# Folded-state compatibility and unfolded-state constraint govern staple-based stabilization: guidelines from a coiled-coil model

**DOI:** 10.1039/d5cb00326a

**Published:** 2026-02-24

**Authors:** Samantha C. Hatfield, Alexa N. Mattingley, Kayla K. Sujeta, Logan D. Humphrey, Taylor Crook, Hiram Aranda, Christian H. Freckleton, Joseph V. Clayson, Chase Renstrom, Joshua L. Price

**Affiliations:** a Department of Chemistry and Biochemistry, Brigham Young University Irvine California USA joshprice@byu.edu

## Abstract

Peptide stapling has emerged as a powerful strategy for stabilizing protein conformation, improving proteolytic resistance, and enhancing biomolecular recognition. Yet design principles for selecting staple sites remain elusive, so advances in stapling have depended largely on trial and error. Here we establish quantitative guidelines for staple placement by exploiting the well-defined geometry of an α-helical coiled coil to compare alternative staple sites in a controlled way. Using both experimental measurements and molecular simulations, we find that (1) staples that link residue pairs that normally form interhelical salt bridges yield greater stabilization than those linking non-salt-bridged pairs; (2) N-terminal staples are more stabilizing than C-terminal staples, where an existing interhelical disulfide constraint reduces their impact; and (3) mismatches between the staple length and site spacing can cause destabilization by forcing the structure into a compressed, non-native geometry. Together, these results show that staple-based stabilization depends on two underlying factors: unfolded-state constraint (the entropic advantage gained when the staple limits how far apart the linked residues can separate in the unfolded ensemble) and folded-state compatibility (how well the staple's maximum accessible span matches the native separation of those residues in the folded structure). These principles provide a predictive framework for rational stapled peptide design, offering a path beyond empirical screening toward principle-guided development of stabilized peptide therapeutics.

## Abstract 2

Peptide stapling has emerged as a powerful strategy for stabilizing protein conformation, improving proteolytic resistance, and enhancing biomolecular recognition.

## Graphical abstract

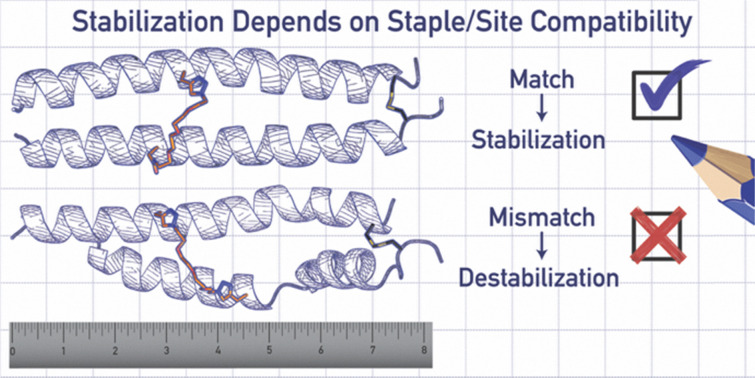

## Introduction

Peptide-based therapeutics continue to expand as a drug class, offering high target specificity and the ability to engage extended protein–protein interaction surfaces that are inaccessible to small molecules,^1–7^ yet their clinical potential is often limited by rapid clearance, conformational instability, and proteolysis.^8^ Macrocyclization or stapling^9–12^ provides a powerful strategy for mitigating these liabilities by covalently linking two residues (referred to here as the anchor positions), thereby constraining a peptide in a conformation that resembles its folded or bound state, effectively prepaying part of the entropic cost of folding or binding. Here we use the term “stapling” for any covalent crosslinking or macrocyclization reaction that connects side-chain and/or backbone groups in a peptide or protein, whether the linked residues reside within a single secondary structural element (*e.g.*, a helix) or across a larger folded architecture. Though early applications of stapling focused primarily on α-helical peptides,^13–15^ subsequent work has applied the same concept broadly to non-helical peptides and folded proteins.^16–21^

Diverse chemistries, including lactams^22,23^ and disulfide linkages,^24^ along with thiol alkylation/arylation,^25–31^ olefin metathesis,^13–15,32–34^ and azide–alkyne cycloaddition,^35–41^ have expanded the accessible range of staples, with additional approaches continuing to emerge.^42–51^ Stapling can improve both conformational stability and proteolytic resistance,^52–54^ and in favorable cases also enhances cell-permeability and target binding affinity.^15,25,27,44,52,55–59^ Despite these successes, the geometric and energetic determinants that make a given site/linker pair stabilizing remain incompletely understood.

Existing design strategies for stapling (most extensively developed for intrahelical stapling of α-helical peptides) typically rely on empirical site selection and iterative screening of multiple linker lengths.^13–15,22–24,41–43,48,51^ Collaborative efforts have yielded qualitative “rules of thumb” for intrahelical stapling,^60^ but there remains little mechanistic guidance on how staple geometry maps onto stabilization energy. Comparable uncertainties arise in the development of PROTACs^61–64^ and antibody–drug conjugates,^65–67^ where some linker choices significantly limit efficacy. Though computational strategies have been applied to optimize linker design in PROTACs,^68–72^ comparable efforts to derive predictive guidelines for peptide stapling are limited, with little attention to why certain staples succeed at particular sites or whether such outcomes could be anticipated. Our previous work showed that staple-induced increases in conformational stability correlate with enhanced proteolytic resistance,^52–54^ suggesting that a quantitative understanding the structural factors that govern staple-based stabilization would meaningfully accelerate the rational design of stapled peptide and proteins with improved proteolytic stability.

To interrogate the geometric factors that govern staple-based stabilization, we used the disulfide-linked α-helical coiled-coil heterodimer A/B^73^ as a spatially well-defined model system. Others have previously applied interhelical stapling to α-helical coiled coils^74–76^ or helix bundles,^77–80^ but with limited attention to the energetic consequences of specific site–staple combinations. A/B folds cooperatively and exhibits a clean two-state thermal unfolding transition, allowing its conformational stability (Δ*G*) to be quantified unambiguously. The regular heptad repeat in A/B creates predictable inter-residue spacings,^81–84^ effectively providing a molecular ruler for varying the positions of the anchor residues and quantifying how site–staple geometry influences stabilization. Its covalent C-terminal disulfide tether fixes its oligomeric state even when unfolded, enabling differences in folding free energy (ΔΔ*G*) to be attributed directly to staple installation. By combining experiment with replica-exchange molecular dynamics simulations, we identify the structural and thermodynamic determinants of staple-based stabilization in A/B—principally folded-state compatibility and unfolded-state constraint—providing mechanistic principles that extend beyond this archetypal coiled-coil scaffold and support the rational design of stapled peptides and proteins. Whereas our prior work on this system (discussed briefly below) examined a small number of representative sites, the present study expands the sequence space systematically, allowing us to isolate the geometric and thermodynamic factors that govern staple-based stabilization in a controlled framework.

## Results and discussion

### Stapling at e/g′ positions: when the staple is just right

**Fig. 1:**
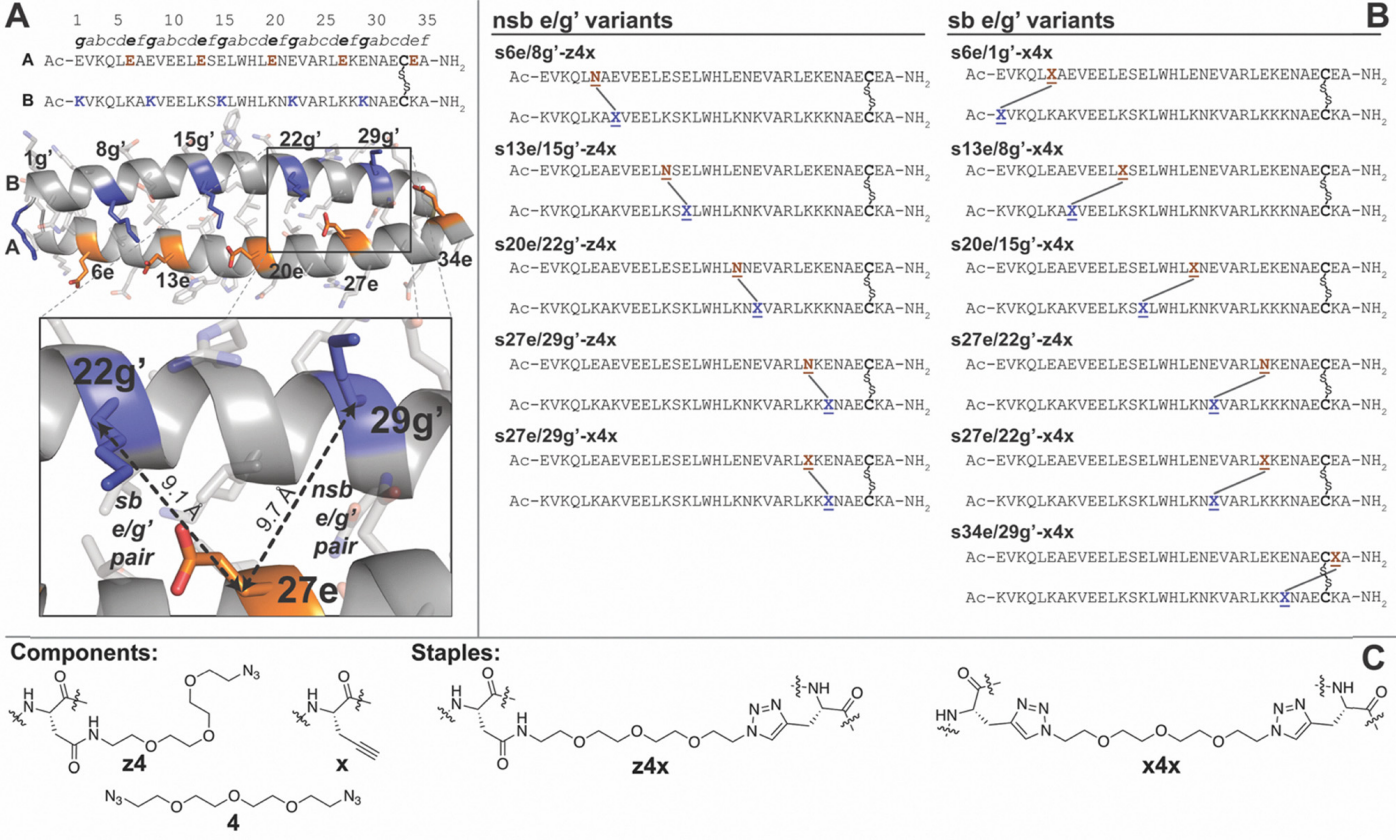
(A) Sequence and structure of coiled coil A/B shown as a ribbon diagram (PDB: 1kd9), with e-positions in subunit A and g-positions in subunit B highlighted in orange and blue, respectively. Inset shows close-up view of nsb e/g′ pair 27e/29g′ *vs.* sb e/g′ pair 27e/22g′. (B) Sequences of stapled variants s6e/8g′-z4x, s13e/15g′-z4x, s20e/22g′-z4x, s27e/29g′-z4x, and s27e/29g′-x4x (in which the staple is incorporated at an nsb e/g′ site) and stapled variants s6e/1g′-x4x, s13e/8g′-x4x, s20e/15g′-x4x, s27e/22g′-z4x, s27e/22g′-x4x, and s34e/29g′-x4x (in which the staple is incorporated at an sb e/g′ site). N̲represents Asn-linked PEG azide z4; X̲represents propargylglycine x; N̲–X̲represents staple z4x and X̲–X̲represents staple x4x. (C) The structures of staples z4x and x4x and components z4, x, and bis-azido PEG 4.

In coiled coil A/B,^73^ e residues pair with g′ residues from the preceding heptad of the opposite subunit: for example, Glu27e from the fourth heptad of subunit A forms a salt bridge with Lys22g′ from the third heptad of subunit B, rather than with Lys29g′ (Fig. 1; apostrophes denote residues in subunit B). Accordingly, we refer to positions like 27e/22g′ as salt-bridged e/g′ pairs (sb) and positions like 27e/29g′ as non-salt-bridged e/g′ pairs (nsb). We previously prepared four variants of A/B in which an Asn-linked four-unit PEG azide (z4) and propargylglycine (x) occupy the e and g′ positions of nsb pairs 6e/8g′, 13e/15g′, 20e/22g′, or 27e/29g′, respectively: we call these variants 6e/8g′-z4x, 13e/15g′-z4x, 20e/22g′-z4x, and 27e/29g′-z4x.^53,54^ We then stapled z4 and x*via* copper-catalyzed azide–alkyne cycloaddition (CuAAC), yielding four stapled variants in which the z4x triazole staple connects the nsb pairs: s6e/8g′-z4x, s13e/15g′-z4x, s20e/22g′-z4x, and s27e/29g′-z4x. We used variable-temperature CD to quantify the stability of each stapled variant relative to its non-stapled counterpart, expressing the effect of the z4x staple as a change in folding free energy (ΔΔ*G*). We found that the z4x staple is most stabilizing (−2.53 ± 0.04 kcal mol^−1^) at the most distal nsb site 6e/8g′, with progressively smaller effects at sites that are more proximal to the C-terminal Cys33-Cys33’ disulfide: −1.33 ± 0.02 kcal mol^−1^ at 13e/15g′; −1.09 ± 0.02 kcal mol^−1^ at 20e/22g′; and −0.65 ± 0.02 kcal mol^−1^ at 27e/29g′ (Table 1). This trend echoes our earlier observations that staples that bridge residues farther apart in sequence can confer greater stabilization than staples at nearby sites.^53,54,85^ Related ideas have emerged from theoretical and experimental studies of disulfide-bonded proteins,^86–92^ which proposed that crosslinks that span longer loops are more stabilizing because they more strongly restrict conformational freedom in the unfolded ensemble. We test this proposal quantitatively using the molecular dynamics simulations described below.

**Table 1:** Observed *vs.* simulated melting temperatures (*T*_m_) for stapled coiled-coil variants and their non-stapled counterparts; the observed *vs.* simulated impact of stapling on coiled-coil conformational stability (ΔΔ*G*); and simulated Cβ–Cβ distances for staple-site residues of each variant at the indicated temperatures, sorted by staple location^a^

Site	Variant	*T*_m_ (°C)		Impact of stapling ΔΔ*G* (kcal mol^−1^)		Cβ–Cβ distances		
		Observed	Simulated	Observed	Simulated	310 K	450 K	Δ 450 K
nsb e/g′	6e/8g′-z4x	39.5 ± 0.2	81.8 ± 0.1			10.5 ± 0.1	59.5 ± 0.2	
	s6e/8g′-z4x	69.1 ± 0.2	101.8 ± 0.3	−2.53 ± 0.04	−2.00 ± 0.06	10.09 ± 0.01	14.40 ± 0.01	−45.1 ± 0.2
	13e/15g′-z4x	42.1 ± 0.1	83.6 ± 0.1			10.18 ± 0.04	50.0 ± 0.3	
	s13e/15g′-z4x	57.7 ± 0.1	92.8 ± 0.2	−1.33 ± 0.02	−1.10 ± 0.04	10.32 ± 0.01	14.65 ± 0.01	−35.3 ± 0.3
	20e/22g′-z4x	41.8 ± 0.2	82.9 ± 0.1			10.45 ± 0.10	38.8 ± 0.1	
	s20e/22g′-z4x	54.3 ± 0.1	89.0 ± 0.1	−1.09 ± 0.02	−0.84 ± 0.02	10.41 ± 0.01	14.07 ± 0.02	−24.8 ± 0.1
	27e/29g′-z4x	41.1 ± 0.2	85.4 ± 0.1			11.6 ± 0.2	23.22 ± 0.04	
	s27e/29g′-z4x	48.2 ± 0.1	87.5 ± 0.1	−0.65 ± 0.02	−0.27 ± 0.02	10.58 ± 0.01	13.55 ± 0.02	−9.68 ± 0.04
	27e/29g′-xx	39.8 ± 0.2	87.4 ± 0.1			11.5 ± 0.1	22.07 ± 0.03	
	s27e/29g′-x4x	52.9 ± 0.2	90.5 ± 0.1	−1.08 ± 0.03	−0.48 ± 0.02	10.55 ± 0.02	13.62 ± 0.02	−8.45 ± 0.04
sb e/g′	6e/1g′-xx	48.7 ± 0.1	87.4 ± 0.1			10.66 ± 0.05	62.5 ± 0.4	
	s6e/1g′-x4x	77.5 ± 0.1	103.6 ± 0.1	−2.96 ± 0.04	−2.03 ± 0.03	9.74 ± 0.02	13.61 ± 0.02	−48.9 ± 0.4
	13e/8g′-xx	41.8 ± 0.2	82.9 ± 0.1			10.01 ± 0.04	53.9 ± 0.3	
	s13e/8g′-x4x	75.6 ± 0.1	101.7 ± 0.4	−3.13 ± 0.05	−1.76 ± 0.08	9.52 ± 0.01	15.08 ± 0.02	−38.8 ± 0.3
	20e/15g′-xx	43.2 ± 0.1	83.8 ± 0.1			9.45 ± 0.01	44.3 ± 0.2	
	s20e/15g′-x4x	74.4 ± 0.1	93.5 ± 0.2	−3.13 ± 0.04	−0.80 ± 0.03	9.71 ± 0.01	14.85 ± 0.02	−29.4 ± 0.2
	27e/22g′-z4x	43.2 ± 0.1	84.6 ± 0.1			9.46 ± 0.02	32.26 ± 0.07	
	s27e/22g′-z4x	63.6 ± 0.1	91.1 ± 0.2	−2.01 ± 0.03	−0.79 ± 0.03	9.62 ± 0.02	14.33 ± 0.01	−17.93 ± 0.07
	27e/22g′-xx	42.5 ± 0.2	85.7 ± 0.1			9.58 ± 0.02	31.3 ± 0.1	
	s27e/22g′-x4x	63.7 ± 0.1	92.0 ± 0.1	−2.07 ± 0.01	−0.72 ± 0.02	9.45 ± 0.01	14.61 ± 0.01	−16.7 ± 0.1
	34e/29g′-xx	51.8 ± 0.1	89.3 ± 0.1			10.02 ± 0.08	14.98 ± 0.02	
	s34e/29g′-x4x	61.9 ± 0.1	92.8 ± 0.1	−1.07 ± 0.01	−0.42 ± 0.02	9.06 ± 0.03	11.20 ± 0.02	−3.78 ± 0.03
f/b′	7f/10b′-xx	48.9 ± 0.2	92.8 ± 0.1			16.11 ± 0.02	49.2 ± 0.3	
	s7f/10b′-x4x	58.0 ± 0.2	98.5 ± 0.4	−0.64 ± 0.01	−0.40 ± 0.05	11.8 ± 0.4	14.73 ± 0.02	−34.5 ± 0.3
	14f/17b′-xx	43.4 ± 0.2	88.3 ± 0.1			16.34 ± 0.01	43.0 ± 0.2	
	s14f/17b′-x4x	—	75.6 ± 0.9	—	0.66 ± 0.06	14.8 ± 0.4	14.65 ± 0.02	−28.4 ± 0.2
	21f/24b′-xx	41.9 ± 0.2	86.6 ± 0.1			16.08 ± 0.04	32.8 ± 0.1	
	s21f/24b′-x4x	—	77.8 ± 0.7	—	0.56 ± 0.06	13.8 ± 0.2	14.14 ± 0.02	−18.6 ± 0.1
	28f/31b′-xx	45.7 ± 0.1	87.2 ± 0.1			16.03 ± 0.05	17.57 ± 0.02	
	s28f/31b′-x4x	37.8 ± 0.3	82.7 ± 0.2	0.62 ± 0.03	0.43 ± 0.03	11.1 ± 0.2	12.79 ± 0.02	−4.78 ± 0.03

a ΔΔ*G* values (observed and simulated) for each stapled variant are given ± standard error relative to its non-stapled counterpart at the *T*_m_ of the non-stapled counterpart (observed or simulated, as appropriate). Observed *T*_m_ and ΔΔG values for stapled variants s6e/8g′-z4x, s13e/15g′-z4x, s20e/22g′-z4x, s27e/29g′-z4x, s27e/29g′-x4x and their non-stapled counterparts are from ref. 53, whereas simulated *T*_m_ and ΔΔ*G* values for these variants are from ref. 93. All other data in the table are newly reported here. In all cases, observed *T*_m_ and ΔΔG and values were obtained from variable temperature CD experiments at 15 µM protein concentration in 20 mM sodium phosphate buffer (pH 7) + 4.0 M GdnHCl, whereas simulated *T*_m_, ΔΔ*G*, and Cβ–Cβ distances were obtained from implicit-solvent T-REMD simulations of each variant (see SI for details). Note that simulated *T*_m_ values are higher in magnitude than the corresponding observed *T*_m_ values. As we have explained previously in ref. 93, this difference likely reflects the fact that our experiments were conducted in high concentrations of denaturant, whereas our simulations were conducted with implicit solvent and without denaturant.

Subsequent implicit-solvent parallel temperature replica exchange molecular dynamics (T-REMD) simulations^93^ on stapled variants s6e/8g′-z4x, s13e/15g′-z4x, s20e/22g′-z4x, and s27e/29g′-z4x and their non-stapled counterparts sampled 16 parallel temperatures from 310 to 450 K, yielding simulated ΔΔG values that follow a similar trend. Although the implicit-solvent model systematically underestimates the absolute ΔΔ*G* values, it reliably captures their relative order. Analysis of the trajectories from each variant provides a compelling rationale for the observations described above. In all trajectories, the Cys33–Cys33′ disulfide constrains the C-termini of subunits A and B to remain close, regardless of stapling. However, in the most distal variant s6e/8g′-z4x, the staple also holds their N-termini together, even at elevated temperatures. As a result, the unfolded ensemble of s6e/8g′-z4x is more compact and has less conformational freedom than its non-stapled counterpart, leading to a higher probability of refolding, and a greater stability at elevated temperature. In contrast, the staple in the most proximal variant s27e/29g′-z4x lies much farther from the N-termini of subunits A and B, which remain nearly as free to separate as when no staple is present. Consequently, the z4x staple in variant s27e/29g′-z4x affords less protection from thermal unfolding than in s6e/8g′-z4x.

Having established that the z4x staple's effects arise from its geometric restraint of the unfolded ensemble (*i.e.*, unfolded-state constraint), we next asked whether a more synthetically convenient staple would show comparable behavior. Our earlier stapling efforts relied on the z4x staple,^53,54^ which requires access to Fmoc-protected derivatives of propargylglycine x (commercially available) and of Asn-linked PEG-azide z4 (synthesized in our lab). As a more synthetically convenient alternative, we envisioned connecting two x residues with a single bis-azido PEG *via* CuAAC: for example, four-unit bis-azido PEG 4 connects two x residues to form the x4x staple (Fig. 1). Previous experiments confirmed that the x4x staple is a reasonable surrogate for the z4x staple: it is moderately more stabilizing at nsb 27e/29g′ (−1.08 ± 0.03 kcal mol^−1^*vs.* −0.65 ± 0.02 kcal mol^−1^), and similarly stabilizing at sb 27e/22g′ (−2.07 ± 0.03 *vs.* −2.01 ± 0.02 kcal mol^−1^), giving an average difference of ΔΔΔ*G* = −0.25 ± 0.03 kcal mol^−1^ for x4x*vs.*z4x.^53^ Previously reported T-REMD simulations of s27e/29g′-x4x and s27e/29g′-z4x,^93^ together with new simulations of s27e/22g′-x4x and s27e/22g′-z4x (Table 1; see SI), are consistent with these observations: the x4x staple is slightly more stabilizing at nsb 27e/29g′ (−0.48 ± 0.02 kcal mol^−1^*vs.* −0.27 ± 0.02 kcal mol^−1^), and similarly stabilizing at sb 27e/22g′ (−0.72 ± 0.02 *vs.* −0.79 ± 0.03 kcal mol^−1^), giving an average simulated difference of ΔΔΔ*G* = −0.07 ± 0.02 kcal mol^−1^ for x4x*vs.*z4x. Therefore, we consider the z4x and x4x staples interchangeable and use the more synthetically convenient x4x staple in the new variants described below.

We next asked whether staple-based stabilization at salt-bridged (sb) e/g′ pairs would show a similar dependence on distance from the C-terminal Cys33–Cys33’ disulfide as observed previously for non-salt-bridged (nsb) e/g′ pairs. We explored this possibility by preparing non-stapled variants 6e/1g′-xx, 13e/8g′-xx, 20e/15g′-xx, 27e/22g′-xx, or 34e/29g′-xx, in which we incorporated propargylglycine x residues at the corresponding sb e/g′ pairs. We next connected the paired x residues in each construct with bis-azido PEG 4*via* CuAAC, yielding stapled variants s6e/1g′-x4x, s13e/8g′-x4x, s20e/15g′-x4x, s27e/22g′-x4x, or s34e/29g′-x4x. Variable temperature CD experiments (Table 1) reveal that x4x-based stabilization at sb e/g′ pairs increases with distance from the C-terminal Cys33–Cys33′ disulfide, rising from proximal 34e/29g′ (−1.07 ± 0.01 kcal mol^−1^) to more distal 27e/22g′ (−2.07 ± 0.03 kcal mol^−1^) and then plateauing beyond 20e/15g′ (−3.13 ± 0.04 kcal mol^−1^ for 20e/15g′; −3.13 ± 0.05 kcal mol^−1^ for 13e/8g′; −2.96 ± 0.04 kcal mol^−1^ for 6e/1g′), an observation examined in more detail below. In contrast, at nsb e/g′ pairs, z4x stapling provides stabilization that increases approximately linearly from proximal 27e/29g′ to distal 6e/8g′, without the leveling off seen at sb sites.

T-REMD simulations on these x4x-stapled variants and their non-stapled counterparts qualitatively mirror the experimental proximal-to-distal stabilization trend described above for sb e/g′ sites, but with an important difference: in the simulations, the plateau in staple-based stabilization occurs later: stabilization is weakest at proximal 34e/29g′ (−0.42 ± 0.02 kcal mol^−1^), increases modestly at 27e/22g′ and 20e/15g′ (−0.72 ± 0.02 kcal mol^−1^; −0.80 ± 0.03 kcal mol^−1^), and reaches the largest predicted values at 13e/8g′ and 6e/1g′ (−1.76 ± 0.08 kcal mol^−1^; −2.03 ± 0.03 kcal mol^−1^). Comparison of simulated and experimental *T*_m_ and Δ*G* values suggests that the simulations underestimate the stability of s20e/15g′-x4x relative to the other variants. Because the implicit-solvent model we used does not fully capture site-specific solvation effects, modest discrepancies of this kind are not unexpected. In any case, the purpose of our simulations was to reveal qualitative mechanistic trends rather than to reproduce absolute ΔΔ*G* values. One possible explanation for this discrepancy is that x4x-based stabilization at 20e/15g′ arises in part from a structural feature not represented in the implicit-solvent model. For example, if stapling at 20e/15g′ protects a critical hydrogen bond or other non-covalent interaction from solvent exposure, the resulting stabilization could exceed that predicted by preorganization alone; however, our current data do not provide a definitive explanation.

Both experiments and simulations show that stapling confers greater stabilization at sb than at nsb e/g′ sites that are similarly distal to the C-terminal disulfide, though they differ in the magnitude of this effect (−1.42 ± 0.02 kcal mol^−1^ in experiments *vs.* −0.28 ± 0.03 kcal mol^−1^ in simulations). One possible explanation is that the z4x and x4x staples may be better matched to the folded-state geometry of the sb than nsb e/g′ sites, an idea we refer to here as folded-state compatibility. Specifically, if these staples comfortably span the side-chain β-carbon distance of the e/g′ anchor positions (the Cβ–Cβ distance, used here as measure of folded-state anchor spacing) at sb sites but approach their maximum span at nsb sites, then the resulting geometric strain could attenuate stabilization at nsb positions. We explored this possibility by examining (1) the intrinsic folded-state anchor spacing of the prospective e/g′ staple site in each non-stapled variant; (2) the folded-state anchor spacing at the same site in the corresponding stapled variant; and (3) several independent estimates of the maximum span accessible to the z4x and x4x staples.

To assess the folded-state compatibility of the z4x and x4x staples with nsb *vs.* sb e/g′ sites, we compared the Cβ–Cβ distance for the prospective e/g′ staple site in the folded conformation of each non-stapled variant with the corresponding distance at the same site its stapled counterpart, using the 310 K T-REMD trajectories described above. In the non-stapled variants, these 310 K Cβ–Cβ distances reflect the intrinsic folded-state anchor spacing of each site: nsb e/g′ positions span 10.1–11.6 Å (average 10.86 ± 0.05 Å), whereas sb e/g′ positions span 9.4–10.7 Å (average 9.86 ± 0.02 Å). This difference is consistent with the possibility that the z4x and x4x staples are well matched to the shorter native spacing of sb e/g′ sites, whereas the slightly longer intrinsic spacing of nsb sites might approach or exceed the staples’ maximum accessible span. However, in the stapled variants, the 310 K Cβ–Cβ distances remain essentially unchanged—10.1–10.6 Å for nsb sites (average 10.39 ± 0.01 Å) and 9.1–9.7 Å for sb sites (average 9.52 ± 0.01 Å)—and each adopts a well-folded coiled coil conformation like that of its non-stapled counterpart. Because neither staple distorts native coiled-coil geometry or forces the anchor residues from their intrinsic folded-state positions, we conclude that z4x and x4x show comparable folded-state compatibility at both nsb and sb e/g′ sites.

**Fig. 2:**
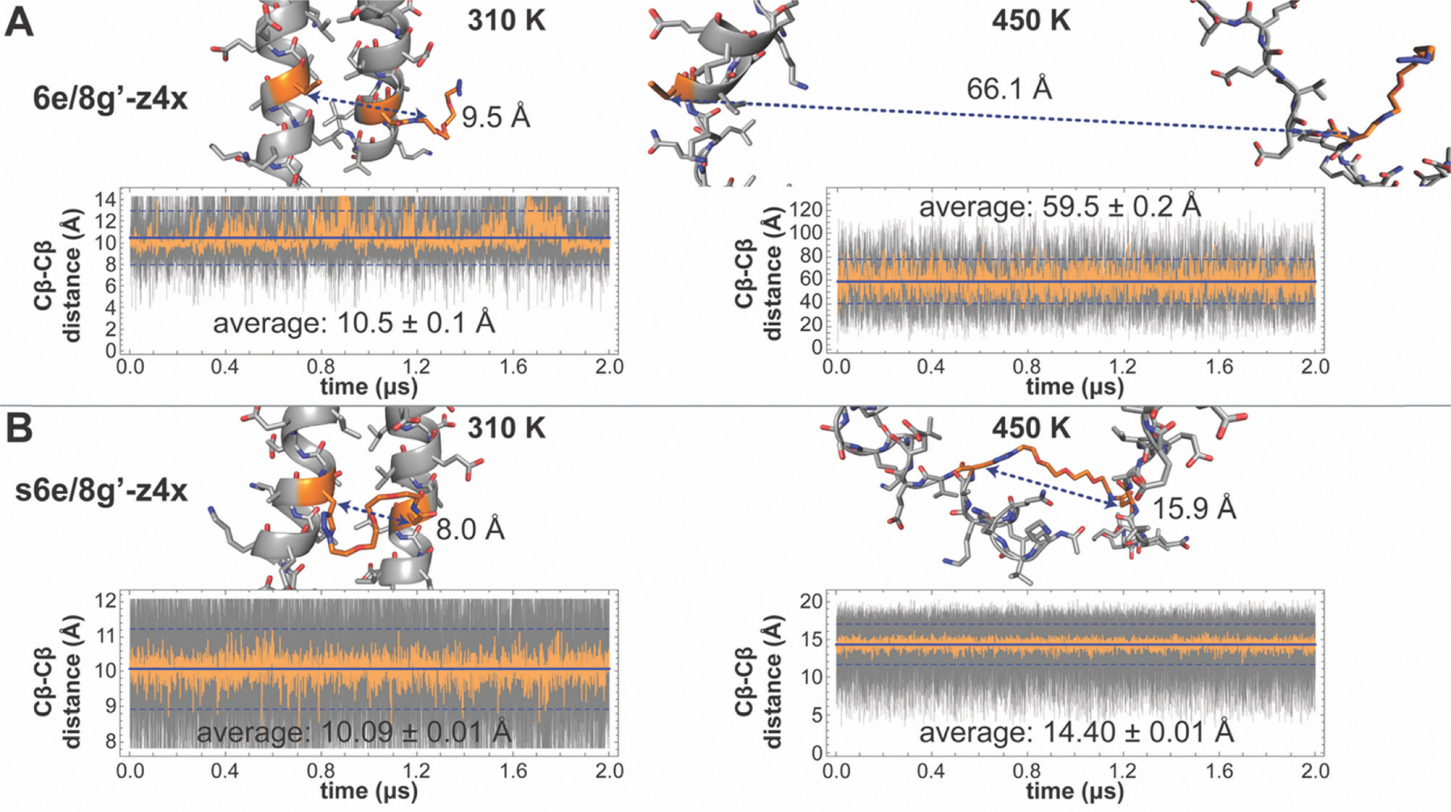
Ribbon diagrams of median-energy frames extracted from the 310 K and 450 K trajectories from T-REMD simulations of (A) non-stapled variant 6e/8g′-z4x and its stapled counterpart s6e/8g′-z4x (B). Side chains are shown as sticks, with nsb site 6e/8g′ highlighted in orange. Cβ–Cβ distances for the nsb site 6e/8g′ within these frames are shown with dashed double-headed arrow; Cβ–Cβ distances for the nsb site 6e/8g′ are plotted *vs.* simulation time for each trajectory (gray lines). Orange lines represent average Cβ–Cβ distance within a moving window of the 50 points that surround each corresponding point in the gray line. The solid blue line shows the average Cβ–Cβ distance over the trajectory from *t* = 40 ns to *t* = 2 µs. Dotted blue lines show this trajectory average Cβ–Cβ distance ± one standard deviation.

We sought to validate this conclusion by examining three independent estimates of the staples’ maximum accessible span and comparing them to the intrinsic folded-state anchor spacings described above for nsb *vs.* sb e/g′ sites. First, DFT-optimized extended conformations of isolated z4x and x4x staple models yield Cβ–Cβ distances of 19.3 and 20.4 Å, respectively, values that likely overestimate the true maximum span, because such fully extended conformations are unlikely when the staple is anchored within a coiled coil. Second, MD simulations of unconstrained z4x and x4x staple models yield average Cβ–Cβ distances of 10.96 ± 0.04 Å and 10.66 ± 0.04 Å, respectively. Third, the z4x and x4x staples reach substantially larger spans in the 450 K T-REMD trajectories of the stapled variants (Fig. 2), where the unfolded peptide chain pulls on the staple from both ends: 13.5–14.7 Å for z4x (average 13.83 ± 0.01 Å) and 11.2–15.1 Å for x4x (average 14.20 ± 0.01 Å). These unfolded-state Cβ–Cβ distances likely provide the most realistic estimate of maximum accessible staple span. In all cases, the accessible staple lengths exceed the intrinsic folded-state spacing of both nsb and sb e/g′ sites, indicating that better folded-state compatibility is not the source of the enhanced stabilization observed at sb sites, and that that the z4x and x4x staples can readily span both classes of anchor positions.

**Fig. 3:**
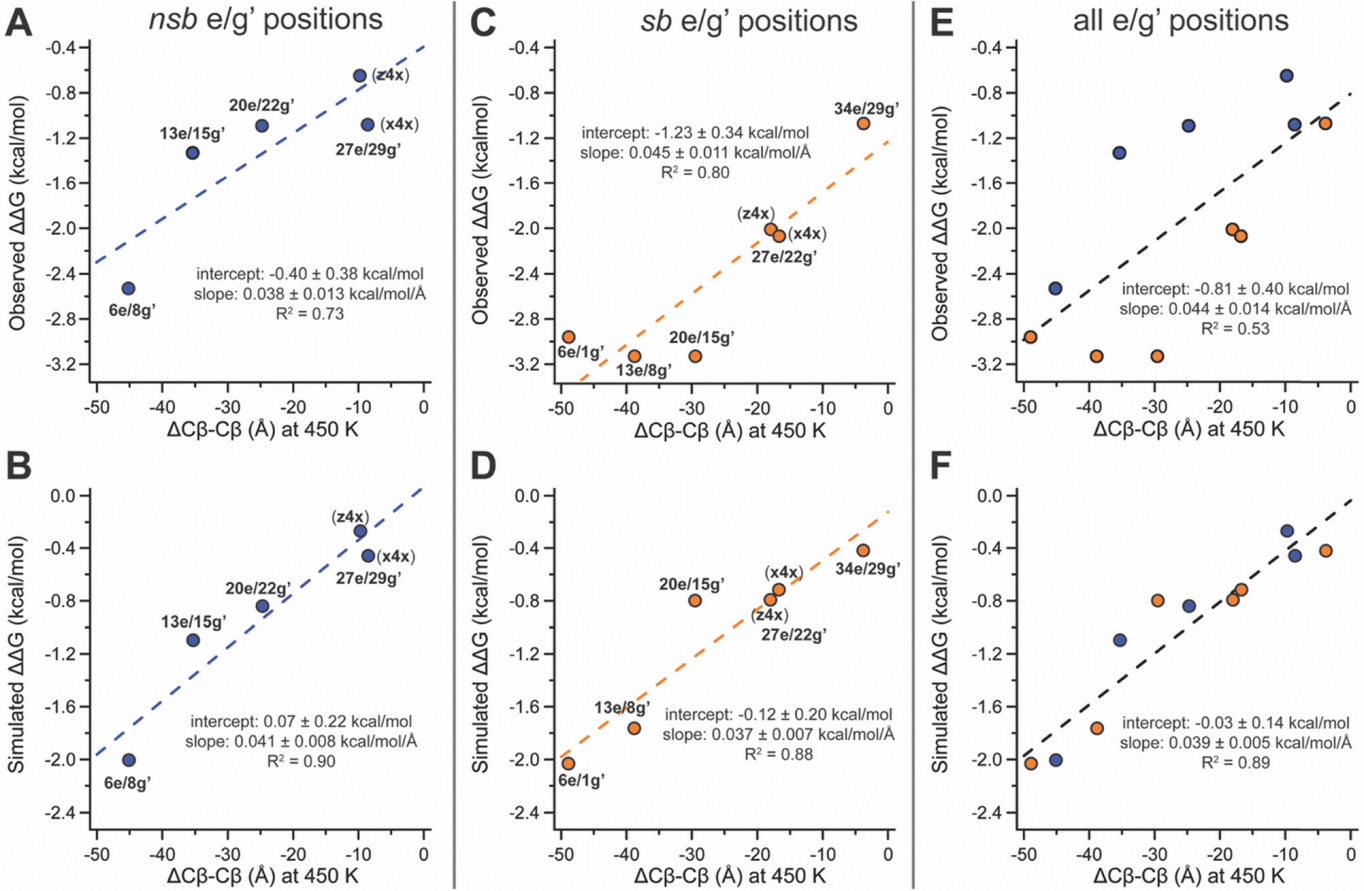
Plots of observed and simulated staple-based stabilization ΔΔ*G vs.* unfolded ΔCβ–Cβ at nsb e/g′ sites (A) and (B); sb e/g′ sites (C) and (D); and all e/g′ sites (E) and (F) together. Dotted lines represent fits of the data to a linear equation, with the indicated intercept, slope, and *R*^2^ values.

Having ruled out folded-state compatibility as the primary source of the different stabilization observed at nsb *vs.* sb e/g′ sites, we next examined how stapling alters the separation of the anchor residues in the unfolded ensemble. The reduction in unfolded-state (450 K) anchor spacing (*i.e.*, Cβ–Cβ distance) in stapled variants relative to their non-stapled counterparts provides a quantitative measure of unfolded-state constraint that we call ΔCβ–Cβ (Fig. 2). Unfolded-state anchor spacings remain similar across the z4x- or x4x-stapled variants (Table 1), but their non-stapled counterparts show much greater variation, increasing sharply with distance from the C-terminal disulfide. For example, at non-stapled nsb e/g′ pairs, unfolded Cβ–Cβ distances are 23.22 ± 0.04 Å (z4x) or 22.07 ± 0.03 Å (x4x) at proximal 27e/29g′, and 38.8 ± 0.1 Å, 50.0 ± 0.3 Å, and 59.5 ± 0.2 Å at 20e/22g′, 13e/15g′, and distal 6e/8g′, respectively. The covalent constraint of the staple collapses these unfolded anchor spacings to the shorter values observed in the stapled variants (Table 1; Fig. 2 illustrates this for s6e/8g-z4x*vs.*6e/8g-z4x), giving ΔCβ–Cβ values that become progressively more negative at more distal sites. For nsb e/g′ pairs, ΔCβ–Cβ is largest in magnitude at distal 6e/8g′ (−45.1 ± 0.2 Å) and decreases at 13e/15g′ (−35.3 ± 0.3 Å), 20e/22g′ (−24.8 ± 0.1 Å), and proximal 27e/29g′ (−9.68 ± 0.04 Å for z4x; −8.45 ± 0.04 for x4x), correlating strongly with the observed and simulated magnitudes of staple-based stabilization (ΔΔ*G*) at these sites (Fig. 3A and B).

We observed similar trends at sb e/g′ pairs: ΔCβ–Cβ is again largest in magnitude at distal 6e/1g′ (−48.9 ± 0.4 Å) and decreases at 13e/8g′ (−38.8 ± 0.3 Å), 20e/15g′ (−29.4 ± 0.2 Å), 27e/22g′ (−16.7 ± 0.1 for x4x; −17.93 ± 0.07 for z4x), and proximal 34e/29g′ (−3.78 ± 0.03 Å), correlating strongly with both the observed and simulated ΔΔG values at these sites (Fig. 3C and D). Across sites located at comparable distances from the C-terminal disulfide, unfolded ΔCβ–Cβ tends to be larger in magnitude for sb *vs.* nsb e/g′ pairs (Table 1; compare sb 6e/1g′ with nsb 6e/8g′), consistent with the notion that stapling overcomes more conformational entropy at sb e/g′ sites and therefore might confer greater stabilization. If unfolded-state constraint (ΔCβ–Cβ) were the sole determinant of ΔΔ*G*, then plotting ΔΔ*G vs.* ΔCβ–Cβ across all e/g′ sites should yield a single linear relationship encompassing both nsb and sb data points. This expectation is borne out in the simulations, where simulated ΔΔ*G* values scale linearly with ΔCβ–Cβ across all nsb and sb e/g′ sites combined (Fig. 3F, *R*^2^ = 0.89); in addition, separate fits of simulated ΔΔ*G vs.* ΔCβ–Cβ for the nsb and sb subsets give similar slopes, intercepts, and *R*^2^ values (Fig. 3B and D). In contrast, the experimental ΔΔ*G* values correlate more weakly with ΔCβ–Cβ when both nsb and sb e/g′ sites are considered together (Fig. 3E, *R*^2^ = 0.53). Separate fits of experimental ΔΔ*G vs.* ΔCβ–Cβ for nsb and sb sites yield similar slopes and *R*^2^ values, but markedly different intercepts (Fig. 3A and C), indicating that although ΔCβ–Cβ influences stabilization similarly within each class, stapling at sb sites is intrinsically more stabilizing than at nsb sites, for reasons not explained by unfolded-state constraint alone.

Taken together, these results suggest that unfolded-state constraint, as captured by ΔCβ–Cβ, provides a useful quantitative descriptor of staple-based stabilization within either the nsb or sb e/g′ subset, but does not fully account for the systematic enhancement observed at sb e/g′ positions. Our implicit-solvent T-REMD simulations reproduce the ΔΔ*G vs.* ΔCβ–Cβ scaling expected if unfolded-state constraint were the dominant contributor and reveal no systematic difference between nsb and sb sites, implying that the additional stabilization observed experimentally at sb sites arises from effects not captured by the implicit-solvent model. One possibility is that solvent-mediated interactions, differential solvent exposure, or other environment-dependent contributions differ between sb and nsb e/g′ sites in ways that are not reflected in ΔCβ–Cβ alone. Despite this limitation, plots of observed *vs.* simulated *T*_m_ and ΔΔ*G* values show good overall agreement, indicating that the simulations correctly identify the most and least stabilizing e/g′ sites, even though they underestimate the additional sb-specific stabilization observed experimentally (see SI).

### Sequence separation as a broadly useful surrogate for unfolded-state constraint

**Fig. 4:**
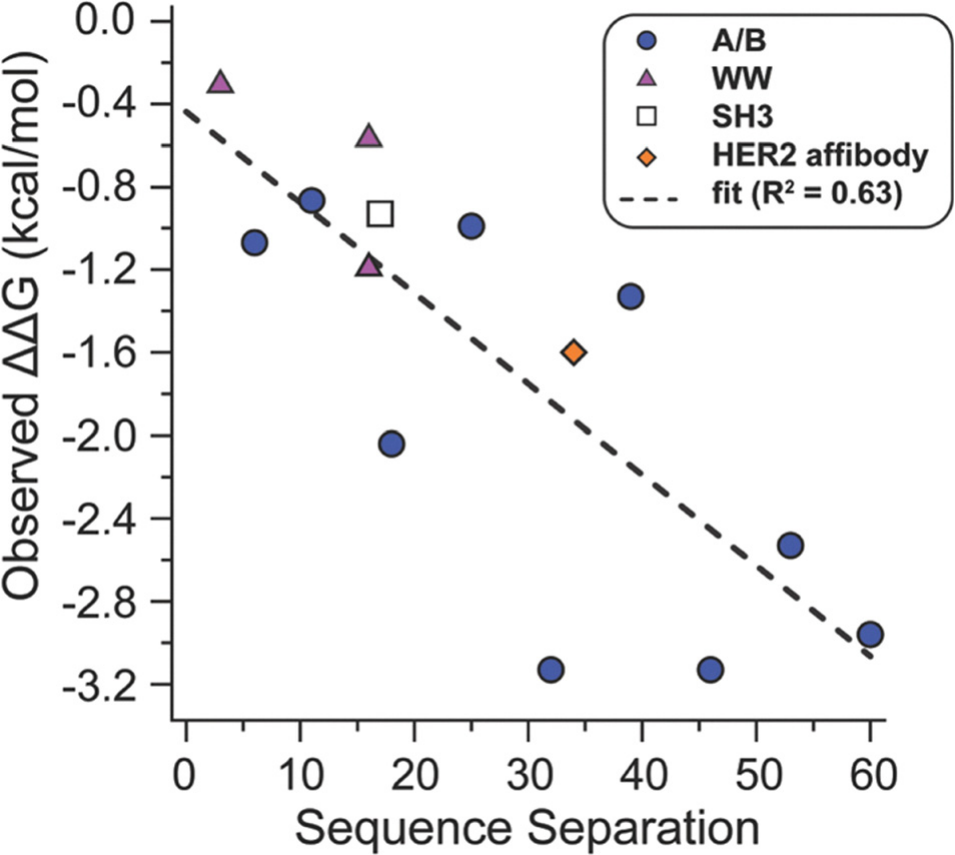
Plot of observed staple-based stabilization (ΔΔ*G*) within coiled-coil A/B, and previously reported WW, SH3, and HER2 affibody scaffolds *vs.* the sequence separation of the staple anchor positions shows a linear relationship (*R*^2^ = 0.63)

The strong correlation between ΔΔ*G* and ΔCβ–Cβ at e/g′ sites suggests that unfolded-state constraint is a key contributor to staple-based stabilization whenever the staple is long enough to accommodate the site's intrinsic folded-state spacing. Because ΔCβ–Cβ requires simulation to compute, we wondered whether a simpler, experimentally accessible metric might capture the same underlying effect. Across the e/g′ positions in A/B, the magnitude of ΔCβ–Cβ tracks closely with the sequence separation between the two anchor residues. This relationship is expected: residues that are widely separated in sequence can access larger average separations in the unfolded ensemble, so constraining them with a staple should yield a larger entropic benefit. To test whether sequence separation serves as a useful predictor beyond the A/B coiled coil variants explored so far, we examined previously reported ΔΔ*G* values for stapled variants in several additional protein scaffolds that differ substantially from A/B: the WW domain, the SH3 domain, and the HER2 helix bundle affibody. Though these systems differ from each other in topology (A/B is a disulfide-bound coiled coil; HER2 affibody is a monomeric three-helix bundle; SH3 and WW domain are both β-sheet rich monomers), a consistent trend emerges cross staple sites. When ΔΔG values from all four scaffolds are plotted against sequence separation, the data fall along a common trajectory: staples between anchor positions that are farther apart in sequence produce greater stabilization, reflecting greater imposed unfolded-state constraint. Consistent with the ΔCβ–Cβ analysis above, the A/B data presented here follow this relationship closely, as do the cross-scaffold data, with only modest scaffold-dependent scatter (Fig. 4).

These results suggest that sequence separation provides a broadly applicable simulation-free surrogate for the unfolded- state constraint contribution to staple-based stabilization, so long as the staple is geometrically compatible with the folded-state spacing of the prospective anchor positions. Practically, sequence separation offers a rapid and intuitive first-pass screen for selecting candidate staple sites: residues that are close in space in the folded state but far apart in the sequence are expected to yield the greatest entropic benefit upon stapling. Having established the general connection between sequence separation, unfolded-state constraint, and stabilization, we next turned to positions where this framework breaks down: the f/b’ sites, where intrinsic folded-state spacing exceeds the span of the z4x and x4x staples. These positions illustrate the contrasting regime where folded-state geometric incompatibility rather than unfolded state entropy dominates the behavior of stapled variants.

### Stapling at f/b′ positions: when the staple is too short

**Fig. 5:**
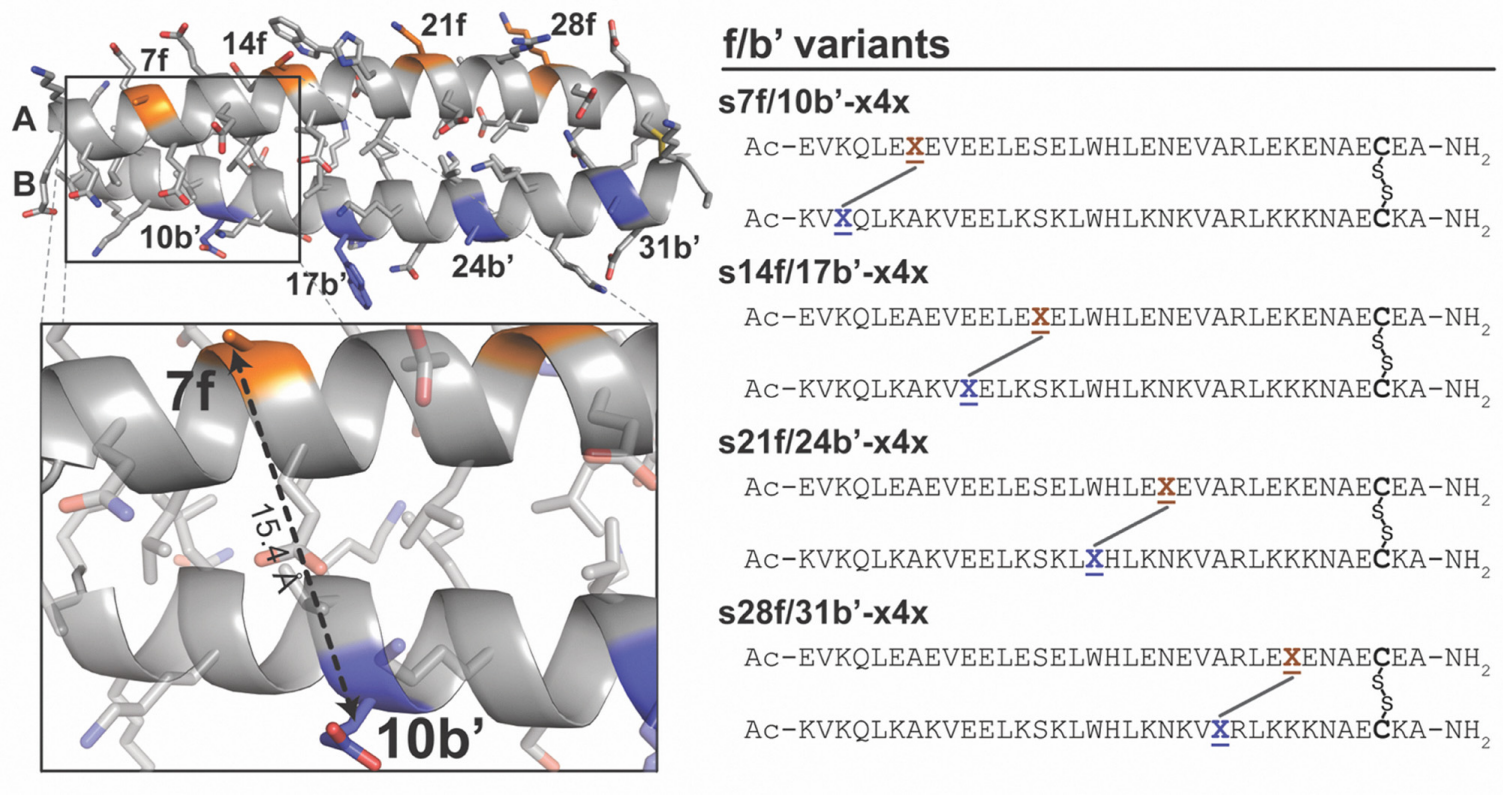
Sequence and structure of coiled coil A/B shown as a ribbon diagram (PDB: 1kd9), with f-positions in subunit A and b-positions in subunit B highlighted in orange and blue, respectively. Inset shows close-up view of f/b′ pair 7f/10b′. Also shown are sequences of stapled variants s7f/10b′-x4x, s14f/17b′-x4x, s21f/24b′-x4x, and s28f/31b′-x4x. X̲represents propargyl glycine x and X̲–X̲represents staple x4x.

Stapling at f/b′ positions presents a qualitatively different geometric challenge from stapling at e/g′ sites and serves as a useful boundary case for assessing the biophysical consequences when staple length becomes limiting. In coiled coil A/B, f residues in subunit A and b′ residues in subunit B point away from the helix-helix interface (Fig. 5), placing them substantially farther apart (Cβ–Cβ distance of 15.4 Å for 7f and 10b′ in the A/B crystal structure) than the nsb or sb e/g′ pairs described above (Table 1). However, the Cβ–Cβ distance for 7f/10b′ is only slightly beyond the ∼14 Å maximum accessible span inferred above for the z4x or x4x staples (Fig. 2 and Table 1), and it was unclear what kind of energetic penalty might be incurred as a result. Based on simple distance comparisons alone, such sites might therefore appear marginally compatible with the z4x or x4x staples. Consistent with this ambiguity, we previously found that stapling at 7f/10b′ with z4x was stabilizing (−0.61 ± 0.03 kcal mol^−1^),^53^ but substantially less so than at nearby nsb 6e/8g′ (−2.53 ± 0.04 kcal mol^−1^), even though the two sites have nearly the same sequence separation. This result suggested that f/b′ positions lie near a geometric threshold where small mismatches between staple span and intrinsic folded-state anchor spacing could lead to qualitatively different energetic outcomes. To determine whether this behavior is specific to 7f/10b′ or reflects a general failure mode for f/b′ sites, and to assess whether such cases could be identified prospectively, we systematically examined the additional f/b′ positions in A/B using both experiment and T-REMD simulations.

We prepared and characterized stapled variants s7f/10b′-x4x, s21f/24b′-x4x, and s28f/31b′-x4x, together with their non-stapled counterparts, but were unable to prepare stapled s14f/17b′-x4x despite repeated efforts. Variable temperature CD experiments reveal that x4x stapling is only weakly stabilizing at distal 7f/10b′ (−0.64 ± 0.01 kcal mol^−1^); moderately destabilizing at proximal 28f/31b′ (0.62 ± 0.03 kcal mol^−1^); and strongly destabilizing at 21f/24b′ where s21f/24b′-x4x was too unstable to obtain reliable *T*_m_ or Δ*G* values). The synthetic intractability of s14f/17b′-x4x is consistent with an expectation of comparable or greater destabilization. Collectively, these results indicate that the intrinsic folded-state geometry of f/b′ positions places the anchor residues beyond the effective reach of the x4x (and by analogy z4x) staples, and that this folded-state geometric incompatibility leads to destabilization rather than stabilization.

T-REMD simulations corroborate these experimental trends. In the simulations, x4x stapling is slightly stabilizing at 7f/10b′ (−0.40 ± 0.05 kcal mol^−1^); strongly destabilizing at 14f/17b’ (0.66 ± 0.06 kcal mol^−1^) and 21f/24b′ (0.56 ± 0.06 kcal mol^−1^); and modestly destabilizing at 28f/31b′ (0.43 ± 0.03 kcal mol^−1^; Table 1). Notably, the simulations predict the greatest destabilization at 14f/17b′: the same site where we were unable to install the staple experimentally. The geometric origin of this trend becomes clear when comparing the intrinsic folded-state anchor spacing of the non-stapled variants with the maximum effective span of the x4x staple. In the unfolded (450 K) conformations of stapled variants s7f/10b′-x4x, s14f/17b′-x4x, s21f/24b′-x4x, and s28f/31b′-x4x, the f/b′ Cβ–Cβ distances converge to 12.79–14.73 Å (average 14.08 ± 0.01 Å) consistent with the maximum effective reach inferred above for x4x at e/g′ sites. In contrast, in the folded (310 K) conformations of the corresponding non-stapled variants, native f/b′ Cβ–Cβ distances (16.03–16.34 Å) exceed the ∼14 Å reach of the x4x staple, indicating that the staple is incompatible with the intrinsic folded-state spacing at these positions. Accordingly, stapling at f/b′ sites necessarily forces the coiled coil into a compressed non-native geometry that shortens the natural f/b’ anchor spacing. Indeed, 310 K T-REMD simulations of the stapled variants show that the f/b′ spacing collapses to 11.1–14.8 Å, confirming that the staple imposes a non-native geometry on the folded state. Thus, folded-state incompatibility rather than unfolded-state constraint dominates the behavior of f/b′ sites and explains the destabilization seen experimentally and computationally.

**Fig. 6:**
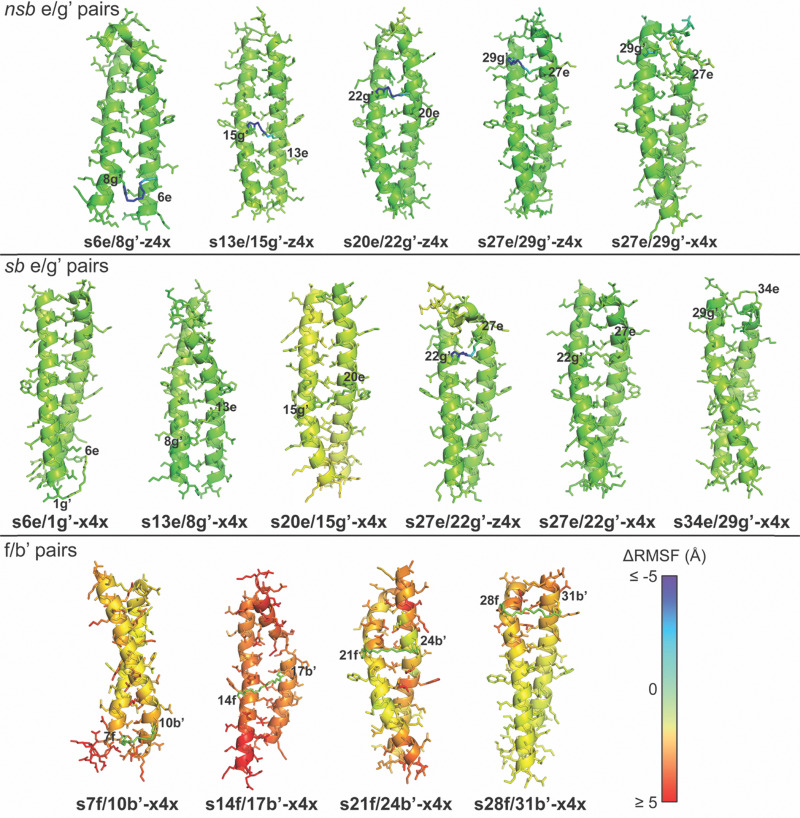
Ribbon diagrams of median-energy frames from 310 K T-REMD trajectories of stapled variants s6e/8g′-z4x, s13e/15g′-z4x, s20e/22g′-z4x, s27e/29g′-z4x, and s27e/29g′-x4x (nsb e/g′ sites); s6e/1g′-x4x, s13e/8g′-x4x, s20e/15g′-x4x, s27e/22g′-z4x, s27e/22g′-x4x, and s34e/29g′-x4x (sb e/g′ sites); s7f/10b′-x4x, s14f/17b′-x4x, s21f/24b′-x4x, and s28f/31b′-x4x (f/b’ sites), with side chains shown as sticks. Atoms in each stapled variant are colored by the difference in root-mean-square fluctuation (ΔRMSF) for that atom relative to the corresponding atom in its non-stapled counterpart, according to the heat-map scale to the right, where red, green, and blue represent increased (≥ 5.0 Å), unchanged (0.0 Å), and decreased (≤−5.0 Å) RMSF, respectively. Because the components of the z4x staple are present in both stapled and non-stapled variants, we can directly compare of RMSF values for the staple atoms before and after stapling, so the atoms of z4x are colored. In contrast, this is not possible for the x4x staple, where the atoms of bis-azido PEG 4 are not present in the non-stapled variants; therefore, the atoms of x4x are colored gray.

We next examined how this staple-enforced contraction propagates structural distortion through the coiled coil by computing atom-wise root-mean-square fluctuation (RMSF) differences between stapled and non-stapled variants in the 310 K T-REMD simulations (Fig. 6). Stapling at distal 7f/10b′ or proximal 28f/31b′ produces modest locally confined RMSF increases, limited primarily to the immediately adjacent residues: N-terminal to the staple for 7f/10b′ or C-terminal for 28f/31b′. In contrast, stapling at the centrally located 14f/17b′ and 21f/24b′ positions generates substantial RMSF increases on both sides of the staple. For example, s21f/24b′-x4x displays elevated RMSF at both termini, and s14f/17b′-x4x exhibits widespread increases throughout the structure, which deviates substantially from ideal coiled-coil geometry (Fig. 5). These differing consequences of folded-state geometric incompatibility at peripheral *vs.* central f/b′ sites suggest that near the termini, the coiled coil can absorb deformation locally, but that in the central region enforced shortening of the intrinsic f/b′ anchor spacing propagates into nearby a and d positions, disrupting the hydrophobic core and strongly destabilizing the fold. In contrast, when the enforced and ideal Cβ–Cβ distances coincide—as with the folded-state compatible z4x or x4x staples at the nsb or sb e/g′ sites—the staple does not introduce substantial RMSF changes. Together, these results indicate that the observed destabilization at f/b′ pairs represents a predictable failure mode for x4x and z4x stapling, arising specifically from geometric incompatibility of the maximum accessible staple span with the intrinsic folded-state anchor spacing. Importantly, this incompatibility can be identified using simulation-derived metrics, providing a prospective means to avoid staples that are intrinsically too short for a given site.

Re-examination of previously published data reveals a similar sensitivity to folded-state geometric compatibility. In the A/B scaffold, we previously found that a shorter z2x staple was destabilizing at the nsb site 27e/29g′ (0.31 ± 0.01 kcal mol^−1^), a location where its longer counterpart z4x was stabilizing (−0.65 ± 0.02 kcal mol^−1^). In contrast, z2x was strongly stabilizing at the nearby sb 27e/22g′ site (−2.04 ± 0.03 kcal mol^−1^), matching the effect of z4x, and illustrating that the shorter staple is compatible with the tighter intrinsic sb spacing, but not with the wider nsb spacing (Table 1). We observed similar behavior in the WW domain: a short two-ethylene-oxide staple linking positions 16 and 19 destabilized the fold by 0.17 ± 0.02 kcal mol^−1^ when installed at anchor positions 16 and 19, whereas a five-unit staple at the same positions was stabilizing (−0.29 ± 0.02 kcal mol^−1^). Together with the f/b′ results above, these observations support the general principle that staple length must be matched to the intrinsic folded-state anchor spacing at a prospective staple site to avoid introducing geometric strain.

## Conclusions

Here we combined experiments and molecular simulations to identify the structural determinants that govern staple-based stabilization in a coiled-coil model system. Three guidelines emerge for staple placement within the A/B scaffold:

(1) Salt-bridged *vs.* non-salt-bridged sites: staples at salt-bridged (sb) e/g′ pairs are more stabilizing than those at non-salt-bridged (nsb) pairs positioned at comparable distances from the C-terminal disulfide.

(2) Positional context: staples placed farther from the C-terminal disulfide provide greater stabilization than proximal staples, where the pre-existing covalent tether limits their entropic contribution.

(3) Geometric compatibility: stabilization requires the staple to match the intrinsic folded-state Cβ–Cβ spacing of the prospective site. When the staple is too short it forces the linked residues into a compressed non-native geometry, as observed for x4x at f/b′ positions.

Although these guidelines were derived from a single coiled-coil scaffold, the two mechanistic principles underlying them appear more broadly applicable to stapled proteins and peptides in general.

(1) Reducing unfolded-state separation enhances stabilization: staples are most stabilizing when they substantially restrict separation of the linked residues in the unfolded ensemble. The larger the reduction in unfolded-state distance upon stapling (*i.e.*, the greater the unfolded-state constraint), the larger the entropic benefit. In practice, this means that (i) staples that link residues close in sequence are typically less stabilizing than those that link residues farther apart, and (ii) staples placed near an existing covalent connection (*e.g.*, a disulfide or another staple) have a diminished effect.

(2) Compatibility with folded-state geometry enhances stabilization: staples should reinforce, rather than distort, the native folded-state spacing between anchor residues. Across the A/B coiled coil and the WW domain, short staples (*e.g.*, x4x at f/b′ sites and z2x at nsb e/g′ sites in A/B, and the two-unit staple at positions 16 and 19 in WW) destabilize the fold when the intrinsic anchor spacing exceeds the staple's accessible span. These observations emphasize a general principle: stabilization requires folded-state compatibility between the intrinsic folded-state spacing of the anchor residues and the finite geometric reach of the staple.

The strong agreement between our experimental measurements and T-REMD simulations suggests straightforward strategies for applying these guidelines prospectively. A useful workflow is: (1) simulate the non-stapled protein; (2) inspect Cβ–Cβ distances in both folded and unfolded ensembles, to identify sites that are close in the folded state yet widely separated in the unfolded state; and (3) match these sites to staples of an appropriate span, using the ∼14 Å effective reach of x4x as a reference point and extending the analysis to other linkers by analogous simulations. This simulation-guided approach offers a rational predictive framework for selecting both staple sites and staple lengths, reducing dependence on empirical screening. More broadly, the mechanistic principles of unfolded-state constraint and folded-state compatibility provide a general foundation for designing stabilizing linkers in diverse protein folds, potential applications in enhancing conformational stability, protease resistance, and biomolecular recognition in peptide- and protein-based therapeutics.

## Author contributions

S. H. performed stapling, purification, and circular dichroism experiments. S. H. and J. L. P. performed molecular dynamic simulations. All other authors assisted with synthesis of peptides. The manuscript was written and revised by S. H. and J. L. P. All authors have given approval to the final version of the manuscript.

## Conflicts of interest

There are no conflicts to declare.

## Supplementary Material

CB-007-D5CB00326A-s001

CB-007-D5CB00326A-s002

CB-007-D5CB00326A-s003

CB-007-D5CB00326A-s004

CB-007-D5CB00326A-s005

CB-007-D5CB00326A-s006

CB-007-D5CB00326A-s007

CB-007-D5CB00326A-s008

CB-007-D5CB00326A-s009

CB-007-D5CB00326A-s010

CB-007-D5CB00326A-s011

CB-007-D5CB00326A-s012

CB-007-D5CB00326A-s013

CB-007-D5CB00326A-s014

CB-007-D5CB00326A-s015

CB-007-D5CB00326A-s016

CB-007-D5CB00326A-s017

CB-007-D5CB00326A-s018

CB-007-D5CB00326A-s019

CB-007-D5CB00326A-s020

CB-007-D5CB00326A-s021

CB-007-D5CB00326A-s022

CB-007-D5CB00326A-s023

CB-007-D5CB00326A-s024

CB-007-D5CB00326A-s025

CB-007-D5CB00326A-s026

CB-007-D5CB00326A-s027

CB-007-D5CB00326A-s028

CB-007-D5CB00326A-s029

CB-007-D5CB00326A-s030

CB-007-D5CB00326A-s031

CB-007-D5CB00326A-s032

CB-007-D5CB00326A-s033

CB-007-D5CB00326A-s034

CB-007-D5CB00326A-s035

CB-007-D5CB00326A-s036

CB-007-D5CB00326A-s037

CB-007-D5CB00326A-s038

CB-007-D5CB00326A-s039

CB-007-D5CB00326A-s040

CB-007-D5CB00326A-s041

CB-007-D5CB00326A-s042

CB-007-D5CB00326A-s043

CB-007-D5CB00326A-s044

CB-007-D5CB00326A-s045

CB-007-D5CB00326A-s046

CB-007-D5CB00326A-s047

CB-007-D5CB00326A-s048

CB-007-D5CB00326A-s049

CB-007-D5CB00326A-s050

CB-007-D5CB00326A-s051

CB-007-D5CB00326A-s052

CB-007-D5CB00326A-s053

CB-007-D5CB00326A-s054

CB-007-D5CB00326A-s055

CB-007-D5CB00326A-s056

CB-007-D5CB00326A-s057

CB-007-D5CB00326A-s058

CB-007-D5CB00326A-s059

CB-007-D5CB00326A-s060

CB-007-D5CB00326A-s061

## Data Availability

The supporting data has been provided as part of the supplementary information (SI), which contains supporting figures, detailed and complete experimental procedures, compound purification and characterization, variable-temperature circular dichroism data, and molecular dynamic simulation data. Video files showing the last 200 ns of each of the 16 temperature-centric trajectories of each variant are available, as are initial PDB files for beginning T-REMD simulations on each variant (note that these PDB files are the starting non-minimized non-equilibrated models built as described in the SI). See DOI: https://doi.org/10.1039/d5cb00326a.
