# Supplementary material for: Folded-state compatibility and unfolded-state constraint govern staple-based stabilization: guidelines from a coiled-coil model
Source: RSC Chem Biol. 2026 Feb 24;7(4):737–50. doi: 10.1039/d5cb00326a (PMC12980645; doi:10.1039/d5cb00326a)
Supplement: CB-007-D5CB00326A-s025 [file CB-007-D5CB00326A-s025.pdf]

Supplementary Information (SI) for RSC Chemical Biology.
This journal is © The Royal Society of Chemistry 2026

Folded-State Compatibility and Unfolded-State Constraint Govern Staple-Based Stabilization: Guidelines from a Coiled-Coil Model

Samantha C. Hatfield, Alexa N. Mattingley, Kayla K. Sujeta, Logan D. Humphrey, Taylor Crook, Hiram Aranda, Christian H. Freckleton, Joseph V. Clayson, Chase Renstrom, Joshua L. Price*

Supplementary Information

Table of Contents

1. Structures of stapled and non-stapled coiled-coil variants....2
2. Synthesis of coiled-coil variants....7
3. Mass spectrometry data....9
4. Analytical HPLC data....23
5. Global fitting of variable temperature CD data....29
6. Building molecular dynamics models of stapled coiled-coil variants and their non-stapled counterparts....40
7. Molecular dynamics simulations of stapled coiled-coil variants and their non-stapled counterparts....42
8. Monitoring passage of coordinates through the sixteen temperature replicas: round trips....43
9. Median energy frames of stapled vs. non-stapled variants from each temperature-centric trajectory....84
10. Generating representative trajectory videos for each variant....104
11. Assessing the foldedness of stapled coiled-coils variants relative to their non-stapled counterparts in the T-REMD simulations: RMSD and residue packing distances....104
6e/1g'-xx....106
s6e/1g'-x4x....122
13e/8g'-xx....138
s13e/8g'-x4x....154
20e/15g'-xx....170
s20e/15g'-x4x....186
27e/22g'-xx....202
s27e/22g'-x4x....218
27e/22g'-z4x....234
s27e/22g'-z4x....250
34e/29g'-xx....266
s34e/29g'-x4x....282
7f/10b'-xx....298
s7f/10b'-x4x....314
14f/17b'-xx....330
s14f/17b'-x4x....346
21f/24b'-xx....362
s21f/24b'-x4x....378
28f/31b'-xx....395
s28f/31b'-x4x....411
12. Measuring Cβ-Cβ distances within stapled and non-stapled coiled-coils at sixteen temperatures....427
13. Fitting the RMSD and residue packing distances to obtain T_m and ΔG for each variant....448
14. Calculating the difference in RMSF for each atom in the 310-K trajectories of each stapled variant relative to its non-stapled counterpart....460
15. Correlation between simulated and observed values of melting temperatures and stabilization of stapled variants compared to their non-stapled counterparts....460

1. Structures of stapled and non-stapled coiled-coil variants

```
        1    5     10    15    20    25    30    35
        gabcdefgabcdefgabcdefgabcdefgabcdef
6e      AcNH-EVKQLXAEVEELESELWHLENEVARLEKENAECEA-CONH2   (HA10051)
1g'     AcNH-XVKQLKAKVEELKSKLWHLKNKVARLKKKNAECKA-CONH2   (HA10052)

6e/1g'  AcNH-EVKQLXAEVEELESELWHLENEVARLEKENAECEA-CONH2   (SH2017)
                                              S
                                              S
        AcNH-XVKQLKAKVEELKSKLWHLKNKVARLKKKNAECKA-CONH2

s6e/1g' AcNH-EVKQLXAEVEELESELWHLENEVARLEKENAECEA-CONH2   (SH2019)
                                              S
                                              S
        AcNH-XVKQLKAKVEELKSKLWHLKNKVARLKKKNAECKA-CONH2
```

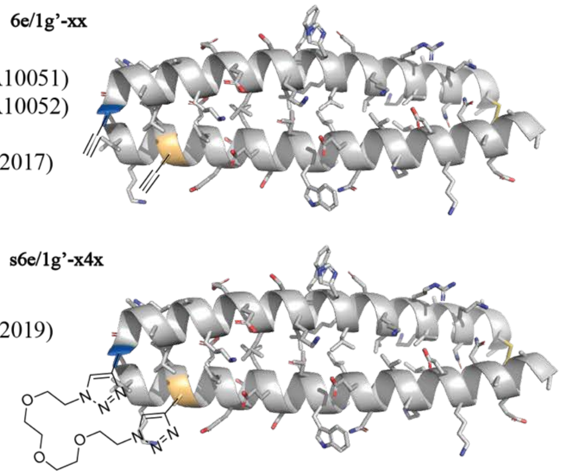

Figure S1. Sequences of (A) acidic monomer **6e-x**, basic monomer **1g'-x**, disulfide-bound coiled-coil heterodimer **6e/1g'-xx**, and its stapled counterpart **s6e/1g'-x4x**. **X** represents propargyl glycine. Also shown are the structures of the side chain of **X** and their stapled counterpart superimposed on the ribbon diagram of parent disulfide-bonded heterodimer **A/B** (PDB ID: 1KD9). Side chains are shown as sticks; position 6e is highlighted in orange, whereas position 1g' is highlighted in blue. The PEG oligomer(s) within each variant have the number of ethylene oxide units indicated in the structural drawings and in the compound names. Numbers in parentheses indicate notebook numbers associated with each compound.

```
         1    5     10    15    20    25    30    35
         gabcdefgabcdefgabcdefgabcdefgabcdef
13e      AcNH-EVKQLEAEVEELXSELWHLENEVARLEKENAECEA-CONH2   (SH10341)
8g'      AcNH-KVKQLKAXVEELKSKLWHLKNKVARLKKKNAECKA-CONH2   (SH10342)

13e/8g'  AcNH-EVKQLEAEVEELXSELWHLENEVARLEKENAECEA-CONH2   (SH2016)
                                               S
                                               S
         AcNH-KVKQLKAXVEELKSKLWHLKNKVARLKKKNAECKA-CONH2   (SH2018)

s13e/8g' AcNH-EVKQLEAEVEELXSELWHLENEVARLEKENAECEA-CONH2
                                               S
                                               S
         AcNH-KVKQLKAXVEELKSKLWHLKNKVARLKKKNAECKA-CONH2
```

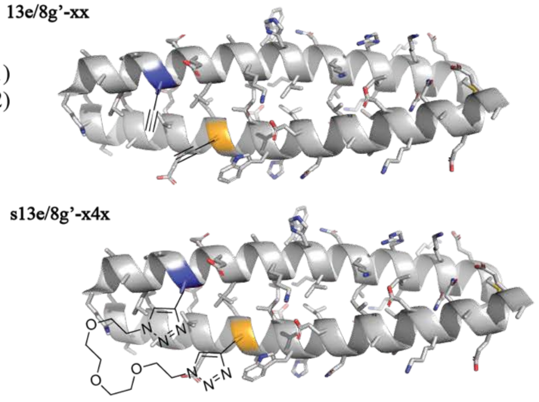

Figure S2. Sequences of (A) acidic monomer **13e-x**, basic monomer **8g'-x**, disulfide-bound coiled-coil heterodimer **13e/8g'-xx**, and its stapled counterpart **s13e/8g'-x4x**. **X** represents propargyl glycine. Also shown are the structures of the side chain of **X** and their stapled counterpart superimposed on the ribbon diagram of parent disulfide-bonded heterodimer **A/B** (PDB ID: 1KD9). Side chains are shown as sticks; position 13e is highlighted in orange, whereas position 8g' is highlighted in blue. The PEG oligomer(s) within each variant have the number of ethylene oxide units indicated in the structural drawings and in the compound names. Numbers in parentheses indicate notebook numbers associated with each compound.

```
        1    5     10    15    20    25    30    35
        gabcdefgabcdefgabcdefgabcdefgabcdef
20e      AcNH-EVKQLEAEVEELESELWHLXNEVARLEKENAECEA-CONH2   (HF1021)
15g'     AcNH-KVKQLKAKVEELKSXLWHLKNKVARLKKKNAECKA-CONH2   (ZB1005)

20e/15g' AcNH-EVKQLEAEVEELESELWHLXNEVARLEKENAECEA-CONH2   (SH2036)
                                              S-S
         AcNH-KVKQLKAKVEELKSXLWHLKNKVARLKKKNAECKA-CONH2

s20e/15g' AcNH-EVKQLEAEVEELESELWHLXNEVARLEKENAECEA-CONH2  (SH2037)
                                              S-S
          AcNH-KVKQLKAKVEELKSXLWHLKNKVARLKKKNAECKA-CONH2
```

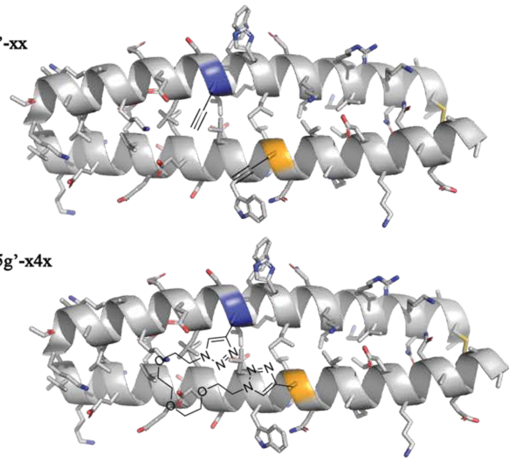

Figure S3. Sequences of (A) acidic monomer **20e-x**, basic monomer **15g'-x**, disulfide-bound coiled-coil heterodimer **20e/15g'-xx**, and its stapled counterpart **s20e/15g'-x4x**. **X** represents propargyl glycine. Also shown are the structures of the side chain of **X** and their stapled counterpart superimposed on the ribbon diagram of parent disulfide-bonded heterodimer **A/B** (PDB ID: 1KD9). Side chains are shown as sticks; position 20e is highlighted in orange, whereas position 15g' is highlighted in blue. The PEG oligomer(s) within each variant have the number of ethylene oxide units indicated in the structural drawings and in the compound names. Numbers in parentheses indicate notebook numbers associated with each compound.

```
        1    5     10    15    20    25    30    35
        gabcdefgabcdefgabcdefgabcdefgabcdef
27e      AcNH-EVKQLEAEVEELESELWHLENEVARLXKENAECEA-CONH2   (SH10343)
22g'     AcNH-KVKQLKAKVEELKSKLWHLKNXVARLKKKNAECKA-CONH2   (SH10344)

27e/22g' AcNH-EVKQLEAEVEELESELWHLENEVARLXKENAECEA-CONH2   (SH2024)
                                              S-S
         AcNH-KVKQLKAKVEELKSKLWHLKNXVARLKKKNAECKA-CONH2

s27e/22g' AcNH-EVKQLEAEVEELESELWHLENEVARLXKENAECEA-CONH2  (SH2027)
                                              S-S
          AcNH-KVKQLKAKVEELKSKLWHLKNXVARLKKKNAECKA-CONH2
```

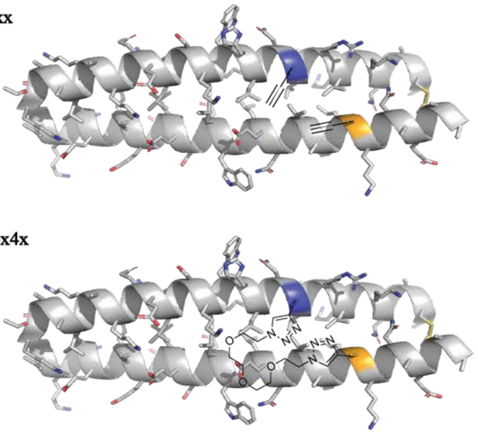

Figure S4. Sequences of (A) acidic monomer **27e-x**, basic monomer **22g'-x**, disulfide-bound coiled-coil heterodimer **27e/22g'-xx**, and its stapled counterpart **s27e/22g'-x4x**. **X** represents propargyl glycine. Also shown are the structures of the side chain of **X** and their stapled counterpart superimposed on the ribbon diagram of parent disulfide-bonded heterodimer **A/B** (PDB ID: 1KD9). Side chains are shown as sticks; position 27e is highlighted in orange, whereas position **22g'** is highlighted in blue. The PEG oligomer(s) within each variant have the number of ethylene oxide units indicated in the structural drawings and in the compound names. Numbers in parentheses indicate notebook numbers associated with each compound.

```
        1    5     10    15    20    25    30    35
        gabcdefgabcdefgabcdefgabcdefgabcdef
34e     AcNH-EVKQLEAEVEELESELWHLENEVARLEKENAECXA-CONH2   (TC10391)
29g'    AcNH-KVKQLKAKVEELKSKLWHLKNKVARLKKXNAECKA-CONH2   (TC10392)

34e/29g'  AcNH-EVKQLEAEVEELESELWHLENEVARLEKENAECXA-CONH2   (SH2021)
                                                 S-S
          AcNH-KVKQLKAKVEELKSKLWHLKNKVARLKKXNAECKA-CONH2

s34e/29g' AcNH-EVKQLEAEVEELESELWHLENEVARLEKENAECXA-CONH2   (SH2023)
                                                 S-S
          AcNH-KVKQLKAKVEELKSKLWHLKNKVARLKKXNAECKA-CONH2
```

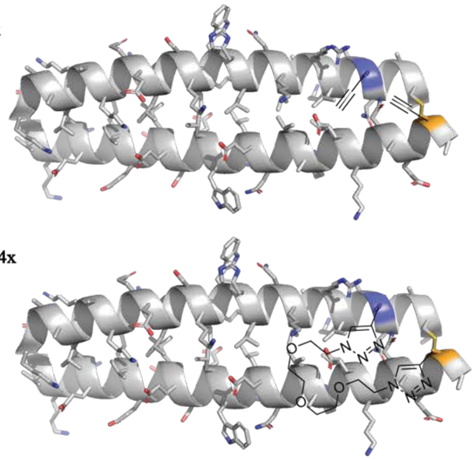

Figure S5. Sequences of (A) acidic monomer **34e-x**, basic monomer **29g'-x**, disulfide-bound coiled-coil heterodimer **34e/29g'-xx**, and its stapled counterpart **s34e/29g'-x4x**. **X** represents propargyl glycine. Also shown are the structures of the side chain of **X** and their stapled counterpart superimposed on the ribbon diagram of parent disulfide-bonded heterodimer **A/B** (PDB ID: 1KD9). Side chains are shown as sticks; position 34e is highlighted in orange, whereas position 29g' is highlighted in blue. The PEG oligomer(s) within each variant have the number of ethylene oxide units indicated in the structural drawings and in the compound names. Numbers in parentheses indicate notebook numbers associated with each compound.

```
        1    5     10    15    20    25    30    35
        gabcdefgabcdefgabcdefgabcdefgabcdef
7f      AcNH-EVKQLEXEVEELESELWHLENEVARLEKENAECEA-CONH2   (ZJ10673)
10b'    AcNH-KVKQLKAKVXELKSKLWHLKNKVARLKKKNAECKA-CONH2   (ZJ10674)

7f/10b'   AcNH-EVKQLEXEVEELESELWHLENEVARLEKENAECEA-CONH2   (LH1029)
                                                 S-S
          AcNH-KVKQLKAKVXELKSKLWHLKNKVARLKKKNAECKA-CONH2

s7f/10b'  AcNH-EVKQLEXEVEELESELWHLENEVARLEKENAECEA-CONH2   (SH1119)
                                                 S-S
          AcNH-KVKQLKAKVXELKSKLWHLKNKVARLKKKNAECKA-CONH2
```

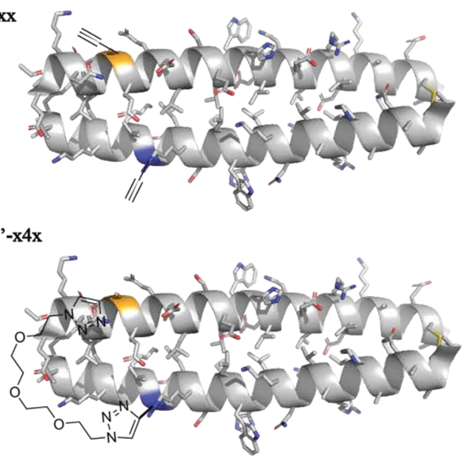

Figure S6. Sequences of (A) acidic monomer **7f-x**, basic monomer **10b'-x**, disulfide-bound coiled-coil heterodimer **7f/10b'-xx**, and its stapled counterpart **s7f/10b'-x4x**. **X** represents propargyl glycine. Also shown are the structures of the side chain of **X** and their stapled counterpart superimposed on the ribbon diagram of parent disulfide-bonded heterodimer **A/B** (PDB ID: 1KD9). Side chains are shown as sticks; position 7f is highlighted in orange, whereas position 10b' is highlighted in blue. The PEG oligomer(s) within each variant have the number of ethylene oxide units indicated in the structural drawings and in the compound names. Numbers in parentheses indicate notebook numbers associated with each compound.

```
              1    5     10    15    20    25    30    35
             gabcdefgabcdefgabcdefgabcdefgabcdef
14f          AcNH-EVKQLEAEVEELEXELWHLENEVARLEKENAECEA-CONH2   (AM1029)
17b'         AcNH-KVKQLKAKVEELKSKLXHLKNKVARLKKKNAECKA-CONH2   (KS1004)

14f/17b'     AcNH-EVKQLEAEVEELEXELWHLENEVARLEKENAECEA-CONH2   (SH2029)
                                                    \
                                                     S
                                                    /
                                                   S
                                                    \
             AcNH-KVKQLKAKVEELKSKLWHLKNKVARLKKKNAECKA-CONH2

s14f/17b'    AcNH-EVKQLEAEVEELEXELWHLENEVARLEKENAECEA-CONH2
                                                    \
                                                     S
                                                    /
                                                   S
                                                    \
             AcNH-KVKQLKAKVEELKSKLXHLKNKVARLKKKNAECKA-CONH2
```

14f/17b'-xx

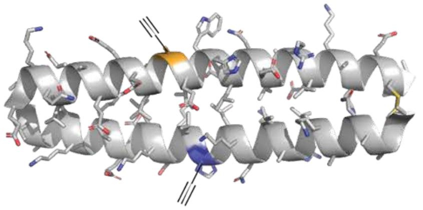

s14f/17b'-x4x

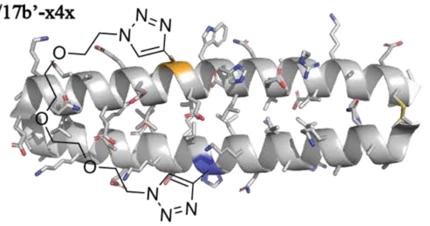

Figure S7. Sequences of (A) acidic monomer **14f-x**, basic monomer **17b'-x**, disulfide-bound coiled-coil heterodimer **14f/17b'-xx**, and its stapled counterpart **s14f/17b'-x4x**. **X** represents propargyl glycine. Also shown are the structures of the side chain of **X** and their stapled counterpart superimposed on the ribbon diagram of parent disulfide-bonded heterodimer **A/B** (PDB ID: 1KD9). Side chains are shown as sticks; position 14f is highlighted in orange, whereas position 17b' is highlighted in blue. The PEG oligomer(s) within each variant have the number of ethylene oxide units indicated in the structural drawings and in the compound names. Numbers in parentheses indicate notebook numbers associated with each compound.

```
              1    5     10    15    20    25    30    35
             gabcdefgabcdefgabcdefgabcdefgabcdef
21f          AcNH-EVKQLEAEVEELESELWHLEXEVARLEKENAECEA-CONH2   (HA10091)
24b'         AcNH-KVKQLKAKVEELKSKLWHLKNKVXRLKKKNAECKA-CONH2   (HA10092)

21f/24b'     AcNH-EVKQLEAEVEELESELWHLEXEVARLEKENAECEA-CONH2   (SH2012)
                                                    \
                                                     S
                                                    /
                                                   S
                                                    \
             AcNH-KVKQLKAKVEELKSKLWHLKNKVXRLKKKNAECKA-CONH2

s21f/24b'    AcNH-EVKQLEAEVEELESELWHLEXEVARLEKENAECEA-CONH2   (SH2014)
                                                    \
                                                     S
                                                    /
                                                   S
                                                    \
             AcNH-KVKQLKAKVEELKSKLWHLKNKVXRLKKKNAECKA-CONH2
```

21f/24b'-xx

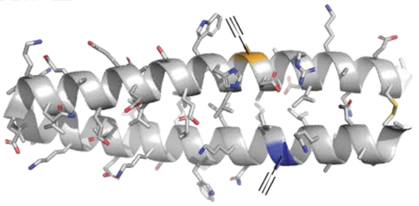

s21f/24b'-x4x

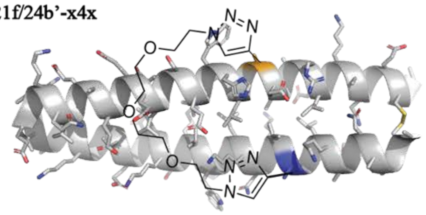

Figure S8. Sequences of (A) acidic monomer **21f-x**, basic monomer **24b'-x**, disulfide-bound coiled-coil heterodimer **21f/24b'-xx**, and its stapled counterpart **s21f/24b'-x4x**. **X** represents propargyl glycine. Also shown are the structures of the side chain of **X** and their stapled counterpart superimposed on the ribbon diagram of parent disulfide-bonded heterodimer **A/B** (PDB ID: 1KD9). Side chains are shown as sticks; position 21f is highlighted in orange, whereas position 24b' is highlighted in blue. The PEG oligomer(s) within each variant have the number of ethylene oxide units indicated in the structural drawings and in the compound names. Numbers in parentheses indicate notebook numbers associated with each compound.

1 5 10 15 20 25 30 35
gabcdefgabcdefgabcdefgabcdefgabcdef

28f/31b'-xx

28f AcNH-EVKQLEAEVEELESELWHLENEVARLE**X**ENAE**C**EA-CONH$_2$ (HA10131/SH2033)

31b' AcNH-KVKQLKAKVEELKSKLWHLKNKVARLKKKN**X**E**C**KA-CONH$_2$ (HA10132/AM1033)

28f/31b' AcNH-EVKQLEAEVEELESELWHLENEVARLE**X**ENAE**C**EA-CONH$_2$ (SH2025)
S
S
AcNH-KVKQLKAKVEELKSKLWHLKNKVARLKKKN**X**E**C**KA-CONH$_2$

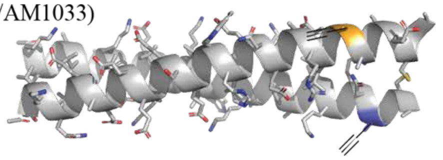

s28f/31b'-x4x

s28f/31b' AcNH-EVKQLEAEVEELESELWHLENEVARLE**X**ENAE**C**EA-CONH$_2$ (SH2039)
S
S
AcNH-KVKQLKAKVEELKSKLWHLKNKVARLKKKN**X**E**C**KA-CONH$_2$

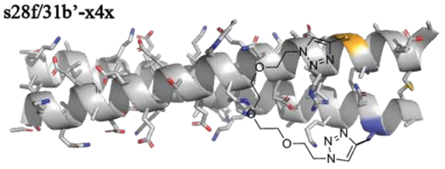

Figure S9. Sequences of (A) acidic monomer **28f-x**, basic monomer **31b'-x**, disulfide-bound coiled-coil heterodimer **28f/31b'-xx**, and its stapled counterpart **s28f/31b'-x4x**. **X** represents propargyl glycine. Also shown are the structures of the side chain of **X** and their stapled counterpart superimposed on the ribbon diagram of parent disulfide-bonded heterodimer **A/B** (PDB ID: 1KD9). Side chains are shown as sticks; position 28f is highlighted in orange, whereas position 31b' is highlighted in blue. The PEG oligomer(s) within each variant have the number of ethylene oxide units indicated in the structural drawings and in the compound names. Numbers in parentheses indicate notebook numbers associated with each compound.

(return to Table of Contents)

2. Synthesis of coiled-coil variants

Peptide Synthesis: Monomers **6*e*-x** (HA10051); **1*g*'-x** (HA10052); **13*e*-x** (SH10341); **8*g*'-x** (SH10342); **20*e*-x** (HF1021); **15*g*'-x** (ZB1005); **27*e*-x** (SH10343); **22*g*'-x** (SH10344); **34*e*-x** (TC10391); **29*g*'-x** (TC10392); **7*f*'-x** (ZJ10673); **10*b*'-x** (ZJ10674); **14*f*-x** (AM1029); **17*b*'-x** (KS1004); **21*f*-x** (HA10091); **24*b*'-x** (HA10092); **28*f*-x** (HA10131/SH2033); **31*b*'-x** (HA10132/AM1033) were prepared as C-terminal amides on Rink amide MBHA LL resin (EMD Biosciences), by microwave-assisted solid phase peptide synthesis using a standard Fmoc Nα protection strategy as described previously,[2] and using room-temperature synthesis. Room temperature synthesis used a standard Fmoc Nα protection strategy with coupling procedure lasting 15 minutes and deprotection being 20 minutes. Fmoc-protected amino acids were purchased from Advanced Chem Tech. Peptides were cleaved from resin and purified by preparative reverse-phase high-performance liquid chromatography (HPLC) on a C18 column using a linear gradient of water in acetonitrile with 0.1% v/v trifluoroacetic acid. Peptide identity was confirmed by electrospray ionization time- of-flight mass spectrometry.

Disulfide formation in the coiled-coil heterodimer: We prepared disulfide-bonded coiled-coil heterodimers **6*e*/1*g*'-x4x** (SH2017); **13*e*/8*g*'-x4x** (SH2016); **20*e*/15*g*'-x4x** (SH2036); **d27*e*/22*g*'-x4x** (SH2024); **34*e*/29*g*'-x4x** (SH2021); **7*f*/10*b*'-x4x** (SH1115); **14*f*/17*b*'-x4x** (SH2029); **21*f*/24*b*'-x4x** (SH2012); **28*f*/31*b*'-x4x** (SH2025) by mixing their purified cysteine-containing precursors in a 1:1 ratio in an aqueous solution of ammonium bicarbonate (8 mg/mL) with exposure to air for 1-3 hrs. Reaction completeness was monitored by analytical HPLC. Disulfide-bonded coiled-coil heterodimers were then purified by preparative HPLC and characterized by ESI-TOF MS.

Two-component stapling via copper-catalyzed azide/alkyne cycloaddition (CuAAC): We prepared triazole-stapled variants **s6*e*/1*g*'-x4x** (SH2019); **s13*e*/8*g*'-x4x** (SH2018); **s20*e*/15*g*'-x4x** (SH2037); **s27*e*/22*g*'-x4x** (SH2027); **s34*e*/29*g*'-x4x** (SH2023); **s7*f*/10*b*'-x4x** (SH1119); **s21*f*/24*b*'-x4x** (SH2014); **s28*f*/31*b*'-x4x** (SH2039) by combining the non-stapled precursors (heterodimers) respectively, in 10:3 (v/v) water/*tert*-butanol with 1.5 eq. of previously synthesized four-unit bis-azido PEG **4**[1], referred to as **x4x** when stapled (Figure S10), in 6.4 eq. copper sulfate pentahydrate and 7.7 eq. sodium ascorbate at 39.5 °C for 30 minutes to an hour. The reaction was monitored by analytical HPLC, where we observed small changes in retention time upon stapling. The triazole-stapled variants were purified via preparative HPLC.

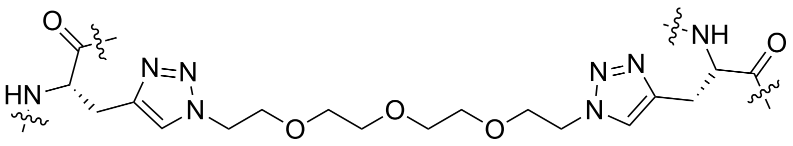

Figure S10. Drawing of staple **x4x**.

Analytical reduction of disulfide bond in stapled variants to confirm the presence of the staple: After purifying each stapled variant, we confirmed the presence of the staple by subjecting a small aliquot of the stapled variant to reduction tris(2-carboxyethyl) phosphine hydrocholoride (purchased from Combi-Blocks). Five eq. of TCEP was added to peptide and heated to 39.5 °C for an hour. Reduced peptide was analyzed by ESI-TOF MS to confirm the presence of the intact reduced stapled heterodimer, which differs from the disulfide-bound stapled heterodimer by two mass units, corresponding to the addition of two hydrogen atoms in the reduction reaction.

(return to Table of Contents)

3. Mass spectrometry data

Mass spectra for monomers, disulfide-bound non-stapled heterodimers, and disulfide-bound stapled heterodimers and TCEP reduced stapled dimers (to confirm that the staple is intact and that the coiled-coil remains a covalent monomer even in the absence of the disulfide bond) appear in Figures S11–S55; data are summarized in Table S1.

Table S1. Summary of the mass spectrum data for stapled disulfide-bonded coiled-coil heterodimers, their non-stapled counterparts, and their monomer components.

Name	Notebook Number	Molecular Formula	z	Expected [M+z·H]/z	Observed [M+z·H]/z
**6e-x**	HA10051	$C_{178}H_{277}N_{46}O_{64}S$	4	1033.752	1033.7552
**1g'-x**	HA10052	$C_{187}H_{322}N_{55}O_{46}S$	4	1031.620	1031.6164
**6e/1g'-xx**	SH2017	$C_{365}H_{599}N_{101}O_{110}S_2$	8	1032.434	1032.430
**s6e/1g'-x4x**	SH2019	$C_{373}H_{615}N_{108}O_{113}S_2$	8	1062.950	1062.9495
**13e-x**	SH10341	$C_{178}H_{277}N_{46}O_{64}S$	4	1033.752	1033.7502
**8g'-x**	SH10342	$C_{187}H_{322}N_{55}O_{46}S$	4	1031.620	1031.6173
**13e/8g'-xx**	SH2016	$C_{365}H_{599}N_{101}O_{110}S_2$	8	1032.434	1032.4309
**s13e/8g'-x4x**	SH2018	$C_{373}H_{615}N_{108}O_{113}S_2$	8	1062.950	1062.9521
**20e-x**	HF1021	$C_{178}H_{277}N_{46}O_{64}S$	4	1033.752	1033.7477
**15g'-x**	ZB1005	$C_{187}H_{322}N_{55}O_{46}S$	4	1031.620	1031.6157
**20e/15g'-xx**	SH2036	$C_{365}H_{599}N_{101}O_{110}S_2$	8	1032.434	1032.4334
**s20e/15g'-x4x**	SH2037	$C_{373}H_{615}N_{108}O_{113}S_2$	8	1062.950	1062.9527
**27e-x**	SH10343	$C_{178}H_{277}N_{46}O_{64}S$	4	1033.752	1033.7705
**22g'-x**	SH10344	$C_{187}H_{322}N_{55}O_{46}S$	4	1031.620	1031.6174
**27e/22g'-xx**	SH2024	$C_{365}H_{599}N_{101}O_{110}S_2$	8	1032.434	1032.4338
**s27e/22g'-x4x**	SH2027	$C_{373}H_{615}N_{108}O_{113}S_2$	8	1062.950	1062.9525
**34e-x**	TC10391	$C_{178}H_{277}N_{46}O_{64}S$	4	1033.752	1033.7454
**29g'-x**	TC10392	$C_{187}H_{322}N_{55}O_{46}S$	4	1031.620	1031.6138
**34e/29g'-xx**	SH2021	$C_{365}H_{599}N_{101}O_{110}S_2$	8	1032.434	1032.4365
**s34e/29g'-x4x**	SH2023	$C_{373}H_{615}N_{108}O_{113}S_2$	8	1062.950	1062.9514
**7f-x**	ZJ10673	$C_{180}H_{279}N_{46}O_{66}S$	4	1048.253	1048.2493
**10b'-x**	ZJ10674	$C_{188}H_{327}N_{56}O_{44}S$	4	1031.383	1031.3676
**7f/10b'-xx**	LH1029	$C_{368}H_{606}N_{102}O_{110}S_2$	8	1039.566	1039.5613
**s7f/10b'-x4x**	SH1119	$C_{376}H_{622}N_{109}O_{113}S_2$	8	1070.082	1070.0847
**14f-x**	AM1029	$C_{180}H_{279}N_{46}O_{65}S$	4	1044.254	1044.2504
**17b'-x**	KS1004	$C_{182}H_{324}N_{55}O_{46}S$	4	1017.123	1017.1161
**14f/17b'-xx**	SH2029	$C_{362}H_{603}N_{101}O_{111}S_2$	8	1030.437	1030.4346
**21f-x**	HA10091	$C_{179}H_{278}N_{45}O_{65}S$	4	1037.502	1037.4933
**24b'-x**	HA10092	$C_{190}H_{329}N_{56}O_{46}S$	4	1045.884	1045.8624
**21f/24b'-xx**	SH2012	$C_{369}H_{607}N_{101}O_{111}S_2$	8	1041.441	1041.4443
**s21f/24b'-x4x**	SH2014	$C_{377}H_{623}N_{108}O_{114}S_2$	8	1071.957	1071.9687
**28f-x**	HA10131	$C_{177}H_{272}N_{45}O_{66}S$	4	1033.989	1033.9875
**28f-x**	SH2033			1033.989	1033.9946
**31b'-x**	HA10132	$C_{190}H_{329}N_{56}O_{46}S$	4	1045.884	1045.8679
**31b'-x**	AM1033			1045.8930	1045.8930
**28f/31b'-xx**	SH2025	$C_{367}H_{601}N_{101}O_{112}S_2$	8	1039.684	1039.6835
**s28f/31b'-x4x**		$C_{375}H_{617}N_{108}O_{115}S_2$	8	1070.200	1070.2129

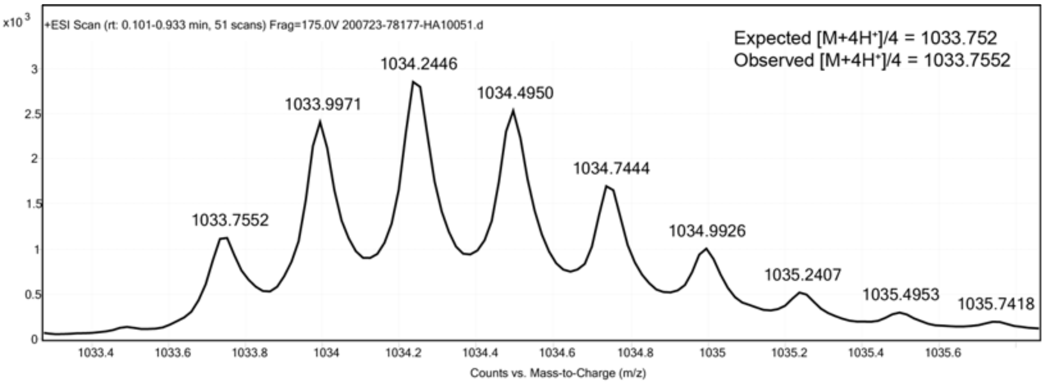

Figure S11. ESI-TOF spectrum for acidic monomer **6e-x** (HA10051, $C_{178}H_{277}N_{46}O_{64}S$). Expected $[M+4H^{+}]/4$ = 1033.752 Da. Observed $[M+4H^{+}]/4$ = 1033.7552 Da.

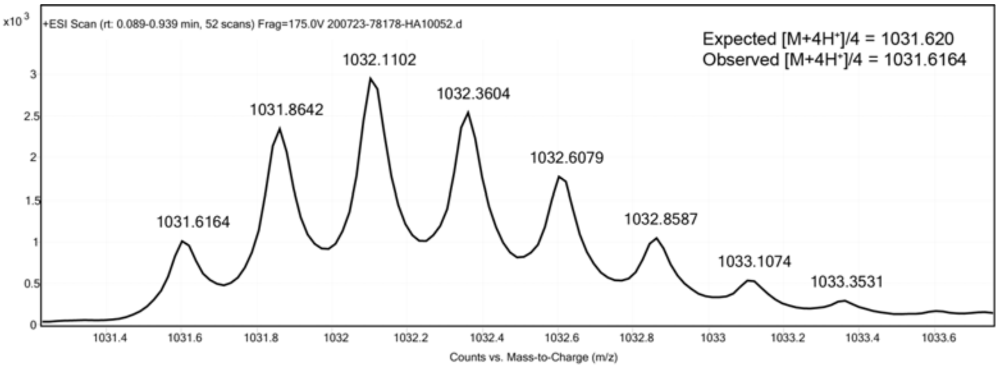

Figure S12. ESI-TOF spectrum for basic monomer **1g'**-x (HA10052, $C_{187}H_{322}N_{55}O_{46}S$). Expected $[M+4H^{+}]/4$ = 1031.620 Da. Observed $[M+4H^{+}]/4$ = 1031.6164 Da.

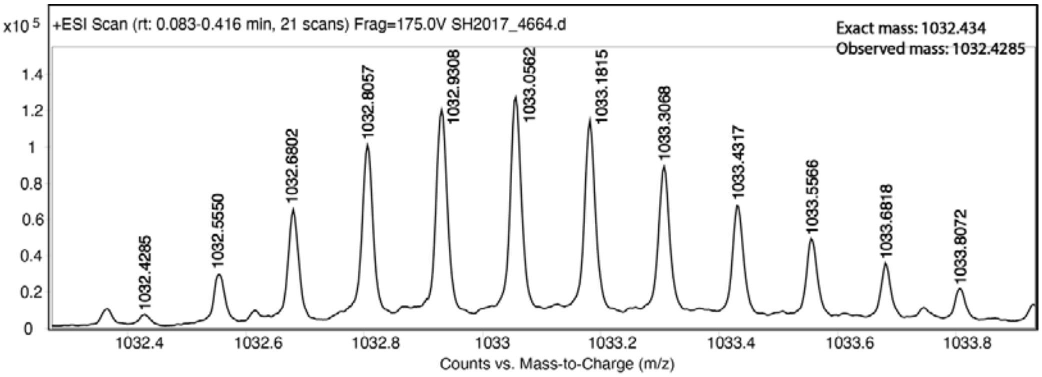

Figure S13. ESI-TOF spectrum for disulfide-bound coiled-coil variant **6e/1g'-xx** (SH2017, $C_{365}H_{599}N_{102}O_{110}S_{2}$). Expected $[M+8H^{+}]/8$ = 1032.434 Da. Observed $[M+4H^{+}]/4$ = 1032.4285 Da.

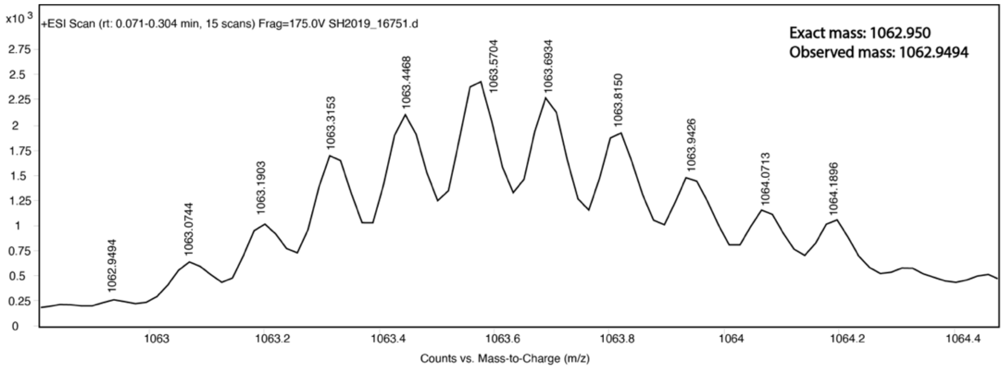

Figure S14. ESI-TOF spectrum for stapled disulfide-bound coiled-coil variant **s6e/1g'-z4x** (SH2019, $C_{373}H_{615}N_{108}O_{113}S_{2}$). Expected $[M+8H^{+}]/8$ = 1062.950 Da. Observed $[M+8H^{+}]/8$ = 1062.9494 Da.

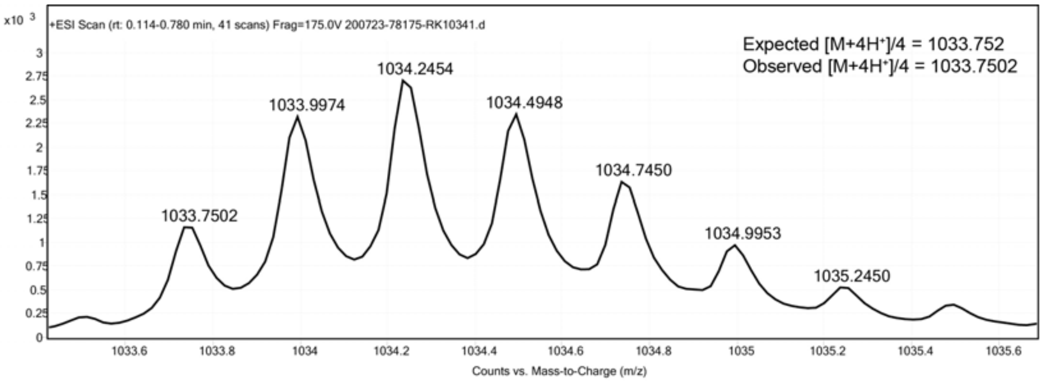

Figure S15. ESI-TOF spectrum for acidic monomer **13e-x** (SH10341; $C_{178}H_{277}N_{46}O_{66}S$; expected $[M+4H^{+}]/4$ = 1033.752 Da. Observed $[M+4H^{+}]/4$ = 1033.7502 Da.

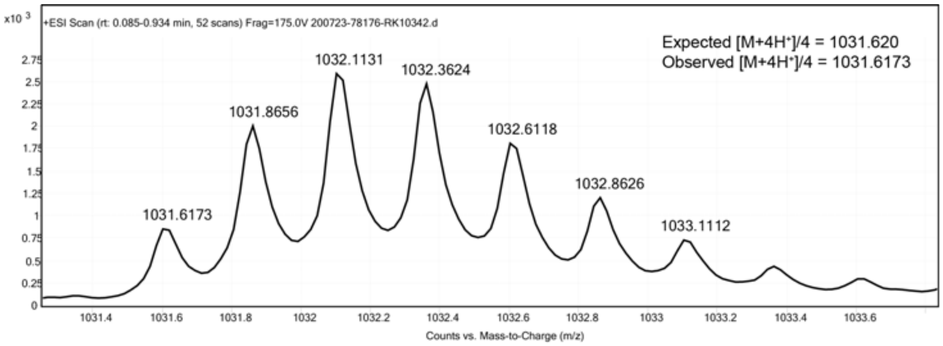

Figure S16. ESI-TOF spectrum for basic variant **8g'-x** (SH10342; $C_{187}H_{322}N_{55}O_{46}S$). Expected $[M+4H^{+}]/4$ = 1031.620 Da. Observed $[M+4H^{+}]/4$ = 1031.6173 Da.

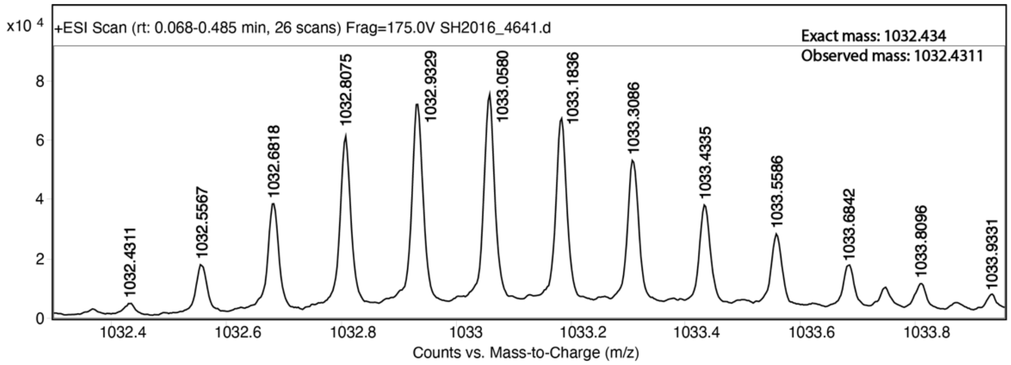

Figure S17. ESI-TOF spectrum for disulfide-bound coiled-coil variant **13e/8g'-xx** (SH2016; $C_{365}H_{599}N_{101}O_{110}S_2$). Expected $[M+8H^{+}]/8$ = 1032.434 Da. Observed $[M+8H^{+}]/8$ = 1032.4311 Da.

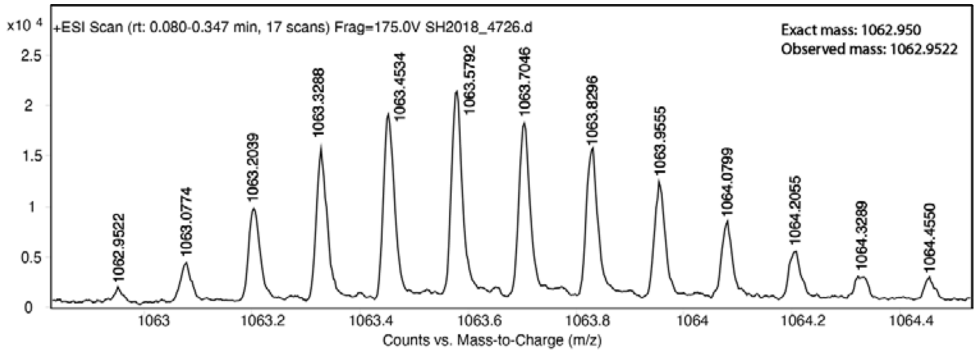

Figure S18. ESI-TOF spectrum for stapled disulfide-bound coiled-coil variant **s13e/8g'-x4x** (SH2018; $C_{373}H_{615}N_{108}O_{113}S_2$). Expected $[M+8H^{+}]/8$ = 1062.950 Da. Observed $[M+8H^{+}]/8$ = 1062.9522 Da.

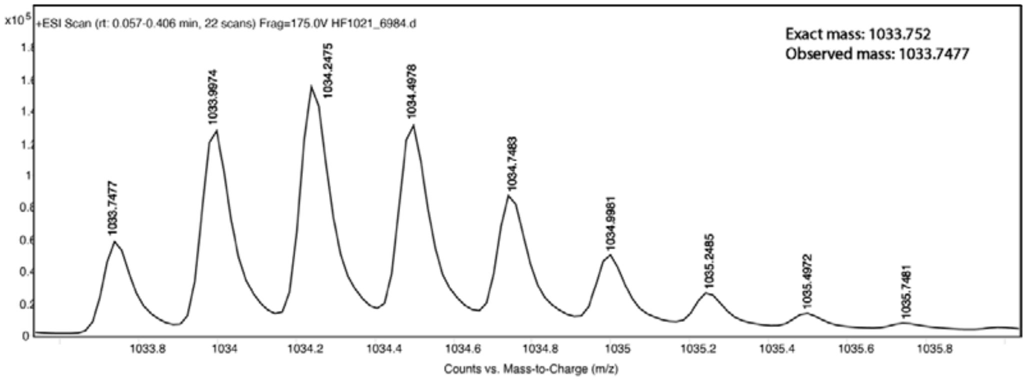

Figure S19. ESI-TOF spectrum for acidic monomer **20e-x** (HF1021, $C_{178}H_{277}N_{46}O_{64}S$). Expected $[M+4H^{+}]/4 = 1033.752$ Da. Observed $[M+4H^{+}]/4 = 1033.7477$ Da.

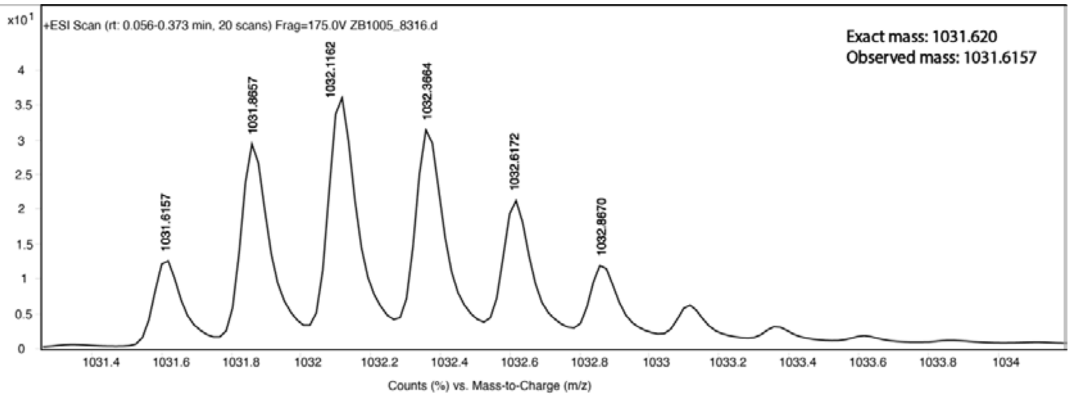

Figure S20. ESI-TOF spectrum for basic monomer **15g'-x** (ZB1005, $C_{187}H_{322}N_{55}O_{46}S$). Expected $[M+4H^{+}]/4 = 1031.620$ Da. Observed $[M+4H^{+}]/4 = 1031.6157$ Da.

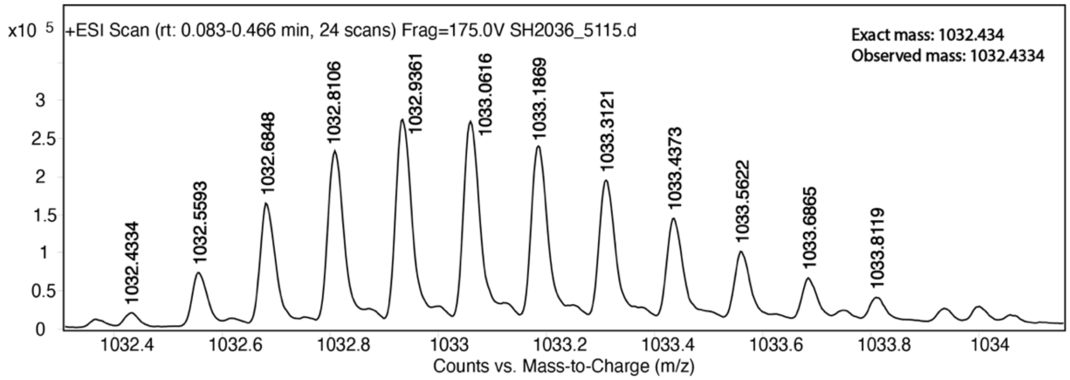

Figure S21. ESI-TOF spectrum for disulfide-bound coiled-coil variant **20e/15g'-xx** (SH2036, $C_{365}H_{599}N_{101}O_{110}S_2$). Expected $[M+8H^{+}]/8 = 1032.434$ Da. Observed $[M+8H^{+}]/8 = 1032.4334$ Da.

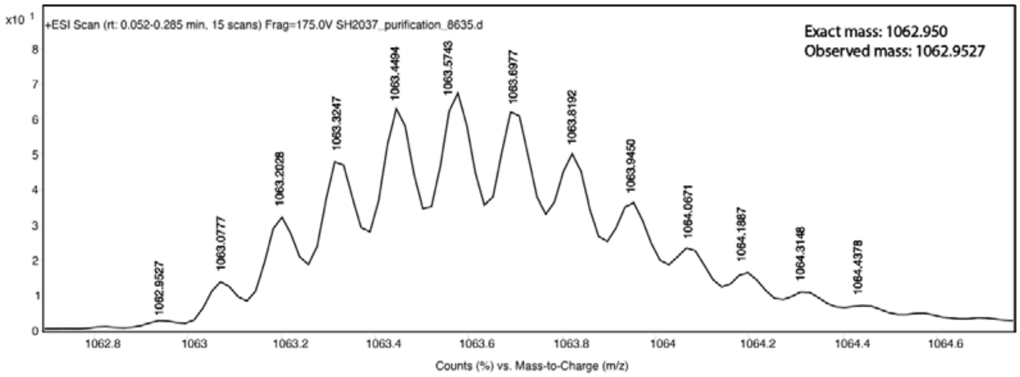

Figure S22. ESI-TOF spectrum for stapled disulfide-bound coiled-coil variant **s20e/15g'-x4x** (2037, $C_{372}H_{617}N_{107}O_{114}S_2$). Expected $[M+8H^{+}]/8 = 1062.950$ Da. Observed $[M+8H^{+}]/8 = 1062.9527$ Da.

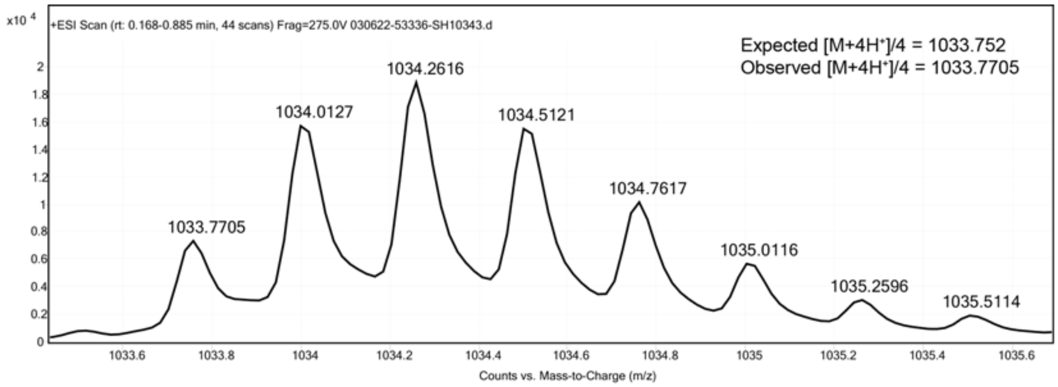

Figure S23. ESI-TOF spectrum for acidic monomer **27e-x** (SH10343, $C_{178}H_{277}N_{46}O_{64}S$). Expected $[M+4H^{+}]/4$ = 1033.752 Da. Observed $[M+4H^{+}]/4$ = 1033.7705 Da.

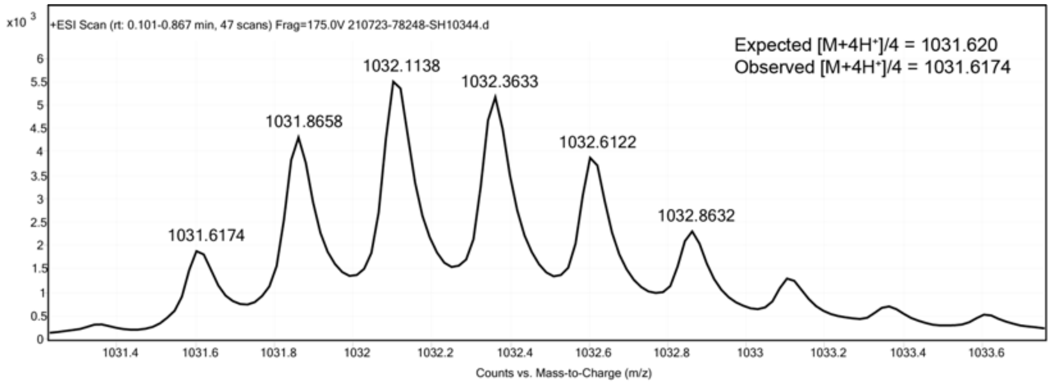

Figure S24. ESI-TOF spectrum for basic monomer **22g'-x** (SH10344, $C_{187}H_{322}N_{55}O_{46}S$). Expected $[M+4H^{+}]/4$ = 1031.620 Da. Observed $[M+4H^{+}]/4$ = 1031.6174 Da.

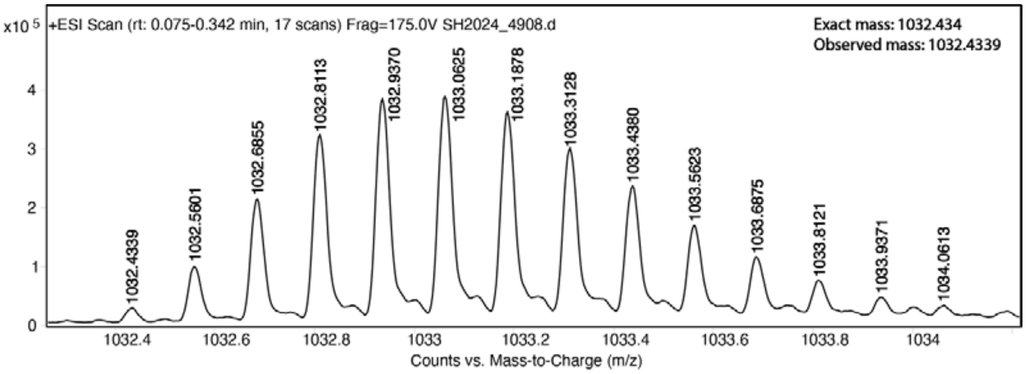

Figure S25. ESI-TOF spectrum for disulfide-bound coiled-coil variant **27e/22g'-xx** (SH2024, $C_{365}H_{599}N_{101}O_{110}S_2$). Expected $[M+8H^{+}]/8$ = 1032.434 Da. Observed $[M+4H^{+}]/4$ = 1032.4339 Da.

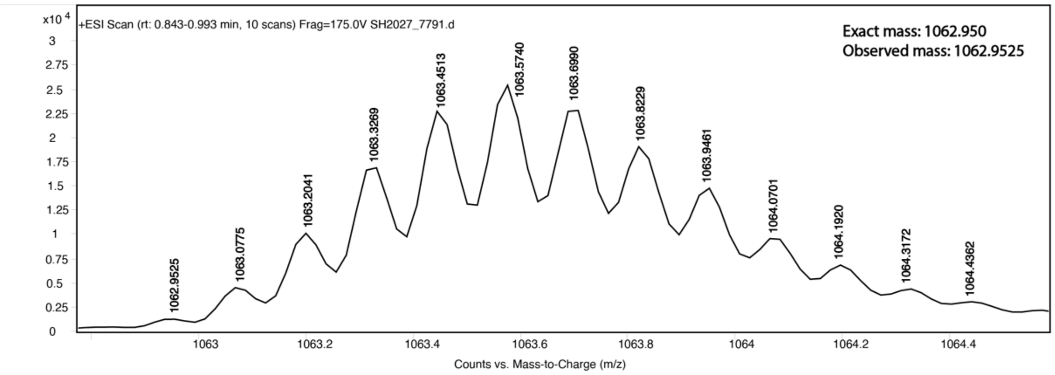

Figure S26. ESI-TOF spectrum for stapled disulfide-bound coiled-coil variant **s27e/22g'-x4x** (SH2027, $C_{373}H_{615}N_{108}O_{113}S_2$). Expected $[M+8H^{+}]/8$ = 1062.950 Da. Observed $[M+H^{+}]/8$ = 1062.9525 Da.

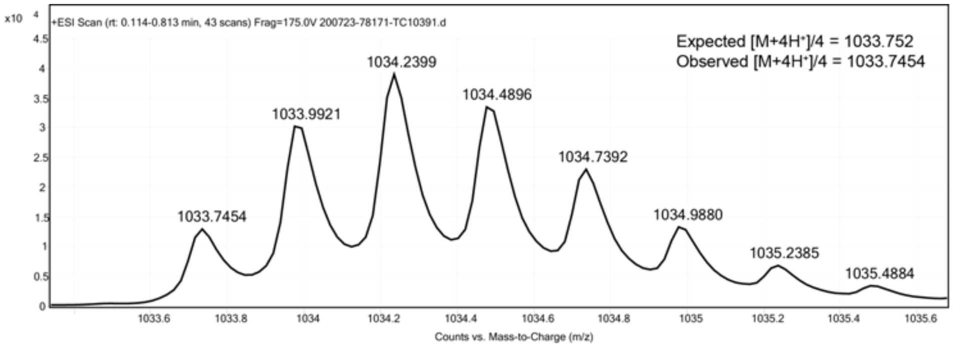

Figure S27. ESI-TOF spectrum for acidic monomer **34e-x** (TC10391; $C_{178}H_{277}N_{46}O_{64}S$). Expected $[M+4H^+]/4 = 1033.752$ Da. Observed $[M+4H^+]/4 = 1033.7454$ Da.

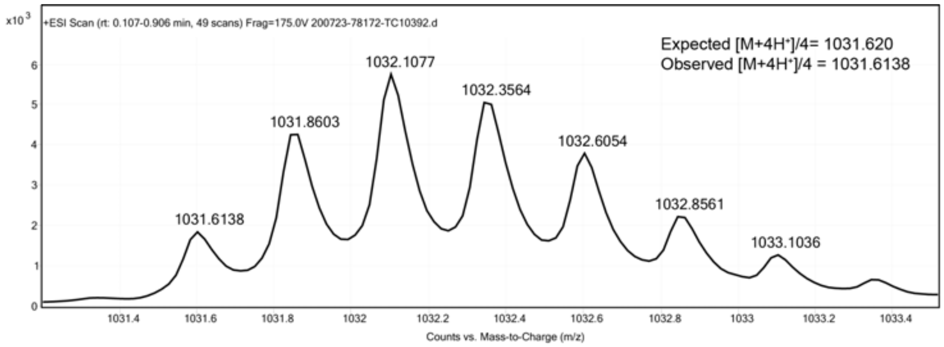

Figure S28. ESI-TOF spectrum for basic monomer ***29g'*-x** (TC10392; $C_{187}H_{322}N_{55}O_{46}S$). Expected $[M+4H^+]/4 = 1031.620$ Da. Observed $[M+4H^+]/4 = 1031.6138$ Da.

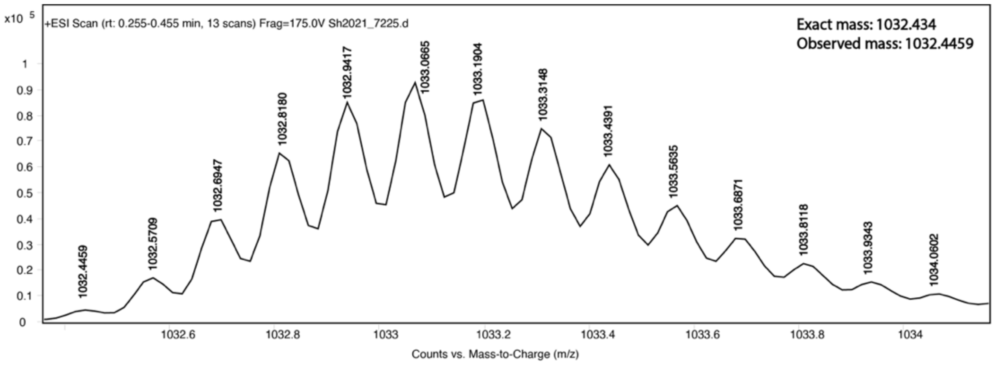

Figure S29. ESI-TOF spectrum for disulfide-bound coiled-coil variant **34e/29g'-xx** (SH2021; $C_{365}H_{599}N_{101}O_{110}S_2$). Expected $[M+8H^+]/8 = 1032.434$ Da. Observed $[M+8H^+]/8 = 1032.4459$ Da.

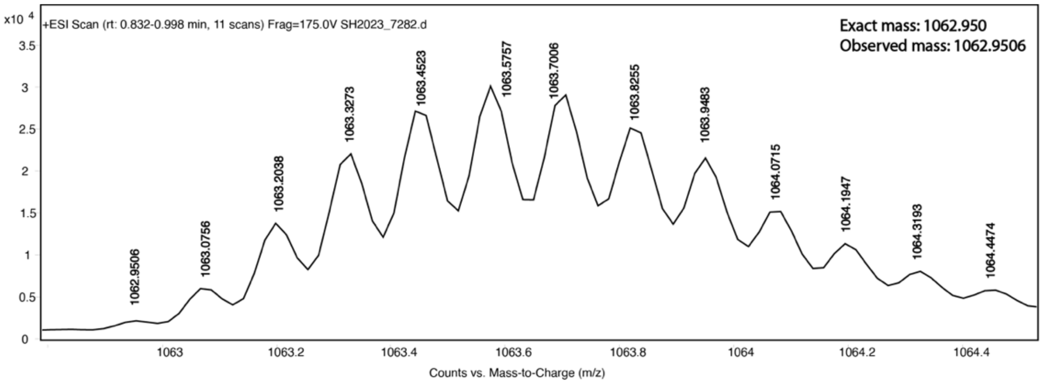

Figure S30. ESI-TOF spectrum for stapled disulfide-bound coiled-coil variant **s34e/29g'-x4x** (SH2023; $C_{373}H_{615}N_{108}O_{113}S_2$). Expected $[M+8H^+]/8 = 1062.950$ Da. Observed $[M+8H^+]/8 = 1062.9506$ Da.

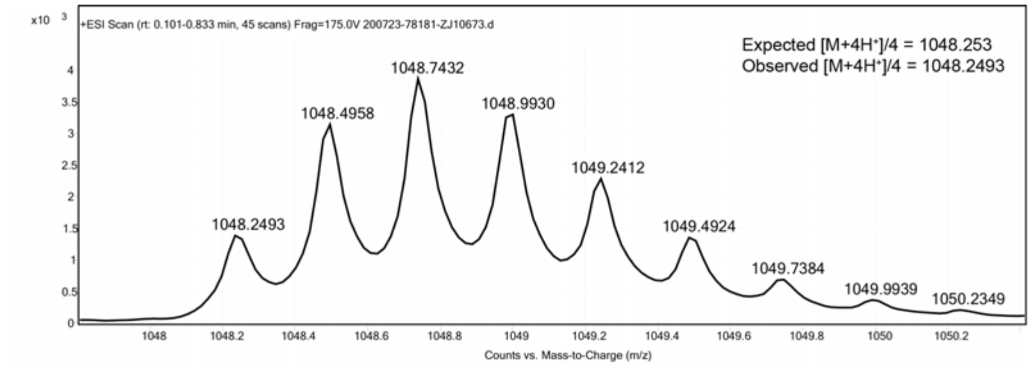

Figure S31. ESI-TOF spectrum for acidic monomer **7f-x** (ZJ10673; $C_{180}H_{279}N_{46}O_{66}S$). Expected [M+4H$^+$]/4 = 1048.253 Da. Observed [M+4H$^+$]/4 = 1048.2493 Da.

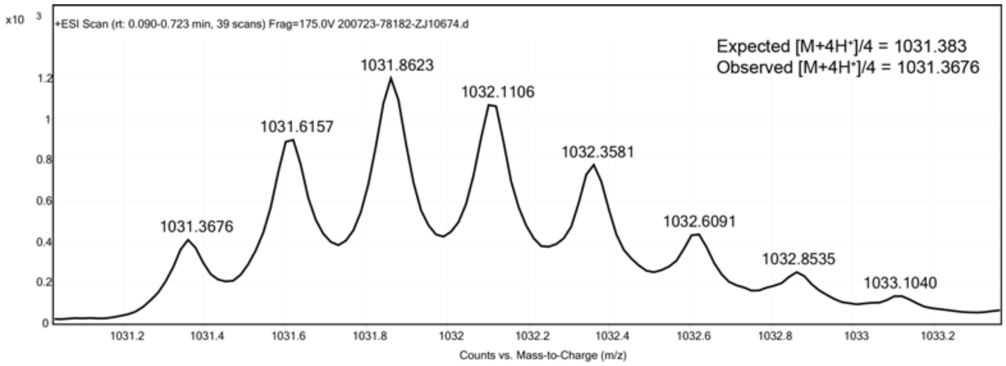

Figure S32. ESI-TOF spectrum for basic monomer **10b'-x** (ZJ10674; $C_{188}H_{327}N_{56}O_{44}S$). Expected [M+4H$^+$]/4 = 1031.383 Da. Observed [M+4H$^+$]/4 = 1031.3676 Da.

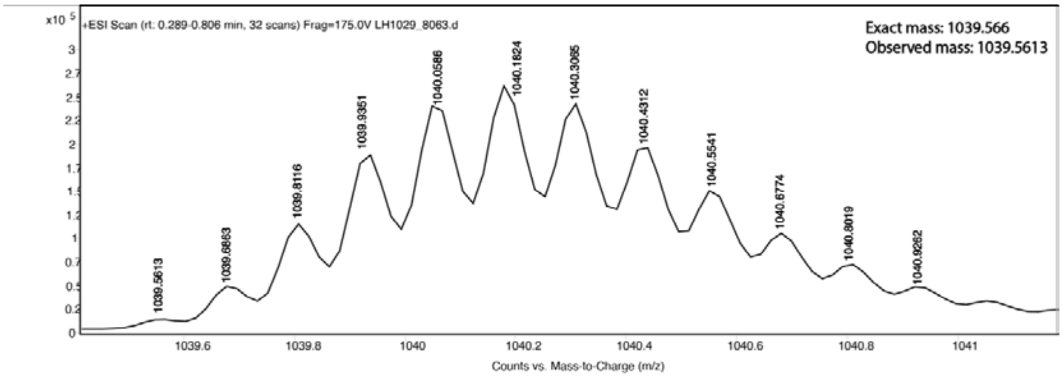

Figure S33. ESI-TOF spectrum for disulfide-bound coiled-coil variant **7f/10b'-xx** (LH1029; $C_{368}H_{606}N_{102}O_{110}S_2$). Expected [M+8H$^+$]/8 = 1039.566 Da. Observed [M+8H$^+$]/8 = 1039.5613 Da.

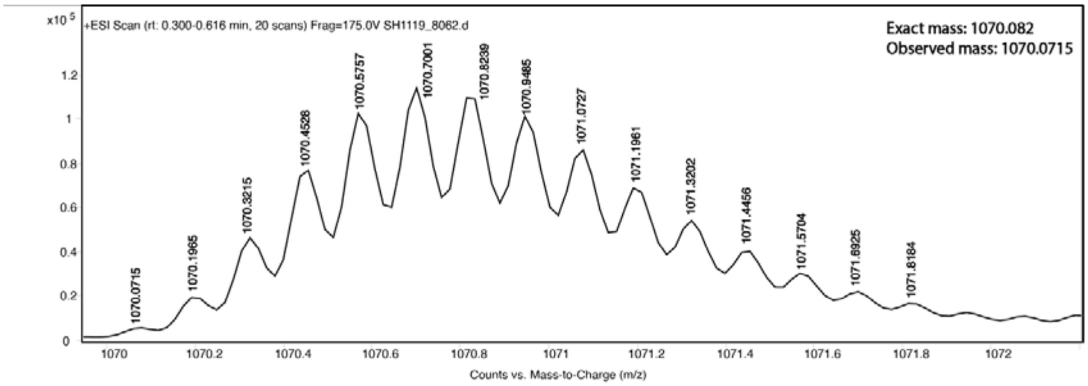

Figure S34. ESI-TOF spectrum for stapled disulfide-bound coiled-coil variant **s7f/10b'-x4x** (SH1119; $C_{376}H_{622}N_{109}O_{113}S_2$). Expected [M+8H$^+$]/8 = 1070.082 Da. Observed [M+8H$^+$]/8 = 1070.0715 Da.

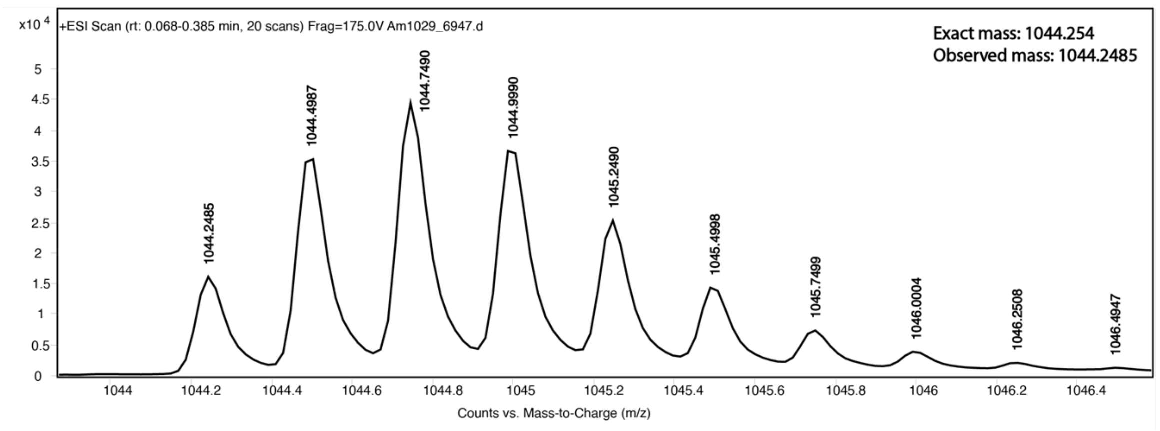

Figure S35. ESI-TOF spectrum for acidic monomer **14f-x** (AM1029, $C_{180}H_{279}N_{46}O_{65}S$). Expected $[M+4H^+]/4$ = 1044.254 Da. Observed $[M+4H^+]/4$ = 1044.2485 Da.

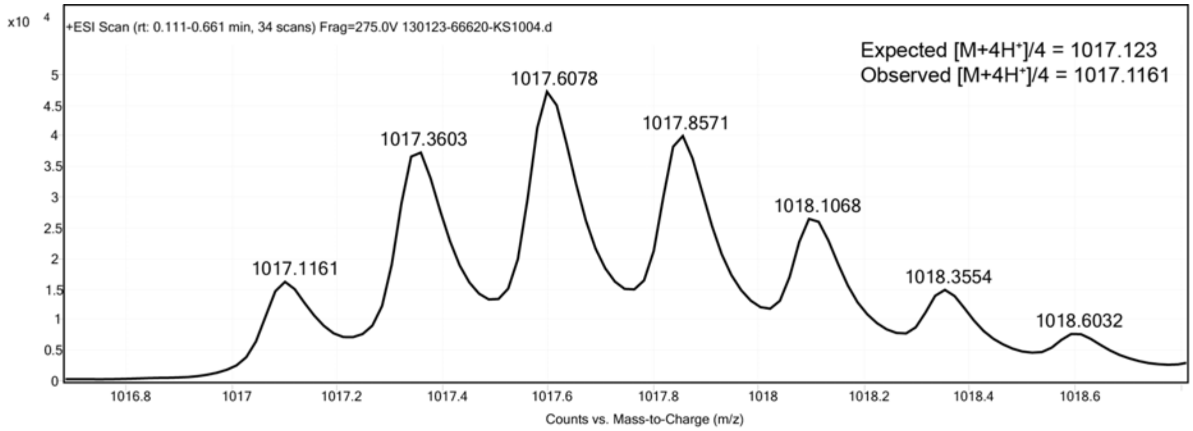

Figure S36. ESI-TOF spectrum for basic monomer **17b'-x** (KS1004; $C_{182}H_{324}N_{55}O_{46}S$). Expected $[M+4H^+]/4$ = 1017.123 Da. Observed $[M+4H^+]/4$ = 1017.1161 Da.

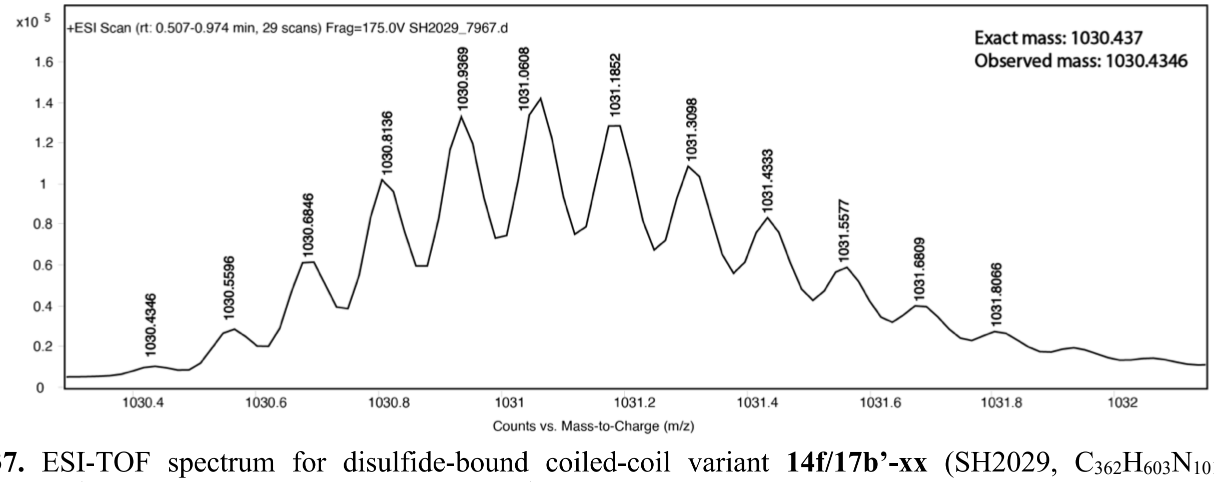

Figure S37. ESI-TOF spectrum for disulfide-bound coiled-coil variant **14f/17b'-xx** (SH2029, $C_{362}H_{603}N_{101}O_{111}S_2$). Expected $[M+8H^+]/8$ = 1030.437 Da. Observed $[M+8H^+]/8$ = 1030.4346 Da.

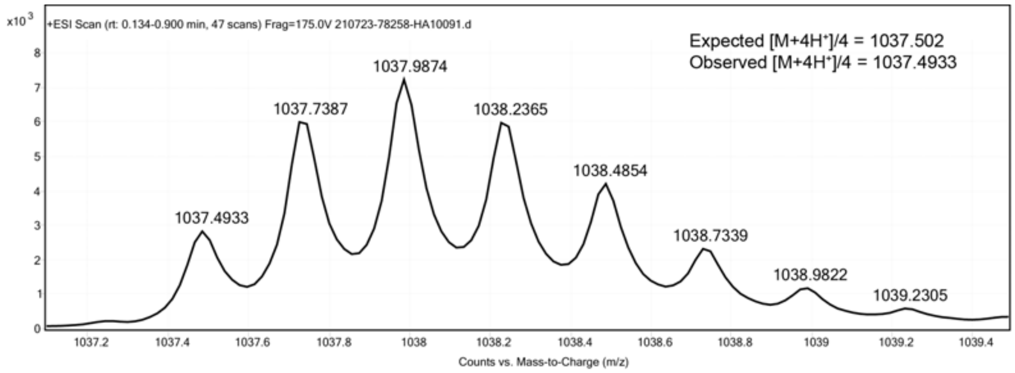

Figure S38. ESI-TOF spectrum for basic monomer variant **21f-x** (HA10091; $C_{179}H_{278}N_{45}O_{65}S$). Expected $[M+4H^+]/4$ = 1037.502 Da. Observed $[M+4H^+]/4$ = 1037.4933 Da.

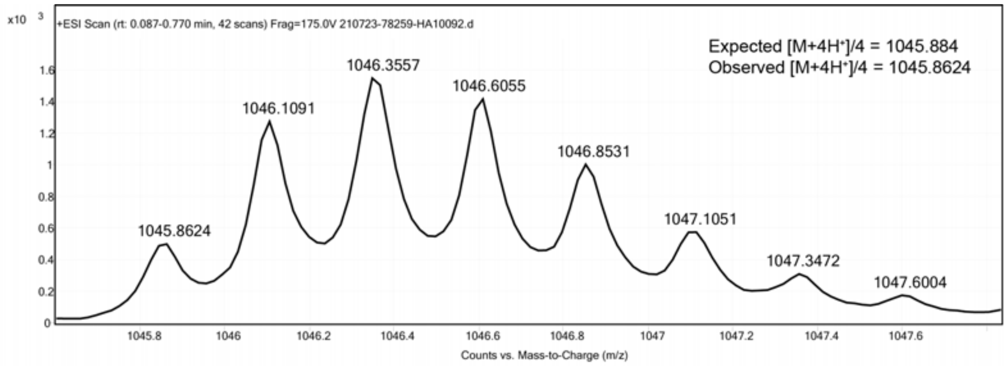

Figure S39. ESI-TOF spectrum for acidic monomer **24b'-x** (HA10092; $C_{190}H_{329}N_{56}O_{46}S$). Expected $[M+4H^+]/4$ = 1045.884 Da. Observed $[M+4H^+]/4$ = 1045.8624 Da.

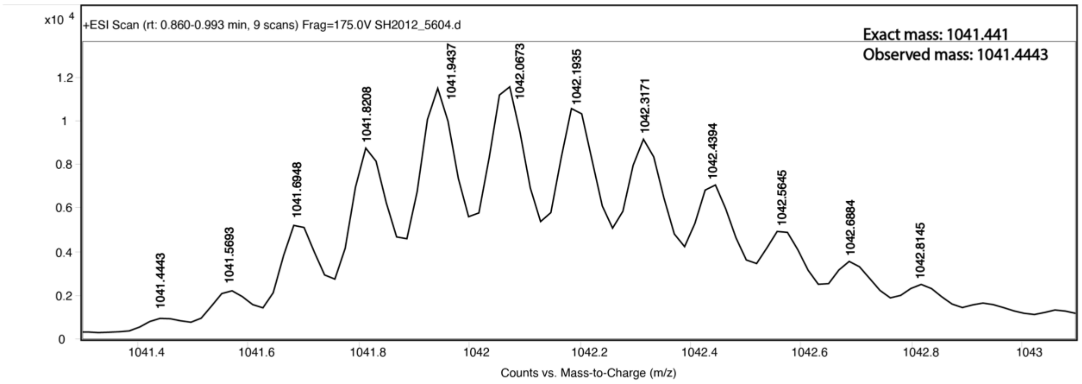

Figure S40. ESI-TOF spectrum for disulfide-bound coiled-coil variant **21f/24b'-xx** (SH2012; $C_{362}H_{607}N_{101}O_{111}S_2$). Expected $[M+8H^+]/8$ = 1041.441 Da. Observed $[M+8H^+]/8$ = 1041.4443 Da.

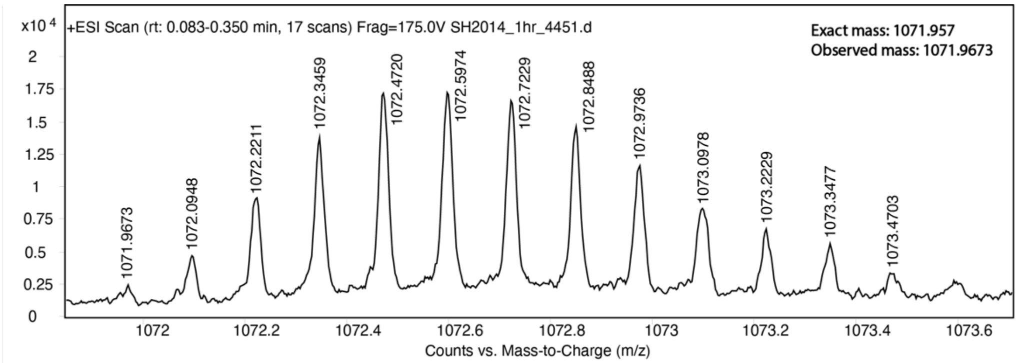

Figure S41. ESI-TOF spectrum for stapled disulfide-bound coiled-coil variant **s21f/24b'-x4x** (SH2014; $C_{377}H_{623}N_{108}O_{114}S_2$). Expected $[M+8H^+]/8$ = 1071.957 Da. Observed $[M+8H^+]/8$ = 1071.9673 Da.

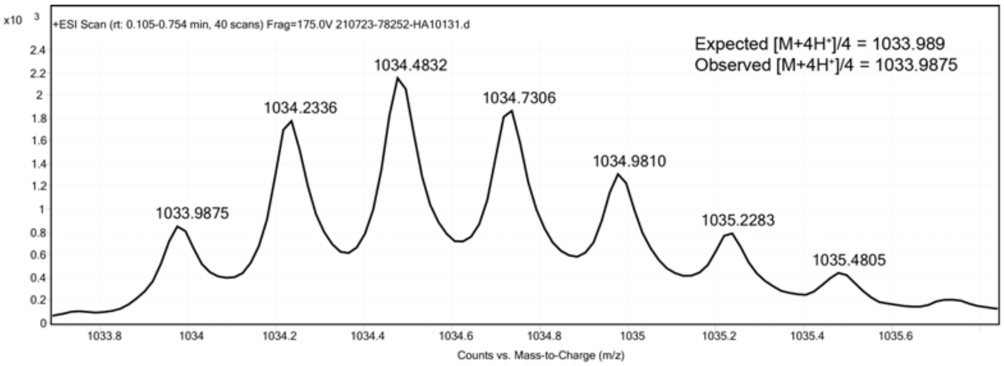

Figure S42. ESI-TOF spectrum for acidic monomer **28f-x** (HA10131; $C_{177}H_{272}N_{45}O_{66}S$). Expected $[M+4H^+]/4 = 1033.989$ Da. Observed $[M+4H^+]/4 = 1033.9875$ Da.

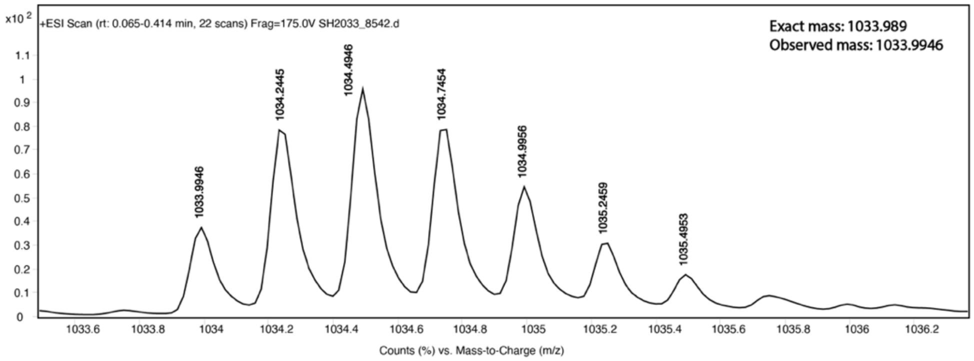

Figure S43. ESI-TOF spectrum for acidic monomer **28f-x** (SH2033; $C_{177}H_{272}N_{45}O_{66}S$). Expected $[M+4H^+]/4 = 1033.989$ Da. Observed $[M+4H^+]/4 = 1033.9875$ Da.

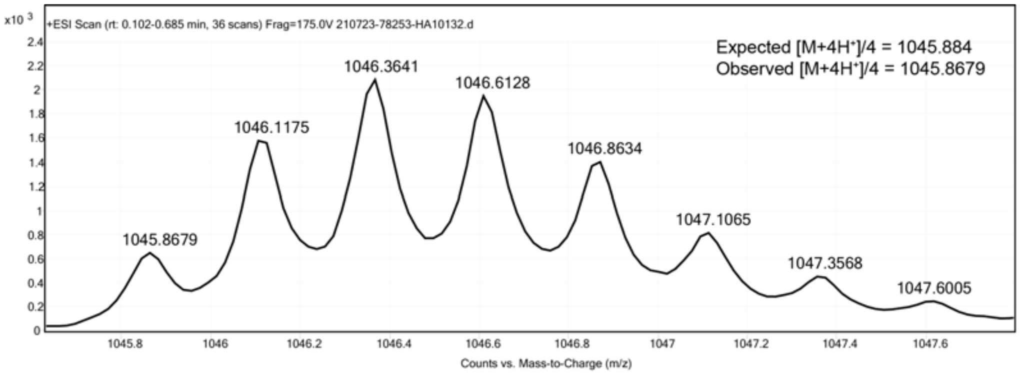

Figure S44. ESI-TOF spectrum for basic monomer **31b'-x** (HA10132; $C_{188}H_{329}N_{57}O_{44}S$). Expected $[M+4H^+]/4 = 1045.884$ Da. Observed $[M+4H^+]/4 = 1045.8679$ Da.

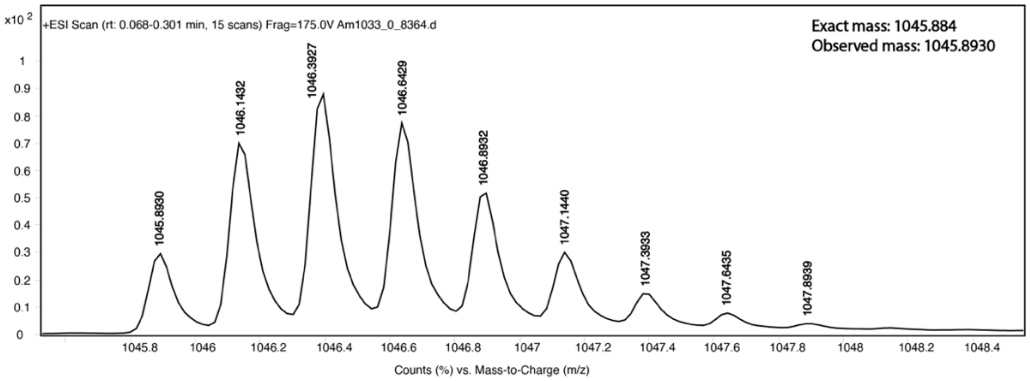

Figure S45. ESI-TOF spectrum for basic monomer **31b'-x** (AM1033; $C_{188}H_{329}N_{57}O_{44}S$). Expected $[M+4H^+]/4 = 1045.884$ Da. Observed $[M+4H^+]/4 = 1045.8679$ Da.

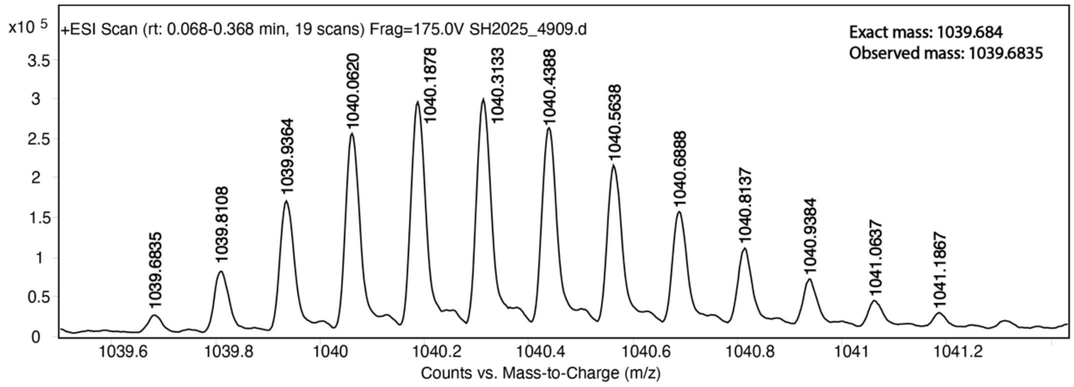

Figure S46. ESI-TOF spectrum for disulfide-bound coiled-coil **28f/31b'-xx** (SH2025; $C_{367}H_{601}N_{101}O_{112}S_2$). Expected $[M+8H^+]/8$ = 1039.684 Da. Observed $[M+8H^+]/8$ = 1039.6835 Da.

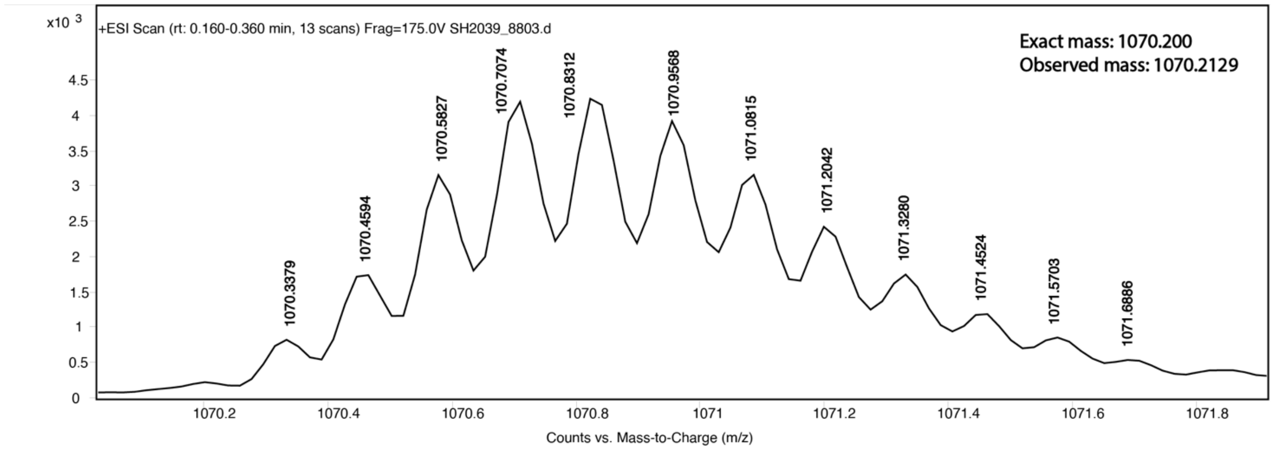

Figure S47. ESI-TOF spectrum for stapled disulfide-bound coiled-coil variant **s28f/31b'-x4x** (SH2039; $C_{375}H_{617}N_{108}O_{115}S_2$). Expected $[M+8H^+]/8$ = 1070.200 Da. Observed $[M+8H^+]/8$ = 1070.1990 Da.

Mass Specs for Reduced Stapled Product

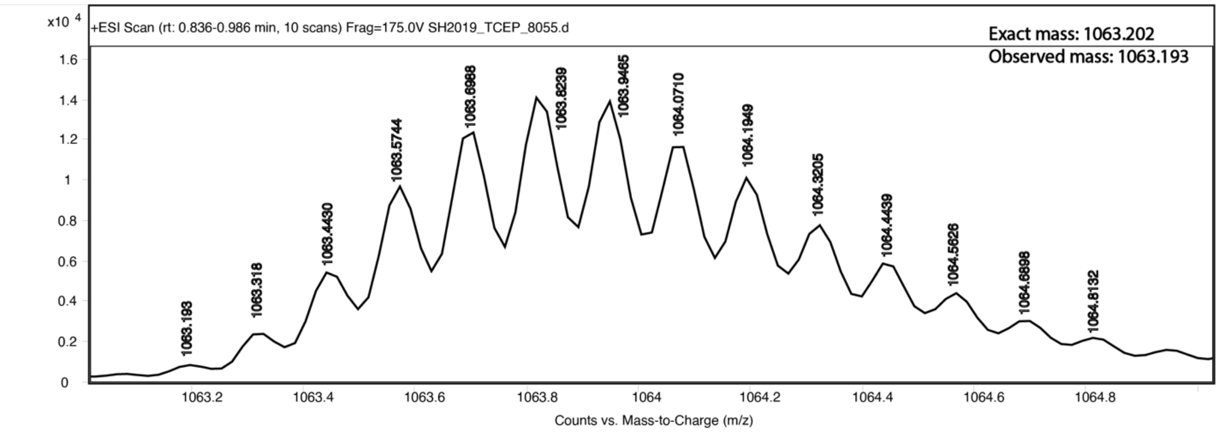

Figure S48. ESI-TOF spectrum for stapled coiled-coil variant **s6*e*/1*g*'-x4x** (SH2019_TCEP, $C_{373}H_{615}N_{108}O_{113}S_2$). Expected $[M+8H^{+}]/8$ = 1063.202 Da. Observed $[M+8H^{+}]/8$ = 1063.193 Da.

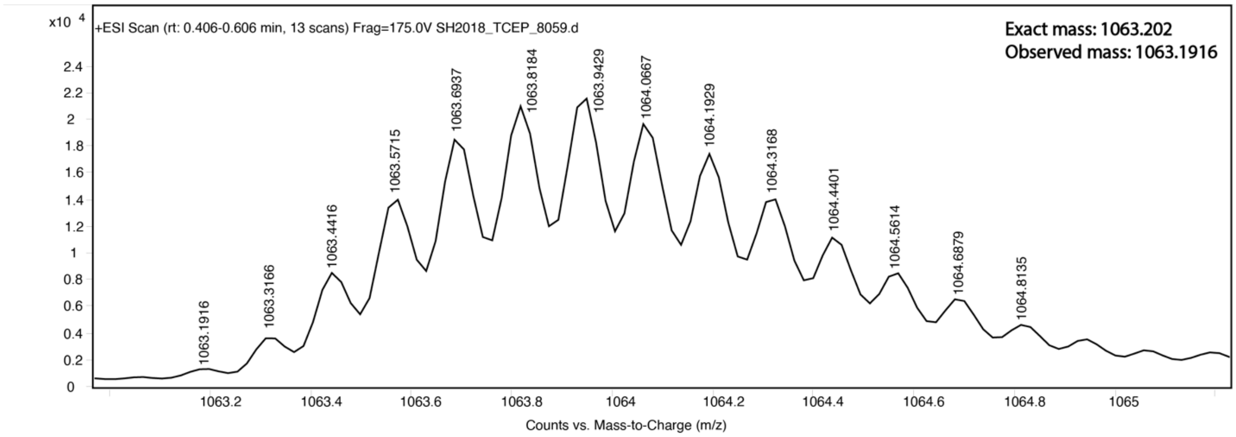

Figure S49. ESI-TOF spectrum for stapled coiled-coil variant **s13*e*/8*g*'-x4x** (SH2018_TCEP; $C_{373}H_{615}N_{108}O_{113}S_2$). Expected $[M+8H^{+}]/8$ = 1063.202 Da. Observed $[M+8H^{+}]/8$ = 1063.1916 Da.

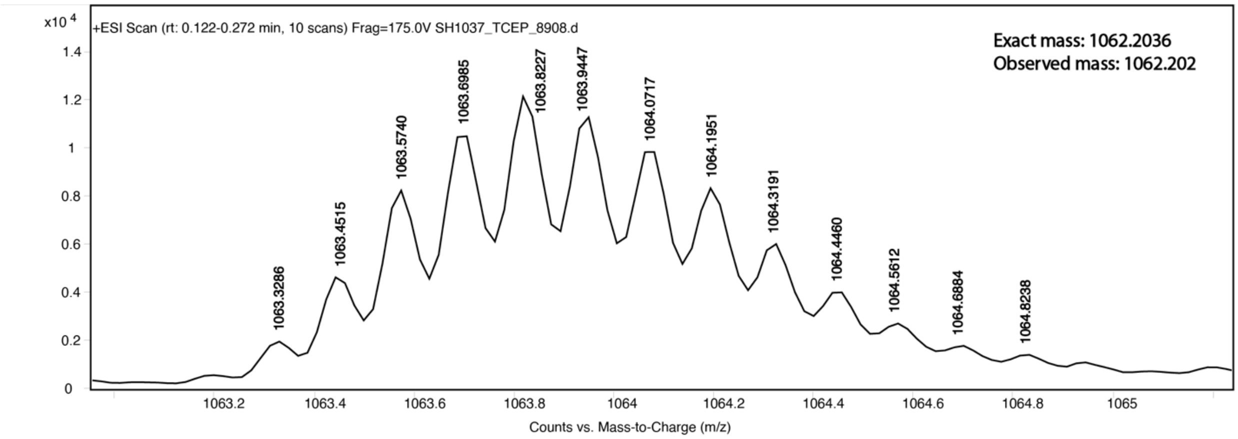

Figure S50. ESI-TOF spectrum for stapled coiled-coil variant **s20e/15g'-x4x** (SH2036, $C_{372}H_{617}N_{107}O_{114}S_2$). Expected $[M+8H^{+}]/8$ = 1063.202 Da. Observed $[M+8H^{+}]/8$ = 1063.1915 Da.

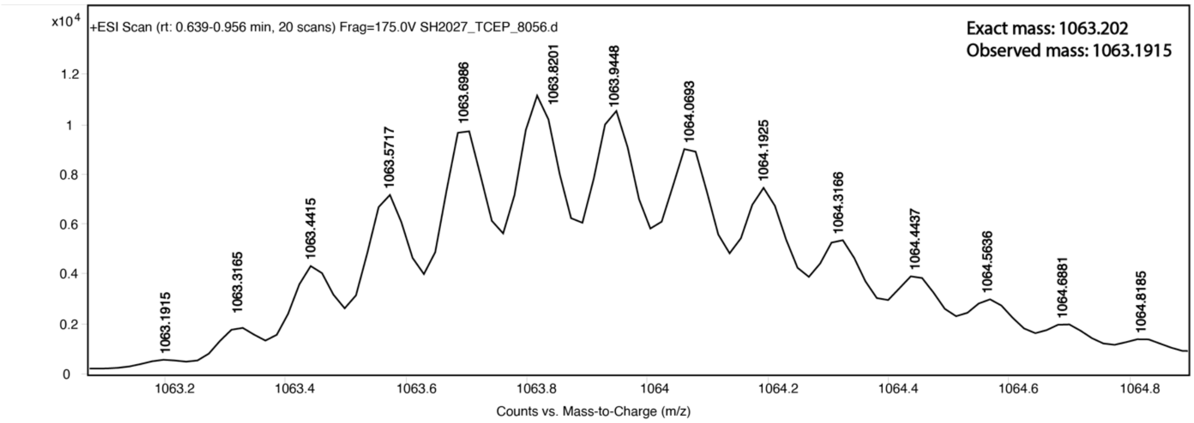

Figure S51. ESI-TOF spectrum for stapled coiled-coil variant **s27*e*/22*g*'-x4x** (SH2027_TCEP, $C_{373}H_{615}N_{108}O_{113}S_2$). Expected $[M+8H^+]/8$ = 1063.202 Da. Observed $[M+H^+]/8$ = 1063.1915 Da.

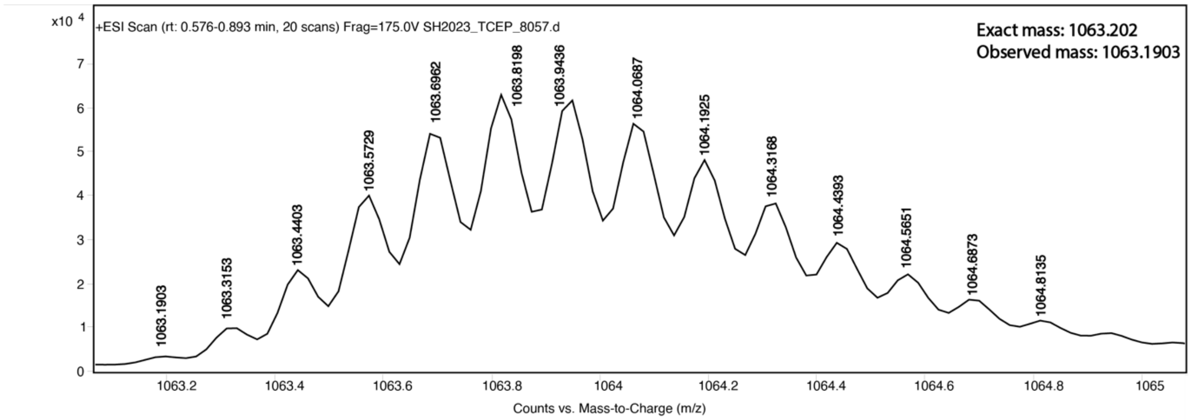

Figure S52. ESI-TOF spectrum for stapled coiled-coil variant **s34*e*/29*g*'-x4x** (SH2023_TCEP; $C_{373}H_{615}N_{108}O_{113}S_2$). Expected $[M+8H^+]/8$ = 1063.202 Da. Observed $[M+8H^+]/8$ = 1063.1903 Da.

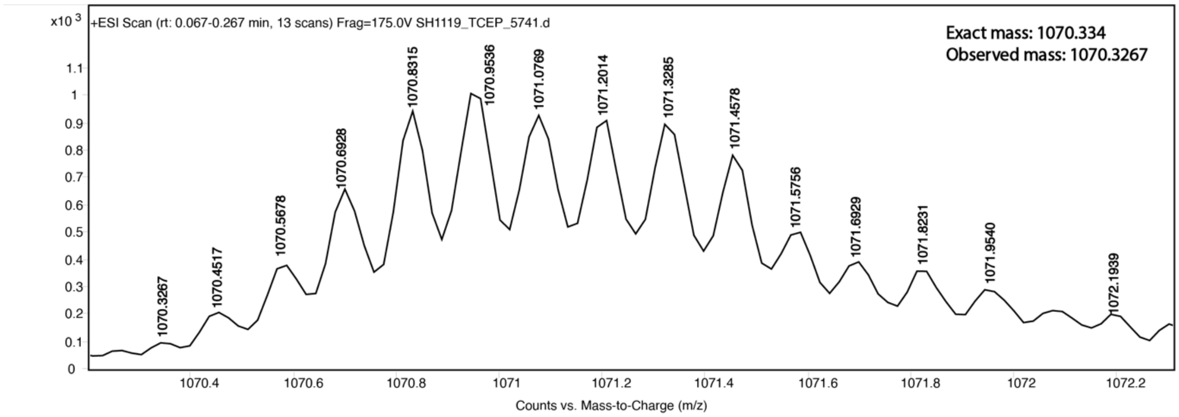

Figure S53. ESI-TOF spectrum for stapled coiled-coil variant **s7*f*/10*b*'-x4x** (SH1119_TCEP; $C_{376}H_{622}N_{109}O_{113}S_2$). Expected $[M+8H^+]/8$ = 1070.334 Da. Observed $[M+8H^+]/8$ = 1070.3267 Da.

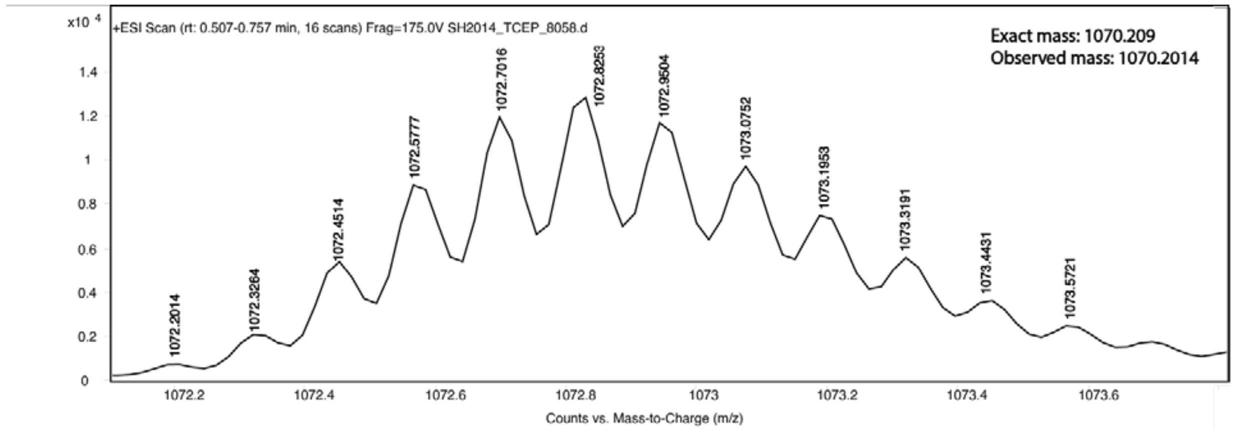

Figure S54. ESI-TOF spectrum for stapled coiled-coil variant **s21*f*/24*b*'-x4x** (SH2014_TCEP; $C_{377}H_{623}N_{108}O_{114}S_2$). Expected [M+8H$^+$]/8 = 1070.209 Da. Observed [M+8H$^+$]/8 = 1070.2014 Da.

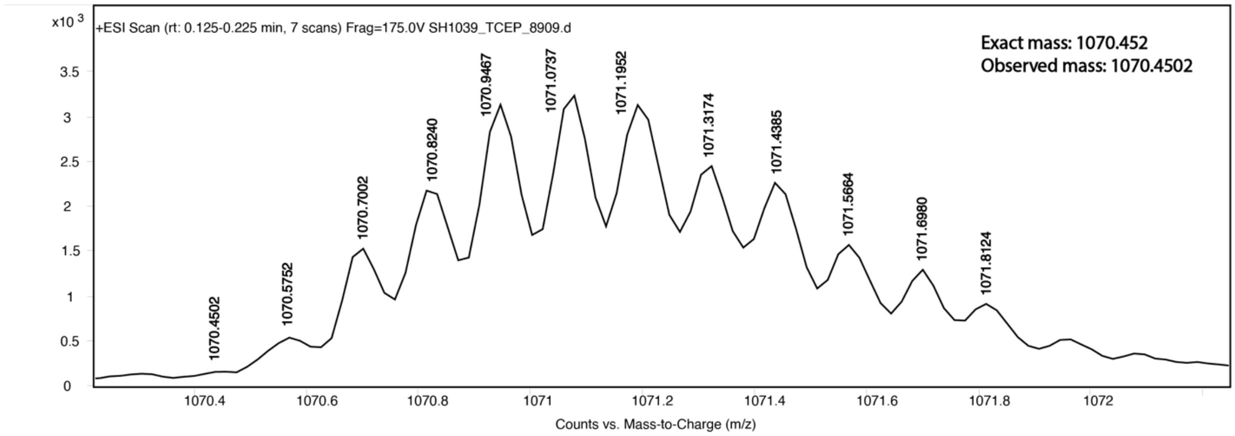

Figure S55. ESI-TOF spectrum for stapled coiled-coil variant **s28*f*/31*b*'-x4x** (2039_TCEP; $C_{375}H_{617}N_{108}O_{115}S_2$). Expected [M+8H$^+$]/8 = 1070.452 Da. Observed [M+8H$^+$]/8 = 1070.4531 Da.

4. Analytical HPLC data

Purity of stapled variants and their non-stapled counterparts was assessed by analytical reverse-phase HPLC. Chromatograms appear below in Figures S56–S72

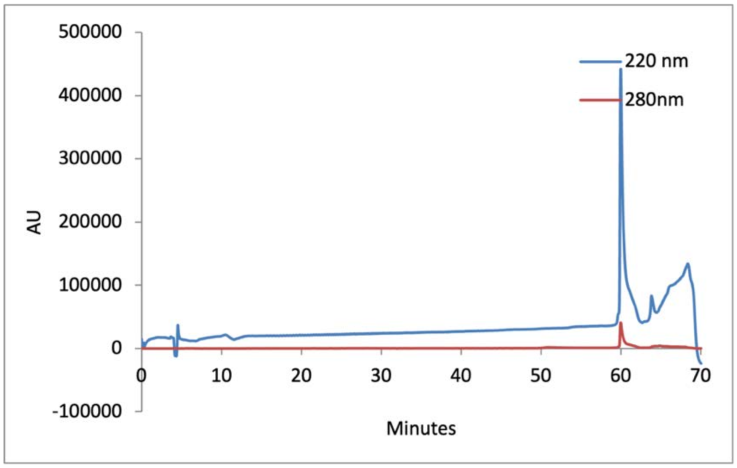

Figure S56. Analytical HPLC Data for variant **6e/1g'-xx** (SH2017). Protein solution was injected onto a C18 analytical column and eluted using a linear gradient of 10-60% B (A = H_2O, 0.1% TFA; B = MeCN, 0.1% TFA) over 50 minutes, followed by a 10-minute rinse (95% B), and a 10-minute column re-equilibration.

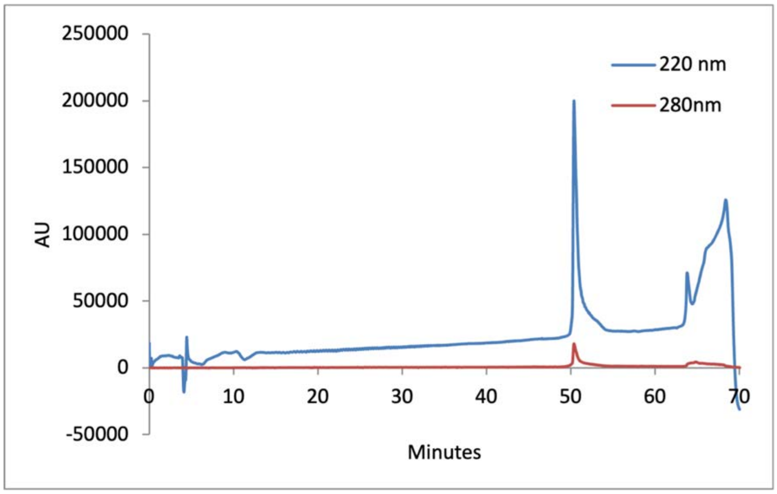

Figure S57. Analytical HPLC Data for variant **s6e/1g'-x4x** (SH2019). Protein solution was injected onto a C18 analytical column and eluted using a linear gradient of 10-60% B (A = H_2O, 0.1% TFA; B = MeCN, 0.1% TFA) over 50 minutes, followed by a 10-minute rinse (95% B), and a 10-minute column re-equilibration.

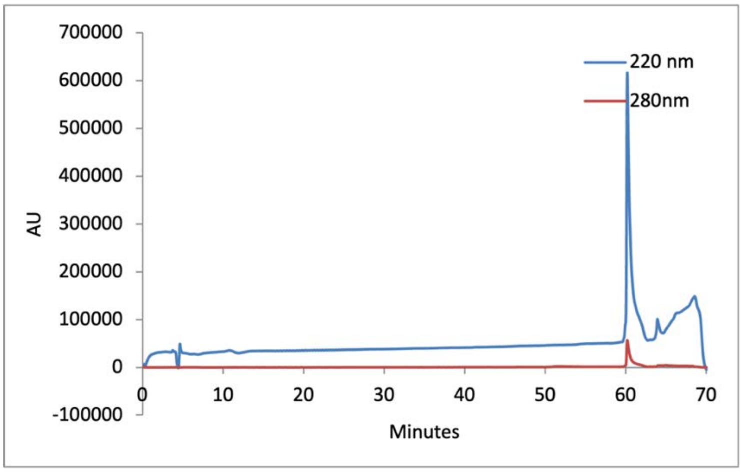

Figure S58. Analytical HPLC Data for variant **13e/8g'-xx** (SH2016). Protein solution was injected onto a C18 analytical column and eluted using a linear gradient of 10-60% B (A = H_2O, 0.1% TFA; B = MeCN, 0.1% TFA) over 50 minutes, followed by a 10-minute rinse (95% B), and a 10-minute column re-equilibration.

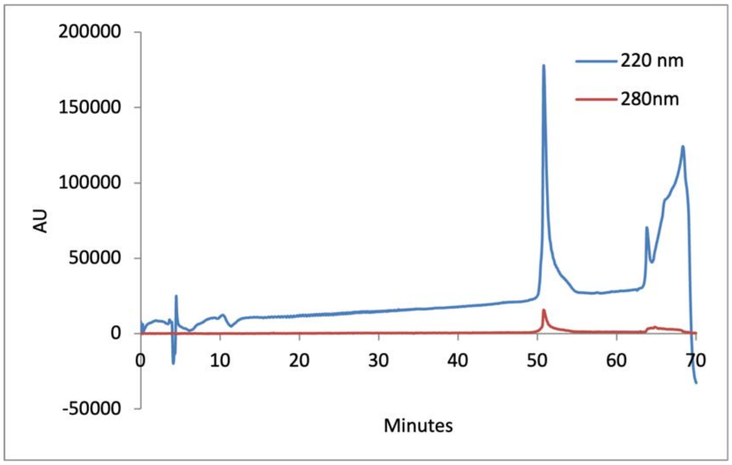

Figure S59. Analytical HPLC Data for variant **s13e/8g'-x4x** (SH2018). Protein solution was injected onto a C18 analytical column and eluted using a linear gradient of 10-60% B (A = H_2O, 0.1% TFA; B = MeCN, 0.1% TFA) over 50 minutes, followed by a 10-minute rinse (95% B), and a 10-minute column re-equilibration.

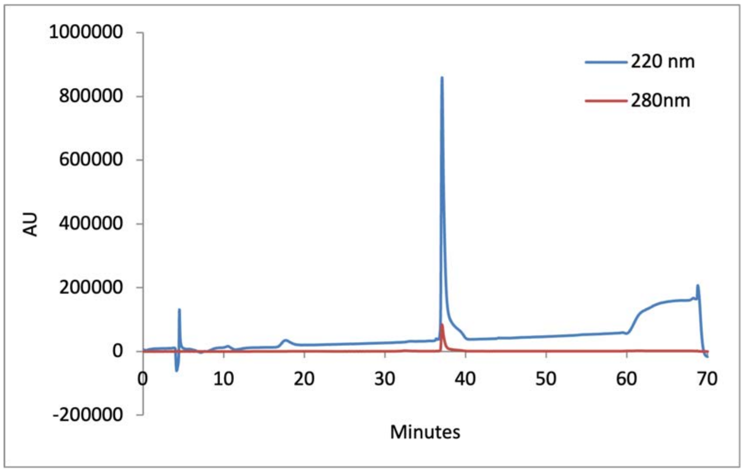

Figure S60. Analytical HPLC Data for variant **20e/15g'-xx** (SH2036). Protein solution was injected onto a C18 analytical column and eluted using a linear gradient of 10-60% B (A = H_2O, 0.1% TFA; B = MeCN, 0.1% TFA) over 50 minutes, followed by a 10-minute rinse (95% B), and a 10-minute column re-equilibration.

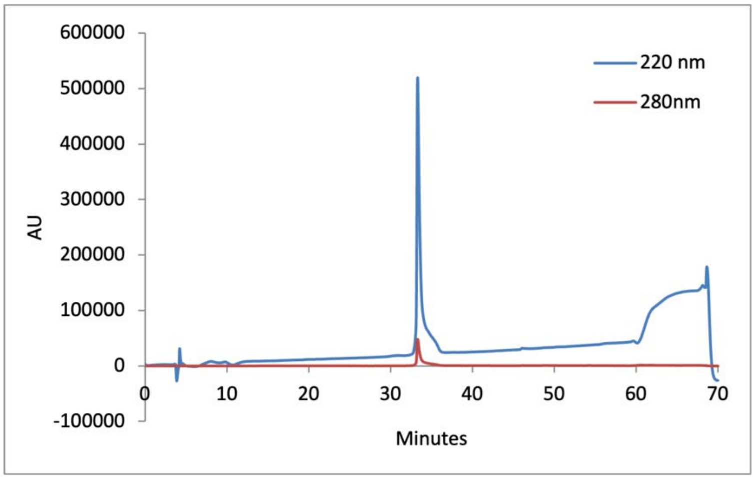

Figure S61. Analytical HPLC Data for variant **s20e/15g’-x4x** (SH2037). Protein solution was injected onto a C18 analytical column and eluted using a linear gradient of 10-60% B (A = H_2O, 0.1% TFA; B = MeCN, 0.1% TFA) over 50 minutes, followed by a 10-minute rinse (95% B), and a 10-minute column re-equilibration.

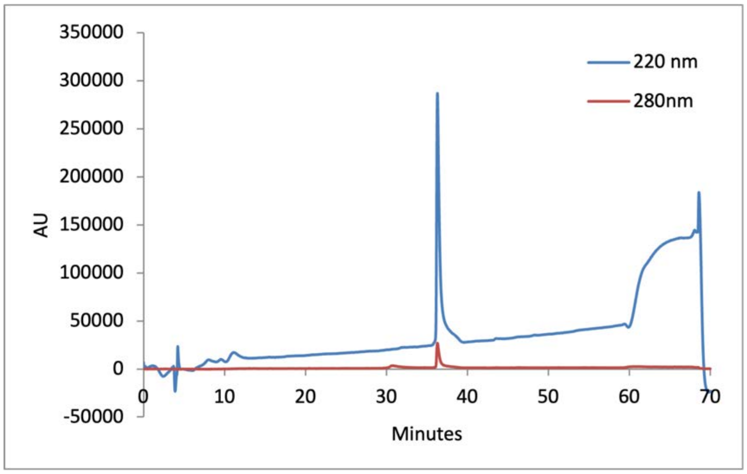

Figure S62. Analytical HPLC Data for variant **27e/22g’-xx** (SH2024). Protein solution was injected onto a C18 analytical column and eluted using a linear gradient of 10-60% B (A = H_2O, 0.1% TFA; B = MeCN, 0.1% TFA) over 50 minutes, followed by a 10-minute rinse (95% B), and a 10-minute column re-equilibration.

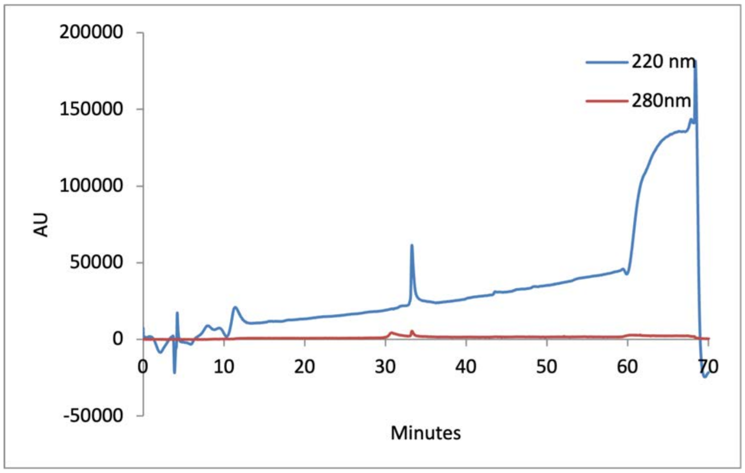

Figure S63. Analytical HPLC Data for variant **s27e/22g’-x4x** (SH2027). Protein solution was injected onto a C18 analytical column and eluted using a linear gradient of 10-60% B (A = H_2O, 0.1% TFA; B = MeCN, 0.1% TFA) over 50 minutes, followed by a 10-minute rinse (95% B), and a 10-minute column re-equilibration.

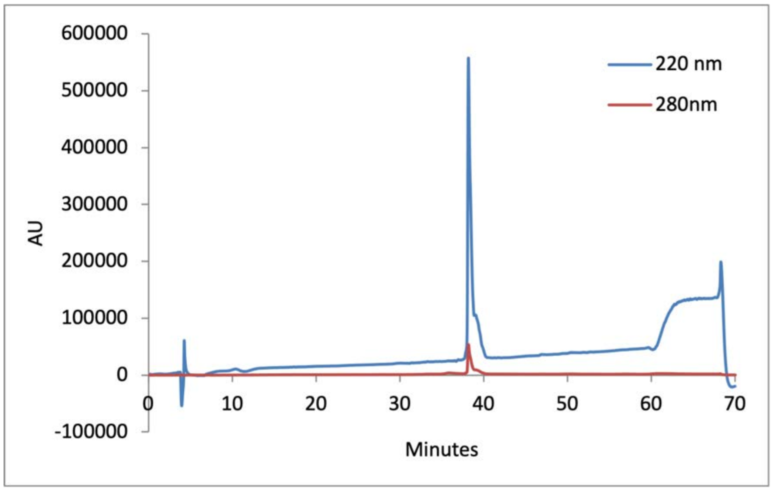

Figure S64. Analytical HPLC Data for variant **34e/29g'-xx** (SH2021). Protein solution was injected onto a C18 analytical column and eluted using a linear gradient of 10-60% B (A = H_2O, 0.1% TFA; B = MeCN, 0.1% TFA) over 50 minutes, followed by a 10-minute rinse (95% B), and a 10-minute column re-equilibration.

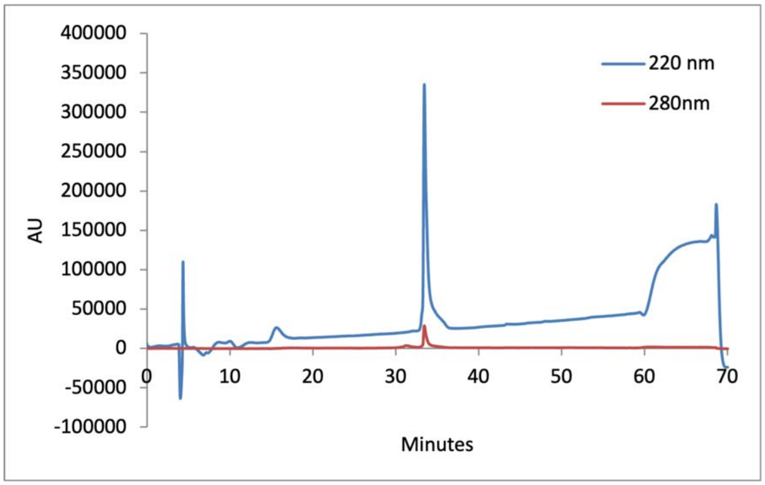

Figure S65. Analytical HPLC Data for variant **s34e/29g'-x4x** (SH2023). Protein solution was injected onto a C18 analytical column and eluted using a linear gradient of 10-60% B (A = H_2O, 0.1% TFA; B = MeCN, 0.1% TFA) over 50 minutes, followed by a 10-minute rinse (95% B), and a 10-minute column re-equilibration.

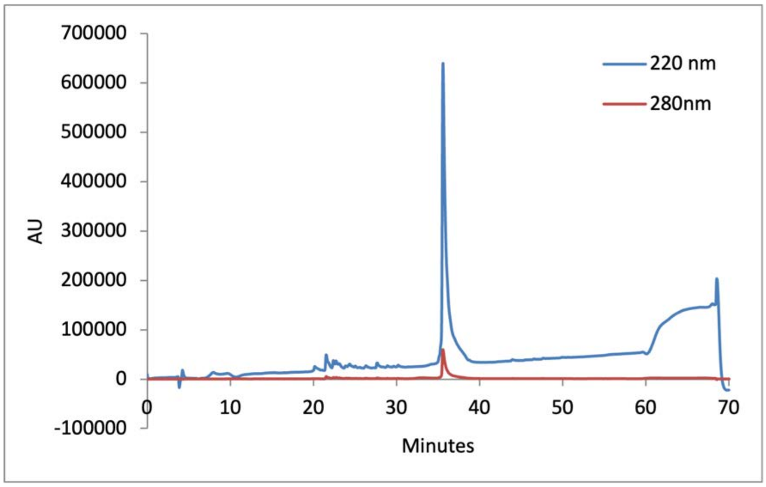

Figure S66. Analytical HPLC Data for variant **7f/10b'-xx** (LH1029). Protein solution was injected onto a C18 analytical column and eluted using a linear gradient of 10-60% B (A = H_2O, 0.1% TFA; B = MeCN, 0.1% TFA) over 50 minutes, followed by a 10-minute rinse (95% B), and a 10-minute column re-equilibration.

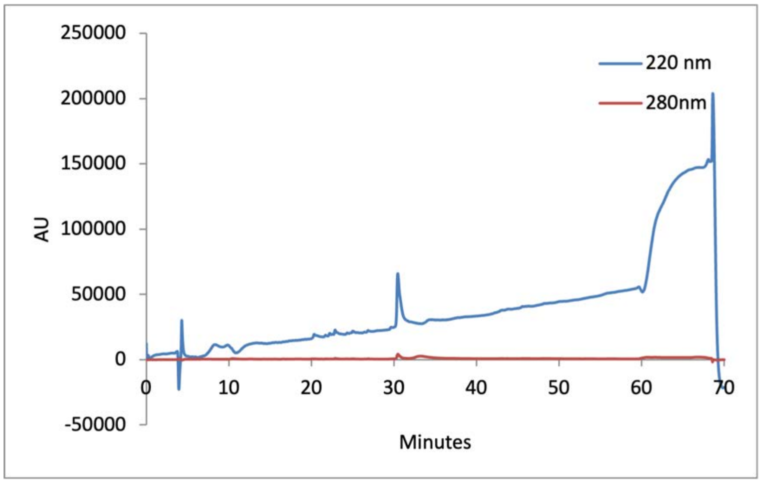

Figure S67. Analytical HPLC Data for variant **s7f/10b'-x4x** (SH1119). Protein solution was injected onto a C18 analytical column and eluted using a linear gradient of 10-60% B (A = H_2O, 0.1% TFA; B = MeCN, 0.1% TFA) over 50 minutes, followed by a 10-minute rinse (95% B), and a 10-minute column re-equilibration.

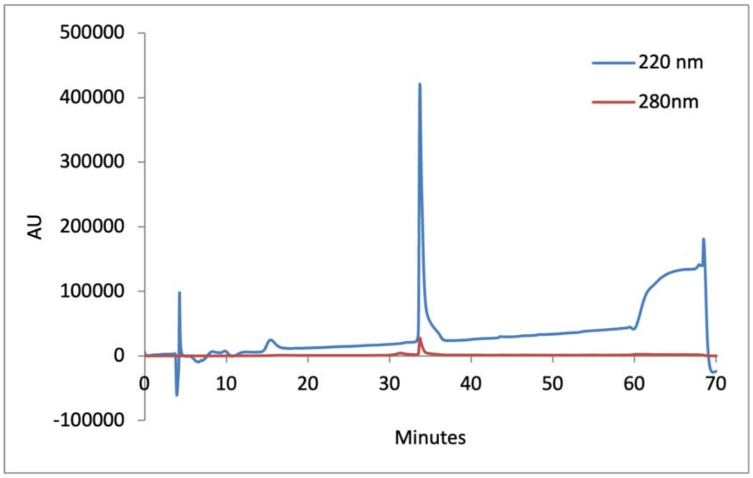

Figure S68. Analytical HPLC Data for variant **14f/17b'-xx** (SH2029). Protein solution was injected onto a C18 analytical column and eluted using a linear gradient of 10-60% B (A = H_2O, 0.1% TFA; B = MeCN, 0.1% TFA) over 50 minutes, followed by a 10-minute rinse (95% B), and a 10-minute column re-equilibration.

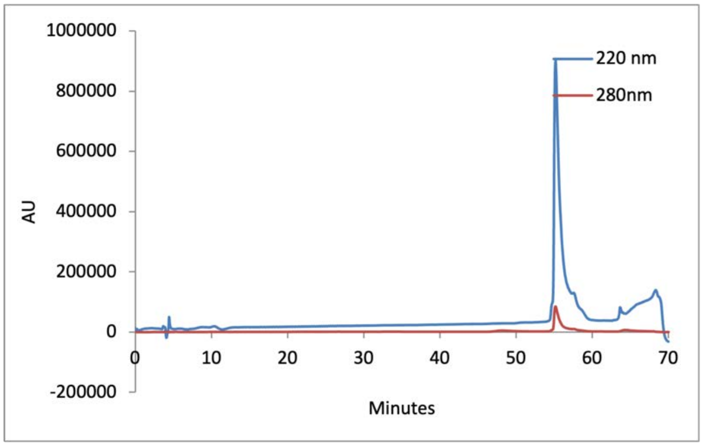

Figure S69. Analytical HPLC Data for variant **21f/24b'-xx** (SH2012). Protein solution was injected onto a C18 analytical column and eluted using a linear gradient of 10-60% B (A = H_2O, 0.1% TFA; B = MeCN, 0.1% TFA) over 50 minutes, followed by a 10-minute rinse (95% B), and a 10-minute column re-equilibration.

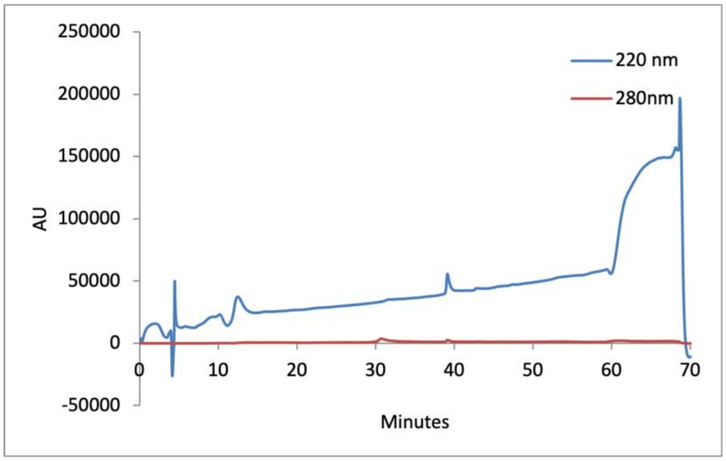

Figure S70. Analytical HPLC Data for variant **s21f/24b'-x4x** (SH2014). Protein solution was injected onto a C18 analytical column and eluted using a linear gradient of 10-60% B (A = H_2O, 0.1% TFA; B = MeCN, 0.1% TFA) over 50 minutes, followed by a 10-minute rinse (95% B), and a 10-minute column re-equilibration.

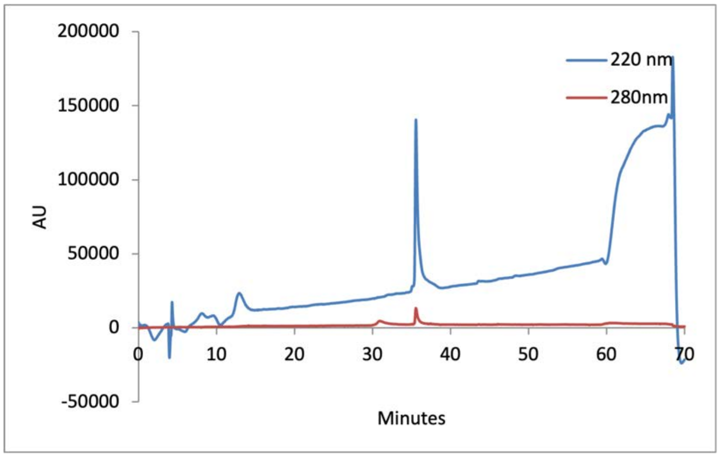

Figure S71. Analytical HPLC Data for variant **28f/31b'-xx** (SH2025). Protein solution was injected onto a C18 analytical column and eluted using a linear gradient of 10-60% B (A = H_2O, 0.1% TFA; B = MeCN, 0.1% TFA) over 50 minutes, followed by a 10-minute rinse (95% B), and a 10-minute column re-equilibration.

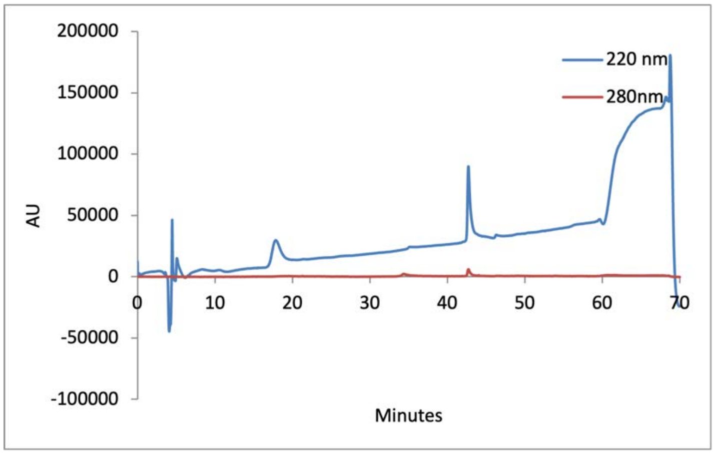

Figure S72. Analytical HPLC Data for variant **s28f/31b'-x4x** (SH2039). Protein solution was injected onto a C18 analytical column and eluted using a linear gradient of 10-60% B (A = H_2O, 0.1% TFA; B = MeCN, 0.1% TFA) over 50 minutes, followed by a 10-minute rinse (95% B), and a 10-minute column re-equilibration.

(return to Table of Contents)

5. Global fitting of variable temperature CD data

The conformational stability of stapled variants and their non-stapled counterparts was assessed by variable-temperature circular dichroism spectropolarimetry in 20 mM sodium phosphate buffer (pH 7) + 4 M guanidinium chloride denaturant. Denaturant was required because some of these variants did not completely unfold even at the highest experimentally accessible temperature (95 °C; at higher temperatures, the boiling and evaporation of water interferes with data collection). Including 4 M guanidinium chloride denaturant allowed each variant to unfold completely over the range of experimentally accessible temperatures, so that we could determine melting temperature and folding free energies under these conditions. Data from three replicate variable temperature CD experiments were each fit globally to a model for a two-state thermally induced unfolding transition using the program Mathematica (Wolfram Research). We used a model based on two-state monomer folding/unfolding equilibrium for stapled coiled-coil variants **s6e/1g'-x4x**, **s13e/8g'-x4x**, **s20e/15g'-x4x**, **s27e/22g'-x4x**, **s34e/29g'-x4x**, **s7f/10b'-x4x**, **s14f/17b'-x4x**, **s21f/24b'-x4x**, and **s28f/31b'-x4x**; and for their non-stapled counterparts variants **6e/1g'-xx**, **13e/8g'-xx**, **20e/15g'-xx**, **27e/22g'-xx**, **34e/29g'-xx**, **7f/10b'-xx**, **14f/17b'-xx**, **21f/24b'-xx**, and **28f/31b'-xx**. This model is described in equation S1:

$$[\theta] = \frac{[K\cdot(a_n+b_n\cdot T)+(c_n+d_n\cdot T)]}{1+K}, \tag{S1}$$

where T is temperature in Kelvin, a_n is the y-intercept and b_n is the slope of the pre-transition baseline for melt n (a_1 and b_1 for replicate 1, a_2 and b_2 for replicate 2, a_3 and b_3 for replicate 3, etc.); c_n is the y-intercept and d_n is the slope of the post-transition baseline for replicate n (c_1 and d_1 for replicate 1, c_2 and d_2 for replicate 2, c_3 and d_3 for replicate 3, etc.); and K is the temperature-dependent folding equilibrium constant. K is related to the temperature-dependent free energy of folding ΔG according to the following equation:

$$K = \exp\left[-\frac{\Delta G}{RT}\right], \tag{S2}$$

where R is the universal gas constant (0.0019872 kcal/mol/K). ΔG is a function of temperature, as shown in the following equation:

$$\Delta G = \frac{\Delta H_0\cdot(T_m-T)}{T_m} + \Delta C_p\cdot\left(T - T_m - T\cdot\ln\left[\frac{T}{T_m}\right]\right), \tag{S3}$$

where T_m is the midpoint of the unfolding transition and the temperature at which $\Delta G_f = 0$; ΔH_0 is the change in enthalpy upon folding at $T = T_m$; and ΔC_p is the change in heat capacity upon folding. In some cases, we found that some fit parameters had sufficiently high standard errors as to render them indistinguishable from zero and therefore not essential to the fit (as judged by their p-values). When this occurred, we repeated the fitting process without the non-essential parameters. We used the fit parameters for each variant to calculate the ΔG values given in the main text; we calculated the uncertainty for each ΔG value by propagation of error using the standard errors

of the fit parameters.

Molar ellipticity data at 222 nm ($[\theta]_{222}$), melting temperatures (T_m), and folding free energies ΔG for variants **s6e/1g'-x4x**, **s13e/8g'-x4x**, **s20e/15g'-x4x**, **s27e/22g'-x4x**, **s34e/29g'-x4x**, **s7f/10b'-x4x, s14f/17b'-x4x, s21f/24b'-x4x,** and **s28f/31b'-x4x**, and for their non-stapled counterparts variants **6e/1g'-xx**, **13e/8g'-xx**, **20e/15g'-xx**, **27e/22g'-xx**, **34e/29g'-xx**, **7f/10b'-xx**, **14f/17b'-xx**, **21f/24b'-xx**, and **28f/31b'-xx** are given in Table S2. $[\theta]_{222}$ is a well-known indicator of α-helical secondary structure, whereas T_m values are indicators of tertiary/quaternary structural stability. Values of the ratio $[\theta]_{222}/[\theta]_{208}$ near 1.1 are also considered diagnostic of coiled-coil tertiary/quaternary structures. However, we were not able to measure $[\theta]_{208}$ due to the excessively high dynode values we observed at wavelengths less than 210 nm in solutions of these variants in 20 mM phosphate buffer (pH 7) + 4 M guanidinium chloride (which was necessary to observe complete or nearly complete thermal unfolding transitions). Therefore, our assessment of coiled-coil tertiary structural stability must rely on T_m values alone.

CD spectra and variable temperature CD data for each variant are shown in Figures S73-S88 along with the fit parameters ± standard error. Standard parameter errors were used to estimate the uncertainty in the thermodynamic values given in the main text by propagation of error.

Table S2. Molar ellipticity, melting temperatures and folding free energies for non-stapled variants and their PEG-stapled counterparts.[a]

Protein	$[\theta]_{222}$ (deg cm^2 dmol^{-1} x 10^3)	T_m (°C)	Impact of Stapling ΔΔG (kcal/mol)
**6e/1g'-xx**	-26.2095	48.7 ± 0.1	
**s6e/1g'-x4x**	-33.5562	77.5 ± 0.1	-2.96 ± 0.04
**13*e*/8*g*'-xx**	-25.9562	41.8 ± 0.2	
**s13*e*/8*g*'-x4x**	-29.7571	75.6 ± 0.1	-3.13 ± 0.05
**20*e*/15*g*'-xx**	-25.1486	43.2 ± 0.1	
**s20*e*/15*g*'-x4x**	-29.6257	74.4 ± 0.1	-3.13 ± 0.04
**27*e*/22*g*'-xx**	-24.3543	42.5 ± 0.2	
**s27*e*/22*g*'-x4x**	-24.819	63.7 ± 0.1	-2.07 ± 0.03
**34*e*/29*g*'-xx**	-30.8857	51.8 ± 0.1	
**s34*e*/29*g*'-x4x**	-28.9333	61.9 ± 0.1	-1.07 ± 0.01
**7*f*/10*b*'-xx**	-22.6714	48.9 ± 0.2	
**s7*f*/10*b*'-x4x**	-24.7019	53.3 ± 0.2	-0.23 ± 0.01
**14*f*/17b'-xx**	-27.7886	43.4 ± 0.2	-
**21*f*/24*b*'-xx**	-26.4581	41.9 ± 0.2	
**s21*f*/24*b*'-x4x**	-3.38095	26.6 ± 1.3	2.16 ± 0.62
**28*f*/31*b*'-xx**	-24.3267	45.7 ± 0.1	
**s28*f*/31*b*'-x4x**	-14.1848	37.8 ± 0.3	0.62 ± 0.03

[a]ΔΔG, ΔΔH, and -TΔΔS values for each variant are given ± std. error in kcal/mol at the melting temperature of its corresponding non-stapled counterpart at 15 μM protein concentration in 20 mM sodium phosphate buffer (pH 7) + 4.0 M GdnHCl.

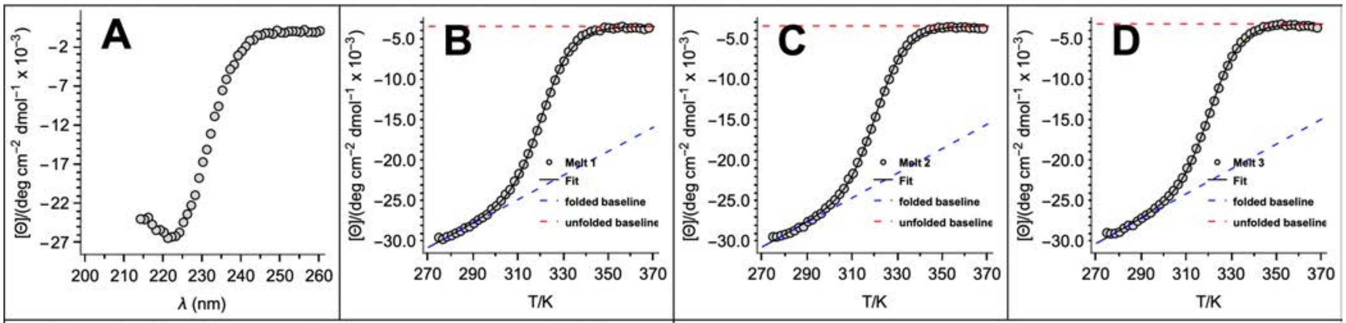

Parameters	Values	Units	P–Values
ΔH_0	−31.16 ± 0.36	kcal/mol	<0.001
T_m	48.7 ± 0.1	°C	<0.001
ΔC_p	---	---	---
a1	−70.8 ± 1.3	deg cm^{-2} dmol^{-1} x 10^{-3}	<0.001
b1	0.149 ± 0.005	deg cm^{-2} dmol^{-1} K^{-1} x 10^{-3}	<0.001
c1	−3.35 ± 0.05	deg cm^{-2} dmol^{-1} x 10^{-3}	<0.001
d1	---	---	---
a2	−71.6 ± 1.3	deg cm^{-2} dmol^{-1} x 10^{-3}	<0.001
b2	0.152 ± 0.005	deg cm^{-2} dmol^{-1} K^{-1} x 10^{-3}	<0.001
c2	−3.31 ± 0.05	deg cm^{-2} dmol^{-1} x 10^{-3}	<0.001
d2	---	---	---
a3	−71.7 ± 1.3	deg cm^{-2} dmol^{-1} x 10^{-3}	<0.001
b3	0.154 ± 0.005	deg cm^{-2} dmol^{-1} K^{-1} x 10^{-3}	<0.001
c3	−3.04 ± 0.05	deg cm^{-2} dmol^{-1} x 10^{-3}	<0.001
d3	---	---	---

$R^2 = 0.99990$
sum residuals^2 = 4.63952

For **6e/1g'–xx**, at 321.8 K,
ΔG = 0.00 ± 0.01 kcal/mol
ΔH = −31.2 ± 0.4 kcal/mol
−TΔS = 31.2 ± 0.4 kcal/mol

Figure S73. (A) CD spectra and (B–D) variable temperature CD data (triplicate) for 15 μM stapled disulfide-bound coiled-coil variant **6e/1g'-xx** (SH2017) in 20 mM sodium phosphate (pH 7) with 4 M GdnHCl. Fit parameters from eq. S1–S3 appear in the table, as do calculated values of ΔG_f, ΔH_f, and -$T\Delta S_f$ ± standard error at the indicated temperature.

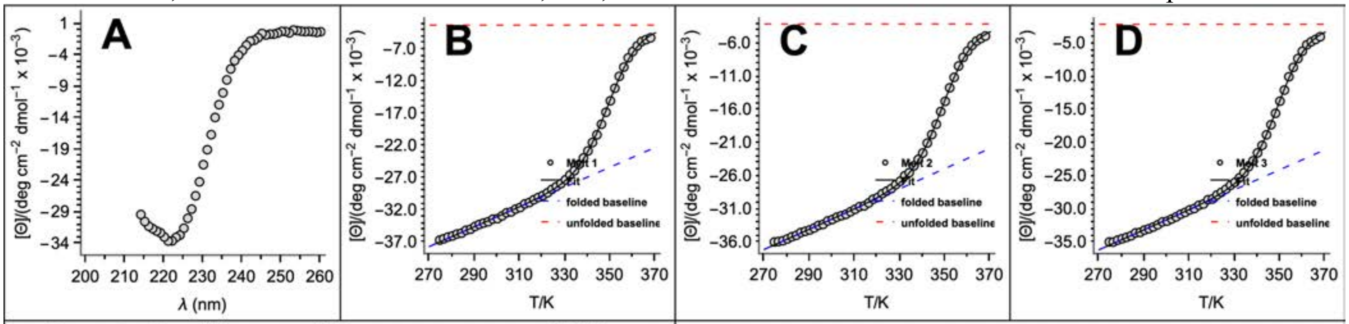

Parameters	Values	Units	P–Values
ΔH_0	−35.96 ± 0.48	kcal/mol	<0.001
T_m	77.5 ± 0.1	°C	---
ΔC_p	---	---	---
a1	−79.2 ± 0.6	deg cm^{-2} dmol^{-1} x 10^{-3}	<0.001
b1	0.154 ± 0.002	deg cm^{-2} dmol^{-1} K^{-1} x 10^{-3}	<0.001
c1	−3.25 ± 0.14	deg cm^{-2} dmol^{-1} x 10^{-3}	<0.001
d1	---	---	---
a2	−78.2 ± 0.6	deg cm^{-2} dmol^{-1} x 10^{-3}	<0.001
b2	0.152 ± 0.002	deg cm^{-2} dmol^{-1} K^{-1} x 10^{-3}	<0.001
c2	−3.06 ± 0.14	deg cm^{-2} dmol^{-1} x 10^{-3}	<0.001
d2	---	---	---
a3	−76.8 ± 0.6	deg cm^{-2} dmol^{-1} x 10^{-3}	<0.001
b3	0.151 ± 0.002	deg cm^{-2} dmol^{-1} K^{-1} x 10^{-3}	<0.001
c3	−2.10 ± 0.14	deg cm^{-2} dmol^{-1} x 10^{-3}	<0.001
d3	---	---	---

$R^2 = 0.99996$
sum residuals^2 = 4.22032

For **s6e/1g'–x4x**, at 321.8 K,
ΔG = −2.96 ± 0.04 kcal/mol
ΔH = −36.0 ± 0.5 kcal/mol
−TΔS = 33.0 ± 0.4 kcal/mol

Figure S74. (A) CD spectra and (B–D) variable temperature CD data (triplicate) for 15 μM disulfide-bound coiled-coil variant **s6e/1g'-x4x** (SH2019) in 20 mM sodium phosphate (pH 7) with 4 M GdnHCl. Fit parameters from eq. S1–S3 appear in the table, as do calculated values of ΔG_f, ΔH_f, and -$T\Delta S_f$ ± standard error at the indicated temperature.

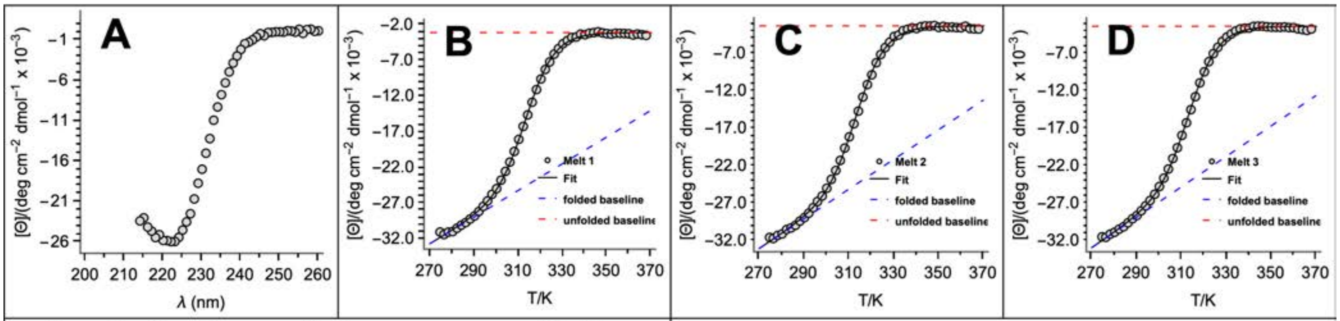

Parameters	Values	Units	P–Values
ΔH_0	−30.48 ± 0.41	kcal/mol	<0.001
T_m	41.8 ± 0.2	°C	<0.001
ΔC_p	---	---	---
a1	−83.2 ± 2.2	deg cm^{-2} dmol^{-1} x 10^{-3}	<0.001
b1	0.187 ± 0.008	deg cm^{-2} dmol^{-1} K^{-1} x 10^{-3}	<0.001
c1	−3.09 ± 0.05	deg cm^{-2} dmol^{-1} x 10^{-3}	<0.001
d1	---	---	---
a2	−86.6 ± 2.2	deg cm^{-2} dmol^{-1} x 10^{-3}	<0.001
b2	0.198 ± 0.008	deg cm^{-2} dmol^{-1} K^{-1} x 10^{-3}	<0.001
c2	−3.34 ± 0.05	deg cm^{-2} dmol^{-1} x 10^{-3}	<0.001
d2	---	---	---
a3	−87.8 ± 2.2	deg cm^{-2} dmol^{-1} x 10^{-3}	<0.001
b3	0.203 ± 0.008	deg cm^{-2} dmol^{-1} K^{-1} x 10^{-3}	<0.001
c3	−3.38 ± 0.05	deg cm^{-2} dmol^{-1} x 10^{-3}	<0.001
d3	---	---	---

R^2 = 0.99985
sum residuals^2 = 6.82415

For **13e/8g'–xx**, at 315.0 K,
ΔG = 0.00 ± 0.02 kcal/mol
ΔH = −30.5 ± 0.4 kcal/mol
−TΔS = 30.5 ± 0.4 kcal/mol

Figure S75. (A) CD spectra and (B–D) variable temperature CD data (triplicate) for 15 μM stapled disulfide-bound coiled-coil variant **13e/8g'-xx** (SH2016) in 20 mM sodium phosphate (pH 7) with 4 M GdnHCl. Fit parameters from eq. S1–S3 appear in the table, as do calculated values of ΔG_f, ΔH_f, and -$T\Delta S_f$ ± standard error at the indicated temperature.

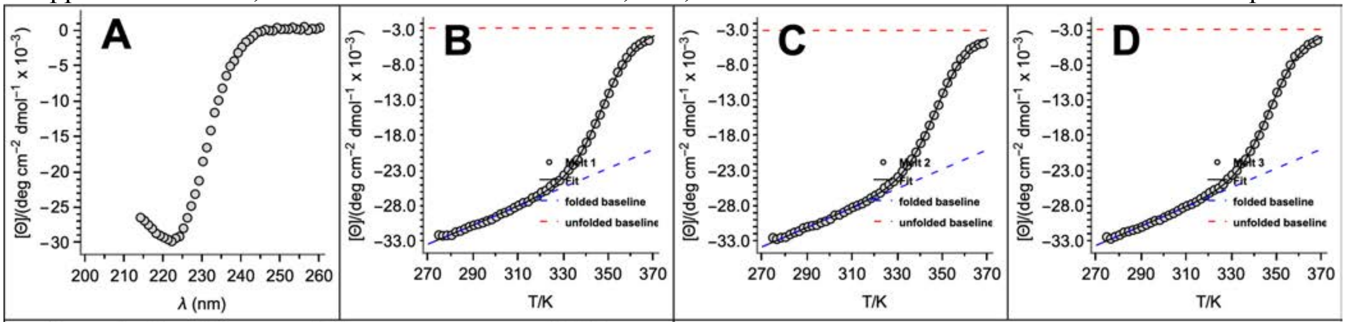

Parameters	Values	Units	P–Values
ΔH_0	−32.36 ± 0.50	kcal/mol	<0.001
T_m	75.6 ± 0.1	°C	<0.001
ΔC_p	---	---	---
a1	−69.6 ± 0.7	deg cm^{-2} dmol^{-1} x 10^{-3}	<0.001
b1	0.135 ± 0.002	deg cm^{-2} dmol^{-1} K^{-1} x 10^{-3}	<0.001
c1	−2.65 ± 0.15	deg cm^{-2} dmol^{-1} x 10^{-3}	<0.001
d1	---	---	---
a2	−70.9 ± 0.7	deg cm^{-2} dmol^{-1} x 10^{-3}	<0.001
b2	0.138 ± 0.002	deg cm^{-2} dmol^{-1} K^{-1} x 10^{-3}	<0.001
c2	−2.99 ± 0.15	deg cm^{-2} dmol^{-1} x 10^{-3}	<0.001
d2	---	---	---
a3	−70.4 ± 0.7	deg cm^{-2} dmol^{-1} x 10^{-3}	<0.001
b3	0.137 ± 0.002	deg cm^{-2} dmol^{-1} K^{-1} x 10^{-3}	<0.001
c3	−2.83 ± 0.15	deg cm^{-2} dmol^{-1} x 10^{-3}	<0.001
d3	---	---	---

R^2 = 0.99994
sum residuals^2 = 4.78685

For **s13e/8g'–x4x**, at 315.0 K,
ΔG = −3.13 ± 0.05 kcal/mol
ΔH = −32.4 ± 0.5 kcal/mol
−TΔS = 29.2 ± 0.5 kcal/mol

Figure S76. (A) CD spectra and (B–D) variable temperature CD data (triplicate) for 15 μM disulfide-bound coiled-coil variant **s13e/8g'-x4x** (SH2018) in 20 mM sodium phosphate (pH 7) with 4 M GdnHCl. Fit parameters from eq. S1–S3 appear in the table, as do calculated values of ΔG_f, ΔH_f, and -$T\Delta S_f$ ± standard error at the indicated temperature.

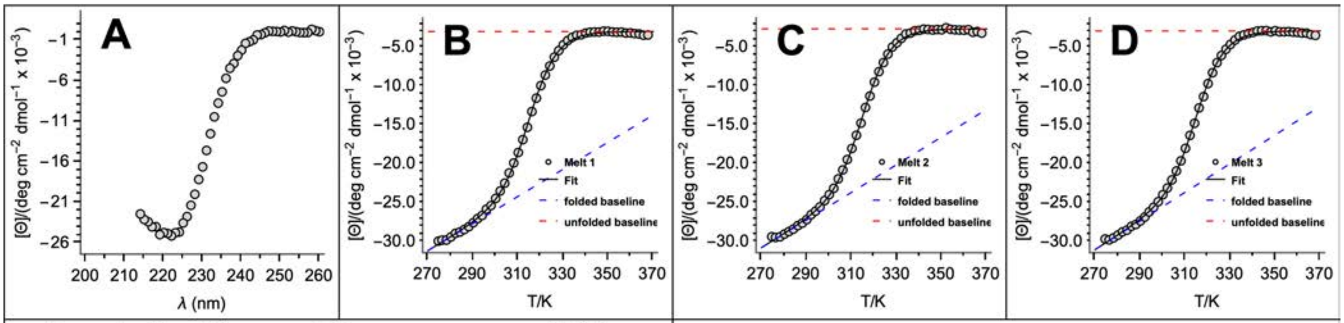

Parameters	Values	Units	P–Values
ΔH_0	−31.99 ± 0.42	kcal/mol	<0.001
T_m	43.2 ± 0.1	°C	<0.001
ΔC_p	---	---	---
a1	−78.1 ± 1.9	deg cm^{-2} dmol^{-1} x 10^{-3}	<0.001
b1	0.174 ± 0.007	deg cm^{-2} dmol^{-1} K^{-1} x 10^{-3}	<0.001
c1	−3.03 ± 0.05	deg cm^{-2} dmol^{-1} x 10^{-3}	<0.001
d1	---	---	---
a2	−78.7 ± 1.9	deg cm^{-2} dmol^{-1} x 10^{-3}	<0.001
b2	0.177 ± 0.007	deg cm^{-2} dmol^{-1} K^{-1} x 10^{-3}	<0.001
c2	−2.69 ± 0.05	deg cm^{-2} dmol^{-1} x 10^{-3}	<0.001
d2	---	---	---
a3	−80.8 ± 1.9	deg cm^{-2} dmol^{-1} x 10^{-3}	<0.001
b3	0.184 ± 0.007	deg cm^{-2} dmol^{-1} K^{-1} x 10^{-3}	<0.001
c3	−2.96 ± 0.05	deg cm^{-2} dmol^{-1} x 10^{-3}	<0.001
d3	---	---	---

R^2 = 0.99985
sum residuals^2 = 6.20323

For **20e/15g'−xx**, at 316.3 K,
ΔG = 0.00 ± 0.01 kcal/mol
ΔH = −32.0 ± 0.4 kcal/mol
−TΔS = 32.0 ± 0.4 kcal/mol

Figure S77. (A) CD spectra and (B–D) variable temperature CD data (triplicate) for 15 μM stapled disulfide-bound coiled-coil variant **20e/15g'-xx** (SH2036) in 20 mM sodium phosphate (pH 7) with 4 M GdnHCl. Fit parameters from eq. S1–S3 appear in the table, as do calculated values of ΔG_f, ΔH_f, and -TΔS_f ± standard error at the indicated temperature.

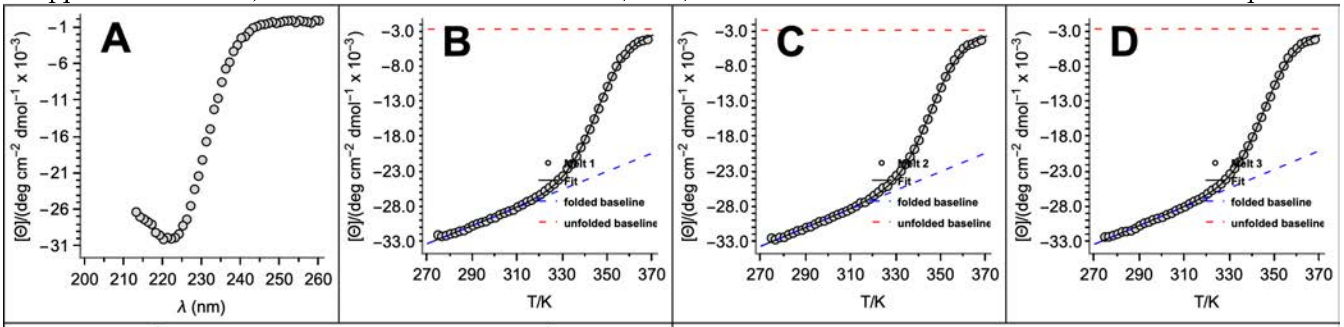

Parameters	Values	Units	P–Values
ΔH_0	−32.82 ± 0.43	kcal/mol	<0.001
T_m	73.8 ± 0.1	°C	---
ΔC_p	---	---	---
a1	−67.9 ± 0.6	deg cm^{-2} dmol^{-1} x 10^{-3}	<0.001
b1	0.129 ± 0.002	deg cm^{-2} dmol^{-1} K^{-1} x 10^{-3}	<0.001
c1	−2.65 ± 0.12	deg cm^{-2} dmol^{-1} x 10^{-3}	<0.001
d1	---	---	---
a2	−69.4 ± 0.6	deg cm^{-2} dmol^{-1} x 10^{-3}	<0.001
b2	0.133 ± 0.002	deg cm^{-2} dmol^{-1} K^{-1} x 10^{-3}	<0.001
c2	−2.80 ± 0.12	deg cm^{-2} dmol^{-1} x 10^{-3}	<0.001
d2	---	---	---
a3	−69.2 ± 0.6	deg cm^{-2} dmol^{-1} x 10^{-3}	<0.001
b3	0.133 ± 0.002	deg cm^{-2} dmol^{-1} K^{-1} x 10^{-3}	<0.001
c3	−2.61 ± 0.12	deg cm^{-2} dmol^{-1} x 10^{-3}	<0.001
d3	---	---	---

R^2 = 0.99995
sum residuals^2 = 4.05589

For **s20e/15g'−x4x**, at 315.8 K,
ΔG = −2.95 ± 0.04 kcal/mol
ΔH = −32.8 ± 0.4 kcal/mol
−TΔS = 29.9 ± 0.4 kcal/mol

Figure S78. (A) CD spectra and (B–D) variable temperature CD data (triplicate) for 15 μM disulfide-bound coiled-coil variant **s20e/15g'-x4x (**SH2037) in 20 mM sodium phosphate (pH 7) with 4 M GdnHCl. Fit parameters from eq. S1–S3 appear in the table, as do calculated values of ΔG_f, ΔH_f, and -TΔS_f ± standard error at the indicated temperature.

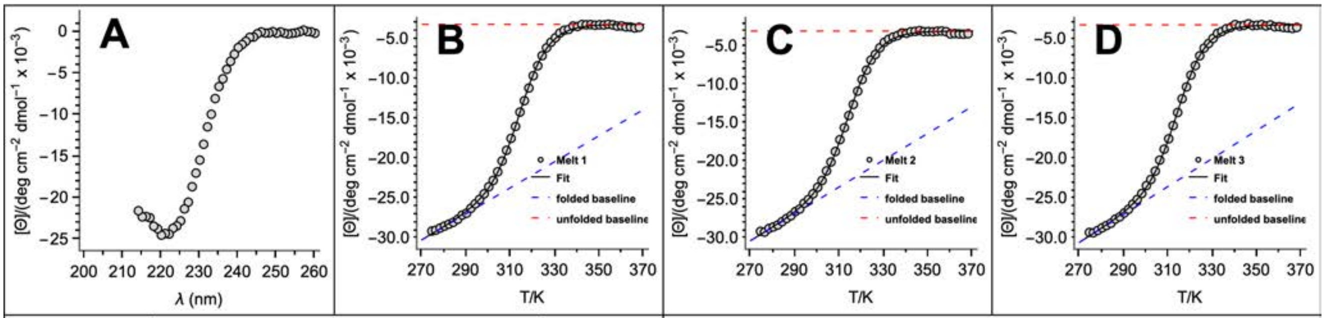

Parameters	Values	Units	P–Values
ΔH_0	−30.82 ± 0.39	kcal/mol	<0.001
T_m	42.5 ± 0.2	°C	<0.001
ΔC_p	---	---	---
a1	−74.4 ± 1.9	deg cm^{-2} dmol^{-1} x 10^{-3}	<0.001
b1	0.164 ± 0.007	deg cm^{-2} dmol^{-1} K^{-1} x 10^{-3}	<0.001
c1	−3.14 ± 0.05	deg cm^{-2} dmol^{-1} x 10^{-3}	<0.001
d1	---	---	---
a2	−77.6 ± 1.9	deg cm^{-2} dmol^{-1} x 10^{-3}	<0.001
b2	0.175 ± 0.007	deg cm^{-2} dmol^{-1} K^{-1} x 10^{-3}	<0.001
c2	−3.03 ± 0.05	deg cm^{-2} dmol^{-1} x 10^{-3}	<0.001
d2	---	---	---
a3	−78.0 ± 1.9	deg cm^{-2} dmol^{-1} x 10^{-3}	<0.001
b3	0.176 ± 0.007	deg cm^{-2} dmol^{-1} K^{-1} x 10^{-3}	<0.001
c3	−3.21 ± 0.05	deg cm^{-2} dmol^{-1} x 10^{-3}	<0.001
d3	---	---	---

$R^2 = 0.99987$
sum residuals^2 = 5.41775

For **27e/22g'–xx**, at 315.7 K,
$\Delta G = 0.00 \pm 0.01$ kcal/mol
$\Delta H = -30.8 \pm 0.4$ kcal/mol
$-T\Delta S = 30.8 \pm 0.4$ kcal/mol

Figure S79. (A) CD spectra and (B–D) variable temperature CD data (triplicate) for 15 μM disulfide-bound coiled-coil variant **27e/22g'-xx** (SH2024) in 20 mM sodium phosphate (pH 7) with 4 M GdnHCl. Fit parameters from eq. S1–S3 appear in the table, as do calculated values for ΔG_f, ΔH_f, and -$T\Delta S_f$ ± standard error at the indicated temperature.

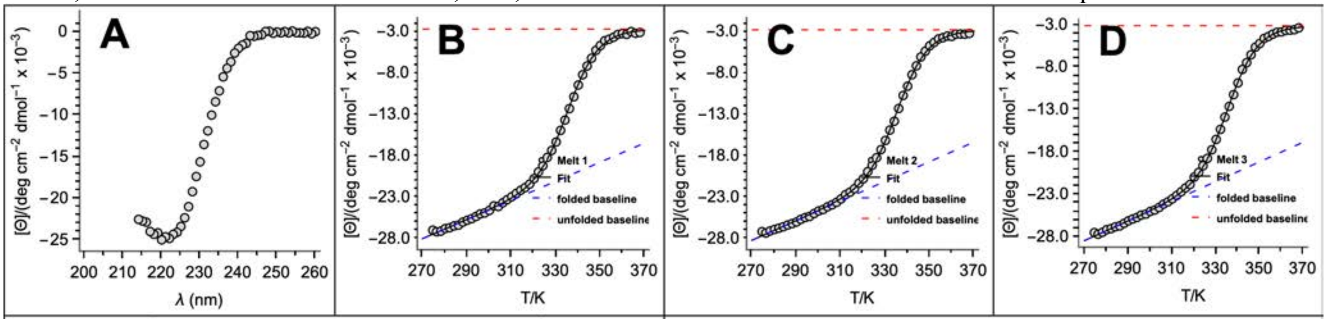

Parameters	Values	Units	P–Values
ΔH_0	−32.93 ± 0.38	kcal/mol	<0.001
T_m	63.7 ± 0.1	°C	---
ΔC_p	---	---	---
a1	−59.2 ± 0.7	deg cm^{-2} dmol^{-1} x 10^{-3}	<0.001
b1	0.115 ± 0.002	deg cm^{-2} dmol^{-1} K^{-1} x 10^{-3}	<0.001
c1	−2.70 ± 0.06	deg cm^{-2} dmol^{-1} x 10^{-3}	<0.001
d1	---	---	---
a2	−60.2 ± 0.7	deg cm^{-2} dmol^{-1} x 10^{-3}	<0.001
b2	0.119 ± 0.002	deg cm^{-2} dmol^{-1} K^{-1} x 10^{-3}	<0.001
c2	−2.80 ± 0.06	deg cm^{-2} dmol^{-1} x 10^{-3}	<0.001
d2	---	---	---
a3	−59.8 ± 0.7	deg cm^{-2} dmol^{-1} x 10^{-3}	<0.001
b3	0.116 ± 0.002	deg cm^{-2} dmol^{-1} K^{-1} x 10^{-3}	<0.001
c3	−3.13 ± 0.06	deg cm^{-2} dmol^{-1} x 10^{-3}	<0.001
d3	---	---	---

$R^2 = 0.99993$
sum residuals^2 = 3.45792

For **s27e/22g'–x4x**, at 315.7 K,
$\Delta G = -2.07 \pm 0.03$ kcal/mol
$\Delta H = -32.9 \pm 0.4$ kcal/mol
$-T\Delta S = 30.9 \pm 0.4$ kcal/mol

Figure S80. (A) CD spectra and (B–D) variable temperature CD data (triplicate) for 15 μM disulfide-bound coiled-coil variant **s27e/22g'-x4x** (SH2027) in 20 mM sodium phosphate (pH 7) with 4 M GdnHCl. Fit parameters from eq. S1–S3 appear in the table, as do calculated values of ΔG_f, ΔH_f, and -$T\Delta S_f$ ± standard error at the indicated temperature.

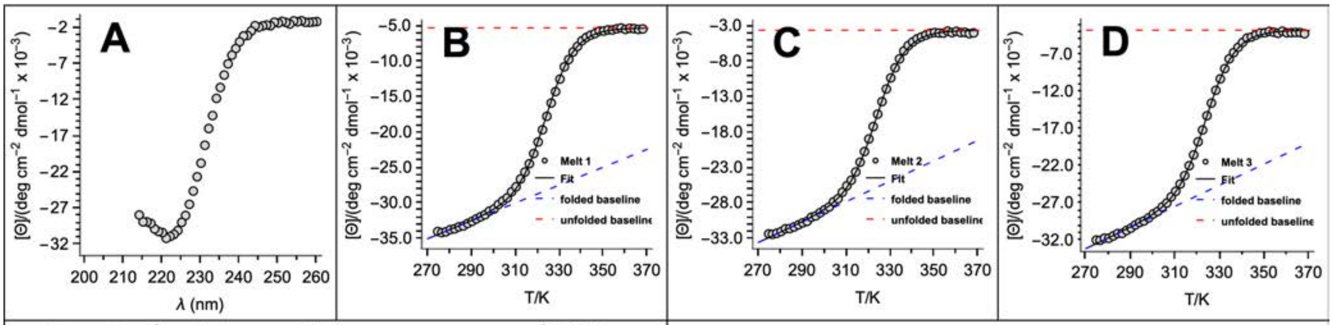

Parameters	Values	Units	P–Values
ΔH_0	−32.76 ± 0.34	kcal/mol	<0.001
T_m	51.8 ± 0.1	°C	---
ΔC_p	---	---	---
a1	−69.1 ± 1.2	deg cm^{-2} dmol^{-1} x 10^{-3}	<0.001
b1	0.126 ± 0.004	deg cm^{-2} dmol^{-1} K^{-1} x 10^{-3}	<0.001
c1	−5.27 ± 0.05	deg cm^{-2} dmol^{-1} x 10^{-3}	<0.001
d1	---	---	---
a2	−72.5 ± 1.2	deg cm^{-2} dmol^{-1} x 10^{-3}	<0.001
b2	0.144 ± 0.004	deg cm^{-2} dmol^{-1} K^{-1} x 10^{-3}	<0.001
c2	−3.60 ± 0.05	deg cm^{-2} dmol^{-1} x 10^{-3}	<0.001
d2	---	---	---
a3	−71.5 ± 1.2	deg cm^{-2} dmol^{-1} x 10^{-3}	<0.001
b3	0.142 ± 0.004	deg cm^{-2} dmol^{-1} K^{-1} x 10^{-3}	<0.001
c3	−3.75 ± 0.05	deg cm^{-2} dmol^{-1} x 10^{-3}	<0.001
d3	---	---	---

R^2 = 0.99993
sum residuals^2 = 4.82343

For **34e/29g'–xx**, at 325.0 K,
ΔG = 0.00 ± 0.01 kcal/mol
ΔH = −32.8 ± 0.3 kcal/mol
−TΔS = 32.8 ± 0.3 kcal/mol

Figure S81. (A) CD spectra and (B–D) variable temperature CD data (triplicate) for 15 µM stapled disulfide-bound coiled-coil variant **34e/29g'-xx** (SH2021) in 20 mM sodium phosphate (pH 7) with 4 M GdnHCl. Fit parameters from eq. S1–S3 appear in the table, as do calculated values of ΔG_f, ΔH_f, and -$T\Delta S_f$ ± standard error at the indicated temperature.

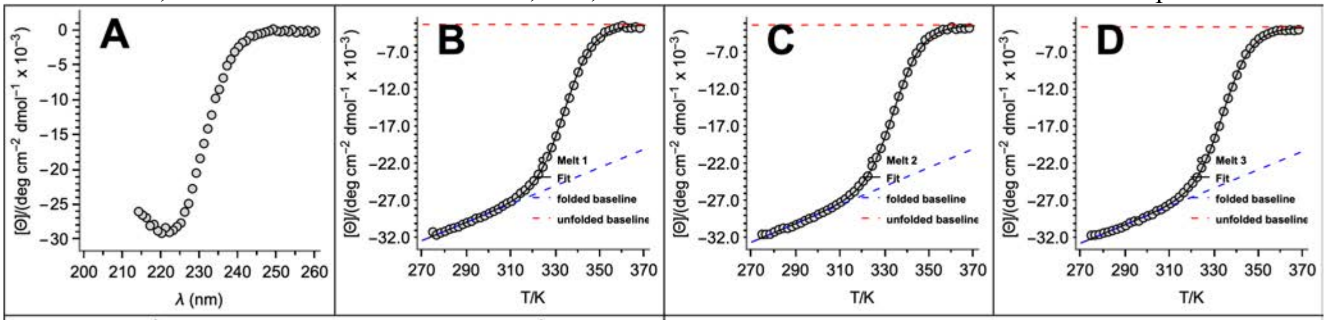

Parameters	Values	Units	P–Values
ΔH_0	−35.50 ± 0.36	kcal/mol	<0.001
T_m	61.9 ± 0.1	°C	---
ΔC_p	---	---	---
a1	−65.5 ± 0.8	deg cm^{-2} dmol^{-1} x 10^{-3}	<0.001
b1	0.123 ± 0.003	deg cm^{-2} dmol^{-1} K^{-1} x 10^{-3}	<0.001
c1	−3.16 ± 0.06	deg cm^{-2} dmol^{-1} x 10^{-3}	<0.001
d1	---	---	---
a2	−66.5 ± 0.8	deg cm^{-2} dmol^{-1} x 10^{-3}	<0.001
b2	0.126 ± 0.003	deg cm^{-2} dmol^{-1} K^{-1} x 10^{-3}	<0.001
c2	−3.24 ± 0.06	deg cm^{-2} dmol^{-1} x 10^{-3}	<0.001
d2	---	---	---
a3	−66.0 ± 0.8	deg cm^{-2} dmol^{-1} x 10^{-3}	<0.001
b3	0.124 ± 0.003	deg cm^{-2} dmol^{-1} K^{-1} x 10^{-3}	<0.001
c3	−3.51 ± 0.06	deg cm^{-2} dmol^{-1} x 10^{-3}	<0.001
d3	---	---	---

R^2 = 0.99994
sum residuals^2 = 4.13955

For **s34e/29g'–x4x**, at 325.0 K,
ΔG = −1.07 ± 0.01 kcal/mol
ΔH = −35.5 ± 0.4 kcal/mol
−TΔS = 34.4 ± 0.3 kcal/mol

Figure S82. (A) CD spectra and (B–D) variable temperature CD data (triplicate) for 15 µM disulfide-bound coiled-coil variant **s34e/29g'-x4x** (SH2023) in 20 mM sodium phosphate (pH 7) with 4 M GdnHCl. Fit parameters from equations S1–S3 appear in the table, as do calculated values for ΔG_f, ΔH_f, and -$T\Delta S_f$ at ± standard error at the indicated temperature.

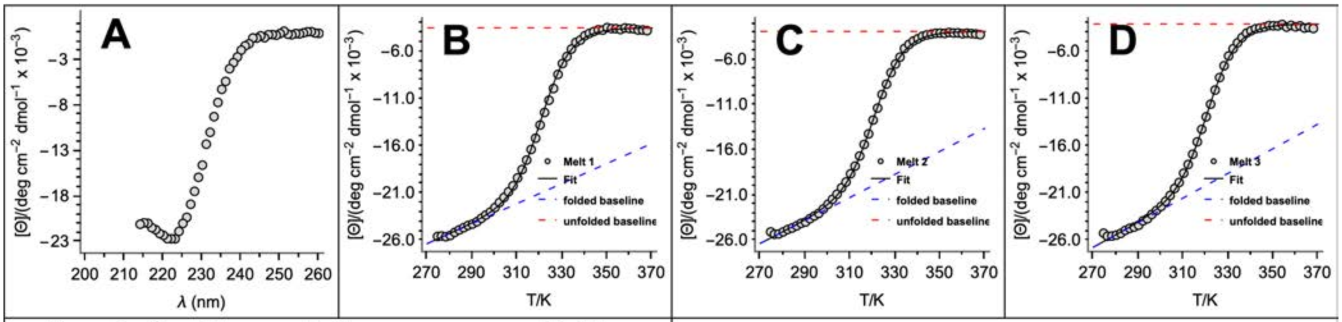

Parameters	Values	Units	P–Values
ΔH_0	−31.79 ± 0.49	kcal/mol	<0.001
T_m	48.9 ± 0.2	°C	<0.001
ΔC_p	---	---	---
a1	−55.0 ± 1.5	deg cm^{-2} dmol^{-1} x 10^{-3}	<0.001
b1	0.106 ± 0.005	deg cm^{-2} dmol^{-1} K^{-1} x 10^{-3}	<0.001
c1	−3.48 ± 0.06	deg cm^{-2} dmol^{-1} x 10^{-3}	<0.001
d1	---	---	---
a2	−60.7 ± 1.5	deg cm^{-2} dmol^{-1} x 10^{-3}	<0.001
b2	0.127 ± 0.005	deg cm^{-2} dmol^{-1} K^{-1} x 10^{-3}	<0.001
c2	−2.89 ± 0.06	deg cm^{-2} dmol^{-1} x 10^{-3}	<0.001
d2	---	---	---
a3	−62.0 ± 1.5	deg cm^{-2} dmol^{-1} x 10^{-3}	<0.001
b3	0.131 ± 0.005	deg cm^{-2} dmol^{-1} K^{-1} x 10^{-3}	<0.001
c3	−3.06 ± 0.06	deg cm^{-2} dmol^{-1} x 10^{-3}	<0.001
d3	---	---	---

R^2 = 0.99983
sum residuals^2 = 6.26341

For **7f/10b'–xx**, at 322.1 K,
ΔG = 0.00 ± 0.02 kcal/mol
ΔH = −31.8 ± 0.5 kcal/mol
−TΔS = 31.8 ± 0.5 kcal/mol

Figure S83. (A) CD spectra and (B–D) variable temperature CD data (triplicate) for 15 μM stapled disulfide-bound coiled-coil variant **7f/10b'-xx** (LH1029) in 20 mM sodium phosphate (pH 7) with 4 M GdnHCl. Fit parameters from equations S1–S3 appear in the table, as do calculated values for ΔG_f, ΔH_f, and -TΔS_f ± standard errors at the indicated temperature.

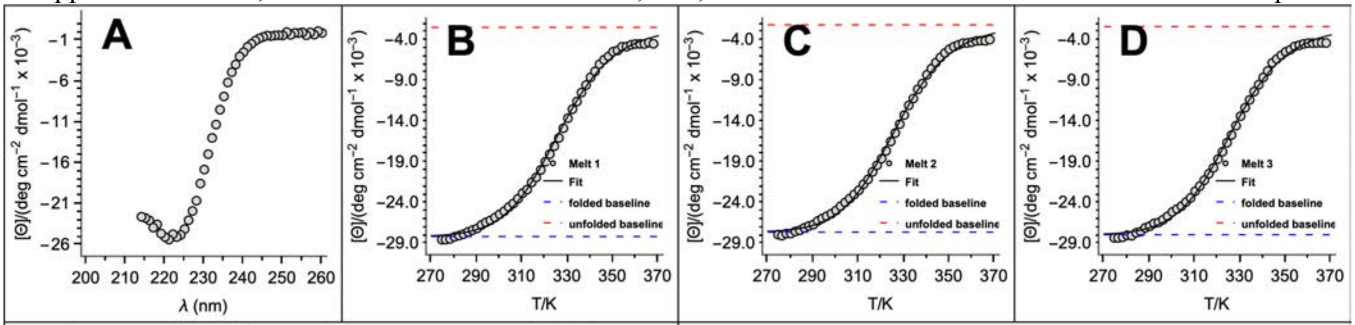

Parameters	Values	Units	P–Values
ΔH_0	−17.43 ± 0.31	kcal/mol	<0.001
T_m	53.3 ± 0.2	°C	<0.001
ΔC_p	---	---	---
a1	−28.1 ± 0.1	deg cm^{-2} dmol^{-1} x 10^{-3}	<0.001
b1	---	---	---
c1	−2.46 ± 0.19	deg cm^{-2} dmol^{-1} x 10^{-3}	<0.001
d1	---	---	---
a2	−27.6 ± 0.1	deg cm^{-2} dmol^{-1} x 10^{-3}	<0.001
b2	---	---	---
c2	−2.15 ± 0.18	deg cm^{-2} dmol^{-1} x 10^{-3}	<0.001
d2	---	---	---
a3	−27.9 ± 0.1	deg cm^{-2} dmol^{-1} x 10^{-3}	<0.001
b3	---	---	---
c3	−2.36 ± 0.18	deg cm^{-2} dmol^{-1} x 10^{-3}	<0.001
d3	---	---	---

R^2 = 0.99958
sum residuals^2 = 21.69073

For **s7f/10b'–x4x**, at 322.1 K,
ΔG = −0.23 ± 0.01 kcal/mol
ΔH = −17.4 ± 0.3 kcal/mol
−TΔS = 17.2 ± 0.3 kcal/mol

Figure S84. (A) CD spectra and (B–D) variable temperature CD data (triplicate) for 15 μM disulfide-bound coiled-coil variant **s7f/10b'-x4x (**SH1119) in 20 mM sodium phosphate (pH 7) with 4 M GdnHCl. Fit parameters from equations S1–S3 appear in the table, as do calculated values for ΔG_f, ΔH_f, and -TΔS_f ± standard error at the indicated temperature.

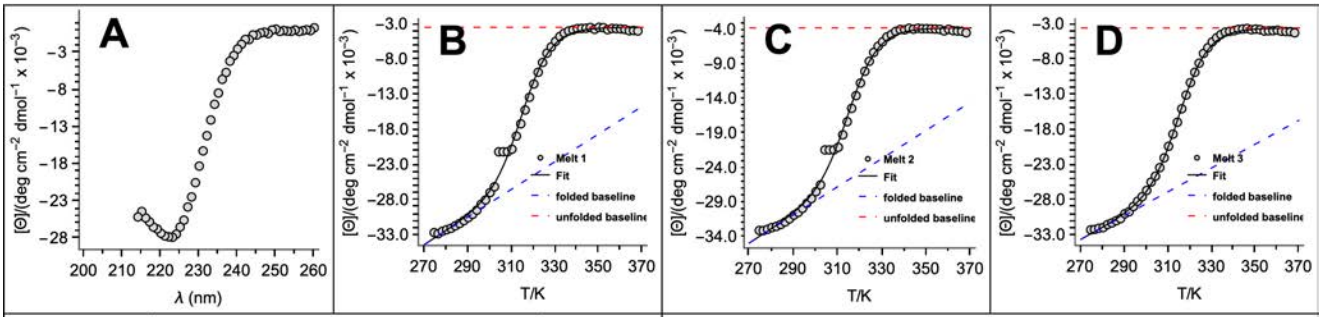

Parameters	Values	Units	P–Values
ΔH_0	−29.88 ± 1.06	kcal/mol	<0.001
T_m	43.2 ± 0.4	°C	<0.001
ΔC_p	---	---	---
a1	−87.5 ± 5.8	deg cm^{-2} dmol^{-1} x 10^{-3}	<0.001
b1	0.197 ± 0.020	deg cm^{-2} dmol^{-1} K^{-1} x 10^{-3}	<0.001
c1	−3.44 ± 0.15	deg cm^{-2} dmol^{-1} x 10^{-3}	<0.001
d1	---	---	---
a2	−90.0 ± 5.8	deg cm^{-2} dmol^{-1} x 10^{-3}	<0.001
b2	0.204 ± 0.020	deg cm^{-2} dmol^{-1} K^{-1} x 10^{-3}	<0.001
c2	−3.73 ± 0.15	deg cm^{-2} dmol^{-1} x 10^{-3}	<0.001
d2	---	---	---
a3	−79.3 ± 5.8	deg cm^{-2} dmol^{-1} x 10^{-3}	<0.001
b3	0.169 ± 0.020	deg cm^{-2} dmol^{-1} K^{-1} x 10^{-3}	<0.001
c3	−3.54 ± 0.16	deg cm^{-2} dmol^{-1} x 10^{-3}	<0.001
d3	---	---	---

R^2 = 0.99900
sum residuals^2 = 52.75047

For **14f/17b'–xx**, at 316.4 K,
ΔG = 0.00 ± 0.04 kcal/mol
ΔH = −29.9 ± 1.1 kcal/mol
−TΔS = 29.9 ± 1.1 kcal/mol

Figure S85. (A) CD spectra and (B–D) variable temperature CD data (triplicate) for 15 µM stapled disulfide-bound coiled-coil variant **14f/17b'-xx** (KS1011) in 20 mM sodium phosphate (pH 7) with 4 M GdnHCl. Fit parameters from equations S1–S3 appear in the table, as do calculated values for ΔG_f, ΔH_f, and -$T\Delta S_f$ ± standard error at the indicated temperature.

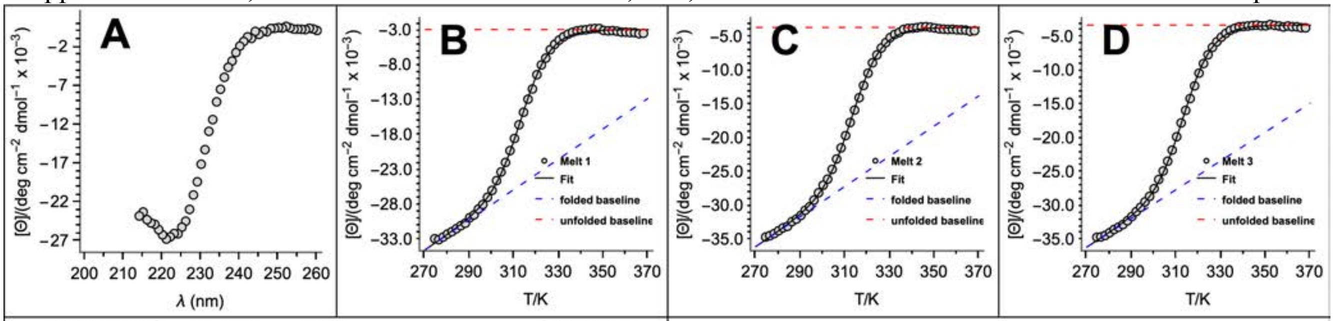

Parameters	Values	Units	P–Values
ΔH_0	−30.88 ± 0.45	kcal/mol	<0.001
T_m	41.9 ± 0.2	°C	<0.001
ΔC_p	---	---	---
a1	−93.2 ± 2.5	deg cm^{-2} dmol^{-1} x 10^{-3}	<0.001
b1	0.217 ± 0.009	deg cm^{-2} dmol^{-1} K^{-1} x 10^{-3}	<0.001
c1	−2.89 ± 0.06	deg cm^{-2} dmol^{-1} x 10^{-3}	<0.001
d1	---	---	---
a2	−96.3 ± 2.5	deg cm^{-2} dmol^{-1} x 10^{-3}	<0.001
b2	0.223 ± 0.009	deg cm^{-2} dmol^{-1} K^{-1} x 10^{-3}	<0.001
c2	−3.61 ± 0.06	deg cm^{-2} dmol^{-1} x 10^{-3}	<0.001
d2	---	---	---
a3	−93.6 ± 2.6	deg cm^{-2} dmol^{-1} x 10^{-3}	<0.001
b3	0.213 ± 0.009	deg cm^{-2} dmol^{-1} K^{-1} x 10^{-3}	<0.001
c3	−3.26 ± 0.06	deg cm^{-2} dmol^{-1} x 10^{-3}	<0.001
d3	---	---	---

R^2 = 0.99983
sum residuals^2 = 9.20516

For **21f/24b'–xx**, at 315.1 K,
ΔG = 0.00 ± 0.02 kcal/mol
ΔH = −30.9 ± 0.4 kcal/mol
−TΔS = 30.9 ± 0.4 kcal/mol

Figure S86. (A) CD spectra and (B–D) variable temperature CD data (triplicate) for 15 µM stapled disulfide-bound coiled-coil variant **21f/24b'-xx** (SH2012) in 20 mM sodium phosphate (pH 7) with 4 M GdnHCl. Fit parameters from eq. S1–S3 appear in the table, as do calculated values for ΔG_f, ΔH_f, and -$T\Delta S_f$ ± standard error at the indicated temperature.

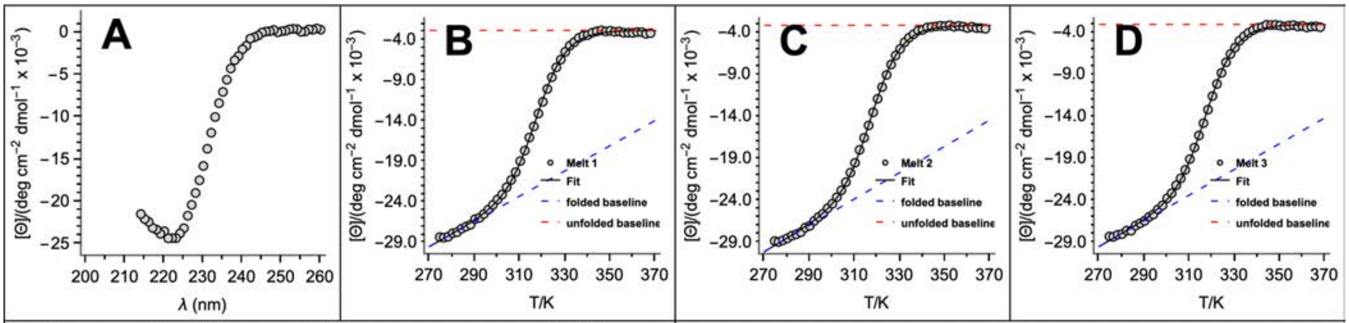

Parameters	Values	Units	P–Values
ΔH_0	−30.99 ± 0.39	kcal/mol	<0.001
T_m	45.7 ± 0.1	°C	<0.001
ΔC_p	---	---	---
a1	−71.5 ± 1.6	deg cm^{-2} dmol^{-1} x 10^{-3}	<0.001
b1	0.156 ± 0.006	deg cm^{-2} dmol^{-1} K^{-1} x 10^{-3}	<0.001
c1	−2.85 ± 0.05	deg cm^{-2} dmol^{-1} x 10^{-3}	<0.001
d1	---	---	---
a2	−72.4 ± 1.6	deg cm^{-2} dmol^{-1} x 10^{-3}	<0.001
b2	0.157 ± 0.006	deg cm^{-2} dmol^{-1} K^{-1} x 10^{-3}	<0.001
c2	−3.21 ± 0.05	deg cm^{-2} dmol^{-1} x 10^{-3}	<0.001
d2	---	---	---
a3	−70.7 ± 1.6	deg cm^{-2} dmol^{-1} x 10^{-3}	<0.001
b3	0.153 ± 0.005	deg cm^{-2} dmol^{-1} K^{-1} x 10^{-3}	<0.001
c3	−3.10 ± 0.05	deg cm^{-2} dmol^{-1} x 10^{-3}	<0.001
d3	---	---	---

$R^2 = 0.99988$
sum residuals^2 = 5.15555

For **28f/31b'–xx**, at 318.9 K,
$\Delta G = 0.00 \pm 0.01$ kcal/mol
$\Delta H = -31.0 \pm 0.4$ kcal/mol
$-T\Delta S = 31.0 \pm 0.4$ kcal/mol

Figure S87. (A) CD spectra and (B–D) variable temperature CD data (triplicate) for 15 μM stapled disulfide-bound coiled-coil variant **28f/31b'-xx** (SH2025) in 20 mM sodium phosphate (pH 7) with 4 M GdnHCl. Fit parameters from equations S1–S3 appear in the table, as do calculated values for ΔG_f, ΔH_f, and $-T\Delta S_f$ ± standard error at the indicated temperature.

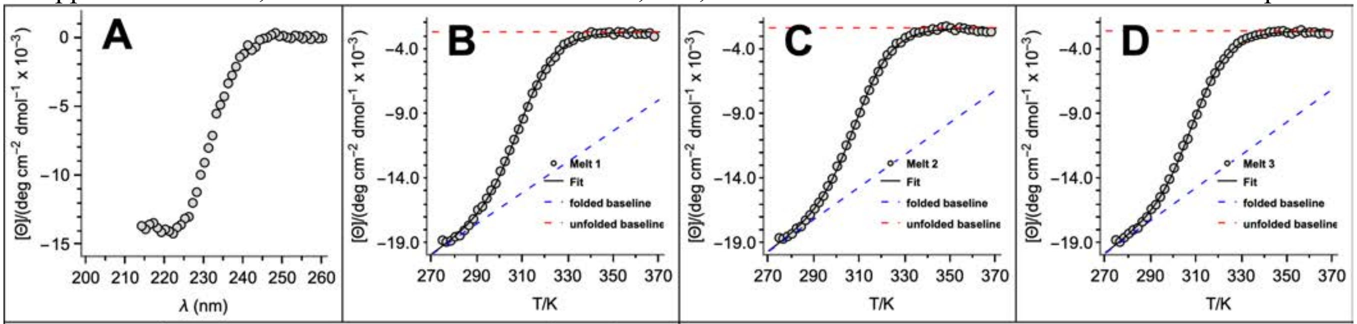

Parameters	Values	Units	P–Values
ΔH_0	−24.38 ± 0.39	kcal/mol	<0.001
T_m	37.8 ± 0.3	°C	<0.001
ΔC_p	---	---	---
a1	−52.1 ± 2.2	deg cm^{-2} dmol^{-1} x 10^{-3}	<0.001
b1	0.120 ± 0.008	deg cm^{-2} dmol^{-1} K^{-1} x 10^{-3}	<0.001
c1	−2.64 ± 0.04	deg cm^{-2} dmol^{-1} x 10^{-3}	<0.001
d1	---	---	---
a2	−53.3 ± 2.2	deg cm^{-2} dmol^{-1} x 10^{-3}	<0.001
b2	0.125 ± 0.008	deg cm^{-2} dmol^{-1} K^{-1} x 10^{-3}	<0.001
c2	−2.33 ± 0.04	deg cm^{-2} dmol^{-1} x 10^{-3}	<0.001
d2	---	---	---
a3	−54.0 ± 2.1	deg cm^{-2} dmol^{-1} x 10^{-3}	<0.001
b3	0.127 ± 0.008	deg cm^{-2} dmol^{-1} K^{-1} x 10^{-3}	<0.001
c3	−2.56 ± 0.04	deg cm^{-2} dmol^{-1} x 10^{-3}	<0.001
d3	---	---	---

$R^2 = 0.99981$
sum residuals^2 = 2.90808

For **s28f/31b'–x4x**, at 318.9 K,
$\Delta G = 0.62 \pm 0.03$ kcal/mol
$\Delta H = -24.4 \pm 0.4$ kcal/mol
$-T\Delta S = 25.0 \pm 0.4$ kcal/mol

Figure S88. (A) CD spectra and (B–D) variable temperature CD data (triplicate) for 15 μM stapled disulfide-bound coiled-coil variant **s28f/31b'-x4x** (SH2039) in 20 mM sodium phosphate (pH 7) with 4 M GdnHCl. Fit parameters from equations S1–S3 appear in the table, as do calculated values for ΔG_f, ΔH_f, and $-T\Delta S_f$ ± standard error at the indicated temperature.

(return to Table of Contents)

6. Building molecular dynamics models of stapled coiled-coil variants and their non-stapled counterparts

We built models of stapled coiled-coil variants **s6e/1g'-x4x**, **s13e/8g'-x4x**, **s20e/15g'-x4x**, **s27e/22g'-x4x**, **s34e/29g'-x4x**, **s7f/10b'-x4x, s14f/17b'-x4x, s21f/24b'-x4x,** and **s28f/31b'-x4x**; for their non-stapled counterparts variants **6e/1g'-xx**, **13e/8g'-xx**, **20e/15g'-xx**, **27e/22g'-xx**, **34e/29g'-xx**, **7f/10b'-xx**, **14f/17b'-xx**, **21f/24b'-xx**, and **28f/31b'-xx** in GaussView6[3], starting with the crystal structure of the parent coiled coil **A/B** (see main text Figure 1, PDB ID: 1kd9). There are three disulfide-bound **A/B** coiled coils in the asymmetric unit of this crystal structure; we chose one of these as our starting point. We used pdb4amber and reduce (AMBER 20[6]) to add missing heavy atoms, remove crystallographic water molecules, and add hydrogens at appropriate locations. We used the previously optimized structure of ACE-PRB-NME_PZ4_ACE-PRB-NME (Figure S89) the bis-N-methyl and bis-N'-acetyl carboxamide version of staple **x4x**; Figure S10), as a starting point for the **x4x** portion of our stapled variants.[4] The three-letter codes PRB and PZ4 refer, respectively, to the propargylglycine **x** and bis-azido PEG **4** segment of ACE-PRB-NME_PZ4_ACE-PRB-NME. This staple was optimized and procedure can be found in our previously submitted paper.

We used GaussView6[3] to modify the starting PDB file for **A/B**, placing PRB residues at the appropriate locations **6e/1g'-xx**, **13e/8g'-xx**, **20e/15g'-xx**, **27e/22g'-xx**, **34e/29g'-xx**, **7f/10b'-xx**, **14f/17b'-xx**, **21f/24b'-xx**, and **28f/31b'-xx**; placing the stapled PRB residues and PZ4 segment at the appropriate locations in **s6e/1g'-x4x**, **s13e/8g'-x4x**, **s20e/15g'-x4x**, **s27e/22g'-x4x**, **s34e/29g'-x4x**, **s7f/10b'-x4x, s14f/17b'-x4x, s21f/24b'-x4x,** and **s28f/31b'-x4x**. Using variant **s27e/22g'-x4x** as an example, this can be accomplished most easily by (1) opening the PDB file for the **A/B** coiled coil in GaussView6[3]; (2) deleting the unnecessary side-chain atoms (Cβ and beyond) at staple sites 27e and 22g'; (3) opening the *.chk file for one of the optimized conformations of model staple ACE-PRB-NME_PZ4_ACE-PRB-NME in GaussView6; (4) copying and pasting the entire structure of model staple ACE-PRB-NME_PZ4_ACE-PRB-NME into the Molecule Group window that contains the coiled coil; (5) deleting the ACE and NME segments of the model staple, along with the backbone atoms of the two PRB residues; (6) making a new single bond between Cβ of the first PRB residue of the model staple and Cα of coiled-coil position 27e; (7) making a new single bond between Cβ of the second PRB residue of the model staple and Cα of coiled-coil position 22g'; (8) using the "clean" function of GaussView6 to do a quick molecular mechanics minimization to correct unrealistic bond angles/lengths; and (9) using the "save as" function of GaussView6 to save the resulting structure as a PDB file. We then used a text editor to edit this PDB file such that (1) all atoms within the PRB residue at coiled-coil position 27e were grouped together with the correct atom names (see Figure S95), residue name PRB, chain identifier A, and residue number 27; (2) all atoms within the PRB residue at coiled-coil position 22g' were grouped together with the correct atom names, residue name PRB, chain identifier B, and residue number 22; and (3) all atoms within the PZ4 segment linking the two PRB residues were grouped together with correct atom names, residue name PZ4, chain identifier C,

and residue number 1. Doing this necessarily required cutting and pasting atoms such that their atom numbers were out of order; however, correct atom ordering can be restored by opening the edited PDB file in GaussView6[3], then using the "save as" function to re-save the PDB file. We used *pdb4amber* and *reduce* (AMBER 20[6]) prepare the final edited PDB files for *tleap*. We then used *tleap* to prepare the appropriate parameter/topology and initial coordinate (or restart) files for coiled-coil variants **s6e/1g'-x4x**, **s13e/8g'-x4x**, **s20e/15g'-x4x**, **s27e/22g'-x4x**, **s34e/29g'-x4x**, **s7f/10b'-x4x, s14f/17b'-x4x, s21f/24b'-x4x,** and **s28f/31b'-x4x**; for their non-stapled counterparts variants **6e/1g'-xx**, **13e/8g'-xx**, **20e/15g'-xx**, **27e/22g'-xx**, **34e/29g'-xx**, **7f/10b'-xx**, **14f/17b'-xx**, **21f/24b'-xx**, and **28f/31b'-xx** using the Amber ff19SB5[5] and GAFF27[7] force fields and the appropriate force-field modification and library files that we generated previously[4] for the PRB residues and PZ4 segment of the **x4x** staple. For the stapled variants, we used the "bond" command of *tleap* to specify the appropriate connections between the PRB residues and the PZ4 segment of the **x4x** staple (CG of PRB1 connected to NF of PZ4; CD of PRB1 connected to NG of PZ4; CG of PRB2 connected to NR of PZ4; CD of PRB2 connected to NO of PZ4, Figure S95).

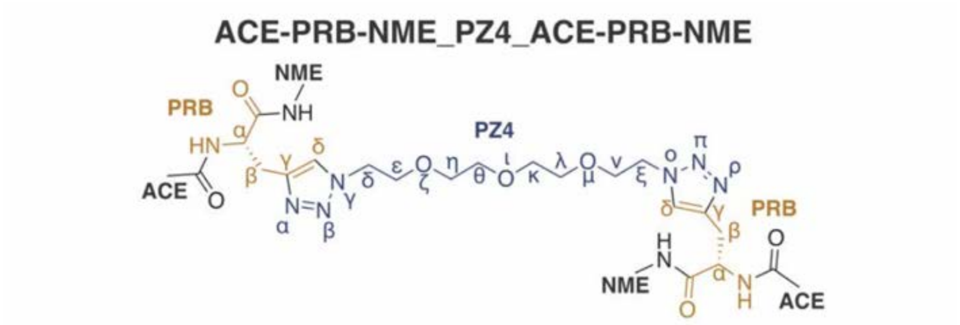

Figure S89. Model structure for staple ACE-PRB-NME_PZ4_ACE-PRB-NME. Atoms in this structure are named according to standard Roman alphabet equivalencies for Greek letters, except for Nα and Cλ, which were given the names NF and CV to avoid the potential confusion with sodium and chlorine atoms that would occur with the prospective abbreviations NA and CL.

(return to Table of Contents)

7. Molecular dynamics simulations of stapled coiled-coil variants and their non-stapled counterparts

Molecular dynamics (MD) simulations were performed with either *pmemd.cuda* or *pmemd.MPI* (AMBER 20[6]) on the CPUs or GPUs of the BYU Office of Research Computing, using the generalized Born implicit solvent model for water[8] (OBC; igb=2); the Langevin thermostat, with collision frequency $\gamma = 1.0\ ps^{-1}$; the SHAKE algorithm[9] to constrain stretching of bonds between heavy atoms and hydrogen; a time step of 2 fs; and a 1000 Å cutoff for non-bonded interactions. MD simulations proceeded in the following stages:

1. **Minimization**: performed on one GPU with *pmemd.cuda*; 1000 cycles via the steepest descent method, followed by a maximum of 4000 additional cycles via the conjugate gradient method, with coordinates of backbone atoms N, CA, C, and O restrained using a harmonic potential of the form $k(\Delta x)^2$, where Δx is the difference between the coordinate of a restrained atom and that of its reference position, and k = 10 kcal/mol/$Å^2$
2. **Heating**: performed in parallel for sixteen different target temperatures (310.00, 317.80, 325.79, 333.99, 342.39, 351.00, 359.83, 368.89, 378.17, 387.68, 397.43, 407.43, 417.68, 428.19, 438.96, or 450.00 K) using *pmemd.MPI* on eight CPUs per heating run; temperature increased from 10 K to the target temperature over 0.6 ns, then held constant at target temperature for 2.4 ns, with coordinates of backbone atoms N, CA, C, and O restrained as described above with k = 1 kcal/mol/$Å^2$
3. **Restrained Equilibration**: performed in parallel for the sixteen target temperatures listed above using *pmemd.MPI* on eight CPUs per equilibration run; temperature held constant for 10 ns, with coordinates of backbone atoms N, CA, C, and O restrained as described above with k = 0.1 kcal/mol/$Å^2$
4. **Replica-Exchange Equilibration**: performed in parallel using *pmemd.MPI* for the sixteen target temperatures listed above; each parallel simulation is called a "replica", eight CPUs per replica. Replica-exchange equilibration consists of two alternating phases: equilibration and exchange. In the equilibration phase, temperature is held constant for 5 ps (i.e., 2500 steps, 0.002 ps per step). In the exchange phase, the S6simulation is paused, and the conformation of a given replica is compared with that of its nearest neighbor. For example, the conformation of the 317.80 K replica would be compared on an alternating basis with that of the 310.00 K replica or of the 325.79 K replica. If the conformations of the replica and its nearest neighbor satisfy the Metropolis Monte Carlo criterion (i.e., are sufficiently similar), their coordinates are swapped; if the conformations are not sufficiently similar, no swap is made. Either way, the simulation resumes with a new equilibration phase. For example, after a swap, the coordinates from the final frame of the equilibration phase for the 310.00 K replica would be used as the initial coordinates for the new equilibration phase for the 317.80 K replica, and vice versa. Over the course of many such alternating cycles of equilibration/exchange, a single set of coordinates can traverse most if not all of the replica temperatures, and the conformational sampling at each temperature is thought to be more complete than would otherwise be possible without the exchanges between replicas.

At the conclusion of the simulations, trajectories for each of the 5-ps equilibration phases can be recombined into a full-length trajectory. Therefore, to obtain a full-length 2 μs trajectory, we needed to run 400000 cycles of equilibration/exchange (1 billion total steps). Doing so in a single run would have far exceeded the wall-time limits adopted by BYU's Office of Research Computing. Therefore, we broke the 400000 cycles of equilibration/exchange into forty serial runs of 10000 cycles each (dt = 0.002 ps; nstlim = 2500, numexchg = 10000; 25 million total steps or 50 ns per serial run; data were written to a trajectory file ever 1000 steps of 2 ps). Each serial run required between 1.5 and 3 days of wall time, for a total wall time of 60–120 days for a complete temperature replica exchange molecular dynamics simulation (T-REMD) on a given coiled-coil variant.

(return to Table of Contents)

8. Monitoring passage of coordinates through the sixteen temperature replicas: round trips

We used the program *cpptraj* to monitor the passage of each set of coordinates (crd1-crd16) through the sixteen temperature replicas during the 400000 equilibration/exchange cycles; to calculate the number of round trips made by each set of coordinates through the sixteen temperature replicas (e.g., from 310.00 K to 450.00 K and back again to 310.00 K); and to calculate the success rate of the exchange attempts between nearest neighbor temperature replicas during the T-REMD simulations of variants **s6e/1g'-x4x**, **s13e/8g'-x4x**, **s20e/15g'-x4x**, **s27e/22g'-x4x**, **s27e/22g'-z4x**, **s34e/29g'-x4x**, **s7f/10b'-x4x, s14f/17b'-x4x, s21f/24b'-x4x,** and **s28f/31b'-x4x**; for their non-stapled counterparts variants **6e/1g'-xx**, **13e/8g'-xx**, **20e/15g'-xx**, **27e/22g'-xx**, **27e/22g'-z4x**, **34e/29g'-xx**, **7f/10b'-xx**, **14f/17b'-xx**, **21f/24b'-xx**, and **28f/31b'-xx**. Figures S90–S109 and Tables S3–S42 show the results of this analysis.

Technically, convergence for a T-REMD simulations means that each of the 16 coordinates should sample the same conformational space over time, meaning that they would spend approximately the same amount of time at each of the 16 temperatures, and make approximately the same number of round trips. The data shown in Figures S90–S109 and Tables S3–S42 indicate that although every coordinate visits every temperature, not every coordinate experiences the same number of round trips. Though it would be reasonable to conclude that additional simulation time would bring these T-REMD simulations closer to convergence, we argue below that the cost of additional simulation time is not worth the payoff in improved convergence given that (1) the average measures of foldedness for each variant described in **Section 11** below are not changing substantially over the course of the 2-μs simulation; and (2) the strong agreement between experimental and simulated the ΔΔG values that reflect the impact of stapling on coiled-coil conformational stability described in **Section 15** below.

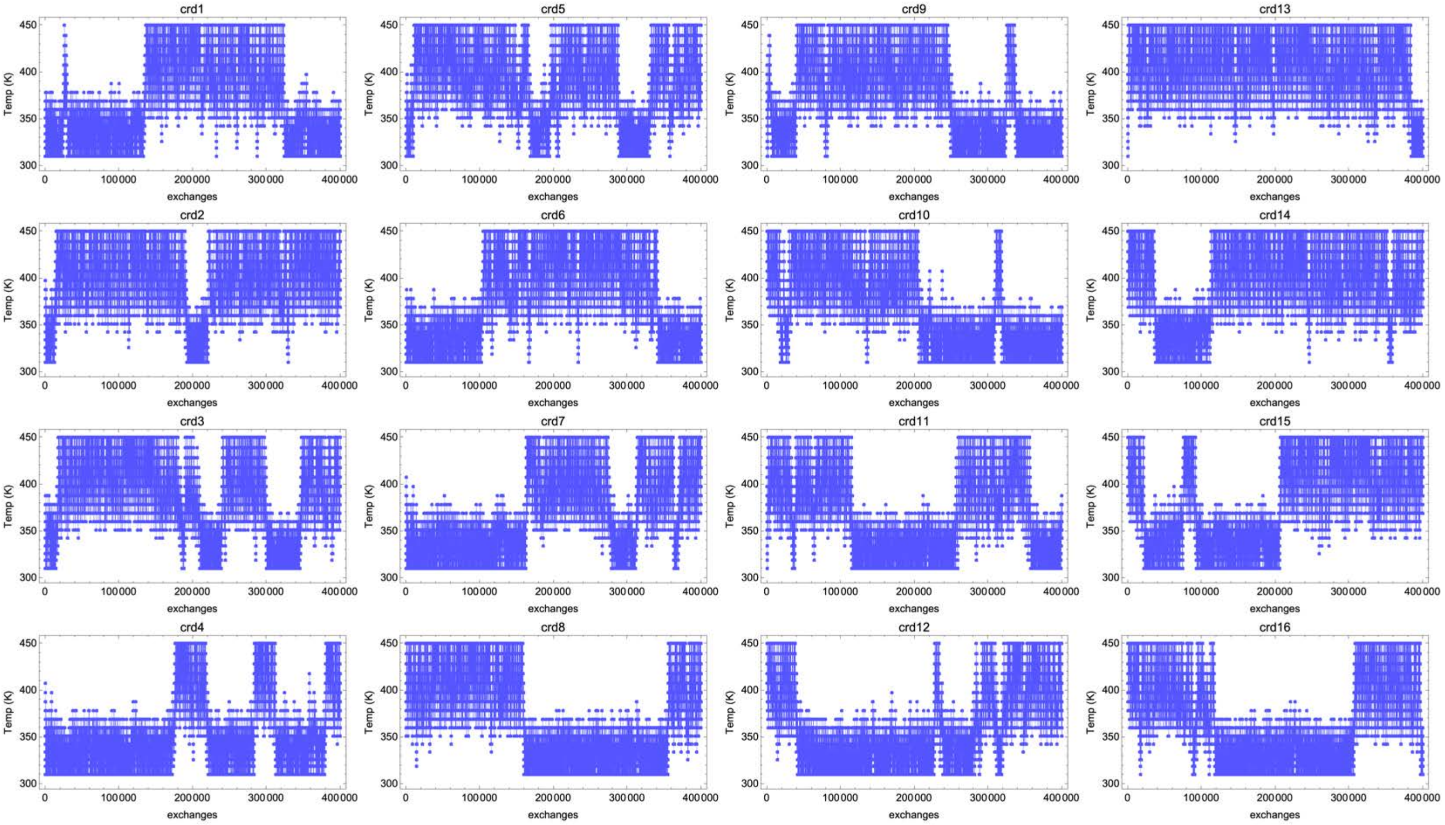

Figure S90. Passage of the 16 sets of coordinates (crd1–crd16) through the 16 available temperature replicas (310.00, 317.80, 325.79, 333.99, 342.39, 351.00, 359.83, 368.89, 378.17, 387.68, 397.43, 407.43, 417.68, 428.19, 438.96, or 450.00 K) during the T-REMD simulation of non-stapled variant **6e/1g'-xx**.

Table S3. Summary of the passage of the 16 sets of coordinates through the 16 available temperature replicas during the T-REMD simulation of non-stapled variant **6e/1g'-xx**.[a]

Coordinate	Round Trips	Exchanges per Round Trip		Minimum Exchanges Per Round Trip	Maximum Exchanges Per Round Trip
		Average	Standard Deviation		
1	4	81037.75	70600.6224	29793	202258
2	2	164679	27243	137436	191922
3	3	99952	66226.317	24030	185408
4	2	156258	64676	91582	220934
5	4	89424	48803.7124	36116	168434
6	3	113668.6667	40695.4295	67026	166188
7	2	181614	96722	84892	278336
8	0	0	0	0	0
9	3	112578	40688.1118	78958	169830
10	3	100082	20733.635	70896	117116
11	3	119073.3333	89276.6214	34306	242490
12	3	90036	76512.7259	23750	197246
13	1	384058	0	384058	384058
14	2	158081	50279	107802	208360
15	1	71036	0	71036	71036
16	4	94921	108741.8515	7668	278886

[a]A round trip constitutes passage of a single coordinate through all the available temperature replicas and back to the starting point (for example, from 310.00 K to 450.00 K and back again to 310.00 K).

Table S4. Replica-exchange success rates for the 16 temperature replicas during T-REMD simulation of non-stapled variant **6e/1g'-xx**.[a]

Replica Temperature (K)	% Success Up Exchange	Up Exchange
310	44.477	0
317.8	44.057	44.477
325.79	43.38	44.057
333.99	42.267	43.38
342.39	38.472	42.267
351	27.418	38.472
359.83	28.326	27.418
368.89	37.828	28.326
378.17	40.517	37.828
387.68	41.212	40.517
397.43	41.721	41.212
407.43	42.179	41.721
417.68	42.498	42.179
428.19	43.035	42.498
438.96	43.66	43.035
450	0	43.66

[a]The up exchange for a given replica is with the adjacent higher-temperature replica, except for the 450.00 K replica, which is with the 310.00 K replica. Similarly, the down exchange for a given replica is with the adjacent lower-temperature replica, except for the 310.00 K replica, which is with the 450.00 K replica.

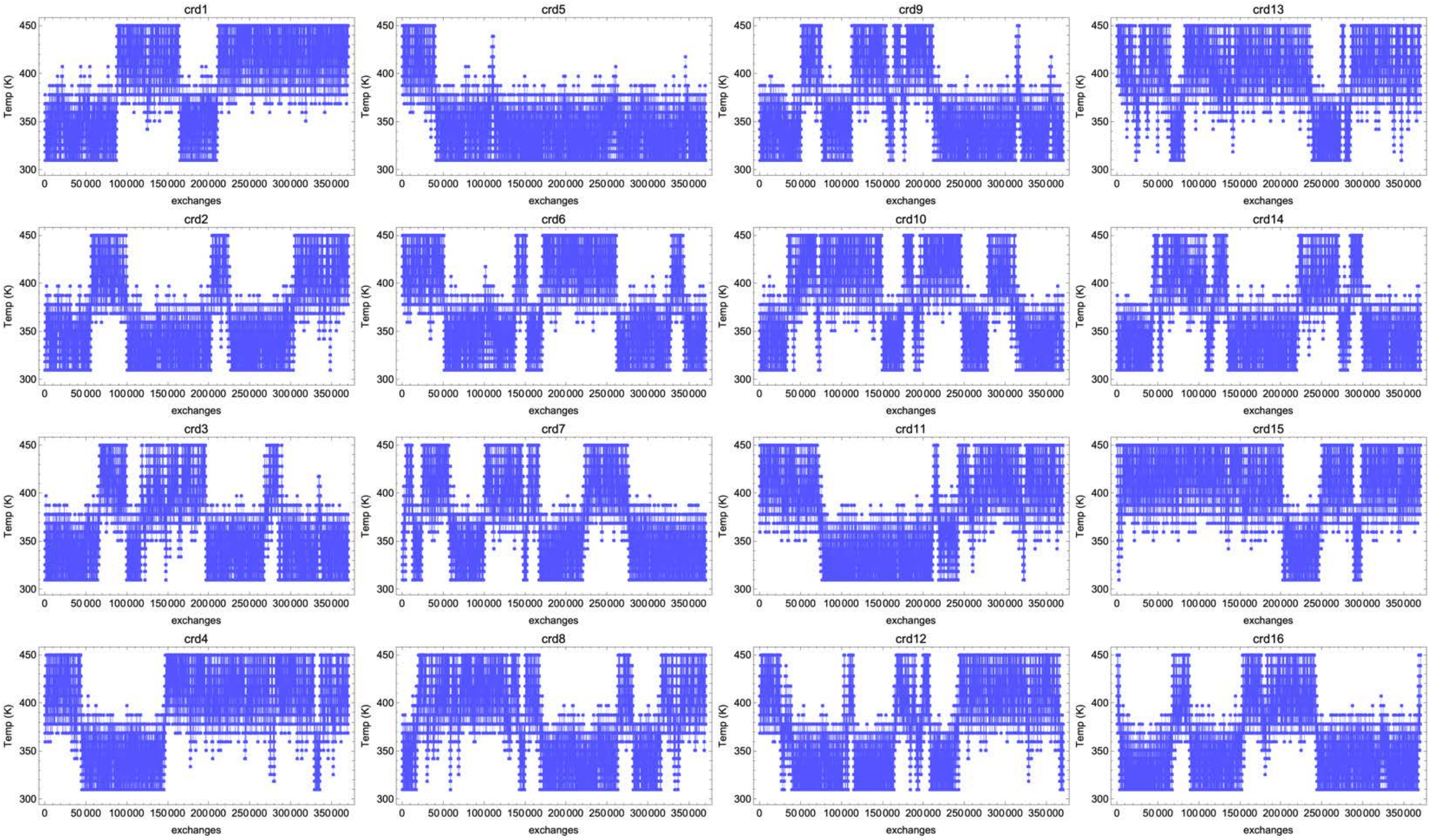

Figure S91. Passage of the 16 sets of coordinates (crd1–crd16) through the 16 available temperature replicas (310.00, 317.80, 325.79, 333.99, 342.39, 351.00, 359.83, 368.89, 378.17, 387.68, 397.43, 407.43, 417.68, 428.19, 438.96, or 450.00 K) during the T-REMD simulation of stapled variant **s6e/1g'-x4x**.

Table S5. Summary of the passage of the 16 sets of coordinates through the 16 available temperature replicas during the T-REMD simulation of stapled variant **s6e/1g'-x4x**.[a]

Coordinate	Round Trips	Exchanges per Round Trip		Minimum Exchanges Per Round Trip	Maximum Exchanges Per Round Trip
		Average	Standard Deviation		
1	1	164582	0	164582	164582
2	3	115957.3333	11335.1811	100192	126354
3	6	48267.3333	34934.4885	4778	99606
4	1	283864	0	283864	283864
5	0	0	0	0	0
6	3	97557.3333	11394.2393	82334	109744
7	5	55330.4	38364.8563	12484	109978
8	6	55992.3333	36241.2684	3468	113794
9	5	63874.8	32294.8377	18274	107452
10	6	52175.3333	16445.2337	30628	77822
11	2	122684	19582	103102	142266
12	6	56588.6667	53344.2014	8220	158906
13	4	80977	54351.2202	39720	172990
14	5	60099.4	40408.6559	26550	137126
15	2	143465	56651	86814	200116
16	2	120374	34190	86184	154564

[a]A round trip constitutes passage of a single coordinate through all the available temperature replicas and back to the starting point (for example, from 310.00 K to 450.00 K and back again to 310.00 K).

Table S6. Replica-exchange success rates for the 16 temperature replicas during T-REMD simulation of stapled variant **s6e/1g'-x4x**.[a]

Replica Temperature (K)	% Success	
	**Up Exchange**	**Up Exchange**
310	43.932	0
317.8	43.932	43.932
325.79	43.608	43.932
333.99	43.134	43.608
342.39	42.361	43.134
351	40.757	42.361
359.83	34.807	40.757
368.89	25.83	34.807
378.17	30.443	25.83
387.68	38.059	30.443
397.43	40.076	38.059
407.43	41.022	40.076
417.68	41.662	41.022
428.19	42.467	41.662
438.96	43.145	42.467
450	0	43.145

[a]The up exchange for a given replica is with the adjacent higher-temperature replica, except for the 450.00 K replica, which is with the 310.00 K replica. Similarly, the down exchange for a given replica is with the adjacent lower-temperature replica, except for the 310.00 K replica, which is with the 450.00 K replica.

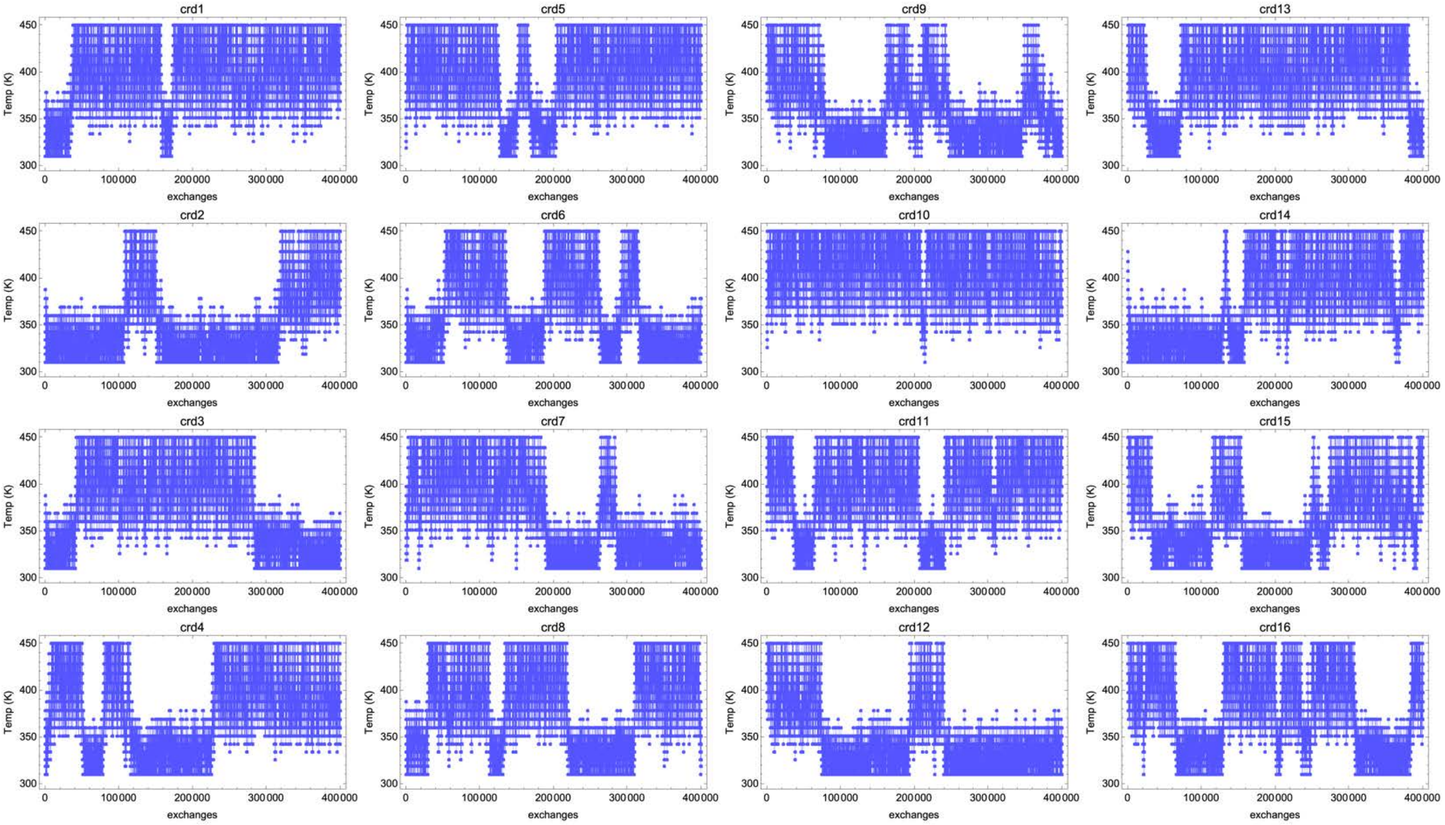

Figure S92. Passage of the 16 sets of coordinates (crd1–crd16) through the 16 available temperature replicas (310.00, 317.80, 325.79, 333.99, 342.39, 351.00, 359.83, 368.89, 378.17, 387.68, 397.43, 407.43, 417.68, 428.19, 438.96, or 450.00 K) during the T-REMD simulation of non-stapled variant **13e/8g'-xx**.

Table S7. Summary of the passage of the 16 sets of coordinates through the 16 available temperature replicas during the T-REMD simulation of non-stapled variant **13e/8g'-xx**.[a]

**Coordinate**	**Round Trips**	**Exchanges per Round Trip**		**Mininum Exchanges Per Round Trip**	**Maximum Exchanges Per Round Trip**
		**Average**	**Standard Deviation**		
1	1	157753	0	157753	157753
2	1	151220	0	151220	151220
3	1	283848	0	283848	283848
4	2	57862	6864	50998	64726
5	1	43544	0	43544	43544
6	3	105338.6667	37641.3886	52454	137046
7	3	89250.6667	37698.2185	40490	132300
8	3	133090	33157.6675	106412	179826
9	4	77929.5	49359.0763	13432	129510
10	0	0	0	0	0
11	2	84907	9633	75274	94540
12	2	82704	70424	12280	153128
13	1	355076	0	355076	355076
14	3	121208.6667	31362.6983	77350	148860
15	4	88762.5	36975.5906	26154	122078
16	4	71816	39336.2157	34412	135450

[a]A round trip constitutes passage of a single coordinate through all the available temperature replicas and back to the starting point (for example, from 310.00 K to 450.00 K and back again to 310.00 K).

Table S8. Replica-exchange success rates for the 16 temperature replicas during T-REMD simulation of non-stapled variant **13e/8g'-xx**.[a]

	% Success	
**Replica Temperature (K)**	**Up Exchange**	**Up Exchange**
310	43.833	0.001
317.8	43.3	43.833
325.79	42.81	43.3
333.99	41.991	42.81
342.39	40.375	41.991
351	38.197	40.375
359.83	32.676	38.197
368.89	29.251	32.676
378.17	33.076	29.251
387.68	37.295	33.076
397.43	39.502	37.295
407.43	40.867	39.502
417.68	41.598	40.867
428.19	42.3	41.598
438.96	43.106	42.3
450	0.001	43.106

[a]The up exchange for a given replica is with the adjacent higher-temperature replica, except for the 450.00 K replica, which is with the 310.00 K replica. Similarly, the down exchange for a given replica is with the adjacent lower-temperature replica, except for the 310.00 K replica, which is with the 450.00 K replica.

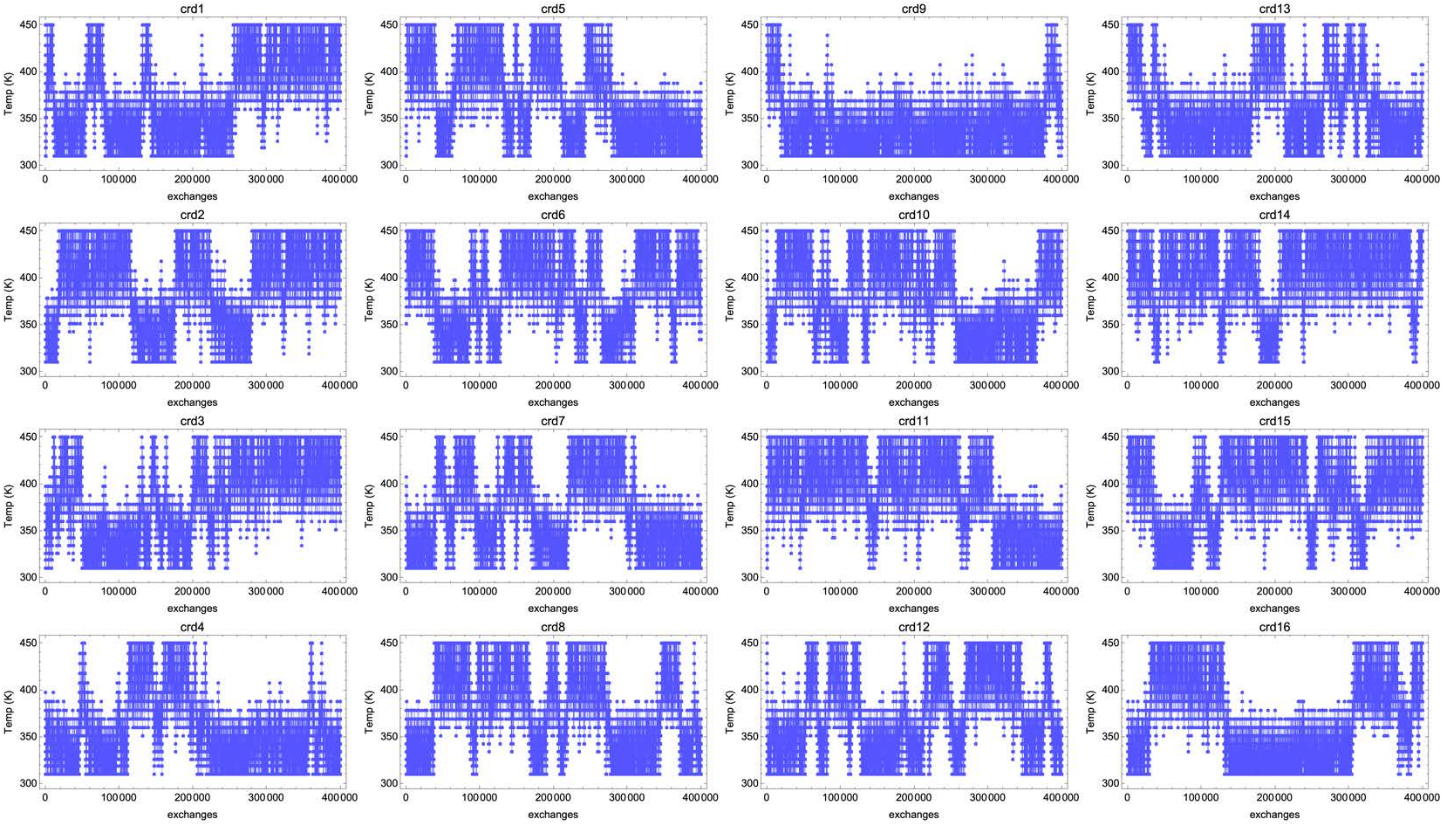

Figure S93. Passage of the 16 sets of coordinates (crd1–crd16) through the 16 available temperature replicas (310.00, 317.80, 325.79, 333.99, 342.39, 351.00, 359.83, 368.89, 378.17, 387.68, 397.43, 407.43, 417.68, 428.19, 438.96, or 450.00 K) during the T-REMD simulation of stapled variant **s13e/8g’-x4x**.

Table S9. Summary of the passage of the 16 sets of coordinates through the 16 available temperature replicas during the T-REMD simulation of stapled variant **s13e/8g’-x4x**.[a]

Coordinate	Round Trips	Exchanges per Round Trip		Mininum Exchanges Per Round Trip	Maximum Exchanges Per Round Trip
		Average	Standard Deviation		
1	3	47842	25592.357	11818	68880
2	4	56436.5	27343.1321	15214	92134
3	6	40698.3333	24859.8335	12688	83730
4	6	62722	45910.6547	12204	144620
5	5	55798	24191.1535	19876	91480
6	6	53666	31241.4161	15122	95102
7	7	44462.2857	37688.0742	9204	130642
8	6	65458	27623.2314	21412	100416
9	1	376586	0	376586	376586
10	7	36402.5714	22142.819	6654	69298
11	5	61207.6	18545.6165	42374	93784
12	8	48435.5	23777.4511	16666	95194
13	6	50175.3333	53858.2972	16750	168956
14	4	87783.5	37535.4683	55080	149020
15	5	54922	25127.4419	6666	76626
16	2	183718.5	52575.5	131143	236294

[a]A round trip constitutes passage of a single coordinate through all the available temperature replicas and back to the starting point (for example, from 310.00 K to 450.00 K and back again to 310.00 K).

Table S10. Replica-exchange success rates for the 16 temperature replicas during T-REMD simulation of stapled variant **s13e/8g'-x4x**.[a]

**Replica Temperature (K)**	**% Success**	
	**Up Exchange**	**Up Exchange**
310	43.833	0.001
317.8	43.3	43.833
325.79	42.81	43.3
333.99	41.991	42.81
342.39	40.375	41.991
351	38.197	40.375
359.83	32.676	38.197
368.89	29.251	32.676
378.17	33.076	29.251
387.68	37.295	33.076
397.43	39.502	37.295
407.43	40.867	39.502
417.68	41.598	40.867
428.19	42.3	41.598
438.96	43.106	42.3
450	0.001	43.106

[a]The up exchange for a given replica is with the adjacent higher-temperature replica, except for the 450.00 K replica, which is with the 310.00 K replica. Similarly, the down exchange for a given replica is with the adjacent lower-temperature replica, except for the 310.00 K replica, which is with the 450.00 K replica.

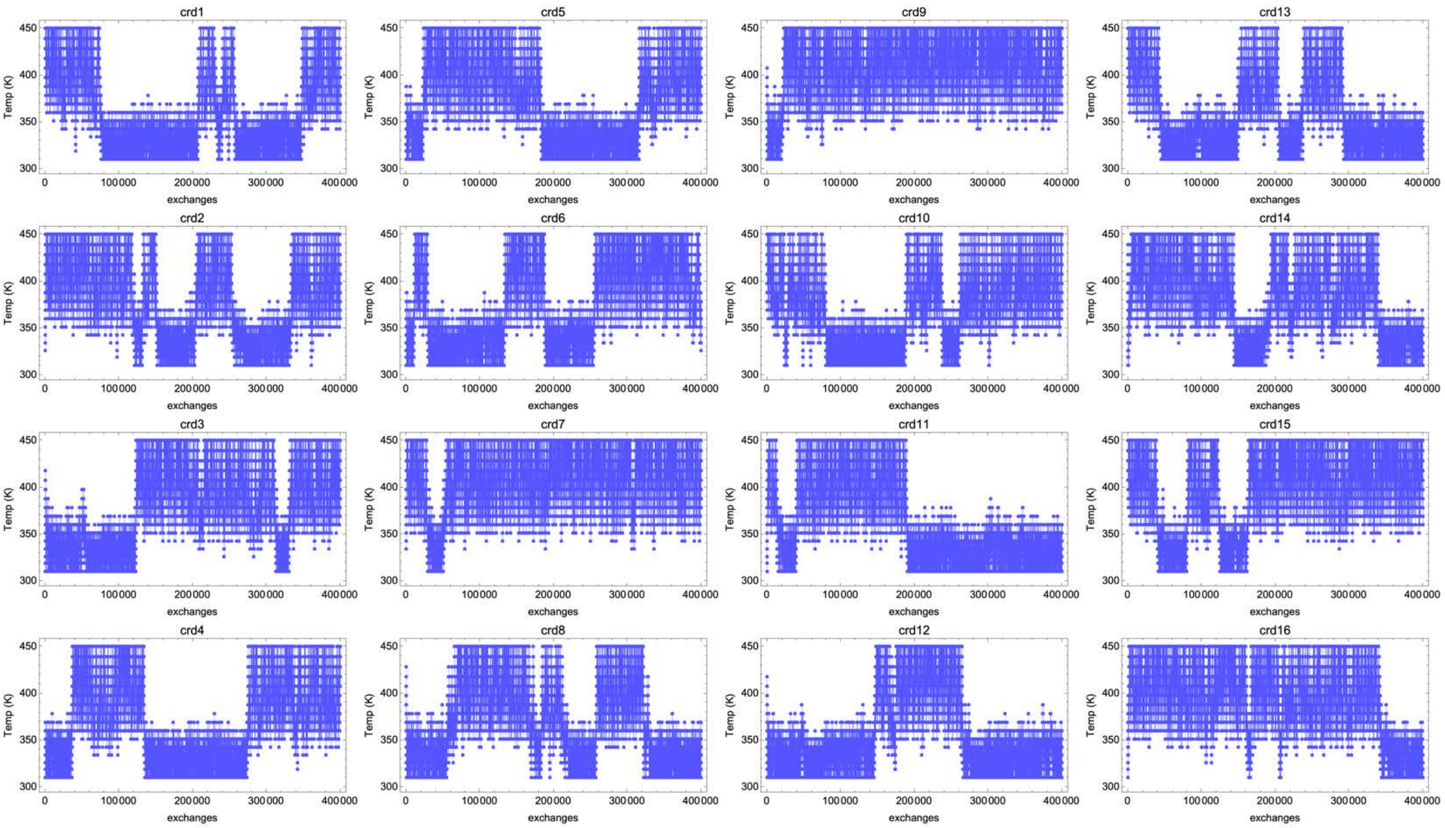

Figure S94. Passage of the 16 sets of coordinates (crd1–crd16) through the 16 available temperature replicas (310.00, 317.80, 325.79, 333.99, 342.39, 351.00, 359.83, 368.89, 378.17, 387.68, 397.43, 407.43, 417.68, 428.19, 438.96, or 450.00 K) during the T-REMD simulation of non-stapled variant **20e/15g'-xx**.

Table S11. Summary of the passage of the 16 sets of coordinates through the 16 available temperature replicas during the T-REMD simulation of non-stapled variant **20e/15g'-xx**.[a]

Coordinate	Round Trips	Exchanges per Round Trip		Mininum Exchanges Per Round Trip	Maximum Exchanges Per Round Trip
		Average	Standard Deviation		
1	3	60474	69142.691	8586	158194
2	3	79847.3333	34910.6482	30480	105060
3	1	312780	0	312780	312780
4	1	135080	0	135080	135080
5	1	183342	0	183342	183342
6	2	94449	64699	29750	159148
7	0	0	0	0	0
8	5	64338	63469.2943	4470	169144
9	0	0	0	0	0
10	5	55390	54300.3295	10412	157988
11	2	95149	79865	15284	175014
12	1	265970	0	265970	265970
13	2	123646	35874	87772	159520
14	2	169916	25912	144004	195828
15	1	83614	0	83614	83614
16	3	114623.6667	52615.8602	41710	163939

[a]A round trip constitutes passage of a single coordinate through all the available temperature replicas and back to the starting point (for example, from 310.00 K to 450.00 K and back again to 310.00 K).

Table S12. Replica-exchange success rates for the 16 temperature replicas during T-REMD simulation of non-stapled variant **20e/15g'-xx**.[a]

Replica Temperature (K)	% Success Up Exchange	Up Exchange
310	44.413	0.001
317.8	44.036	44.413
325.79	43.294	44.036
333.99	41.617	43.294
342.39	34.133	41.617
351	22.834	34.133
359.83	32.592	22.834
368.89	39.215	32.592
378.17	41.103	39.215
387.68	41.558	41.103
397.43	42.026	41.558
407.43	42.451	42.026
417.68	42.809	42.451
428.19	43.572	42.809
438.96	43.944	43.572
450	0.001	43.944

[a]The up exchange for a given replica is with the adjacent higher-temperature replica, except for the 450.00 K replica, which is with the 310.00 K replica. Similarly, the down exchange for a given replica is with the adjacent lower-temperature replica, except for the 310.00 K replica, which is with the 450.00 K replica.

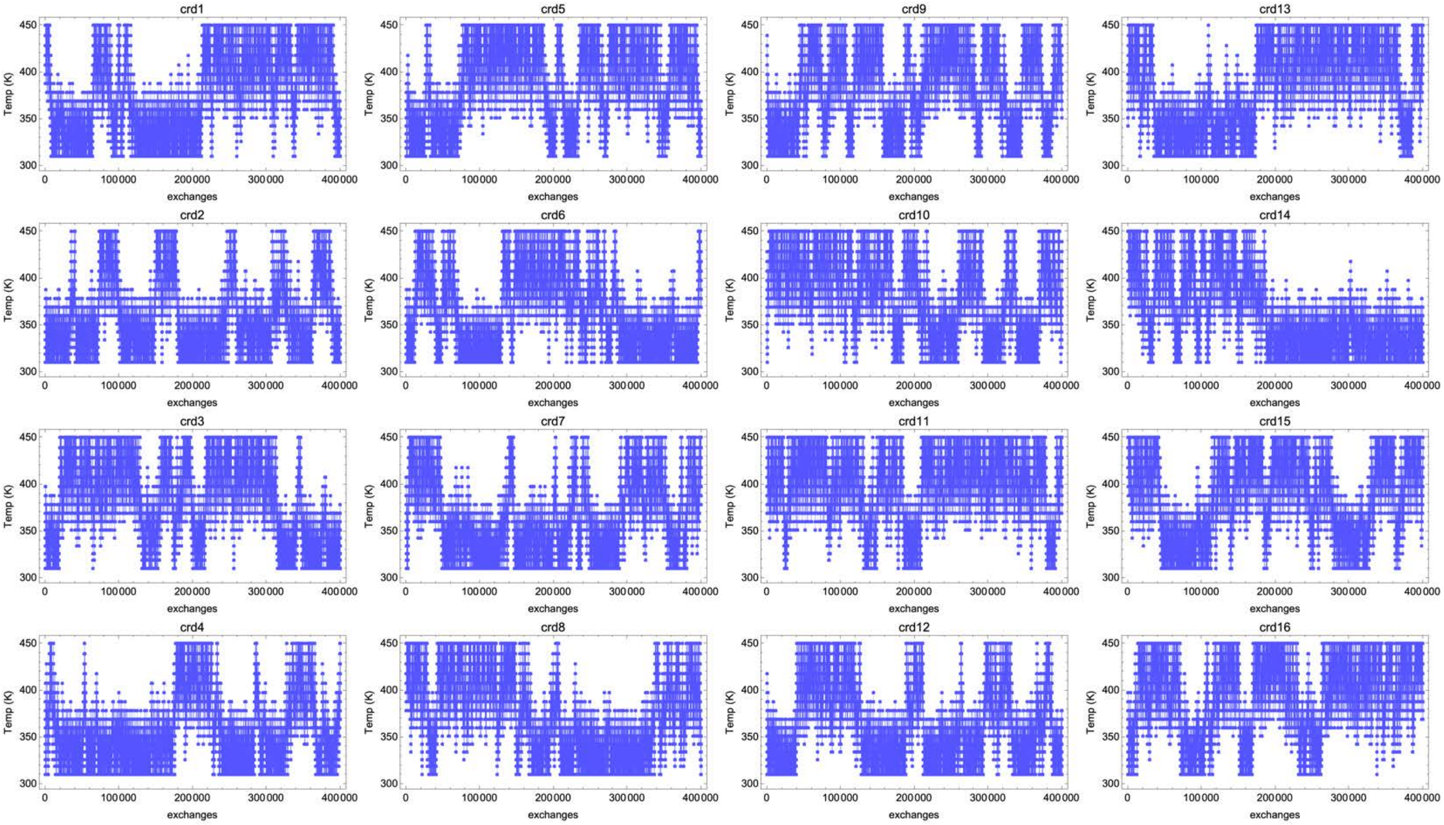

Figure S95. Passage of the 16 sets of coordinates (crd1–crd16) through the 16 available temperature replicas (310.00, 317.80, 325.79, 333.99, 342.39, 351.00, 359.83, 368.89, 378.17, 387.68, 397.43, 407.43, 417.68, 428.19, 438.96, or 450.00 K) during the T-REMD simulation of stapled variant **s20e/15g'-x4x**.

Table S13. Summary of the passage of the 16 sets of coordinates through the 16 available temperature replicas during the T-REMD simulation of stapled variant **s20e/15g'-x4x**.[a]

Coordinate	Round Trips	Exchanges per Round Trip		Mininum Exchanges Per Round Trip	Maximum Exchanges Per Round Trip
		Average	Standard Deviation		
1	8	48122.25	31824.1638	10882	108928
2	8	48814.25	20448.859	11554	70090
3	7	49684.2857	14635.0505	26464	66628
4	7	52374.8571	51320.4849	7826	172458
5	7	56527.7143	47213.5654	3562	159800
6	12	23984.1667	20683.1562	3350	75090
7	7	53529.1429	33641.4062	16068	105860
8	7	54007.4286	41855.4908	9152	134566
9	9	41486.8889	22095.4345	3126	87948
10	8	49774	29131.2062	9858	104588
11	6	59224.6667	60871.7464	7292	173024
12	7	55296.8571	36036.3925	18112	127786
13	3	117789.3333	101125.4577	19250	256820
14	6	26457.3333	10927.2638	13114	43388
15	7	45363.1429	27040.7128	10772	83994
16	5	67546.2	25290.9377	38608	106258

[a]A round trip constitutes passage of a single coordinate through all the available temperature replicas and back to the starting point (for example, from 310.00 K to 450.00 K and back again to 310.00 K).

Table S14. Replica-exchange success rates for the 16 temperature replicas during T-REMD simulation of stapled variant **s20e/15g'-x4x**.[a]

Replica Temperature (K)	% Success Up Exchange	Up Exchange
310	43.516	0.001
317.8	43.382	43.516
325.79	42.444	43.382
333.99	41.367	42.444
342.39	39.49	41.367
351	35.279	39.49
359.83	31.317	35.279
368.89	33.428	31.317
378.17	36.312	33.428
387.68	38.236	36.312
397.43	39.306	38.236
407.43	40.127	39.306
417.68	41.257	40.127
428.19	42.085	41.257
438.96	42.723	42.085
450	0.001	42.723

[a]The up exchange for a given replica is with the adjacent higher-temperature replica, except for the 450.00 K replica, which is with the 310.00 K replica. Similarly, the down exchange for a given replica is with the adjacent lower-temperature replica, except for the 310.00 K replica, which is with the 450.00 K replica.

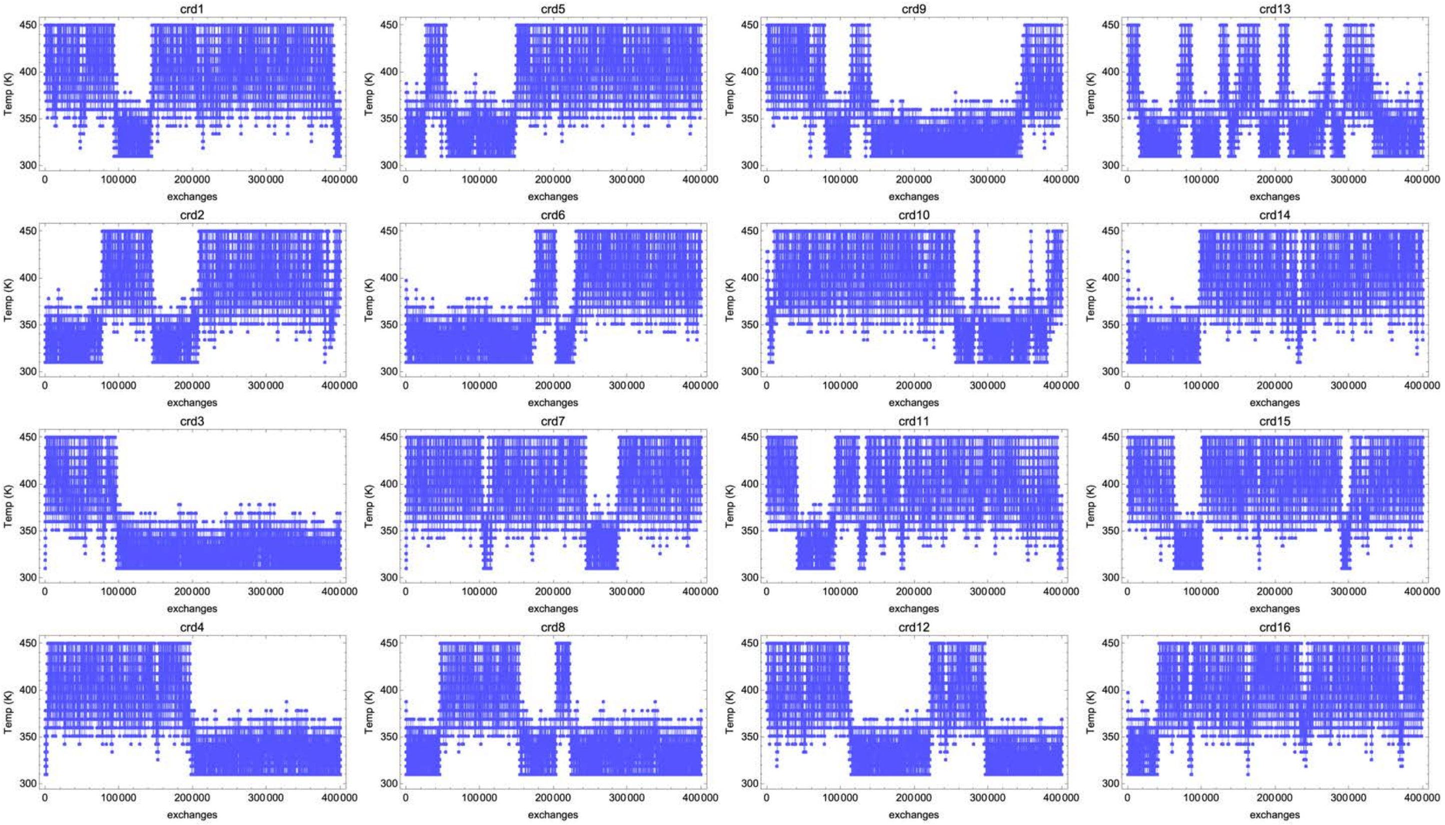

Figure S96. Passage of the 16 sets of coordinates (crd1–crd16) through the 16 available temperature replicas (310.00, 317.80, 325.79, 333.99, 342.39, 351.00, 359.83, 368.89, 378.17, 387.68, 397.43, 407.43, 417.68, 428.19, 438.96, or 450.00 K) during the T-REMD simulation of non-stapled variant **27e/22g'-xx**.

Table S15. Summary of the passage of the 16 sets of coordinates through the 16 available temperature replicas during the T-REMD simulation of non-stapled variant **27e/22g'-xx**.[a]

Coordinate	Round Trips	Exchanges per Round Trip		Mininum Exchanges Per Round Trip	Maximum Exchanges Per Round Trip
		Average	Standard Deviation		
1	1	299124	0	299124	299124
2	2	189498	43644	145854	233142
3	1	97056	0	97056	97056
4	1	197808	0	197808	197808
5	2	27914	19722	8192	47636
6	1	203324	0	203324	203324
7	3	81416.6667	52877.7089	8166	131074
8	2	111896	42152	69744	154048
9	3	27415.3333	19873.8602	7340	54488
10	3	120078	95975.8931	30816	253260
11	3	118008	68461.4824	56730	213564
12	1	181952	0	181952	181952
13	6	52775	10776.1782	39696	71596
14	1	229964	0	229964	229964
15	2	113681	1611	112070	115292
16	2	81414.5	4402.5	77012	85817

[a]A round trip constitutes passage of a single coordinate through all the available temperature replicas and back to the starting point (for example, from 310.00 K to 450.00 K and back again to 310.00 K).

Table S16. Replica-exchange success rates for the 16 temperature replicas during T-REMD simulation of non-stapled variant **27e/22g’-xx**.[a]

Replica Temperature (K)	% Success Up Exchange	Up Exchange
310	43.883	0.001
317.8	43.387	43.883
325.79	42.783	43.387
333.99	41.247	42.783
342.39	35.093	41.247
351	25.561	35.093
359.83	30.005	25.561
368.89	39.316	30.005
378.17	41.17	39.316
387.68	41.796	41.17
397.43	42.221	41.796
407.43	42.756	42.221
417.68	43.163	42.756
428.19	43.575	43.163
438.96	43.924	43.575
450	0.001	43.924

[a]The up exchange for a given replica is with the adjacent higher-temperature replica, except for the 450.00 K replica, which is with the 310.00 K replica. Similarly, the down exchange for a given replica is with the adjacent lower-temperature replica, except for the 310.00 K replica, which is with the 450.00 K replica.

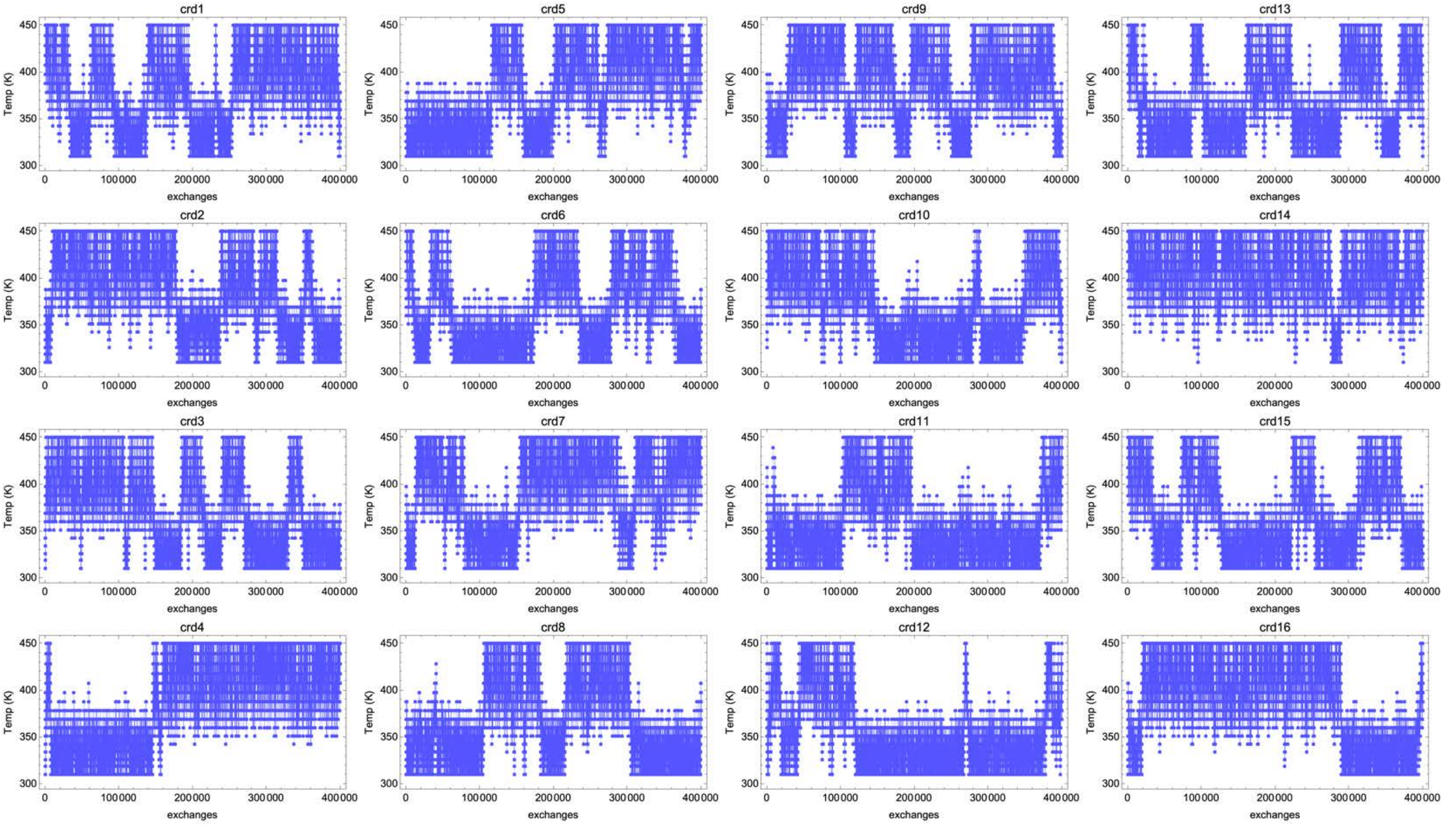

Figure S97. Passage of the 16 sets of coordinates (crd1–crd16) through the 16 available temperature replicas (310.00, 317.80, 325.79, 333.99, 342.39, 351.00, 359.83, 368.89, 378.17, 387.68, 397.43, 407.43, 417.68, 428.19, 438.96, or 450.00 K) during the T-REMD simulation of stapled variant **s27e/22g'-x4x**.

Table S17. Summary of the passage of the 16 sets of coordinates through the 16 available temperature replicas during the T-REMD simulation of stapled variant **s27e/22g'-x4x**.[a]

Coordinate	Round Trips	Exchanges per Round Trip		Mininum Exchanges Per Round Trip	Maximum Exchanges Per Round Trip
		Average	Standard Deviation		
1	4	90983.5	47801.1256	38500	163718
2	4	91025.5	57901.6095	30772	179236
3	8	43688.5	20449.917	12682	79484
4	2	77841	70423	7418	148264
5	3	125826	24463.1547	101870	159420
6	4	88245.5	52276.4479	37882	171948
7	4	84405.5	73392.1517	26224	210338
8	4	76204.5	59105.1862	13336	146690
9	4	97282.5	27909.4712	68228	138966
10	4	81208.5	47642.7072	24468	143650
11	2	98064	90490	7574	188554
12	5	79454.8	51662.9427	18828	153544
13	6	64227.6667	34731.1104	11284	120984
14	3	92823.3333	33871.7749	49248	131840
15	6	56369	24867.4296	23874	102990
16	1	289349	0	289349	289349

[a]A round trip constitutes passage of a single coordinate through all the available temperature replicas and back to the starting point (for example, from 310.00 K to 450.00 K and back again to 310.00 K).

Table S18. Replica-exchange success rates for the 16 temperature replicas during T-REMD simulation of stapled variant **s27e/22g’-x4x**.[a]

Replica Temperature (K)	% Success	
	**Up Exchange**	**Up Exchange**
310	43.592	0.001
317.8	43.522	43.592
325.79	42.728	43.522
333.99	41.88	42.728
342.39	39.642	41.88
351	32.547	39.642
359.83	28.548	32.547
368.89	32.903	28.548
378.17	38.011	32.903
387.68	39.681	38.011
397.43	40.477	39.681
407.43	41.094	40.477
417.68	41.581	41.094
428.19	42.163	41.581
438.96	42.748	42.163
450	0.001	42.748

[a]The up exchange for a given replica is with the adjacent higher-temperature replica, except for the 450.00 K replica, which is with the 310.00 K replica. Similarly, the down exchange for a given replica is with the adjacent lower-temperature replica, except for the 310.00 K replica, which is with the 450.00 K replica.

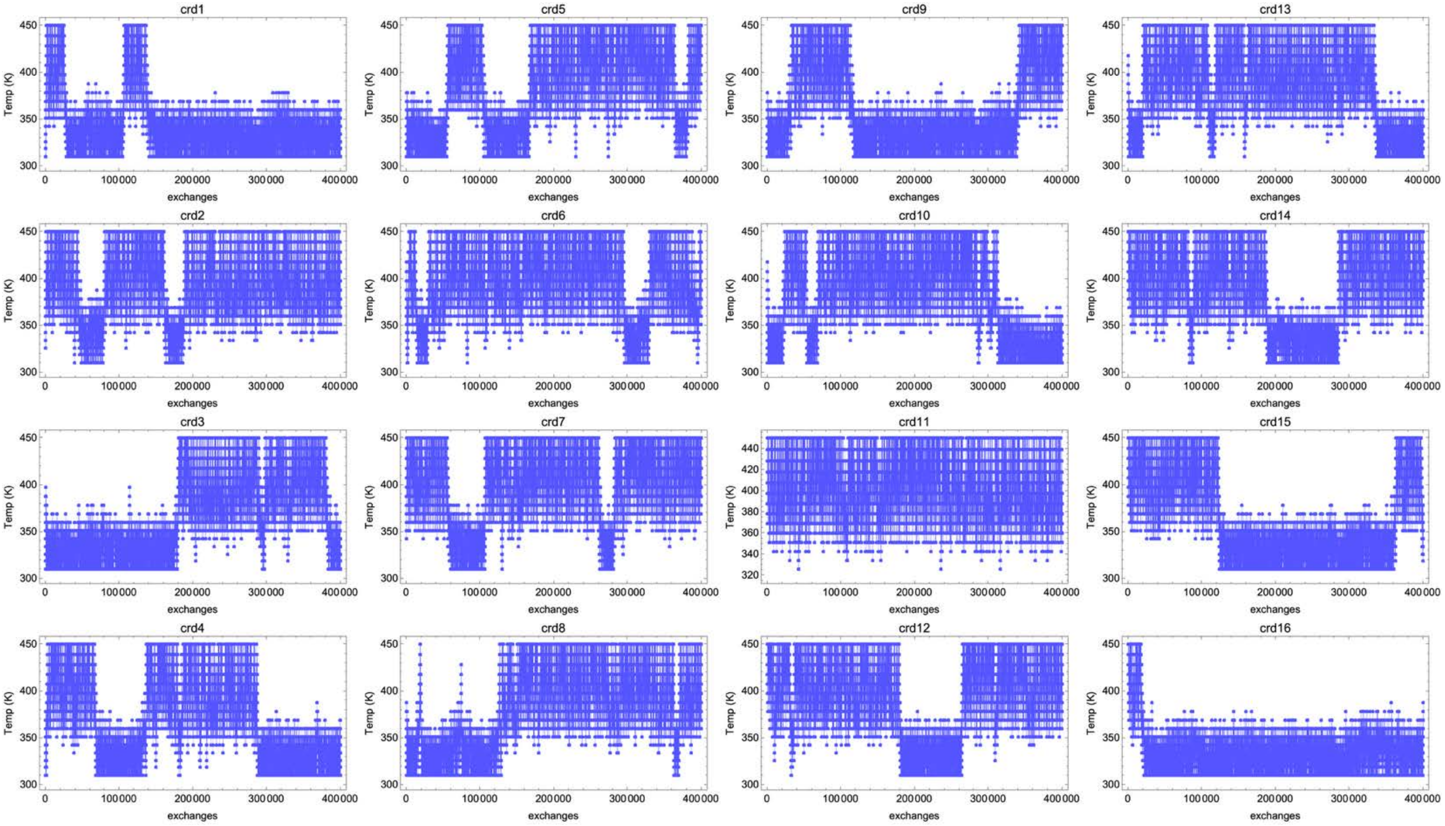

Figure S98. Passage of the 16 sets of coordinates (crd1–crd16) through the 16 available temperature replicas (310.00, 317.80, 325.79, 333.99, 342.39, 351.00, 359.83, 368.89, 378.17, 387.68, 397.43, 407.43, 417.68, 428.19, 438.96, or 450.00 K) during the T-REMD simulation of non-stapled variant **27e/22g’-z4x**.

Table S19. Summary of the passage of the 16 sets of coordinates through the 16 available temperature replicas during the T-REMD simulation of non-stapled variant **27e/22g’-z4x**.[a]

Coordinate	Round Trips	Exchanges per Round Trip		Mininum Exchanges Per Round Trip	Maximum Exchanges Per Round Trip
		Average	Standard Deviation		
1	2	70242.5	42019.5	28223	112262
2	2	61850	52620	9230	114470
3	2	191607	102517	89090	294124
4	3	95769.3333	19712.1804	68092	112494
5	4	91293	29741.5658	44056	124770
6	4	98463	72883.4652	13742	213080
7	2	101086	31324	69762	132410
8	3	120913.3333	88473.284	19892	235356
9	1	117004	0	117004	117004
10	3	104228.6667	91328.1172	26702	232454
11	0	0	0	0	0
12	1	148228	0	148228	148228
13	3	112084.6667	54053.5356	46616	178996
14	1	105462	0	105462	105462
15	0	0	0	0	0
16	0	0	0	0	0

[a]A round trip constitutes passage of a single coordinate through all the available temperature replicas and back to the starting point (for example, from 310.00 K to 450.00 K and back again to 310.00 K).

Table S20. Replica-exchange success rates for the 16 temperature replicas during T-REMD simulation of non-stapled variant **27e/22g'-z4x**.[a]

Replica Temperature (K)	% Success Up Exchange	Up Exchange
310	43.574	0
317.8	43.404	43.574
325.79	42.475	43.404
333.99	41.289	42.475
342.39	36.45	41.289
351	21.052	36.45
359.83	31.802	21.052
368.89	38.983	31.802
378.17	40.947	38.983
387.68	41.492	40.947
397.43	41.813	41.492
407.43	42.085	41.813
417.68	42.709	42.085
428.19	43.256	42.709
438.96	44.008	43.256
450	0	44.008

[a]The up exchange for a given replica is with the adjacent higher-temperature replica, except for the 450.00 K replica, which is with the 310.00 K replica. Similarly, the down exchange for a given replica is with the adjacent lower-temperature replica, except for the 310.00 K replica, which is with the 450.00 K replica.

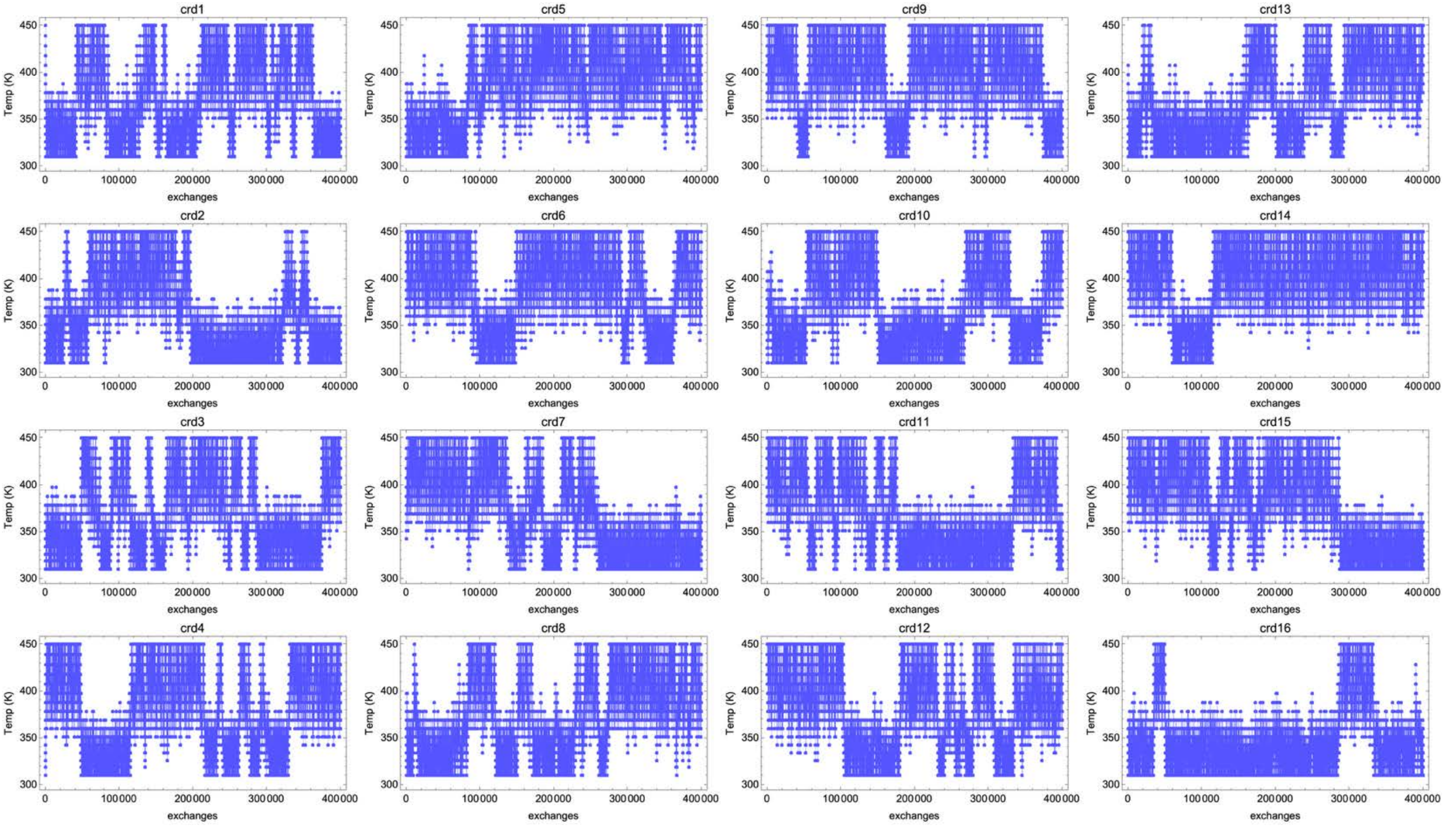

Figure S99. Passage of the 16 sets of coordinates (crd1–crd16) through the 16 available temperature replicas (310.00, 317.80, 325.79, 333.99, 342.39, 351.00, 359.83, 368.89, 378.17, 387.68, 397.43, 407.43, 417.68, 428.19, 438.96, or 450.00 K) during the T-REMD simulation of stapled variant **s27e/22g’-z4x**.

Table S21. Summary of the passage of the 16 sets of coordinates through the 16 available temperature replicas during the T-REMD simulation of stapled variant **s27e/22g’-z4x**.[a]

Coordinate	Round Trips	Exchanges per Round Trip		Mininum Exchanges Per Round Trip	Maximum Exchanges Per Round Trip
		Average	Standard Deviation		
1	7	51854.8571	25799.0684	12710	85006
2	5	71461.2	49044.9056	18070	142486
3	8	35834.25	19481.4396	15952	74240
4	5	59828	54808.7745	21868	167826
5	2	66243	31025	35218	97268
6	2	114804	82584	32220	197388
7	4	43834.5	8490.6858	31556	55318
8	6	61054.6667	35849.7023	14644	106156
9	4	82987	42725.8633	14616	119574
10	4	82328	62720.2958	6632	178742
11	6	56360.6667	71854.6039	15754	216416
12	5	48156	40536.2941	13376	126934
13	5	55131.6	43588.4591	7204	130130
14	0	0	0	0	0
15	3	58880.6667	41655.993	27024	117724
16	2	166564.5	115819.5	50745	282384

[a]A round trip constitutes passage of a single coordinate through all the available temperature replicas and back to the starting point (for example, from 310.00 K to 450.00 K and back again to 310.00 K).

Table S22. Replica-exchange success rates for the 16 temperature replicas during T-REMD simulation of stapled variant **s27e/22g'-z4x**.[a]

Replica Temperature (K)	% Success	
	Up Exchange	Up Exchange
310	43.261	0.001
317.8	42.977	43.261
325.79	42.412	42.977
333.99	41.056	42.412
342.39	38.682	41.056
351	32.779	38.682
359.83	27.847	32.779
368.89	34.383	27.847
378.17	38.435	34.383
387.68	40.085	38.435
397.43	40.803	40.085
407.43	41.441	40.803
417.68	41.89	41.441
428.19	42.428	41.89
438.96	42.974	42.428
450	0.001	42.974

[a]The up exchange for a given replica is with the adjacent higher-temperature replica, except for the 450.00 K replica, which is with the 310.00 K replica. Similarly, the down exchange for a given replica is with the adjacent lower-temperature replica, except for the 310.00 K replica, which is with the 450.00 K replica.

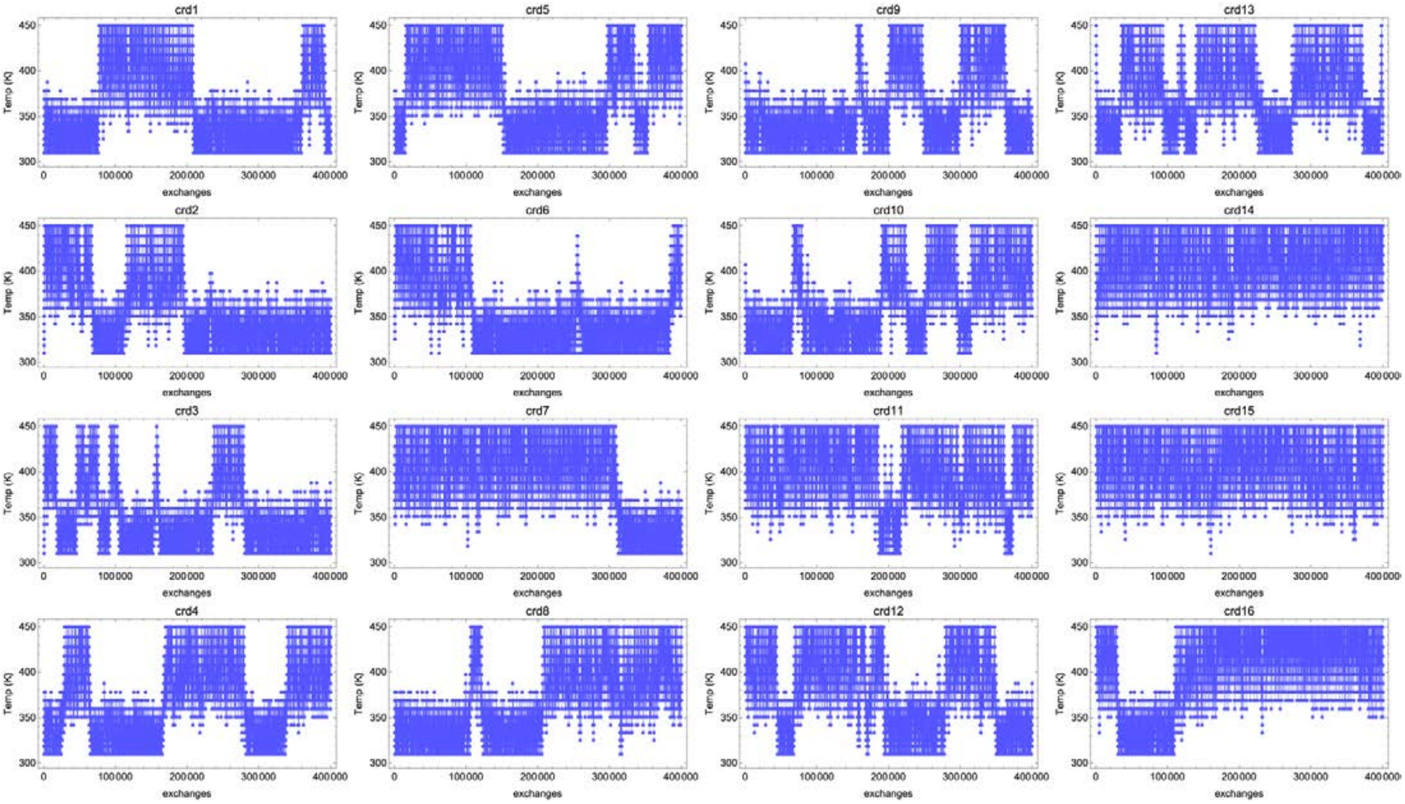

Figure S100. Passage of the 16 sets of coordinates (crd1–crd16) through the 16 available temperature replicas (310.00, 317.80, 325.79, 333.99, 342.39, 351.00, 359.83, 368.89, 378.17, 387.68, 397.43, 407.43, 417.68, 428.19, 438.96, or 450.00 K) during the T-REMD simulation of non-stapled variant **34e/29g'-xx**.

Table S23. Summary of the passage of the 16 sets of coordinates through the 16 available temperature replicas during the T-REMD simulation of non-stapled variant **34e/29g'-xx**.[a]

Coordinate	Round Trips	Exchanges per Round Trip		Mininum Exchanges Per Round Trip	Maximum Exchanges Per Round Trip
		Average	Standard Deviation		
1	2	196199.5	12113.5	184086	208313
2	2	97951	29753	68198	127704
3	5	56017.6	35084.2232	18222	118982
4	2	140853	76429	64424	217282
5	2	167573	16191	151382	183764
6	2	28704	17750	10954	46454
7	0	0	0	0	0
8	2	157099	34155	122944	191254
9	3	121210	33583.5781	83462	165046
10	4	74244.5	35838.4943	22468	123412
11	1	175954	0	175954	175954
12	3	101792	56331.3393	24236	156338
13	5	79754	45505.1409	26908	146086
14	0	0	0	0	0
15	0	0	0	0	0
16	0	0	0	0	0

[a]A round trip constitutes passage of a single coordinate through all the available temperature replicas and back to the starting point (for example, from 310.00 K to 450.00 K and back again to 310.00 K).

Table S24. Replica-exchange success rates for the 16 temperature replicas during T-REMD simulation of non-stapled variant **34e/29g'-xx**.[a]

Replica Temperature (K)	% Success	
	**Up Exchange**	**Up Exchange**
310	44.393	0
317.8	43.728	44.393
325.79	43.284	43.728
333.99	42.32	43.284
342.39	38.665	42.32
351	29.165	38.665
359.83	27.079	29.165
368.89	36.484	27.079
378.17	40.231	36.484
387.68	41.072	40.231
397.43	41.684	41.072
407.43	42.151	41.684
417.68	42.505	42.151
428.19	43.112	42.505
438.96	43.902	43.112
450	0	43.902

[a]The up exchange for a given replica is with the adjacent higher-temperature replica, except for the 450.00 K replica, which is with the 310.00 K replica. Similarly, the down exchange for a given replica is with the adjacent lower-temperature replica, except for the 310.00 K replica, which is with the 450.00 K replica.

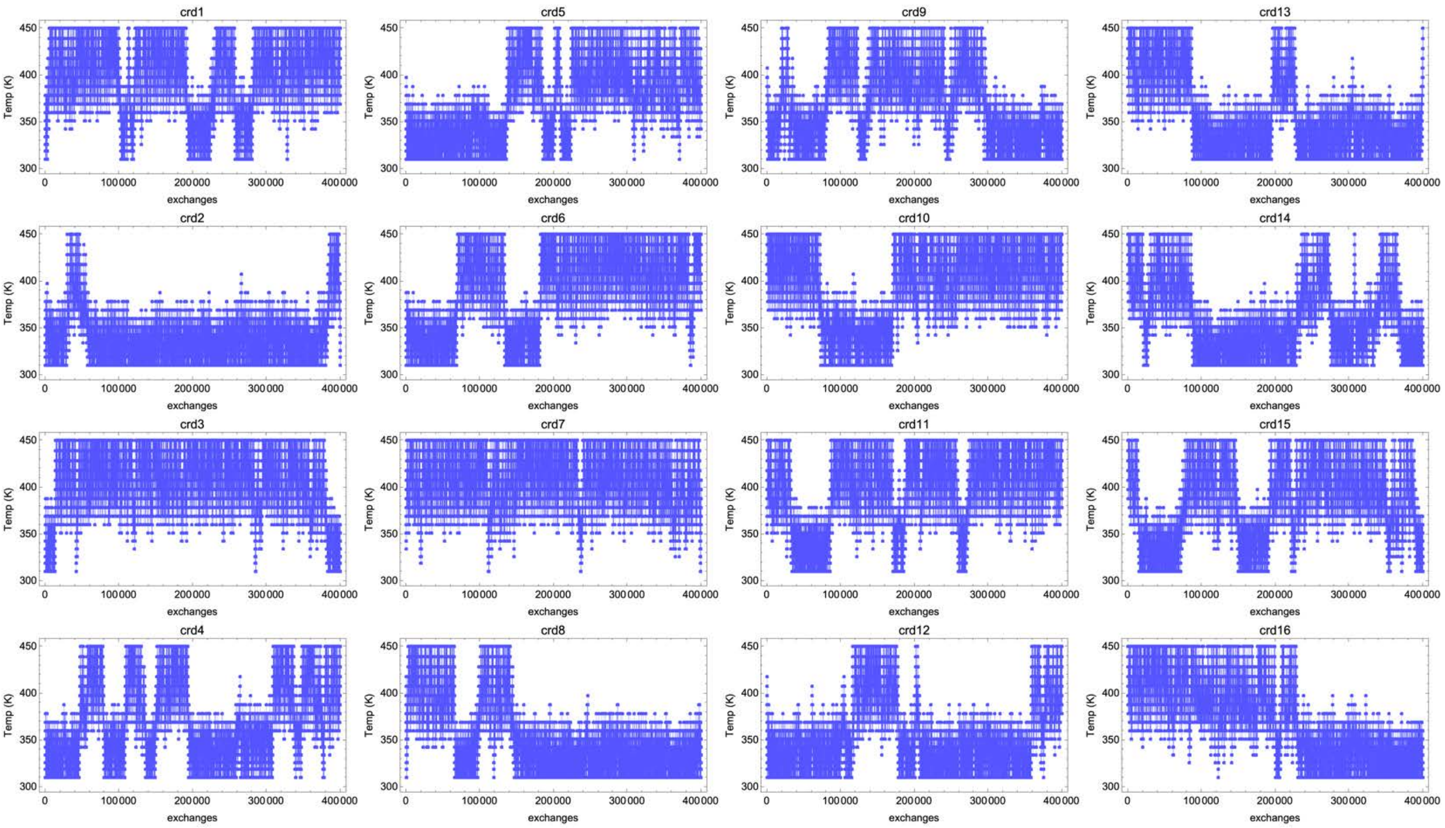

Figure S101. Passage of the 16 sets of coordinates (crd1–crd16) through the 16 available temperature replicas (310.00, 317.80, 325.79, 333.99, 342.39, 351.00, 359.83, 368.89, 378.17, 387.68, 397.43, 407.43, 417.68, 428.19, 438.96, or 450.00 K) during the T-REMD simulation of stapled variant **s34e/29g'-x4x**.

Table S25. Summary of the passage of the 16 sets of coordinates through the 16 available temperature replicas during the T-REMD simulation of stapled variant **s34e/29g'-x4x**.[a]

Coordinate	Round Trips	Exchanges per Round Trip		Mininum Exchanges Per Round Trip	Maximum Exchanges Per Round Trip
		Average	Standard Deviation		
1	5	65595.8	28642.3257	14718	102549
2	2	199922	142568	57354	342490
3	3	127662	84738.9743	41982	243064
4	5	75304	37434.2585	37060	145134
5	4	92636	59881.4697	24266	185618
6	2	192705	59095	133610	251800
7	2	143872	18676	125196	162548
8	3	48800	15737.9097	27712	65508
9	5	58676	42374.98	6066	117732
10	0	0	0	0	0
11	2	112723	24051	88672	136774
12	3	124928.6667	68036.8627	28882	177932
13	1	141068	0	141068	141068
14	4	87220.5	59129.3735	33414	187262
15	5	75079.2	50846.025	18650	134460
16	2	54080	23684	30396	77764

[a]A round trip constitutes passage of a single coordinate through all the available temperature replicas and back to the starting point (for example, from 310.00 K to 450.00 K and back again to 310.00 K).

Table S26. Replica-exchange success rates for the 16 temperature replicas during T-REMD simulation of stapled variant **s34e/29g'-x4x**.[a]

Replica Temperature (K)	% Success Up Exchange	Up Exchange
310	43.752	0
317.8	43.368	43.752
325.79	42.719	43.368
333.99	41.627	42.719
342.39	39.027	41.627
351	32.823	39.027
359.83	26.682	32.823
368.89	32.094	26.682
378.17	38.268	32.094
387.68	40.212	38.268
397.43	40.501	40.212
407.43	41.172	40.501
417.68	41.931	41.172
428.19	42.679	41.931
438.96	43.224	42.679
450	0	43.224

[a]The up exchange for a given replica is with the adjacent higher-temperature replica, except for the 450.00 K replica, which is with the 310.00 K replica. Similarly, the down exchange for a given replica is with the adjacent lower-temperature replica, except for the 310.00 K replica, which is with the 450.00 K replica.

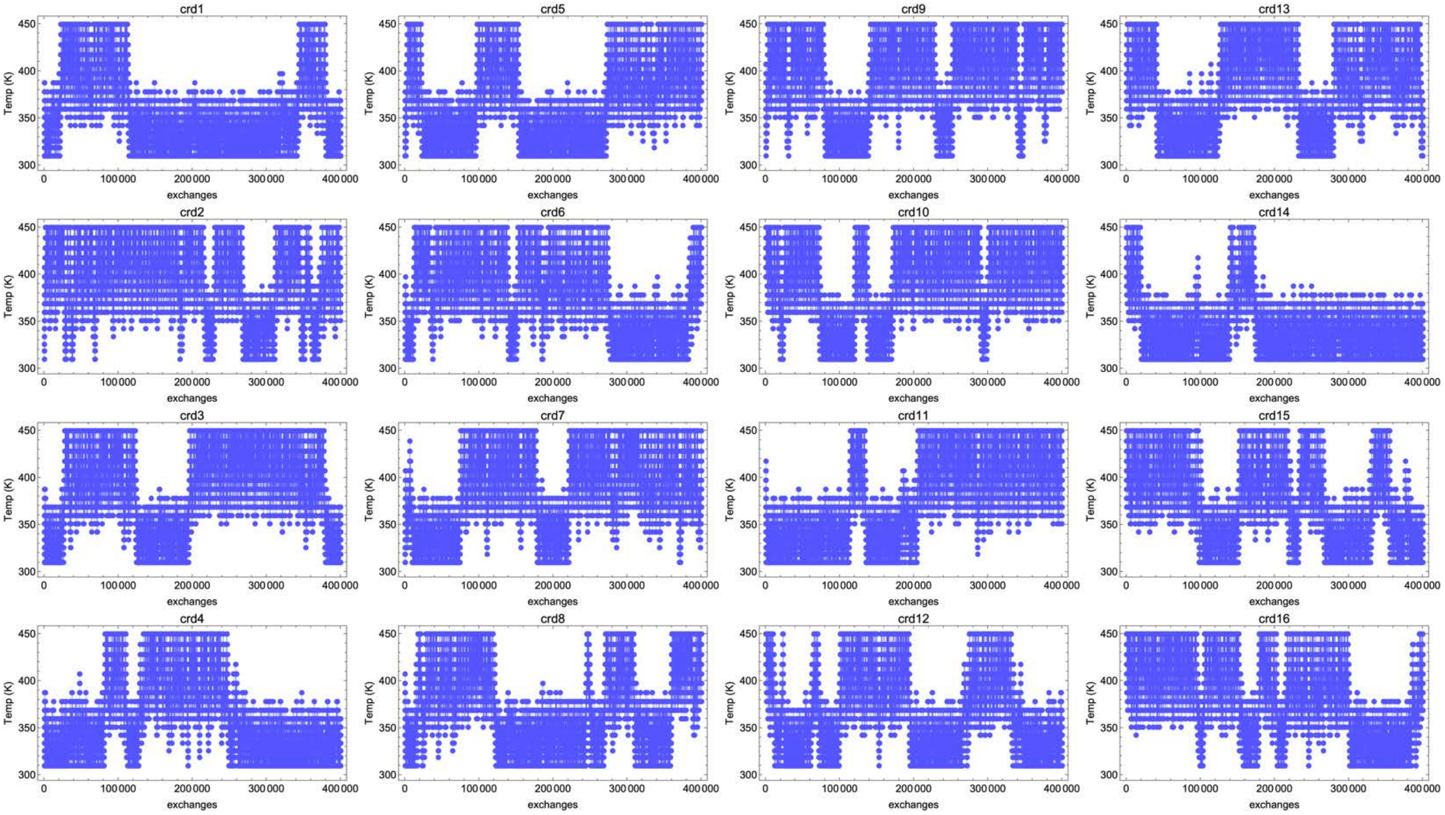

Figure S102. Passage of the 16 sets of coordinates (crd1–crd16) through the 16 available temperature replicas (310.00, 317.80, 325.79, 333.99, 342.39, 351.00, 359.83, 368.89, 378.17, 387.68, 397.43, 407.43, 417.68, 428.19, 438.96, or 450.00 K) during the T-REMD simulation of non-stapled variant **7f/10b’-xx**.

Table S27. Summary of the passage of the 16 sets of coordinates through the 16 available temperature replicas during the T-REMD simulation of non-stapled variant **7f/10b’-xx**.[a]

Coordinate	Round Trips	Exchanges per Round Trip: Average	Exchanges per Round Trip: Standard Deviation	Mininum Exchanges Per Round Trip	Maximum Exchanges Per Round Trip
1	2	190005.5	75754.5	114251	265760
2	8	45178.25	33248.8122	8940	114972
3	2	189609	65351	124258	254960
4	3	83032.6667	23218.3348	55360	112176
5	2	76709	53893	22816	130602
6	4	69300	28399.4303	37364	102088
7	2	185163	6877	178286	192040
8	4	77550	39629.2863	22972	127788
9	4	85381	47996.6518	30444	150464
10	3	90689.3333	45049.6009	53746	154112
11	1	134962	0	134962	134962
12	5	64320	43147.5704	11814	138724
13	2	178296	12478	165818	190774
14	1	155136	0	155136	155136
15	3	85881.3333	29779.3277	48150	120948
16	4	50509.5	6587.0657	42534	58448

[a]A round trip constitutes passage of a single coordinate through all the available temperature replicas and back to the starting point (for example, from 310.00 K to 450.00 K and back again to 310.00 K).

Table S28. Replica-exchange success rates for the 16 temperature replicas during T-REMD simulation of non-stapled variant **7f/10b’-xx**.[a]

Replica Temperature (K)	% Success	
	Up Exchange	Up Exchange
310	44.044	0
317.8	43.645	44.044
325.79	42.8	43.645
333.99	41.869	42.8
342.39	39.856	41.869
351	32.874	39.856
359.83	24.992	32.874
368.89	33.031	24.992
378.17	38.547	33.031
387.68	40.215	38.547
397.43	41.066	40.215
407.43	41.867	41.066
417.68	42.486	41.867
428.19	43.513	42.486
438.96	43.988	43.513
450	0	43.988

[a]The up exchange for a given replica is with the adjacent higher-temperature replica, except for the 450.00 K replica, which is with the 310.00 K replica. Similarly, the down exchange for a given replica is with the adjacent lower-temperature replica, except for the 310.00 K replica, which is with the 450.00 K replica.

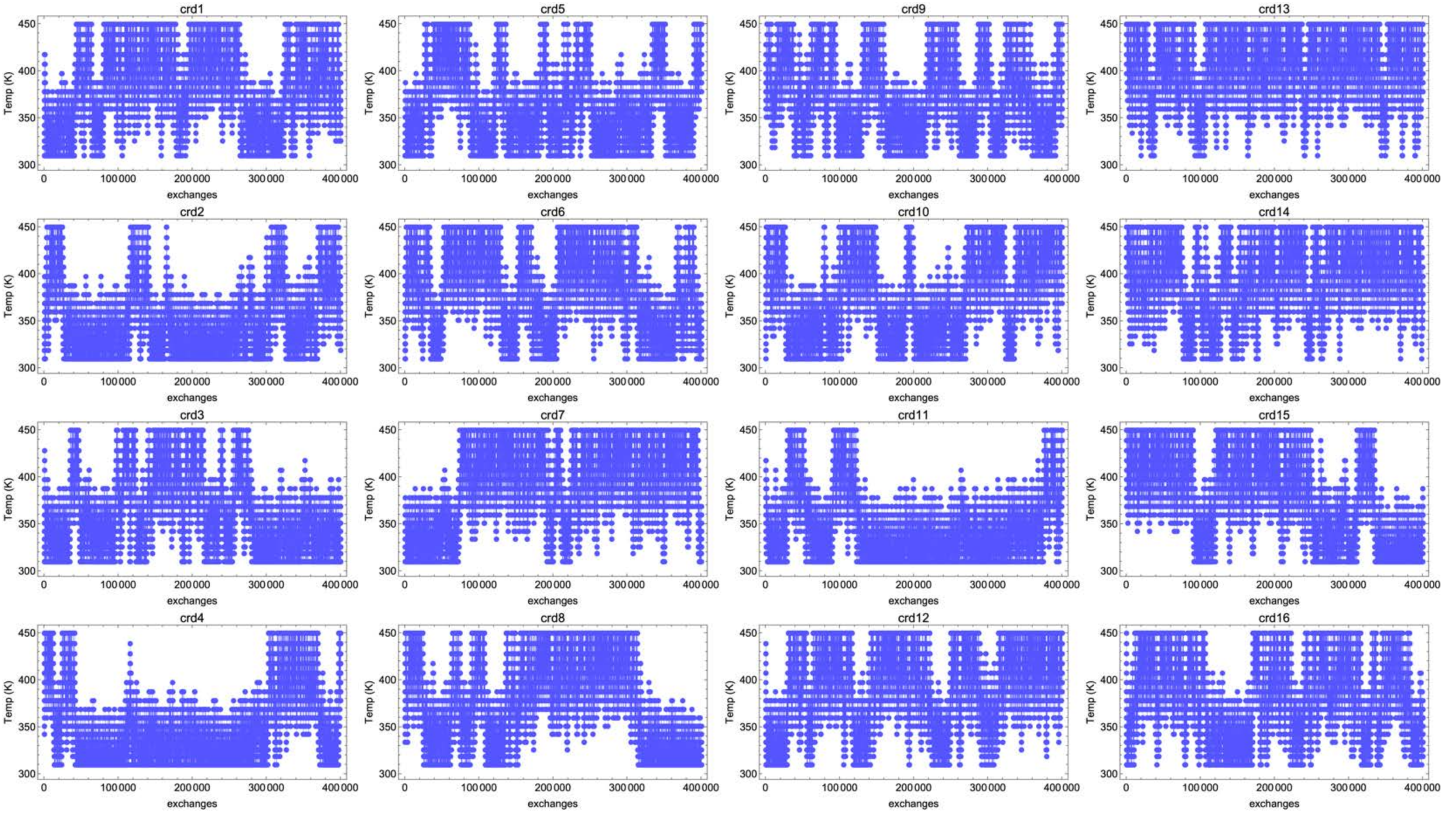

Figure S103. Passage of the 16 sets of coordinates (crd1–crd16) through the 16 available temperature replicas (310.00, 317.80, 325.79, 333.99, 342.39, 351.00, 359.83, 368.89, 378.17, 387.68, 397.43, 407.43, 417.68, 428.19, 438.96, or 450.00 K) during the T-REMD simulation of stapled variant **s7f/10b’-x4x**.

Table S29. Summary of the passage of the 16 sets of coordinates through the 16 available temperature replicas during the T-REMD simulation of stapled variant **s7f/10b’-x4x**.[a]

Coordinate	Round Trips	Exchanges per Round Trip		Mininum Exchanges Per Round Trip	Maximum Exchanges Per Round Trip
		Average	Standard Deviation		
1	8	44689.125	23956.8779	10594	85672
2	5	65194.8	56386.4879	12794	158280
3	9	31093.3333	18900.1076	8494	61982
4	3	119412.6667	106978.6939	28106	269536
5	9	39057.5556	27776.7841	5784	99898
6	9	43688.4444	28289.5899	10660	95968
7	6	66158.3333	72308.1376	2038	191606
8	5	57930.8	55341.0313	9150	165432
9	6	53508.6667	25907.714	15742	100122
10	6	53894	33632.5758	19748	123600
11	4	98251	101985.7738	2782	269858
12	7	41591.4286	18792.9982	12932	75256
13	8	46220.25	28166.6888	13612	86058
14	10	32191.4	23140.0847	5864	81894
15	5	48912.8	26268.3118	10380	84296
16	12	31950.1667	21745.0523	2118	73492

[a]A round trip constitutes passage of a single coordinate through all the available temperature replicas and back to the starting point (for example, from 310.00 K to 450.00 K and back again to 310.00 K).

Table S30. Replica-exchange success rates for the 16 temperature replicas during T-REMD simulation of stapled variant **s7f/10b'-x4x**.[a]

Replica Temperature (K)	% Success	
	**Up Exchange**	**Up Exchange**
310	41.745	0
317.8	41.868	41.745
325.79	41.492	41.868
333.99	40.775	41.492
342.39	39.475	40.775
351	36.461	39.475
359.83	33.151	36.461
368.89	32.206	33.151
378.17	34.907	32.206
387.68	37.848	34.907
397.43	39.629	37.848
407.43	40.855	39.629
417.68	41.718	40.855
428.19	42.61	41.718
438.96	42.929	42.61
450	0	42.929

[a]The up exchange for a given replica is with the adjacent higher-temperature replica, except for the 450.00 K replica, which is with the 310.00 K replica. Similarly, the down exchange for a given replica is with the adjacent lower-temperature replica, except for the 310.00 K replica, which is with the 450.00 K replica.

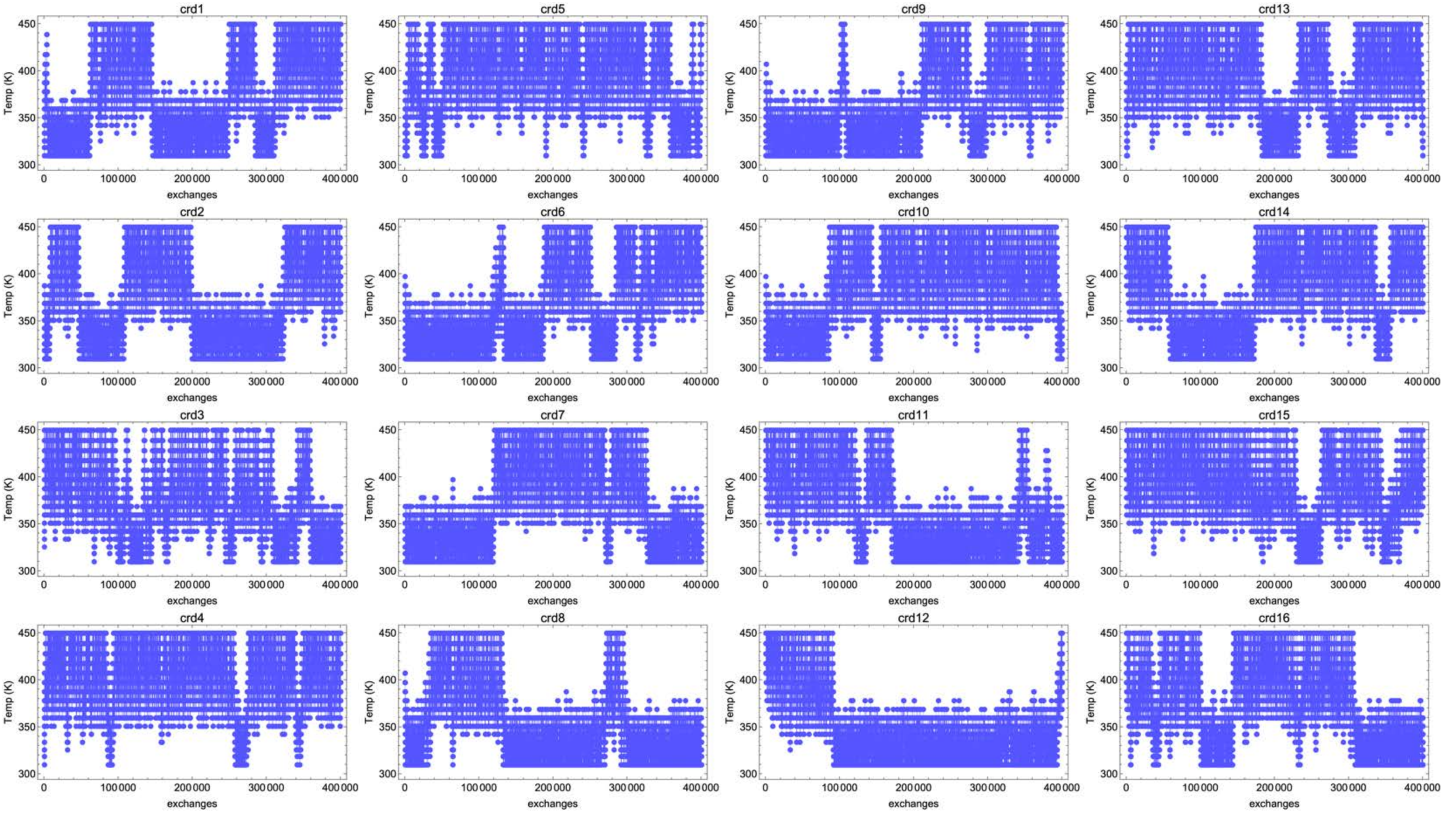

Figure S104. Passage of the 16 sets of coordinates (crd1–crd16) through the 16 available temperature replicas (310.00, 317.80, 325.79, 333.99, 342.39, 351.00, 359.83, 368.89, 378.17, 387.68, 397.43, 407.43, 417.68, 428.19, 438.96, or 450.00 K) during the T-REMD simulation of non-stapled variant **14f/17b'-xx**.

Table S31. Summary of the passage of the 16 sets of coordinates through the 16 available temperature replicas during the T-REMD simulation of non-stapled variant **14f/17b'-xx**.[a]

Coordinate	Round Trips	Exchanges per Round Trip		Mininum Exchanges Per Round Trip	Maximum Exchanges Per Round Trip
		Average	Standard Deviation		
1	2	142420.5	4042.5	138378	146463
2	2	99638	52726	46912	152364
3	9	32539.1111	21773.4525	3364	80002
4	3	113726.6667	40827.869	83488	171444
5	7	55956.8571	44163.3734	16382	150888
6	3	103773.3333	31542.6554	60114	133528
7	2	163525	107537	55988	271062
8	4	73726.5	55378.3616	3854	158934
9	3	118581.3333	35858.937	80540	166644
10	2	196776	52530	144246	249306
11	2	116811	66651	50160	183462
12	0	0	0	0	0
13	3	133164	37885.724	90654	182662
14	1	277690	0	277690	277690
15	2	80222	34842	45380	115064
16	4	75703.5	36240.1005	30730	131342

[a]A round trip constitutes passage of a single coordinate through all the available temperature replicas and back to the starting point (for example, from 310.00 K to 450.00 K and back again to 310.00 K).

Table S32. Replica-exchange success rates for the 16 temperature replicas during T-REMD simulation of non-stapled variant **14f/17b'-xx**.[a]

Replica Temperature (K)	% Success	
	Up Exchange	Up Exchange
310	44.073	0
317.8	43.901	44.073
325.79	43.224	43.901
333.99	42.103	43.224
342.39	38.343	42.103
351	26.971	38.343
359.83	28.002	26.971
368.89	36.795	28.002
378.17	39.659	36.795
387.68	41.154	39.659
397.43	41.756	41.154
407.43	42.396	41.756
417.68	42.939	42.396
428.19	43.41	42.939
438.96	43.813	43.41
450	0	43.813

[a]The up exchange for a given replica is with the adjacent higher-temperature replica, except for the 450.00 K replica, which is with the 310.00 K replica. Similarly, the down exchange for a given replica is with the adjacent lower-temperature replica, except for the 310.00 K replica, which is with the 450.00 K replica.

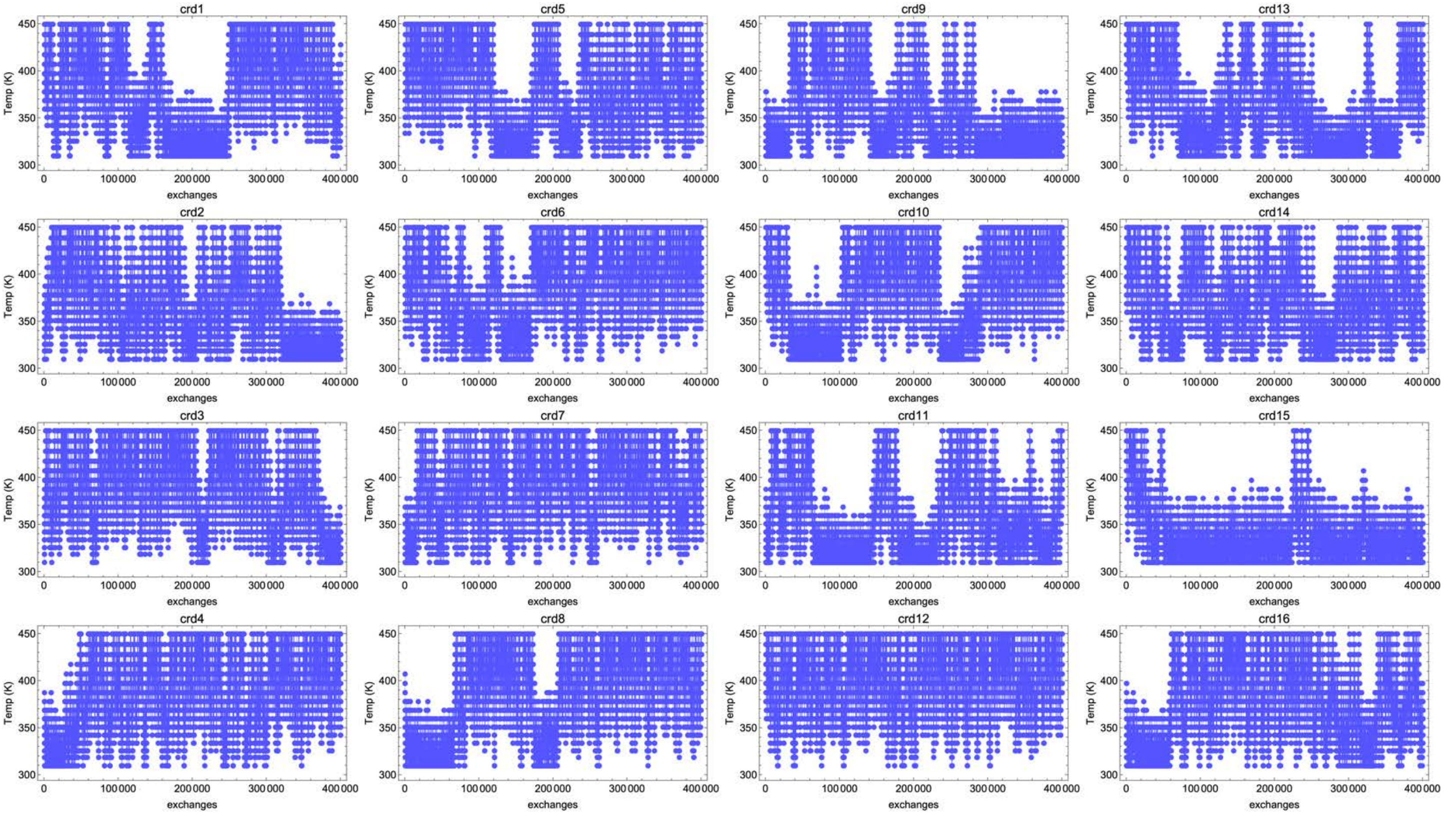

Figure S105. Passage of the 16 sets of coordinates (crd1–crd16) through the 16 available temperature replicas (310.00, 317.80, 325.79, 333.99, 342.39, 351.00, 359.83, 368.89, 378.17, 387.68, 397.43, 407.43, 417.68, 428.19, 438.96, or 450.00 K) during the T-REMD simulation of stapled variant **s14f/17b'-x4x**.

Table S33. Summary of the passage of the 16 sets of coordinates through the 16 available temperature replicas during the T-REMD simulation of stapled variant **s14f/17b'-x4x**.[a]

Coordinate	Round Trips	Exchanges per Round Trip		Mininum Exchanges Per Round Trip	Maximum Exchanges Per Round Trip
		Average	Standard Deviation		
1	6	62851	75940.2367	6018	230050
2	21	15172.2857	9127.5746	5360	39944
3	8	46384.75	43378.2302	7174	134250
4	15	23097.2	12931.0814	7204	51508
5	17	16962.2353	14578.8663	3208	58098
6	11	26249.8182	23109.3023	5122	87246
7	13	28604.4615	20201.883	3754	80310
8	7	46809.7143	27381.9067	6850	85672
9	13	21618.4615	15199.2996	4132	49076
10	4	83007.5	30482.4557	43068	128542
11	17	23318.5882	23332.0558	1902	92172
12	11	26007.4545	16985.2637	1614	60488
13	8	37043.5	29629.6867	5704	94574
14	20	18263.9	13963.0896	2722	52926
15	5	44892.4	69494.053	5414	183386
16	19	20855.3158	16634.6224	3808	74599

[a]A round trip constitutes passage of a single coordinate through all the available temperature replicas and back to the starting point (for example, from 310.00 K to 450.00 K and back again to 310.00 K).

Table S34. Replica-exchange success rates for the 16 temperature replicas during T-REMD simulation of stapled variant **s14f/17b'-x4x**.[a]

**Replica Temperature (K)**	**% Success**	
	**Up Exchange**	**Up Exchange**
310	41.474	0.001
317.8	40.626	41.474
325.79	38.876	40.626
333.99	36.842	38.876
342.39	35.776	36.842
351	36.459	35.776
359.83	36.693	36.459
368.89	37.658	36.693
378.17	38.591	37.658
387.68	38.964	38.591
397.43	39.82	38.964
407.43	40.797	39.82
417.68	41.581	40.797
428.19	42.255	41.581
438.96	43.376	42.255
450	0.001	43.376

[a]The up exchange for a given replica is with the adjacent higher-temperature replica, except for the 450.00 K replica, which is with the 310.00 K replica. Similarly, the down exchange for a given replica is with the adjacent lower-temperature replica, except for the 310.00 K replica, which is with the 450.00 K replica.

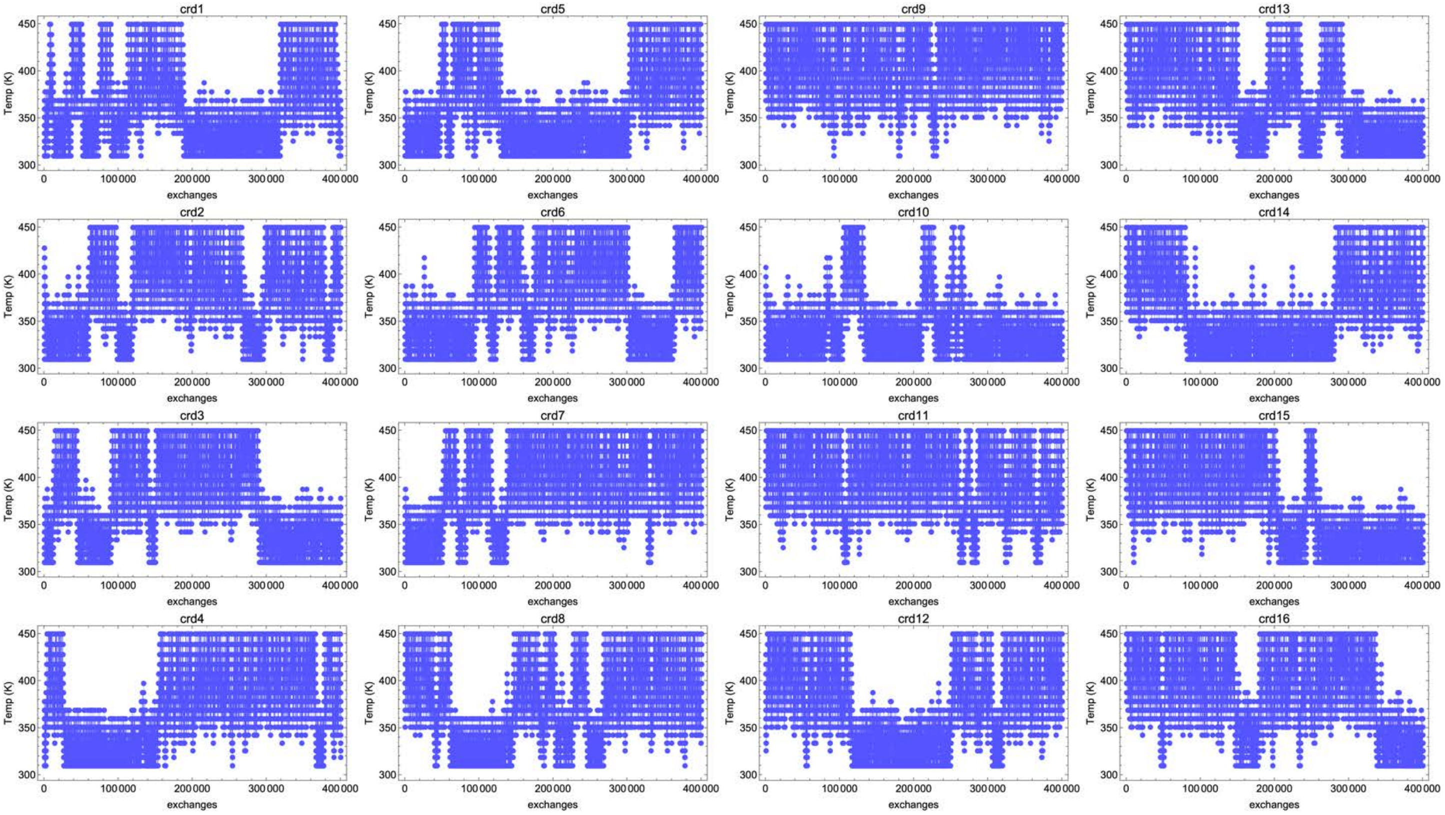

Figure S106. Passage of the 16 sets of coordinates (crd1–crd16) through the 16 available temperature replicas (310.00, 317.80, 325.79, 333.99, 342.39, 351.00, 359.83, 368.89, 378.17, 387.68, 397.43, 407.43, 417.68, 428.19, 438.96, or 450.00 K) during the T-REMD simulation of non-stapled variant **21f/24b'-xx**.

Table S35. Summary of the passage of the 16 sets of coordinates through the 16 available temperature replicas during the T-REMD simulation of non-stapled variant **21f/24b'-xx**.[a]

Coordinate	Round Trips	Exchanges per Round Trip		Mininum Exchanges Per Round Trip	Maximum Exchanges Per Round Trip
		Average	Standard Deviation		
1	6	66311.5	65503.9447	12353	209752
2	4	94811	49668.33	30696	169312
3	3	96766.6667	42442.3913	44982	148942
4	4	91665.5	40990.8865	26192	136148
5	3	43168.6667	9486.5701	36002	56574
6	3	100390	41795.2673	43496	142714
7	3	109449.3333	72657.6406	45784	211130
8	5	67505.6	51051.5412	16000	131812
9	2	67065	20877	46188	87942
10	4	66584.5	50003.4113	10516	133440
11	4	64621	53856.2958	16576	155984
12	2	125825	63577	62248	189402
13	2	71112	13820	57292	84932
14	0	0	0	0	0
15	3	81624	72750.0324	13144	182360
16	4	72870.5	30431.1803	35710	105114

[a]A round trip constitutes passage of a single coordinate through all the available temperature replicas and back to the starting point (for example, from 310.00 K to 450.00 K and back again to 310.00 K).

Table S36. Replica-exchange success rates for the 16 temperature replicas during T-REMD simulation of non-stapled variant **21f/24b'-xx**.[a]

Replica Temperature (K)	% Success	
	Up Exchange	Up Exchange
310	43.984	0
317.8	43.489	43.984
325.79	42.773	43.489
333.99	40.869	42.773
342.39	35.142	40.869
351	27.81	35.142
359.83	29.965	27.81
368.89	37.327	29.965
378.17	39.948	37.327
387.68	40.858	39.948
397.43	41.697	40.858
407.43	42.235	41.697
417.68	42.664	42.235
428.19	43.125	42.664
438.96	43.966	43.125
450	0	43.966

[a]The up exchange for a given replica is with the adjacent higher-temperature replica, except for the 450.00 K replica, which is with the 310.00 K replica. Similarly, the down exchange for a given replica is with the adjacent lower-temperature replica, except for the 310.00 K replica, which is with the 450.00 K replica.

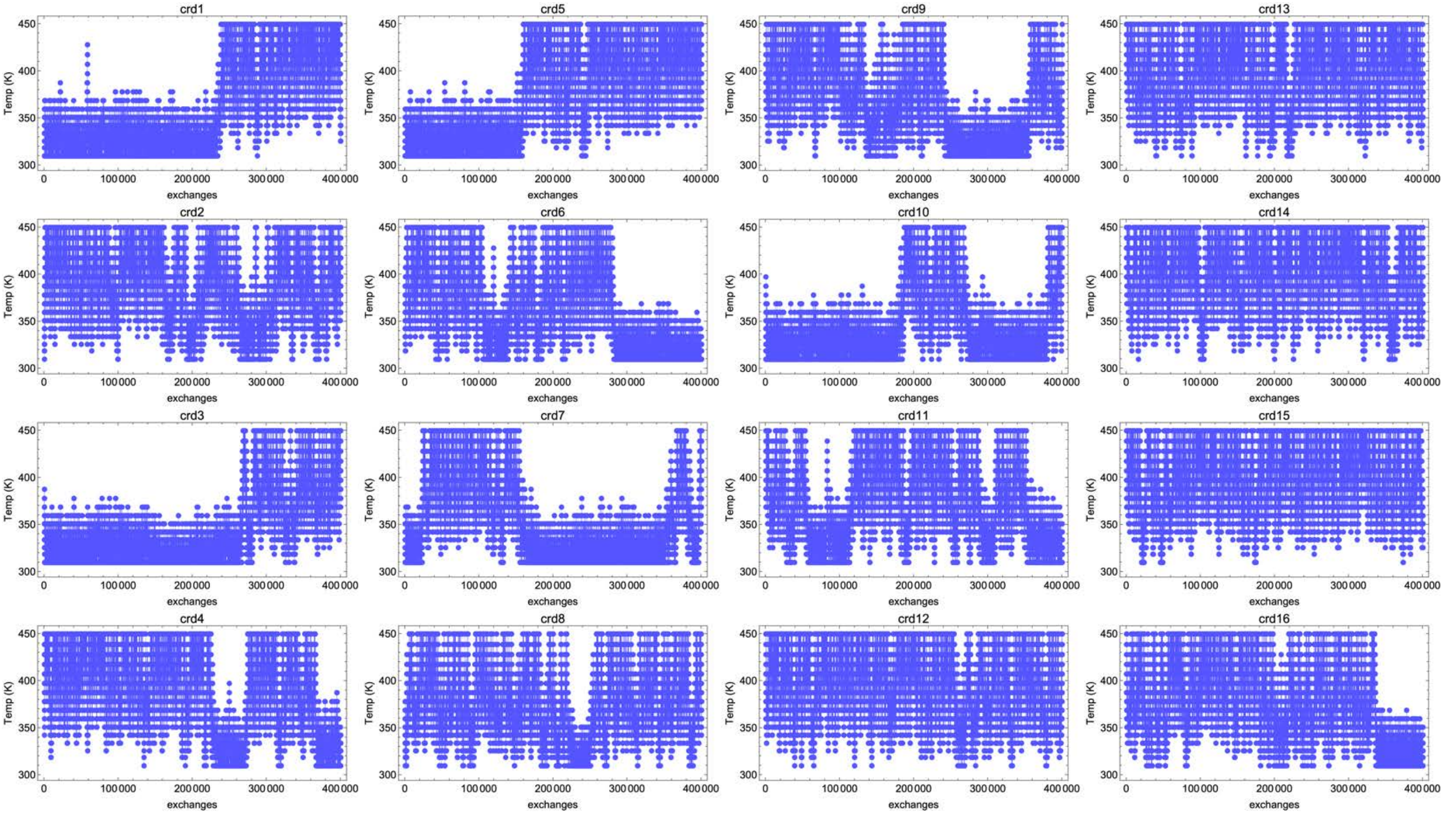

Figure S107. Passage of the 16 sets of coordinates (crd1–crd16) through the 16 available temperature replicas (310.00, 317.80, 325.79, 333.99, 342.39, 351.00, 359.83, 368.89, 378.17, 387.68, 397.43, 407.43, 417.68, 428.19, 438.96, or 450.00 K) during the T-REMD simulation of stapled variant **s21f/24b'-x4x**.

Table S37. Summary of the passage of the 16 sets of coordinates through the 16 available temperature replicas during the T-REMD simulation of stapled variant **s21f/24b'-x4x**.[a]

Coordinate	Round Trips	Exchanges per Round Trip		Mininum Exchanges Per Round Trip	Maximum Exchanges Per Round Trip
		Average	Standard Deviation		
1	1	287483	0	287483	287483
2	10	38058.8	25639.4619	14752	98992
3	3	112220	115049.188	11676	273274
4	5	46445.2	26168.1797	12240	87782
5	1	238408	0	238408	238408
6	8	30040.75	20081.5182	8352	69398
7	3	128943.3333	84068.1885	25906	231830
8	13	29472.1538	17816.1665	4146	61032
9	4	43858	17976.7764	22842	69484
10	6	64664.6667	75601.8897	8202	211284
11	8	40565.25	21170.8491	16854	83364
12	8	42378	31846.8194	9222	116018
13	7	40365.4286	32676.4868	14312	105416
14	5	67656.4	31477.5288	25460	99880
15	3	117682	71667.1808	25562	200344
16	13	23799.8462	25400.5223	2618	101222

[a]A round trip constitutes passage of a single coordinate through all the available temperature replicas and back to the starting point (for example, from 310.00 K to 450.00 K and back again to 310.00 K).

Table S38. Replica-exchange success rates for the 16 temperature replicas during T-REMD simulation of stapled variant **s21f/24b’-x4x**.[a]

**Replica Temperature (K)**	**% Success**	
	**Up Exchange**	**Up Exchange**
310	42.505	0
317.8	41.599	42.505
325.79	39.12	41.599
333.99	34.47	39.12
342.39	31.435	34.47
351	33.911	31.435
359.83	36.896	33.911
368.89	38.099	36.896
378.17	39.092	38.099
387.68	39.519	39.092
397.43	40.232	39.519
407.43	40.871	40.232
417.68	41.672	40.871
428.19	42.189	41.672
438.96	42.853	42.189
450	0	42.853

[a]The up exchange for a given replica is with the adjacent higher-temperature replica, except for the 450.00 K replica, which is with the 310.00 K replica. Similarly, the down exchange for a given replica is with the adjacent lower-temperature replica, except for the 310.00 K replica, which is with the 450.00 K replica.

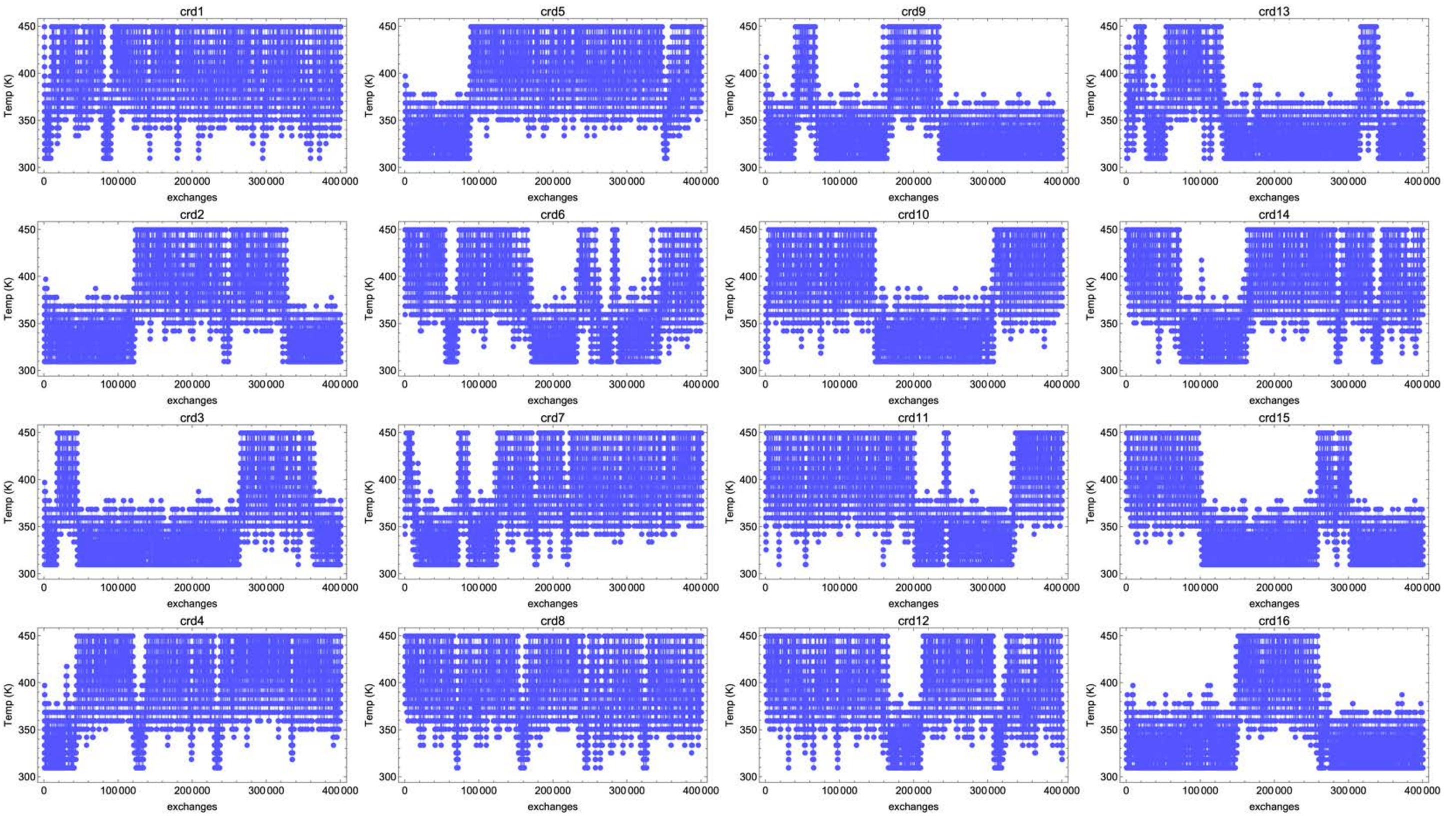

Figure S108. Passage of the 16 sets of coordinates (crd1–crd16) through the 16 available temperature replicas (310.00, 317.80, 325.79, 333.99, 342.39, 351.00, 359.83, 368.89, 378.17, 387.68, 397.43, 407.43, 417.68, 428.19, 438.96, or 450.00 K) during the T-REMD simulation of non-stapled variant **28f/31b'-xx**.

Table S39. Summary of the passage of the 16 sets of coordinates through the 16 available temperature replicas during the T-REMD simulation of non-stapled variant **28f/31b'-xx**.[a]

**Coordinate**	**Round Trips**	**Exchanges per Round Trip**		**Mininum Exchanges Per Round Trip**	**Maximum Exchanges Per Round Trip**
		**Average**	**Standard Deviation**		
1	7	52976.8571	25567.9572	12152	86702
2	4	81953.5	96807.3251	4478	242252
3	3	121662.6667	125000.8028	22438	297978
4	2	114874	8828	106046	123702
5	1	350046	0	350046	350046
6	5	56214.8	38080.8731	8552	115916
7	3	68326	18707.3747	44046	89566
8	5	50400.4	34295.3168	6518	86638
9	3	77762.6667	9377.5727	67314	90060
10	1	147598	0	147598	147598
11	4	57405.5	27788.8342	35604	105063
12	4	69533	45042.2118	32410	143458
13	5	67865.6	73901.4413	7524	207788
14	3	96705.3333	79889.6849	30778	209128
15	2	100019	82333	17686	182352
16	1	259615	0	259615	259615

[a]A round trip constitutes passage of a single coordinate through all the available temperature replicas and back to the starting point (for example, from 310.00 K to 450.00 K and back again to 310.00 K).

Table S40. Replica-exchange success rates for the 16 temperature replicas during T-REMD simulation of non-stapled variant **28f/31b'-xx**.[a]

Replica Temperature (K)	% Success	
	Up Exchange	Up Exchange
310	44.215	0.001
317.8	43.747	44.215
325.79	42.954	43.747
333.99	41.417	42.954
342.39	36.753	41.417
351	26.704	36.753
359.83	30.657	26.704
368.89	36.502	30.657
378.17	39.498	36.502
387.68	40.611	39.498
397.43	41.607	40.611
407.43	42.178	41.607
417.68	42.551	42.178
428.19	43.609	42.551
438.96	43.8	43.609
450	0.001	43.8

[a]The up exchange for a given replica is with the adjacent higher-temperature replica, except for the 450.00 K replica, which is with the 310.00 K replica. Similarly, the down exchange for a given replica is with the adjacent lower-temperature replica, except for the 310.00 K replica, which is with the 450.00 K replica.

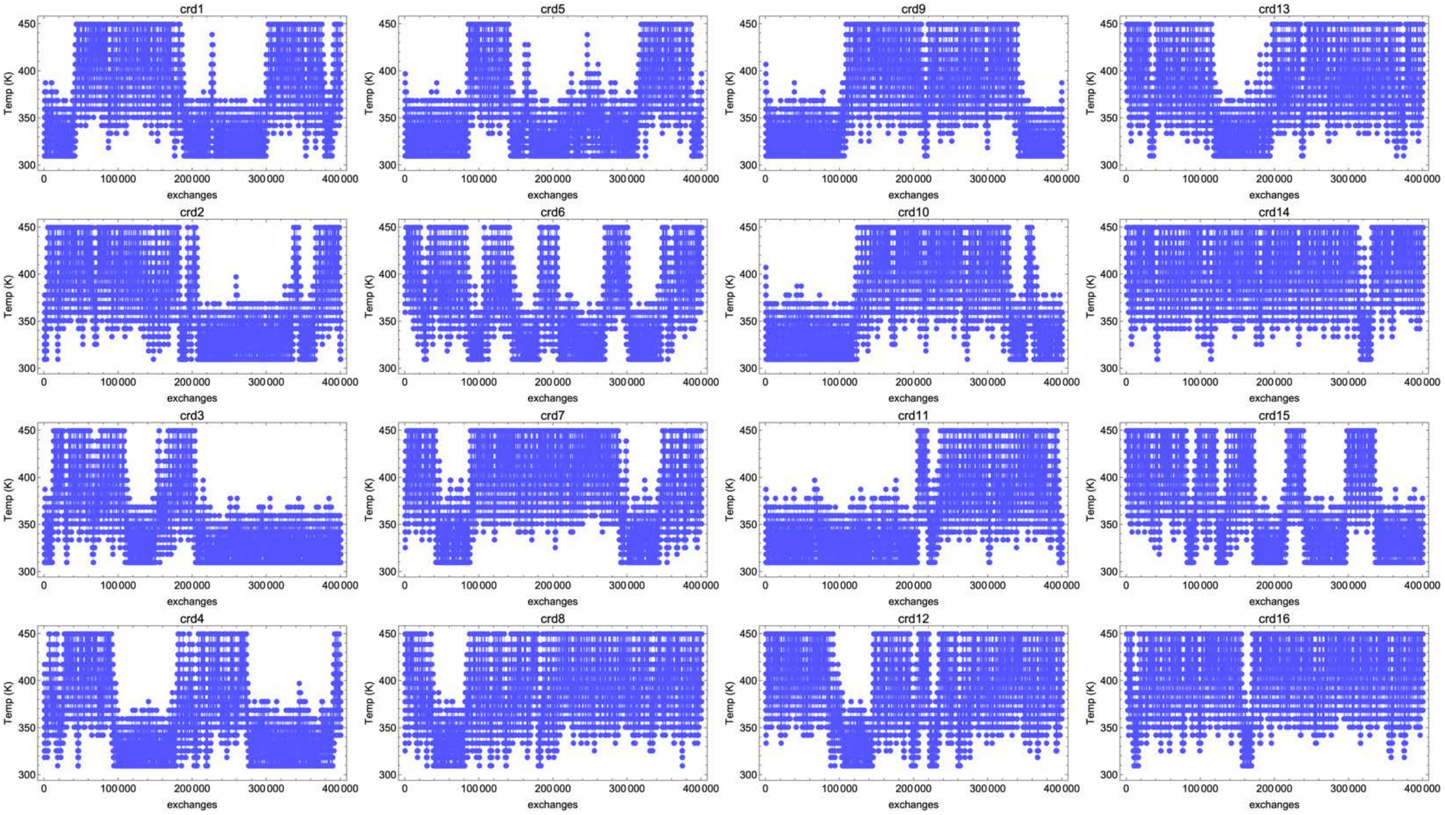

Figure S109. Passage of the 16 sets of coordinates (crd1–crd16) through the 16 available temperature replicas (310.00, 317.80, 325.79, 333.99, 342.39, 351.00, 359.83, 368.89, 378.17, 387.68, 397.43, 407.43, 417.68, 428.19, 438.96, or 450.00 K) during the T-REMD simulation of stapled variant **s28f/31b’-x4x**.

Table S41. Summary of the passage of the 16 sets of coordinates through the 16 available temperature replicas during the T-REMD simulation of stapled variant **s28f/31b’-x4x**.[a]

Coordinate	Round Trips	Exchanges per Round Trip		Mininum Exchanges Per Round Trip	Maximum Exchanges Per Round Trip
		Average	Standard Deviation		
1	4	94605.75	75764.7342	12688	175665
2	7	57052.5714	50361.2367	8502	137516
3	5	40794.4	6867.8845	29988	48228
4	6	42983.3333	26582.042	15792	91326
5	3	128912.6667	49931.5281	62184	182282
6	6	54796.6667	22167.2891	26458	94626
7	2	156000	93670	62330	249670
8	6	57462.3333	63881.5263	8858	194396
9	2	170265	44061	126204	214326
10	3	120497.3333	107262.449	32266	271474
11	3	131972.6667	62488.145	81100	219988
12	4	42932.5	36798.9432	5402	103348
13	4	85598	46526.0451	9574	127972
14	2	137389	64627	72762	202016
15	4	63510.5	21080.758	40036	95524
16	1	146412	0	146412	146412

[a]A round trip constitutes passage of a single coordinate through all the available temperature replicas and back to the starting point (for example, from 310.00 K to 450.00 K and back again to 310.00 K).

Table S42. Replica-exchange success rates for the 16 temperature replicas during T-REMD simulation of stapled variant **s28f/31b'-x4x**.[a]

Replica Temperature (K)	% Success	
	Up Exchange	Up Exchange
310	42.627	0
317.8	42.152	42.627
325.79	41.159	42.152
333.99	38.895	41.159
342.39	33.735	38.895
351	28.182	33.735
359.83	33.919	28.182
368.89	37.484	33.919
378.17	39.009	37.484
387.68	39.974	39.009
397.43	40.532	39.974
407.43	41.109	40.532
417.68	41.839	41.109
428.19	42.73	41.839
438.96	43.262	42.73
450	0	43.262

[a]The up exchange for a given replica is with the adjacent higher-temperature replica, except for the 450.00 K replica, which is with the 310.00 K replica. Similarly, the down exchange for a given replica is with the adjacent lower-temperature replica, except for the 310.00 K replica, which is with the 450.00 K replica.

(return to Table of Contents)

9. Median energy frames of stapled vs. non-stapled variants from each temperature-centric trajectory

Using cpptraj, we converted the sixteen coordinate-centric trajectory files for each T-REMD simulation (which follow the same set of coordinates through the various temperature replicas) into the corresponding sixteen temperature-centric trajectory files, in which each frame has the same target temperature (310.00, 317.80, 325.79, 333.99, 342.39, 351.00, 359.83, 368.89, 378.17, 387.68, 397.43, 407.43, 417.68, 428.19, 438.96, or 450.00 K). Shown in Figures S110-S129 are ribbon diagrams of median-energy frames extracted from the 2000-ns T-REMD trajectories of variants **s6e/1g'-x4x**, **s13e/8g'-x4x**, **s20e/15g'-x4x**, **s27e/22g'-x4x**, **s27e/22g'-z4x**, **s34e/29g'-x4x**, **s7f/10b'-x4x, s14f/17b'-x4x, s21f/24b'-x4x,** and **s28f/31b'-x4x**; for their non-stapled counterparts variants **6e/1g'-xx**, **13e/8g'-xx**, **20e/15g'-xx**, **27e/22g'-xx**, **27e/22g'-z4x**, **34e/29g'-xx**, **7f/10b'-xx**, **14f/17b'-xx**, **21f/24b'-xx**, and **28f/31b'-xx** at the sixteen target temperatures listed above.

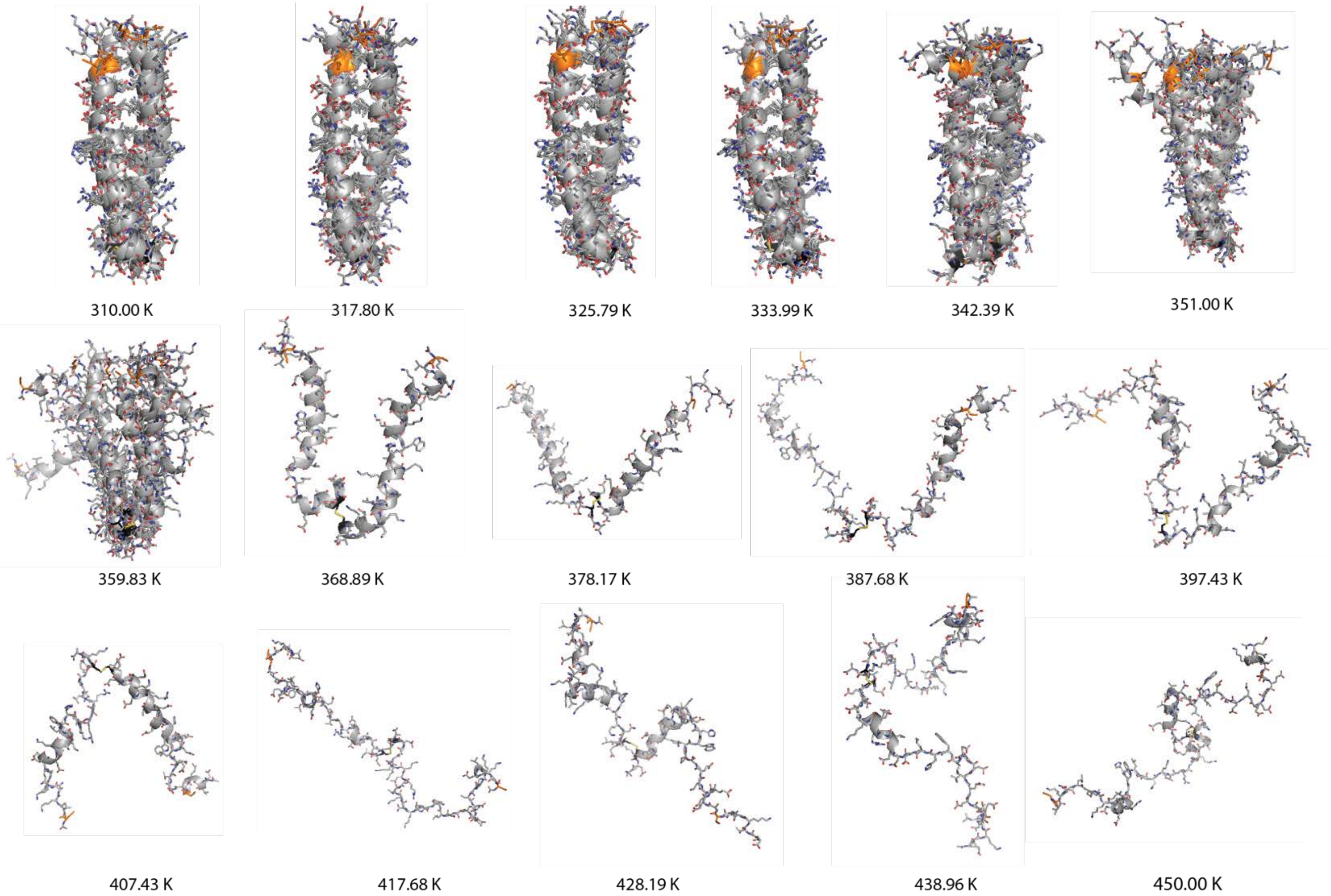

Figure S110. Ribbon diagrams of median-energy frames extracted from 2000-ns T-REMD simulations of non-stapled coiled-coil variant **6e/1g'-xx** at 310.00, 317.80, 325.79, 333.99, 342.39, 351.00, 359.83, 368.89, 378.17, 387.68, 397.43, 407.43, 417.68, 428.19, 438.96, and 450.00 K. Side chains are shown as sticks, with nitrogens and oxygens colored blue and red, respectively, and carbons colored gray, except for residues involved in the staple (where carbons are orange) or in the Cys33-Cys33' disulfide (where carbons are black). Structures for 310.00, 317.80, 325.79, 333.99, 342.39, 351.00, and 359.83 K show the five median-energy frames overlaid. At higher temperatures, the coiled coil becomes less folded, such that the five median-energy frames diverge considerably in structure. Therefore, for clarity, only the single median-energy frame is shown at 368.89, 378.17, 387.68, 397.43, 407.43, 417.68, 428.19, 438.96, and 450.00 K.

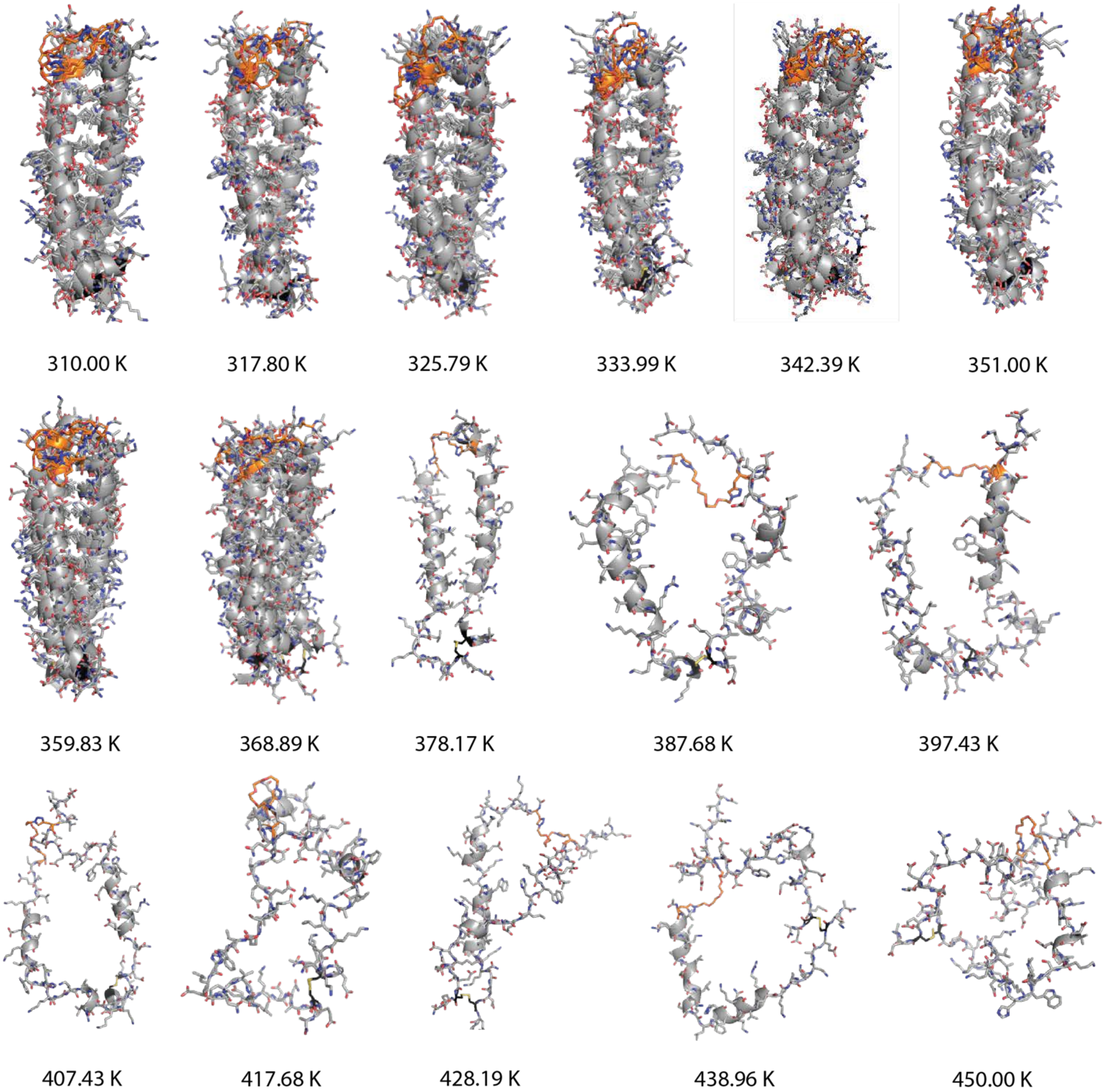

Figure S111. Ribbon diagrams of median-energy frames extracted from the last 1850 ns of the 2000-ns T-REMD simulations of stapled coiled-coil variant **s6e/1g'-x4x** at 310.00, 317.80, 325.79, 333.99, 342.39, 351.00, 359.83, 368.89, 378.17, 387.68, 397.43, 407.43, 417.68, 428.19, 438.96, and 450.00 K. Side chains are shown as sticks, with nitrogens and oxygens colored blue and red, respectively, and carbons colored gray, except for residues involved in the staple (where carbons are orange) or in the Cys33-Cys33' disulfide (where carbons are black). Structures for 310.00, 317.80, 325.79, 333.99, 342.39, 351.00, 359.83, and 368.89 K show the five median-energy frames overlaid. At higher temperatures, the coiled coil becomes less folded, such that the five median-energy frames diverge considerably in structure. Therefore, for clarity, only the single median-energy frame is shown at 378.17, 387.68, 397.43, 407.43, 417.68, 428.19, 438.96, and 450.00 K.

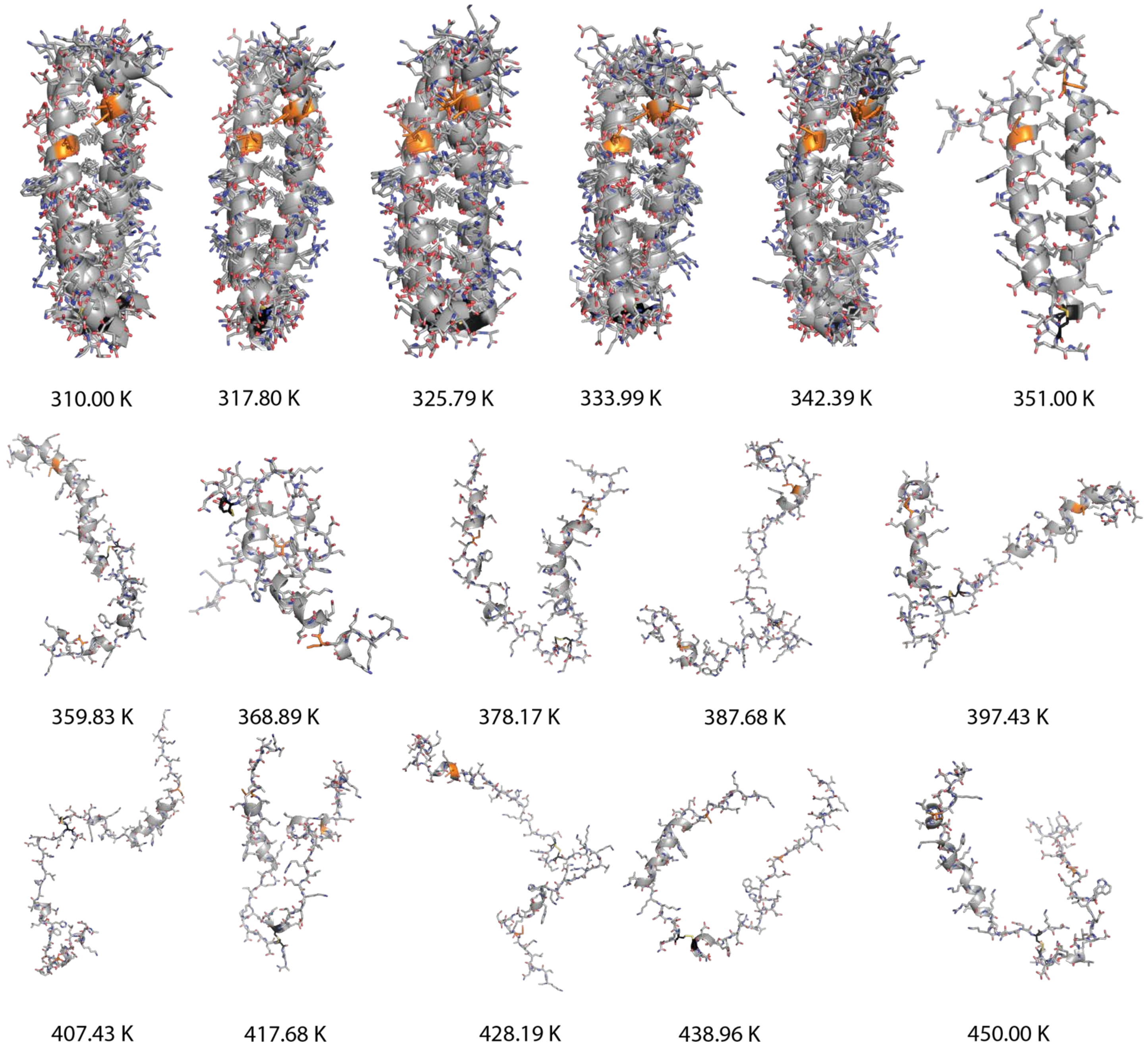

Figure S112. Ribbon diagrams of median-energy frames extracted from 2000-ns T-REMD simulations of non-stapled coiled-coil variant **13e/8g'-xx** at 310.00, 317.80, 325.79, 333.99, 342.39, 351.00, 359.83, 368.89, 378.17, 387.68, 397.43, 407.43, 417.68, 428.19, 438.96, and 450.00 K. Side chains are shown as sticks, with nitrogens and oxygens colored blue and red, respectively, and carbons colored gray, except for residues involved in the staple (where carbons are orange) or in the Cys33-Cys33' disulfide (where carbons are black). Structures for 310.00, 317.80, 325.79, 333.99, and 342.39 K show the five median-energy frames overlaid. At higher temperatures, the coiled coil becomes less folded, such that the five median-energy frames diverge considerably in structure. Therefore, for clarity, only the single median-energy frame is shown at 351.00, 359.83, 368.89, 378.17, 387.68, 397.43, 407.43, 417.68, 428.19, 438.96, and 450.00 K.

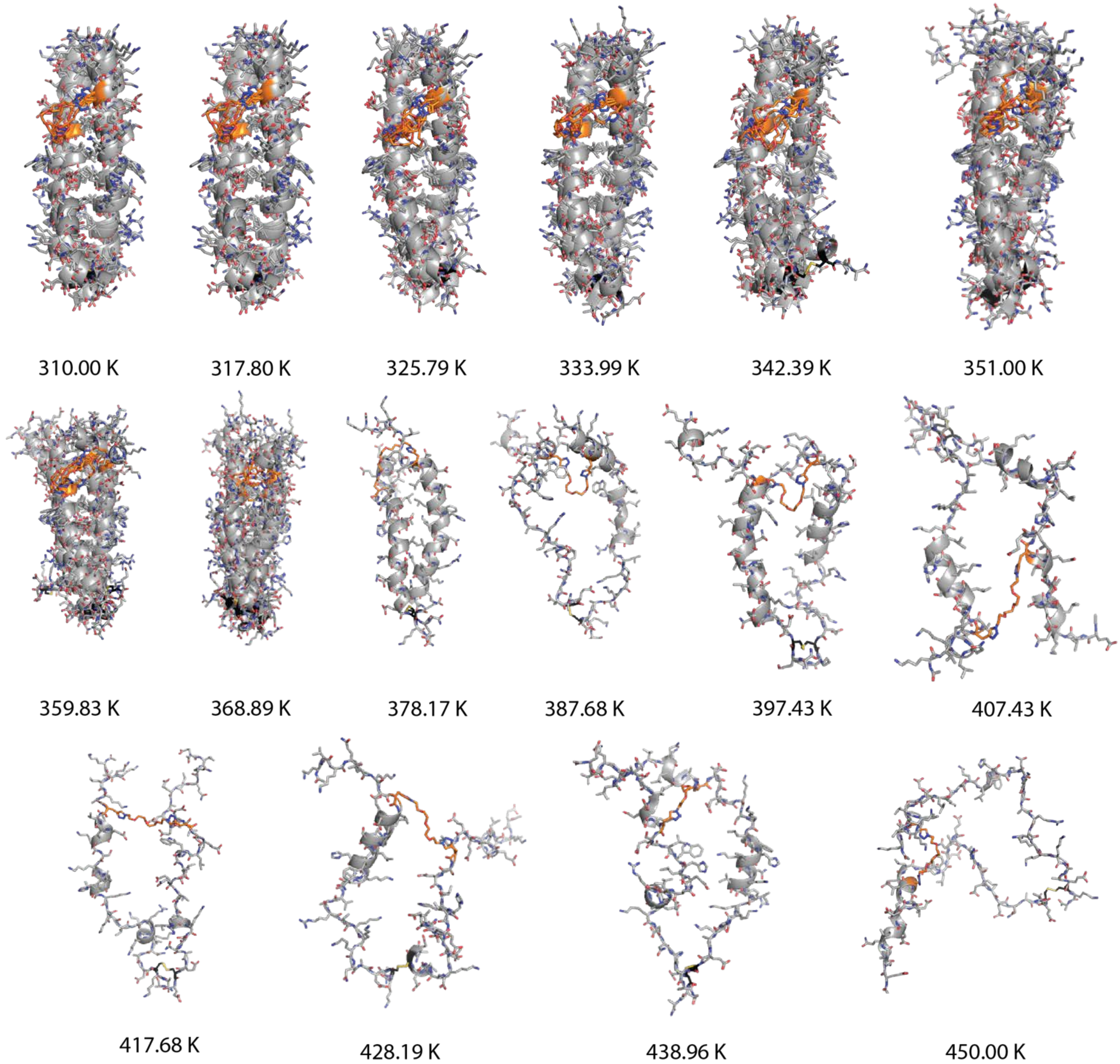

Figure S113. Ribbon diagrams of median-energy frames extracted from 2000-ns T-REMD simulations of stapled coiled-coil variant **s13e/8g'-x4x** at 310.00, 317.80, 325.79, 333.99, 342.39, 351.00, 359.83, 368.89, 378.17, 387.68, 397.43, 407.43, 417.68, 428.19, 438.96, and 450.00 K. Side chains are shown as sticks, with nitrogens and oxygens colored blue and red, respectively, and carbons colored gray, except for residues involved in the staple (where carbons are orange) or in the Cys33-Cys33' disulfide (where carbons are black). Structures for 310.00, 317.80, 325.79, 333.99, 342.39 K, 351.00, 359.83, and 368.89 show the five median-energy frames overlaid. At higher temperatures, the coiled coil becomes less folded, such that the five median-energy frames diverge considerably in structure. Therefore, for clarity, only the single median-energy frame is shown at 378.17, 387.68, 397.43, 407.43, 417.68, 428.19, 438.96, and 450.00 K.

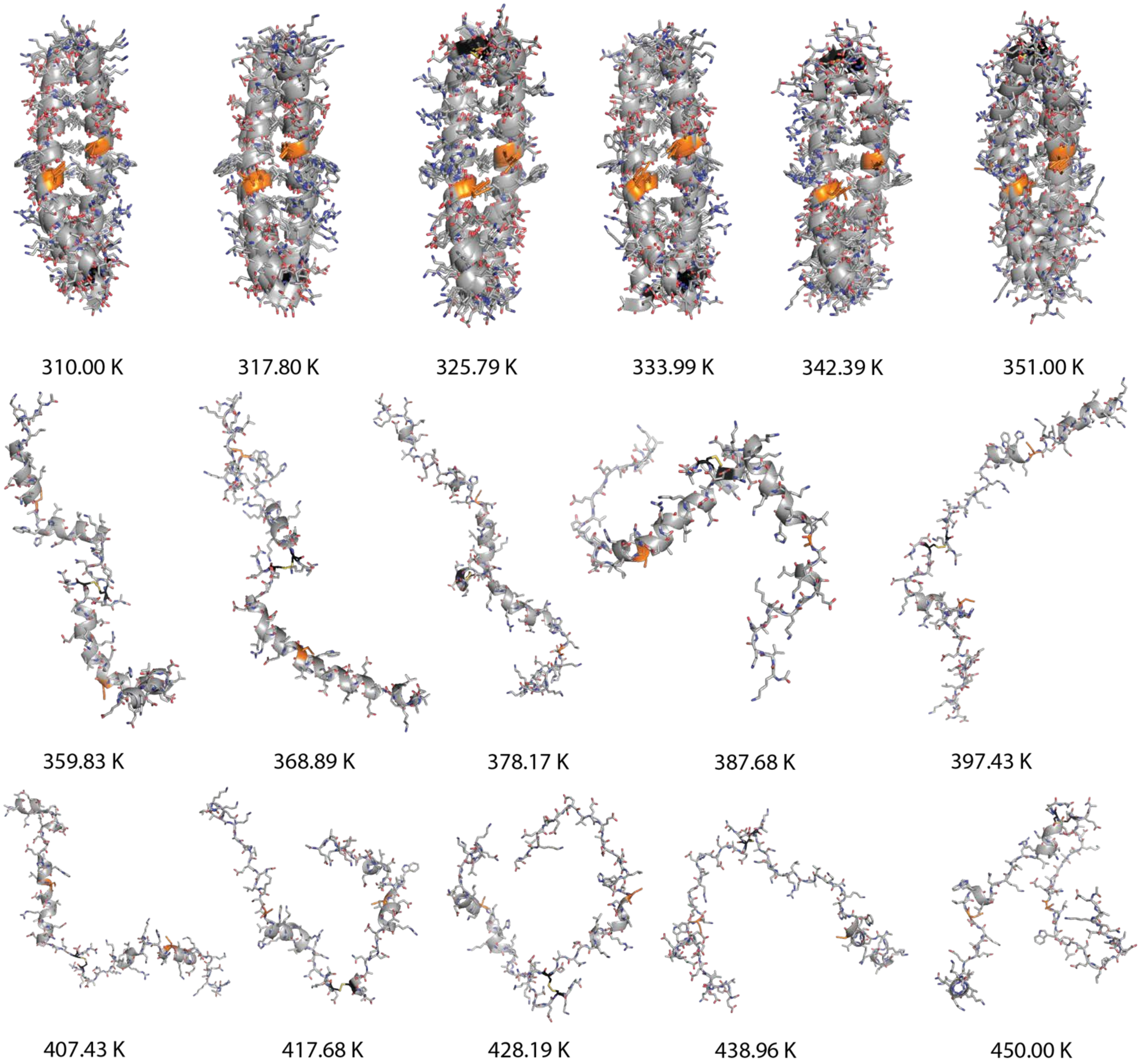

Figure S114. Ribbon diagrams of median-energy frames extracted from 2000-ns T-REMD simulations of non-stapled coiled-coil variant **20e/15g'-xx** at 310.00, 317.80, 325.79, 333.99, 342.39, 351.00, 359.83, 368.89, 378.17, 387.68, 397.43, 407.43, 417.68, 428.19, 438.96, and 450.00 K. Side chains are shown as sticks, with nitrogens and oxygens colored blue and red, respectively, and carbons colored gray, except for residues involved in the staple (where carbons are orange) or in the Cys33-Cys33' disulfide (where carbons are black). Structures for 310.00, 317.80, 325.79, 333.99, 342.39 K, and 351.00 show the five median-energy frames overlaid. At higher temperatures, the coiled coil becomes less folded, such that the five median-energy frames diverge considerably in structure. Therefore, for clarity, only the single median-energy frame is shown at 359.83, 368.89, 378.17, 387.68, 397.43, 407.43, 417.68, 428.19, 438.96, and 450.00 K.

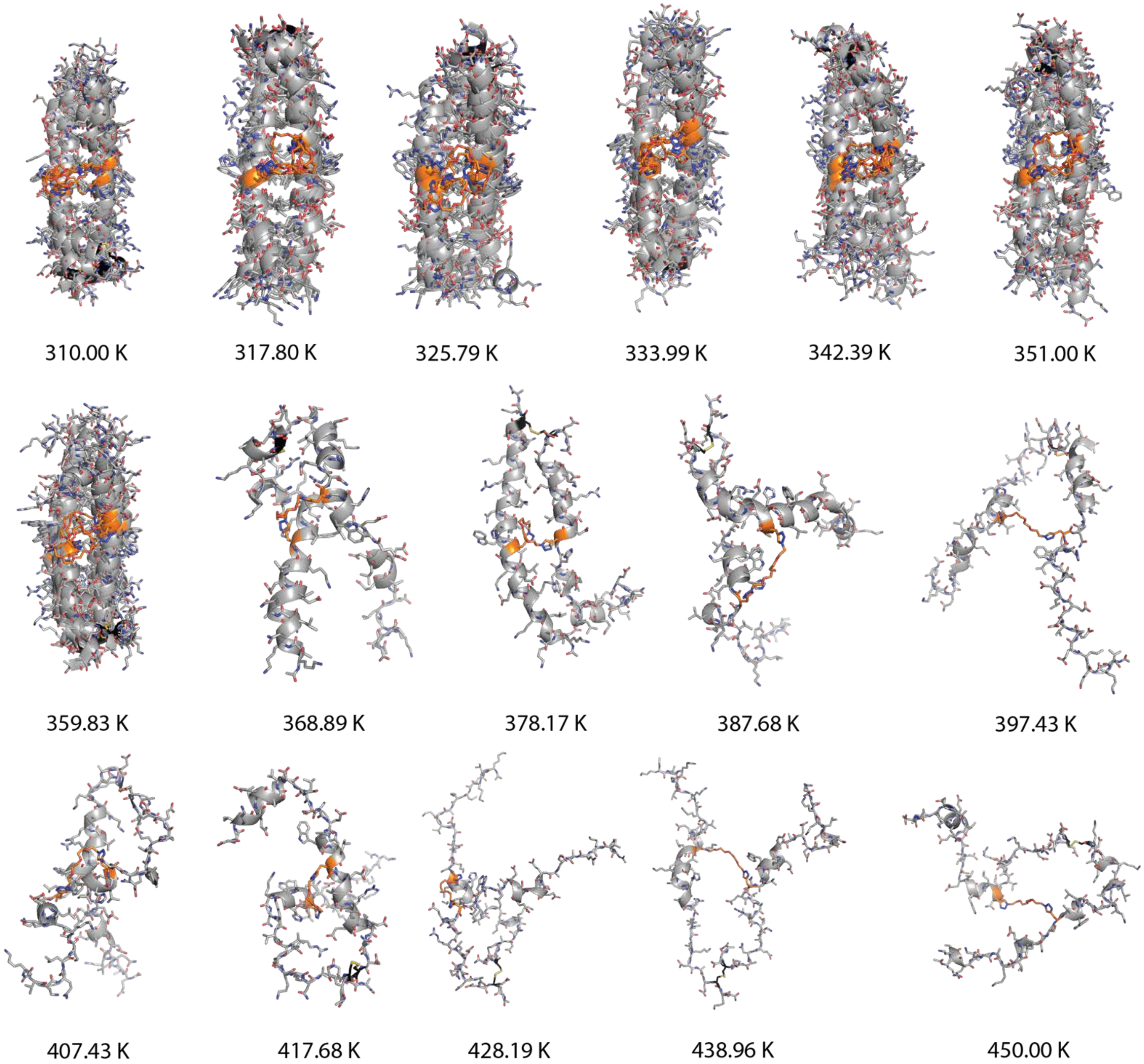

Figure S115. Ribbon diagrams of median-energy frames extracted from 2000-ns T-REMD simulations of stapled coiled-coil variant **s20e/15g'-x4x** at 310.00, 317.80, 325.79, 333.99, 342.39, 351.00, 359.83, 368.89, 378.17, 387.68, 397.43, 407.43, 417.68, 428.19, 438.96, and 450.00 K. Side chains are shown as sticks, with nitrogens and oxygens colored blue and red, respectively, and carbons colored gray, except for residues involved in the staple (where carbons are orange) or in the Cys33-Cys33' disulfide (where carbons are black). Structures for 310.00, 317.80, 325.79, 333.99, 342.39 K, 351.00, and 359.83 show the five median-energy frames overlaid. At higher temperatures, the coiled coil becomes less folded, such that the five median-energy frames diverge considerably in structure. Therefore, for clarity, only the single median-energy frame is shown at 368.89, 378.17, 387.68, 397.43, 407.43, 417.68, 428.19, 438.96, and 450.00 K.

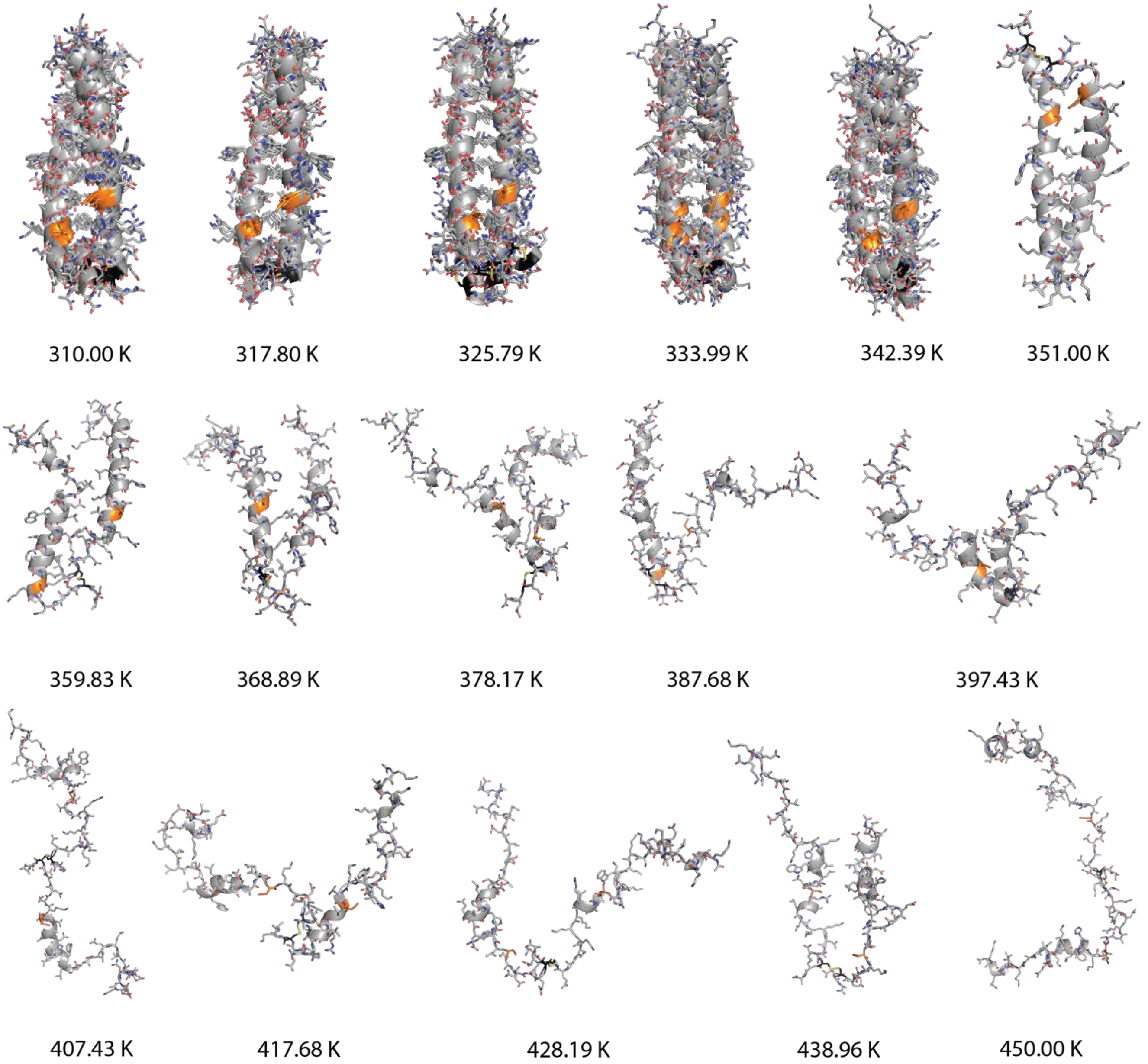

Figure S116. Ribbon diagrams of median-energy frames extracted from 2000-ns T-REMD simulations of non-stapled coiled-coil variant **27e/22g'-xx** at 310.00, 317.80, 325.79, 333.99, 342.39, 351.00, 359.83, 368.89, 378.17, 387.68, 397.43, 407.43, 417.68, 428.19, 438.96, and 450.00 K. Side chains are shown as sticks, with nitrogens and oxygens colored blue and red, respectively, and carbons colored gray, except for residues involved in the staple (where carbons are orange) or in the Cys33-Cys33' disulfide (where carbons are black). Structures for 310.00, 317.80, 325.79, 333.99, and 342.39 K show the five median-energy frames overlaid. At higher temperatures, the coiled coil becomes less folded, such that the five median-energy frames diverge considerably in structure. Therefore, for clarity, only the single median-energy frame is shown at 351.00, 359.83, 368.89, 378.17, 387.68, 397.43, 407.43, 417.68, 428.19, 438.96, and 450.00 K.

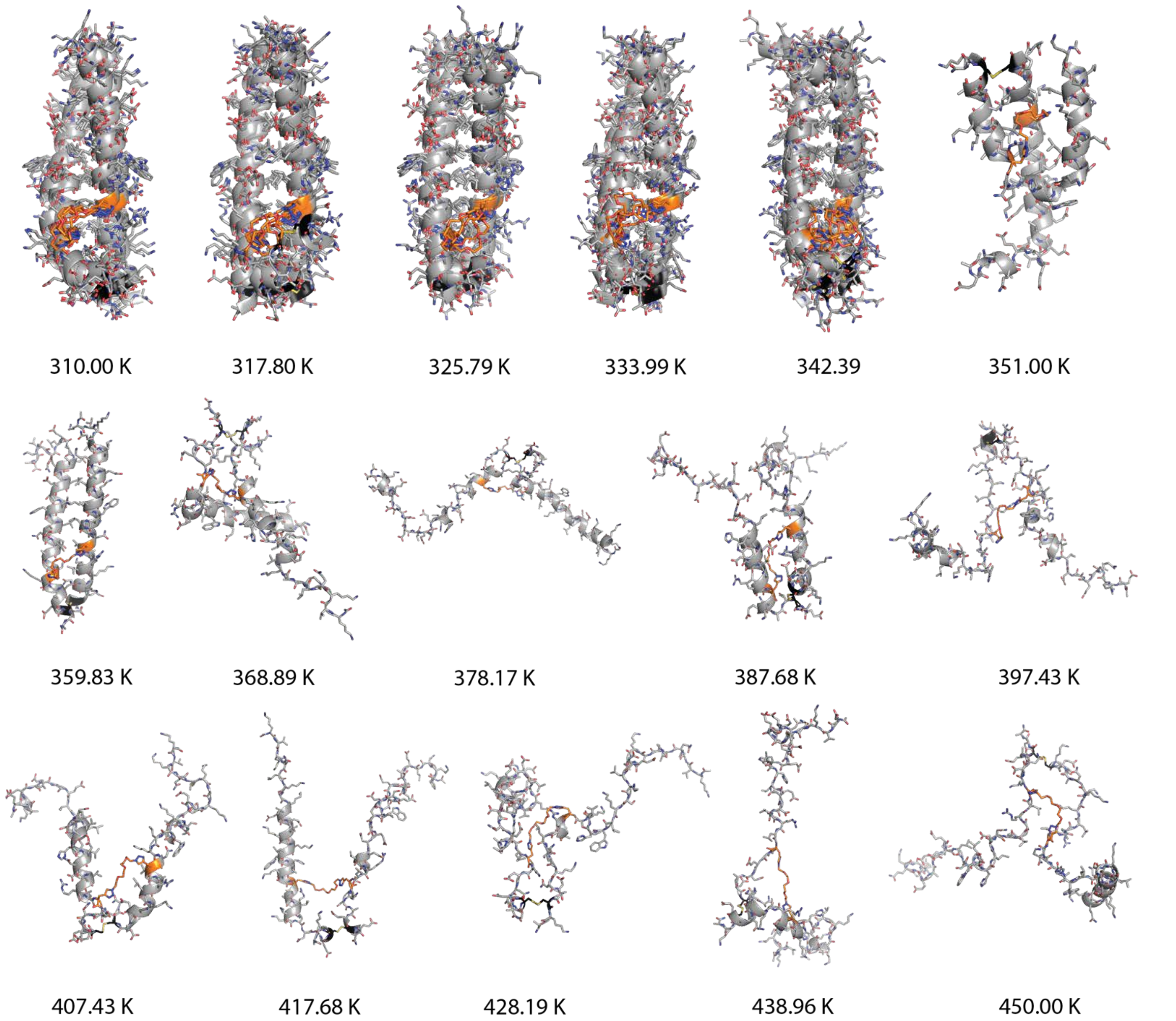

Figure S117. Ribbon diagrams of median-energy frames extracted from 2000-ns T-REMD simulations of stapled coiled-coil variant **s27e/22g'-x4x** at 310.00, 317.80, 325.79, 333.99, 342.39, 351.00, 359.83, 368.89, 378.17, 387.68, 397.43, 407.43, 417.68, 428.19, 438.96, and 450.00 K. Side chains are shown as sticks, with nitrogens and oxygens colored blue and red, respectively, and carbons colored gray, except for residues involved in the staple (where carbons are orange) or in the Cys33-Cys33' disulfide (where carbons are black). Structures for 310.00, 317.80, 325.79, 333.99, and 342.39 K show the five median-energy frames overlaid. At higher temperatures, the coiled coil becomes less folded, such that the five median-energy frames diverge considerably in structure. Therefore, for clarity, only the single median-energy frame is shown at 351.00, 359.83 K, 368.89, 378.17, 387.68, 397.43, 407.43, 417.68, 428.19, 438.96, and 450.00 K.

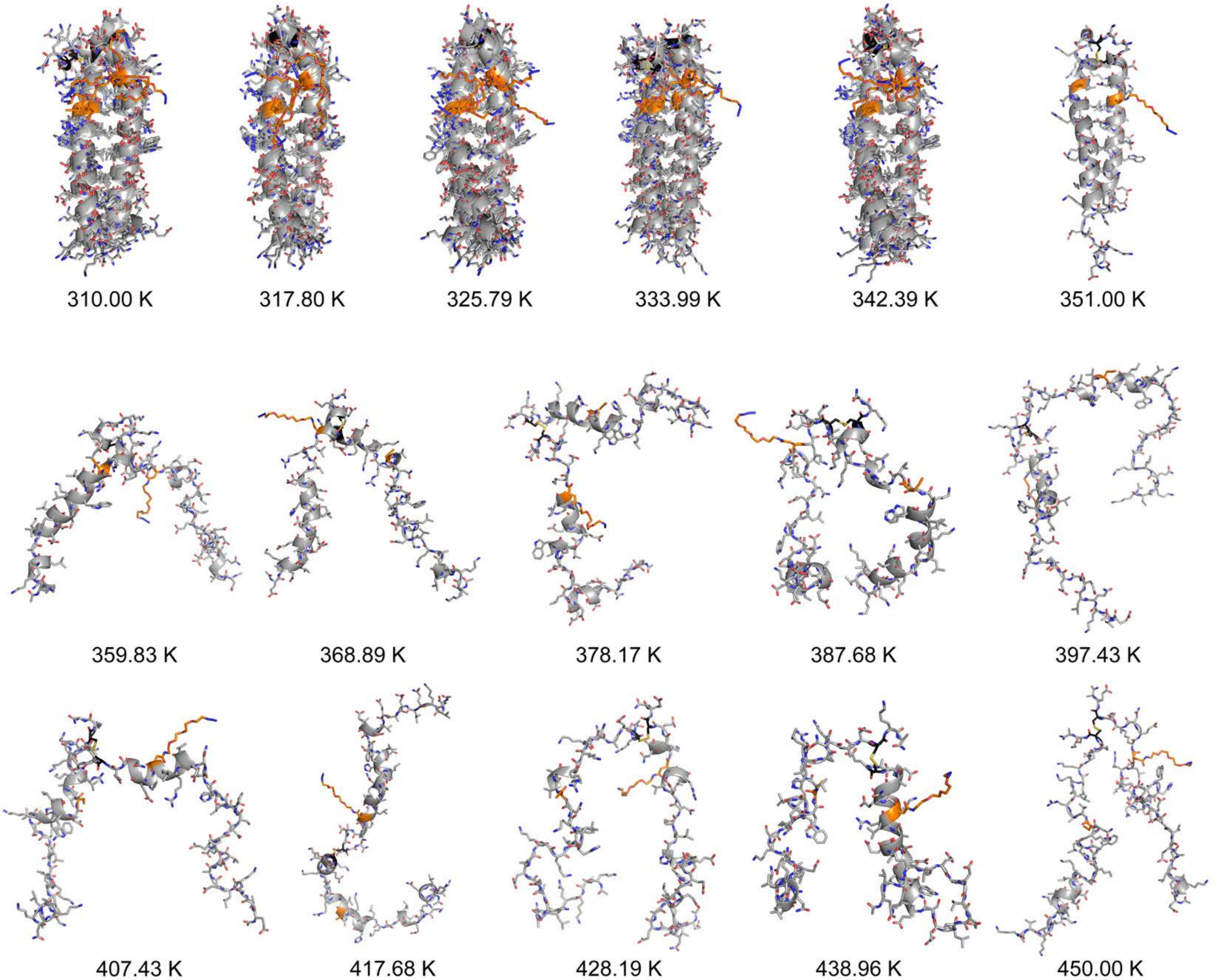

Figure S118. Ribbon diagrams of median-energy frames extracted from 2000-ns T-REMD simulations of non-stapled coiled-coil variant **27e/22g'-z4x** at 310.00, 317.80, 325.79, 333.99, 342.39, 351.00, 359.83, 368.89, 378.17, 387.68, 397.43, 407.43, 417.68, 428.19, 438.96, and 450.00 K. Side chains are shown as sticks, with nitrogens and oxygens colored blue and red, respectively, and carbons colored gray, except for residues involved in the staple (where carbons are orange) or in the Cys33-Cys33' disulfide (where carbons are black). Structures for 310.00, 317.80, 325.79, 333.99, and 342.39 K show the five median-energy frames overlaid. At higher temperatures, the coiled coil becomes less folded, such that the five median-energy frames diverge considerably in structure. Therefore, for clarity, only the single median-energy frame is shown at 351.00, 359.83, 368.89, 378.17, 387.68, 397.43, 407.43, 417.68, 428.19, 438.96, and 450.00 K.

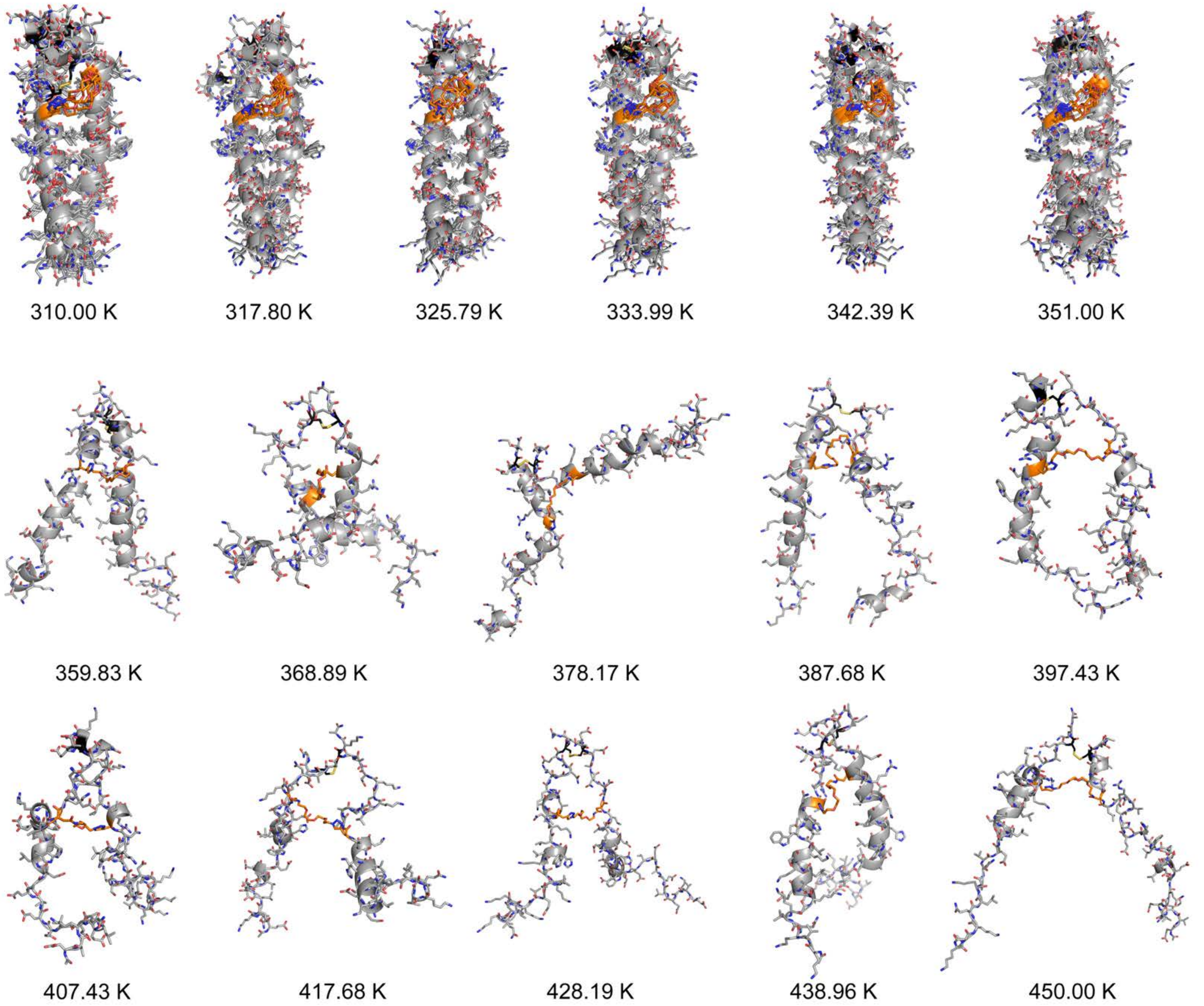

Figure S119. Ribbon diagrams of median-energy frames extracted from 2000-ns T-REMD simulations of stapled coiled-coil variant **s27e/22g’-z4x** at 310.00, 317.80, 325.79, 333.99, 342.39, 351.00, 359.83, 368.89, 378.17, 387.68, 397.43, 407.43, 417.68, 428.19, 438.96, and 450.00 K. Side chains are shown as sticks, with nitrogens and oxygens colored blue and red, respectively, and carbons colored gray, except for residues involved in the staple (where carbons are orange) or in the Cys33-Cys33’ disulfide (where carbons are black). Structures for 310.00, 317.80, 325.79, 333.99, 342.39, and 351.00 K show the five median-energy frames overlaid. At higher temperatures, the coiled coil becomes less folded, such that the five median-energy frames diverge considerably in structure. Therefore, for clarity, only the single median-energy frame is shown at 359.83, 368.89, 378.17, 387.68, 397.43, 407.43, 417.68, 428.19, 438.96, and 450.00 K.

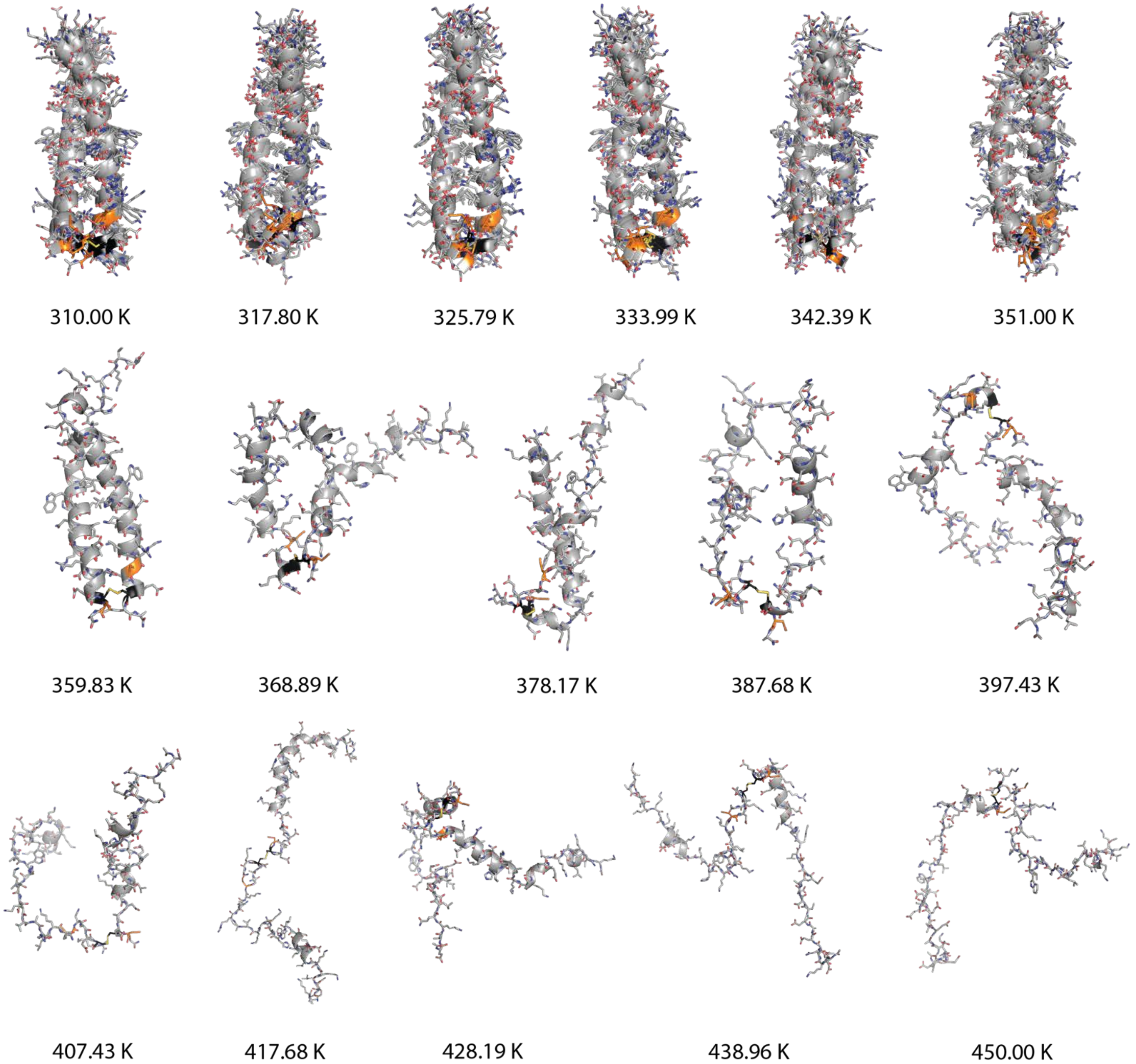

Figure S120. Ribbon diagrams of median-energy frames extracted from 2000-ns T-REMD simulations of non-stapled coiled-coil variant **34e/29g'-xx** at 310.00, 317.80, 325.79, 333.99, 342.39, 351.00, 359.83, 368.89, 378.17, 387.68, 397.43, 407.43, 417.68, 428.19, 438.96, and 450.00 K. Side chains are shown as sticks, with nitrogens and oxygens colored blue and red, respectively, and carbons colored gray, except for residues involved in the staple (where carbons are orange) or in the Cys33-Cys33' disulfide (where carbons are black). Structures for 310.00, 317.80, 325.79, 333.99, 342.39, and 351.00 K show the five median-energy frames overlaid. At higher temperatures, the coiled coil becomes less folded, such that the five median-energy frames diverge considerably in structure. Therefore, for clarity, only the single median-energy frame is shown at 359.83 K, 368.89, 378.17, 387.68, 397.43, 407.43, 417.68, 428.19, 438.96, and 450.00 K.

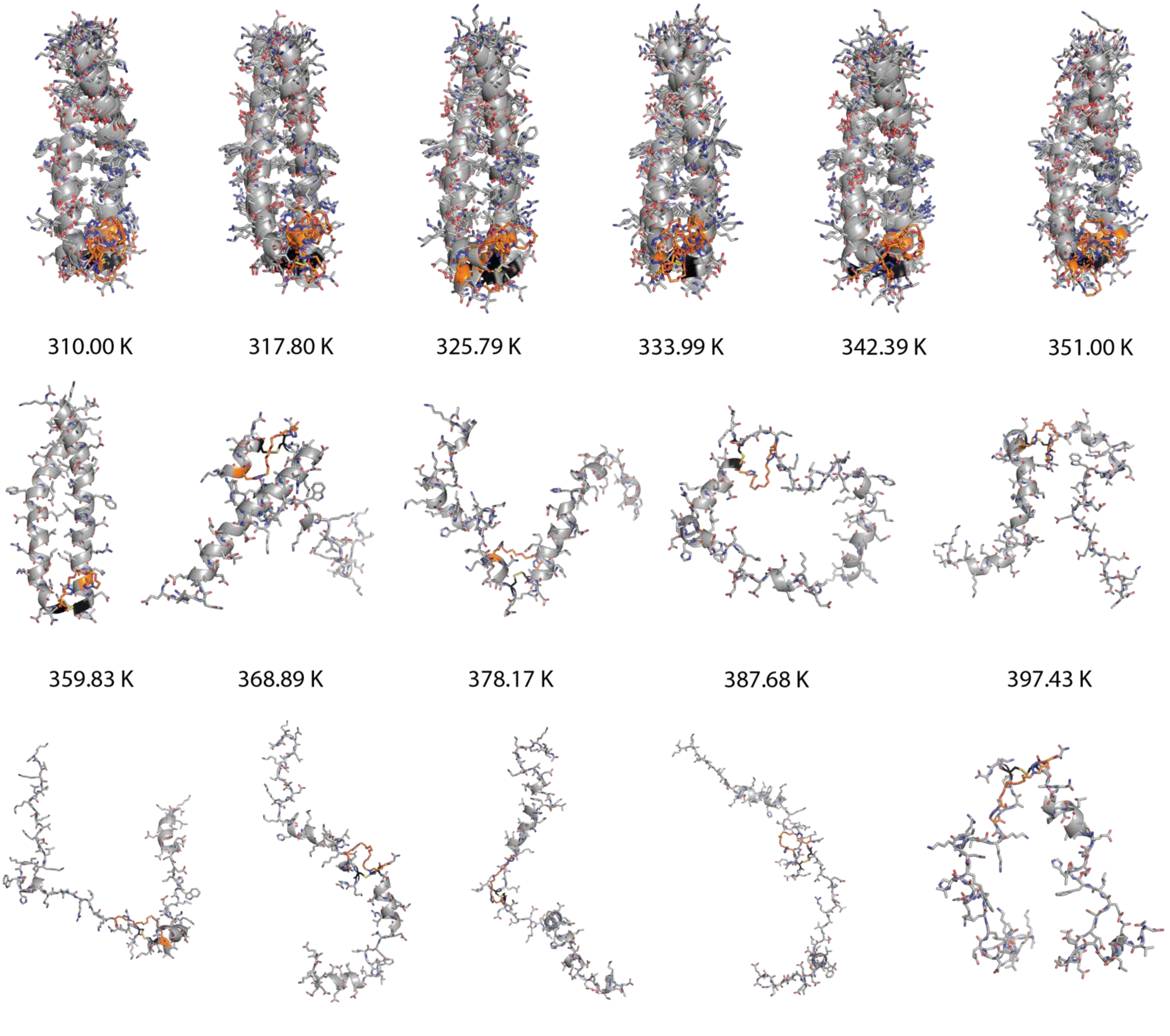

310.00 K 317.80 K 325.79 K 333.99 K 342.39 K 351.00 K

359.83 K 368.89 K 378.17 K 387.68 K 397.43 K

407.43 K 417.68 K 428.19 K 438.96 K 450.00 K

Figure S121. Ribbon diagrams of median-energy frames extracted from 2000-ns T-REMD simulations of stapled coiled-coil variant **s34e/29g'-x4x** at 310.00, 317.80, 325.79, 333.99, 342.39, 351.00, 359.83, 368.89, 378.17, 387.68, 397.43, 407.43, 417.68, 428.19, 438.96, and 450.00 K. Side chains are shown as sticks, with nitrogens and oxygens colored blue and red, respectively, and carbons colored gray, except for residues involved in the staple (where carbons are orange) or in the Cys33-Cys33' disulfide (where carbons are black). Structures for 310.00, 317.80, 325.79, 333.99, 342.39, and 351.00 K show the five median-energy frames overlaid. At higher temperatures, the coiled coil becomes less folded, such that the five median-energy frames diverge considerably in structure. Therefore, for clarity, only the single median-energy frame is shown at 359.83 K, 368.89, 378.17, 387.68, 397.43, 407.43, 417.68, 428.19, 438.96, and 450.00 K.

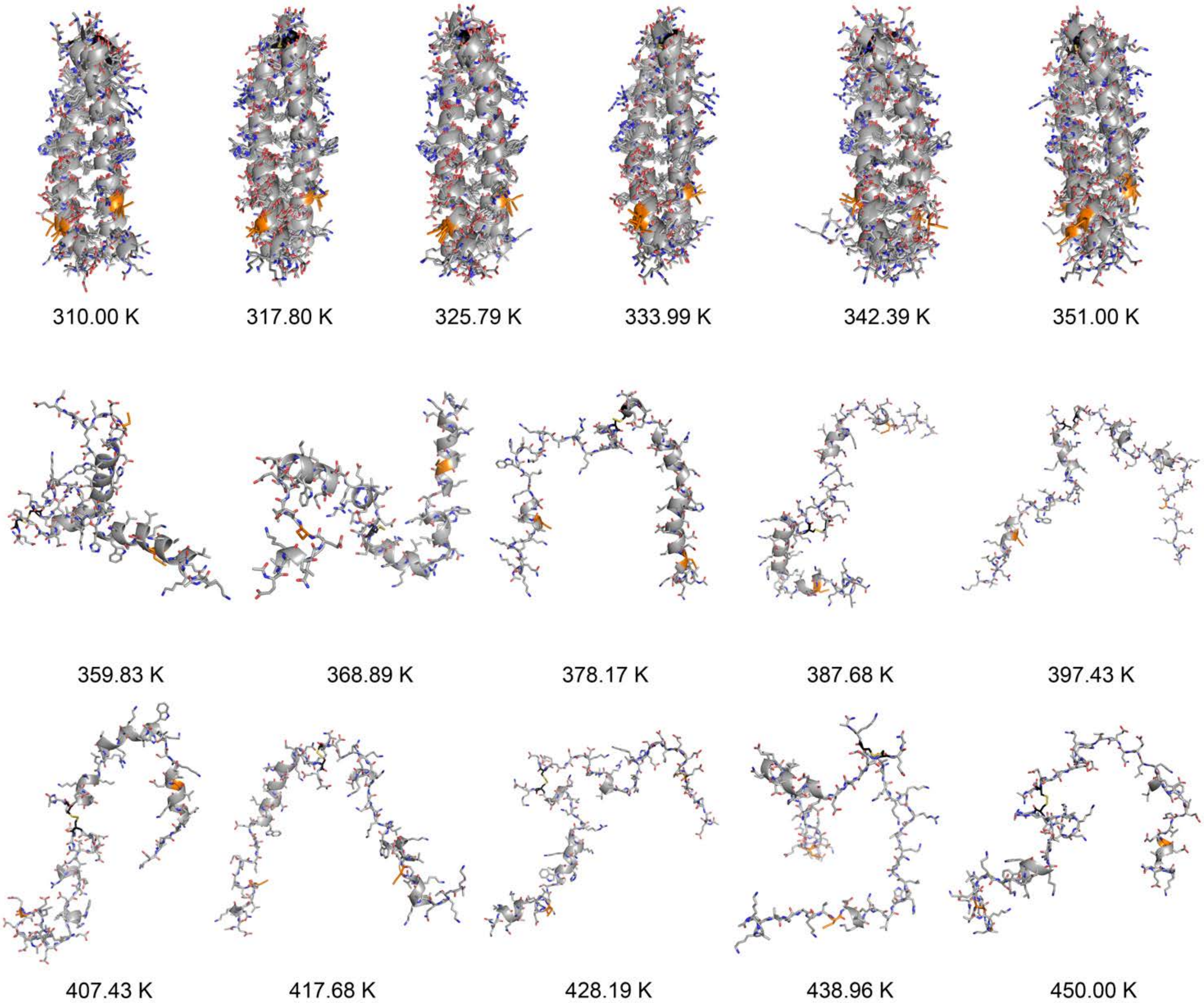

Figure S122. Ribbon diagrams of median-energy frames extracted from 2000-ns T-REMD simulations of non-stapled coiled-coil variant **7f/10b'-xx** at 310.00, 317.80, 325.79, 333.99, 342.39, 351.00, 359.83, 368.89, 378.17, 387.68, 397.43, 407.43, 417.68, 428.19, 438.96, and 450.00 K. Side chains are shown as sticks, with nitrogens and oxygens colored blue and red, respectively, and carbons colored gray, except for residues involved in the staple (where carbons are orange) or in the Cys33-Cys33' disulfide (where carbons are black). Structures for 310.00, 317.80, 325.79, 333.99, 342.39, and 351.00 K show the five median-energy frames overlaid. At higher temperatures, the coiled coil becomes less folded, such that the five median-energy frames diverge considerably in structure. Therefore, for clarity, only the single median-energy frame is shown at 359.83 K, 368.89, 378.17, 387.68, 397.43, 407.43, 417.68, 428.19, 438.96, and 450.00 K.

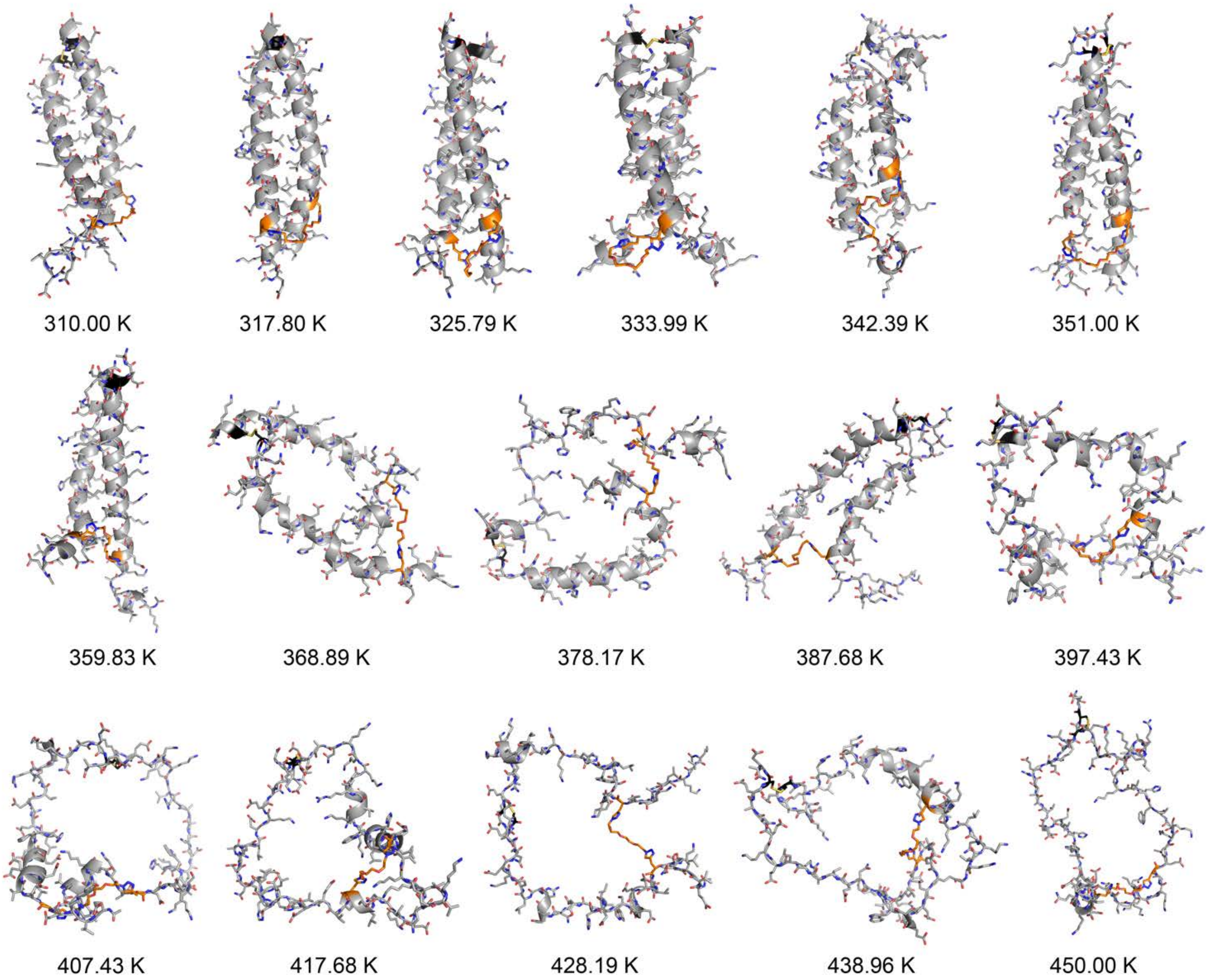

Figure S123. Ribbon diagrams of median-energy frames extracted from 2000-ns T-REMD simulations of stapled coiled-coil variant **s7f/10b'-x4x** at 310.00, 317.80, 325.79, 333.99, 342.39, 351.00, 359.83, 368.89, 378.17, 387.68, 397.43, 407.43, 417.68, 428.19, 438.96, and 450.00 K. Side chains are shown as sticks, with nitrogens and oxygens colored blue and red, respectively, and carbons colored gray, except for residues involved in the staple (where carbons are orange) or in the Cys33-Cys33' disulfide (where carbons are black).

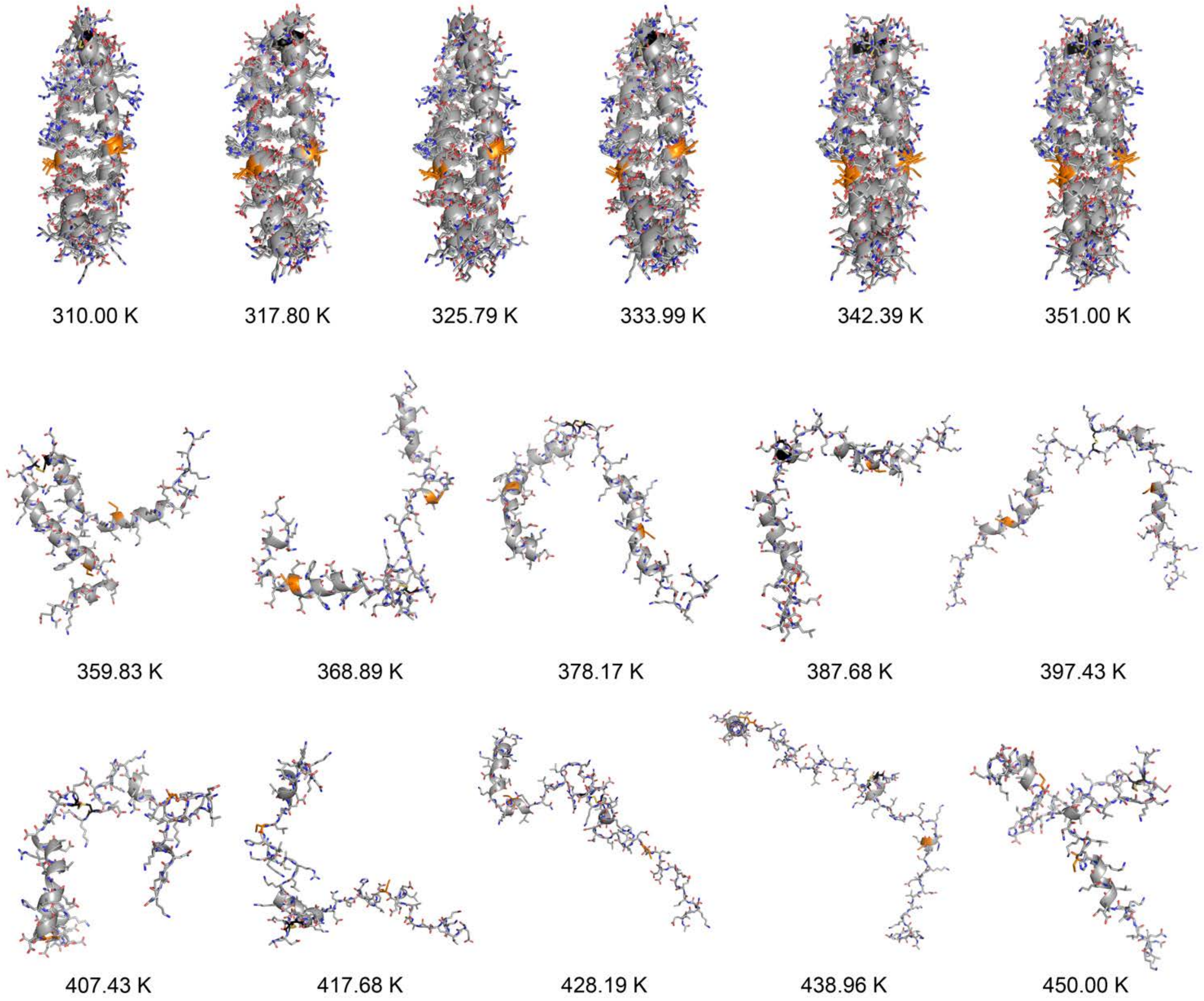

Figure S124. Ribbon diagrams of median-energy frames extracted from 2000-ns T-REMD simulations of non-stapled coiled-coil variant **14f/17b'-xx** at 310.00, 317.80, 325.79, 333.99, 342.39, 351.00, 359.83, 368.89, 378.17, 387.68, 397.43, 407.43, 417.68, 428.19, 438.96, and 450.00 K. Side chains are shown as sticks, with nitrogens and oxygens colored blue and red, respectively, and carbons colored gray, except for residues involved in the staple (where carbons are orange) or in the Cys33-Cys33' disulfide (where carbons are black). Structures for 310.00, 317.80, 325.79, 333.99, 342.39, and 351.00 K show the five median-energy frames overlaid. At higher temperatures, the coiled coil becomes less folded, such that the five median-energy frames diverge considerably in structure. Therefore, for clarity, only the single median-energy frame is shown at 359.83 K, 368.89, 378.17, 387.68, 397.43, 407.43, 417.68, 428.19, 438.96, and 450.00 K.

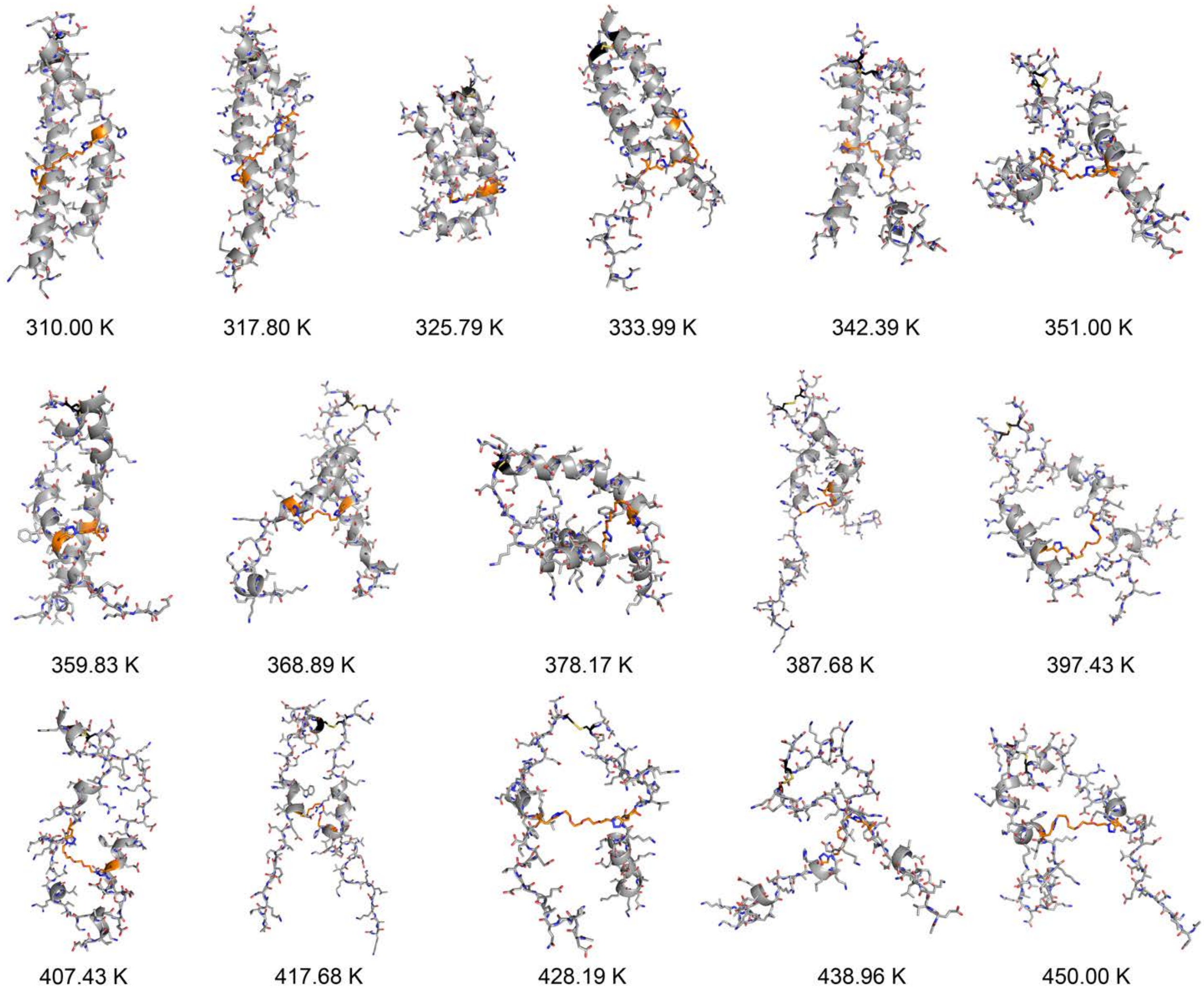

Figure S125. Ribbon diagrams of median-energy frames extracted from 2000-ns T-REMD simulations of stapled coiled-coil variant **s14f/17b'-x4x** at 310.00, 317.80, 325.79, 333.99, 342.39, 351.00, 359.83, 368.89, 378.17, 387.68, 397.43, 407.43, 417.68, 428.19, 438.96, and 450.00 K. Side chains are shown as sticks, with nitrogens and oxygens colored blue and red, respectively, and carbons colored gray, except for residues involved in the staple (where carbons are orange) or in the Cys33-Cys33' disulfide (where carbons are black).

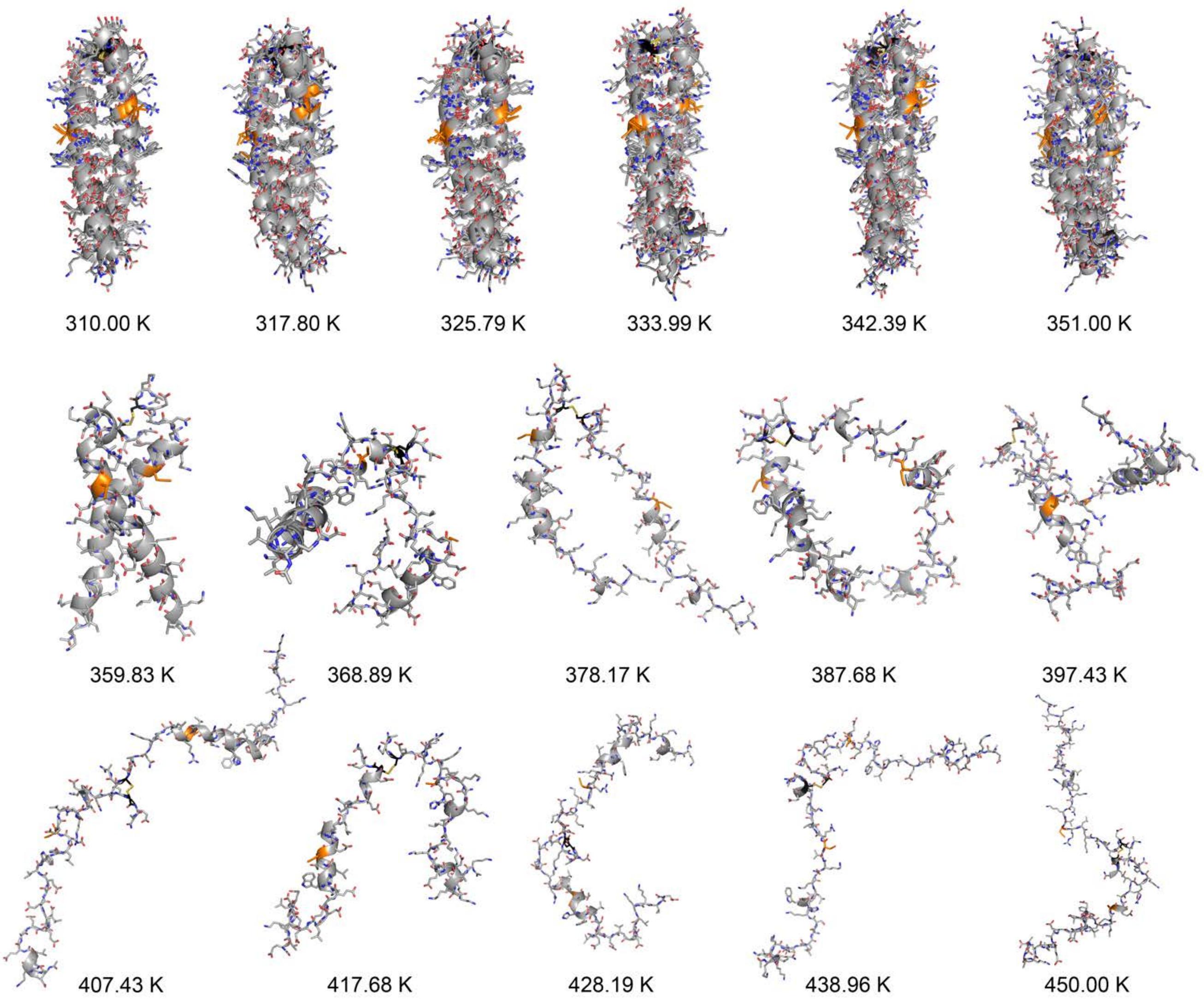

Figure S126. Ribbon diagrams of median-energy frames extracted from 2000-ns T-REMD simulations of non-stapled coiled-coil variant **21f/24b'-xx** at 310.00, 317.80, 325.79, 333.99, 342.39, 351.00, 359.83, 368.89, 378.17, 387.68, 397.43, 407.43, 417.68, 428.19, 438.96, and 450.00 K. Side chains are shown as sticks, with nitrogens and oxygens colored blue and red, respectively, and carbons colored gray, except for residues involved in the staple (where carbons are orange) or in the Cys33-Cys33' disulfide (where carbons are black). Structures for 310.00, 317.80, 325.79, 333.99, 342.39, and 351.00 K show the five median-energy frames overlaid. At higher temperatures, the coiled coil becomes less folded, such that the five median-energy frames diverge considerably in structure. Therefore, for clarity, only the single median-energy frame is shown at 359.83 K, 368.89, 378.17, 387.68, 397.43, 407.43, 417.68, 428.19, 438.96, and 450.00 K.

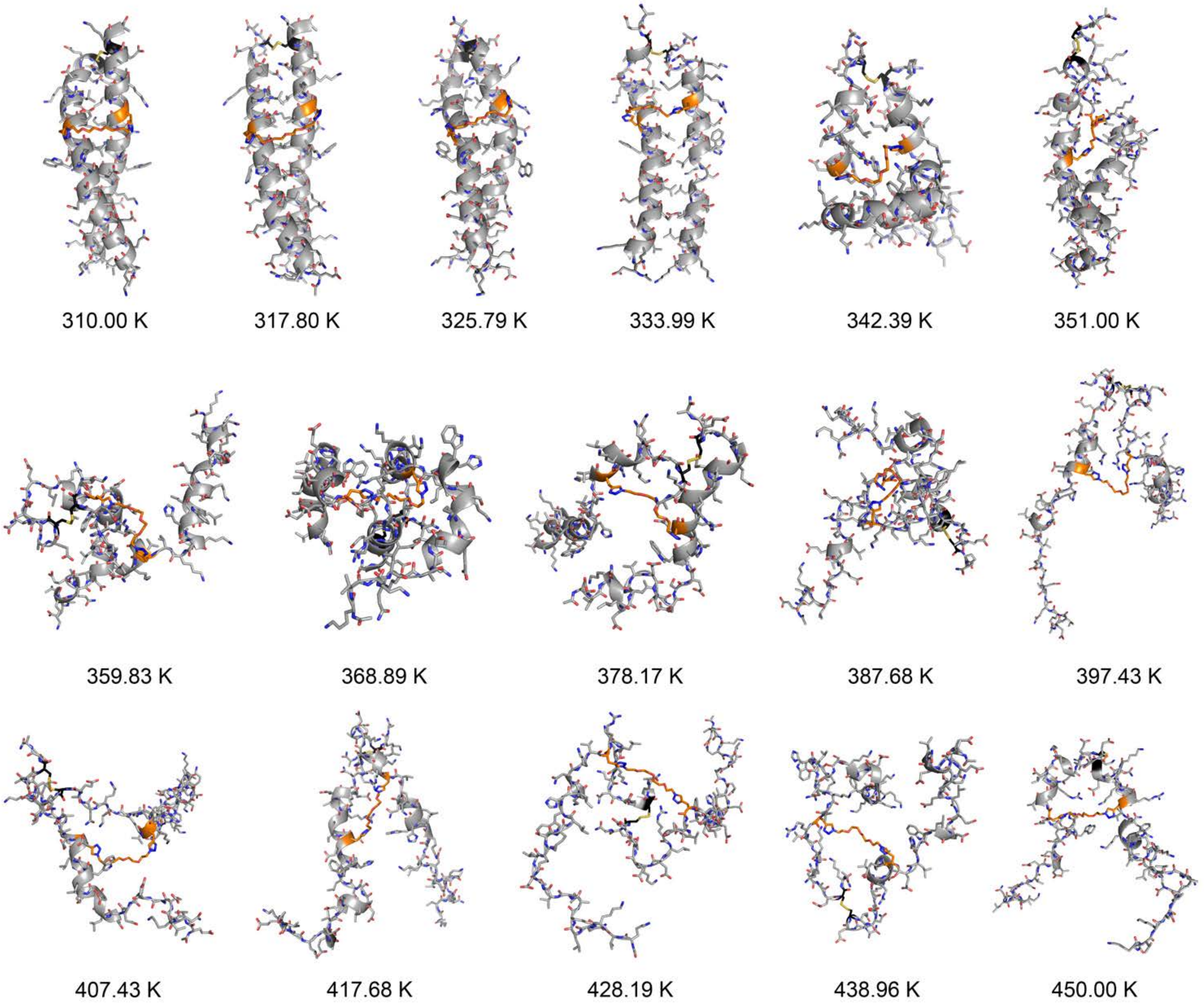

Figure S127. Ribbon diagrams of median-energy frames extracted from 2000-ns T-REMD simulations of stapled coiled-coil variant **s21f/24b'-x4x** at 310.00, 317.80, 325.79, 333.99, 342.39, 351.00, 359.83, 368.89, 378.17, 387.68, 397.43, 407.43, 417.68, 428.19, 438.96, and 450.00 K. Side chains are shown as sticks, with nitrogens and oxygens colored blue and red, respectively, and carbons colored gray, except for residues involved in the staple (where carbons are orange) or in the Cys33-Cys33' disulfide (where carbons are black).

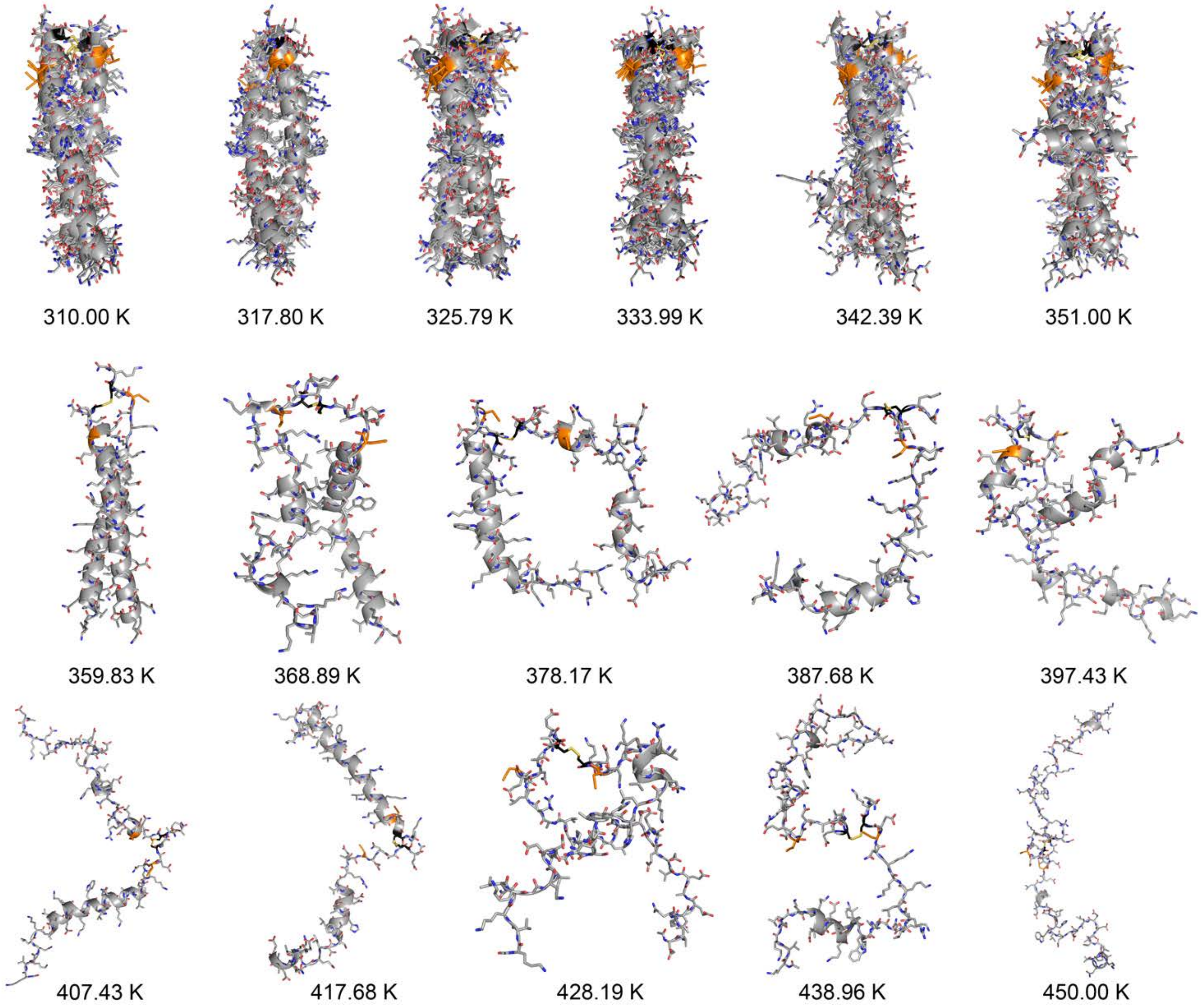

Figure S128. Ribbon diagrams of median-energy frames extracted from 2000-ns T-REMD simulations of non-stapled coiled-coil variant **28f/31b’-xx** at 310.00, 317.80, 325.79, 333.99, 342.39, 351.00, 359.83, 368.89, 378.17, 387.68, 397.43, 407.43, 417.68, 428.19, 438.96, and 450.00 K. Side chains are shown as sticks, with nitrogens and oxygens colored blue and red, respectively, and carbons colored gray, except for residues involved in the staple (where carbons are orange) or in the Cys33-Cys33’ disulfide (where carbons are black). Structures for 310.00, 317.80, 325.79, 333.99, 342.39, and 351.00 K show the five median-energy frames overlaid. At higher temperatures, the coiled coil becomes less folded, such that the five median-energy frames diverge considerably in structure. Therefore, for clarity, only the single median-energy frame is shown at 359.83 K, 368.89, 378.17, 387.68, 397.43, 407.43, 417.68, 428.19, 438.96, and 450.00 K.

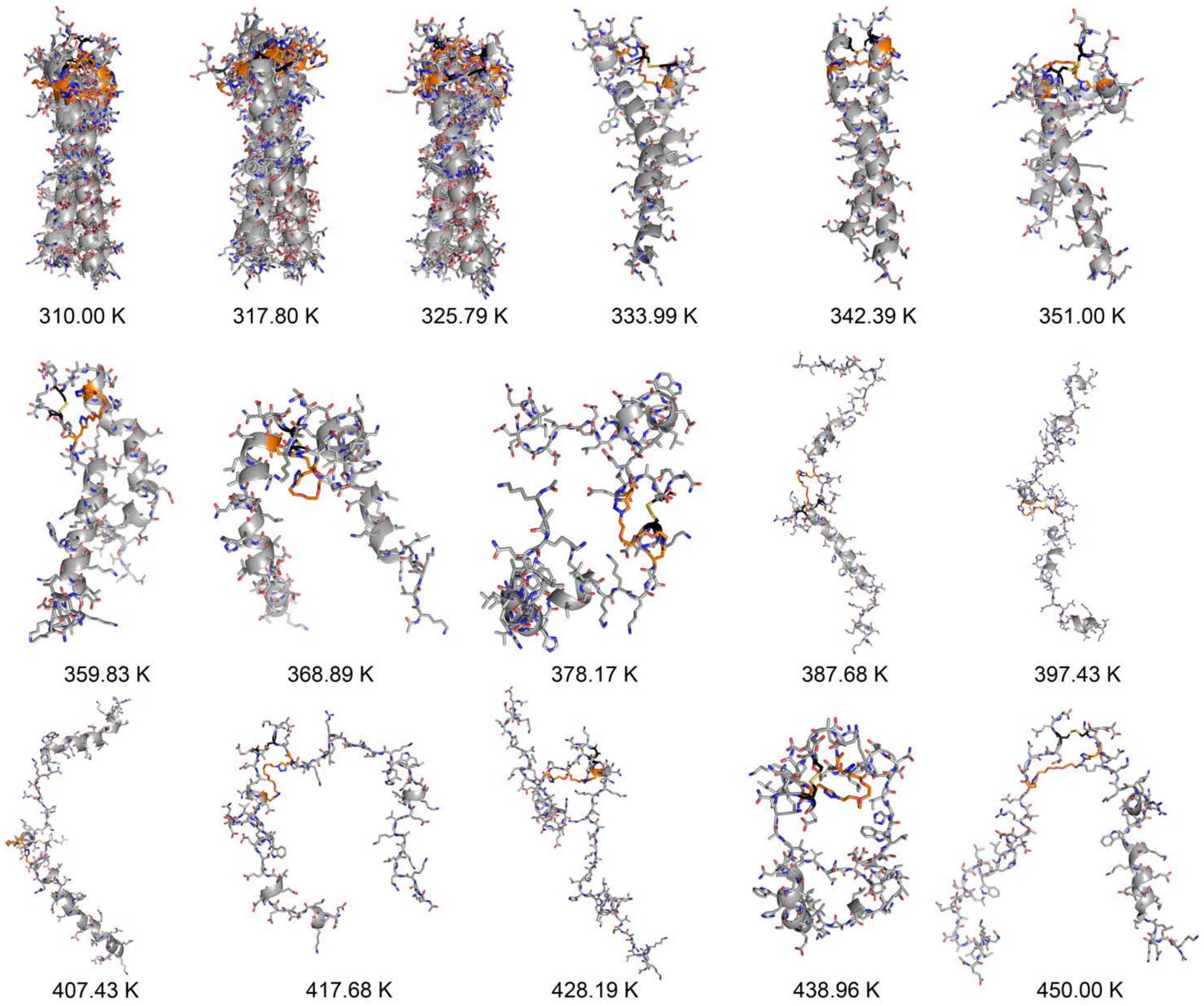

Figure S129. Ribbon diagrams of median-energy frames extracted from 2000-ns T-REMD simulations of stapled coiled-coil variant **s28f/31b'-x4x** at 310.00, 317.80, 325.79, 333.99, 342.39, 351.00, 359.83, 368.89, 378.17, 387.68, 397.43, 407.43, 417.68, 428.19, 438.96, and 450.00 K. Side chains are shown as sticks, with nitrogens and oxygens colored blue and red, respectively, and carbons colored gray, except for residues involved in the staple (where carbons are orange) or in the Cys33-Cys33' disulfide (where carbons are black). Structures for 310.00, 317.80, and 325.79 K show the five median-energy frames overlaid. At higher temperatures, the coiled coil becomes less folded, such that the five median-energy frames diverge considerably in structure. Therefore, for clarity, only the single median-energy frame is shown at 333.99, 342.39, and 351.00 359.83, 368.89, 378.17, 387.68, 397.43, 407.43, 417.68, 428.19, 438.96, and 450.00 K.

(return to Table of Contents)

10. Generating representative trajectory videos for each variant

We visualized the full 2-μs trajectory for each variant at each temperature using Pymol. However, because data were written to the trajectory files in 2 ps intervals, a complete 2-μs trajectory for a single variant at a single temperature would comprise 1 million frames; a video showing a full trajectory at each of the sixteen replica temperatures (16 million frames) would be prohibitively long. Therefore, we only visualized every 1000th frame within the final 200 ns of each of the sixteen temperature-centric trajectories for variants **s6e/1g'-x4x**, **s13e/8g'-x4x**, **s20e/15g'-x4x**, **s27e/22g'-x4x**, **s27e/22g'-z4x**, **s34e/29g'-x4x**, **s7f/10b'-x4x, s14f/17b'-x4x, s21f/24b'-x4x,** and **s28f/31b'-x4x**; for their non-stapled counterparts variants **6e/1g'-xx**, **13e/8g'-xx**, **20e/15g'-xx**, **27e/22g'-xx**, **27e/22g'-z4x**, **34e/29g'-xx**, **7f/10b'-xx**, **14f/17b'-xx**, **21f/24b'-xx**, and **28f/31b'-xx** resulting in eighteen videos, one for each variant. Each video comprises 100 frames at each of the sixteen replica temperatures (spaced 2 ns apart) for a total of 1600 frames. Playback rate is set at ~30 frames per second of play (i.e., 60 ns of simulation per playback second); videos are uploaded separately from this document in MPEG4 format. In these videos, variants are shown as stick diagrams; nitrogens are blue; oxygens are red; and carbons are gray, except for residues involved in the staple (orange) or in the Cys33-Cys33' disulfide (black). Qualitative observations from these videos are described in the main text.

(return to Table of Contents)

11. Assessing the foldedness of stapled coiled-coils variants relative to their non-stapled counterparts in the T-REMD simulations: RMSD and residue packing distances

We sought to support these qualitative observations with quantitative analysis of the coordinates recorded in the trajectory files for variants **s6e/1g'-x4x**, **s13e/8g'-x4x**, **s20e/15g'-x4x**, **s27e/22g'-x4x**, **s27e/22g'-z4x**, **s34e/29g'-x4x**, **s7f/10b'-x4x, s14f/17b'-x4x, s21f/24b'-x4x,** and **s28f/31b'-x4x**; for their non-stapled counterparts variants **6e/1g'-xx**, **13e/8g'-xx**, **20e/15g'-xx**, **27e/22g'-xx**, **27e/29g'-z4x**, **34e/29g'-xx**, **7f/10b'-xx**, **14f/17b'-xx**, **21f/24b'-xx**, and **28f/31b'-xx** at each of the sixteen replica temperatures. To facilitate reasonable output file sizes, we limited subsequent analysis to every 10th frame of these temperature-centric trajectory files. In each of the selected frames, we calculated the root-mean-square deviation (RMSD) of the backbone atoms (N, CA, C, and O) of each variant relative to the corresponding atoms in the crystal structure of the parent coiled coil **A/B** (PDB ID: 1kd9). We also calculated the packing distances between the side chains on each helix that comprise the hydrophobic core of the coiled coil. In each of the stapled and non-stapled variants described here, Val2a, Leu16a, Val23a, and Asn30a occupy the canonical heptad a-positions within helical subunit A, whereas Val2a', Leu16a', Val23a', and Asn30a' occupy the corresponding *a*-positions within helical subunit B. Similarly, Leu5d, Leu12d, Leu19d, and Leu26d occupy the *d*-positions within helical subunit A, whereas Leu5d', Leu12d', Leu19d', and Leu26d' occupy the corresponding *d*-positions within helical subunit B. In the subsequent discussion, we define residue packing distance 2a/2a' as the distance between the average positions

of the CA and CB atoms of Val2a vs. Val2a', respectively. Similarly, we define residue packing distances 9a/9a', 16a/16a', 23a/23a', 30a/30a' 5d/5d', 12d/12d', 19d/19d' and 26d/26d' as the distances between the average positions of the CA and CB atoms of Val9a vs. Val9a', Leu16a vs. Leu16a', Val23a vs. Val23a', Asn30a vs. Asn30a', Leu5d vs. Leu5d', Leu12d vs. Leu12d', Leu19d vs. Leu19d', and Leu26d vs. Leu26d', respectively.

Plots of RMSD and residue packing distances 2a/2a', 5d/5d', 9a/9a', 12d/12d', 16a/16a', 19d/19d', 23a/23a', 26d/26d', and 30a/30a' as a function of simulation step number (where each simulation step is 2 fs, or 1 billion steps within one 2000-ns trajectory) for the sixteen temperature-centric trajectories of variant **6e/1g'-x4x** are shown below in Figures S126–S141. In these plots, the gray lines represent RMSD or residue packing distances as a function of simulation step number. The orange lines represent the average RMSD or residue packing distances within a moving window of the 50 points that surround each corresponding point in the gray line. The RMSD and residue packing distances occasionally showed some systematic deviation from the average RMSD values and residue packing distances averages calculated for the entire 1-billion-step trajectory during the first 20 million steps of the simulation. Therefore, we excluded these first 20 million steps from our calculation of the average trajectory RMSD values and the individual average trajectory residue packing distances that we used to assess folding. The solid blue line shows the average RMSD or residue packing distance over the trajectory from t = 40 ns (i.e., the 20 millionth step) to t = 2 μs (i.e., the billionth step). Dotted blue lines show this trajectory average RMSD or residue packing distance ± one standard deviation. Generally, higher trajectory average RMSD values or residue packing distances correspond to less foldedness.

How do we know whether these average RMSD values or residue packing distances are stable over the course of the simulation? To answer this question, we used linear regression to calculate the slope of RMSD or residue packing distances (the gray lines in the plots) vs. time from t = 40 ns to t = 2 μs; values of these slopes (in units of Å / μs) appear below the plots in Figures S130–S145, along with the trajectory average RMSD and residue packing distances. In each case, the slope is substantially smaller than the corresponding standard deviation, suggesting that the average RMSD values or residue packing distances are not changing systematically over time outside of what would be expected given the observed standard deviation. Similar plots and analyses are shown for all other variants and their stapled components in Figures S146-S450.

6e/1g'-xx

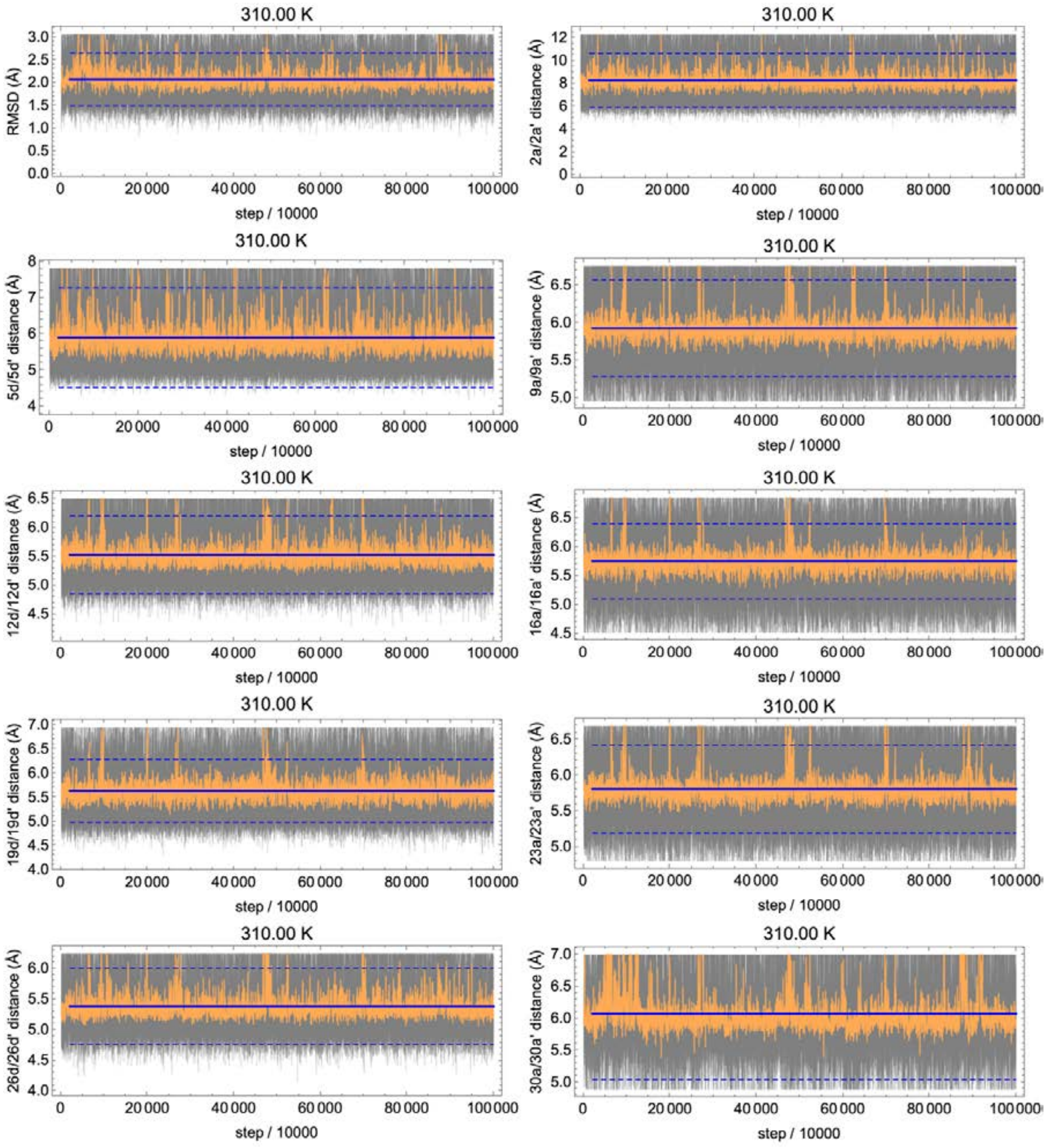

Measure at 310.00 K	Average (Å)	Standard Deviation (Å)	Slope (Å / μs)
RMSD	2.07947	0.582398	−0.0490092
5d/5d' distance	5.90152	1.38265	−0.0643797
12d/12d' distance	5.5288	0.673675	−0.00883352
19d/19d' distance	5.63851	0.6544	−0.00317344
26d/26d' distance	5.38513	0.622346	−0.0100055
2a/2a' distance	8.28662	2.37592	−0.0723512
9a/9a' distance	5.9282	0.644515	−0.0125905
16a/16a' distance	5.7496	0.650327	−0.00941555
23a/23a' distance	5.80938	0.613116	−0.0115813
30a/30a' distance	6.07911	1.03213	−0.0426018
Core Distance	6.0341	1.38141	

Figure S130. Plots of RMSD and residue packing distances for T-REMD simulations of variant **6e/1g'-xx** at 310.00 K.

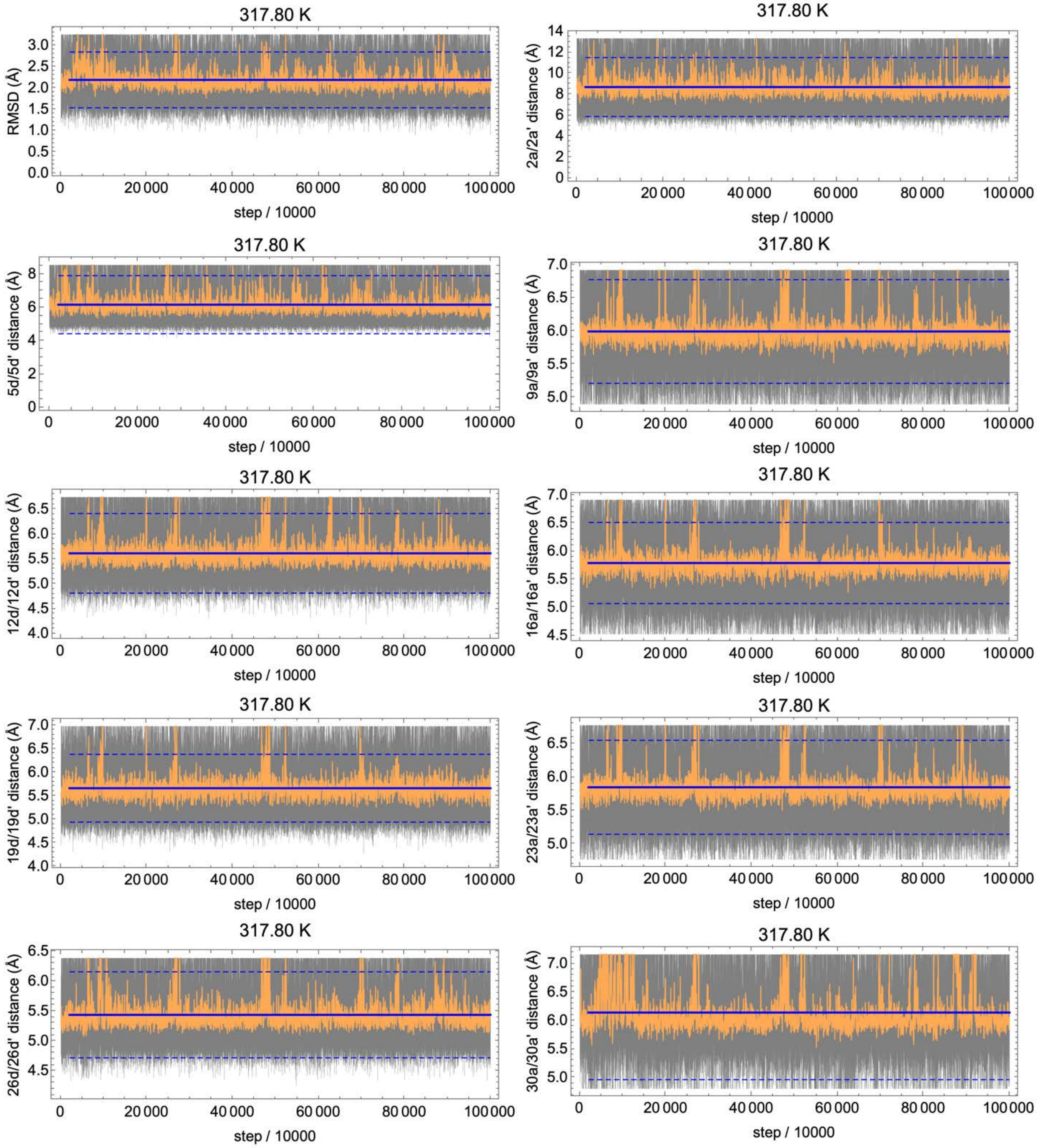

Measure at 317.80 K	Average (Å)	Standard Deviation (Å)	Slope (Å / μs)
RMSD	2.19034	0.654727	−0.0621403
5d/5d' distance	6.16258	1.74121	−0.107548
12d/12d' distance	5.61318	0.799866	−0.0213974
19d/19d' distance	5.66406	0.720091	−0.0125351
26d/26d' distance	5.43739	0.719126	−0.0101996
2a/2a' distance	8.69166	2.81153	−0.148669
9a/9a' distance	5.99499	0.78114	−0.0227787
16a/16a' distance	5.79076	0.721645	−0.0162041
23a/23a' distance	5.84686	0.701302	−0.00964005
30a/30a' distance	6.14153	1.1779	−0.0528825
Core Distance	6.14923	1.61105	

Figure S131. Plots of RMSD and residue packing distances for T-REMD simulations of variant **6e/1g'-xx** at 317.80 K.

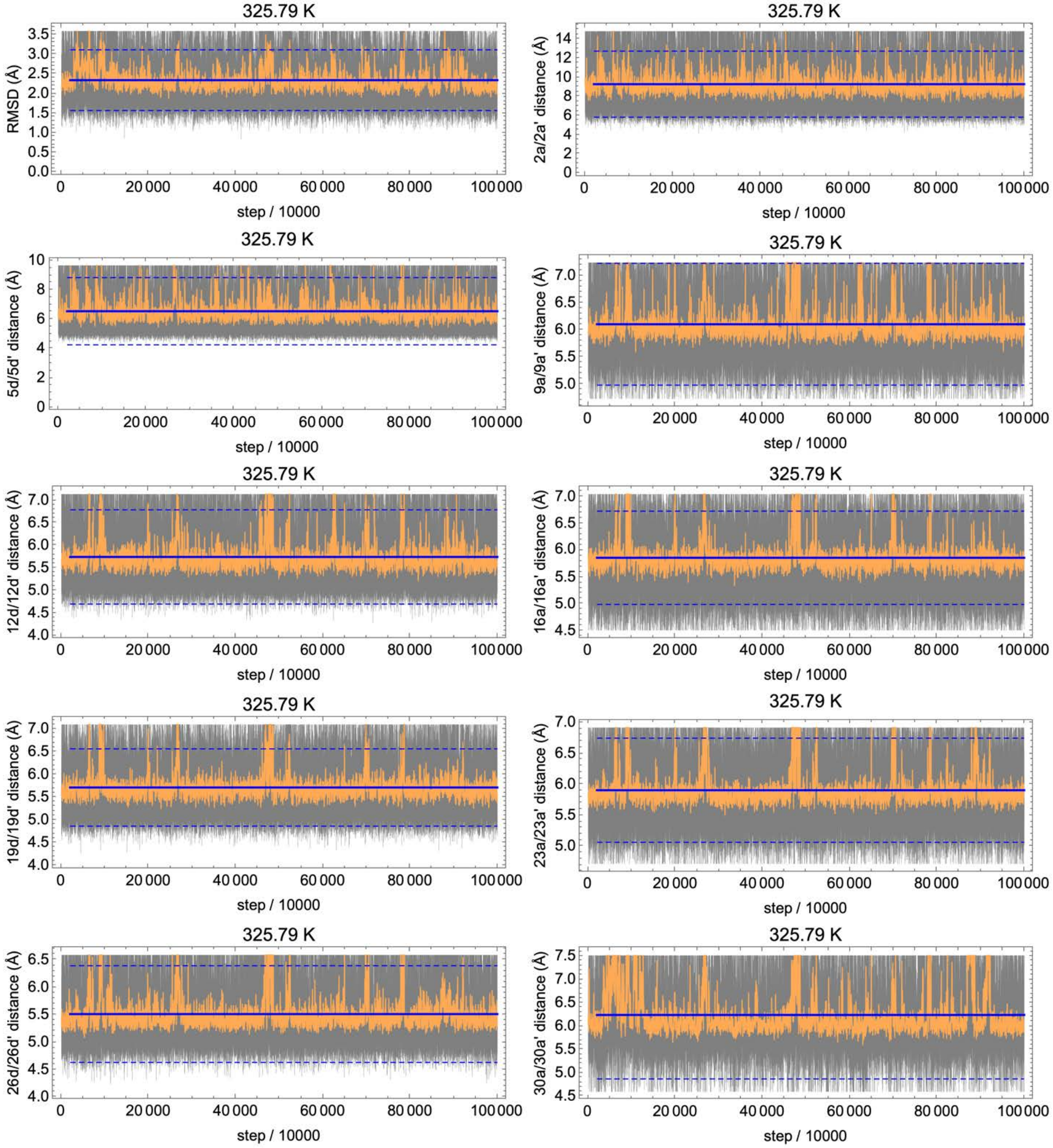

Measure at 325.79 K	Average (Å)	Standard Deviation (Å)	Slope (Å / μs)
RMSD	2.33915	0.773888	−0.0830999
5d/5d' distance	6.54369	2.29131	−0.112611
12d/12d' distance	5.74148	1.04574	−0.0222457
19d/19d' distance	5.71086	0.849353	−0.0233042
26d/26d' distance	5.51102	0.881858	−0.0197306
2a/2a' distance	9.2793	3.4498	−0.208359
9a/9a' distance	6.10337	1.11987	−0.0230312
16a/16a' distance	5.85931	0.870497	−0.0245377
23a/23a' distance	5.90451	0.840976	−0.023496
30a/30a' distance	6.23771	1.36986	−0.0874012
Core Distance	6.32125	1.97049	

Figure S132. Plots of RMSD and residue packing distances for T-REMD simulations of variant **6e/1g'-xx** at 325.79 K.

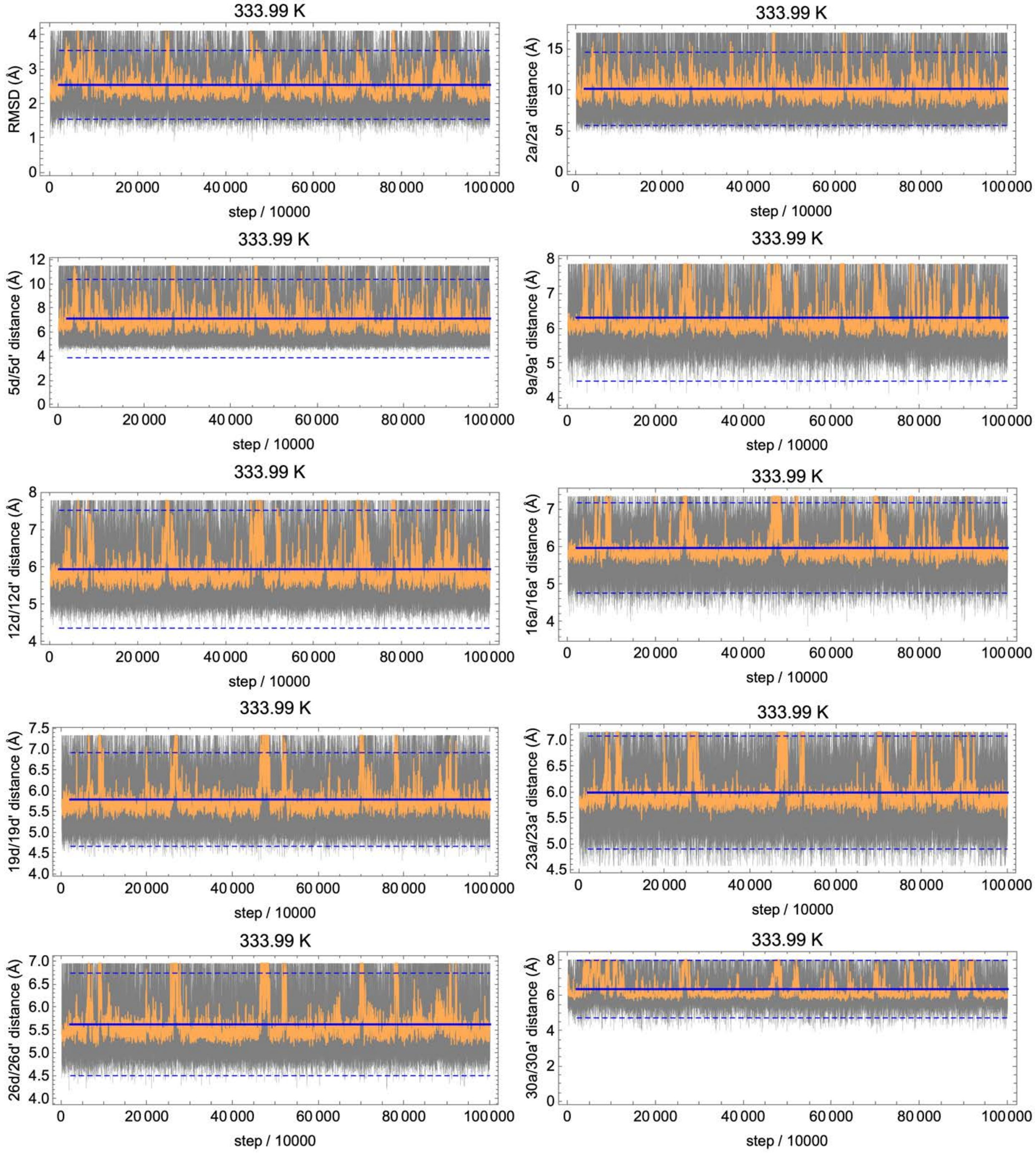

Measure at 333.99 K	Average (Å)	Standard Deviation (Å)	Slope (Å / μs)
RMSD	2.55899	1.00006	−0.0701653
5d/5d' distance	7.16685	3.24593	−0.0871093
12d/12d' distance	5.95475	1.5805	0.0143678
19d/19d' distance	5.80067	1.12698	−0.00646789
26d/26d' distance	5.6308	1.11922	−0.0229799
2a/2a' distance	10.1696	4.47311	−0.192427
9a/9a' distance	6.32121	1.82204	0.00392722
16a/16a' distance	5.97898	1.20561	−0.0094074
23a/23a' distance	5.9973	1.08998	−0.0209917
30a/30a' distance	6.38254	1.62964	−0.110806
Core Distance	6.6003	2.58444	

Figure S133. Plots of RMSD and residue packing distances for T-REMD simulations of variant **6e/1g'-xx** at 333.99 K.

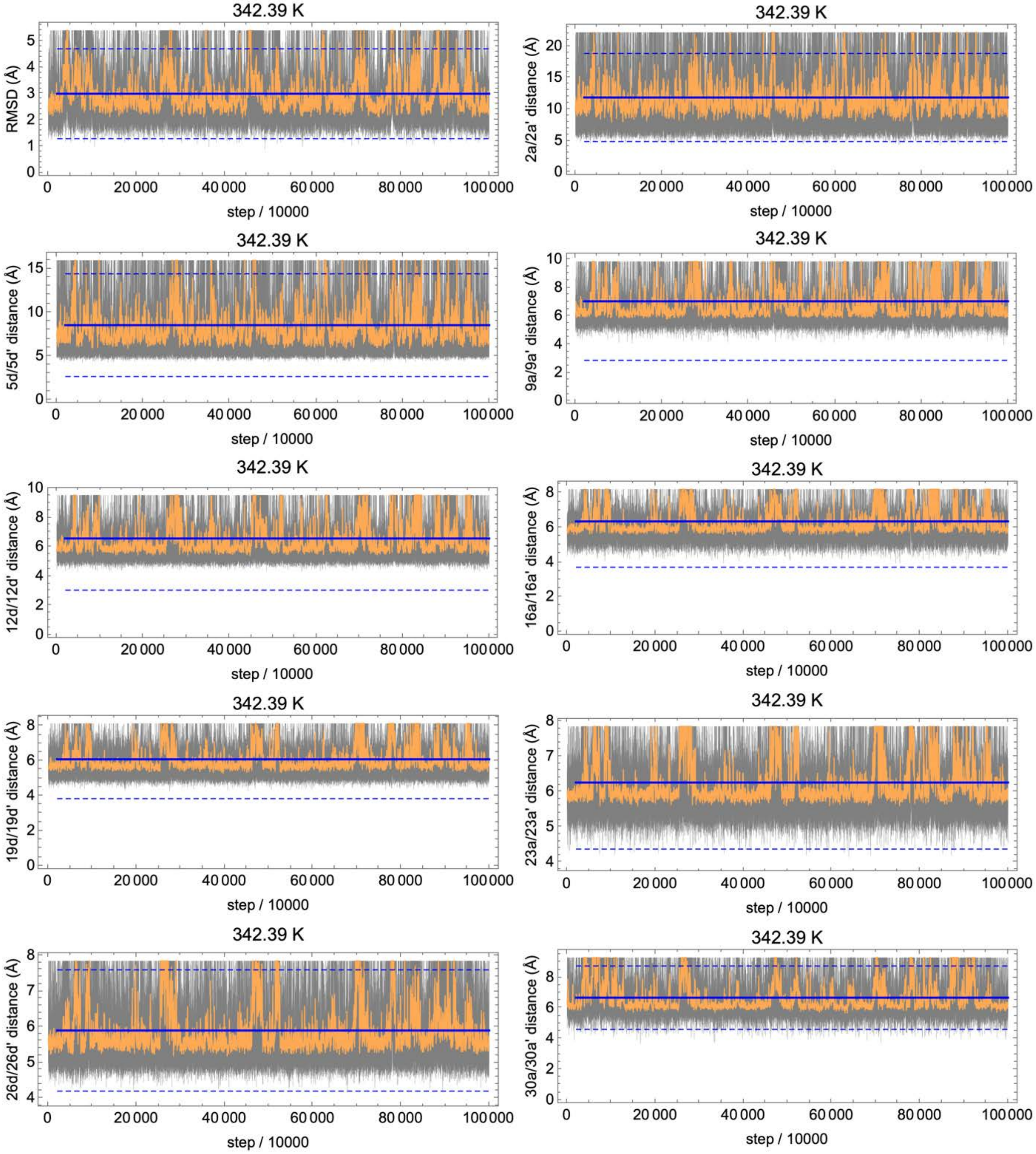

Measure at 342.39 K	Average (Å)	Standard Deviation (Å)	Slope (Å / μs)
RMSD	2.99458	1.70359	0.0491195
5d/5d' distance	8.52064	5.8627	0.326097
12d/12d' distance	6.56002	3.52203	0.295295
19d/19d' distance	6.08115	2.24878	0.145729
26d/26d' distance	5.8925	1.6998	0.0474566
2a/2a' distance	11.8387	6.99917	0.247755
9a/9a' distance	7.02895	4.15944	0.337922
16a/16a' distance	6.3418	2.63719	0.179094
23a/23a' distance	6.25732	1.8877	0.0778984
30a/30a' distance	6.67345	2.07087	−0.097827
Core Distance	7.24384	4.27348	

Figure S134. Plots of RMSD and residue packing distances for T-REMD simulations of variant **6e/1g'-xx** at 342.39 K.

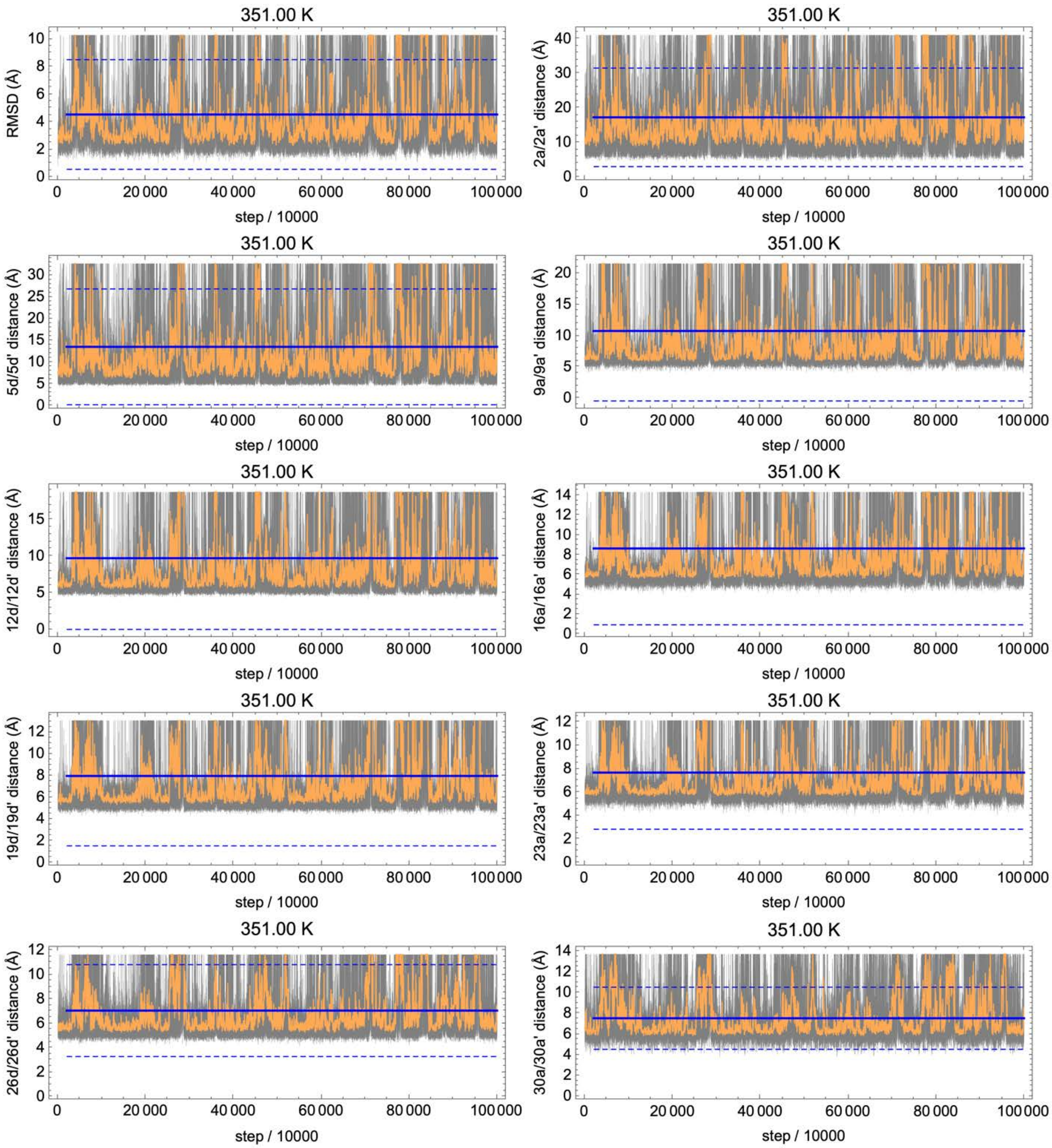

Measure at 351.00 K	Average (Å)	Standard Deviation (Å)	Slope (Å / μs)
RMSD	4.52977	3.97225	0.974255
5d/5d' distance	13.4917	13.3098	3.33949
12d/12d' distance	9.71676	9.71888	2.50454
19d/19d' distance	7.95165	6.42788	1.5416
26d/26d' distance	7.04072	3.76368	0.717184
2a/2a' distance	17.2812	14.2537	3.36185
9a/9a' distance	10.7454	11.195	2.93196
16a/16a' distance	8.63422	7.70807	1.88956
23a/23a' distance	7.66709	4.83236	1.02796
30a/30a' distance	7.52057	2.99888	0.219315
Core Distance	10.0055	9.65857	

Figure S135. Plots of RMSD and residue packing distances for T-REMD simulations of variant **6e/1g'-xx** at 351.00 K.

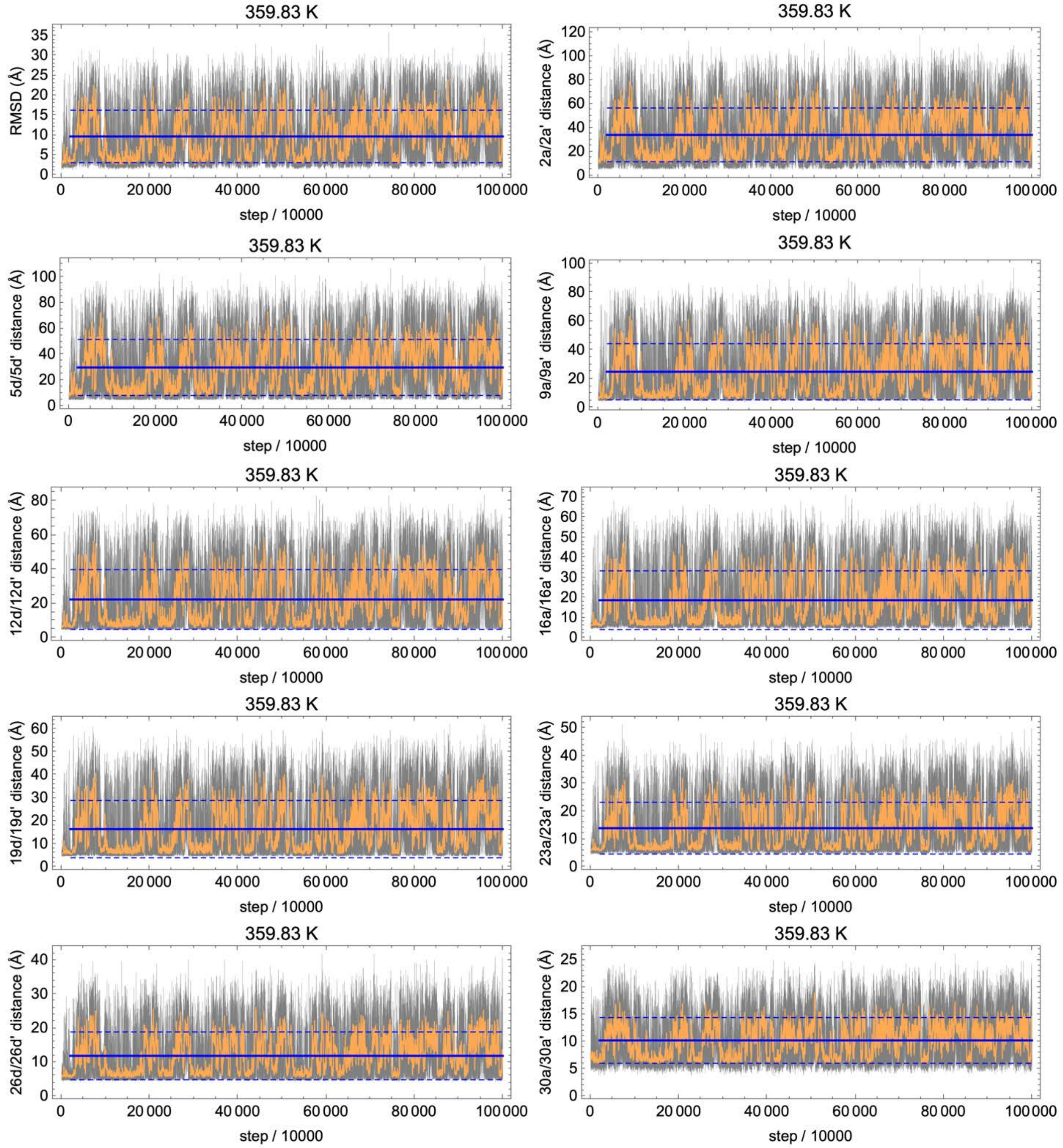

Measure at 359.83 K	Average (Å)	Standard Deviation (Å)	Slope (Å / μs)
RMSD	9.65767	6.5945	2.25837
5d/5d' distance	29.8565	21.7297	7.26035
12d/12d' distance	22.3367	17.4504	5.72468
19d/19d' distance	16.4412	12.4595	3.81652
26d/26d' distance	11.8624	7.03195	2.05038
2a/2a' distance	34.0277	22.5714	7.27576
9a/9a' distance	25.0019	19.5303	6.5173
16a/16a' distance	18.7017	14.6397	4.53755
23a/23a' distance	13.9659	9.31153	2.82422
30a/30a' distance	10.2213	4.19676	1.16669
Core Distance	20.2684	17.4077	

Figure S136. Plots of RMSD and residue packing distances for T-REMD simulations of variant **6e/1g’-xx** at 359.83 K.

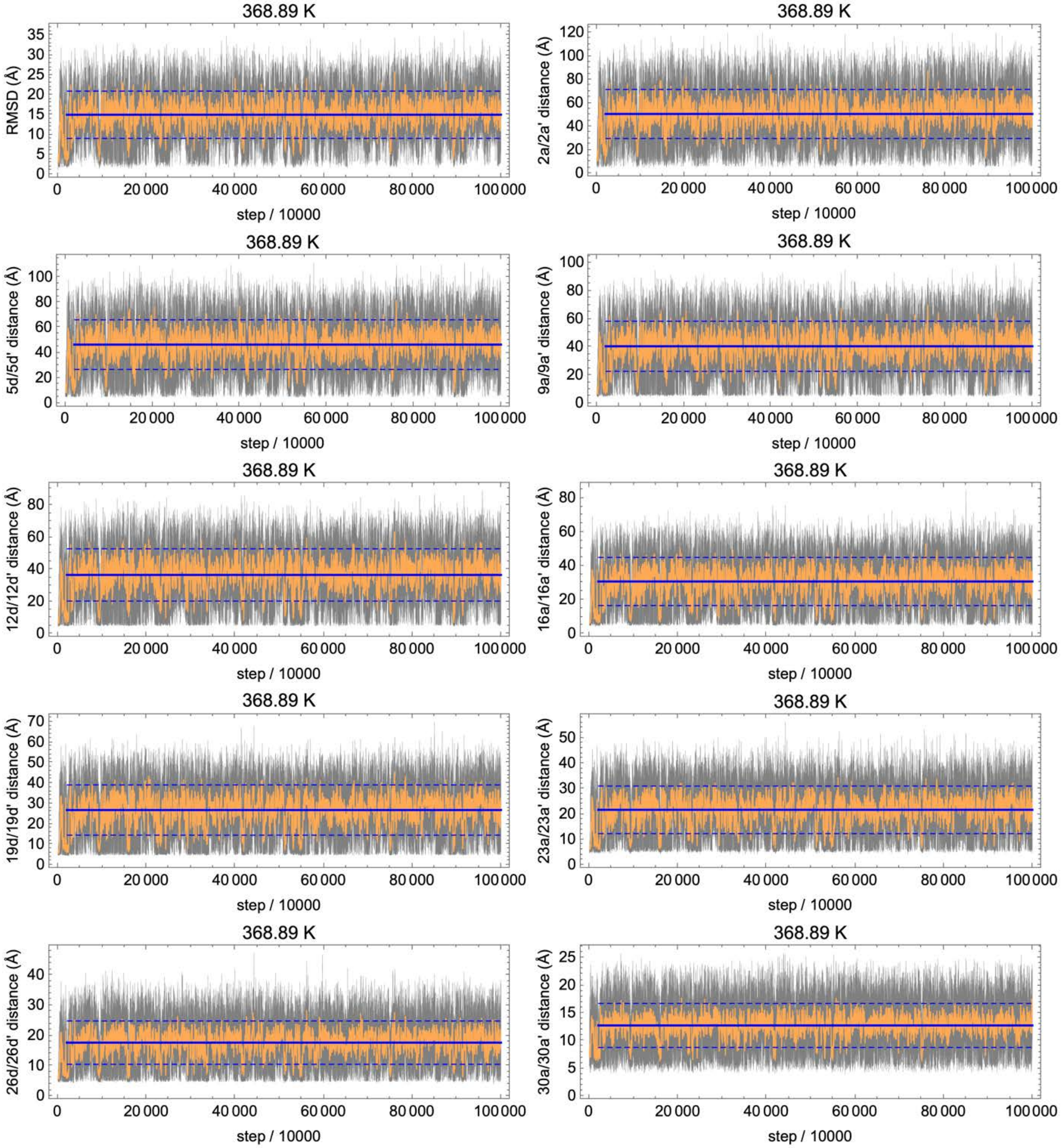

Measure at 368.89 K	Average (Å)	Standard Deviation (Å)	Slope (Å / μs)
RMSD	14.9892	5.93064	0.565193
5d/5d' distance	46.4381	19.6979	1.7559
12d/12d' distance	36.51	16.2477	1.48964
19d/19d' distance	26.859	12.2881	1.02995
26d/26d' distance	17.6691	7.15534	0.544647
2a/2a' distance	50.5775	20.9124	1.66692
9a/9a' distance	40.5099	17.8151	1.68213
16a/16a' distance	30.7495	14.1941	1.1973
23a/23a' distance	21.6702	9.37359	0.768005
30a/30a' distance	12.7612	3.97181	0.358662
Core Distance	31.5272	19.057	

Figure S137. Plots of RMSD and residue packing distances for T-REMD simulations of variant **6e/1g'-xx** at 368.89 K.

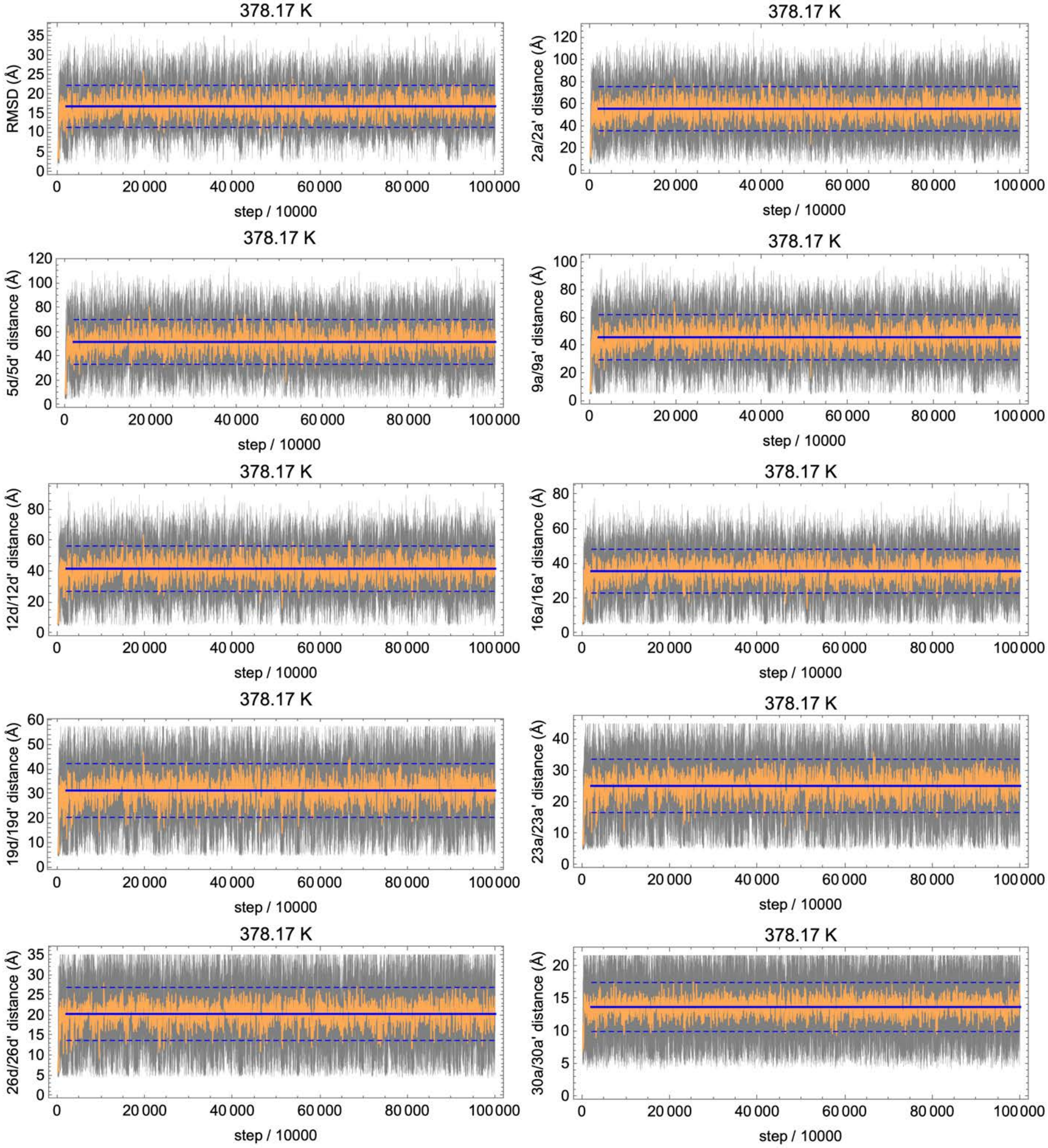

Measure at 378.17 K	Average (Å)	Standard Deviation (Å)	Slope (Å / μs)
RMSD	16.8835	5.41397	0.170649
5d/5d' distance	51.8955	18.3954	0.623004
12d/12d' distance	41.7992	14.7042	0.500259
19d/19d' distance	31.4012	10.9504	0.263713
26d/26d' distance	20.3653	6.60876	0.146396
2a/2a' distance	55.8391	19.9566	0.553384
9a/9a' distance	45.9842	16.2603	0.567848
16a/16a' distance	35.7369	12.6392	0.294796
23a/23a' distance	25.1548	8.50671	0.184006
30a/30a' distance	13.7313	3.76163	0.0932653
Core Distance	35.7675	19.106	

Figure S138. Plots of RMSD and residue packing distances for T-REMD simulations of variant **6e/1g'-xx** at 378.17 K.

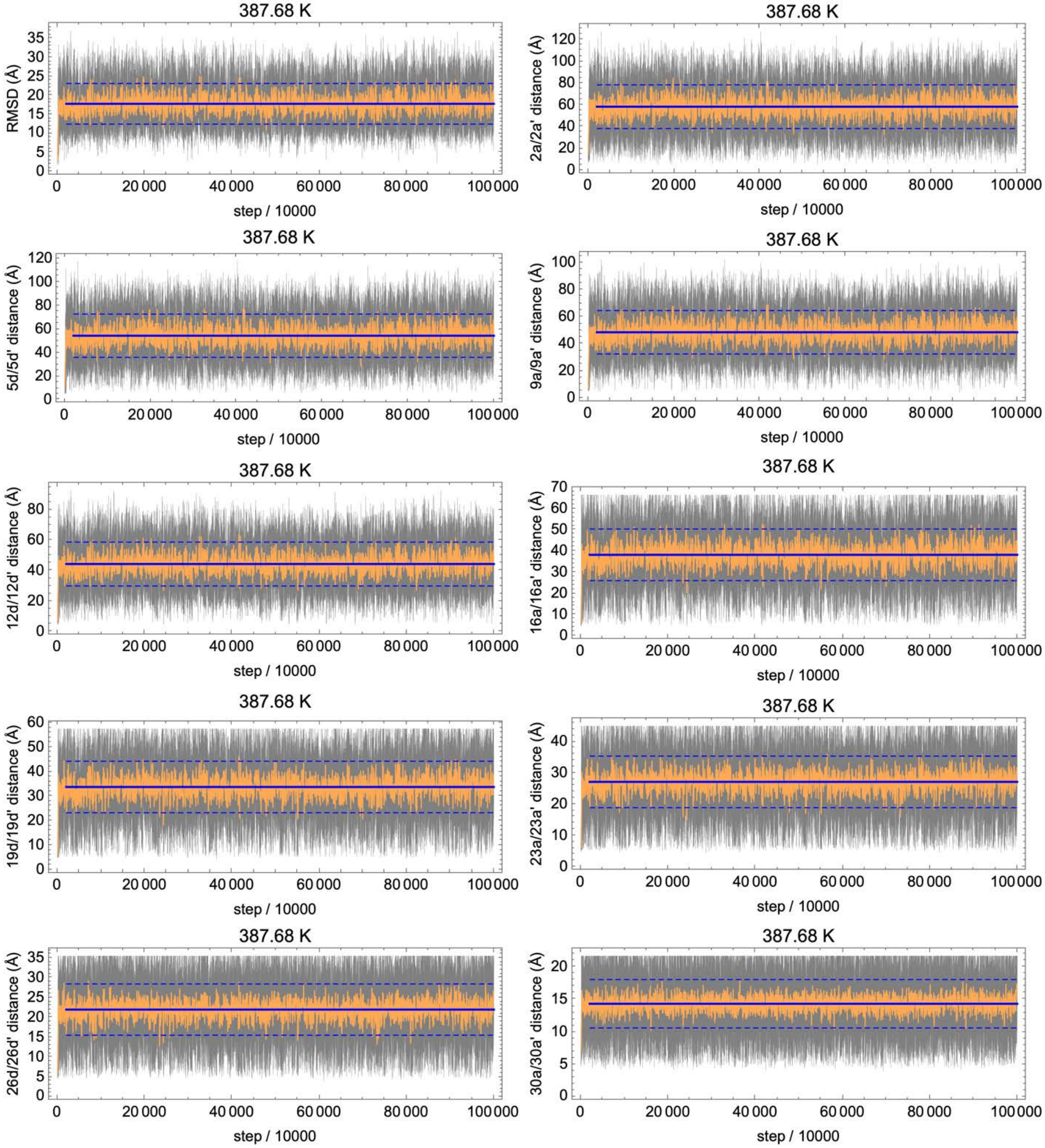

Measure at 387.68 K	Average (Å)	Standard Deviation (Å)	Slope (Å / μs)
RMSD	17.7957	5.34365	0.214538
5d/5d' distance	54.3349	18.3677	0.731794
12d/12d' distance	44.2656	14.4041	0.59242
19d/19d' distance	33.7293	10.5171	0.324515
26d/26d' distance	21.9685	6.4607	0.183573
2a/2a' distance	58.2292	20.0814	0.663667
9a/9a' distance	48.4542	16.0362	0.71464
16a/16a' distance	38.1454	12.1726	0.40374
23a/23a' distance	27.1012	8.23466	0.217451
30a/30a' distance	14.3166	3.72272	0.0857425
Core Distance	37.8383	19.3413	

Figure S139. Plots of RMSD and residue packing distances for T-REMD simulations of variant **6e/1g'-xx** at 387.68 K.

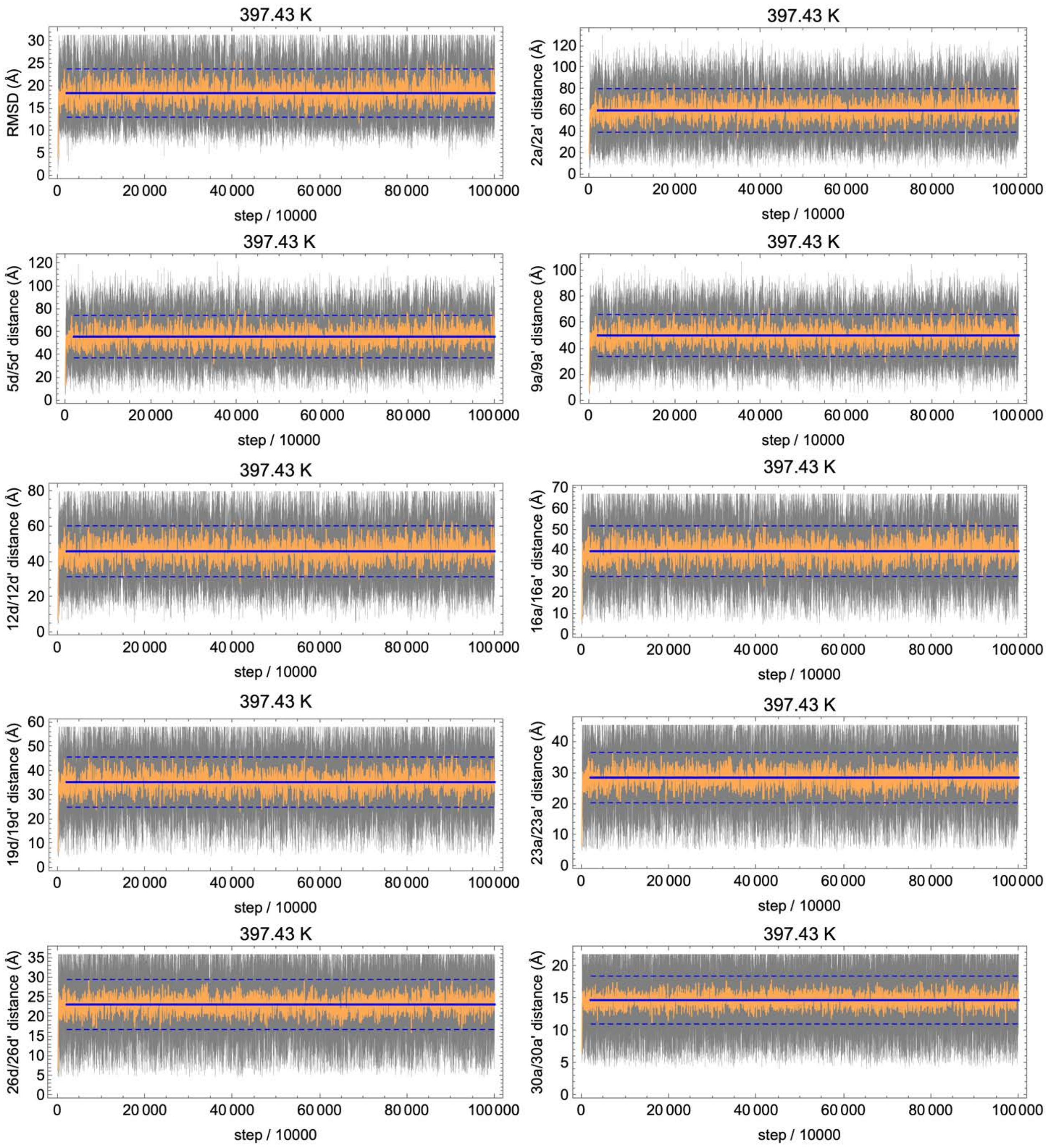

Measure at 397.43 K	Average (Å)	Standard Deviation (Å)	Slope (Å / μs)
RMSD	18.4501	5.36599	0.244253
5d/5d' distance	56.0373	18.615	0.849899
12d/12d' distance	45.9863	14.4184	0.625233
19d/19d' distance	35.473	10.3663	0.334738
26d/26d' distance	23.195	6.38354	0.129167
2a/2a' distance	59.9171	20.3664	0.851481
9a/9a' distance	50.1456	16.1574	0.772459
16a/16a' distance	39.8978	12.0564	0.420895
23a/23a' distance	28.5319	8.12157	0.205881
30a/30a' distance	14.7746	3.71155	0.077847
Core Distance	39.3287	19.6155	

Figure S140. Plots of RMSD and residue packing distances for T-REMD simulations of variant **6e/1g'-xx** at 397.43 K.

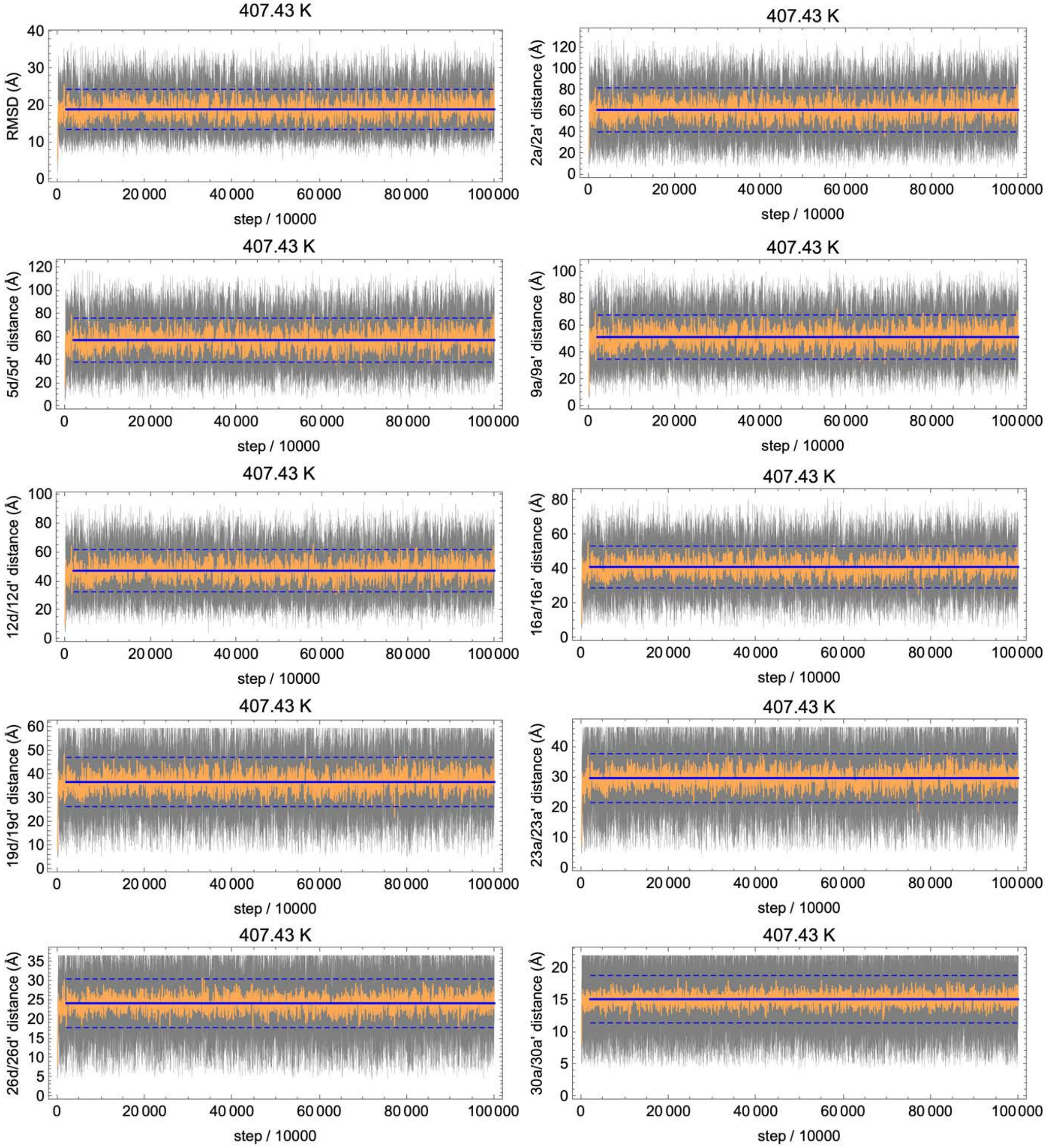

Measure at 407.43 K	Average (Å)	Standard Deviation (Å)	Slope (Å / μs)
RMSD	18.9753	5.4279	0.243428
5d/5d' distance	57.3206	18.958	0.81586
12d/12d' distance	47.3298	14.6389	0.575197
19d/19d' distance	36.8263	10.398	0.392992
26d/26d' distance	24.2388	6.32183	0.160089
2a/2a' distance	61.1797	20.8236	0.78492
9a/9a' distance	51.476	16.4199	0.737659
16a/16a' distance	41.2502	12.162	0.465275
23a/23a' distance	29.757	8.09398	0.264332
30a/30a' distance	15.1678	3.70099	0.0881515
Core Distance	40.5051	19.9038	

Figure S141. Plots of RMSD and residue packing distances for T-REMD simulations of variant **6e/1g'-xx** at 407.43 K.

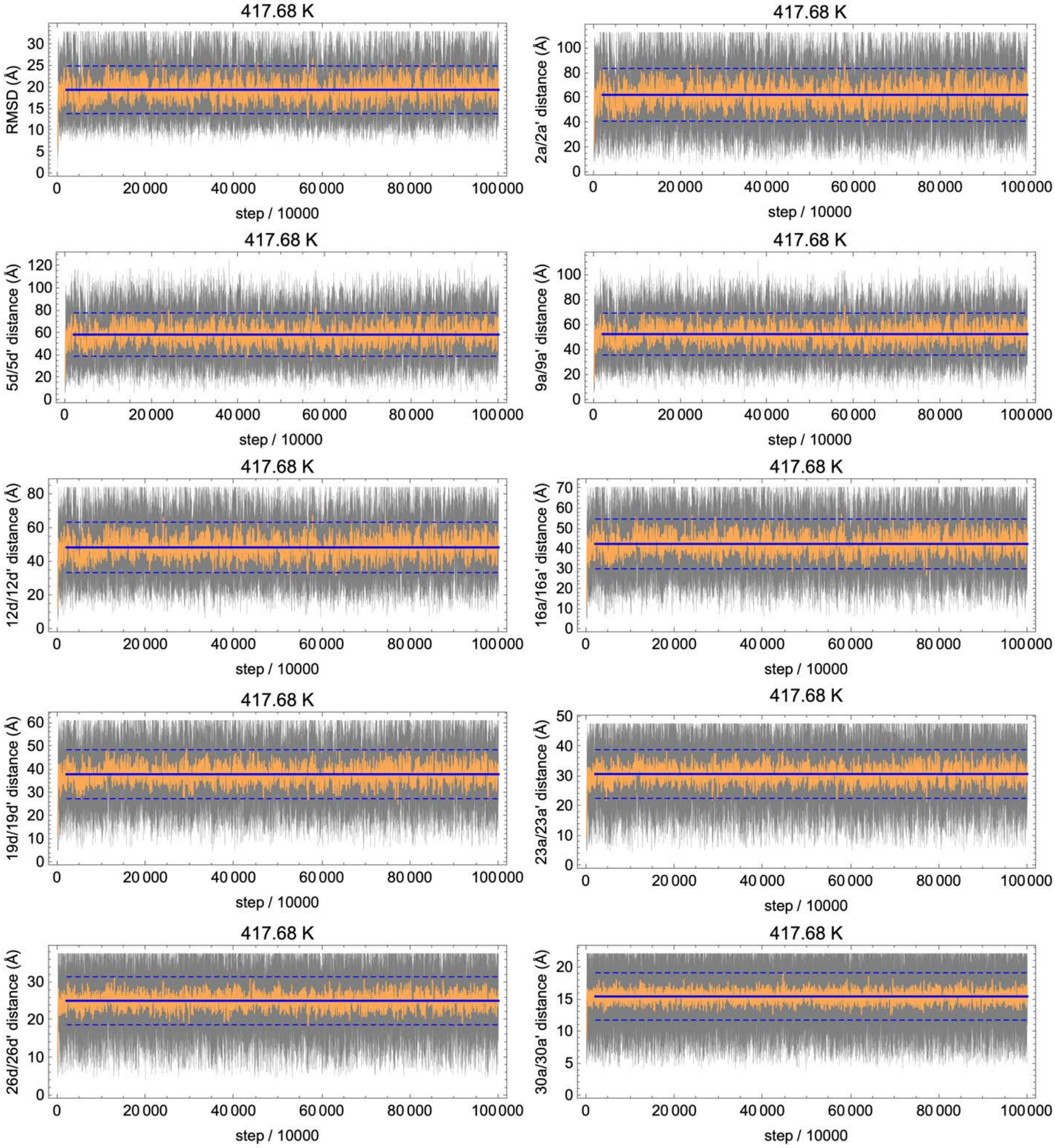

Measure at 417.68 K	Average (Å)	Standard Deviation (Å)	Slope (Å / μs)
RMSD	19.4648	5.53038	0.172673
5d/5d' distance	58.539	19.4319	0.621615
12d/12d' distance	48.5095	14.9626	0.419937
19d/19d' distance	37.9897	10.5302	0.238783
26d/26d' distance	25.1062	6.35371	0.039009
2a/2a' distance	62.4207	21.317	0.648285
9a/9a' distance	52.6632	16.8008	0.534059
16a/16a' distance	42.4242	12.3786	0.26336
23a/23a' distance	30.8095	8.16674	0.0747939
30a/30a' distance	15.498	3.69317	0.00883673
Core Distance	41.5511	20.2677	

Figure S142. Plots of RMSD and residue packing distances for T-REMD simulations of variant **6e/1g'-xx** at 417.68 K.

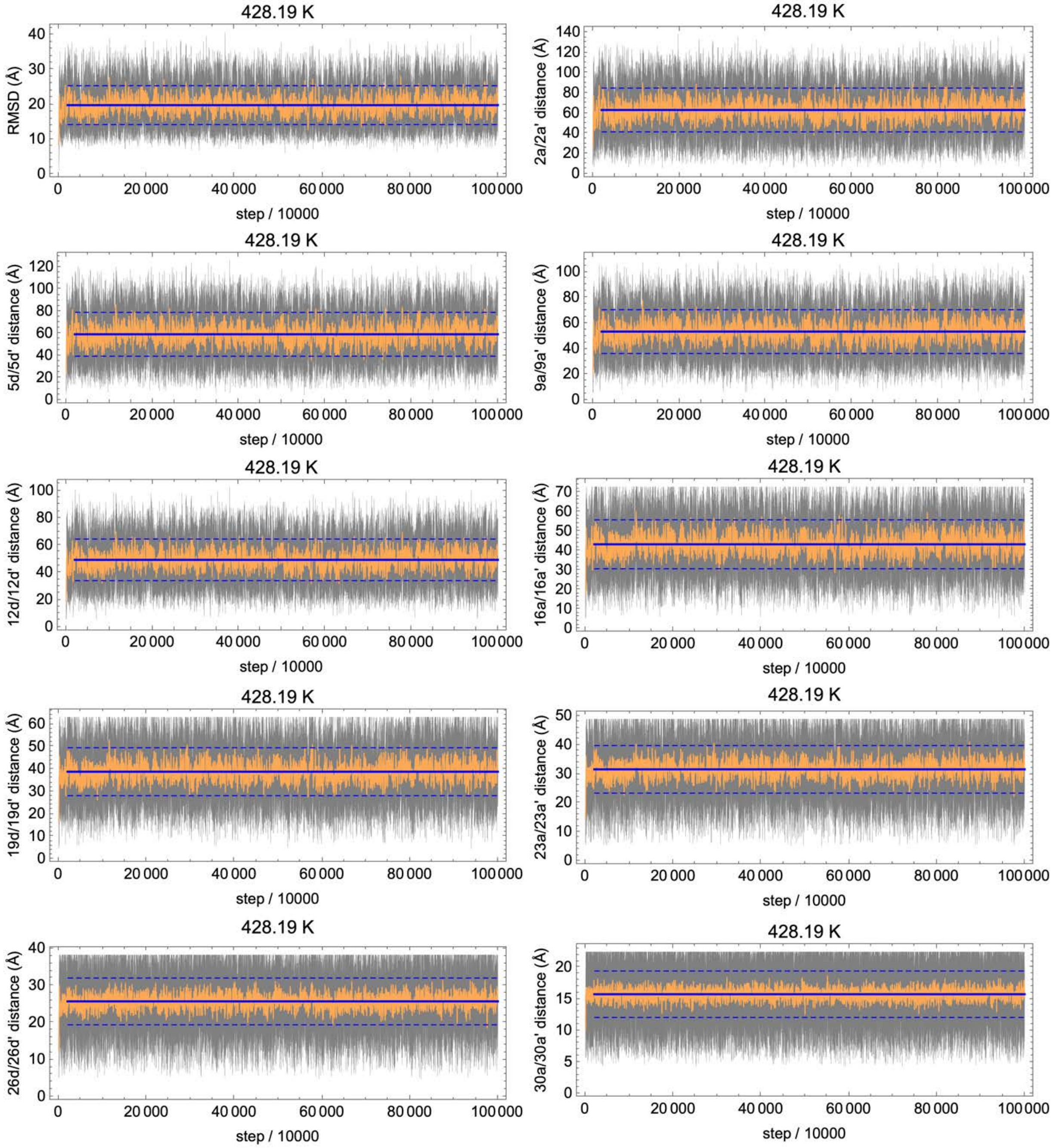

Measure at 428.19 K	Average (Å)	Standard Deviation (Å)	Slope (Å / μs)
RMSD	19.7769	5.5854	0.135365
5d/5d' distance	59.1953	19.8046	0.495233
12d/12d' distance	49.2729	15.2586	0.252363
19d/19d' distance	38.8003	10.6819	0.202752
26d/26d' distance	25.7144	6.31352	0.0962036
2a/2a' distance	63.1098	21.6792	0.462632
9a/9a' distance	53.3507	17.1235	0.399153
16a/16a' distance	43.2488	12.5805	0.198962
23a/23a' distance	31.5583	8.21269	0.117401
30a/30a' distance	15.7502	3.68434	0.0344801
Core Distance	42.2223	20.4917	

Figure S143. Plots of RMSD and residue packing distances for T-REMD simulations of variant **6e/1g'-xx** at 428.19 K.

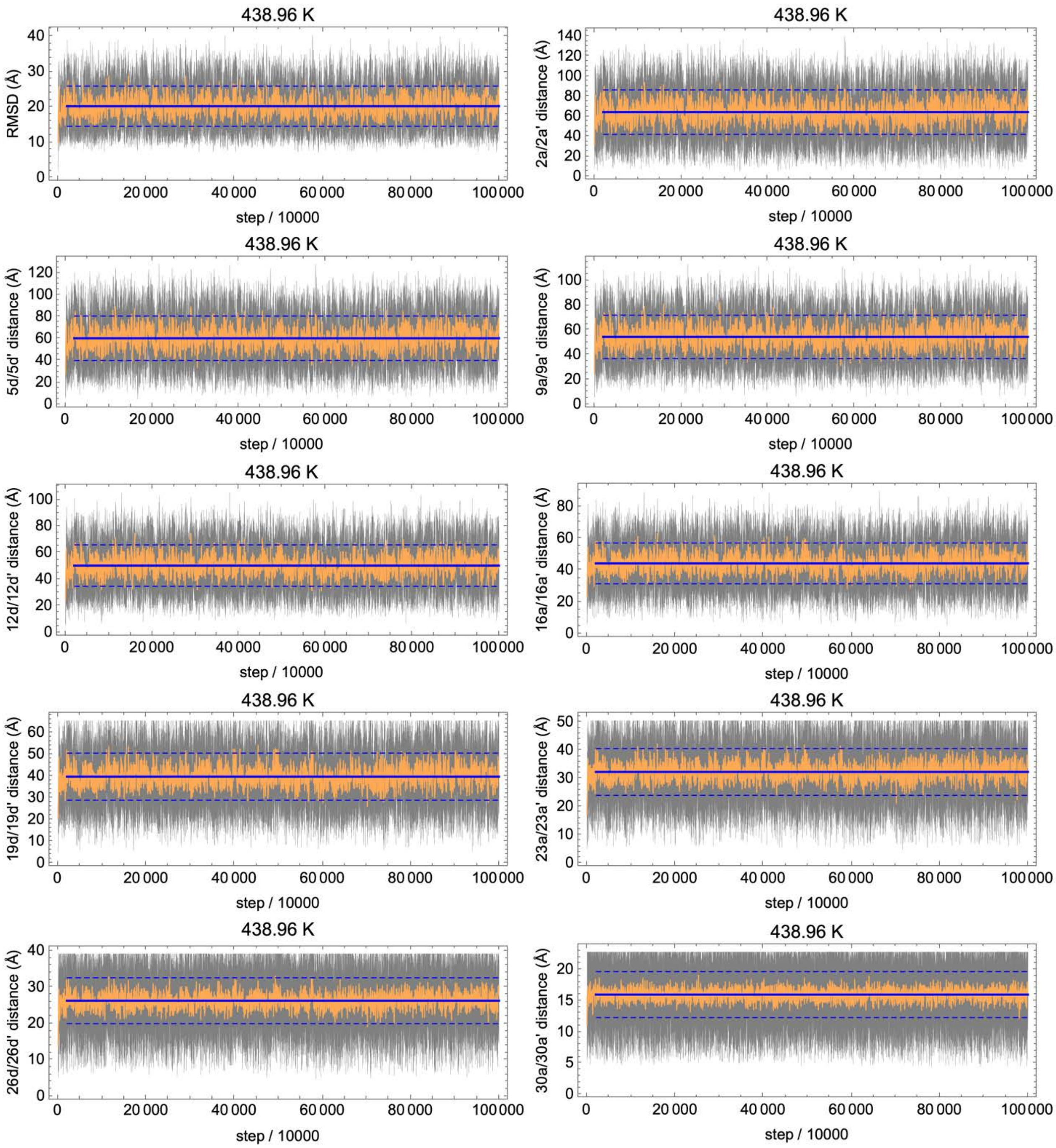

Measure at 438.96 K	Average (Å)	Standard Deviation (Å)	Slope (Å / μs)
RMSD	20.1826	5.6788	0.116067
5d/5d' distance	60.2195	20.2729	0.28718
12d/12d' distance	50.2473	15.6161	0.262776
19d/19d' distance	39.6572	10.8326	0.215798
26d/26d' distance	26.2571	6.31437	0.159682
2a/2a' distance	64.1312	22.2033	0.264225
9a/9a' distance	54.3619	17.556	0.319824
16a/16a' distance	44.1722	12.8088	0.252143
23a/23a' distance	32.3058	8.2995	0.168111
30a/30a' distance	15.9745	3.67858	0.0301558
Core Distance	43.0363	20.8753	

Figure S144. Plots of RMSD and residue packing distances for T-REMD simulations of variant **6e/1g'-xx** at 438.96 K.

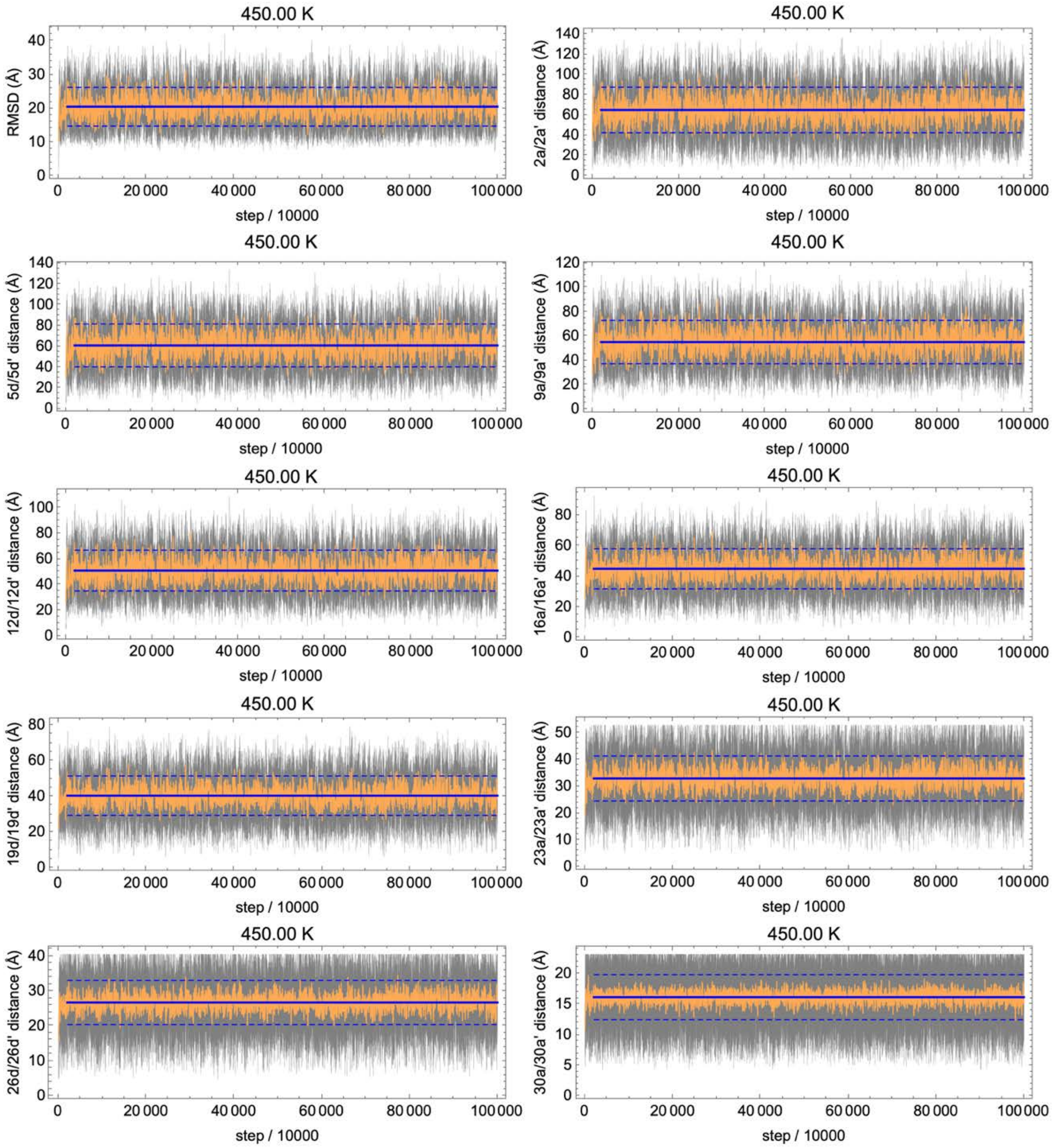

Measure at 450.00 K	Average (Å)	Standard Deviation (Å)	Slope (Å / μs)
RMSD	20.5083	5.73357	0.0682275
5d/5d' distance	60.9662	20.5944	0.208645
12d/12d' distance	50.9996	15.9234	0.259596
19d/19d' distance	40.3642	11.043	0.15938
26d/26d' distance	26.721	6.3237	0.137933
2a/2a' distance	64.9478	22.5343	0.104368
9a/9a' distance	55.1234	17.8482	0.253159
16a/16a' distance	44.9451	13.1027	0.209711
23a/23a' distance	32.9473	8.40189	0.117136
30a/30a' distance	16.1457	3.67776	0.0121498
Core Distance	43.6845	21.1632	

Figure S145. Plots of RMSD and residue packing distances for T-REMD simulations of variant **6e/1g'-xx** at 450.00 K.

(return to Table of Contents)

s6e/1g'-x4x

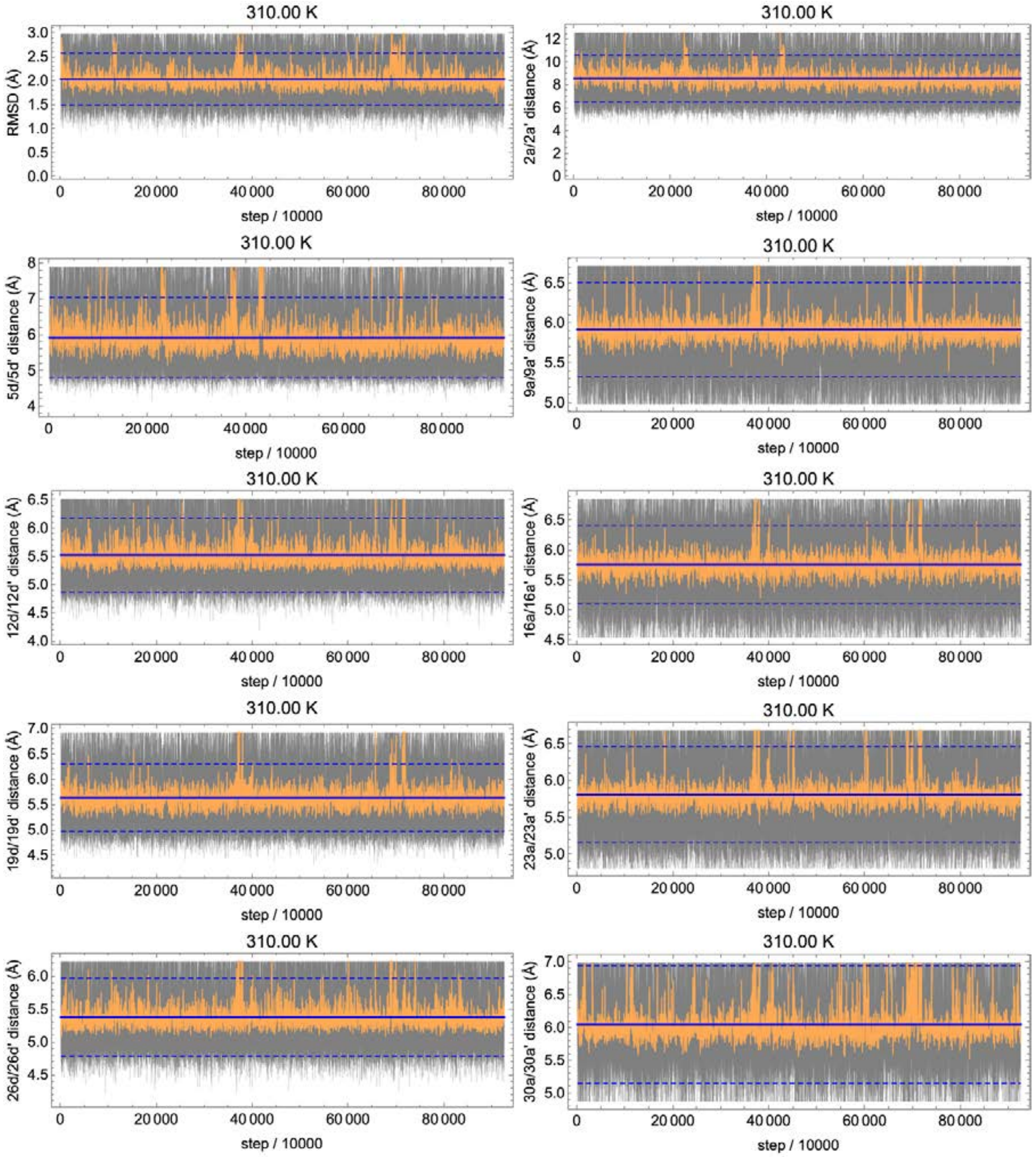

Measure at 310.00 K	Average (Å)	Standard Deviation (Å)	Slope (Å / μs)
RMSD	2.04224	0.545518	−0.00916253
5d/5d' distance	5.93152	1.11996	−0.105849
12d/12d' distance	5.52987	0.654407	−0.00477869
19d/19d' distance	5.6428	0.662351	0.0160294
26d/26d' distance	5.38266	0.593646	0.00323221
2a/2a' distance	8.60656	2.05099	−0.280371
9a/9a' distance	5.91932	0.591363	−0.00232328
16a/16a' distance	5.76679	0.648123	0.0159842
23a/23a' distance	5.81796	0.652374	0.0169486
30a/30a' distance	6.05191	0.898438	0.0462122
Core Distance	6.07215	1.34417	

Figure S146. Plots of RMSD and residue packing distances for T-REMD simulations of variant **s6e/1g'-x4x** at 310.00 K.

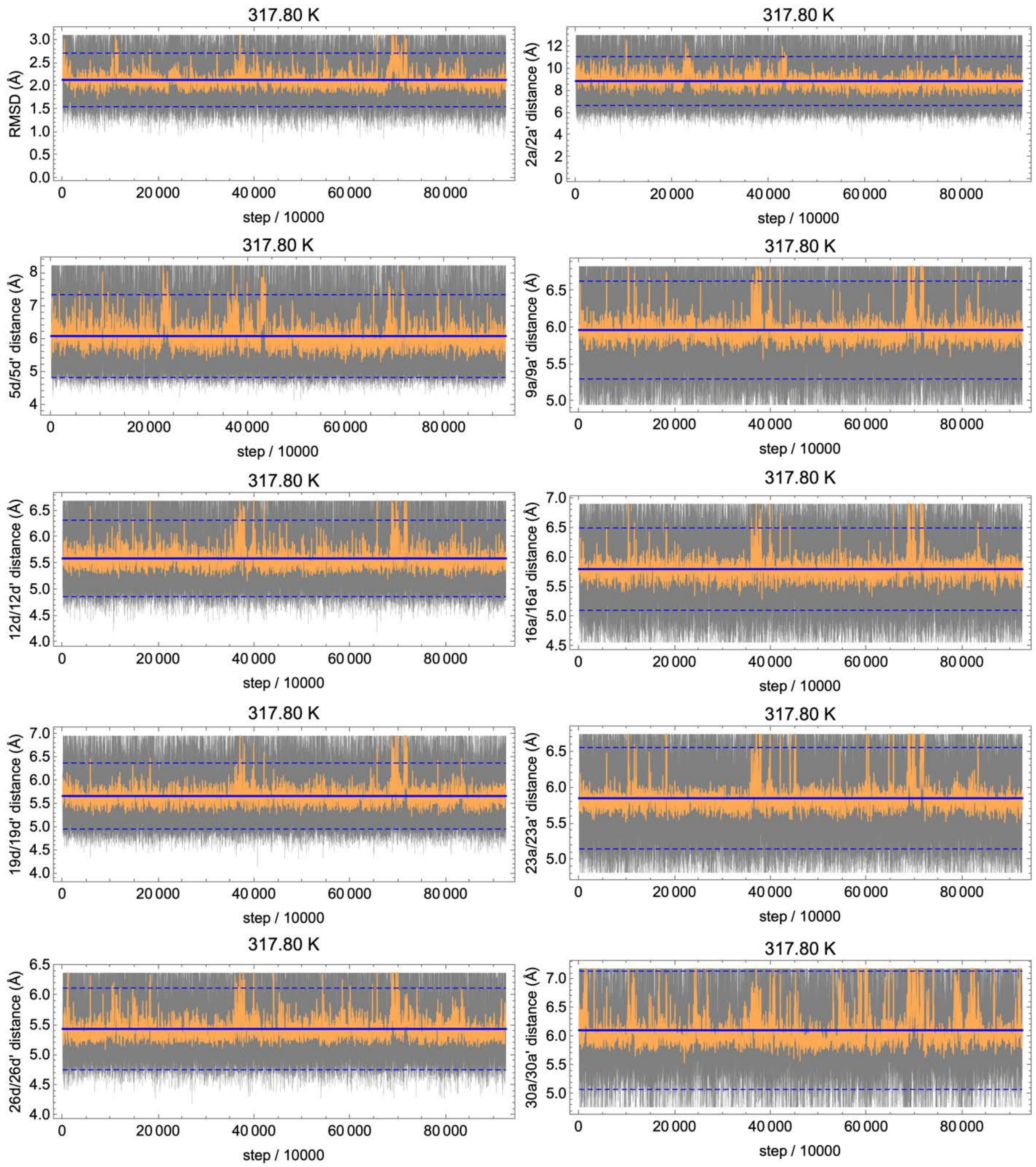

Measure at 317.80 K	Average (Å)	Standard Deviation (Å)	Slope (Å / μs)
RMSD	2.13178	0.581365	−0.01574
5d/5d' distance	6.08496	1.25864	−0.121761
12d/12d' distance	5.59357	0.728395	−0.0161538
19d/19d' distance	5.66538	0.709048	0.017922
26d/26d' distance	5.43694	0.681316	0.00399623
2a/2a' distance	8.86944	2.206	−0.293195
9a/9a' distance	5.96813	0.664978	−0.00671018
16a/16a' distance	5.80274	0.696698	0.014226
23a/23a' distance	5.85314	0.707001	0.01827
30a/30a' distance	6.1082	1.0292	0.0516136
Core Distance	6.15361	1.45795	

Figure S147. Plots of RMSD and residue packing distances for T-REMD simulations of variant **s6e/1g’-x4x** at 317.80 K.

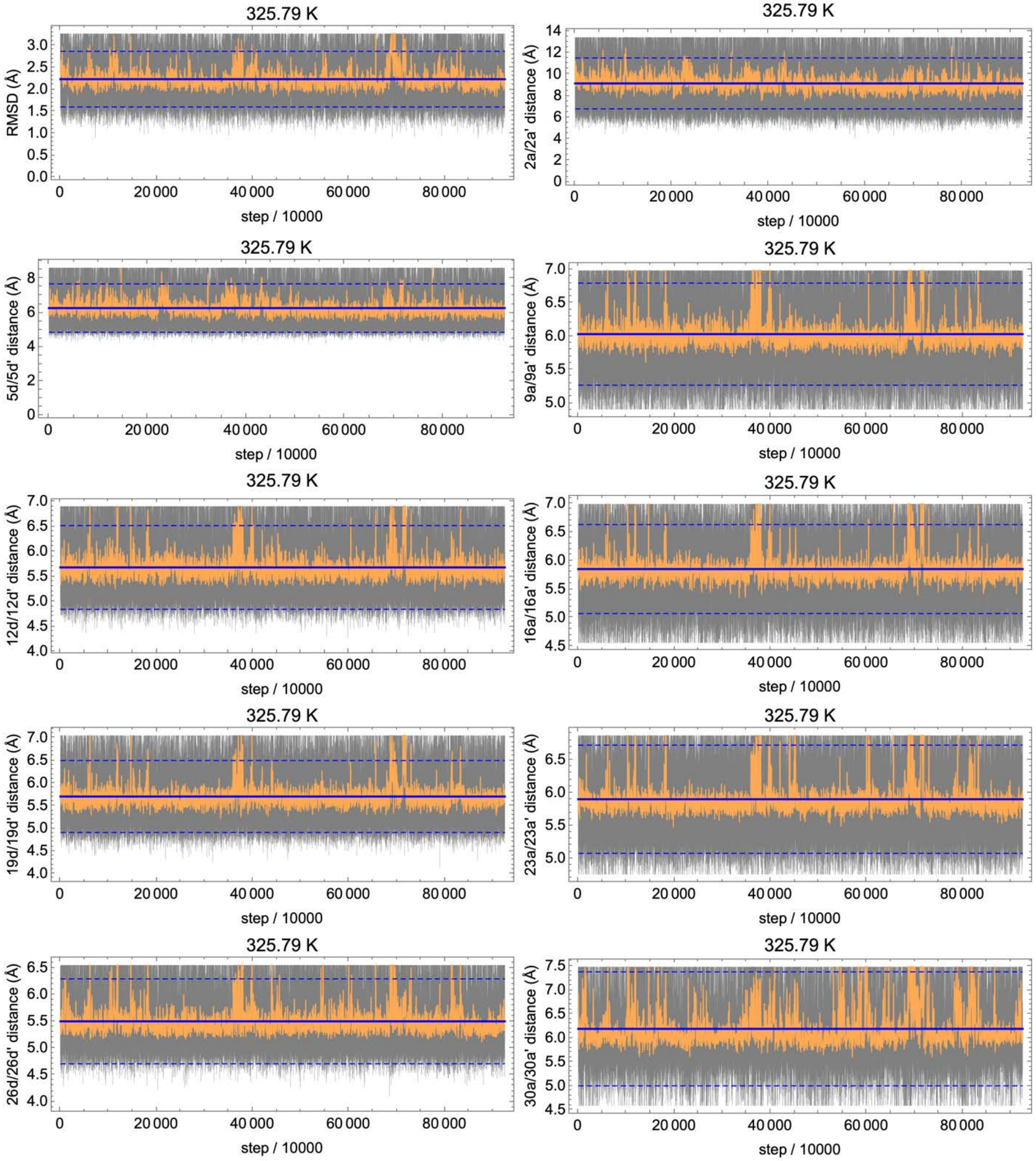

Measure at 325.79 K	Average (Å)	Standard Deviation (Å)	Slope (Å / μs)
RMSD	2.23258	0.632886	−0.0143918
5d/5d' distance	6.26493	1.41703	−0.126469
12d/12d' distance	5.67877	0.835907	−0.0135528
19d/19d' distance	5.70385	0.794258	0.0205032
26d/26d' distance	5.4991	0.792456	−0.00797398
2a/2a' distance	9.15337	2.36736	−0.29489
9a/9a' distance	6.03113	0.764574	−0.0107421
16a/16a' distance	5.85058	0.778833	0.00890712
23a/23a' distance	5.90033	0.823234	0.0152841
30a/30a' distance	6.19017	1.18698	0.0755552
Core Distance	6.25247	1.59161	

Figure S148. Plots of RMSD and residue packing distances for T-REMD simulations of variant **s6e/1g'-x4x** at 325.79 K.

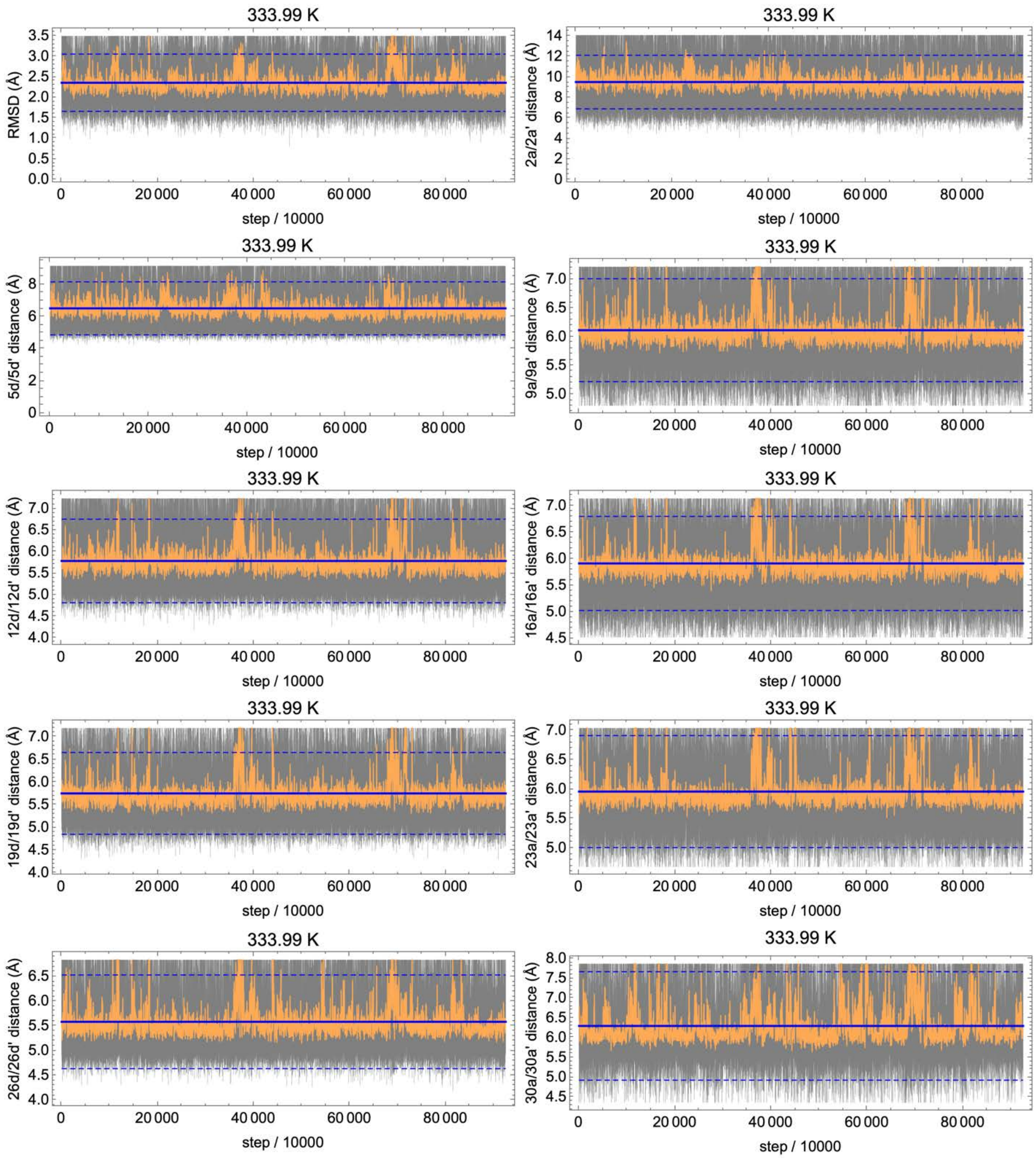

Measure at 333.99 K	Average (Å)	Standard Deviation (Å)	Slope (Å / μs)
RMSD	2.35777	0.698217	−0.00863421
5d/5d' distance	6.51248	1.64939	−0.156012
12d/12d' distance	5.78383	0.967277	−0.0255129
19d/19d' distance	5.75228	0.901114	0.0239608
26d/26d' distance	5.58394	0.946798	−0.00367891
2a/2a' distance	9.5191	2.61317	−0.321544
9a/9a' distance	6.11428	0.895483	−0.0179254
16a/16a' distance	5.91226	0.886435	0.0119334
23a/23a' distance	5.95975	0.95343	0.0172855
30a/30a' distance	6.29686	1.37005	0.0873531
Core Distance	6.38164	1.77308	

Figure S149. Plots of RMSD and residue packing distances for T-REMD simulations of variant **s6e/1g'-x4x** at 333.99 K.

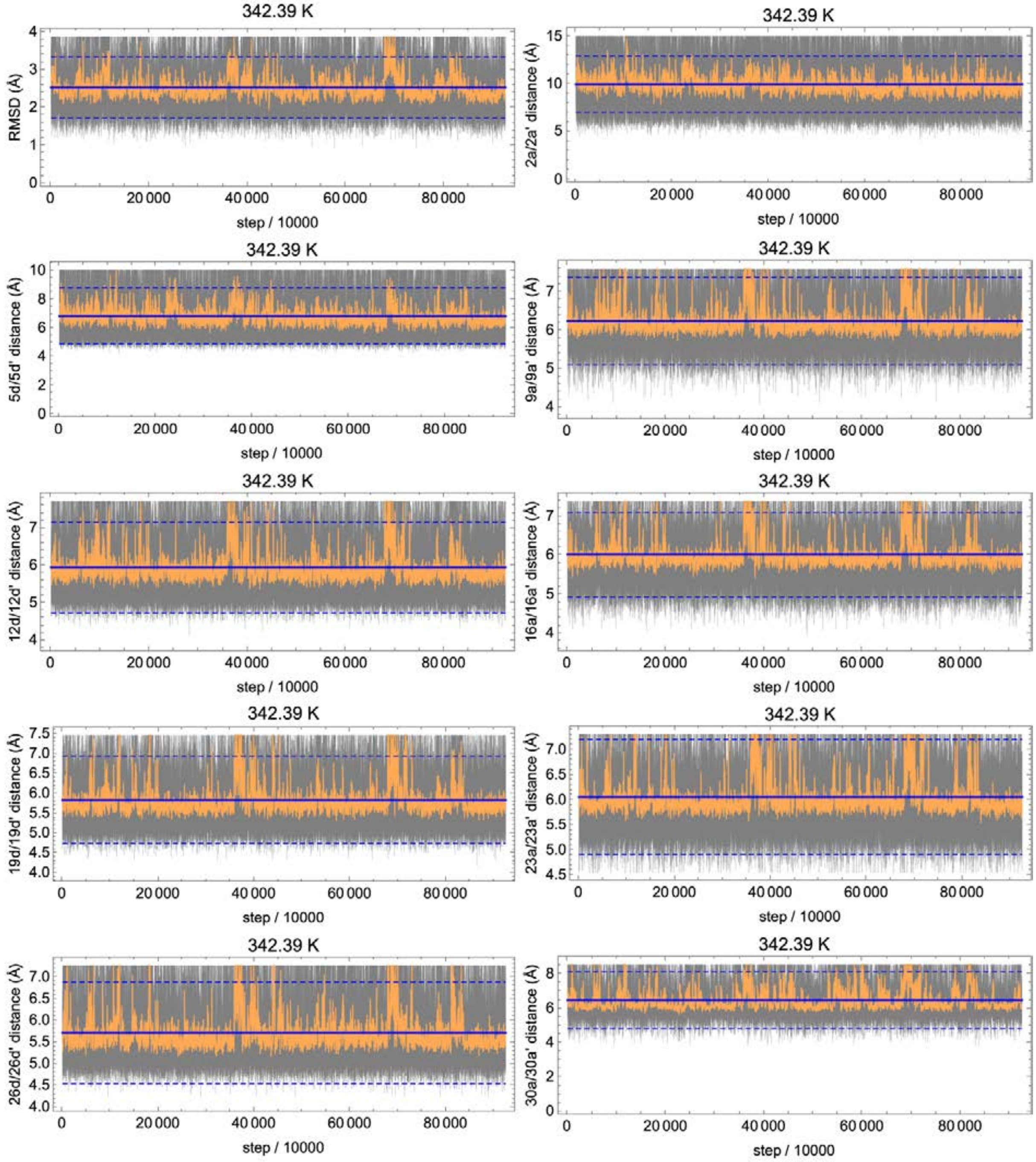

Measure at 342.39 K	Average (Å)	Standard Deviation (Å)	Slope (Å / μs)
RMSD	2.52895	0.804064	−0.0182668
5d/5d' distance	6.84831	1.96168	−0.213139
12d/12d' distance	5.94198	1.21259	−0.0438409
19d/19d' distance	5.8414	1.10132	0.0104953
26d/26d' distance	5.71657	1.17239	−0.01984
2a/2a' distance	9.99428	2.93135	−0.393838
9a/9a' distance	6.24091	1.13588	−0.0451412
16a/16a' distance	6.01864	1.08318	−0.00447645
23a/23a' distance	6.05783	1.14832	0.0106318
30a/30a' distance	6.47075	1.64866	0.0956843
Core Distance	6.57007	2.03142	

Figure S150. Plots of RMSD and residue packing distances for T-REMD simulations of variant **s6e/1g'-x4x** at 342.39 K.

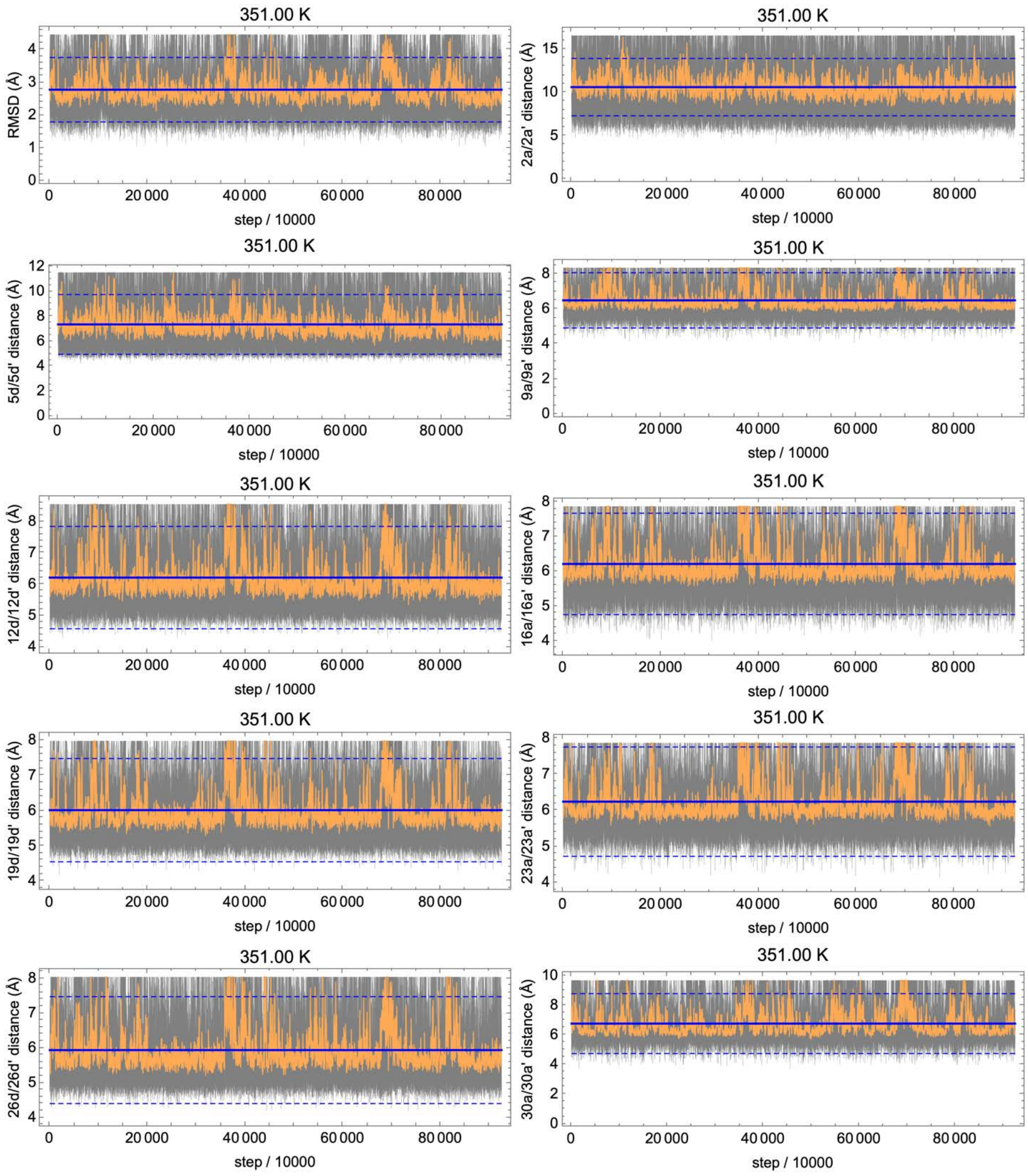

Measure at 351.00 K	Average (Å)	Standard Deviation (Å)	Slope (Å / μs)
RMSD	2.7726	0.982621	−0.0323247
5d/5d' distance	7.3397	2.39804	−0.272869
12d/12d' distance	6.20982	1.63229	−0.080984
19d/19d' distance	6.0116	1.4661	−0.00753775
26d/26d' distance	5.9354	1.5356	−0.0222023
2a/2a' distance	10.5987	3.30762	−0.421642
9a/9a' distance	6.48173	1.57801	−0.0849569
16a/16a' distance	6.2082	1.45874	−0.0206072
23a/23a' distance	6.23809	1.51417	0.00577353
30a/30a' distance	6.75665	2.01944	0.115308
Core Distance	6.86443	2.40421	

Figure S151. Plots of RMSD and residue packing distances for T-REMD simulations of variant **s6e/1g'-x4x** at 351.00 K.

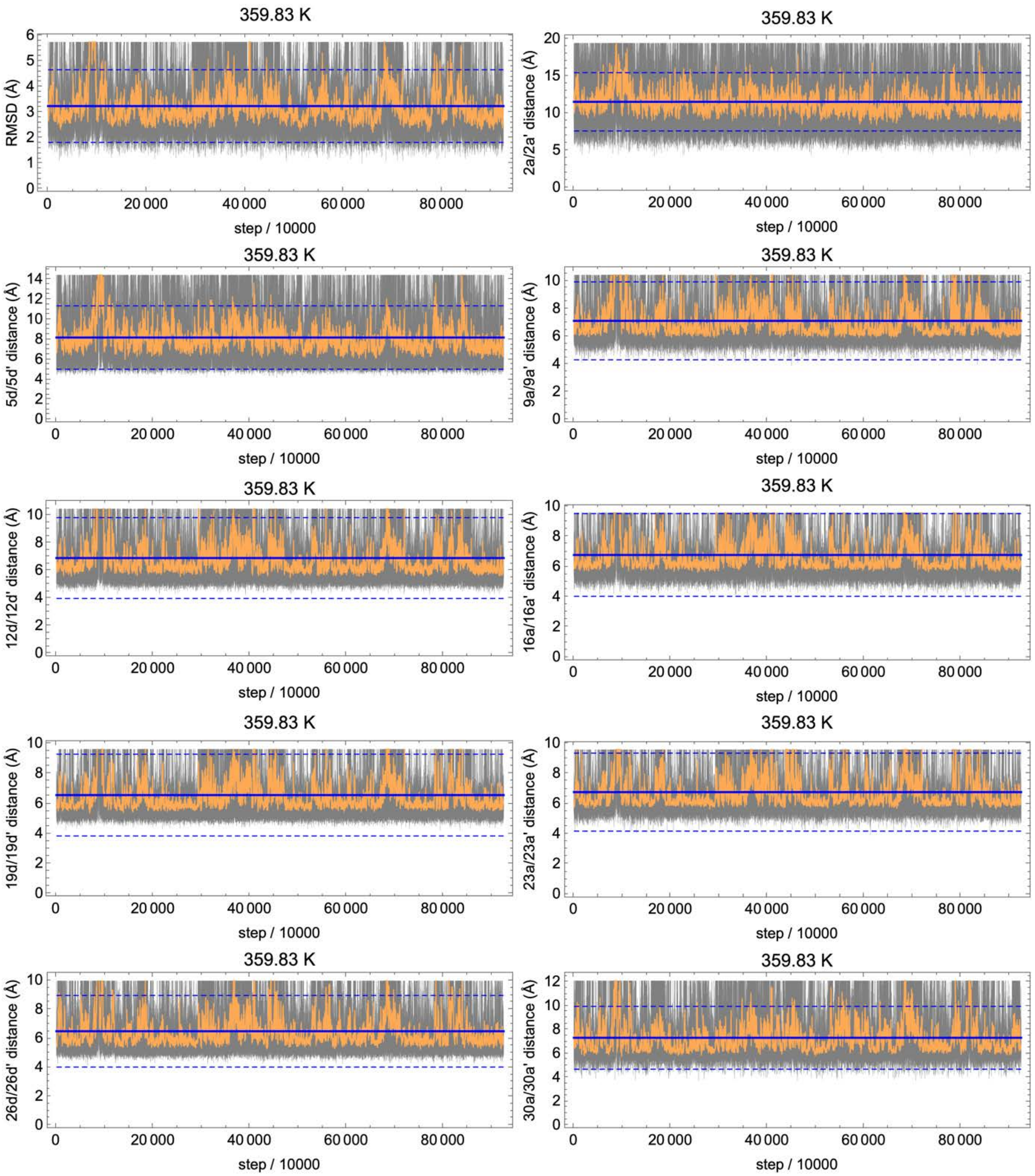

Measure at 359.83 K	Average (Å)	Standard Deviation (Å)	Slope (Å / μs)
RMSD	3.24186	1.42124	−0.168825
5d/5d' distance	8.18371	3.16448	−0.553273
12d/12d' distance	6.89458	2.92593	−0.438746
19d/19d' distance	6.55956	2.72058	−0.257797
26d/26d' distance	6.49055	2.47103	−0.198477
2a/2a' distance	11.5227	3.91791	−0.652632
9a/9a' distance	7.12019	2.81366	−0.392245
16a/16a' distance	6.77809	2.74072	−0.291598
23a/23a' distance	6.76605	2.58657	−0.185066
30a/30a' distance	7.31966	2.63196	−0.00276668
Core Distance	7.51501	3.27672	

Figure S152. Plots of RMSD and residue packing distances for T-REMD simulations of variant **s6e/1g'-x4x** at 359.83 K.

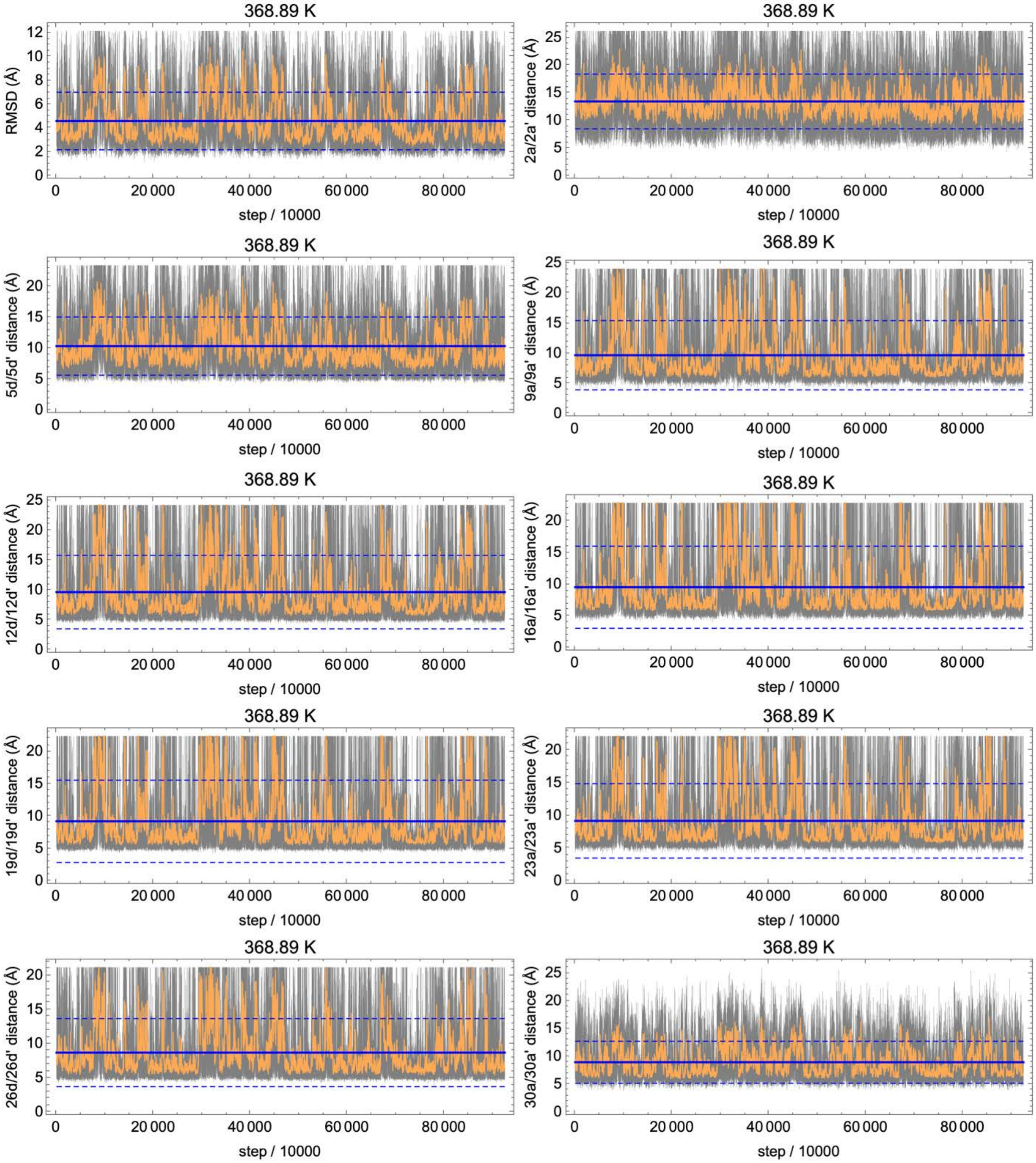

Measure at 368.89 K	Average (Å)	Standard Deviation (Å)	Slope (Å / μs)
RMSD	4.60191	2.41712	−0.323914
5d/5d' distance	10.352	4.70368	−0.777531
12d/12d' distance	9.66525	6.14561	−0.824275
19d/19d' distance	9.21077	6.35338	−0.701577
26d/26d' distance	8.68766	4.97281	−0.557335
2a/2a' distance	13.4493	4.95027	−0.759208
9a/9a' distance	9.6859	5.74597	−0.769183
16a/16a' distance	9.52743	6.4684	−0.701712
23a/23a' distance	9.17297	5.68216	−0.624315
30a/30a' distance	8.9581	3.77325	−0.255687
Core Distance	9.8566	5.64955	

Figure S153. Plots of RMSD and residue packing distances for T-REMD simulations of variant **s6e/1g'-x4x** at 368.89 K.

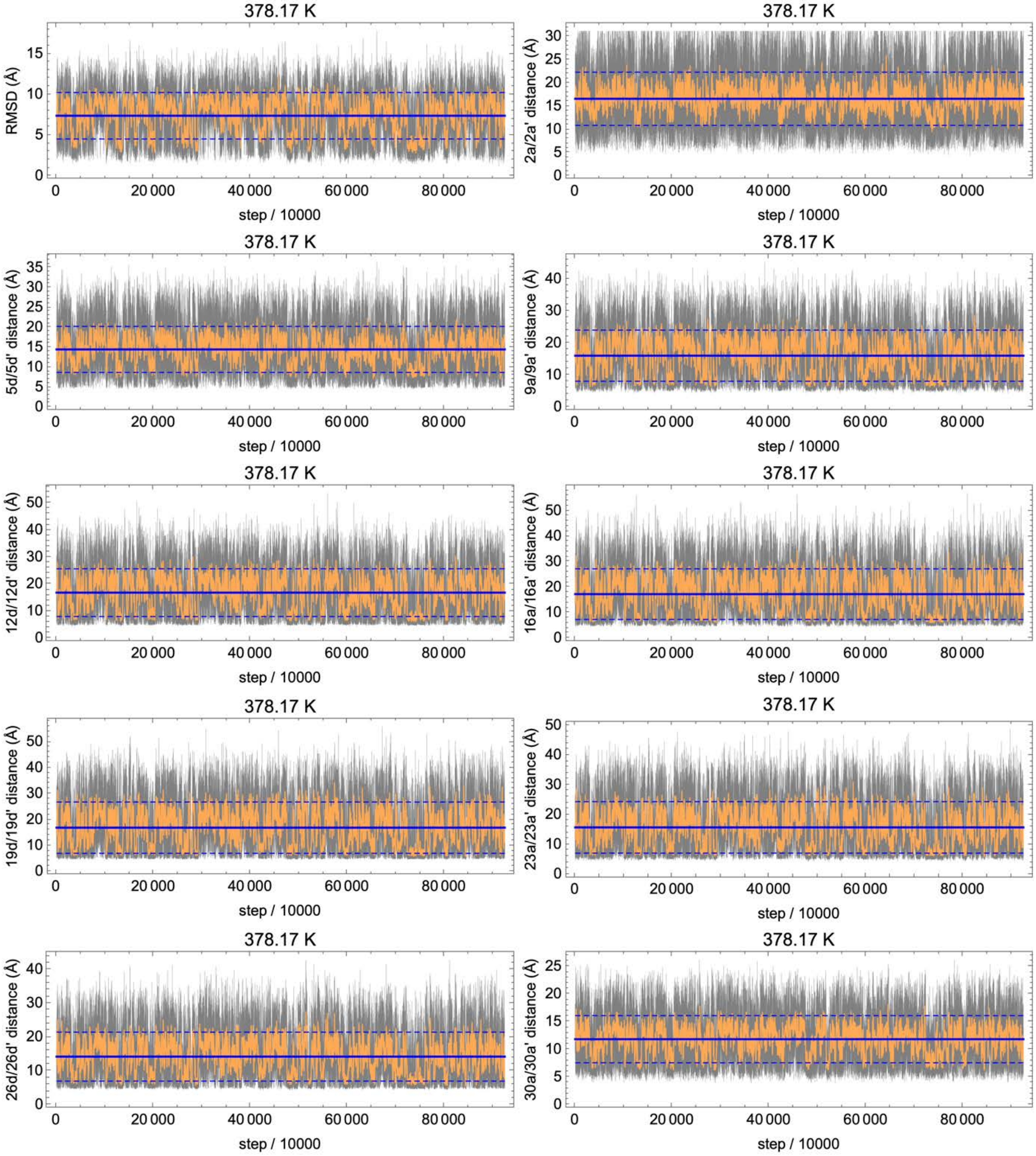

Measure at 378.17 K	Average (Å)	Standard Deviation (Å)	Slope (Å / μs)
RMSD	7.37721	2.85527	−0.193387
5d/5d' distance	14.4425	5.78099	−0.469696
12d/12d' distance	16.8035	8.82799	−0.726662
19d/19d' distance	16.8519	9.90077	−0.712488
26d/26d' distance	14.2052	7.24136	−0.588257
2a/2a' distance	16.5778	5.72841	−0.253346
9a/9a' distance	16.0298	7.9932	−0.584919
16a/16a' distance	17.2565	9.9335	−0.712751
23a/23a' distance	15.6756	8.64667	−0.671965
30a/30a' distance	11.836	4.26765	−0.189372
Core Distance	15.5199	7.98937	

Figure S154. Plots of RMSD and residue packing distances for T-REMD simulations of variant **s6e/1g'-x4x** at 378.17 K.

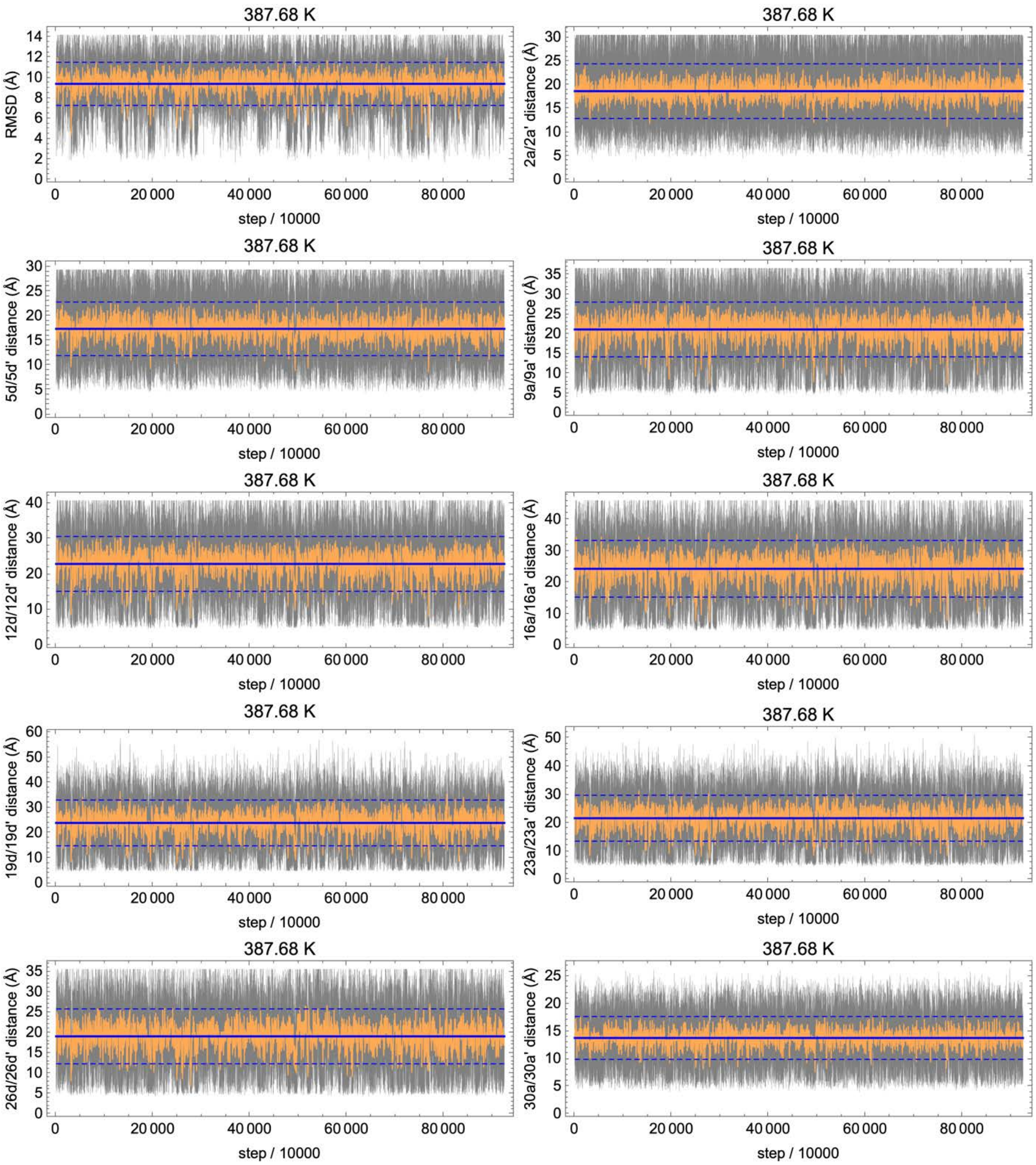

Measure at 387.68 K	Average (Å)	Standard Deviation (Å)	Slope (Å / μs)
RMSD	9.40219	2.11744	−0.0109965
5d/5d' distance	17.3834	5.4478	−0.218283
12d/12d' distance	22.8631	7.71804	−0.254064
19d/19d' distance	23.9538	9.09477	−0.117555
26d/26d' distance	19.0696	6.75376	−0.0607882
2a/2a' distance	18.6638	5.76742	0.0370658
9a/9a' distance	21.1271	6.93532	−0.173374
16a/16a' distance	24.3116	9.01522	−0.16502
23a/23a' distance	21.6668	8.11449	−0.0530986
30a/30a' distance	13.7847	3.88955	−0.00125885
Core Distance	20.3138	7.85203	

Figure S155. Plots of RMSD and residue packing distances for T-REMD simulations of variant **s6e/1g'-x4x** at 387.68 K.

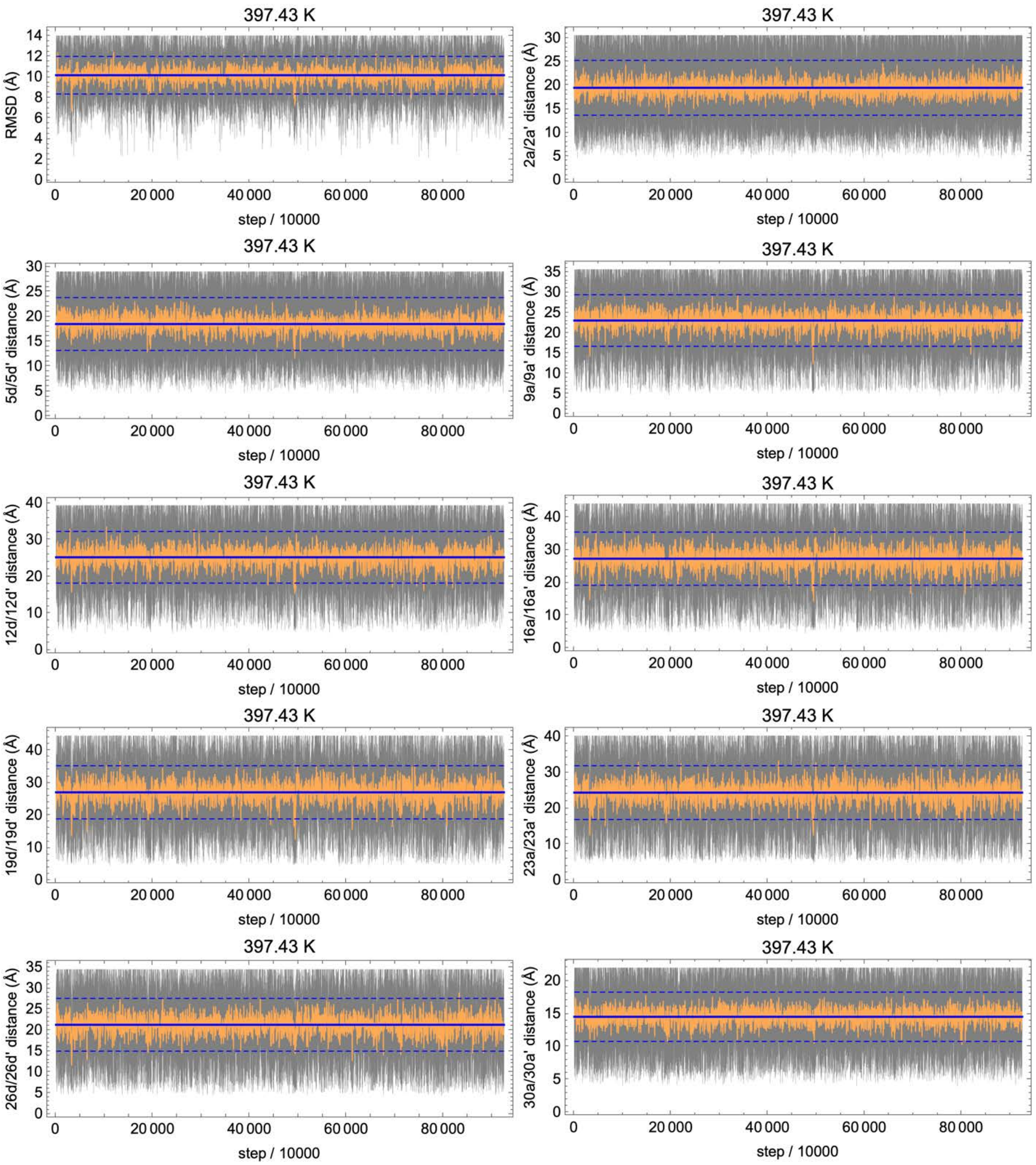

Measure at 397.43 K	Average (Å)	Standard Deviation (Å)	Slope (Å / μs)
RMSD	10.1906	1.826	−0.0274775
5d/5d' distance	18.5282	5.30558	−0.253451
12d/12d' distance	25.2813	7.03839	−0.305962
19d/19d' distance	27.0958	8.18226	−0.13009
26d/26d' distance	21.2975	6.29659	−0.0328633
2a/2a' distance	19.4929	5.80489	0.0643195
9a/9a' distance	23.0793	6.36066	−0.211989
16a/16a' distance	27.3452	8.11529	−0.203033
23a/23a' distance	24.4223	7.48462	−0.0902457
30a/30a' distance	14.5505	3.75289	0.030257
Core Distance	22.3437	7.74596	

Figure S156. Plots of RMSD and residue packing distances for T-REMD simulations of variant **s6e/1g'-x4x** at 397.43 K.

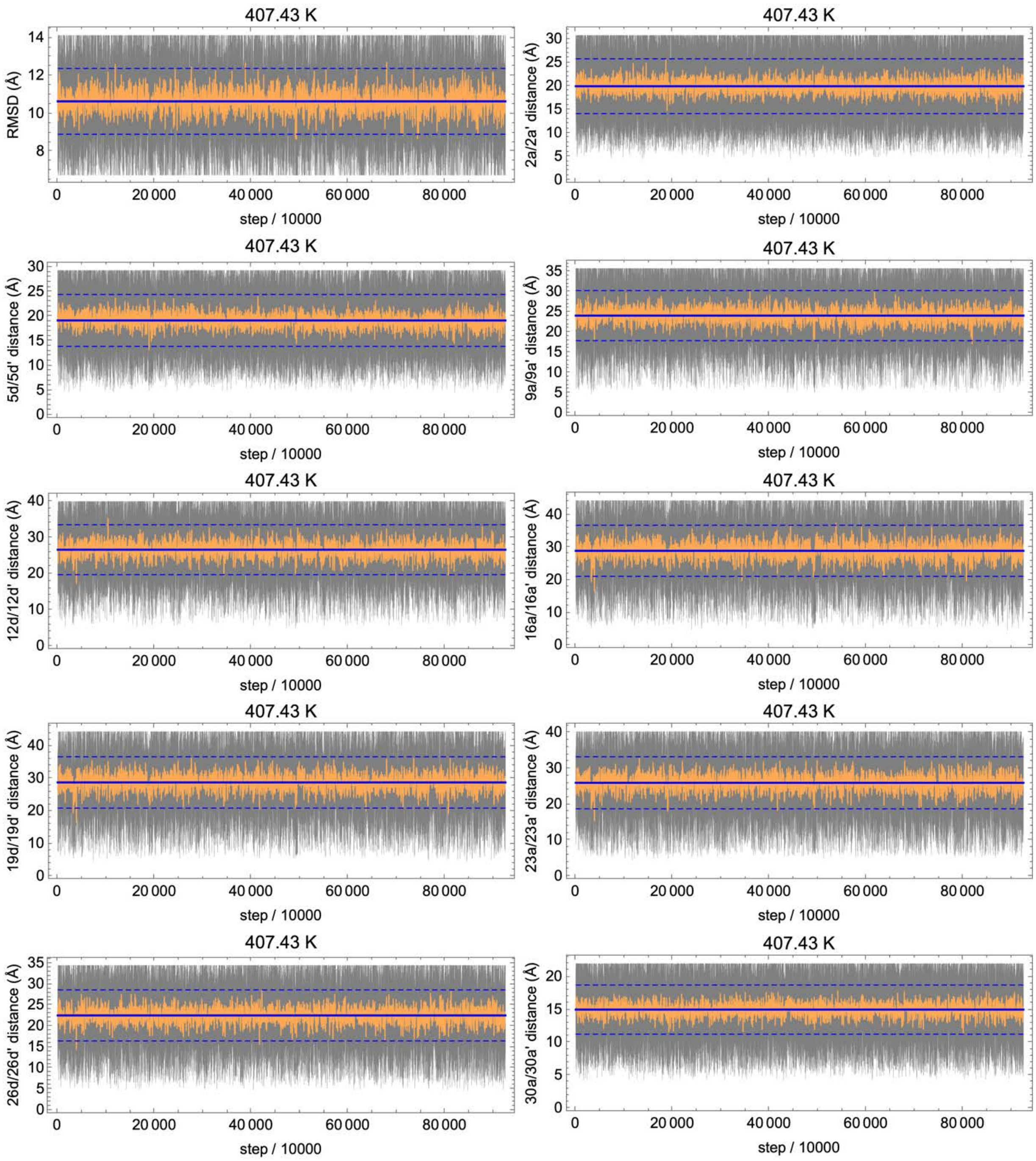

Measure at 407.43 K	Average (Å)	Standard Deviation (Å)	Slope (Å / μs)
RMSD	10.6432	1.73834	−0.0259139
5d/5d' distance	19.1593	5.26534	−0.349708
12d/12d' distance	26.6031	6.88811	−0.200184
19d/19d' distance	28.7587	7.87546	−0.0785833
26d/26d' distance	22.5424	6.08721	−0.0297495
2a/2a' distance	19.9476	5.83533	−0.0701375
9a/9a' distance	24.0779	6.18846	−0.216148
16a/16a' distance	28.938	7.83975	−0.112608
23a/23a' distance	25.9923	7.24881	−0.082964
30a/30a' distance	14.9916	3.736	0.0169724
Core Distance	23.4457	7.83654	

Figure S157. Plots of RMSD and residue packing distances for T-REMD simulations of variant **s6e/1g'-x4x** at 407.43 K.

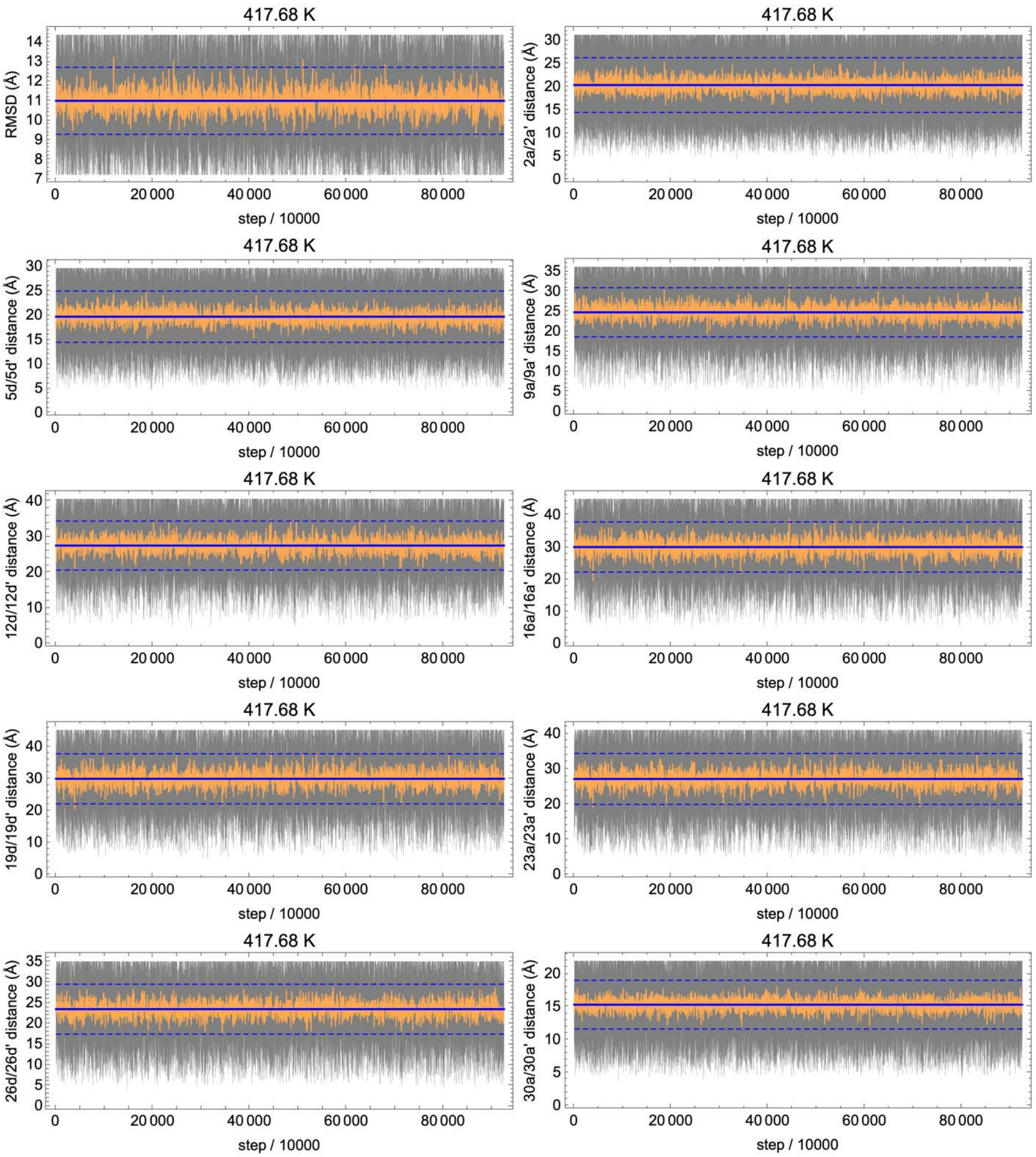

Measure at 417.68 K	Average (Å)	Standard Deviation (Å)	Slope (Å / μs)
RMSD	10.9986	1.71234	−0.0174513
5d/5d' distance	19.7157	5.25868	−0.345129
12d/12d' distance	27.5377	6.88342	−0.199989
19d/19d' distance	29.9631	7.82191	0.0482364
26d/26d' distance	23.4843	6.05245	0.0826202
2a/2a' distance	20.3712	5.89716	−0.0834697
9a/9a' distance	24.7762	6.13978	−0.268047
16a/16a' distance	30.0503	7.80654	−0.00267924
23a/23a' distance	27.1438	7.22557	0.031572
30a/30a' distance	15.327	3.69932	−0.00669343
Core Distance	24.2633	7.98407	

Figure S158. Plots of RMSD and residue packing distances for T-REMD simulations of variant **s6e/1g'-x4x** at 417.68 K.

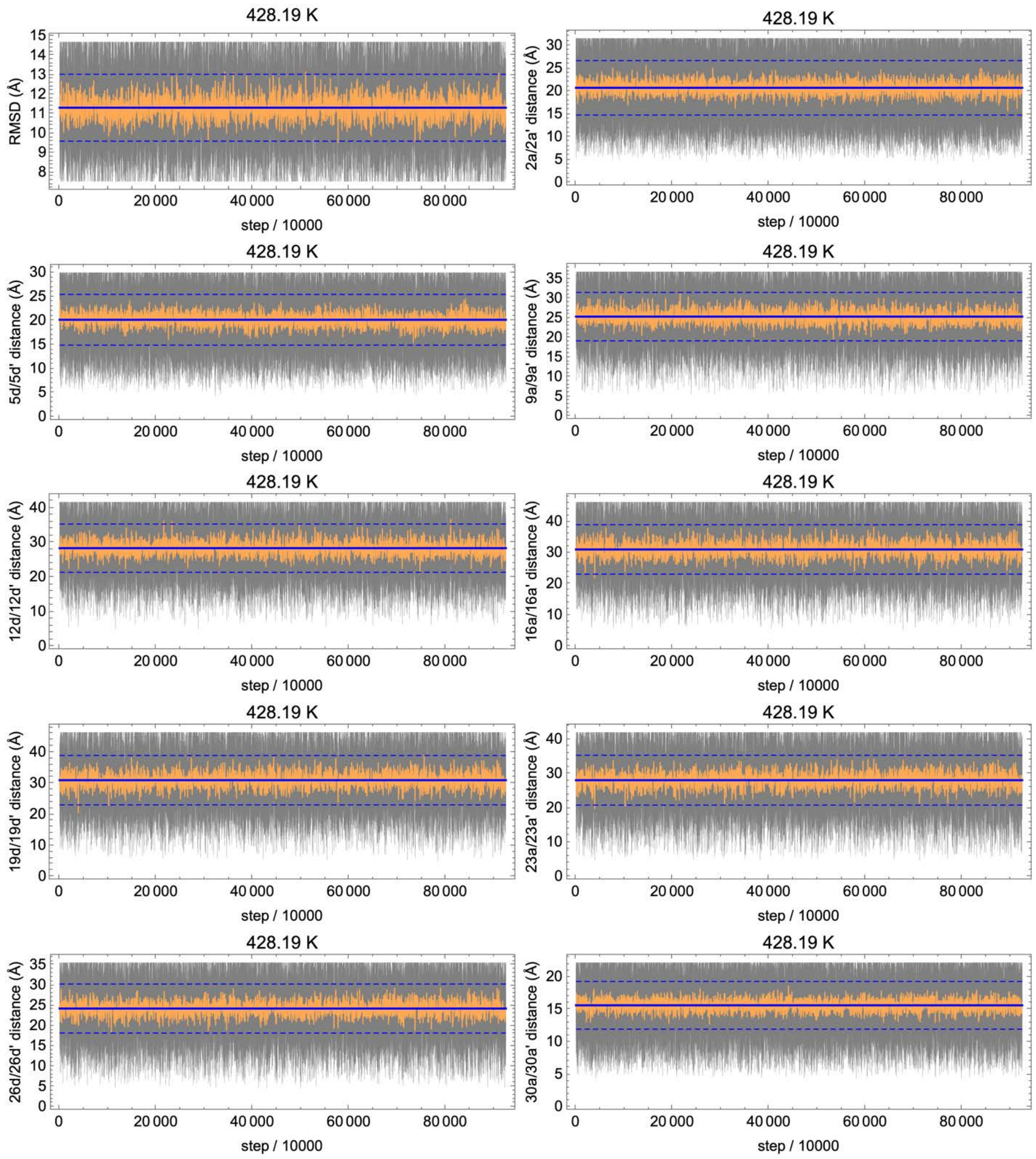

Measure at 428.19 K	Average (Å)	Standard Deviation (Å)	Slope (Å / μs)
RMSD	11.3147	1.71647	0.00393728
5d/5d' distance	20.1677	5.27065	−0.318916
12d/12d' distance	28.353	6.97426	−0.118611
19d/19d' distance	30.9811	7.94255	0.146235
26d/26d' distance	24.2296	6.04327	0.112114
2a/2a' distance	20.7298	5.95982	−0.119171
9a/9a' distance	25.3621	6.18482	−0.216577
16a/16a' distance	31.0147	7.9139	0.0275397
23a/23a' distance	28.0956	7.26475	0.128407
30a/30a' distance	15.6264	3.67563	0.00601255
Core Distance	24.9511	8.1761	

Figure S159. Plots of RMSD and residue packing distances for T-REMD simulations of variant **s6e/1g'-x4x** at 428.19 K.

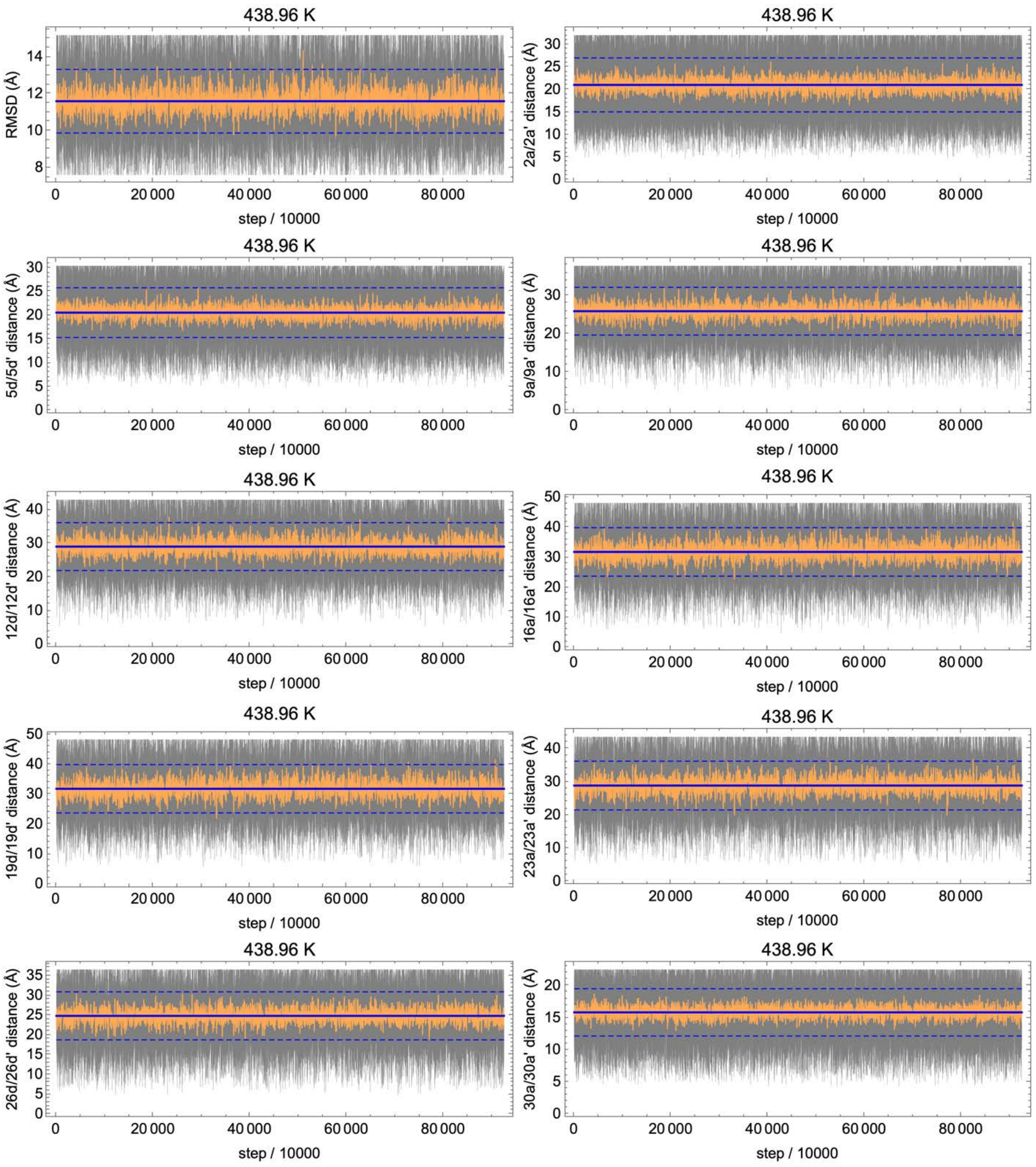

Measure at 438.96 K	Average (Å)	Standard Deviation (Å)	Slope (Å / μs)
RMSD	11.5964	1.72232	0.0199352
5d/5d' distance	20.5632	5.23461	−0.254598
12d/12d' distance	29.0423	7.11962	0.00526379
19d/19d' distance	31.8143	8.07399	0.256888
26d/26d' distance	24.8148	6.06031	0.126202
2a/2a' distance	21.037	5.99209	−0.0235466
9a/9a' distance	25.8959	6.23001	−0.105053
16a/16a' distance	31.8087	8.06796	0.191951
23a/23a' distance	28.8743	7.36222	0.182745
30a/30a' distance	15.844	3.68055	−0.0163365
Core Distance	25.5216	8.36206	

Figure S160. Plots of RMSD and residue packing distances for T-REMD simulations of variant **s6e/1g'-x4x** at 438.96 K.

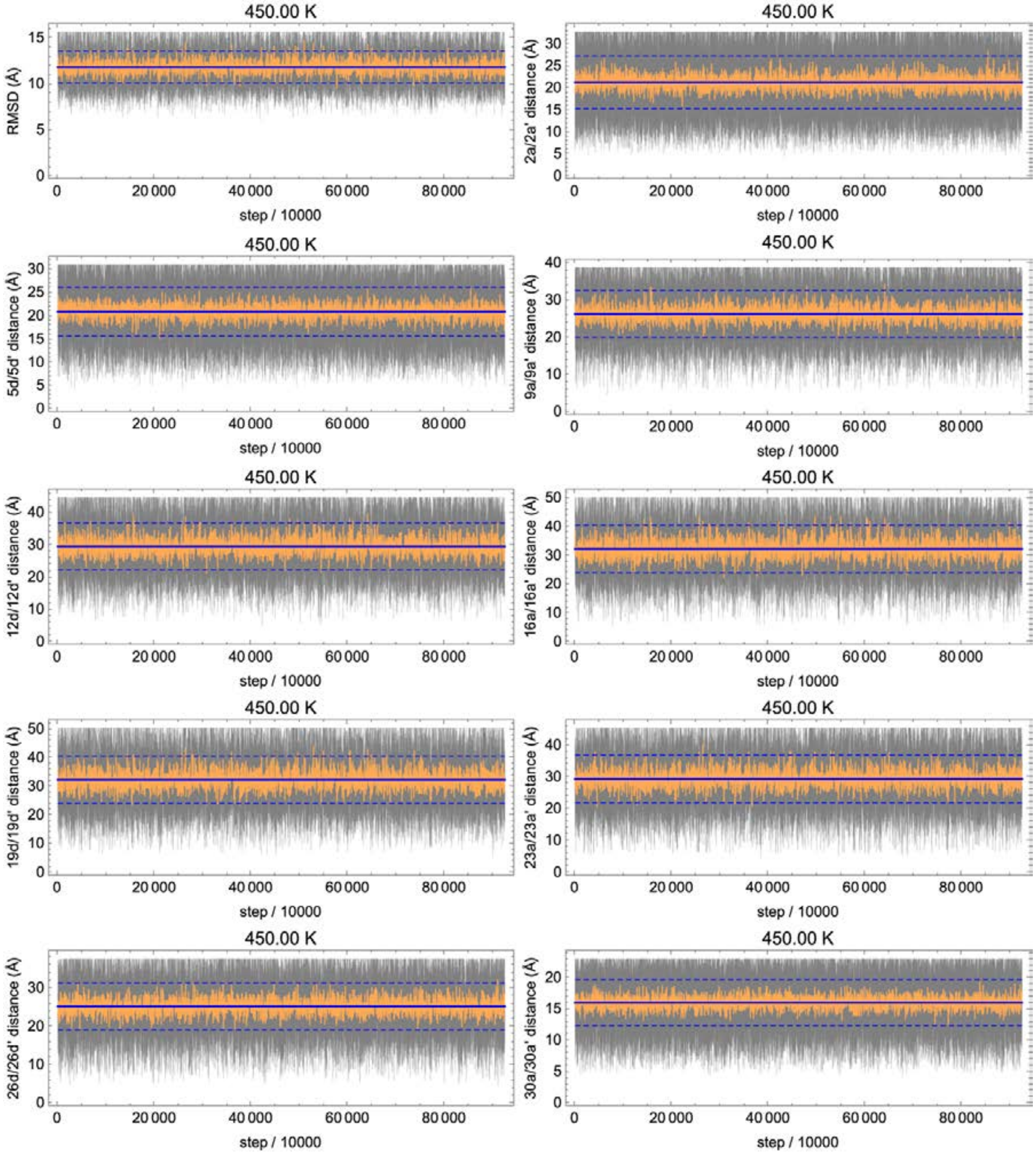

Measure at 450.00 K	Average (Å)	Standard Deviation (Å)	Slope (Å / μs)
RMSD	11.8299	1.73322	−0.00340833
5d/5d' distance	20.9285	5.25306	−0.213333
12d/12d' distance	29.5884	7.29364	−0.0195633
19d/19d' distance	32.3616	8.25603	0.116203
26d/26d' distance	25.2602	6.07288	0.103084
2a/2a' distance	21.3197	6.00595	−0.0758977
9a/9a' distance	26.3194	6.28587	−0.113507
16a/16a' distance	32.3477	8.26461	0.142056
23a/23a' distance	29.4395	7.47352	0.143587
30a/30a' distance	16.0425	3.67504	0.000280181
Core Distance	25.9564	8.51114	

Figure S161. Plots of RMSD and residue packing distances for T-REMD simulations of variant **s6e/1g’-x4x** at 450.00 K.

(return to Table of Contents)

13e/8g'-xx

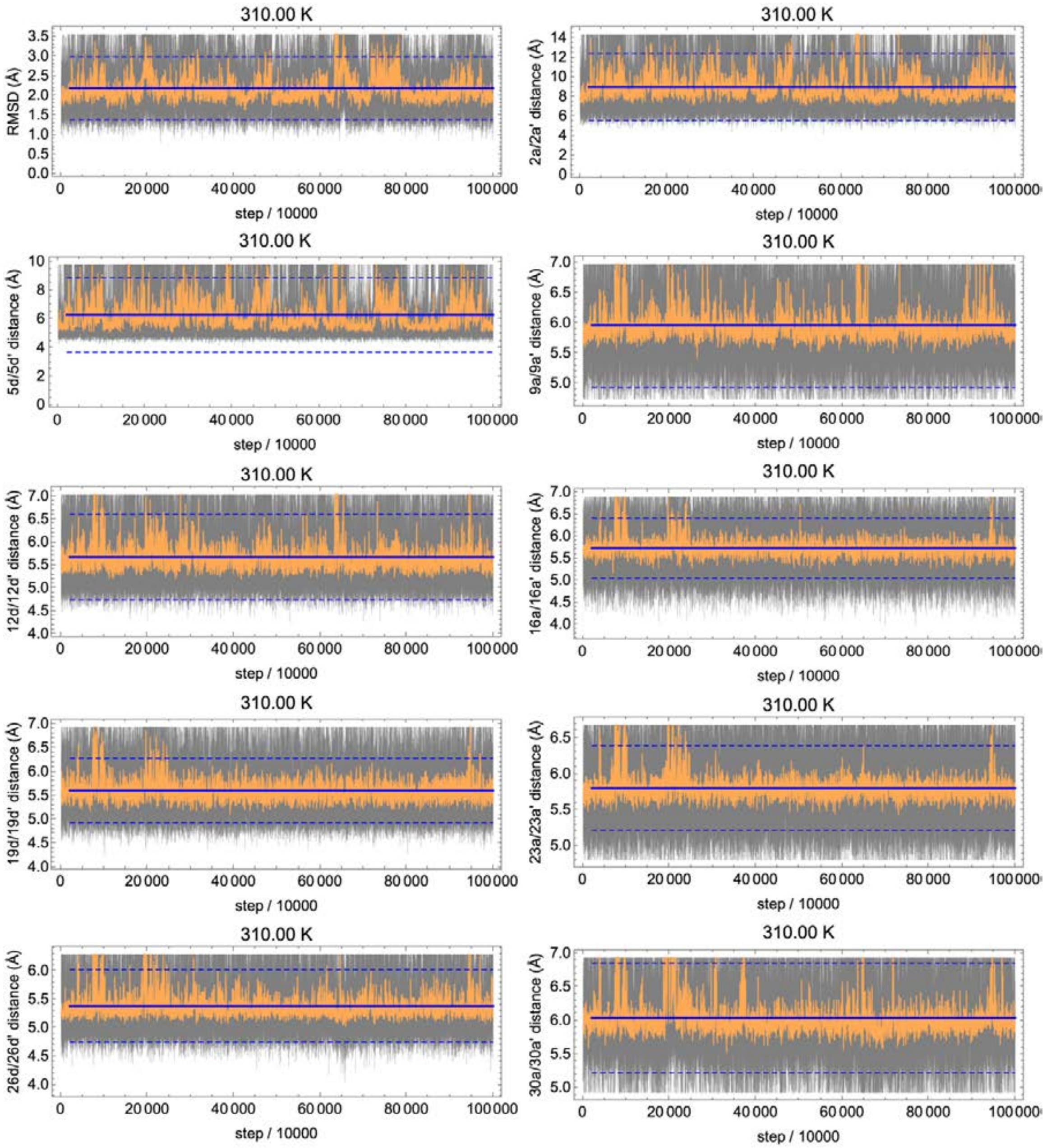

Measure at 310.00 K	Average (Å)	Standard Deviation (Å)	Slope (Å / μs)
RMSD	2.18444	0.802786	−0.0177865
5d/5d' distance	6.28637	2.60603	−0.156093
12d/12d' distance	5.67592	0.928358	−0.0571861
19d/19d' distance	5.61131	0.674258	−0.0442688
26d/26d' distance	5.38709	0.634156	−0.0388117
2a/2a' distance	8.99528	3.44713	−0.246575
9a/9a' distance	5.966	1.03749	−0.0273257
16a/16a' distance	5.73774	0.683255	−0.0448109
23a/23a' distance	5.8052	0.586862	−0.0327383
30a/30a' distance	6.03998	0.816922	−0.0917903
Core Distance	6.16721	1.90003	

Figure S162. Plots of RMSD and residue packing distances for T-REMD simulations of variant **13e/8g'-xx** at 310.00 K.

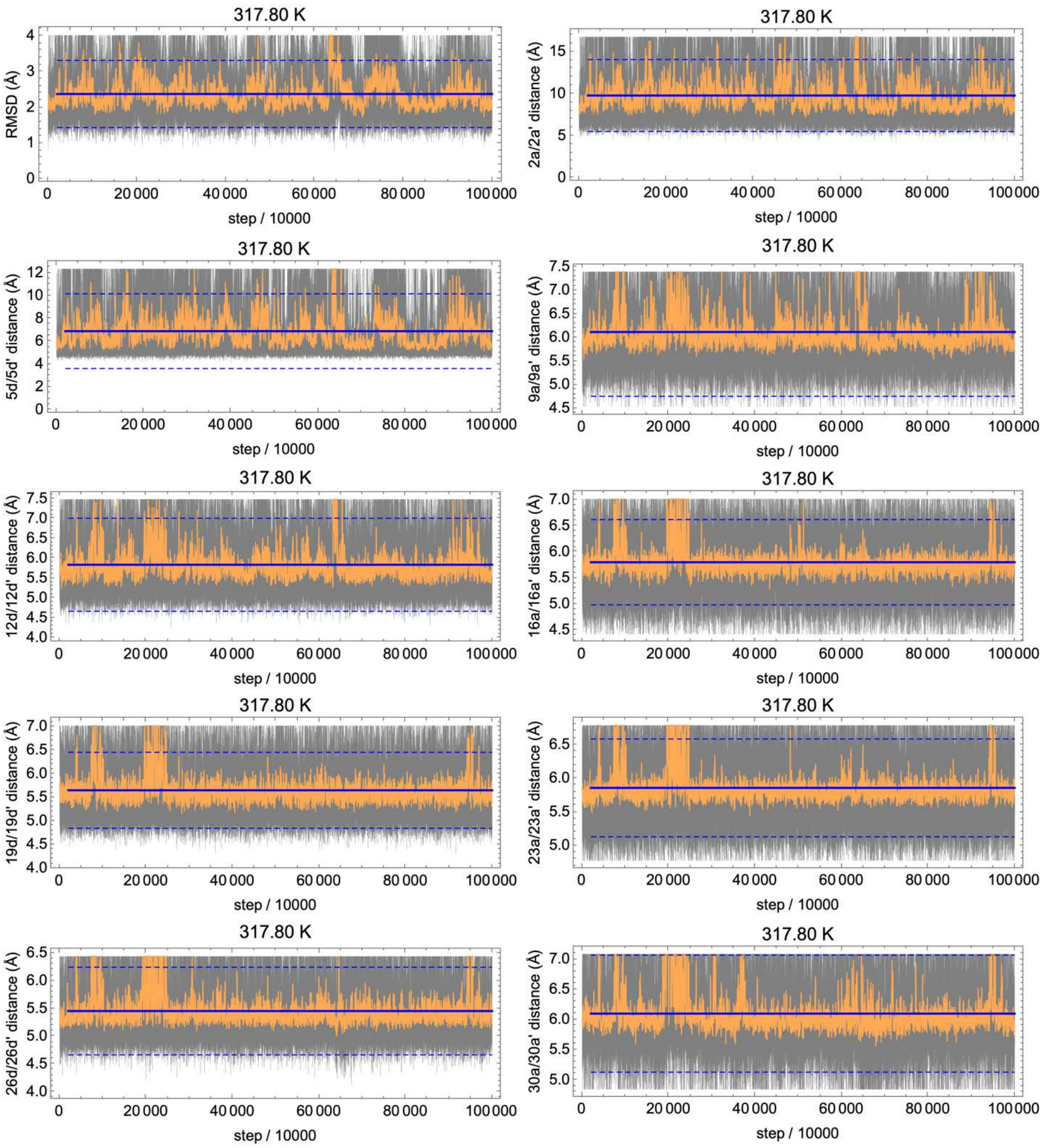

Measure at 317.80 K	Average (Å)	Standard Deviation (Å)	Slope (Å / μs)
RMSD	2.37192	0.938951	−0.0388526
5d/5d' distance	6.88254	3.27362	−0.212439
12d/12d' distance	5.83493	1.17213	−0.0824134
19d/19d' distance	5.64788	0.804407	−0.0736368
26d/26d' distance	5.45458	0.789366	−0.0771494
2a/2a' distance	9.77365	4.27655	−0.326064
9a/9a' distance	6.11474	1.36324	−0.0467091
16a/16a' distance	5.80071	0.818379	−0.0800893
23a/23a' distance	5.85575	0.72712	−0.0655699
30a/30a' distance	6.09713	0.969418	−0.122851
Core Distance	6.38466	2.35385	

Figure S163. Plots of RMSD and residue packing distances for T-REMD simulations of variant **13e/8g'-xx** at 317.80 K.

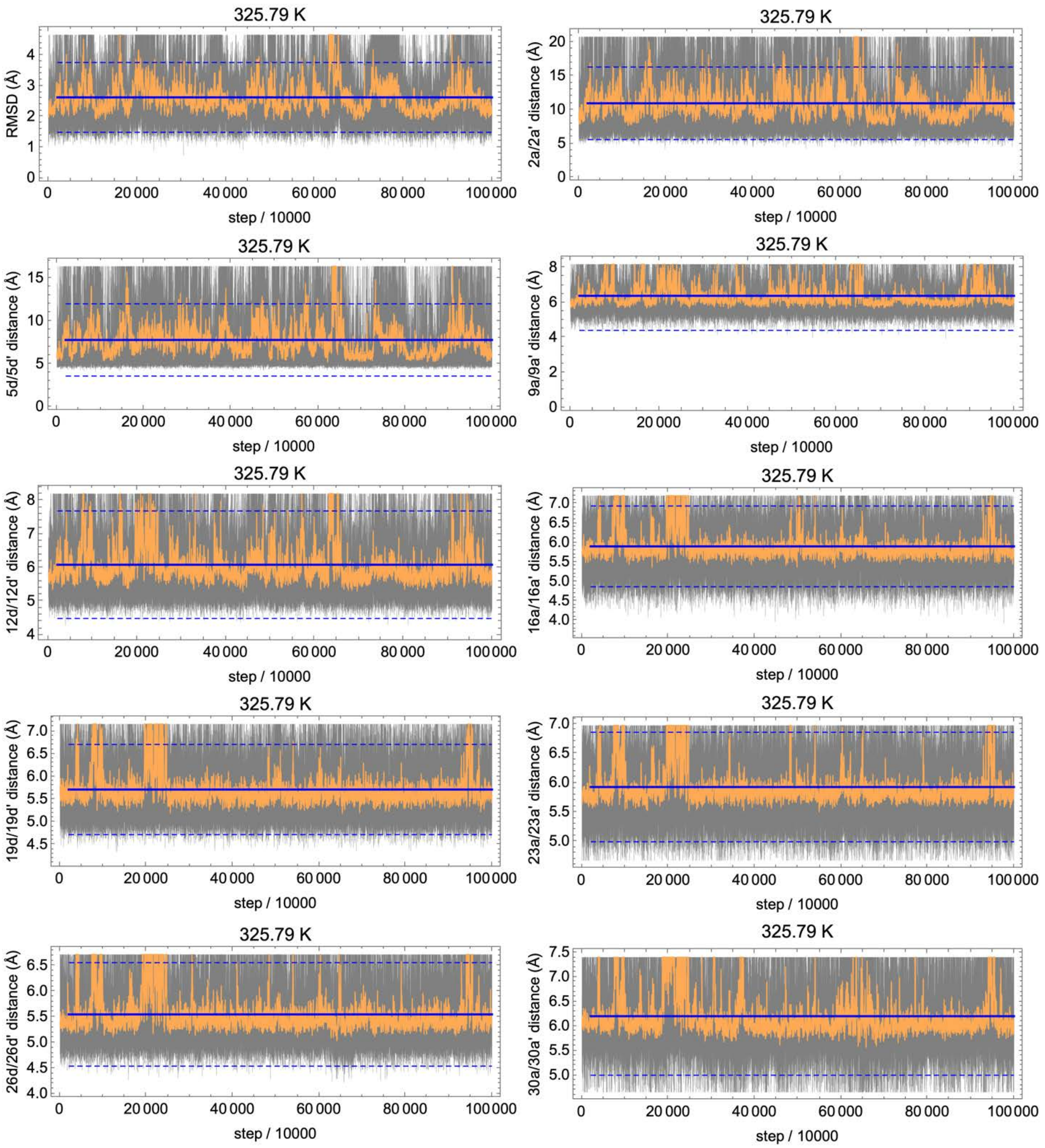

Measure at 325.79 K	Average (Å)	Standard Deviation (Å)	Slope (Å / μs)
RMSD	2.62674	1.13595	−0.0710318
5d/5d' distance	7.79409	4.21052	−0.327039
12d/12d' distance	6.08692	1.60133	−0.1357
19d/19d' distance	5.71306	0.993798	−0.116829
26d/26d' distance	5.54963	1.00963	−0.129304
2a/2a' distance	10.9502	5.36308	−0.455076
9a/9a' distance	6.38319	1.97422	−0.0689149
16a/16a' distance	5.89992	1.03668	−0.129496
23a/23a' distance	5.92634	0.933449	−0.114241
30a/30a' distance	6.20113	1.20181	−0.170014
Core Distance	6.72272	3.01602	

Figure S164. Plots of RMSD and residue packing distances for T-REMD simulations of variant **13e/8g'-xx** at 325.79 K.

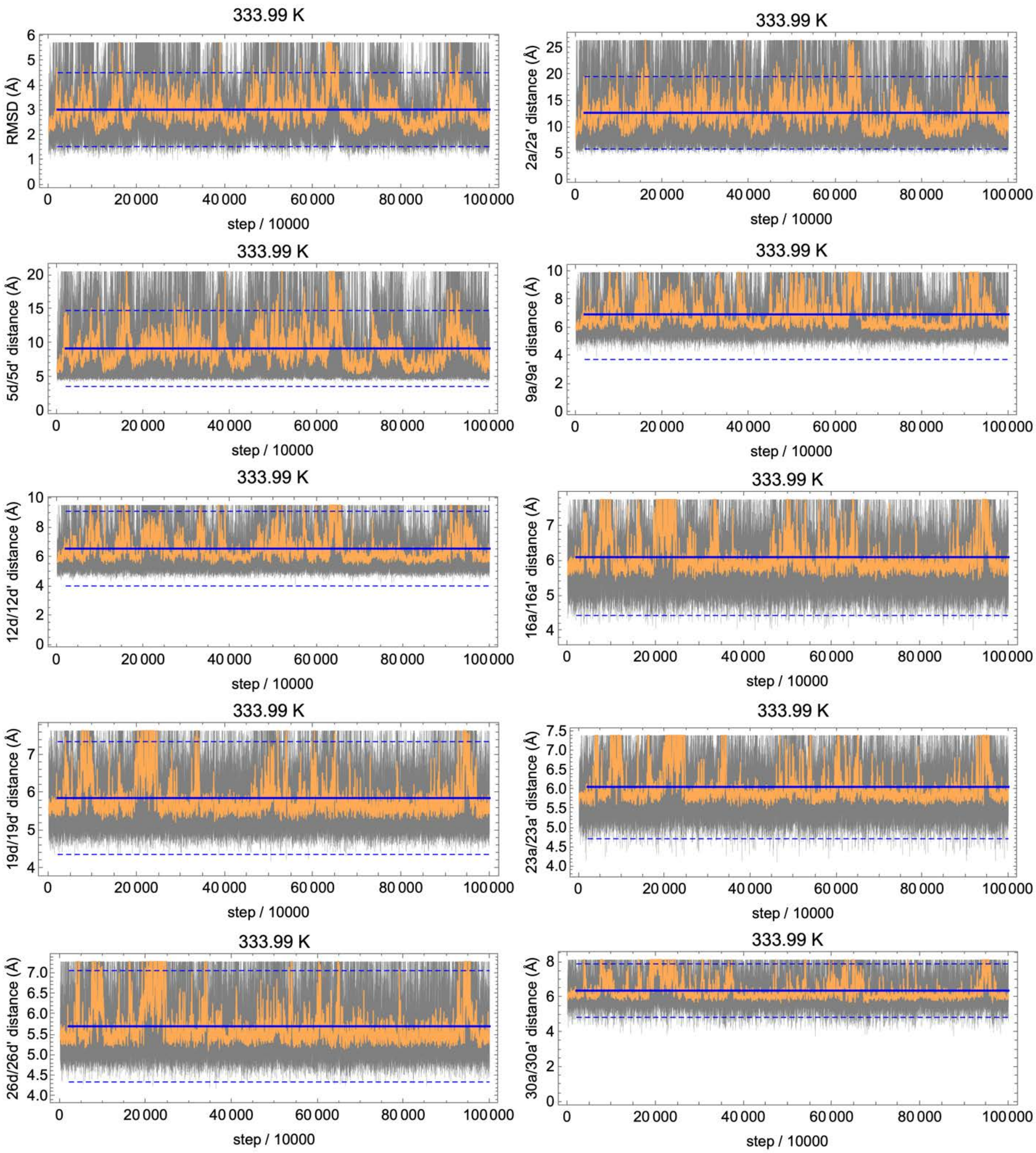

Measure at 333.99 K	Average (Å)	Standard Deviation (Å)	Slope (Å / μs)
RMSD	3.03052	1.48914	−0.0936097
5d/5d' distance	9.21252	5.59147	−0.394823
12d/12d' distance	6.5638	2.54871	−0.171523
19d/19d' distance	5.86031	1.49202	−0.152006
26d/26d' distance	5.70593	1.35839	−0.174695
2a/2a' distance	12.7307	6.86835	−0.564072
9a/9a' distance	6.94125	3.20552	−0.0870936
16a/16a' distance	6.10534	1.6667	−0.158907
23a/23a' distance	6.06649	1.34491	−0.152468
30a/30a' distance	6.37783	1.53099	−0.200432
Core Distance	7.2849	4.06123	

Figure S165. Plots of RMSD and residue packing distances for T-REMD simulations of variant **13e/8g’-xx** at 333.99 K.

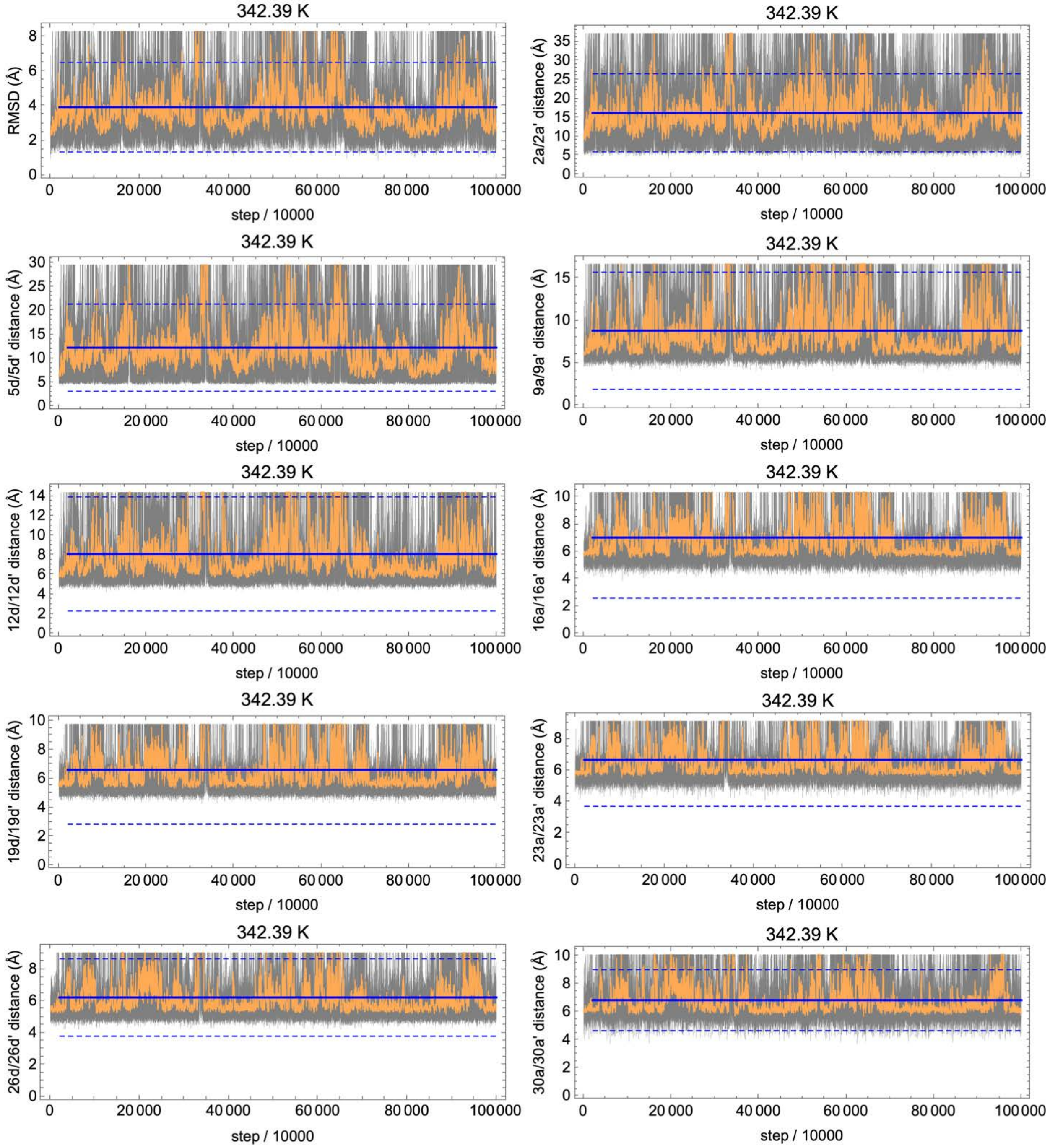

Measure at 342.39 K	Average (Å)	Standard Deviation (Å)	Slope (Å / μs)
RMSD	3.92388	2.58528	−0.121724
5d/5d' distance	12.2339	9.1162	−0.462113
12d/12d' distance	8.11165	5.80771	−0.239899
19d/19d' distance	6.59601	3.7507	−0.19083
26d/26d' distance	6.21827	2.42869	−0.229825
2a/2a' distance	16.2208	10.275	−0.596547
9a/9a' distance	8.78853	6.92182	−0.115379
16a/16a' distance	7.01371	4.42446	−0.196406
23a/23a' distance	6.64619	2.93115	−0.197574
30a/30a' distance	6.83411	2.18027	−0.230289
Core Distance	8.74035	6.78008	

Figure S166. Plots of RMSD and residue packing distances for T-REMD simulations of variant **13e/8g'-xx** at 342.39 K.

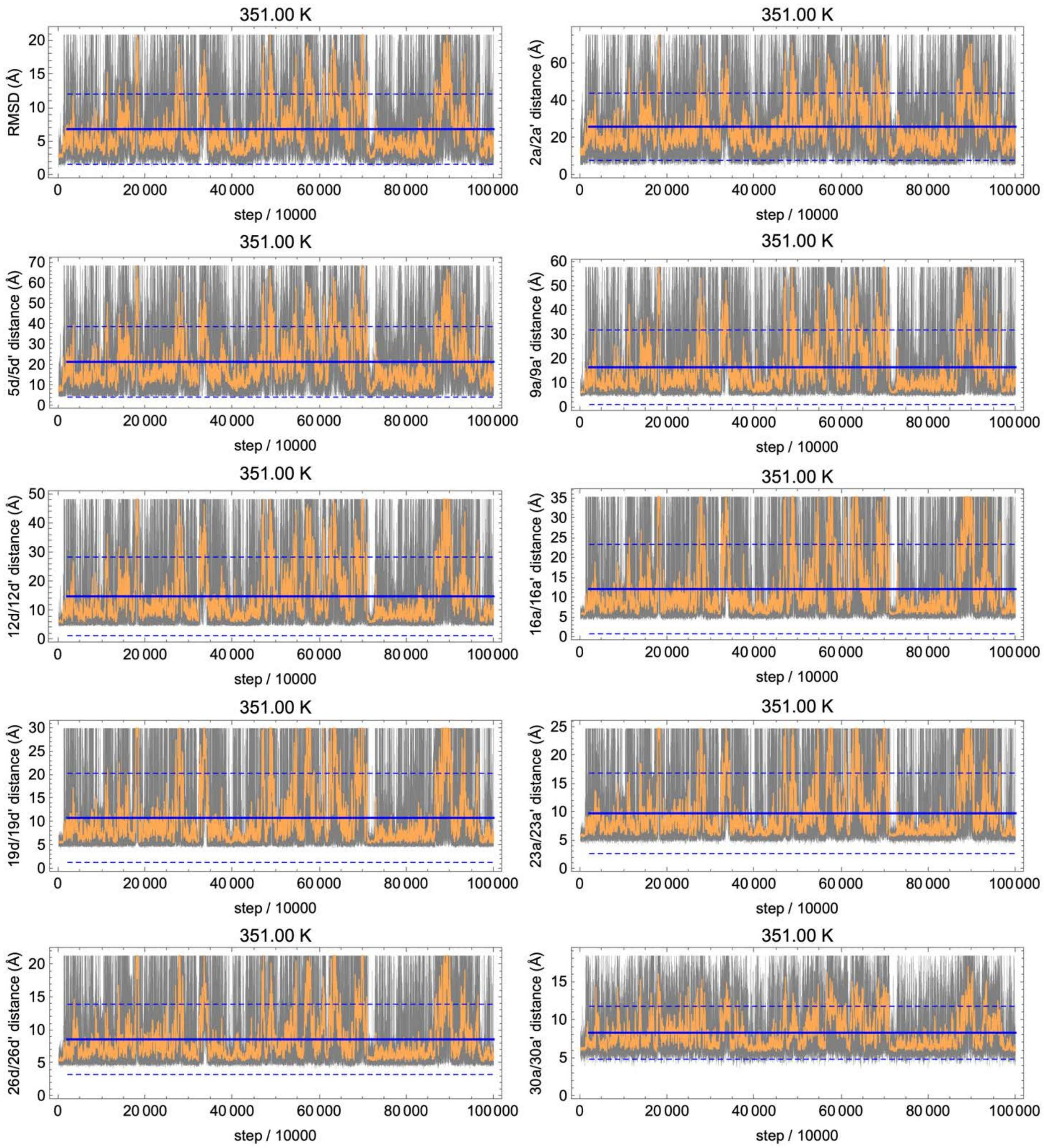

Measure at 351.00 K	Average (Å)	Standard Deviation (Å)	Slope (Å / μs)
RMSD	6.86062	5.22665	0.589057
5d/5d' distance	21.6454	17.2677	1.6657
12d/12d' distance	14.931	13.5457	1.63122
19d/19d' distance	10.8751	9.52462	1.13342
26d/26d' distance	8.63748	5.34495	0.436478
2a/2a' distance	26.0688	18.0549	1.62028
9a/9a' distance	16.6094	15.3593	1.87838
16a/16a' distance	12.1844	11.2981	1.40378
23a/23a' distance	9.82244	7.09729	0.74749
30a/30a' distance	8.34825	3.48583	0.180617
Core Distance	14.3469	13.5602	

Figure S167. Plots of RMSD and residue packing distances for T-REMD simulations of variant **13e/8g'-xx** at 351.00 K.

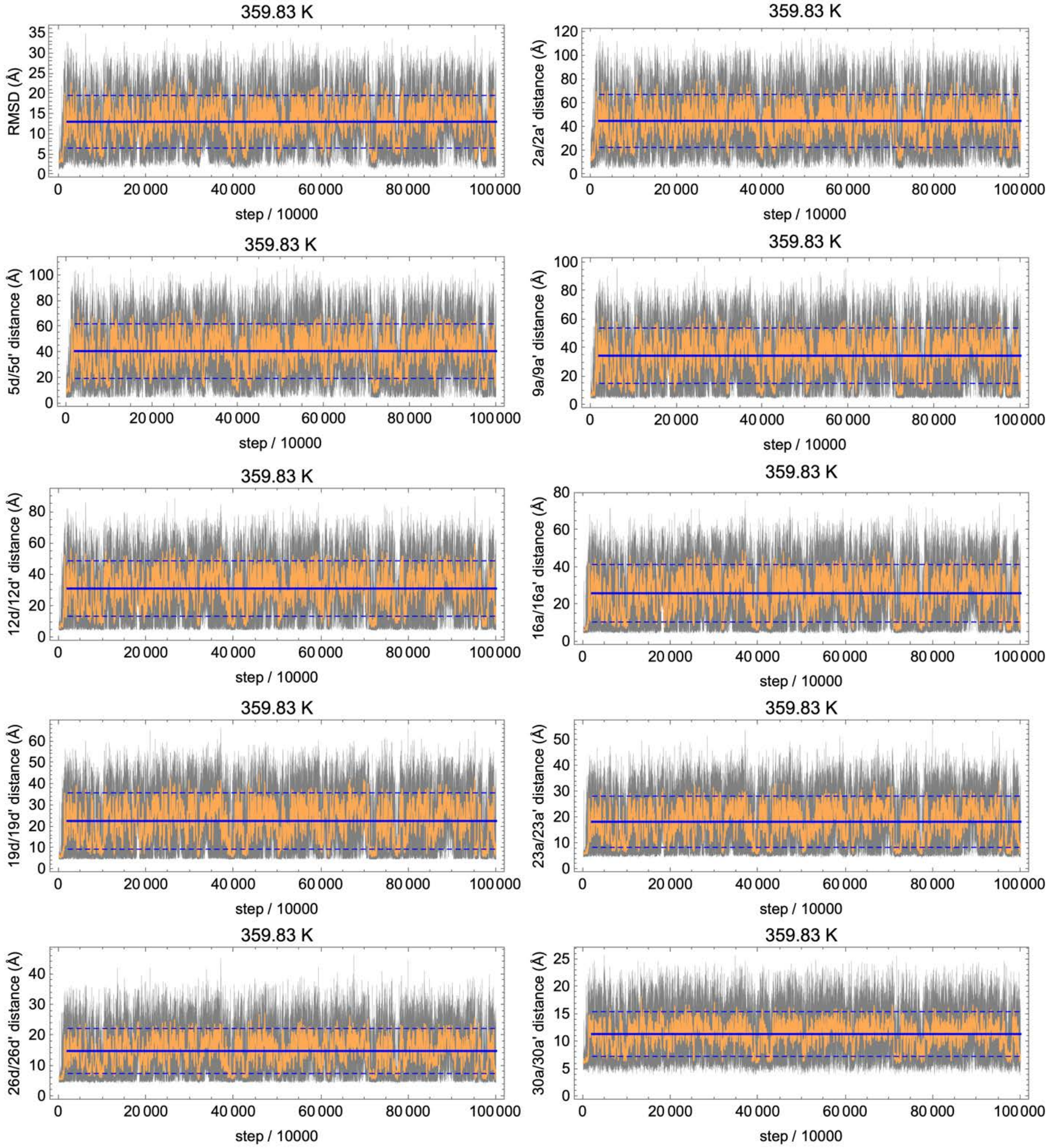

Measure at 359.83 K	Average (Å)	Standard Deviation (Å)	Slope (Å / μs)
RMSD	13.0992	6.50697	0.213865
5d/5d' distance	40.9419	21.308	0.447542
12d/12d' distance	31.2703	17.6455	0.520791
19d/19d' distance	22.6781	13.2423	0.48454
26d/26d' distance	14.9245	7.37063	0.148788
2a/2a' distance	45.2576	22.2834	0.507538
9a/9a' distance	34.6952	19.4426	0.576248
16a/16a' distance	26.0808	15.482	0.55952
23a/23a' distance	18.364	9.90833	0.319712
30a/30a' distance	11.3901	4.07604	−0.0227449
Core Distance	27.2892	19.1837	

Figure S168. Plots of RMSD and residue packing distances for T-REMD simulations of variant **13e/8g'-xx** at 359.83 K.

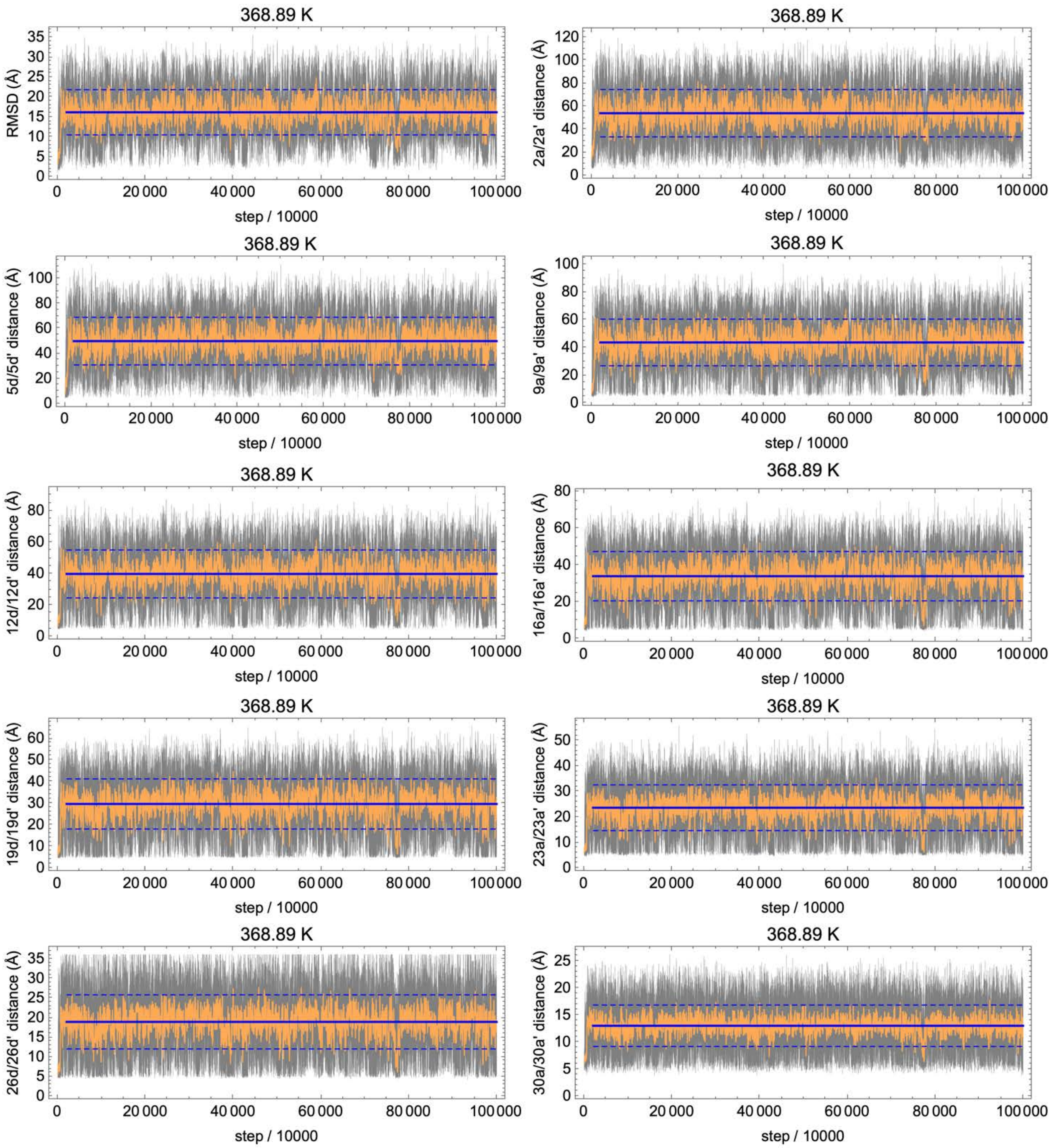

Measure at 368.89 K	Average (Å)	Standard Deviation (Å)	Slope (Å / μs)
RMSD	16.1729	5.67543	0.0151775
5d/5d' distance	49.9596	19.0373	−0.0644902
12d/12d' distance	39.8331	15.2628	−0.0153296
19d/19d' distance	29.693	11.6164	0.0618624
26d/26d' distance	18.9504	6.89206	0.00955178
2a/2a' distance	54.0027	20.5893	−0.0375387
9a/9a' distance	43.7068	16.7844	−0.0254666
16a/16a' distance	33.9832	13.3925	0.103041
23a/23a' distance	23.6472	8.96732	0.0489425
30a/30a' distance	13.0584	3.83824	−0.0503502
Core Distance	34.0927	19.2403	

Figure S169. Plots of RMSD and residue packing distances for T-REMD simulations of variant **13e/8g’-xx** at 368.89 K.

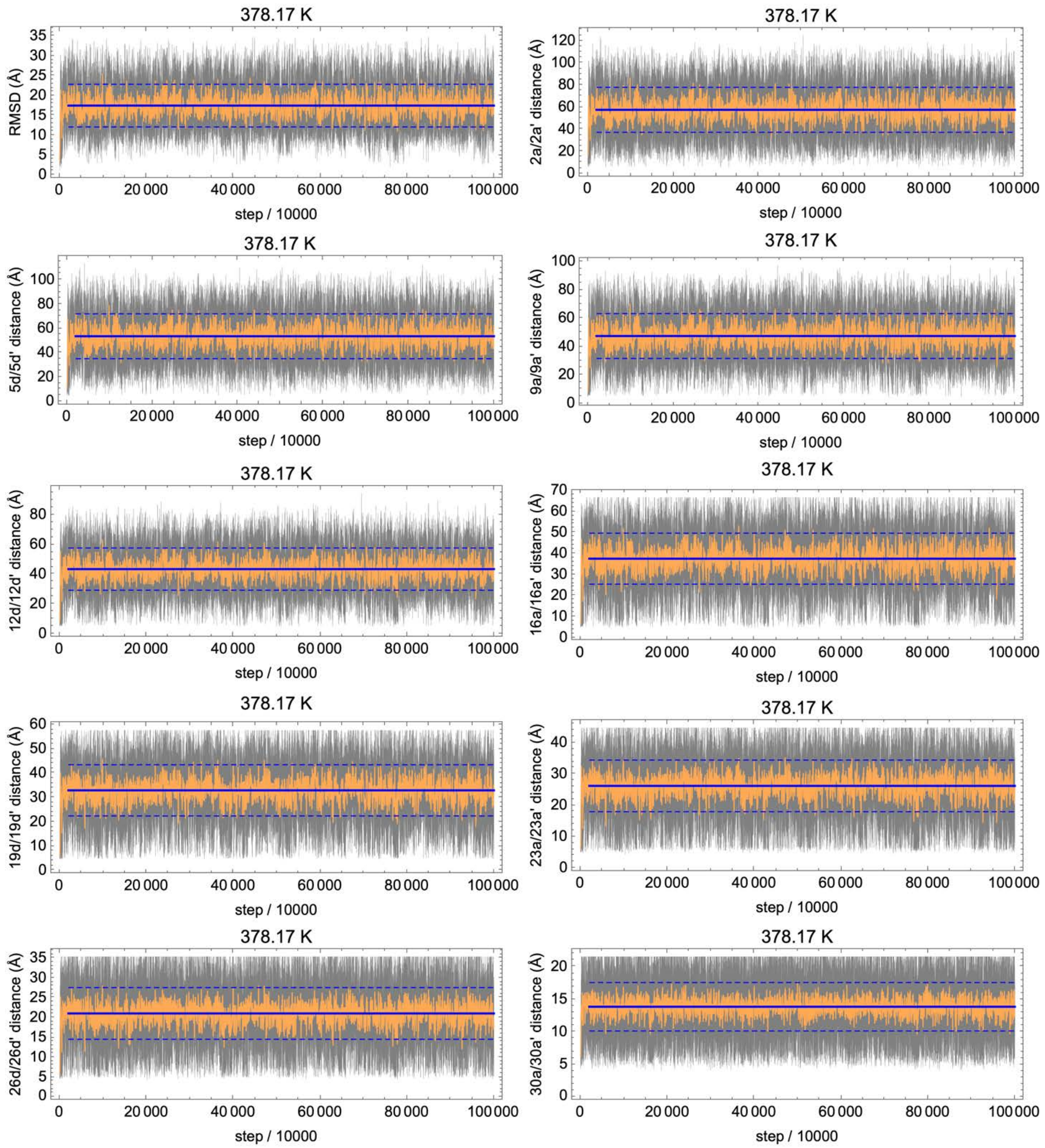

Measure at 378.17 K	Average (Å)	Standard Deviation (Å)	Slope (Å / μs)
RMSD	17.4494	5.38167	0.0224197
5d/5d' distance	53.479	18.4485	−0.0389061
12d/12d' distance	43.4064	14.1849	0.0333073
19d/19d' distance	32.919	10.5172	0.0766748
26d/26d' distance	21.0236	6.51757	0.0484797
2a/2a' distance	57.5183	20.3135	−0.015329
9a/9a' distance	47.3366	15.8126	0.0434015
16a/16a' distance	37.4589	12.1191	0.111559
23a/23a' distance	26.1852	8.23627	0.126345
30a/30a' distance	13.8412	3.72673	−0.0142602
Core Distance	37.0187	19.2981	

Figure S170. Plots of RMSD and residue packing distances for T-REMD simulations of variant **13e/8g'-xx** at 378.17 K.

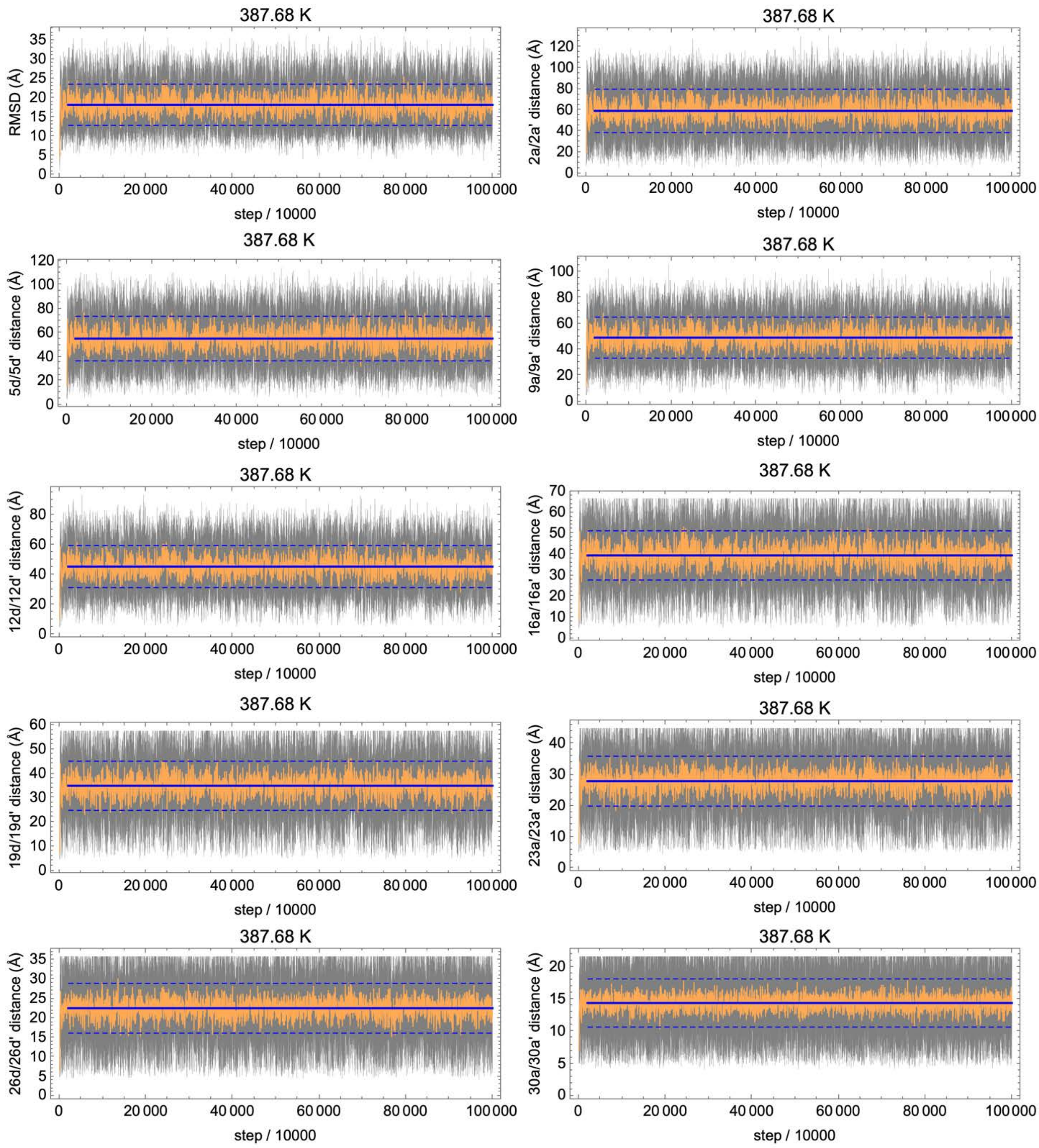

Measure at 387.68 K	Average (Å)	Standard Deviation (Å)	Slope (Å / μs)
RMSD	18.1708	5.3462	−0.0424071
5d/5d' distance	55.2158	18.602	−0.144659
12d/12d' distance	45.357	14.039	−0.0811688
19d/19d' distance	34.9335	10.1271	−0.0254859
26d/26d' distance	22.5169	6.37325	0.0450914
2a/2a' distance	59.2474	20.5959	−0.130744
9a/9a' distance	49.2205	15.7875	−0.0922634
16a/16a' distance	39.4466	11.7224	−0.0641791
23a/23a' distance	27.8976	8.01094	0.0223954
30a/30a' distance	14.3984	3.71632	−0.0316869
Core Distance	38.6926	19.4622	

Figure S171. Plots of RMSD and residue packing distances for T-REMD simulations of variant **13e/8g'-xx** at 387.68 K.

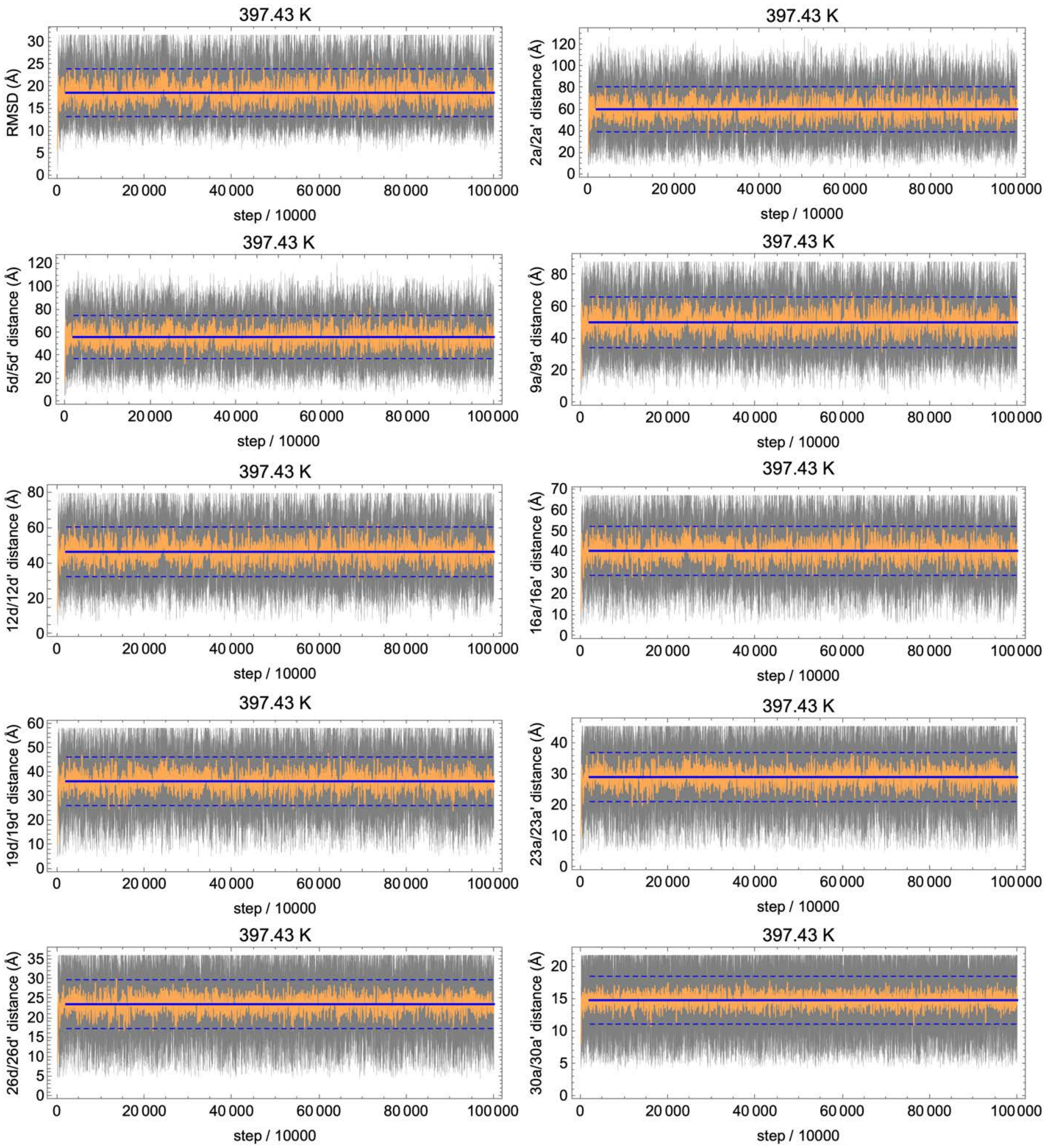

Measure at 397.43 K	Average (Å)	Standard Deviation (Å)	Slope (Å / μs)
RMSD	18.6367	5.35178	−0.0442413
5d/5d' distance	56.2657	18.7911	−0.108612
12d/12d' distance	46.6052	14.0678	−0.0988021
19d/19d' distance	36.2803	10.0492	−0.0366729
26d/26d' distance	23.5817	6.25933	0.0385938
2a/2a' distance	60.2543	20.8668	−0.0828387
9a/9a' distance	50.3752	15.885	−0.073822
16a/16a' distance	40.753	11.7006	−0.0558899
23a/23a' distance	29.1029	7.93791	0.0335332
30a/30a' distance	14.8294	3.69269	−0.00145032
Core Distance	39.7831	19.5914	

Figure S172. Plots of RMSD and residue packing distances for T-REMD simulations of variant **13e/8g'-xx** at 397.43 K.

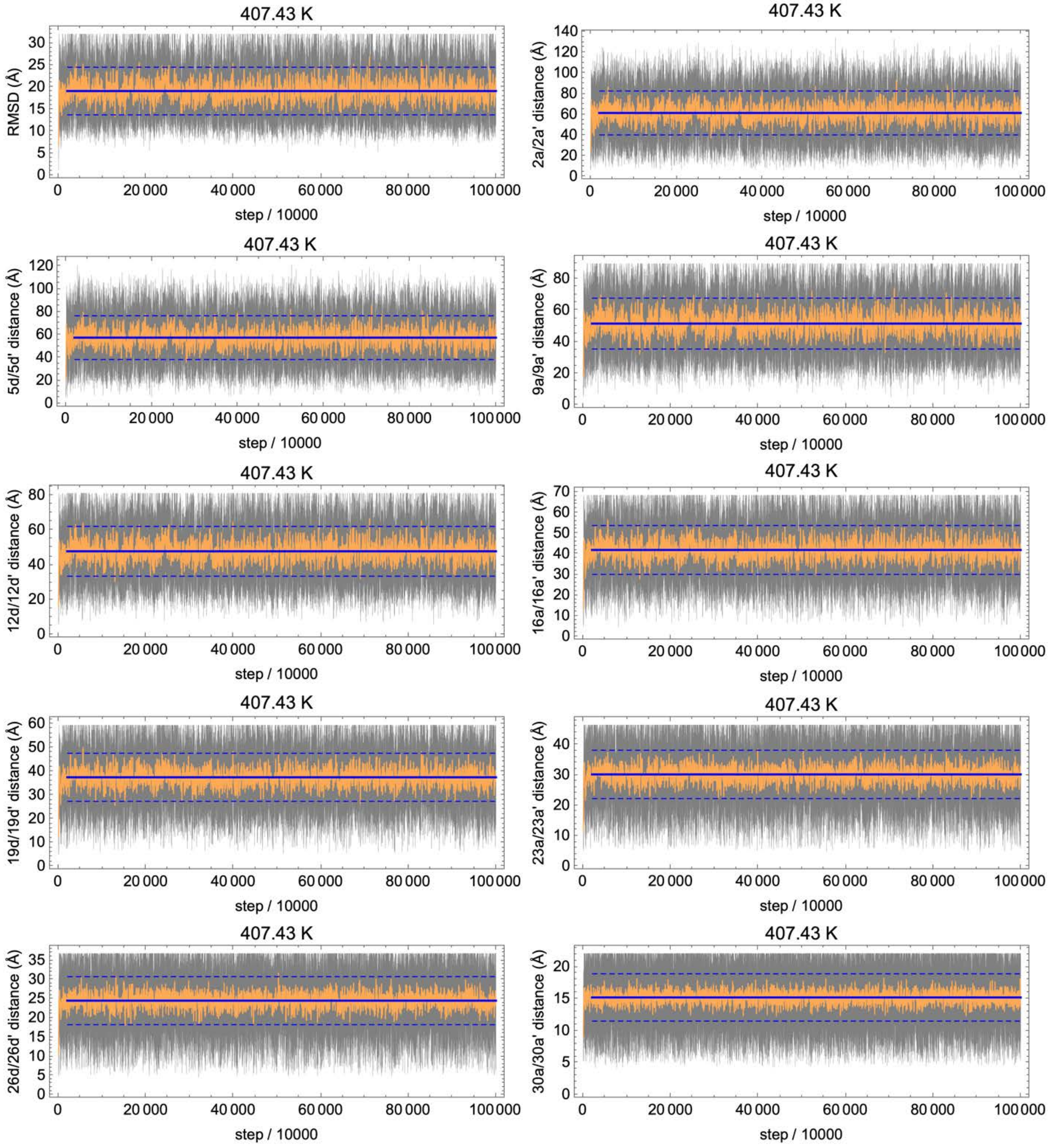

Measure at 407.43 K	Average (Å)	Standard Deviation (Å)	Slope (Å / μs)
RMSD	19.1167	5.40742	−0.0878383
5d/5d' distance	57.4133	19.1099	−0.233601
12d/12d' distance	47.7847	14.3069	−0.176848
19d/19d' distance	37.4724	10.121	−0.145946
26d/26d' distance	24.5107	6.25337	−0.0299976
2a/2a' distance	61.4642	21.2238	−0.262306
9a/9a' distance	51.5445	16.1345	−0.162667
16a/16a' distance	41.9181	11.8323	−0.164425
23a/23a' distance	30.1859	7.94439	−0.0939053
30a/30a' distance	15.2135	3.6954	−0.00346313
Core Distance	40.8342	19.8639	

Figure S173. Plots of RMSD and residue packing distances for T-REMD simulations of variant **13e/8g'-xx** at 407.43 K.

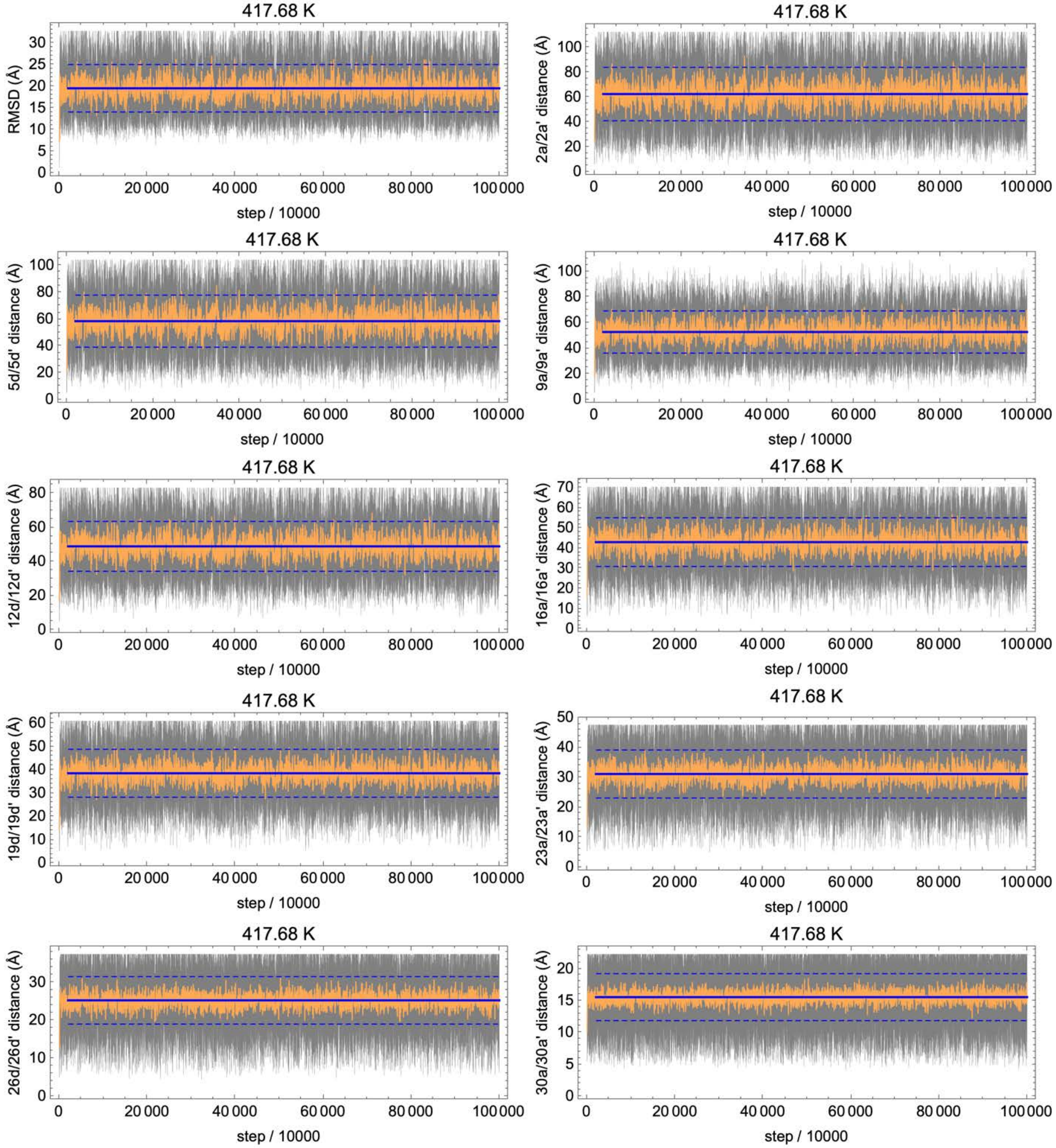

Measure at 417.68 K	Average (Å)	Standard Deviation (Å)	Slope (Å / μs)
RMSD	19.5583	5.47016	−0.0429575
5d/5d' distance	58.4706	19.4178	−0.0399442
12d/12d' distance	48.8812	14.5887	−0.11118
19d/19d' distance	38.5481	10.2789	−0.0216949
26d/26d' distance	25.2815	6.24898	0.0692717
2a/2a' distance	62.5544	21.5269	−0.0534872
9a/9a' distance	52.6647	16.4611	−0.110879
16a/16a' distance	43.009	12.0527	−0.0486292
23a/23a' distance	31.1357	8.02954	0.0592879
30a/30a' distance	15.5661	3.684	0.0779543
Core Distance	41.7902	20.1548	

Figure S174. Plots of RMSD and residue packing distances for T-REMD simulations of variant **13e/8g'-xx** at 417.68 K.

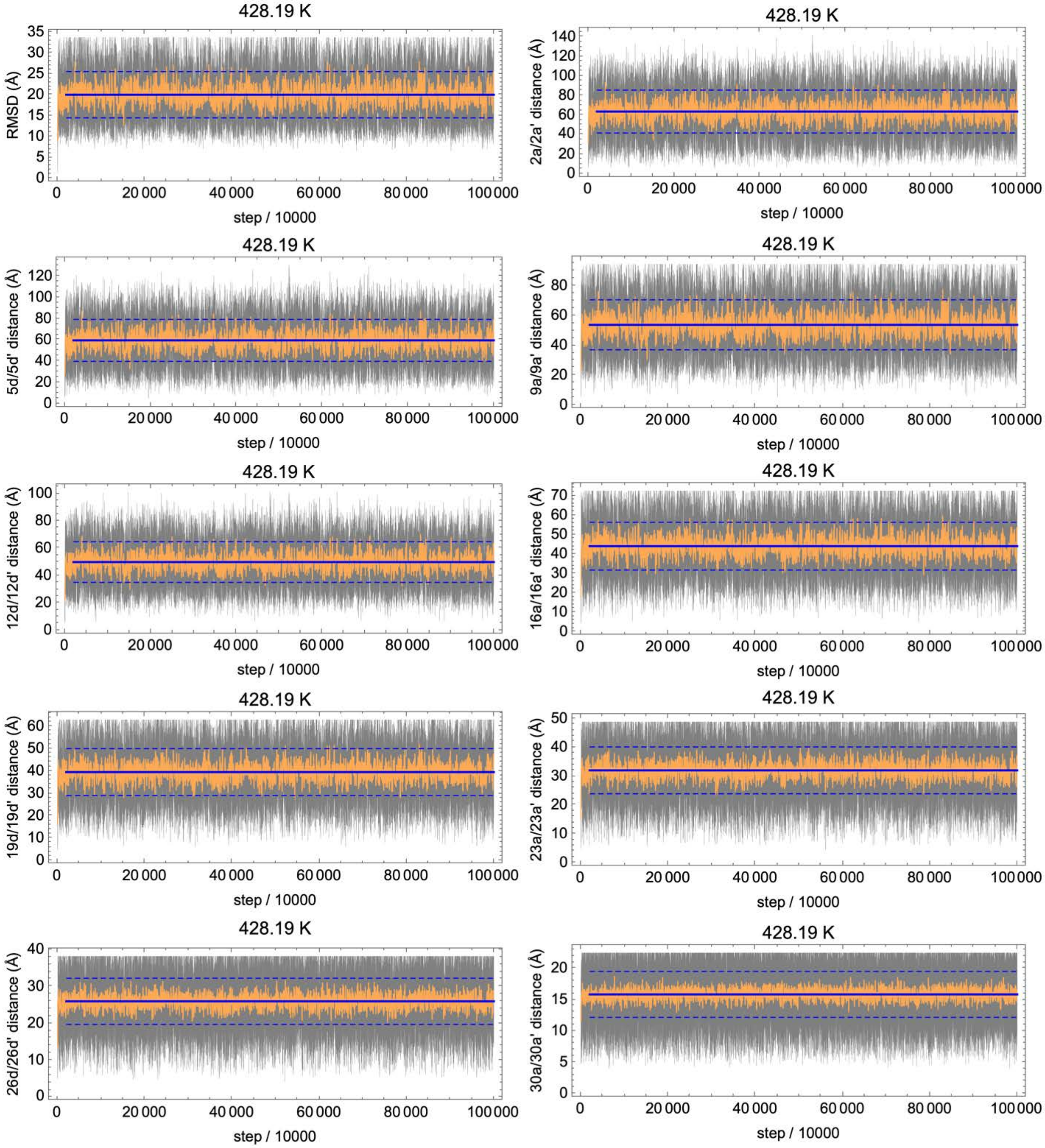

Measure at 428.19 K	Average (Å)	Standard Deviation (Å)	Slope (Å / μs)
RMSD	19.9761	5.5505	−0.0551199
5d/5d' distance	59.4729	19.7617	−0.132433
12d/12d' distance	49.8686	14.888	−0.119642
19d/19d' distance	39.4787	10.4897	0.0147336
26d/26d' distance	25.9469	6.25648	−0.00308558
2a/2a' distance	63.5167	21.9444	−0.136385
9a/9a' distance	53.6991	16.7648	−0.177479
16a/16a' distance	43.9924	12.3074	−0.0542319
23a/23a' distance	32.0023	8.12915	0.014678
30a/30a' distance	15.8256	3.6646	0.0526658
Core Distance	42.6448	20.4704	

Figure S175. Plots of RMSD and residue packing distances for T-REMD simulations of variant **13e/8g'-xx** at 428.19 K.

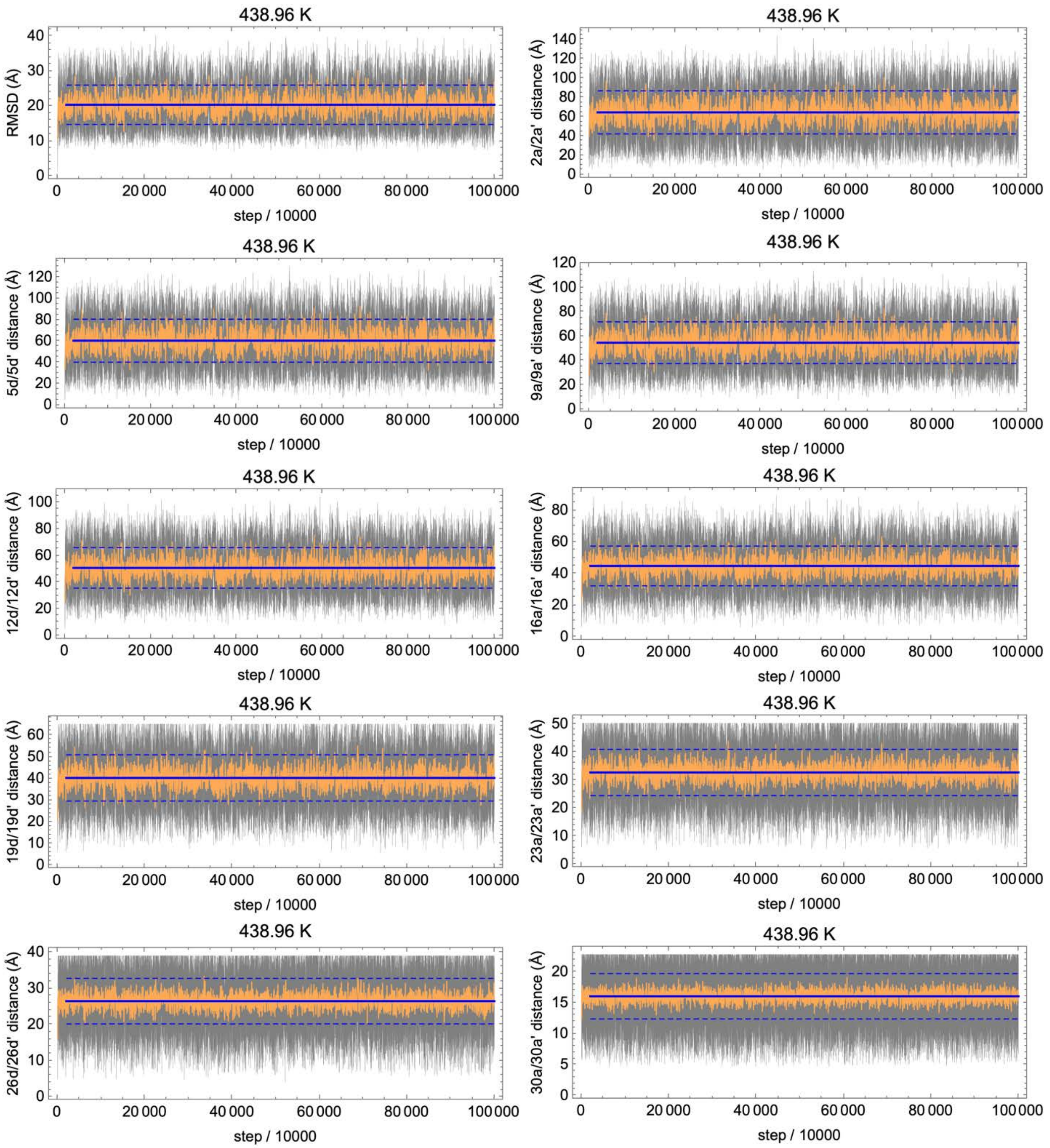

Measure at 438.96 K	Average (Å)	Standard Deviation (Å)	Slope (Å / μs)
RMSD	20.3524	5.62605	−0.0395318
5d/5d' distance	60.3745	20.1659	−0.0116278
12d/12d' distance	50.7252	15.2123	−0.0978673
19d/19d' distance	40.2546	10.6726	−0.0834102
26d/26d' distance	26.4648	6.29961	−0.0382215
2a/2a' distance	64.5056	22.3518	0.0183965
9a/9a' distance	54.5947	17.1559	−0.0968534
16a/16a' distance	44.8304	12.5585	−0.0999368
23a/23a' distance	32.6986	8.2426	−0.108113
30a/30a' distance	16.0576	3.66638	0.0271818
Core Distance	43.3895	20.8116	

Figure S176. Plots of RMSD and residue packing distances for T-REMD simulations of variant **13e/8g'-xx** at 438.96 K.

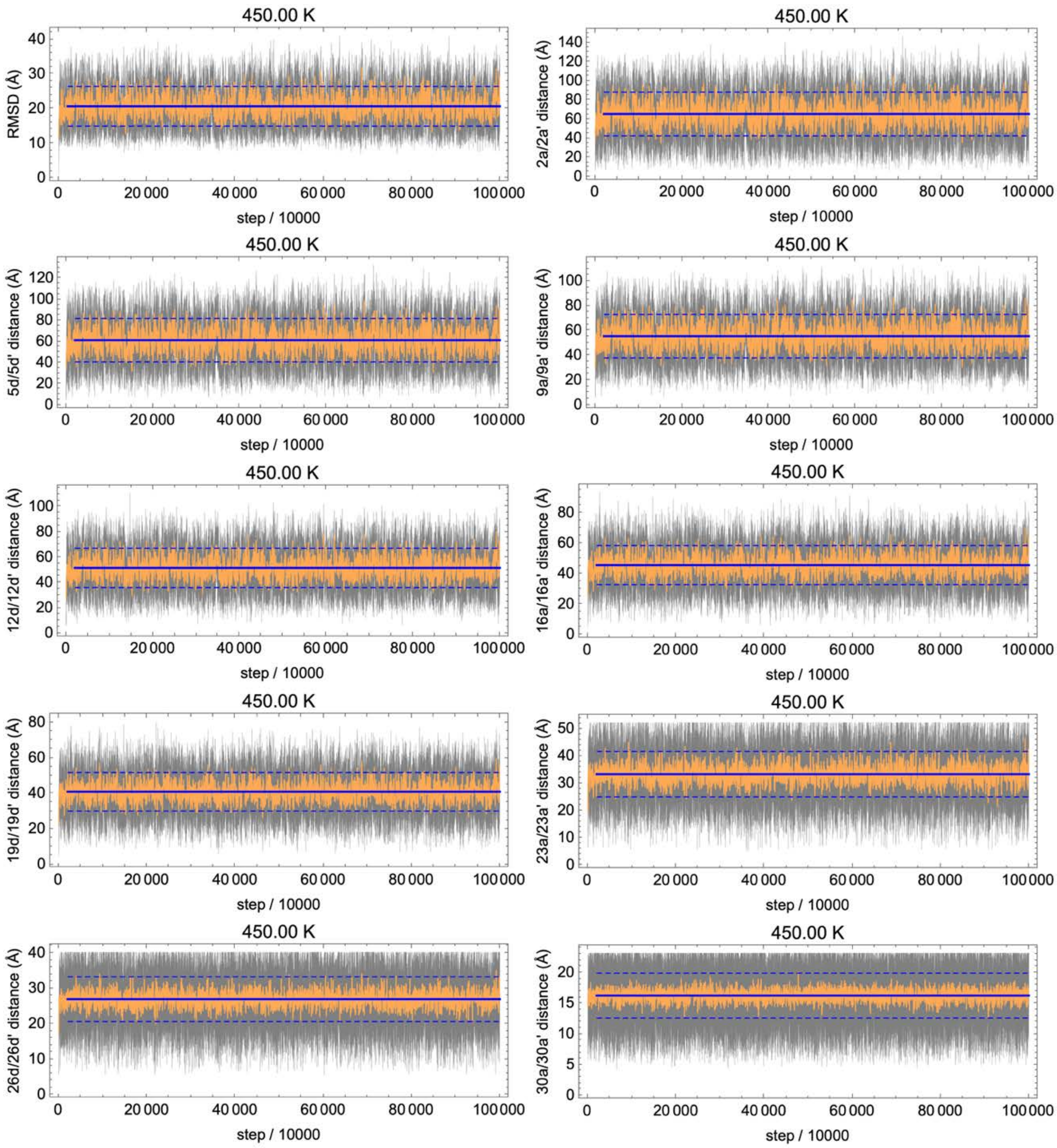

Measure at 450.00 K	Average (Å)	Standard Deviation (Å)	Slope (Å / μs)
RMSD	20.6902	5.7162	−0.00503144
5d/5d' distance	61.2208	20.5684	0.0689689
12d/12d' distance	51.5416	15.5303	0.0734475
19d/19d' distance	40.9555	10.8723	0.0748212
26d/26d' distance	26.9578	6.30744	0.0278145
2a/2a' distance	65.3788	22.8316	0.0578096
9a/9a' distance	55.4533	17.5274	0.0314284
16a/16a' distance	45.617	12.846	0.10602
23a/23a' distance	33.3094	8.33173	−0.00947422
30a/30a' distance	16.2456	3.65198	0.00161778
Core Distance	44.0755	21.1529	

Figure S177. Plots of RMSD and residue packing distances for T-REMD simulations of variant **13e/8g’-xx** at 450.00 K.

(return to Table of Contents)

s13e/8g'-x4x

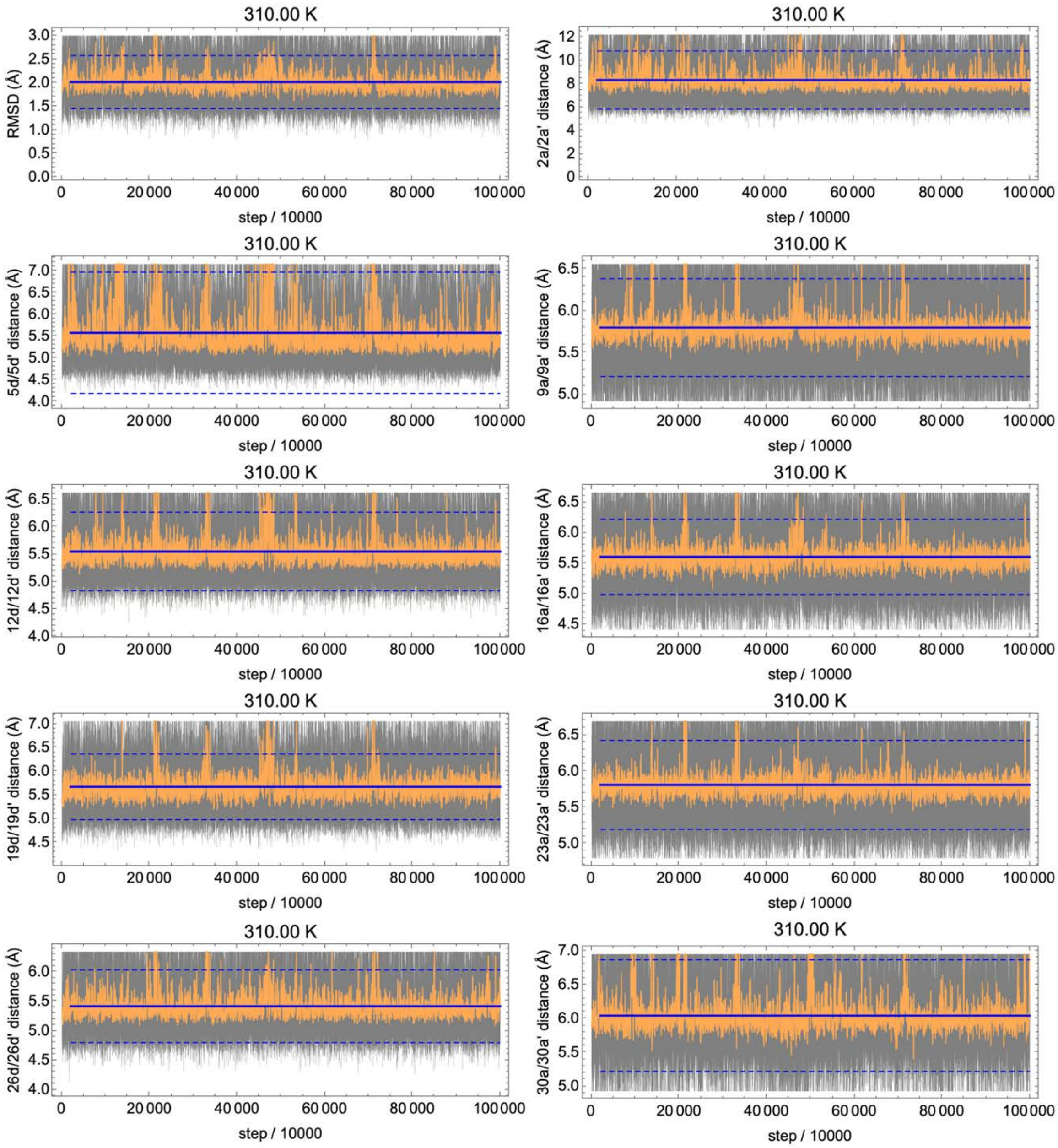

Measure at 310.00 K	Average (Å)	Standard Deviation (Å)	Slope (Å / μs)
RMSD	2.01642	0.561831	−0.0532616
5d/5d' distance	5.58041	1.39151	−0.129837
12d/12d' distance	5.54806	0.716172	−0.0309871
19d/19d' distance	5.67354	0.692222	0.00116821
26d/26d' distance	5.41676	0.617875	−0.0178326
2a/2a' distance	8.3256	2.48976	−0.222746
9a/9a' distance	5.79817	0.586167	−0.0360715
16a/16a' distance	5.6022	0.613073	−0.0151096
23a/23a' distance	5.81082	0.614392	−0.0270324
30a/30a' distance	6.04372	0.823502	−0.0200941
Core Distance	5.9777	1.40442	

Figure S178. Plots of RMSD and residue packing distances for T-REMD simulations of variant **s13e/8g'-x4x** at 310.00 K.

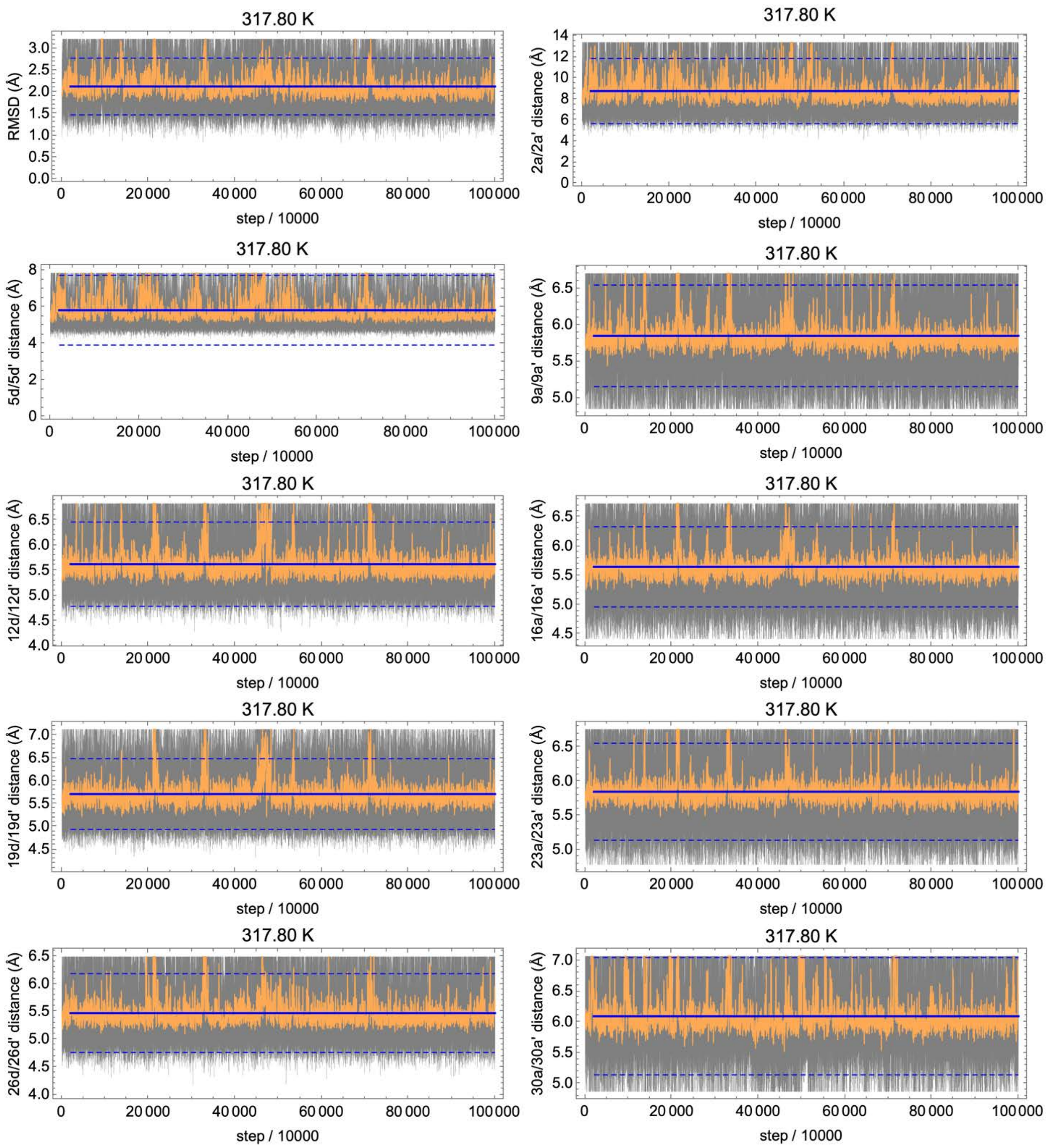

Measure at 317.80 K	Average (Å)	Standard Deviation (Å)	Slope (Å / μs)
RMSD	2.12382	0.651923	−0.0841402
5d/5d' distance	5.81697	1.9068	−0.23557
12d/12d' distance	5.62387	0.836541	−0.0487512
19d/19d' distance	5.71193	0.773528	−0.0143535
26d/26d' distance	5.47171	0.712326	−0.0310287
2a/2a' distance	8.75315	3.09298	−0.386225
9a/9a' distance	5.84856	0.696667	−0.0494547
16a/16a' distance	5.64746	0.684763	−0.0271368
23a/23a' distance	5.84674	0.708175	−0.042993
30a/30a' distance	6.09397	0.951645	−0.0426367
Core Distance	6.09049	1.68628	

Figure S179. Plots of RMSD and residue packing distances for T-REMD simulations of variant **s13e/8g'-x4x** at 317.80 K.

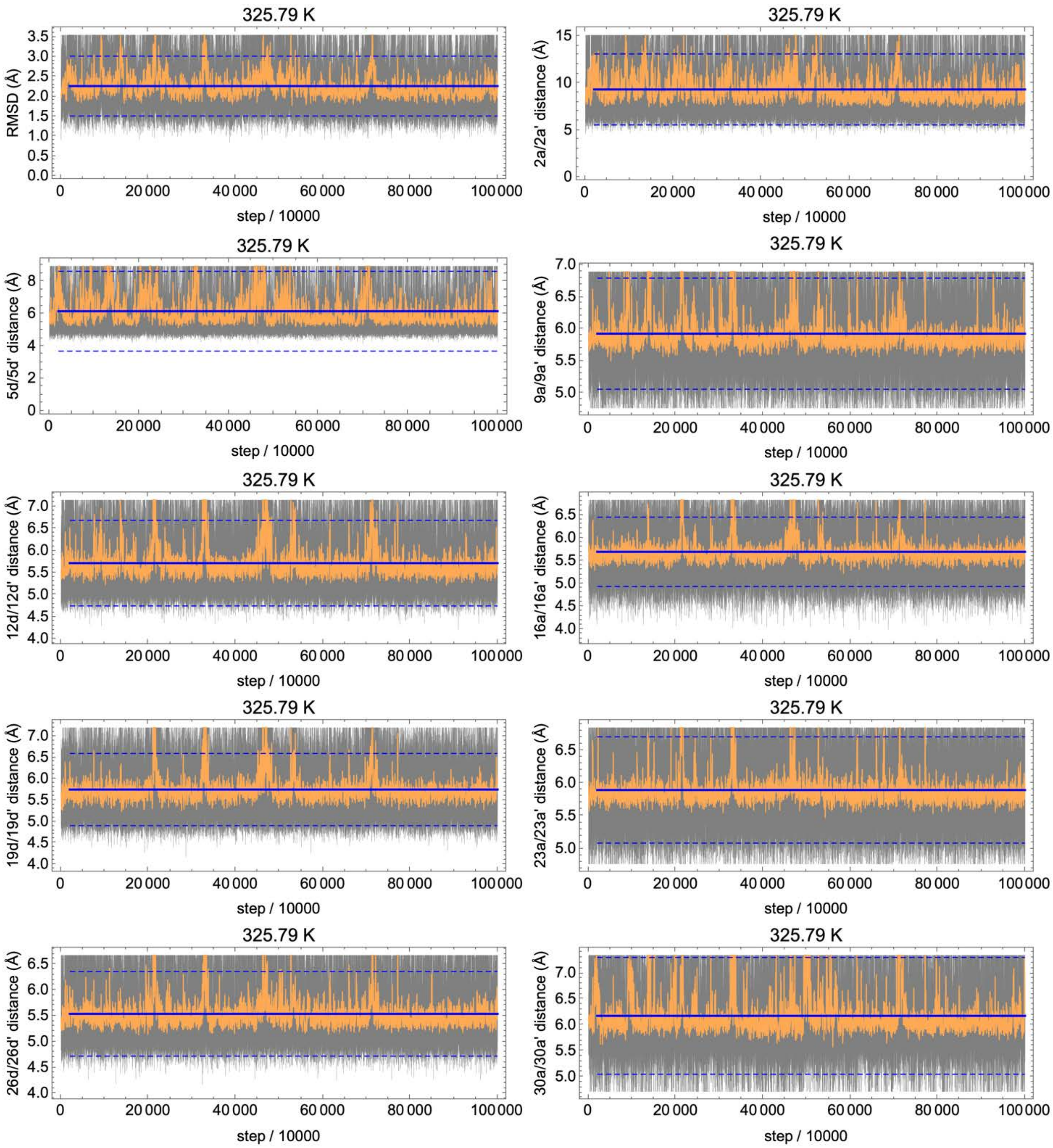

Measure at 325.79 K	Average (Å)	Standard Deviation (Å)	Slope (Å / μs)
RMSD	2.26132	0.752066	−0.10662
5d/5d' distance	6.15246	2.46349	−0.324676
12d/12d' distance	5.71544	0.968709	−0.0707058
19d/19d' distance	5.74915	0.844974	−0.0246159
26d/26d' distance	5.53811	0.818128	−0.036244
2a/2a' distance	9.3247	3.77326	−0.513901
9a/9a' distance	5.92424	0.867871	−0.0769602
16a/16a' distance	5.69426	0.759275	−0.0285689
23a/23a' distance	5.89535	0.809528	−0.0484039
30a/30a' distance	6.1768	1.12613	−0.0526335
Core Distance	6.24117	2.02575	

Figure S180. Plots of RMSD and residue packing distances for T-REMD simulations of variant **s13e/8g'-x4x** at 325.79 K.

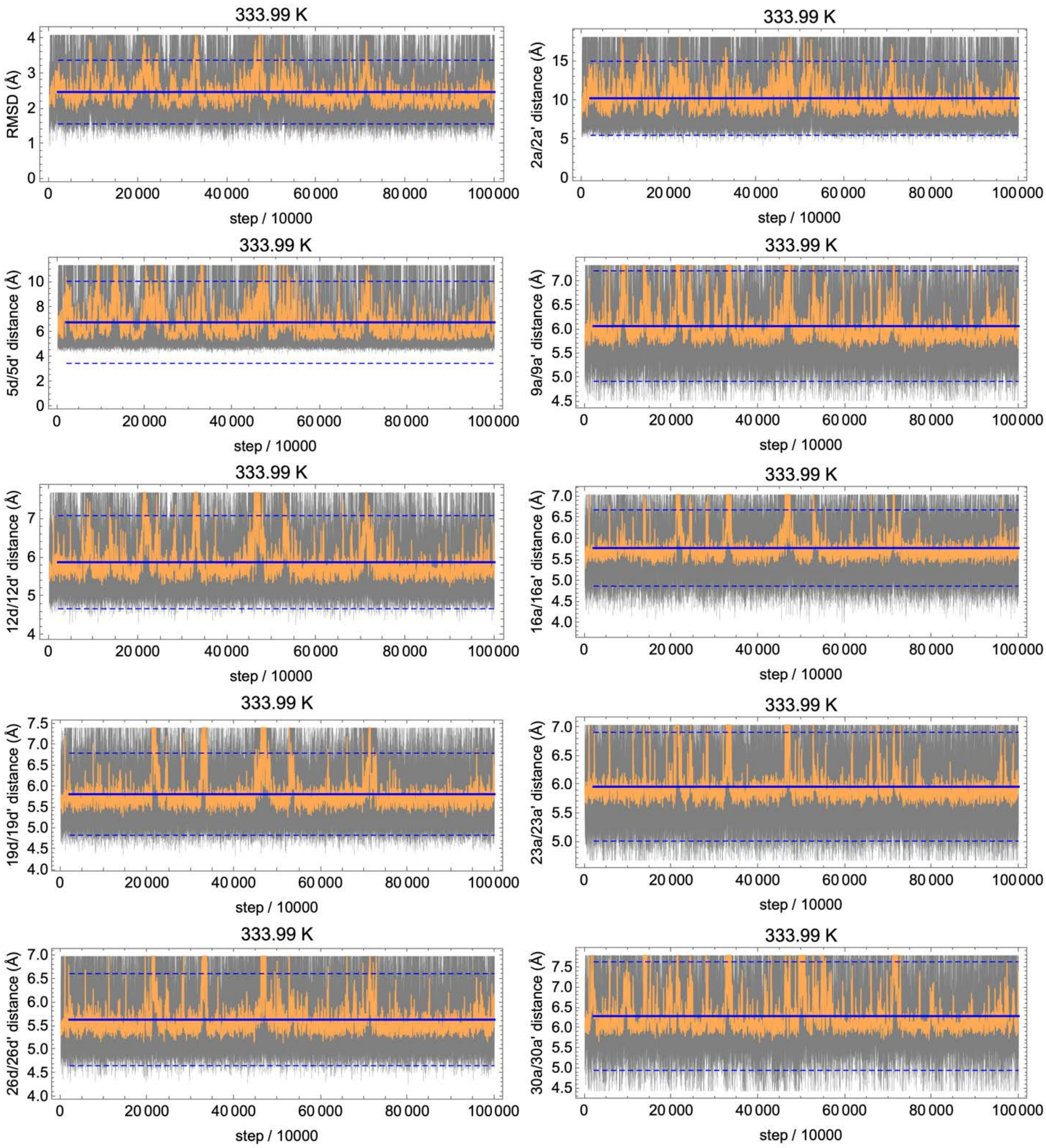

Measure at 333.99 K	Average (Å)	Standard Deviation (Å)	Slope (Å / μs)
RMSD	2.47189	0.907128	−0.160163
5d/5d' distance	6.77002	3.30156	−0.545445
12d/12d' distance	5.88032	1.20493	−0.118435
19d/19d' distance	5.81608	0.984175	−0.0406891
26d/26d' distance	5.63511	0.977578	−0.044448
2a/2a' distance	10.2661	4.75826	−0.809799
9a/9a' distance	6.06794	1.14846	−0.123678
16a/16a' distance	5.77809	0.905323	−0.0479966
23a/23a' distance	5.96555	0.951646	−0.0604142
30a/30a' distance	6.29408	1.3448	−0.0663397
Core Distance	6.49703	2.55303	

Figure S181. Plots of RMSD and residue packing distances for T-REMD simulations of variant **s13e/8g'-x4x** at 333.99 K.

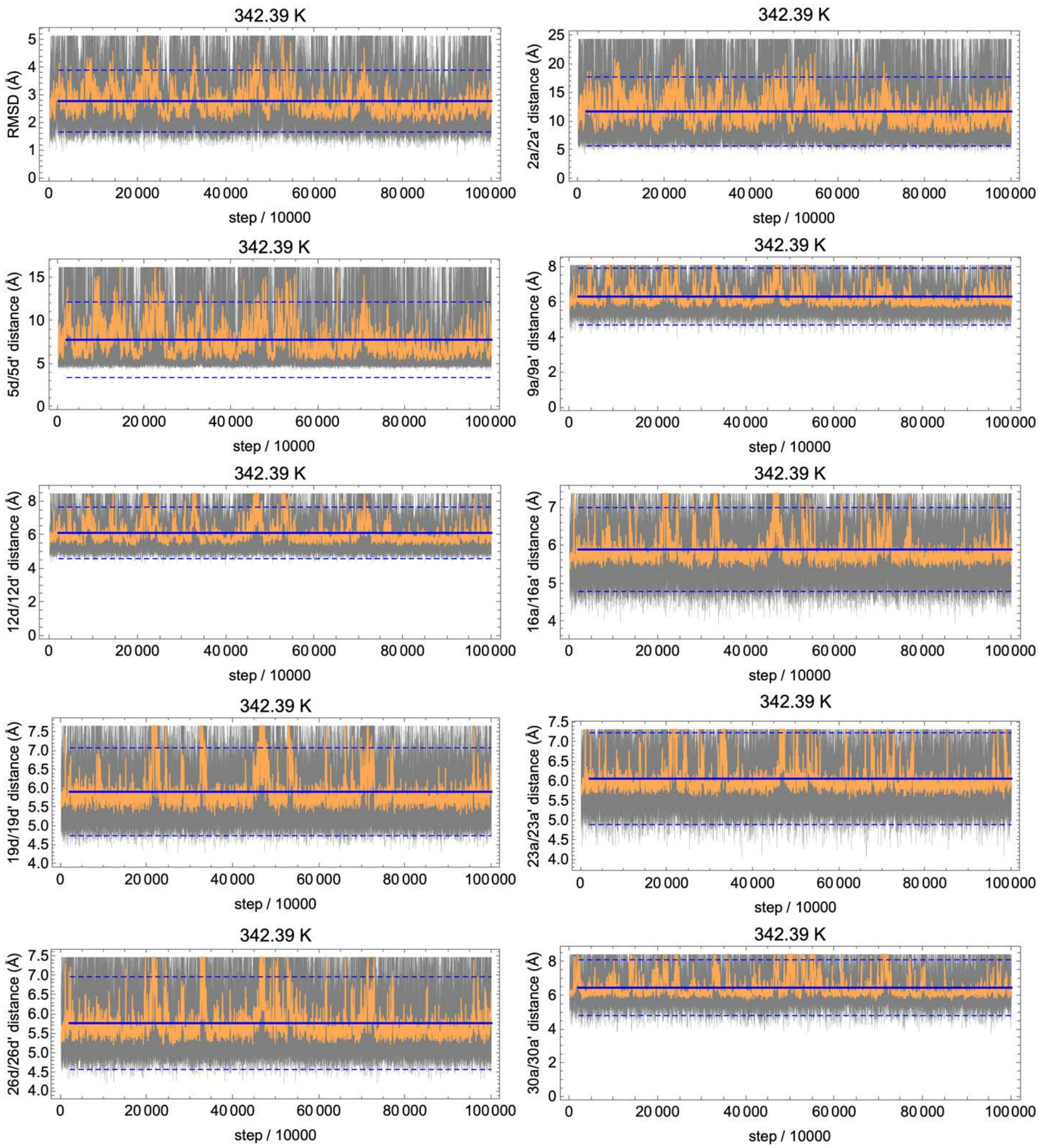

Measure at 342.39 K	Average (Å)	Standard Deviation (Å)	Slope (Å / μs)
RMSD	2.78774	1.11691	−0.243475
5d/5d' distance	7.81456	4.38645	−0.925427
12d/12d' distance	6.14164	1.53507	−0.184676
19d/19d' distance	5.9164	1.16927	−0.0557226
26d/26d' distance	5.77755	1.19434	−0.054995
2a/2a' distance	11.7626	6.01983	−1.29428
9a/9a' distance	6.32298	1.61575	−0.207166
16a/16a' distance	5.90028	1.10515	−0.0680796
23a/23a' distance	6.07221	1.17113	−0.0763297
30a/30a' distance	6.47858	1.65396	−0.0771742
Core Distance	6.90965	3.30062	

Figure S182. Plots of RMSD and residue packing distances for T-REMD simulations of variant **s13e/8g'-x4x** at 342.39 K.

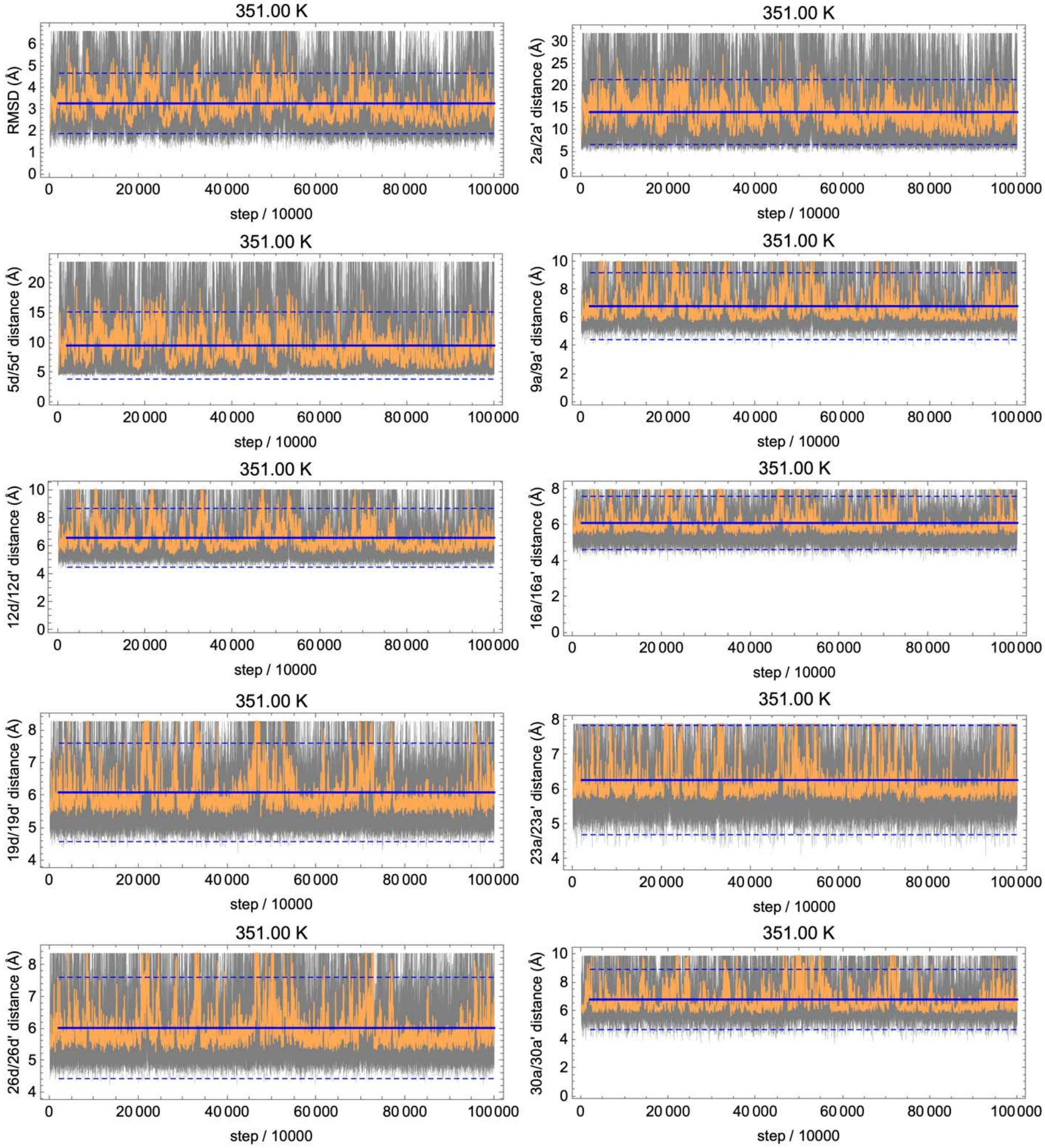

Measure at 351.00 K	Average (Å)	Standard Deviation (Å)	Slope (Å / μs)
RMSD	3.28245	1.39205	−0.30692
5d/5d' distance	9.54444	5.62698	−1.19859
12d/12d' distance	6.60976	2.10147	−0.29072
19d/19d' distance	6.09536	1.51189	−0.101312
26d/26d' distance	6.02805	1.59125	−0.0736313
2a/2a' distance	14.0368	7.37201	−1.6229
9a/9a' distance	6.8355	2.3827	−0.342046
16a/16a' distance	6.12467	1.48665	−0.118217
23a/23a' distance	6.27222	1.57631	−0.090326
30a/30a' distance	6.81381	2.11583	−0.0790411
Core Distance	7.59563	4.2968	

Figure S183. Plots of RMSD and residue packing distances for T-REMD simulations of variant **s13e/8g'-x4x** at 351.00 K.

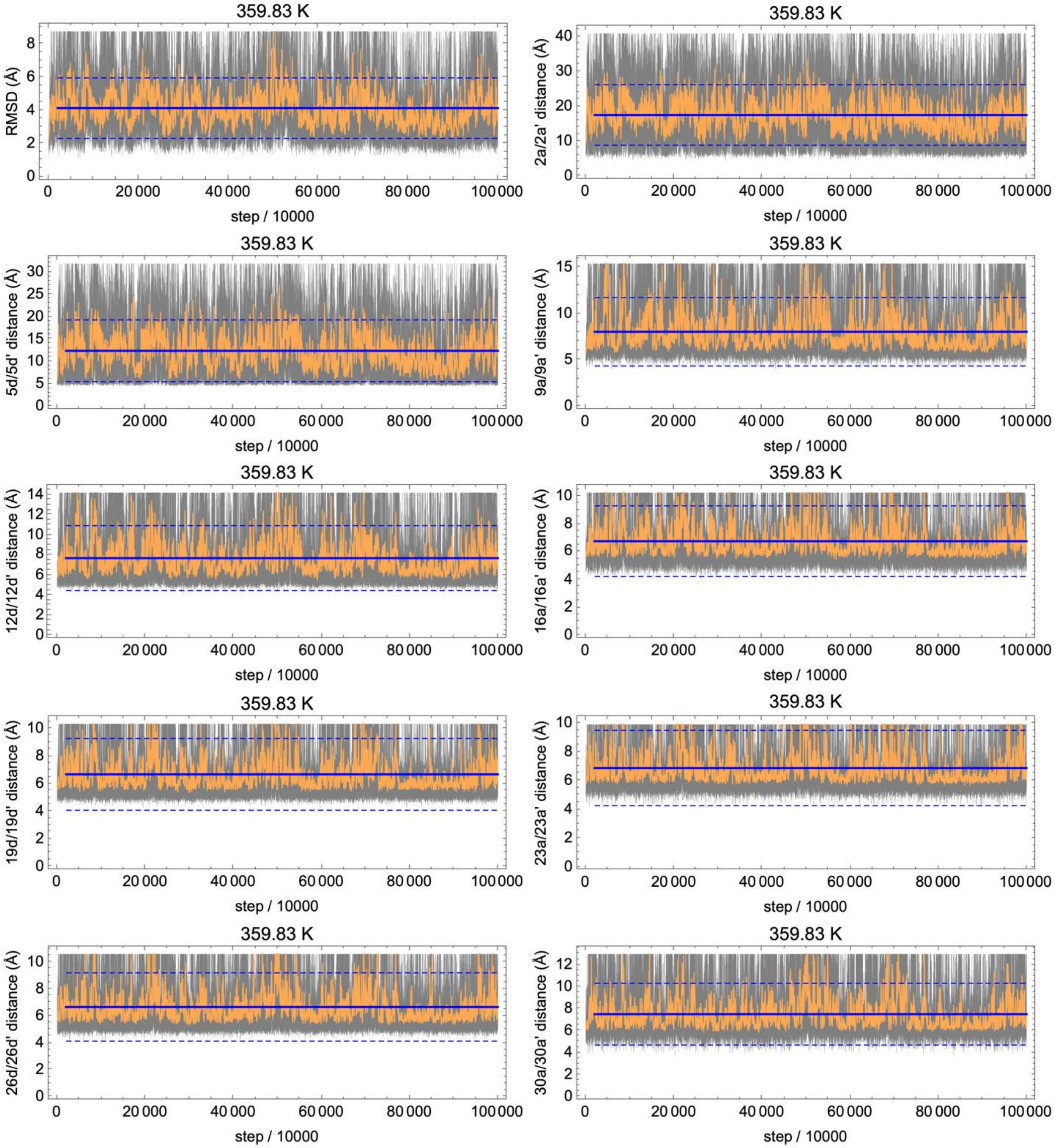

Measure at 359.83 K	Average (Å)	Standard Deviation (Å)	Slope (Å / μs)
RMSD	4.11862	1.82278	−0.311938
5d/5d' distance	12.3416	6.90324	−1.1533
12d/12d' distance	7.66347	3.23859	−0.411407
19d/19d' distance	6.66668	2.5993	−0.184433
26d/26d' distance	6.64935	2.53856	−0.106934
2a/2a' distance	17.4263	8.66431	−1.46239
9a/9a' distance	8.01187	3.69042	−0.469246
16a/16a' distance	6.74796	2.53947	−0.193362
23a/23a' distance	6.86199	2.61746	−0.131832
30a/30a' distance	7.48449	2.80536	−0.111255
Core Distance	8.87264	5.66566	

Figure S184. Plots of RMSD and residue packing distances for T-REMD simulations of variant **s13e/8g’-x4x** at 359.83 K.

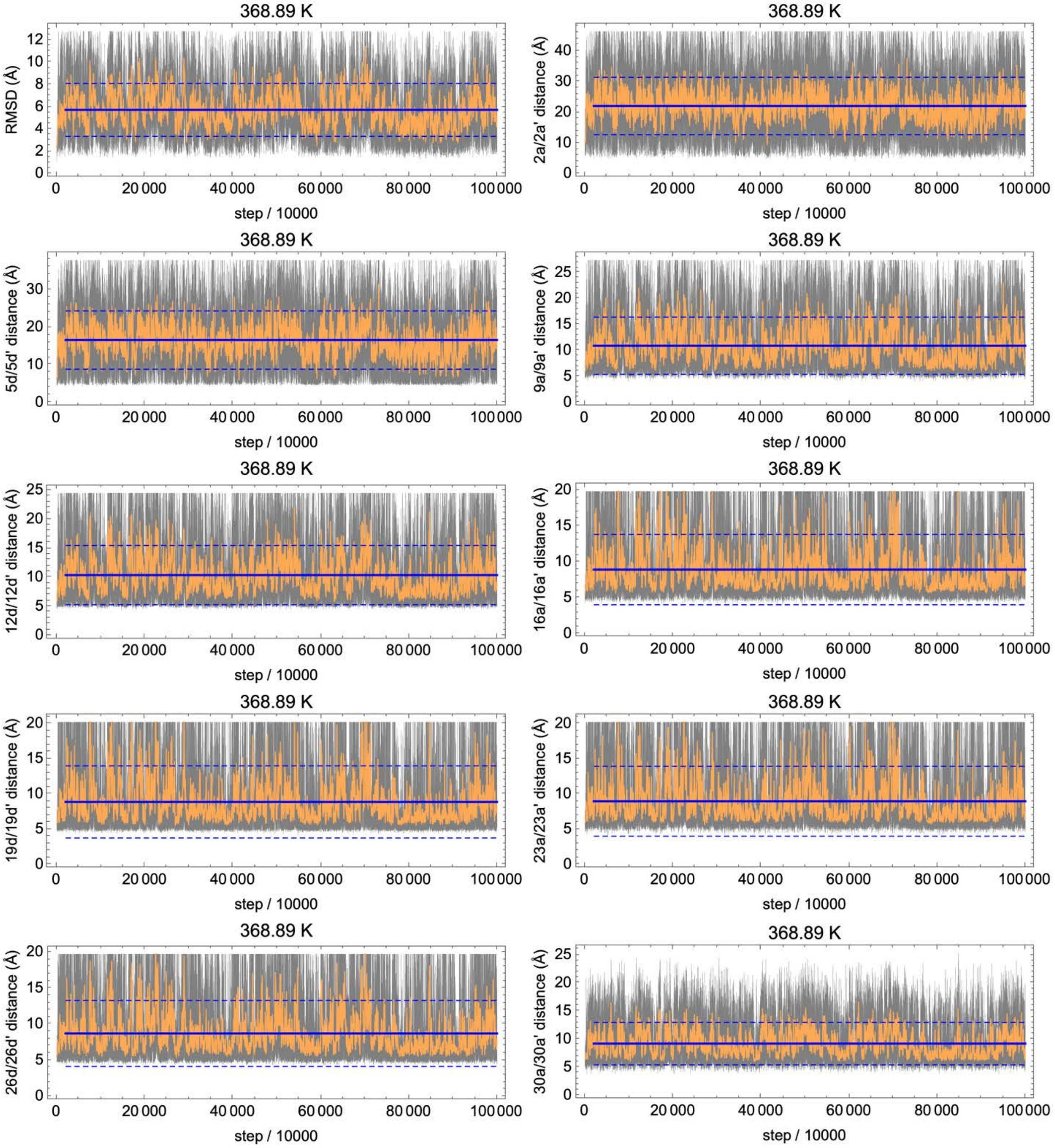

Measure at 368.89 K	Average (Å)	Standard Deviation (Å)	Slope (Å / μs)
RMSD	5.71034	2.37686	−0.495748
5d/5d' distance	16.5425	7.74859	−1.17508
12d/12d' distance	10.3479	5.10596	−0.84002
19d/19d' distance	8.85193	5.11425	−0.804752
26d/26d' distance	8.69681	4.57994	−0.580058
2a/2a' distance	22.017	9.36549	−1.36754
9a/9a' distance	10.8841	5.51029	−0.881797
16a/16a' distance	8.91227	4.89951	−0.720676
23a/23a' distance	8.95588	4.96533	−0.624944
30a/30a' distance	9.19668	3.78805	−0.43638
Core Distance	11.6006	7.34332	

Figure S185. Plots of RMSD and residue packing distances for T-REMD simulations of variant **s13e/8g'-x4x** at 368.89 K.

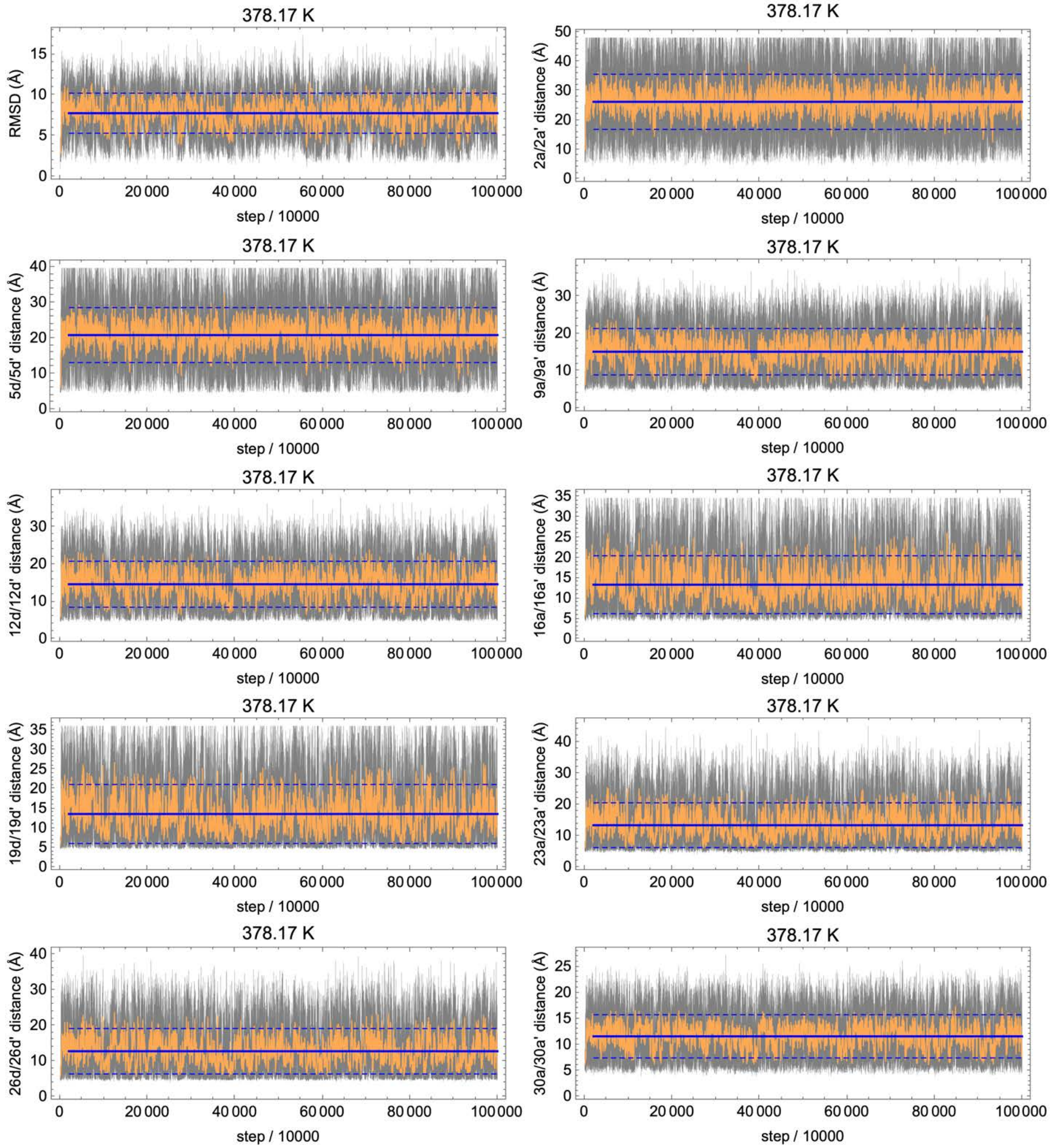

Measure at 378.17 K	Average (Å)	Standard Deviation (Å)	Slope (Å / μs)
RMSD	7.76962	2.45695	−0.129874
5d/5d' distance	20.8368	7.72809	−0.24435
12d/12d' distance	14.6288	6.17103	−0.252243
19d/19d' distance	13.5805	7.54097	−0.384728
26d/26d' distance	12.7818	6.37816	−0.245716
2a/2a' distance	26.257	9.38672	−0.360891
9a/9a' distance	15.1326	6.21656	−0.124634
16a/16a' distance	13.3501	7.09853	−0.358589
23a/23a' distance	13.4071	7.16969	−0.250627
30a/30a' distance	11.5976	4.15778	−0.113451
Core Distance	15.7303	8.3073	

Figure S186. Plots of RMSD and residue packing distances for T-REMD simulations of variant **s13e/8g'-x4x** at 378.17 K.

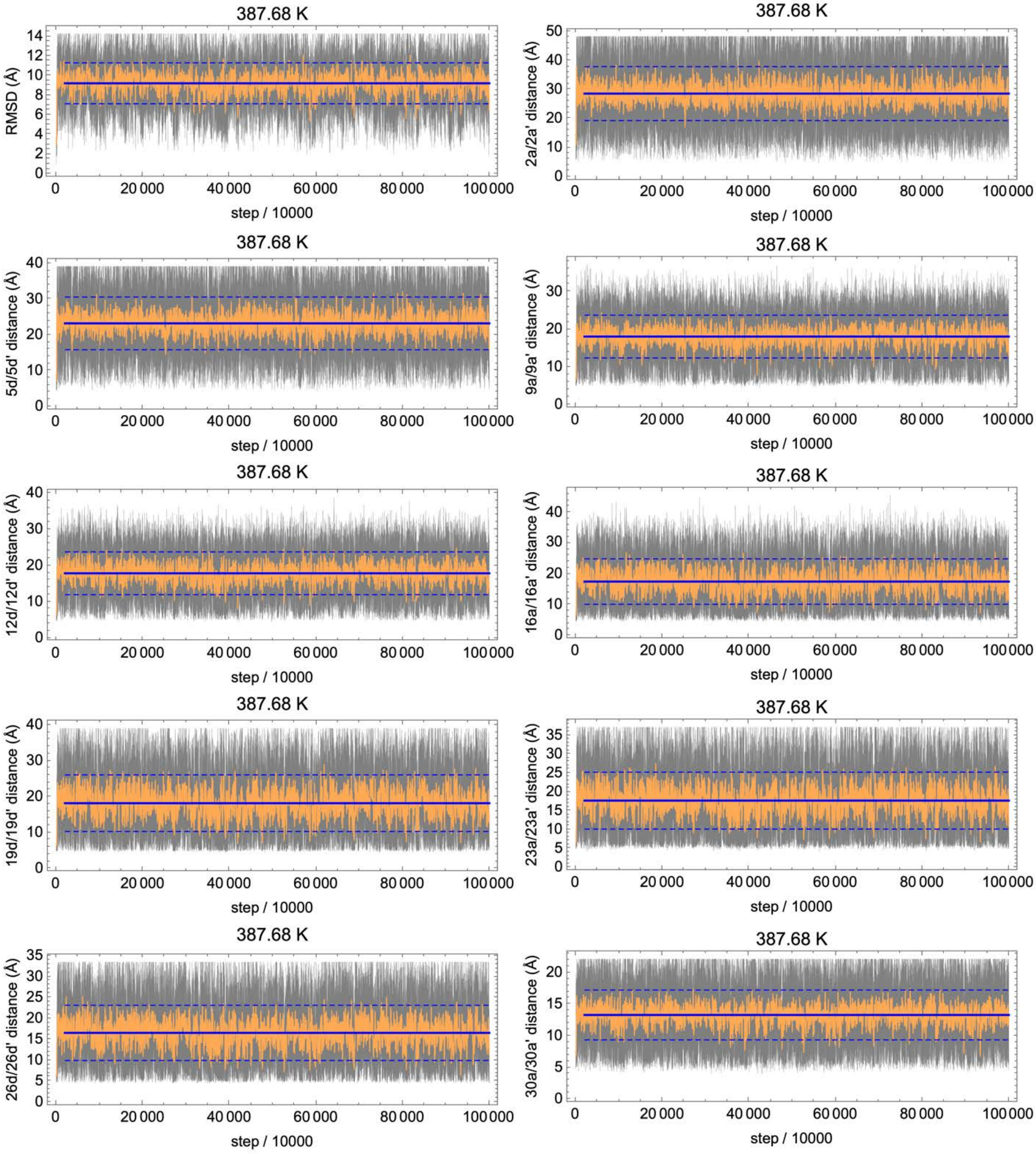

Measure at 387.68 K	Average (Å)	Standard Deviation (Å)	Slope (Å / μs)
RMSD	9.20865	2.08557	−0.061106
5d/5d' distance	23.2092	7.3669	−0.0485446
12d/12d' distance	17.8344	5.90373	−0.100113
19d/19d' distance	18.1686	7.89253	−0.134343
26d/26d' distance	16.5356	6.60408	−0.178514
2a/2a' distance	28.5619	9.29926	−0.241768
9a/9a' distance	18.0495	5.69107	0.0104847
16a/16a' distance	17.4707	7.39219	−0.179551
23a/23a' distance	17.6511	7.55026	−0.132018
30a/30a' distance	13.2889	3.94494	−0.0604168
Core Distance	18.9744	8.13802	

Figure S187. Plots of RMSD and residue packing distances for T-REMD simulations of variant **s13e/8g'-x4x** at 387.68 K.

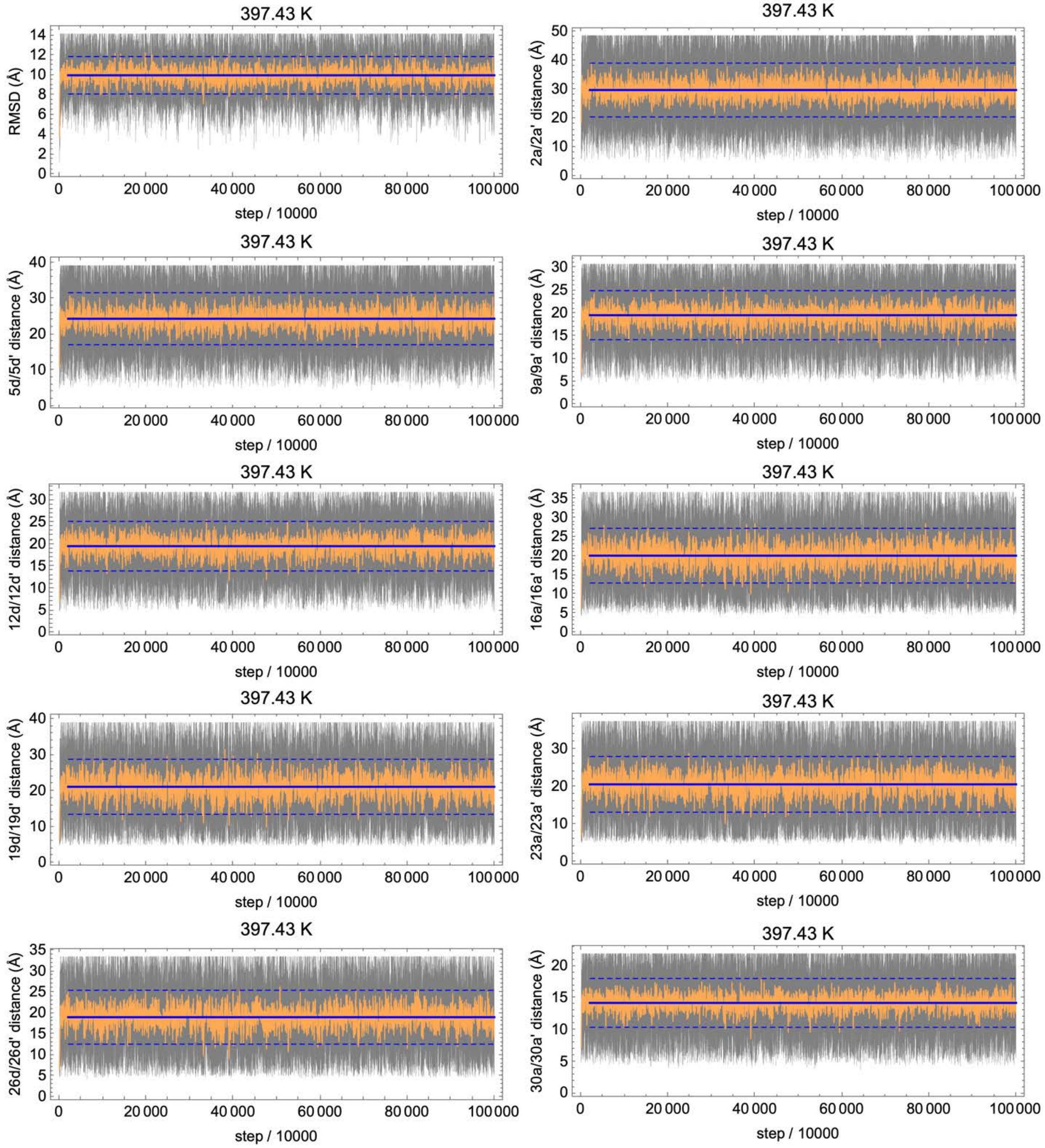

Measure at 397.43 K	Average (Å)	Standard Deviation (Å)	Slope (Å / μs)
RMSD	9.96884	1.88899	−0.0156431
5d/5d' distance	24.3616	7.27941	−0.0375551
12d/12d' distance	19.5423	5.61757	0.0123012
19d/19d' distance	21.1257	7.65852	0.0105753
26d/26d' distance	19.0121	6.43694	−0.0315173
2a/2a' distance	29.7234	9.32816	−0.147937
9a/9a' distance	19.5271	5.37177	0.0706989
16a/16a' distance	20.0691	7.15892	0.0159466
23a/23a' distance	20.5818	7.42058	−0.0317713
30a/30a' distance	14.2181	3.81407	0.0121908
Core Distance	20.9068	7.91609	

Figure S188. Plots of RMSD and residue packing distances for T-REMD simulations of variant **s13e/8g’-x4x** at 397.43 K.

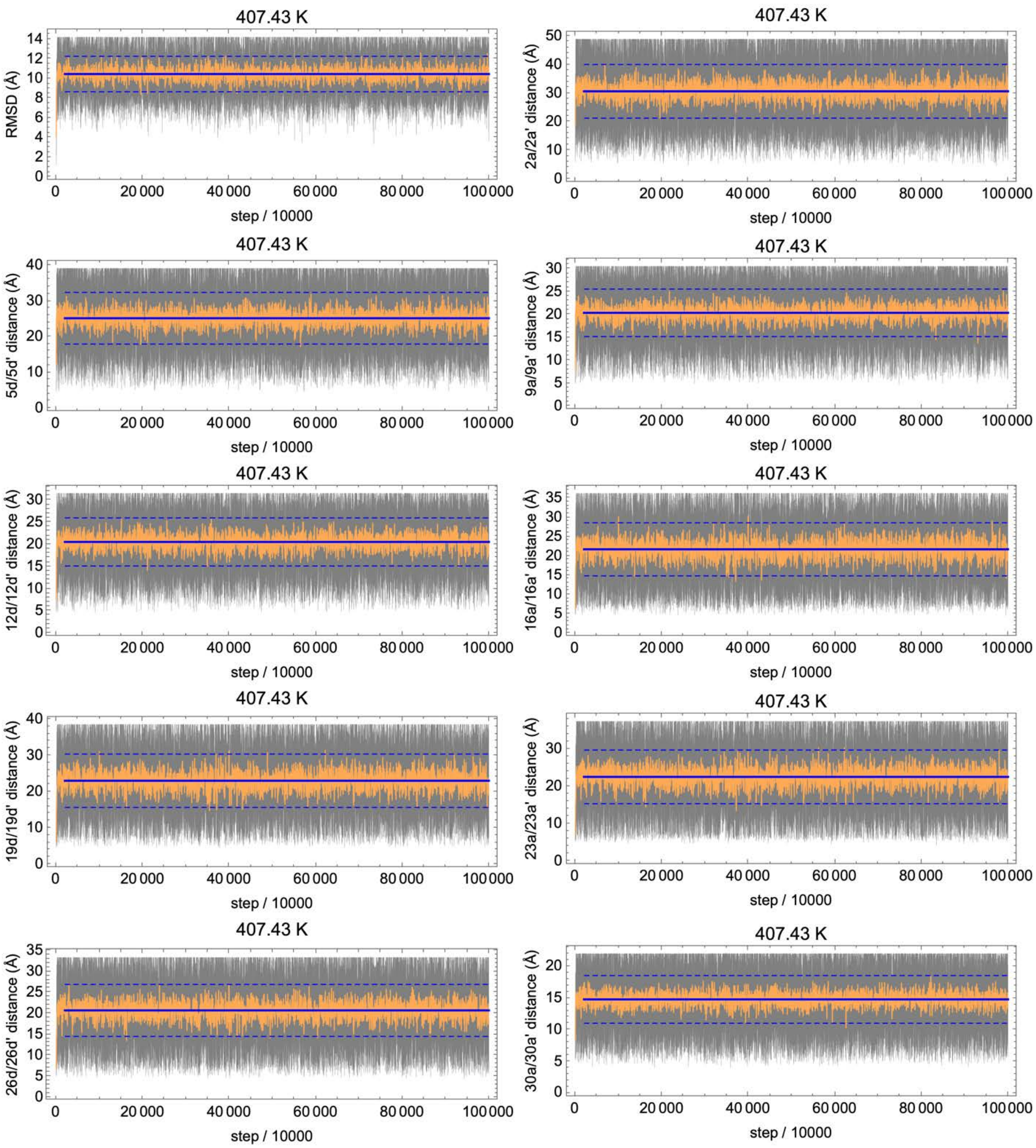

Measure at 407.43 K	Average (Å)	Standard Deviation (Å)	Slope (Å / μs)
RMSD	10.4545	1.80459	−0.0249235
5d/5d' distance	25.1333	7.23139	0.0186118
12d/12d' distance	20.525	5.42046	0.0287106
19d/19d' distance	22.9903	7.36178	0.00635221
26d/26d' distance	20.6721	6.2043	−0.042826
2a/2a' distance	30.6292	9.39046	−0.105596
9a/9a' distance	20.3575	5.1941	0.0793742
16a/16a' distance	21.6822	6.88373	0.0357326
23a/23a' distance	22.4918	7.175	−0.0189425
30a/30a' distance	14.7584	3.74367	0.0391643
Core Distance	22.1378	7.79538	

Figure S189. Plots of RMSD and residue packing distances for T-REMD simulations of variant **s13e/8g’-x4x** at 407.43 K.

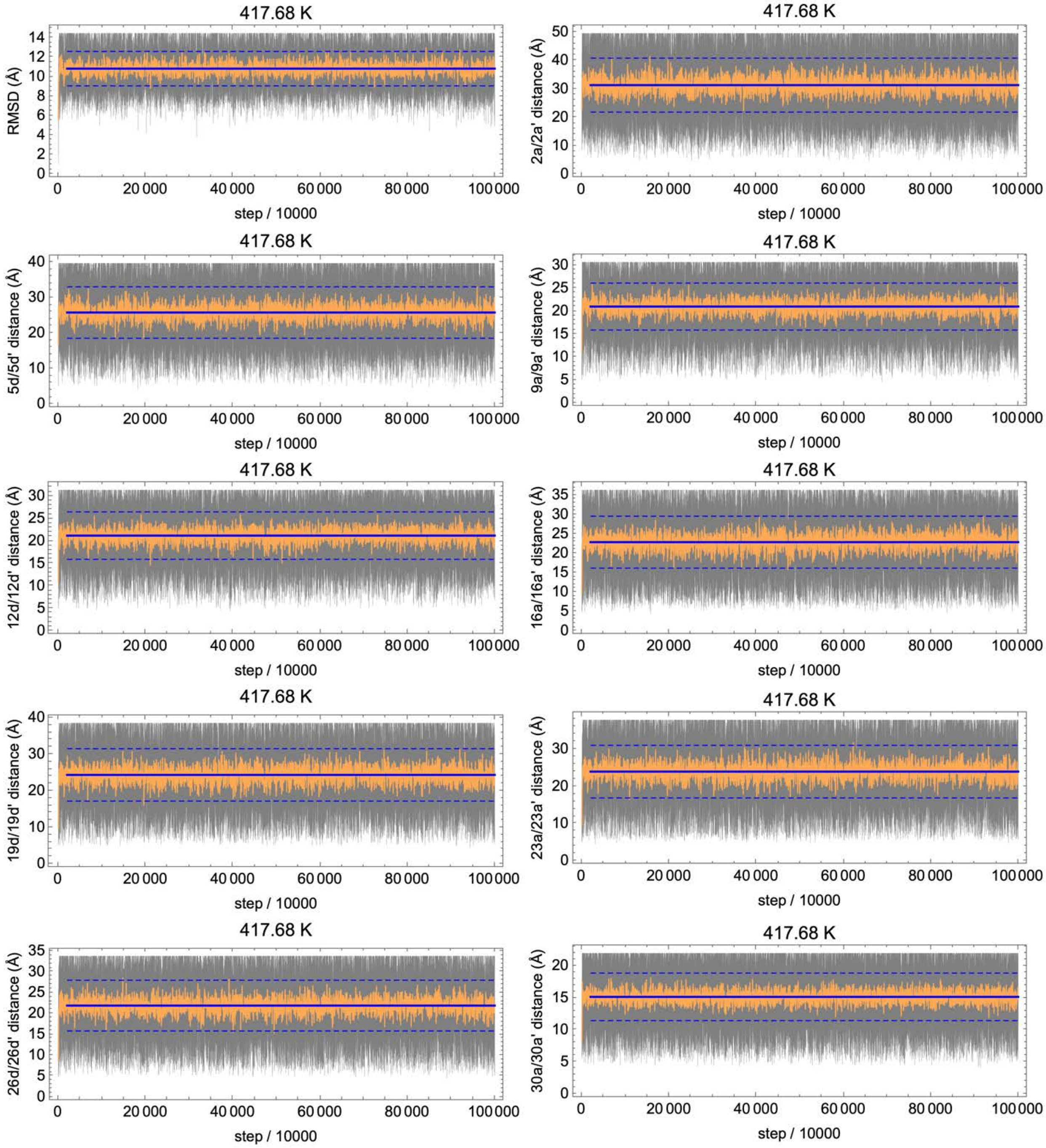

Measure at 417.68 K	Average (Å)	Standard Deviation (Å)	Slope (Å / μs)
RMSD	10.8329	1.76837	−0.0087095
5d/5d' distance	25.7769	7.24285	0.00377778
12d/12d' distance	21.2044	5.28441	0.101341
19d/19d' distance	24.3432	7.164	0.0589823
26d/26d' distance	21.881	6.08299	−0.0154234
2a/2a' distance	31.3278	9.50043	−0.0274602
9a/9a' distance	20.967	5.10793	0.0376414
16a/16a' distance	22.8527	6.69653	0.181741
23a/23a' distance	23.9392	7.06802	−0.0546012
30a/30a' distance	15.1454	3.70856	−0.00125829
Core Distance	23.0486	7.7722	

Figure S190. Plots of RMSD and residue packing distances for T-REMD simulations of variant **s13e/8g'-x4x** at 417.68 K.

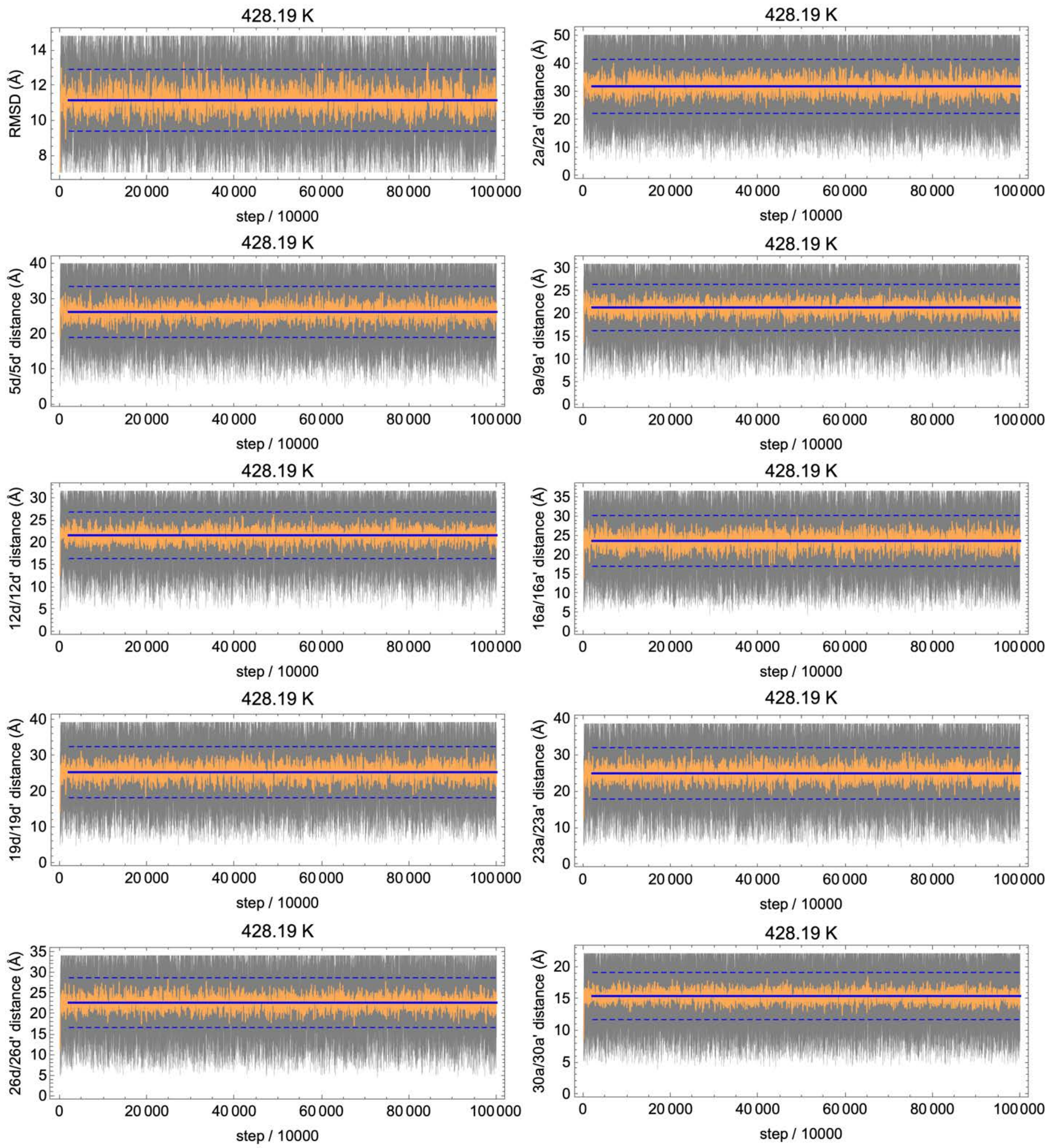

Measure at 428.19 K	Average (Å)	Standard Deviation (Å)	Slope (Å / μs)
RMSD	11.1683	1.76063	−0.0221778
5d/5d' distance	26.3224	7.2614	0.0326655
12d/12d' distance	21.7039	5.23347	0.109867
19d/19d' distance	25.4075	7.13463	0.0353403
26d/26d' distance	22.7572	6.04432	−0.0861392
2a/2a' distance	31.9894	9.62984	−0.0864354
9a/9a' distance	21.4088	5.09449	0.0498568
16a/16a' distance	23.7328	6.62874	0.12697
23a/23a' distance	25.038	7.0631	−0.0556338
30a/30a' distance	15.4566	3.70241	−0.0736496
Core Distance	23.7574	7.8324	

Figure S191. Plots of RMSD and residue packing distances for T-REMD simulations of variant **s13e/8g'-x4x** at 428.19 K.

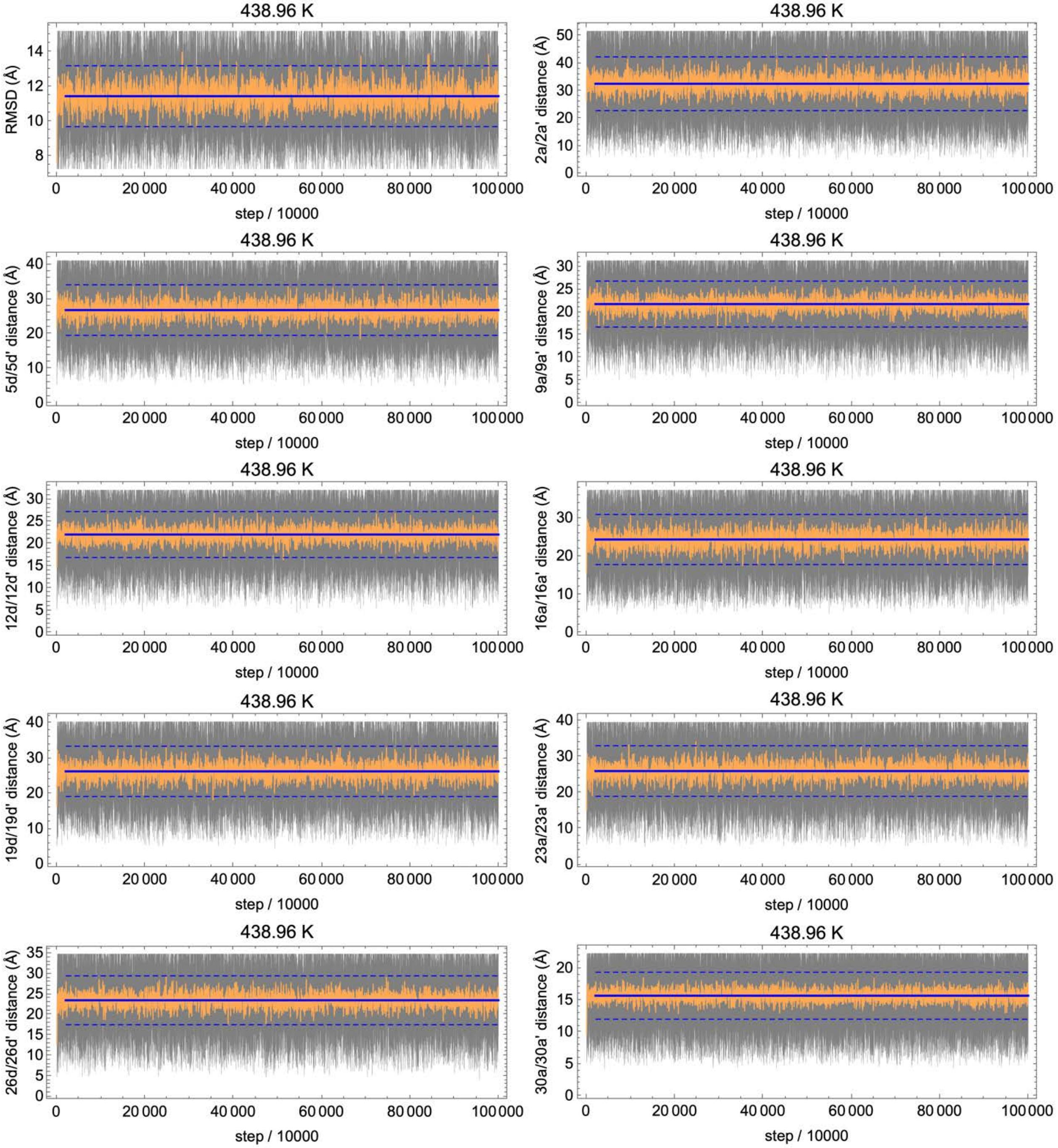

Measure at 438.96 K	Average (Å)	Standard Deviation (Å)	Slope (Å / μs)
RMSD	11.4493	1.75286	0.00608291
5d/5d' distance	26.8113	7.29228	0.030198
12d/12d' distance	22.0608	5.17221	0.117726
19d/19d' distance	26.2489	7.11314	0.0716536
26d/26d' distance	23.5025	5.97044	−0.0497682
2a/2a' distance	32.5752	9.75043	−0.052024
9a/9a' distance	21.7618	5.05393	0.119774
16a/16a' distance	24.3834	6.55991	0.213315
23a/23a' distance	25.9759	7.01569	−0.0163971
30a/30a' distance	15.7081	3.68209	−0.0592514
Core Distance	24.3364	7.88734	

Figure S192. Plots of RMSD and residue packing distances for T-REMD simulations of variant **s13e/8g’-x4x** at 438.96 K.

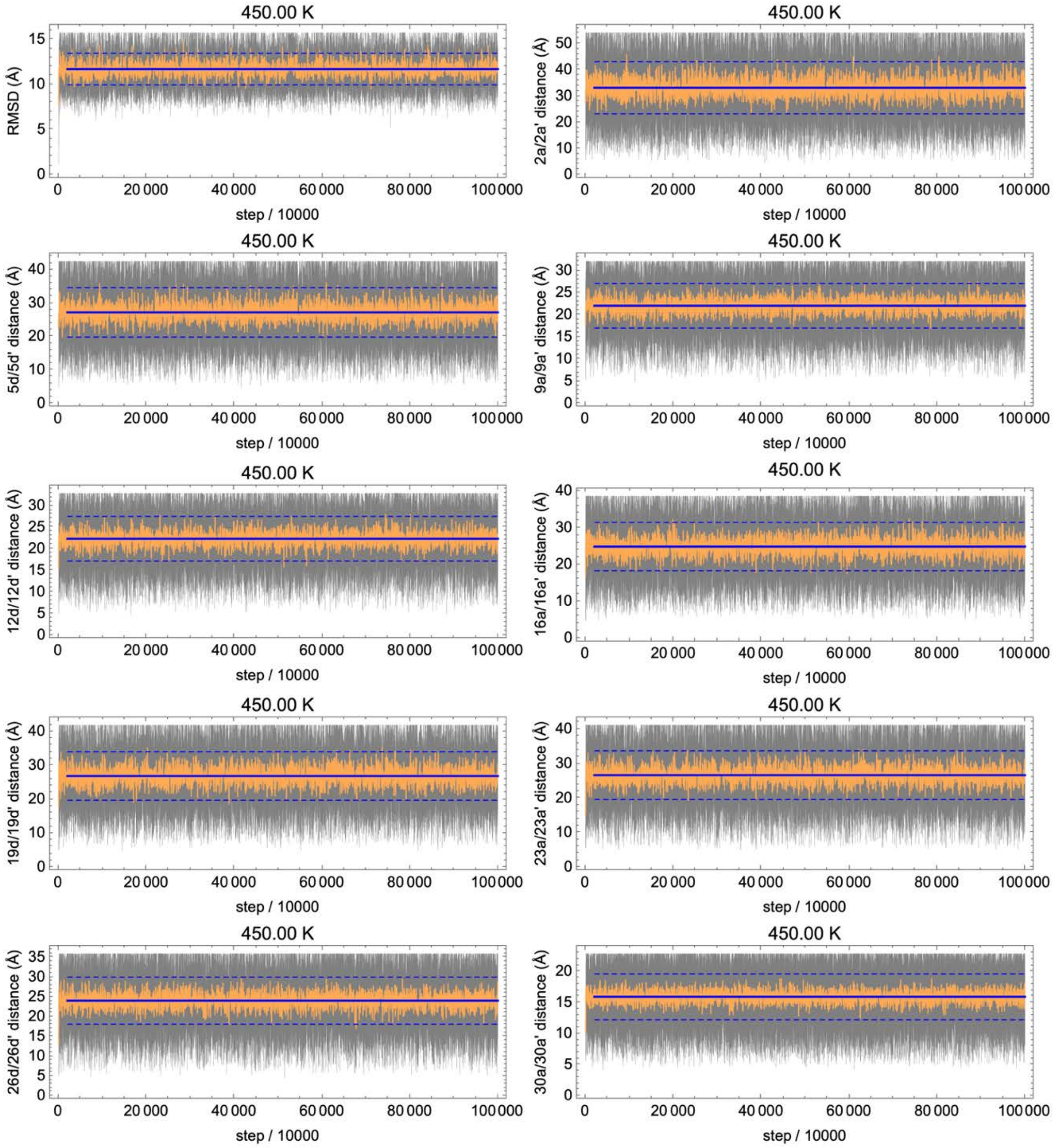

Measure at 450.00 K	Average (Å)	Standard Deviation (Å)	Slope (Å / μs)
RMSD	11.6964	1.75175	0.0265236
5d/5d' distance	27.2074	7.37266	0.100425
12d/12d' distance	22.3267	5.17194	0.0990146
19d/19d' distance	26.8567	7.14682	0.0194707
26d/26d' distance	24.0272	5.94703	−0.00451108
2a/2a' distance	33.1465	9.93111	0.0188535
9a/9a' distance	22.0342	5.03147	0.09421
16a/16a' distance	24.8645	6.56593	0.101793
23a/23a' distance	26.642	7.04758	0.0158559
30a/30a' distance	15.9084	3.6835	−0.0265536
Core Distance	24.7793	7.98849	

Figure S193. Plots of RMSD and residue packing distances for T-REMD simulations of variant **s13e/8g’-x4x** at 450.00 K.

(return to Table of Contents)

20e/15g’-xx

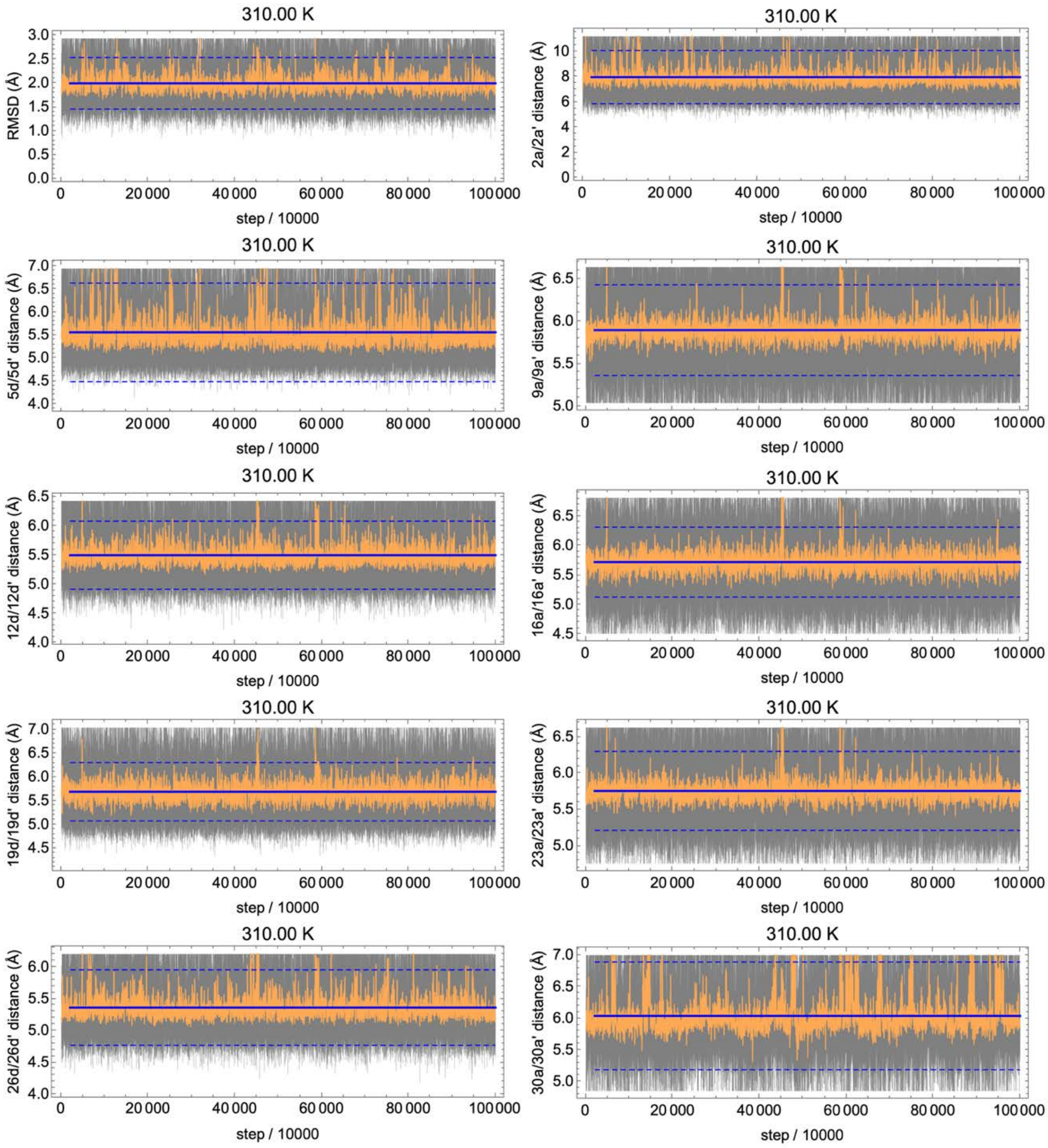

Measure at 310.00 K	Average (Å)	Standard Deviation (Å)	Slope (Å / μs)
RMSD	1.9966	0.539354	−0.0179112
5d/5d' distance	5.55927	1.07074	−0.0457761
12d/12d' distance	5.5004	0.583822	−0.00546945
19d/19d' distance	5.69036	0.614493	0.0120639
26d/26d' distance	5.36361	0.592618	−0.00876646
2a/2a' distance	7.9706	2.10634	−0.0998277
9a/9a' distance	5.89587	0.534217	−0.0037296
16a/16a' distance	5.72262	0.592504	0.00065937
23a/23a' distance	5.75549	0.544065	−0.00597751
30a/30a' distance	6.03981	0.851038	0.0377677
Core Distance	5.94422	1.21375	

Figure S194. Plots of RMSD and residue packing distances for T-REMD simulations of variant **20e/15g’-xx** at 310.00 K.

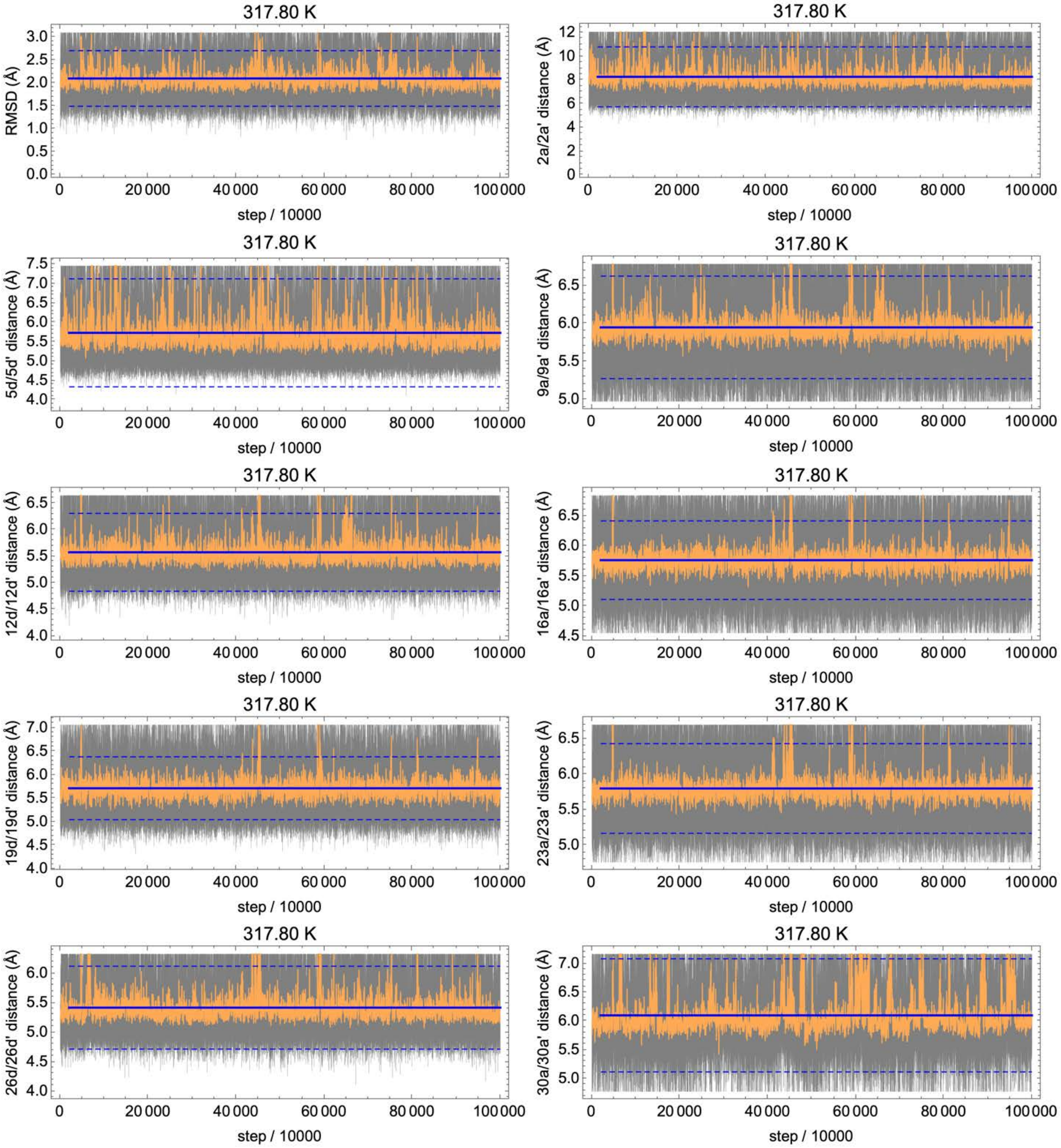

Measure at 317.80 K	Average (Å)	Standard Deviation (Å)	Slope (Å / μs)
RMSD	2.09611	0.605809	−0.0224153
5d/5d' distance	5.72748	1.39149	−0.0706381
12d/12d' distance	5.57348	0.73221	−0.00812231
19d/19d' distance	5.7129	0.669188	0.00449957
26d/26d' distance	5.41675	0.702247	−0.018166
2a/2a' distance	8.27038	2.52347	−0.157607
9a/9a' distance	5.94705	0.675281	−0.00730378
16a/16a' distance	5.76251	0.653576	−0.00100288
23a/23a' distance	5.79628	0.631173	−0.00703882
30a/30a' distance	6.09737	0.982656	0.0589716
Core Distance	6.0338	1.4127	

Figure S195. Plots of RMSD and residue packing distances for T-REMD simulations of variant **20e/15g'-xx** at 317.80 K.

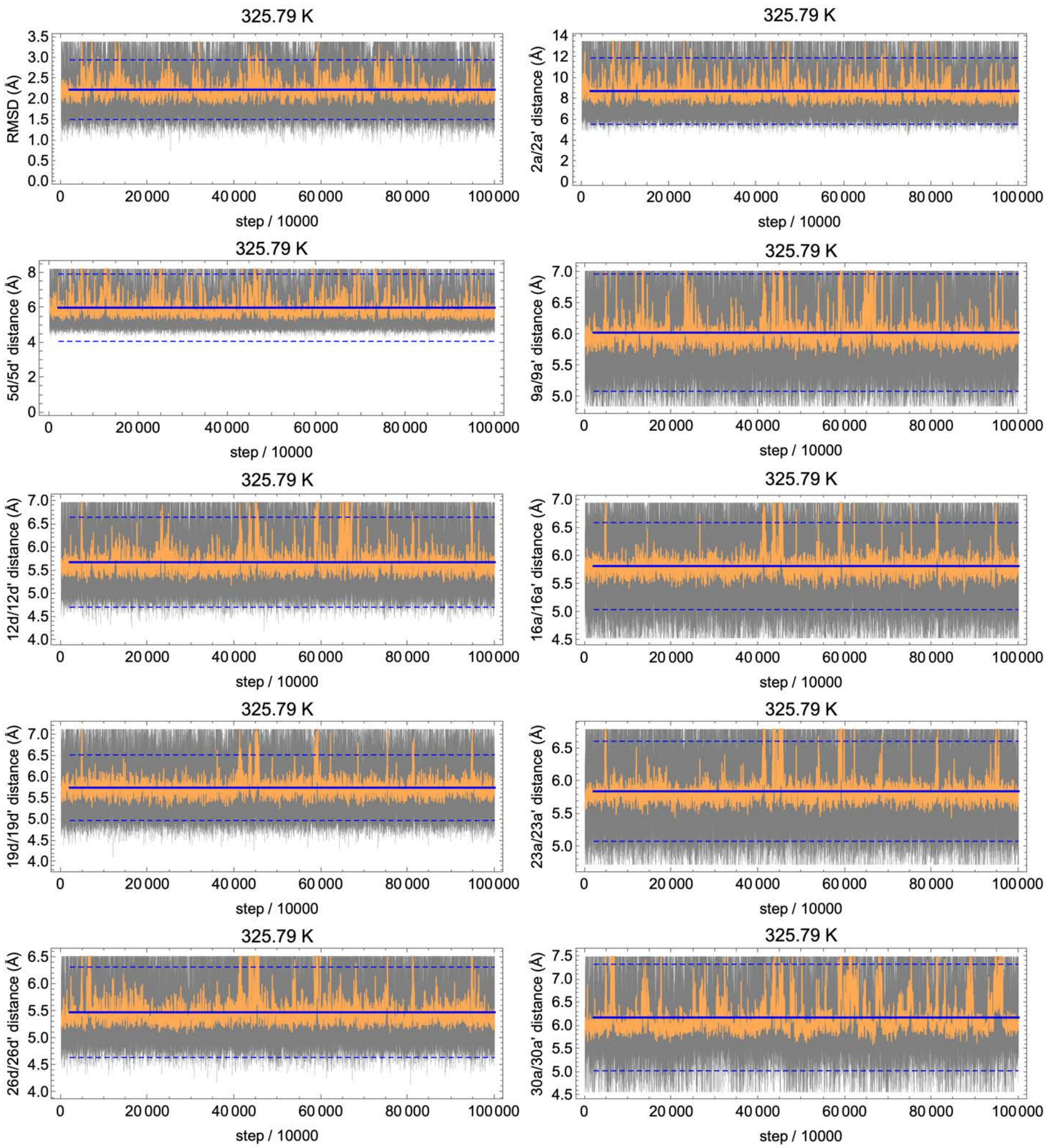

Measure at 325.79 K	Average (Å)	Standard Deviation (Å)	Slope (Å / μs)
RMSD	2.23126	0.723033	−0.0320372
5d/5d' distance	6.01268	1.92622	−0.112123
12d/12d' distance	5.68298	0.969515	0.0000946839
19d/19d' distance	5.75019	0.772349	0.00854362
26d/26d' distance	5.48264	0.836765	−0.0216245
2a/2a' distance	8.74547	3.182	−0.219352
9a/9a' distance	6.03324	0.940652	−0.00482615
16a/16a' distance	5.82281	0.775242	0.000832563
23a/23a' distance	5.84792	0.764191	−0.00297382
30a/30a' distance	6.17878	1.14892	0.0666997
Core Distance	6.17297	1.74	

Figure S196. Plots of RMSD and residue packing distances for T-REMD simulations of variant **20e/15g’-xx** at 325.79 K.

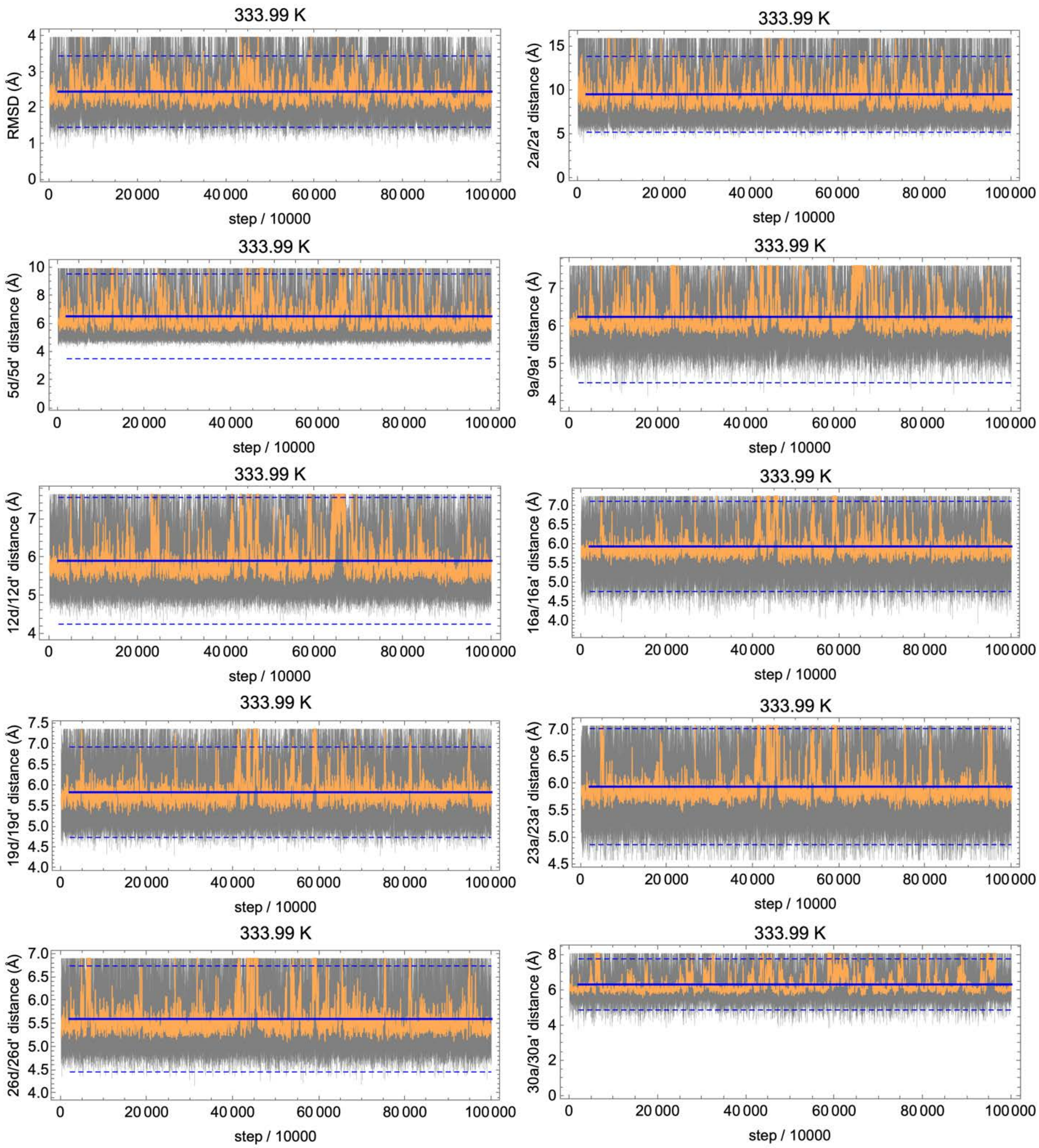

Measure at 333.99 K	Average (Å)	Standard Deviation (Å)	Slope (Å / μs)
RMSD	2.4518	0.993505	−0.0370053
5d/5d' distance	6.53886	3.01639	−0.128616
12d/12d' distance	5.91051	1.65974	0.0103721
19d/19d' distance	5.83761	1.09523	0.0151809
26d/26d' distance	5.6053	1.142	−0.0285537
2a/2a' distance	9.53244	4.31056	−0.286656
9a/9a' distance	6.24654	1.76215	−0.00773614
16a/16a' distance	5.9422	1.17386	0.00453763
23a/23a' distance	5.9444	1.07881	−0.0034354
30a/30a' distance	6.33648	1.4518	0.113991
Core Distance	6.4327	2.40708	

Figure S197. Plots of RMSD and residue packing distances for T-REMD simulations of variant **20e/15g'-xx** at 333.99 K.

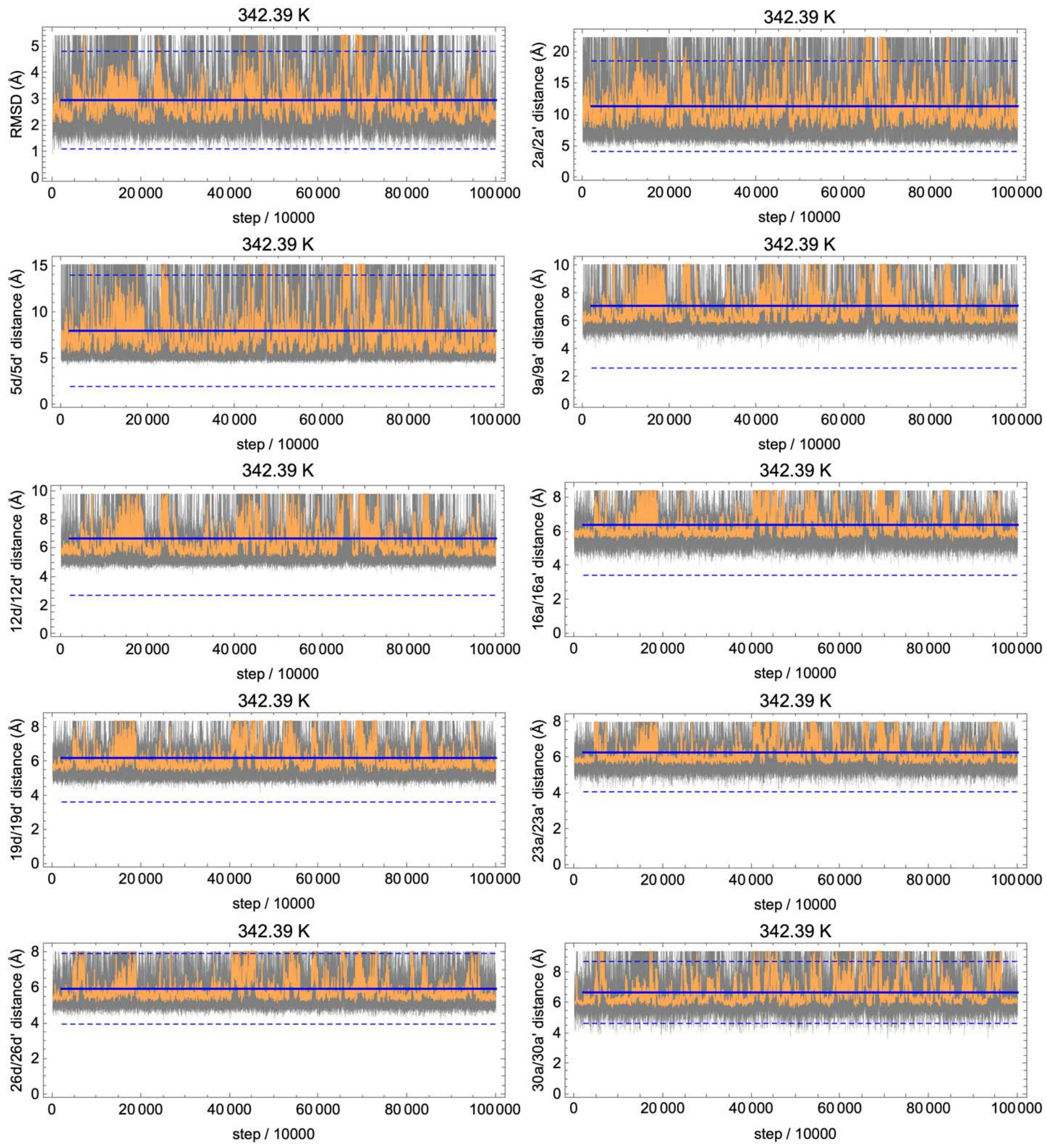

Measure at 342.39 K	Average (Å)	Standard Deviation (Å)	Slope (Å / μs)
RMSD	2.96839	1.85061	−0.0215995
5d/5d' distance	8.01076	6.02679	−0.147435
12d/12d' distance	6.70973	3.97458	0.0391166
19d/19d' distance	6.2119	2.58133	0.0313723
26d/26d' distance	5.93926	1.98313	−0.00538609
2a/2a' distance	11.3856	7.20807	−0.308761
9a/9a' distance	7.11656	4.47492	−0.0166216
16a/16a' distance	6.39848	2.96232	0.0207653
23a/23a' distance	6.28508	2.19612	0.0200263
30a/30a' distance	6.68702	2.0327	0.179077
Core Distance	7.19383	4.41157	

Figure S198. Plots of RMSD and residue packing distances for T-REMD simulations of variant **20e/15g'-xx** at 342.39 K.

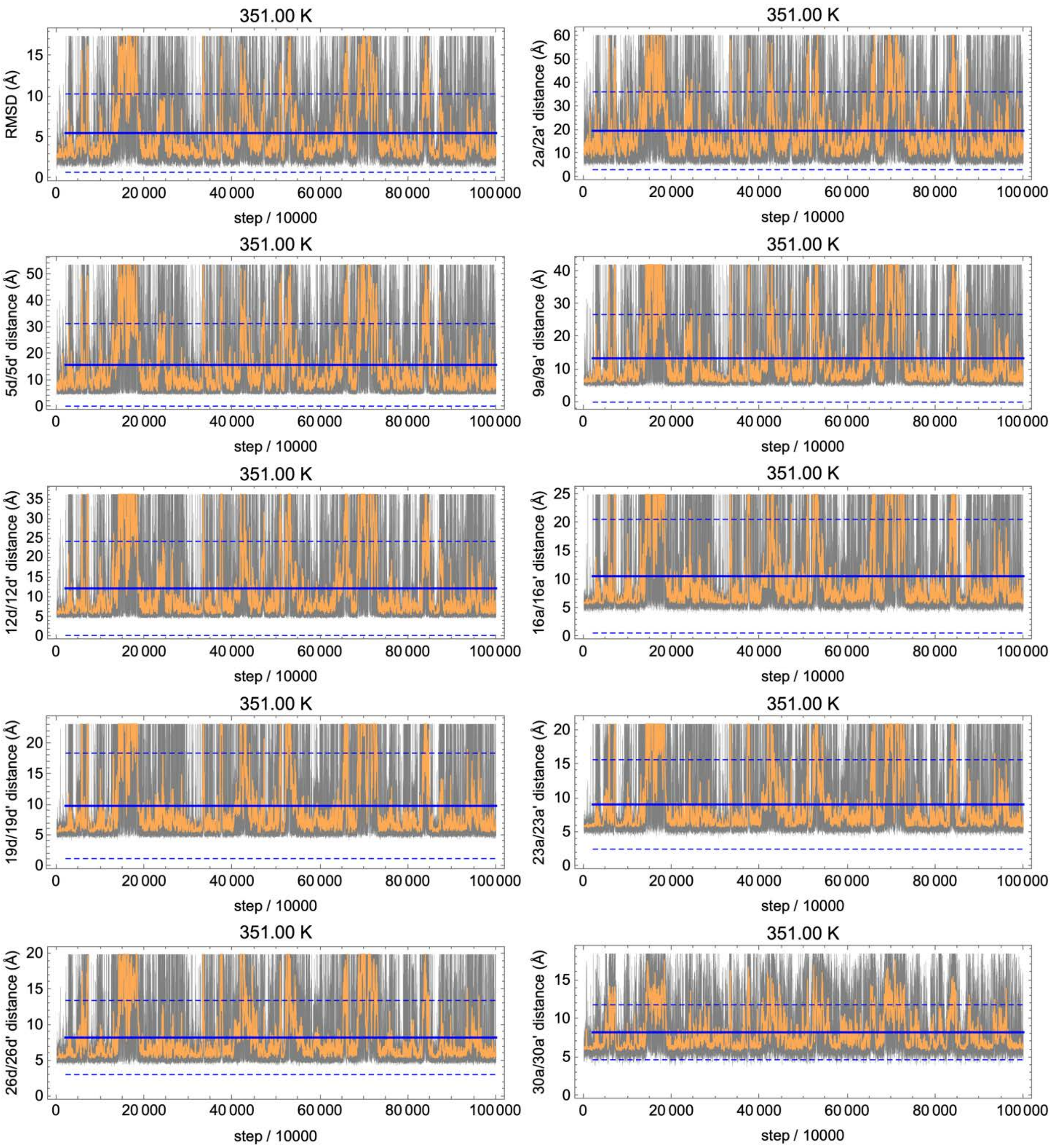

Measure at 351.00 K	Average (Å)	Standard Deviation (Å)	Slope (Å / μs)
RMSD	5.52154	4.8026	−0.504853
5d/5d' distance	15.8131	15.6034	−1.60768
12d/12d' distance	12.2376	12.0073	−1.09429
19d/19d' distance	9.80641	8.59775	−0.750781
26d/26d' distance	8.23984	5.19548	−0.41939
2a/2a' distance	19.6997	16.5506	−1.8567
9a/9a' distance	13.3354	13.4181	−1.24826
16a/16a' distance	10.6054	9.9948	−0.869175
23a/23a' distance	9.07561	6.58967	−0.536014
30a/30a' distance	8.2346	3.56917	−0.0514553
Core Distance	11.8942	11.6321	

Figure S199. Plots of RMSD and residue packing distances for T-REMD simulations of variant **20e/15g’-xx** at 351.00 K.

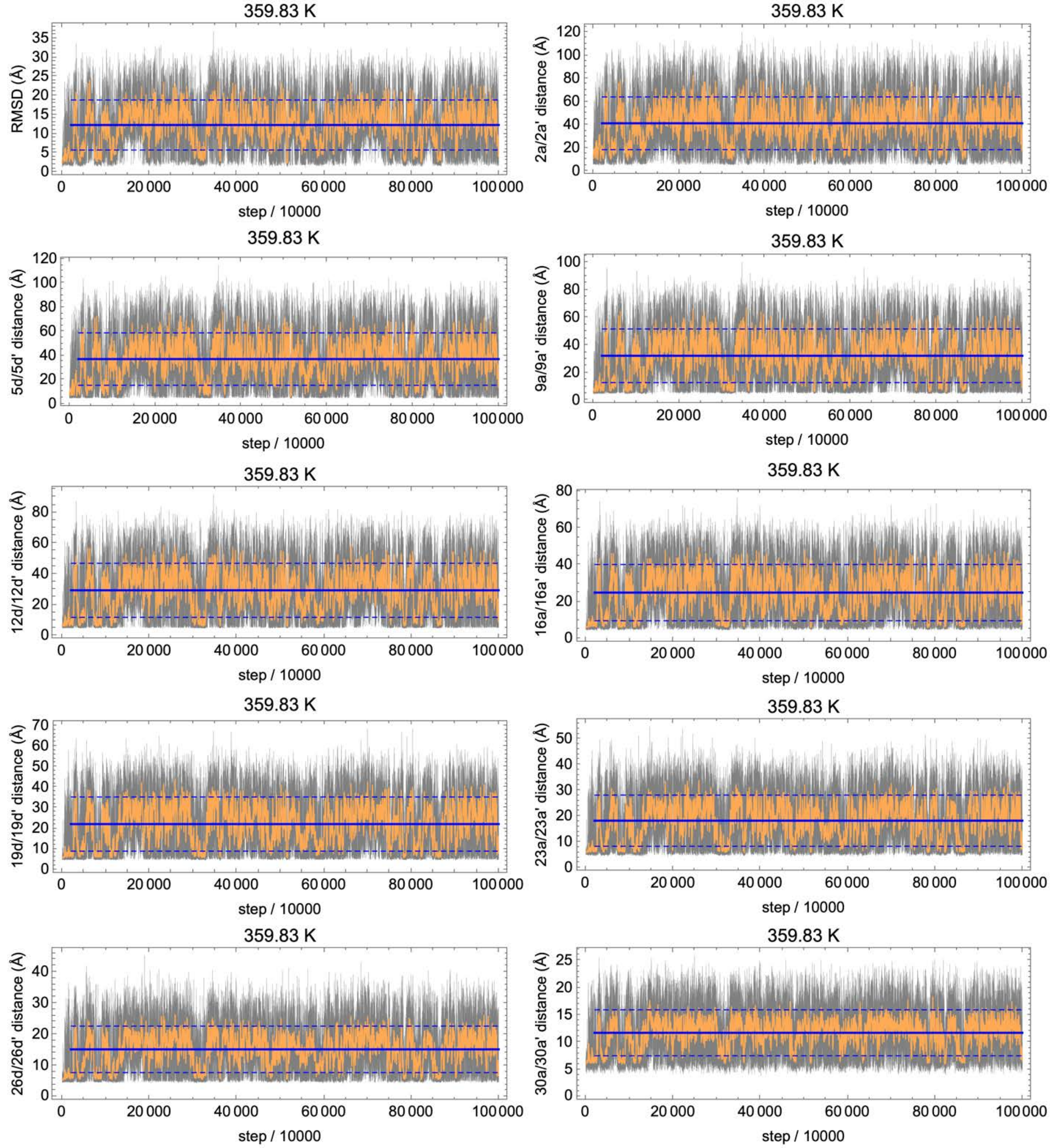

Measure at 359.83 K	Average (Å)	Standard Deviation (Å)	Slope (Å / μs)
RMSD	12.2939	6.54328	0.726585
5d/5d' distance	37.0592	21.7012	2.38976
12d/12d' distance	29.3301	17.5663	1.81248
19d/19d' distance	22.0839	13.1798	1.23223
26d/26d' distance	15.1614	7.44434	0.845995
2a/2a' distance	41.2374	22.7689	2.29403
9a/9a' distance	32.2285	19.4046	2.15923
16a/16a' distance	24.8507	15.2907	1.36456
23a/23a' distance	18.2368	9.92804	1.01055
30a/30a' distance	11.7306	4.21684	0.520508
Core Distance	25.7687	18.426	

Figure S200. Plots of RMSD and residue packing distances for T-REMD simulations of variant **20e/15g'-xx** at 359.83 K.

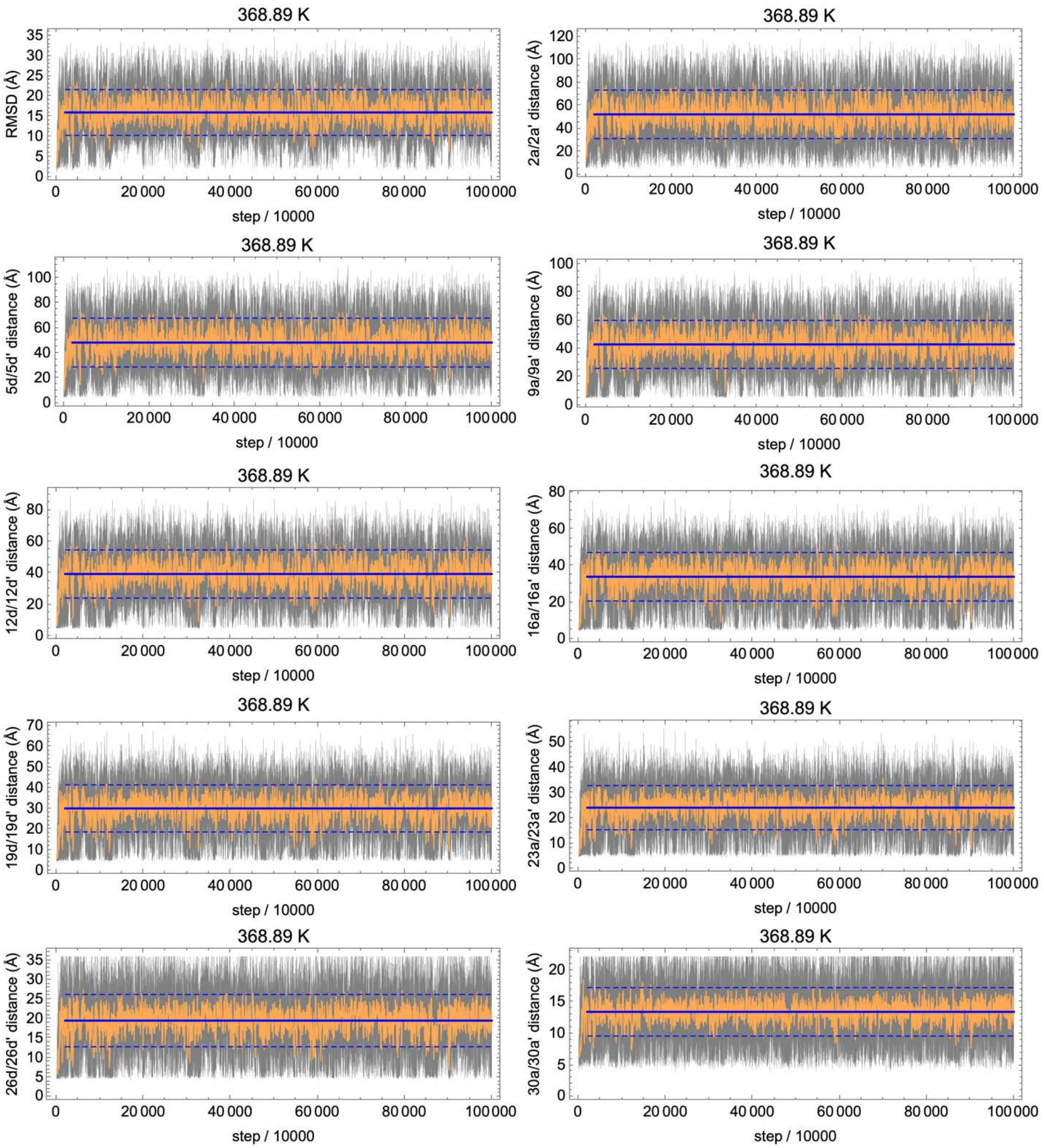

Measure at 368.89 K	Average (Å)	Standard Deviation (Å)	Slope (Å / μs)
RMSD	15.9877	5.66343	−0.0688613
5d/5d' distance	48.3836	19.5172	−0.245569
12d/12d' distance	39.3747	15.2987	−0.193891
19d/19d' distance	30.0846	11.3895	−0.150509
26d/26d' distance	19.5295	6.69704	0.111733
2a/2a' distance	52.4369	21.1076	−0.35007
9a/9a' distance	42.9401	17.0168	−0.202834
16a/16a' distance	33.9125	13.2377	−0.24502
23a/23a' distance	24.1311	8.76369	0.00281312
30a/30a' distance	13.4165	3.81789	0.0906976
Core Distance	33.8011	18.8335	

Figure S201. Plots of RMSD and residue packing distances for T-REMD simulations of variant **20e/15g’-xx** at 368.89 K.

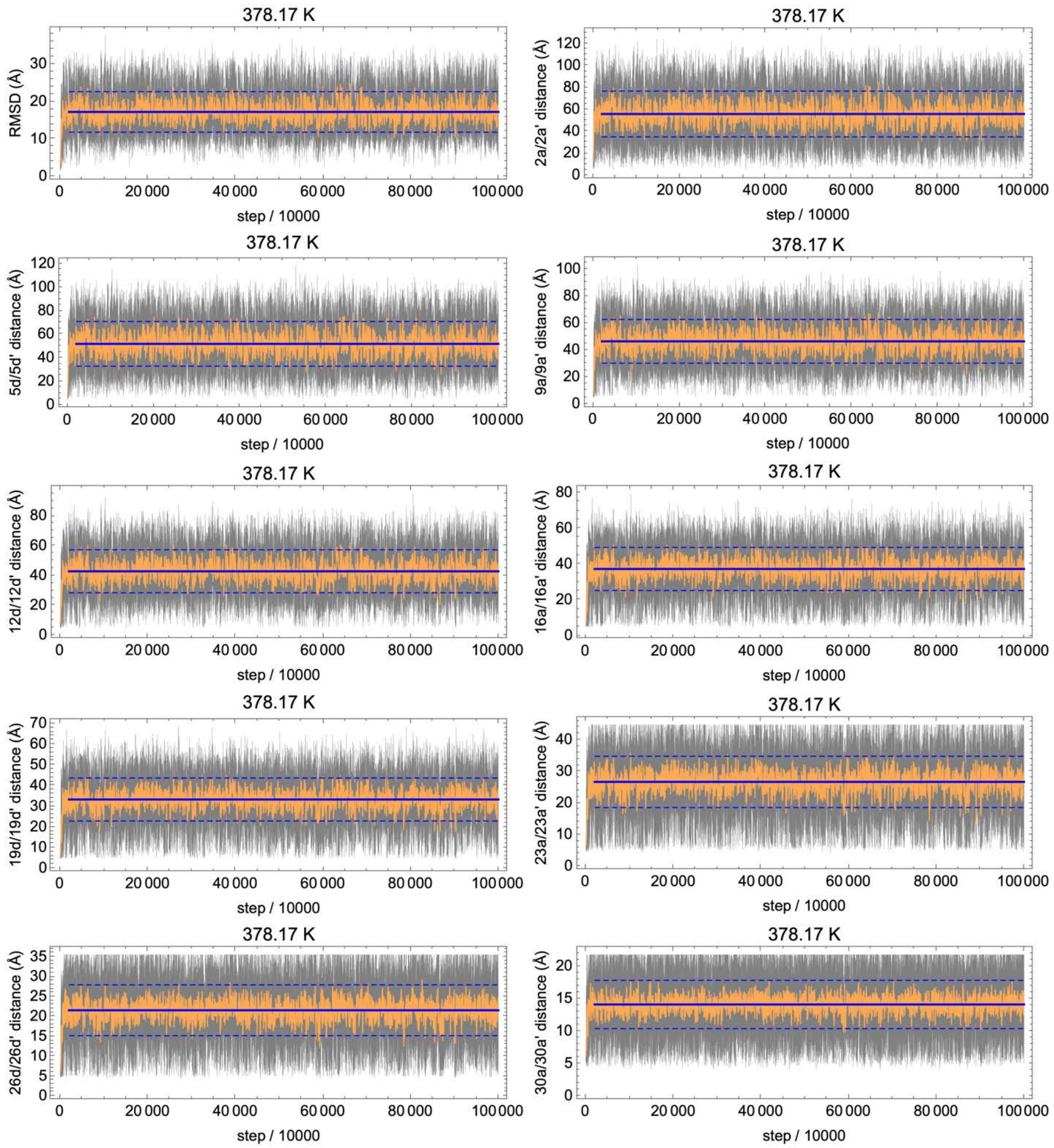

Measure at 378.17 K	Average (Å)	Standard Deviation (Å)	Slope (Å / μs)
RMSD	17.2111	5.4061	−0.111378
5d/5d' distance	51.789	19.0163	−0.37515
12d/12d' distance	42.7795	14.4075	−0.292704
19d/19d' distance	33.2558	10.388	−0.137514
26d/26d' distance	21.5442	6.38611	0.025215
2a/2a' distance	55.7459	20.9209	−0.447232
9a/9a' distance	46.3914	16.2337	−0.346861
16a/16a' distance	37.2771	12.0547	−0.263861
23a/23a' distance	26.6707	8.13301	−0.0802231
30a/30a' distance	14.0891	3.71879	0.0359913
Core Distance	36.6159	18.9131	

Figure S202. Plots of RMSD and residue packing distances for T-REMD simulations of variant **20e/15g'-xx** at 378.17 K.

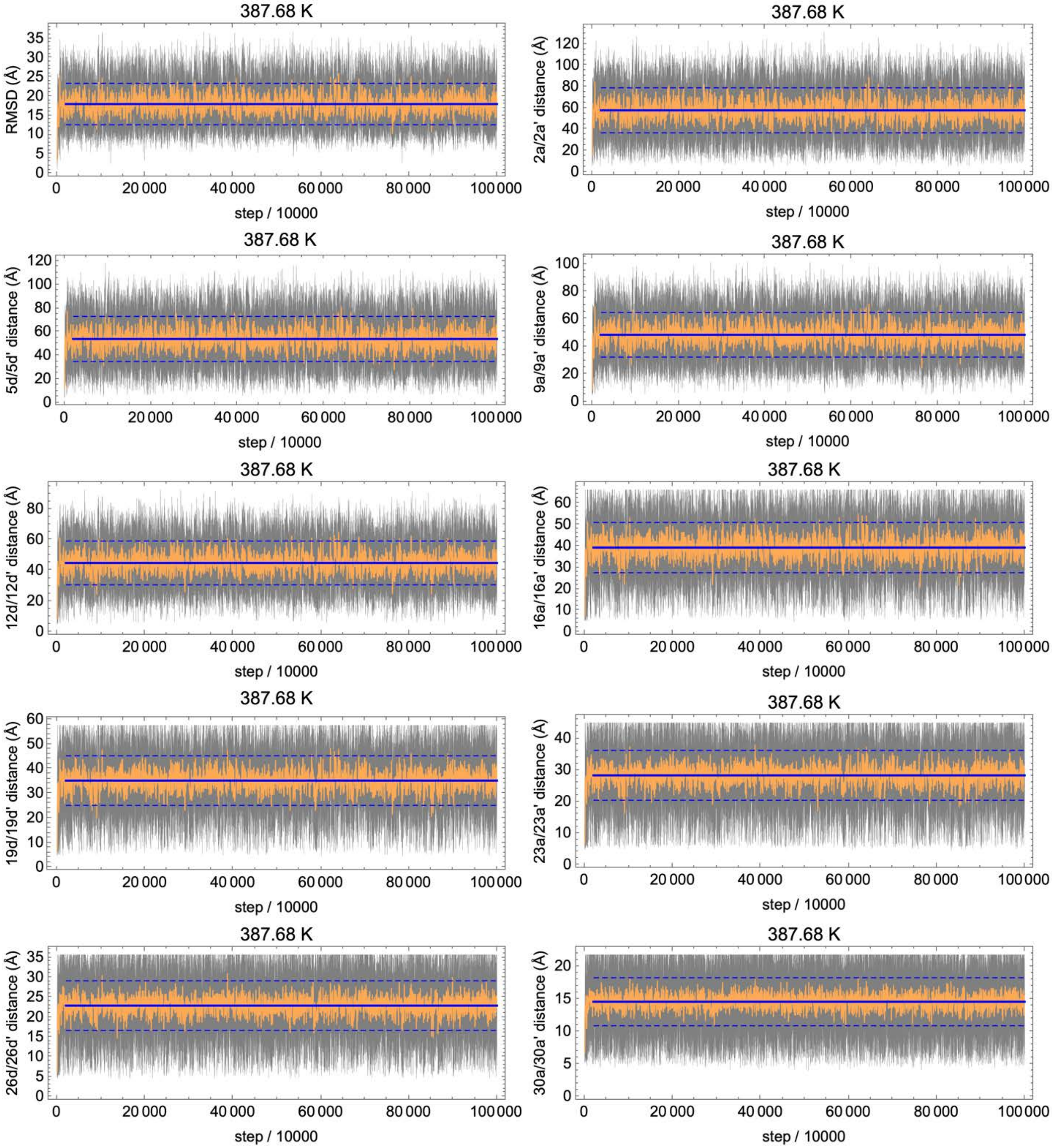

Measure at 387.68 K	Average (Å)	Standard Deviation (Å)	Slope (Å / μs)
RMSD	17.9758	5.37657	−0.0483532
5d/5d' distance	53.8648	19.1206	−0.251623
12d/12d' distance	44.812	14.2613	−0.0542309
19d/19d' distance	35.1576	10.0615	0.0157598
26d/26d' distance	22.8864	6.24258	−0.0235592
2a/2a' distance	57.7983	21.1005	−0.362002
9a/9a' distance	48.462	16.1741	−0.135916
16a/16a' distance	39.2304	11.7164	−0.0578598
23a/23a' distance	28.2875	7.88648	−0.0144344
30a/30a' distance	14.5603	3.68481	−0.058992
Core Distance	38.3399	19.1755	

Figure S203. Plots of RMSD and residue packing distances for T-REMD simulations of variant **20e/15g’-xx** at 387.68 K.

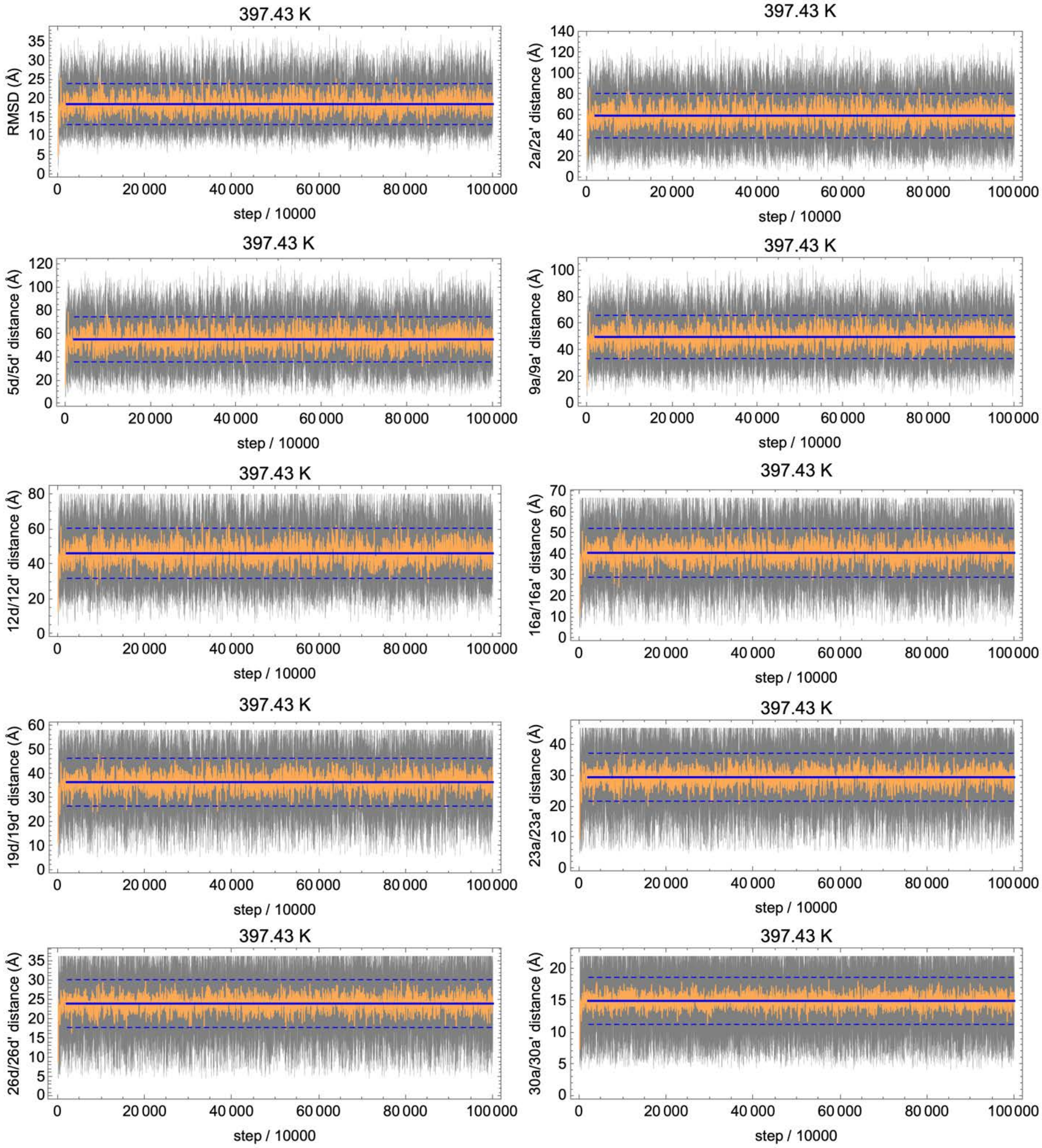

Measure at 397.43 K	Average (Å)	Standard Deviation (Å)	Slope (Å / μs)
RMSD	18.5711	5.40223	0.0663654
5d/5d' distance	55.446	19.3587	0.14435
12d/12d' distance	46.3306	14.351	0.271877
19d/19d' distance	36.5989	9.94685	0.179907
26d/26d' distance	24.0218	6.18492	0.0822555
2a/2a' distance	59.3879	21.4276	0.119845
9a/9a' distance	49.9986	16.3167	0.250154
16a/16a' distance	40.6861	11.6345	0.203268
23a/23a' distance	29.583	7.78523	0.104293
30a/30a' distance	14.9889	3.68766	−0.0130343
Core Distance	39.6713	19.4588	

Figure S204. Plots of RMSD and residue packing distances for T-REMD simulations of variant **20e/15g'-xx** at 397.43 K.

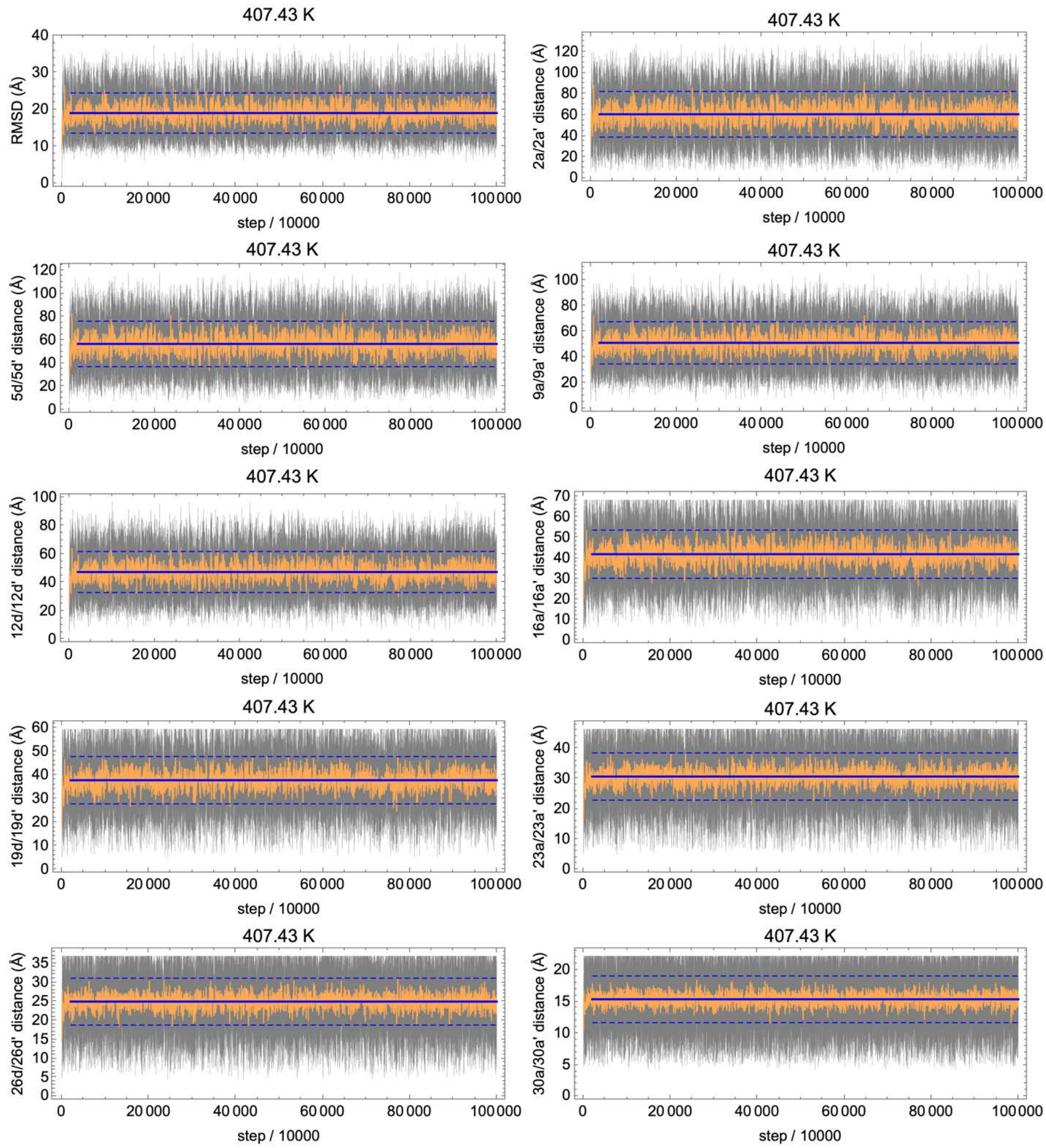

Measure at 407.43 K	Average (Å)	Standard Deviation (Å)	Slope (Å / μs)
RMSD	19.0449	5.4408	0.0226299
5d/5d' distance	56.6415	19.593	0.00981577
12d/12d' distance	47.4918	14.4758	0.120692
19d/19d' distance	37.7897	10.0241	0.138862
26d/26d' distance	24.9333	6.1617	0.0365547
2a/2a' distance	60.5929	21.7012	−0.0736815
9a/9a' distance	51.1615	16.5016	0.109861
16a/16a' distance	41.8834	11.731	0.131436
23a/23a' distance	30.6918	7.80745	0.0798802
30a/30a' distance	15.3589	3.6891	−0.0704315
Core Distance	40.7272	19.702	

Figure S205. Plots of RMSD and residue packing distances for T-REMD simulations of variant **20e/15g'-xx** at 407.43 K.

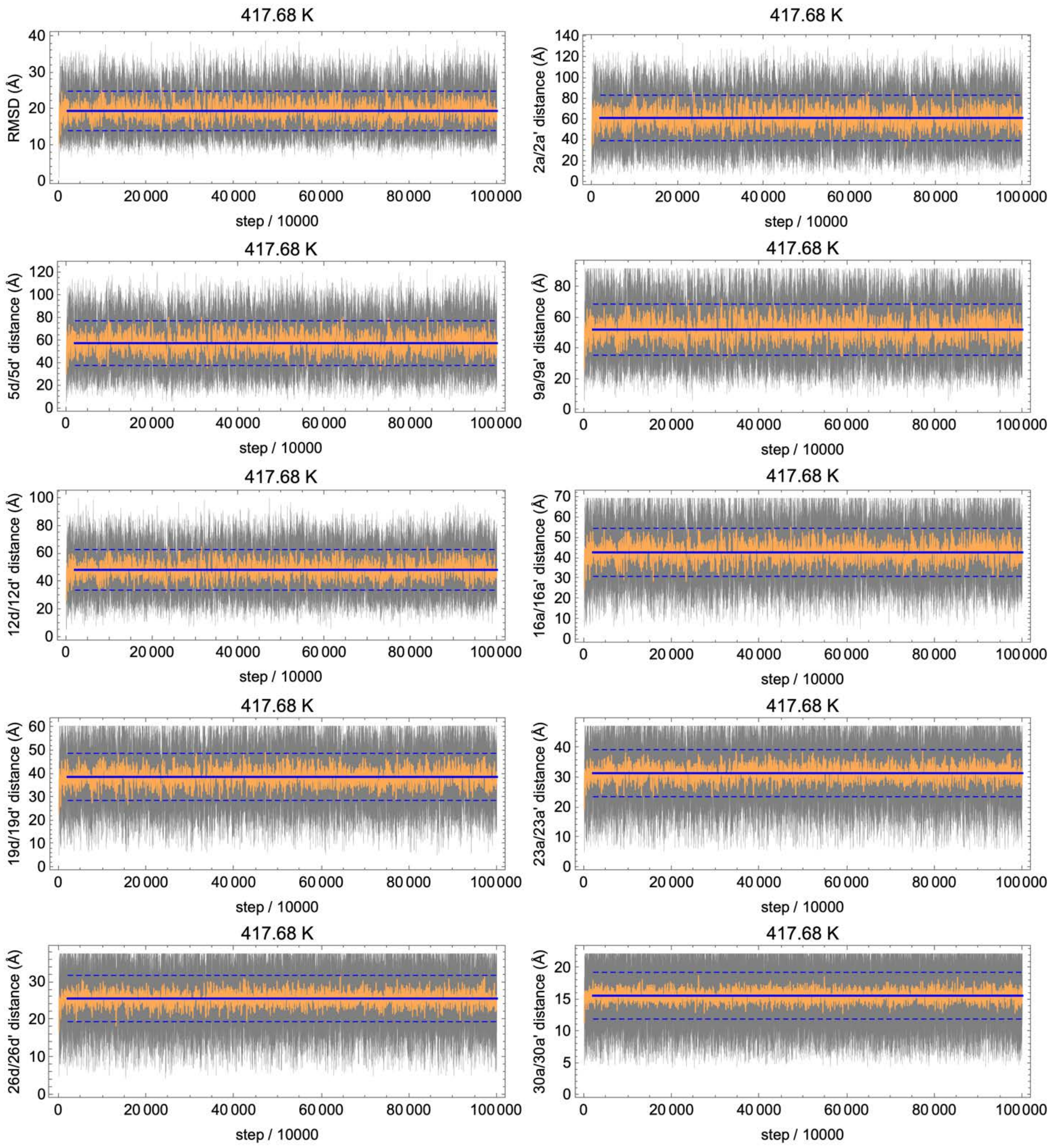

Measure at 417.68 K	Average (Å)	Standard Deviation (Å)	Slope (Å / μs)
RMSD	19.4335	5.45271	0.0331606
5d/5d' distance	57.6198	19.7404	0.0000732002
12d/12d' distance	48.3983	14.5834	0.128657
19d/19d' distance	38.727	10.0846	0.16861
26d/26d' distance	25.6391	6.15646	0.0808557
2a/2a' distance	61.5639	21.8965	−0.0532454
9a/9a' distance	52.1026	16.6147	0.0754905
16a/16a' distance	42.8132	11.8205	0.127306
23a/23a' distance	31.5239	7.87319	0.1595
30a/30a' distance	15.6362	3.67782	−0.0270547
Core Distance	41.5582	19.9012	

Figure S206. Plots of RMSD and residue packing distances for T-REMD simulations of variant **20e/15g'-xx** at 417.68 K.

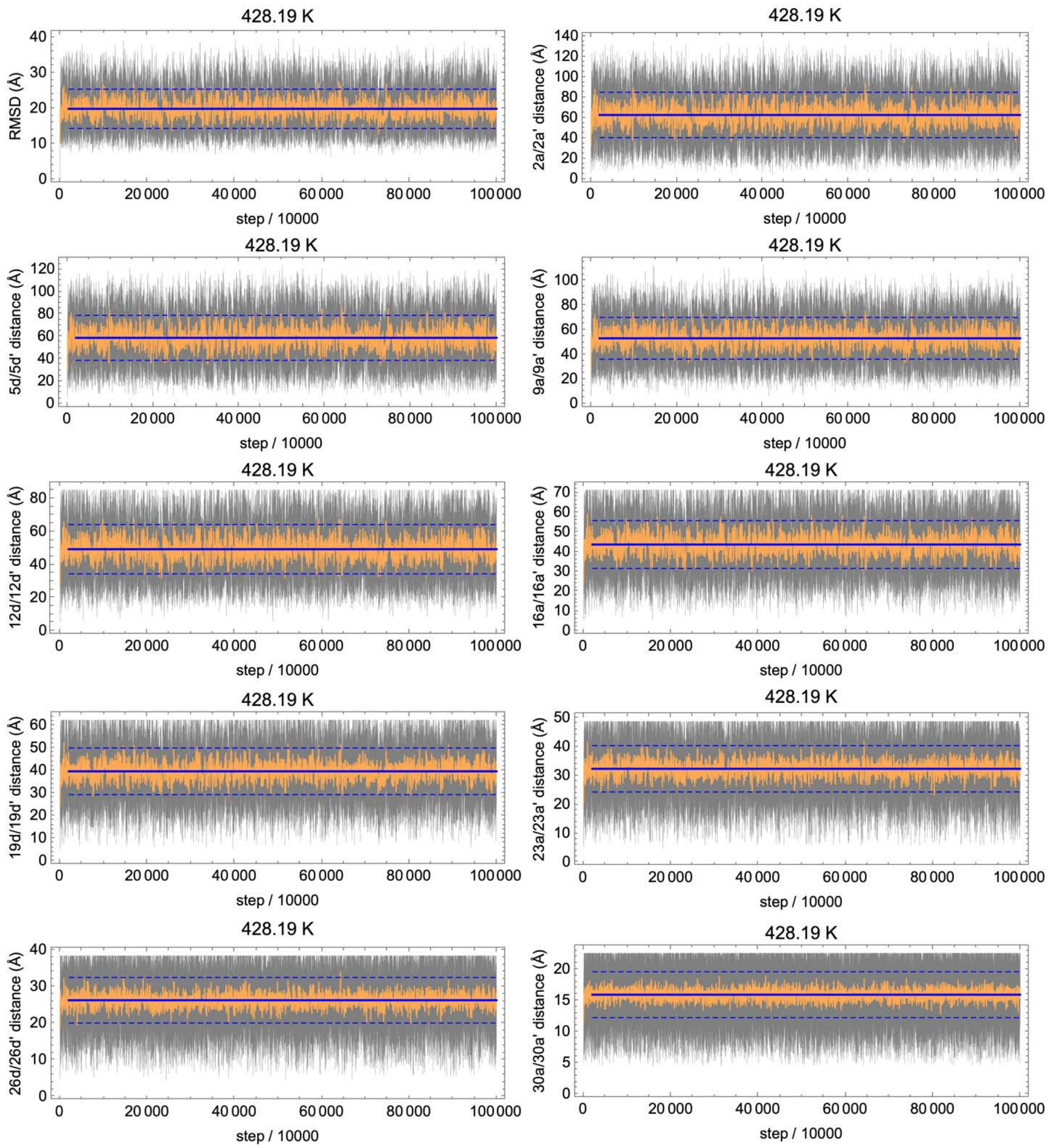

Measure at 428.19 K	Average (Å)	Standard Deviation (Å)	Slope (Å / μs)
RMSD	19.8567	5.53596	0.00850255
5d/5d' distance	58.7207	20.0853	−0.0460464
12d/12d' distance	49.3507	14.9153	0.0107931
19d/19d' distance	39.6107	10.3275	0.124769
26d/26d' distance	26.2627	6.20651	0.0503929
2a/2a' distance	62.7691	22.2487	−0.0175935
9a/9a' distance	53.0844	16.9494	−0.0678626
16a/16a' distance	43.7056	12.1036	0.0845281
23a/23a' distance	32.3191	8.01589	0.0782772
30a/30a' distance	15.9092	3.67834	−0.0280679
Core Distance	42.4147	20.2612	

Figure S207. Plots of RMSD and residue packing distances for T-REMD simulations of variant **20e/15g'-xx** at 428.19 K.

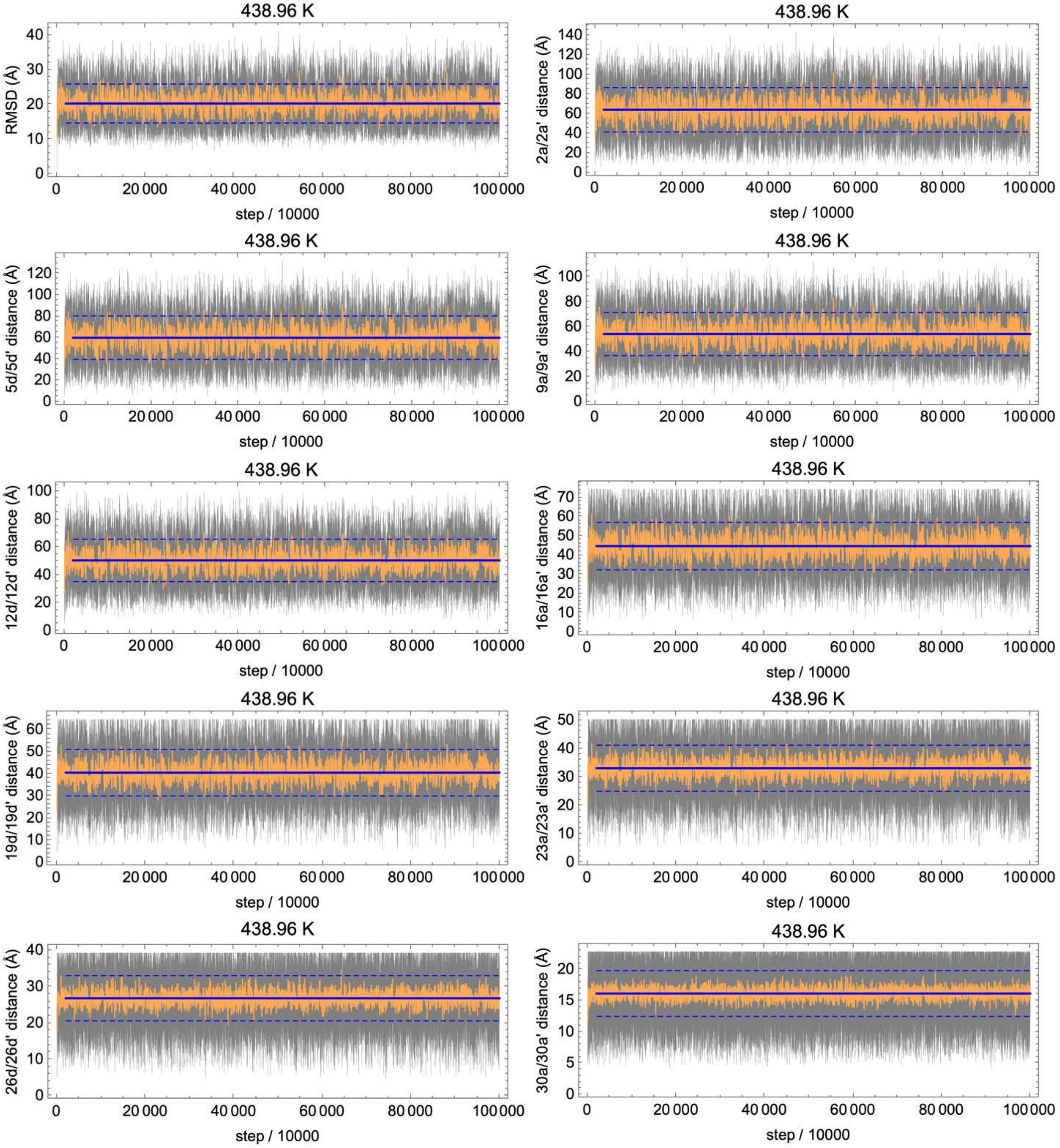

Measure at 438.96 K	Average (Å)	Standard Deviation (Å)	Slope (Å / μs)
RMSD	20.2995	5.60128	0.0983794
5d/5d' distance	59.8957	20.3757	0.154767
12d/12d' distance	50.3917	15.2102	0.277737
19d/19d' distance	40.5201	10.5211	0.301193
26d/26d' distance	26.8485	6.23049	0.099562
2a/2a' distance	64.0043	22.5806	0.259511
9a/9a' distance	54.2019	17.264	0.132274
16a/16a' distance	44.6802	12.3491	0.270855
23a/23a' distance	33.1051	8.11758	0.1676
30a/30a' distance	16.1411	3.65575	0.0385939
Core Distance	43.3098	20.631	

Figure S208. Plots of RMSD and residue packing distances for T-REMD simulations of variant **20e/15g'-xx** at 438.96 K.

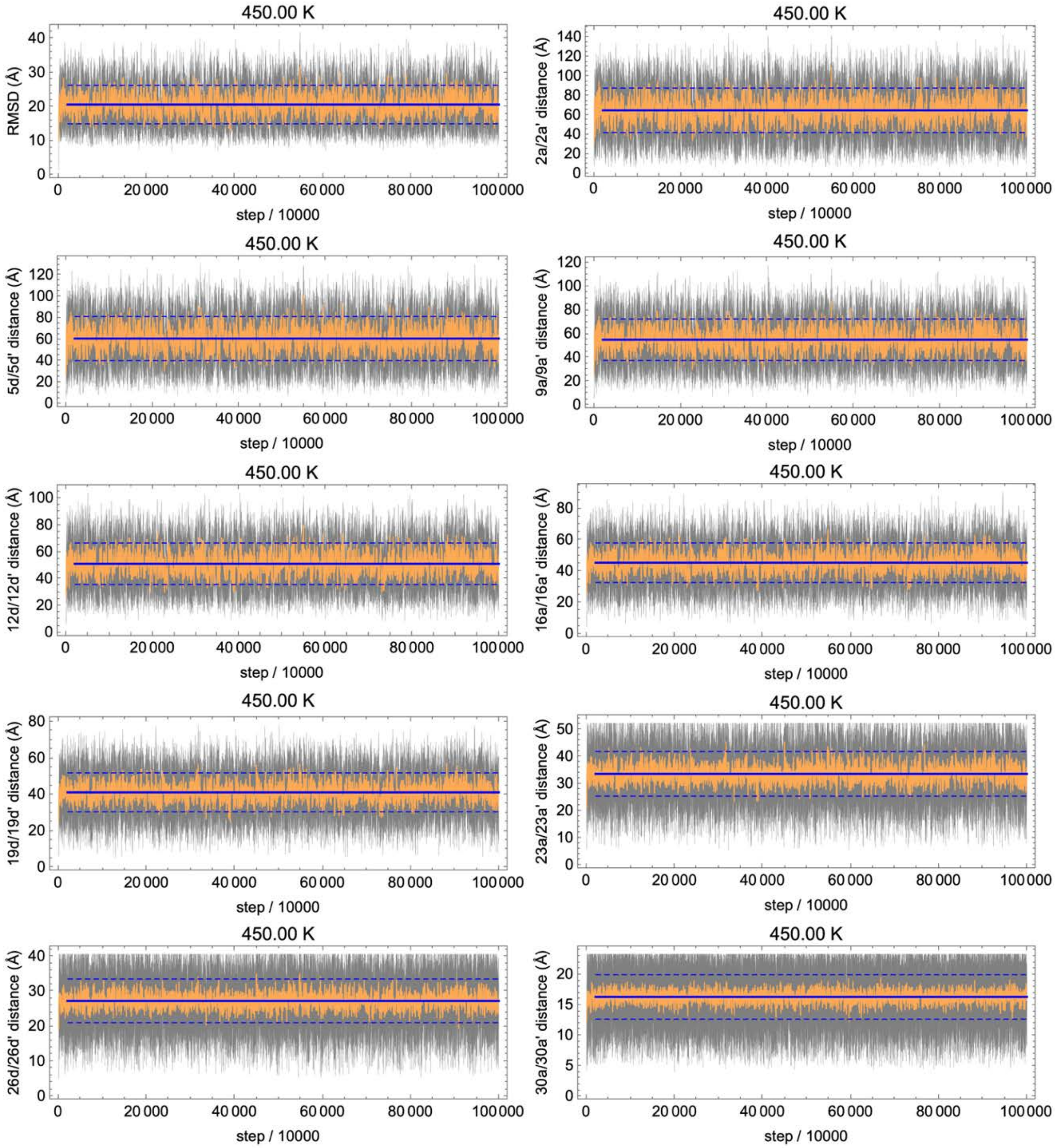

Measure at 450.00 K	Average (Å)	Standard Deviation (Å)	Slope (Å / μs)
RMSD	20.6437	5.64598	0.0646762
5d/5d' distance	60.762	20.6173	0.0596504
12d/12d' distance	51.1827	15.4396	0.156768
19d/19d' distance	41.2303	10.6927	0.275166
26d/26d' distance	27.2735	6.24586	0.0237976
2a/2a' distance	64.8966	22.8412	0.176069
9a/9a' distance	55.0561	17.5155	0.037392
16a/16a' distance	45.4673	12.585	0.266975
23a/23a' distance	33.6962	8.23304	0.114775
30a/30a' distance	16.3317	3.65054	−0.0302306
Core Distance	43.9885	20.9166	

Figure S209. Plots of RMSD and residue packing distances for T-REMD simulations of variant **20e/15g'-xx** at 450.00 K.

(return to Table of Contents)

s20e/15g'-x4x

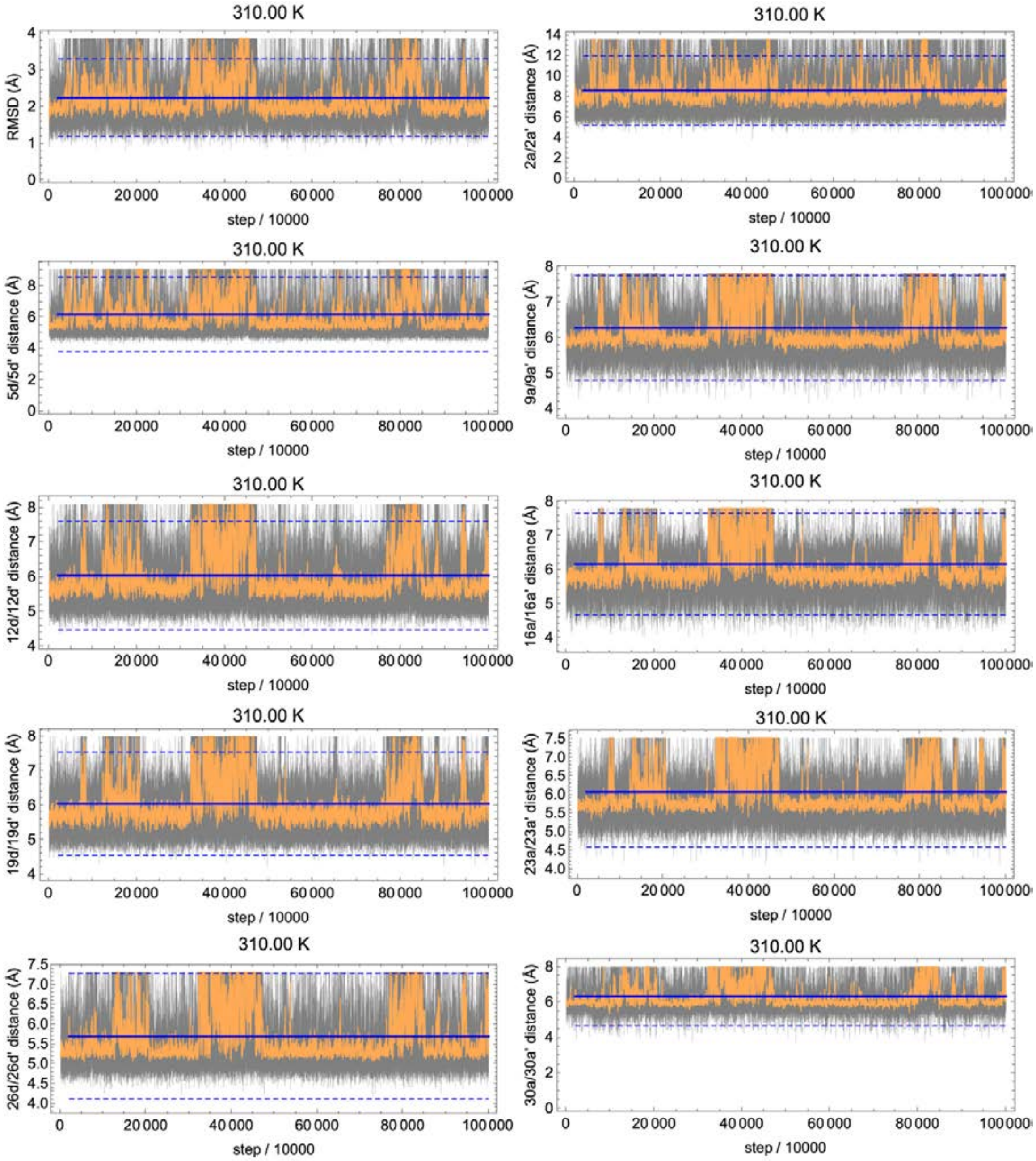

Measure at 310.00 K	Average (Å)	Standard Deviation (Å)	Slope (Å / μs)
RMSD	2.253	1.05535	−0.0667694
5d/5d' distance	6.19938	2.39905	−0.19463
12d/12d' distance	6.04357	1.56717	−0.0303918
19d/19d' distance	6.05676	1.49457	−0.0157812
26d/26d' distance	5.70471	1.58257	−0.015504
2a/2a' distance	8.62923	3.38428	−0.285354
9a/9a' distance	6.2844	1.47567	−0.0221388
16a/16a' distance	6.17325	1.49251	−0.00692598
23a/23a' distance	6.0828	1.47689	−0.0359807
30a/30a' distance	6.35893	1.66701	−0.000347719
Core Distance	6.39256	2.09954	

Figure S210. Plots of RMSD and residue packing distances for T-REMD simulations of variant **s20e/15g'-x4x** at 310.00 K.

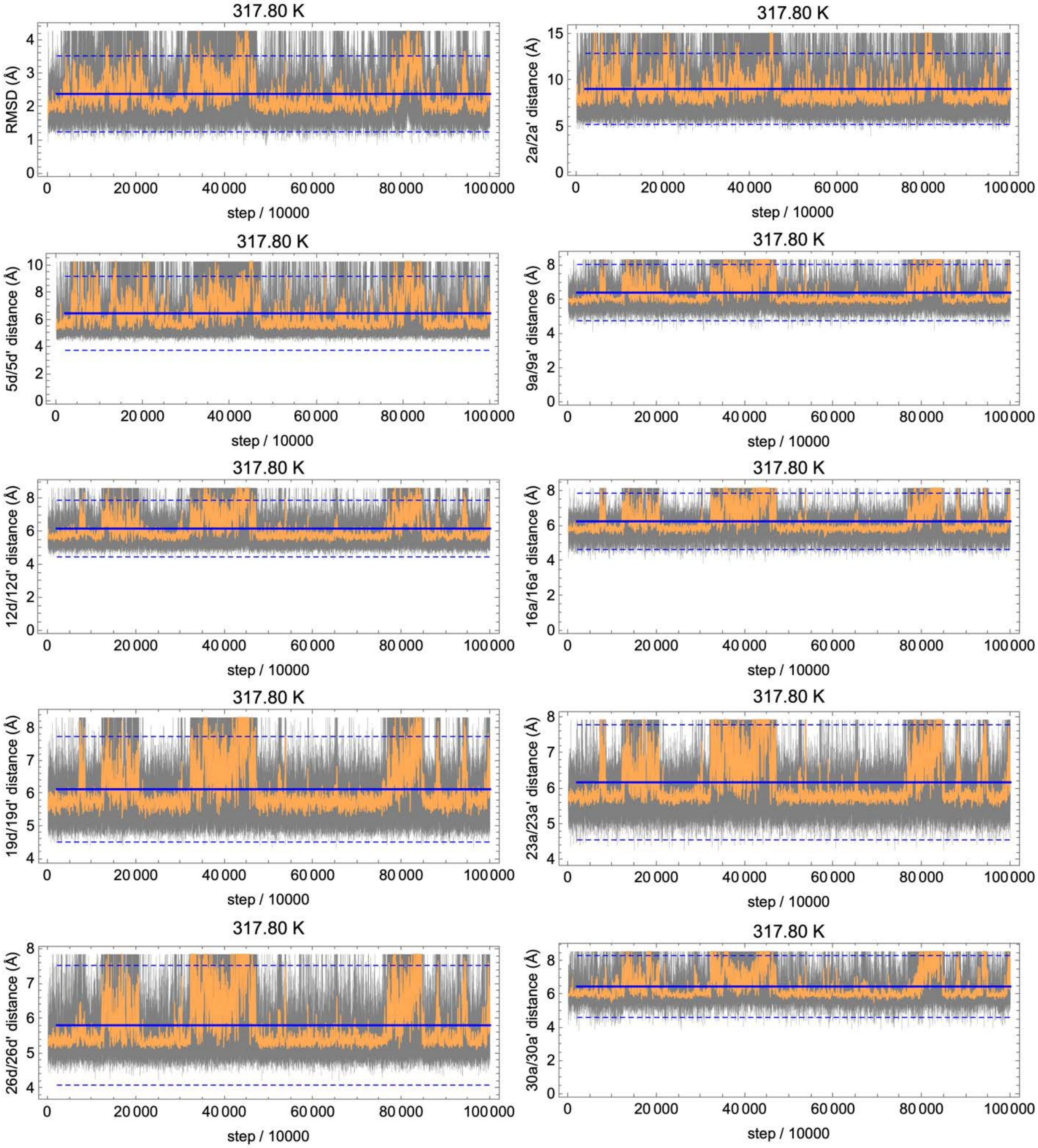

Measure at 317.80 K	Average (Å)	Standard Deviation (Å)	Slope (Å / μs)
RMSD	2.39216	1.14028	−0.0844578
5d/5d' distance	6.48694	2.71867	−0.25782
12d/12d' distance	6.1867	1.71559	−0.0207299
19d/19d' distance	6.1356	1.61587	−0.0024158
26d/26d' distance	5.81321	1.71924	−0.00695726
2a/2a' distance	9.07532	3.83894	−0.419012
9a/9a' distance	6.40369	1.64577	−0.0137621
16a/16a' distance	6.26696	1.61429	0.00139368
23a/23a' distance	6.17548	1.61778	−0.0397397
30a/30a' distance	6.47916	1.863	0.0217885
Core Distance	6.55812	2.34556	

Figure S211. Plots of RMSD and residue packing distances for T-REMD simulations of variant **s20e/15g'-x4x** at 317.80 K.

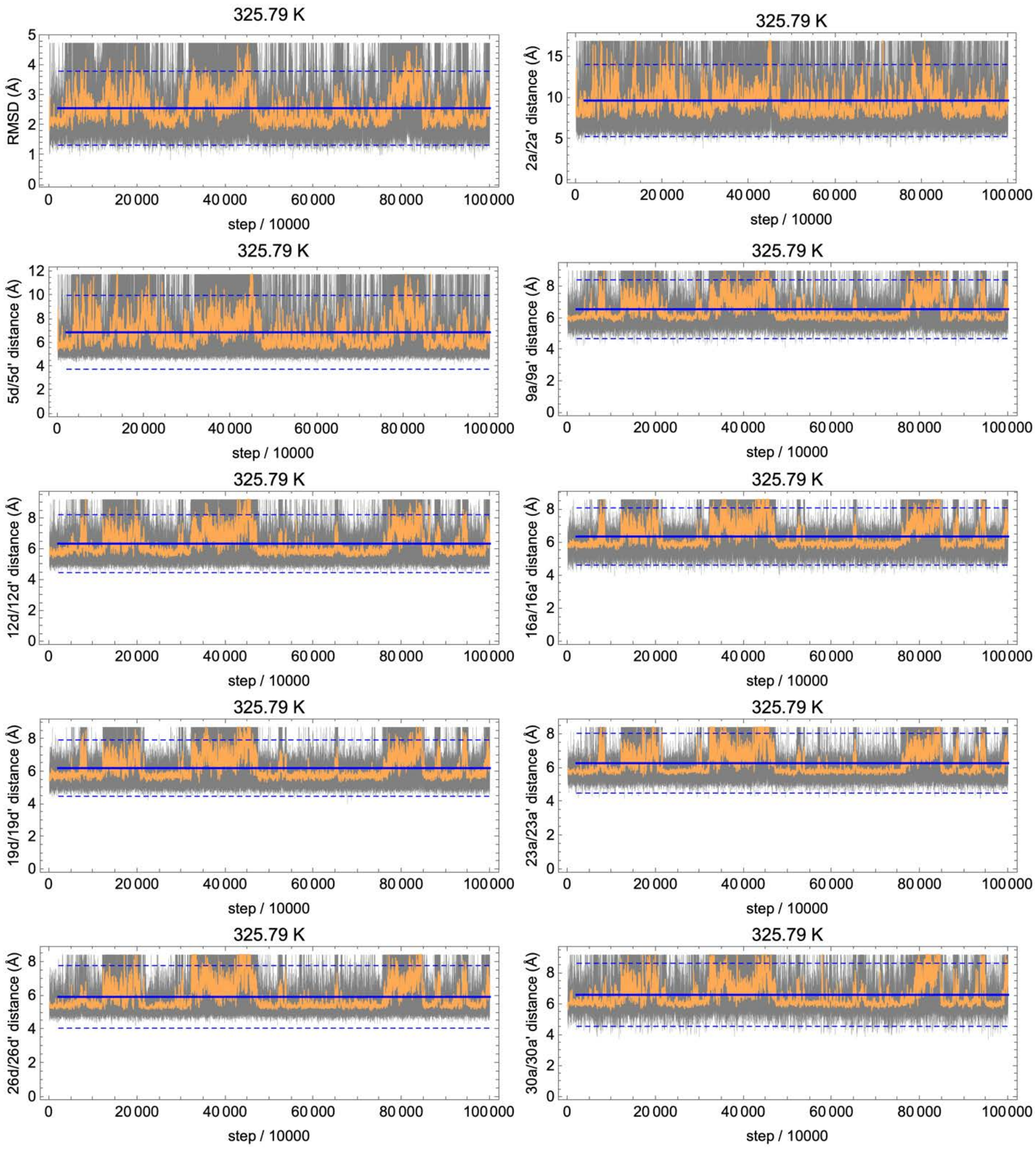

Measure at 325.79 K	Average (Å)	Standard Deviation (Å)	Slope (Å / μs)
RMSD	2.56987	1.23624	−0.100171
5d/5d' distance	6.88173	3.11776	−0.324171
12d/12d' distance	6.3578	1.87991	−0.022962
19d/19d' distance	6.2197	1.73172	−0.00776993
26d/26d' distance	5.93099	1.85591	−0.00472726
2a/2a' distance	9.68609	4.37954	−0.534875
9a/9a' distance	6.5571	1.85782	−0.0200991
16a/16a' distance	6.36743	1.73229	−0.0158493
23a/23a' distance	6.27715	1.76938	−0.0428033
30a/30a' distance	6.60771	2.04191	0.0163297
Core Distance	6.76508	2.64153	

Figure S212. Plots of RMSD and residue packing distances for T-REMD simulations of variant **s20e/15g'-x4x** at 325.79 K.

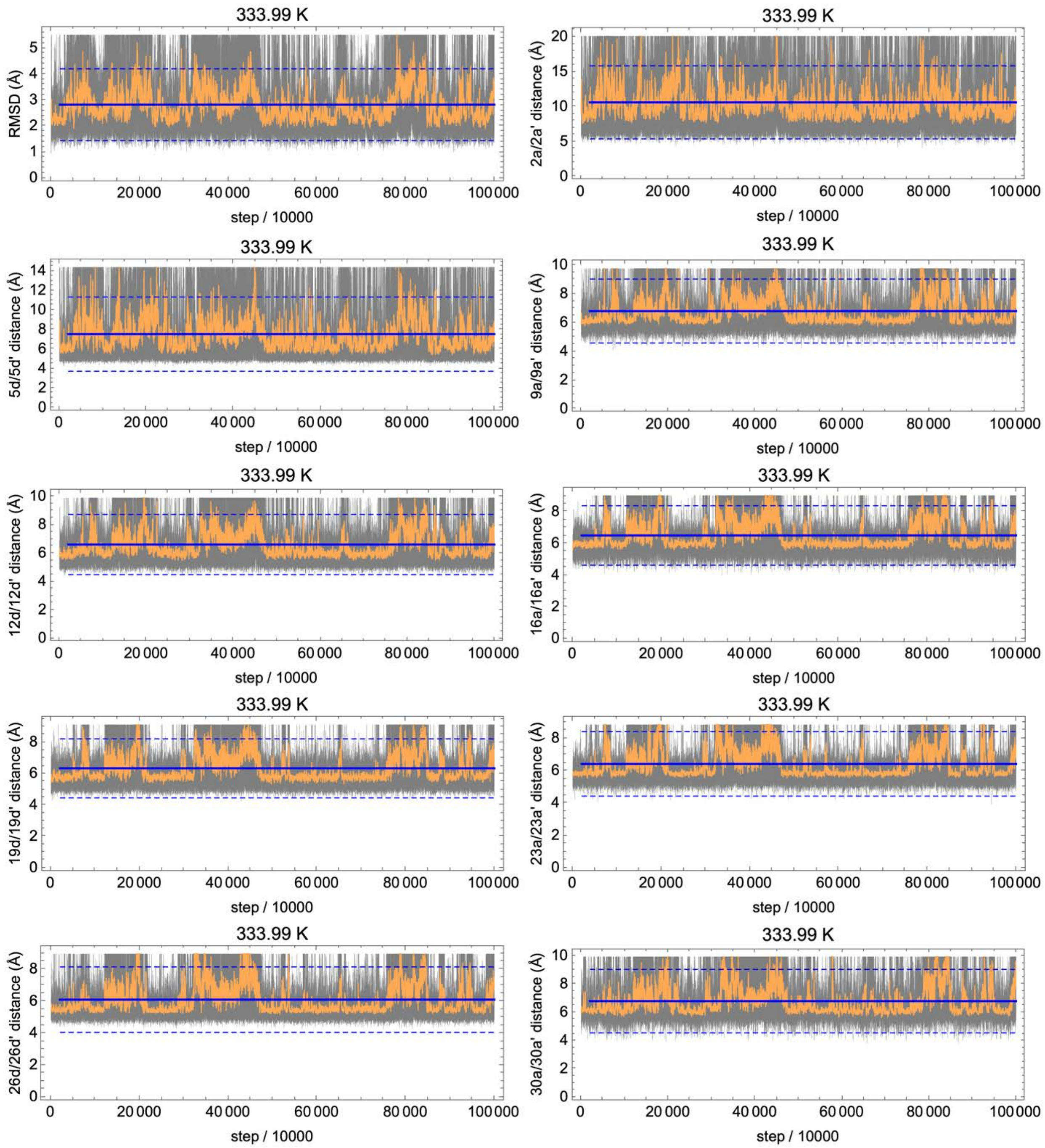

Measure at 333.99 K	Average (Å)	Standard Deviation (Å)	Slope (Å / μs)
RMSD	2.82019	1.3822	−0.120389
5d/5d' distance	7.52592	3.80236	−0.380198
12d/12d' distance	6.60101	2.12228	−0.0201537
19d/19d' distance	6.33544	1.87904	−0.0173319
26d/26d' distance	6.09435	2.04784	−0.00419792
2a/2a' distance	10.6086	5.23057	−0.686744
9a/9a' distance	6.79802	2.21927	−0.00473605
16a/16a' distance	6.50858	1.87546	−0.0232376
23a/23a' distance	6.42025	1.99238	−0.0558517
30a/30a' distance	6.79208	2.2475	0.010736
Core Distance	7.07603	3.10622	

Figure S213. Plots of RMSD and residue packing distances for T-REMD simulations of variant **s20e/15g'-x4x** at 333.99 K.

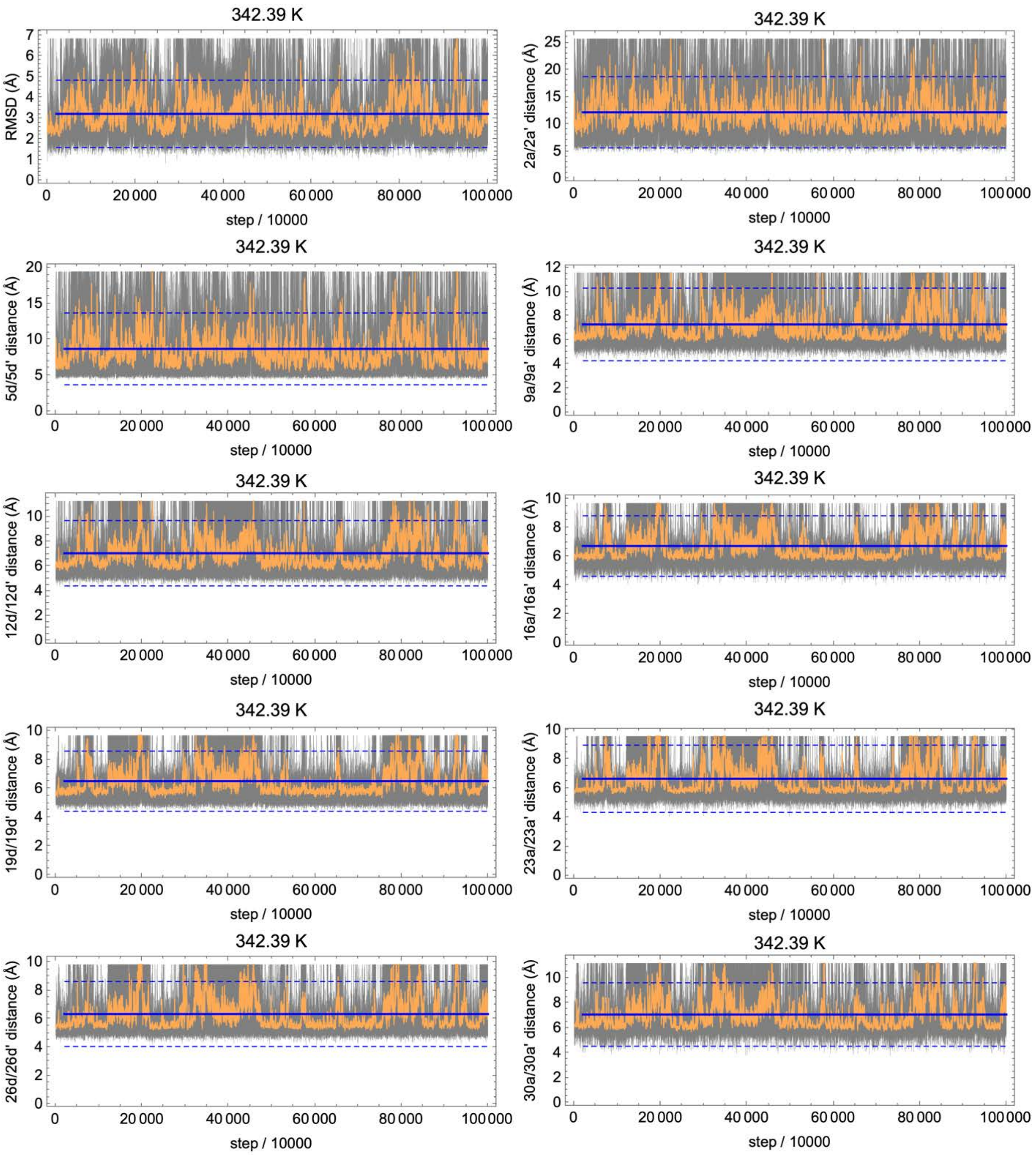

Measure at 342.39 K	Average (Å)	Standard Deviation (Å)	Slope (Å / μs)
RMSD	3.22088	1.62502	−0.09076
5d/5d' distance	8.68758	5.00408	−0.327855
12d/12d' distance	7.03743	2.63271	0.0454705
19d/19d' distance	6.51563	2.08654	0.0148528
26d/26d' distance	6.34143	2.28917	0.0401432
2a/2a' distance	12.1651	6.55595	−0.671709
9a/9a' distance	7.28867	3.01557	0.08238
16a/16a' distance	6.72197	2.09993	0.00907936
23a/23a' distance	6.64885	2.30561	−0.00734228
30a/30a' distance	7.07682	2.54386	0.0599314
Core Distance	7.60927	3.90128	

Figure S214. Plots of RMSD and residue packing distances for T-REMD simulations of variant **s20e/15g'-x4x** at 342.39 K.

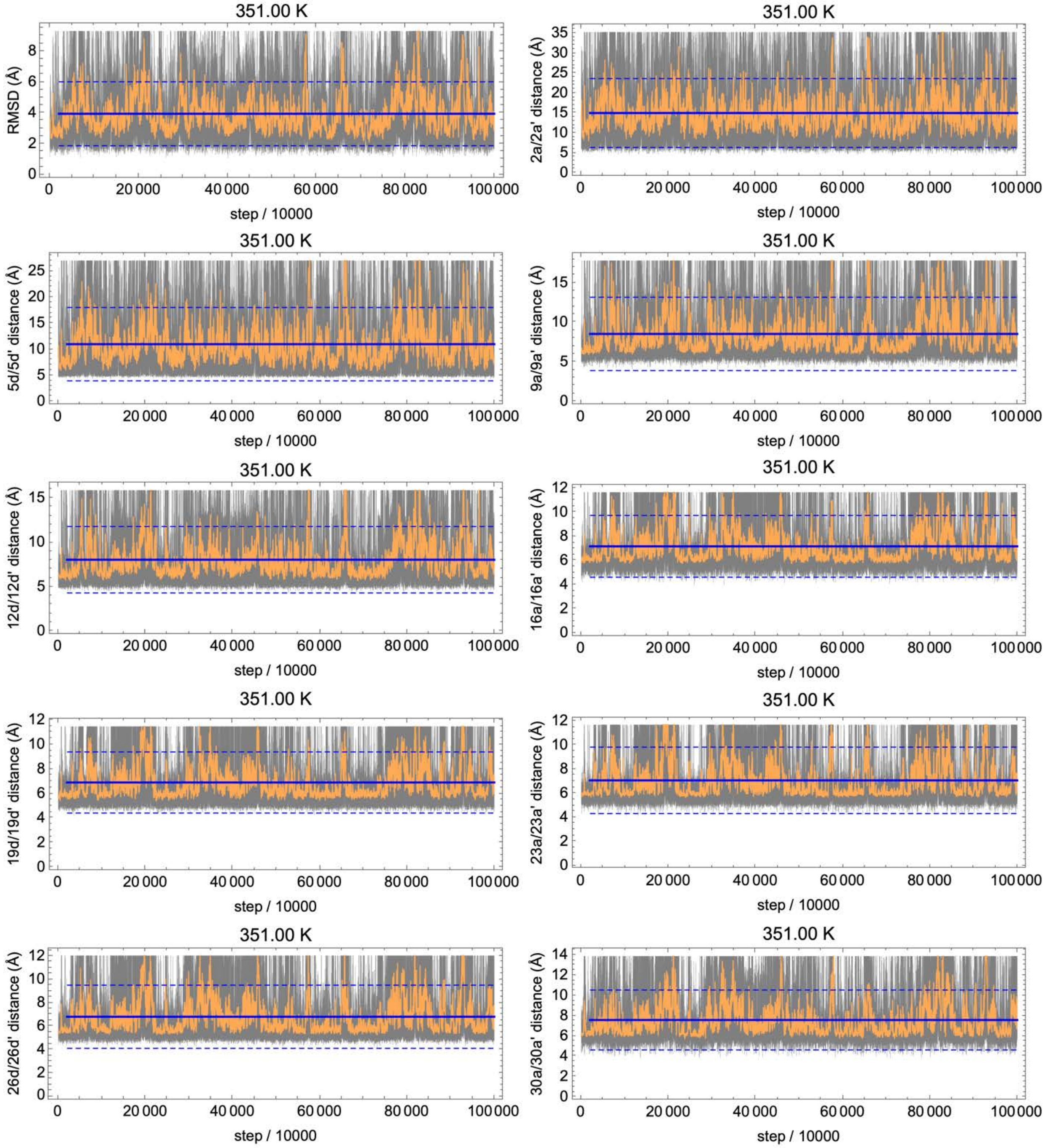

Measure at 351.00 K	Average (Å)	Standard Deviation (Å)	Slope (Å / μs)
RMSD	3.93815	2.0726	0.0638141
5d/5d' distance	10.9621	7.0383	0.19944
12d/12d' distance	8.03218	3.74601	0.305923
19d/19d' distance	6.89861	2.49056	0.0579881
26d/26d' distance	6.80035	2.70265	0.0821183
2a/2a' distance	14.9782	8.62598	−0.0628461
9a/9a' distance	8.52616	4.64224	0.426883
16a/16a' distance	7.17292	2.55931	0.068747
23a/23a' distance	7.06643	2.75759	0.024787
30a/30a' distance	7.57765	2.95321	0.0967656
Core Distance	8.6683	5.31271	

Figure S215. Plots of RMSD and residue packing distances for T-REMD simulations of variant **s20e/15g'-x4x** at 351.00 K.

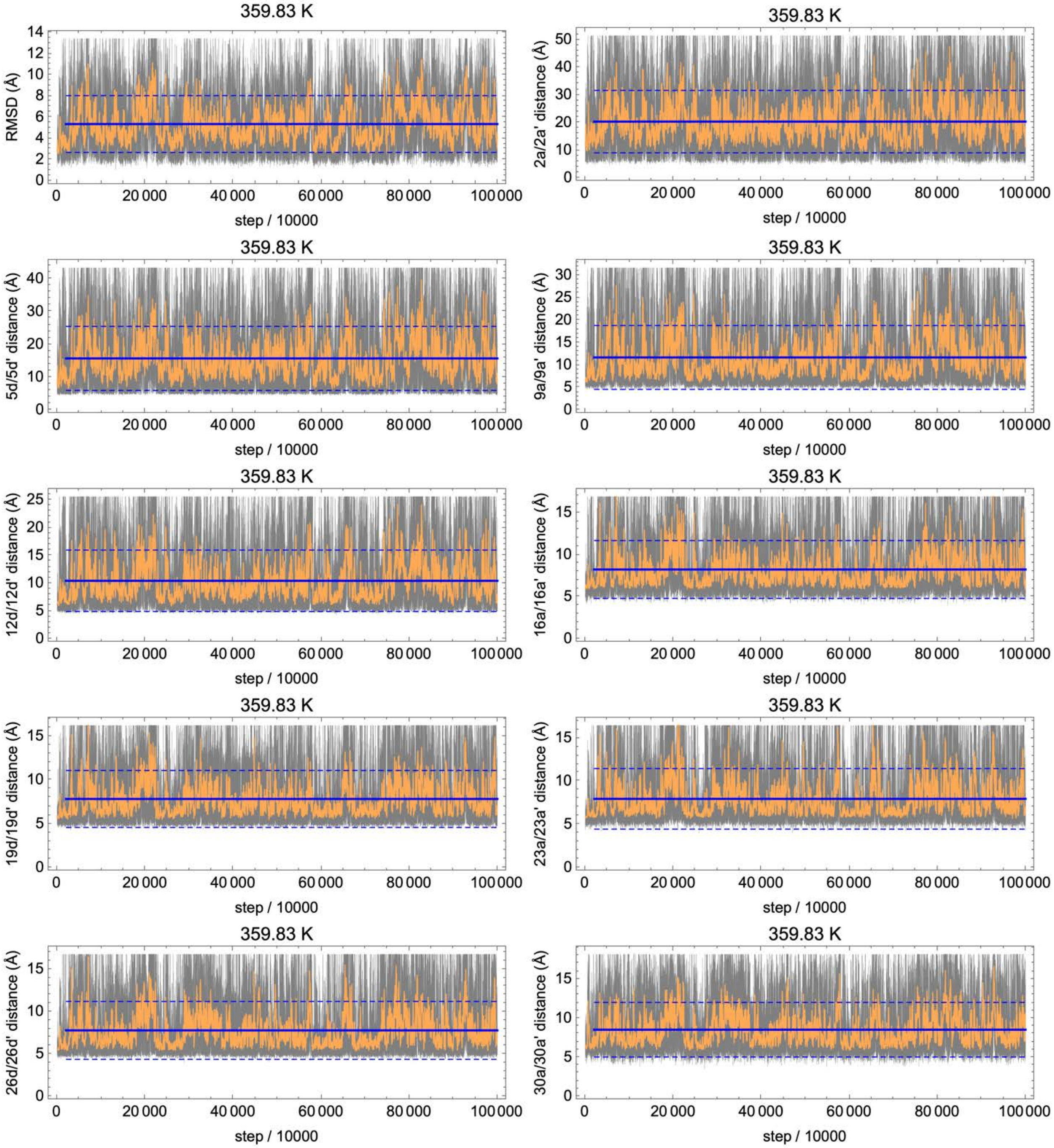

Measure at 359.83 K	Average (Å)	Standard Deviation (Å)	Slope (Å / μs)
RMSD	5.34453	2.68179	0.244135
5d/5d' distance	15.7008	9.76734	0.91296
12d/12d' distance	10.4272	5.53022	0.6089
19d/19d' distance	7.82357	3.24955	0.127602
26d/26d' distance	7.77808	3.4155	0.113636
2a/2a' distance	20.3216	11.2878	0.731756
9a/9a' distance	11.6802	7.10879	0.843838
16a/16a' distance	8.23137	3.43823	0.180738
23a/23a' distance	7.94738	3.50549	0.101429
30a/30a' distance	8.51833	3.48688	0.112734
Core Distance	10.9365	7.57028	

Figure S216. Plots of RMSD and residue packing distances for T-REMD simulations of variant **s20e/15g'-x4x** at 359.83 K.

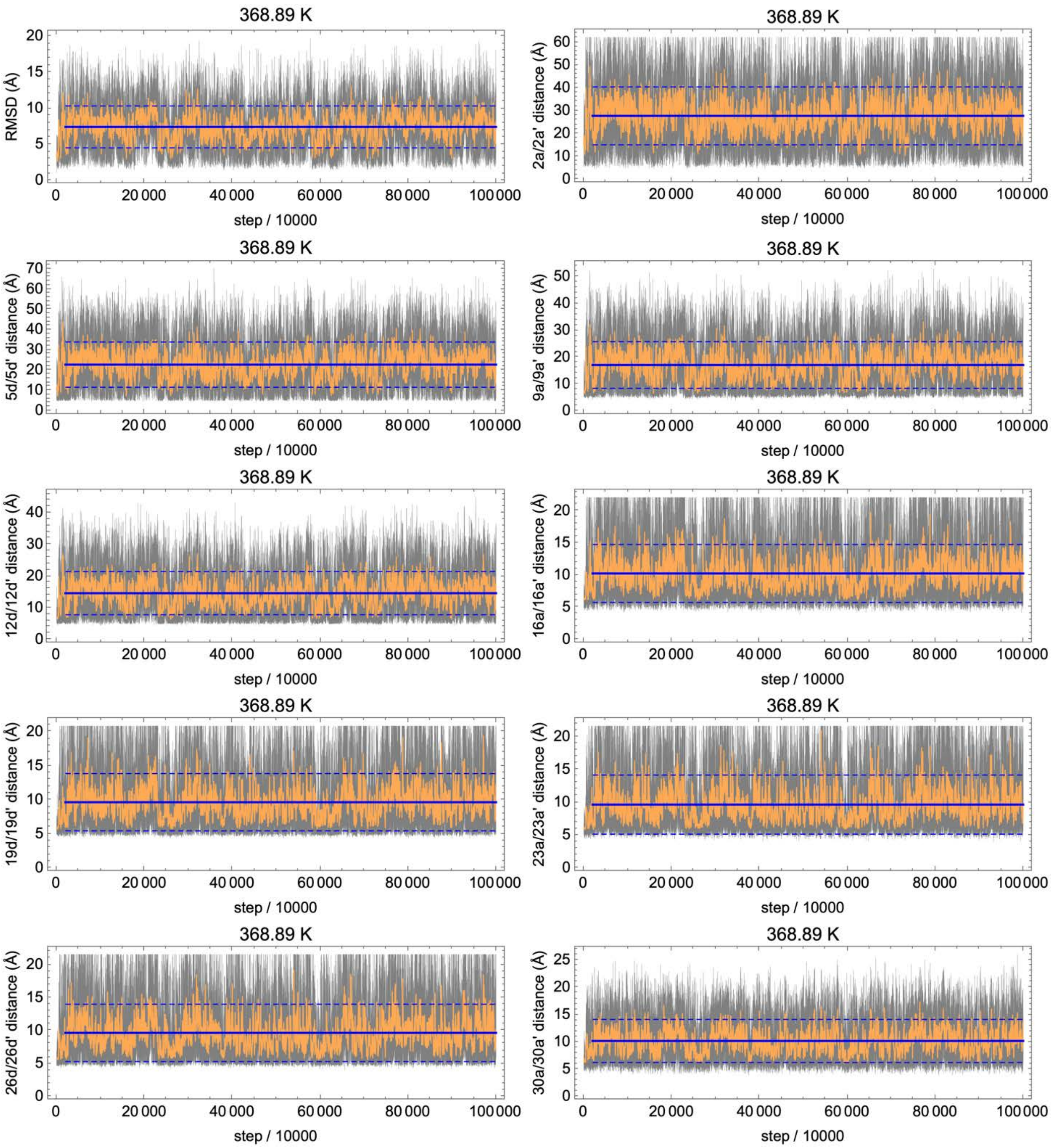

Measure at 368.89 K	Average (Å)	Standard Deviation (Å)	Slope (Å / μs)
RMSD	7.40466	2.90498	0.129294
5d/5d' distance	22.6578	11.1827	0.490021
12d/12d' distance	14.5702	6.79408	0.315824
19d/19d' distance	9.63854	4.20626	0.13561
26d/26d' distance	9.63824	4.35555	0.165284
2a/2a' distance	27.7146	12.6799	0.405921
9a/9a' distance	17.0747	8.67262	0.469709
16a/16a' distance	10.2423	4.50477	0.130913
23a/23a' distance	9.63313	4.50059	0.175364
30a/30a' distance	10.1564	3.95508	0.0747851
Core Distance	14.5918	9.75103	

Figure S217. Plots of RMSD and residue packing distances for T-REMD simulations of variant **s20e/15g'-x4x** at 368.89 K.

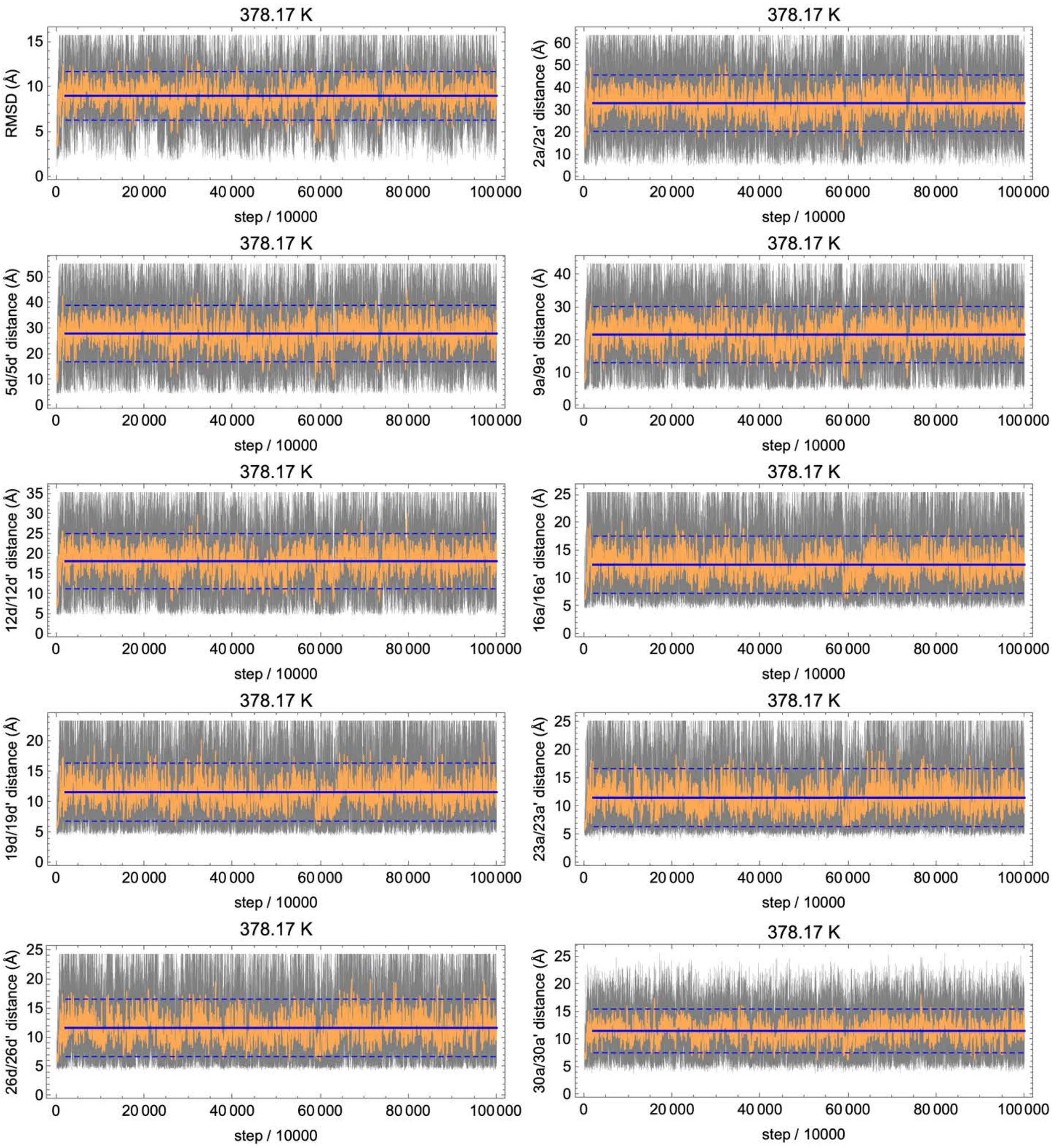

Measure at 378.17 K	Average (Å)	Standard Deviation (Å)	Slope (Å / μs)
RMSD	9.03082	2.70369	−0.0498558
5d/5d' distance	28.0606	10.9907	−0.114831
12d/12d' distance	18.2511	6.89678	−0.136345
19d/19d' distance	11.6507	4.78558	−0.00926698
26d/26d' distance	11.68	4.94037	0.0438953
2a/2a' distance	33.2077	12.6565	−0.14574
9a/9a' distance	21.7275	8.61609	−0.0631303
16a/16a' distance	12.4679	5.13768	−0.0614855
23a/23a' distance	11.5719	5.15105	0.0106548
30a/30a' distance	11.5572	3.97127	−0.0336255
Core Distance	17.7972	10.8451	

Figure S218. Plots of RMSD and residue packing distances for T-REMD simulations of variant **s20e/15g'-x4x** at 378.17 K.

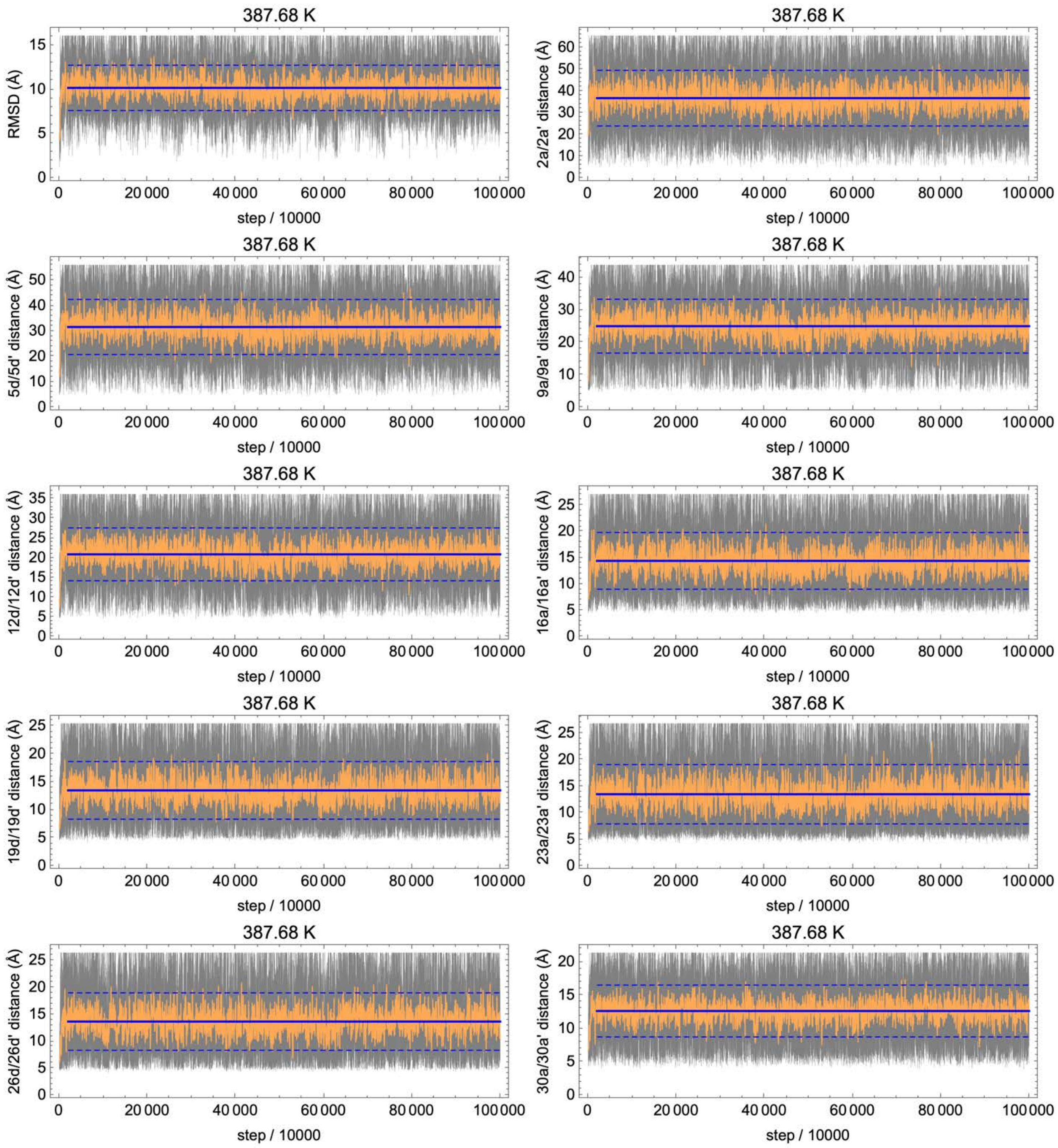

Measure at 387.68 K	Average (Å)	Standard Deviation (Å)	Slope (Å / μs)
RMSD	10.1696	2.55713	−0.143102
5d/5d' distance	31.6061	10.8378	−0.525915
12d/12d' distance	20.8823	6.71357	−0.26121
19d/19d' distance	13.4882	5.14903	0.0189879
26d/26d' distance	13.6466	5.32805	0.0855679
2a/2a' distance	36.7378	12.7965	−0.631059
9a/9a' distance	25.0001	8.28627	−0.320639
16a/16a' distance	14.3993	5.39409	−0.0555859
23a/23a' distance	13.49	5.58986	0.0308206
30a/30a' distance	12.6312	3.89193	0.0102286
Core Distance	20.209	11.4328	

Figure S219. Plots of RMSD and residue packing distances for T-REMD simulations of variant **s20e/15g'-x4x** at 387.68 K.

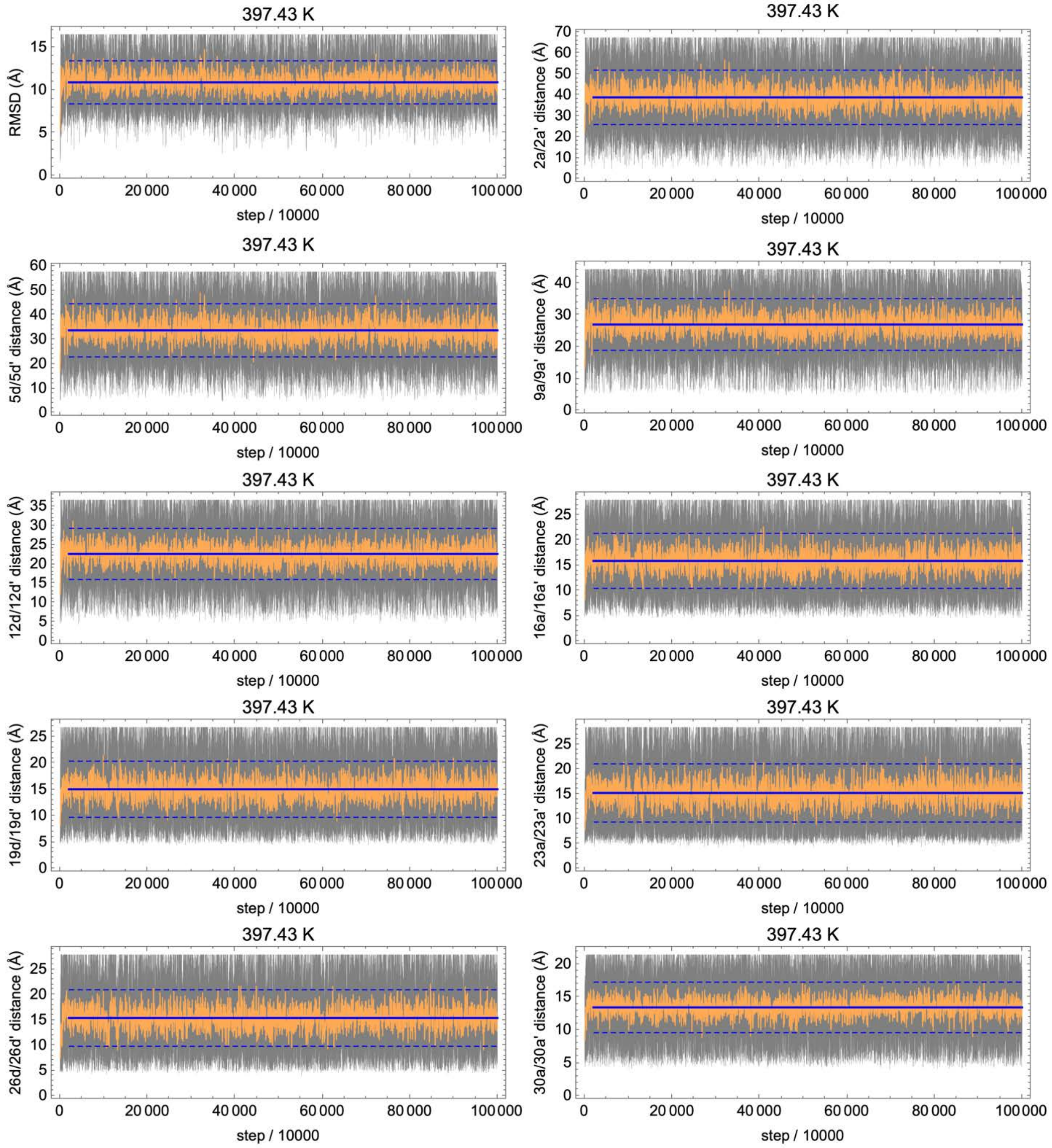

Measure at 397.43 K	Average (Å)	Standard Deviation (Å)	Slope (Å / μs)
RMSD	10.9131	2.52079	−0.112452
5d/5d' distance	33.7161	10.8449	−0.432129
12d/12d' distance	22.6035	6.62578	−0.147323
19d/19d' distance	15.0491	5.33138	0.0677783
26d/26d' distance	15.3874	5.57809	0.169709
2a/2a' distance	38.8602	12.9847	−0.483447
9a/9a' distance	27.0048	8.12322	−0.246404
16a/16a' distance	15.898	5.44597	−0.0187703
23a/23a' distance	15.213	5.86266	0.15847
30a/30a' distance	13.4228	3.83405	0.0527932
Core Distance	21.9061	11.6911	

Figure S220. Plots of RMSD and residue packing distances for T-REMD simulations of variant **s20e/15g’-x4x** at 397.43 K.

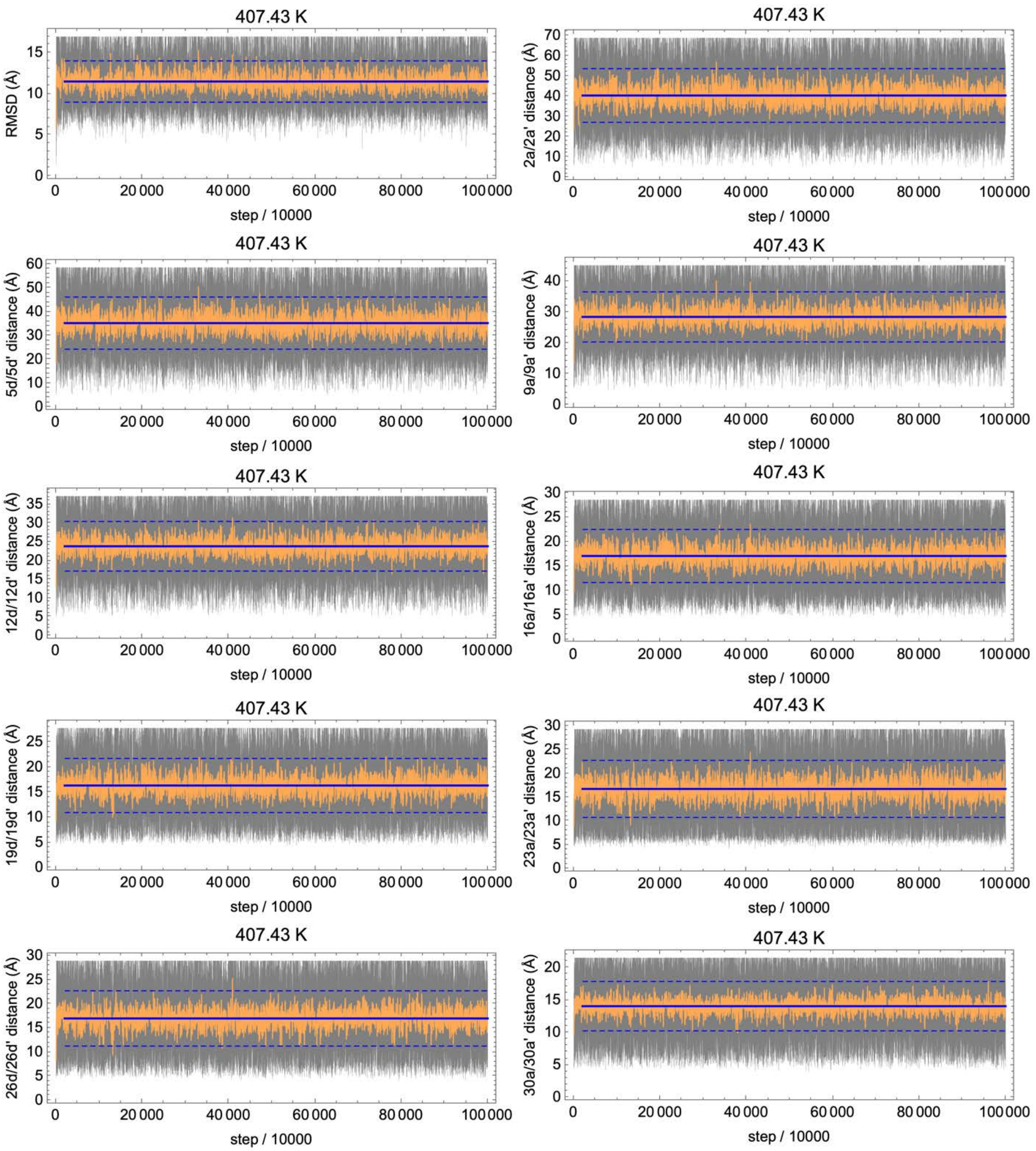

Measure at 407.43 K	Average (Å)	Standard Deviation (Å)	Slope (Å / μs)
RMSD	11.4813	2.50708	−0.0561671
5d/5d' distance	35.2361	10.971	−0.209863
12d/12d' distance	23.8006	6.61272	0.0264993
19d/19d' distance	16.3273	5.38948	0.100112
26d/26d' distance	16.9688	5.73024	0.125851
2a/2a' distance	40.4013	13.2462	−0.191903
9a/9a' distance	28.4251	8.09947	−0.103115
16a/16a' distance	17.074	5.43784	0.0460914
23a/23a' distance	16.7516	5.99414	0.130081
30a/30a' distance	14.0306	3.80399	0.0184581
Core Distance	23.224	11.854	

Figure S221. Plots of RMSD and residue packing distances for T-REMD simulations of variant **s20e/15g'-x4x** at 407.43 K.

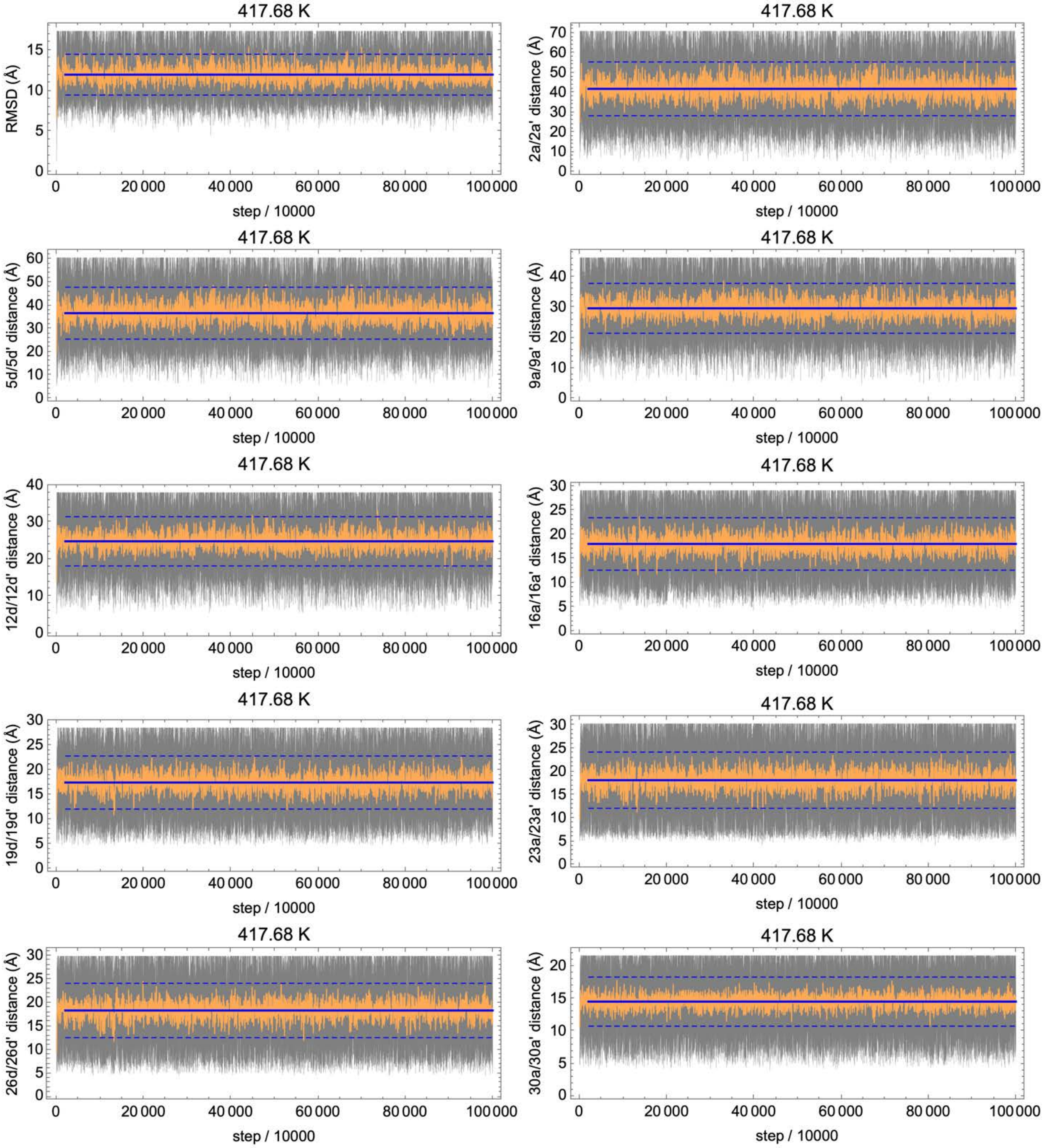

Measure at 417.68 K	Average (Å)	Standard Deviation (Å)	Slope (Å / μs)
RMSD	11.9834	2.5201	−0.0378866
5d/5d' distance	36.6352	11.1648	−0.262907
12d/12d' distance	24.8376	6.65095	0.0275188
19d/19d' distance	17.4094	5.37473	0.070296
26d/26d' distance	18.3313	5.76928	0.104652
2a/2a' distance	41.8465	13.56	−0.250492
9a/9a' distance	29.6582	8.21492	−0.131019
16a/16a' distance	18.058	5.42736	0.00510784
23a/23a' distance	18.1427	6.05264	0.0908157
30a/30a' distance	14.5369	3.76358	0.0360706
Core Distance	24.384	12.0505	

Figure S222. Plots of RMSD and residue packing distances for T-REMD simulations of variant **s20e/15g’-x4x** at 417.68 K.

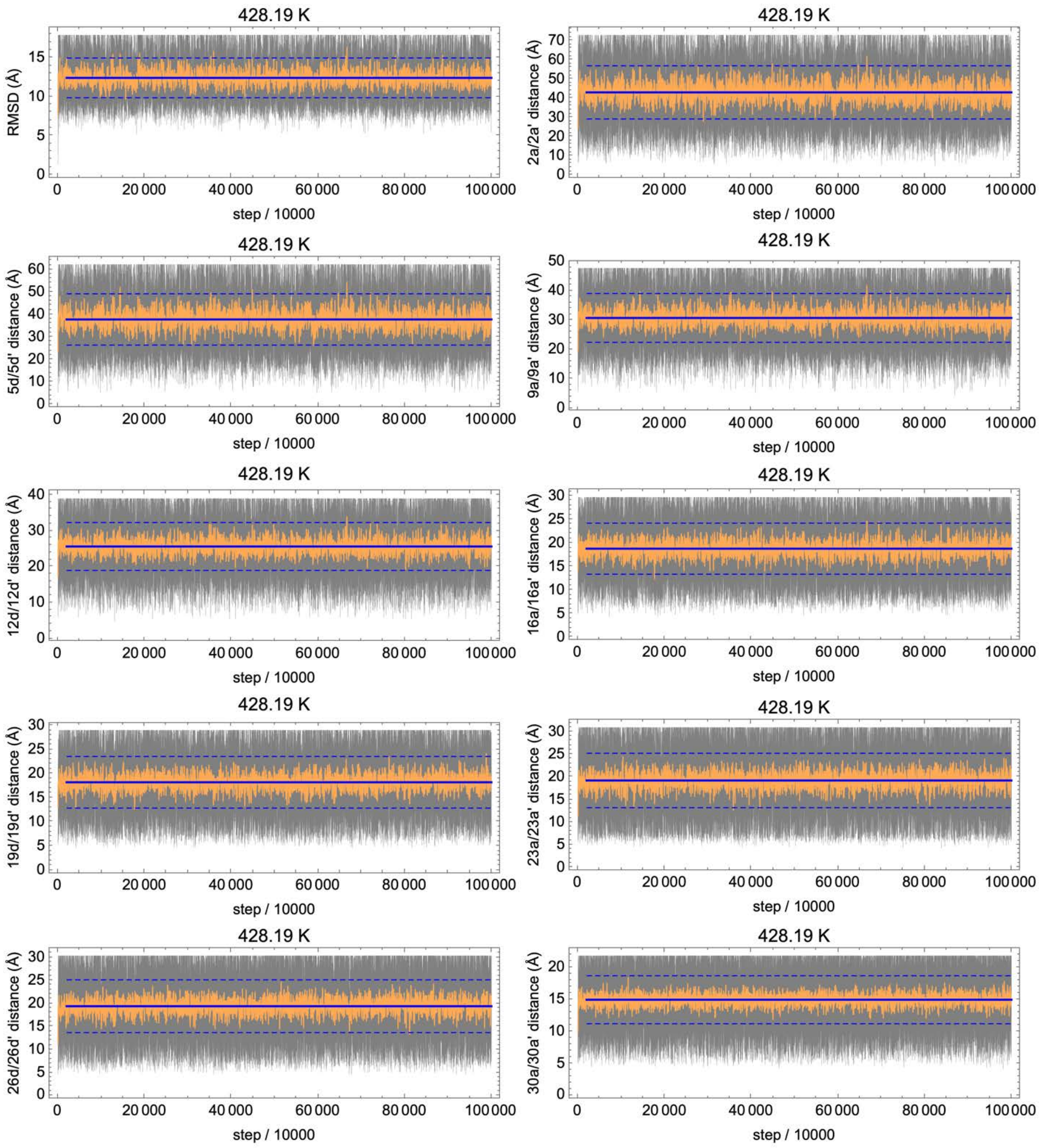

Measure at 428.19 K	Average (Å)	Standard Deviation (Å)	Slope (Å / μs)
RMSD	12.399	2.54378	−0.0319641
5d/5d' distance	37.7171	11.4057	−0.235808
12d/12d' distance	25.5925	6.67759	0.0243652
19d/19d' distance	18.1725	5.34963	0.138397
26d/26d' distance	19.3767	5.7209	0.162526
2a/2a' distance	42.9812	13.8627	−0.355925
9a/9a' distance	30.6495	8.3047	−0.130488
16a/16a' distance	18.7773	5.4225	0.0534629
23a/23a' distance	19.1845	6.01894	0.154986
30a/30a' distance	14.897	3.73059	0.0579288
Core Distance	25.2609	12.2305	

Figure S223. Plots of RMSD and residue packing distances for T-REMD simulations of variant **s20e/15g'-x4x** at 428.19 K.

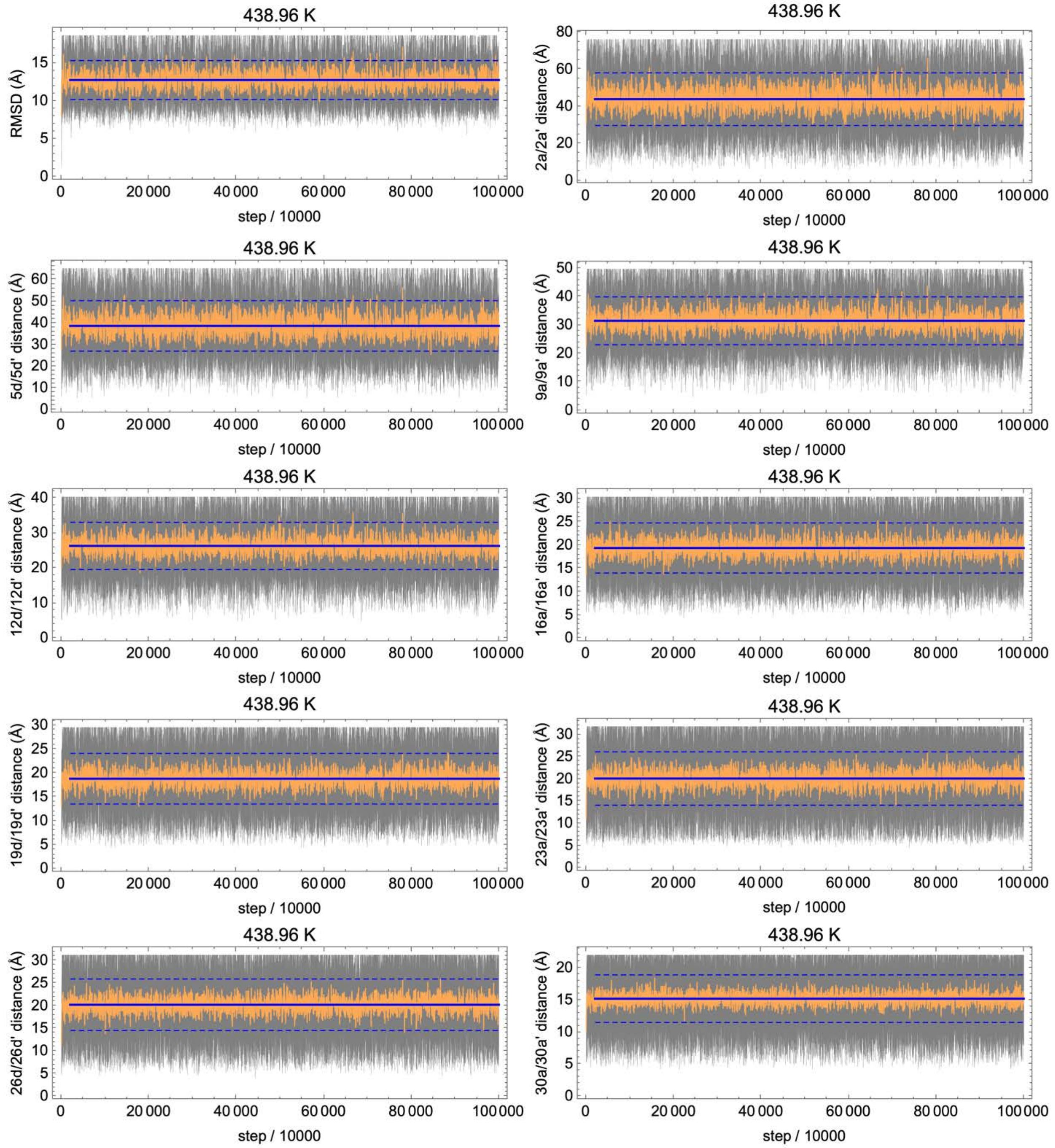

Measure at 438.96 K	Average (Å)	Standard Deviation (Å)	Slope (Å / μs)
RMSD	12.7507	2.56329	−0.024388
5d/5d' distance	38.6008	11.6448	−0.301702
12d/12d' distance	26.3105	6.73544	0.0151129
19d/19d' distance	18.8088	5.29512	0.0980782
26d/26d' distance	20.2369	5.69807	0.0763257
2a/2a' distance	43.8558	14.1843	−0.382221
9a/9a' distance	31.49	8.42306	−0.154245
16a/16a' distance	19.3867	5.38678	0.0214378
23a/23a' distance	20.0915	6.01065	0.106811
30a/30a' distance	15.2185	3.71055	0.0019862
Core Distance	25.9999	12.3836	

Figure S224. Plots of RMSD and residue packing distances for T-REMD simulations of variant **s20e/15g'-x4x** at 438.96 K.

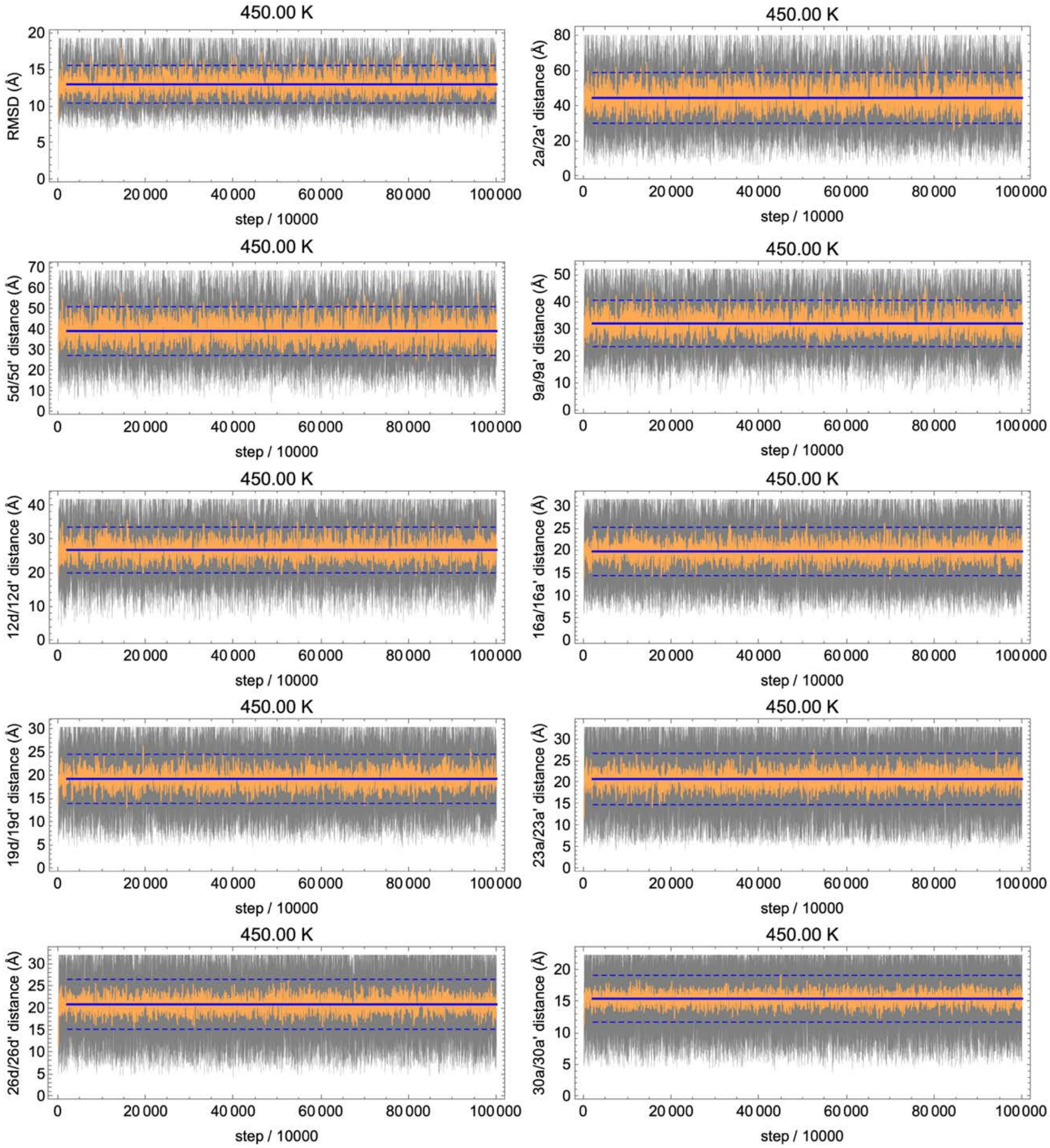

Measure at 450.00 K	Average (Å)	Standard Deviation (Å)	Slope (Å / μs)
RMSD	13.0554	2.57756	−0.0307053
5d/5d' distance	39.3453	11.8706	−0.380848
12d/12d' distance	26.8822	6.78585	−0.0769875
19d/19d' distance	19.3365	5.28799	0.0871608
26d/26d' distance	20.9051	5.65662	0.100299
2a/2a' distance	44.6919	14.4514	−0.440609
9a/9a' distance	32.185	8.56667	−0.192992
16a/16a' distance	19.9437	5.4008	0.0240832
23a/23a' distance	20.821	5.98246	0.14286
30a/30a' distance	15.4691	3.70043	0.012266
Core Distance	26.62	12.5415	

Figure S225. Plots of RMSD and residue packing distances for T-REMD simulations of variant **s20e/15g'-x4x** at 450.00 K.

(return to Table of Contents)

27e/22g'-xx

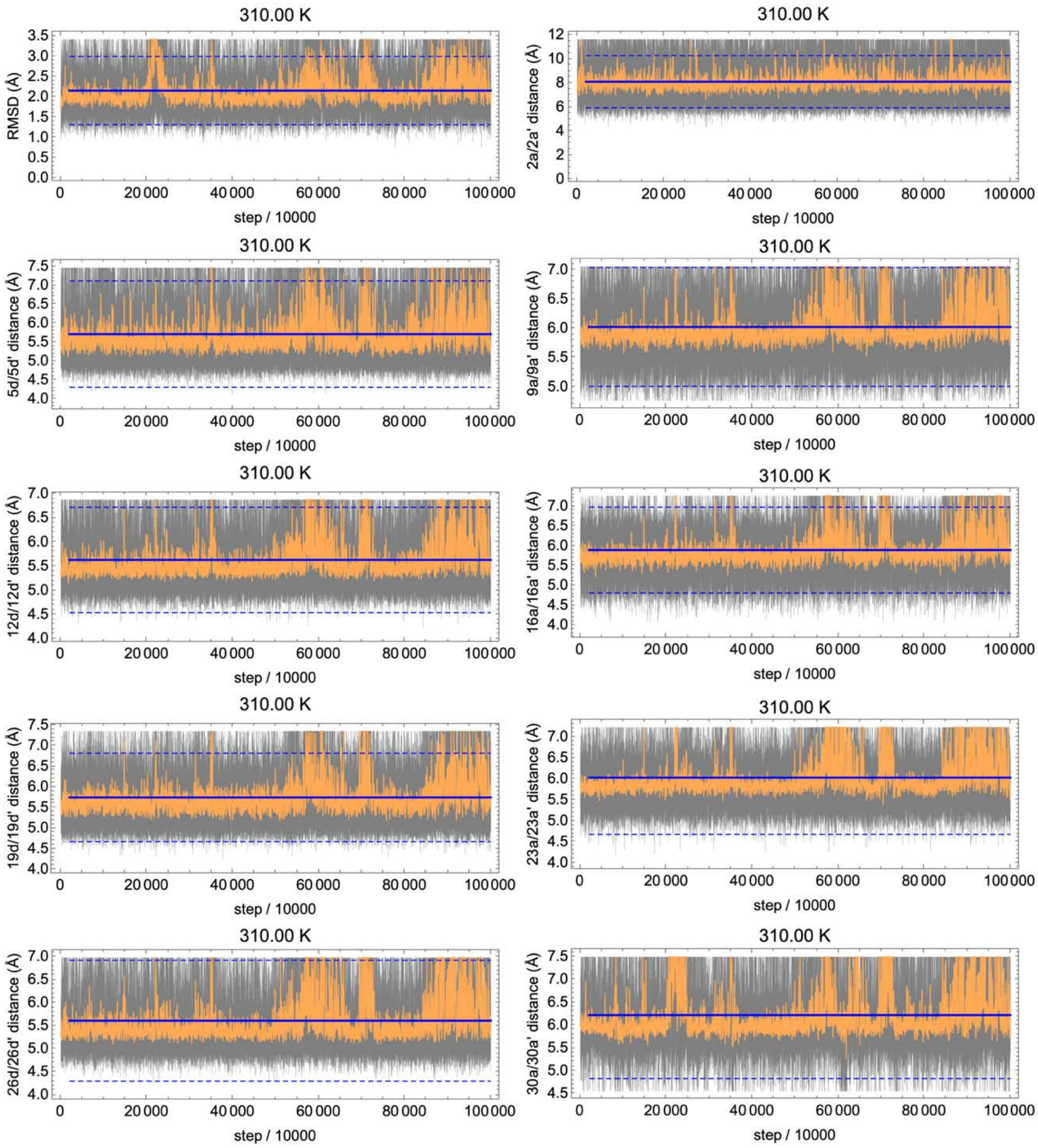

Measure at 310.00 K	Average (Å)	Standard Deviation (Å)	Slope (Å / μs)
RMSD	2.15515	0.841773	0.123476
5d/5d' distance	5.71227	1.40468	0.21218
12d/12d' distance	5.62941	1.08978	0.201598
19d/19d' distance	5.7448	1.07348	0.192588
26d/26d' distance	5.6088	1.31043	0.251855
2a/2a' distance	8.11024	2.16987	0.159987
9a/9a' distance	6.02465	1.01725	0.18386
16a/16a' distance	5.8932	1.08246	0.207607
23a/23a' distance	6.02936	1.35096	0.244617
30a/30a' distance	6.21943	1.39191	0.179926
Core Distance	6.10802	1.54758	

Figure S226. Plots of RMSD and residue packing distances for T-REMD simulations of variant **27e/22g'-xx** at 310.00 K.

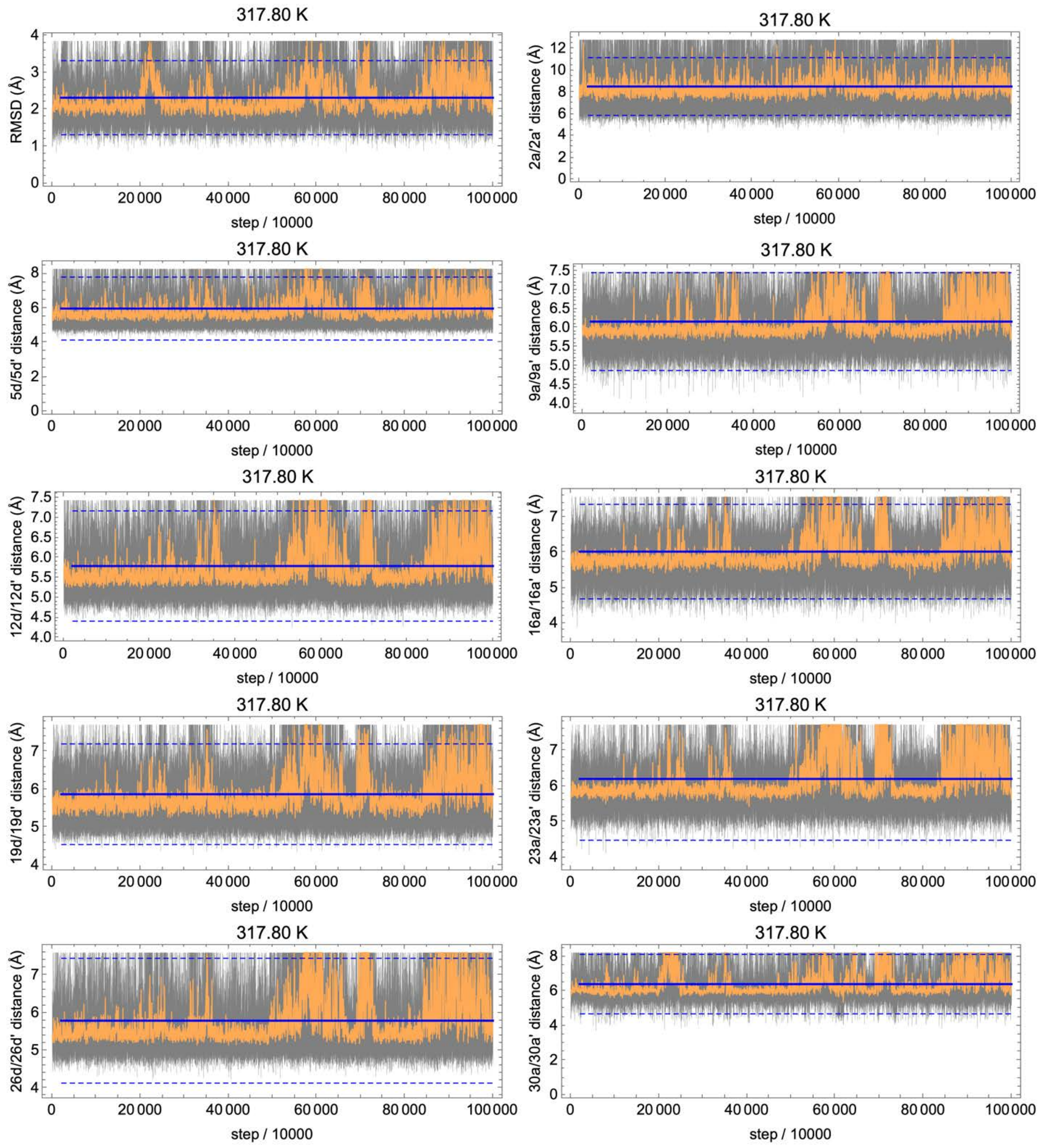

Measure at 317.80 K	Average (Å)	Standard Deviation (Å)	Slope (Å / μs)
RMSD	2.31642	1.00259	0.217947
5d/5d' distance	5.98021	1.83377	0.366047
12d/12d' distance	5.78986	1.37998	0.332301
19d/19d' distance	5.87338	1.32793	0.310791
26d/26d' distance	5.78977	1.65601	0.426551
2a/2a' distance	8.51408	2.64456	0.292252
9a/9a' distance	6.16085	1.29147	0.304961
16a/16a' distance	6.02327	1.33719	0.328101
23a/23a' distance	6.2015	1.71841	0.430568
30a/30a' distance	6.39602	1.71626	0.332221
Core Distance	6.30322	1.88392	

Figure S227. Plots of RMSD and residue packing distances for T-REMD simulations of variant **27e/22g’-xx** at 317.80 K.

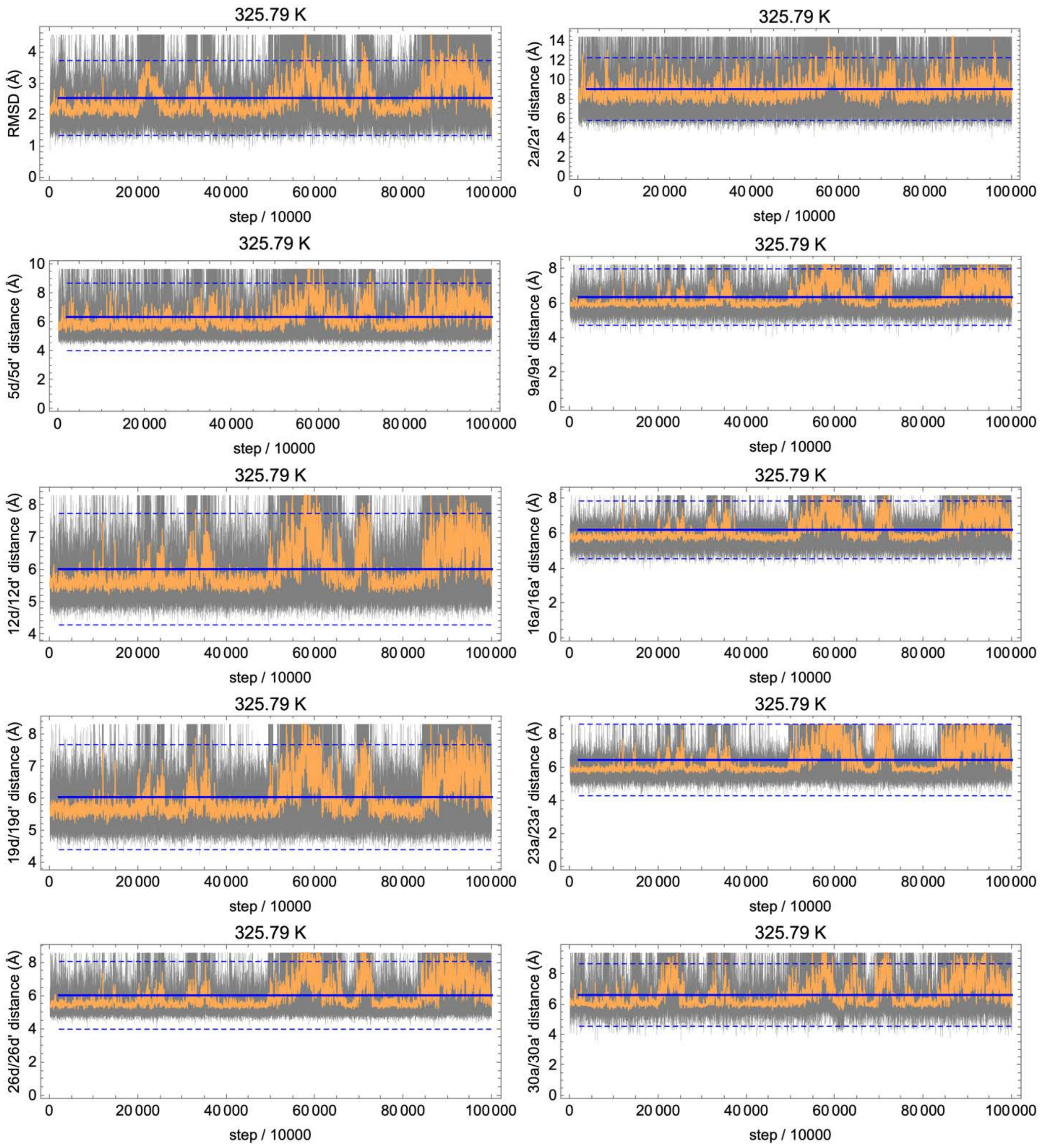

Measure at 325.79 K	Average (Å)	Standard Deviation (Å)	Slope (Å / μs)
RMSD	2.54088	1.19299	0.342564
5d/5d' distance	6.36741	2.3322	0.576104
12d/12d' distance	6.01934	1.72245	0.505345
19d/19d' distance	6.05034	1.63736	0.472058
26d/26d' distance	6.04467	2.04042	0.652418
2a/2a' distance	9.06111	3.23991	0.454926
9a/9a' distance	6.36072	1.62832	0.468054
16a/16a' distance	6.21295	1.6552	0.494398
23a/23a' distance	6.45983	2.16383	0.680295
30a/30a' distance	6.64549	2.06301	0.510128
Core Distance	6.58021	2.29396	

Figure S228. Plots of RMSD and residue packing distances for T-REMD simulations of variant **27e/22g'-xx** at 325.79 K.

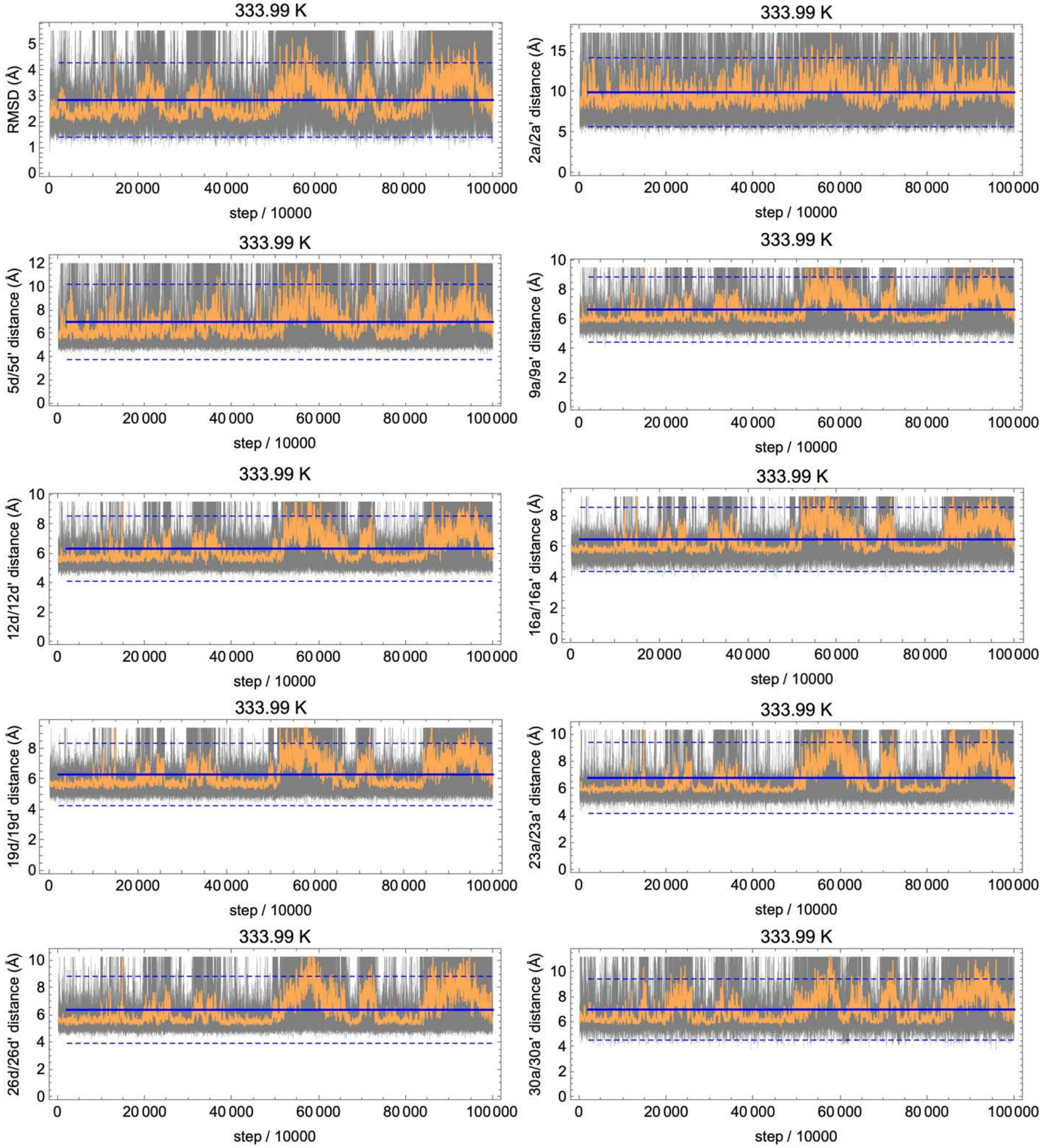

Measure at 333.99 K	Average (Å)	Standard Deviation (Å)	Slope (Å / μs)
RMSD	2.85511	1.44087	0.463498
5d/5d' distance	7.01427	3.23252	0.764895
12d/12d' distance	6.35936	2.22042	0.666728
19d/19d' distance	6.32149	2.04831	0.643008
26d/26d' distance	6.40344	2.45635	0.881745
2a/2a' distance	9.95907	4.26042	0.619238
9a/9a' distance	6.67902	2.21393	0.62873
16a/16a' distance	6.49393	2.08246	0.659452
23a/23a' distance	6.80729	2.62409	0.92544
30a/30a' distance	7.00834	2.44639	0.694246
Core Distance	7.00513	2.91065	

Figure S229. Plots of RMSD and residue packing distances for T-REMD simulations of variant **27e/22g'-xx** at 333.99 K.

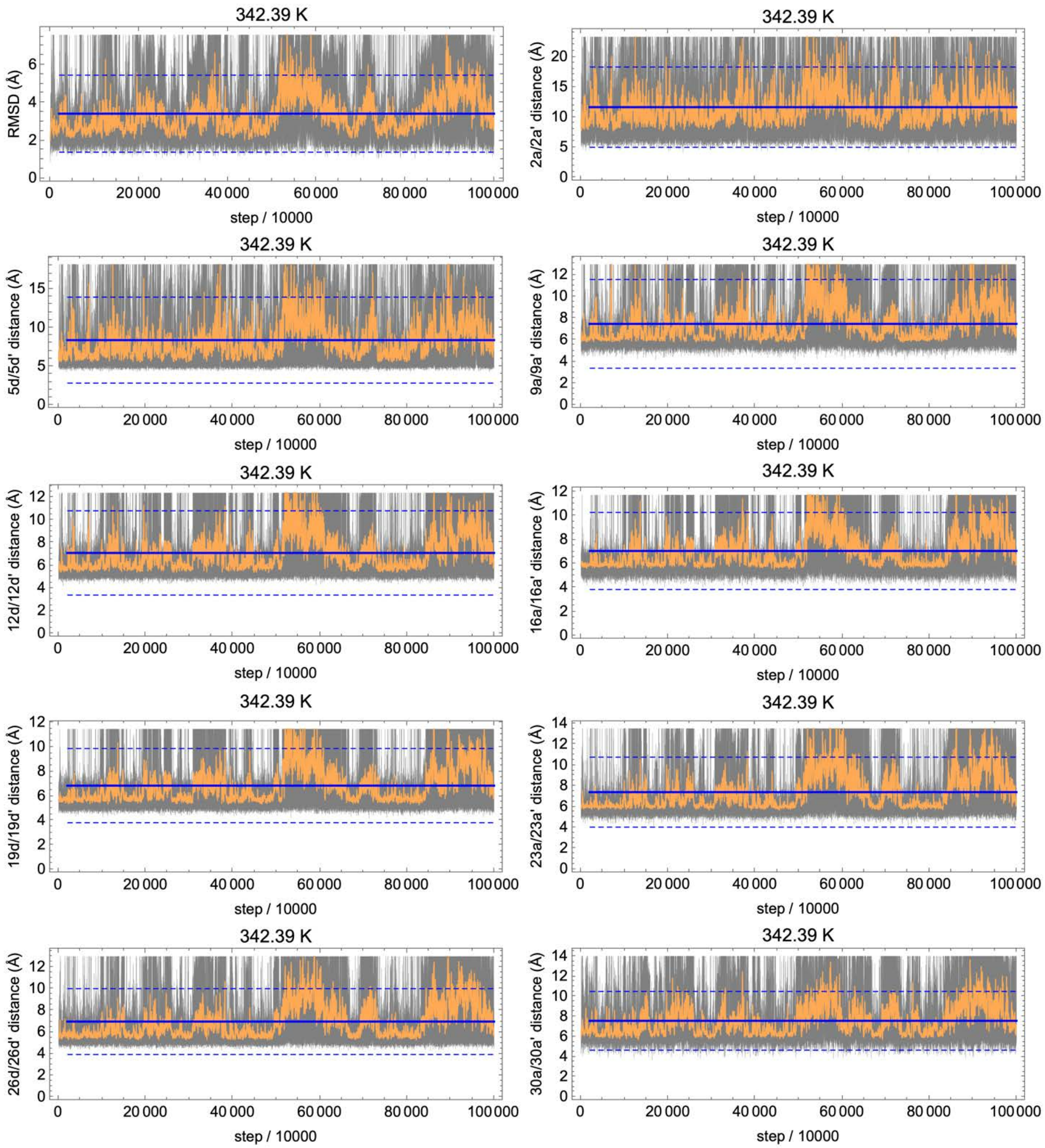

Measure at 342.39 K	Average (Å)	Standard Deviation (Å)	Slope (Å / μs)
RMSD	3.41418	2.03657	0.592085
5d/5d' distance	8.38311	5.55664	1.02919
12d/12d' distance	7.09412	3.71245	0.87153
19d/19d' distance	6.8465	3.02541	0.831976
26d/26d' distance	6.9698	3.04025	1.11249
2a/2a' distance	11.6503	6.64689	0.906413
9a/9a' distance	7.46617	4.08166	0.839457
16a/16a' distance	7.05884	3.21416	0.849392
23a/23a' distance	7.3979	3.36419	1.19118
30a/30a' distance	7.57058	2.92494	0.854175
Core Distance	7.82637	4.37453	

Figure S230. Plots of RMSD and residue packing distances for T-REMD simulations of variant **27e/22g'-xx** at 342.39 K.

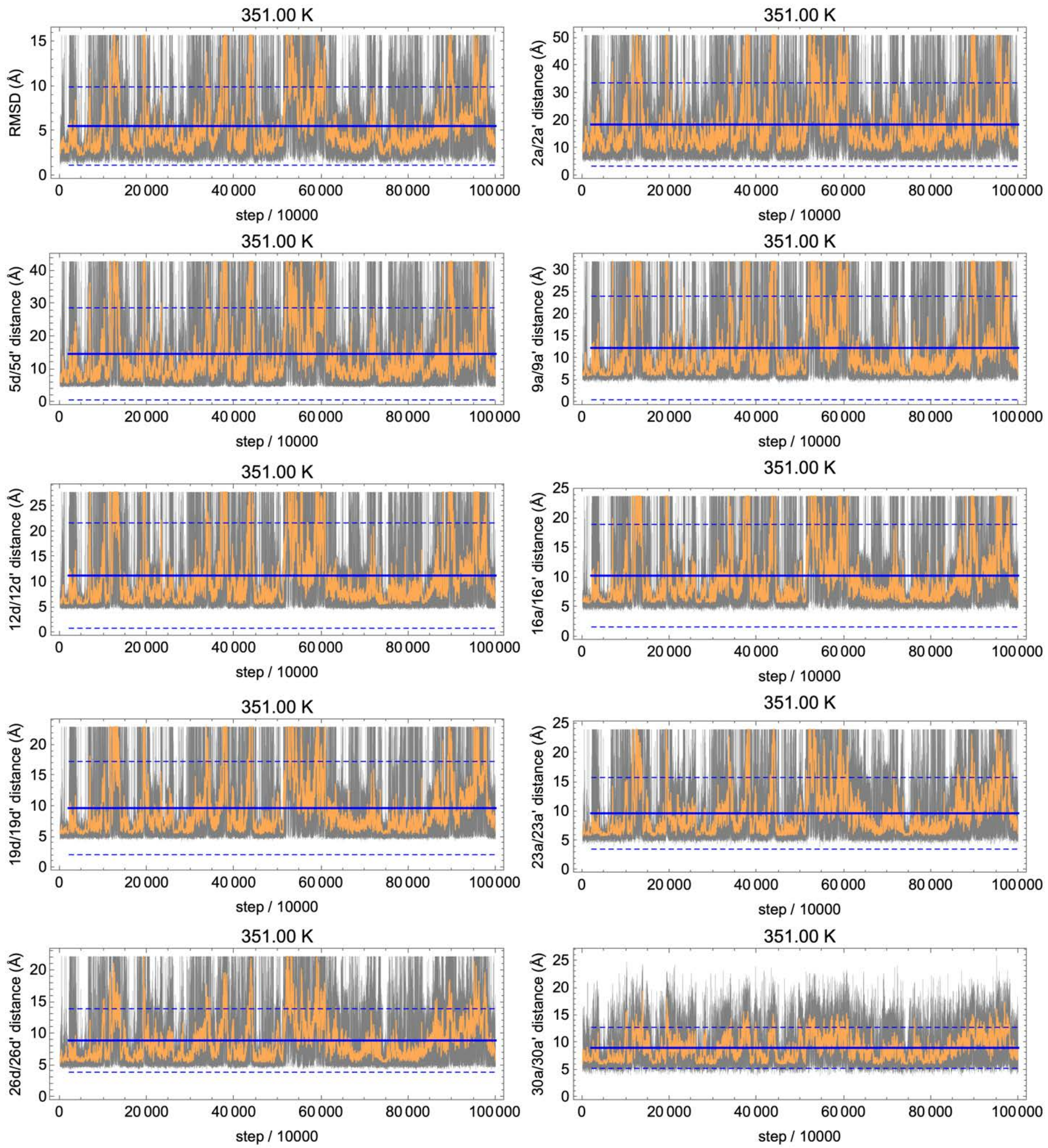

Measure at 351.00 K	Average (Å)	Standard Deviation (Å)	Slope (Å / μs)
RMSD	5.55784	4.37787	0.617502
5d/5d' distance	14.693	14.0509	1.11787
12d/12d' distance	11.2844	10.4139	0.776639
19d/19d' distance	9.70238	7.60999	0.802171
26d/26d' distance	8.92483	5.00591	1.00082
2a/2a' distance	18.5999	15.1211	1.12054
9a/9a' distance	12.2844	11.7655	0.877961
16a/16a' distance	10.3171	8.66041	0.727718
23a/23a' distance	9.68034	6.11676	1.05709
30a/30a' distance	9.02771	3.76089	0.748475
Core Distance	11.6127	10.3511	

Figure S231. Plots of RMSD and residue packing distances for T-REMD simulations of variant **27e/22g'-xx** at 351.00 K.

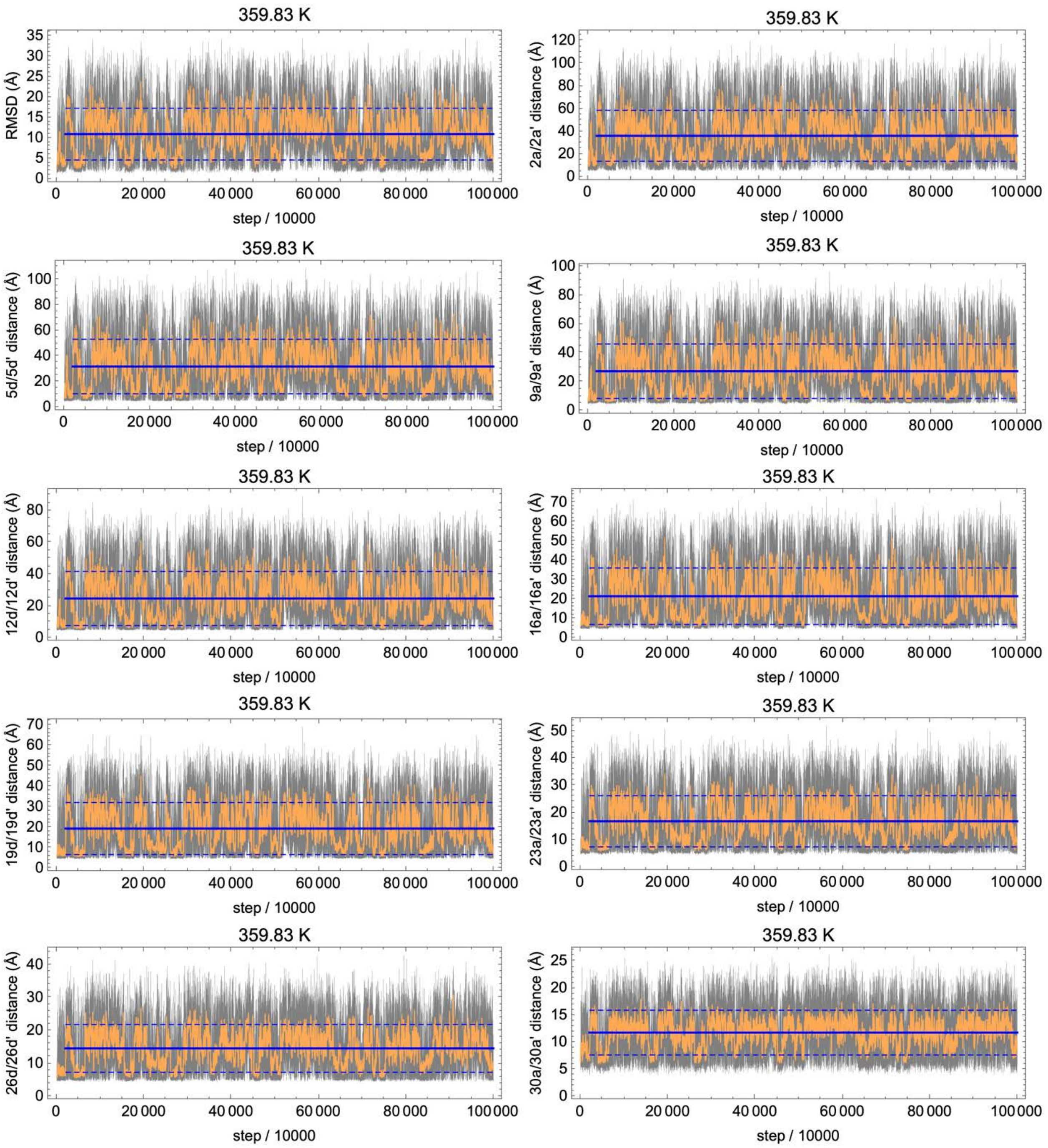

Measure at 359.83 K	Average (Å)	Standard Deviation (Å)	Slope (Å / μs)
RMSD	10.9298	6.34618	0.268957
5d/5d' distance	31.7157	21.367	0.386468
12d/12d' distance	24.661	17.0128	0.105318
19d/19d' distance	19.3535	12.714	0.18452
26d/26d' distance	14.5557	7.27507	0.456186
2a/2a' distance	36.0257	22.4702	0.478243
9a/9a' distance	27.1964	18.8989	0.220377
16a/16a' distance	21.4436	14.5855	0.124468
23a/23a' distance	16.7833	9.40268	0.358787
30a/30a' distance	11.7646	4.13705	0.299407
Core Distance	22.611	17.1834	

Figure S232. Plots of RMSD and residue packing distances for T-REMD simulations of variant **27e/22g’-xx** at 359.83 K.

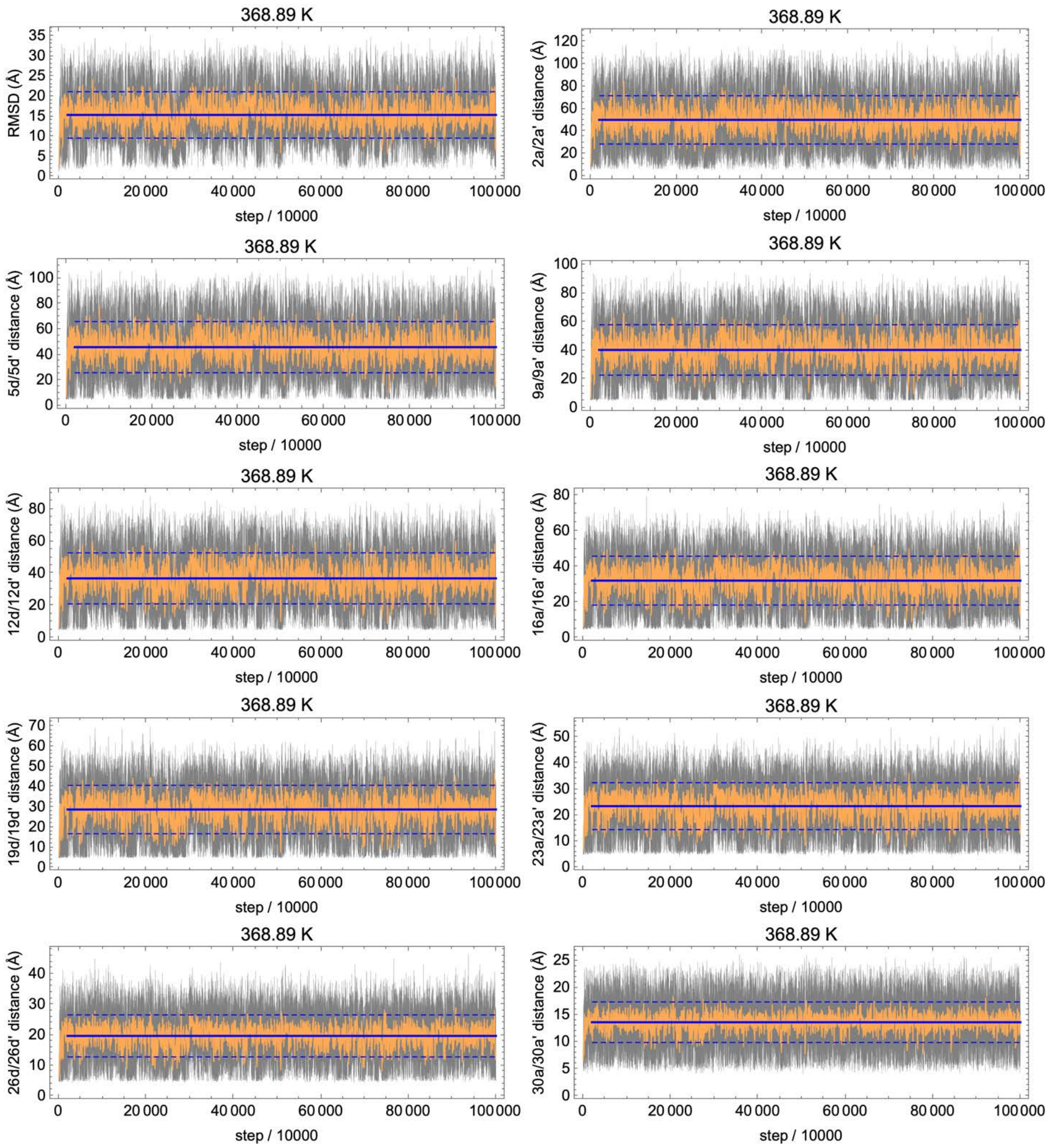

Measure at 368.89 K	Average (Å)	Standard Deviation (Å)	Slope (Å / μs)
RMSD	15.3245	5.77427	−0.204186
5d/5d' distance	45.7921	20.0795	−0.891194
12d/12d' distance	36.8768	15.8527	−0.687901
19d/19d' distance	28.7147	11.9679	−0.337804
26d/26d' distance	19.6561	6.8813	−0.0289322
2a/2a' distance	50.0117	21.5754	−0.924861
9a/9a' distance	40.3284	17.5953	−0.770986
16a/16a' distance	32.0709	13.7011	−0.448358
23a/23a' distance	23.4419	8.98537	−0.117911
30a/30a' distance	13.6058	3.74259	0.0550341
Core Distance	32.2776	18.5127	

Figure S233. Plots of RMSD and residue packing distances for T-REMD simulations of variant **27e/22g’-xx** at 368.89 K.

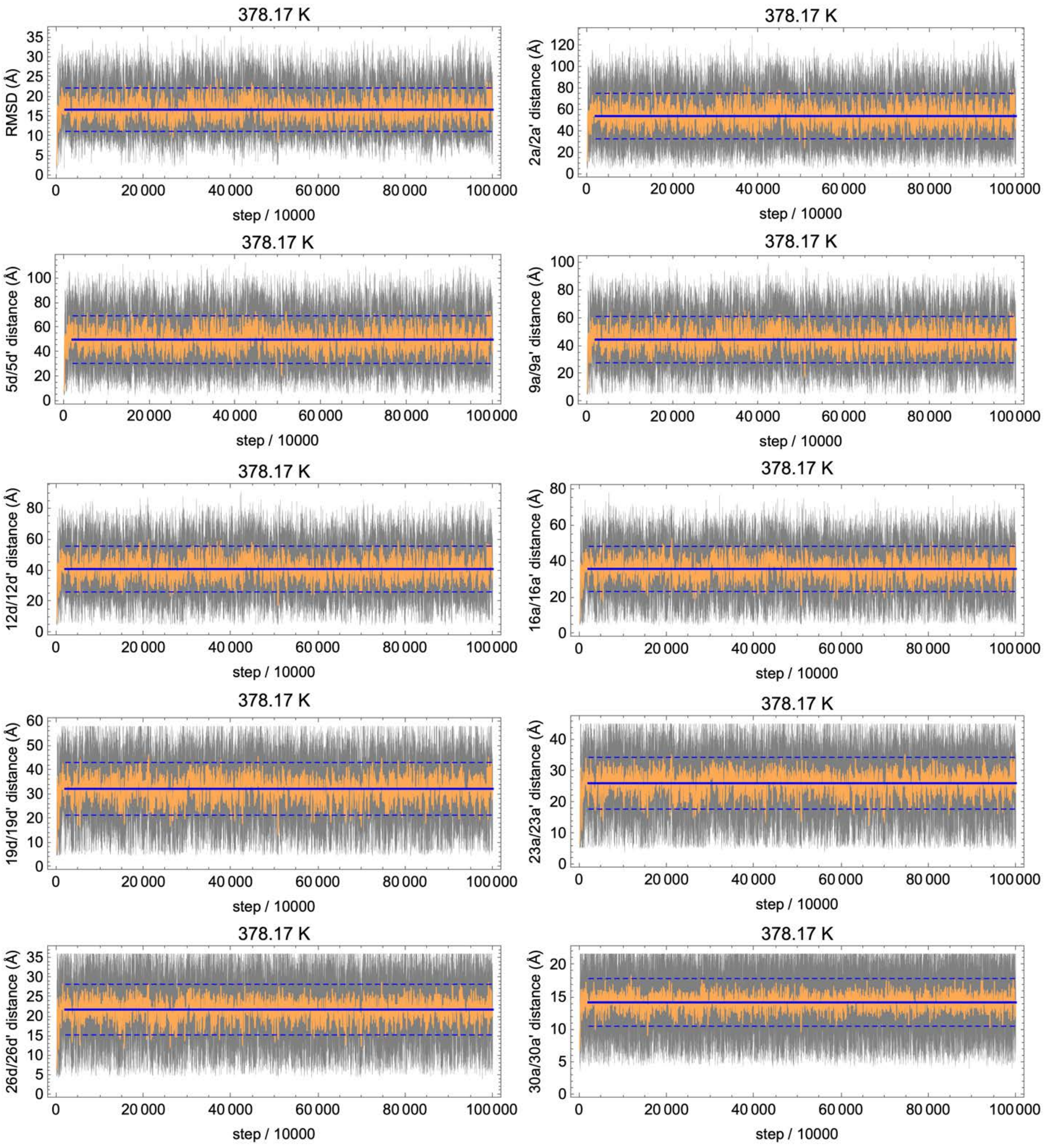

Measure at 378.17 K	Average (Å)	Standard Deviation (Å)	Slope (Å / μs)
RMSD	16.751	5.50737	−0.064548
5d/5d' distance	50.119	19.4226	−0.230568
12d/12d' distance	40.9919	14.7938	−0.176657
19d/19d' distance	32.277	10.8534	−0.0586531
26d/26d' distance	21.7948	6.48866	−0.0188268
2a/2a' distance	54.2408	21.2431	−0.19184
9a/9a' distance	44.6044	16.6666	−0.20774
16a/16a' distance	35.931	12.5229	−0.0792548
23a/23a' distance	26.1523	8.31516	−0.0313335
30a/30a' distance	14.2282	3.67531	0.060217
Core Distance	35.5933	18.6806	

Figure S234. Plots of RMSD and residue packing distances for T-REMD simulations of variant **27e/22g'-xx** at 378.17 K.

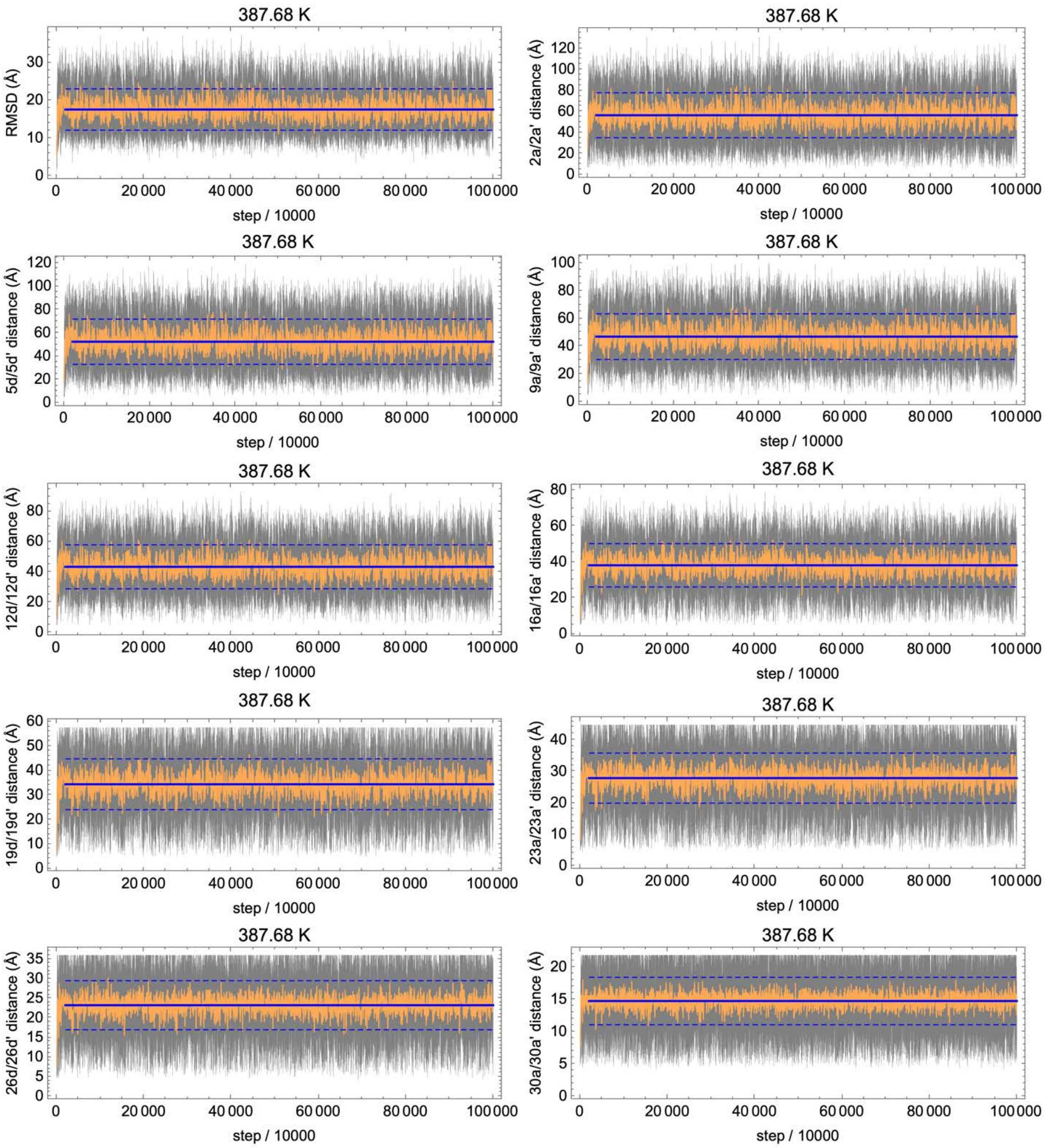

Measure at 387.68 K	Average (Å)	Standard Deviation (Å)	Slope (Å / μs)
RMSD	17.5506	5.45859	−0.0890424
5d/5d' distance	52.3664	19.4157	−0.319678
12d/12d' distance	43.2618	14.5218	−0.156349
19d/19d' distance	34.3881	10.3665	−0.0986285
26d/26d' distance	23.2336	6.26133	0.0419695
2a/2a' distance	56.4105	21.3964	−0.297005
9a/9a' distance	46.8694	16.4658	−0.235604
16a/16a' distance	38.1544	12.0526	−0.12511
23a/23a' distance	27.8928	7.95595	−0.00853701
30a/30a' distance	14.7338	3.66961	0.0266668
Core Distance	37.479	18.8836	

Figure S235. Plots of RMSD and residue packing distances for T-REMD simulations of variant **27e/22g'-xx** at 387.68 K.

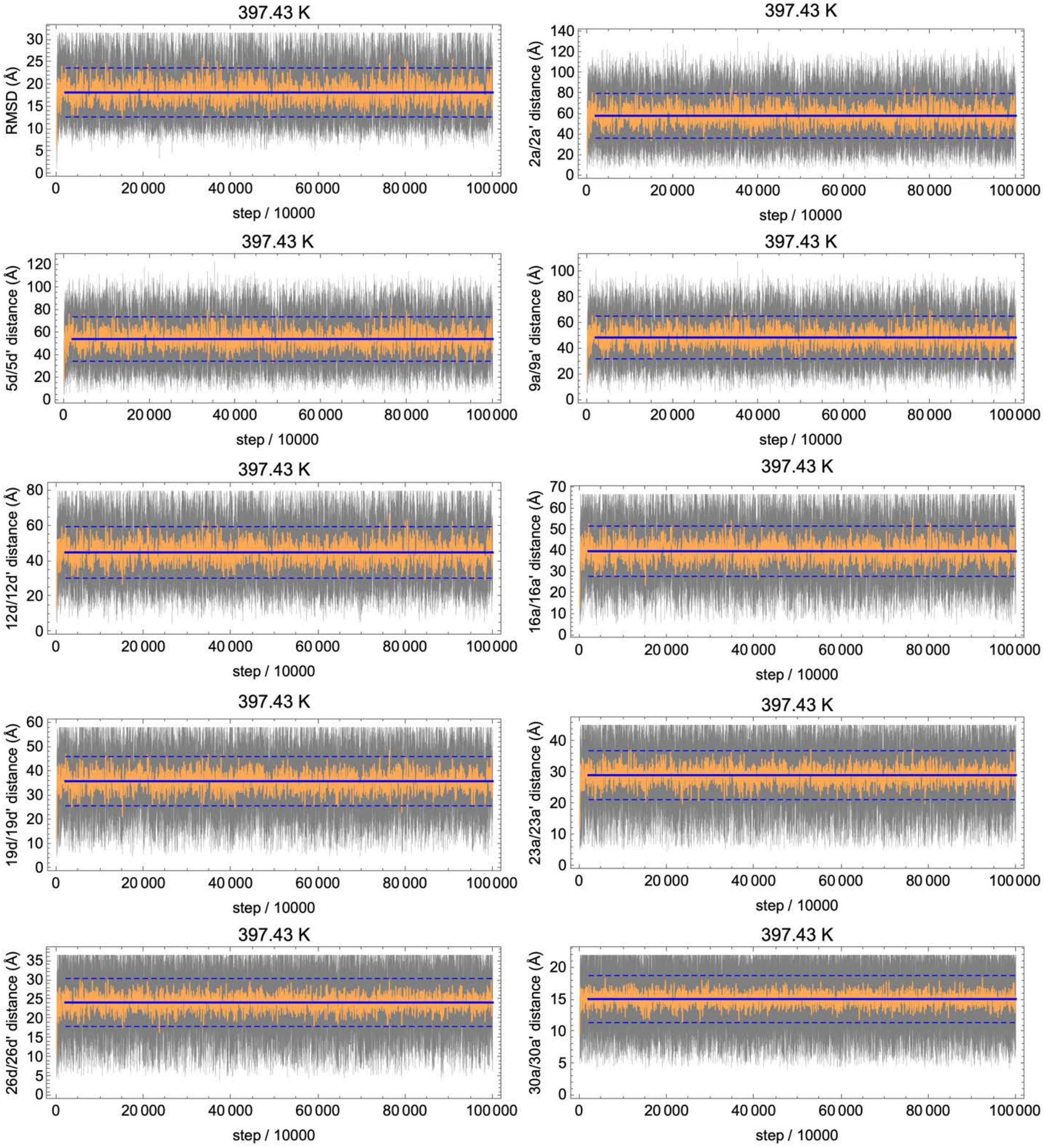

Measure at 397.43 K	Average (Å)	Standard Deviation (Å)	Slope (Å / μs)
RMSD	18.1988	5.49086	−0.147607
5d/5d' distance	54.1916	19.6872	−0.48884
12d/12d' distance	44.9789	14.5866	−0.324256
19d/19d' distance	35.8922	10.1696	−0.253274
26d/26d' distance	24.2277	6.21198	−0.0599616
2a/2a' distance	58.2184	21.7316	−0.421426
9a/9a' distance	48.6571	16.6128	−0.387494
16a/16a' distance	39.7611	11.9192	−0.273857
23a/23a' distance	29.1177	7.85867	−0.114323
30a/30a' distance	15.1048	3.67164	−0.0439392
Core Distance	38.9055	19.2471	

Figure S236. Plots of RMSD and residue packing distances for T-REMD simulations of variant **27e/22g’-xx** at 397.43 K.

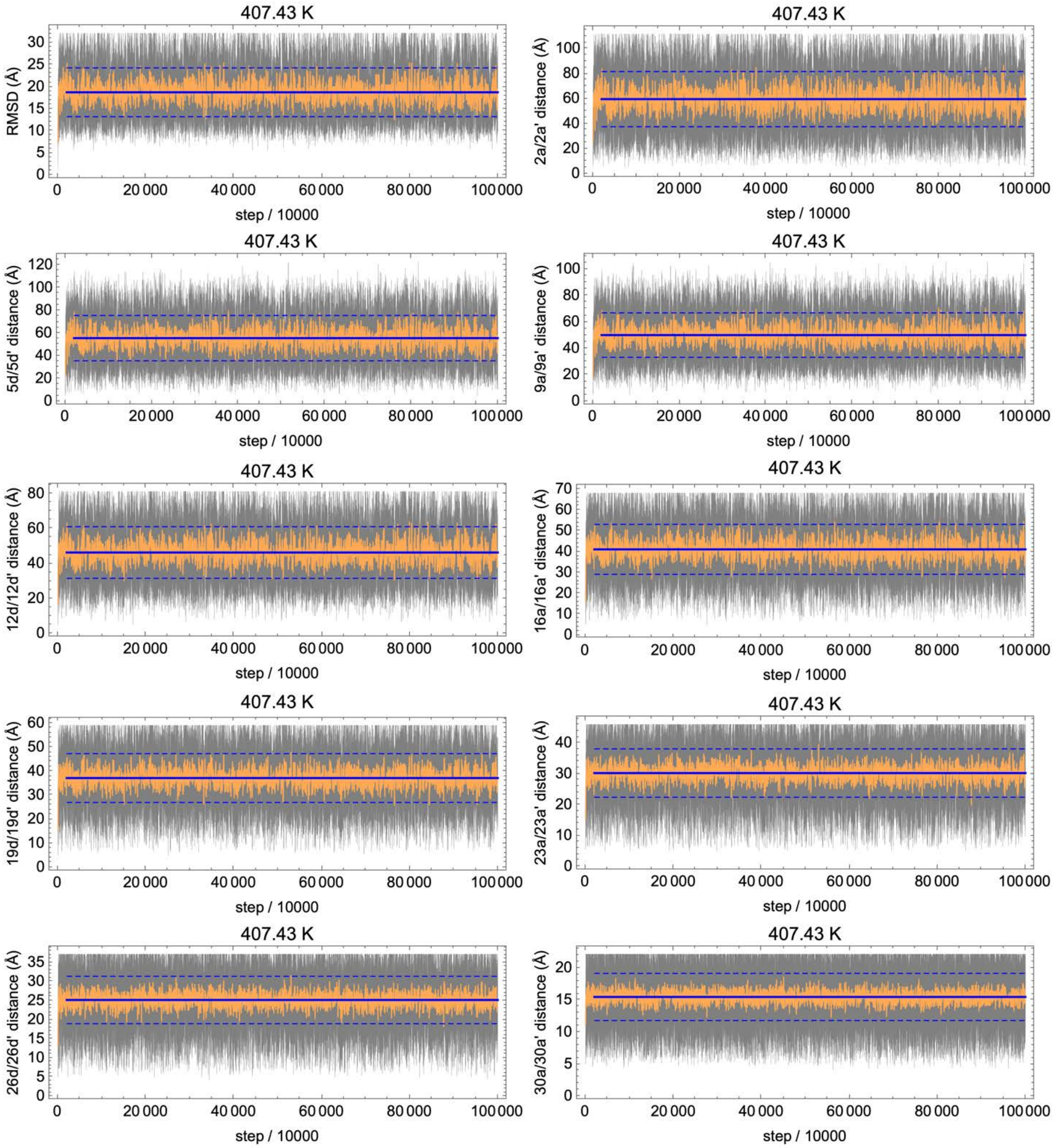

Measure at 407.43 K	Average (Å)	Standard Deviation (Å)	Slope (Å / μs)
RMSD	18.7222	5.52346	−0.147925
5d/5d' distance	55.5258	19.9756	−0.421076
12d/12d' distance	46.3251	14.7774	−0.286291
19d/19d' distance	37.1676	10.1638	−0.298378
26d/26d' distance	25.1665	6.2041	−0.123908
2a/2a' distance	59.5622	22.0802	−0.313179
9a/9a' distance	49.9987	16.8332	−0.3161
16a/16a' distance	41.0881	11.9769	−0.287532
23a/23a' distance	30.2598	7.83494	−0.203599
30a/30a' distance	15.4807	3.68035	−0.0758018
Core Distance	40.0639	19.5432	

Figure S237. Plots of RMSD and residue packing distances for T-REMD simulations of variant **27e/22g'-xx** at 407.43 K.

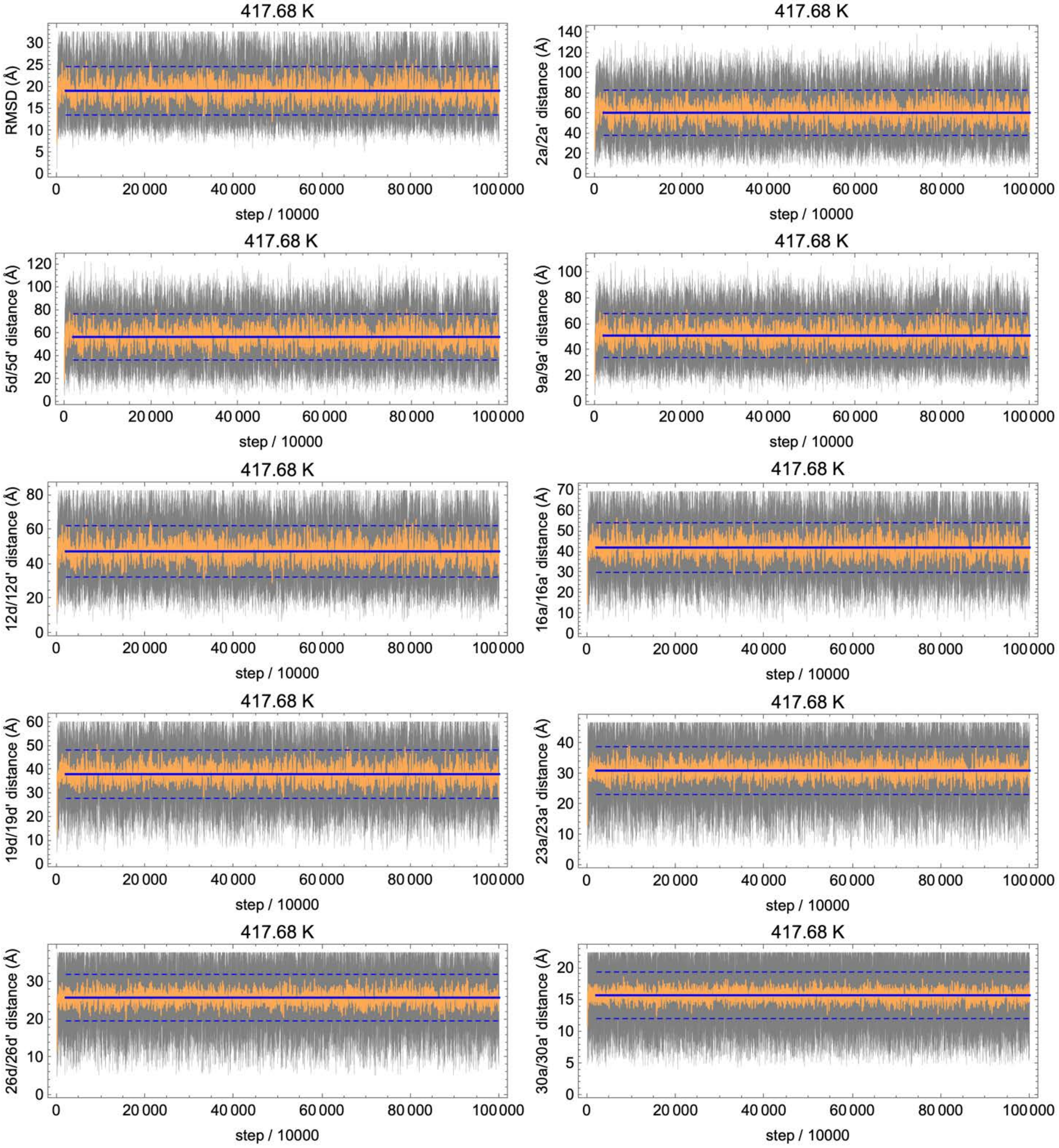

Measure at 417.68 K	Average (Å)	Standard Deviation (Å)	Slope (Å / μs)
RMSD	19.1527	5.5472	−0.219403
5d/5d' distance	56.6313	20.1656	−0.639942
12d/12d' distance	47.4305	14.8933	−0.475574
19d/19d' distance	38.1468	10.2074	−0.340027
26d/26d' distance	25.8201	6.15898	−0.114402
2a/2a' distance	60.6859	22.3406	−0.623811
9a/9a' distance	51.0923	16.9923	−0.547335
16a/16a' distance	42.1305	12.0498	−0.405048
23a/23a' distance	31.0824	7.8408	−0.177195
30a/30a' distance	15.748	3.66804	−0.0850008
Core Distance	40.9742	19.8032	

Figure S238. Plots of RMSD and residue packing distances for T-REMD simulations of variant **27e/22g'-xx** at 417.68 K.

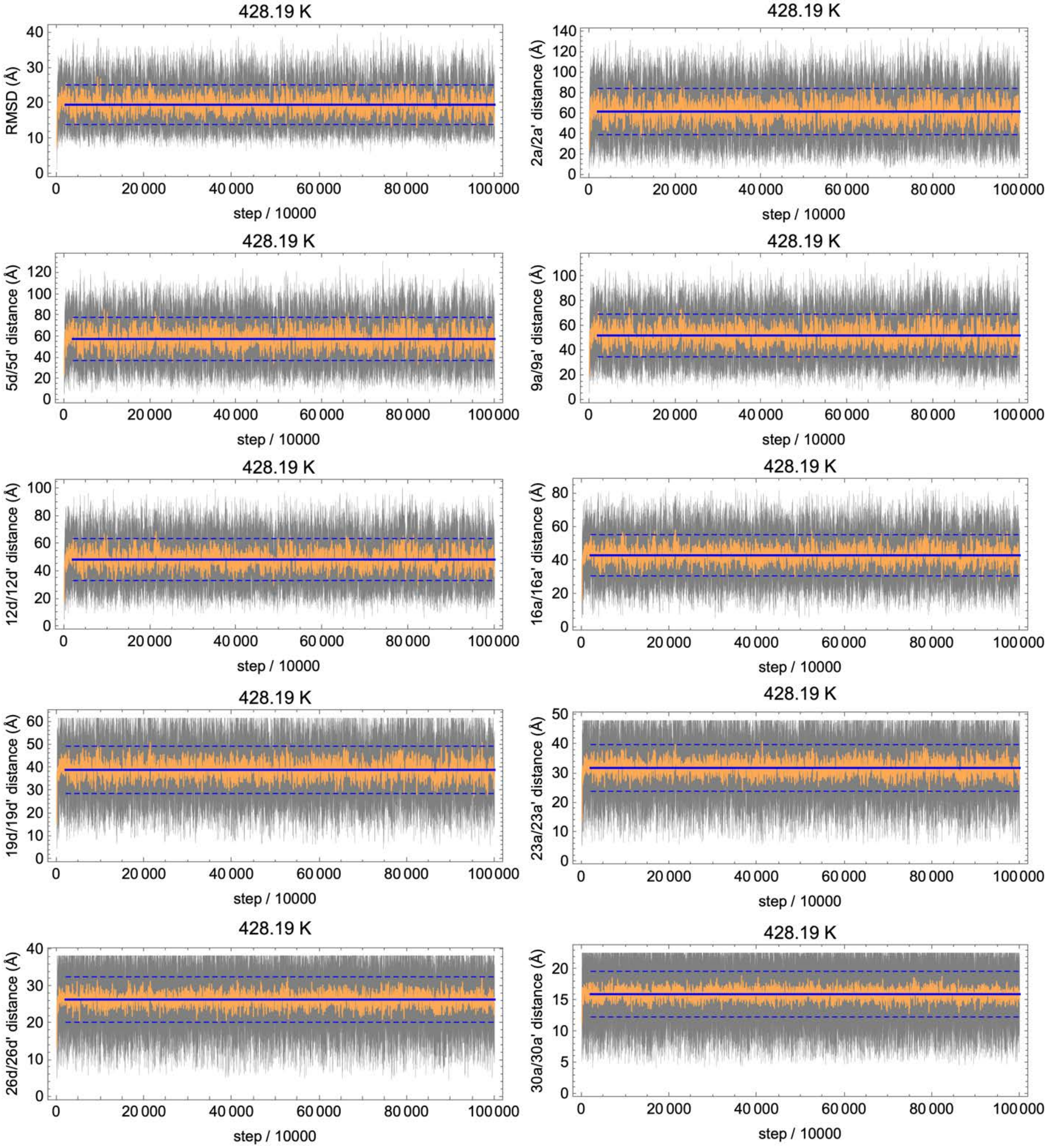

Measure at 428.19 K	Average (Å)	Standard Deviation (Å)	Slope (Å / μs)
RMSD	19.5996	5.61569	−0.211911
5d/5d' distance	57.8056	20.4623	−0.625997
12d/12d' distance	48.5034	15.1982	−0.425267
19d/19d' distance	39.0524	10.3489	−0.246842
26d/26d' distance	26.4032	6.16434	−0.1042
2a/2a' distance	61.9118	22.6012	−0.653383
9a/9a' distance	52.2186	17.3125	−0.48147
16a/16a' distance	43.1041	12.2905	−0.354933
23a/23a' distance	31.8687	7.9376	−0.158474
30a/30a' distance	16.0175	3.65976	−0.055667
Core Distance	41.8761	20.1565	

Figure S239. Plots of RMSD and residue packing distances for T-REMD simulations of variant **27e/22g’-xx** at 428.19 K.

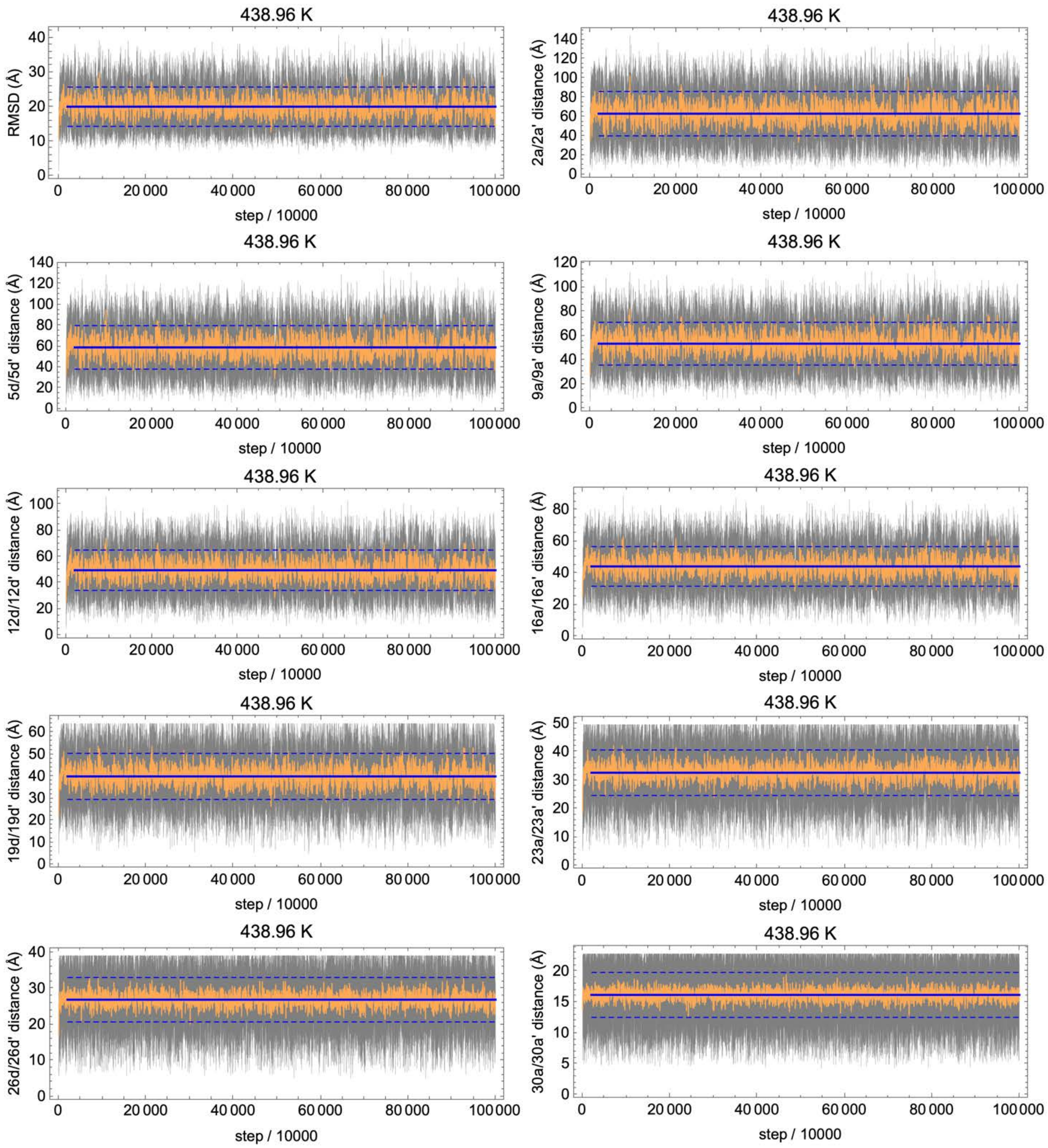

Measure at 438.96 K	Average (Å)	Standard Deviation (Å)	Slope (Å / μs)
RMSD	20.0356	5.68999	−0.194359
5d/5d' distance	58.9443	20.8573	−0.500456
12d/12d' distance	49.5757	15.4602	−0.406771
19d/19d' distance	39.9905	10.4628	−0.296525
26d/26d' distance	26.9143	6.1645	−0.157225
2a/2a' distance	63.0791	23.0505	−0.560107
9a/9a' distance	53.3396	17.633	−0.432202
16a/16a' distance	44.1386	12.4748	−0.356256
23a/23a' distance	32.647	8.00121	−0.216549
30a/30a' distance	16.1862	3.64321	−0.0780098
Core Distance	42.7572	20.5452	

Figure S240. Plots of RMSD and residue packing distances for T-REMD simulations of variant **27e/22g'-xx** at 438.96 K.

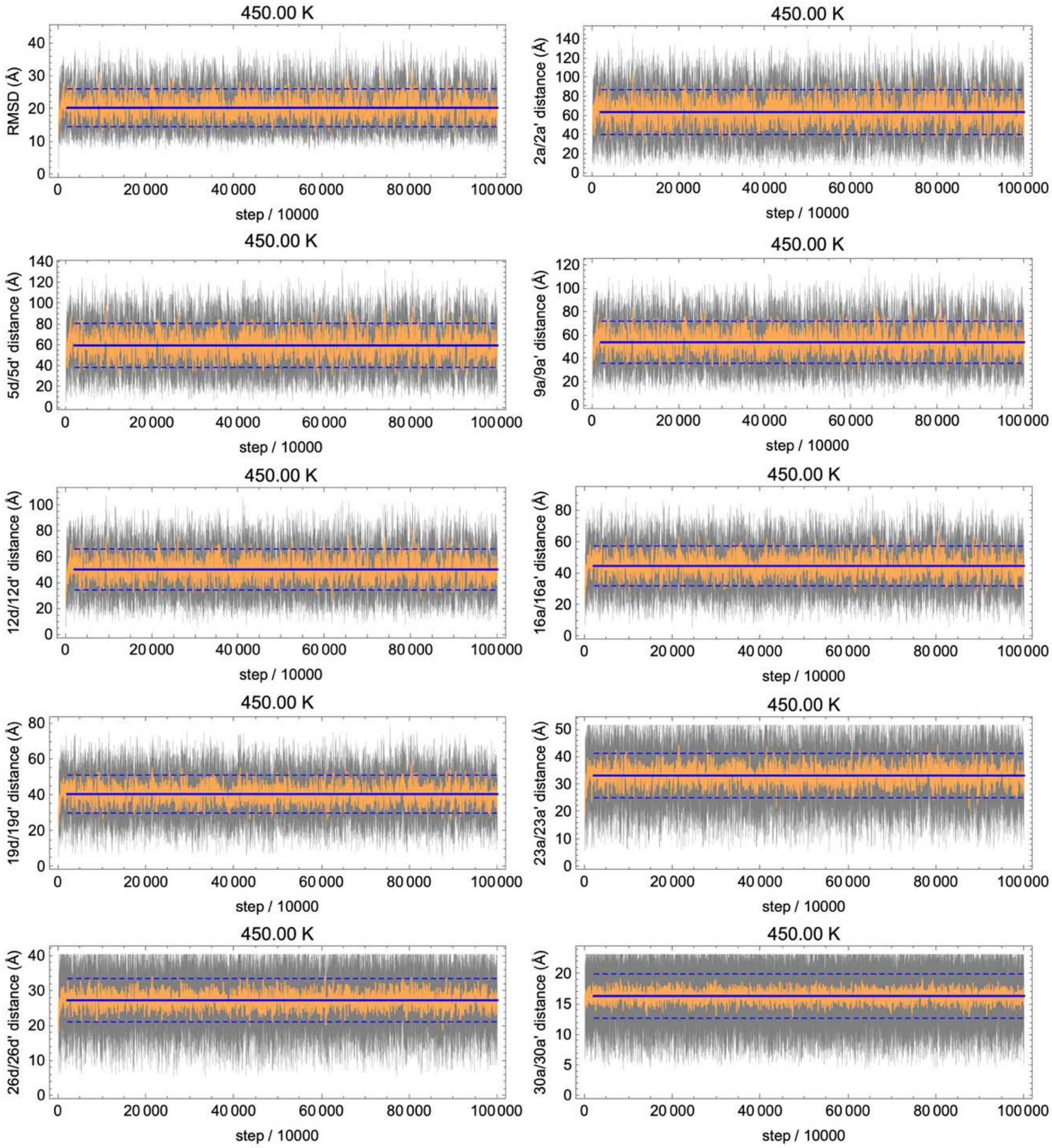

Measure at 450.00 K	Average (Å)	Standard Deviation (Å)	Slope (Å / μs)
RMSD	20.4118	5.76107	−0.157952
5d/5d' distance	59.8885	21.2151	−0.313817
12d/12d' distance	50.5021	15.7583	−0.318317
19d/19d' distance	40.7072	10.6246	−0.244191
26d/26d' distance	27.3758	6.20763	−0.0707927
2a/2a' distance	64.0345	23.4248	−0.360748
9a/9a' distance	54.2793	17.989	−0.345997
16a/16a' distance	44.9697	12.7224	−0.301335
23a/23a' distance	33.2797	8.10312	−0.130886
30a/30a' distance	16.3933	3.63673	−0.0704984
Core Distance	43.4922	20.89	

Figure S241. Plots of RMSD and residue packing distances for T-REMD simulations of variant **27e/22g'-xx** at 450.00 K.

(return to Table of Contents)

s27e/22g'-x4x

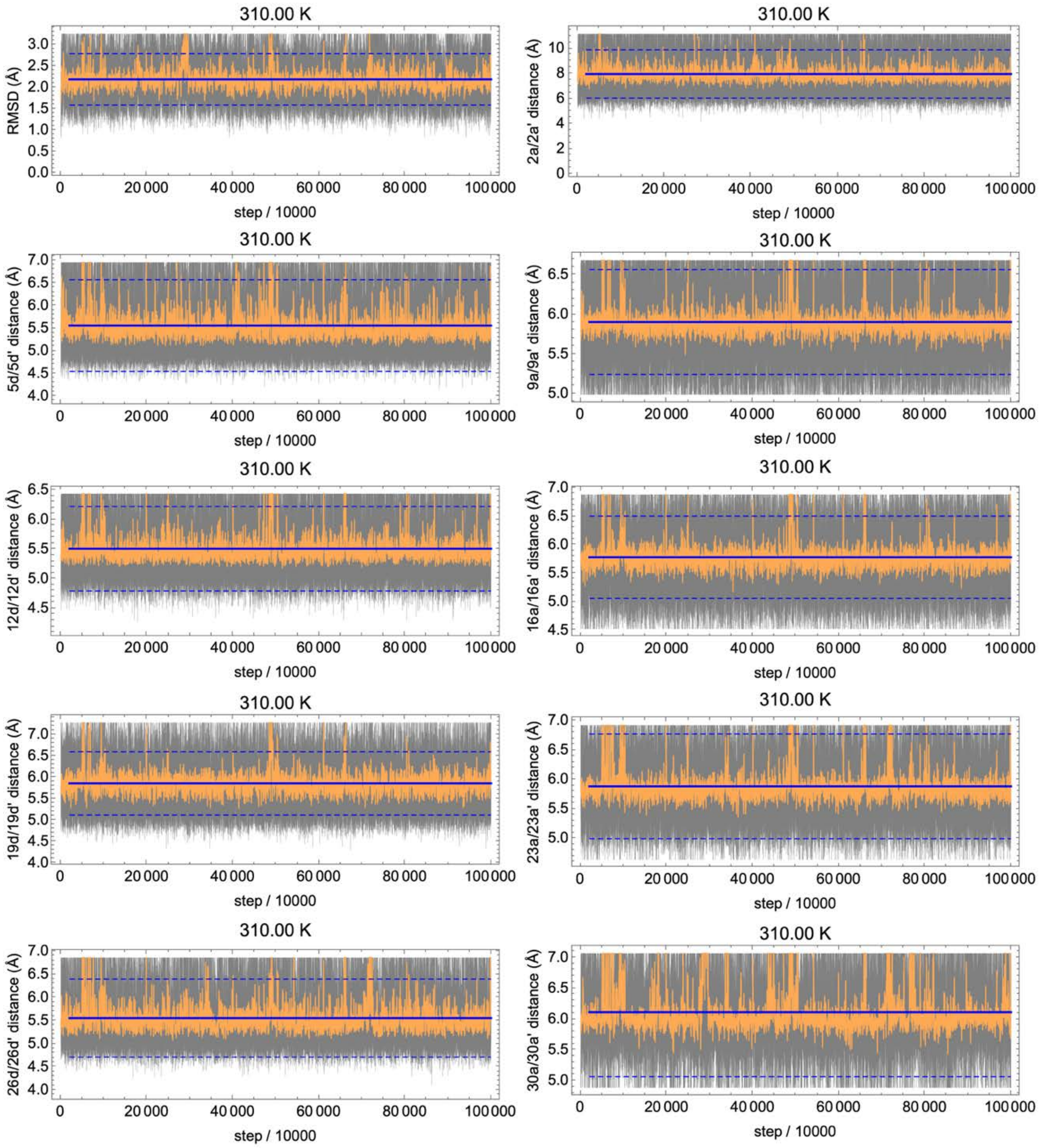

Measure at 310.00 K	Average (Å)	Standard Deviation (Å)	Slope (Å / μs)
RMSD	2.18389	0.600447	−0.0307161
5d/5d' distance	5.55651	1.01305	−0.0592661
12d/12d' distance	5.50619	0.712916	−0.024973
19d/19d' distance	5.85574	0.737158	−0.0303508
26d/26d' distance	5.55452	0.842863	−0.0369576
2a/2a' distance	7.95979	1.92432	−0.0883925
9a/9a' distance	5.90285	0.661505	−0.0285956
16a/16a' distance	5.77988	0.720957	−0.0290069
23a/23a' distance	5.87921	0.891748	−0.0469323
30a/30a' distance	6.11066	1.04779	−0.0766393
Core Distance	6.01171	1.24391	

Figure S242. Plots of RMSD and residue packing distances for T-REMD simulations of variant **s27e/22g'-x4x** at 310.00 K.

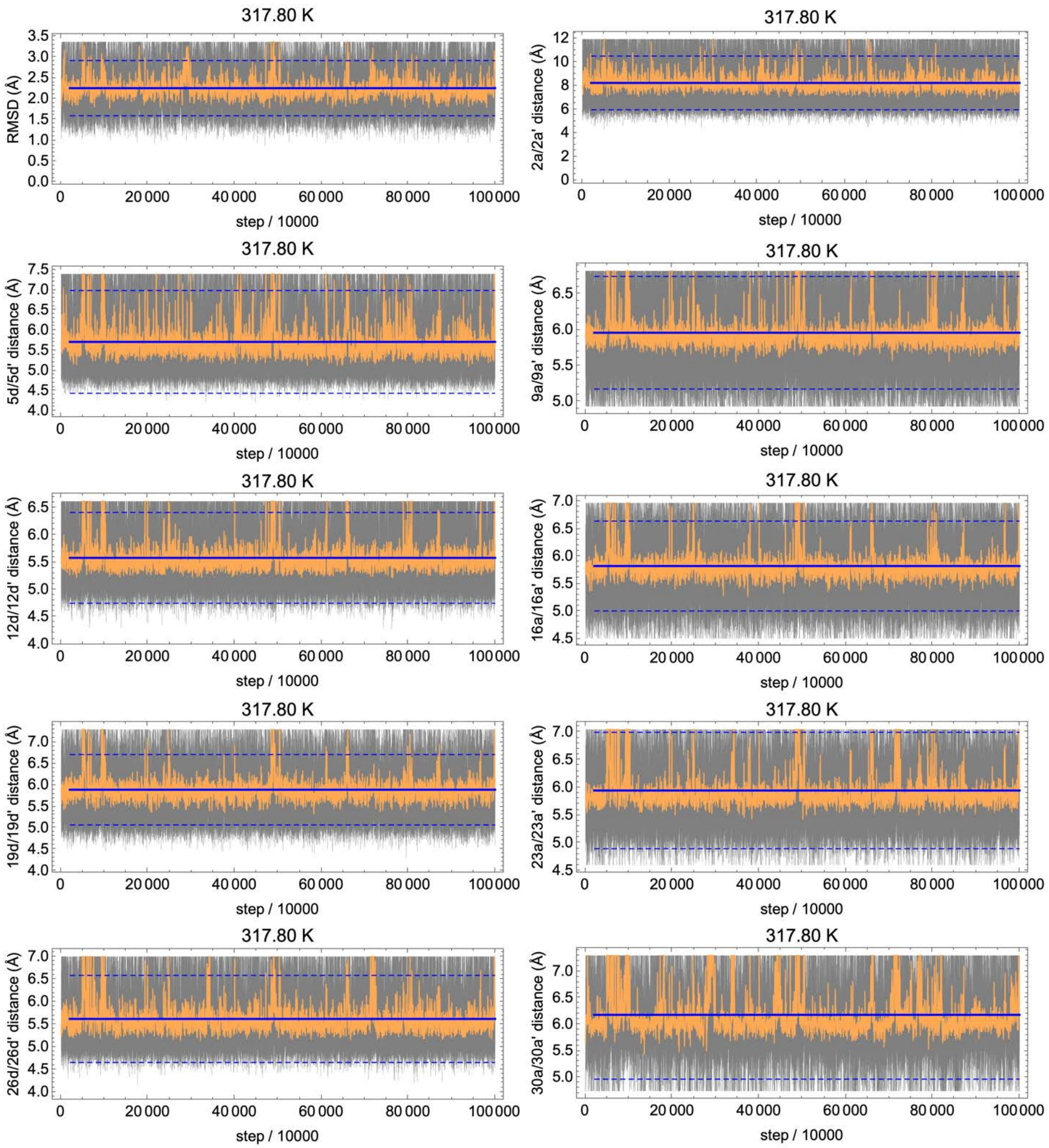

Measure at 317.80 K	Average (Å)	Standard Deviation (Å)	Slope (Å / μs)
RMSD	2.25387	0.662788	−0.0367843
5d/5d' distance	5.71239	1.27563	−0.080345
12d/12d' distance	5.58061	0.832816	−0.0390156
19d/19d' distance	5.88964	0.820488	−0.0334835
26d/26d' distance	5.61969	0.963963	−0.0596097
2a/2a' distance	8.23496	2.27408	−0.133714
9a/9a' distance	5.95906	0.783905	−0.0427749
16a/16a' distance	5.82184	0.817645	−0.0441209
23a/23a' distance	5.94615	1.05036	−0.0669815
30a/30a' distance	6.18051	1.21729	−0.0802698
Core Distance	6.10498	1.42762	

Figure S243. Plots of RMSD and residue packing distances for T-REMD simulations of variant **s27e/22g'-x4x** at 317.80 K.

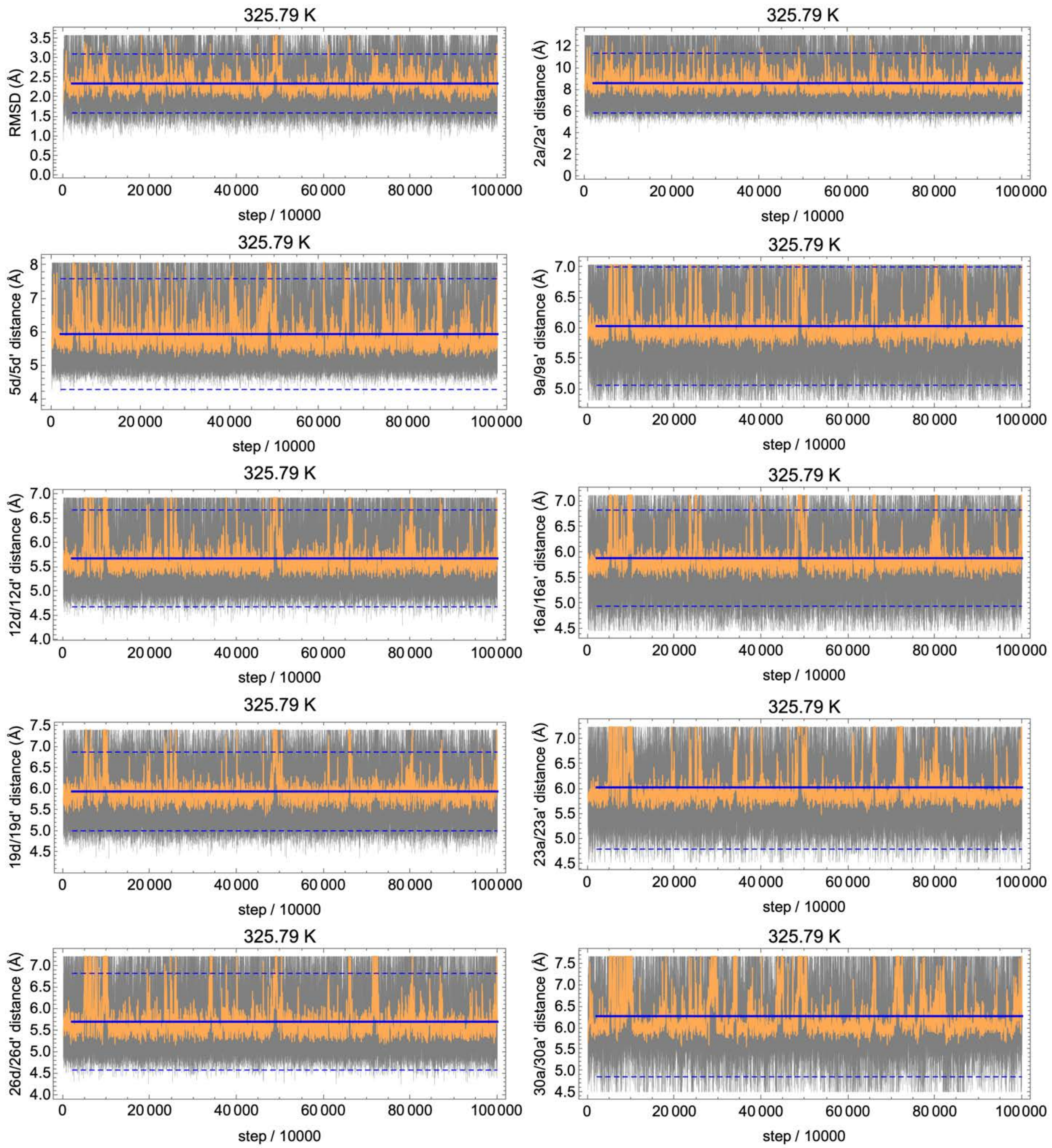

Measure at 325.79 K	Average (Å)	Standard Deviation (Å)	Slope (Å / μs)
RMSD	2.35002	0.754043	−0.0372373
5d/5d' distance	5.94026	1.65801	−0.120232
12d/12d' distance	5.67697	0.99801	−0.0530966
19d/19d' distance	5.94154	0.93509	−0.0326322
26d/26d' distance	5.7026	1.1223	−0.0705478
2a/2a' distance	8.61674	2.7569	−0.190301
9a/9a' distance	6.03596	0.970444	−0.0573547
16a/16a' distance	5.88882	0.941915	−0.0421541
23a/23a' distance	6.03305	1.23618	−0.088579
30a/30a' distance	6.27913	1.41409	−0.0985595
Core Distance	6.23501	1.68255	

Figure S244. Plots of RMSD and residue packing distances for T-REMD simulations of variant **s27e/22g’-x4x** at 325.79 K.

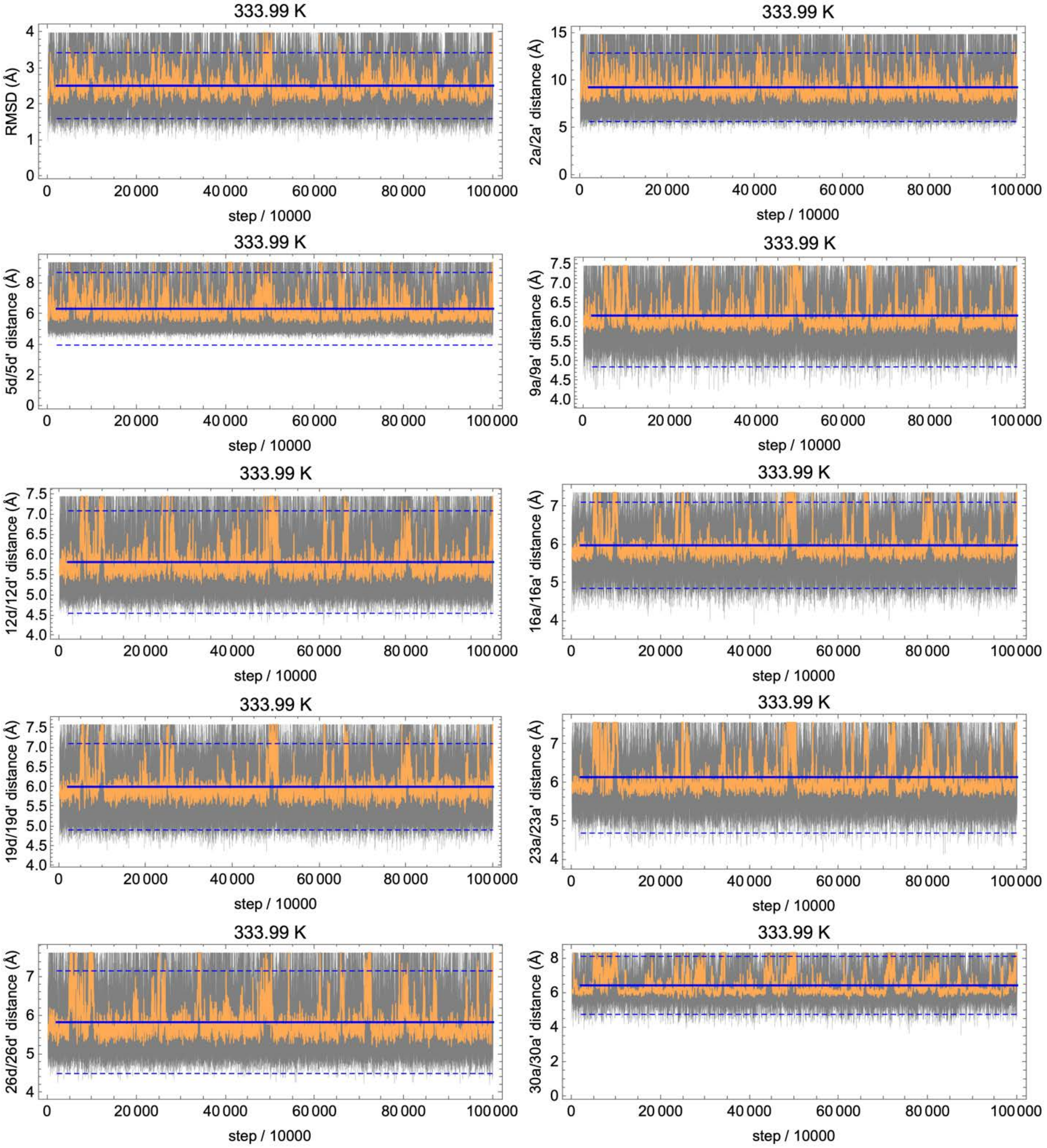

Measure at 333.99 K	Average (Å)	Standard Deviation (Å)	Slope (Å / μs)
RMSD	2.51231	0.914924	−0.0417318
5d/5d' distance	6.34669	2.36903	−0.136149
12d/12d' distance	5.82508	1.26867	−0.0583095
19d/19d' distance	6.00806	1.09705	−0.0364497
26d/26d' distance	5.82917	1.3344	−0.0820266
2a/2a' distance	9.27765	3.61963	−0.241356
9a/9a' distance	6.17845	1.32417	−0.0610342
16a/16a' distance	5.9815	1.13078	−0.0350094
23a/23a' distance	6.14654	1.45427	−0.0961964
30a/30a' distance	6.43687	1.68225	−0.0934957
Core Distance	6.44778	2.12518	

Figure S245. Plots of RMSD and residue packing distances for T-REMD simulations of variant **s27e/22g'-x4x** at 333.99 K.

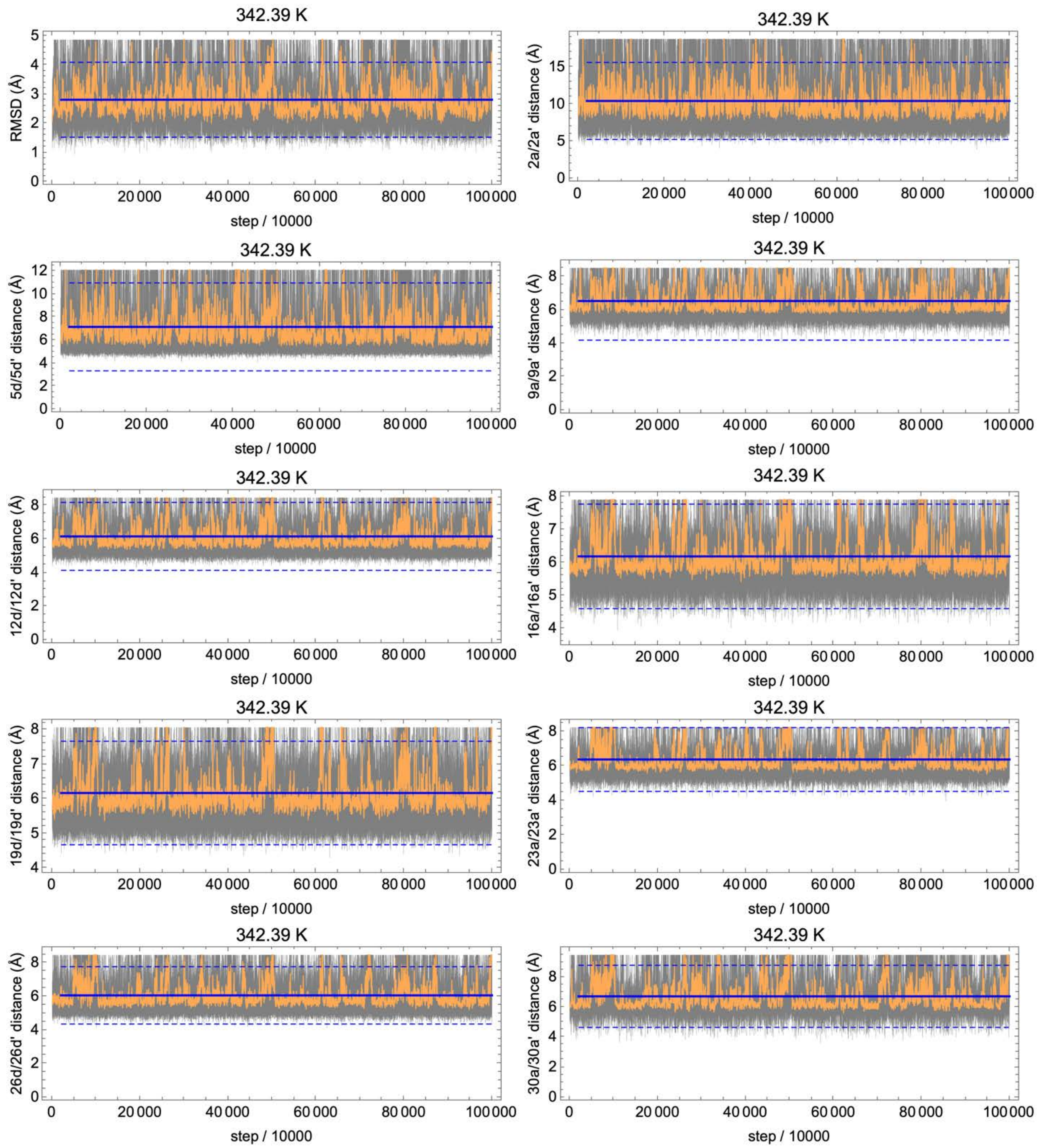

Measure at 342.39 K	Average (Å)	Standard Deviation (Å)	Slope (Å / μs)
RMSD	2.8125	1.28294	−0.0349691
5d/5d' distance	7.15084	3.813	−0.202817
12d/12d' distance	6.15636	2.02306	−0.0532333
19d/19d' distance	6.16706	1.49308	−0.00892481
26d/26d' distance	6.06233	1.70667	−0.0602612
2a/2a' distance	10.4263	5.17696	−0.355622
9a/9a' distance	6.53309	2.34569	−0.0746684
16a/16a' distance	6.18181	1.585	−0.0226973
23a/23a' distance	6.3724	1.84854	−0.0775399
30a/30a' distance	6.71185	2.06781	−0.0843655
Core Distance	6.86245	3.00966	

Figure S246. Plots of RMSD and residue packing distances for T-REMD simulations of variant **s27e/22g'-x4x** at 342.39 K.

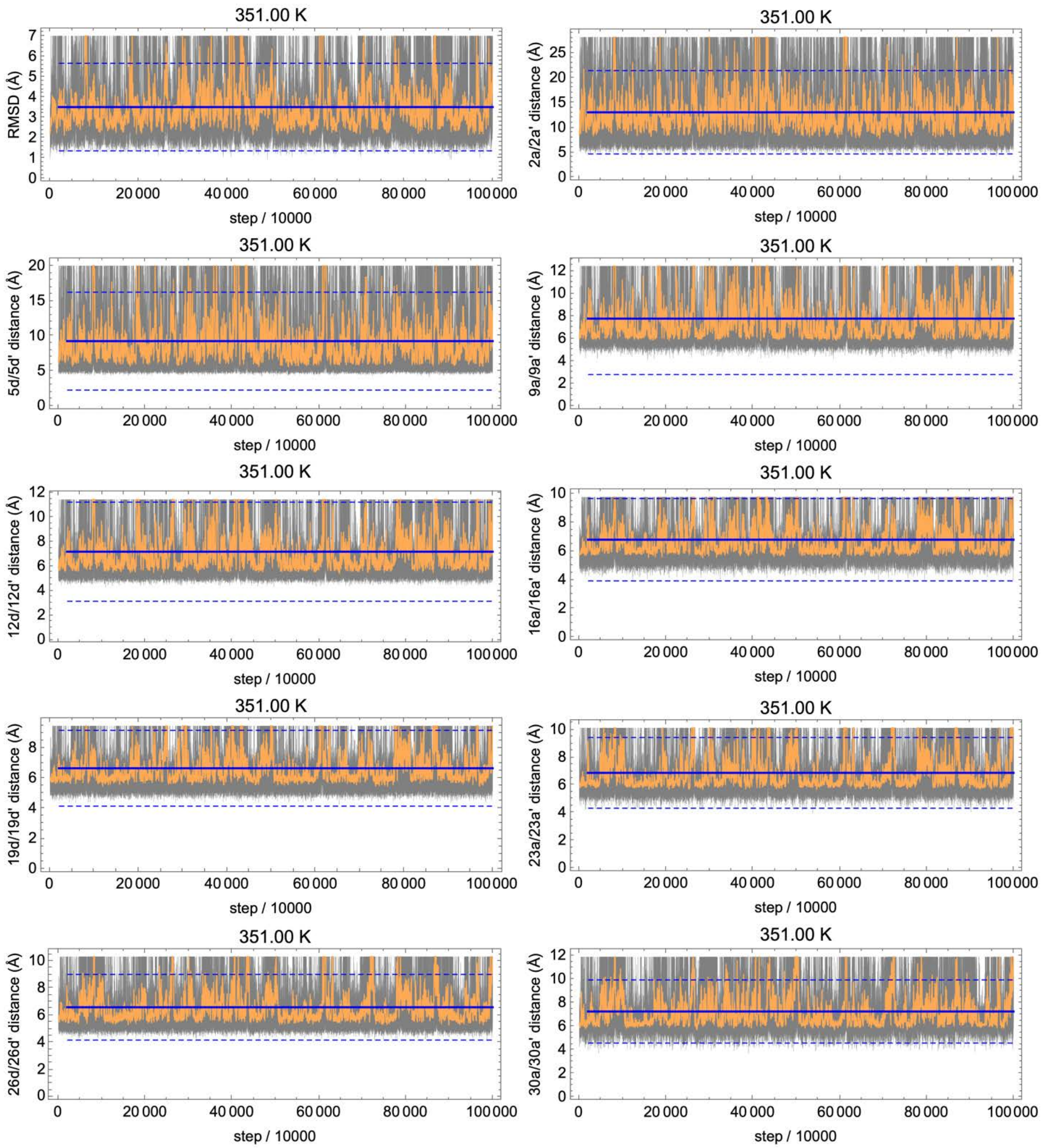

Measure at 351.00 K	Average (Å)	Standard Deviation (Å)	Slope (Å / μs)
RMSD	3.51661	2.14819	0.00935719
5d/5d' distance	9.25189	6.99549	−0.148061
12d/12d' distance	7.17719	4.04351	0.0403491
19d/19d' distance	6.65369	2.51002	0.118714
26d/26d' distance	6.58996	2.41445	0.0482981
2a/2a' distance	13.0586	8.36846	−0.326141
9a/9a' distance	7.76084	4.9567	0.0129964
16a/16a' distance	6.79324	2.8693	0.0967907
23a/23a' distance	6.88034	2.55603	0.0285911
30a/30a' distance	7.25746	2.67694	0.00775877
Core Distance	7.93591	5.04273	

Figure S247. Plots of RMSD and residue packing distances for T-REMD simulations of variant **s27e/22g'-x4x** at 351.00 K.

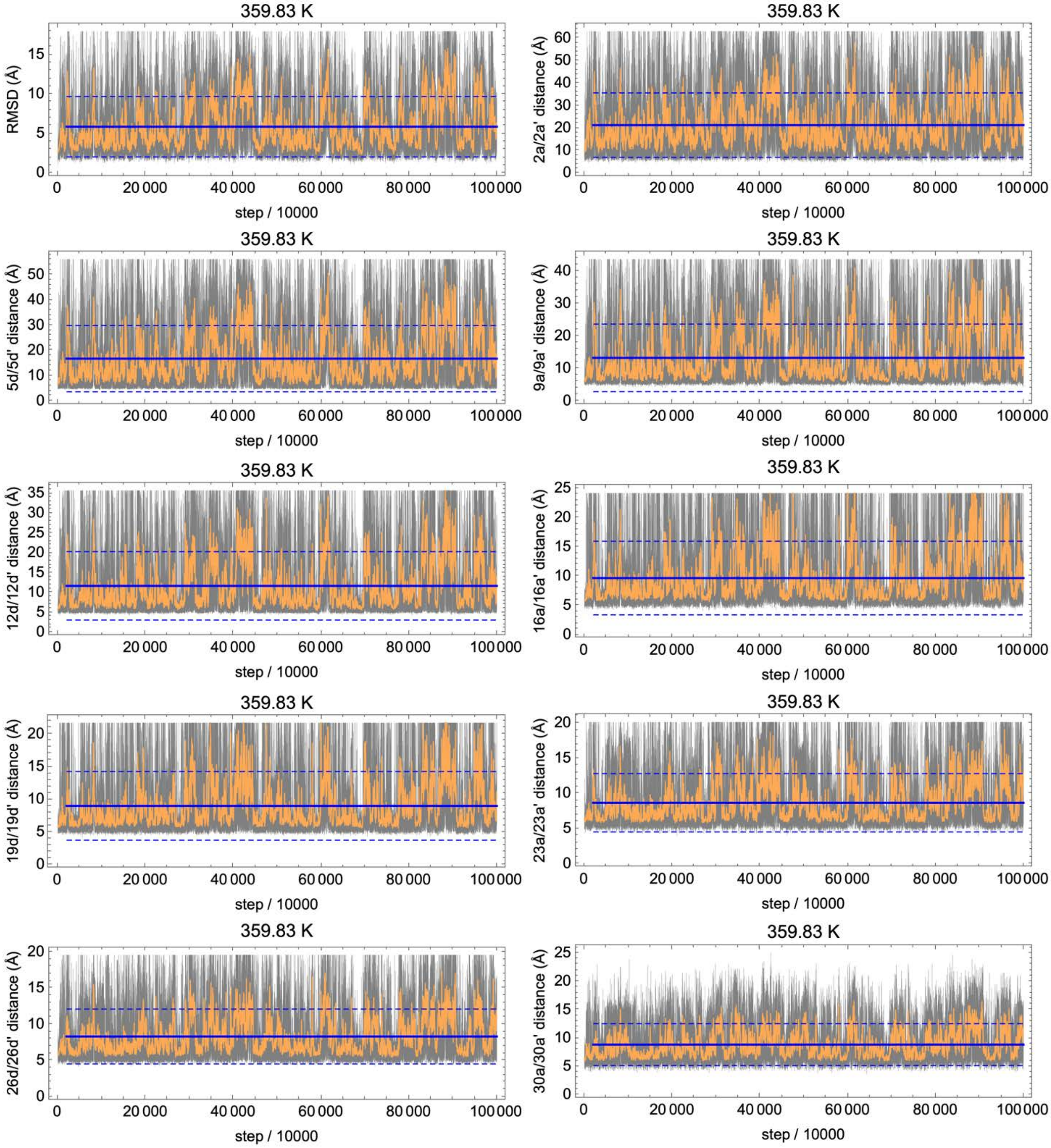

Measure at 359.83 K	Average (Å)	Standard Deviation (Å)	Slope (Å / μs)
RMSD	5.85253	3.83086	0.933052
5d/5d' distance	16.7363	13.1329	2.71045
12d/12d' distance	11.6612	8.61095	1.82411
19d/19d' distance	8.98156	5.25816	1.22662
26d/26d' distance	8.30736	3.79383	0.824304
2a/2a' distance	21.254	14.3418	2.6119
9a/9a' distance	13.2534	10.4103	2.32373
16a/16a' distance	9.68211	6.27809	1.33209
23a/23a' distance	8.65189	4.13801	0.816791
30a/30a' distance	8.82518	3.69541	0.679094
Core Distance	11.9281	9.61498	

Figure S248. Plots of RMSD and residue packing distances for T-REMD simulations of variant **s27e/22g'-x4x** at 359.83 K.

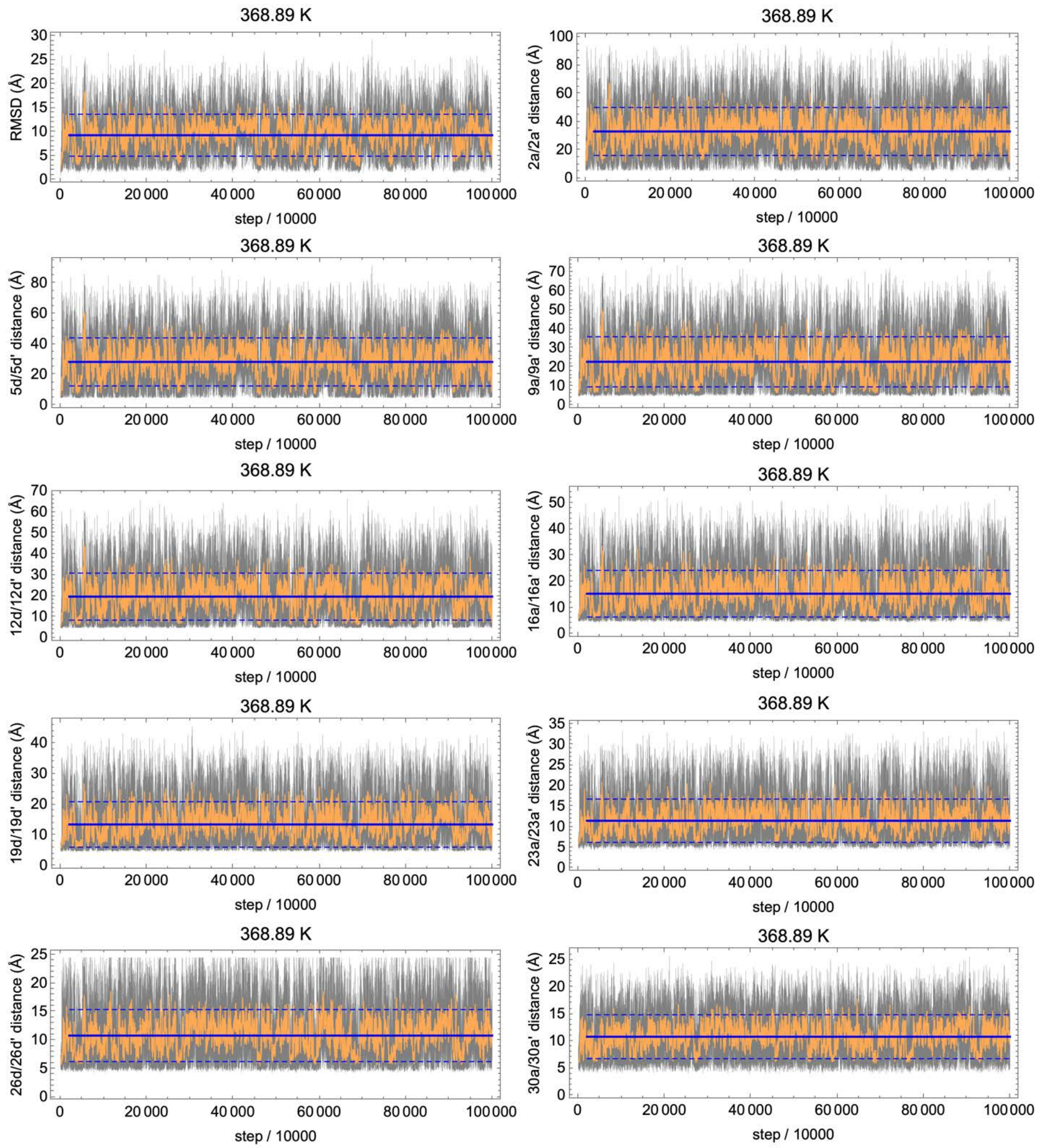

Measure at 368.89 K	Average (Å)	Standard Deviation (Å)	Slope (Å / μs)
RMSD	9.2769	4.37474	0.338134
5d/5d' distance	28.302	15.7458	0.873577
12d/12d' distance	19.6462	11.2264	0.628299
19d/19d' distance	13.4611	7.424	0.688381
26d/26d' distance	10.7989	4.57242	0.262191
2a/2a' distance	33.1341	16.9724	0.816482
9a/9a' distance	22.7692	13.1712	0.782176
16a/16a' distance	15.3116	8.91214	0.591942
23a/23a' distance	11.4689	5.26801	0.284095
30a/30a' distance	10.8122	4.04815	0.183784
Core Distance	18.4116	13.2001	

Figure S249. Plots of RMSD and residue packing distances for T-REMD simulations of variant **s27e/22g'-x4x** at 368.89 K.

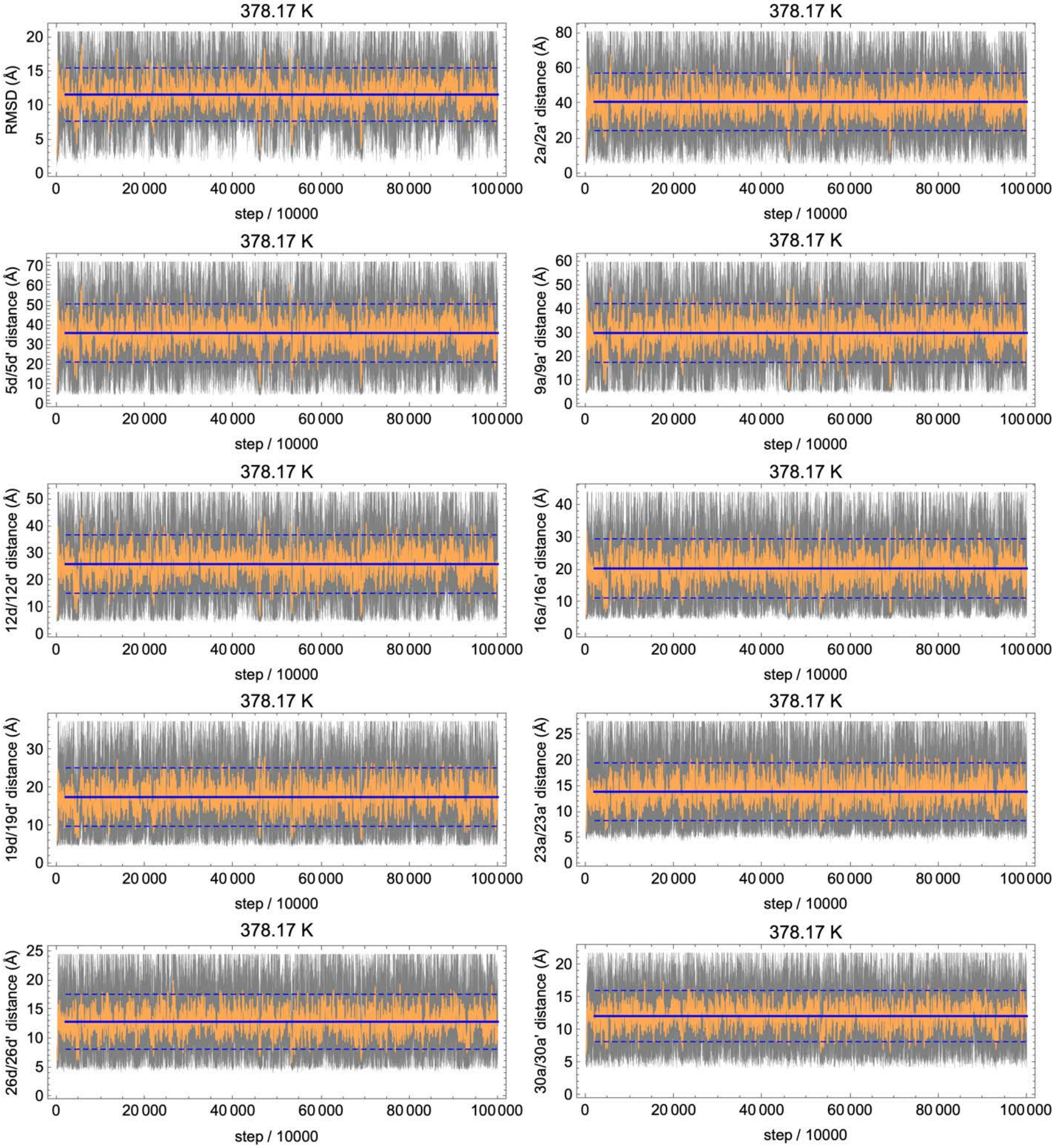

Measure at 378.17 K	Average (Å)	Standard Deviation (Å)	Slope (Å / μs)
RMSD	11.6299	3.90878	0.197697
5d/5d' distance	36.1428	14.7823	0.493532
12d/12d' distance	26.0501	10.8046	0.526383
19d/19d' distance	17.5729	7.70279	0.562473
26d/26d' distance	12.8973	4.73566	0.0614352
2a/2a' distance	40.9031	16.3556	0.359312
9a/9a' distance	30.0136	12.3449	0.518064
16a/16a' distance	20.4034	9.13021	0.511096
23a/23a' distance	13.9367	5.58744	0.199329
30a/30a' distance	12.1265	3.93167	0.0289498
Core Distance	23.3385	14.3616	

Figure S250. Plots of RMSD and residue packing distances for T-REMD simulations of variant **s27e/22g'-x4x** at 378.17 K.

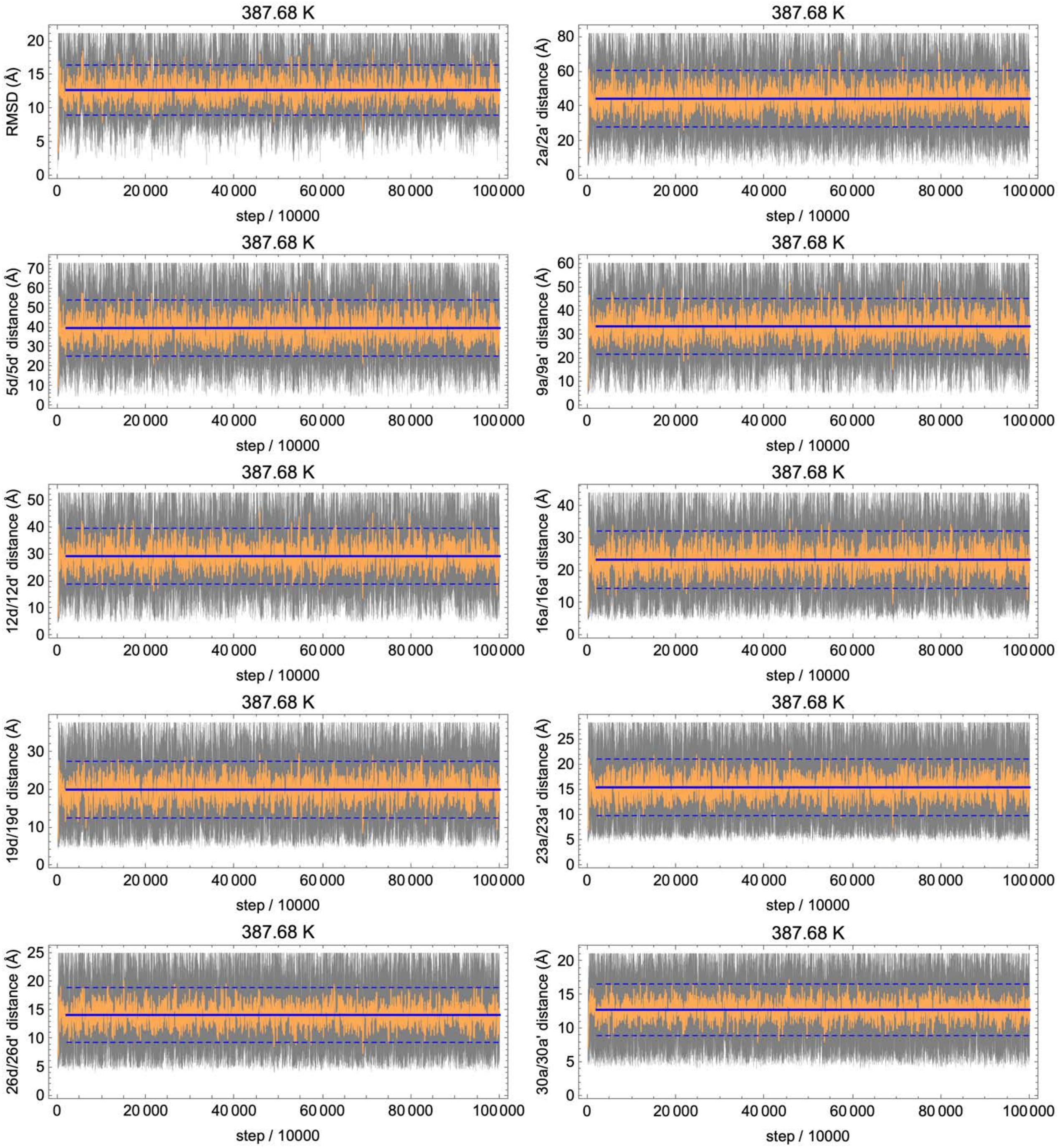

Measure at 387.68 K	Average (Å)	Standard Deviation (Å)	Slope (Å / μs)
RMSD	12.757	3.71742	0.0620129
5d/5d' distance	39.8407	14.3895	0.103258
12d/12d' distance	29.4033	10.3337	0.15313
19d/19d' distance	20.0548	7.52395	0.2256
26d/26d' distance	14.1809	4.81266	−0.0717038
2a/2a' distance	44.4855	16.2893	0.0161692
9a/9a' distance	33.5852	11.789	0.138119
16a/16a' distance	23.413	8.86277	0.110512
23a/23a' distance	15.5013	5.63596	0.0805789
30a/30a' distance	12.8118	3.83078	−0.0651462
Core Distance	25.9196	14.874	

Figure S251. Plots of RMSD and residue packing distances for T-REMD simulations of variant **s27e/22g’-x4x** at 387.68 K.

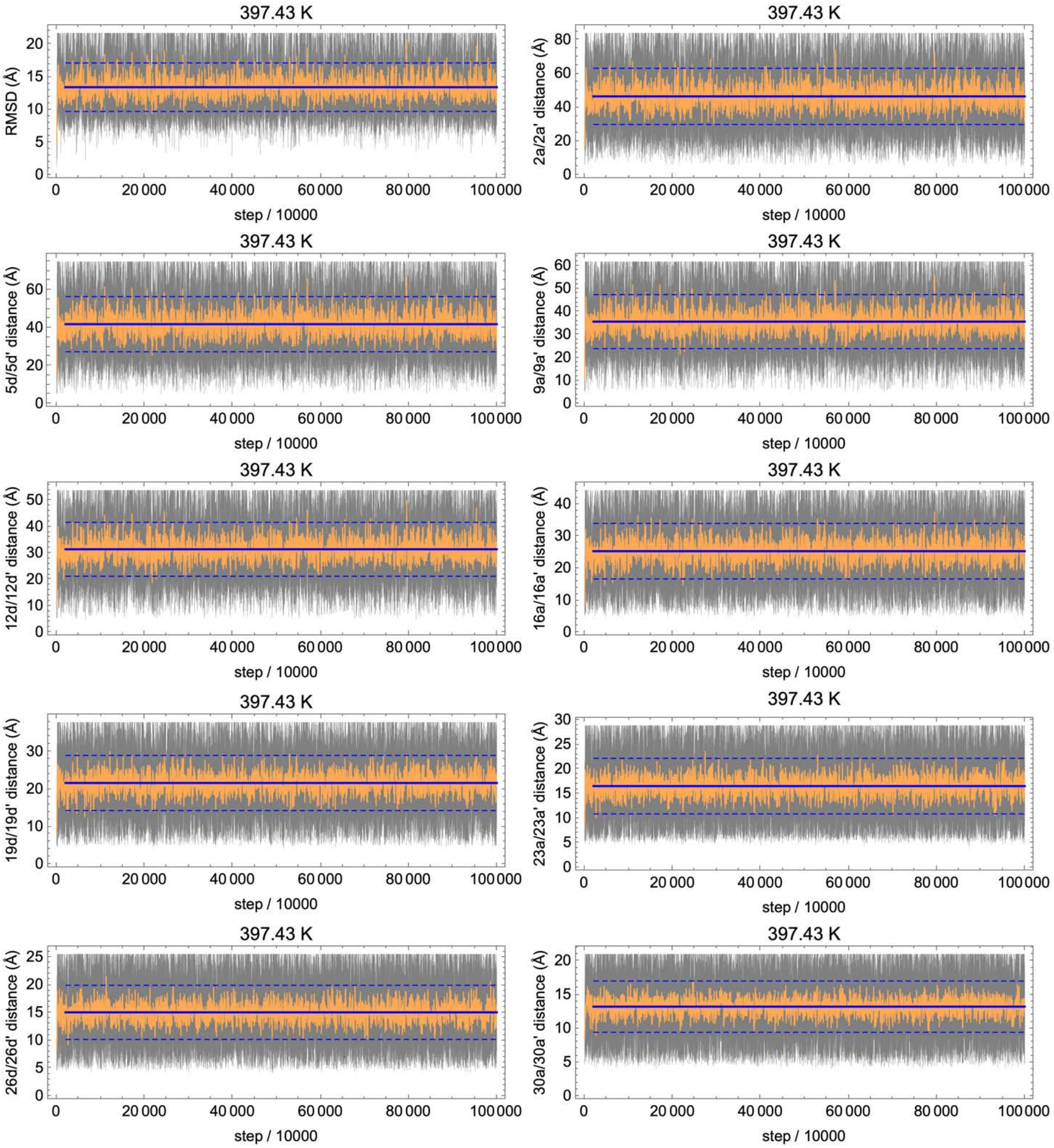

Measure at 397.43 K	Average (Å)	Standard Deviation (Å)	Slope (Å / μs)
RMSD	13.4289	3.7075	0.0439393
5d/5d' distance	41.9293	14.5399	0.0205406
12d/12d' distance	31.4434	10.2066	0.161799
19d/19d' distance	21.6621	7.3567	0.211033
26d/26d' distance	15.0689	4.86903	0.00138791
2a/2a' distance	46.5207	16.6246	−0.103237
9a/9a' distance	35.67	11.7367	0.0914573
16a/16a' distance	25.3329	8.67262	0.168383
23a/23a' distance	16.5485	5.64617	0.14772
30a/30a' distance	13.2376	3.79357	−0.0338637
Core Distance	27.4904	15.2679	

Figure S252. Plots of RMSD and residue packing distances for T-REMD simulations of variant **s27e/22g’-x4x** at 397.43 K.

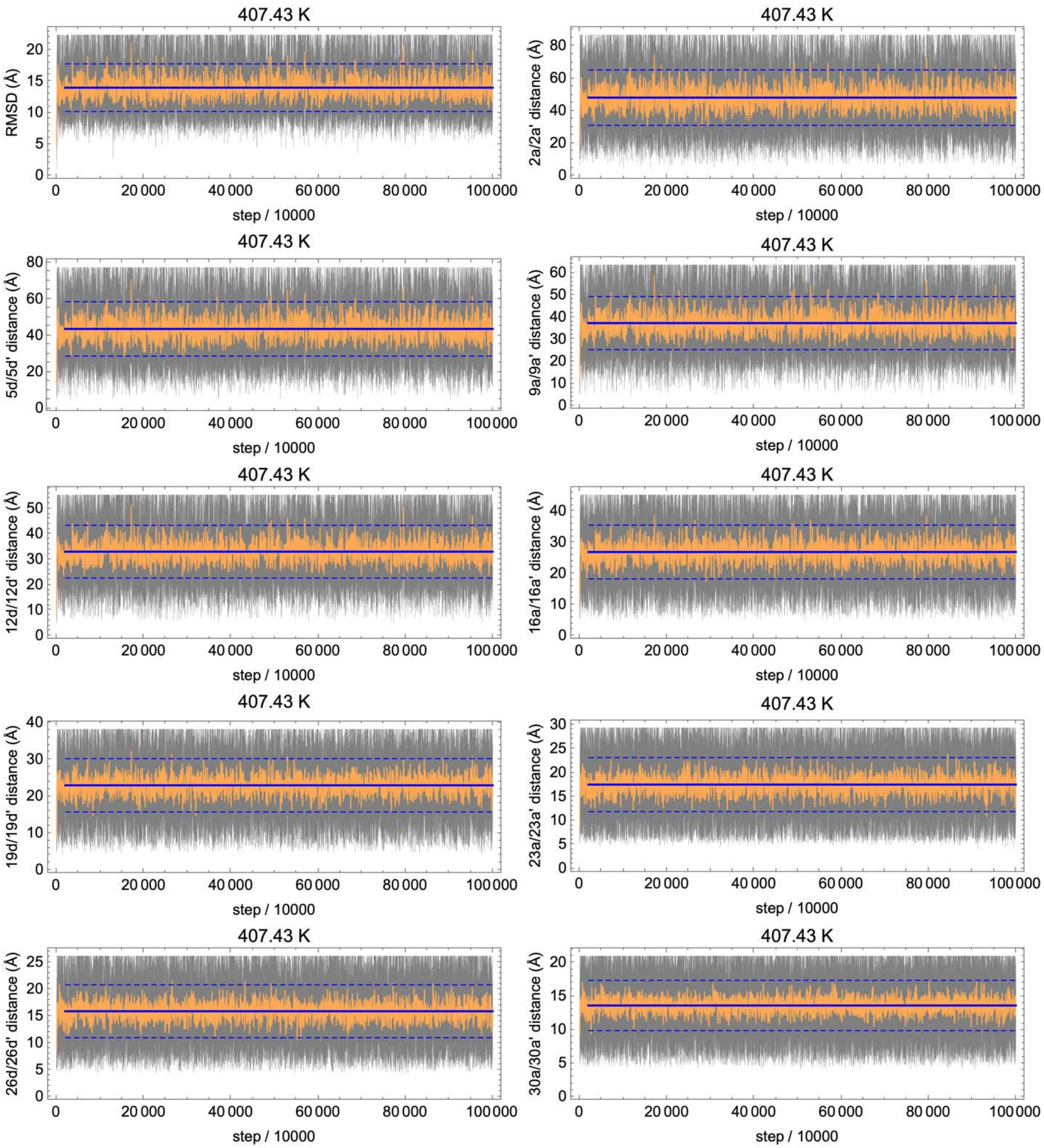

Measure at 407.43 K	Average (Å)	Standard Deviation (Å)	Slope (Å / μs)
RMSD	13.9859	3.78504	0.0168325
5d/5d' distance	43.5679	14.9289	0.0297812
12d/12d' distance	33.0468	10.372	0.0939605
19d/19d' distance	22.998	7.2297	0.153134
26d/26d' distance	15.9021	4.91909	−0.0234167
2a/2a' distance	48.1163	17.107	−0.0597747
9a/9a' distance	37.3118	11.9979	0.0569489
16a/16a' distance	26.9126	8.63714	0.153357
23a/23a' distance	17.514	5.63716	0.115786
30a/30a' distance	13.6173	3.74686	−0.025271
Core Distance	28.7763	15.6599	

Figure S253. Plots of RMSD and residue packing distances for T-REMD simulations of variant **s27e/22g'-x4x** at 407.43 K.

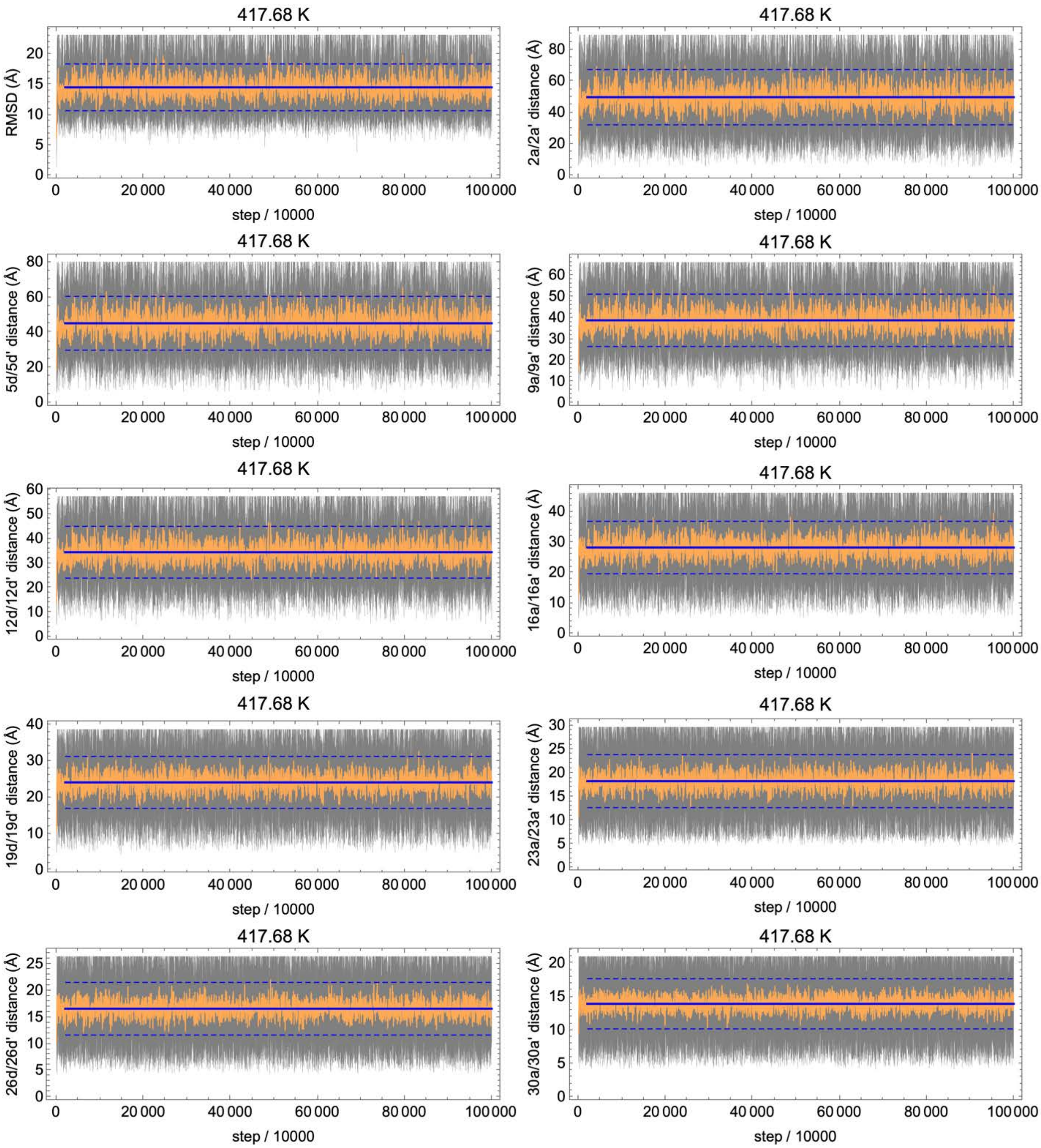

Measure at 417.68 K	Average (Å)	Standard Deviation (Å)	Slope (Å / μs)
RMSD	14.5173	3.85735	0.0139446
5d/5d' distance	45.1566	15.3517	0.0240886
12d/12d' distance	34.5027	10.553	0.0450099
19d/19d' distance	24.0938	7.14813	0.0771353
26d/26d' distance	16.6069	4.94754	−0.00935247
2a/2a' distance	49.7547	17.5702	−0.137064
9a/9a' distance	38.8237	12.3175	0.0729247
16a/16a' distance	28.2432	8.61084	0.0730469
23a/23a' distance	18.2779	5.59718	0.0590025
30a/30a' distance	13.9225	3.73923	−0.00160821
Core Distance	29.9313	16.1031	

Figure S254. Plots of RMSD and residue packing distances for T-REMD simulations of variant **s27e/22g'-x4x** at 417.68 K.

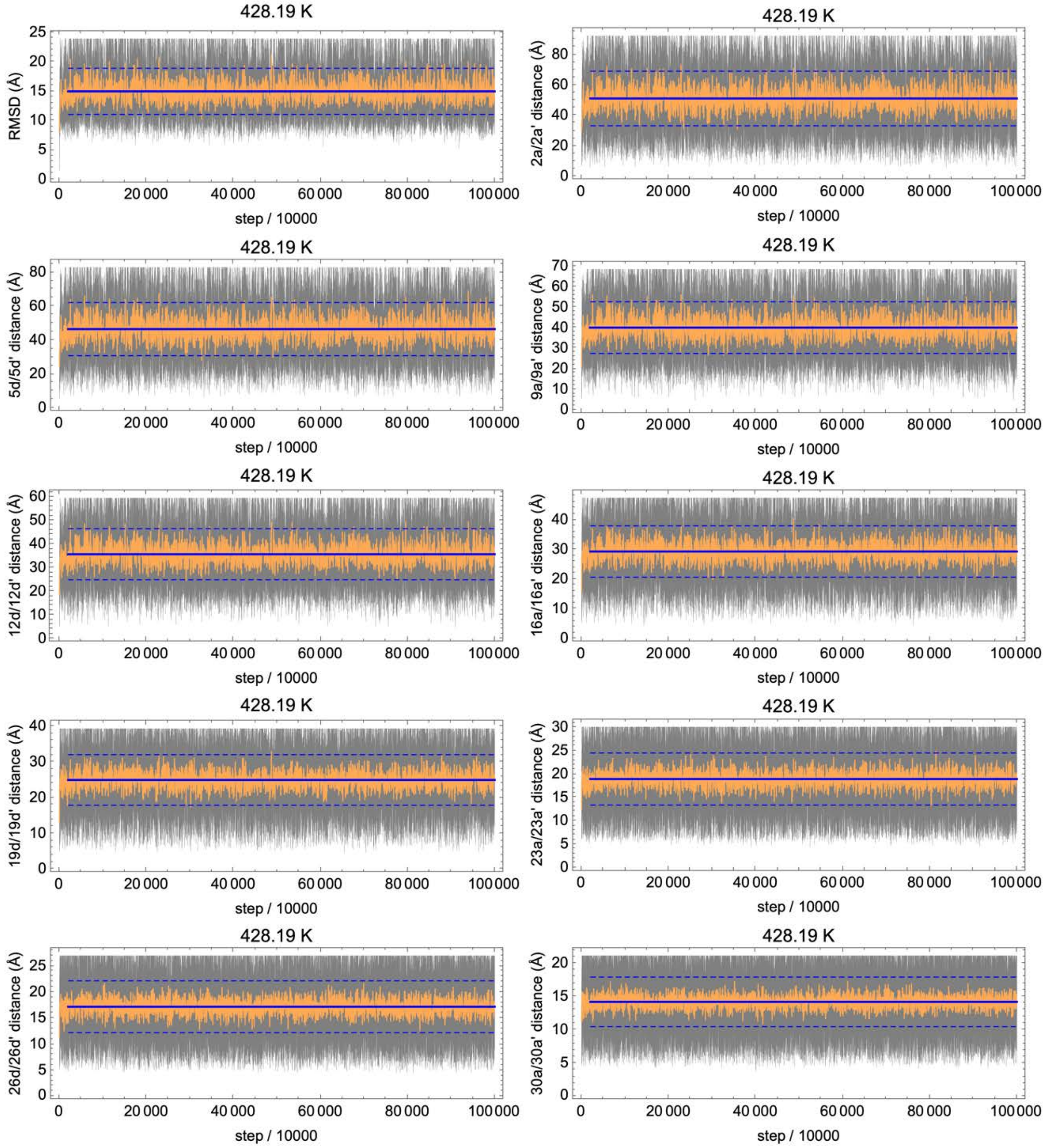

Measure at 428.19 K	Average (Å)	Standard Deviation (Å)	Slope (Å / μs)
RMSD	14.9617	3.93239	−0.0458476
5d/5d' distance	46.4527	15.677	−0.161984
12d/12d' distance	35.6599	10.7925	−0.105112
19d/19d' distance	24.9493	7.0766	−0.0268801
26d/26d' distance	17.1697	4.97649	−0.0549842
2a/2a' distance	51.1052	17.9199	−0.269858
9a/9a' distance	40.035	12.601	−0.127669
16a/16a' distance	29.3047	8.66683	−0.0387657
23a/23a' distance	18.9174	5.57086	−0.00551181
30a/30a' distance	14.1839	3.72348	0.00891272
Core Distance	30.8642	16.4746	

Figure S255. Plots of RMSD and residue packing distances for T-REMD simulations of variant **s27e/22g'-x4x** at 428.19 K.

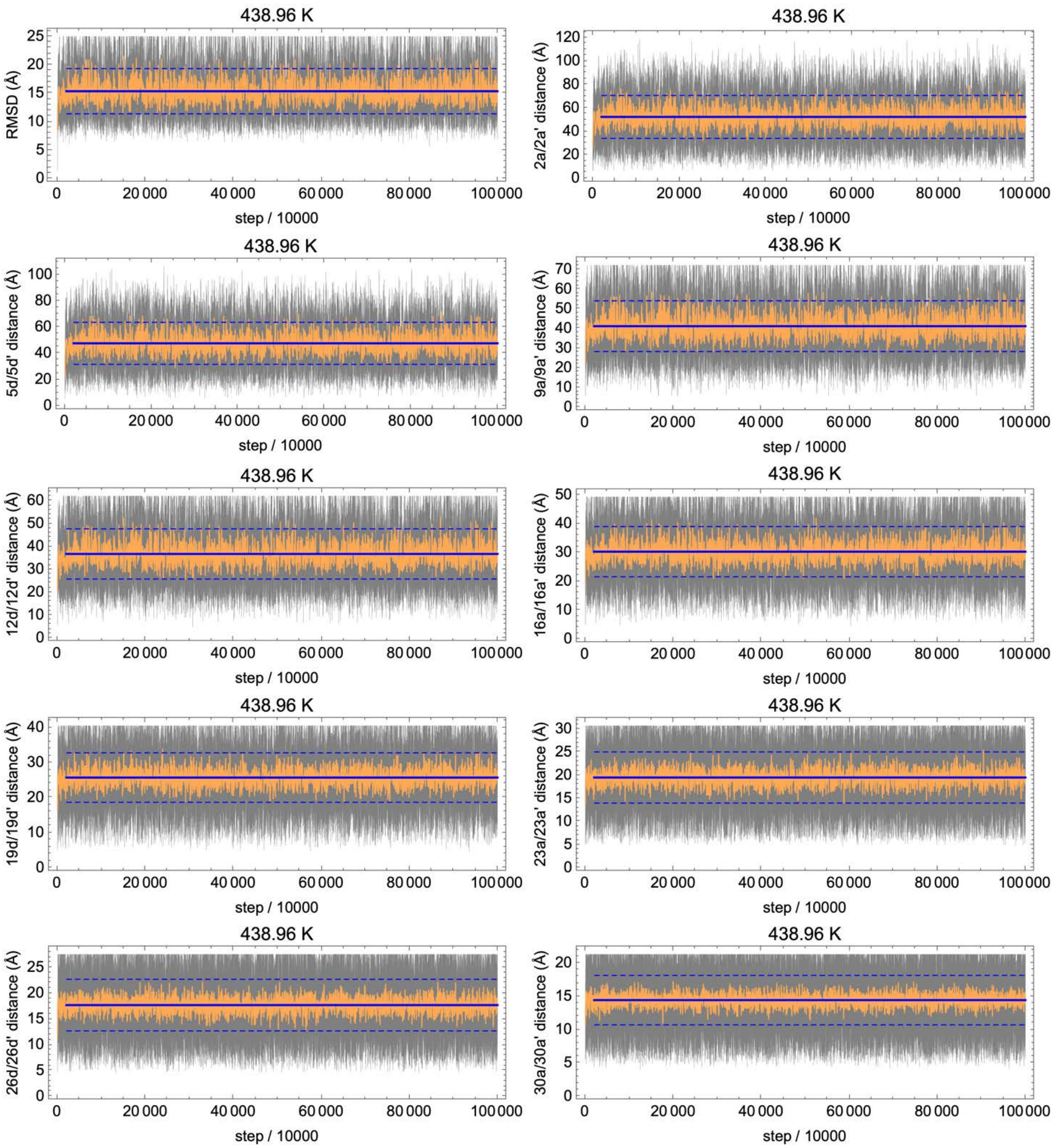

Measure at 438.96 K	Average (Å)	Standard Deviation (Å)	Slope (Å / μs)
RMSD	15.3542	3.98193	−0.0381009
5d/5d' distance	47.5738	16.0241	−0.131953
12d/12d' distance	36.7268	10.9622	−0.04797
19d/19d' distance	25.7112	7.05443	0.0230946
26d/26d' distance	17.6804	5.00077	−0.0125218
2a/2a' distance	52.2647	18.3181	−0.190602
9a/9a' distance	41.1552	12.8853	−0.0670905
16a/16a' distance	30.2675	8.71827	0.00315909
23a/23a' distance	19.5076	5.53396	0.0703896
30a/30a' distance	14.4229	3.70844	0.0126108
Core Distance	31.7011	16.8155	

Figure S256. Plots of RMSD and residue packing distances for T-REMD simulations of variant **s27e/22g'-x4x** at 438.96 K.

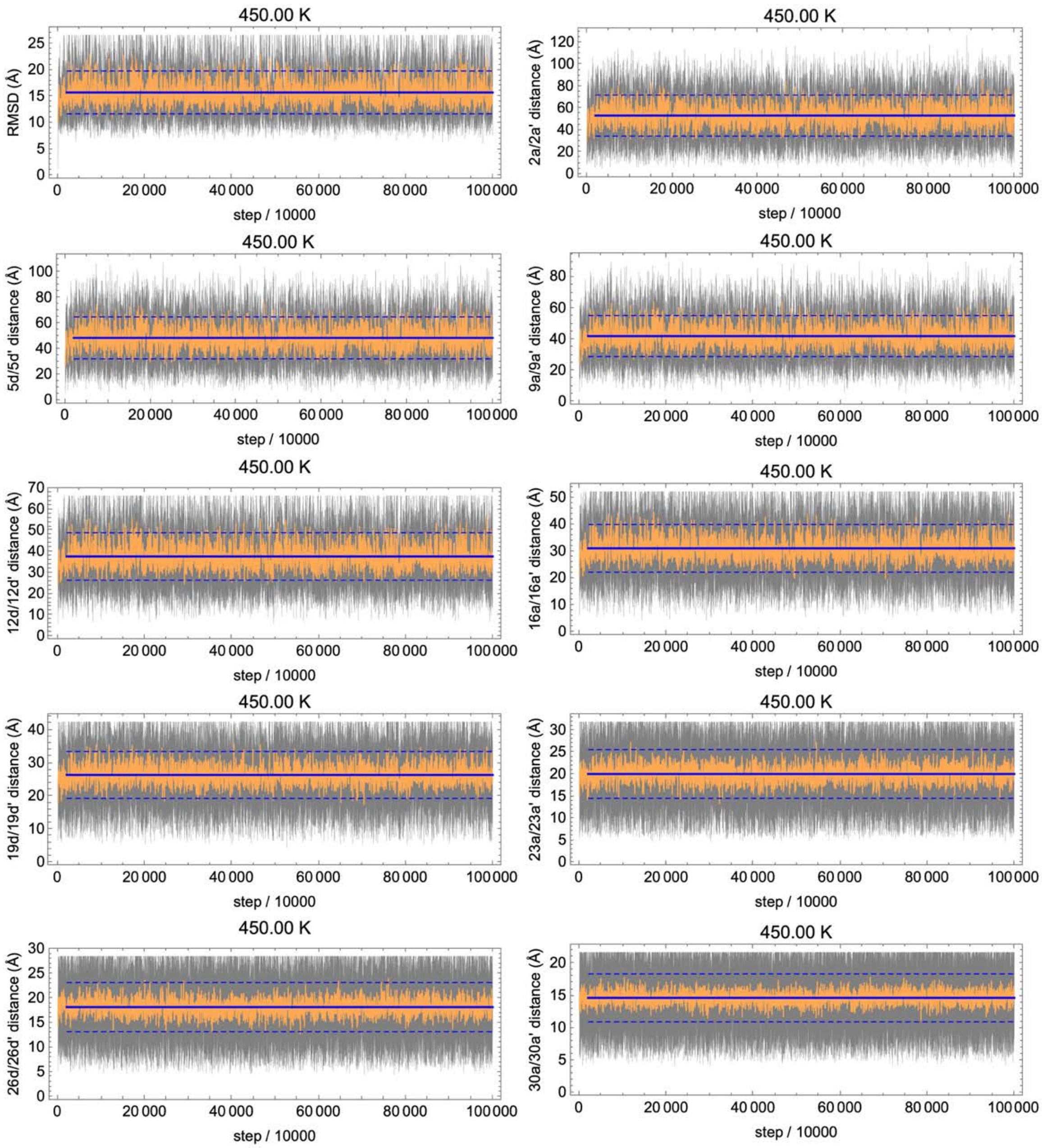

Measure at 450.00 K	Average (Å)	Standard Deviation (Å)	Slope (Å / μs)
RMSD	15.7383	4.06458	−0.0755991
5d/5d' distance	48.5951	16.3921	−0.2322
12d/12d' distance	37.6782	11.238	−0.237048
19d/19d' distance	26.386	7.10469	−0.0977611
26d/26d' distance	18.1731	4.994	−0.0261339
2a/2a' distance	53.2868	18.7079	−0.170444
9a/9a' distance	42.1623	13.2213	−0.238729
16a/16a' distance	31.0918	8.87416	−0.178679
23a/23a' distance	20.0544	5.54578	−0.0351941
30a/30a' distance	14.6415	3.69987	0.0500794
Core Distance	32.4521	17.1533	

Figure S257. Plots of RMSD and residue packing distances for T-REMD simulations of variant **s27e/22g'-x4x** at 450.00 K.

(return to Table of Contents)

27e/22g'-z4x

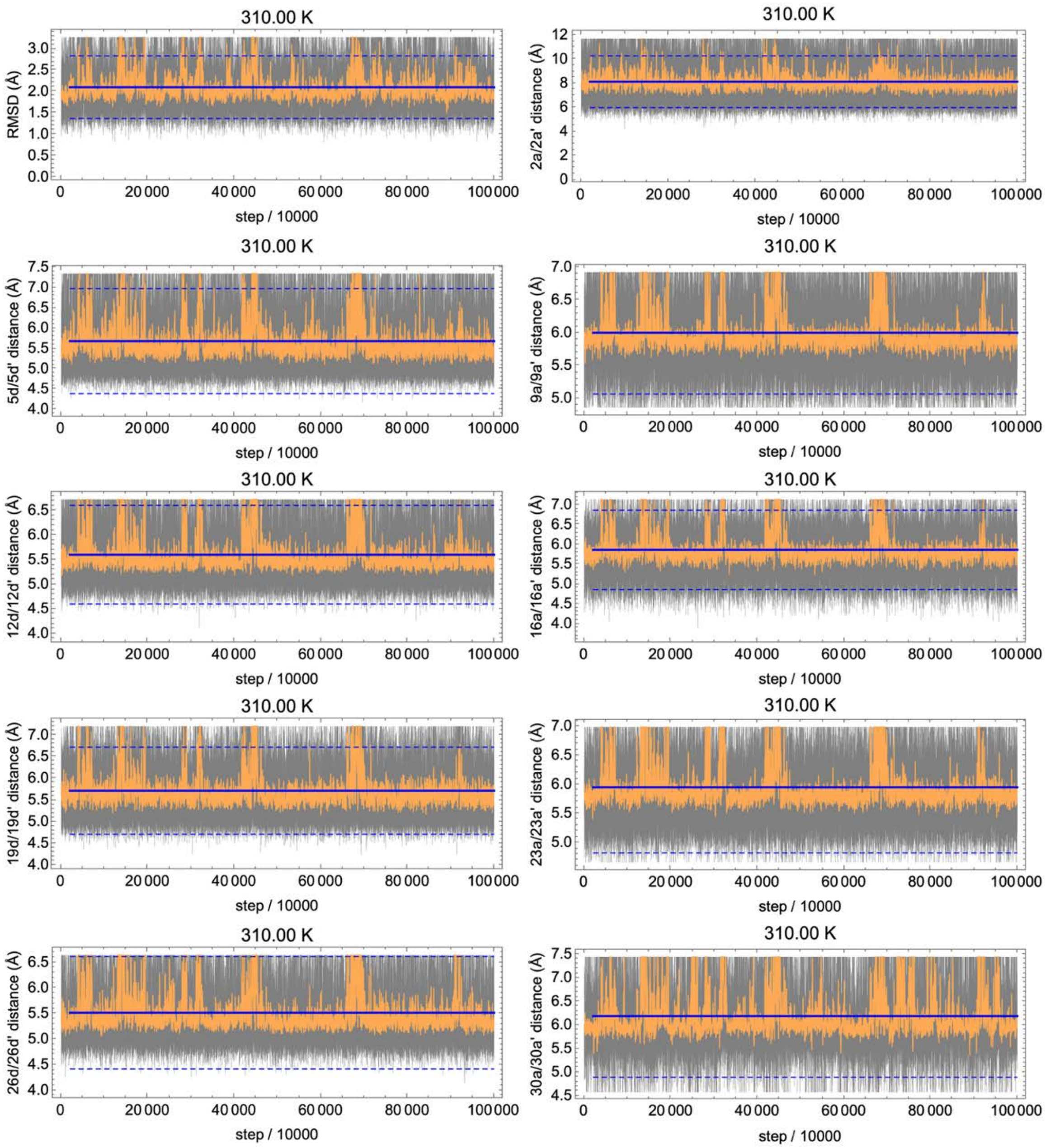

Measure at 310.00 K	Average (Å)	Standard Deviation (Å)	Slope (Å / μs)
RMSD	2.09874	0.732073	−0.0466265
5d/5d' distance	5.68111	1.29409	−0.126241
12d/12d' distance	5.60064	1.00289	−0.0828477
19d/19d' distance	5.71392	1.00772	−0.0805241
26d/26d' distance	5.51586	1.09737	−0.11679
2a/2a' distance	8.13229	2.14487	−0.167336
9a/9a' distance	6.00077	0.935975	−0.0841009
16a/16a' distance	5.86559	1.00236	−0.0769804
23a/23a' distance	5.94974	1.12956	−0.111975
30a/30a' distance	6.1878	1.29501	−0.0206733
Core Distance	6.07197	1.47074	

Figure S258. Plots of RMSD and residue packing distances for T-REMD simulations of variant **27e/22g'-z4x** at 310.00 K.

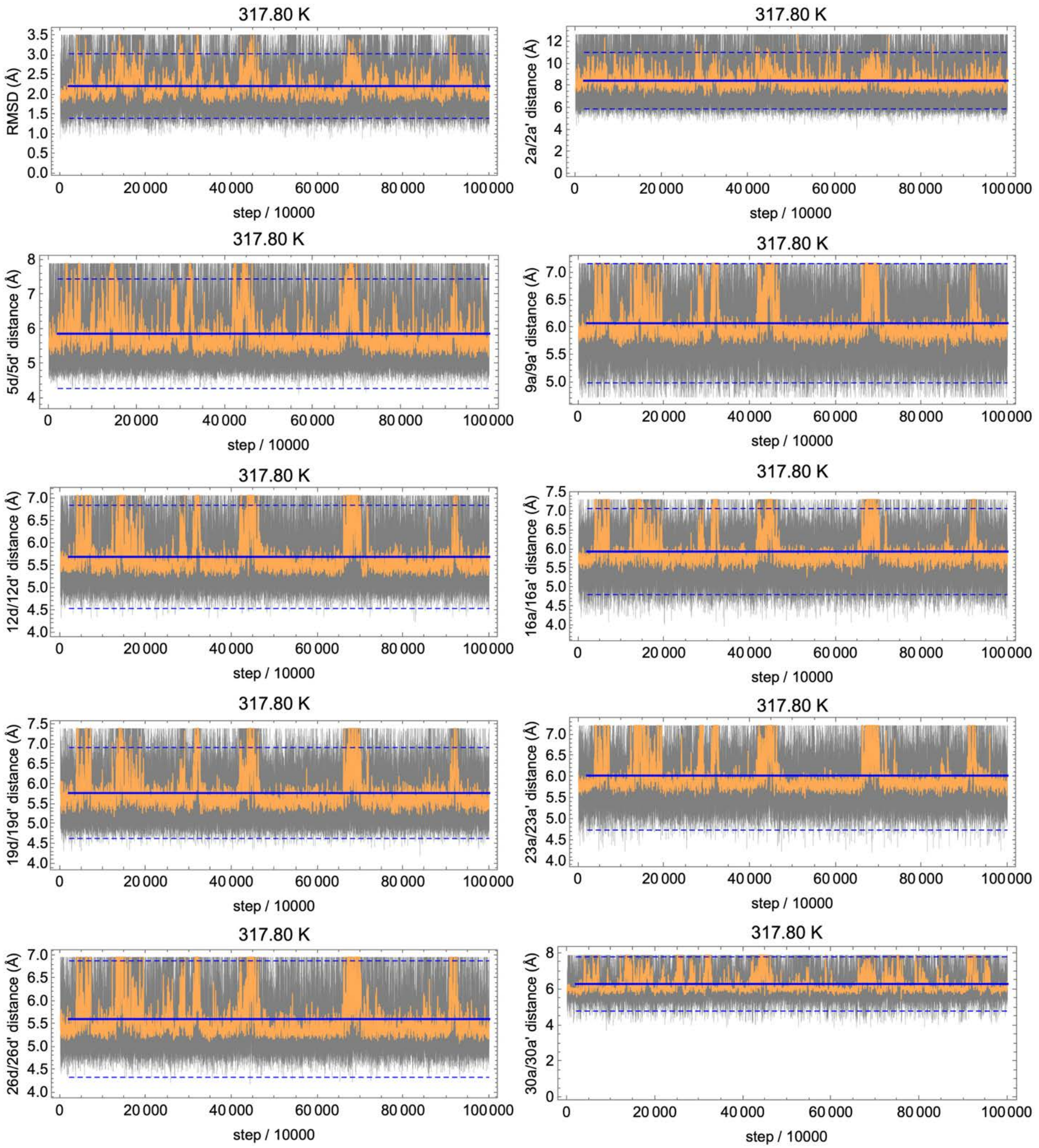

Measure at 317.80 K	Average (Å)	Standard Deviation (Å)	Slope (Å / μs)
RMSD	2.21493	0.818675	−0.0625658
5d/5d' distance	5.86913	1.58436	−0.163662
12d/12d' distance	5.6958	1.154	−0.109726
19d/19d' distance	5.77728	1.14146	−0.102577
26d/26d' distance	5.60883	1.27431	−0.124943
2a/2a' distance	8.47001	2.56468	−0.202358
9a/9a' distance	6.07855	1.08927	−0.1098
16a/16a' distance	5.93826	1.13321	−0.0995822
23a/23a' distance	6.03135	1.29259	−0.133392
30a/30a' distance	6.30261	1.50598	−0.0360544
Core Distance	6.19687	1.69714	

Figure S259. Plots of RMSD and residue packing distances for T-REMD simulations of variant **27e/22g’-z4x** at 317.80 K.

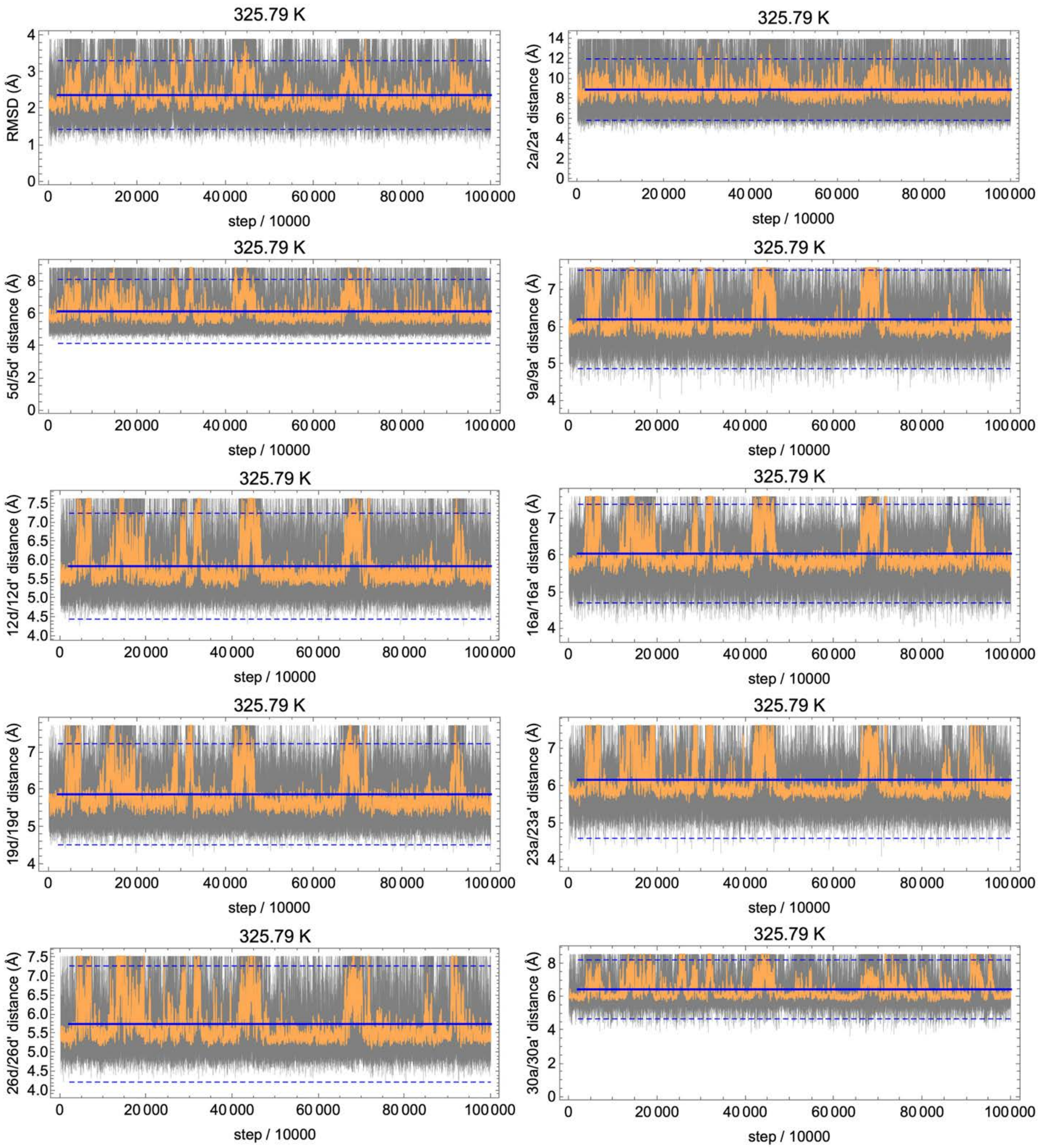

Measure at 325.79 K	Average (Å)	Standard Deviation (Å)	Slope (Å / μs)
RMSD	2.37203	0.938143	−0.0920508
5d/5d' distance	6.15244	1.98426	−0.216323
12d/12d' distance	5.85024	1.39868	−0.153276
19d/19d' distance	5.87985	1.35484	−0.149277
26d/26d' distance	5.75276	1.52824	−0.176254
2a/2a' distance	8.92567	3.06031	−0.251479
9a/9a' distance	6.20321	1.33362	−0.156067
16a/16a' distance	6.05315	1.34962	−0.154168
23a/23a' distance	6.16374	1.57329	−0.191145
30a/30a' distance	6.46796	1.76983	−0.0757072
Core Distance	6.38322	2.00794	

Figure S260. Plots of RMSD and residue packing distances for T-REMD simulations of variant **27e/22g’-z4x** at 325.79 K.

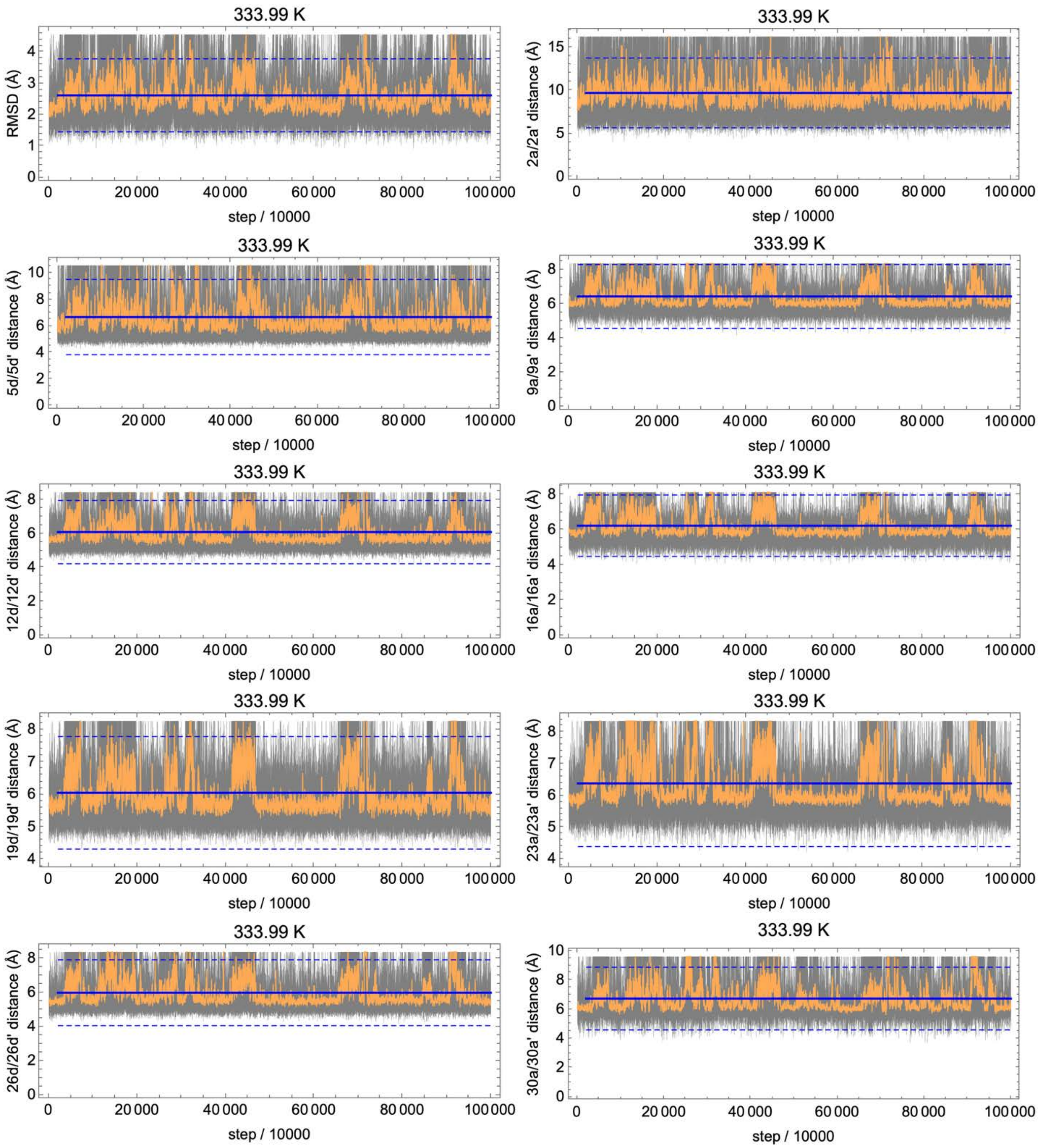

Measure at 333.99 K	Average (Å)	Standard Deviation (Å)	Slope (Å / μs)
RMSD	2.61757	1.16236	−0.111107
5d/5d' distance	6.67355	2.83496	−0.271626
12d/12d' distance	6.08675	1.8685	−0.185388
19d/19d' distance	6.0496	1.73342	−0.159811
26d/26d' distance	5.98913	1.92744	−0.196988
2a/2a' distance	9.71285	4.04187	−0.314512
9a/9a' distance	6.43029	1.86959	−0.201063
16a/16a' distance	6.23203	1.74825	−0.169935
23a/23a' distance	6.38051	1.99607	−0.216622
30a/30a' distance	6.73143	2.14743	−0.0781187
Core Distance	6.69846	2.59292	

Figure S261. Plots of RMSD and residue packing distances for T-REMD simulations of variant **27e/22g'-z4x** at 333.99 K.

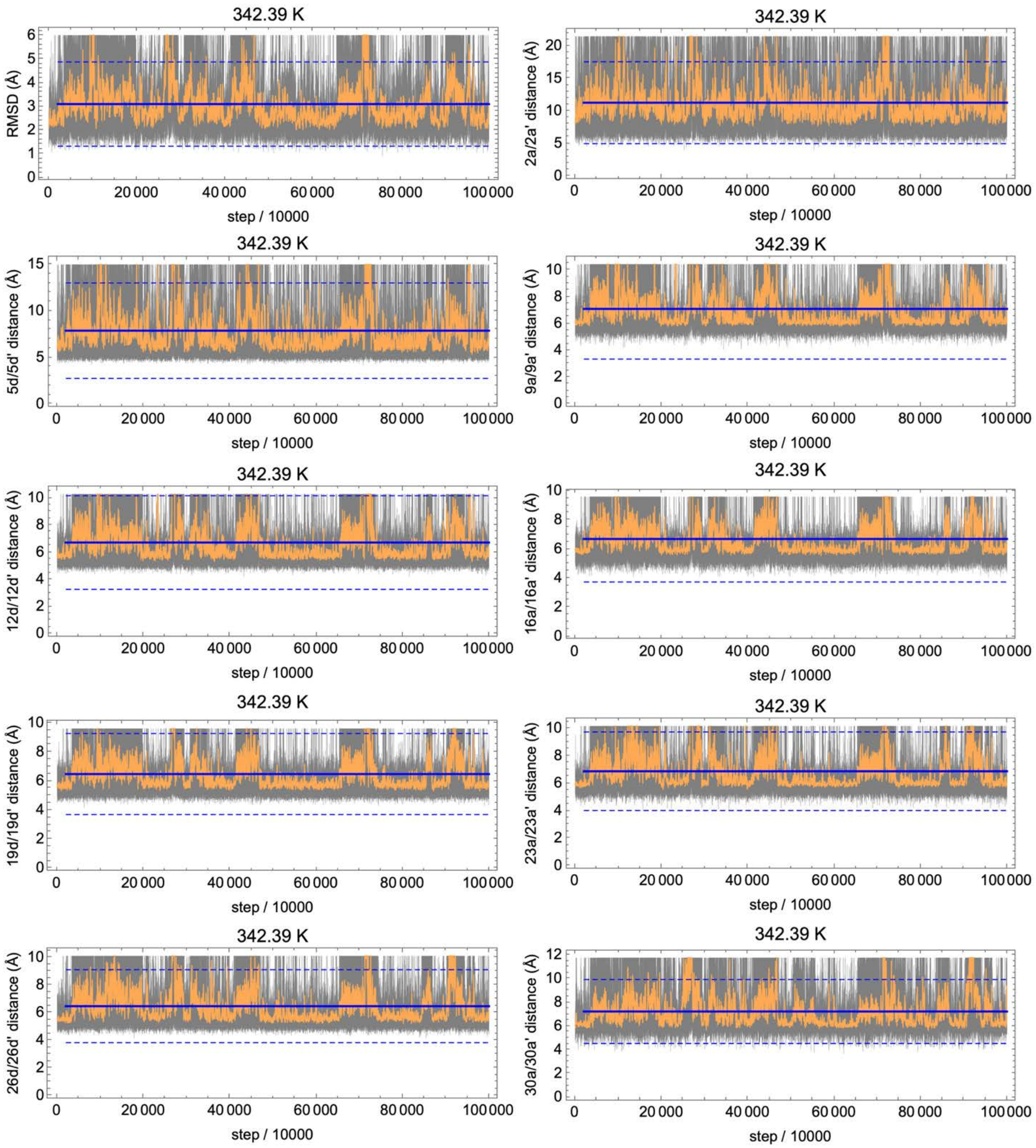

Measure at 342.39 K	Average (Å)	Standard Deviation (Å)	Slope (Å / μs)
RMSD	3.09798	1.77815	−0.22595
5d/5d' distance	7.86686	5.11511	−0.545856
12d/12d' distance	6.70922	3.44966	−0.321765
19d/19d' distance	6.47449	2.78937	−0.24498
26d/26d' distance	6.46186	2.6394	−0.321654
2a/2a' distance	11.2582	6.29028	−0.683395
9a/9a' distance	7.088	3.75017	−0.372992
16a/16a' distance	6.68986	2.95613	−0.269103
23a/23a' distance	6.86863	2.86131	−0.355881
30a/30a' distance	7.22502	2.69341	−0.216656
Core Distance	7.40469	4.06686	

Figure S262. Plots of RMSD and residue packing distances for T-REMD simulations of variant **27e/22g'-z4x** at 342.39 K.

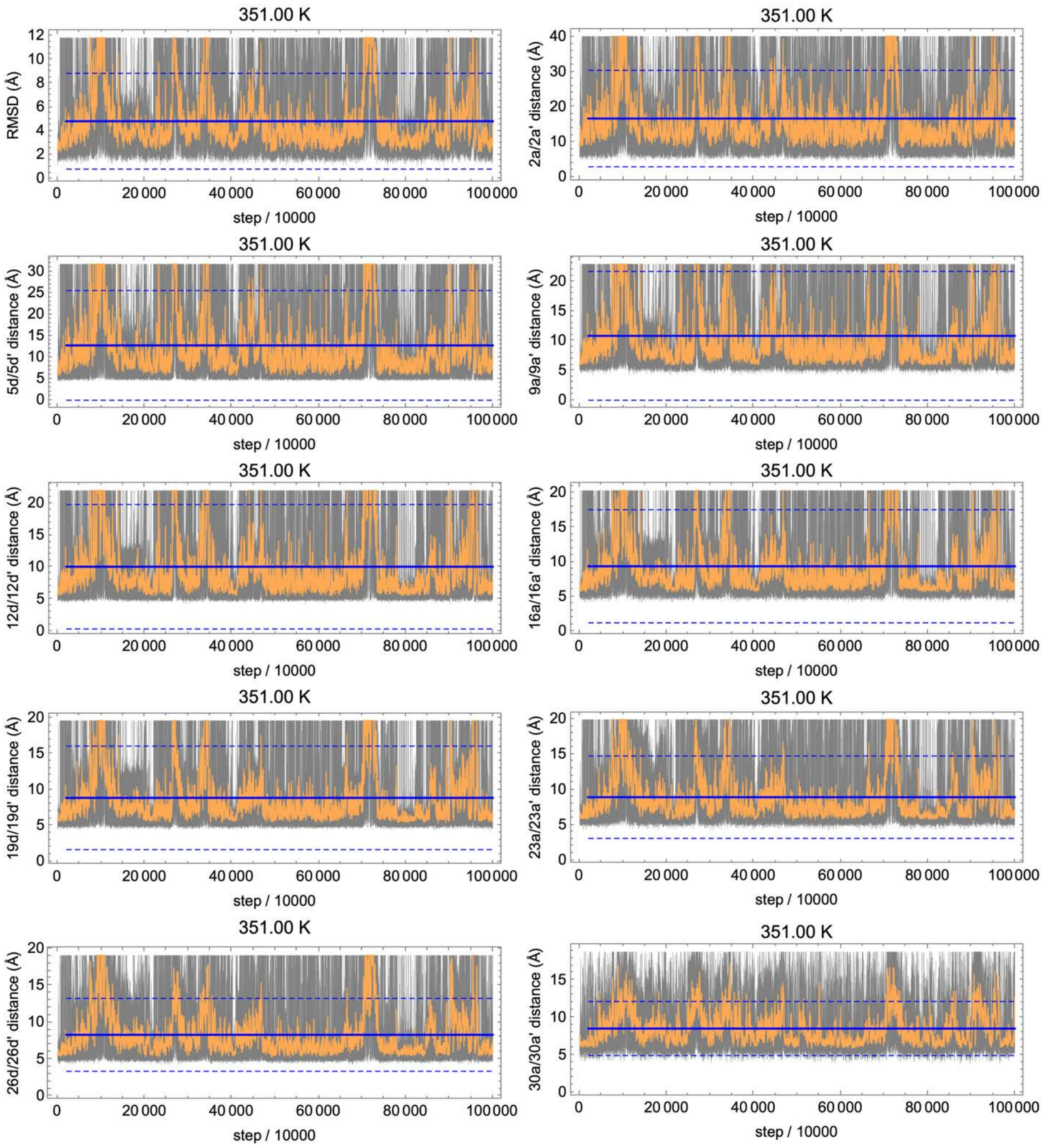

Measure at 351.00 K	Average (Å)	Standard Deviation (Å)	Slope (Å / μs)
RMSD	4.80262	4.02683	−0.582171
5d/5d' distance	12.7363	12.7973	−1.5235
12d/12d' distance	10.0536	9.71588	−0.994237
19d/19d' distance	8.84288	7.22043	−0.699281
26d/26d' distance	8.29705	4.94063	−0.717667
2a/2a' distance	16.6589	13.7538	−1.79502
9a/9a' distance	10.8332	10.8432	−1.10362
16a/16a' distance	9.36265	8.16685	−0.778744
23a/23a' distance	8.92348	5.83499	−0.821277
30a/30a' distance	8.53132	3.61835	−0.511647
Core Distance	10.4711	9.50893	

Figure S263. Plots of RMSD and residue packing distances for T-REMD simulations of variant **27e/22g’-z4x** at 351.00 K.

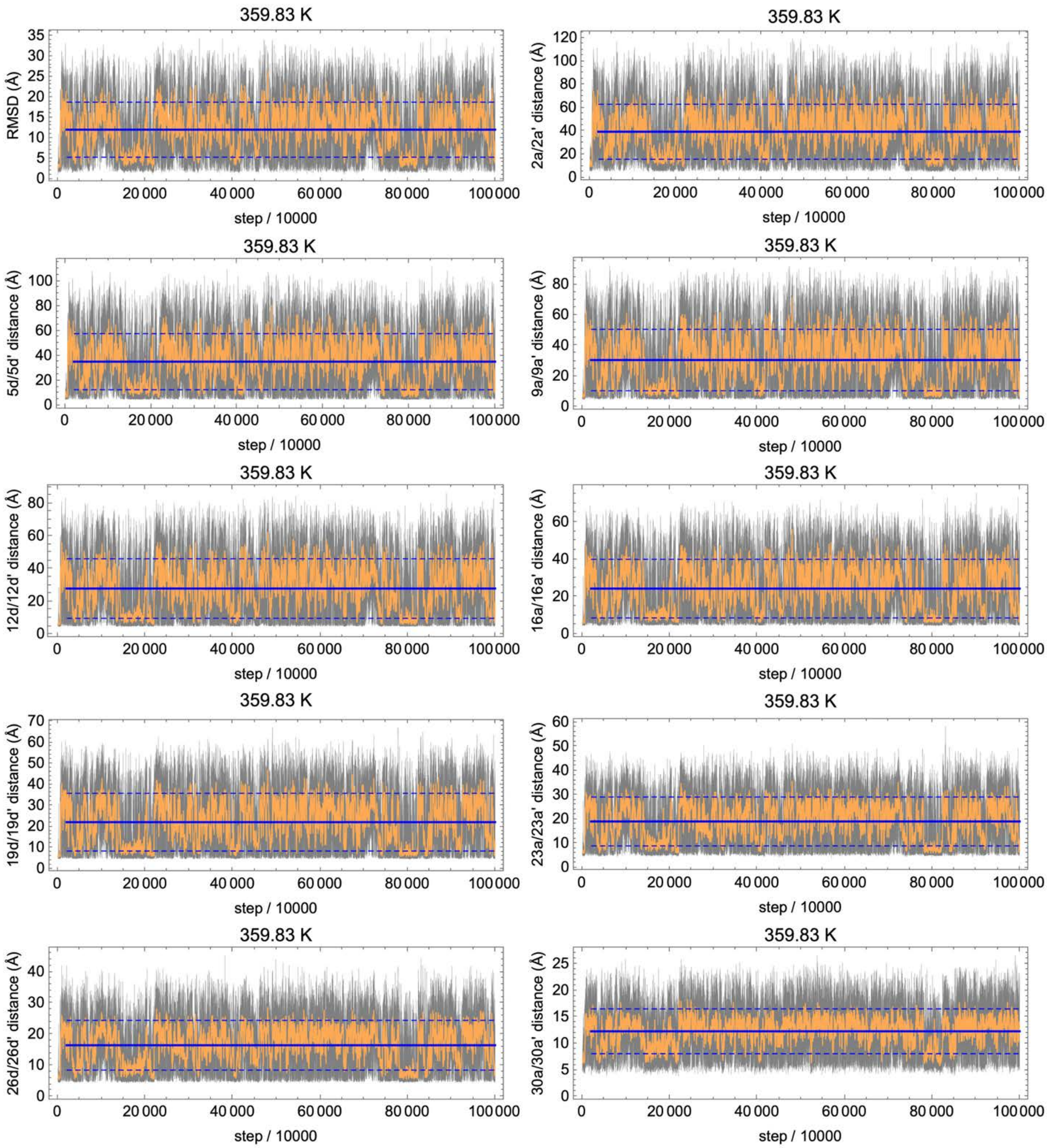

Measure at 359.83 K	Average (Å)	Standard Deviation (Å)	Slope (Å / μs)
RMSD	12.0097	6.7399	0.720891
5d/5d' distance	35.1128	22.5966	2.30591
12d/12d' distance	27.9085	18.2154	1.97467
19d/19d' distance	22.07	13.7134	1.64244
26d/26d' distance	16.4702	7.9821	0.846176
2a/2a' distance	39.3784	23.6306	2.31882
9a/9a' distance	30.5395	20.0842	2.23767
16a/16a' distance	24.3709	15.6977	1.67588
23a/23a' distance	19.0459	10.1096	1.04116
30a/30a' distance	12.3059	4.22366	0.429874
Core Distance	25.2447	18.4039	

Figure S264. Plots of RMSD and residue packing distances for T-REMD simulations of variant **27e/22g'-z4x** at 359.83 K.

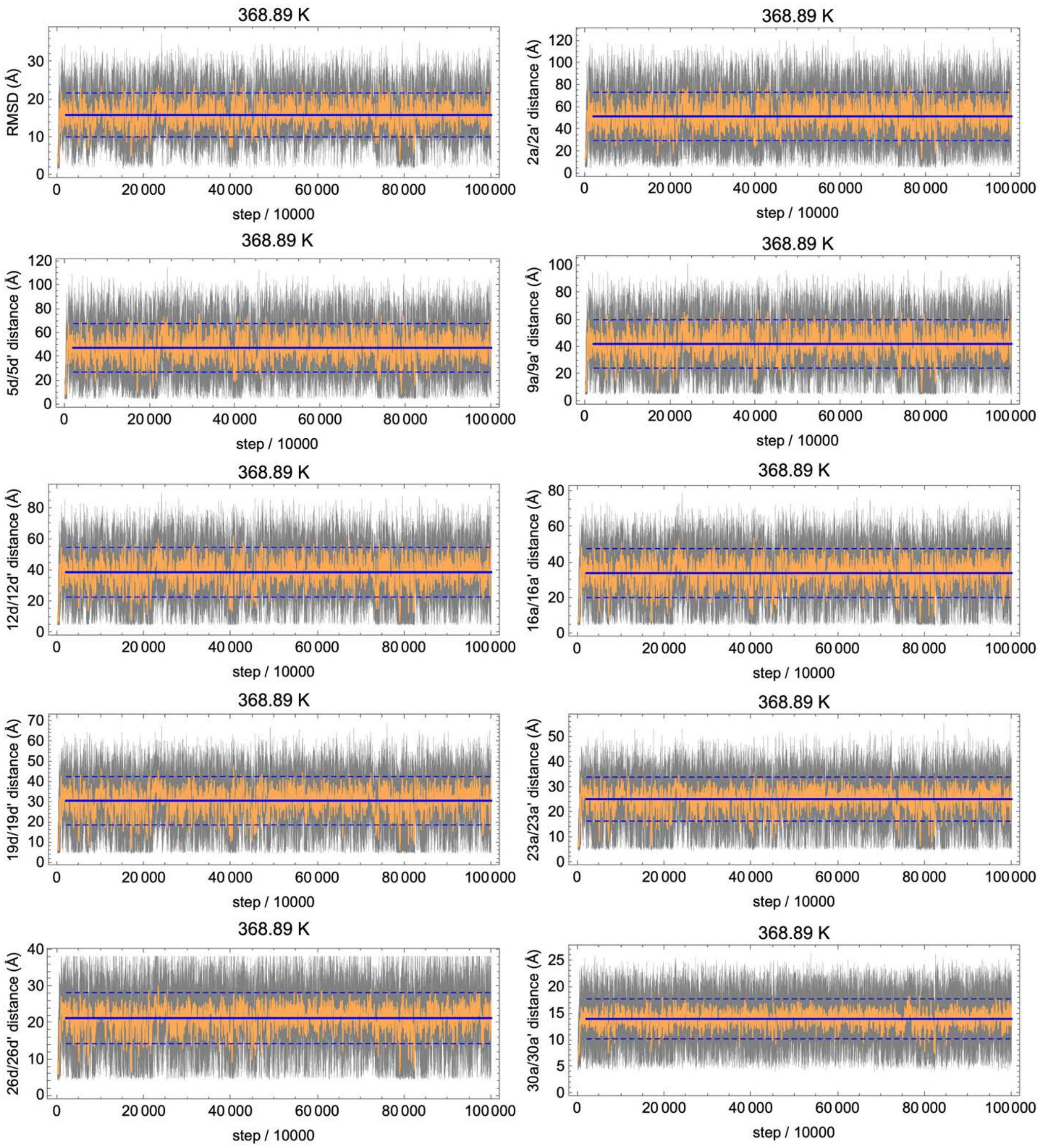

Measure at 368.89 K	Average (Å)	Standard Deviation (Å)	Slope (Å / μs)
RMSD	15.9524	5.84368	0.271294
5d/5d' distance	47.5847	20.3544	1.07466
12d/12d' distance	38.8082	15.9858	0.878956
19d/19d' distance	30.6276	11.9518	0.652852
26d/26d' distance	21.3331	6.98214	0.45587
2a/2a' distance	51.7157	21.8621	1.03509
9a/9a' distance	42.2104	17.7618	0.946601
16a/16a' distance	34.0172	13.7448	0.700522
23a/23a' distance	25.2365	8.791	0.521113
30a/30a' distance	14.0037	3.76236	0.11084
Core Distance	33.9486	18.7752	

Figure S265. Plots of RMSD and residue packing distances for T-REMD simulations of variant **27e/22g'-z4x** at 368.89 K.

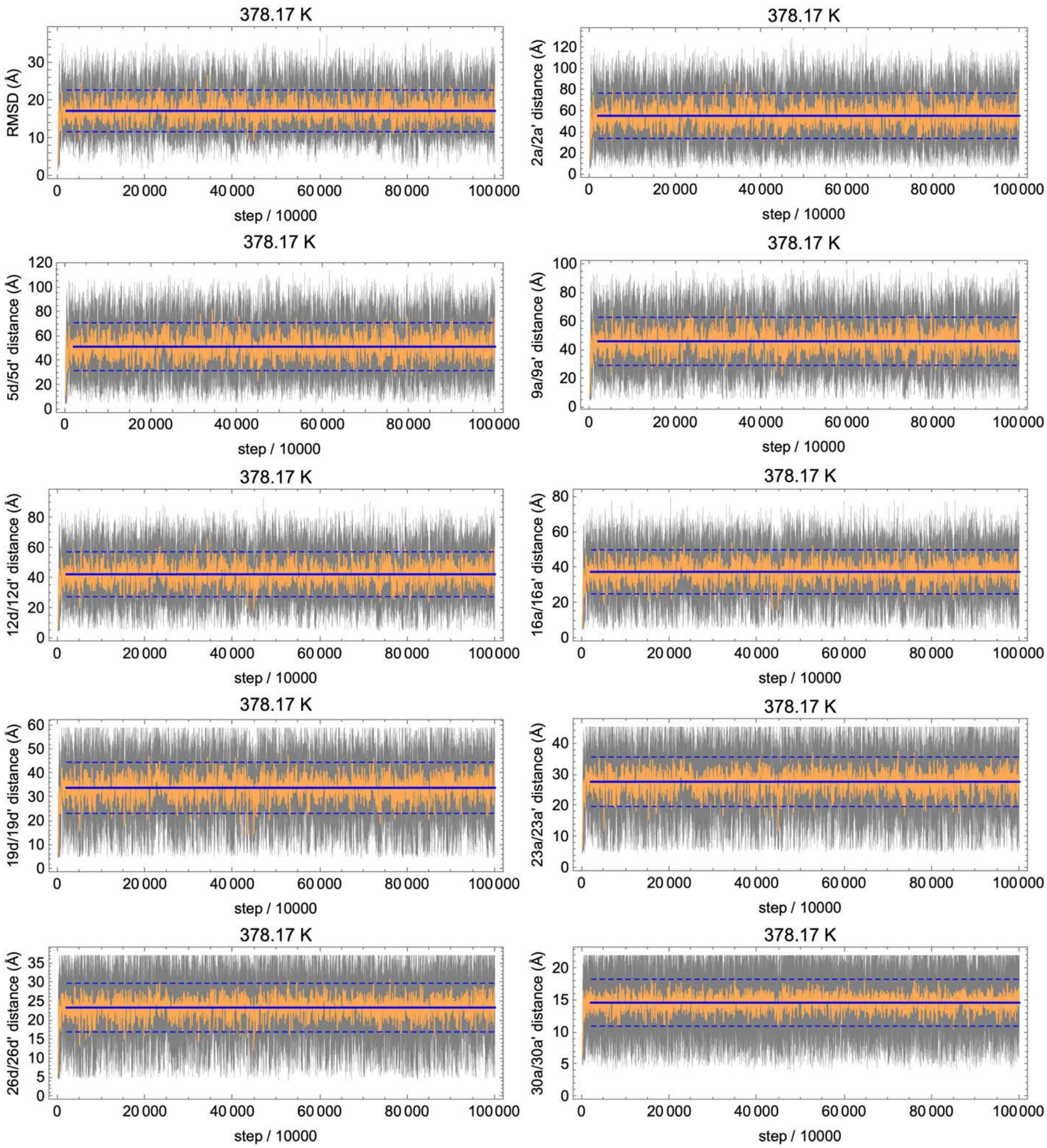

Measure at 378.17 K	Average (Å)	Standard Deviation (Å)	Slope (Å / μs)
RMSD	17.2808	5.53138	0.111926
5d/5d' distance	51.5185	19.6341	0.48272
12d/12d' distance	42.646	14.8899	0.411076
19d/19d' distance	33.9635	10.7006	0.322609
26d/26d' distance	23.4208	6.40952	0.207135
2a/2a' distance	55.5385	21.4259	0.487904
9a/9a' distance	46.1388	16.7961	0.387005
16a/16a' distance	37.6459	12.4543	0.372608
23a/23a' distance	27.7564	7.96187	0.29487
30a/30a' distance	14.6595	3.66114	0.0477296
Core Distance	37.032	18.8098	

Figure S266. Plots of RMSD and residue packing distances for T-REMD simulations of variant **27e/22g'-z4x** at 378.17 K.

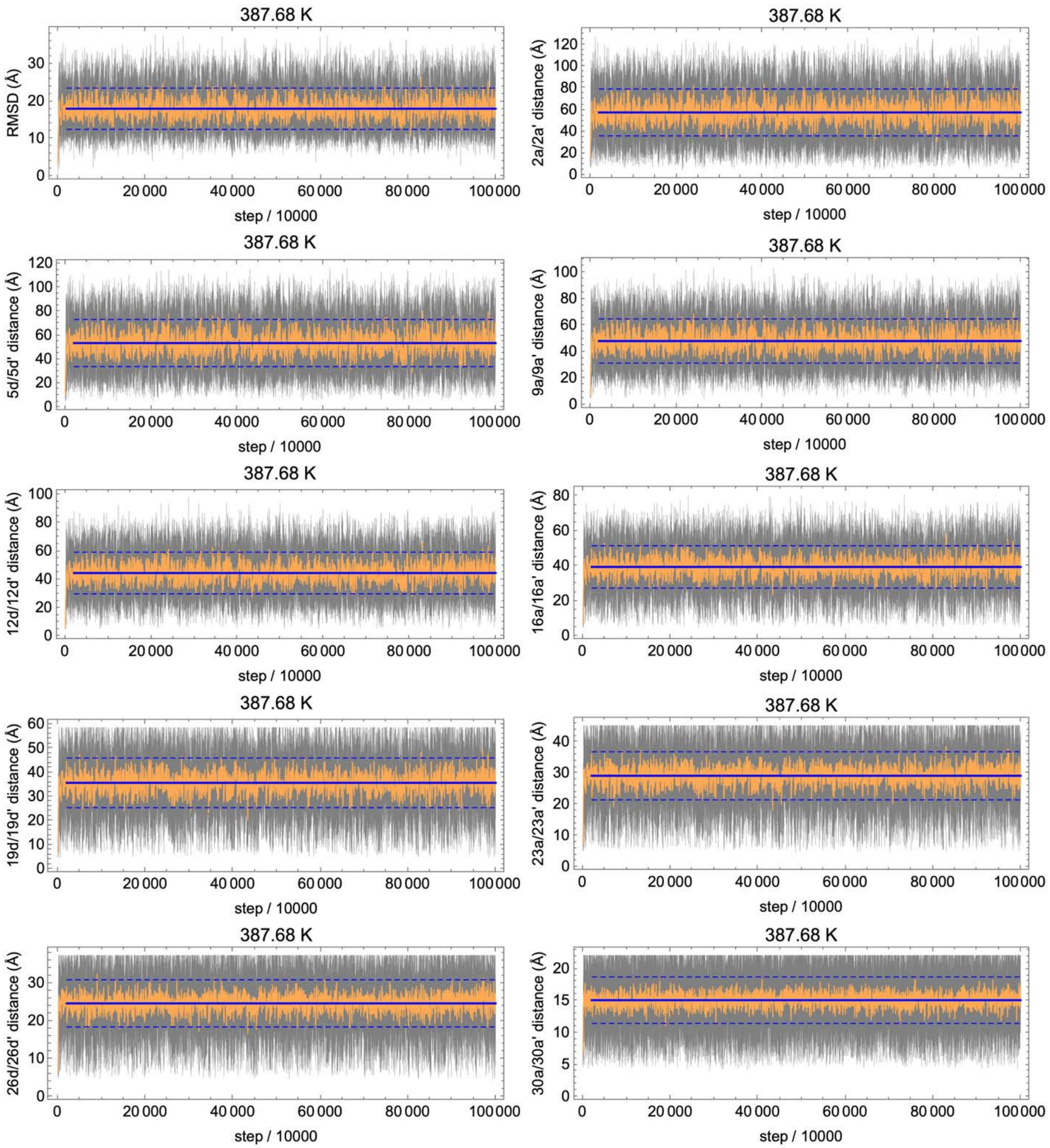

Measure at 387.68 K	Average (Å)	Standard Deviation (Å)	Slope (Å / μs)
RMSD	17.9942	5.49756	0.107159
5d/5d' distance	53.5102	19.7087	0.431593
12d/12d' distance	44.5552	14.6975	0.297431
19d/19d' distance	35.6934	10.2993	0.241252
26d/26d' distance	24.6394	6.23139	0.187694
2a/2a' distance	57.527	21.6213	0.506803
9a/9a' distance	48.1029	16.7066	0.262157
16a/16a' distance	39.453	12.0877	0.264153
23a/23a' distance	29.1669	7.70837	0.223909
30a/30a' distance	15.0938	3.63525	0.0929074
Core Distance	38.638	19.0513	

Figure S267. Plots of RMSD and residue packing distances for T-REMD simulations of variant **27e/22g’-z4x** at 387.68 K.

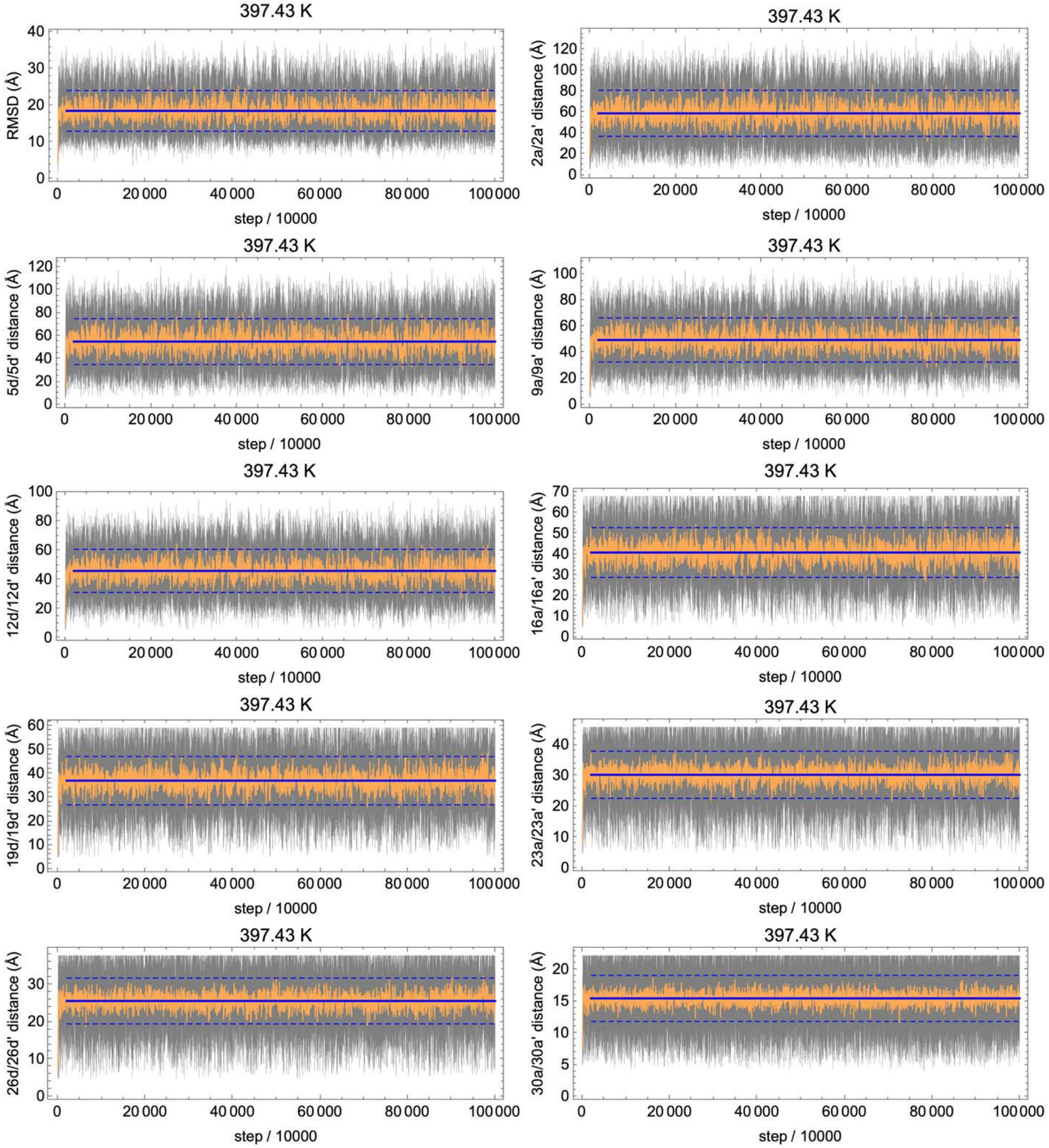

Measure at 397.43 K	Average (Å)	Standard Deviation (Å)	Slope (Å / μs)
RMSD	18.53	5.5448	0.0199655
5d/5d' distance	54.9356	19.9725	0.0259835
12d/12d' distance	45.9548	14.7907	−0.017305
19d/19d' distance	37.0188	10.1556	0.0169719
26d/26d' distance	25.5881	6.12922	0.0649809
2a/2a' distance	58.9339	22.0094	0.0563668
9a/9a' distance	49.5099	16.8811	−0.0757537
16a/16a' distance	40.8305	12.0027	0.0498942
23a/23a' distance	30.2888	7.63987	0.0896876
30a/30a' distance	15.4761	3.62929	0.0190267
Core Distance	39.8374	19.3321	

Figure S268. Plots of RMSD and residue packing distances for T-REMD simulations of variant **27e/22g'-z4x** at 397.43 K.

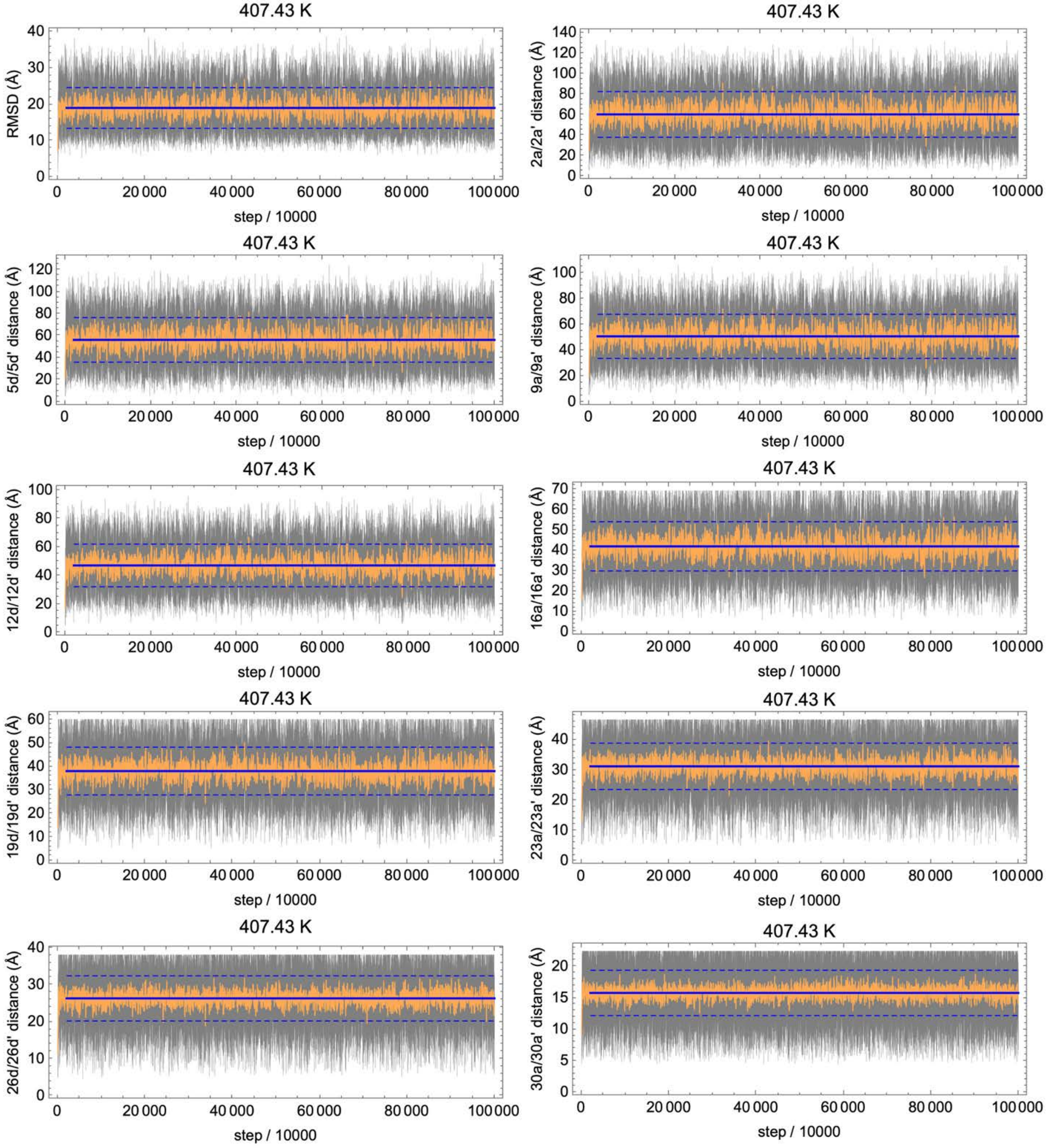

Measure at 407.43 K	Average (Å)	Standard Deviation (Å)	Slope (Å / μs)
RMSD	19.0034	5.5891	−0.0787817
5d/5d' distance	56.1889	20.2534	−0.2665
12d/12d' distance	47.1239	14.9668	−0.287637
19d/19d' distance	38.0423	10.1675	−0.138768
26d/26d' distance	26.3381	6.09555	0.0570454
2a/2a' distance	60.2253	22.3023	−0.294039
9a/9a' distance	50.7294	17.1048	−0.322526
16a/16a' distance	41.9188	12.08	−0.191026
23a/23a' distance	31.1914	7.66087	−0.0337802
30a/30a' distance	15.7885	3.60284	0.0336345
Core Distance	40.8385	19.6382	

Figure S269. Plots of RMSD and residue packing distances for T-REMD simulations of variant **27e/22g’-z4x** at 407.43 K.

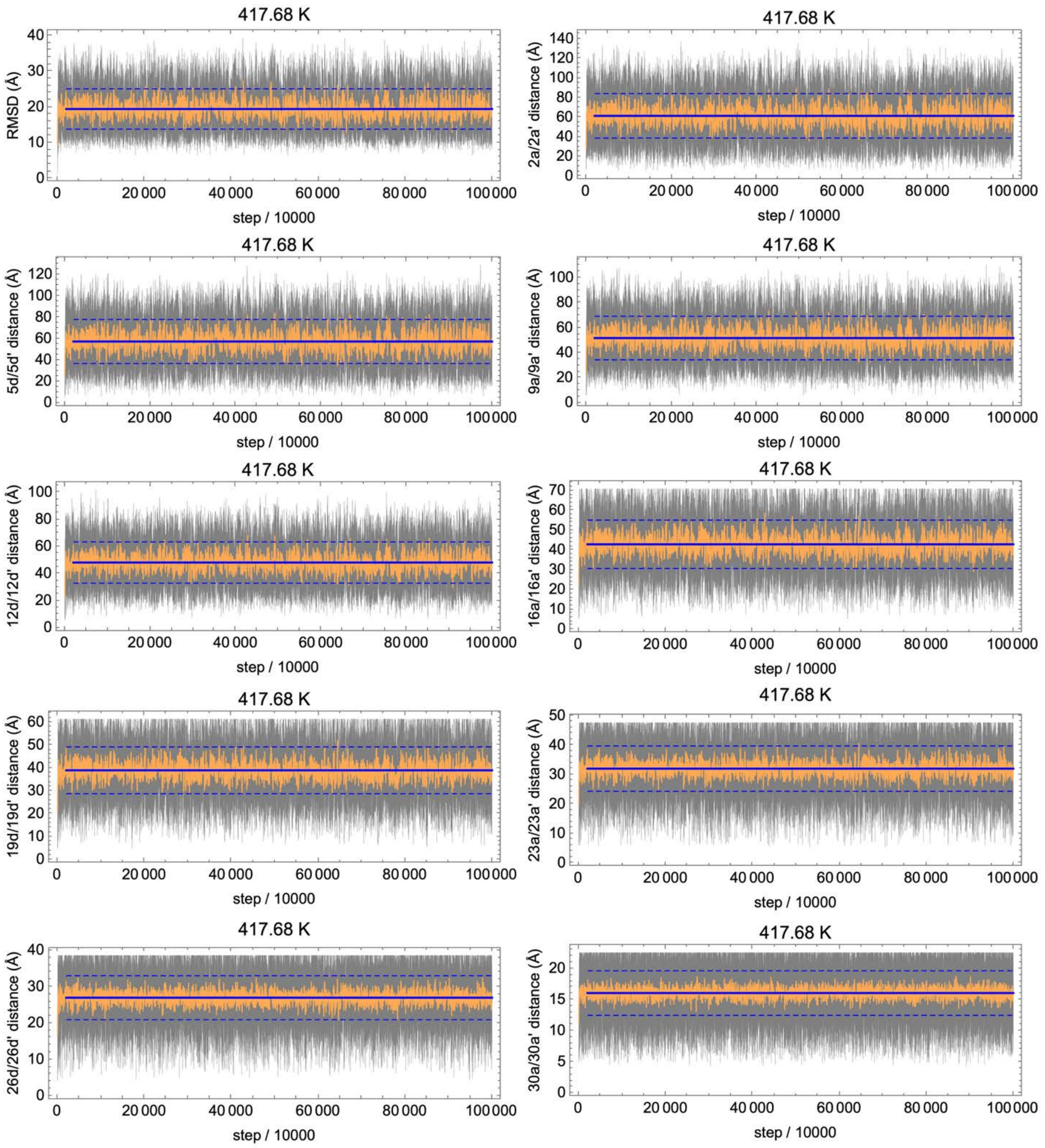

Measure at 417.68 K	Average (Å)	Standard Deviation (Å)	Slope (Å / μs)
RMSD	19.4229	5.64762	−0.102503
5d/5d' distance	57.2929	20.5903	−0.367756
12d/12d' distance	48.138	15.21	−0.370097
19d/19d' distance	38.9643	10.2303	−0.270364
26d/26d' distance	26.9797	6.04968	−0.0324986
2a/2a' distance	61.4009	22.6813	−0.320425
9a/9a' distance	51.7497	17.4208	−0.420507
16a/16a' distance	42.88	12.2216	−0.321598
23a/23a' distance	32.0494	7.70213	−0.173757
30a/30a' distance	16.0557	3.57554	0.0102714
Core Distance	41.7234	19.961	

Figure S270. Plots of RMSD and residue packing distances for T-REMD simulations of variant **27e/22g'-z4x** at 417.68 K.

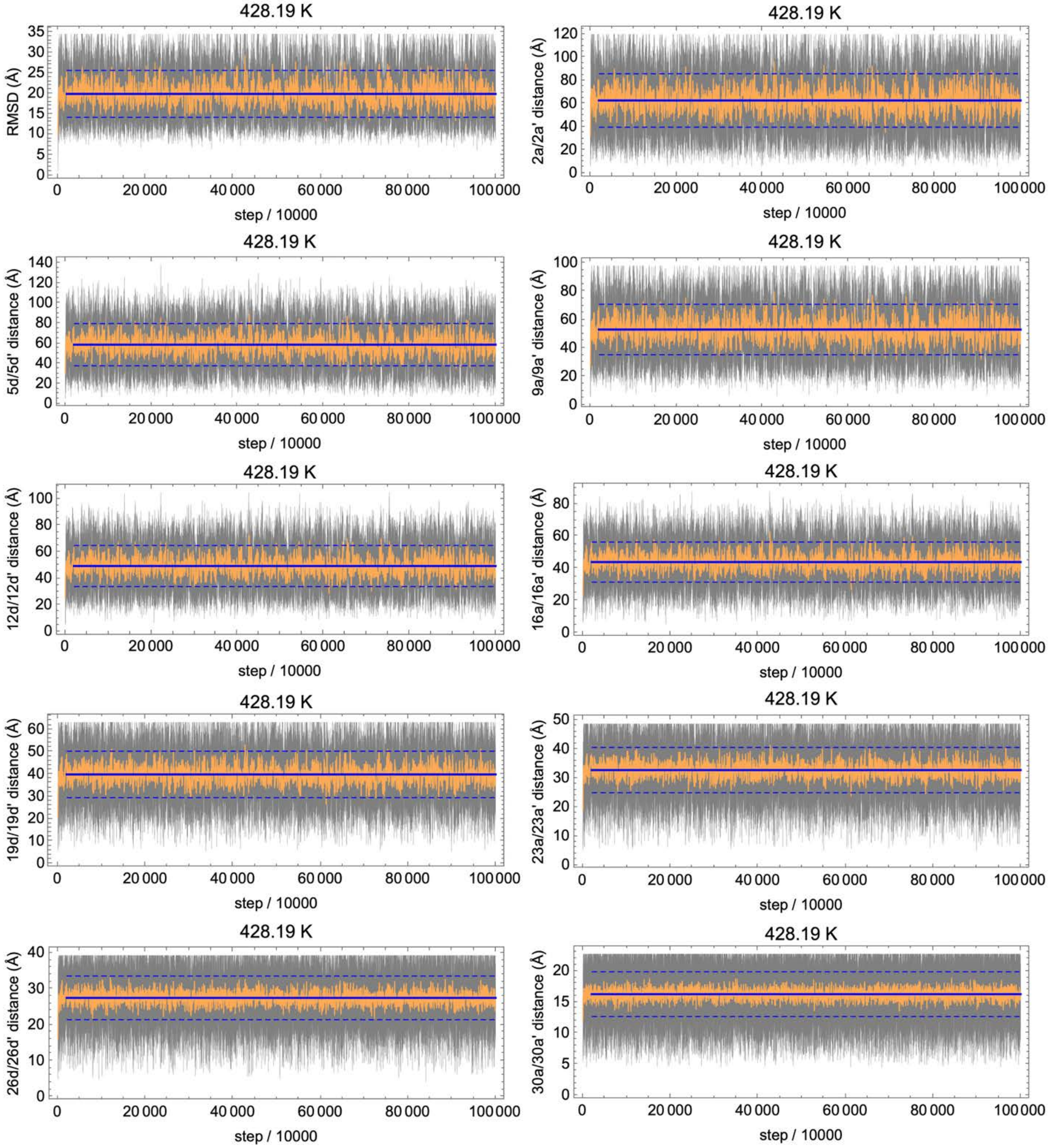

Measure at 428.19 K	Average (Å)	Standard Deviation (Å)	Slope (Å / μs)
RMSD	19.8862	5.74126	−0.128969
5d/5d' distance	58.6206	20.9584	−0.381331
12d/12d' distance	49.2406	15.4991	−0.454572
19d/19d' distance	39.8187	10.3937	−0.34507
26d/26d' distance	27.4788	6.07224	−0.140858
2a/2a' distance	62.7365	23.0956	−0.335682
9a/9a' distance	52.9876	17.7557	−0.45308
16a/16a' distance	43.8684	12.4343	−0.414522
23a/23a' distance	32.725	7.80004	−0.271073
30a/30a' distance	16.2573	3.59323	−0.000434495
Core Distance	42.6371	20.3987	

Figure S271. Plots of RMSD and residue packing distances for T-REMD simulations of variant **27e/22g'-z4x** at 428.19 K.

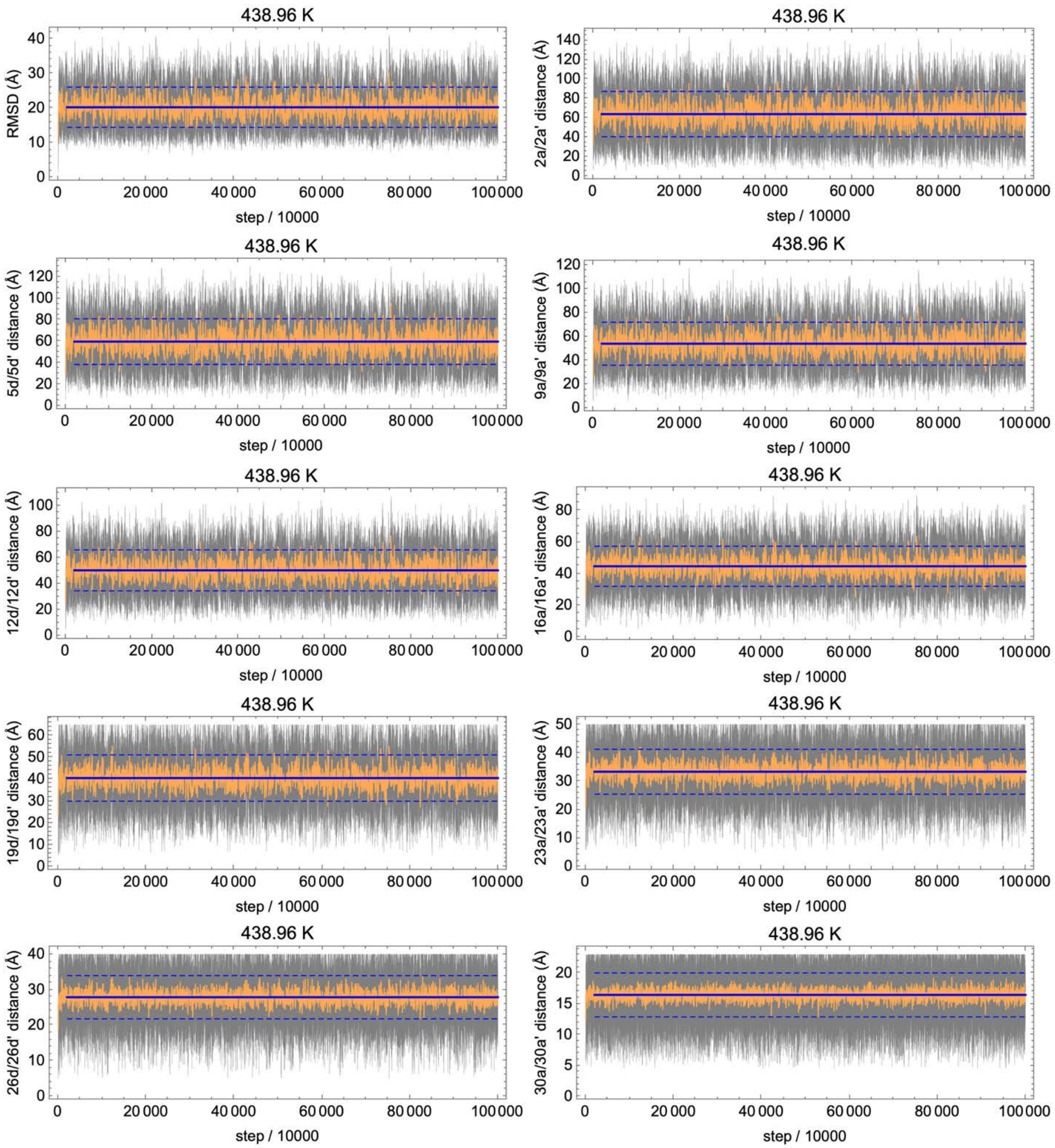

Measure at 438.96 K	Average (Å)	Standard Deviation (Å)	Slope (Å / μs)
RMSD	20.2655	5.80817	−0.0233917
5d/5d' distance	59.6603	21.2276	−0.0592769
12d/12d' distance	50.1525	15.7786	−0.195202
19d/19d' distance	40.5124	10.5794	−0.196782
26d/26d' distance	27.9089	6.08933	−0.0720784
2a/2a' distance	63.8486	23.3853	0.0232487
9a/9a' distance	53.9482	18.0254	−0.171841
16a/16a' distance	44.6837	12.6871	−0.241084
23a/23a' distance	33.3644	7.90664	−0.142693
30a/30a' distance	16.4694	3.56579	0.0105803
Core Distance	43.3943	20.7415	

Figure S272. Plots of RMSD and residue packing distances for T-REMD simulations of variant **27e/22g'-z4x** at 438.96 K.

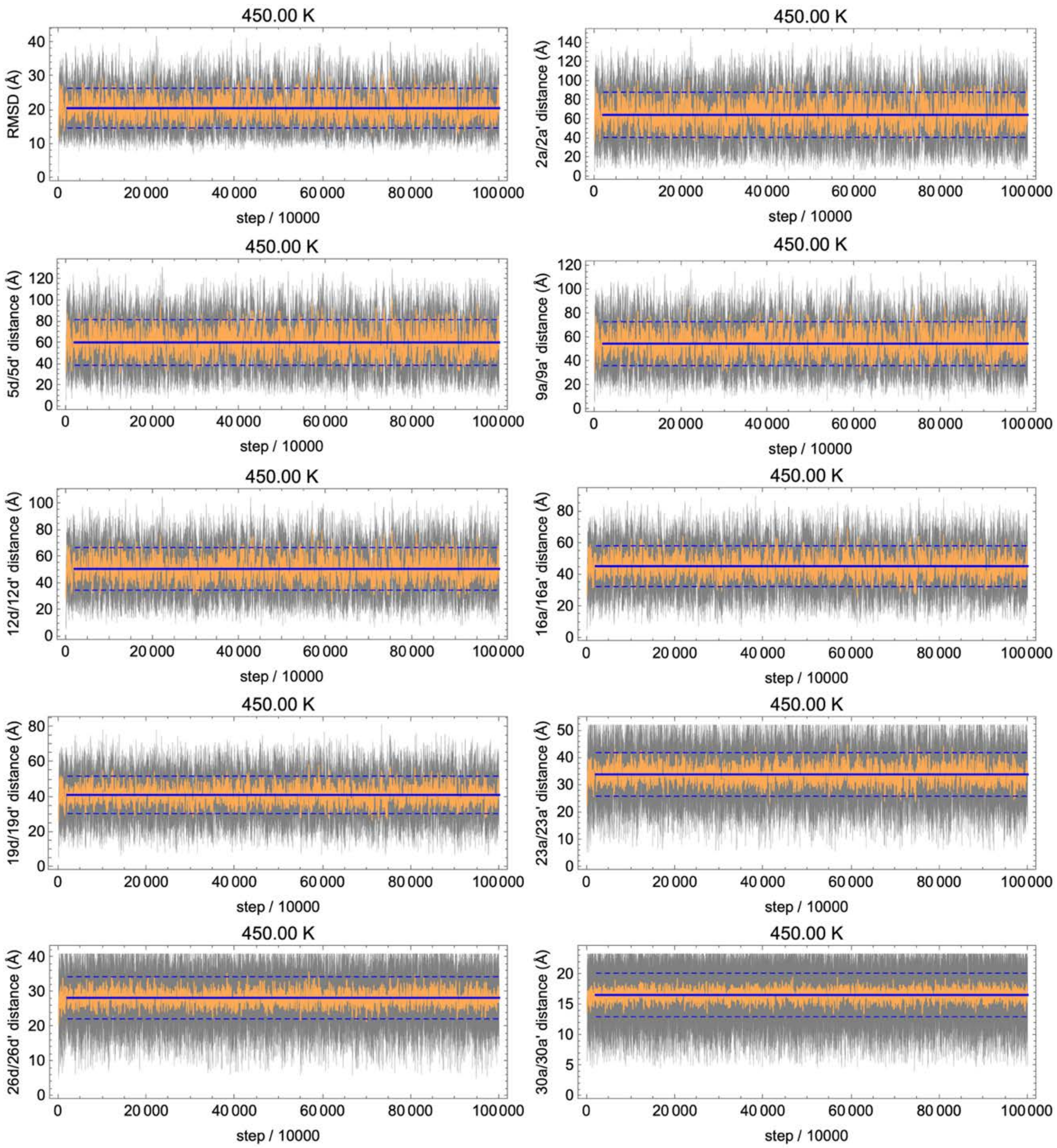

Measure at 450.00 K	Average (Å)	Standard Deviation (Å)	Slope (Å / μs)
RMSD	20.5785	5.86128	−0.00134675
5d/5d' distance	60.4197	21.5107	0.0621571
12d/12d' distance	50.9361	16.0379	−0.134587
19d/19d' distance	41.1667	10.7047	−0.157393
26d/26d' distance	28.3074	6.09072	−0.0697653
2a/2a' distance	64.5817	23.714	0.182469
9a/9a' distance	54.7481	18.3227	−0.0770583
16a/16a' distance	45.3957	12.887	−0.243323
23a/23a' distance	33.9244	7.992	−0.156155
30a/30a' distance	16.607	3.58477	0.0402397
Core Distance	44.0097	21.0223	

Figure S273. Plots of RMSD and residue packing distances for T-REMD simulations of variant **27e/22g'-z4x** at 450.00 K.

(return to Table of Contents)

s27e/22g'-z4x

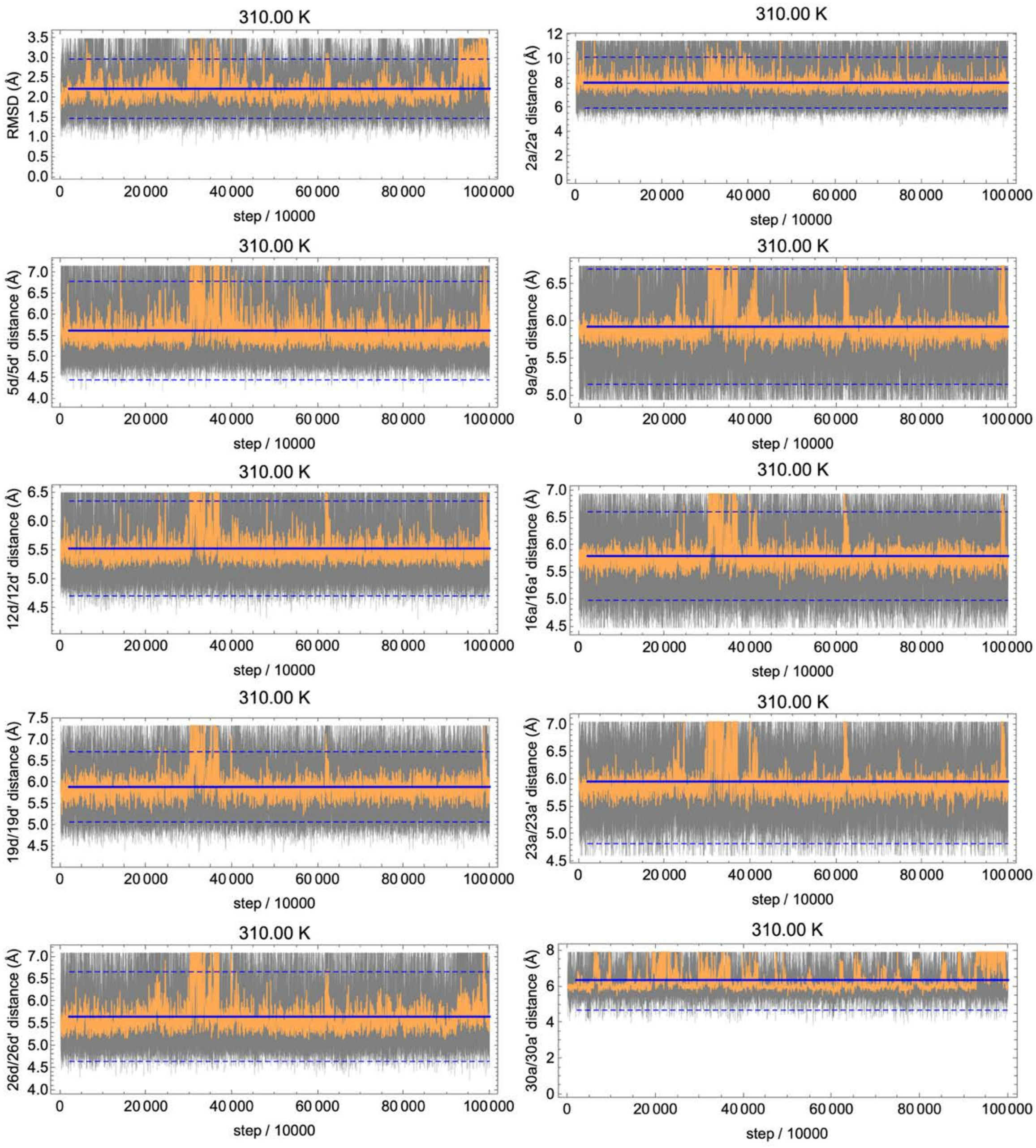

Measure at 310.00 K	Average (Å)	Standard Deviation (Å)	Slope (Å / μs)
RMSD	2.22291	0.745173	0.0715987
5d/5d' distance	5.62253	1.17409	−0.0840494
12d/12d' distance	5.53342	0.823583	−0.0336603
19d/19d' distance	5.89722	0.822589	−0.0376851
26d/26d' distance	5.65523	1.00796	−0.0286086
2a/2a' distance	8.05644	2.09527	−0.132877
9a/9a' distance	5.92938	0.774569	−0.0376298
16a/16a' distance	5.79731	0.814389	−0.0392758
23a/23a' distance	5.95943	1.13314	−0.0848751
30a/30a' distance	6.37163	1.6798	0.202801
Core Distance	6.0914	1.42741	

Figure S274. Plots of RMSD and residue packing distances for T-REMD simulations of variant **s27e/22g'-z4x** at 310.00 K.

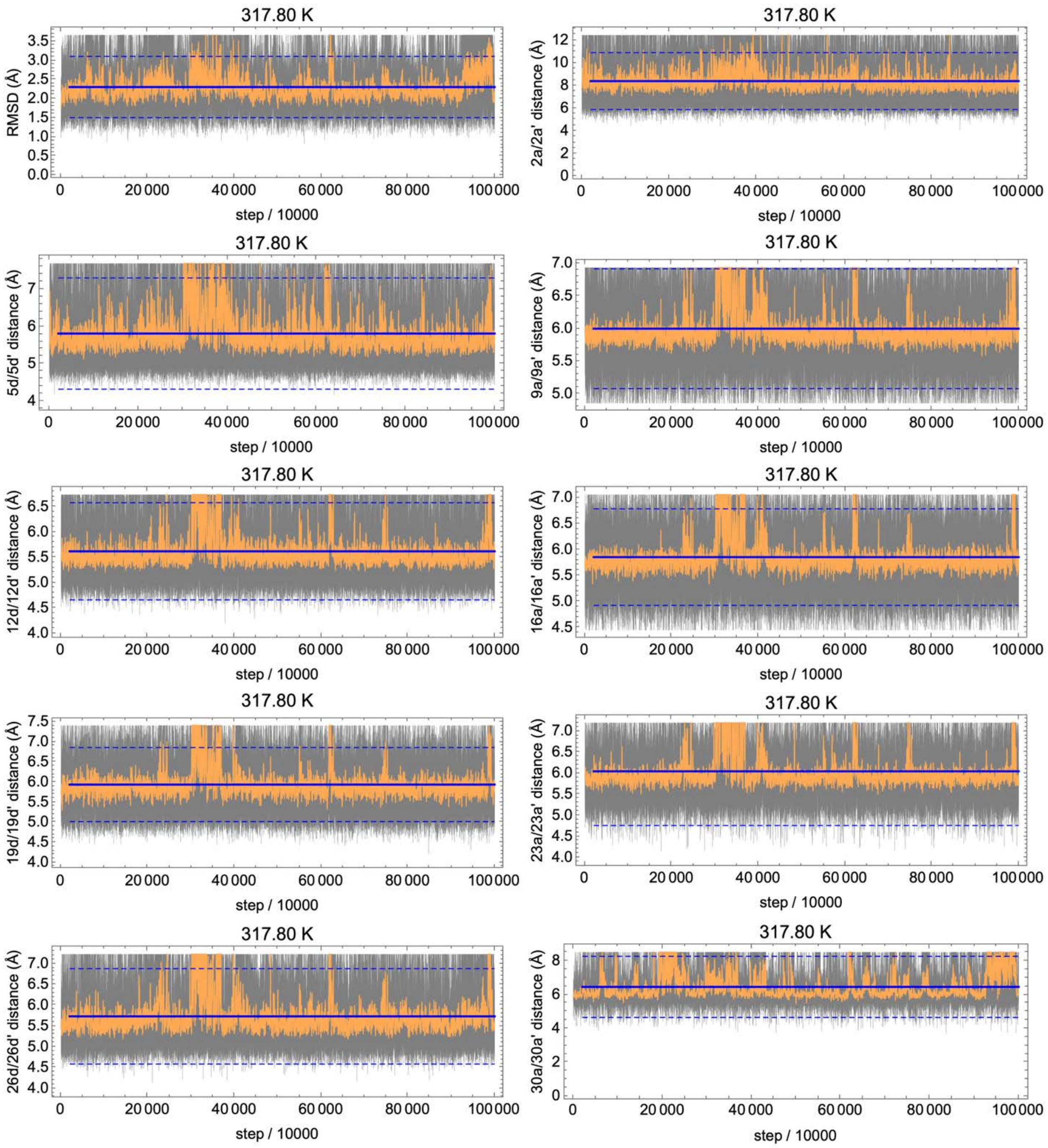

Measure at 317.80 K	Average (Å)	Standard Deviation (Å)	Slope (Å / μs)
RMSD	2.3031	0.800959	0.018472
5d/5d' distance	5.80655	1.48595	−0.111294
12d/12d' distance	5.6179	0.960226	−0.0446448
19d/19d' distance	5.93584	0.921231	−0.0444749
26d/26d' distance	5.73114	1.14648	−0.0562024
2a/2a' distance	8.38758	2.50921	−0.181759
9a/9a' distance	5.99711	0.917574	−0.0512603
16a/16a' distance	5.85387	0.928806	−0.0429222
23a/23a' distance	6.04296	1.28447	−0.093399
30a/30a' distance	6.455	1.80875	0.0921496
Core Distance	6.20311	1.63417	

Figure S275. Plots of RMSD and residue packing distances for T-REMD simulations of variant **s27e/22g'-z4x** at 317.80 K.

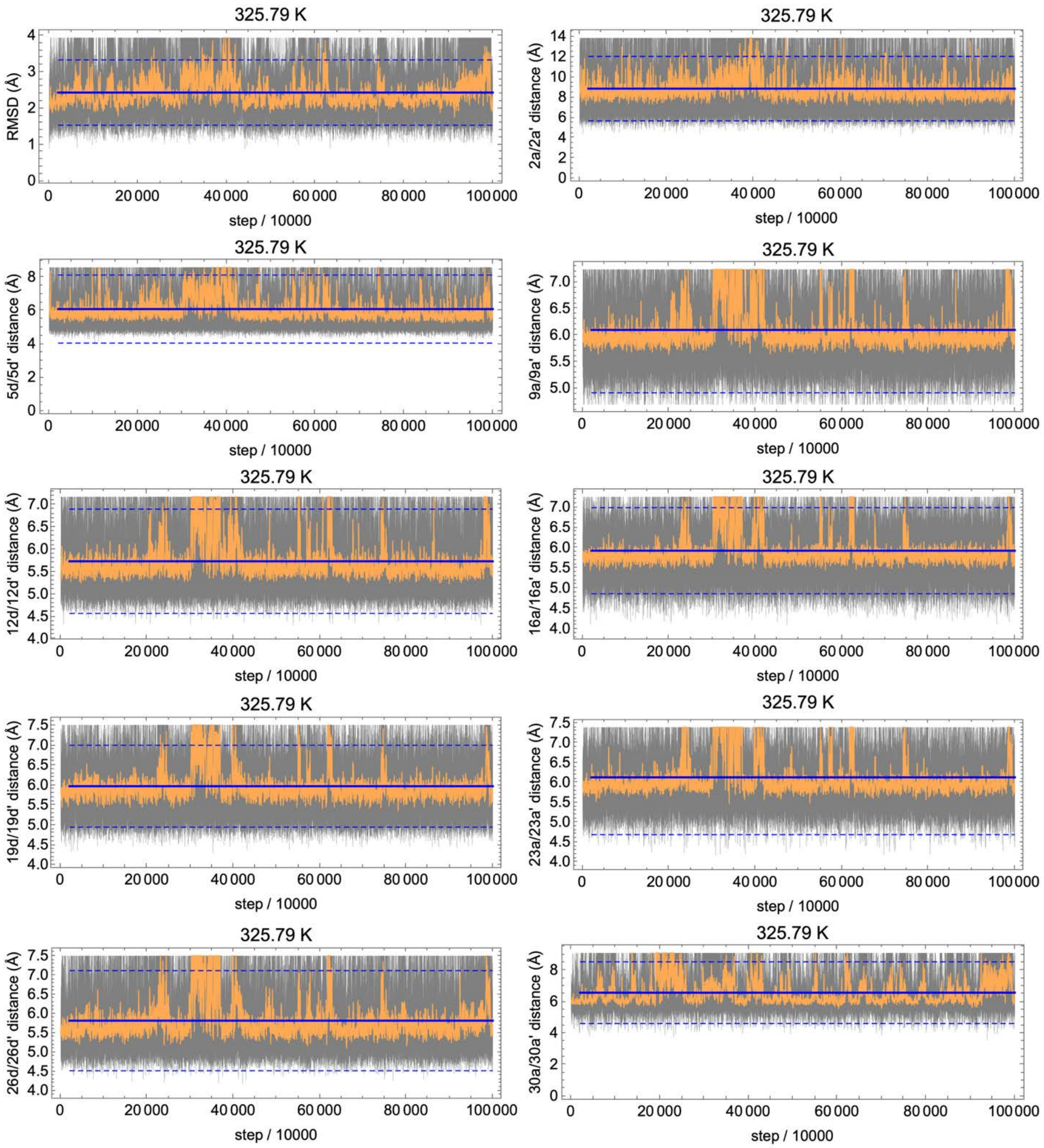

Measure at 325.79 K	Average (Å)	Standard Deviation (Å)	Slope (Å / μs)
RMSD	2.43376	0.898451	−0.0212627
5d/5d' distance	6.09743	2.03911	−0.15472
12d/12d' distance	5.73409	1.16474	−0.0632652
19d/19d' distance	5.97551	1.02617	−0.0600531
26d/26d' distance	5.82055	1.30018	−0.076544
2a/2a' distance	8.87296	3.18024	−0.235992
9a/9a' distance	6.09914	1.18407	−0.0662995
16a/16a' distance	5.92906	1.06108	−0.0549174
23a/23a' distance	6.13576	1.44691	−0.127929
30a/30a' distance	6.58003	1.9584	−0.0158284
Core Distance	6.3605	1.95492	

Figure S276. Plots of RMSD and residue packing distances for T-REMD simulations of variant **s27e/22g'-z4x** at 325.79 K.

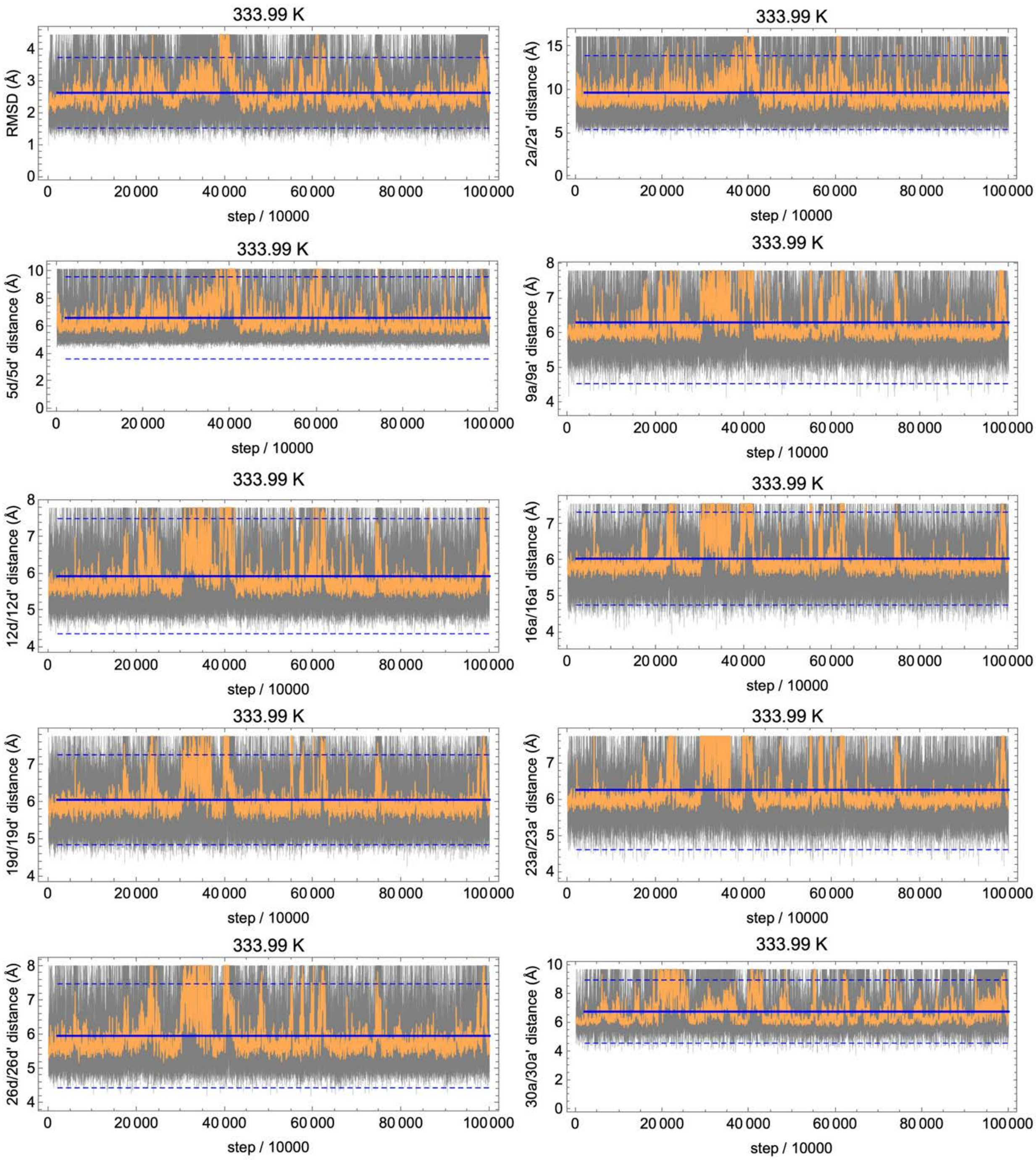

Measure at 333.99 K	Average (Å)	Standard Deviation (Å)	Slope (Å / μs)
RMSD	2.63982	1.10588	−0.0753308
5d/5d' distance	6.61872	2.98859	−0.198298
12d/12d' distance	5.93038	1.57099	−0.0855455
19d/19d' distance	6.05924	1.20451	−0.0665114
26d/26d' distance	5.96319	1.52304	−0.10064
2a/2a' distance	9.68077	4.27467	−0.317535
9a/9a' distance	6.30506	1.76658	−0.104124
16a/16a' distance	6.0454	1.2935	−0.077255
23a/23a' distance	6.26998	1.65518	−0.13574
30a/30a' distance	6.77691	2.19658	−0.155658
Core Distance	6.62774	2.51588	

Figure S277. Plots of RMSD and residue packing distances for T-REMD simulations of variant **s27e/22g’-z4x** at 333.99 K.

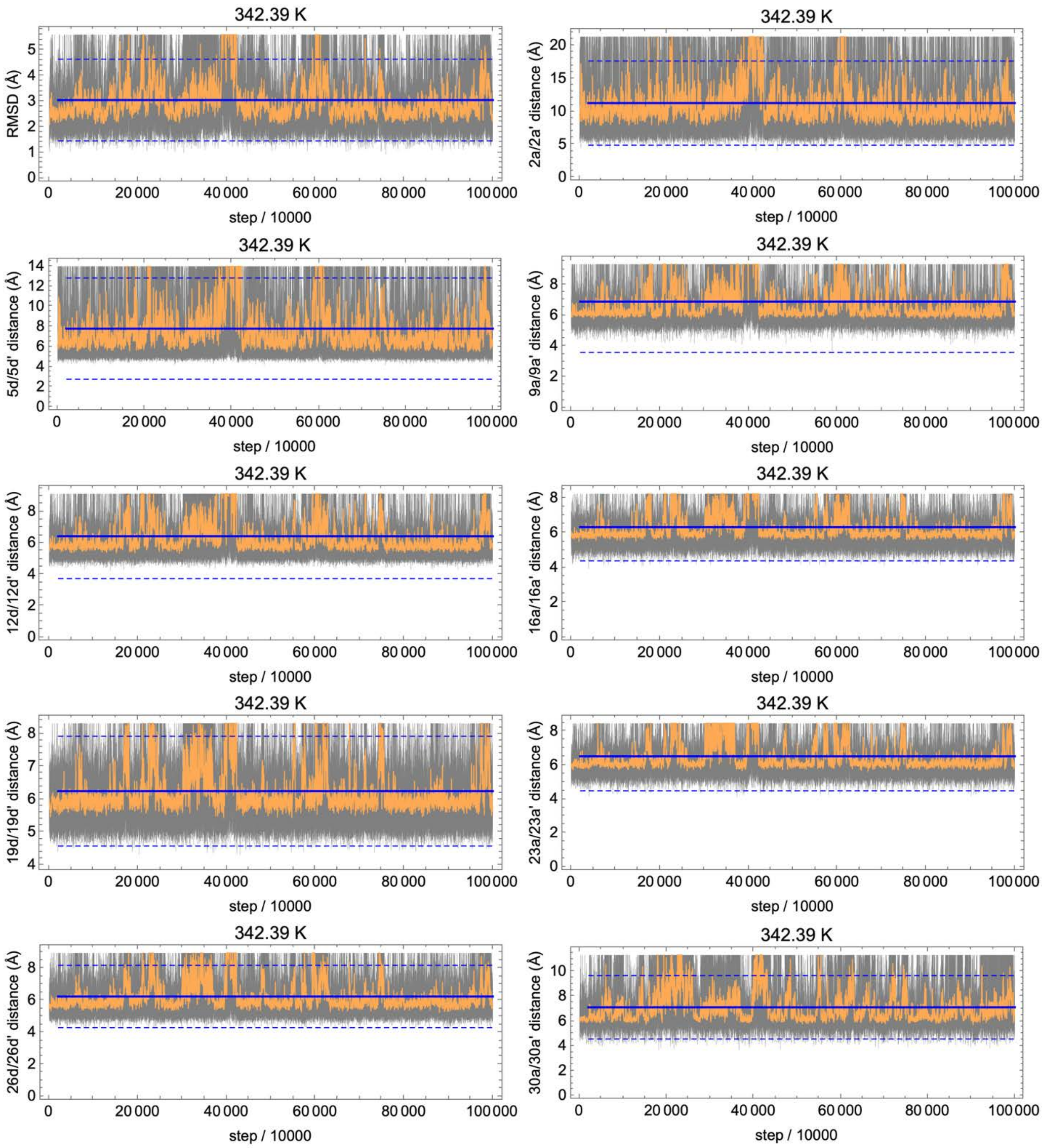

Measure at 342.39 K	Average (Å)	Standard Deviation (Å)	Slope (Å / μs)
RMSD	3.04851	1.5951	−0.13817
5d/5d' distance	7.79711	5.0335	−0.356386
12d/12d' distance	6.42694	2.70218	−0.190492
19d/19d' distance	6.24602	1.67381	−0.0957885
26d/26d' distance	6.23521	1.94554	−0.133254
2a/2a' distance	11.2624	6.38353	−0.539828
9a/9a' distance	6.88545	3.30081	−0.217535
16a/16a' distance	6.32165	1.93457	−0.13346
23a/23a' distance	6.51958	2.0357	−0.16747
30a/30a' distance	7.10853	2.54795	−0.238997
Core Distance	7.20033	3.73999	

Figure S278. Plots of RMSD and residue packing distances for T-REMD simulations of variant **s27e/22g'-z4x** at 342.39 K.

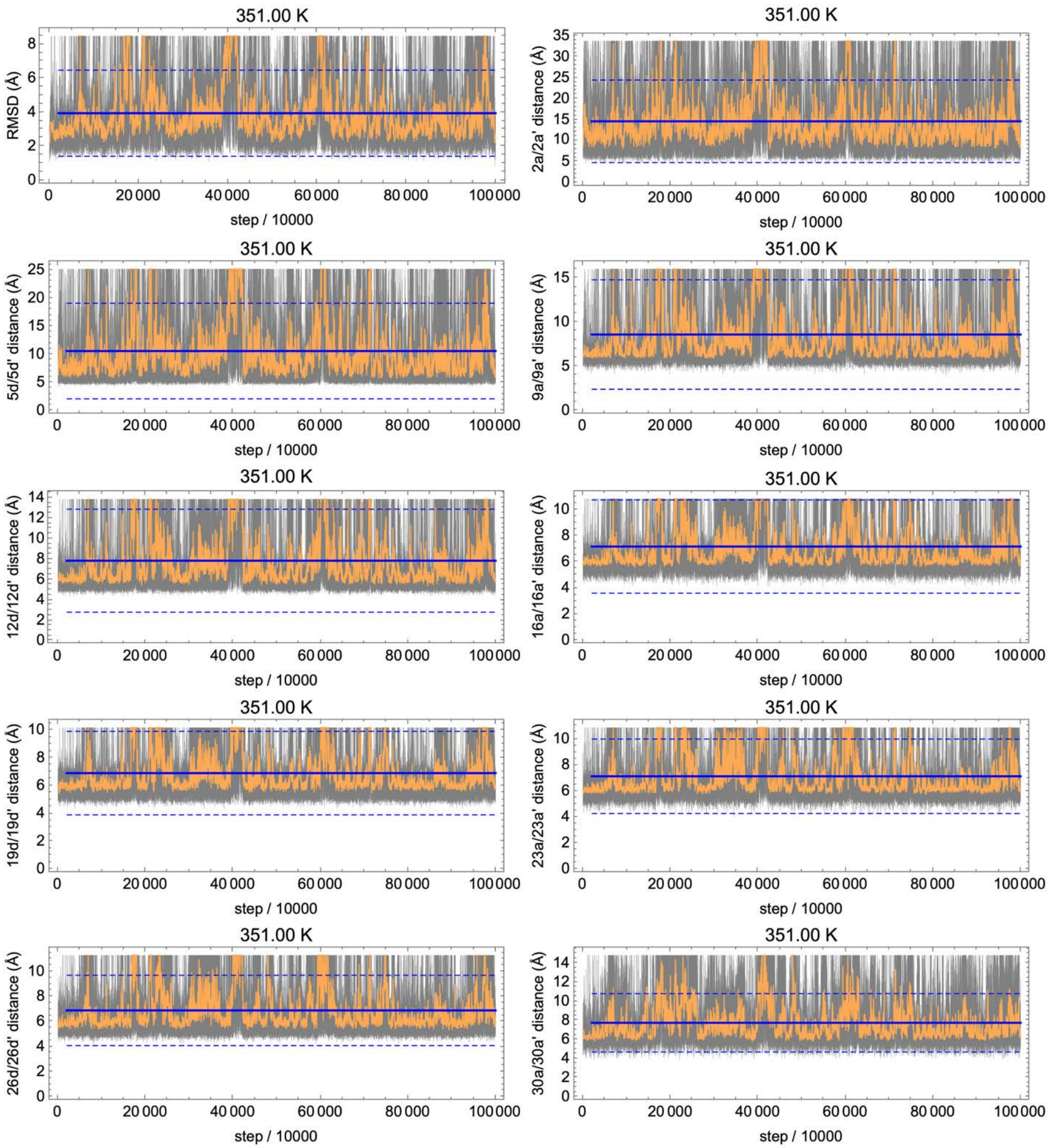

Measure at 351.00 K	Average (Å)	Standard Deviation (Å)	Slope (Å / μs)
RMSD	3.93671	2.52986	−0.181262
5d/5d' distance	10.5728	8.5099	−0.424557
12d/12d' distance	7.82305	5.0425	−0.219275
19d/19d' distance	6.89028	3.00059	−0.122085
26d/26d' distance	6.87983	2.80604	−0.168921
2a/2a' distance	14.5847	9.85888	−0.636124
9a/9a' distance	8.58648	6.19524	−0.239964
16a/16a' distance	7.15781	3.56377	−0.179961
23a/23a' distance	7.12418	2.85882	−0.214941
30a/30a' distance	7.73979	3.05966	−0.346384
Core Distance	8.59544	6.07111	

Figure S279. Plots of RMSD and residue packing distances for T-REMD simulations of variant **s27e/22g'-z4x** at 351.00 K.

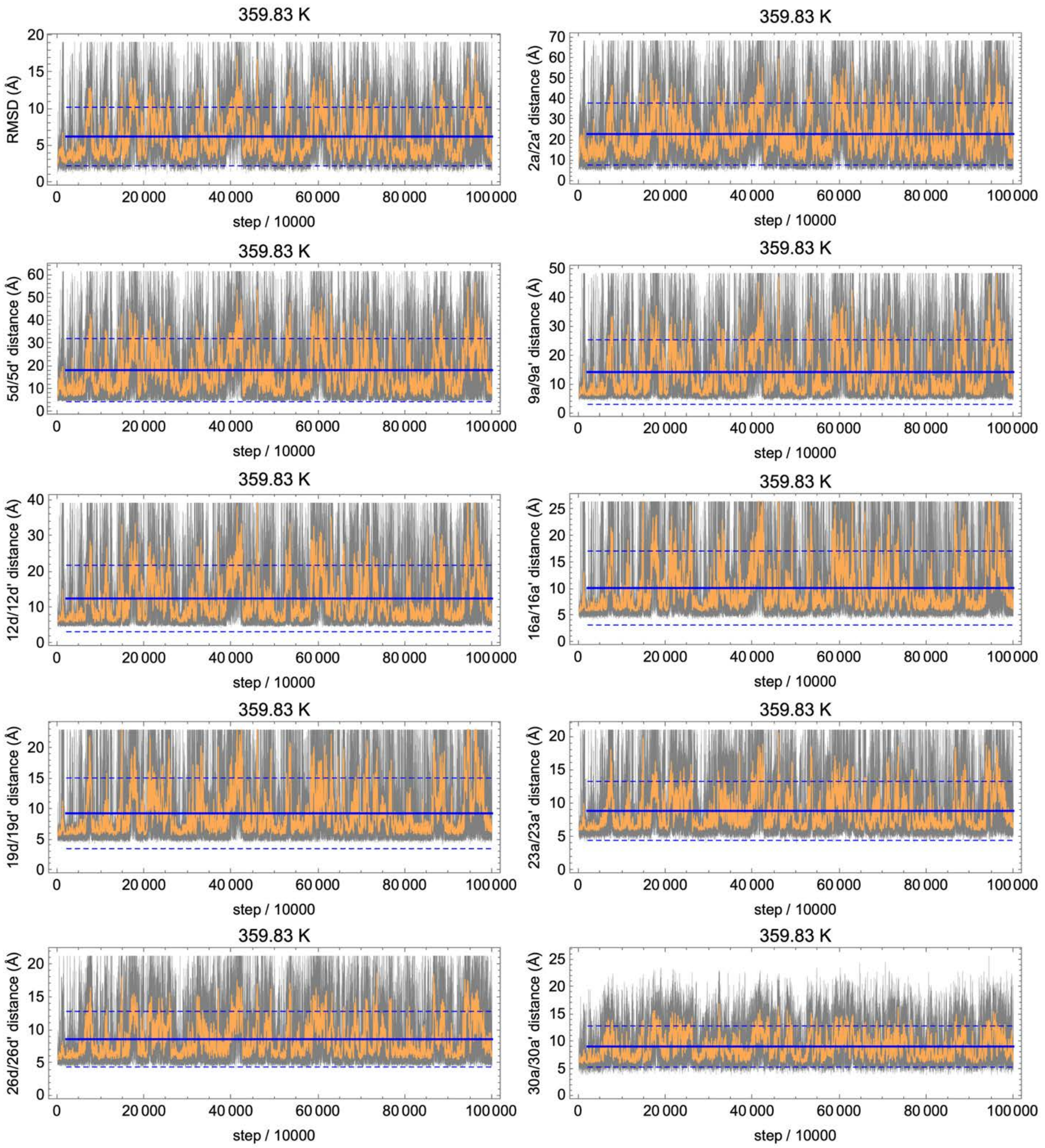

Measure at 359.83 K	Average (Å)	Standard Deviation (Å)	Slope (Å / μs)
RMSD	6.2428	3.98731	0.406125
5d/5d' distance	18.2763	13.8682	1.39301
12d/12d' distance	12.52	9.31439	1.00442
19d/19d' distance	9.31614	5.7957	0.650365
26d/26d' distance	8.64452	4.2146	0.387338
2a/2a' distance	22.9342	15.1322	1.28589
9a/9a' distance	14.3514	11.1707	1.31255
16a/16a' distance	10.1862	6.98046	0.724638
23a/23a' distance	8.8829	4.41827	0.368319
30a/30a' distance	9.10078	3.76792	0.135639
Core Distance	12.6903	10.3654	

Figure S280. Plots of RMSD and residue packing distances for T-REMD simulations of variant **s27e/22g'-z4x** at 359.83 K.

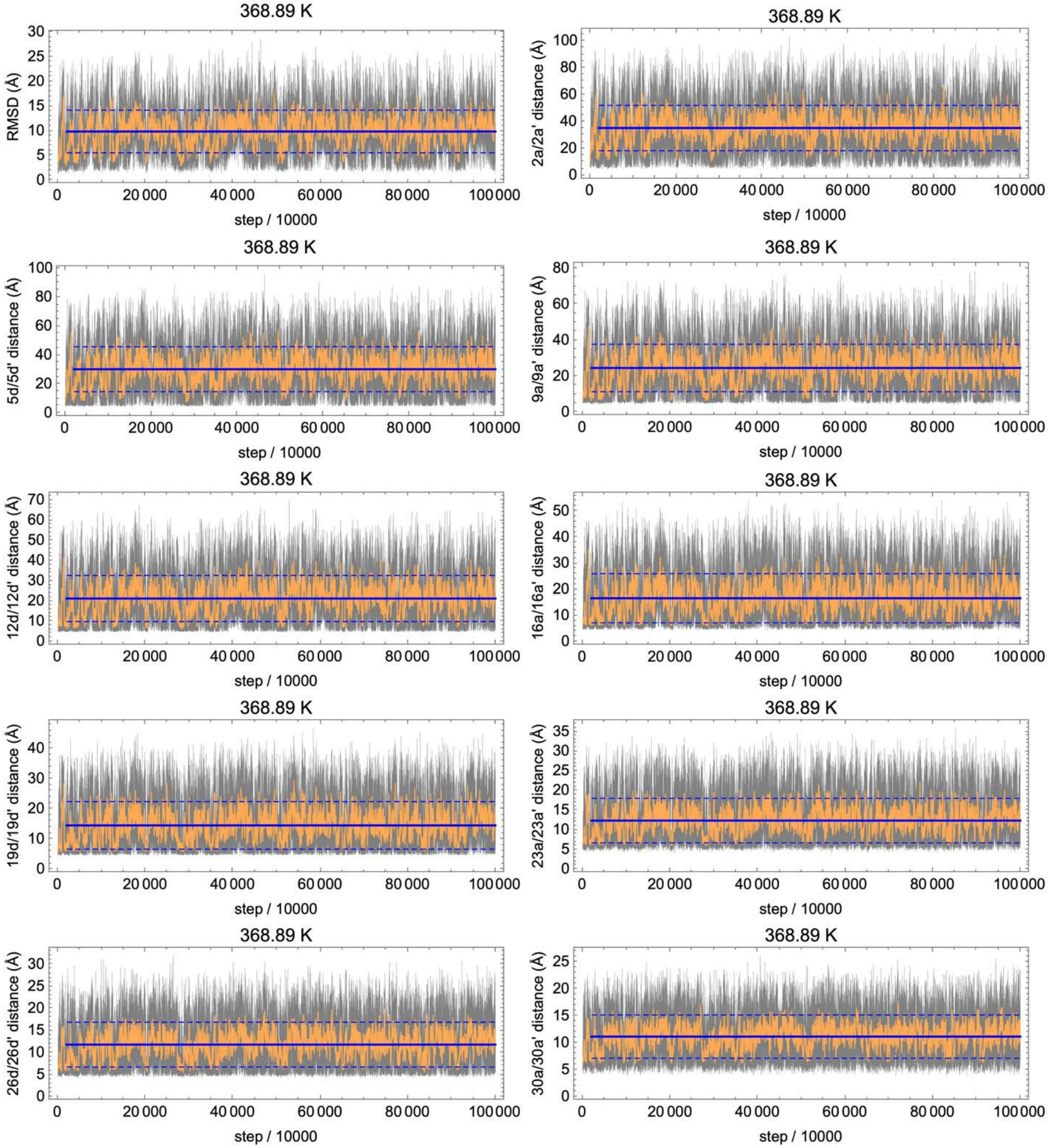

Measure at 368.89 K	Average (Å)	Standard Deviation (Å)	Slope (Å / μs)
RMSD	9.86123	4.31887	0.727497
5d/5d' distance	30.2415	15.529	2.29527
12d/12d' distance	21.2374	11.4333	1.84933
19d/19d' distance	14.5158	7.87821	1.21305
26d/26d' distance	11.815	5.07417	0.758421
2a/2a' distance	35.1134	16.8496	2.16324
9a/9a' distance	24.5723	13.1893	2.23577
16a/16a' distance	16.6193	9.40714	1.38475
23a/23a' distance	12.3603	5.65879	0.764697
30a/30a' distance	11.1564	4.01153	0.463357
Core Distance	19.7368	13.5579	

Figure S281. Plots of RMSD and residue packing distances for T-REMD simulations of variant **s27e/22g'-z4x** at 368.89 K.

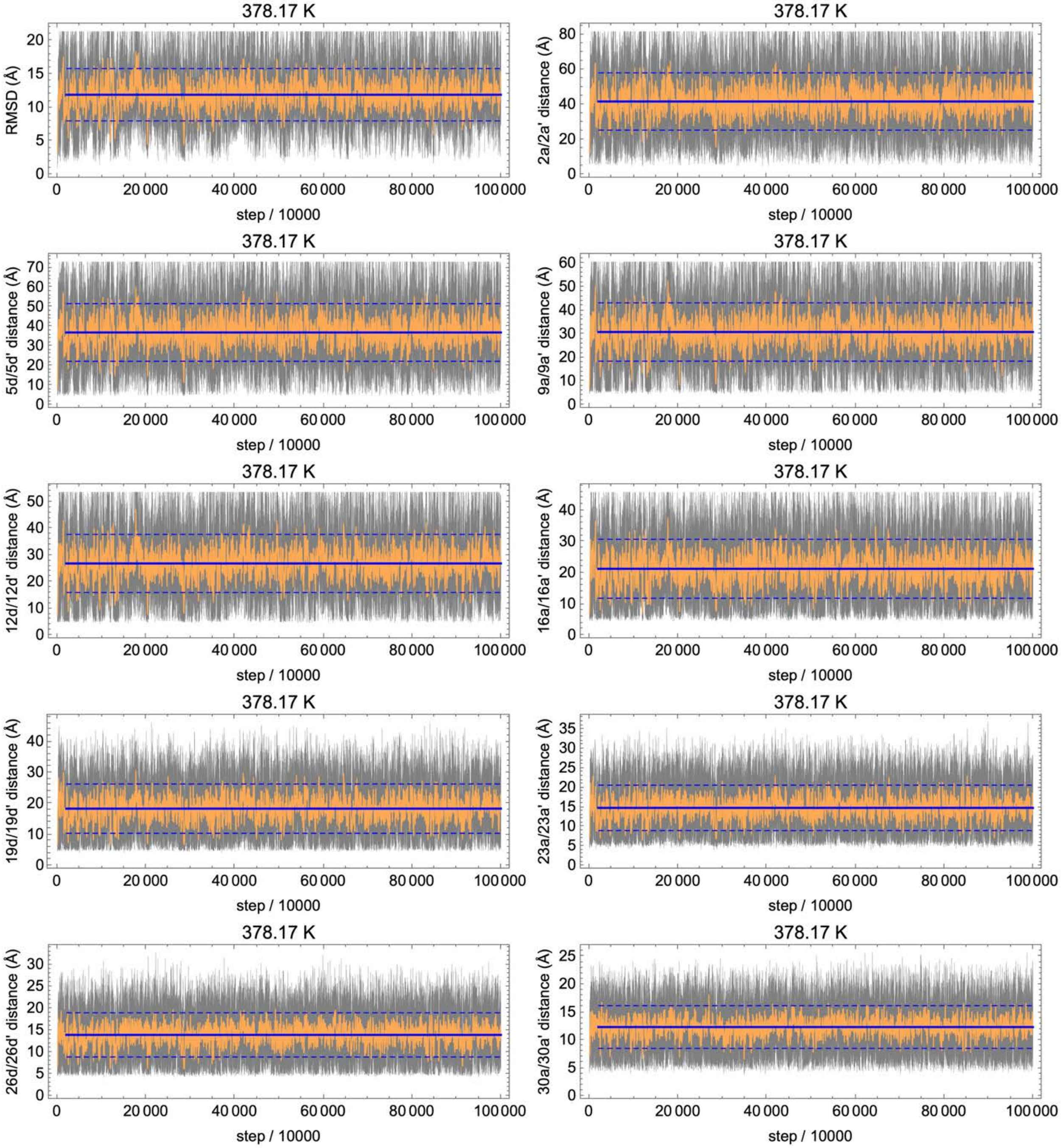

Measure at 378.17 K	Average (Å)	Standard Deviation (Å)	Slope (Å / μs)
RMSD	11.893	3.90572	0.163385
5d/5d' distance	36.942	14.7008	0.510675
12d/12d' distance	26.8842	10.8931	0.466647
19d/19d' distance	18.3716	7.96371	0.398474
26d/26d' distance	13.9219	5.04559	0.316342
2a/2a' distance	41.6828	16.3514	0.429103
9a/9a' distance	30.8406	12.3484	0.583425
16a/16a' distance	21.2985	9.44573	0.423468
23a/23a' distance	14.7886	5.82741	0.276969
30a/30a' distance	12.3476	3.83063	0.16929
Core Distance	24.1197	14.4441	

Figure S282. Plots of RMSD and residue packing distances for T-REMD simulations of variant **s27e/22g'-z4x** at 378.17 K.

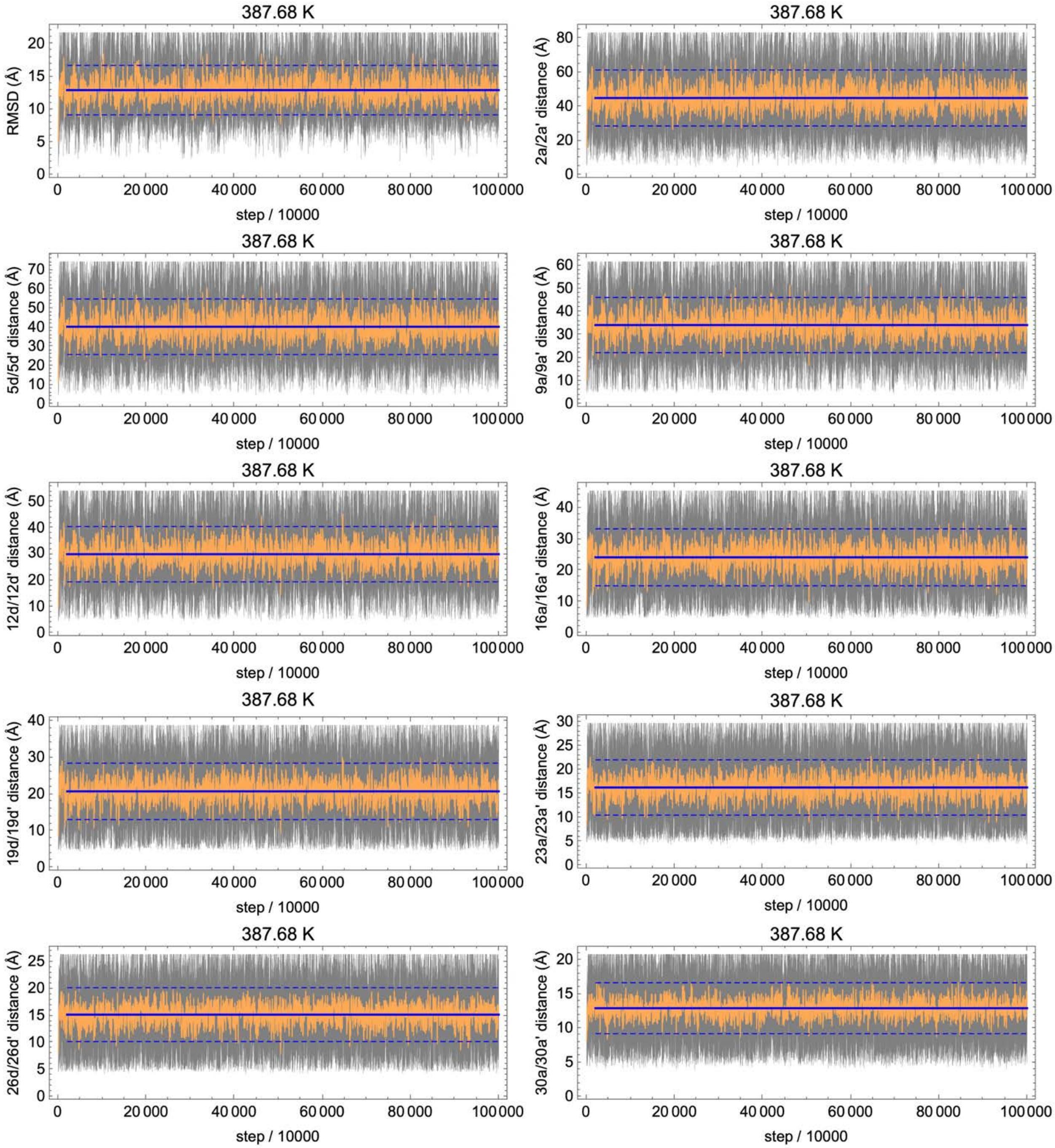

Measure at 387.68 K	Average (Å)	Standard Deviation (Å)	Slope (Å / μs)
RMSD	12.9277	3.77607	−0.0658479
5d/5d' distance	40.304	14.5027	−0.118661
12d/12d' distance	30.042	10.5349	−0.0761543
19d/19d' distance	20.7569	7.69136	−0.0190441
26d/26d' distance	15.1802	4.9854	0.114758
2a/2a' distance	44.985	16.4062	−0.211307
9a/9a' distance	34.1419	11.951	−0.0444073
16a/16a' distance	24.1753	9.10384	−0.096072
23a/23a' distance	16.2852	5.77885	−0.00329177
30a/30a' distance	12.9281	3.72424	0.0704189
Core Distance	26.5332	14.9283	

Figure S283. Plots of RMSD and residue packing distances for T-REMD simulations of variant **s27e/22g'-z4x** at 387.68 K.

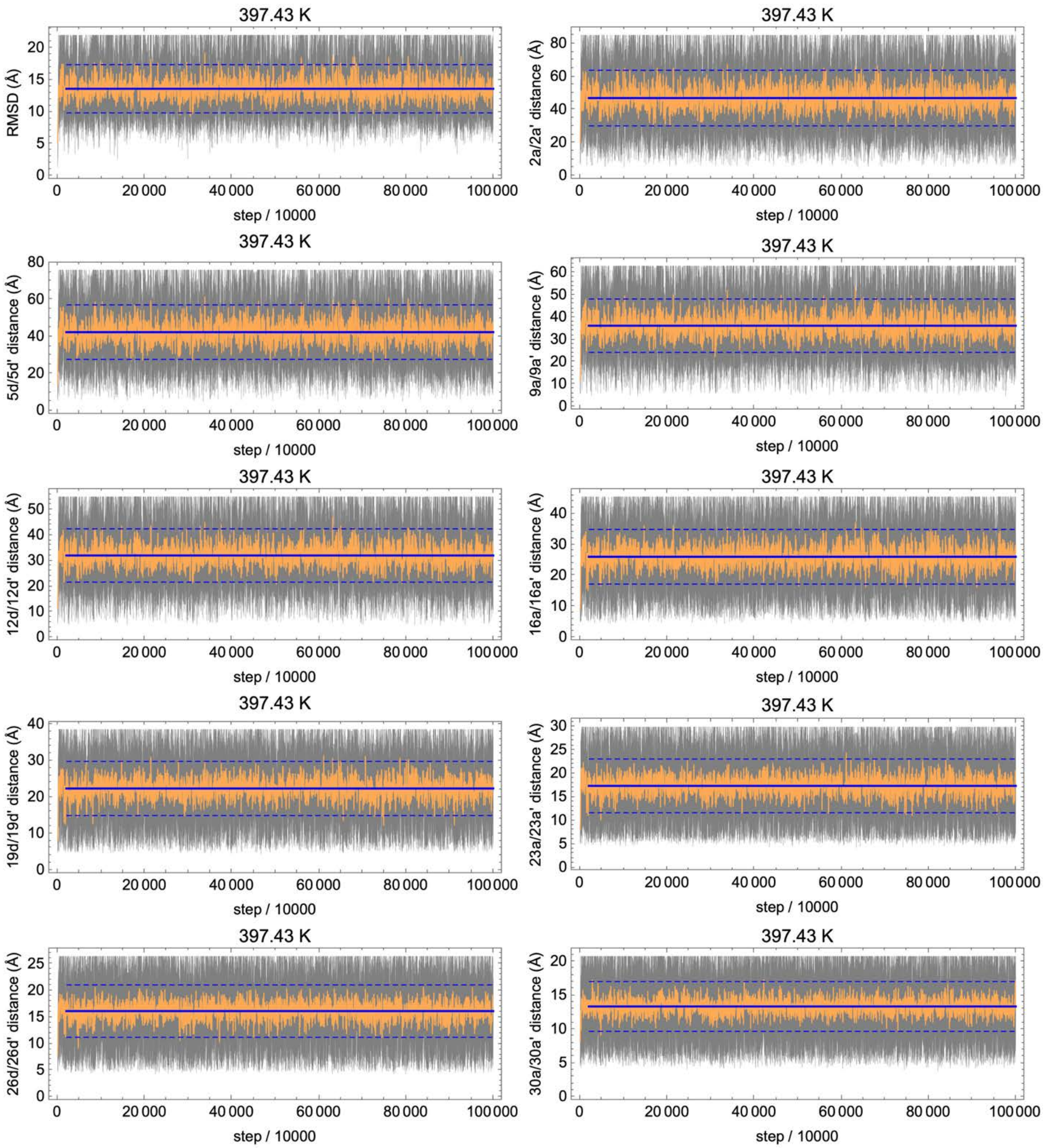

Measure at 397.43 K	Average (Å)	Standard Deviation (Å)	Slope (Å / μs)
RMSD	13.6089	3.7712	−0.0754377
5d/5d' distance	42.3721	14.7405	−0.054273
12d/12d' distance	32.0321	10.4222	−0.0437848
19d/19d' distance	22.3567	7.42832	−0.00266047
26d/26d' distance	16.138	4.93103	0.0208546
2a/2a' distance	46.9807	16.8042	−0.104797
9a/9a' distance	36.1766	11.9438	−0.0426306
16a/16a' distance	26.051	8.83185	−0.0556094
23a/23a' distance	17.3511	5.70564	−0.0478957
30a/30a' distance	13.357	3.68224	0.0023054
Core Distance	28.0906	15.3064	

Figure S284. Plots of RMSD and residue packing distances for T-REMD simulations of variant **s27e/22g'-z4x** at 397.43 K.

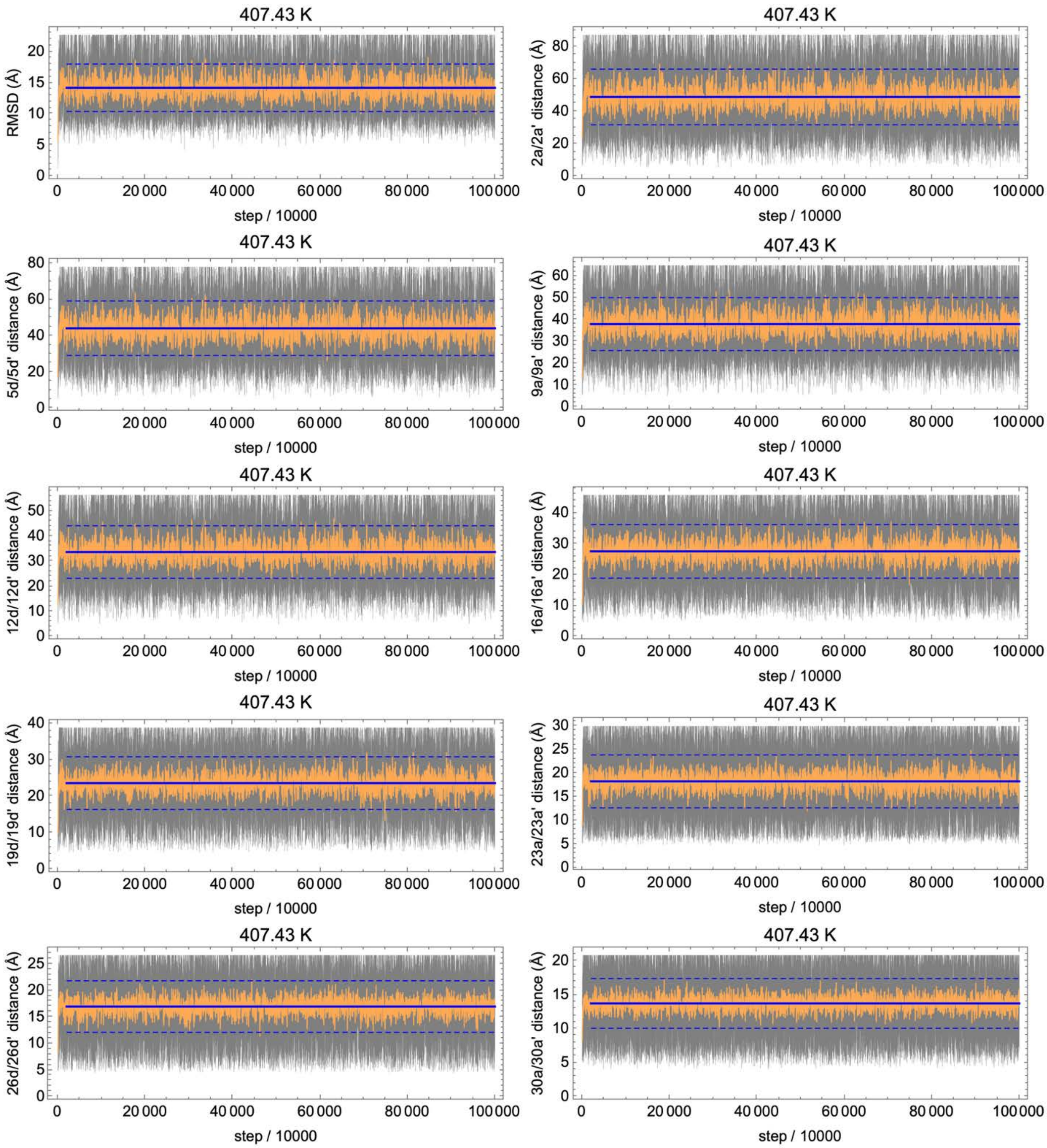

Measure at 407.43 K	Average (Å)	Standard Deviation (Å)	Slope (Å / μs)
RMSD	14.1911	3.8158	−0.0499454
5d/5d' distance	44.1044	15.0339	−0.0957682
12d/12d' distance	33.6167	10.4682	−0.0282688
19d/19d' distance	23.5951	7.24208	0.0706546
26d/26d' distance	16.9537	4.85538	0.0445546
2a/2a' distance	48.7746	17.1669	−0.129826
9a/9a' distance	37.8438	12.1284	−0.0735395
16a/16a' distance	27.5261	8.67778	0.000192313
23a/23a' distance	18.2085	5.58985	0.0289839
30a/30a' distance	13.7193	3.66421	0.0278383
Core Distance	29.3713	15.6961	

Figure S285. Plots of RMSD and residue packing distances for T-REMD simulations of variant **s27e/22g'-z4x** at 407.43 K.

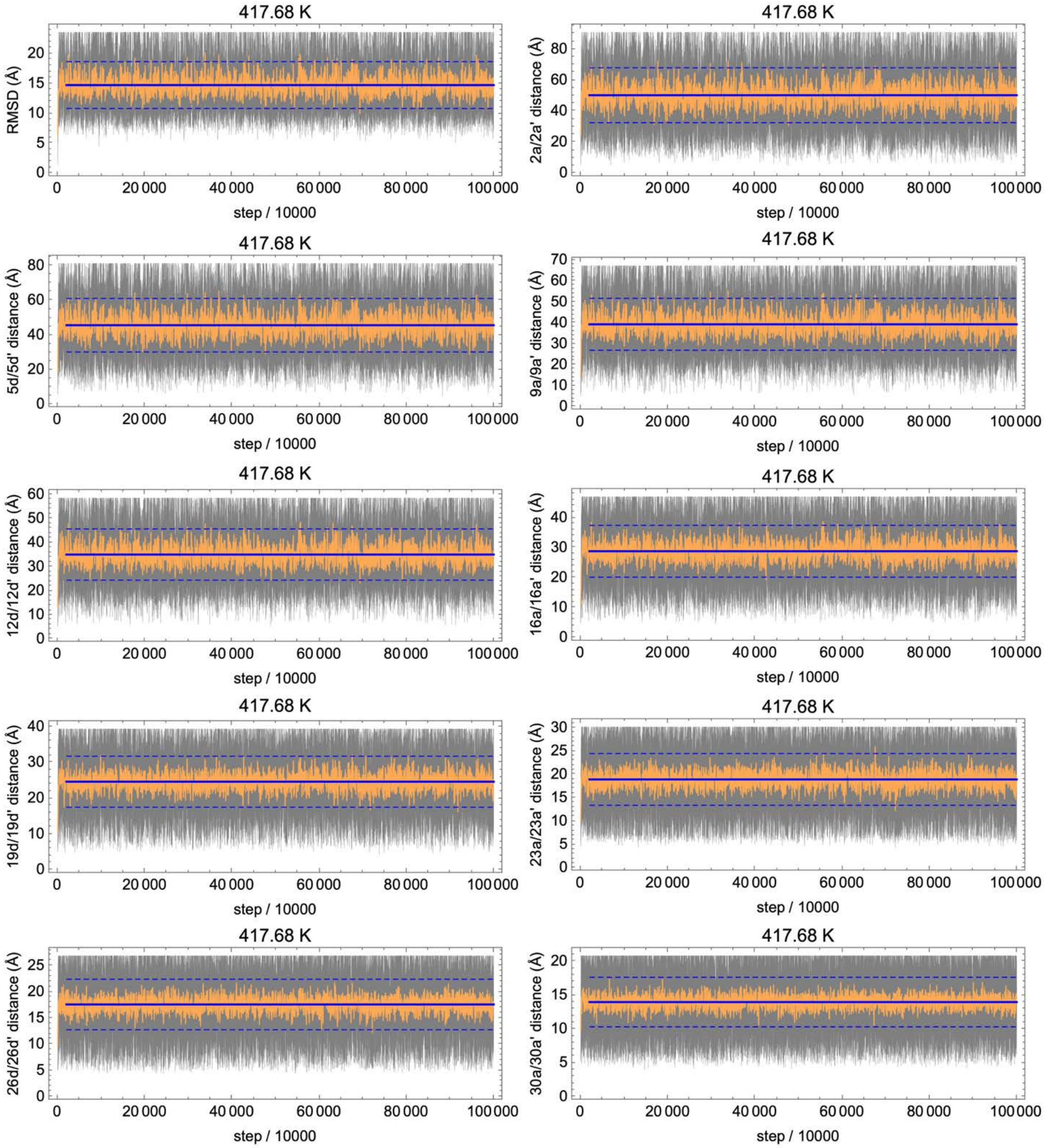

Measure at 417.68 K	Average (Å)	Standard Deviation (Å)	Slope (Å / μs)
RMSD	14.7178	3.91343	−0.0354512
5d/5d' distance	45.6086	15.457	−0.014899
12d/12d' distance	35.0197	10.6391	−0.0255621
19d/19d' distance	24.6114	7.12866	0.0668817
26d/26d' distance	17.5705	4.8206	0.0368855
2a/2a' distance	50.2602	17.7219	−0.0451473
9a/9a' distance	39.3076	12.3888	−0.0667582
16a/16a' distance	28.7753	8.63554	−0.0152738
23a/23a' distance	18.9201	5.54509	0.0264402
30a/30a' distance	13.9763	3.65977	0.021641
Core Distance	30.45	16.1283	

Figure S286. Plots of RMSD and residue packing distances for T-REMD simulations of variant **s27e/22g’-z4x** at 417.68 K.

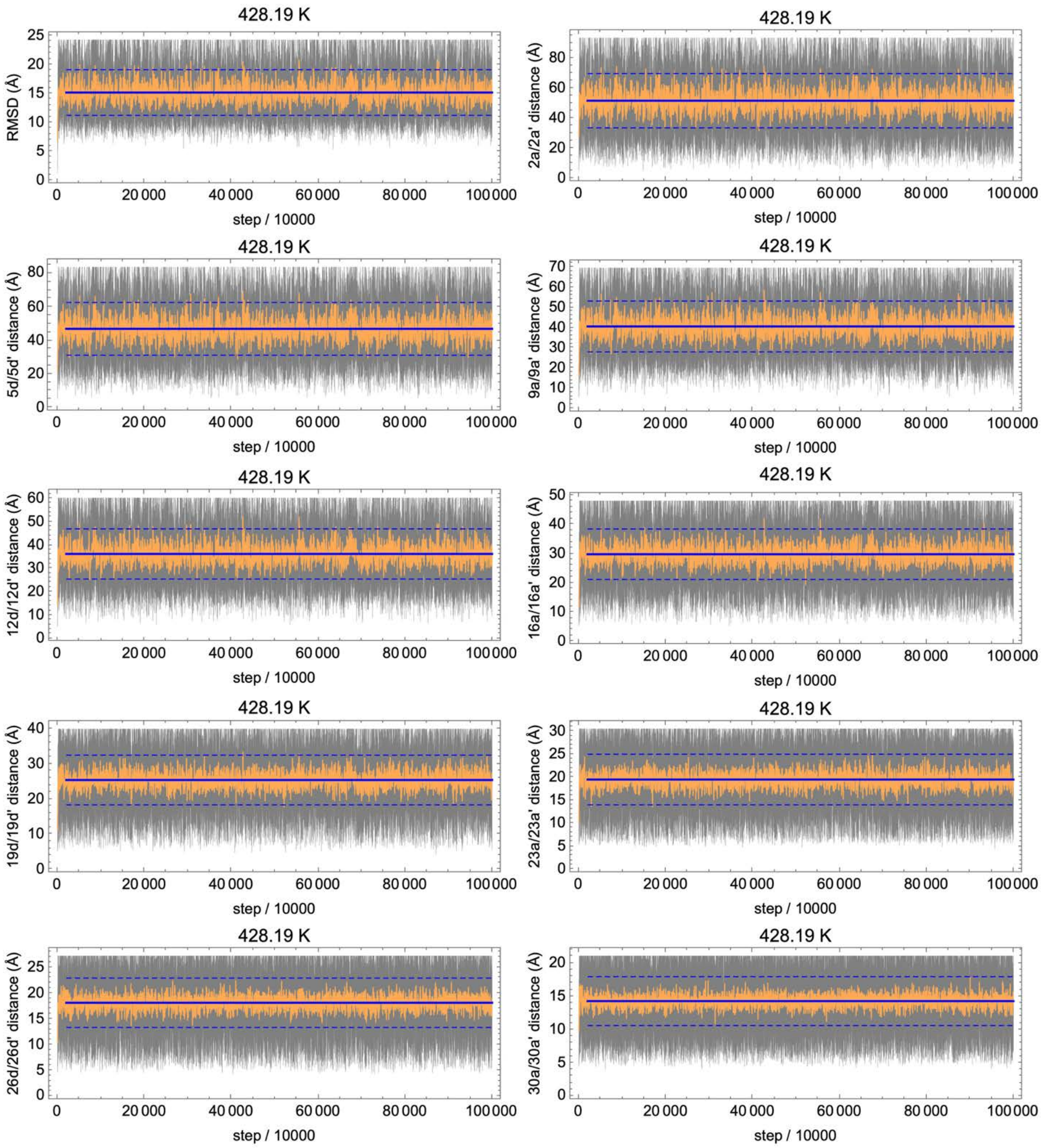

Measure at 428.19 K	Average (Å)	Standard Deviation (Å)	Slope (Å / μs)
RMSD	15.1522	3.94183	−0.0307978
5d/5d' distance	46.8978	15.7514	−0.0986497
12d/12d' distance	36.1502	10.7512	−0.0476908
19d/19d' distance	25.3914	7.07181	0.0823978
26d/26d' distance	18.1227	4.79166	0.0458731
2a/2a' distance	51.5222	18.0648	−0.138571
9a/9a' distance	40.5187	12.5855	−0.0644714
16a/16a' distance	29.7621	8.63549	0.0133273
23a/23a' distance	19.4694	5.5004	0.058286
30a/30a' distance	14.254	3.6594	0.0778633
Core Distance	31.3432	16.4714	

Figure S287. Plots of RMSD and residue packing distances for T-REMD simulations of variant **s27e/22g'-z4x** at 428.19 K.

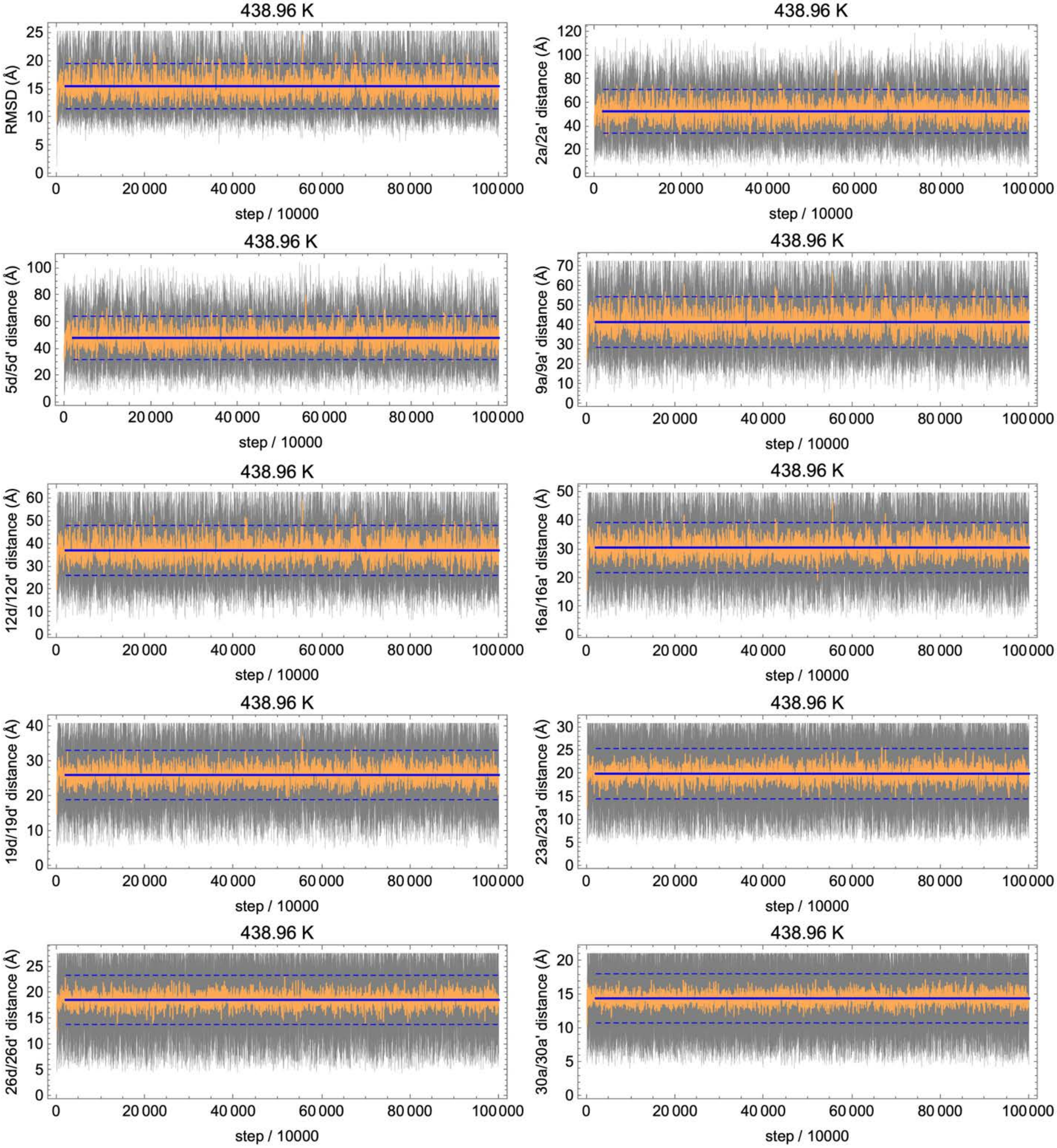

Measure at 438.96 K	Average (Å)	Standard Deviation (Å)	Slope (Å / μs)
RMSD	15.551	4.01424	0.00965506
5d/5d' distance	48.04	16.1463	0.0276571
12d/12d' distance	37.1603	10.9914	0.0392466
19d/19d' distance	26.0665	7.05845	0.11604
26d/26d' distance	18.6063	4.76644	0.0257033
2a/2a' distance	52.7298	18.5259	0.0095296
9a/9a' distance	41.625	12.9037	0.0576554
16a/16a' distance	30.6281	8.72981	0.0487512
23a/23a' distance	20.0025	5.45807	0.0958795
30a/30a' distance	14.4522	3.63638	0.060585
Core Distance	32.1456	16.857	

Figure S288. Plots of RMSD and residue packing distances for T-REMD simulations of variant **s27e/22g'-z4x** at 438.96 K.

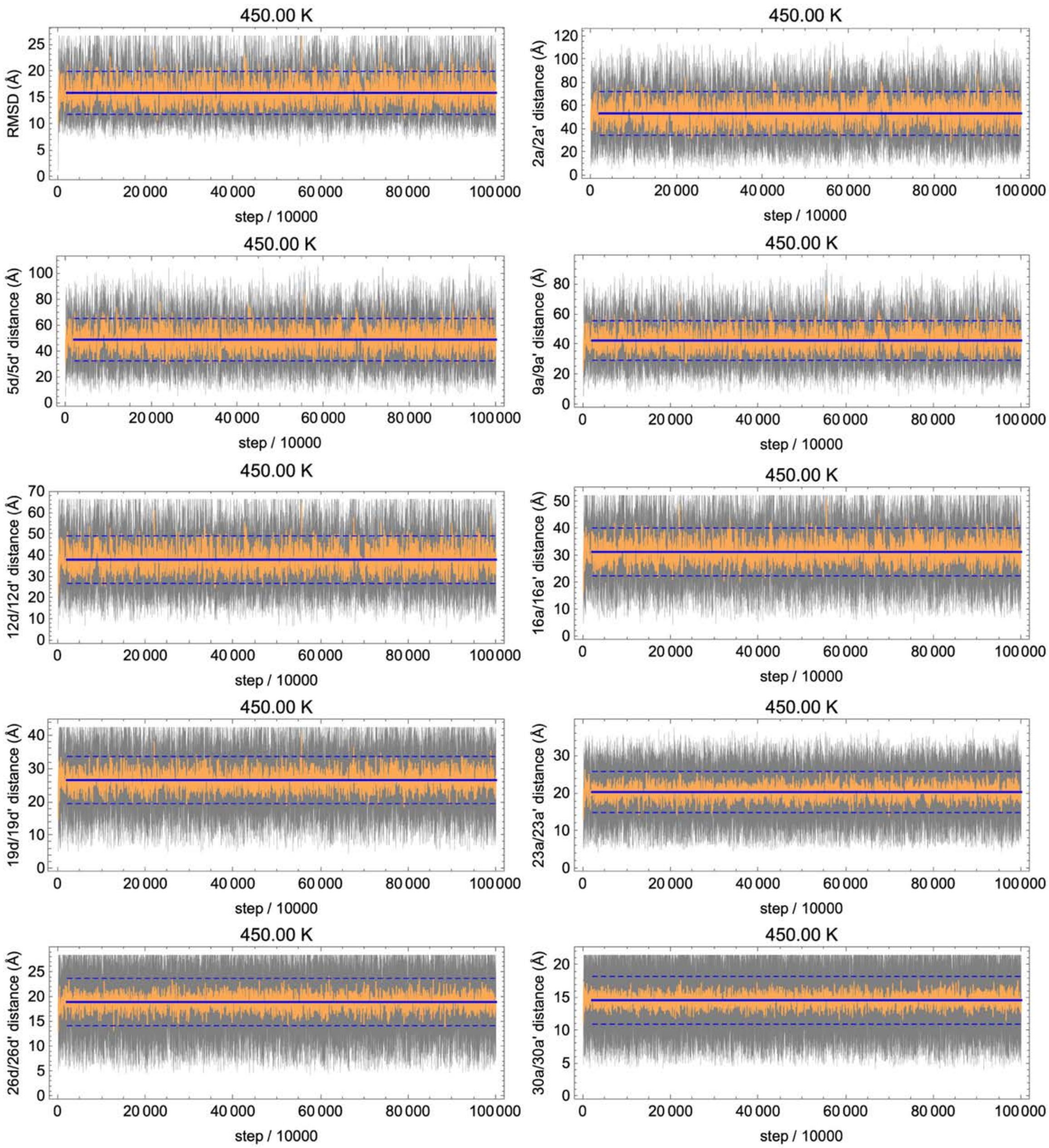

Measure at 450.00 K	Average (Å)	Standard Deviation (Å)	Slope (Å / μs)
RMSD	15.9121	4.05484	0.0517165
5d/5d' distance	49.069	16.3973	0.264738
12d/12d' distance	38.121	11.2025	0.0449667
19d/19d' distance	26.6503	7.08282	0.124398
26d/26d' distance	18.9855	4.76727	0.0229578
2a/2a' distance	53.7505	18.8293	0.398524
9a/9a' distance	42.6388	13.1456	0.0976684
16a/16a' distance	31.4098	8.86131	0.0435137
23a/23a' distance	20.4315	5.46212	0.0719956
30a/30a' distance	14.6357	3.63792	0.0651529
Core Distance	32.8547	17.1865	

Figure S289. Plots of RMSD and residue packing distances for T-REMD simulations of variant **s27e/22g'-z4x** at 450.00 K.

(return to Table of Contents)

34e/29g’-xx

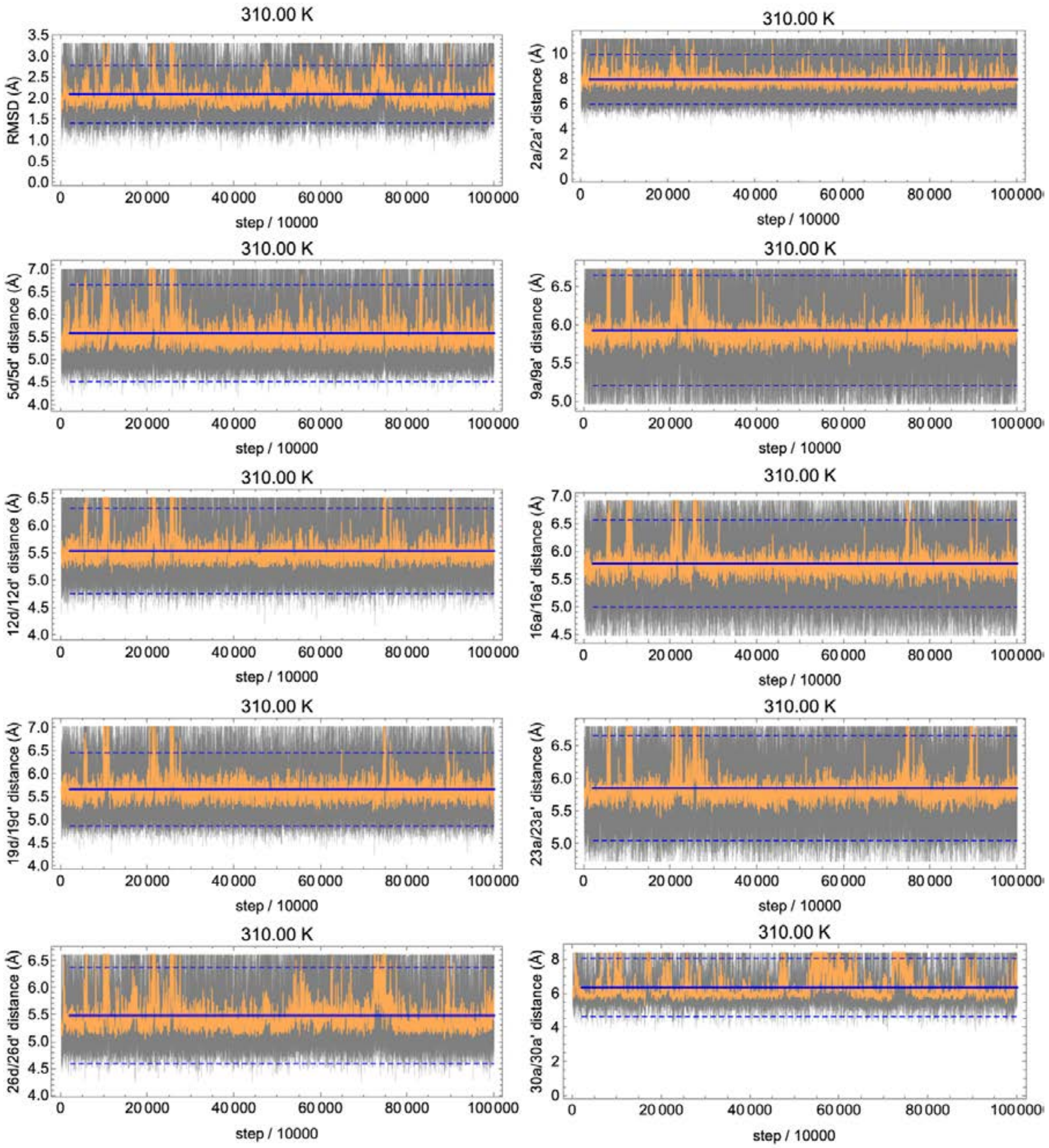

Measure at 310.00 K	Average (Å)	Standard Deviation (Å)	Slope (Å / μs)
RMSD	2.10731	0.683601	−0.00067507
5d/5d' distance	5.58984	1.07055	−0.0880501
12d/12d' distance	5.54625	0.78249	−0.0569181
19d/19d' distance	5.6726	0.791998	−0.059297
26d/26d' distance	5.49243	0.896712	−0.03276
2a/2a' distance	7.98569	1.96634	−0.15091
9a/9a' distance	5.93533	0.719035	−0.0547216
16a/16a' distance	5.79127	0.786607	−0.0561287
23a/23a' distance	5.85898	0.803235	−0.0456795
30a/30a' distance	6.37451	1.69764	0.0834332
Core Distance	6.02743	1.35819	

Figure S290. Plots of RMSD and residue packing distances for T-REMD simulations of variant **34e/29g’-xx** at 310.00 K.

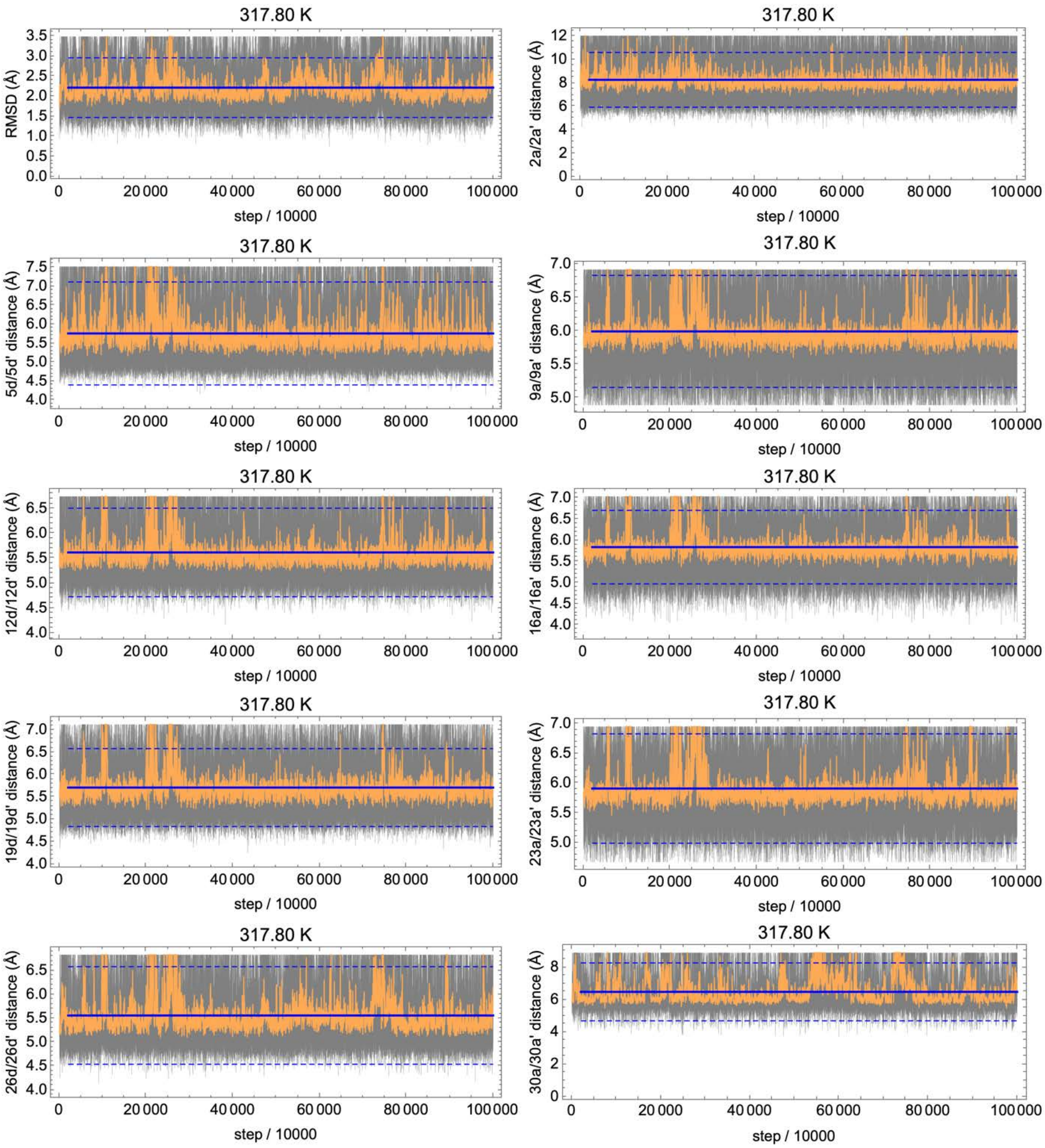

Measure at 317.80 K	Average (Å)	Standard Deviation (Å)	Slope (Å / μs)
RMSD	2.21326	0.742823	−0.0100861
5d/5d' distance	5.75524	1.35699	−0.113893
12d/12d' distance	5.6165	0.889277	−0.0601825
19d/19d' distance	5.70753	0.871539	−0.0565436
26d/26d' distance	5.55696	1.02106	−0.0404163
2a/2a' distance	8.26113	2.33187	−0.181102
9a/9a' distance	5.99289	0.837332	−0.0629568
16a/16a' distance	5.83596	0.868534	−0.054072
23a/23a' distance	5.90846	0.917257	−0.042238
30a/30a' distance	6.47342	1.78999	0.0618749
Core Distance	6.12312	1.53106	

Figure S291. Plots of RMSD and residue packing distances for T-REMD simulations of variant **34e/29g'-xx** at 317.80 K.

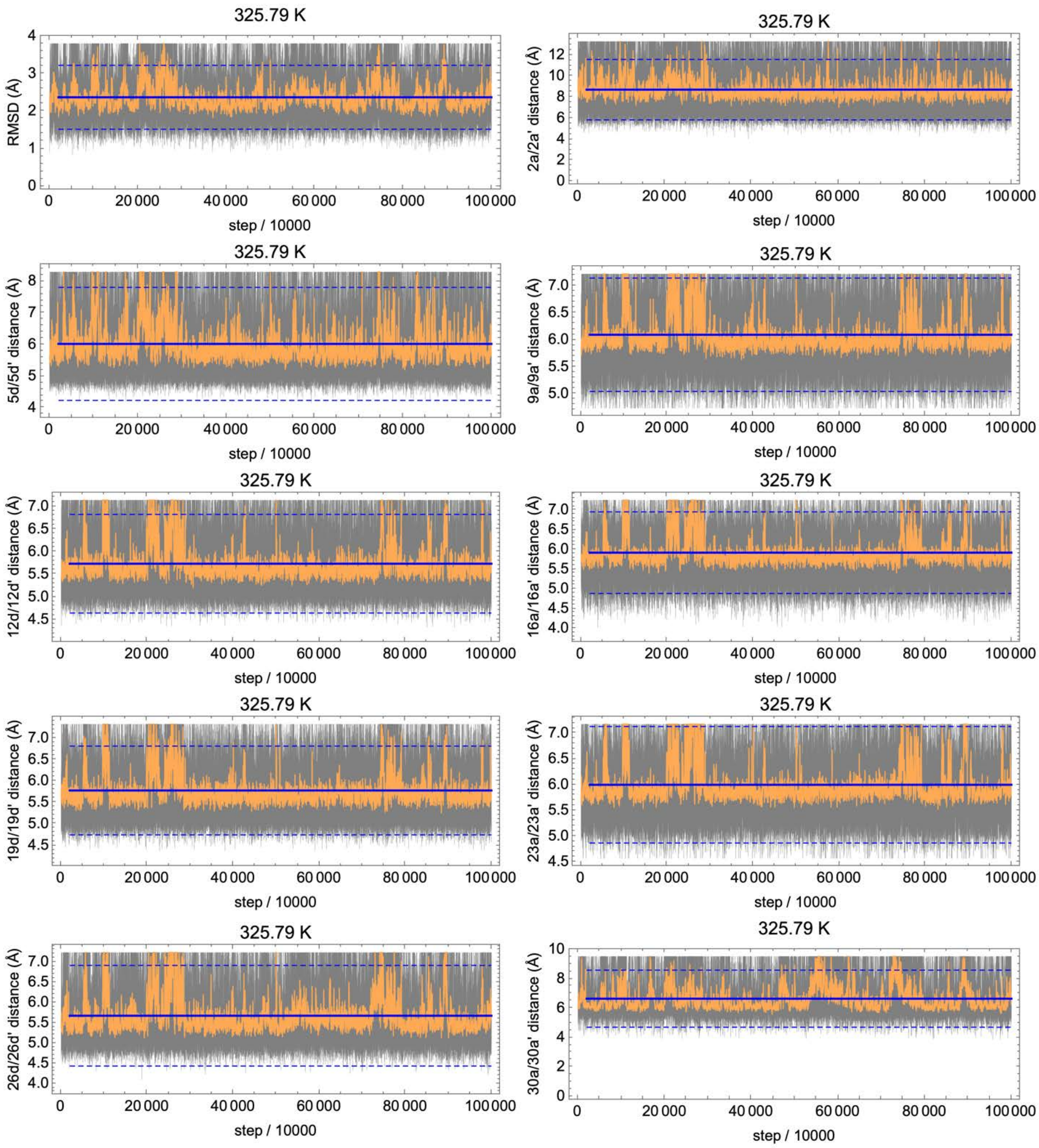

Measure at 325.79 K	Average (Å)	Standard Deviation (Å)	Slope (Å / μs)
RMSD	2.36681	0.851519	−0.028757
5d/5d' distance	6.015	1.78512	−0.159842
12d/12d' distance	5.7326	1.09076	−0.0789878
19d/19d' distance	5.77741	1.03473	−0.0679174
26d/26d' distance	5.67104	1.24178	−0.0480136
2a/2a' distance	8.69644	2.8703	−0.261706
9a/9a' distance	6.09095	1.04948	−0.0836802
16a/16a' distance	5.91585	1.03426	−0.0690534
23a/23a' distance	5.99688	1.13142	−0.0500617
30a/30a' distance	6.63727	1.94021	0.083103
Core Distance	6.28149	1.81511	

Figure S292. Plots of RMSD and residue packing distances for T-REMD simulations of variant **34e/29g’-xx** at 325.79 K.

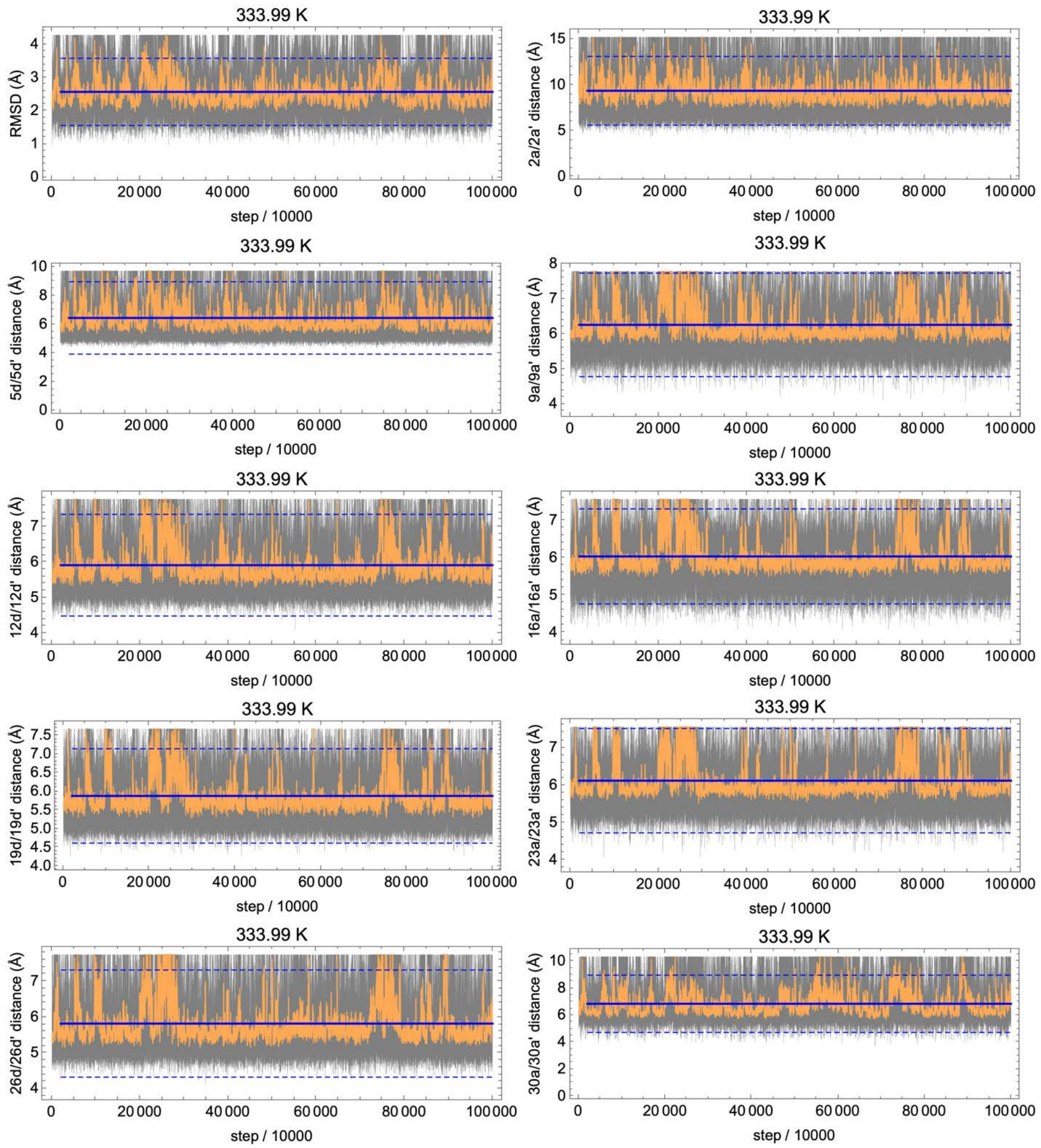

Measure at 333.99 K	Average (Å)	Standard Deviation (Å)	Slope (Å / μs)
RMSD	2.57154	1.01143	−0.0505122
5d/5d' distance	6.44299	2.52282	−0.249466
12d/12d' distance	5.91062	1.43028	−0.0867064
19d/19d' distance	5.87807	1.26344	−0.0673652
26d/26d' distance	5.81771	1.49549	−0.0509912
2a/2a' distance	9.3599	3.75116	−0.369508
9a/9a' distance	6.25666	1.47585	−0.0856912
16a/16a' distance	6.03959	1.27407	−0.0644739
23a/23a' distance	6.11704	1.39135	−0.0409321
30a/30a' distance	6.84246	2.11875	0.0810051
Core Distance	6.51834	2.27192	

Figure S293. Plots of RMSD and residue packing distances for T-REMD simulations of variant **34e/29g'-xx** at 333.99 K.

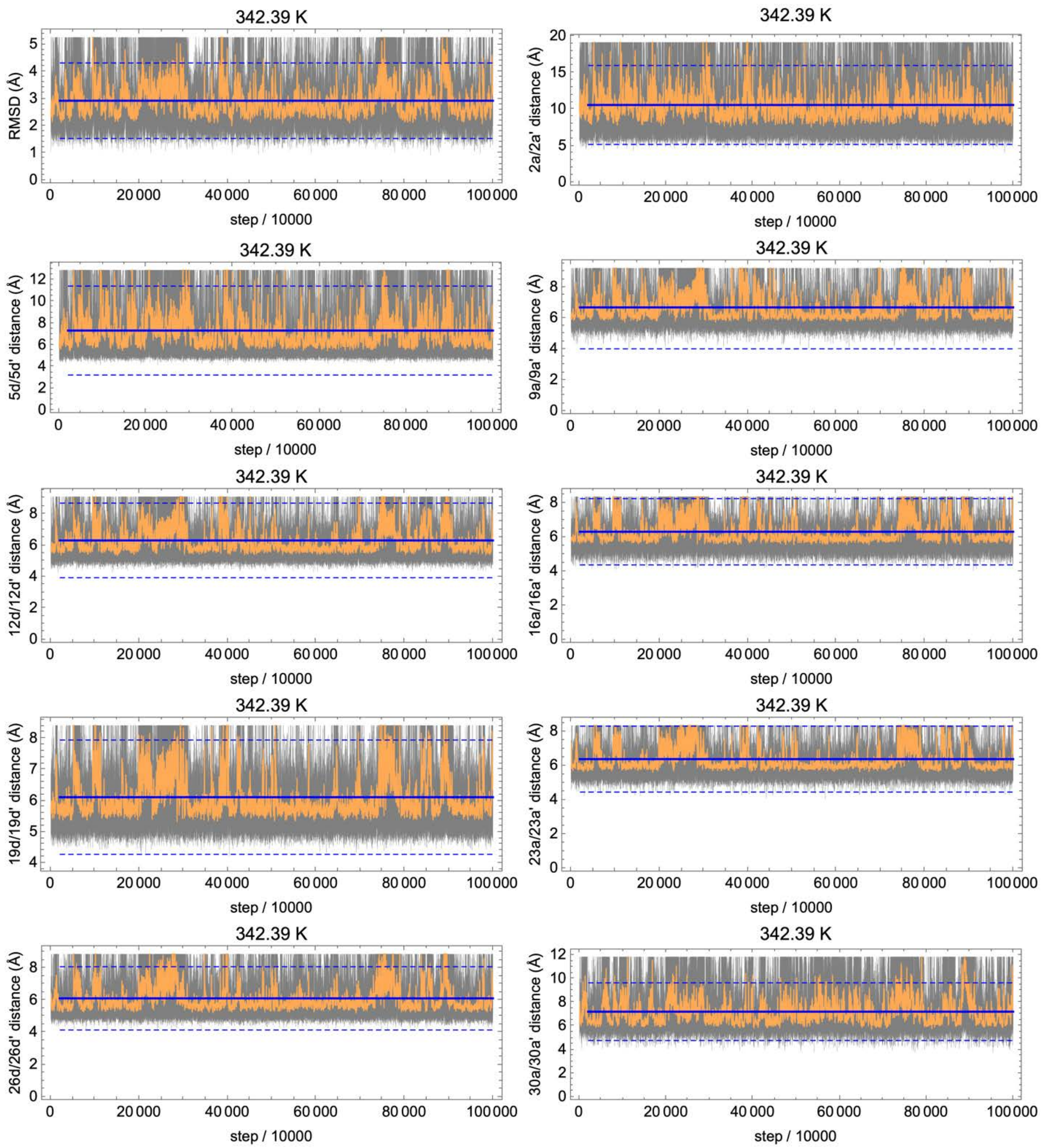

Measure at 342.39 K	Average (Å)	Standard Deviation (Å)	Slope (Å / μs)
RMSD	2.92254	1.39042	−0.0513241
5d/5d' distance	7.32168	4.09116	−0.307087
12d/12d' distance	6.30177	2.37229	−0.0648427
19d/19d' distance	6.10831	1.83523	−0.0399173
26d/26d' distance	6.10482	1.96232	−0.0323579
2a/2a' distance	10.5761	5.39893	−0.489185
9a/9a' distance	6.68109	2.6767	−0.0658358
16a/16a' distance	6.30216	1.93872	−0.0457285
23a/23a' distance	6.36456	1.9236	−0.00791967
30a/30a' distance	7.20108	2.4372	0.111026
Core Distance	6.99573	3.25413	

Figure S294. Plots of RMSD and residue packing distances for T-REMD simulations of variant **34e/29g'-xx** at 342.39 K.

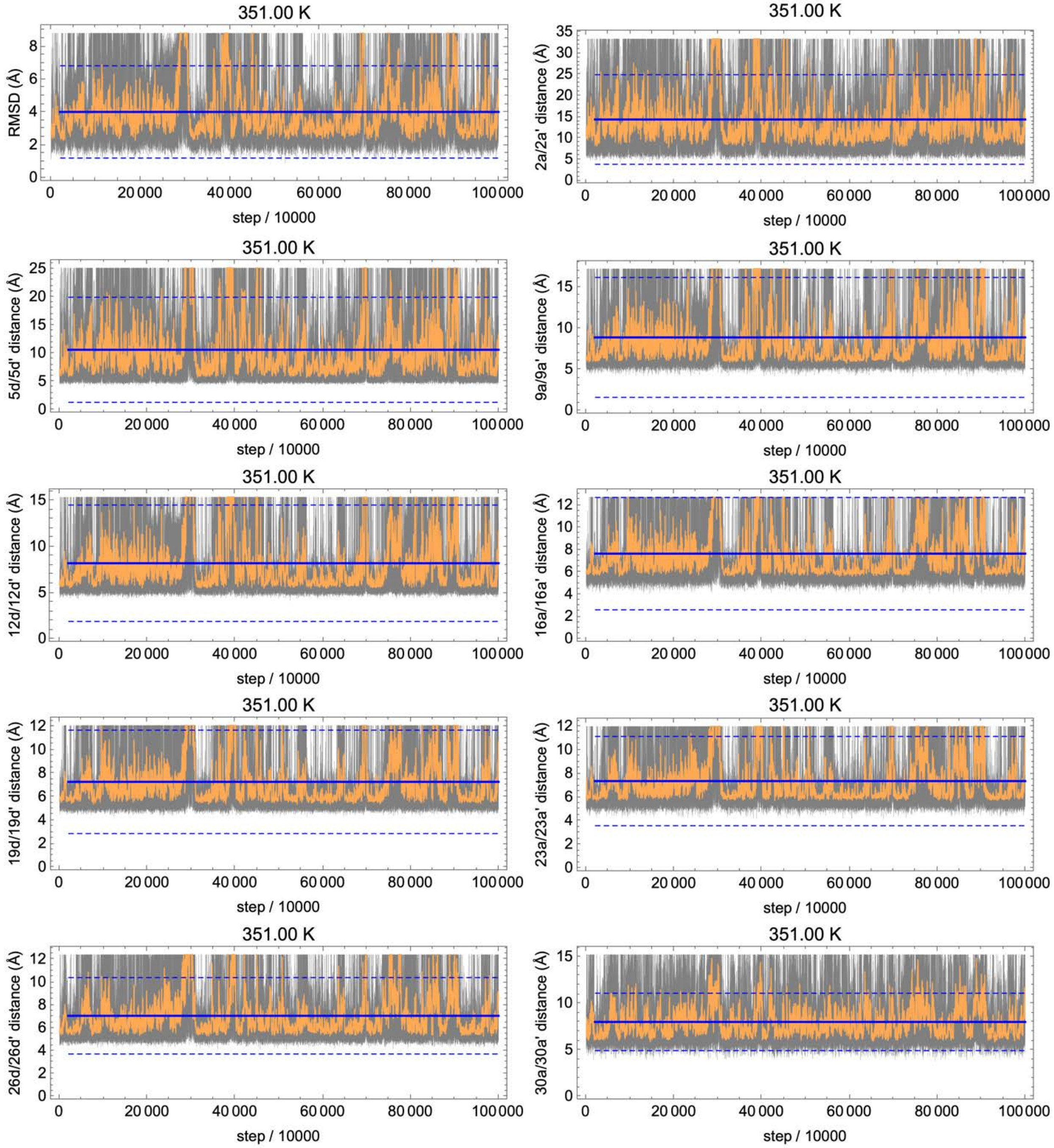

Measure at 351.00 K	Average (Å)	Standard Deviation (Å)	Slope (Å / μs)
RMSD	4.01133	2.81635	−0.083749
5d/5d' distance	10.625	9.32004	−0.531753
12d/12d' distance	8.19875	6.31141	−0.146449
19d/19d' distance	7.26934	4.40186	−0.0801224
26d/26d' distance	7.06051	3.3462	−0.0158287
2a/2a' distance	14.3805	10.5393	−0.72135
9a/9a' distance	8.88093	7.27401	−0.151737
16a/16a' distance	7.64457	5.04112	−0.105583
23a/23a' distance	7.36541	3.77797	−0.00249262
30a/30a' distance	8.00302	3.08482	0.163397
Core Distance	8.82534	6.78797	

Figure S295. Plots of RMSD and residue packing distances for T-REMD simulations of variant **34e/29g'-xx** at 351.00 K.

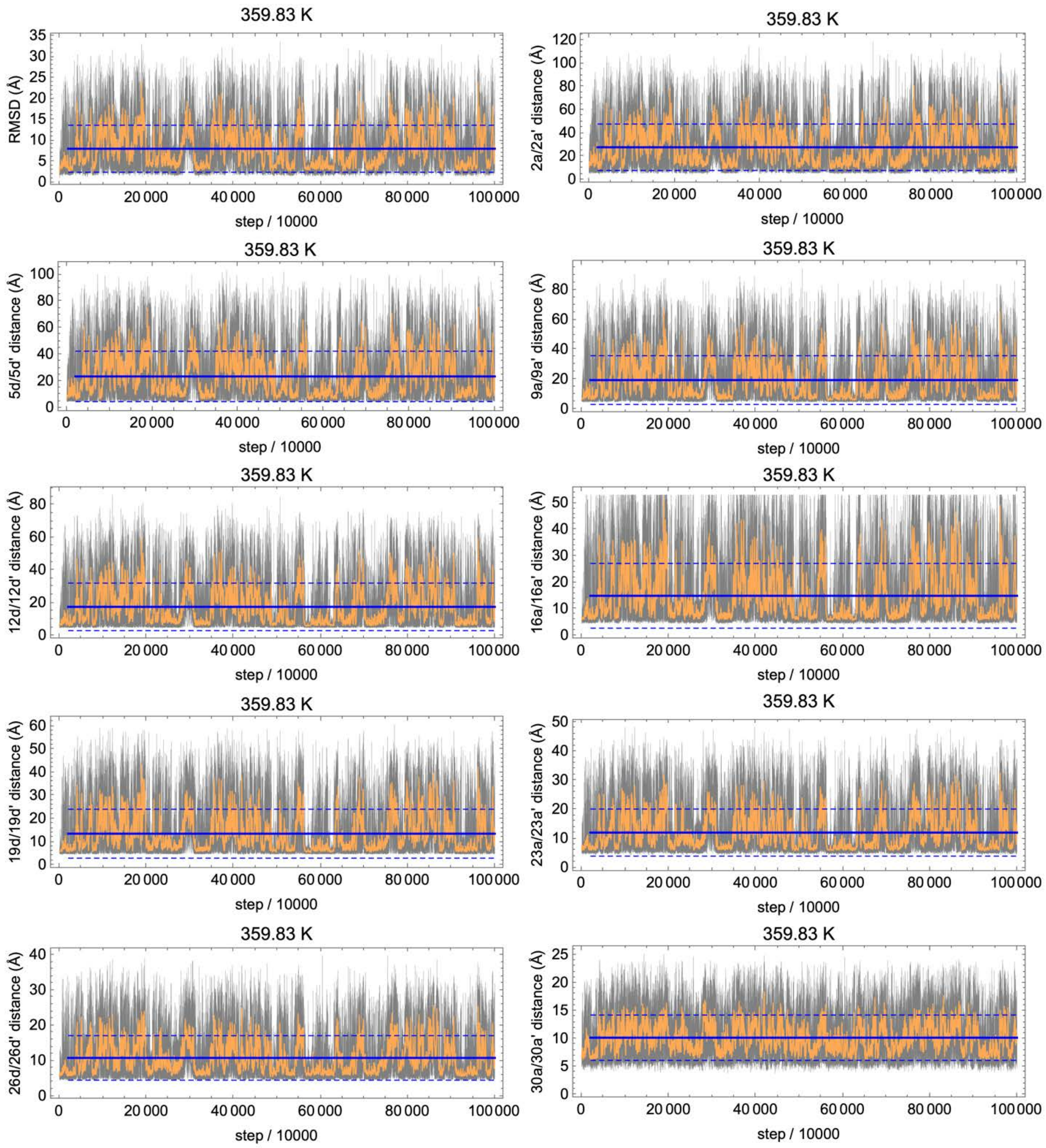

Measure at 359.83 K	Average (Å)	Standard Deviation (Å)	Slope (Å / μs)
RMSD	7.99471	5.62092	−0.290066
5d/5d' distance	23.4574	18.9278	−1.12016
12d/12d' distance	17.5037	14.5421	−0.658979
19d/19d' distance	13.5067	10.5662	−0.444975
26d/26d' distance	10.8187	6.31058	−0.401379
2a/2a' distance	27.8165	19.9644	−1.12786
9a/9a' distance	19.4757	16.3307	−0.777954
16a/16a' distance	14.9673	12.2346	−0.579039
23a/23a' distance	12.067	8.0867	−0.375271
30a/30a' distance	10.1768	4.06076	−0.194039
Core Distance	16.6433	14.5579	

Figure S296. Plots of RMSD and residue packing distances for T-REMD simulations of variant **34e/29g’-xx** at 359.83 K.

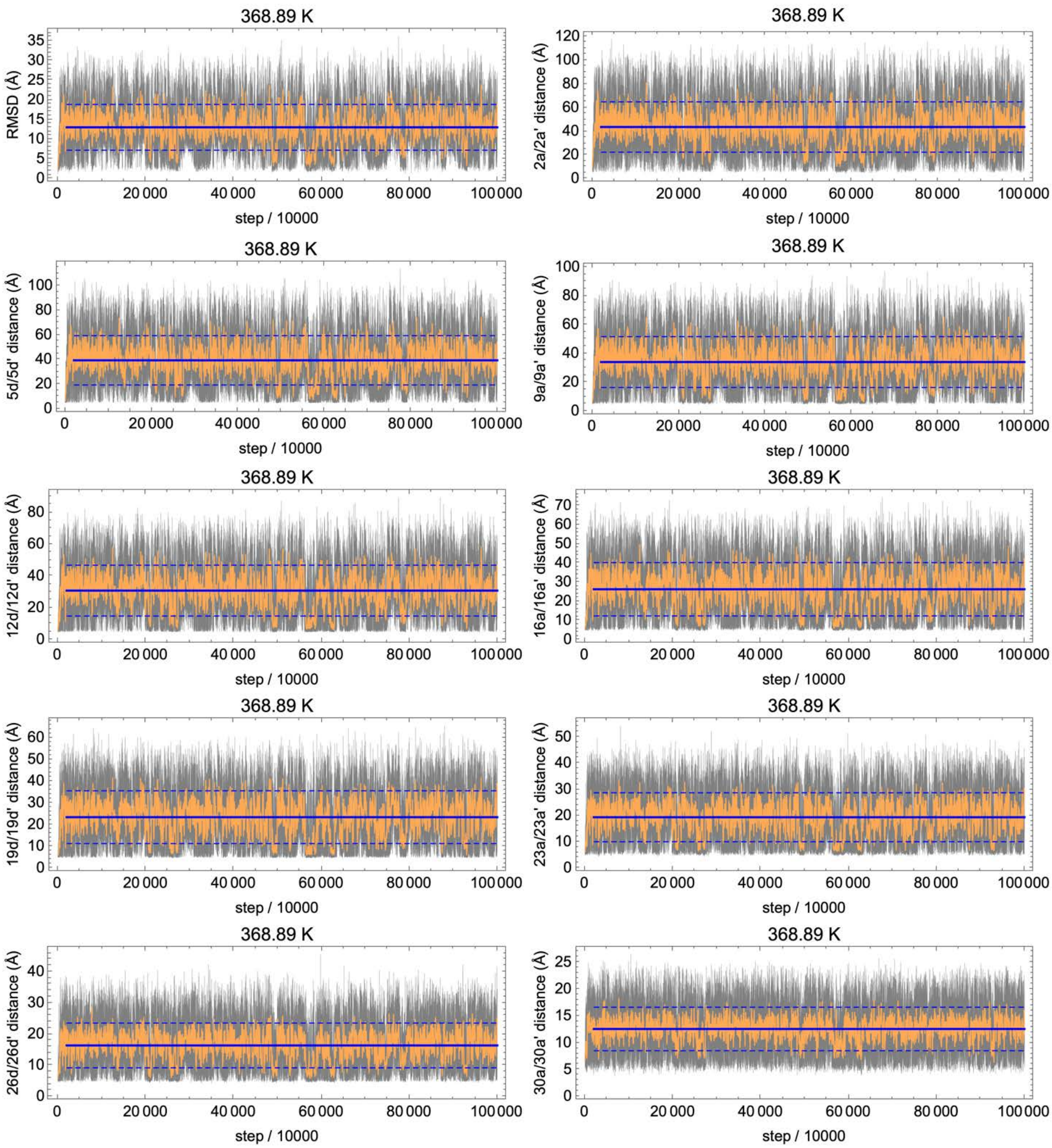

Measure at 368.89 K	Average (Å)	Standard Deviation (Å)	Slope (Å / μs)
RMSD	13.0047	5.82905	−0.343841
5d/5d' distance	39.2673	20.0465	−1.3649
12d/12d' distance	30.7651	15.9755	−1.02956
19d/19d' distance	23.3864	12.1563	−0.602484
26d/26d' distance	16.3389	7.17123	−0.302327
2a/2a' distance	43.571	21.2254	−1.32334
9a/9a' distance	33.9603	17.6703	−1.12682
16a/16a' distance	26.2548	13.9172	−0.818018
23a/23a' distance	19.3552	9.34347	−0.417789
30a/30a' distance	12.5724	4.06231	0.00673433
Core Distance	27.2746	17.6302	

Figure S297. Plots of RMSD and residue packing distances for T-REMD simulations of variant **34e/29g'-xx** at 368.89 K.

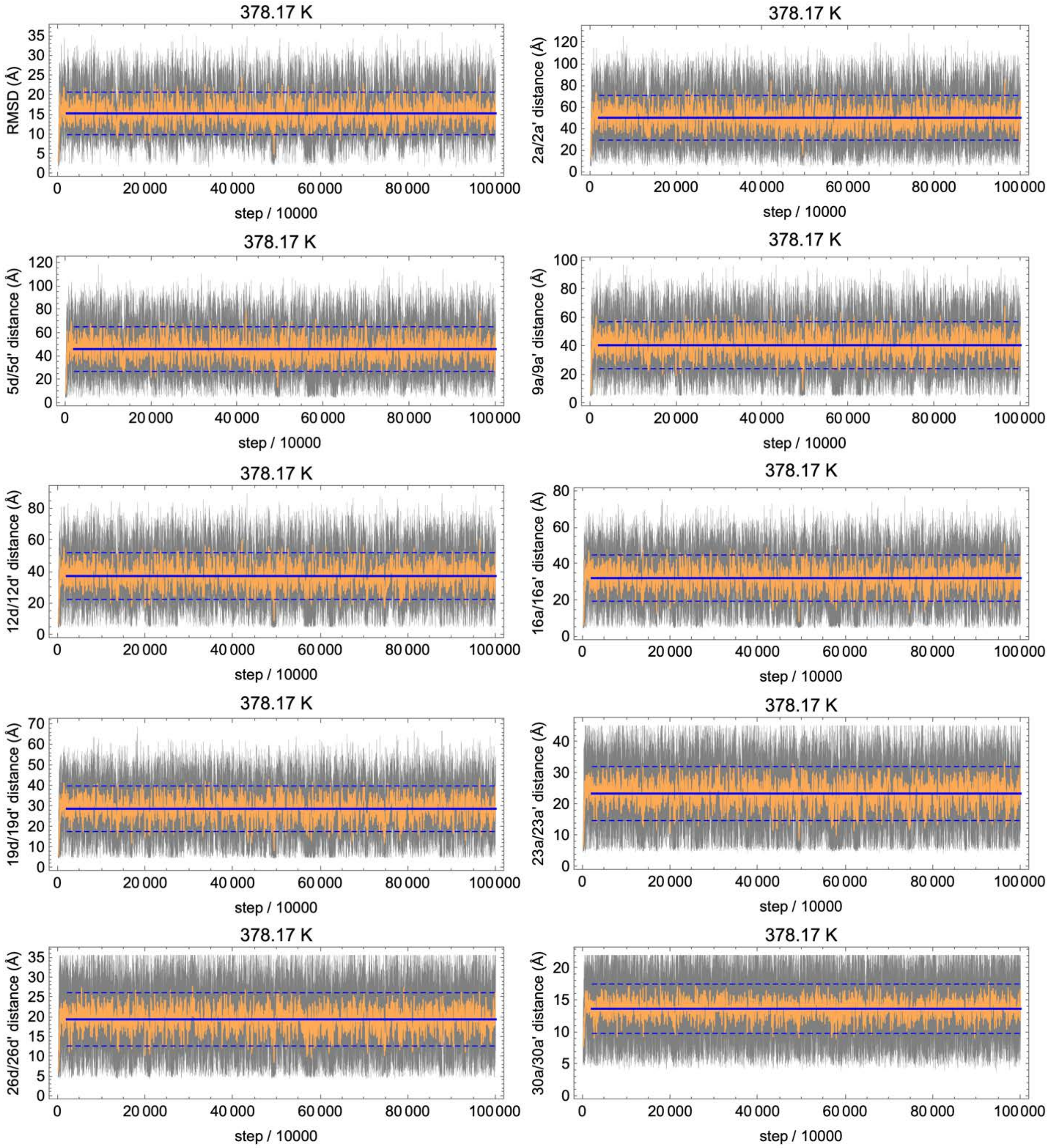

Measure at 378.17 K	Average (Å)	Standard Deviation (Å)	Slope (Å / μs)
RMSD	15.3642	5.42436	−0.141672
5d/5d' distance	46.3345	19.0964	−0.51102
12d/12d' distance	37.387	14.7772	−0.426928
19d/19d' distance	28.8765	11.1022	−0.384523
26d/26d' distance	19.491	6.73353	−0.208068
2a/2a' distance	50.4454	20.7102	−0.428911
9a/9a' distance	40.8796	16.4944	−0.457217
16a/16a' distance	32.3677	12.7281	−0.470677
23a/23a' distance	23.501	8.65714	−0.289825
30a/30a' distance	13.7031	3.85759	0.0131319
Core Distance	32.554	18.0389	

Figure S298. Plots of RMSD and residue packing distances for T-REMD simulations of variant **34e/29g’-xx** at 378.17 K.

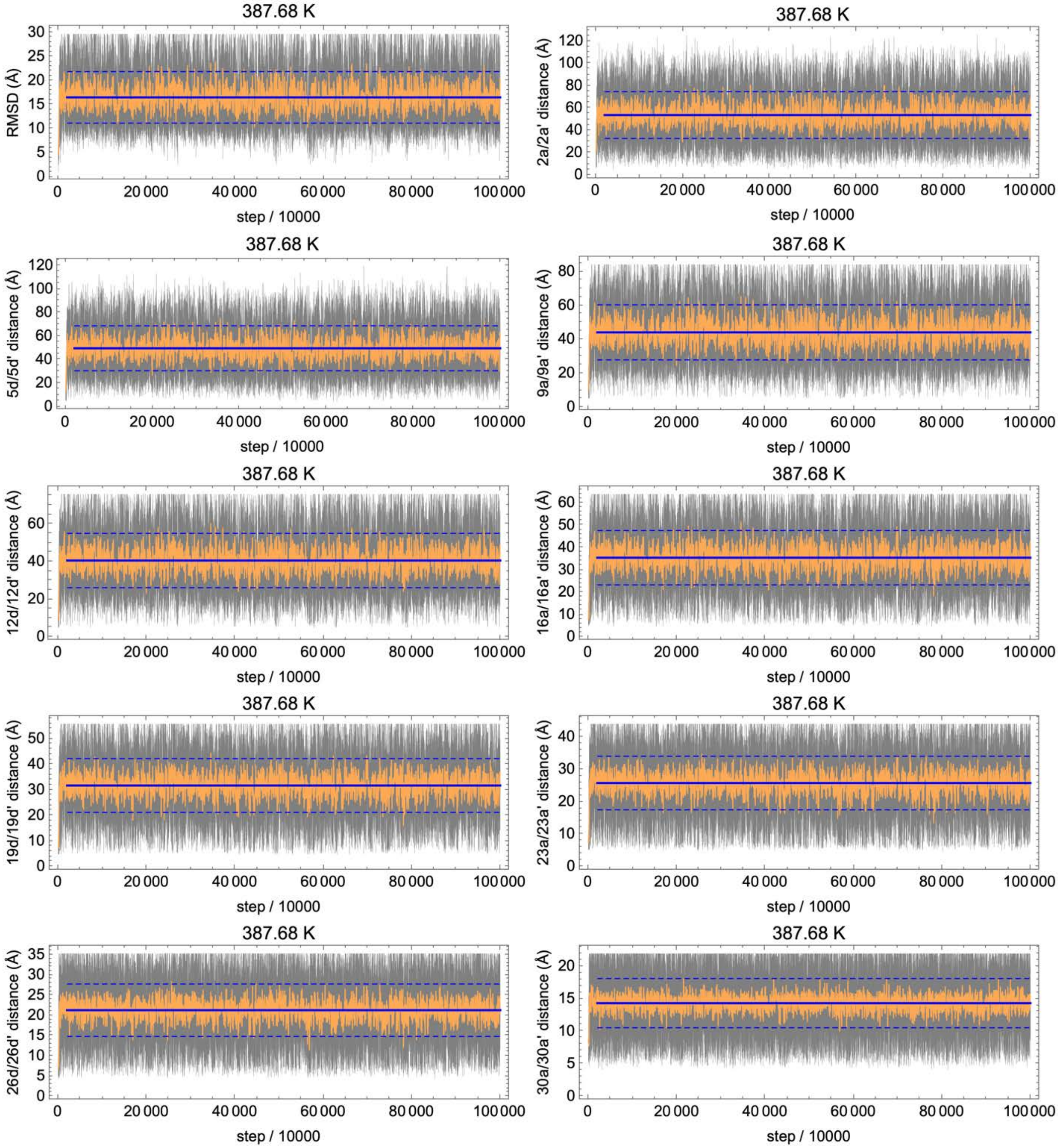

Measure at 387.68 K	Average (Å)	Standard Deviation (Å)	Slope (Å / μs)
RMSD	16.5045	5.35555	−0.0268905
5d/5d' distance	49.5834	19.1148	−0.0329036
12d/12d' distance	40.5326	14.4294	−0.0619455
19d/19d' distance	31.7126	10.5182	−0.203437
26d/26d' distance	21.28	6.48206	−0.153319
2a/2a' distance	53.6031	20.9515	0.0178774
9a/9a' distance	44.0964	16.2495	−0.0500108
16a/16a' distance	35.4048	12.1531	−0.199239
23a/23a' distance	25.7658	8.26494	−0.188696
30a/30a' distance	14.3169	3.78483	0.00263626
Core Distance	35.144	18.3901	

Figure S299. Plots of RMSD and residue packing distances for T-REMD simulations of variant **34e/29g’-xx** at 387.68 K.

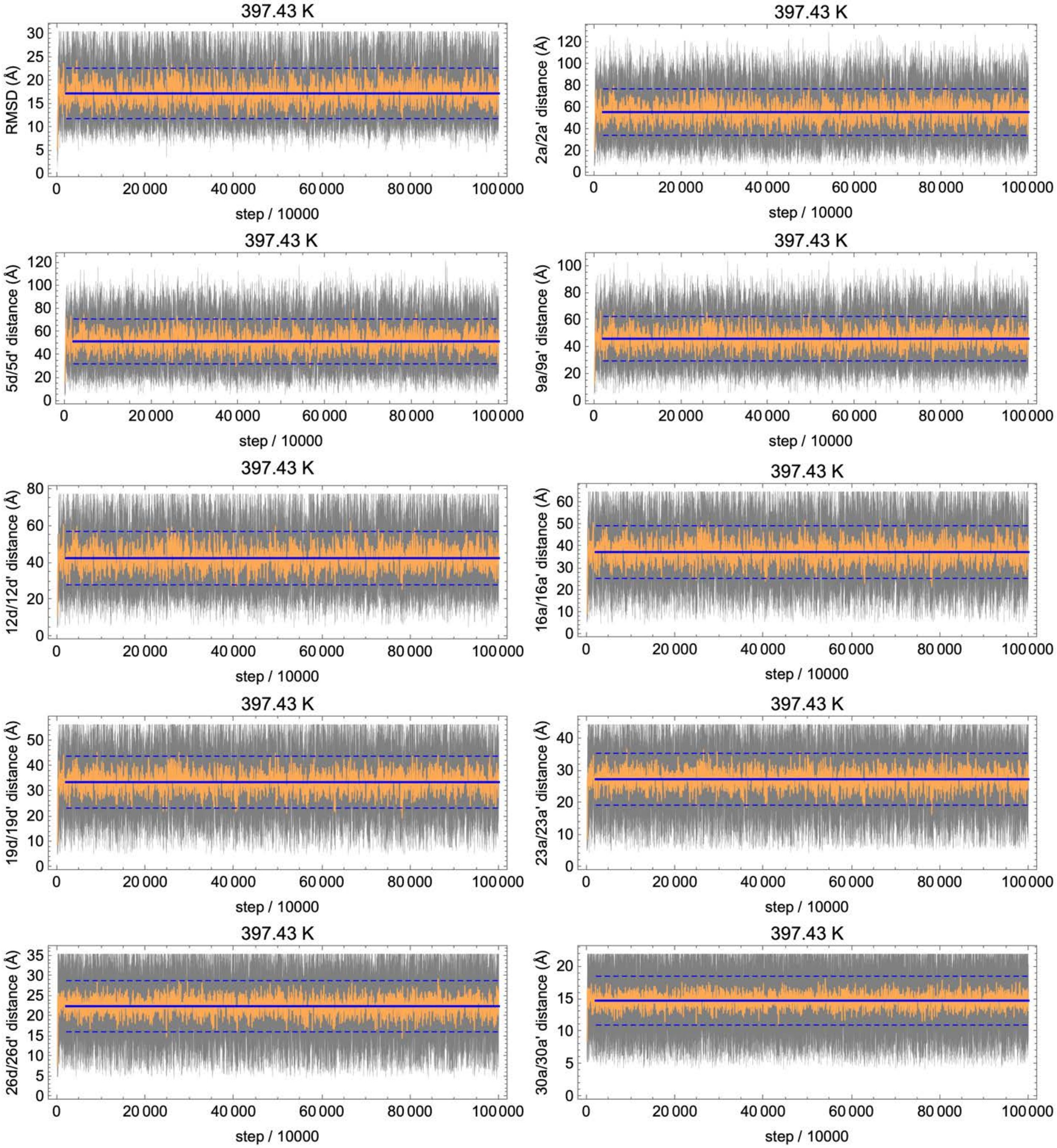

Measure at 397.43 K	Average (Å)	Standard Deviation (Å)	Slope (Å / μs)
RMSD	17.2804	5.40736	0.0476198
5d/5d' distance	51.6847	19.4451	0.230912
12d/12d' distance	42.5982	14.5184	0.168105
19d/19d' distance	33.595	10.2801	−0.0268044
26d/26d' distance	22.5062	6.37669	−0.0675015
2a/2a' distance	55.7215	21.3725	0.271898
9a/9a' distance	46.1716	16.4631	0.171851
16a/16a' distance	37.3701	11.9881	0.0381388
23a/23a' distance	27.3609	8.08095	−0.0718487
30a/30a' distance	14.7569	3.75542	0.0317749
Core Distance	36.8628	18.7885	

Figure S300. Plots of RMSD and residue packing distances for T-REMD simulations of variant **34e/29g'-xx** at 397.43 K.

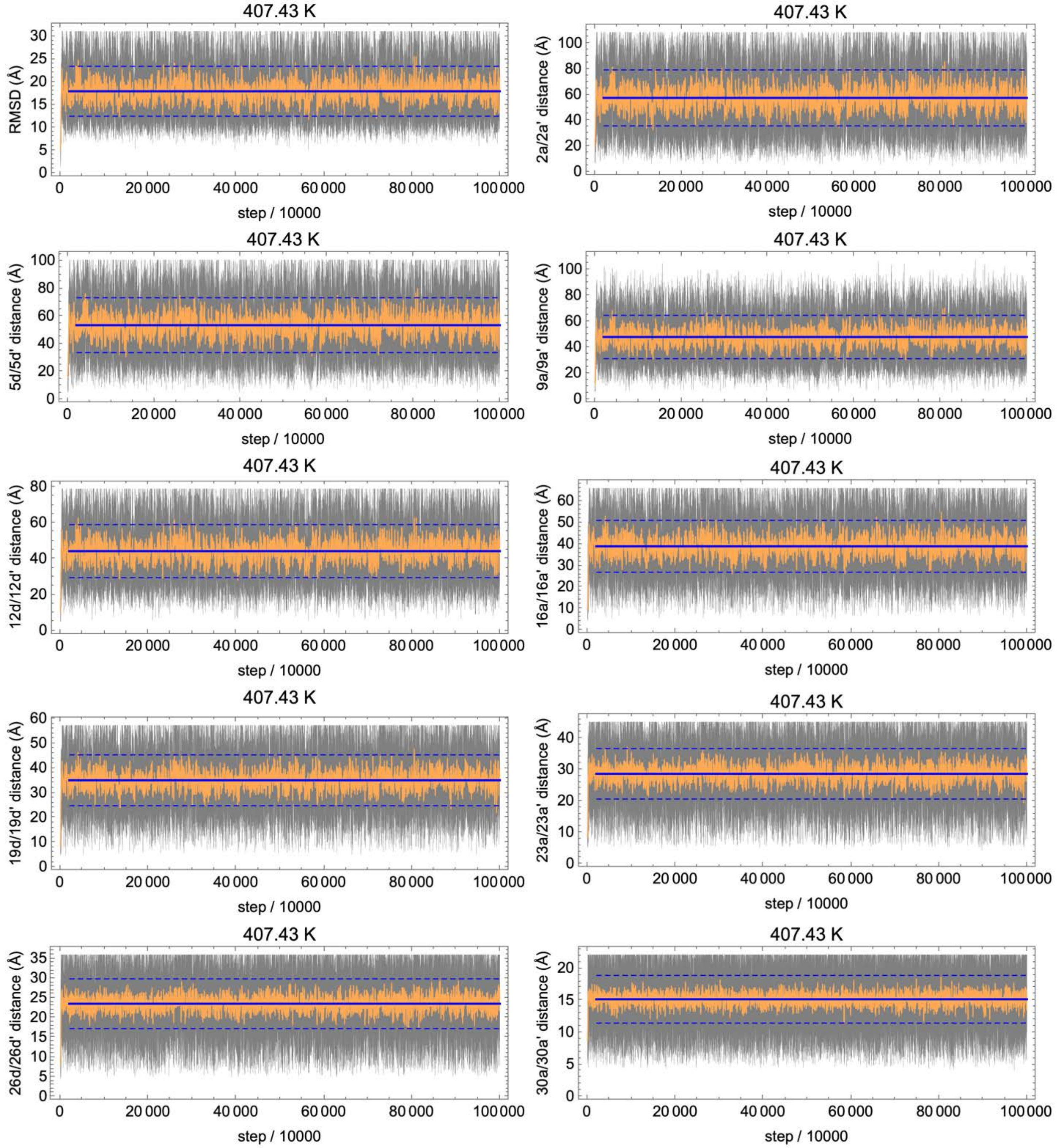

Measure at 407.43 K	Average (Å)	Standard Deviation (Å)	Slope (Å / μs)
RMSD	17.9391	5.47387	0.160509
5d/5d' distance	53.4872	19.7939	0.606348
12d/12d' distance	44.2622	14.7701	0.503376
19d/19d' distance	35.0715	10.2674	0.257052
26d/26d' distance	23.5403	6.30484	0.0610794
2a/2a' distance	57.519	21.7955	0.581506
9a/9a' distance	47.9073	16.7508	0.589815
16a/16a' distance	38.9364	12.051	0.38039
23a/23a' distance	28.6207	8.02133	0.0604987
30a/30a' distance	15.1819	3.7208	0.0222724
Core Distance	38.2807	19.2031	

Figure S301. Plots of RMSD and residue packing distances for T-REMD simulations of variant **34e/29g'-xx** at 407.43 K.

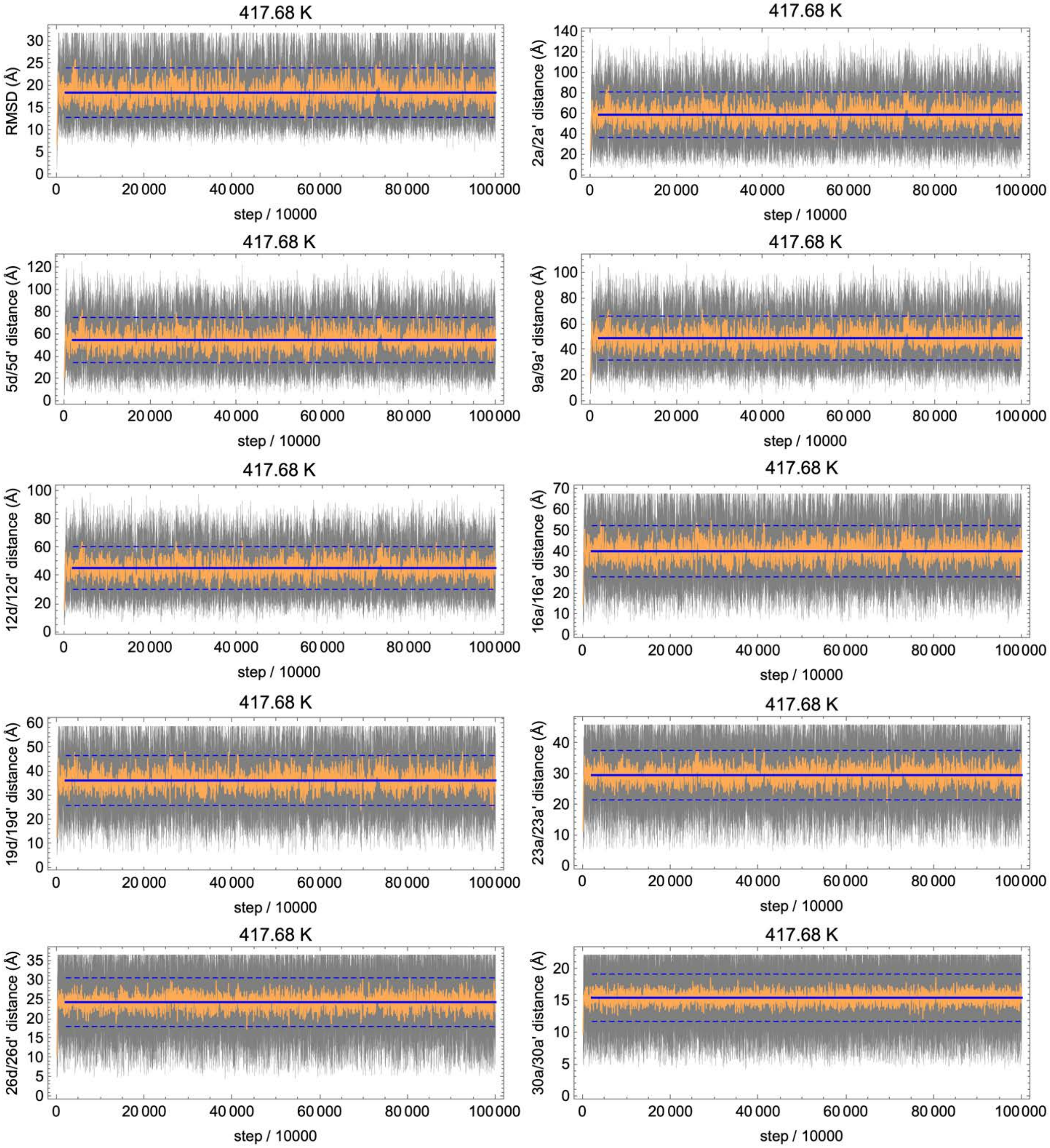

Measure at 417.68 K	Average (Å)	Standard Deviation (Å)	Slope (Å / μs)
RMSD	18.4755	5.57168	0.126526
5d/5d' distance	54.8688	20.2284	0.520619
12d/12d' distance	45.5552	15.1135	0.422374
19d/19d' distance	36.3075	10.3574	0.118537
26d/26d' distance	24.3686	6.29345	0.0309625
2a/2a' distance	59.0155	22.2584	0.528801
9a/9a' distance	49.2243	17.147	0.501323
16a/16a' distance	40.2045	12.2412	0.243745
23a/23a' distance	29.6691	8.05596	0.0422213
30a/30a' distance	15.4961	3.71252	0.018381
Core Distance	39.4122	19.6162	

Figure S302. Plots of RMSD and residue packing distances for T-REMD simulations of variant **34e/29g'-xx** at 417.68 K.

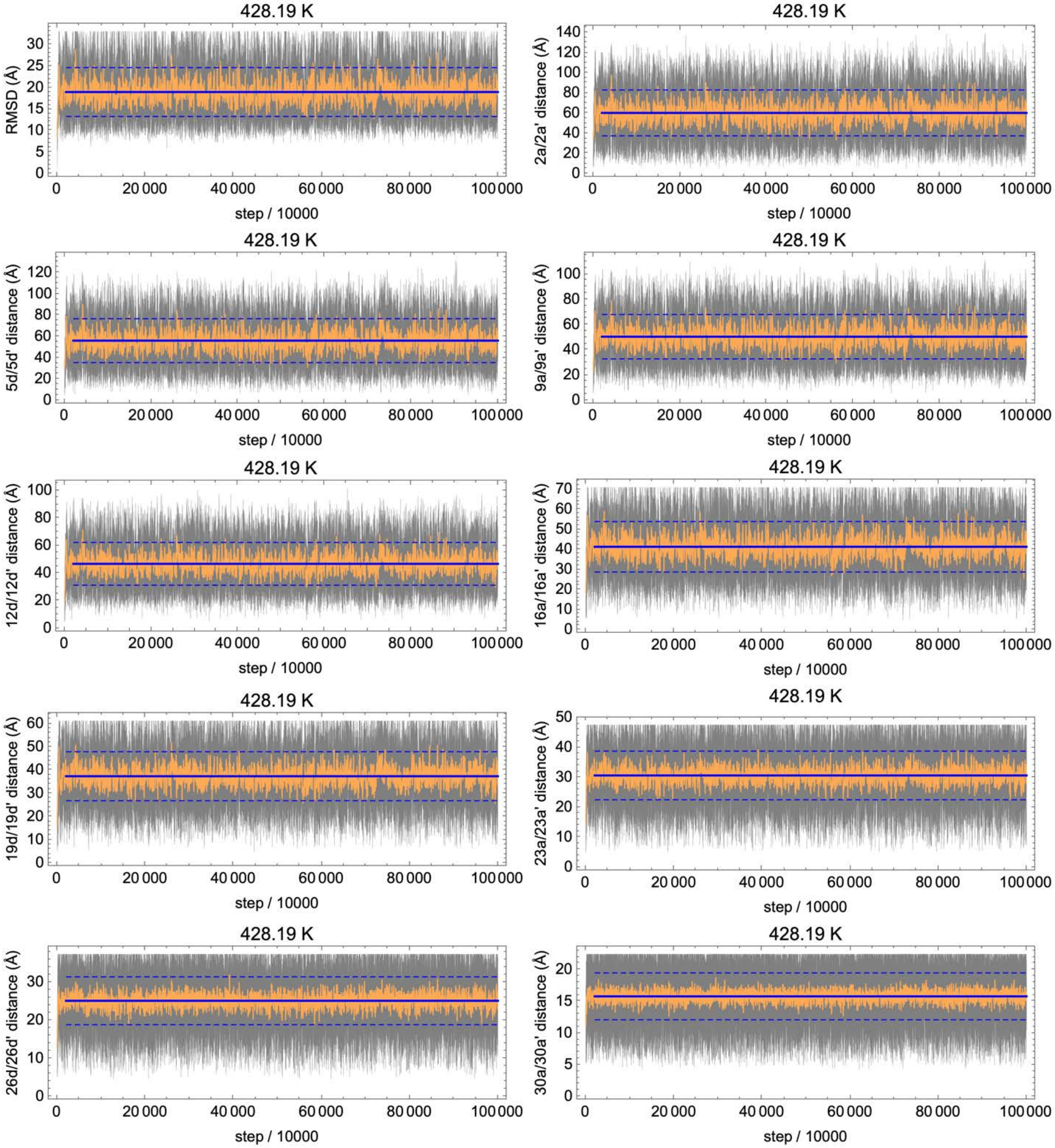

Measure at 428.19 K	Average (Å)	Standard Deviation (Å)	Slope (Å / μs)
RMSD	18.9362	5.67029	0.0971304
5d/5d' distance	56.0078	20.6607	0.370072
12d/12d' distance	46.6834	15.4968	0.286569
19d/19d' distance	37.3528	10.5439	0.167845
26d/26d' distance	25.0978	6.27467	0.0539244
2a/2a' distance	60.1655	22.7388	0.353998
9a/9a' distance	50.3556	17.5912	0.349659
16a/16a' distance	41.2709	12.5649	0.164091
23a/23a' distance	30.6203	8.12075	0.0645889
30a/30a' distance	15.7755	3.69201	0.0454867
Core Distance	40.37	20.0016	

Figure S303. Plots of RMSD and residue packing distances for T-REMD simulations of variant **34e/29g'-xx** at 428.19 K.

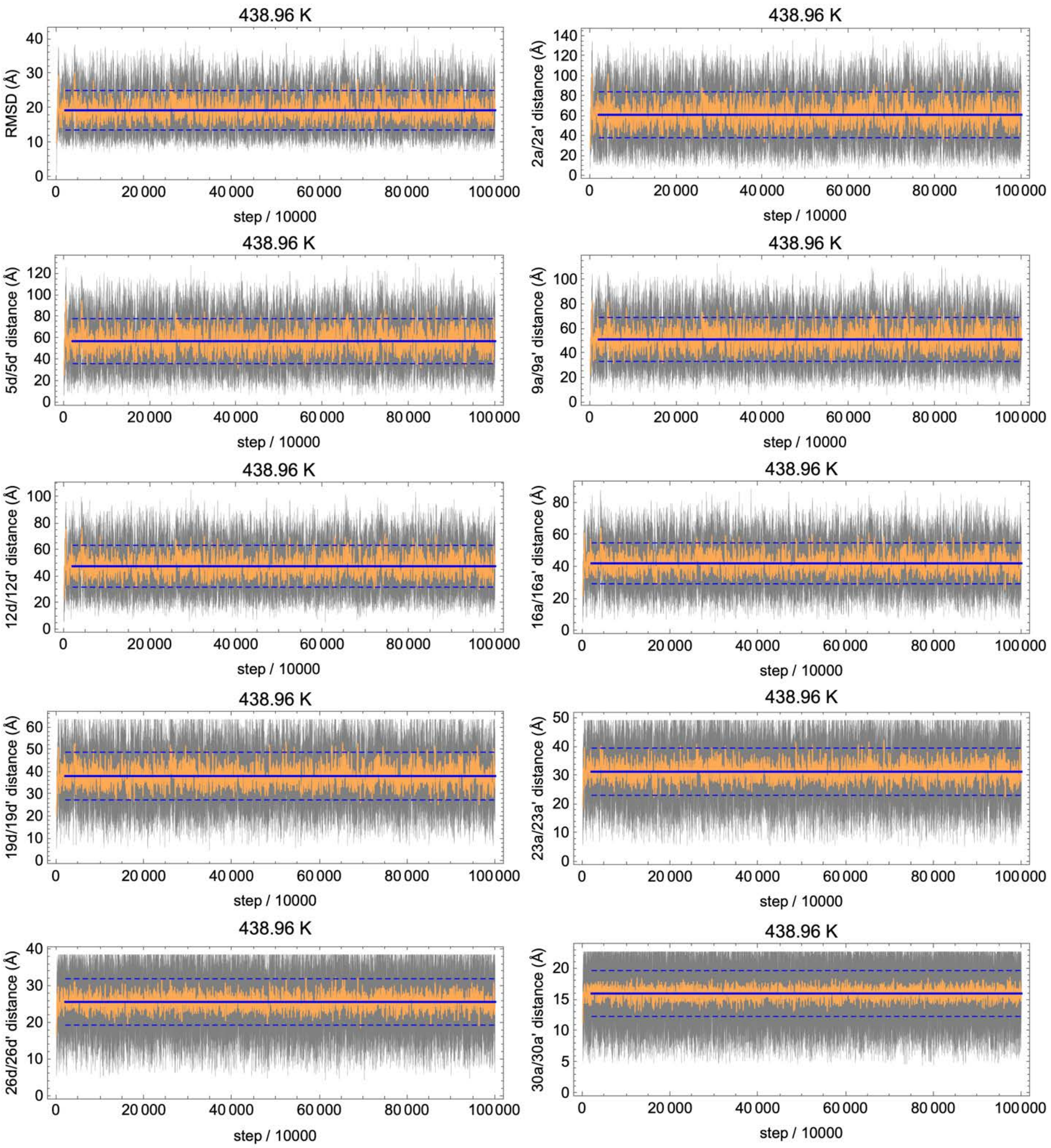

Measure at 438.96 K	Average (Å)	Standard Deviation (Å)	Slope (Å / μs)
RMSD	19.3711	5.72885	0.0896743
5d/5d' distance	57.1504	20.9809	0.443493
12d/12d' distance	47.6962	15.8092	0.288251
19d/19d' distance	38.2155	10.7365	0.131606
26d/26d' distance	25.694	6.31056	−0.00194453
2a/2a' distance	61.3013	23.0598	0.417116
9a/9a' distance	51.4543	17.9184	0.338352
16a/16a' distance	42.2385	12.8091	0.164245
23a/23a' distance	31.3737	8.23168	−0.00459915
30a/30a' distance	16.0174	3.67778	−0.0131206
Core Distance	41.2379	20.3589	

Figure S304. Plots of RMSD and residue packing distances for T-REMD simulations of variant **34e/29g'-xx** at 438.96 K.

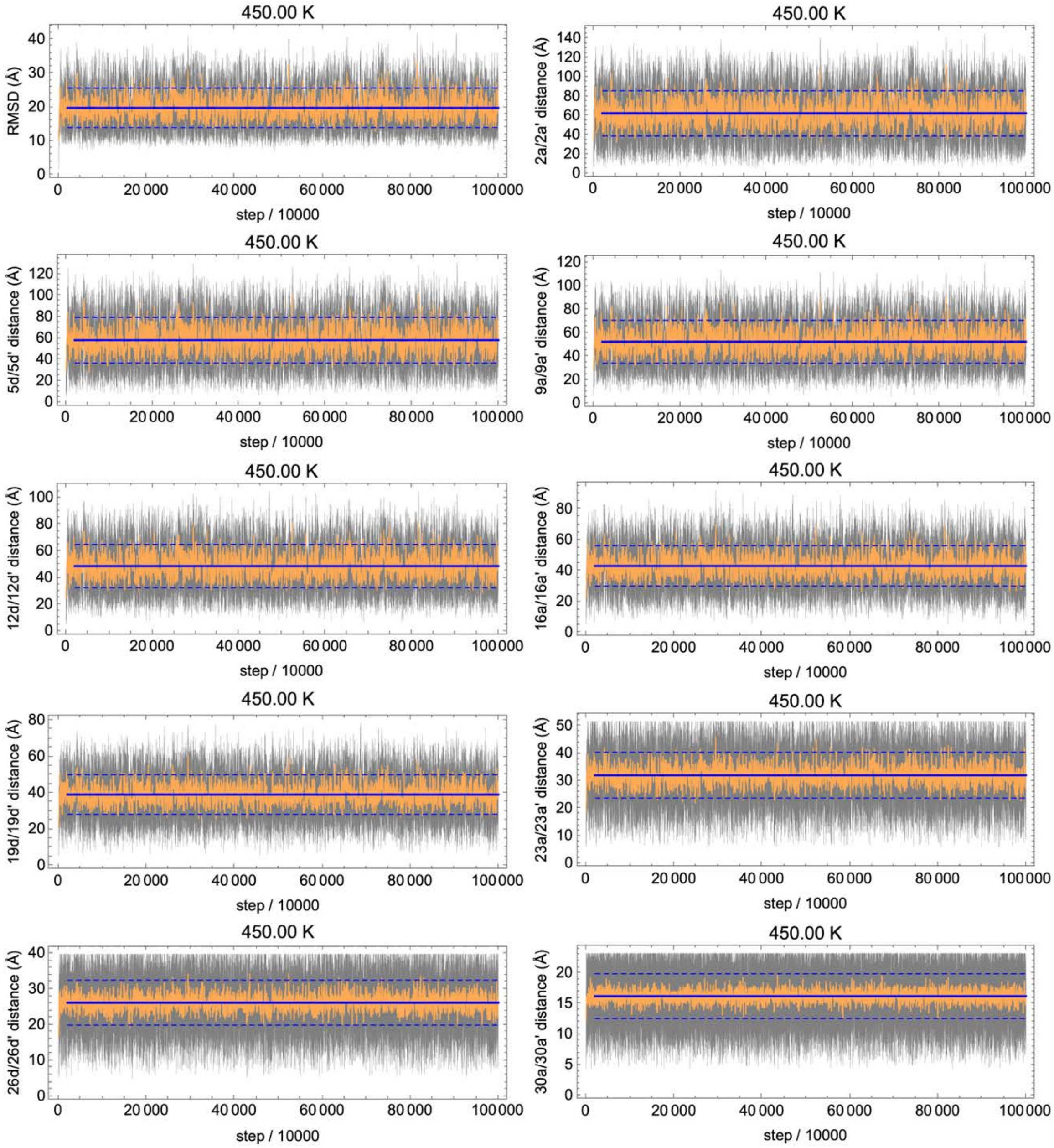

Measure at 450.00 K	Average (Å)	Standard Deviation (Å)	Slope (Å / μs)
RMSD	19.7587	5.83816	0.0895228
5d/5d' distance	58.1069	21.4326	0.377175
12d/12d' distance	48.6404	16.1703	0.290225
19d/19d' distance	39.0152	10.9361	0.17807
26d/26d' distance	26.1802	6.27718	0.0792445
2a/2a' distance	62.3246	23.4788	0.363969
9a/9a' distance	52.4224	18.3246	0.346687
16a/16a' distance	43.1233	13.1096	0.175137
23a/23a' distance	32.0307	8.30306	0.0937446
30a/30a' distance	16.2124	3.6473	0.03627
Core Distance	42.0062	20.7392	

Figure S305. Plots of RMSD and residue packing distances for T-REMD simulations of variant **34e/29g’-xx** at 450.00 K.

(return to Table of Contents)

s34e/29g'-x4x

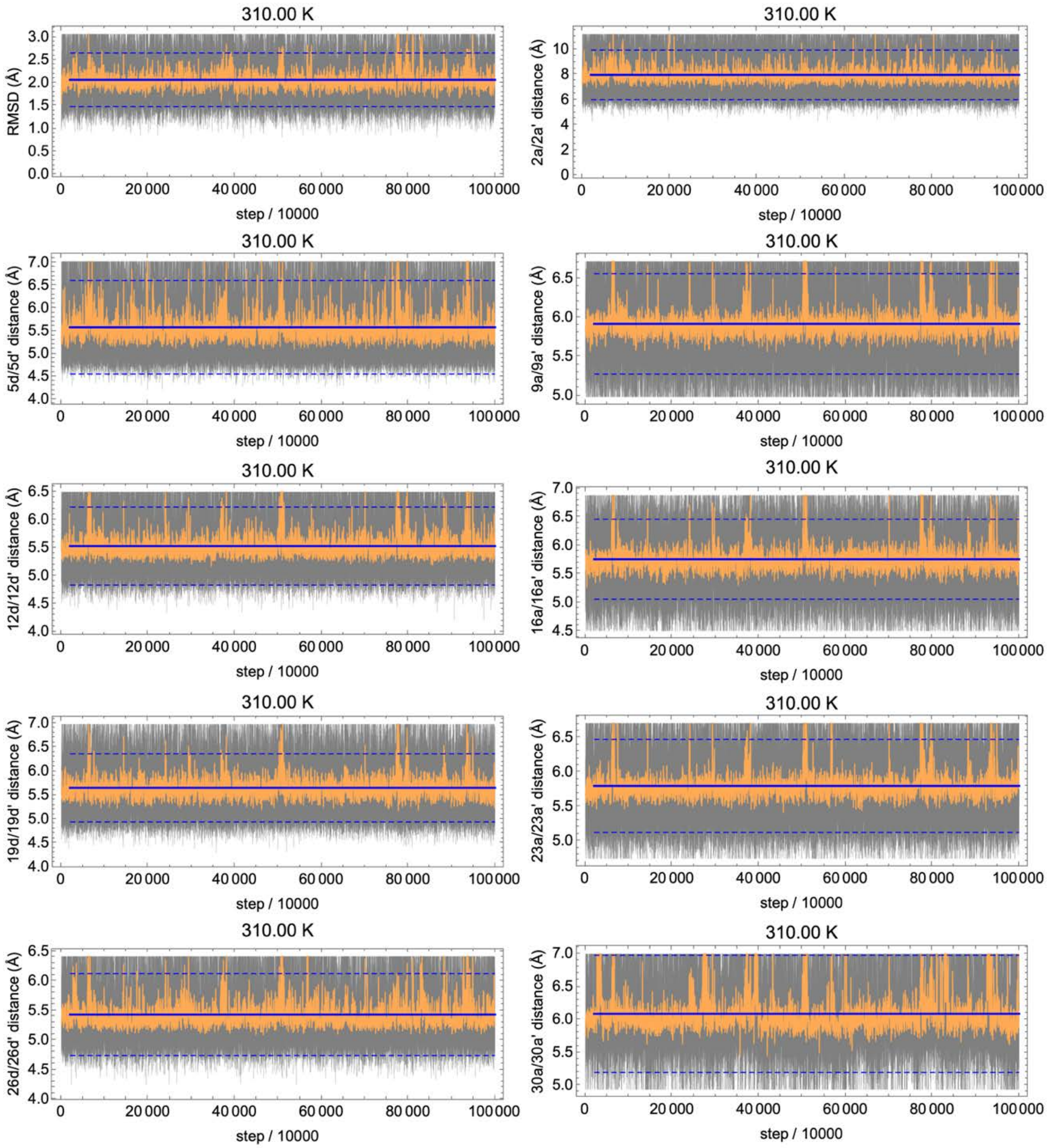

Measure at 310.00 K	Average (Å)	Standard Deviation (Å)	Slope (Å / μs)
RMSD	2.06348	0.585296	0.00928639
5d/5d' distance	5.57859	1.02714	−0.0124166
12d/12d' distance	5.52929	0.69689	0.0188641
19d/19d' distance	5.6515	0.710164	0.0118903
26d/26d' distance	5.43305	0.692275	0.0198203
2a/2a' distance	7.97533	1.96869	−0.0773834
9a/9a' distance	5.9163	0.643598	0.00814351
16a/16a' distance	5.75949	0.701862	0.0103912
23a/23a' distance	5.79807	0.676061	0.0197352
30a/30a' distance	6.08511	0.889624	−0.00282537
Core Distance	5.96964	1.22037	

Figure S306. Plots of RMSD and residue packing distances for T-REMD simulations of variant **s34e/29g'-x4x** at 310.00 K.

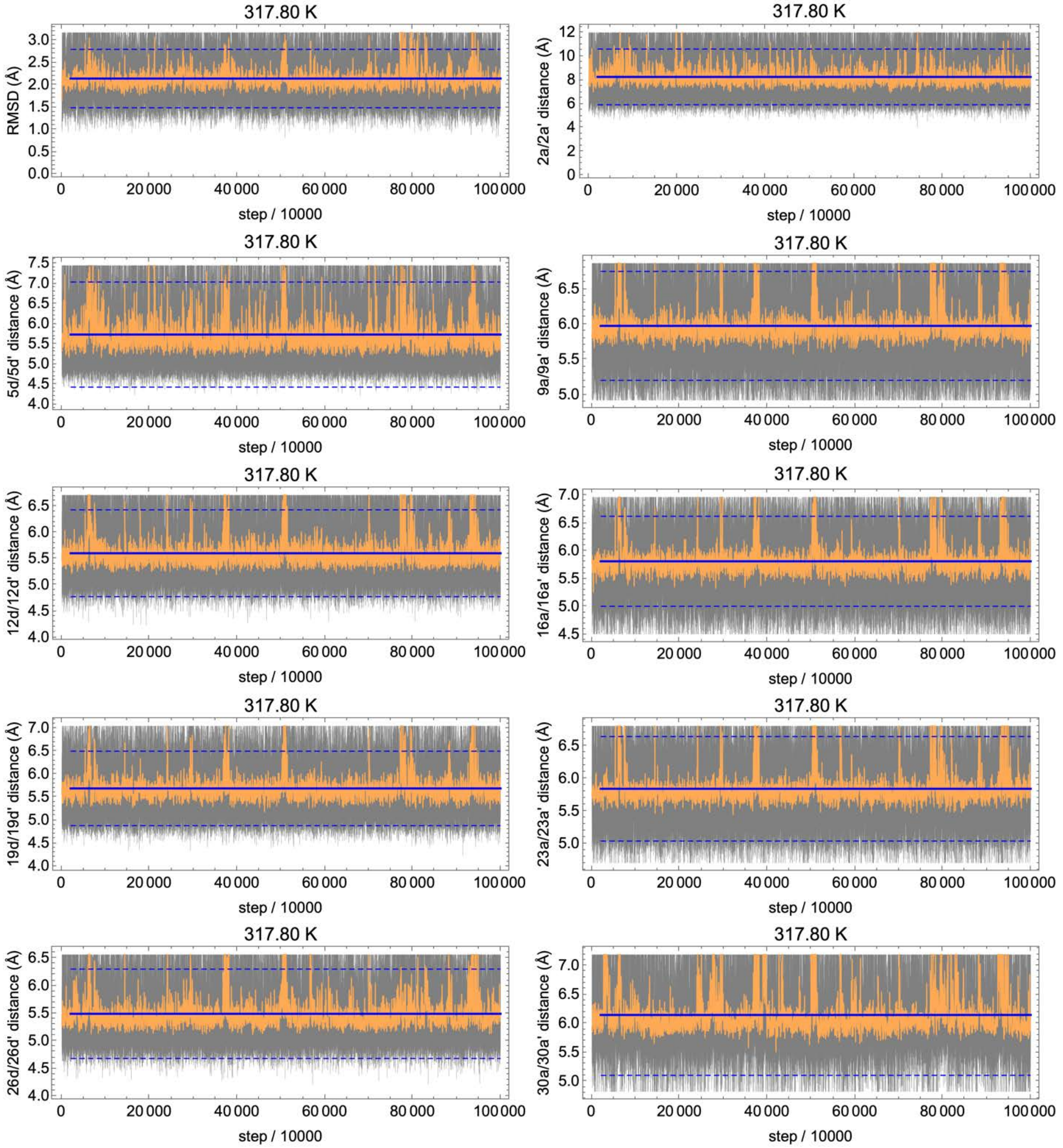

Measure at 317.80 K	Average (Å)	Standard Deviation (Å)	Slope (Å / μs)
RMSD	2.14251	0.655034	0.00482998
5d/5d' distance	5.73096	1.30398	−0.034295
12d/12d' distance	5.60402	0.826878	0.0194726
19d/19d' distance	5.69141	0.810111	0.0138844
26d/26d' distance	5.4921	0.809293	0.0318901
2a/2a' distance	8.26518	2.34227	−0.145659
9a/9a' distance	5.97555	0.772007	0.0189737
16a/16a' distance	5.81331	0.807398	0.019526
23a/23a' distance	5.84367	0.800537	0.0266449
30a/30a' distance	6.14755	1.0437	0.00892145
Core Distance	6.06264	1.41104	

Figure S307. Plots of RMSD and residue packing distances for T-REMD simulations of variant **s34e/29g'-x4x** at 317.80 K.

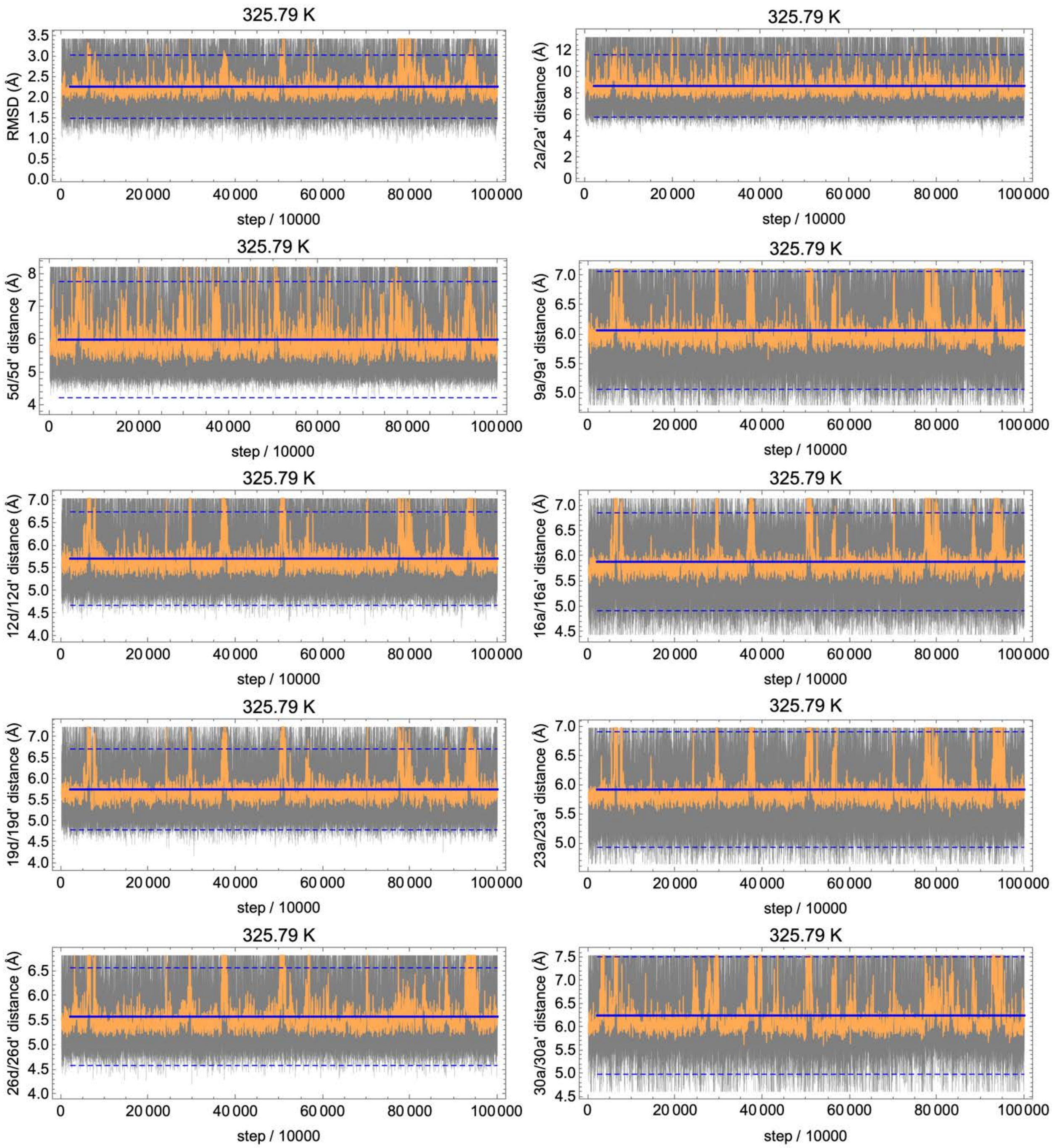

Measure at 325.79 K	Average (Å)	Standard Deviation (Å)	Slope (Å / μs)
RMSD	2.26705	0.770457	0.0250177
5d/5d' distance	5.99779	1.77149	−0.0199125
12d/12d' distance	5.71407	1.034	0.0400754
19d/19d' distance	5.7528	0.960792	0.0442229
26d/26d' distance	5.5803	0.994428	0.0567334
2a/2a' distance	8.7051	2.91208	−0.148314
9a/9a' distance	6.06929	1.0032	0.0354727
16a/16a' distance	5.88772	0.967523	0.0466323
23a/23a' distance	5.9246	0.991993	0.0560852
30a/30a' distance	6.25087	1.26389	0.0382474
Core Distance	6.20917	1.71439	

Figure S308. Plots of RMSD and residue packing distances for T-REMD simulations of variant **s34e/29g'-x4x** at 325.79 K.

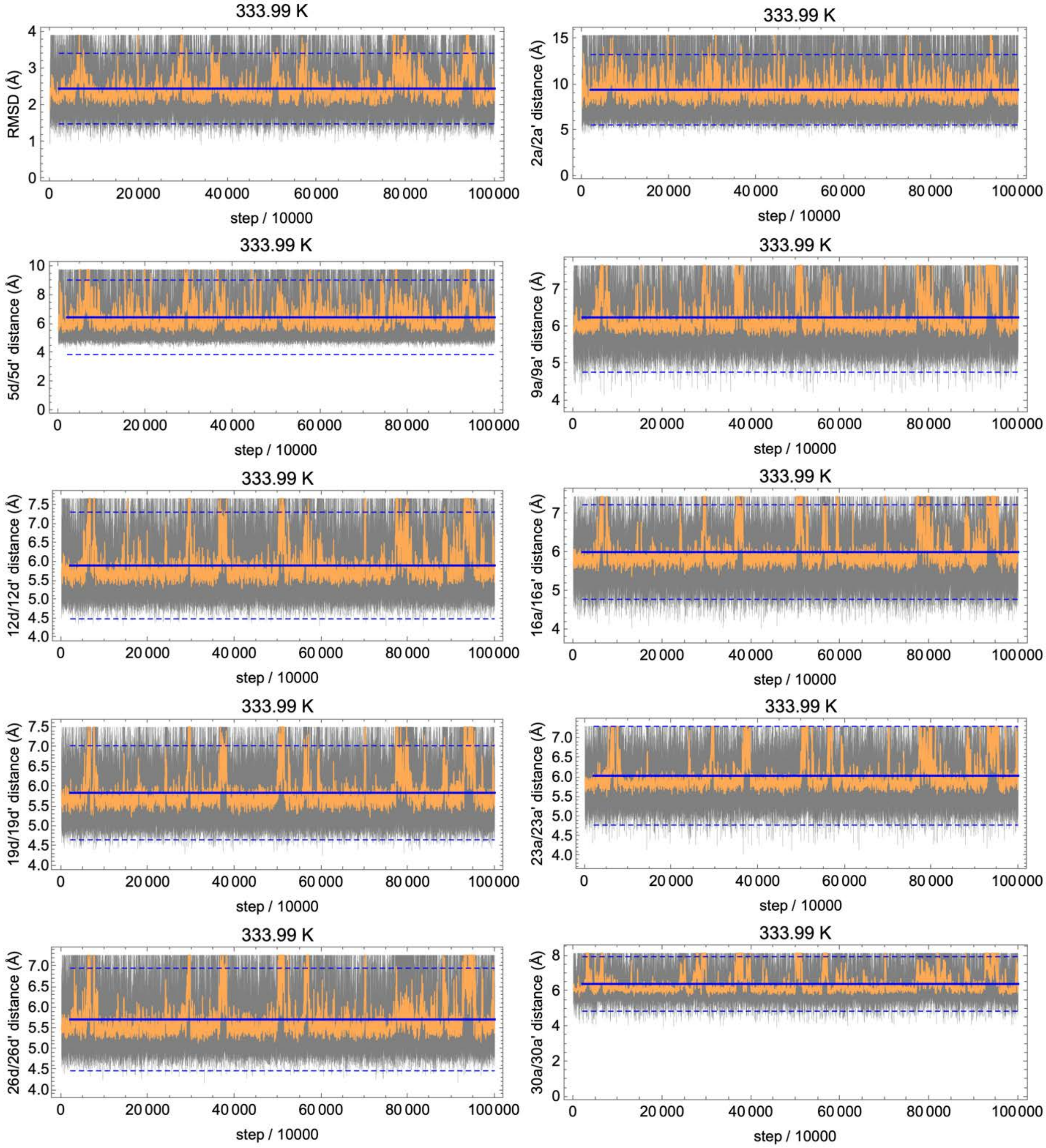

Measure at 333.99 K	Average (Å)	Standard Deviation (Å)	Slope (Å / μs)
RMSD	2.45722	0.962655	0.0642883
5d/5d' distance	6.46281	2.58502	0.0250462
12d/12d' distance	5.9017	1.41043	0.108721
19d/19d' distance	5.84593	1.18917	0.10126
26d/26d' distance	5.71388	1.24481	0.111567
2a/2a' distance	9.42471	3.83889	−0.172921
9a/9a' distance	6.24767	1.48144	0.110848
16a/16a' distance	6.00745	1.22764	0.107333
23a/23a' distance	6.04166	1.25948	0.11694
30a/30a' distance	6.40661	1.54685	0.0999697
Core Distance	6.45027	2.22398	

Figure S309. Plots of RMSD and residue packing distances for T-REMD simulations of variant **s34e/29g'-x4x** at 333.99 K.

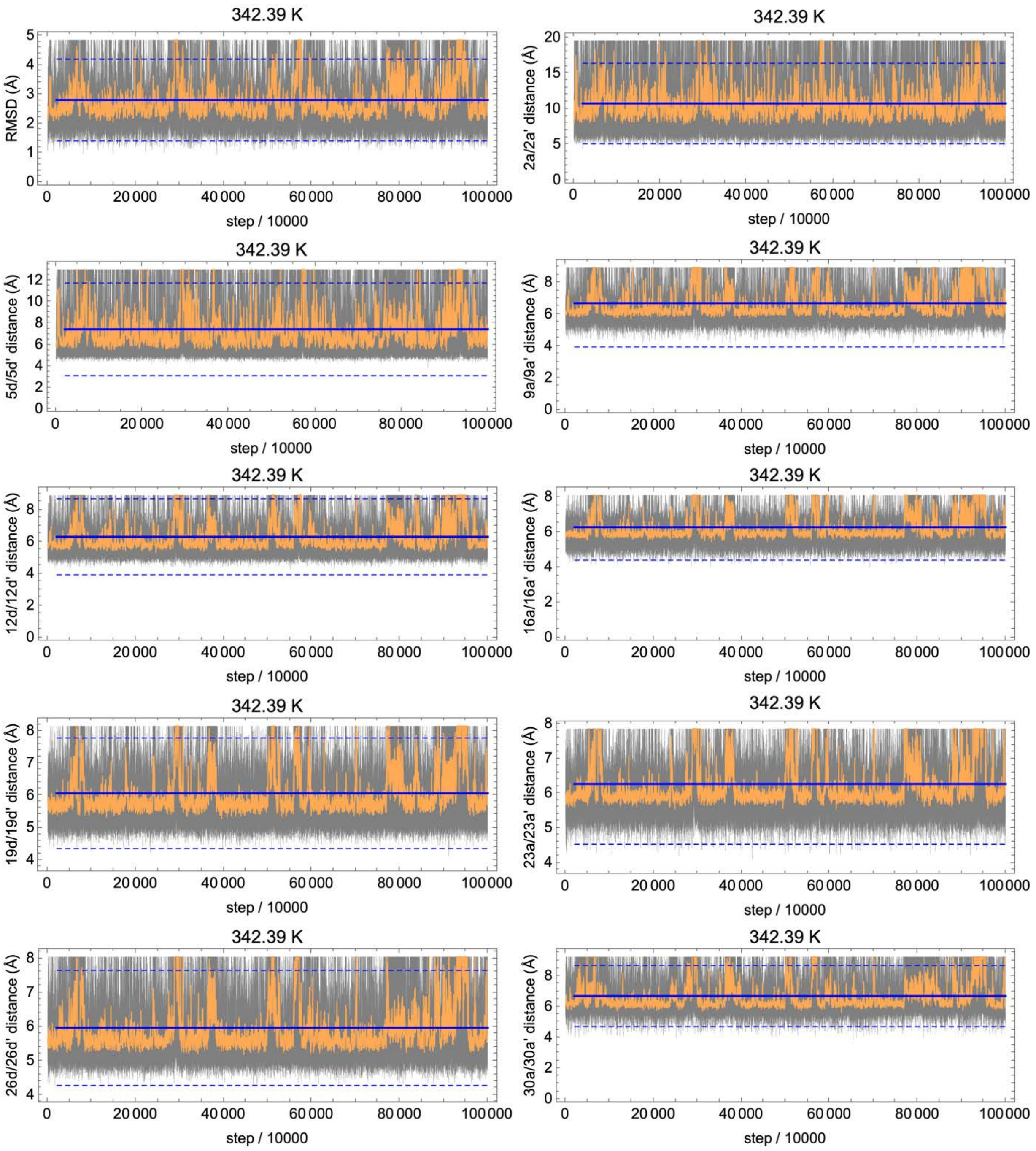

Measure at 342.39 K	Average (Å)	Standard Deviation (Å)	Slope (Å / μs)
RMSD	2.79847	1.39433	0.160856
5d/5d' distance	7.43306	4.32433	0.202578
12d/12d' distance	6.30976	2.38763	0.282017
19d/19d' distance	6.06592	1.7164	0.219194
26d/26d' distance	5.96074	1.68672	0.232262
2a/2a' distance	10.7527	5.6552	−0.0247319
9a/9a' distance	6.69214	2.7497	0.32501
16a/16a' distance	6.27231	1.87821	0.237986
23a/23a' distance	6.26446	1.73697	0.23902
30a/30a' distance	6.69372	1.99411	0.199613
Core Distance	6.93831	3.30284	

Figure S310. Plots of RMSD and residue packing distances for T-REMD simulations of variant **s34e/29g'-x4x** at 342.39 K.

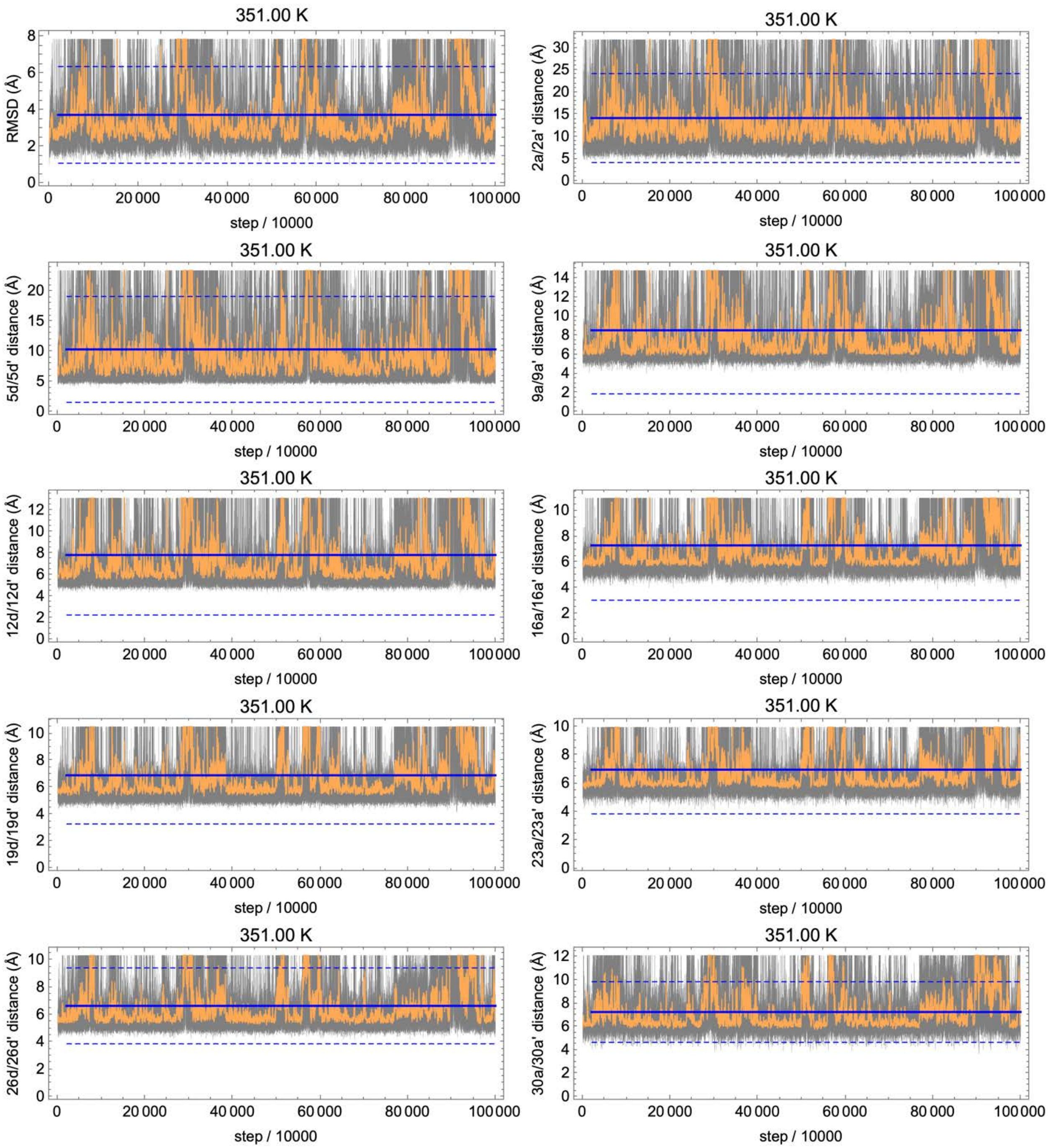

Measure at 351.00 K	Average (Å)	Standard Deviation (Å)	Slope (Å / μs)
RMSD	3.72153	2.63334	0.592373
5d/5d' distance	10.3399	8.7715	1.36573
12d/12d' distance	7.84319	5.57851	1.08428
19d/19d' distance	6.90346	3.62209	0.686176
26d/26d' distance	6.64334	2.77347	0.619277
2a/2a' distance	14.1864	10.0143	1.07567
9a/9a' distance	8.53299	6.65502	1.37182
16a/16a' distance	7.2999	4.25661	0.829183
23a/23a' distance	6.96788	3.11505	0.658137
30a/30a' distance	7.25631	2.59655	0.491325
Core Distance	8.44149	6.28455	

Figure S311. Plots of RMSD and residue packing distances for T-REMD simulations of variant **s34e/29g'-x4x** at 351.00 K.

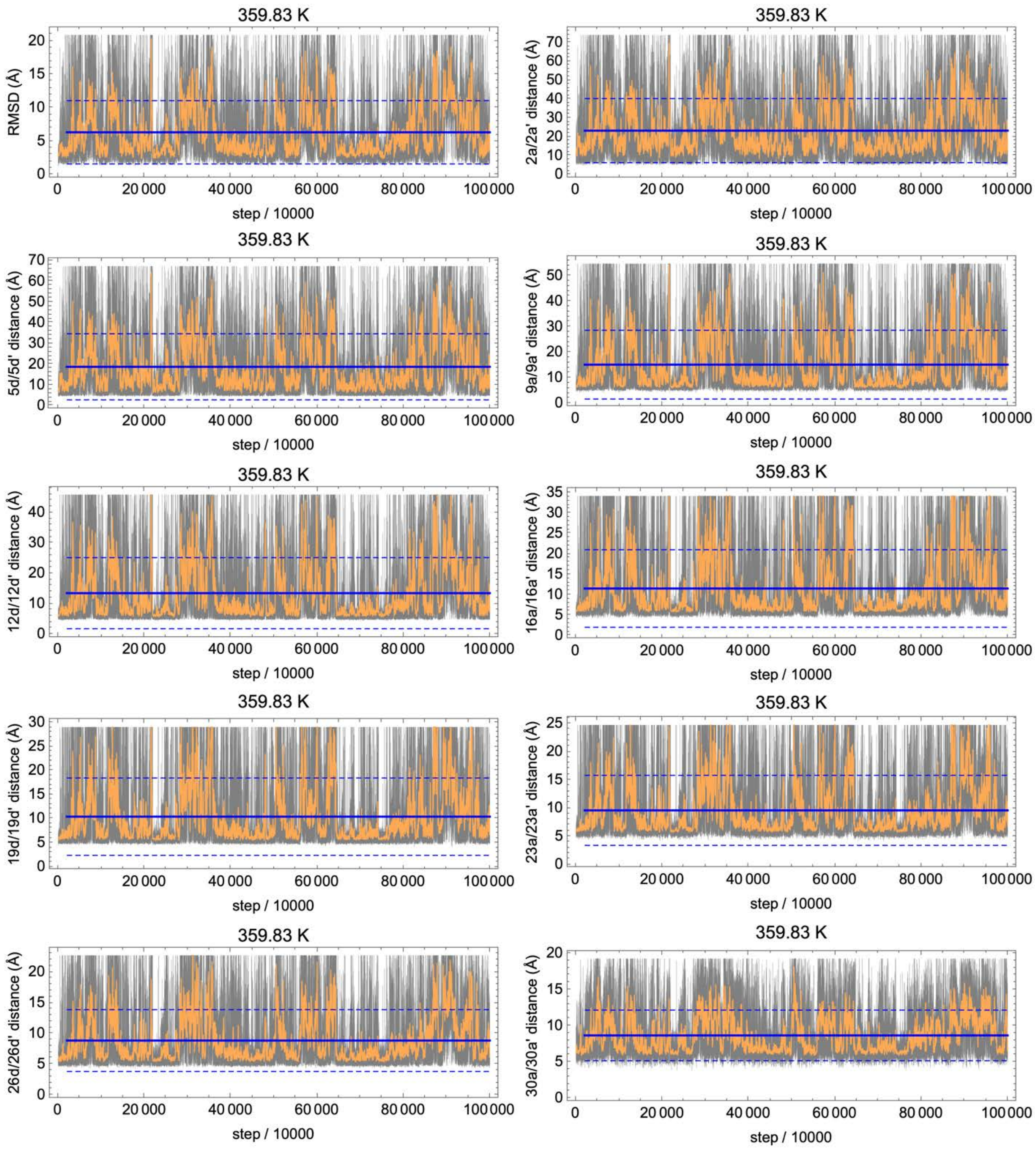

Measure at 359.83 K	Average (Å)	Standard Deviation (Å)	Slope (Å / μs)
RMSD	6.3112	4.74231	0.466051
5d/5d' distance	18.742	15.9294	0.87797
12d/12d' distance	13.4736	11.6937	0.789213
19d/19d' distance	10.3846	8.04021	0.591157
26d/26d' distance	8.8426	5.0259	0.459839
2a/2a' distance	23.2204	16.9983	0.523268
9a/9a' distance	15.113	13.4799	1.02571
16a/16a' distance	11.5015	9.48259	0.73172
23a/23a' distance	9.58767	6.20352	0.461754
30a/30a' distance	8.65504	3.49364	0.325864
Core Distance	13.28	11.9719	

Figure S312. Plots of RMSD and residue packing distances for T-REMD simulations of variant **s34e/29g'-x4x** at 359.83 K.

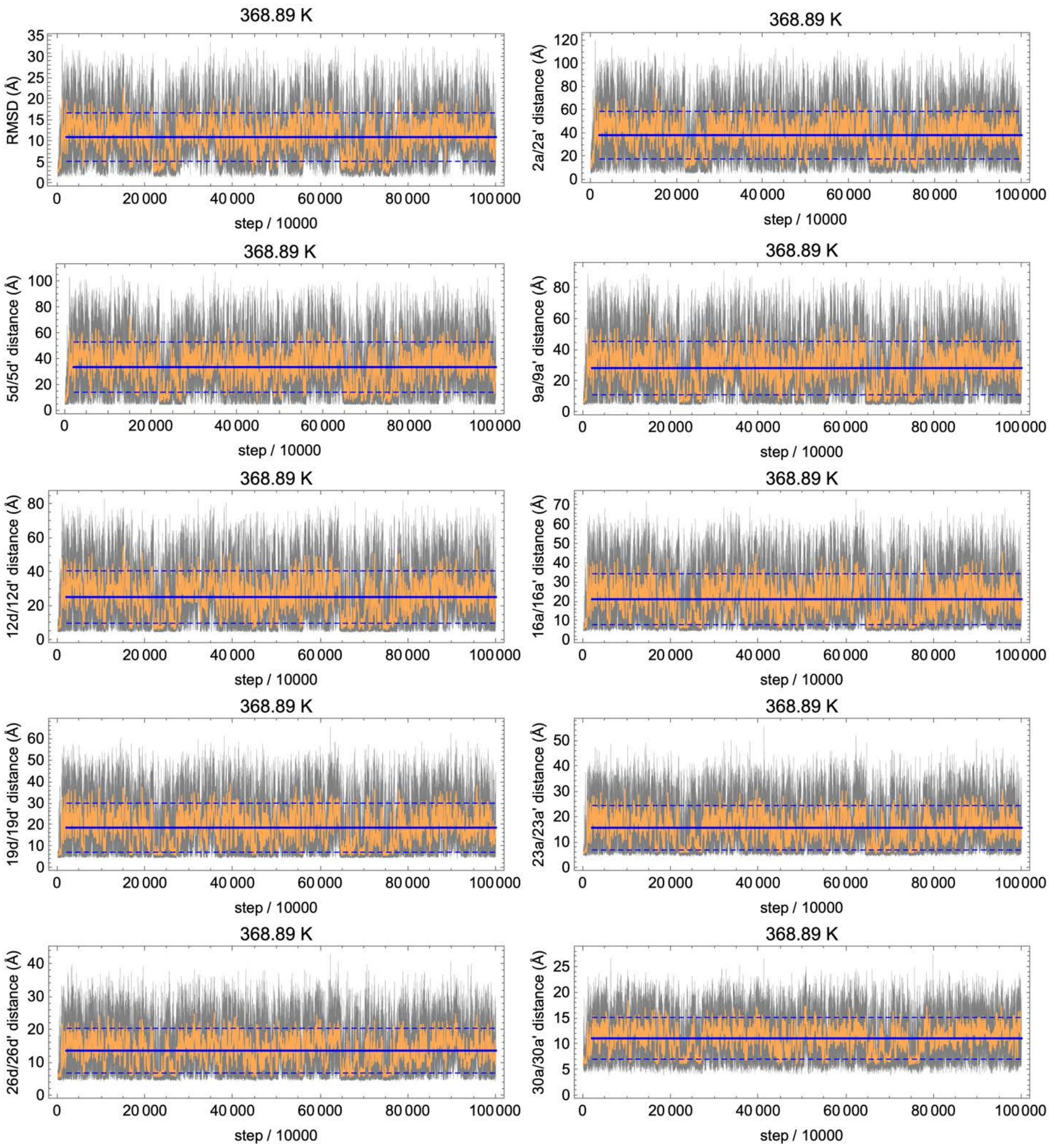

Measure at 368.89 K	Average (Å)	Standard Deviation (Å)	Slope (Å / μs)
RMSD	11.0772	5.75469	−0.139411
5d/5d' distance	33.8827	19.4733	−0.766453
12d/12d' distance	25.3659	15.4326	−0.653844
19d/19d' distance	18.7568	11.4332	−0.590844
26d/26d' distance	13.697	6.79922	−0.355327
2a/2a' distance	38.5135	20.5267	−0.984789
9a/9a' distance	28.5046	17.2308	−0.583508
16a/16a' distance	21.259	13.2424	−0.585132
23a/23a' distance	15.8104	8.75447	−0.491584
30a/30a' distance	11.1529	4.05196	−0.0571523
Core Distance	22.9936	16.5851	

Figure S313. Plots of RMSD and residue packing distances for T-REMD simulations of variant **s34e/29g'-x4x** at 368.89 K.

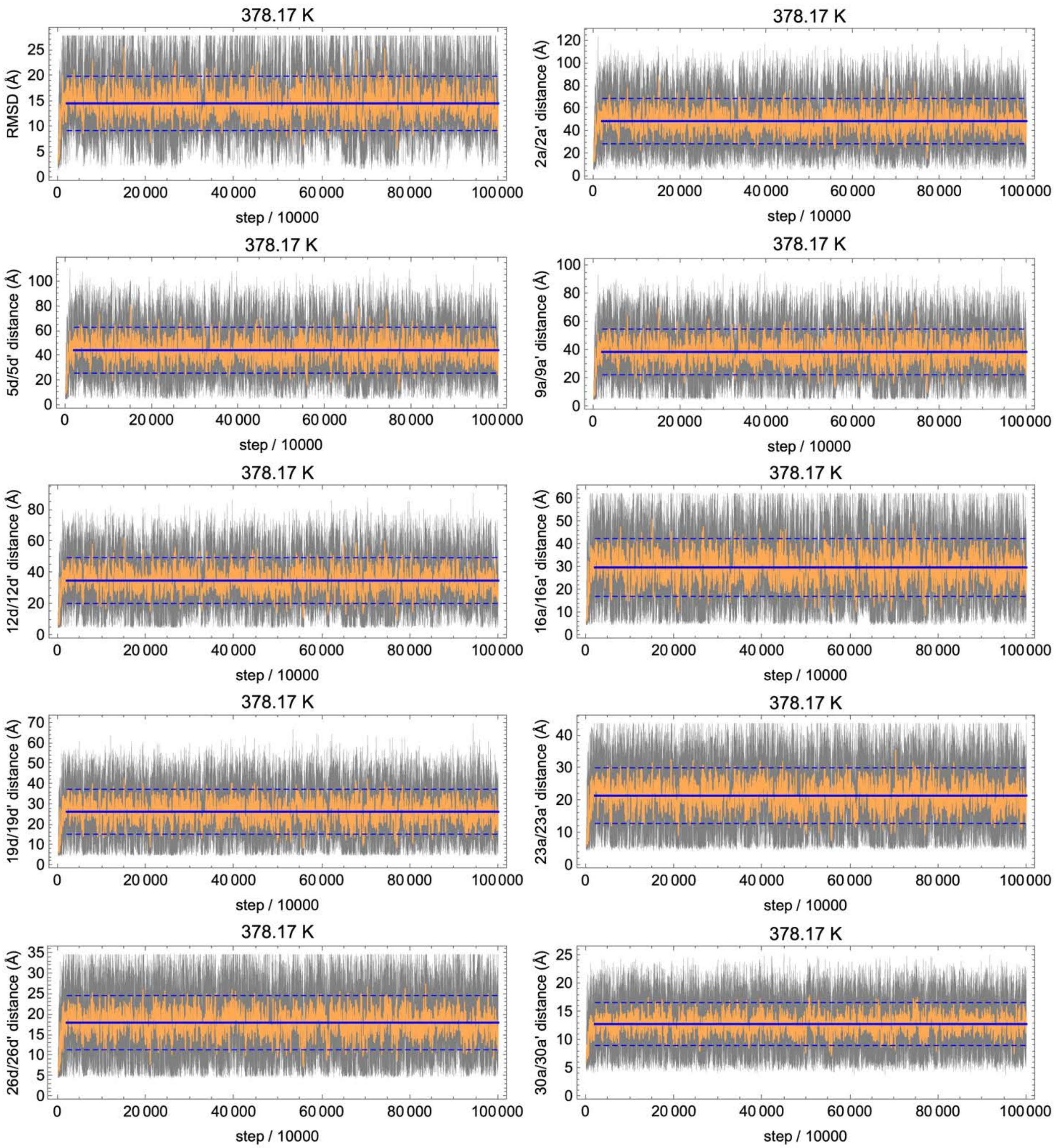

Measure at 378.17 K	Average (Å)	Standard Deviation (Å)	Slope (Å / μs)
RMSD	14.5666	5.32526	−0.265088
5d/5d' distance	44.483	18.5894	−0.955929
12d/12d' distance	34.9824	14.6147	−0.787125
19d/19d' distance	26.3846	11.0641	−0.629571
26d/26d' distance	18.0232	6.62401	−0.394416
2a/2a' distance	48.8366	20.109	−1.08025
9a/9a' distance	38.7197	16.2043	−0.82656
16a/16a' distance	29.863	12.6902	−0.738141
23a/23a' distance	21.5077	8.57995	−0.493383
30a/30a' distance	12.8356	3.80171	−0.0590997
Core Distance	30.6262	17.7221	

Figure S314. Plots of RMSD and residue packing distances for T-REMD simulations of variant **s34e/29g'-x4x** at 378.17 K.

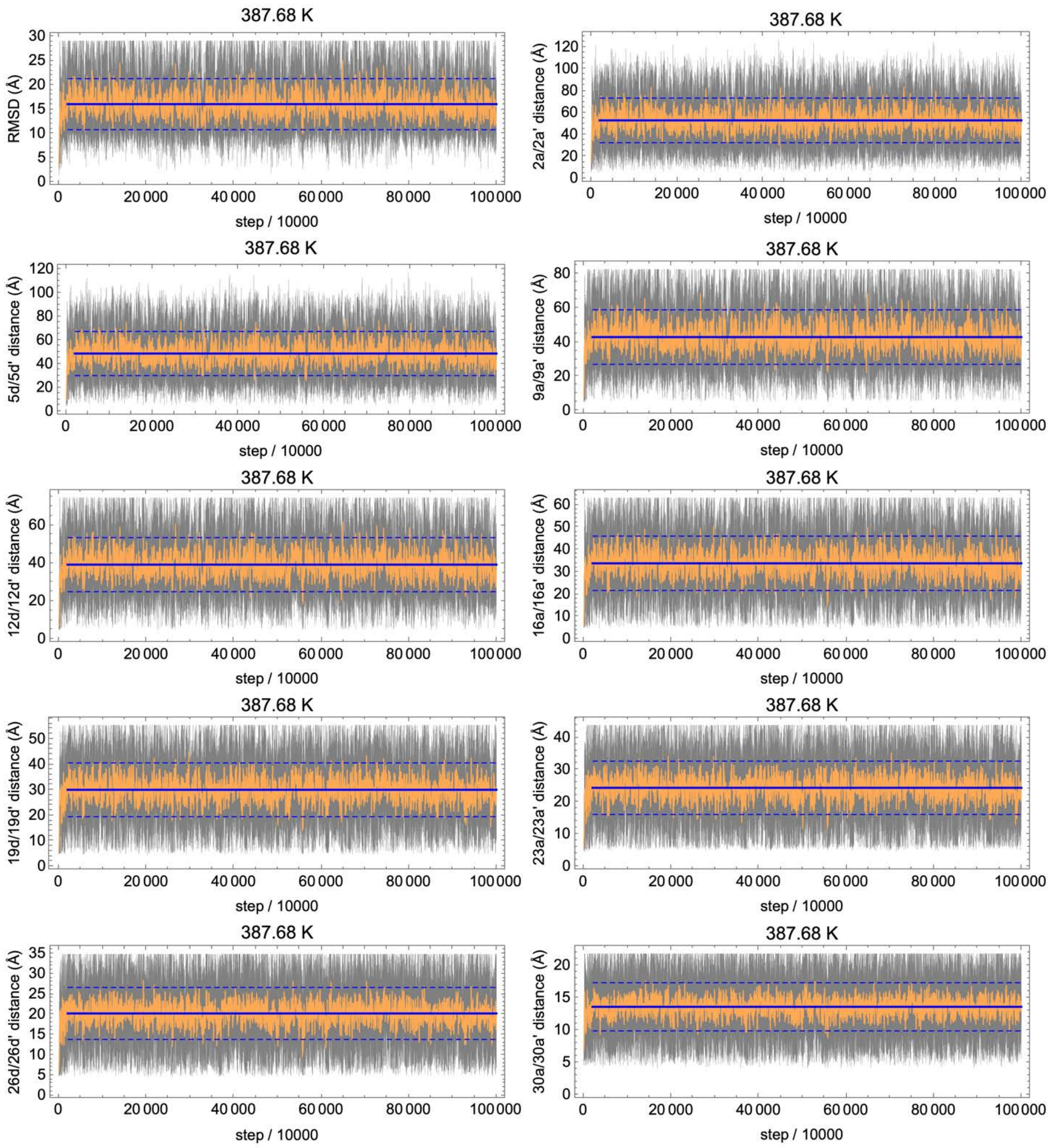

Measure at 387.68 K	Average (Å)	Standard Deviation (Å)	Slope (Å / μs)
RMSD	16.0389	5.25808	−0.263299
5d/5d' distance	48.7576	18.6192	−0.892643
12d/12d' distance	39.1645	14.254	−0.734865
19d/19d' distance	30.0575	10.5606	−0.52391
26d/26d' distance	20.2074	6.41554	−0.285457
2a/2a' distance	52.9965	20.381	−0.949413
9a/9a' distance	42.9852	15.9555	−0.837951
16a/16a' distance	33.829	12.1547	−0.644727
23a/23a' distance	24.3799	8.2764	−0.364599
30a/30a' distance	13.6137	3.72464	−0.0359192
Core Distance	33.999	18.2653	

Figure S315. Plots of RMSD and residue packing distances for T-REMD simulations of variant **s34e/29g'-x4x** at 387.68 K.

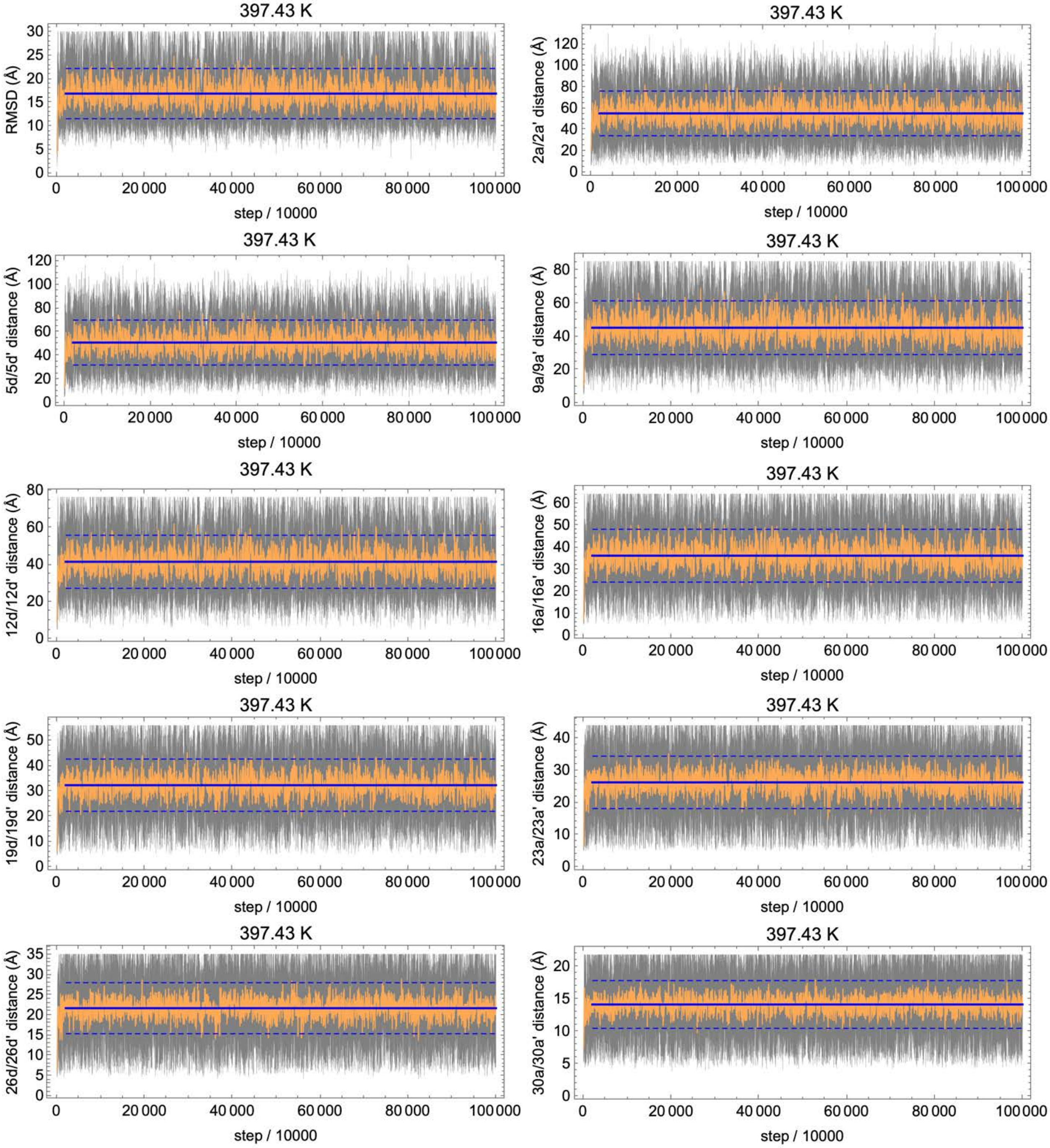

Measure at 397.43 K	Average (Å)	Standard Deviation (Å)	Slope (Å / μs)
RMSD	16.9028	5.32172	−0.259094
5d/5d' distance	51.0283	19.0371	−0.915867
12d/12d' distance	41.5469	14.3221	−0.624512
19d/19d' distance	32.3604	10.3236	−0.3615
26d/26d' distance	21.7111	6.32451	−0.080235
2a/2a' distance	55.2231	20.9637	−1.06063
9a/9a' distance	45.2979	16.155	−0.767962
16a/16a' distance	36.2155	11.9783	−0.481423
23a/23a' distance	26.2778	8.12864	−0.186557
30a/30a' distance	14.1729	3.67922	−0.0226918
Core Distance	35.9815	18.6745	

Figure S316. Plots of RMSD and residue packing distances for T-REMD simulations of variant **s34e/29g'-x4x** at 397.43 K.

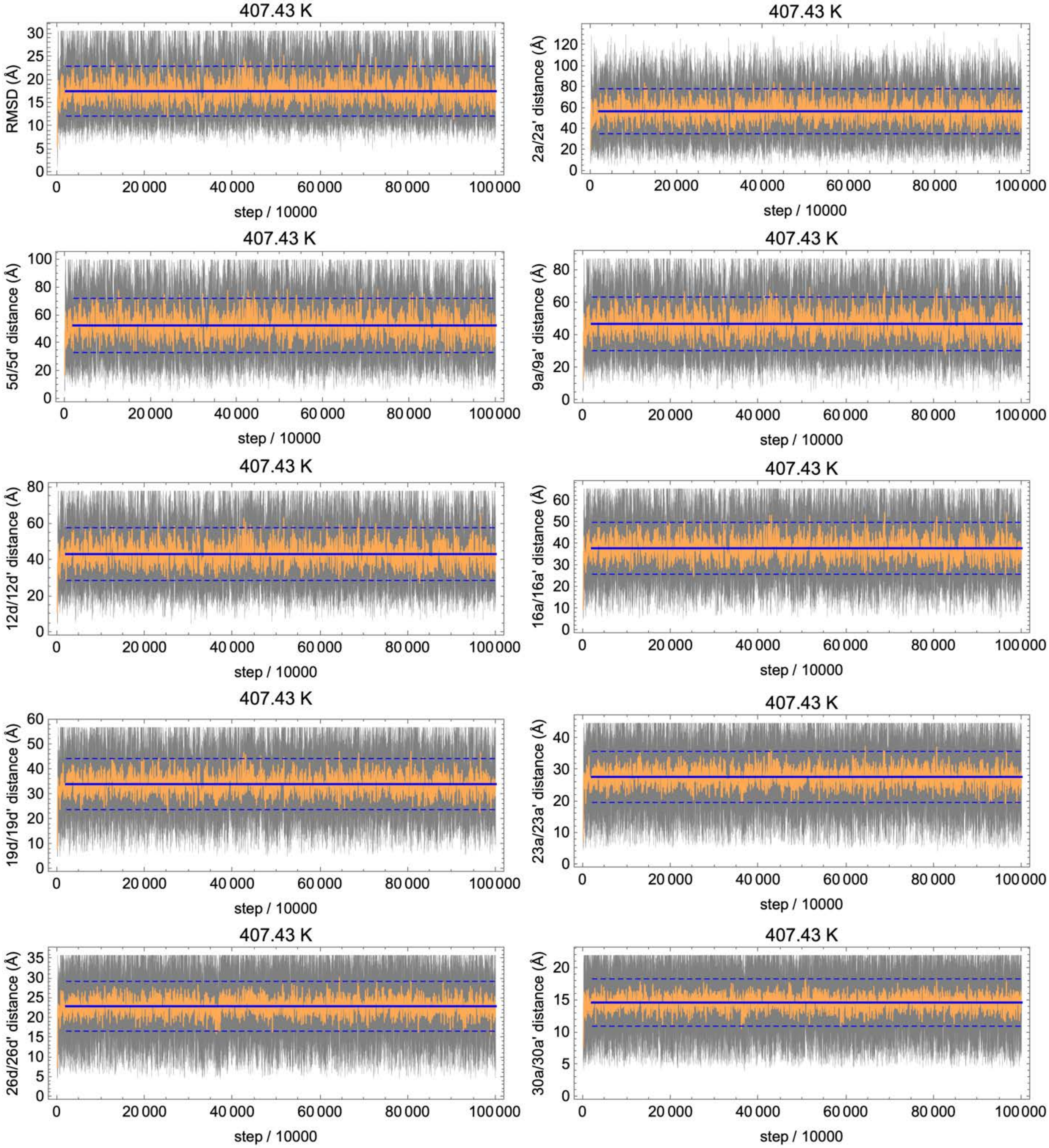

Measure at 407.43 K	Average (Å)	Standard Deviation (Å)	Slope (Å / μs)
RMSD	17.5641	5.40142	−0.208159
5d/5d' distance	52.695	19.4802	−0.747916
12d/12d' distance	43.2419	14.5638	−0.471252
19d/19d' distance	34.0836	10.2686	−0.282068
26d/26d' distance	22.9326	6.31212	−0.113512
2a/2a' distance	56.8866	21.4713	−0.947128
9a/9a' distance	46.9622	16.4903	−0.5692
16a/16a' distance	37.9433	12.0034	−0.337981
23a/23a' distance	27.7838	8.10109	−0.190198
30a/30a' distance	14.6622	3.67597	−0.0693394
Core Distance	37.4657	19.0388	

Figure S317. Plots of RMSD and residue packing distances for T-REMD simulations of variant **s34e/29g'-x4x** at 407.43 K.

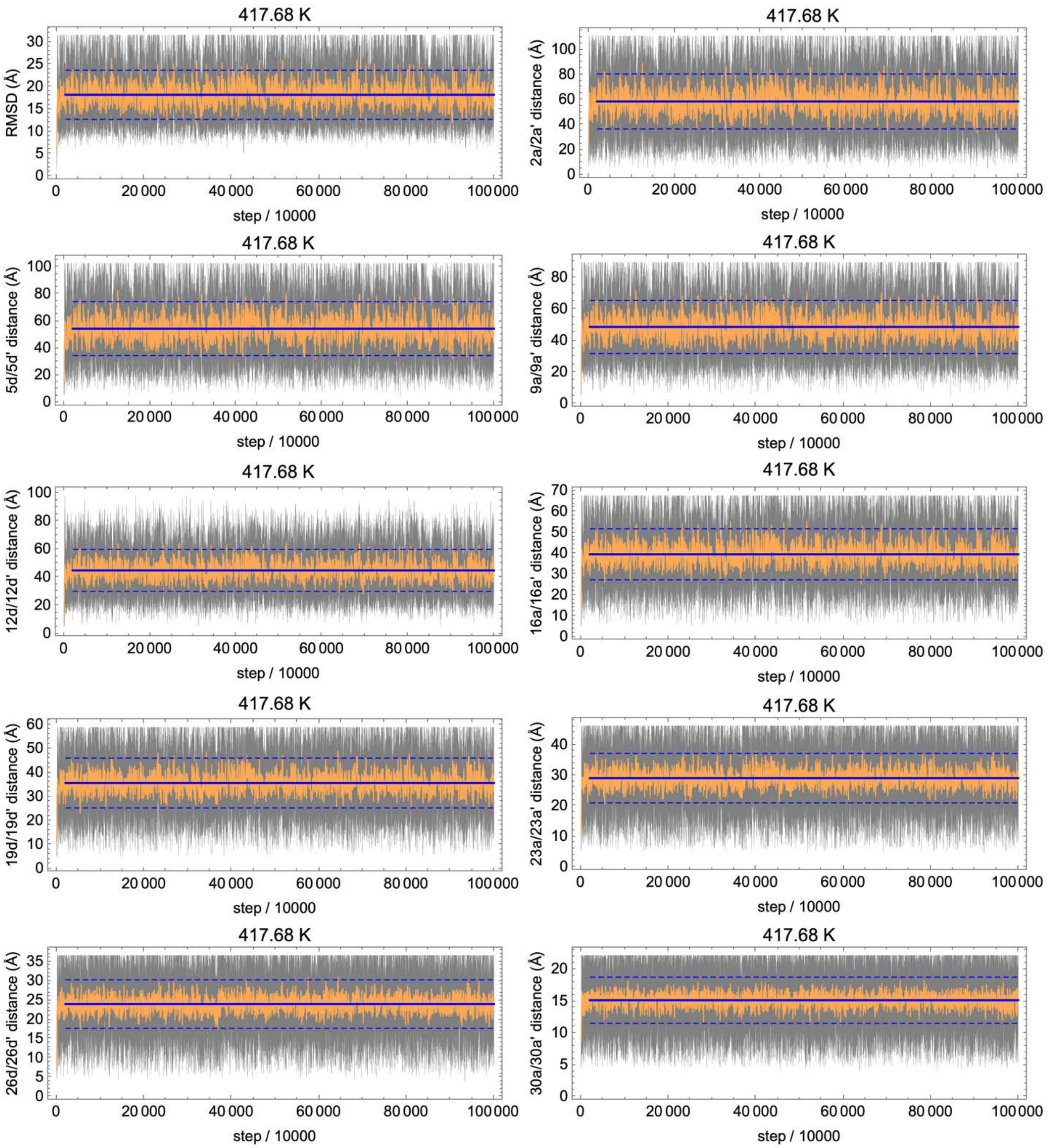

Measure at 417.68 K	Average (Å)	Standard Deviation (Å)	Slope (Å / μs)
RMSD	18.2082	5.49431	−0.0805211
5d/5d' distance	54.3086	19.9023	−0.347645
12d/12d' distance	44.8922	14.8739	−0.11359
19d/19d' distance	35.6429	10.4052	−0.0105026
26d/26d' distance	23.9939	6.28157	0.044867
2a/2a' distance	58.4902	21.9546	−0.539204
9a/9a' distance	48.604	16.8596	−0.169193
16a/16a' distance	39.5398	12.218	−0.0248335
23a/23a' distance	29.1103	8.112	0.0412715
30a/30a' distance	15.136	3.62963	−0.0532979
Core Distance	38.8576	19.4496	

Figure S318. Plots of RMSD and residue packing distances for T-REMD simulations of variant **s34e/29g'-x4x** at 417.68 K.

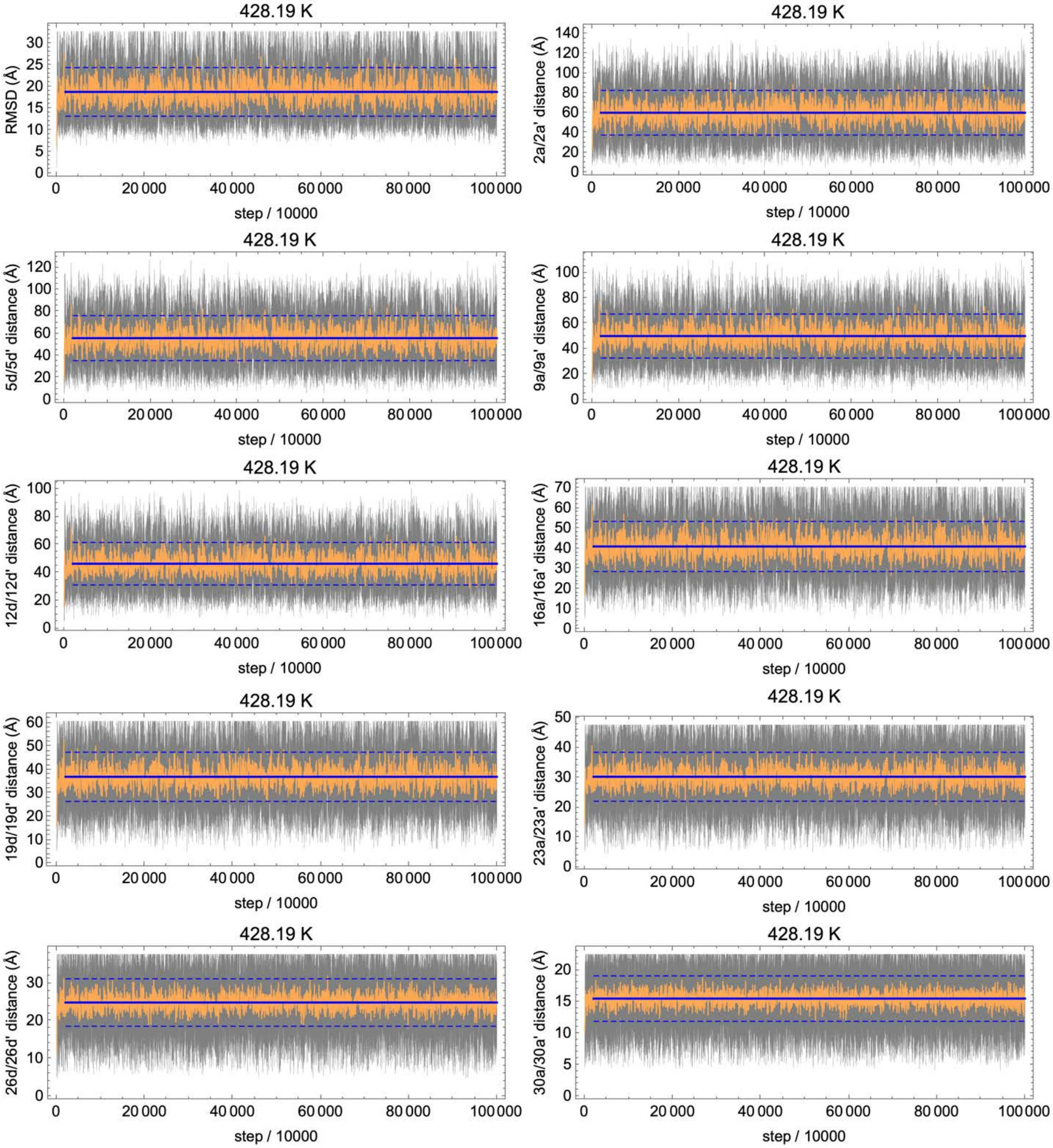

Measure at 428.19 K	Average (Å)	Standard Deviation (Å)	Slope (Å / μs)
RMSD	18.7898	5.60206	−0.0449329
5d/5d' distance	55.8683	20.4013	−0.235002
12d/12d' distance	46.3393	15.2384	−0.0976584
19d/19d' distance	36.9292	10.5193	0.0365561
26d/26d' distance	24.8492	6.28123	0.0430669
2a/2a' distance	60.0668	22.5007	−0.253832
9a/9a' distance	50.1183	17.3088	−0.16044
16a/16a' distance	40.9268	12.4291	−0.0204051
23a/23a' distance	30.2158	8.19937	0.0555765
30a/30a' distance	15.4797	3.59881	−0.0519855
Core Distance	40.0881	19.9244	

Figure S319. Plots of RMSD and residue packing distances for T-REMD simulations of variant **s34e/29g’-x4x** at 428.19 K.

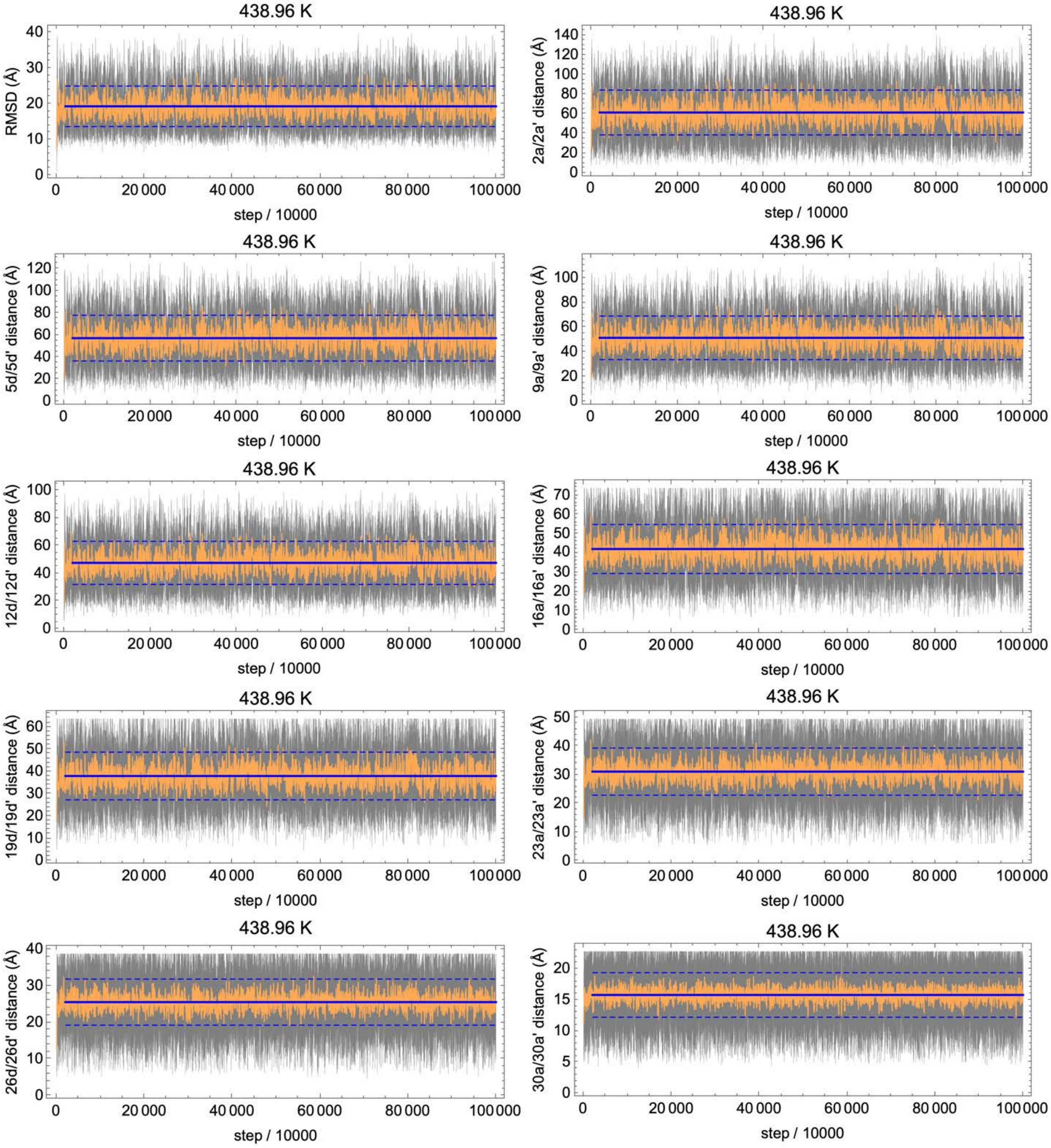

Measure at 438.96 K	Average (Å)	Standard Deviation (Å)	Slope (Å / μs)
RMSD	19.2628	5.6799	0.106287
5d/5d' distance	57.1199	20.7048	0.385721
12d/12d' distance	47.5593	15.5535	0.285741
19d/19d' distance	37.9702	10.707	0.123114
26d/26d' distance	25.541	6.29184	0.0825447
2a/2a' distance	61.3286	22.8006	0.302608
9a/9a' distance	51.3824	17.6551	0.336865
16a/16a' distance	42.0451	12.71	0.15033
23a/23a' distance	31.1336	8.22526	0.187681
30a/30a' distance	15.782	3.57865	−0.0514998
Core Distance	41.0958	20.289	

Figure S320. Plots of RMSD and residue packing distances for T-REMD simulations of variant **s34e/29g'-x4x** at 438.96 K.

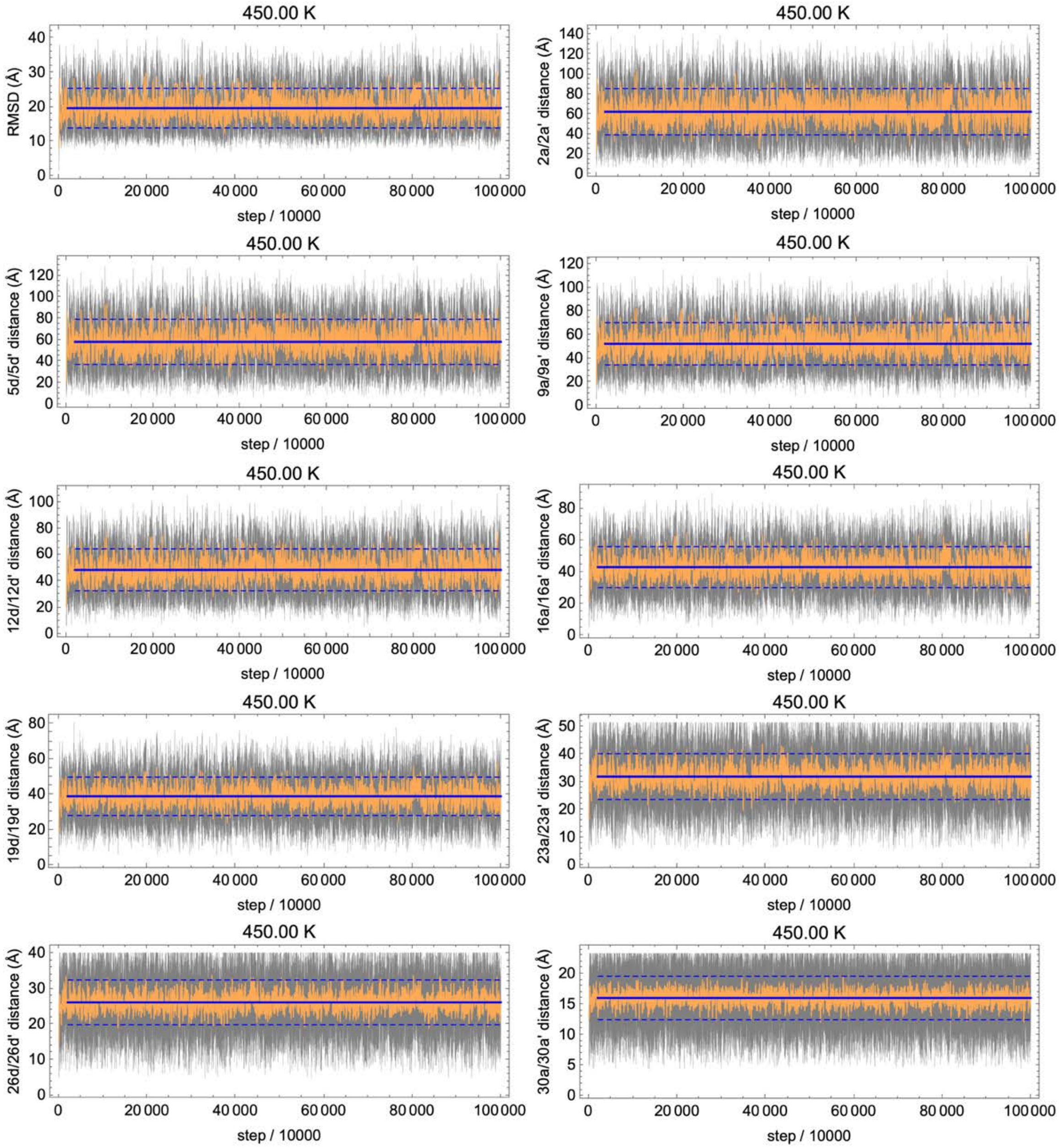

Measure at 450.00 K	Average (Å)	Standard Deviation (Å)	Slope (Å / μs)
RMSD	19.6721	5.74818	0.177822
5d/5d' distance	58.093	21.0691	0.686051
12d/12d' distance	48.5556	15.8825	0.472303
19d/19d' distance	38.895	10.882	0.0513156
26d/26d' distance	26.141	6.29344	0.00542954
2a/2a' distance	62.2978	23.2227	0.742417
9a/9a' distance	52.37	17.9805	0.591278
16a/16a' distance	43.0507	12.9745	0.257697
23a/23a' distance	31.9359	8.28723	0.0629275
30a/30a' distance	16.0383	3.56828	−0.12416
Core Distance	41.9308	20.6169	

Figure S321. Plots of RMSD and residue packing distances for T-REMD simulations of variant **s34e/29g’-x4x** at 450.00 K.

(return to Table of Contents)

7f/10b'-xx

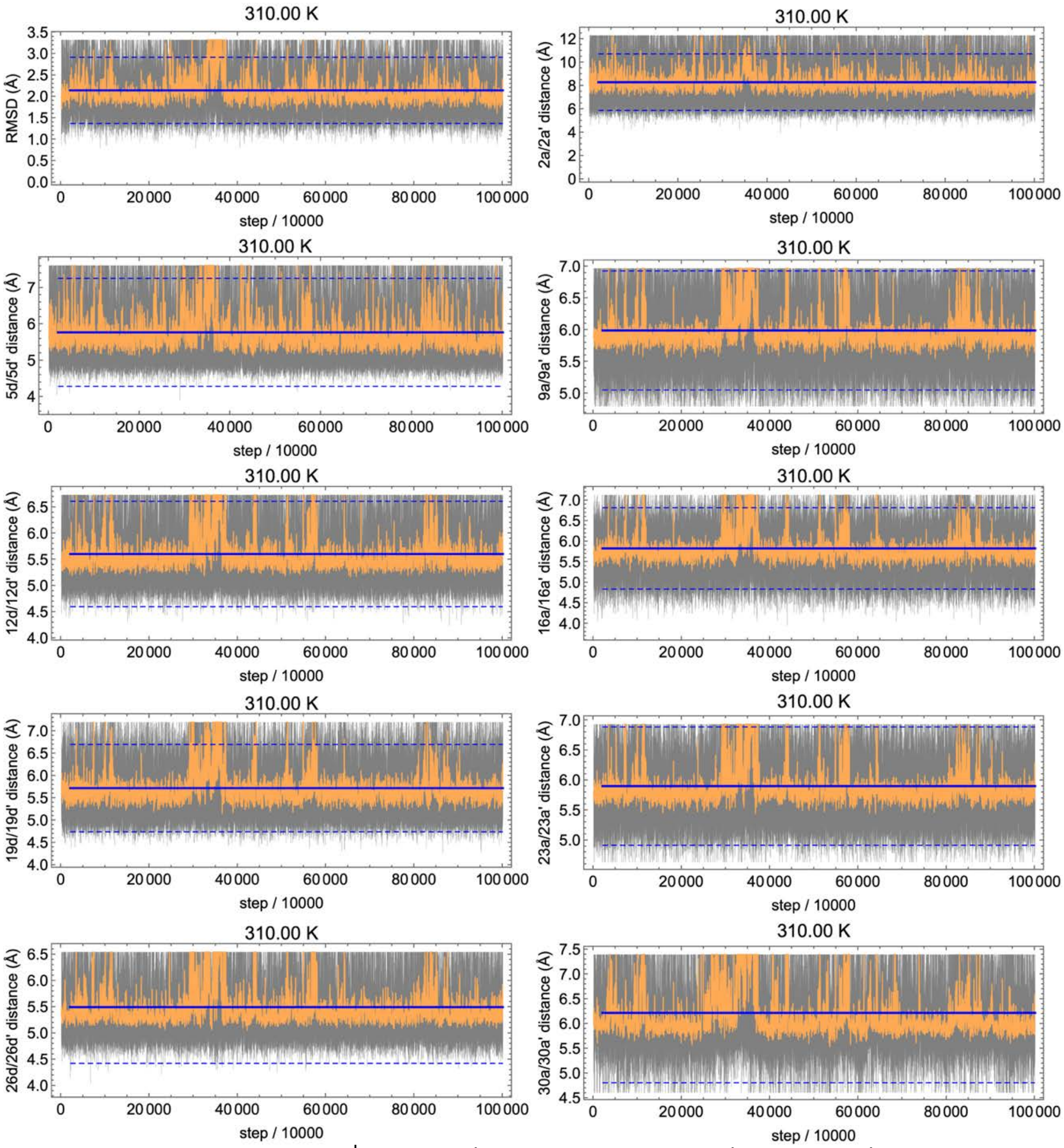

Measure at 310.00 K	Average (Å)	Standard Deviation (Å)	Slope (Å / μs)
RMSD	2.15226	0.772585	−0.0957482
5d/5d' distance	5.7833	1.48604	−0.120194
12d/12d' distance	5.61289	1.00555	−0.0575605
19d/19d' distance	5.72846	0.978474	−0.0472616
26d/26d' distance	5.50455	1.0699	−0.0738665
2a/2a' distance	8.33272	2.41746	−0.231059
9a/9a' distance	5.99682	0.937697	−0.0533614
16a/16a' distance	5.83965	0.989429	−0.0595939
23a/23a' distance	5.90818	0.986012	−0.0625689
30a/30a' distance	6.22704	1.41118	−0.203596
Core Distance	6.10373	1.56112	

Figure S322. Plots of RMSD and residue packing distances for T-REMD simulations of variant **7f/10b'-xx** at 310.00 K.

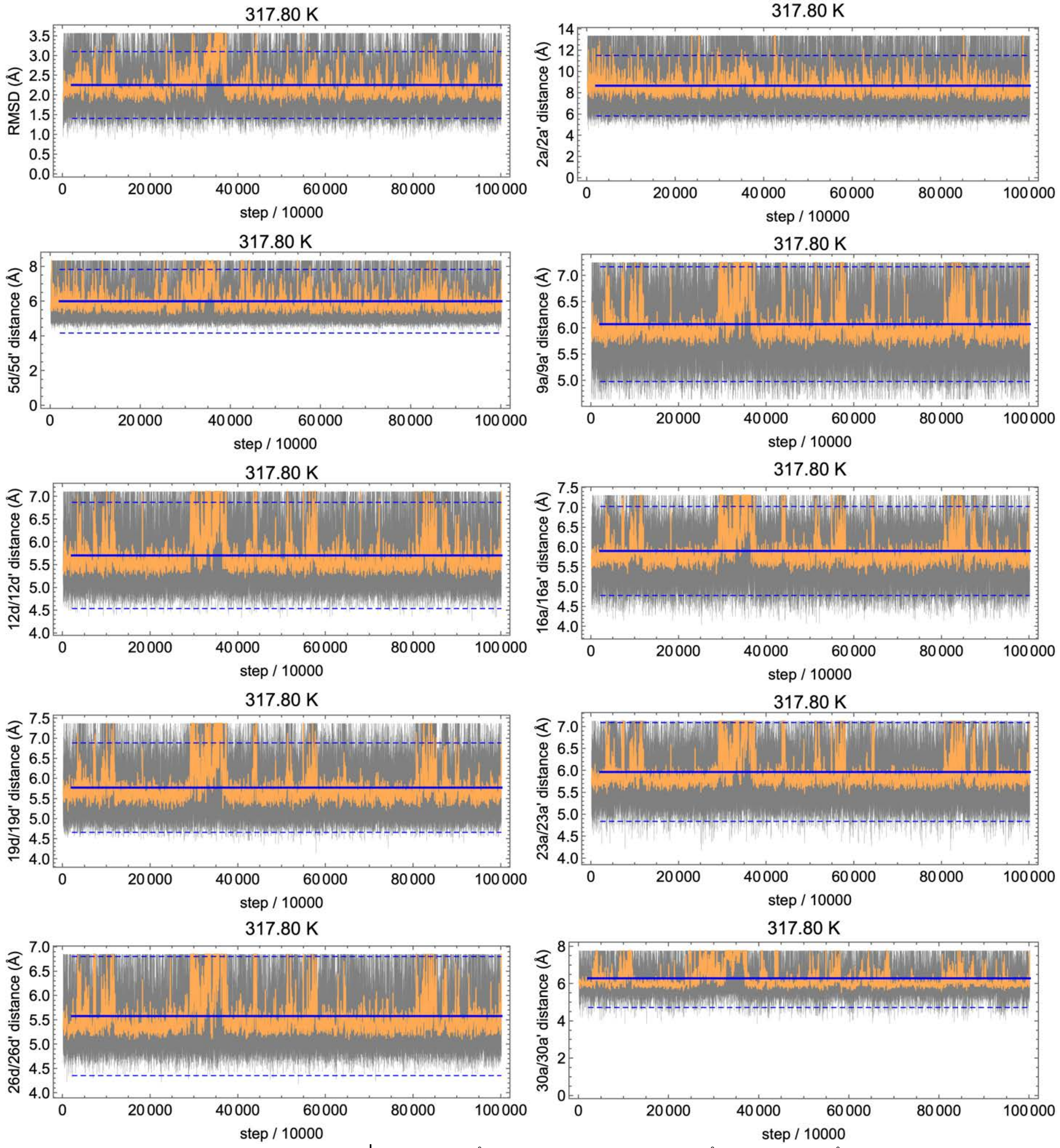

Measure at 317.80 K	Average (Å)	Standard Deviation (Å)	Slope (Å / μs)
RMSD	2.26932	0.846687	−0.0909951
5d/5d' distance	6.03186	1.83322	−0.118812
12d/12d' distance	5.71714	1.16615	−0.0593765
19d/19d' distance	5.78701	1.11246	−0.0641351
26d/26d' distance	5.5904	1.22467	−0.0853878
2a/2a' distance	8.72221	2.84558	−0.241064
9a/9a' distance	6.08386	1.09312	−0.0575991
16a/16a' distance	5.91688	1.12234	−0.0644457
23a/23a' distance	5.97932	1.12694	−0.0724176
30a/30a' distance	6.31819	1.56818	−0.22578
Core Distance	6.23854	1.79651	

Figure S323. Plots of RMSD and residue packing distances for T-REMD simulations of variant **7f/10b'-xx** at 317.80 K.

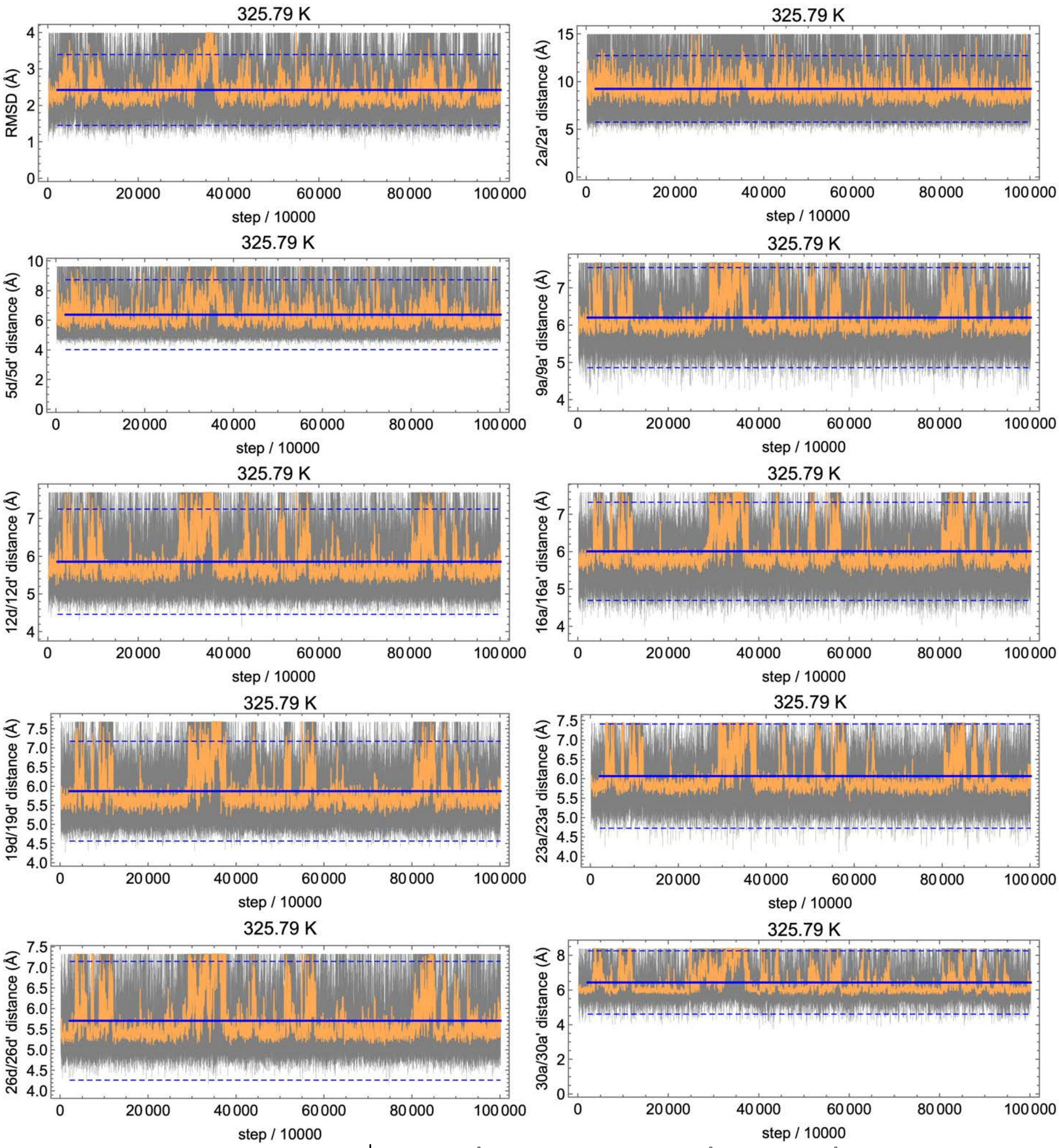

Measure at 325.79 K	Average (Å)	Standard Deviation (Å)	Slope (Å / μs)
RMSD	2.44118	0.972078	−0.120984
5d/5d' distance	6.42232	2.35405	−0.17379
12d/12d' distance	5.87228	1.39401	−0.0888018
19d/19d' distance	5.88641	1.30427	−0.0721601
26d/26d' distance	5.72018	1.44247	−0.120409
2a/2a' distance	9.30972	3.48832	−0.323738
9a/9a' distance	6.22008	1.34346	−0.0781349
16a/16a' distance	6.0264	1.3149	−0.0823051
23a/23a' distance	6.08476	1.34241	−0.106264
30a/30a' distance	6.47132	1.82471	−0.271923
Core Distance	6.44594	2.15585	

Figure S324. Plots of RMSD and residue packing distances for T-REMD simulations of variant **7f/10b'-xx** at 325.79 K.

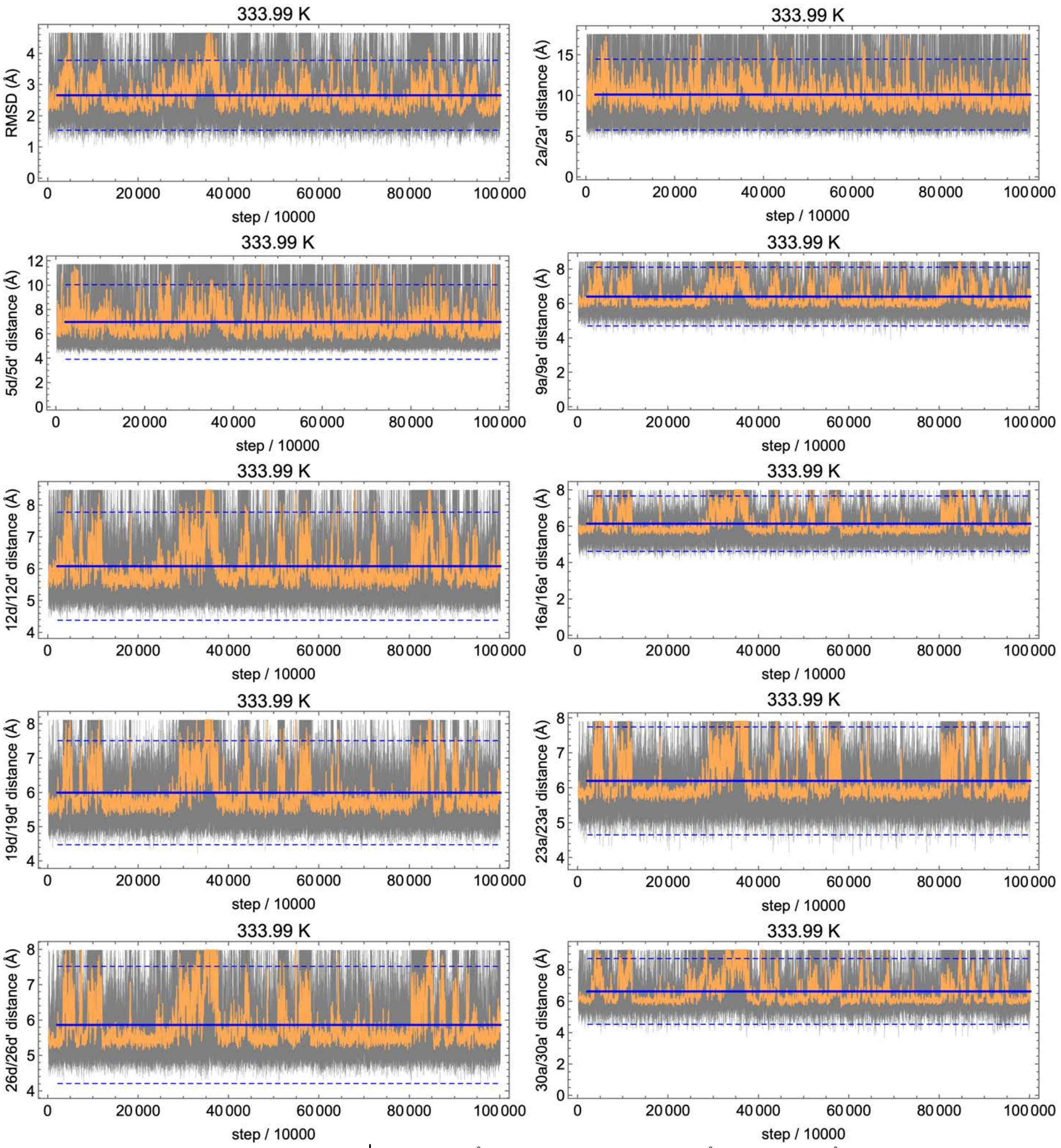

Measure at 333.99 K	Average (Å)	Standard Deviation (Å)	Slope (Å / μs)
RMSD	2.67772	1.12083	−0.127653
5d/5d' distance	7.02978	3.061	−0.19901
12d/12d' distance	6.10221	1.69666	−0.0754106
19d/19d' distance	6.0102	1.51756	−0.0661893
26d/26d' distance	5.88063	1.65499	−0.116265
2a/2a' distance	10.1903	4.36025	−0.442199
9a/9a' distance	6.43506	1.70527	−0.0738211
16a/16a' distance	6.18034	1.52367	−0.0752723
23a/23a' distance	6.21575	1.54488	−0.0947817
30a/30a' distance	6.66696	2.09783	−0.285386
Core Distance	6.74569	2.63884	

Figure S325. Plots of RMSD and residue packing distances for T-REMD simulations of variant **7f/10b'-xx** at 333.99 K.

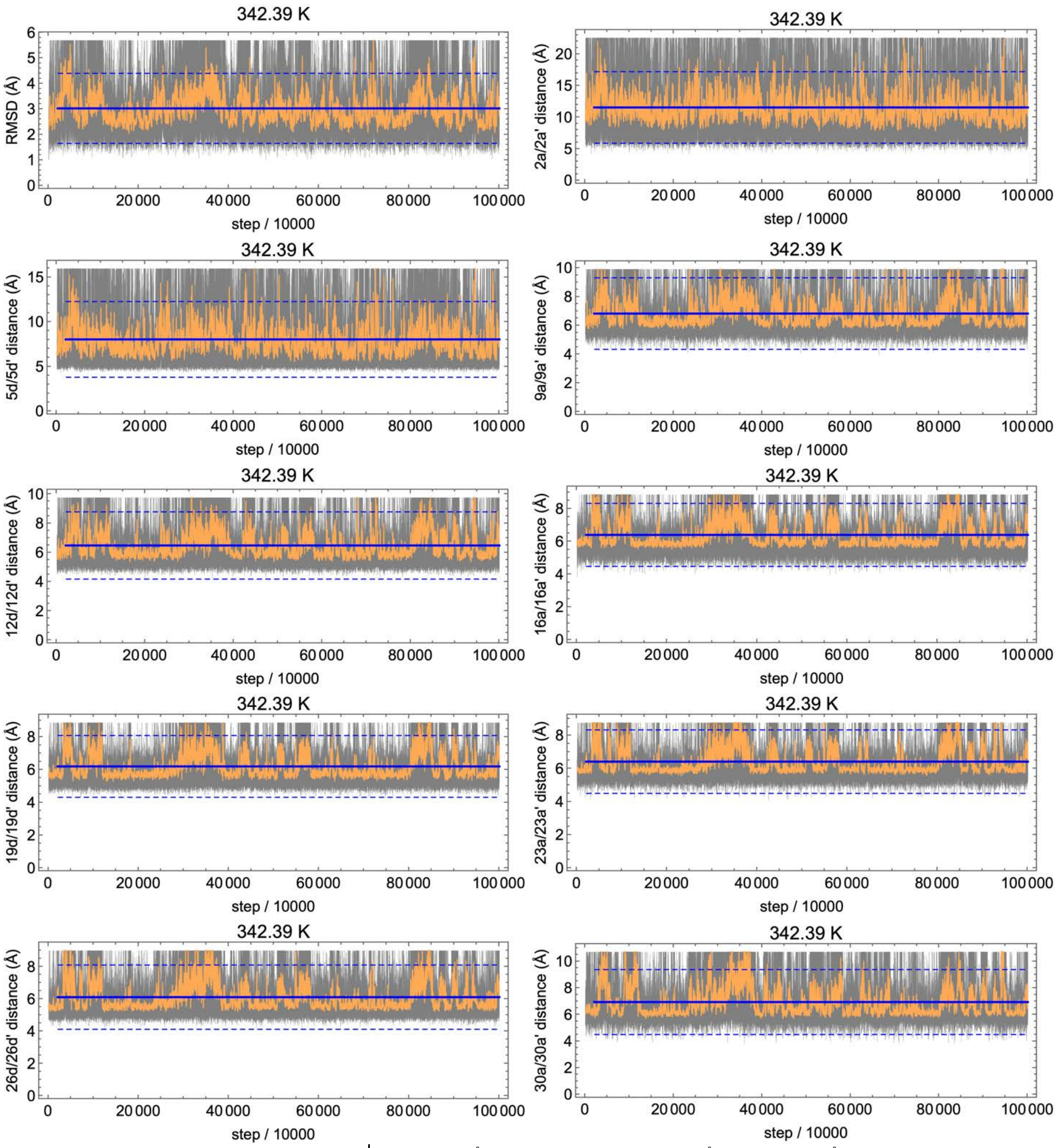

Measure at 342.39 K	Average (Å)	Standard Deviation (Å)	Slope (Å / μs)
RMSD	3.04128	1.37464	−0.125719
5d/5d' distance	8.07669	4.23996	−0.165004
12d/12d' distance	6.50628	2.30023	−0.055106
19d/19d' distance	6.22385	1.88576	−0.0551785
26d/26d' distance	6.13428	1.99009	−0.13698
2a/2a' distance	11.607	5.6644	−0.461693
9a/9a' distance	6.85257	2.48846	−0.0284755
16a/16a' distance	6.42216	1.92145	−0.0696742
23a/23a' distance	6.43584	1.91453	−0.100874
30a/30a' distance	6.96756	2.43987	−0.303552
Core Distance	7.24736	3.43958	

Figure S326. Plots of RMSD and residue packing distances for T-REMD simulations of variant **7f/10b'-xx** at 342.39 K.

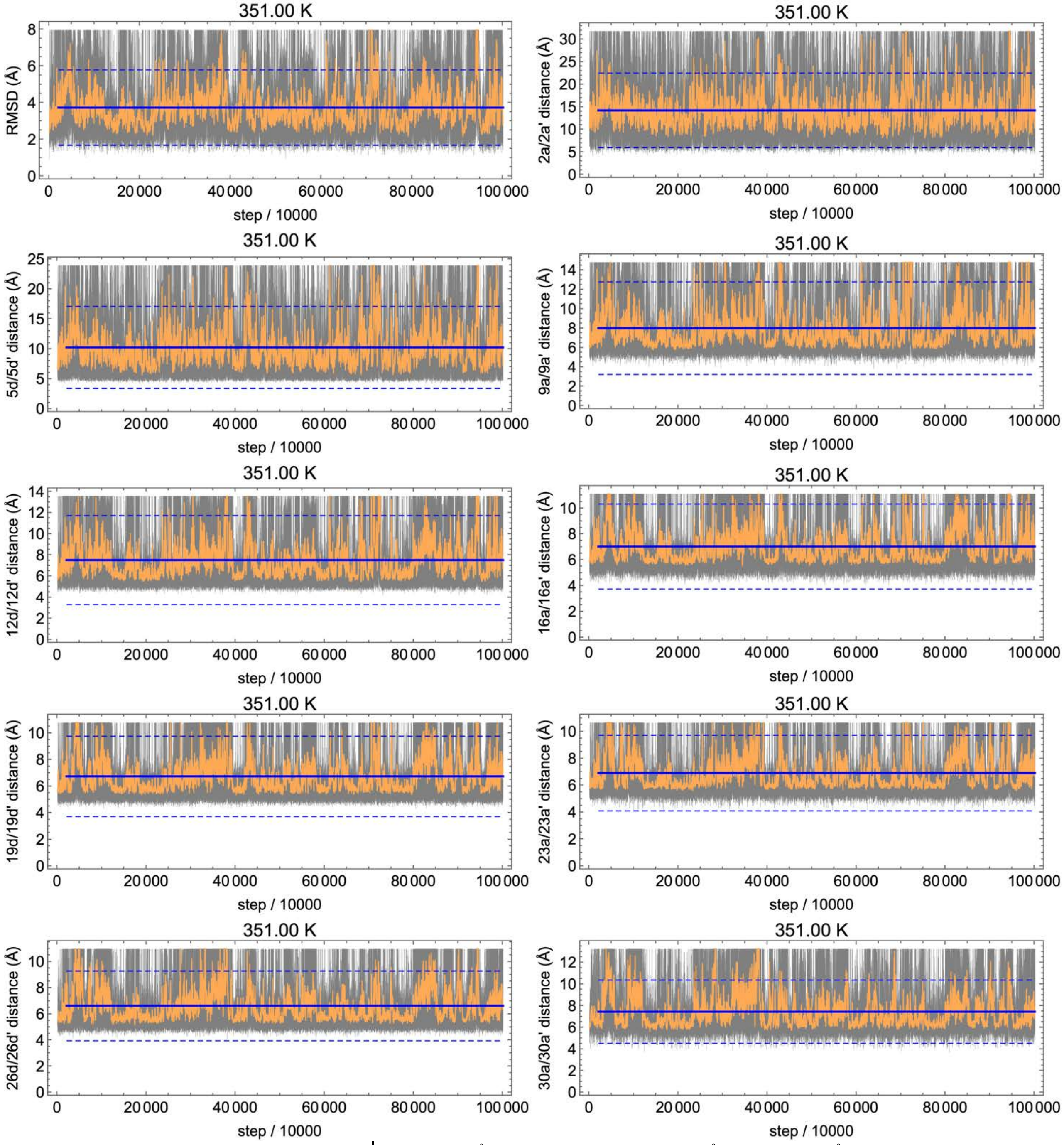

Measure at 351.00 K	Average (Å)	Standard Deviation (Å)	Slope (Å / μs)
RMSD	3.76241	2.05448	0.00765119
5d/5d' distance	10.3096	6.84404	0.38653
12d/12d' distance	7.56895	4.20489	0.230587
19d/19d' distance	6.77429	3.02453	0.0715092
26d/26d' distance	6.6504	2.66726	−0.0611159
2a/2a' distance	14.3363	8.26483	0.135696
9a/9a' distance	8.04981	4.78917	0.330943
16a/16a' distance	7.06663	3.29413	0.102406
23a/23a' distance	6.94565	2.81482	0.0144091
30a/30a' distance	7.48961	2.92	−0.328325
Core Distance	8.35459	5.26449	

Figure S327. Plots of RMSD and residue packing distances for T-REMD simulations of variant **7f/10b’-xx** at 351.00 K.

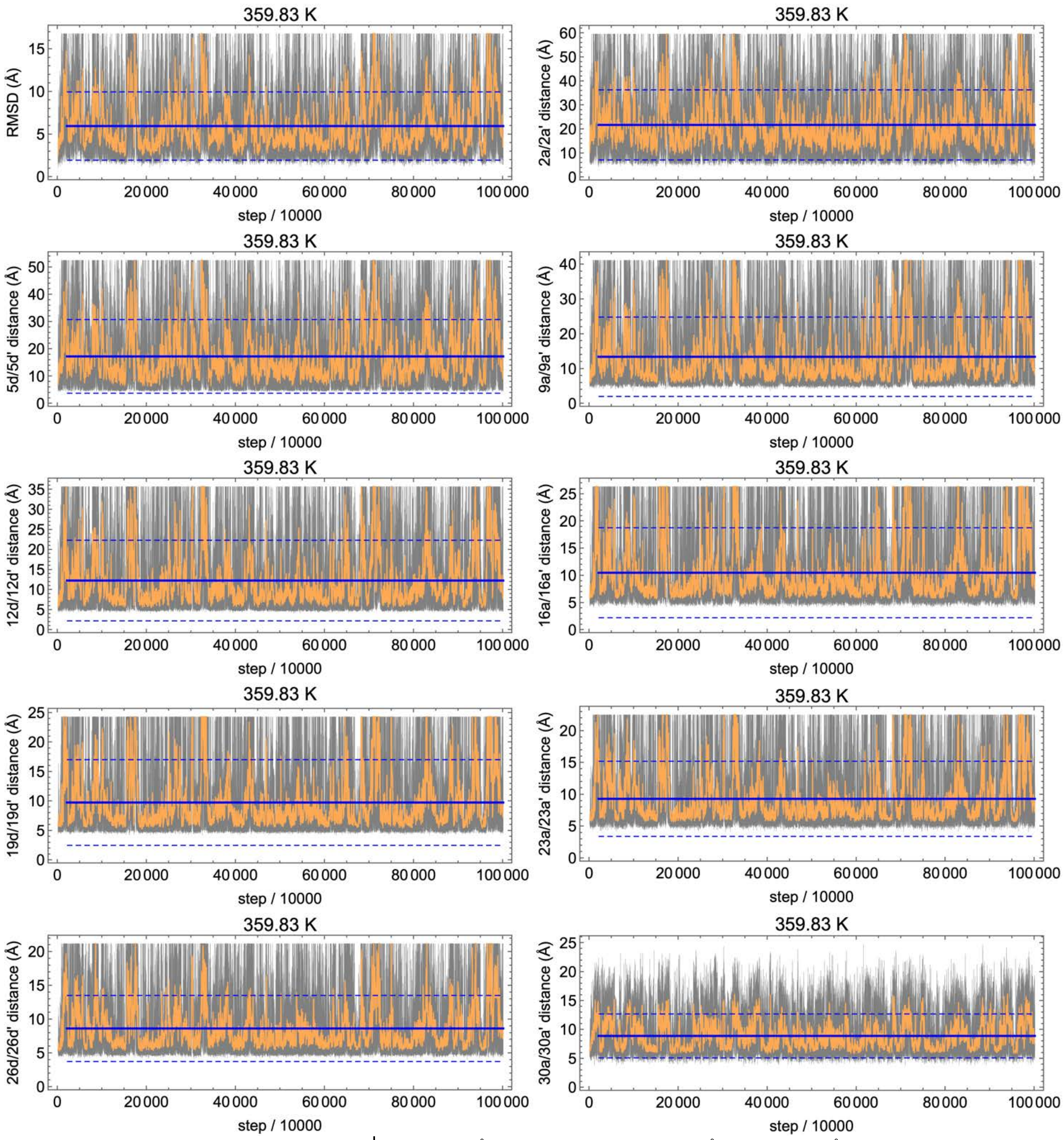

Measure at 359.83 K	Average (Å)	Standard Deviation (Å)	Slope (Å / μs)
RMSD	6.01245	4.00502	0.312759
5d/5d' distance	17.4344	13.5034	1.2736
12d/12d' distance	12.3981	10.0488	0.949275
19d/19d' distance	9.83606	7.27208	0.633451
26d/26d' distance	8.71154	4.88695	0.326742
2a/2a' distance	21.9412	14.5865	1.02124
9a/9a' distance	13.5581	11.4024	1.15834
16a/16a' distance	10.5872	8.27344	0.715938
23a/23a' distance	9.3819	5.89769	0.452832
30a/30a' distance	8.97931	3.80405	−0.0316374
Core Distance	12.5364	10.4477	

Figure S328. Plots of RMSD and residue packing distances for T-REMD simulations of variant **7f/10b'-xx** at 359.83 K.

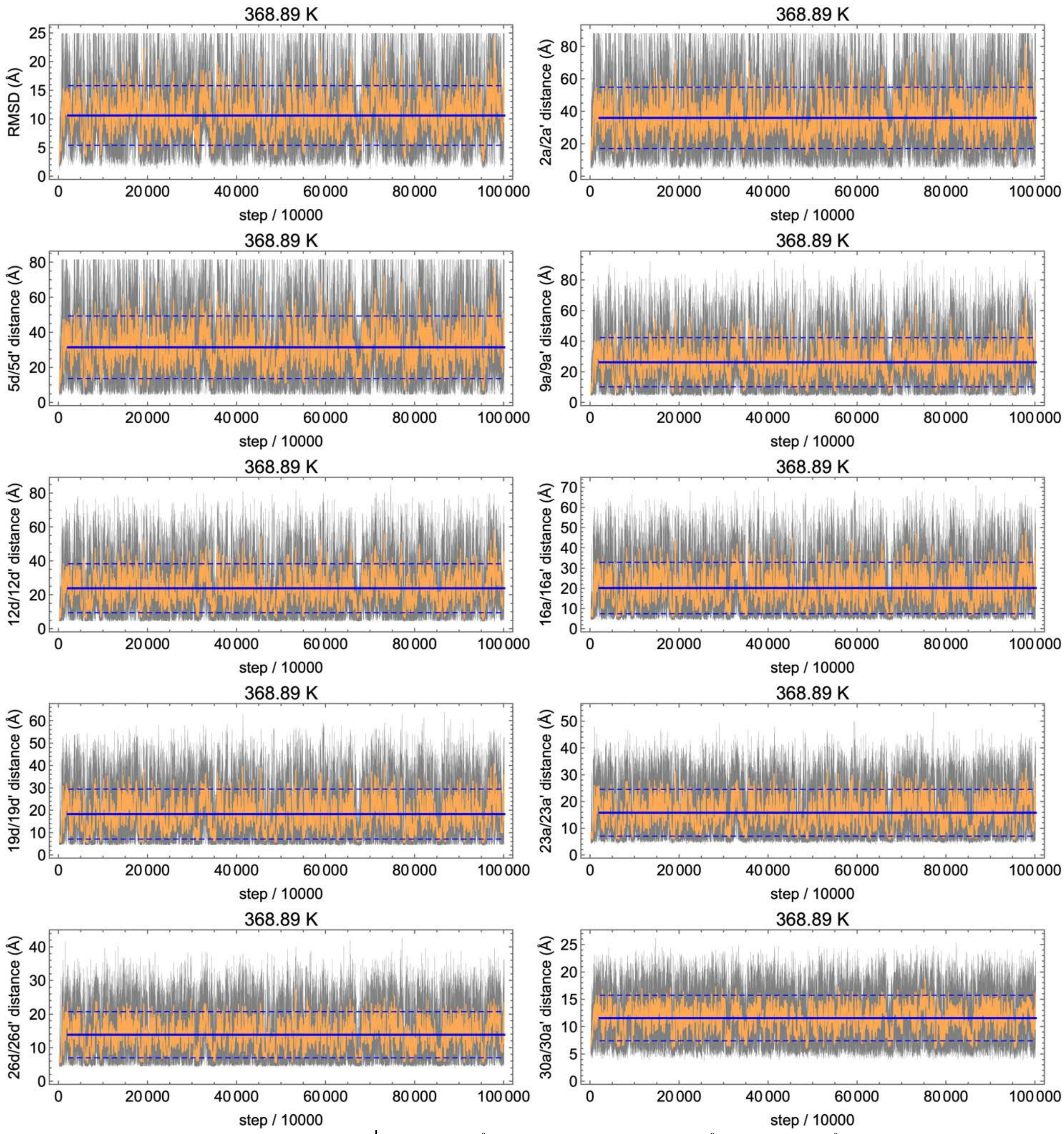

Measure at 368.89 K	Average (Å)	Standard Deviation (Å)	Slope (Å / μs)
RMSD	10.7145	5.19735	0.283206
5d/5d' distance	31.8754	17.8531	0.97279
12d/12d' distance	24.2737	14.4281	0.735905
19d/19d' distance	18.5762	11.1624	0.531576
26d/26d' distance	14.0385	6.86411	0.316122
2a/2a' distance	36.3811	18.9094	0.98399
9a/9a' distance	26.7038	16.0451	0.850561
16a/16a' distance	20.4888	12.7287	0.606489
23a/23a' distance	16.0452	8.74173	0.407567
30a/30a' distance	11.6883	4.17386	0.057849
Core Distance	22.2301	15.3472	

Figure S329. Plots of RMSD and residue packing distances for T-REMD simulations of variant **7f/10b'-xx** at 368.89 K.

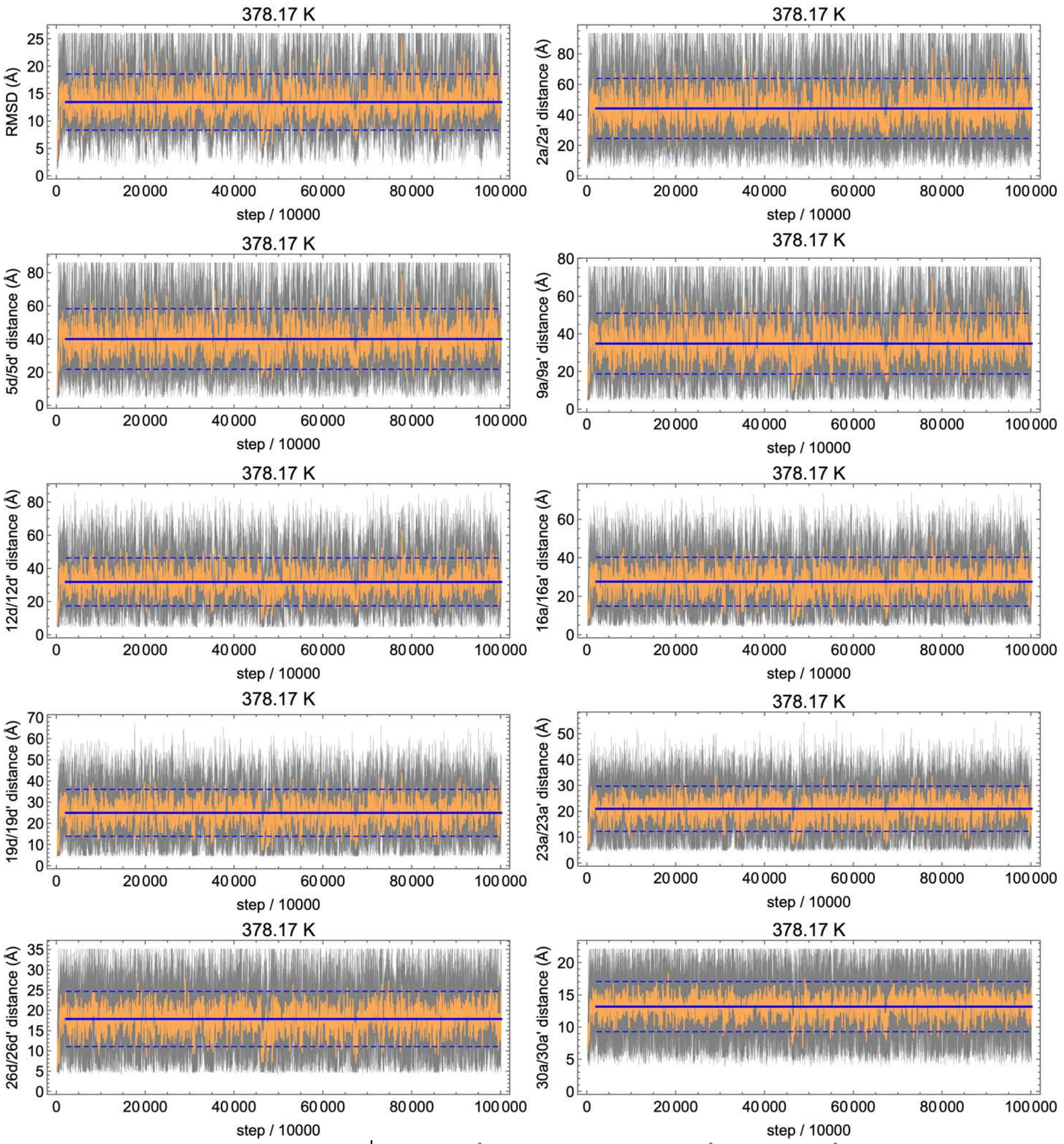

Measure at 378.17 K	Average (Å)	Standard Deviation (Å)	Slope (Å / μs)
RMSD	13.5829	5.11812	0.231016
5d/5d' distance	40.3708	18.2474	0.718368
12d/12d' distance	32.2191	14.4517	0.537017
19d/19d' distance	25.2822	11.0783	0.32754
26d/26d' distance	18.0315	6.81367	0.240051
2a/2a' distance	44.6391	19.6951	0.687839
9a/9a' distance	35.132	16.1249	0.66177
16a/16a' distance	27.9285	12.6933	0.406449
23a/23a' distance	21.2307	8.71831	0.214354
30a/30a' distance	13.2717	3.89463	0.0424975
Core Distance	28.6784	16.5834	

Figure S330. Plots of RMSD and residue packing distances for T-REMD simulations of variant **7f/10b'-xx** at 378.17 K.

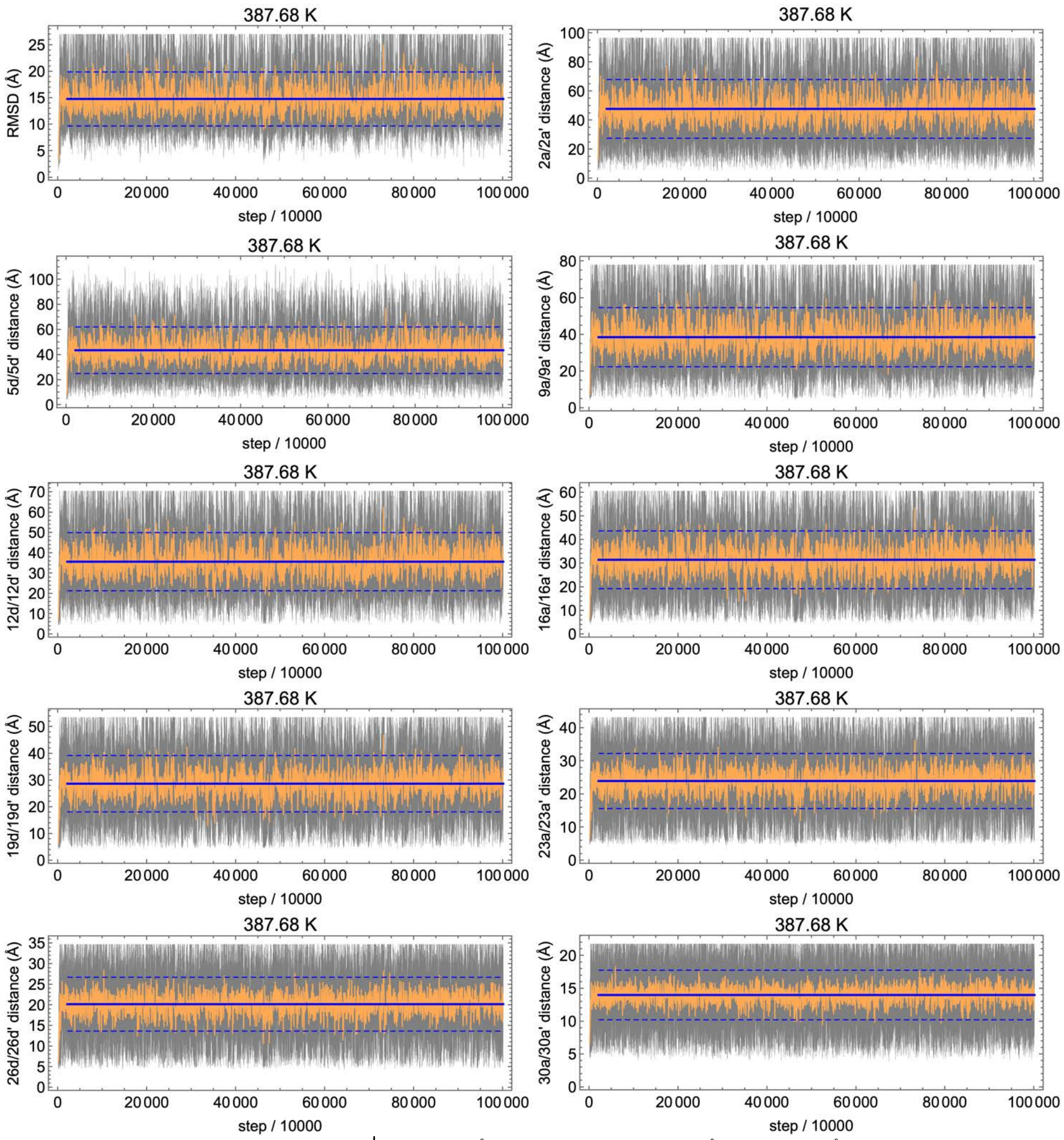

Measure at 387.68 K	Average (Å)	Standard Deviation (Å)	Slope (Å / μs)
RMSD	14.8874	5.0958	0.235433
5d/5d' distance	43.9294	18.5588	0.600233
12d/12d' distance	35.9083	14.3141	0.59283
19d/19d' distance	28.8577	10.5459	0.480502
26d/26d' distance	20.318	6.53564	0.315891
2a/2a' distance	48.1311	20.1862	0.538307
9a/9a' distance	38.8152	16.1144	0.636872
16a/16a' distance	31.6737	12.2182	0.579169
23a/23a' distance	24.1081	8.30879	0.366525
30a/30a' distance	14.073	3.76955	0.0571482
Core Distance	31.7572	16.9861	

Figure S331. Plots of RMSD and residue packing distances for T-REMD simulations of variant **7f/10b'-xx** at 387.68 K.

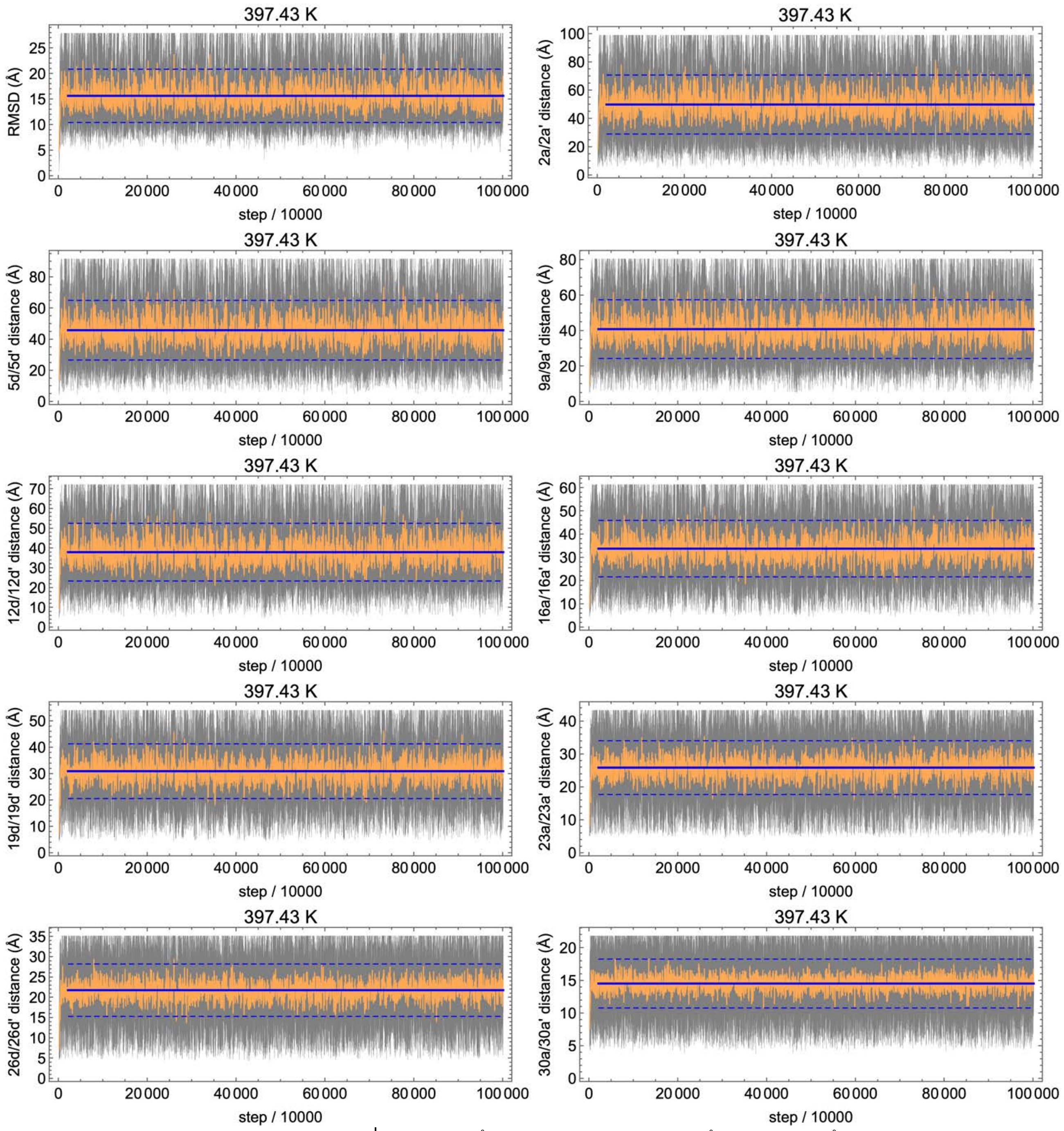

Measure at 397.43 K	Average (Å)	Standard Deviation (Å)	Slope (Å / μs)
RMSD	15.7579	5.2092	0.153525
5d/5d' distance	46.1633	19.145	0.392888
12d/12d' distance	38.2505	14.5946	0.347619
19d/19d' distance	31.1649	10.3689	0.271849
26d/26d' distance	21.9029	6.45523	0.190399
2a/2a' distance	50.3073	20.8749	0.348717
9a/9a' distance	41.1784	16.5601	0.415463
16a/16a' distance	34.0478	12.1482	0.31588
23a/23a' distance	26.0587	8.17015	0.193556
30a/30a' distance	14.6107	3.74025	0.00555571
Core Distance	33.7427	17.4588	

Figure S332. Plots of RMSD and residue packing distances for T-REMD simulations of variant **7f/10b'-xx** at 397.43 K.

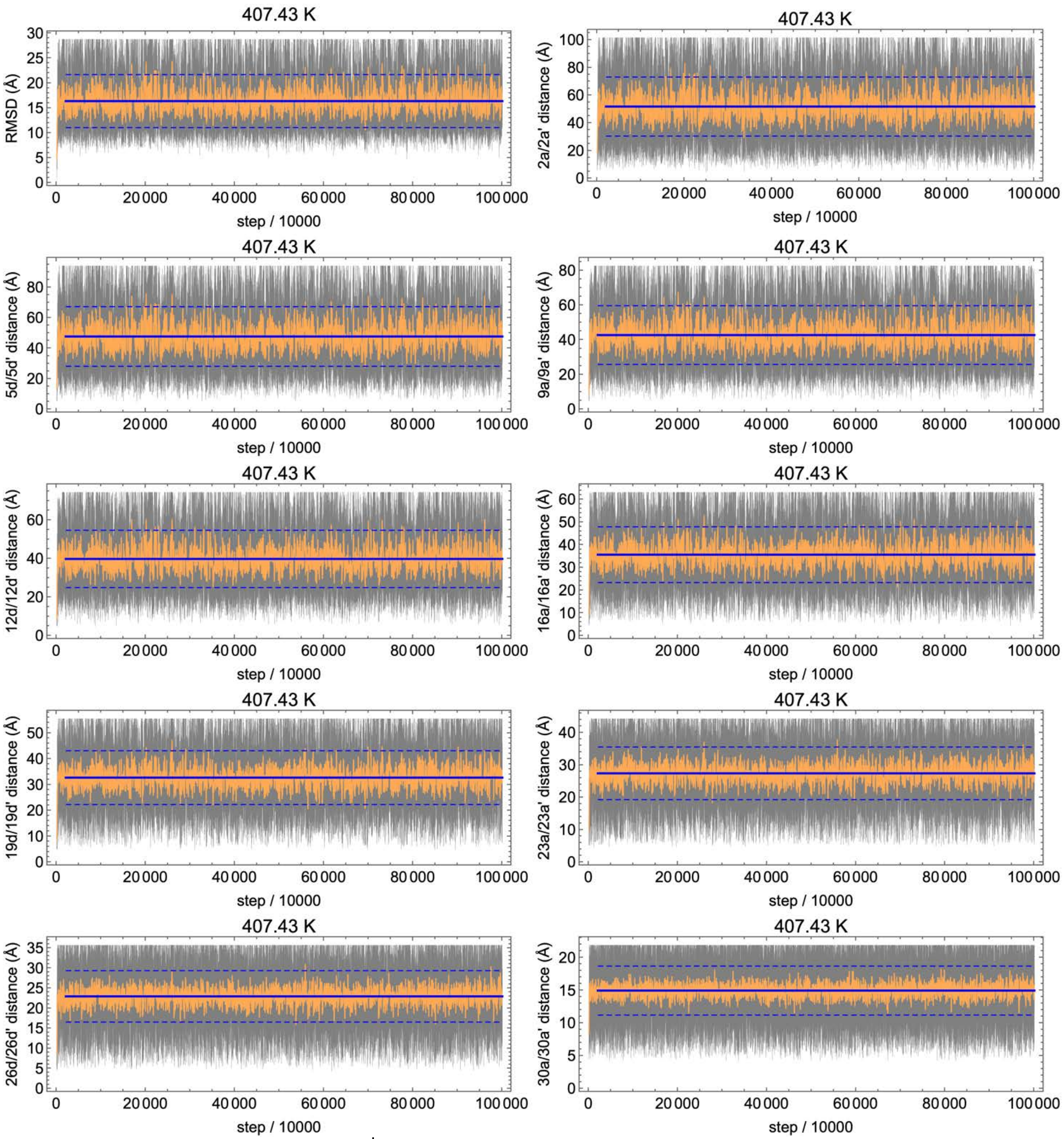

Measure at 407.43 K	Average (Å)	Standard Deviation (Å)	Slope (Å / μs)
RMSD	16.4448	5.31354	0.194809
5d/5d' distance	47.9501	19.5588	0.590588
12d/12d' distance	40.0106	14.8763	0.412296
19d/19d' distance	32.8545	10.4203	0.25224
26d/26d' distance	23.0455	6.3965	0.13022
2a/2a' distance	52.0943	21.3165	0.579877
9a/9a' distance	42.9604	16.9405	0.477129
16a/16a' distance	35.8226	12.2681	0.34453
23a/23a' distance	27.5341	8.1361	0.154598
30a/30a' distance	15.0185	3.74189	0.0435457
Core Distance	35.2545	17.8774	

Figure S333. Plots of RMSD and residue packing distances for T-REMD simulations of variant **7f/10b'-xx** at 407.43 K.

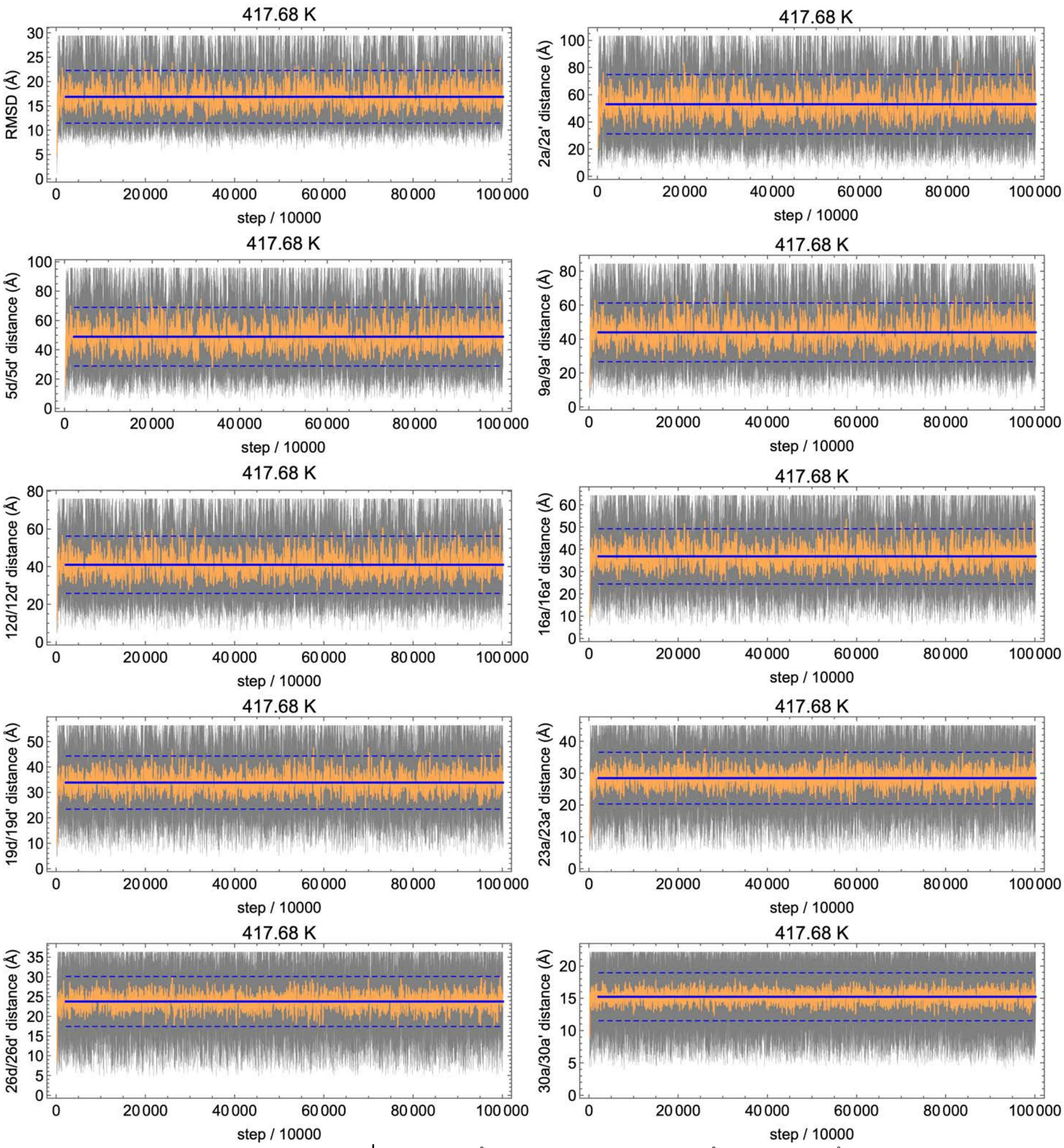

Measure at 417.68 K	Average (Å)	Standard Deviation (Å)	Slope (Å / μs)
RMSD	17.0002	5.40604	0.17982
5d/5d' distance	49.3875	19.9956	0.543666
12d/12d' distance	41.3652	15.2063	0.448224
19d/19d' distance	34.1382	10.4557	0.244752
26d/26d' distance	23.9333	6.33907	0.0459025
2a/2a' distance	53.5372	21.8416	0.540713
9a/9a' distance	44.3732	17.3156	0.503049
16a/16a' distance	37.1546	12.387	0.315615
23a/23a' distance	28.653	8.12275	0.0730342
30a/30a' distance	15.3332	3.71093	−0.011243
Core Distance	36.4306	18.2761	

Figure S334. Plots of RMSD and residue packing distances for T-REMD simulations of variant **7f/10b'-xx** at 417.68 K.

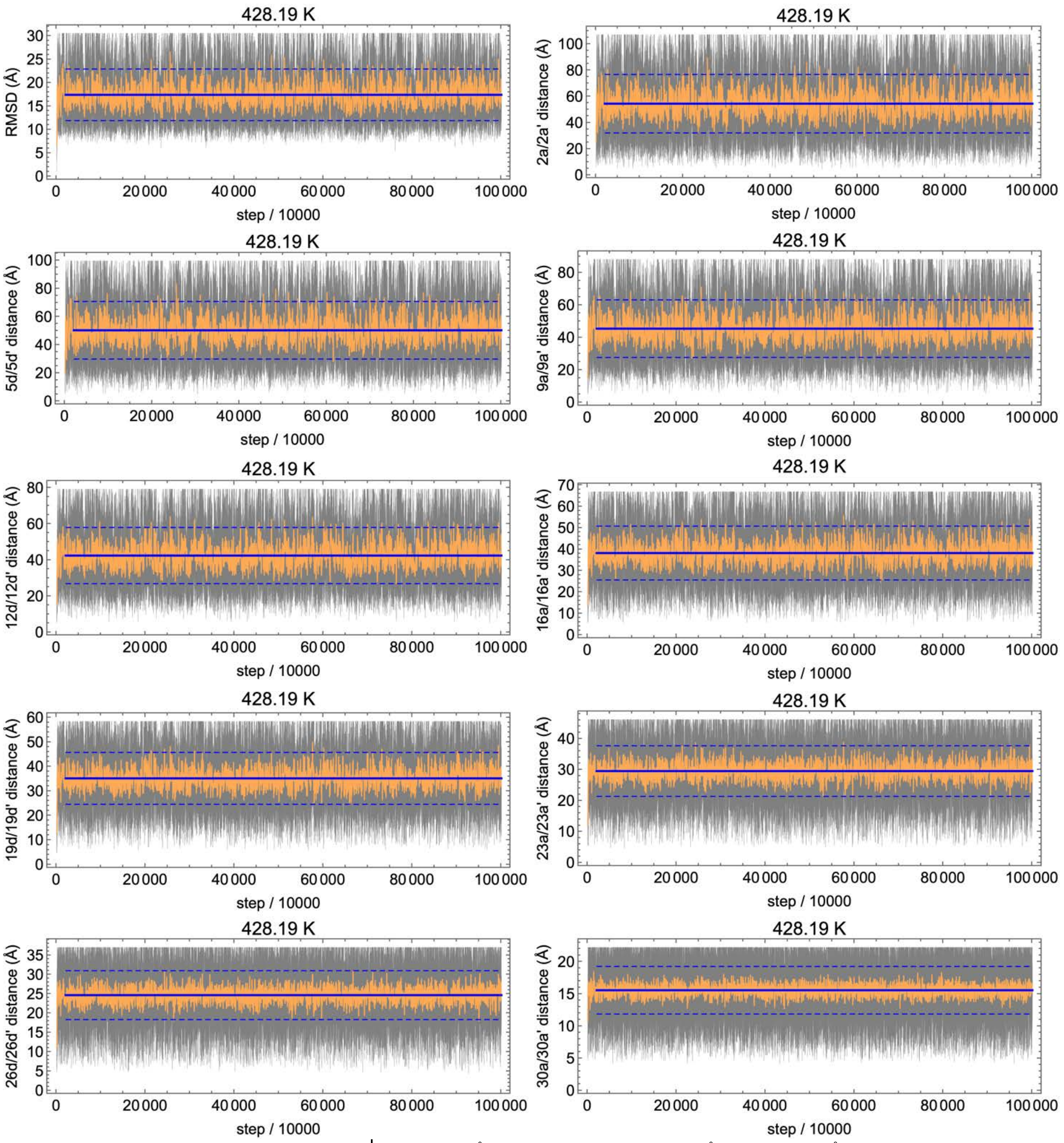

Measure at 428.19 K	Average (Å)	Standard Deviation (Å)	Slope (Å / μs)
RMSD	17.5	5.50973	0.255152
5d/5d' distance	50.6022	20.4453	0.842632
12d/12d' distance	42.635	15.5576	0.706244
19d/19d' distance	35.3148	10.5916	0.337266
26d/26d' distance	24.7661	6.3218	0.0791562
2a/2a' distance	54.7458	22.321	0.903918
9a/9a' distance	45.6515	17.73	0.719203
16a/16a' distance	38.4003	12.5878	0.463278
23a/23a' distance	29.6607	8.16786	0.143031
30a/30a' distance	15.6279	3.69688	−0.0145215
Core Distance	37.4894	18.658	

Figure S335. Plots of RMSD and residue packing distances for T-REMD simulations of variant **7f/10b'-xx** at 428.19 K.

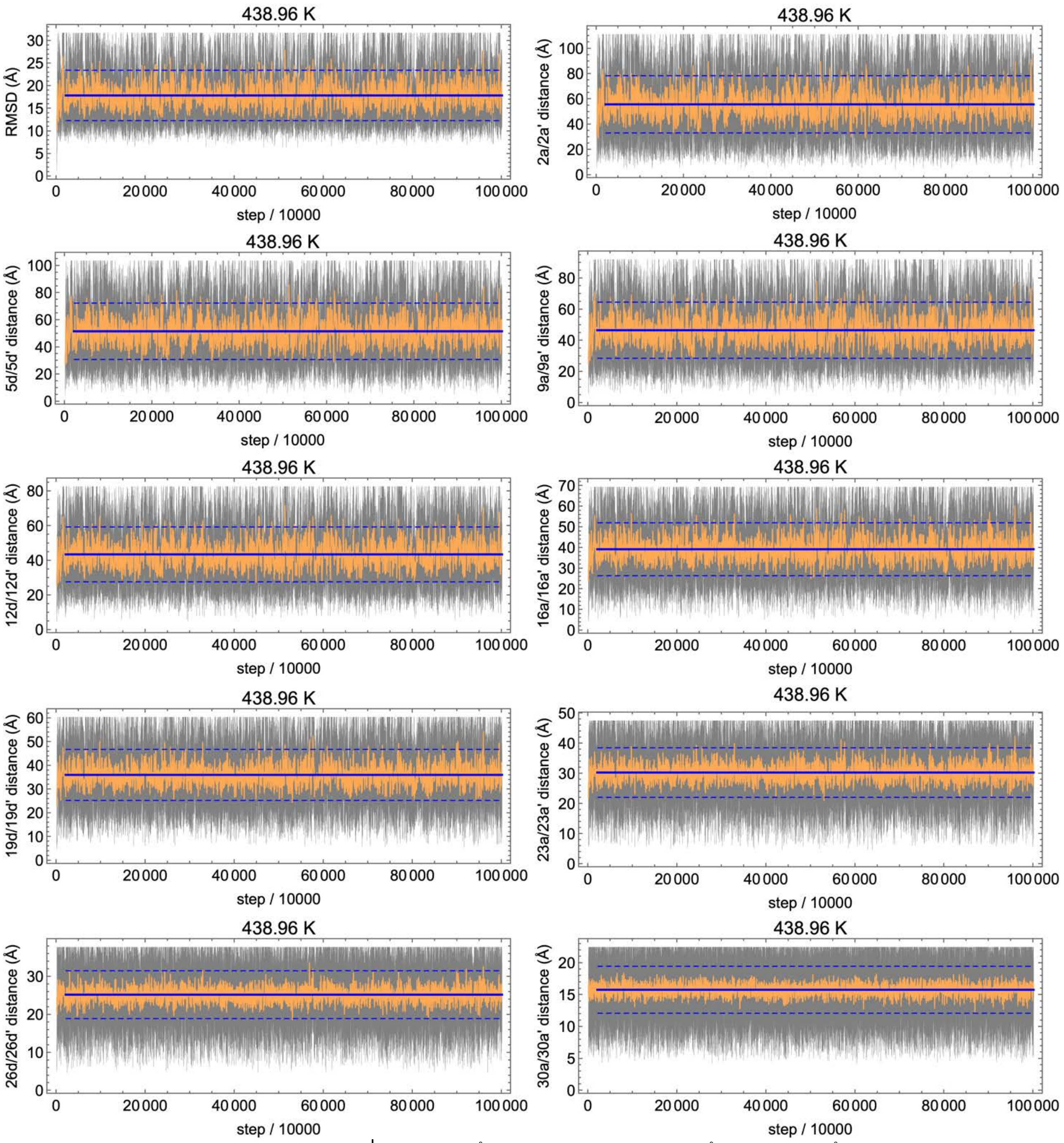

Measure at 438.96 K	Average (Å)	Standard Deviation (Å)	Slope (Å / μs)
RMSD	17.9825	5.5788	0.187511
5d/5d' distance	51.9451	20.7701	0.524573
12d/12d' distance	43.7688	15.8288	0.616285
19d/19d' distance	36.2515	10.7726	0.27376
26d/26d' distance	25.3674	6.30121	0.0693786
2a/2a' distance	56.158	22.6346	0.424166
9a/9a' distance	46.8703	18.0249	0.600644
16a/16a' distance	39.4523	12.8038	0.418616
23a/23a' distance	30.4775	8.21908	0.122721
30a/30a' distance	15.8561	3.68042	−0.0444391
Core Distance	38.4608	19.0492	

Figure S336. Plots of RMSD and residue packing distances for T-REMD simulations of variant **7f/10b’-xx** at 438.96 K.

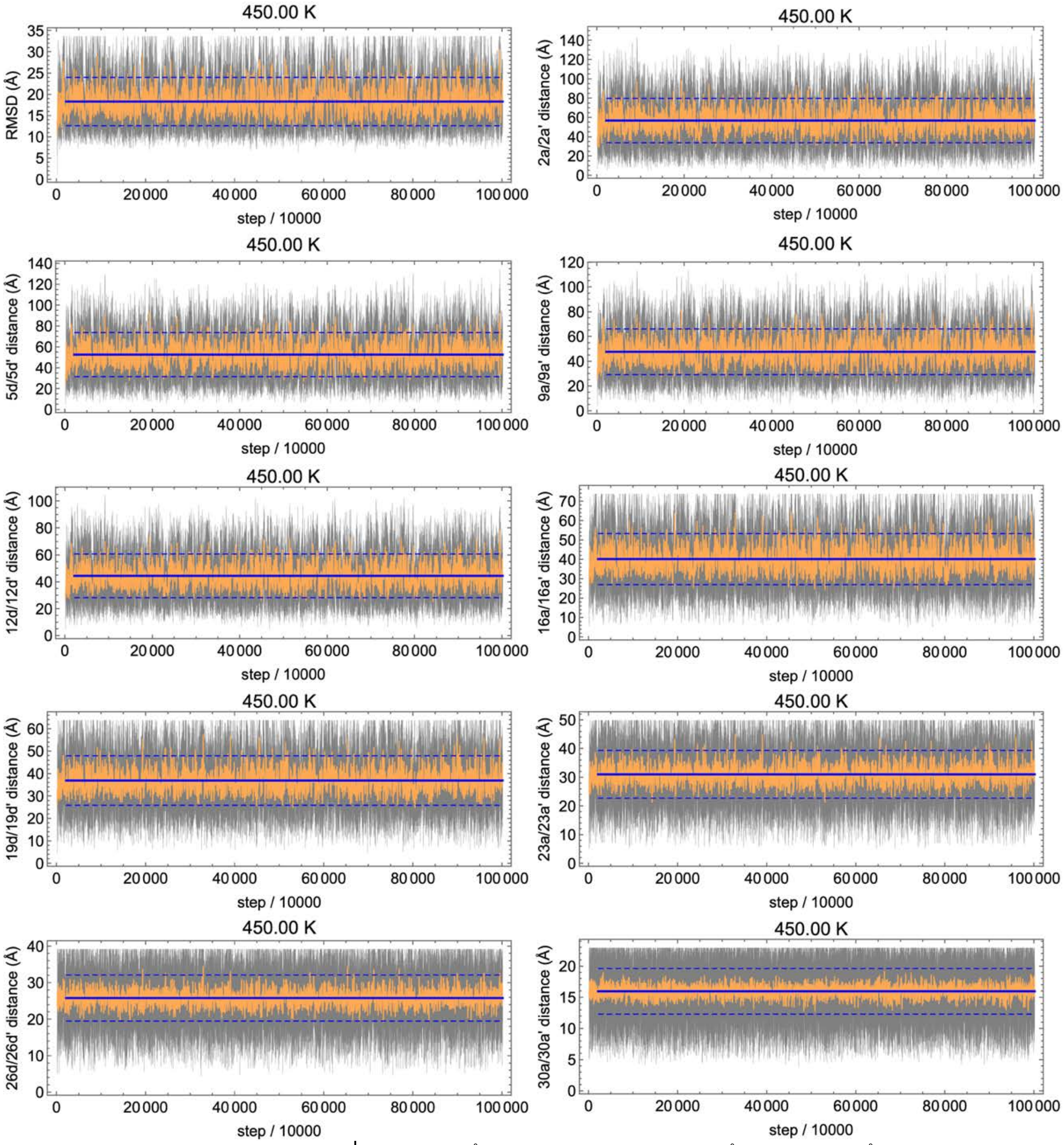

Measure at 450.00 K	Average (Å)	Standard Deviation (Å)	Slope (Å / μs)
RMSD	18.459	5.69268	0.196312
5d/5d' distance	53.2412	21.1317	0.509306
12d/12d' distance	44.9036	16.2493	0.63053
19d/19d' distance	37.2022	11.0114	0.422617
26d/26d' distance	25.9601	6.30901	0.128861
2a/2a' distance	57.4686	23.0122	0.346779
9a/9a' distance	48.116	18.4515	0.607738
16a/16a' distance	40.4674	13.1496	0.491077
23a/23a' distance	31.2395	8.32486	0.2251
30a/30a' distance	16.0633	3.66055	0.0108865
Core Distance	39.4069	19.4918	

Figure S337. Plots of RMSD and residue packing distances for T-REMD simulations of variant **7f/10b’-xx** at 450.00 K.

(return to Table of Contents)

s7f/10b'-x4x

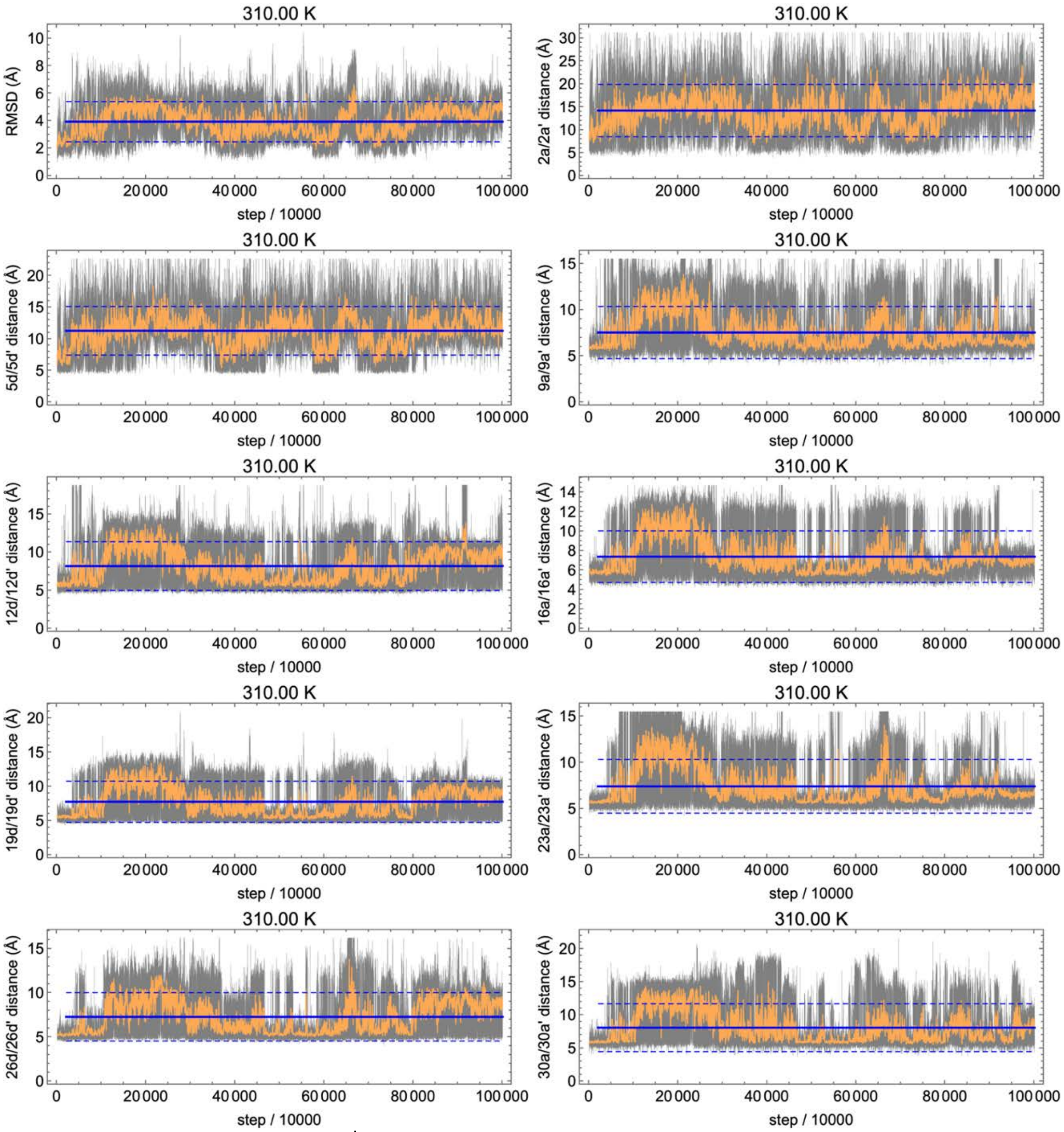

Measure at 310.00 K	Average (Å)	Standard Deviation (Å)	Slope (Å / μs)
RMSD	3.96179	1.46315	0.100594
5d/5d' distance	11.3413	3.84586	0.275231
12d/12d' distance	8.24099	3.20352	−0.0129631
19d/19d' distance	7.81696	3.00412	−0.204693
26d/26d' distance	7.32285	2.73793	0.414322
2a/2a' distance	14.2852	5.69699	1.18903
9a/9a' distance	7.59253	2.82269	−1.22359
16a/16a' distance	7.42143	2.64975	−1.01739
23a/23a' distance	7.45938	2.91039	−1.34786
30a/30a' distance	8.14395	3.60932	−1.41778
Core Distance	8.84717	4.1645	

Figure S338. Plots of RMSD and residue packing distances for T-REMD simulations of variant **s7f/10b'-x4x** at 310.00 K.

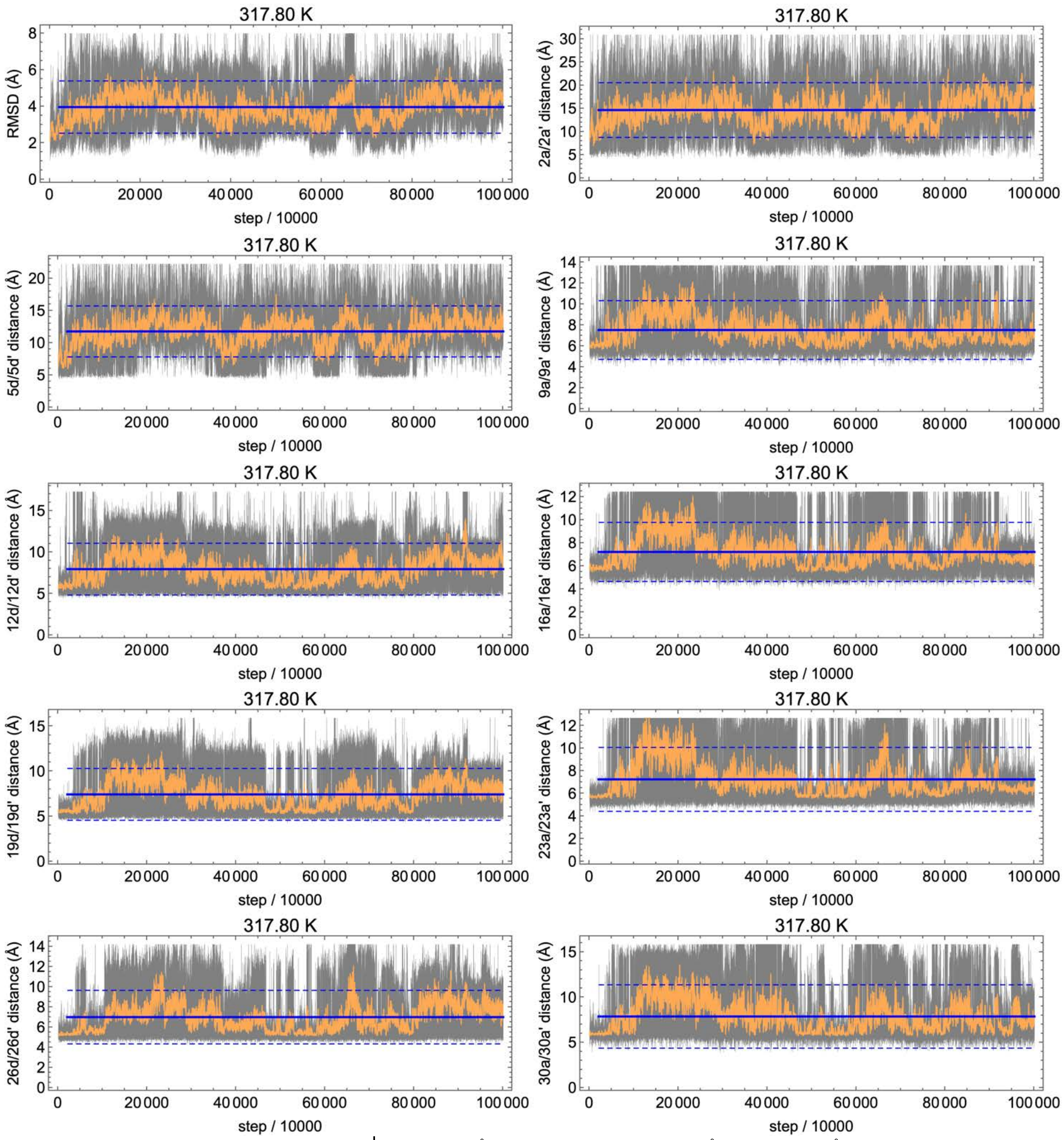

Measure at 317.80 K	Average (Å)	Standard Deviation (Å)	Slope (Å / μs)
RMSD	3.98187	1.43114	0.110889
5d/5d' distance	11.8271	3.95343	0.40555
12d/12d' distance	7.99778	3.11834	0.178078
19d/19d' distance	7.47109	2.87593	−0.0567665
26d/26d' distance	7.03237	2.65369	0.458462
2a/2a' distance	14.7488	5.90533	1.10938
9a/9a' distance	7.56354	2.80775	−0.727389
16a/16a' distance	7.24653	2.56486	−0.673861
23a/23a' distance	7.28236	2.82312	−0.95988
30a/30a' distance	7.91467	3.50543	−0.935072
Core Distance	8.78714	4.31241	

Figure S339. Plots of RMSD and residue packing distances for T-REMD simulations of variant **s7f/10b'-x4x** at 317.80 K.

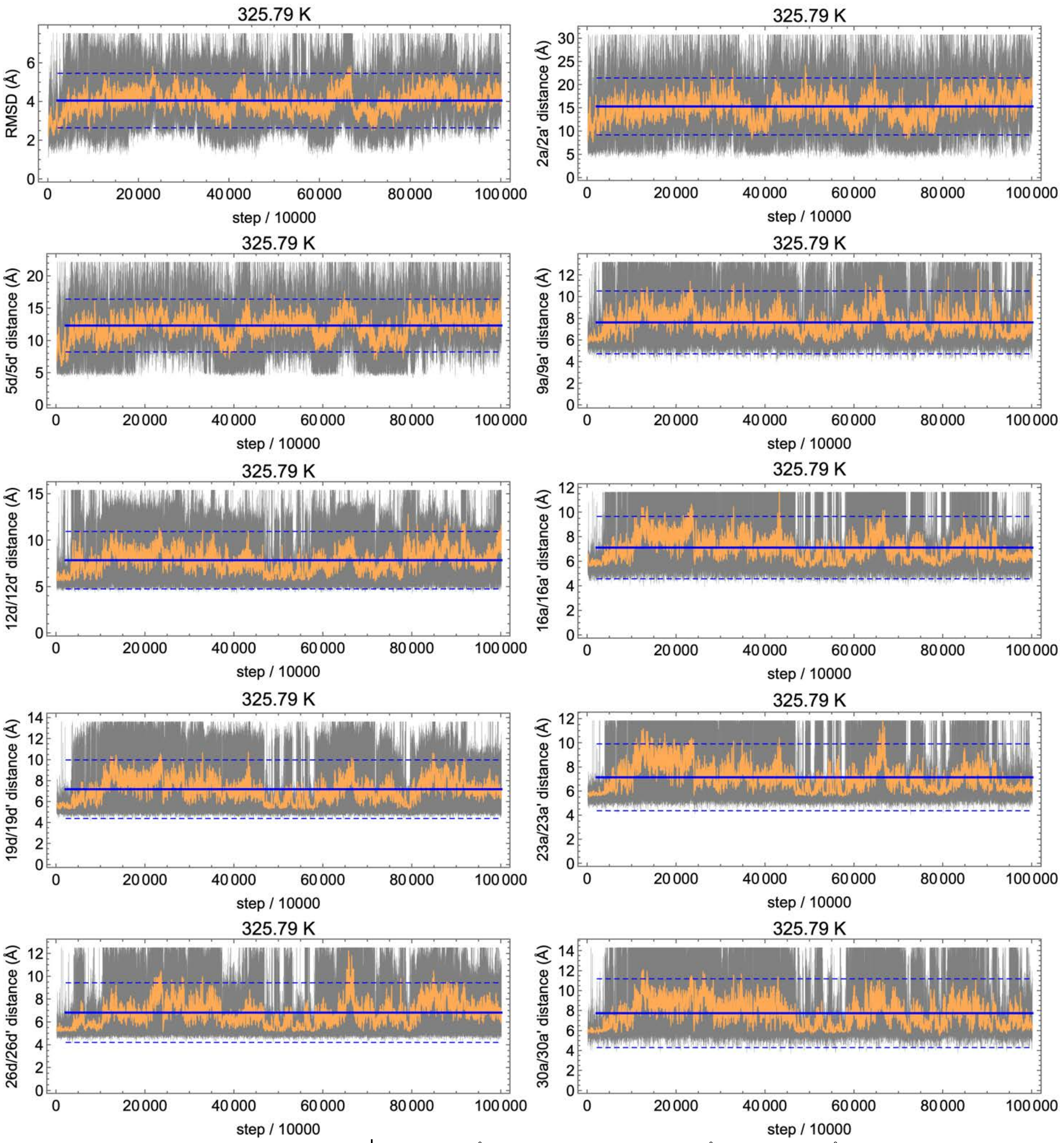

Measure at 325.79 K	Average (Å)	Standard Deviation (Å)	Slope (Å / μs)
RMSD	4.08194	1.41078	0.124705
5d/5d' distance	12.4065	4.09056	0.569479
12d/12d' distance	7.91137	3.09696	0.234575
19d/19d' distance	7.23753	2.79313	−0.0497235
26d/26d' distance	6.87154	2.61287	0.371112
2a/2a' distance	15.4432	6.13168	0.99397
9a/9a' distance	7.68024	2.90053	−0.339039
16a/16a' distance	7.16684	2.53975	−0.45369
23a/23a' distance	7.19571	2.77287	−0.649462
30a/30a' distance	7.80385	3.44343	−0.591192
Core Distance	8.85742	4.52775	

Figure S340. Plots of RMSD and residue packing distances for T-REMD simulations of variant **s7f/10b'-x4x** at 325.79 K.

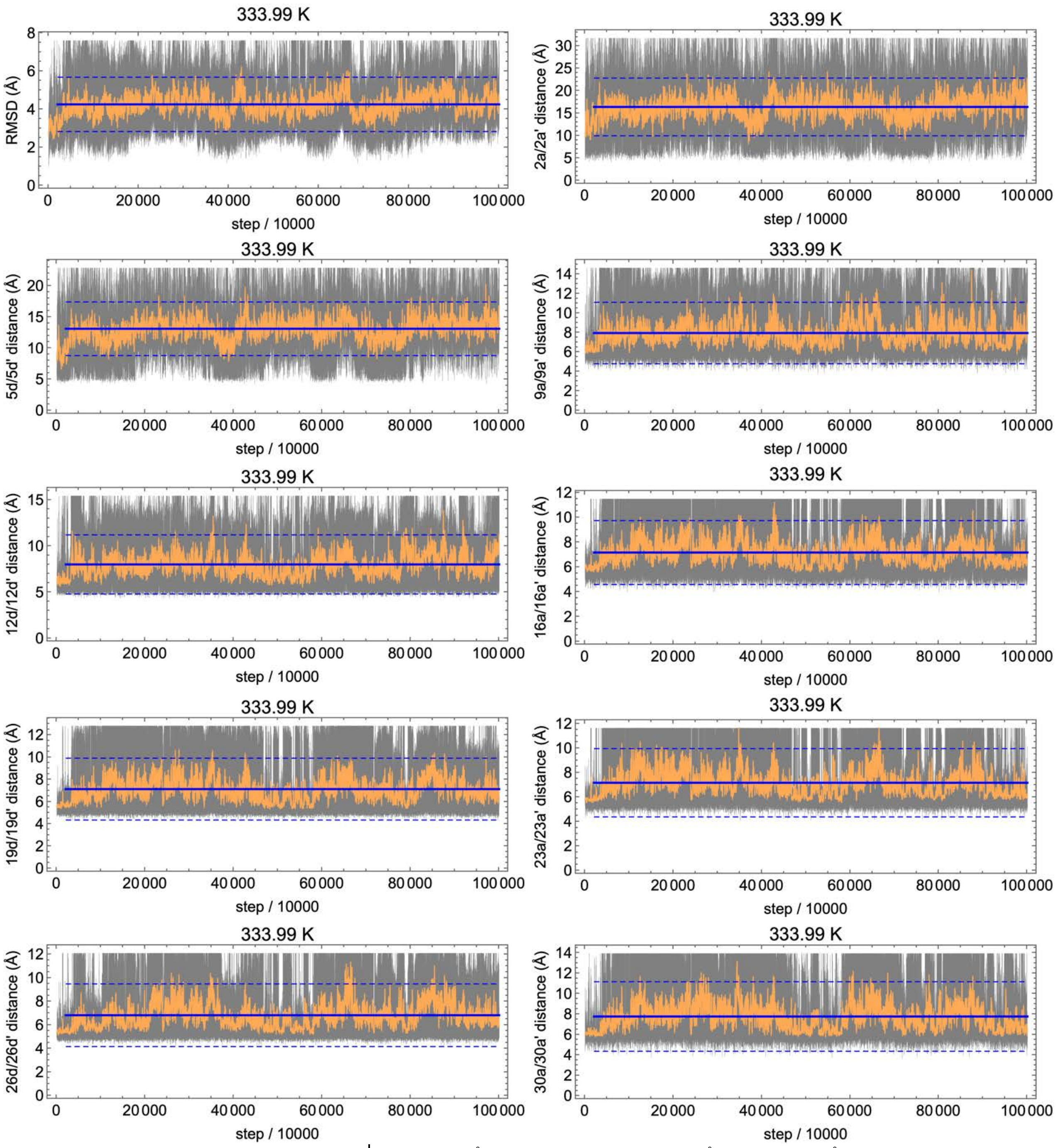

Measure at 333.99 K	Average (Å)	Standard Deviation (Å)	Slope (Å / μs)
RMSD	4.27357	1.42507	0.157548
5d/5d' distance	13.1642	4.30563	0.649233
12d/12d' distance	8.04357	3.20338	0.320017
19d/19d' distance	7.16088	2.78275	0.00664936
26d/26d' distance	6.85385	2.65801	0.323941
2a/2a' distance	16.4817	6.43306	0.825918
9a/9a' distance	7.98406	3.15551	0.0227408
16a/16a' distance	7.1973	2.57729	−0.246367
23a/23a' distance	7.19821	2.79068	−0.345911
30a/30a' distance	7.79821	3.4007	−0.321792
Core Distance	9.098	4.85398	

Figure S341. Plots of RMSD and residue packing distances for T-REMD simulations of variant **s7f/10b’-x4x** at 333.99 K.

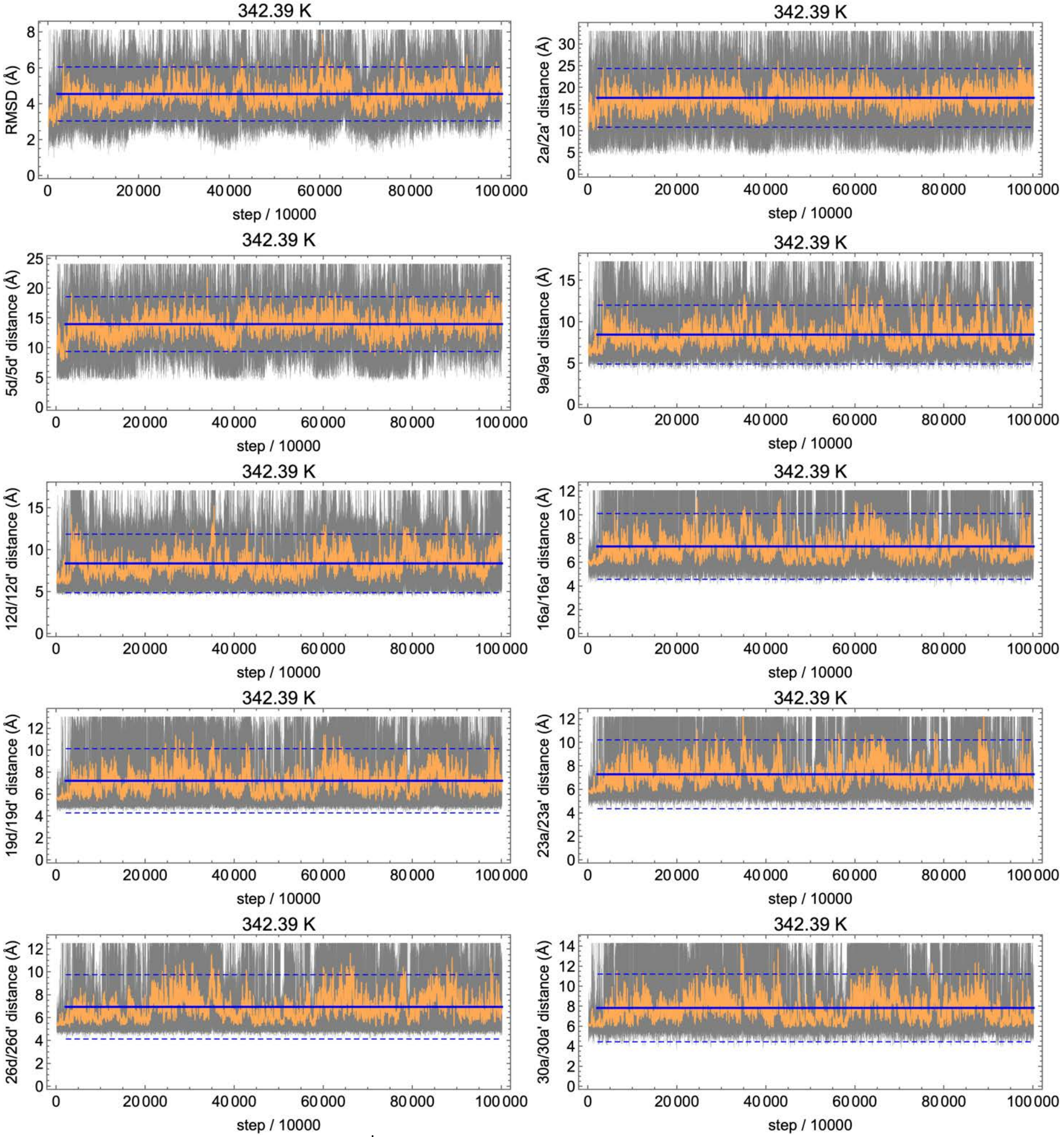

Measure at 342.39 K	Average (Å)	Standard Deviation (Å)	Slope (Å / μs)
RMSD	4.57682	1.50491	0.225519
5d/5d' distance	14.0653	4.6053	0.65668
12d/12d' distance	8.42947	3.49117	0.463125
19d/19d' distance	7.27052	2.92876	0.155981
26d/26d' distance	6.99402	2.80959	0.365395
2a/2a' distance	17.7349	6.76666	0.731185
9a/9a' distance	8.51287	3.55146	0.350517
16a/16a' distance	7.38405	2.76809	−0.0245279
23a/23a' distance	7.33758	2.93095	−0.0547278
30a/30a' distance	7.88981	3.38399	−0.0410187
Core Distance	9.51317	5.26775	

Figure S342. Plots of RMSD and residue packing distances for T-REMD simulations of variant **s7f/10b’-x4x** at 342.39 K.

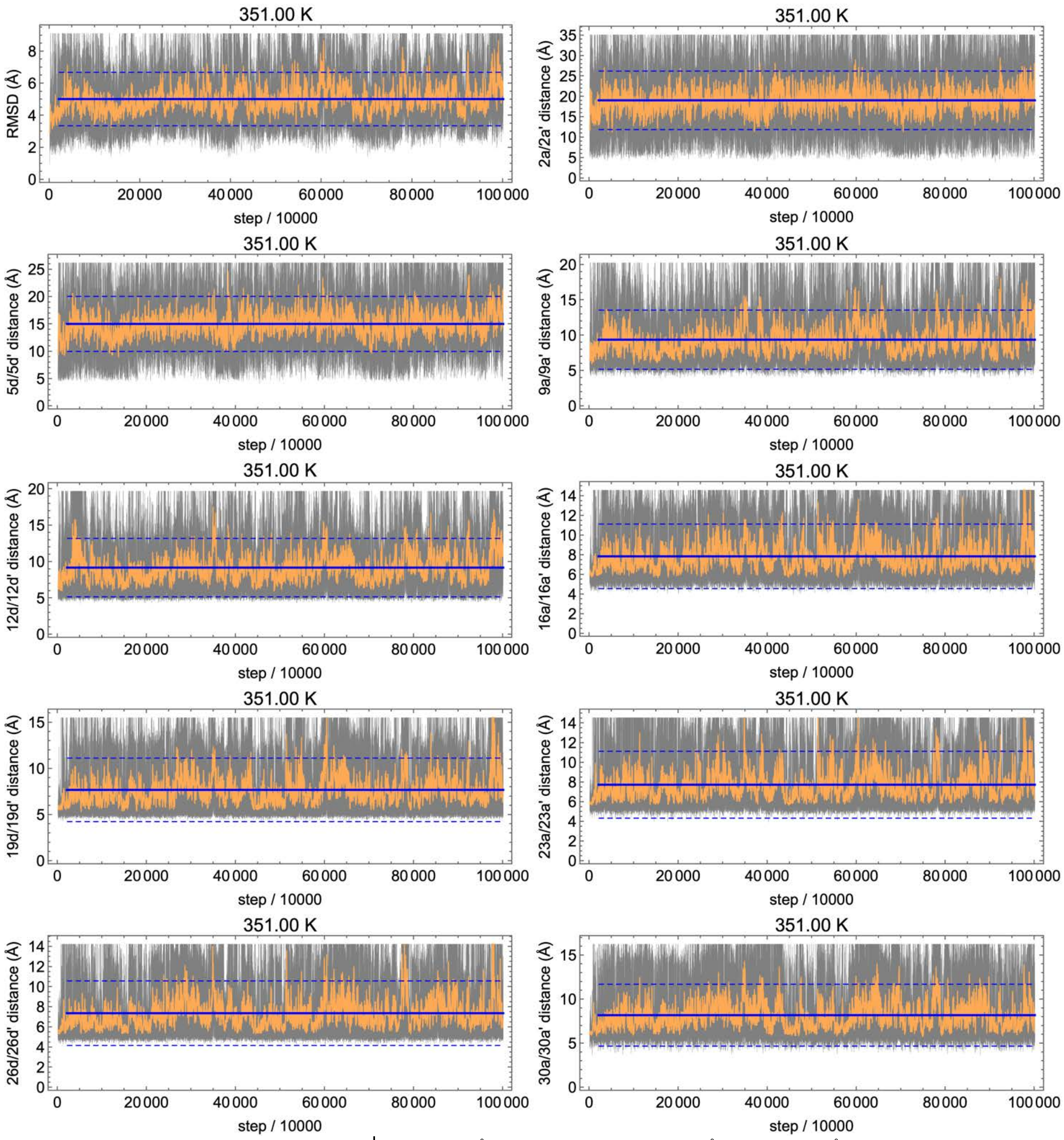

Measure at 351.00 K	Average (Å)	Standard Deviation (Å)	Slope (Å / μs)
RMSD	5.04754	1.6671	0.319199
5d/5d' distance	15.113	5.03842	0.693917
12d/12d' distance	9.24638	4.01933	0.685857
19d/19d' distance	7.74165	3.43841	0.392586
26d/26d' distance	7.42293	3.20735	0.500932
2a/2a' distance	19.1781	7.17151	0.596249
9a/9a' distance	9.44987	4.18208	0.71959
16a/16a' distance	7.89775	3.28472	0.276598
23a/23a' distance	7.78641	3.39622	0.267606
30a/30a' distance	8.24693	3.50888	0.153123
Core Distance	10.2315	5.79376	

Figure S343. Plots of RMSD and residue packing distances for T-REMD simulations of variant **s7f/10b’-x4x** at 351.00 K.

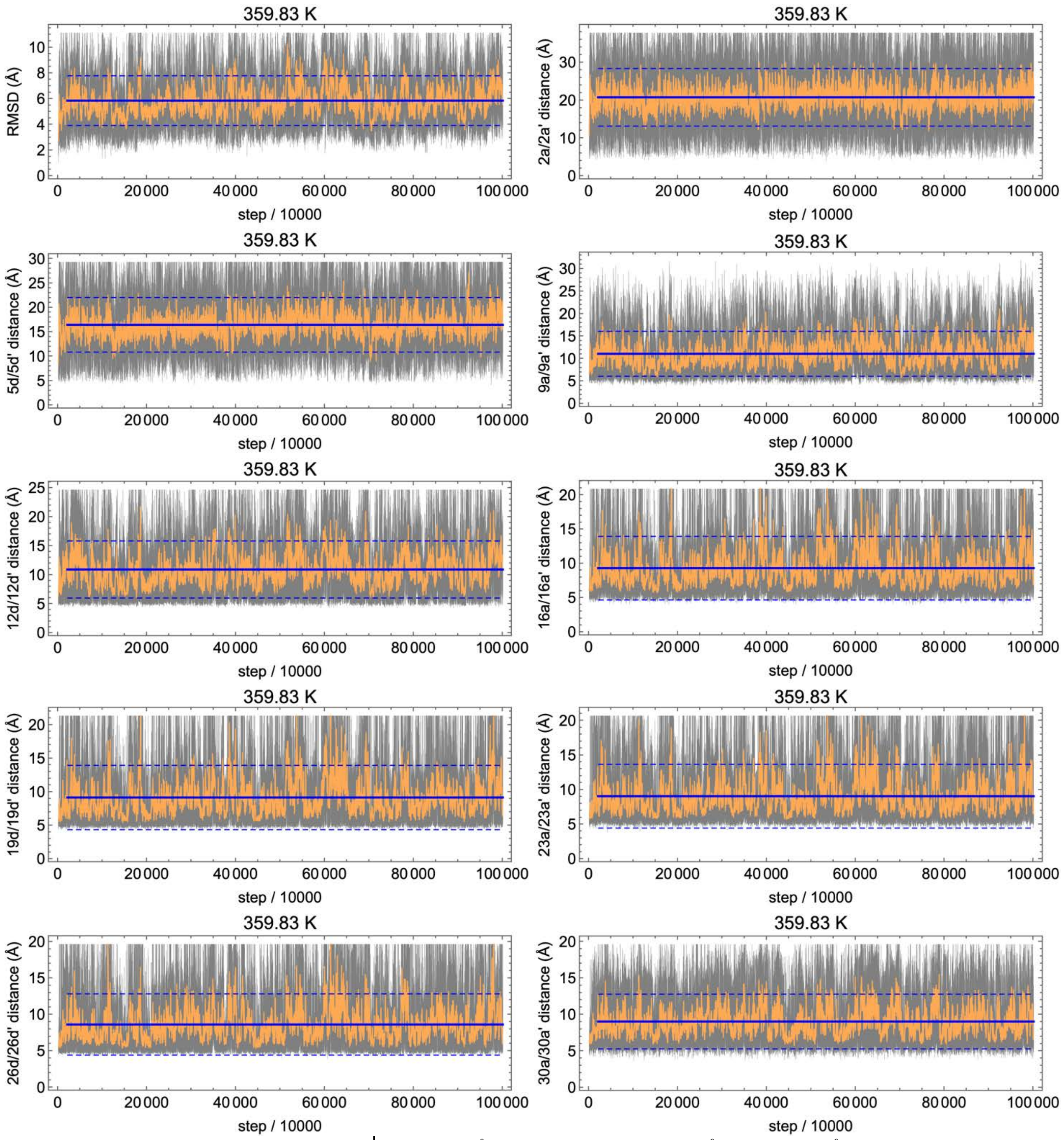

Measure at 359.83 K	Average (Å)	Standard Deviation (Å)	Slope (Å / μs)
RMSD	5.88951	1.93193	0.313059
5d/5d' distance	16.5435	5.58807	0.630845
12d/12d' distance	10.9949	4.91935	0.727081
19d/19d' distance	9.21594	4.79561	0.496377
26d/26d' distance	8.6878	4.20569	0.52784
2a/2a' distance	20.8819	7.60438	0.473148
9a/9a' distance	11.1762	5.00544	0.803443
16a/16a' distance	9.37637	4.63865	0.414002
23a/23a' distance	9.11866	4.60699	0.460754
30a/30a' distance	9.09884	3.75133	0.188422
Core Distance	11.6771	6.48427	

Figure S344. Plots of RMSD and residue packing distances for T-REMD simulations of variant **s7f/10b'-x4x** at 359.83 K.

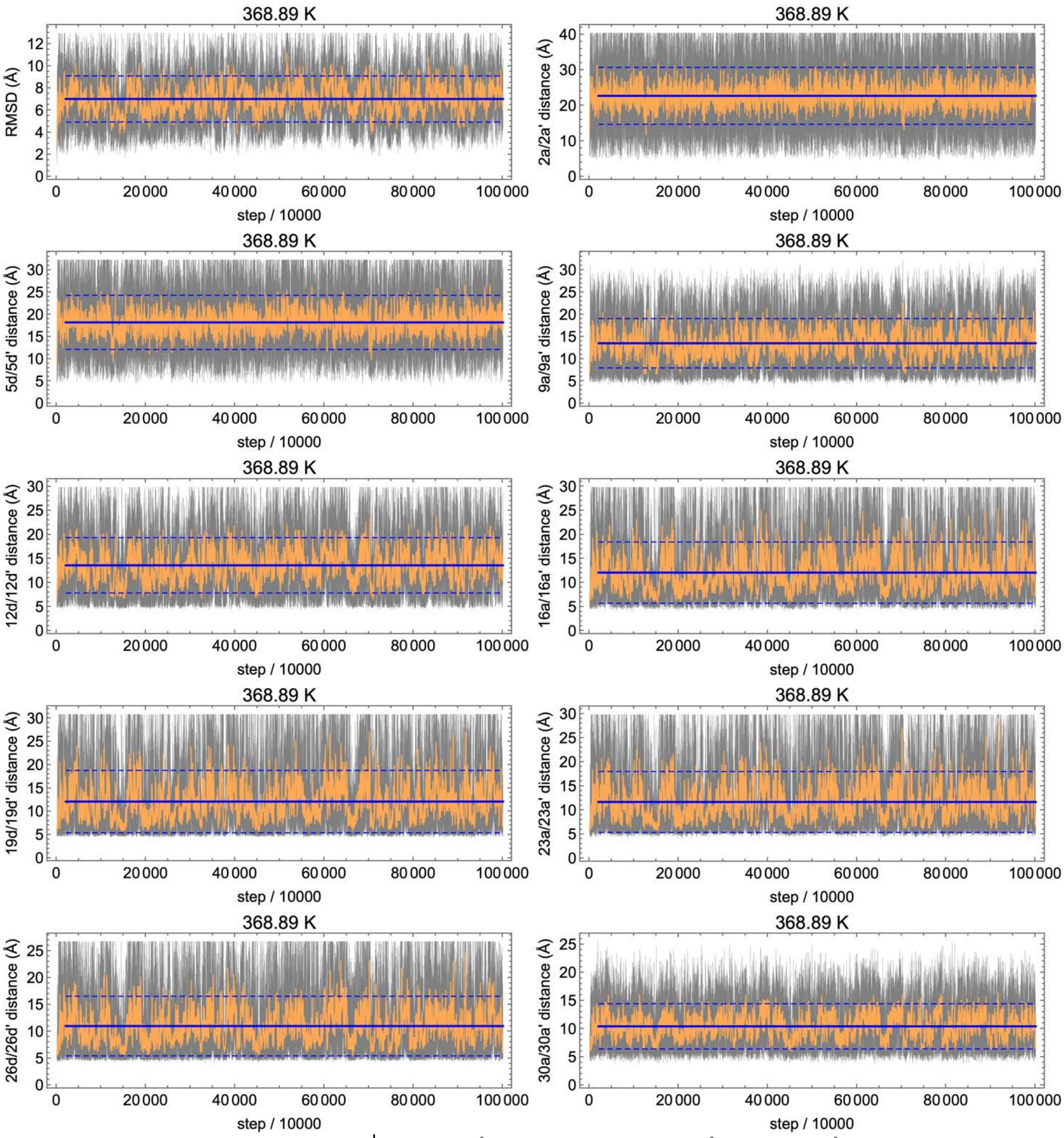

Measure at 368.89 K	Average (Å)	Standard Deviation (Å)	Slope (Å / μs)
RMSD	7.05032	2.08421	0.276294
5d/5d' distance	18.2963	6.09101	0.323466
12d/12d' distance	13.6804	5.7522	0.759411
19d/19d' distance	12.1788	6.69867	0.637085
26d/26d' distance	11.0663	5.55518	0.599262
2a/2a' distance	22.8194	8.00945	0.174334
9a/9a' distance	13.6085	5.54945	0.704979
16a/16a' distance	12.1667	6.35922	0.618846
23a/23a' distance	11.7656	6.3187	0.657774
30a/30a' distance	10.5041	4.02084	0.194565
Core Distance	14.0096	7.19869	

Figure S345. Plots of RMSD and residue packing distances for T-REMD simulations of variant **s7f/10b'-x4x** at 368.89 K.

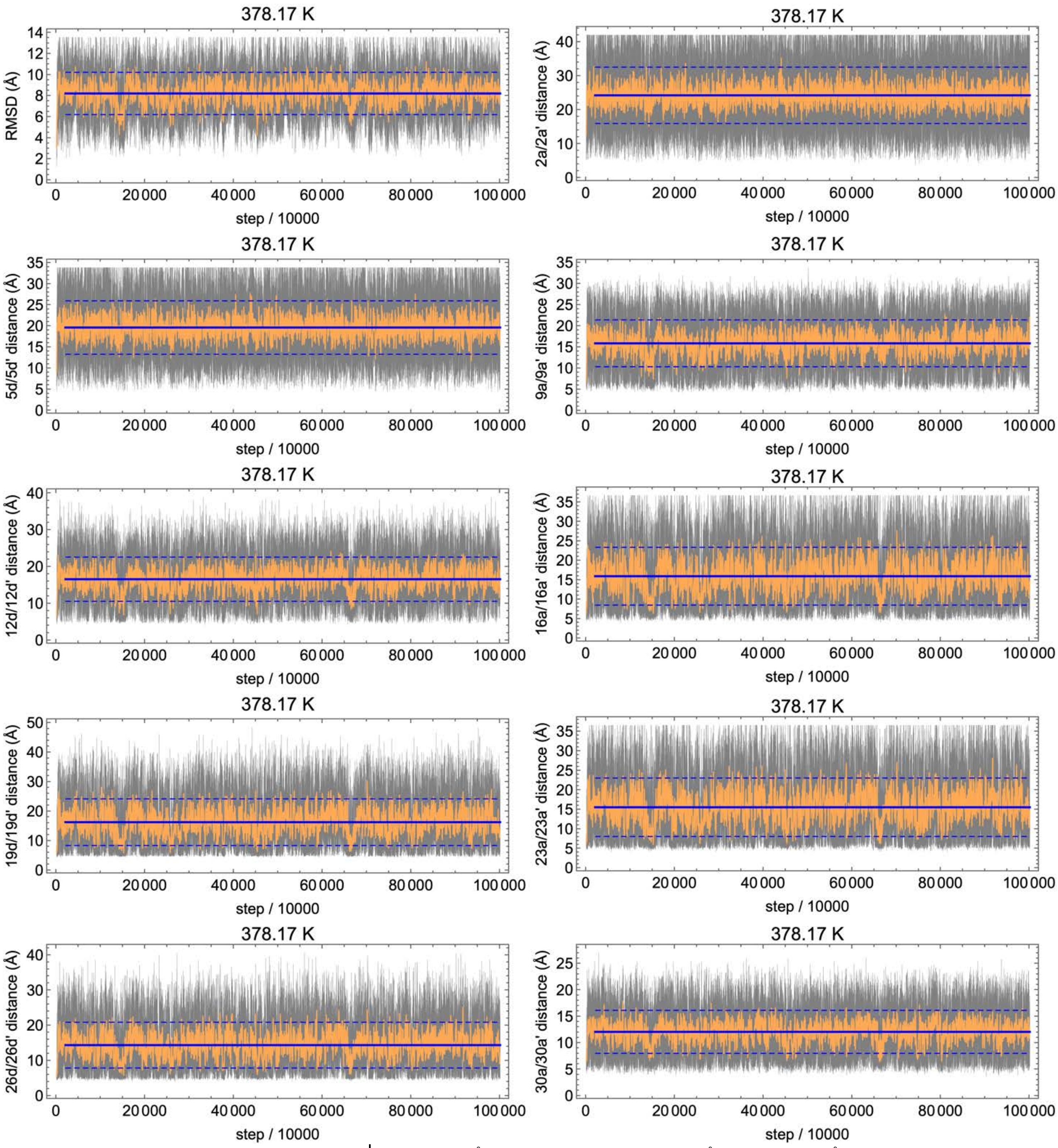

Measure at 378.17 K	Average (Å)	Standard Deviation (Å)	Slope (Å / μs)
RMSD	8.25893	2.0077	0.185329
5d/5d' distance	19.7379	6.3344	0.0296454
12d/12d' distance	16.6985	6.01255	0.541888
19d/19d' distance	16.4197	7.9149	0.574043
26d/26d' distance	14.5062	6.50577	0.53007
2a/2a' distance	24.3924	8.28841	−0.0402713
9a/9a' distance	15.9975	5.53425	0.469742
16a/16a' distance	16.0552	7.4373	0.562798
23a/23a' distance	15.6693	7.4921	0.632929
30a/30a' distance	12.1559	4.06502	0.182024
Core Distance	16.8481	7.4852	

Figure S346. Plots of RMSD and residue packing distances for T-REMD simulations of variant **s7f/10b'-x4x** at 378.17 K.

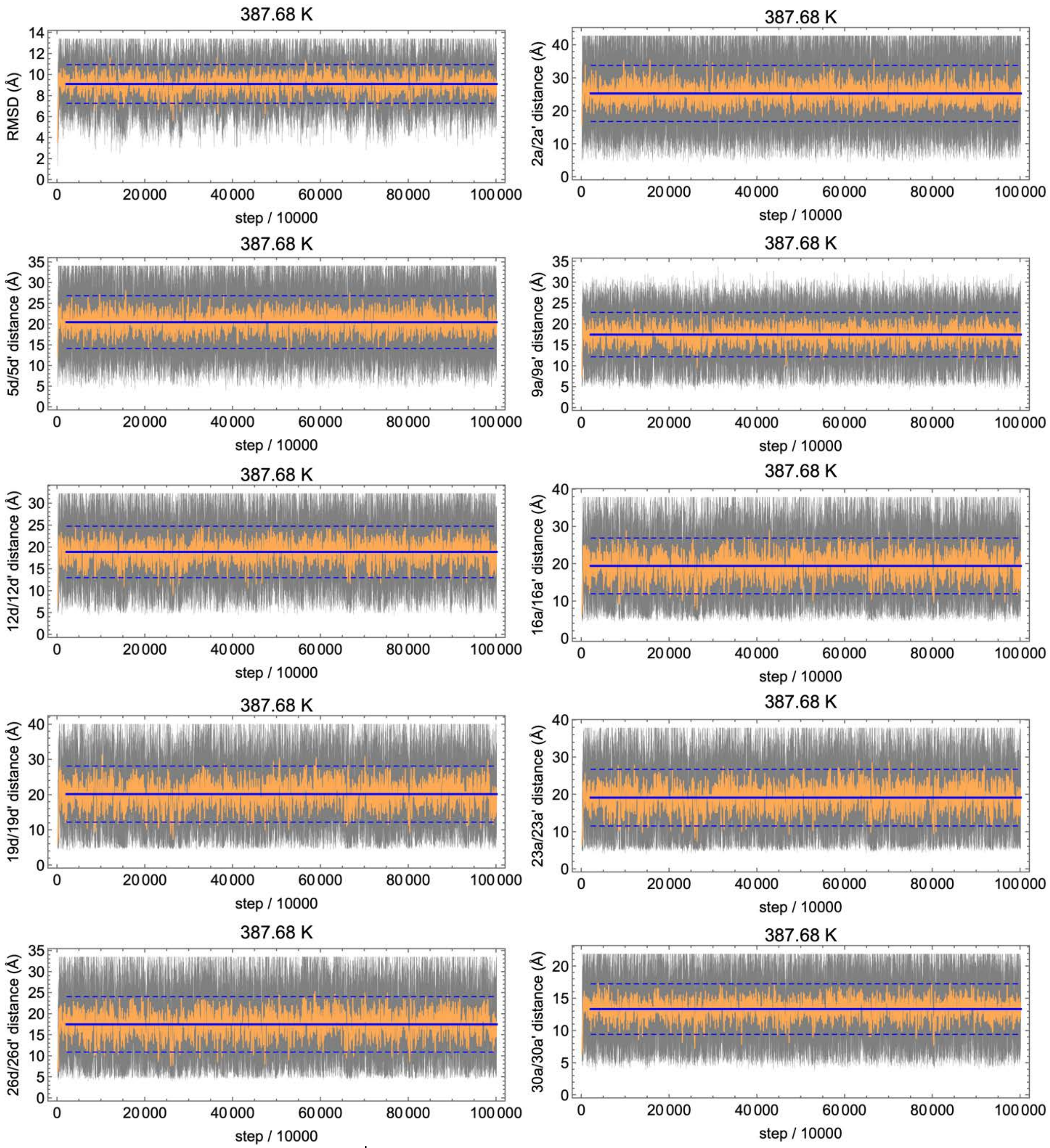

Measure at 387.68 K	Average (Å)	Standard Deviation (Å)	Slope (Å / μs)
RMSD	9.16284	1.84085	0.0710066
5d/5d' distance	20.6251	6.37444	−0.118627
12d/12d' distance	19.0131	5.88578	0.221547
19d/19d' distance	20.3281	7.9655	0.390354
26d/26d' distance	17.6154	6.58407	0.327037
2a/2a' distance	25.4557	8.50396	−0.122849
9a/9a' distance	17.6125	5.31704	0.155359
16a/16a' distance	19.5802	7.48748	0.30058
23a/23a' distance	19.2951	7.60137	0.424565
30a/30a' distance	13.426	3.92349	0.115019
Core Distance	19.2168	7.40038	

Figure S347. Plots of RMSD and residue packing distances for T-REMD simulations of variant **s7f/10b'-x4x** at 387.68 K.

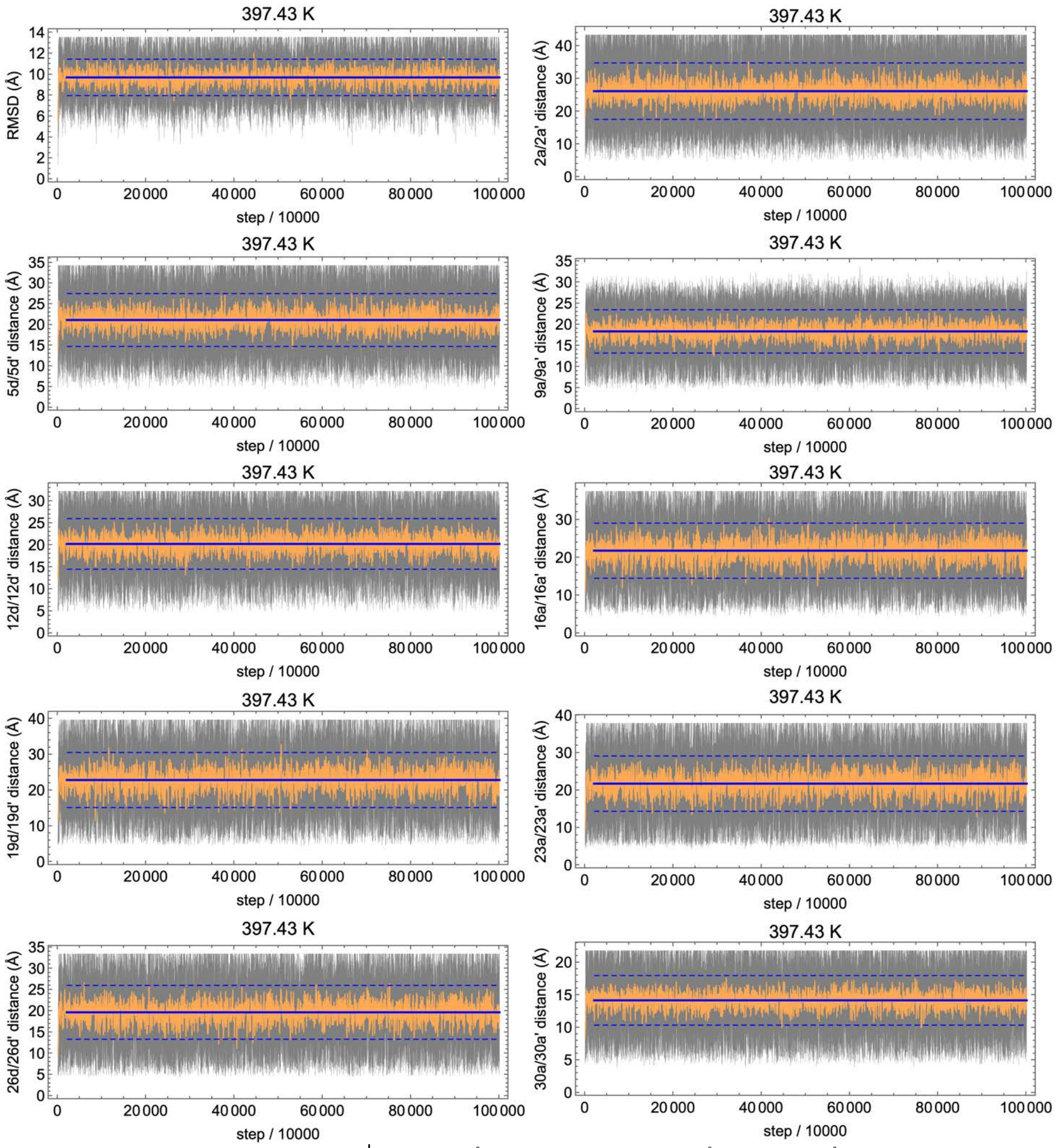

Measure at 397.43 K	Average (Å)	Standard Deviation (Å)	Slope (Å / μs)
RMSD	9.74427	1.73832	0.0401085
5d/5d' distance	21.2224	6.38743	−0.132932
12d/12d' distance	20.3367	5.73746	0.12539
19d/19d' distance	22.9617	7.69631	0.183386
26d/26d' distance	19.7634	6.34481	0.18475
2a/2a' distance	26.3048	8.61707	−0.153691
9a/9a' distance	18.4497	5.1266	0.0615281
16a/16a' distance	21.8993	7.27613	0.117328
23a/23a' distance	21.8621	7.37837	0.242297
30a/30a' distance	14.2349	3.80356	0.0345962
Core Distance	20.7817	7.32568	

Figure S348. Plots of RMSD and residue packing distances for T-REMD simulations of variant **s7f/10b'-x4x** at 397.43 K.

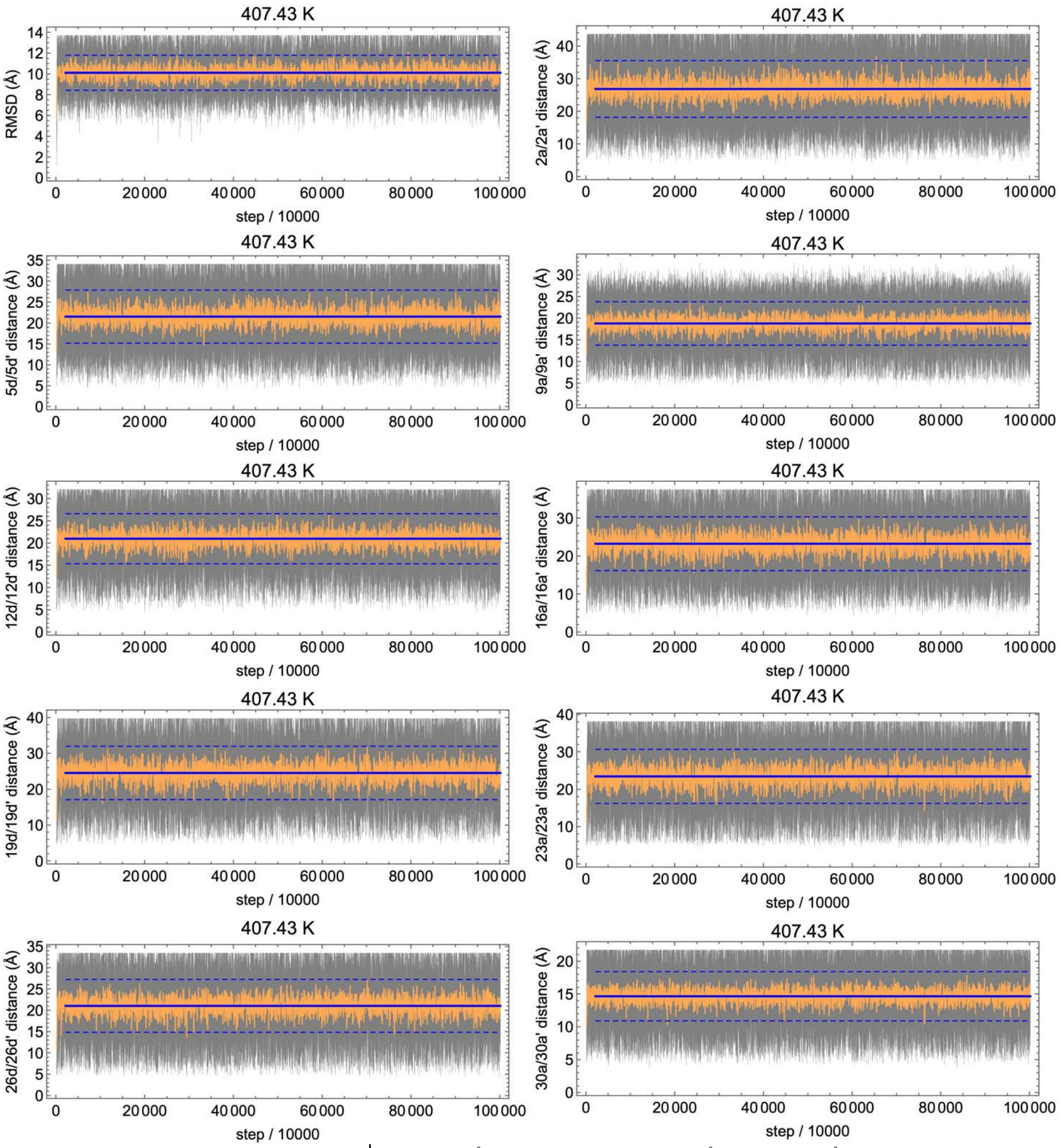

Measure at 407.43 K	Average (Å)	Standard Deviation (Å)	Slope (Å / μs)
RMSD	10.1732	1.68323	0.0246222
5d/5d' distance	21.6884	6.3477	−0.0878537
12d/12d' distance	21.1226	5.62506	0.0647569
19d/19d' distance	24.7265	7.44786	0.0348824
26d/26d' distance	21.1998	6.20094	0.108056
2a/2a' distance	27.0161	8.67407	−0.204889
9a/9a' distance	18.9274	5.00365	0.0496775
16a/16a' distance	23.4254	7.07845	−0.0177812
23a/23a' distance	23.6074	7.23077	0.115376
30a/30a' distance	14.7522	3.74905	0.00656478
Core Distance	21.8295	7.32576	

Figure S349. Plots of RMSD and residue packing distances for T-REMD simulations of variant **s7f/10b'-x4x** at 407.43 K.

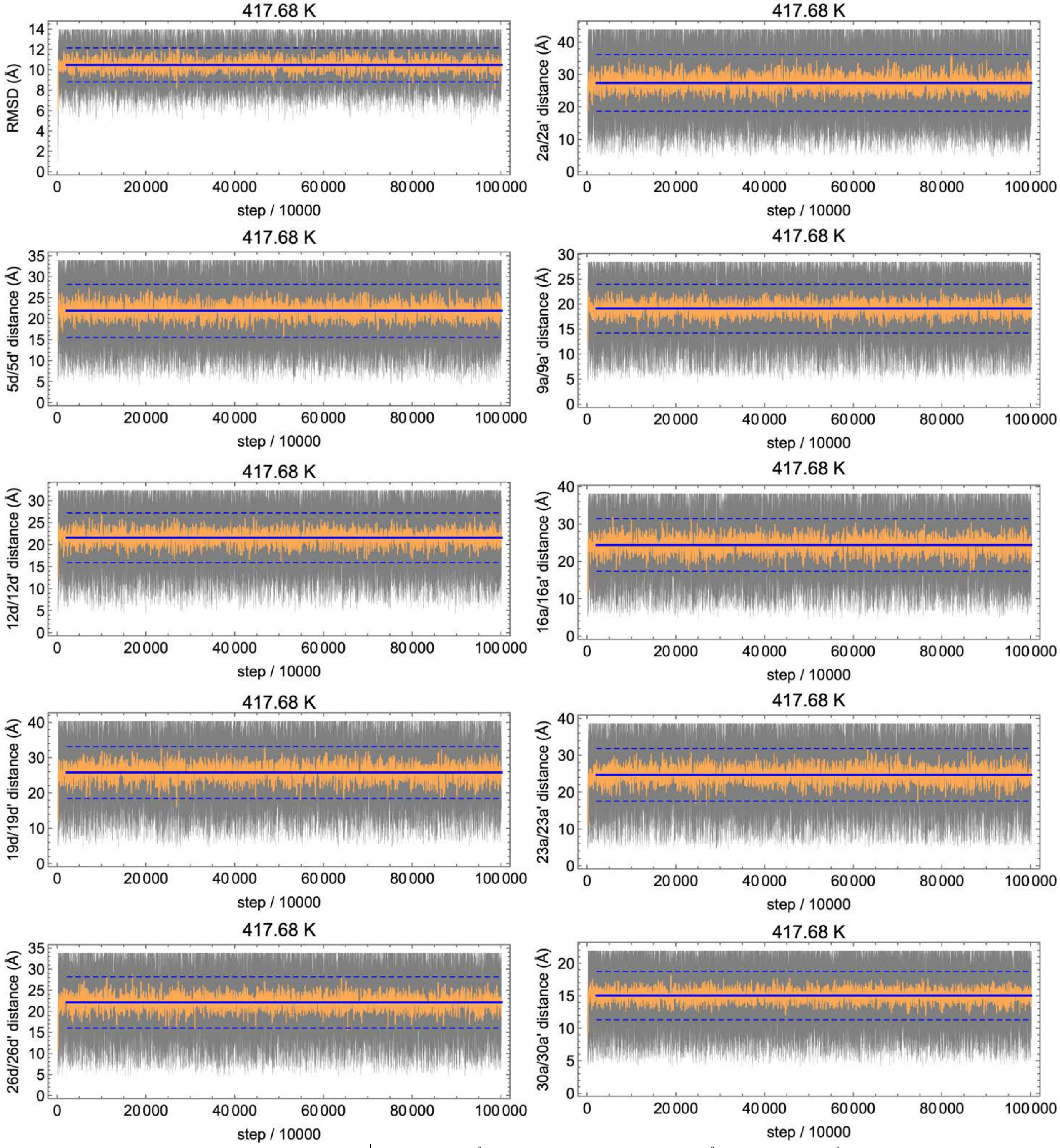

Measure at 417.68 K	Average (Å)	Standard Deviation (Å)	Slope (Å / μs)
RMSD	10.537	1.66653	−0.00975221
5d/5d' distance	22.0529	6.3285	−0.148641
12d/12d' distance	21.7289	5.61375	−0.0797166
19d/19d' distance	25.9827	7.37784	−0.12032
26d/26d' distance	22.2583	6.08953	−0.0395909
2a/2a' distance	27.6049	8.75733	−0.156558
9a/9a' distance	19.2554	4.89313	−0.00818053
16a/16a' distance	24.5696	7.03017	−0.0888131
23a/23a' distance	24.8845	7.14126	−0.0945186
30a/30a' distance	15.1369	3.73143	−0.0382894
Core Distance	22.6082	7.39145	

Figure S350. Plots of RMSD and residue packing distances for T-REMD simulations of variant **s7f/10b’-x4x** at 417.68 K.

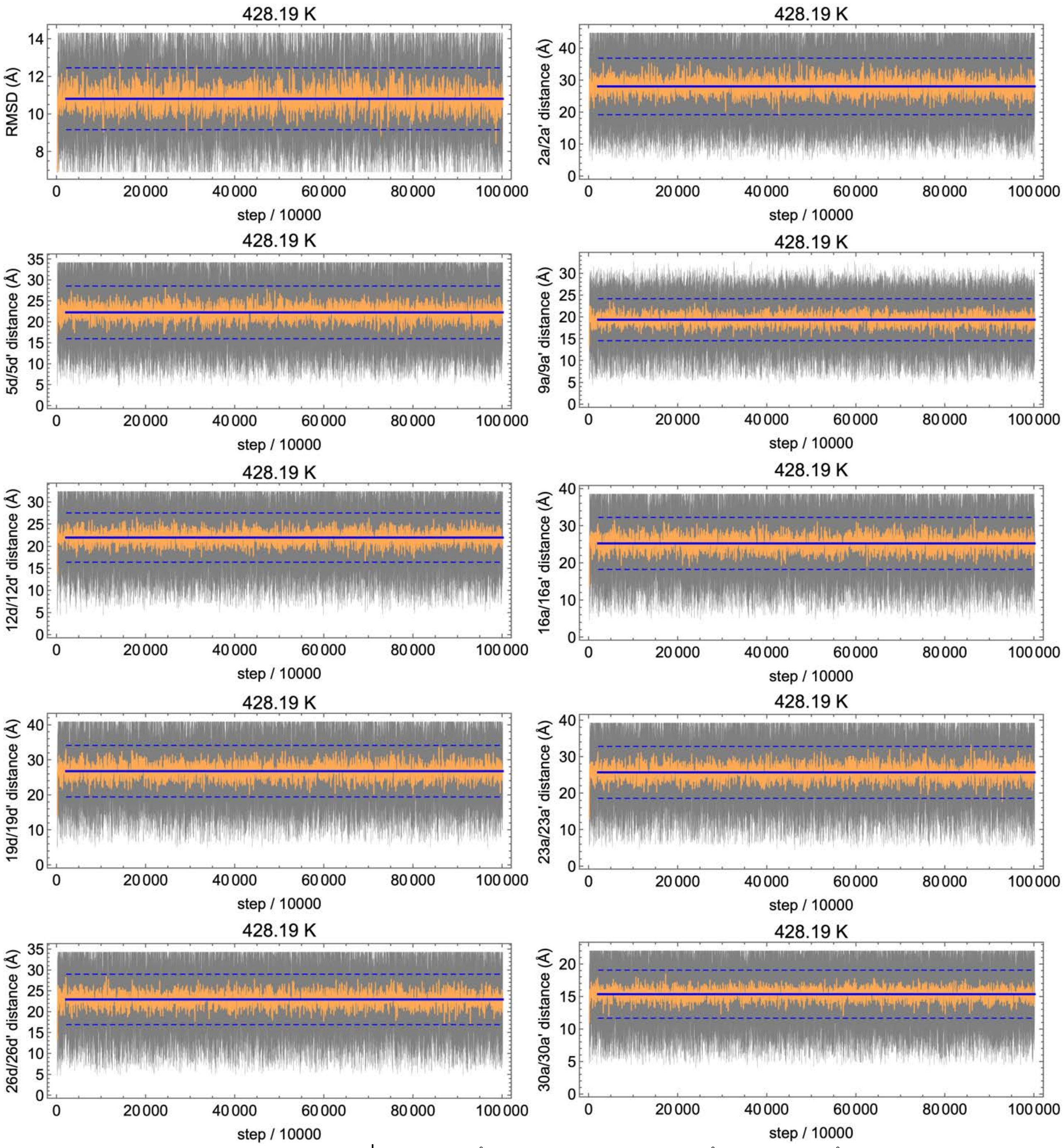

Measure at 428.19 K	Average (Å)	Standard Deviation (Å)	Slope (Å / μs)
RMSD	10.8445	1.64655	−0.0328128
5d/5d' distance	22.4255	6.28934	−0.176652
12d/12d' distance	22.1152	5.573	−0.0125802
19d/19d' distance	26.9561	7.35467	−0.0433765
26d/26d' distance	23.0915	6.03491	−0.0140634
2a/2a' distance	28.2242	8.83193	−0.226386
9a/9a' distance	19.5167	4.81258	−0.0433854
16a/16a' distance	25.4076	6.99959	−0.00675968
23a/23a' distance	25.8649	7.11288	0.0215263
30a/30a' distance	15.4569	3.69669	−0.0119062
Core Distance	23.2287	7.4697	

Figure S351. Plots of RMSD and residue packing distances for T-REMD simulations of variant **s7f/10b'-x4x** at 428.19 K.

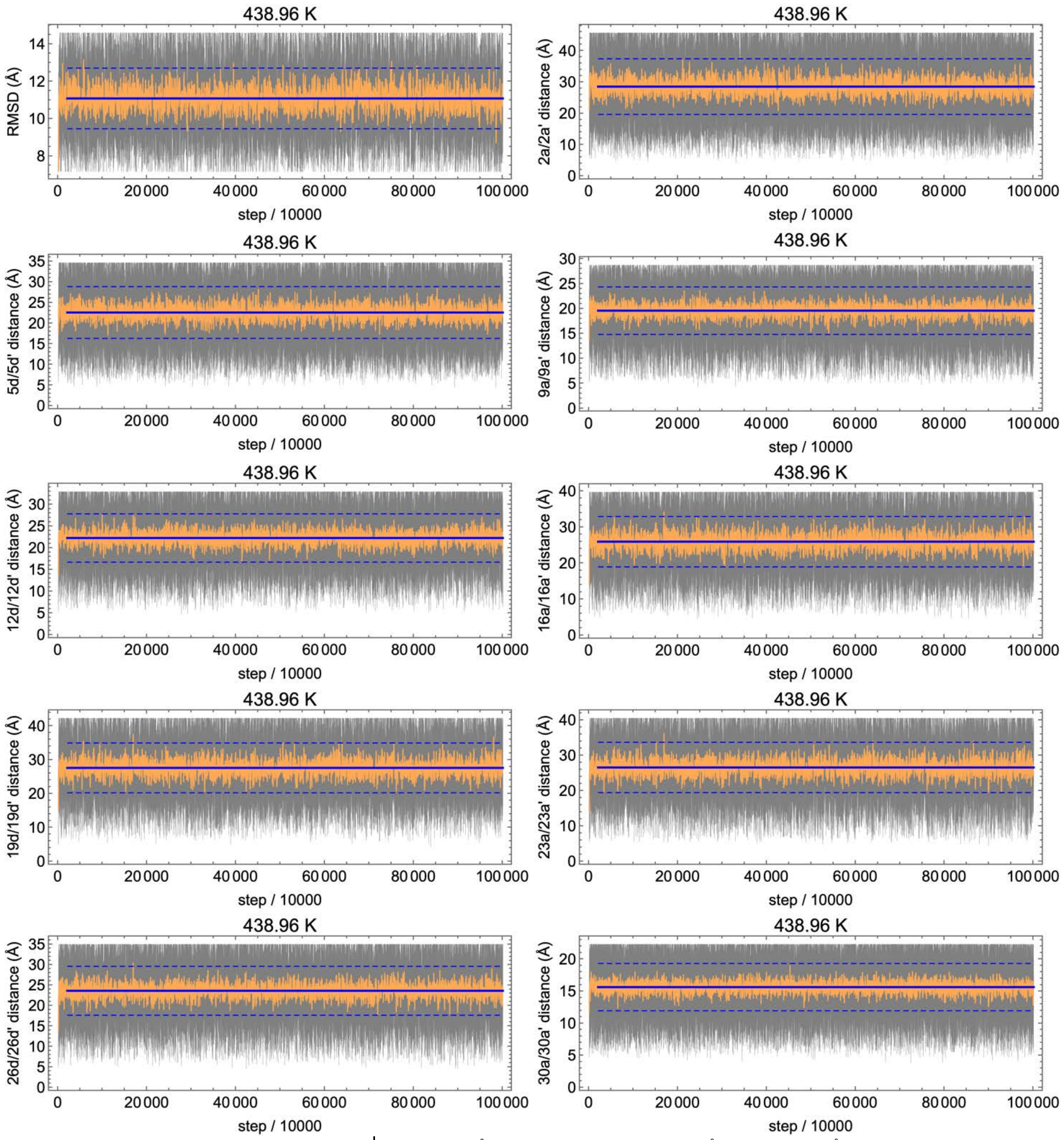

Measure at 438.96 K	Average (Å)	Standard Deviation (Å)	Slope (Å / μs)
RMSD	11.1049	1.62426	−0.0221051
5d/5d' distance	22.6847	6.26875	−0.128091
12d/12d' distance	22.3789	5.55715	0.0223492
19d/19d' distance	27.7263	7.36283	−0.0100086
26d/26d' distance	23.7278	5.97298	0.0490645
2a/2a' distance	28.6669	8.88287	−0.227154
9a/9a' distance	19.6695	4.76446	−0.016585
16a/16a' distance	26.0661	6.99881	−0.0176392
23a/23a' distance	26.6998	7.12436	0.0259573
30a/30a' distance	15.6971	3.68789	−0.0119574
Core Distance	23.7019	7.55393	

Figure S352. Plots of RMSD and residue packing distances for T-REMD simulations of variant **s7f/10b'-x4x** at 438.96 K.

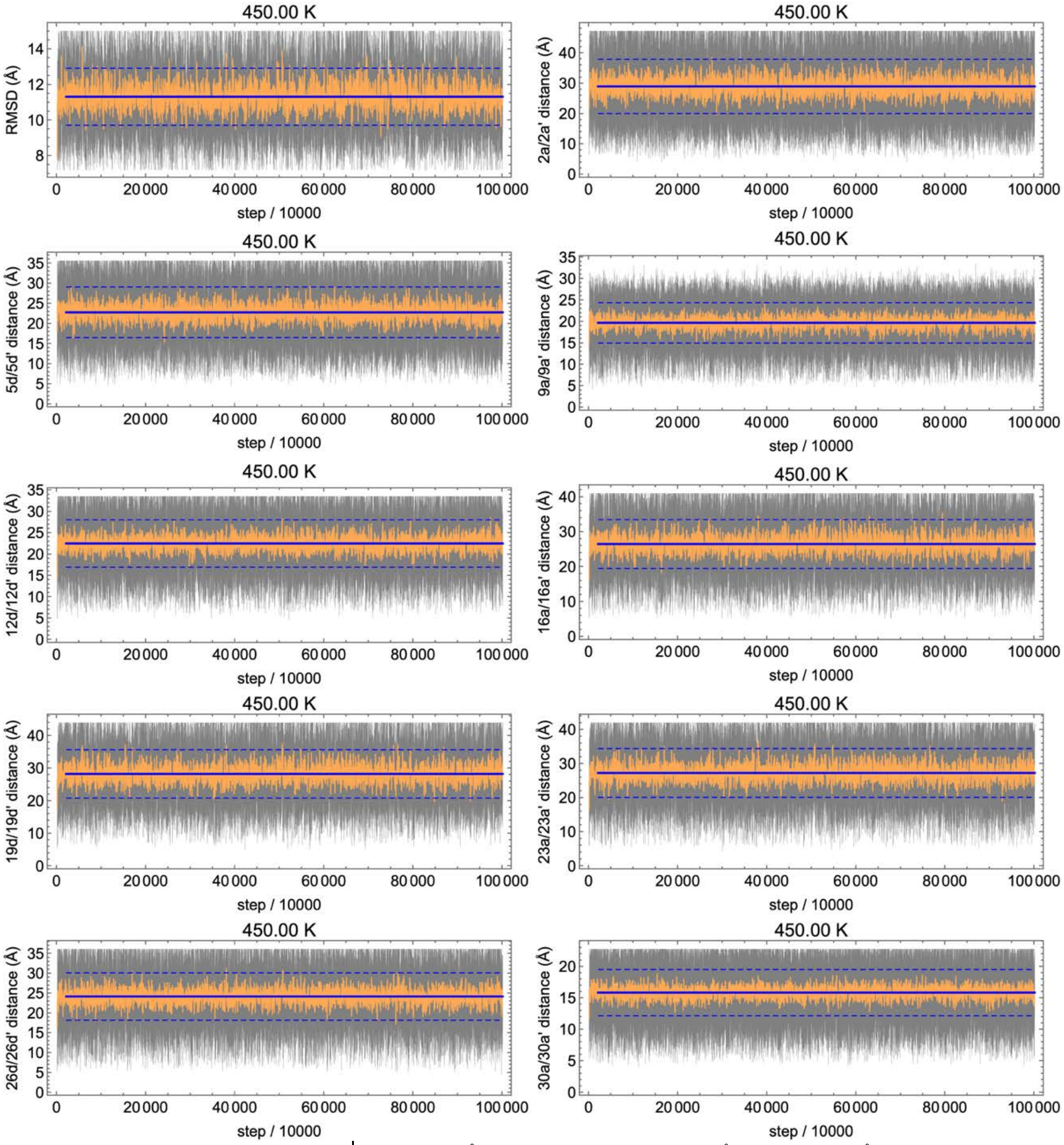

Measure at 450.00 K	Average (Å)	Standard Deviation (Å)	Slope (Å / μs)
RMSD	11.3439	1.60547	−0.015771
5d/5d' distance	22.9126	6.29319	−0.101192
12d/12d' distance	22.612	5.55182	−0.000422111
19d/19d' distance	28.3927	7.41286	0.0010475
26d/26d' distance	24.2832	5.97335	0.126022
2a/2a' distance	29.1292	8.93205	−0.136557
9a/9a' distance	19.7967	4.70039	0.0299582
16a/16a' distance	26.6256	6.99682	0.0285512
23a/23a' distance	27.4257	7.13821	0.0606356
30a/30a' distance	15.9154	3.66367	0.0380549
Core Distance	24.1214	7.64912	

Figure S353. Plots of RMSD and residue packing distances for T-REMD simulations of variant **s7f/10b'-x4x** at 450.00 K.

(return to Table of Contents)

14f/17b'-xx

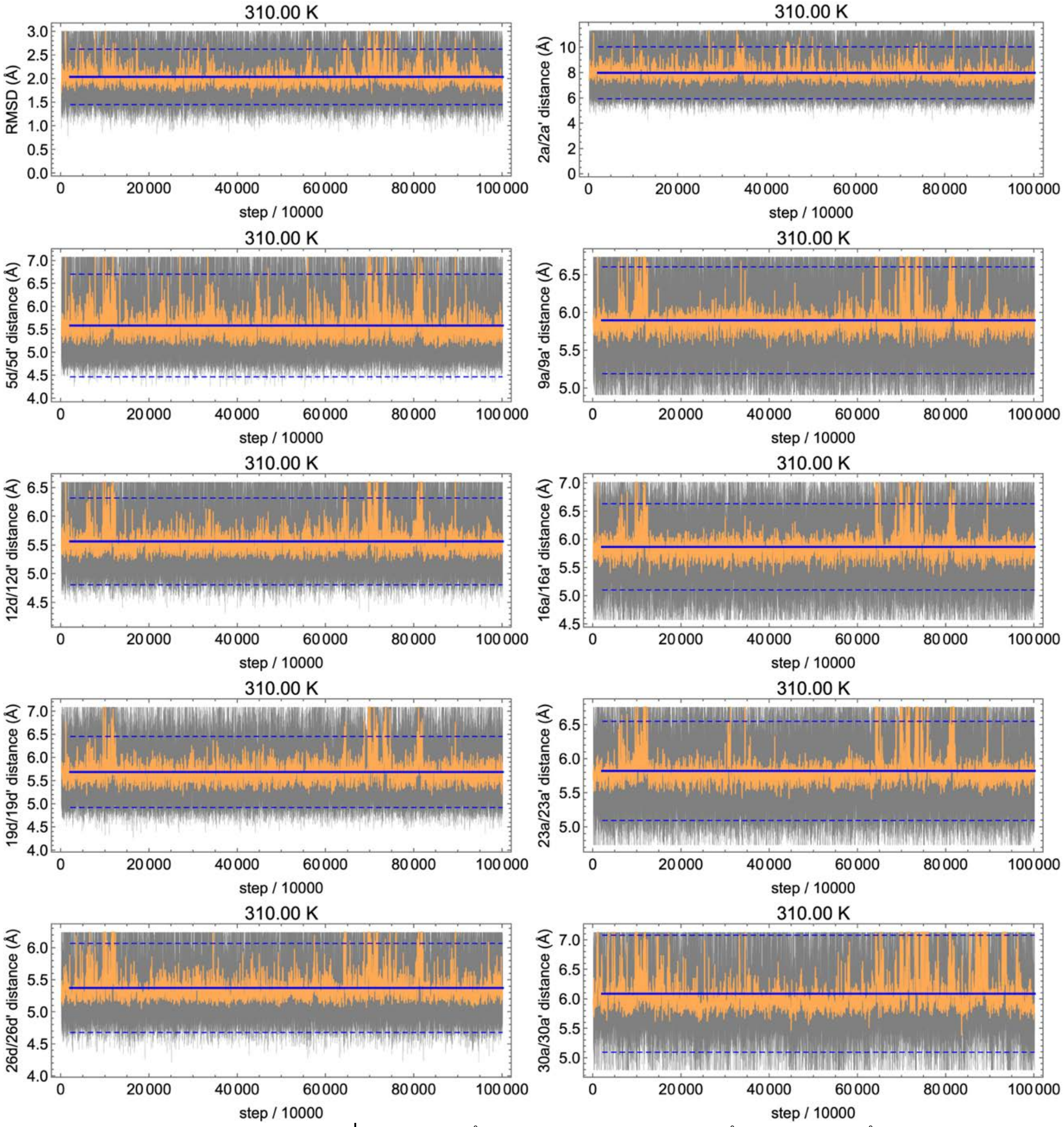

Measure at 310.00 K	Average (Å)	Standard Deviation (Å)	Slope (Å / μs)
RMSD	2.04971	0.586081	0.0111764
5d/5d' distance	5.59468	1.11996	−0.0279074
12d/12d' distance	5.5722	0.756506	0.00675605
19d/19d' distance	5.70004	0.766521	0.015721
26d/26d' distance	5.38195	0.69469	0.00979447
2a/2a' distance	8.03035	2.05352	−0.105774
9a/9a' distance	5.90531	0.707865	0.00485351
16a/16a' distance	5.87721	0.764434	0.0145253
23a/23a' distance	5.82966	0.72807	0.017912
30a/30a' distance	6.09809	0.992428	0.0768295
Core Distance	5.99883	1.27882	

Figure S354. Plots of RMSD and residue packing distances for T-REMD simulations of variant **14f/17b'-xx** at 310.00 K.

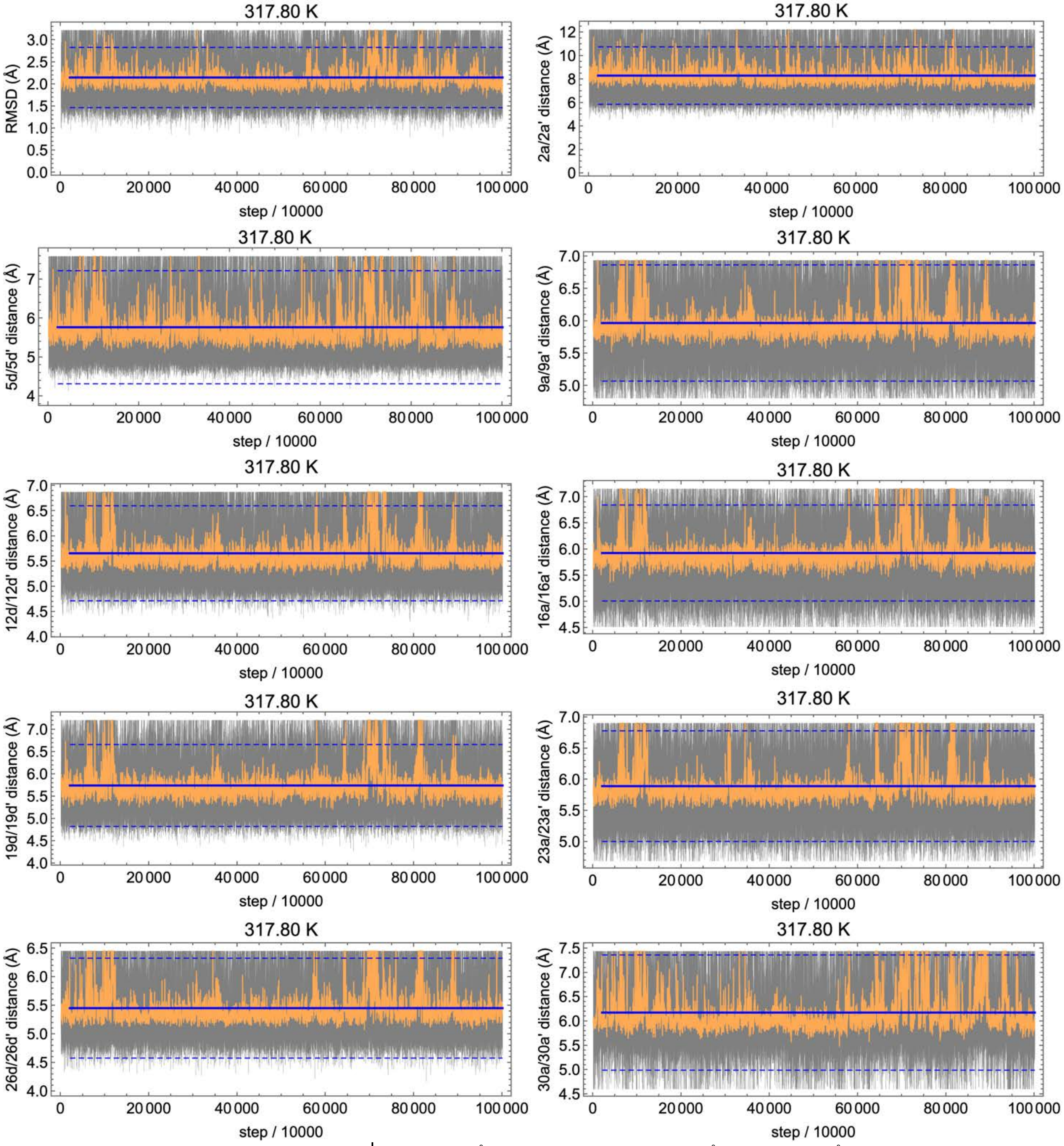

Measure at 317.80 K	Average (Å)	Standard Deviation (Å)	Slope (Å / μs)
RMSD	2.15872	0.68183	0.0170288
5d/5d' distance	5.77969	1.45346	−0.0228636
12d/12d' distance	5.6664	0.94229	0.0130494
19d/19d' distance	5.75235	0.917727	0.0330093
26d/26d' distance	5.4615	0.87355	0.028534
2a/2a' distance	8.34439	2.44869	−0.113983
9a/9a' distance	5.97545	0.897924	0.0159659
16a/16a' distance	5.93614	0.916776	0.0269458
23a/23a' distance	5.89887	0.888274	0.0254305
30a/30a' distance	6.18513	1.18532	0.10728
Core Distance	6.1111	1.5051	

Figure S355. Plots of RMSD and residue packing distances for T-REMD simulations of variant **14f/17b'-xx** at 317.80 K.

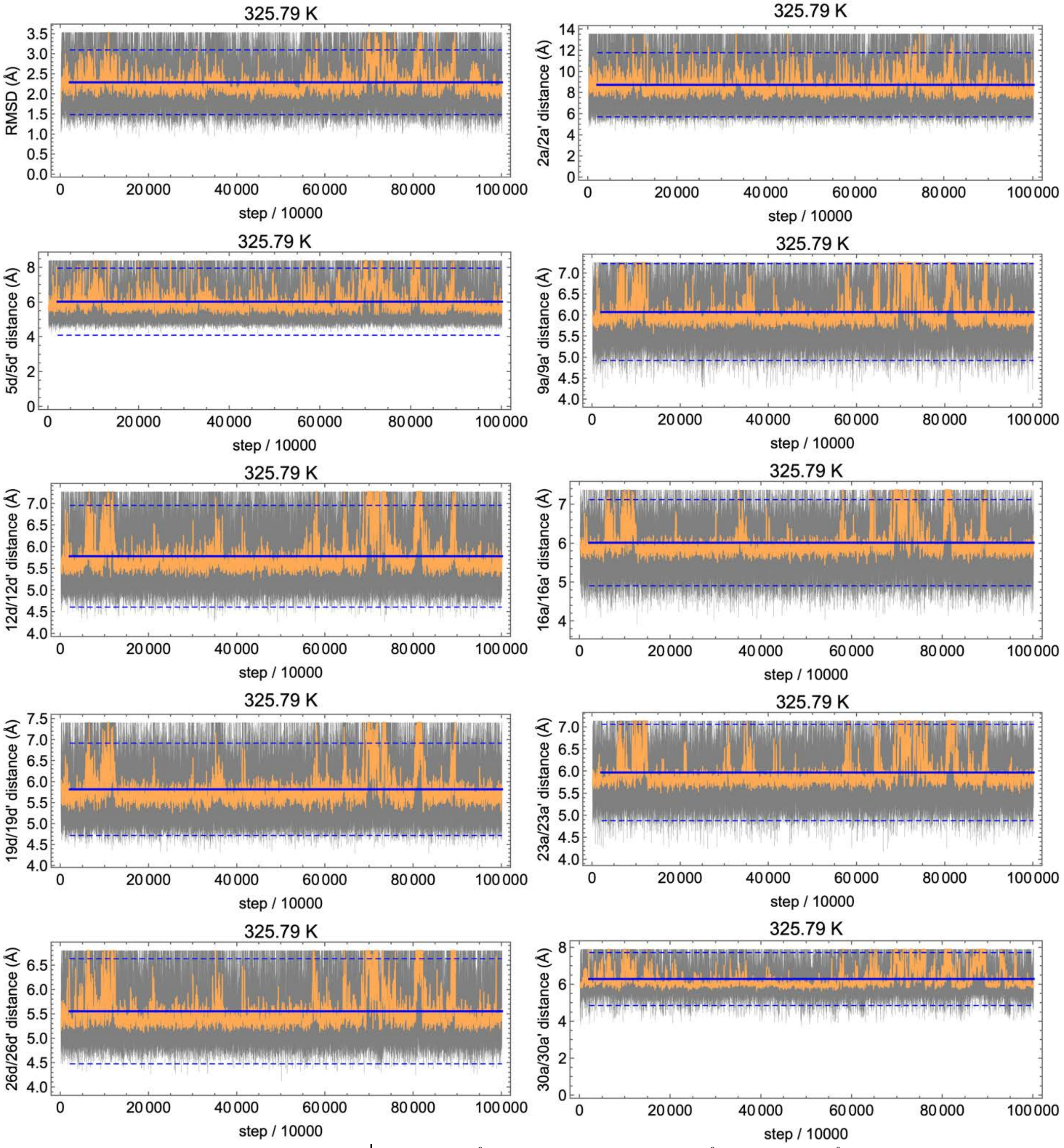

Measure at 325.79 K	Average (Å)	Standard Deviation (Å)	Slope (Å / μs)
RMSD	2.30521	0.805128	0.0391085
5d/5d' distance	6.05816	1.92621	0.00809706
12d/12d' distance	5.79353	1.17298	0.0548963
19d/19d' distance	5.83236	1.0995	0.0710812
26d/26d' distance	5.56588	1.07667	0.0622079
2a/2a' distance	8.79577	3.03395	−0.119556
9a/9a' distance	6.0876	1.15396	0.0536145
16a/16a' distance	6.02748	1.10686	0.0628484
23a/23a' distance	5.98331	1.09345	0.0630154
30a/30a' distance	6.3194	1.43272	0.18232
Core Distance	6.27372	1.82512	

Figure S356. Plots of RMSD and residue packing distances for T-REMD simulations of variant **14f/17b'-xx** at 325.79 K.

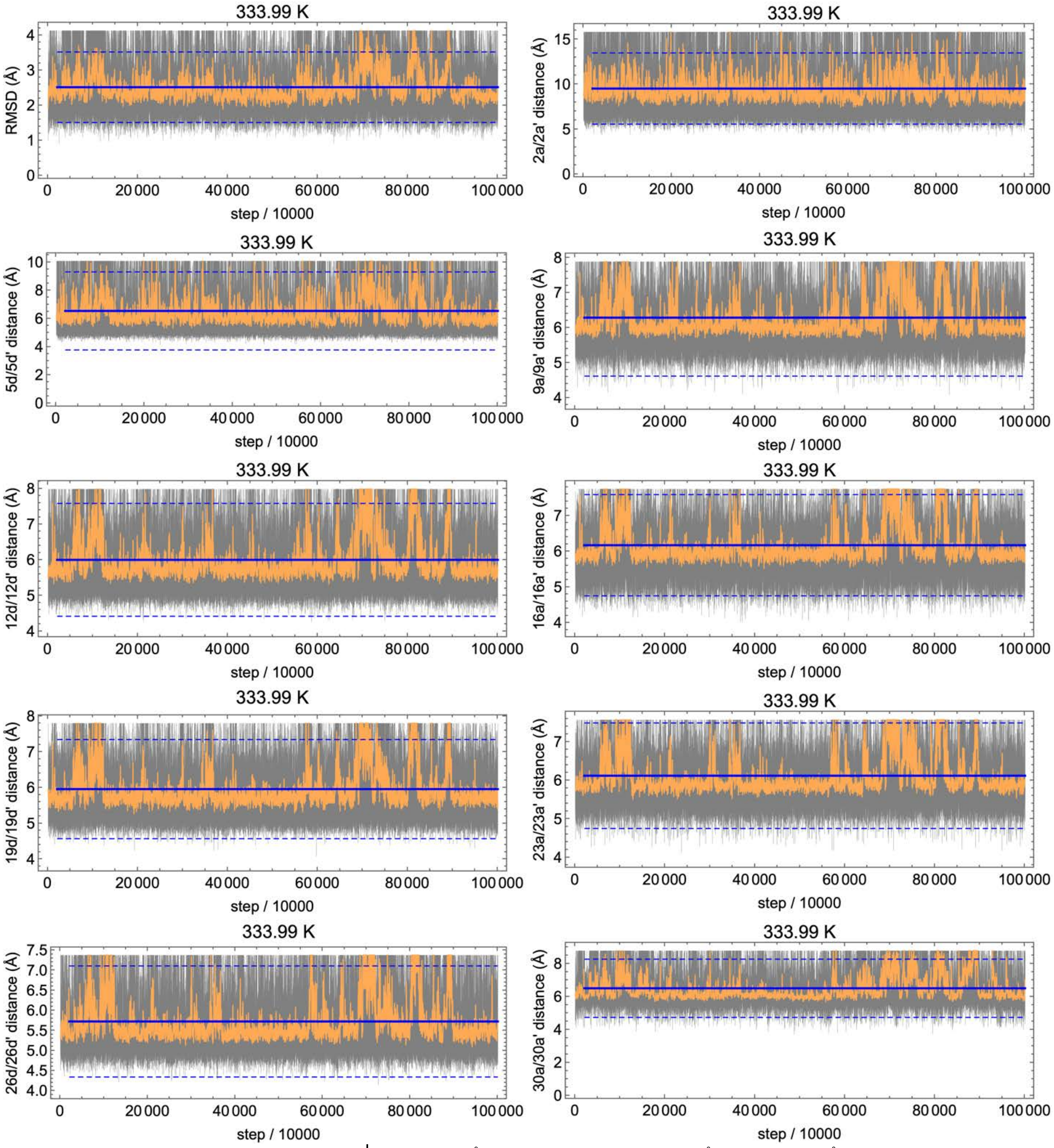

Measure at 333.99 K	Average (Å)	Standard Deviation (Å)	Slope (Å / μs)
RMSD	2.52728	1.00506	0.0606239
5d/5d' distance	6.56712	2.7667	0.0377028
12d/12d' distance	6.01254	1.58346	0.0716974
19d/19d' distance	5.9686	1.38635	0.095795
26d/26d' distance	5.73411	1.38318	0.0933342
2a/2a' distance	9.55914	3.96089	−0.0810538
9a/9a' distance	6.29679	1.66934	0.0939534
16a/16a' distance	6.18266	1.41886	0.0914931
23a/23a' distance	6.12926	1.3693	0.0867399
30a/30a' distance	6.52764	1.76495	0.249452
Core Distance	6.5531	2.36172	

Figure S357. Plots of RMSD and residue packing distances for T-REMD simulations of variant **14f/17b'-xx** at 333.99 K.

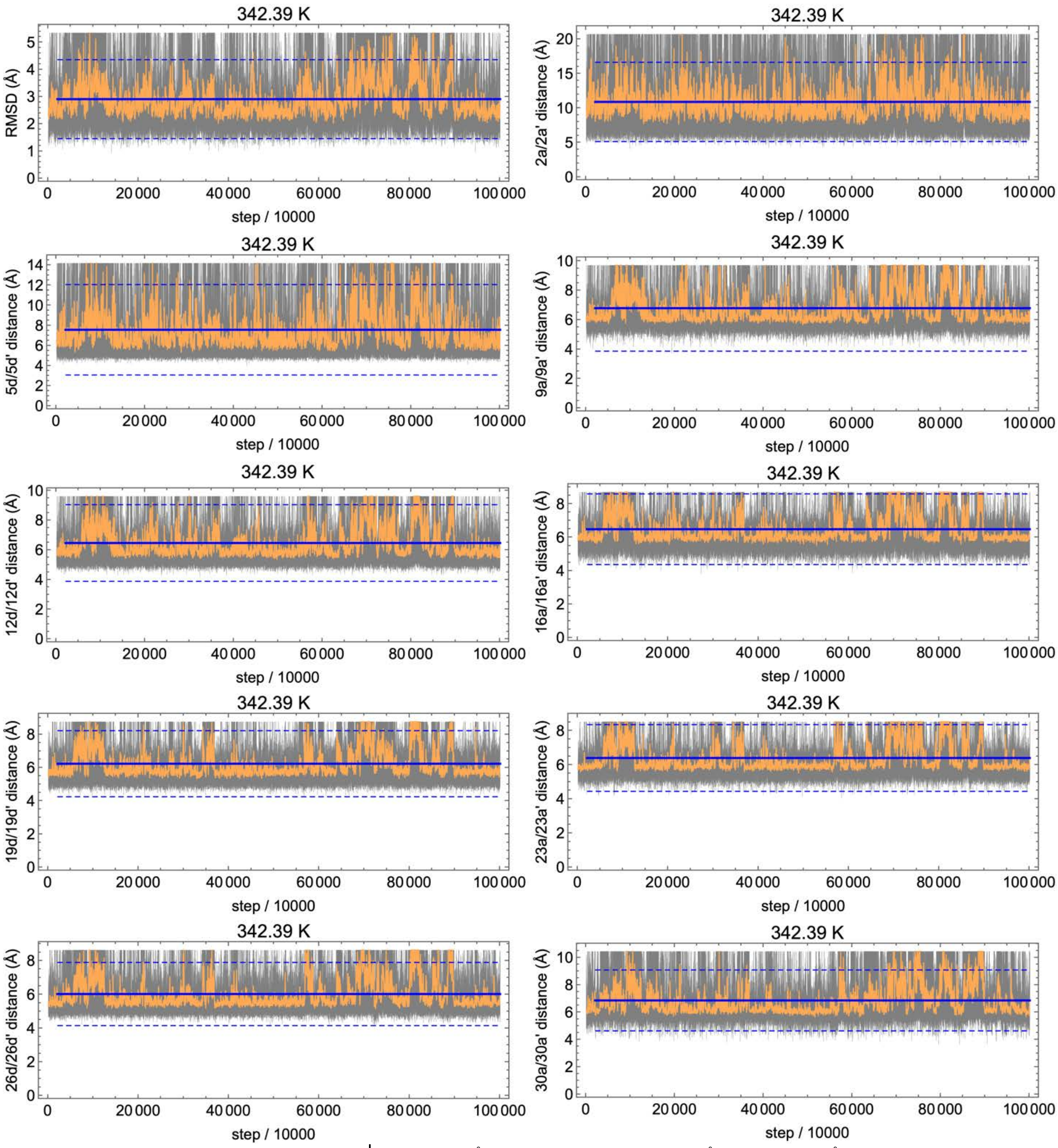

Measure at 342.39 K	Average (Å)	Standard Deviation (Å)	Slope (Å / μs)
RMSD	2.92474	1.44975	0.0821687
5d/5d' distance	7.61238	4.49281	0.115711
12d/12d' distance	6.49407	2.58201	0.0876756
19d/19d' distance	6.26162	1.98949	0.0988027
26d/26d' distance	6.04966	1.87291	0.126623
2a/2a' distance	10.9483	5.74882	0.00779687
9a/9a' distance	6.81518	2.9207	0.106427
16a/16a' distance	6.50835	2.11323	0.091799
23a/23a' distance	6.42335	1.95203	0.118307
30a/30a' distance	6.89046	2.22553	0.336442
Core Distance	7.11148	3.4529	

Figure S358. Plots of RMSD and residue packing distances for T-REMD simulations of variant **14f/17b'-xx** at 342.39 K.

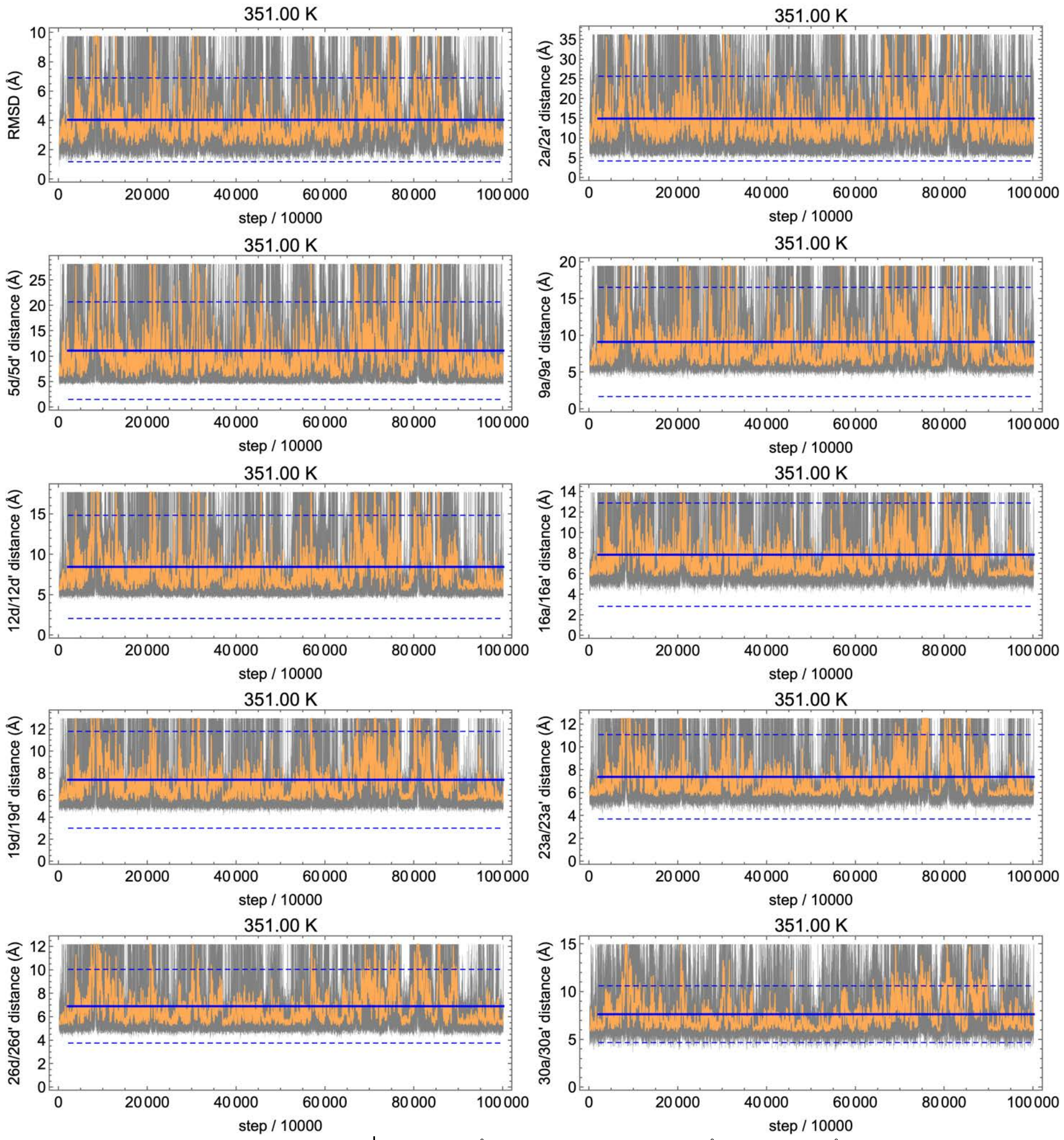

Measure at 351.00 K	Average (Å)	Standard Deviation (Å)	Slope (Å / μs)
RMSD	4.08341	2.85582	−0.132791
5d/5d' distance	11.2134	9.60467	−0.504951
12d/12d' distance	8.52366	6.39221	−0.370263
19d/19d' distance	7.45704	4.39655	−0.300092
26d/26d' distance	6.95096	3.14212	−0.106542
2a/2a' distance	15.0766	10.7461	−0.564418
9a/9a' distance	9.18616	7.42583	−0.394191
16a/16a' distance	7.90961	5.03179	−0.347341
23a/23a' distance	7.43601	3.6891	−0.10171
30a/30a' distance	7.7001	2.97044	0.178586
Core Distance	9.0504	6.94943	

Figure S359. Plots of RMSD and residue packing distances for T-REMD simulations of variant **14f/17b'-xx** at 351.00 K.

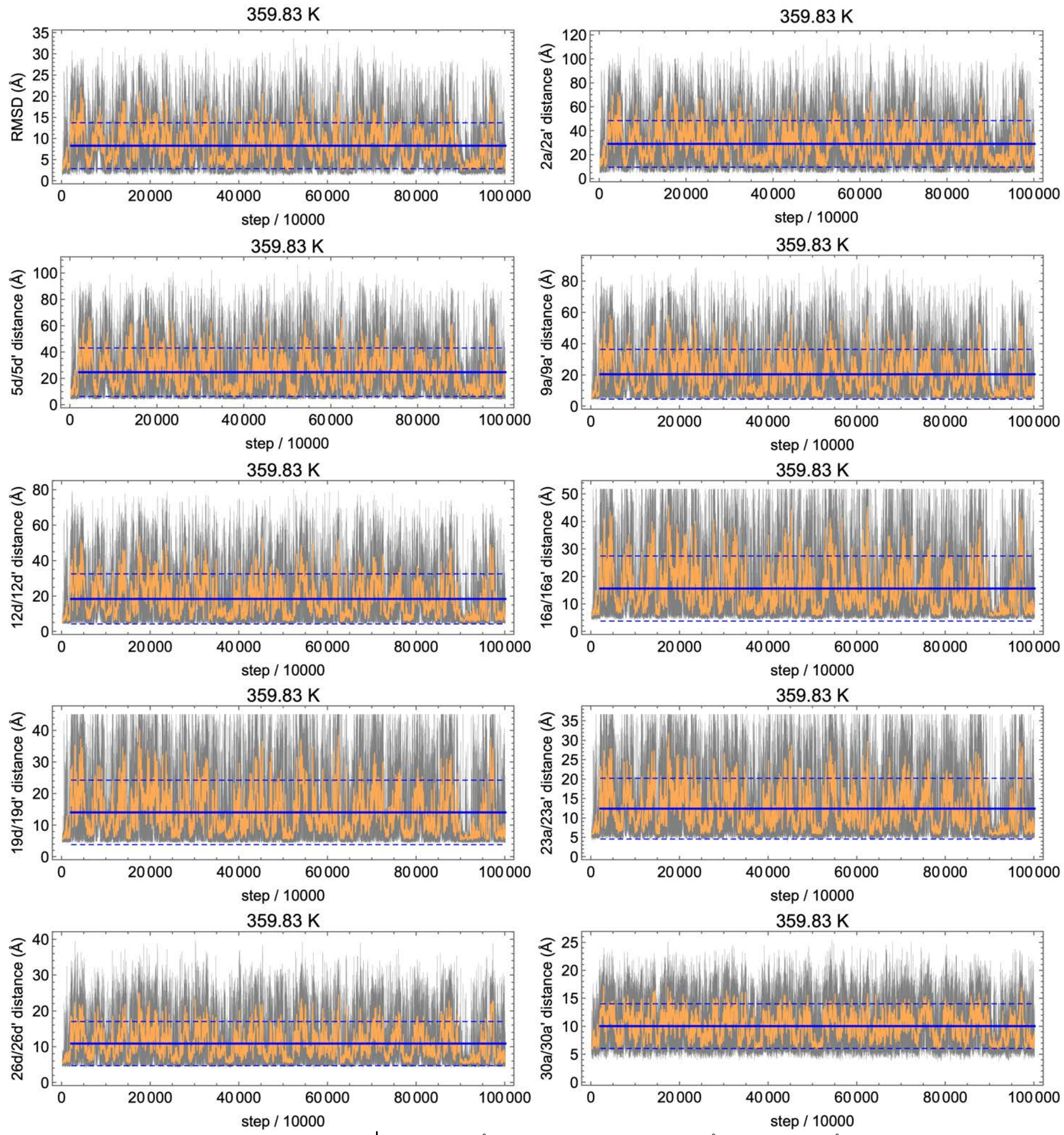

Measure at 359.83 K	Average (Å)	Standard Deviation (Å)	Slope (Å / μs)
RMSD	8.44814	5.4251	−1.38222
5d/5d' distance	25.2178	18.3965	−4.31284
12d/12d' distance	18.7393	14.134	−3.43813
19d/19d' distance	14.25	10.2118	−2.40429
26d/26d' distance	11.0538	6.1554	−1.23962
2a/2a' distance	29.6026	19.4234	−4.36918
9a/9a' distance	20.8158	15.9302	−3.8135
16a/16a' distance	15.844	11.8403	−2.84136
23a/23a' distance	12.5515	7.82428	−1.5223
30a/30a' distance	10.1466	3.98682	−0.612575
Core Distance	17.5802	14.4554	

Figure S360. Plots of RMSD and residue packing distances for T-REMD simulations of variant **14f/17b’-xx** at 359.83 K.

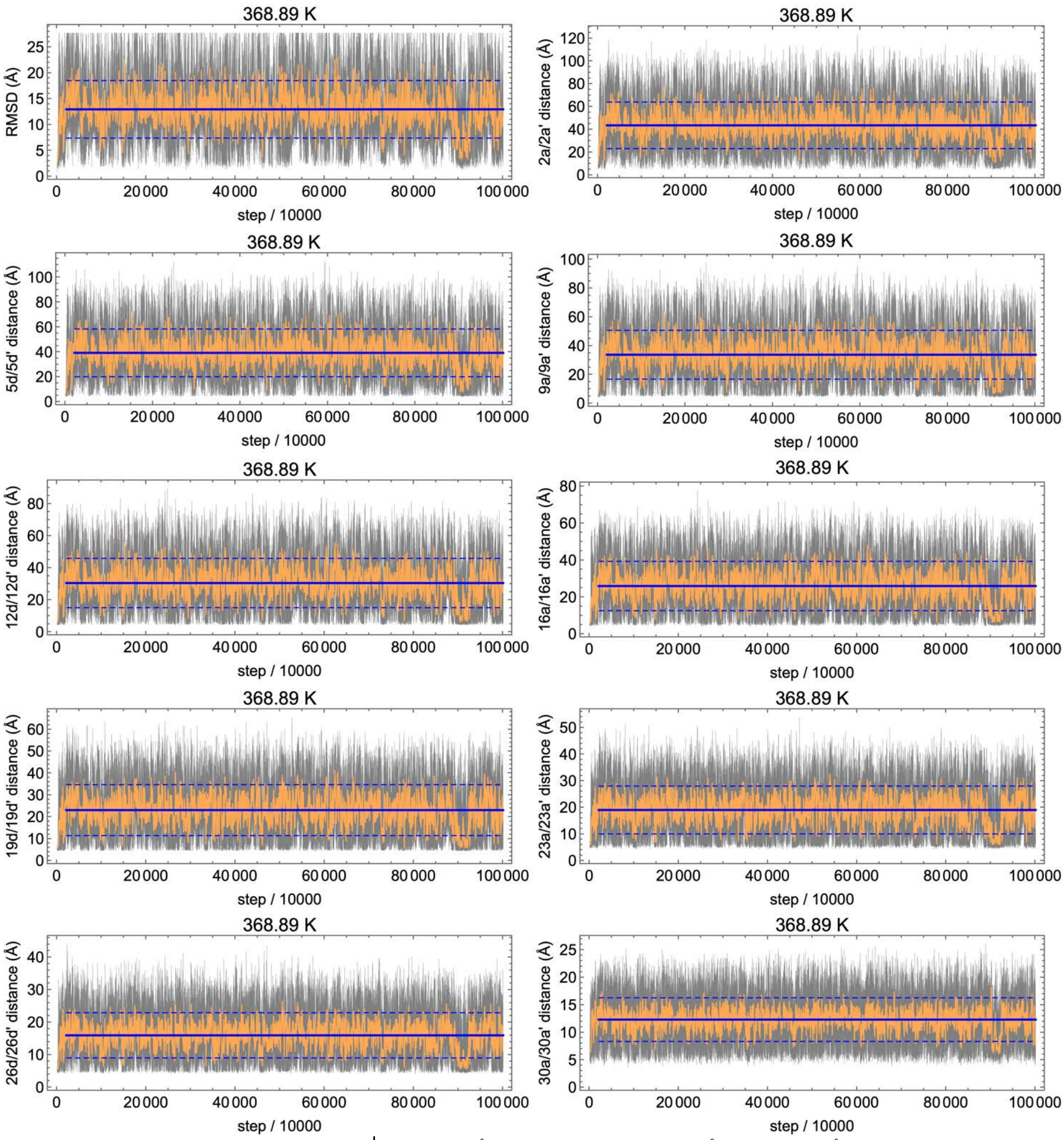

Measure at 368.89 K	Average (Å)	Standard Deviation (Å)	Slope (Å / μs)
RMSD	13.047	5.55507	−0.797554
5d/5d' distance	39.6524	19.1539	−2.48737
12d/12d' distance	30.8186	15.3715	−2.09893
19d/19d' distance	23.3001	11.6353	−1.44666
26d/26d' distance	16.1322	6.94997	−0.59242
2a/2a' distance	43.9885	20.4267	−2.55578
9a/9a' distance	34.0946	16.9593	−2.32887
16a/16a' distance	26.2278	13.3612	−1.75209
23a/23a' distance	19.2124	8.99138	−0.850818
30a/30a' distance	12.4004	3.96694	−0.206307
Core Distance	27.3141	17.2746	

Figure S361. Plots of RMSD and residue packing distances for T-REMD simulations of variant **14f/17b'-xx** at 368.89 K.

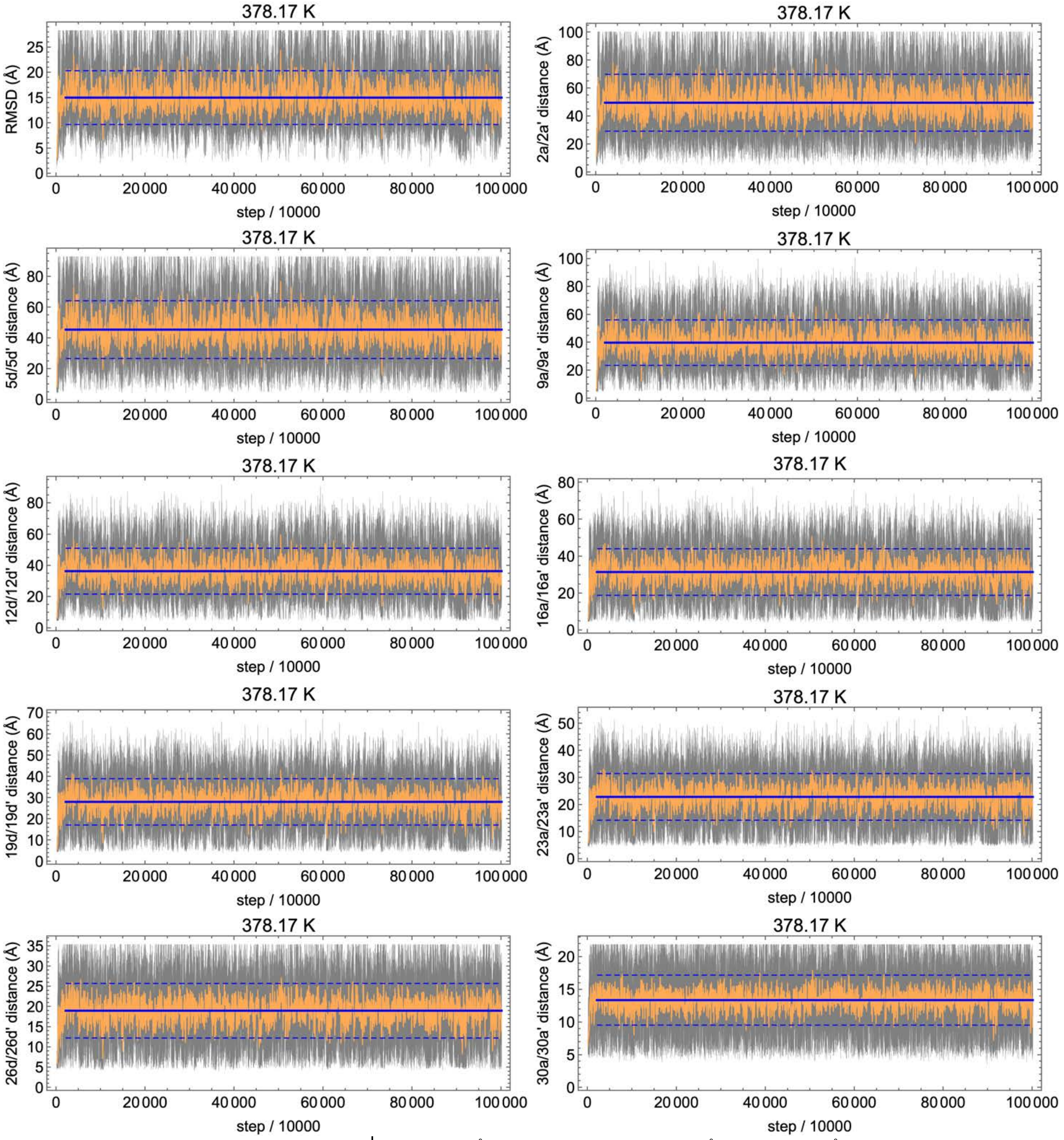

Measure at 378.17 K	Average (Å)	Standard Deviation (Å)	Slope (Å / μs)
RMSD	15.1145	5.32186	−0.332082
5d/5d' distance	45.7874	18.7579	−1.01094
12d/12d' distance	36.6824	14.6849	−0.741815
19d/19d' distance	28.2927	10.9296	−0.389179
26d/26d' distance	19.1132	6.7467	−0.0125302
2a/2a' distance	49.9538	20.3405	−1.12168
9a/9a' distance	40.1684	16.2942	−0.883398
16a/16a' distance	31.7307	12.589	−0.571632
23a/23a' distance	23.0596	8.61039	−0.124379
30a/30a' distance	13.4431	3.82266	0.0317583
Core Distance	32.0257	17.8394	

Figure S362. Plots of RMSD and residue packing distances for T-REMD simulations of variant **14f/17b'-xx** at 378.17 K.

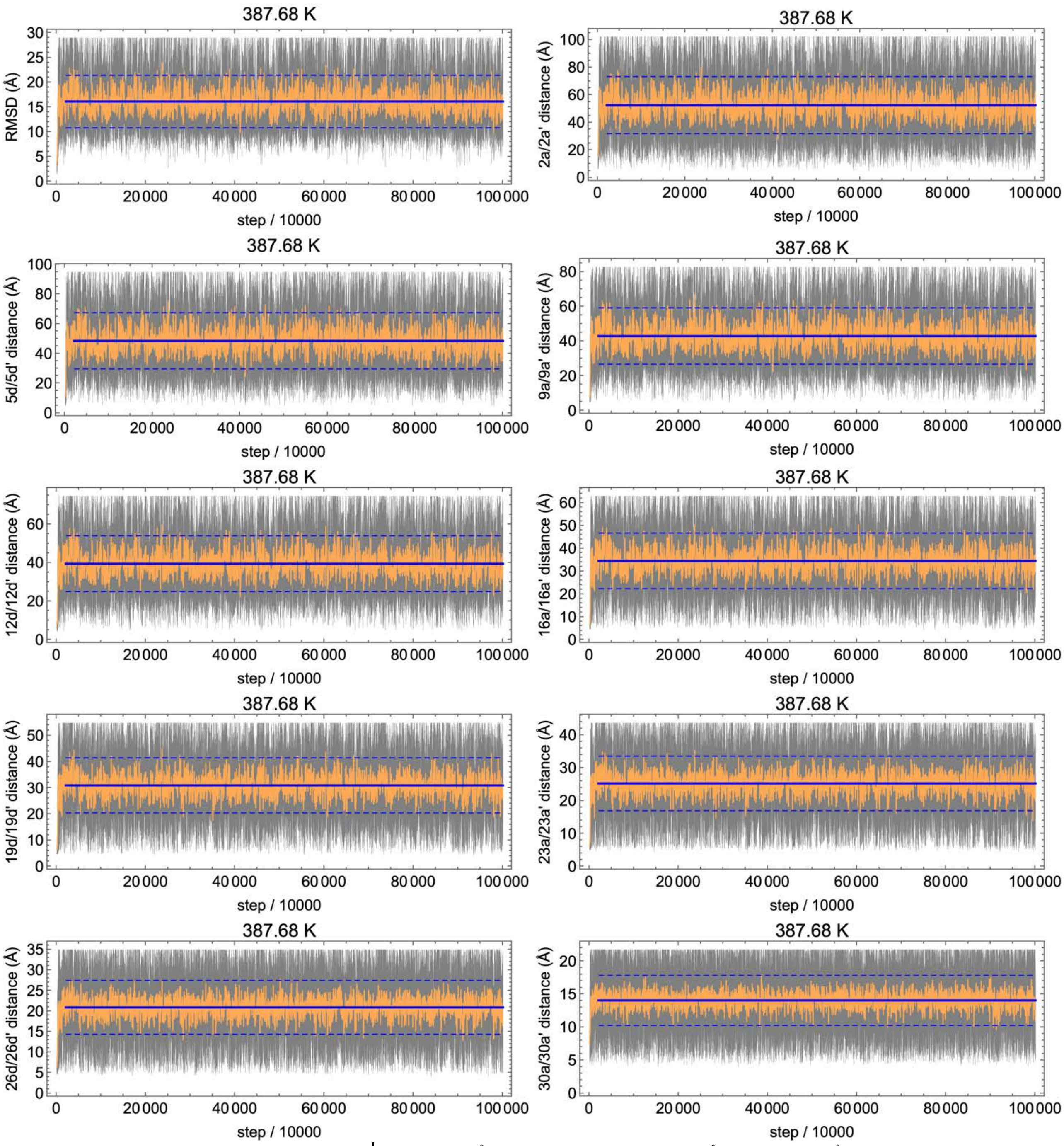

Measure at 387.68 K	Average (Å)	Standard Deviation (Å)	Slope (Å / μs)
RMSD	16.1985	5.3081	−0.27025
5d/5d' distance	48.789	18.9444	−0.895646
12d/12d' distance	39.6938	14.5247	−0.595765
19d/19d' distance	31.1348	10.5084	−0.298925
26d/26d' distance	20.9949	6.53775	0.00295972
2a/2a' distance	52.9023	20.6934	−1.03056
9a/9a' distance	43.1845	16.2595	−0.784585
16a/16a' distance	34.6823	12.2044	−0.442236
23a/23a' distance	25.4081	8.31809	−0.0693907
30a/30a' distance	14.1177	3.78139	−0.0105418
Core Distance	34.5453	18.212	

Figure S363. Plots of RMSD and residue packing distances for T-REMD simulations of variant **14f/17b'-xx** at 387.68 K.

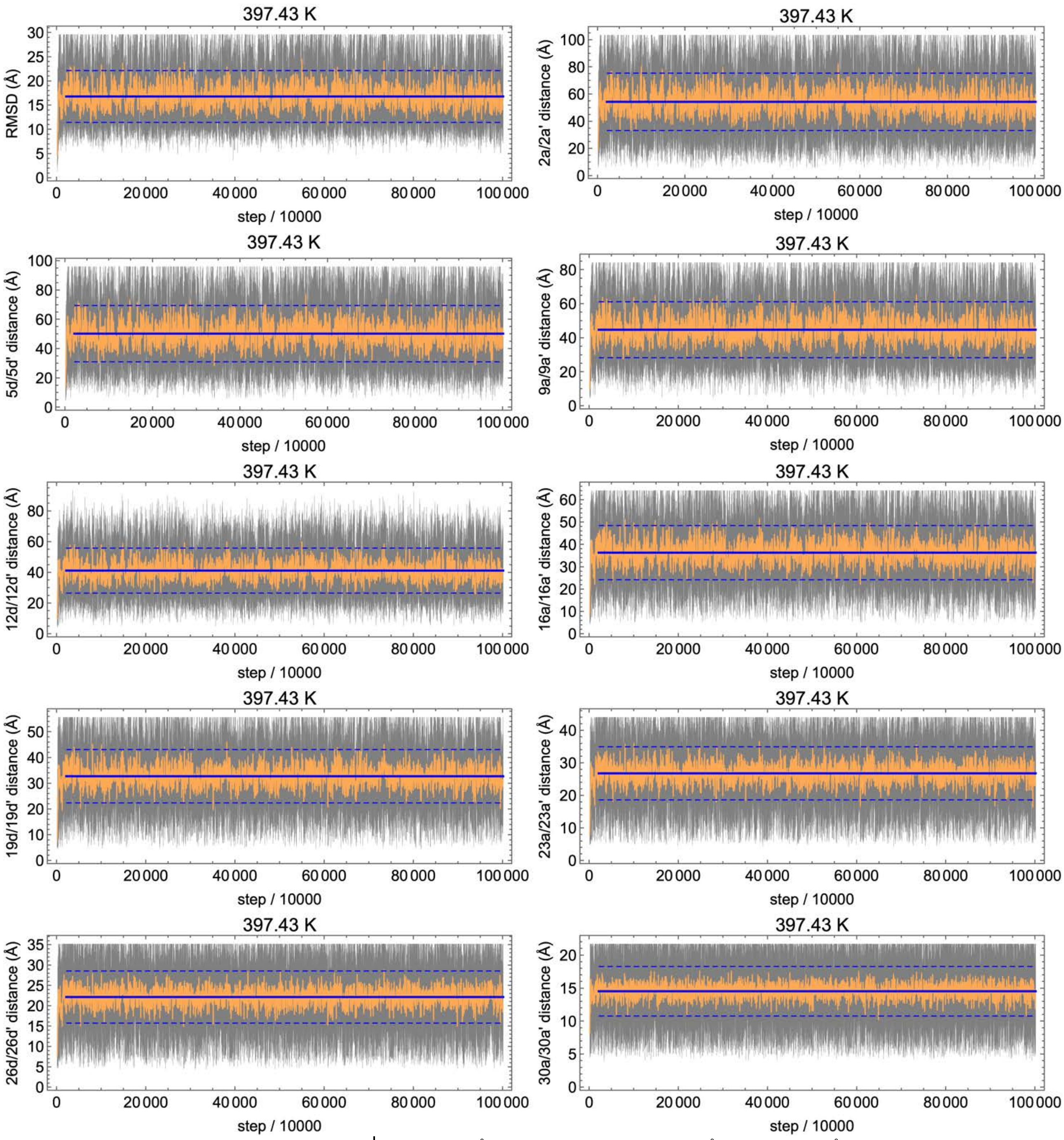

Measure at 397.43 K	Average (Å)	Standard Deviation (Å)	Slope (Å / μs)
RMSD	16.9207	5.35124	−0.190304
5d/5d' distance	50.6569	19.242	−0.651902
12d/12d' distance	41.5655	14.6398	−0.491527
19d/19d' distance	32.9606	10.3646	−0.188643
26d/26d' distance	22.3039	6.40318	−0.0502428
2a/2a' distance	54.7732	21.1067	−0.717156
9a/9a' distance	45.0759	16.449	−0.591499
16a/16a' distance	36.5859	12.1289	−0.317534
23a/23a' distance	26.9684	8.16023	−0.114441
30a/30a' distance	14.6203	3.75141	−0.0706828
Core Distance	36.1678	18.5383	

Figure S364. Plots of RMSD and residue packing distances for T-REMD simulations of variant **14f/17b'-xx** at 397.43 K.

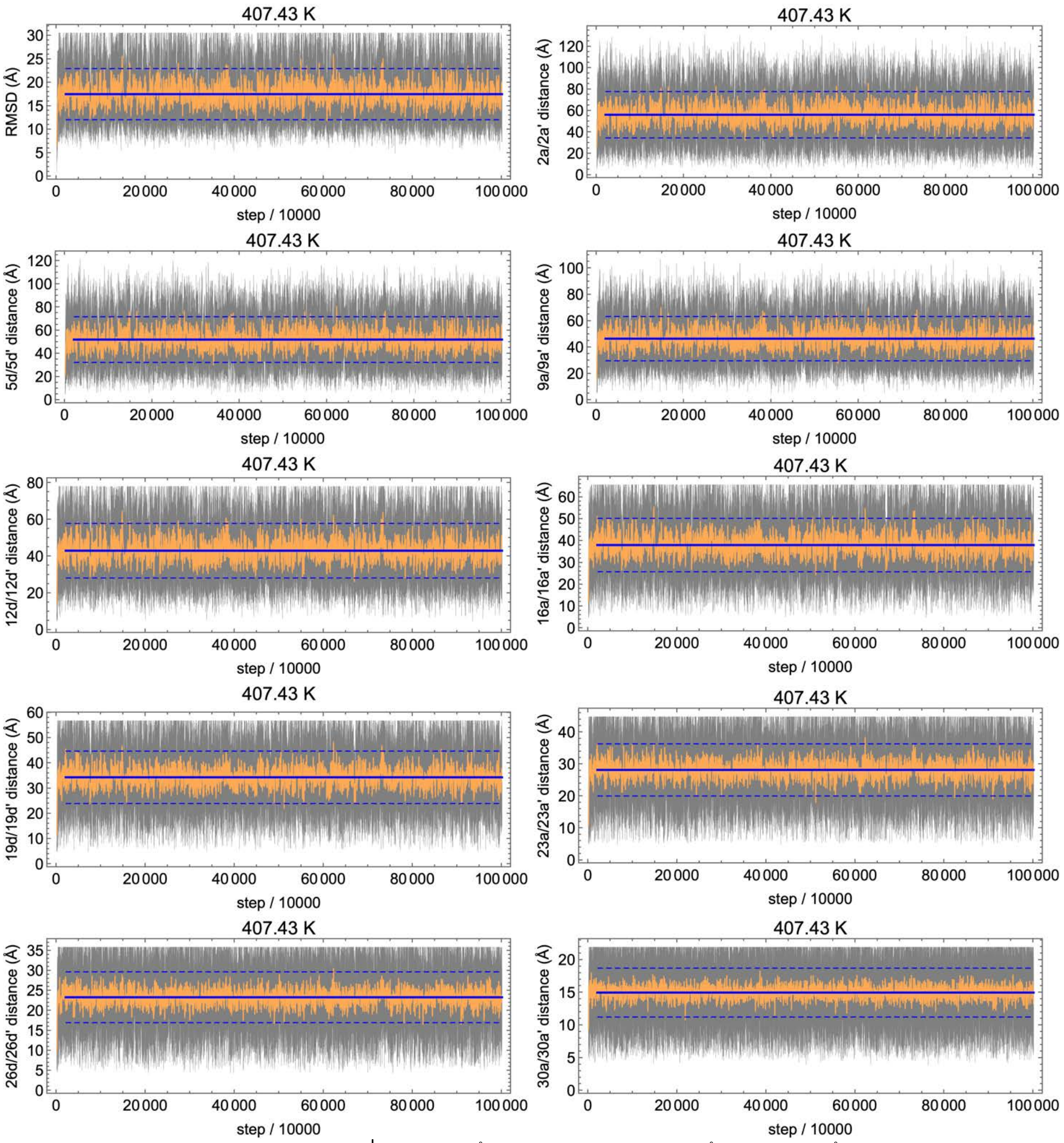

Measure at 407.43 K	Average (Å)	Standard Deviation (Å)	Slope (Å / μs)
RMSD	17.595	5.45174	−0.128877
5d/5d' distance	52.4214	19.695	−0.418845
12d/12d' distance	43.2593	14.8584	−0.39248
19d/19d' distance	34.529	10.391	−0.232155
26d/26d' distance	23.4258	6.35243	−0.0827364
2a/2a' distance	56.561	21.6425	−0.429331
9a/9a' distance	46.8266	16.7914	−0.451122
16a/16a' distance	38.2186	12.2346	−0.301639
23a/23a' distance	28.3202	8.14752	−0.176343
30a/30a' distance	15.0443	3.73656	−0.0389761
Core Distance	37.6229	18.9716	

Figure S365. Plots of RMSD and residue packing distances for T-REMD simulations of variant **14f/17b'-xx** at 407.43 K.

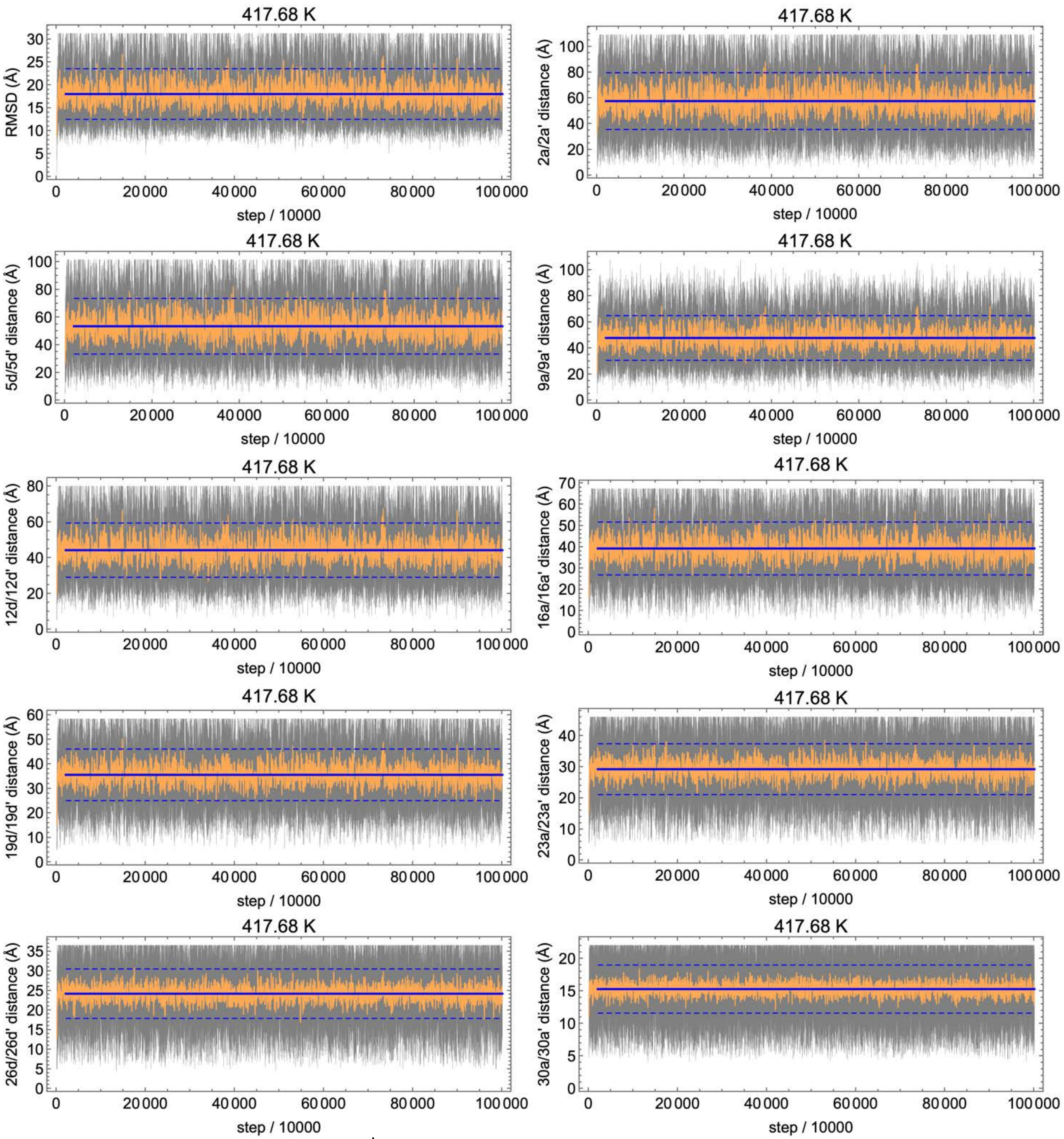

Measure at 417.68 K	Average (Å)	Standard Deviation (Å)	Slope (Å / μs)
RMSD	18.1293	5.52577	−0.117595
5d/5d' distance	53.7903	20.0687	−0.475561
12d/12d' distance	44.5037	15.1462	−0.344874
19d/19d' distance	35.7571	10.5216	−0.241195
26d/26d' distance	24.3132	6.31051	−0.0837687
2a/2a' distance	57.9901	22.0387	−0.413561
9a/9a' distance	48.0962	17.1365	−0.434386
16a/16a' distance	39.4716	12.435	−0.299194
23a/23a' distance	29.409	8.17243	−0.181935
30a/30a' distance	15.3683	3.70071	−0.0129615
Core Distance	38.7444	19.3387	

Figure S366. Plots of RMSD and residue packing distances for T-REMD simulations of variant **14f/17b’-xx** at 417.68 K.

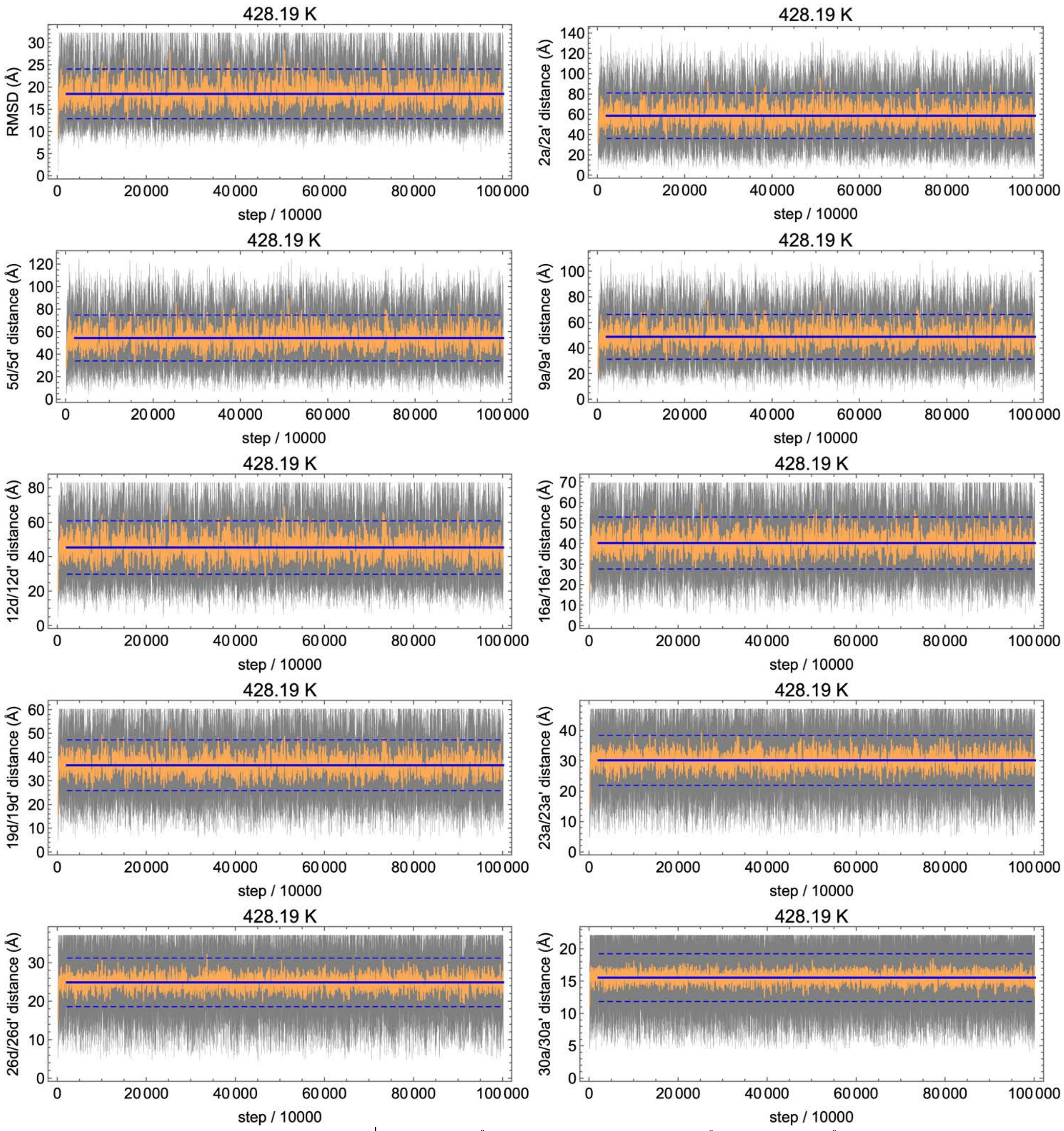

Measure at 428.19 K	Average (Å)	Standard Deviation (Å)	Slope (Å / μs)
RMSD	18.6095	5.59128	−0.0248331
5d/5d' distance	54.9967	20.4018	−0.190208
12d/12d' distance	45.6846	15.4699	−0.257867
19d/19d' distance	36.8194	10.7074	−0.15689
26d/26d' distance	25.0703	6.3215	−0.0410457
2a/2a' distance	59.2315	22.4033	−0.148551
9a/9a' distance	49.3081	17.4445	−0.238452
16a/16a' distance	40.6001	12.6835	−0.185636
23a/23a' distance	30.3654	8.22145	−0.116547
30a/30a' distance	15.6509	3.69128	−0.0484998
Core Distance	39.7474	19.694	

Figure S367. Plots of RMSD and residue packing distances for T-REMD simulations of variant **14f/17b'-xx** at 428.19 K.

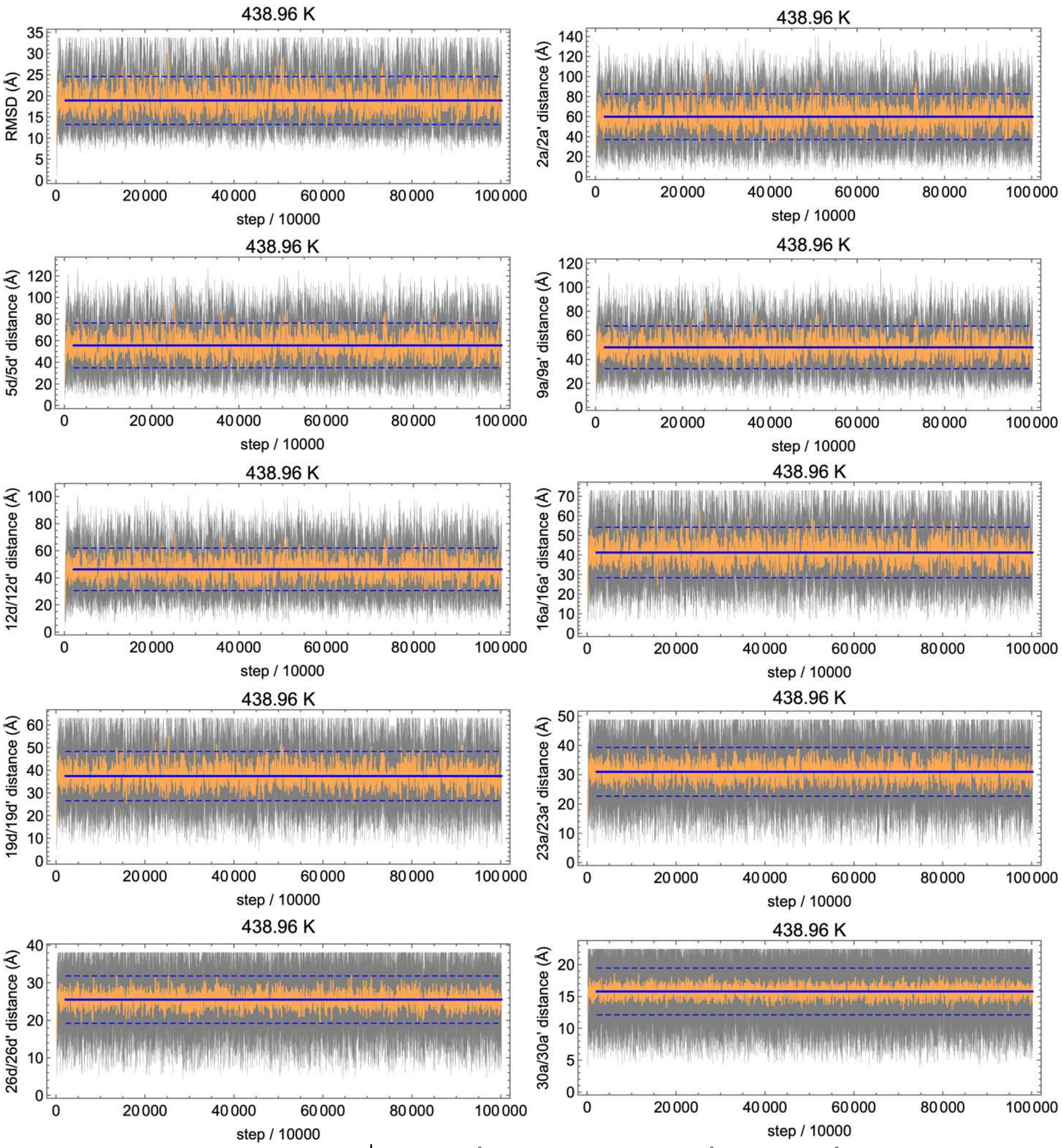

Measure at 438.96 K	Average (Å)	Standard Deviation (Å)	Slope (Å / μs)
RMSD	19.0733	5.67023	−0.0154412
5d/5d' distance	56.2237	20.7486	−0.183288
12d/12d' distance	46.7474	15.689	−0.177297
19d/19d' distance	37.7334	10.8708	−0.205432
26d/26d' distance	25.6833	6.31331	−0.100369
2a/2a' distance	60.5205	22.8106	−0.115719
9a/9a' distance	50.5007	17.7479	−0.209122
16a/16a' distance	41.5938	12.8773	−0.175087
23a/23a' distance	31.1955	8.30851	−0.205231
30a/30a' distance	15.8997	3.6728	−0.0520981
Core Distance	40.6776	20.0701	

Figure S368. Plots of RMSD and residue packing distances for T-REMD simulations of variant **14f/17b'-xx** at 438.96 K.

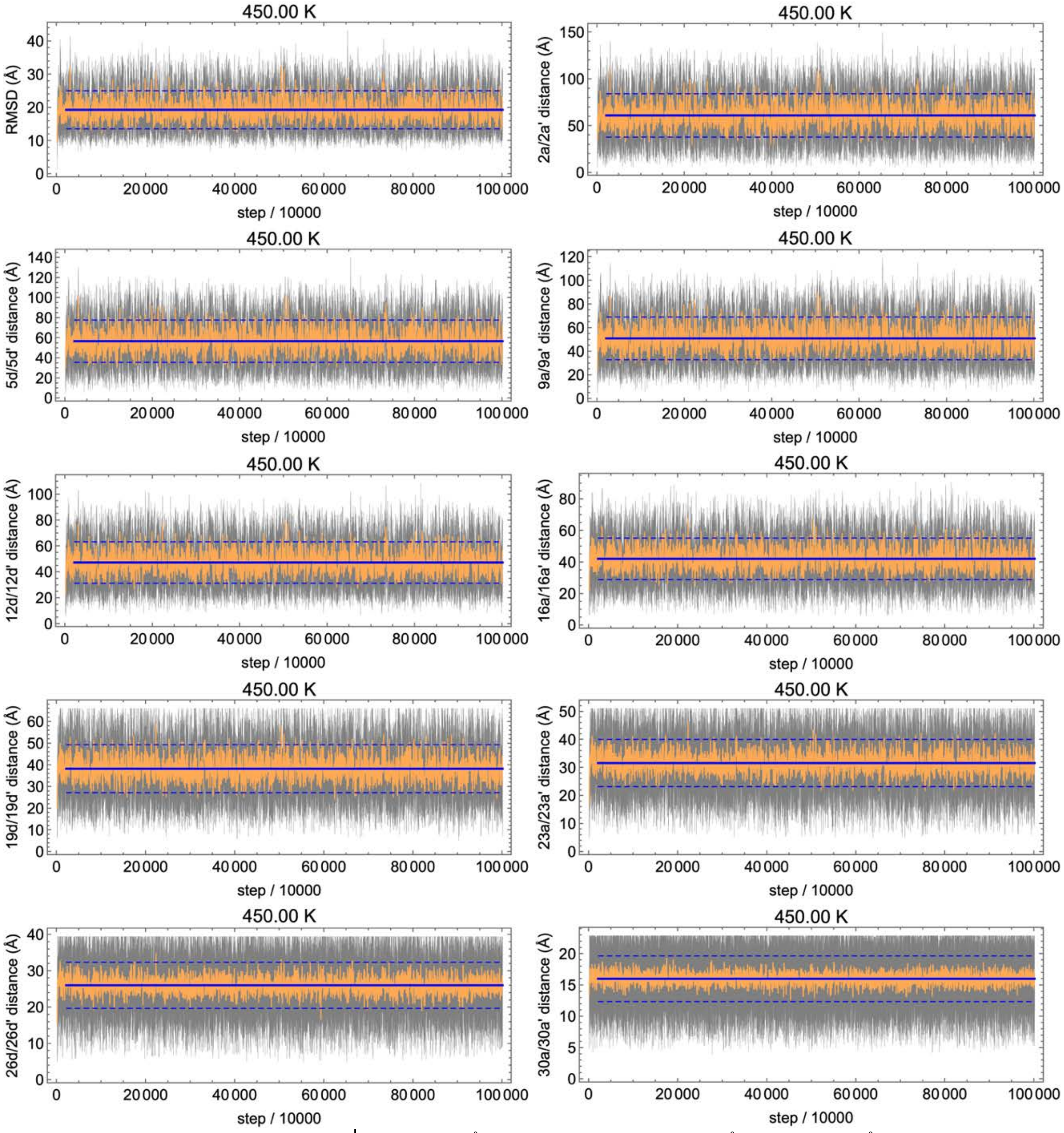

Measure at 450.00 K	Average (Å)	Standard Deviation (Å)	Slope (Å / μs)
RMSD	19.4578	5.74347	−0.0438091
5d/5d' distance	57.2146	21.064	−0.17329
12d/12d' distance	47.6563	15.9934	−0.202951
19d/19d' distance	38.5064	11.0943	−0.231089
26d/26d' distance	26.1859	6.33817	−0.178527
2a/2a' distance	61.4912	23.1541	−0.165951
9a/9a' distance	51.4292	18.0854	−0.175831
16a/16a' distance	42.3969	13.1752	−0.204064
23a/23a' distance	31.8089	8.42428	−0.252107
30a/30a' distance	16.0967	3.64518	−0.0599376
Core Distance	41.4207	20.4133	

Figure S369. Plots of RMSD and residue packing distances for T-REMD simulations of variant **14f/17b'-xx** at 450.00 K.

(return to Table of Contents)

s14f/17b'-x4x

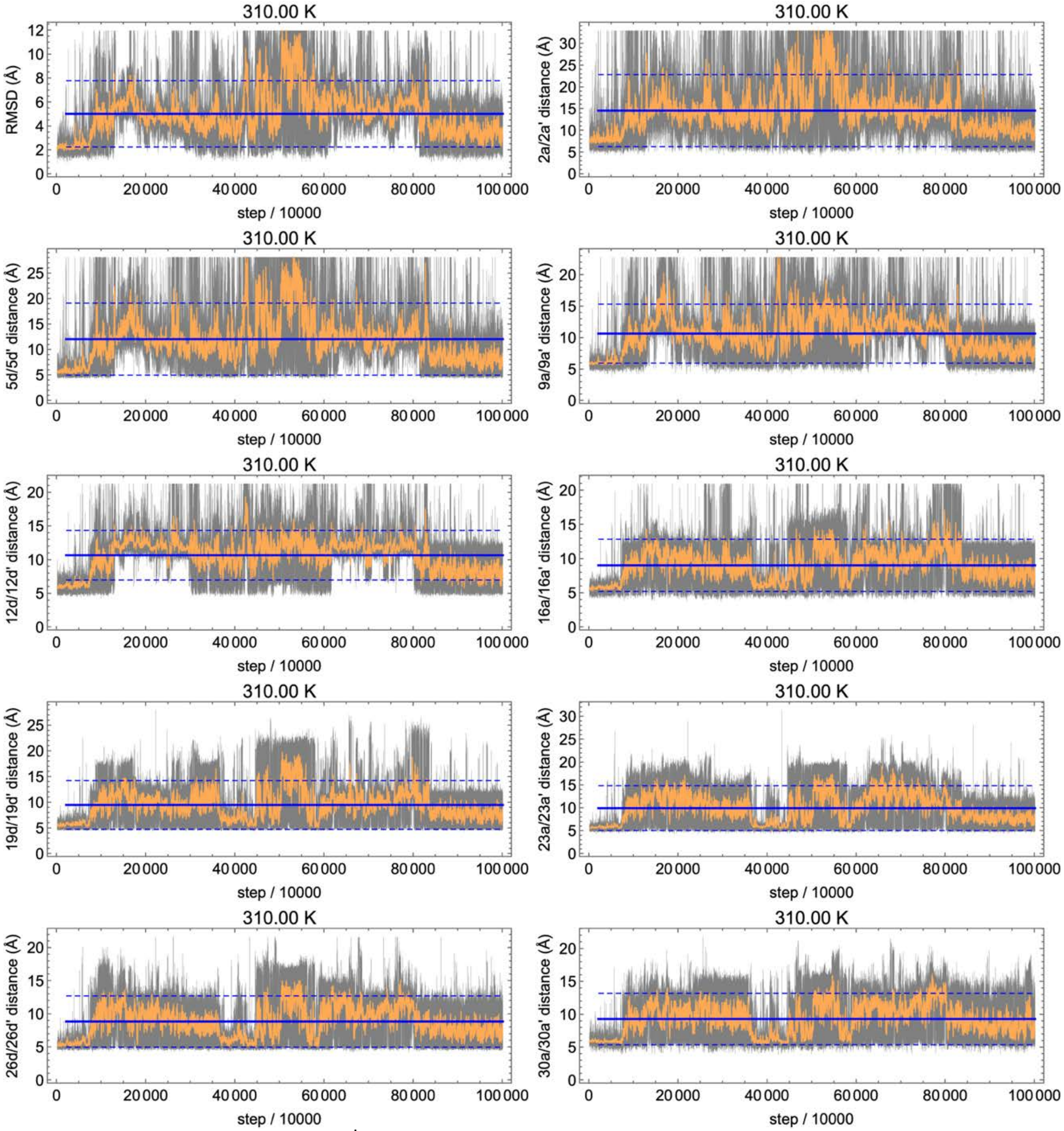

Measure at 310.00 K	Average (Å)	Standard Deviation (Å)	Slope (Å / μs)
RMSD	5.06667	2.76565	0.05659
5d/5d' distance	12.1534	7.07025	−0.522995
12d/12d' distance	10.7459	3.67374	−0.48583
19d/19d' distance	9.58894	4.74354	−0.0304073
26d/26d' distance	8.9315	3.8651	0.222092
2a/2a' distance	14.6853	8.28361	−0.674082
9a/9a' distance	10.7319	4.69101	−0.393592
16a/16a' distance	9.11163	3.8188	0.384248
23a/23a' distance	10.059	4.90039	−0.0537873
30a/30a' distance	9.38239	3.90893	0.589292
Core Distance	10.5989	5.50113	

Figure S370. Plots of RMSD and residue packing distances for T-REMD simulations of variant **s14f/17b'-x4x** at 310.00 K.

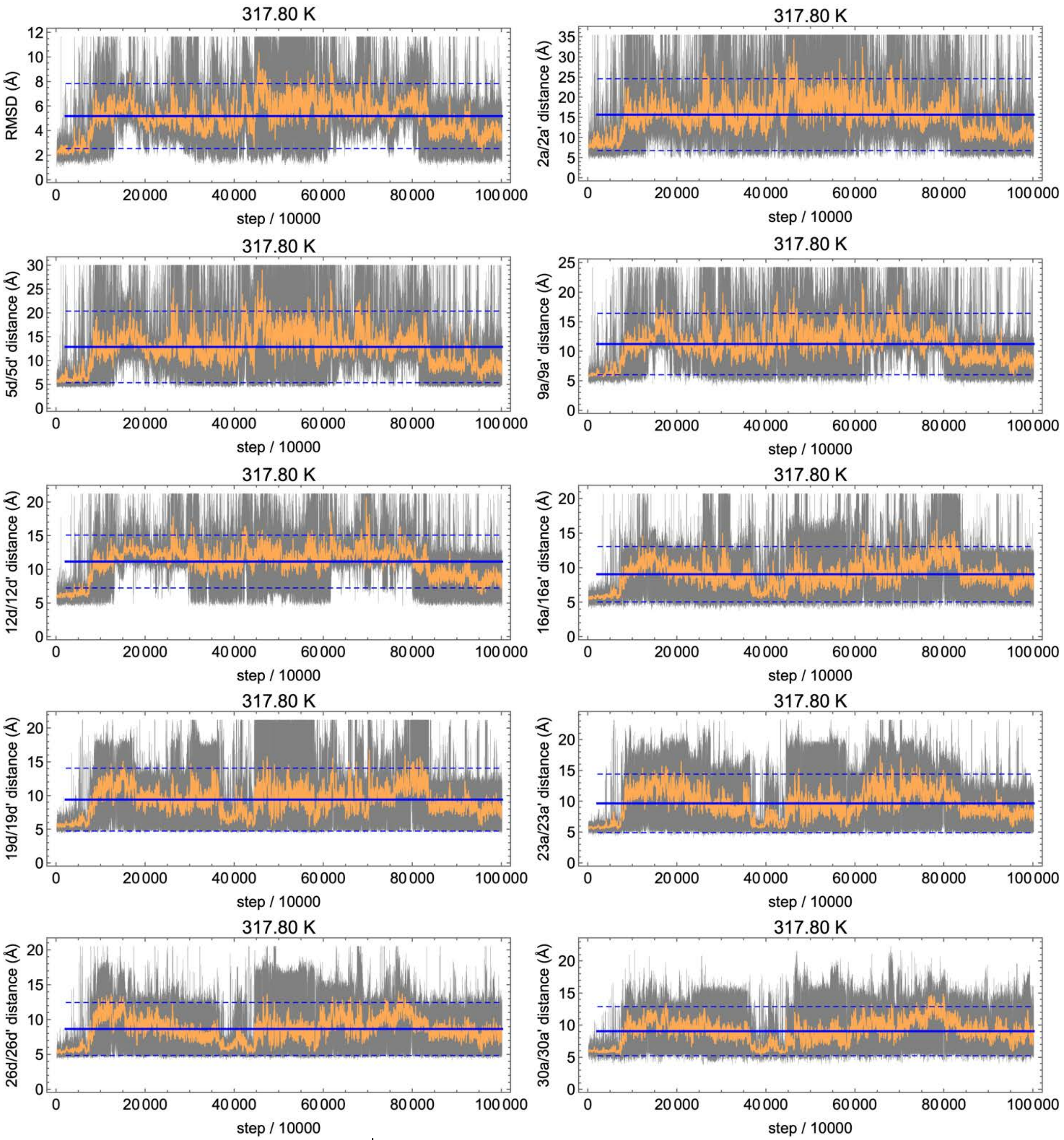

Measure at 317.80 K	Average (Å)	Standard Deviation (Å)	Slope (Å / μs)
RMSD	5.2358	2.64396	0.118799
5d/5d' distance	12.9876	7.50872	−0.34426
12d/12d' distance	11.2533	3.92474	−0.320513
19d/19d' distance	9.49107	4.66114	0.176581
26d/26d' distance	8.7474	3.79667	0.295459
2a/2a' distance	15.8242	8.90648	−0.531695
9a/9a' distance	11.3329	5.19658	−0.217841
16a/16a' distance	9.14454	4.01225	0.571832
23a/23a' distance	9.75529	4.74521	−0.0323603
30a/30a' distance	9.15276	3.82183	0.706754
Core Distance	10.8543	5.87307	

Figure S371. Plots of RMSD and residue packing distances for T-REMD simulations of variant **s14f/17b’-x4x** at 317.80 K.

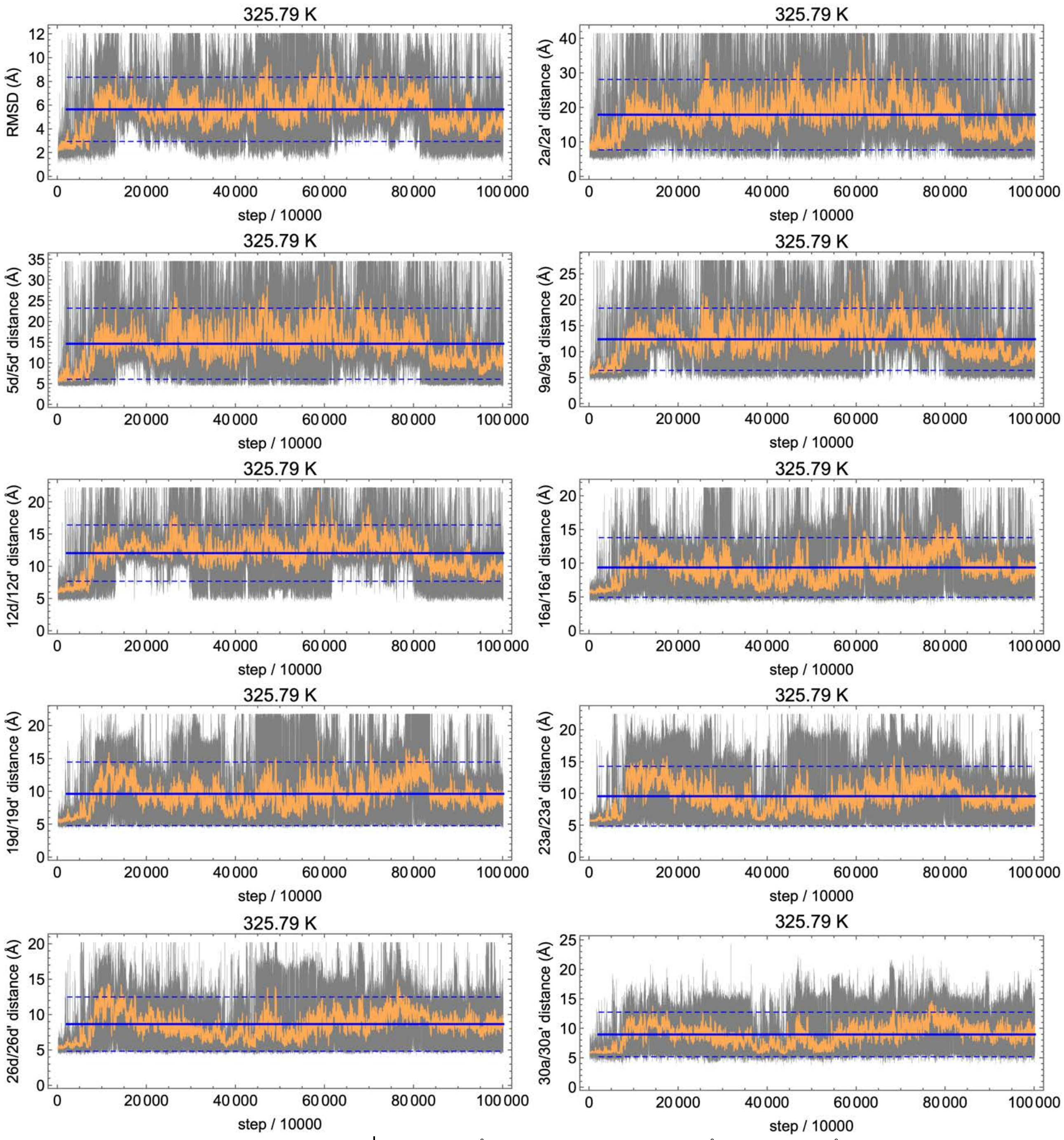

Measure at 325.79 K	Average (Å)	Standard Deviation (Å)	Slope (Å / μs)
RMSD	5.70223	2.70593	0.158816
5d/5d' distance	14.7781	8.57563	−0.263563
12d/12d' distance	12.1504	4.36841	−0.188518
19d/19d' distance	9.72514	4.83566	0.540397
26d/26d' distance	8.75068	3.835	0.473921
2a/2a' distance	18.0693	10.2067	−0.502607
9a/9a' distance	12.5194	5.9983	−0.125832
16a/16a' distance	9.47461	4.42121	0.835807
23a/23a' distance	9.67453	4.69518	0.10362
30a/30a' distance	9.07508	3.78363	0.899784
Core Distance	11.5797	6.71737	

Figure S372. Plots of RMSD and residue packing distances for T-REMD simulations of variant **s14f/17b'-x4x** at 325.79 K.

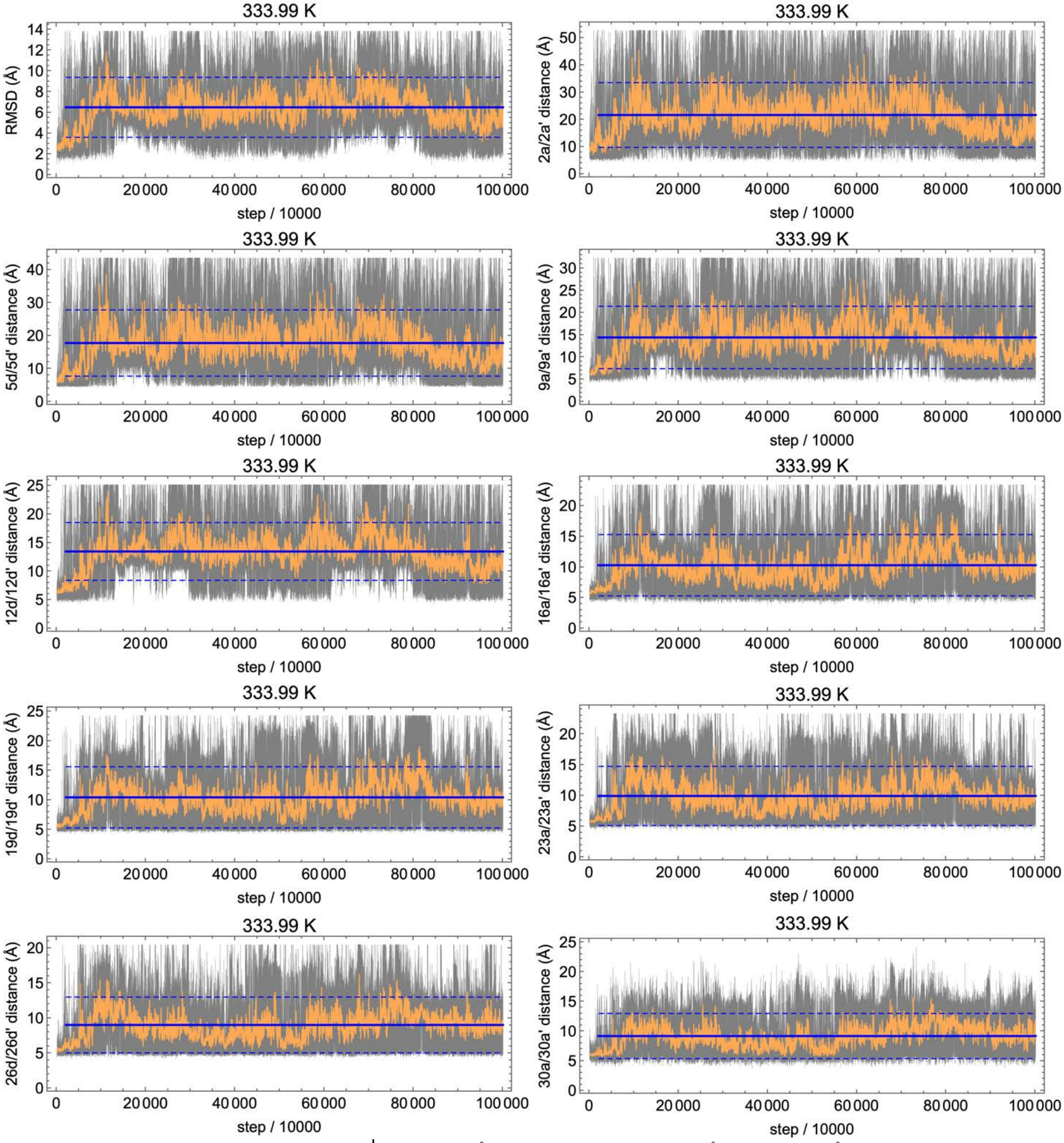

Measure at 333.99 K	Average (Å)	Standard Deviation (Å)	Slope (Å / μs)
RMSD	6.53669	2.88975	0.102089
5d/5d' distance	17.8861	10.0595	−0.477123
12d/12d' distance	13.5335	5.05889	−0.176382
19d/19d' distance	10.5131	5.17198	0.924245
26d/26d' distance	9.08151	3.98025	0.646347
2a/2a' distance	21.7912	11.8917	−0.783121
9a/9a' distance	14.5104	7.0261	−0.273077
16a/16a' distance	10.3702	5.00581	1.04749
23a/23a' distance	9.99738	4.80208	0.323363
30a/30a' distance	9.24323	3.80732	1.0302
Core Distance	12.9918	8.0144	

Figure S373. Plots of RMSD and residue packing distances for T-REMD simulations of variant **s14f/17b’-x4x** at 333.99 K.

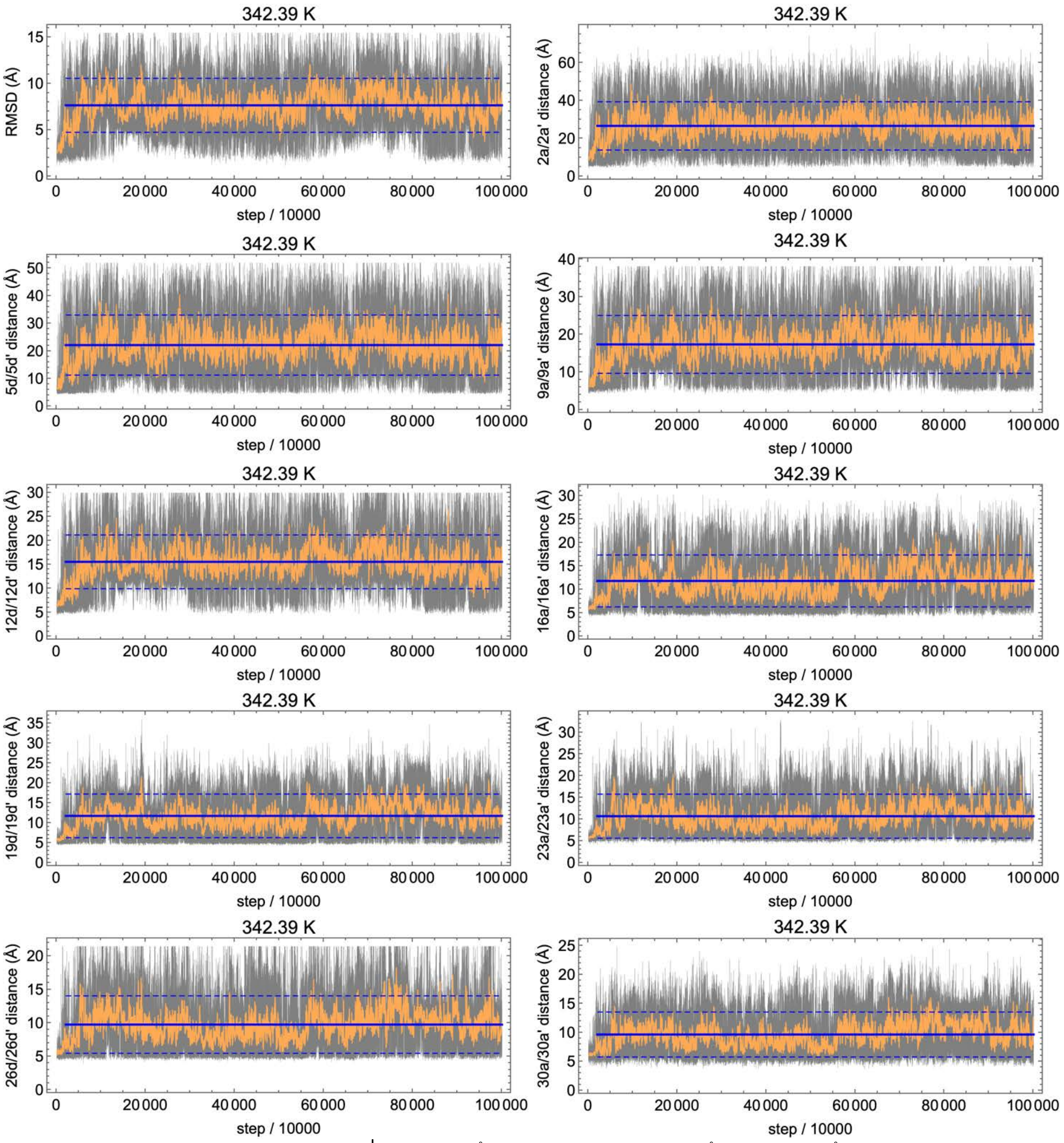

Measure at 342.39 K	Average (Å)	Standard Deviation (Å)	Slope (Å / μs)
RMSD	7.69322	2.90904	0.250302
5d/5d' distance	22.2726	10.8735	0.517585
12d/12d' distance	15.6207	5.61341	0.218151
19d/19d' distance	11.8428	5.47631	1.18673
26d/26d' distance	9.80682	4.29607	0.679199
2a/2a' distance	26.8038	12.7386	0.352775
9a/9a' distance	17.4579	7.68885	0.32483
16a/16a' distance	11.8851	5.54345	1.19098
23a/23a' distance	10.7909	5.06467	0.575519
30a/30a' distance	9.7133	3.88314	1.04972
Core Distance	15.1327	9.32041	

Figure S374. Plots of RMSD and residue packing distances for T-REMD simulations of variant **s14f/17b'-x4x** at 342.39 K.

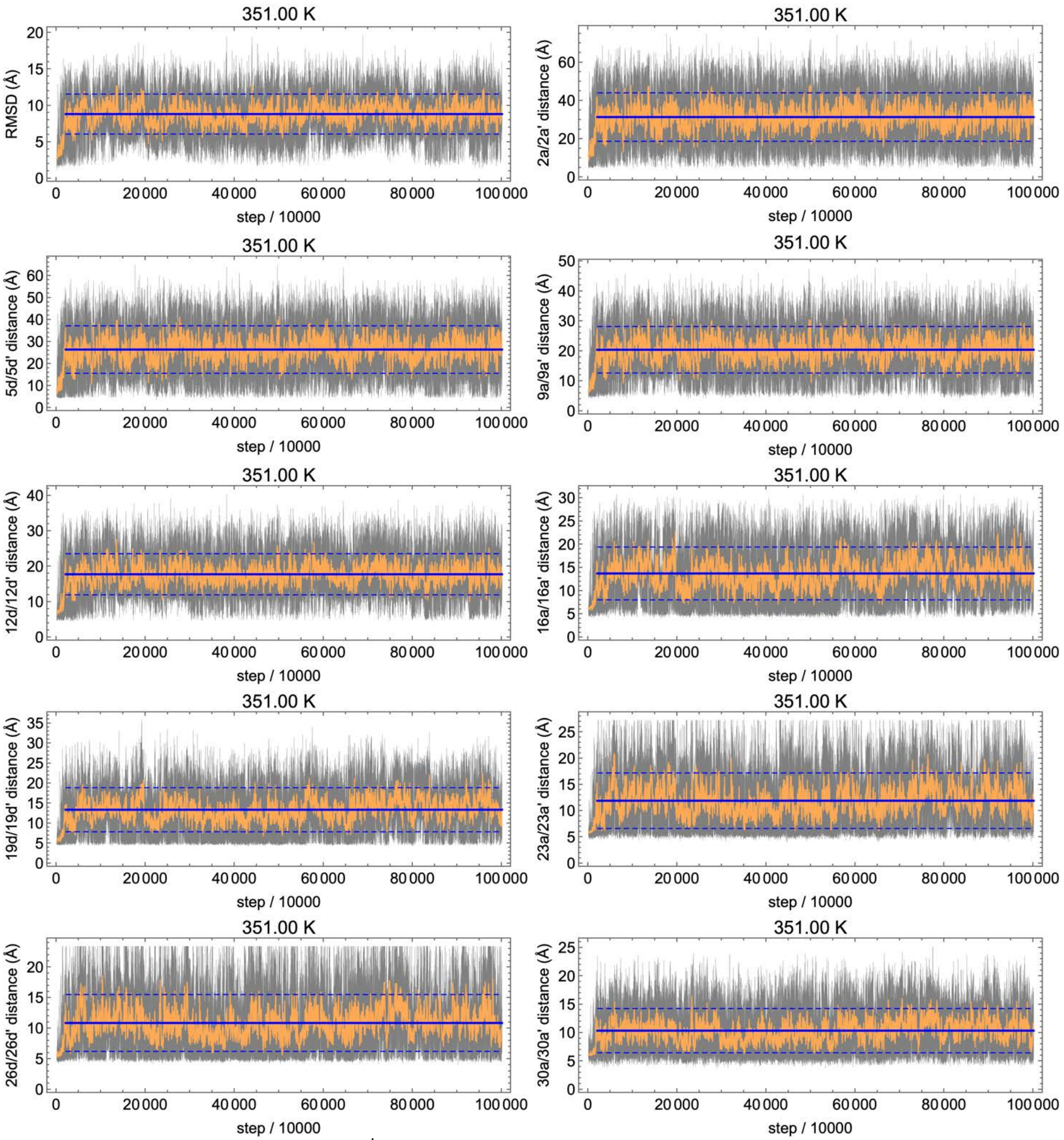

Measure at 351.00 K	Average (Å)	Standard Deviation (Å)	Slope (Å / μs)
RMSD	8.88274	2.74506	0.253395
5d/5d' distance	26.6258	10.7788	0.609693
12d/12d' distance	17.8926	5.81309	0.257459
19d/19d' distance	13.4912	5.50791	1.11373
26d/26d' distance	10.9424	4.6478	0.430356
2a/2a' distance	31.6246	12.653	0.643956
9a/9a' distance	20.5702	7.76058	0.349386
16a/16a' distance	13.8253	5.69672	1.0055
23a/23a' distance	12.0024	5.28724	0.513854
30a/30a' distance	10.4439	3.90096	0.656061
Core Distance	17.4909	10.2351	

Figure S375. Plots of RMSD and residue packing distances for T-REMD simulations of variant **s14f/17b'-x4x** at 351.00 K.

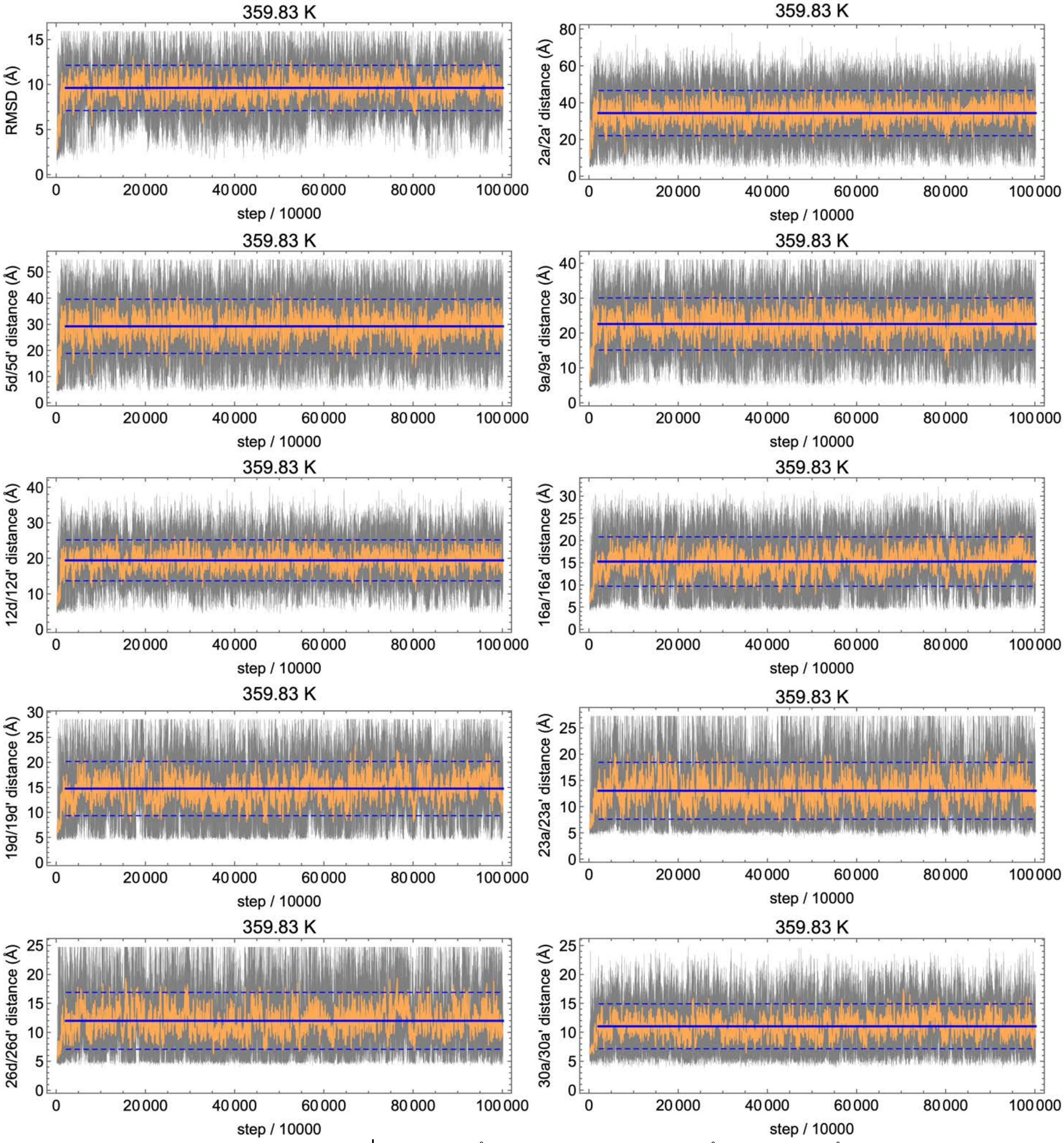

Measure at 359.83 K	Average (Å)	Standard Deviation (Å)	Slope (Å / μs)
RMSD	9.69131	2.51442	0.16299
5d/5d' distance	29.4766	10.3602	0.523289
12d/12d' distance	19.6112	5.76285	0.231031
19d/19d' distance	14.8932	5.41968	0.738918
26d/26d' distance	12.1009	4.9087	0.167303
2a/2a' distance	34.6861	12.2768	0.590931
9a/9a' distance	22.7684	7.46796	0.333967
16a/16a' distance	15.4057	5.55178	0.633043
23a/23a' distance	13.1703	5.41938	0.237697
30a/30a' distance	11.1484	3.89151	0.246713
Core Distance	19.2512	10.6389	

Figure S376. Plots of RMSD and residue packing distances for T-REMD simulations of variant **s14f/17b'-x4x** at 359.83 K.

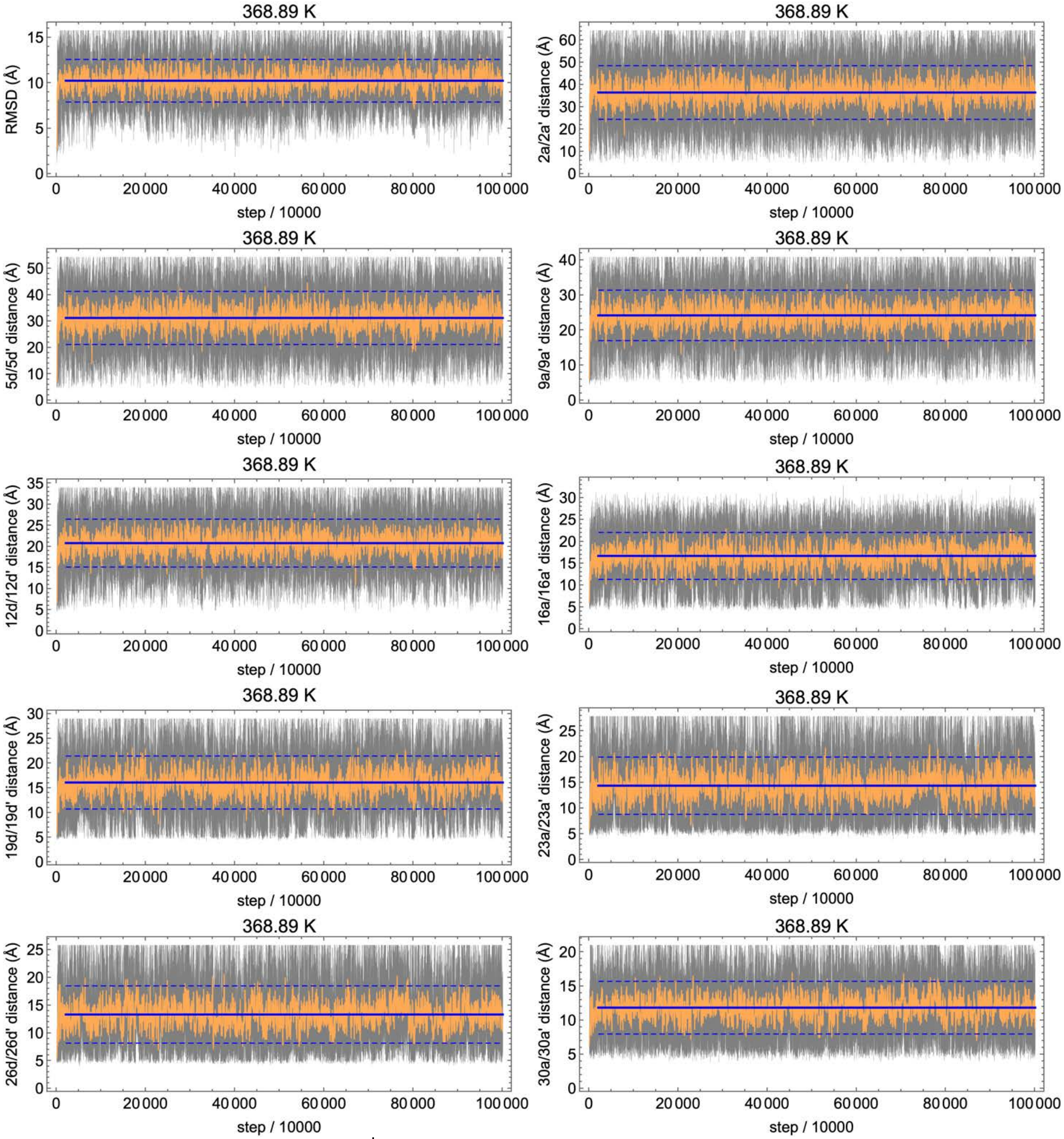

Measure at 368.89 K	Average (Å)	Standard Deviation (Å)	Slope (Å / μs)
RMSD	10.2915	2.3441	0.074133
5d/5d' distance	31.3907	10.0616	0.253347
12d/12d' distance	20.9087	5.67183	0.124056
19d/19d' distance	16.181	5.36499	0.370899
26d/26d' distance	13.4131	5.15975	0.107521
2a/2a' distance	36.6914	12.0484	0.338511
9a/9a' distance	24.3372	7.20035	0.188577
16a/16a' distance	16.7972	5.37049	0.345034
23a/23a' distance	14.4587	5.55708	0.113409
30a/30a' distance	11.9069	3.85801	0.124661
Core Distance	20.6761	10.7947	

Figure S377. Plots of RMSD and residue packing distances for T-REMD simulations of variant **s14f/17b'-x4x** at 368.89 K.

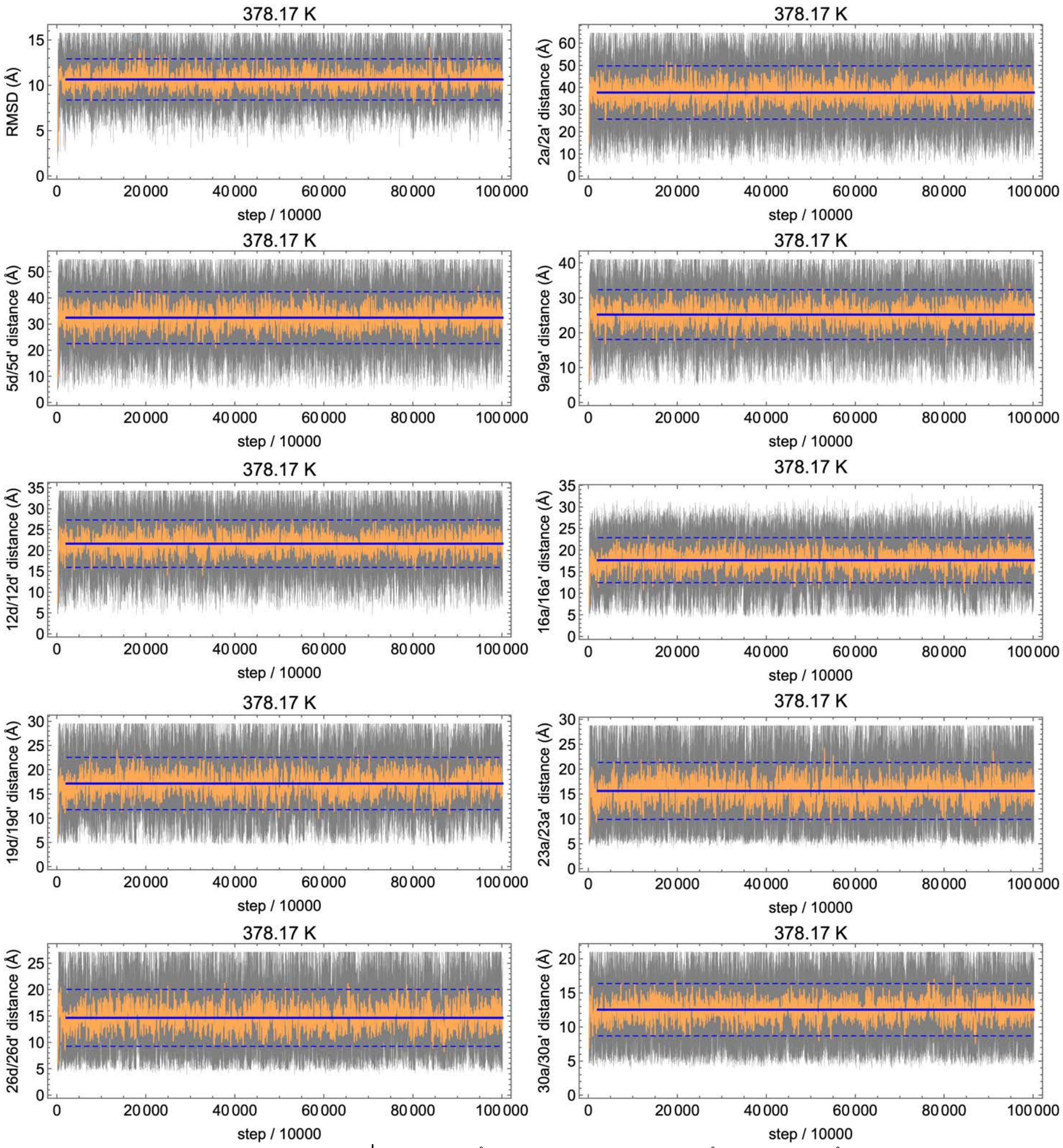

Measure at 378.17 K	Average (Å)	Standard Deviation (Å)	Slope (Å / μs)
RMSD	10.7173	2.26638	0.0557955
5d/5d' distance	32.6782	9.9156	0.228709
12d/12d' distance	21.7879	5.68492	0.0891898
19d/19d' distance	17.301	5.39617	0.154715
26d/26d' distance	14.7811	5.39727	0.0597642
2a/2a' distance	38.0102	12.0265	0.315065
9a/9a' distance	25.3678	7.12189	0.162571
16a/16a' distance	17.8142	5.21016	0.167983
23a/23a' distance	15.7485	5.71185	0.0391185
30a/30a' distance	12.6247	3.83997	0.0876375
Core Distance	21.7904	10.8464	

Figure S378. Plots of RMSD and residue packing distances for T-REMD simulations of variant **s14f/17b'-x4x** at 378.17 K.

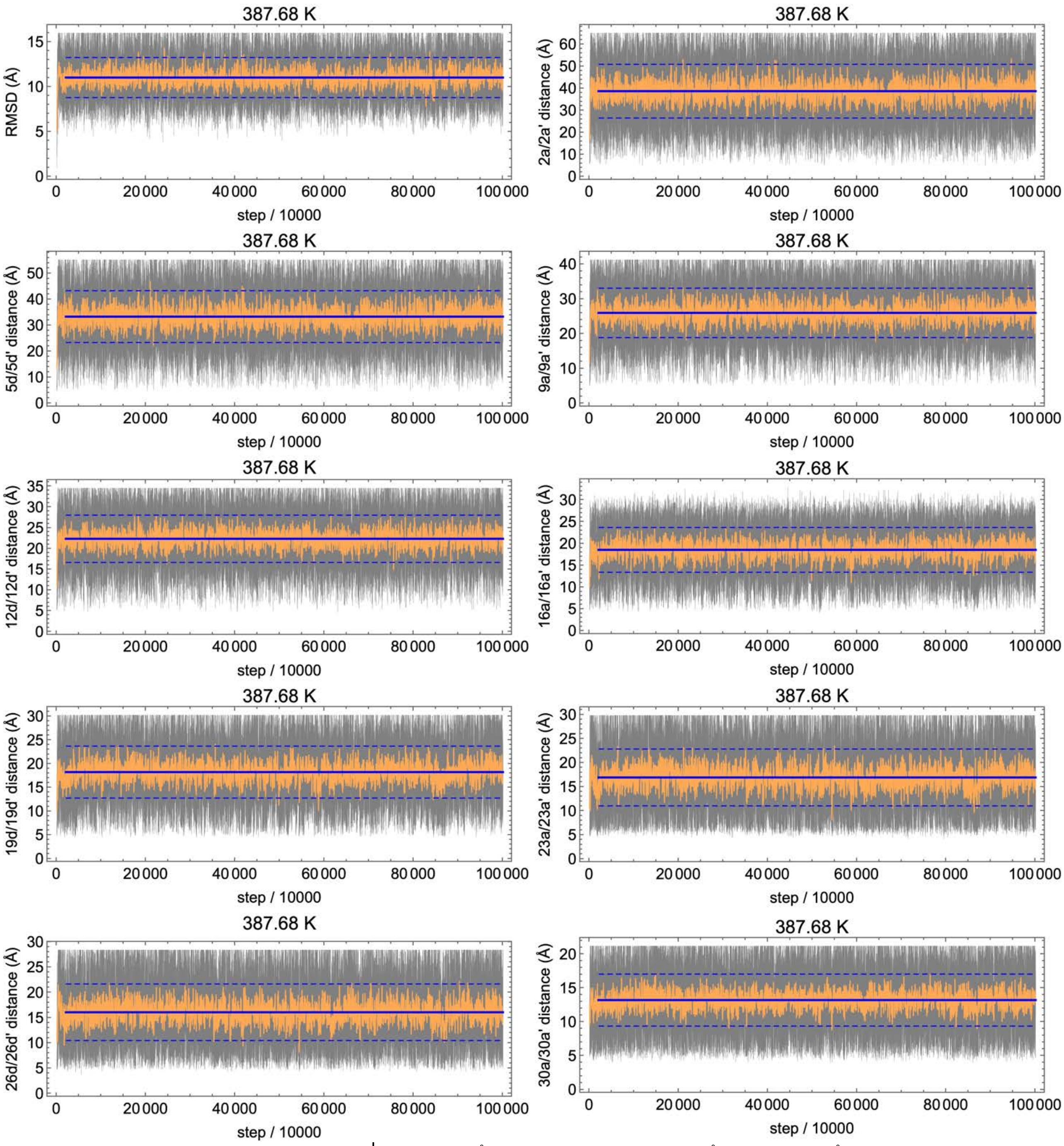

Measure at 387.68 K	Average (Å)	Standard Deviation (Å)	Slope (Å / μs)
RMSD	11.0623	2.23269	0.00290372
5d/5d' distance	33.4899	9.979	0.172256
12d/12d' distance	22.4431	5.68719	0.0041143
19d/19d' distance	18.3152	5.47148	−0.0638461
26d/26d' distance	16.1536	5.59862	−0.0560502
2a/2a' distance	38.9079	12.189	0.139573
9a/9a' distance	26.1012	7.10605	0.144587
16a/16a' distance	18.634	5.1312	0.0255461
23a/23a' distance	17.0158	5.90767	−0.152566
30a/30a' distance	13.2684	3.83727	0.0329483
Core Distance	22.7032	10.8467	

Figure S379. Plots of RMSD and residue packing distances for T-REMD simulations of variant **s14f/17b'-x4x** at 387.68 K.

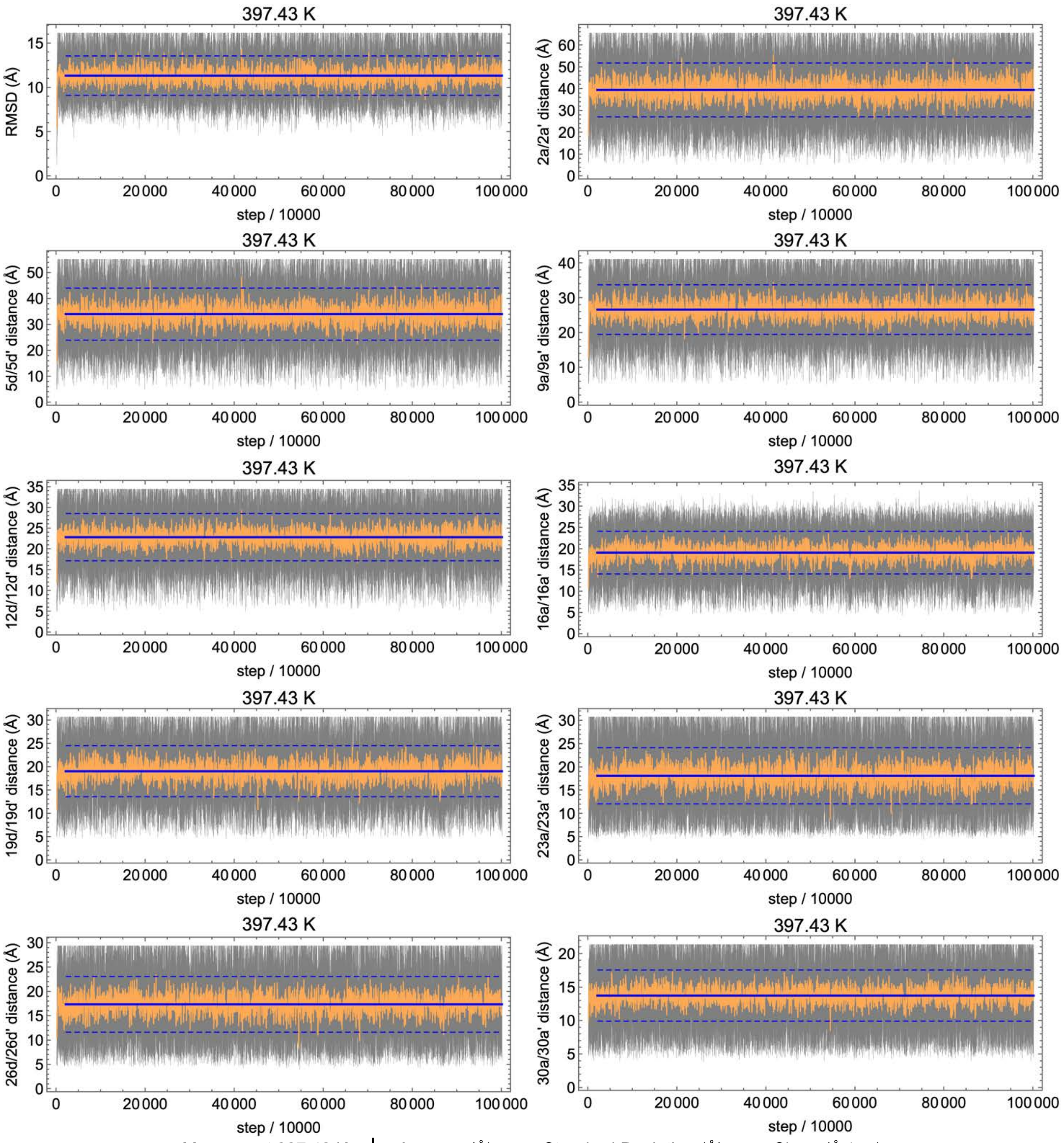

Measure at 397.43 K	Average (Å)	Standard Deviation (Å)	Slope (Å / μs)
RMSD	11.3935	2.22586	−0.00358488
5d/5d' distance	34.2256	10.0521	0.0161932
12d/12d' distance	22.9876	5.68221	−0.0350338
19d/19d' distance	19.1576	5.47684	−0.0656958
26d/26d' distance	17.4701	5.72315	−0.00375378
2a/2a' distance	39.6916	12.3557	0.0685423
9a/9a' distance	26.7761	7.11478	0.0177762
16a/16a' distance	19.2308	5.01949	0.0178404
23a/23a' distance	18.2207	6.03508	−0.0714678
30a/30a' distance	13.8293	3.82483	−0.00719412
Core Distance	23.5099	10.8456	

Figure S380. Plots of RMSD and residue packing distances for T-REMD simulations of variant **s14f/17b'-x4x** at 397.43 K.

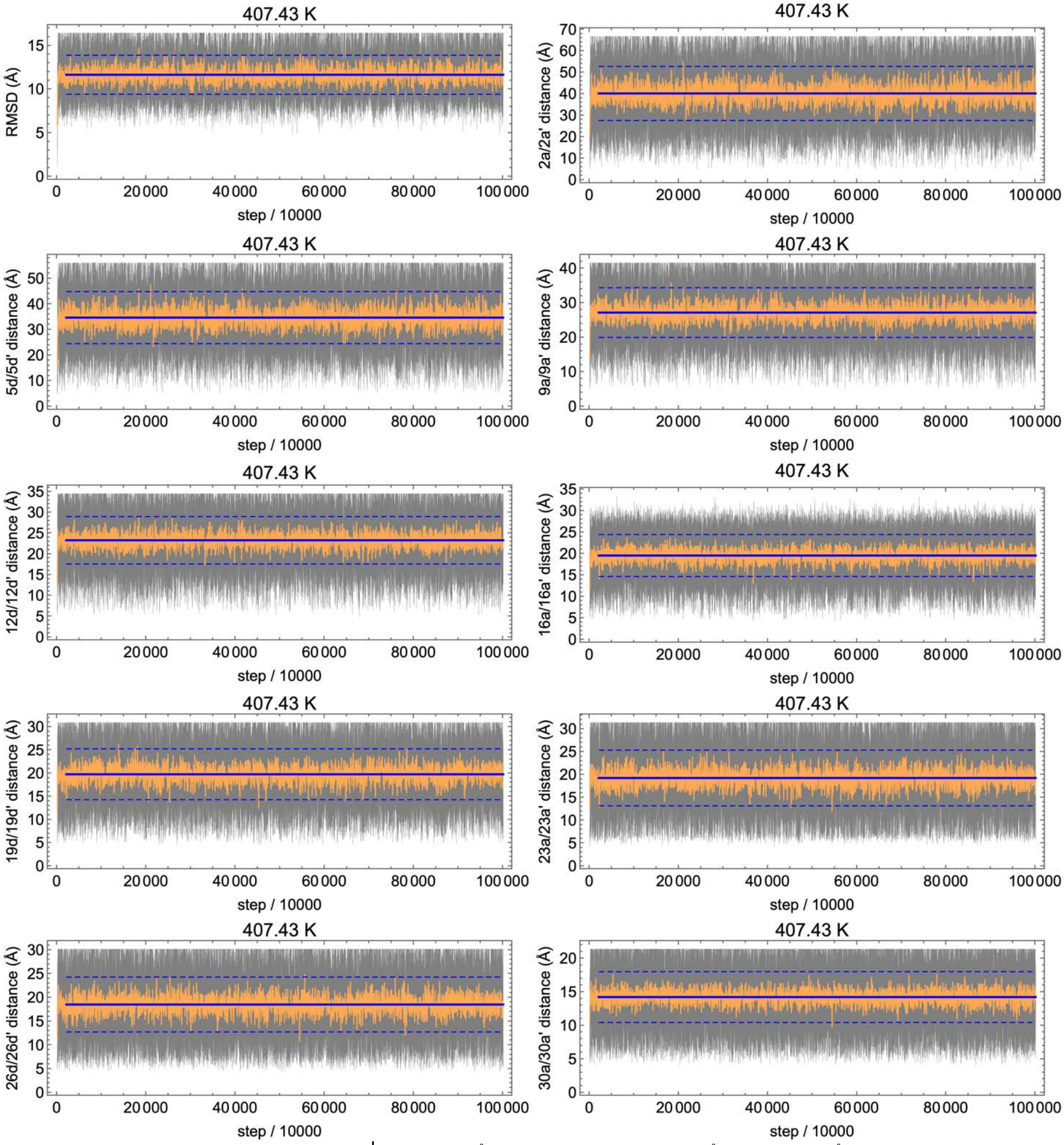

Measure at 407.43 K	Average (Å)	Standard Deviation (Å)	Slope (Å / μs)
RMSD	11.6974	2.23981	0.009908
5d/5d' distance	34.8304	10.1733	0.104818
12d/12d' distance	23.3833	5.70392	−0.00425646
19d/19d' distance	19.8589	5.48394	−0.0507374
26d/26d' distance	18.5969	5.77046	−0.0053505
2a/2a' distance	40.3774	12.5847	0.0872677
9a/9a' distance	27.2854	7.19744	0.0399424
16a/16a' distance	19.6462	4.89522	0.018409
23a/23a' distance	19.3493	6.09536	−0.0449543
30a/30a' distance	14.2939	3.78846	−0.0592353
Core Distance	24.1802	10.8657	

Figure S381. Plots of RMSD and residue packing distances for T-REMD simulations of variant **s14f/17b'-x4x** at 407.43 K.

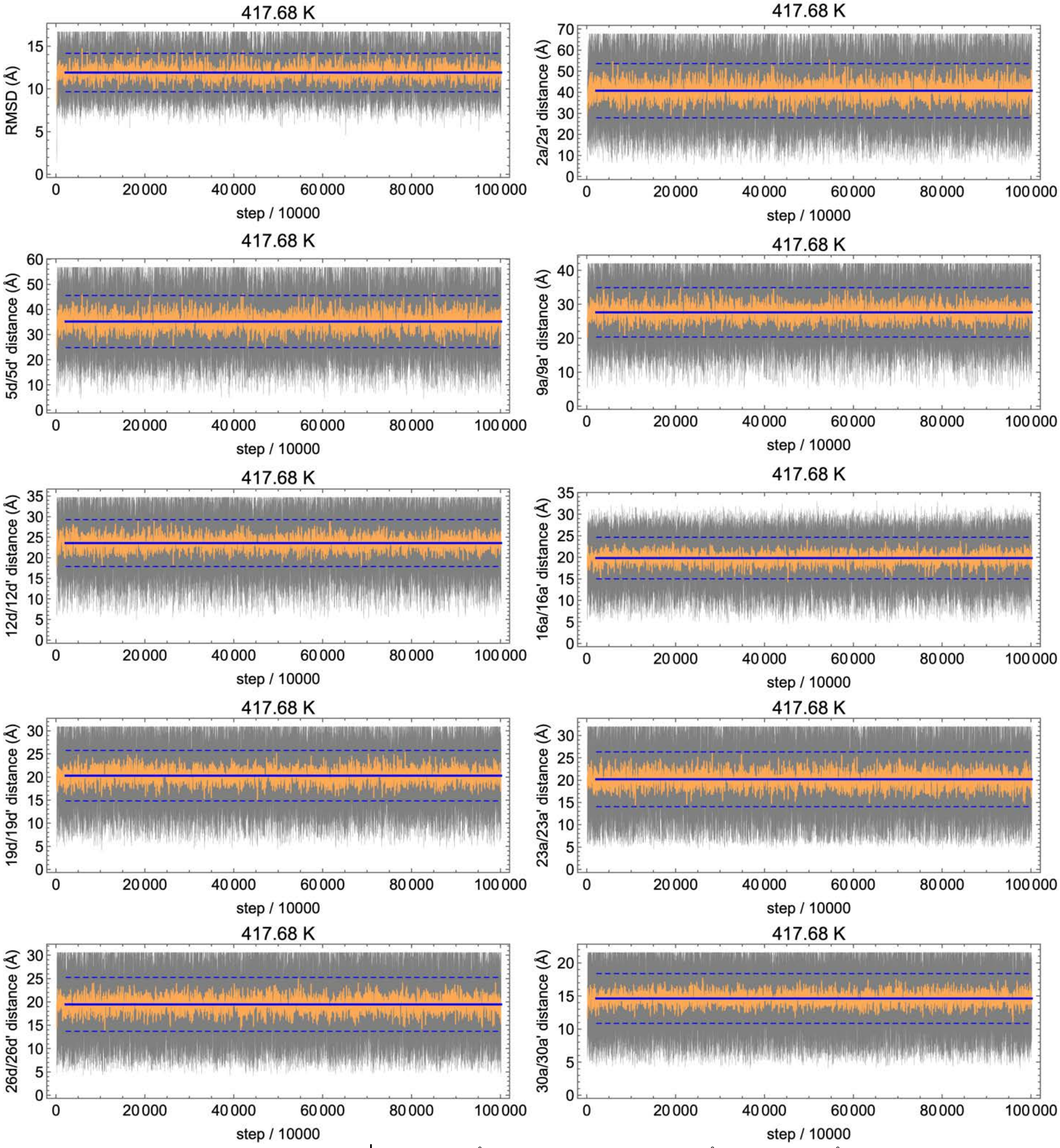

Measure at 417.68 K	Average (Å)	Standard Deviation (Å)	Slope (Å / μs)
RMSD	11.9927	2.24745	−0.0141787
5d/5d' distance	35.4589	10.3657	0.0627324
12d/12d' distance	23.7441	5.69595	−0.0277721
19d/19d' distance	20.4393	5.46283	−0.0827739
26d/26d' distance	19.6091	5.77575	−0.0453241
2a/2a' distance	41.0519	12.8702	0.0162924
9a/9a' distance	27.8409	7.28065	−0.0243253
16a/16a' distance	19.9841	4.82131	−0.0235752
23a/23a' distance	20.3485	6.14142	−0.153158
30a/30a' distance	14.7261	3.76232	−0.0182838
Core Distance	24.8003	10.9311	

Figure S382. Plots of RMSD and residue packing distances for T-REMD simulations of variant **s14f/17b'-x4x** at 417.68 K.

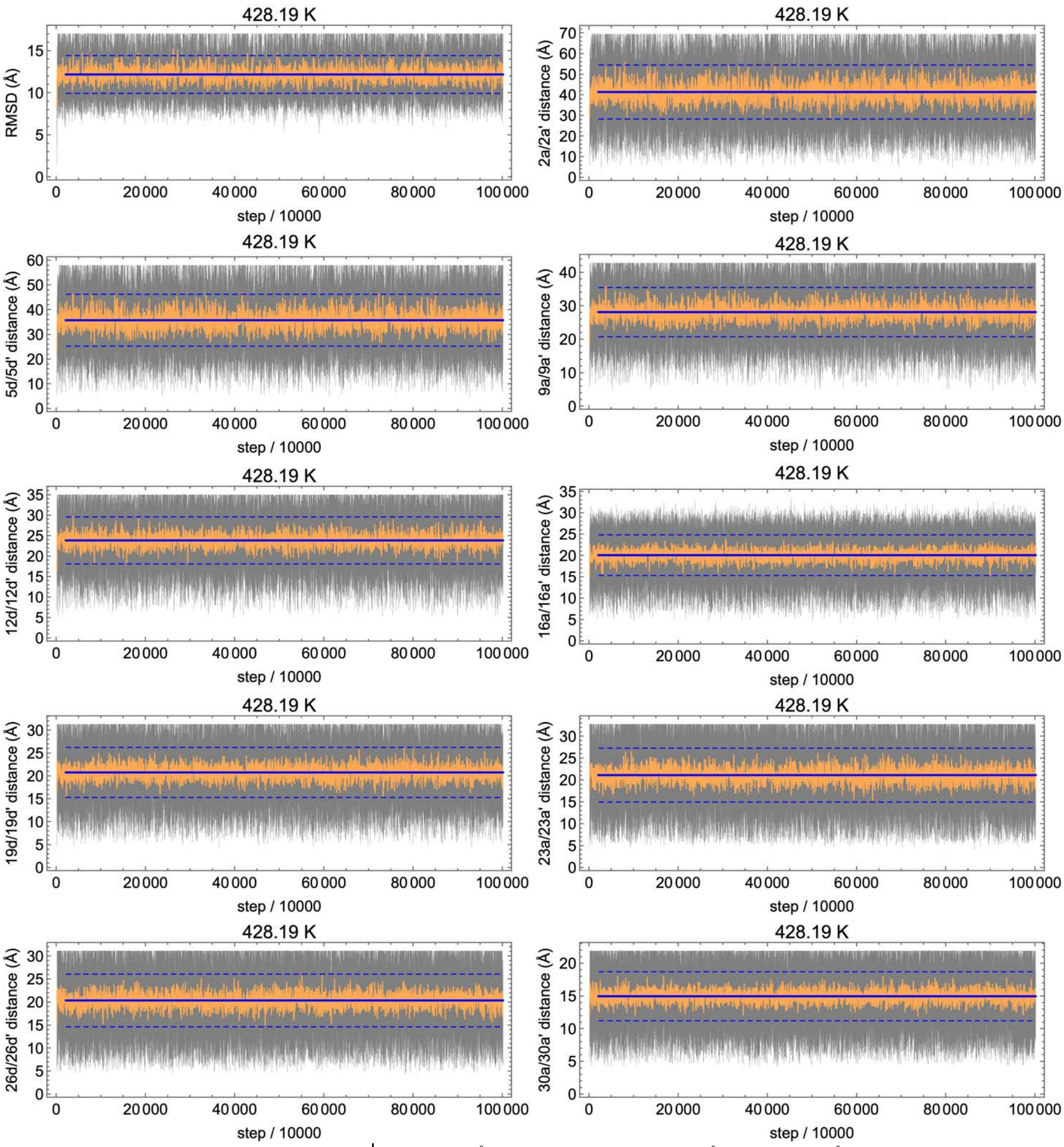

Measure at 428.19 K	Average (Å)	Standard Deviation (Å)	Slope (Å / μs)
RMSD	12.2506	2.24435	0.00571974
5d/5d' distance	35.9912	10.491	0.206686
12d/12d' distance	23.9912	5.73938	0.0598337
19d/19d' distance	20.898	5.46331	−0.0371712
26d/26d' distance	20.4825	5.72918	0.0355305
2a/2a' distance	41.6593	13.0838	0.218088
9a/9a' distance	28.2894	7.34765	0.0819302
16a/16a' distance	20.2202	4.75841	−0.00735813
23a/23a' distance	21.2462	6.14855	−0.087879
30a/30a' distance	15.0499	3.74503	0.0152972
Core Distance	25.3142	10.9938	

Figure S383. Plots of RMSD and residue packing distances for T-REMD simulations of variant **s14f/17b'-x4x** at 428.19 K.

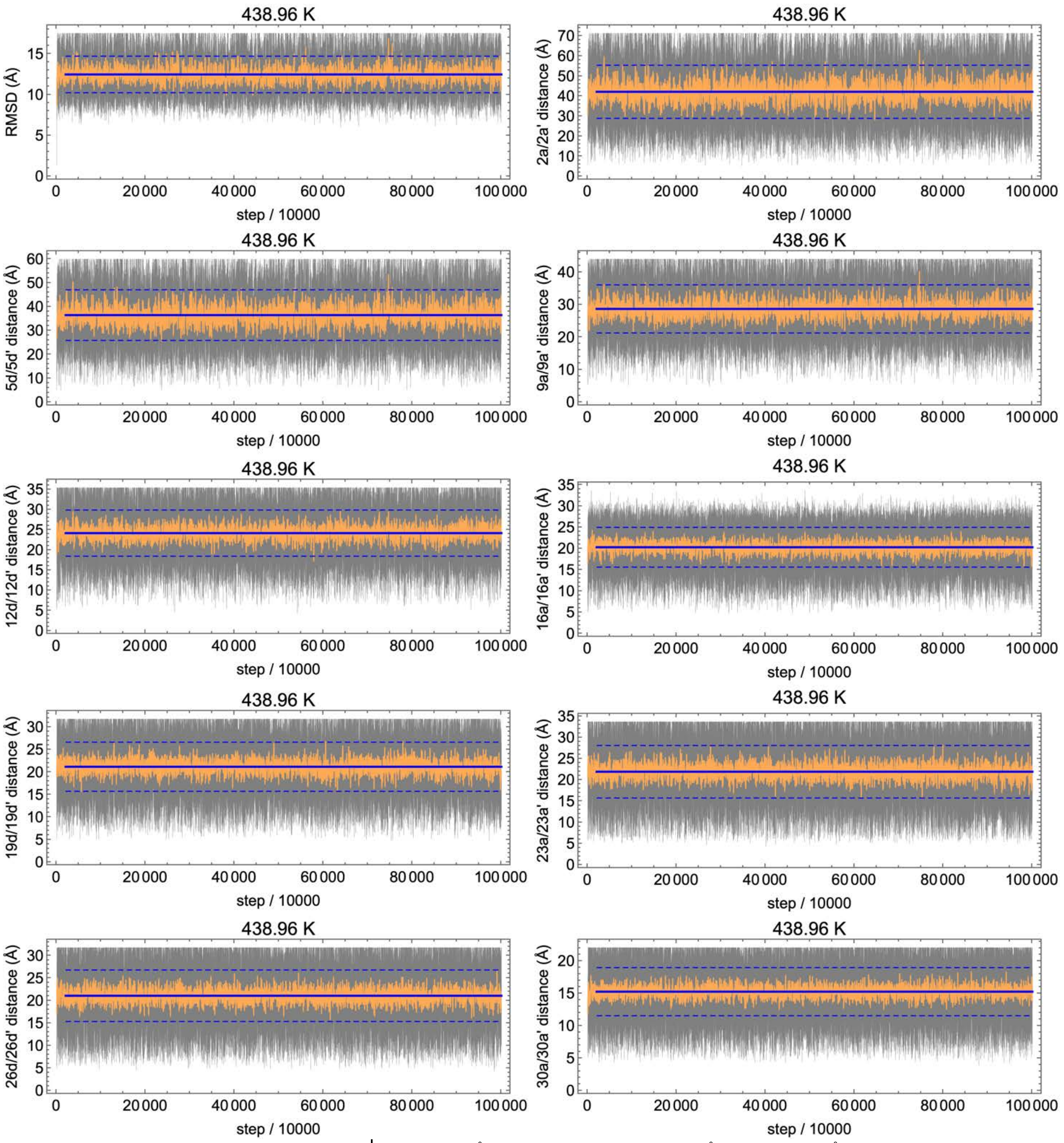

Measure at 438.96 K	Average (Å)	Standard Deviation (Å)	Slope (Å / μs)
RMSD	12.5079	2.24	−0.0118791
5d/5d' distance	36.6074	10.6021	0.19689
12d/12d' distance	24.2566	5.70879	0.039618
19d/19d' distance	21.2549	5.45363	−0.122802
26d/26d' distance	21.1494	5.72159	−0.0795894
2a/2a' distance	42.3139	13.2461	0.209269
9a/9a' distance	28.7905	7.39996	0.0903531
16a/16a' distance	20.3839	4.67767	−0.07434
23a/23a' distance	21.9946	6.18713	−0.185433
30a/30a' distance	15.3191	3.71102	0.0245393
Core Distance	25.7856	11.0976	

Figure S384. Plots of RMSD and residue packing distances for T-REMD simulations of variant **s14f/17b'-x4x** at 438.96 K.

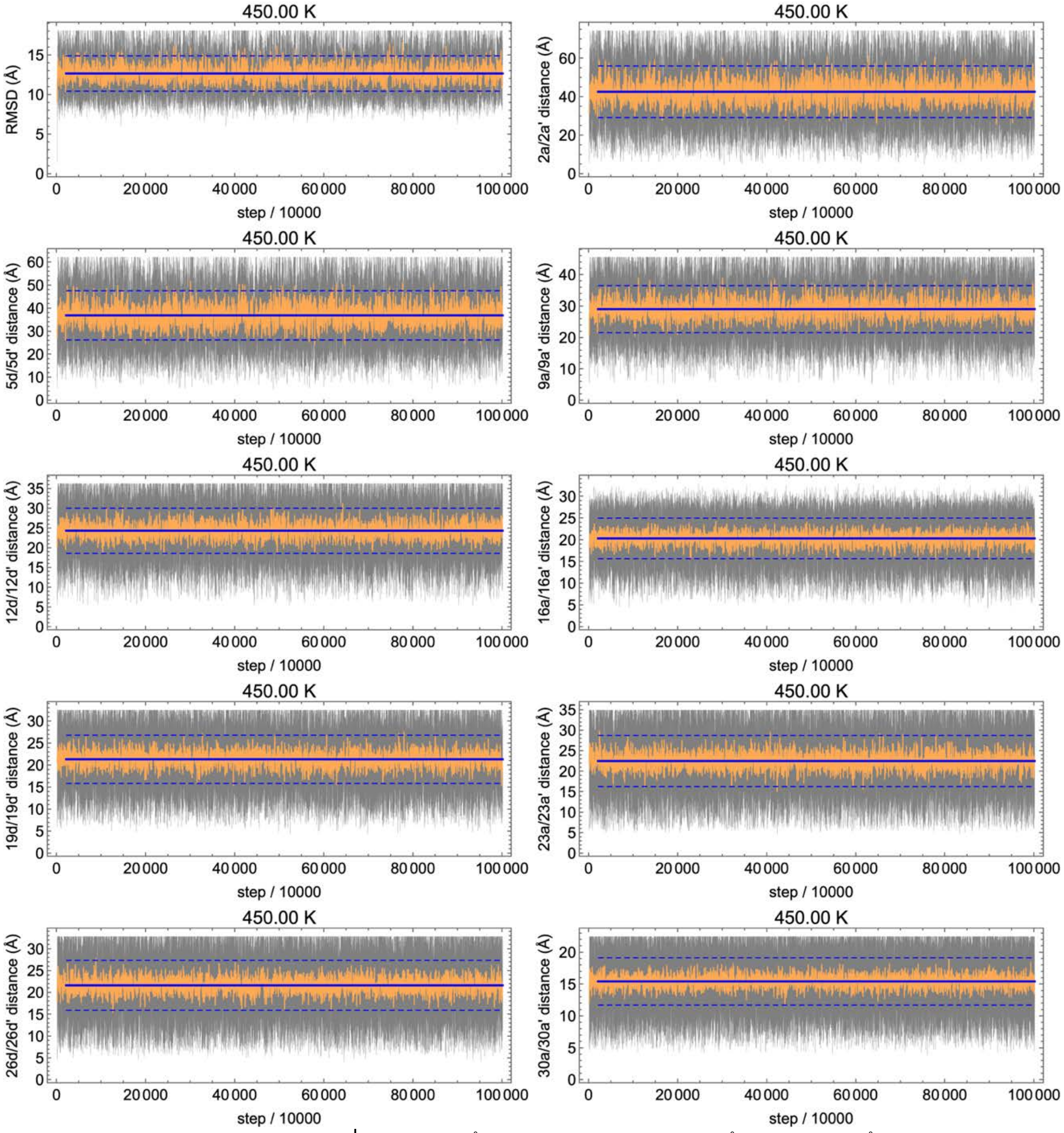

Measure at 450.00 K	Average (Å)	Standard Deviation (Å)	Slope (Å / μs)
RMSD	12.7347	2.2361	−0.0257275
5d/5d' distance	37.1311	10.6979	0.17503
12d/12d' distance	24.4741	5.717	−0.00511228
19d/19d' distance	21.4677	5.47387	−0.110004
26d/26d' distance	21.7716	5.72453	−0.0125698
2a/2a' distance	42.8781	13.4153	0.219871
9a/9a' distance	29.2268	7.48876	0.0796239
16a/16a' distance	20.4548	4.66606	−0.0832661
23a/23a' distance	22.6391	6.23079	−0.0931173
30a/30a' distance	15.521	3.70629	−0.00606158
Core Distance	26.1738	11.2139	

Figure S385. Plots of RMSD and residue packing distances for T-REMD simulations of variant **s14f/17b'-x4x** at 450.00 K.

(return to Table of Contents)

21f/24b'-xx

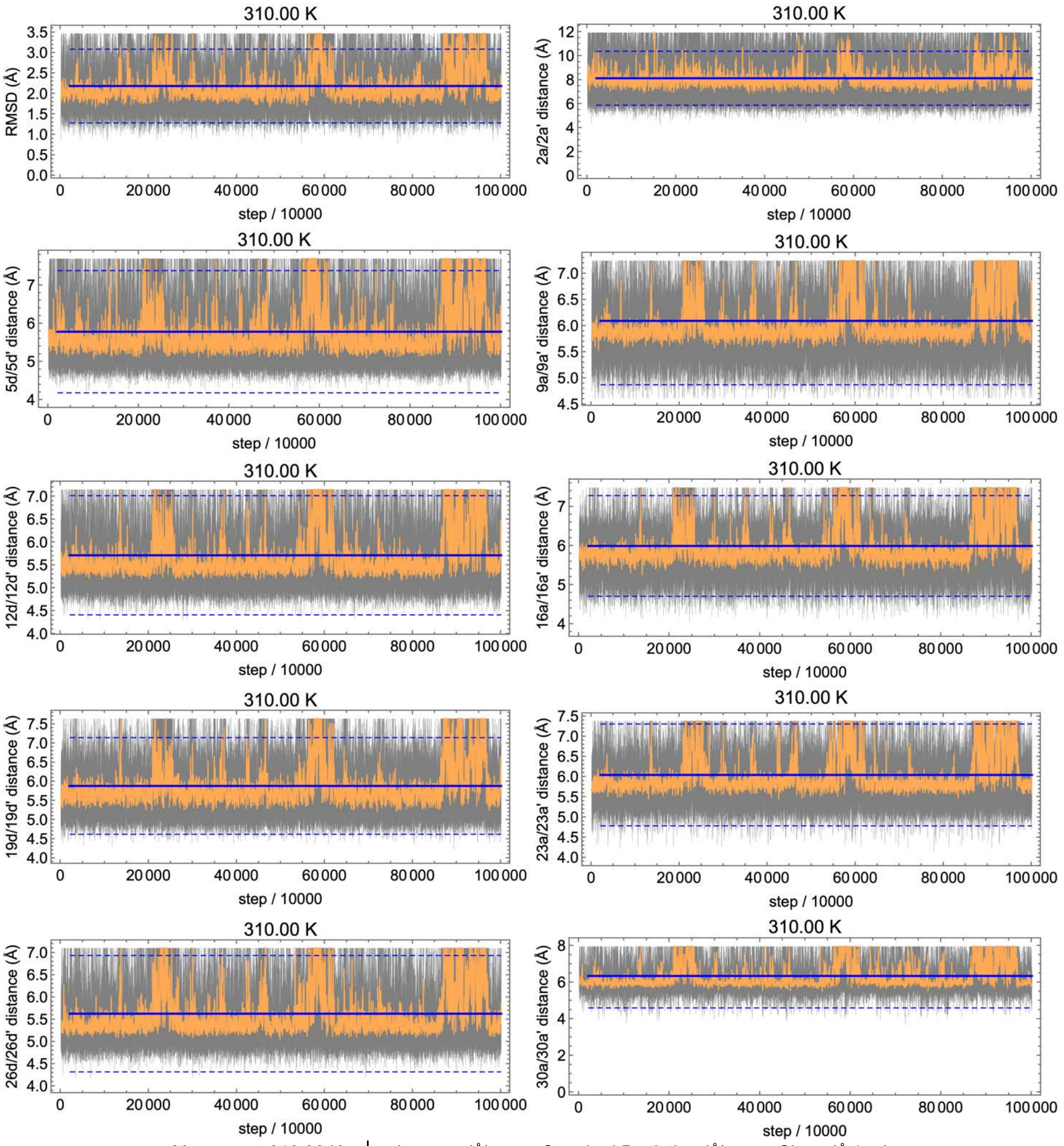

Measure at 310.00 K	Average (Å)	Standard Deviation (Å)	Slope (Å / μs)
RMSD	2.19565	0.902151	0.143509
5d/5d' distance	5.79302	1.59882	0.262387
12d/12d' distance	5.72304	1.30229	0.246172
19d/19d' distance	5.89305	1.26346	0.221579
26d/26d' distance	5.63587	1.30921	0.219833
2a/2a' distance	8.16689	2.25102	0.193505
9a/9a' distance	6.10167	1.22272	0.235085
16a/16a' distance	6.00313	1.28615	0.244434
23a/23a' distance	6.05585	1.26138	0.197728
30a/30a' distance	6.36487	1.74327	0.297235
Core Distance	6.19304	1.67262	

Figure S386. Plots of RMSD and residue packing distances for T-REMD simulations of variant **21f/24b'-xx** at 310.00 K.

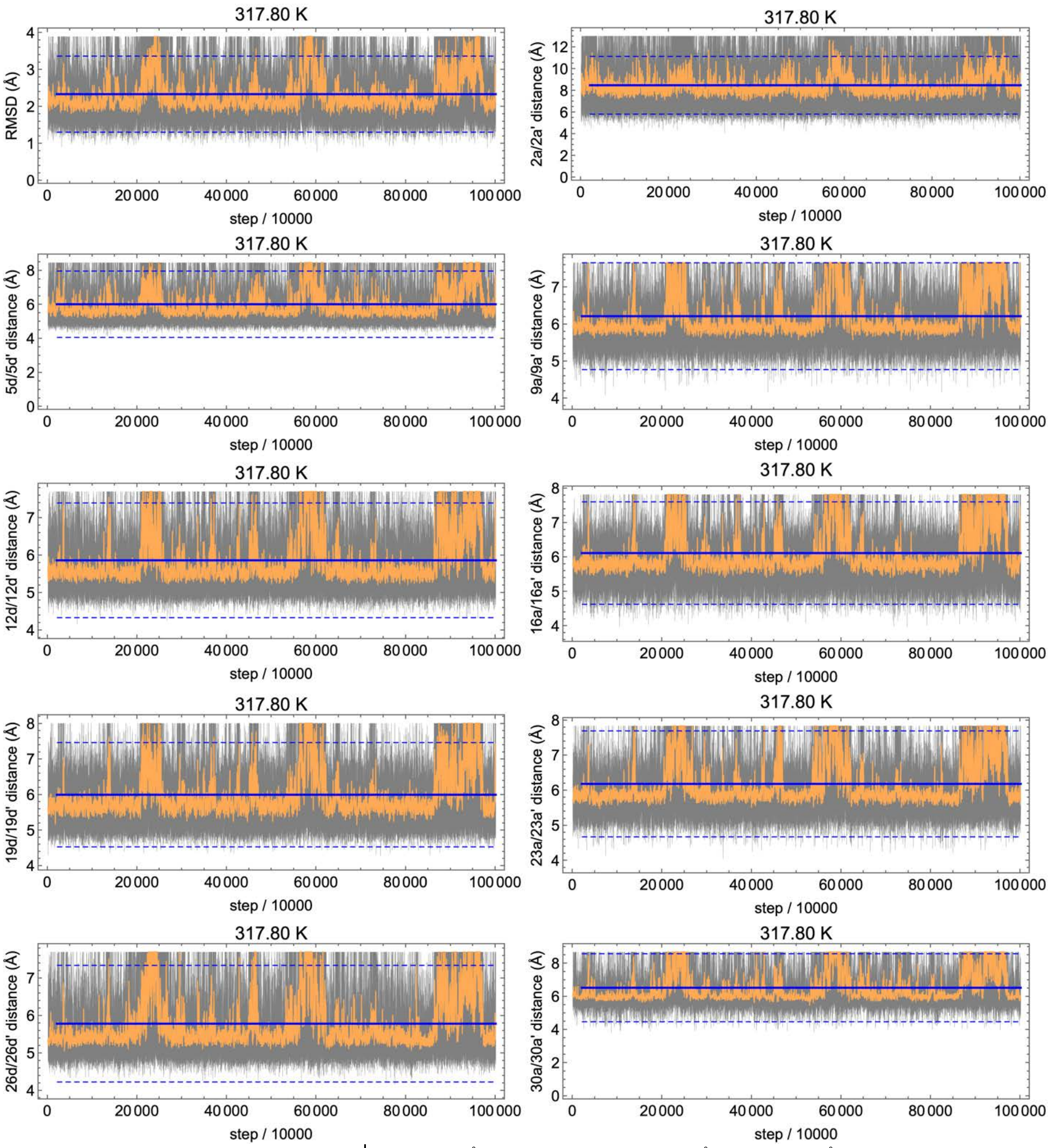

Measure at 317.80 K	Average (Å)	Standard Deviation (Å)	Slope (Å / μs)
RMSD	2.34866	1.02963	0.14763
5d/5d' distance	6.0436	1.95093	0.299572
12d/12d' distance	5.87686	1.52995	0.273063
19d/19d' distance	6.01214	1.46676	0.255902
26d/26d' distance	5.79675	1.55842	0.222124
2a/2a' distance	8.52997	2.65988	0.23751
9a/9a' distance	6.22892	1.44182	0.263632
16a/16a' distance	6.13123	1.48881	0.270909
23a/23a' distance	6.19782	1.51087	0.219998
30a/30a' distance	6.55488	2.05203	0.312141
Core Distance	6.37469	1.94944	

Figure S387. Plots of RMSD and residue packing distances for T-REMD simulations of variant **21f/24b'-xx** at 317.80 K.

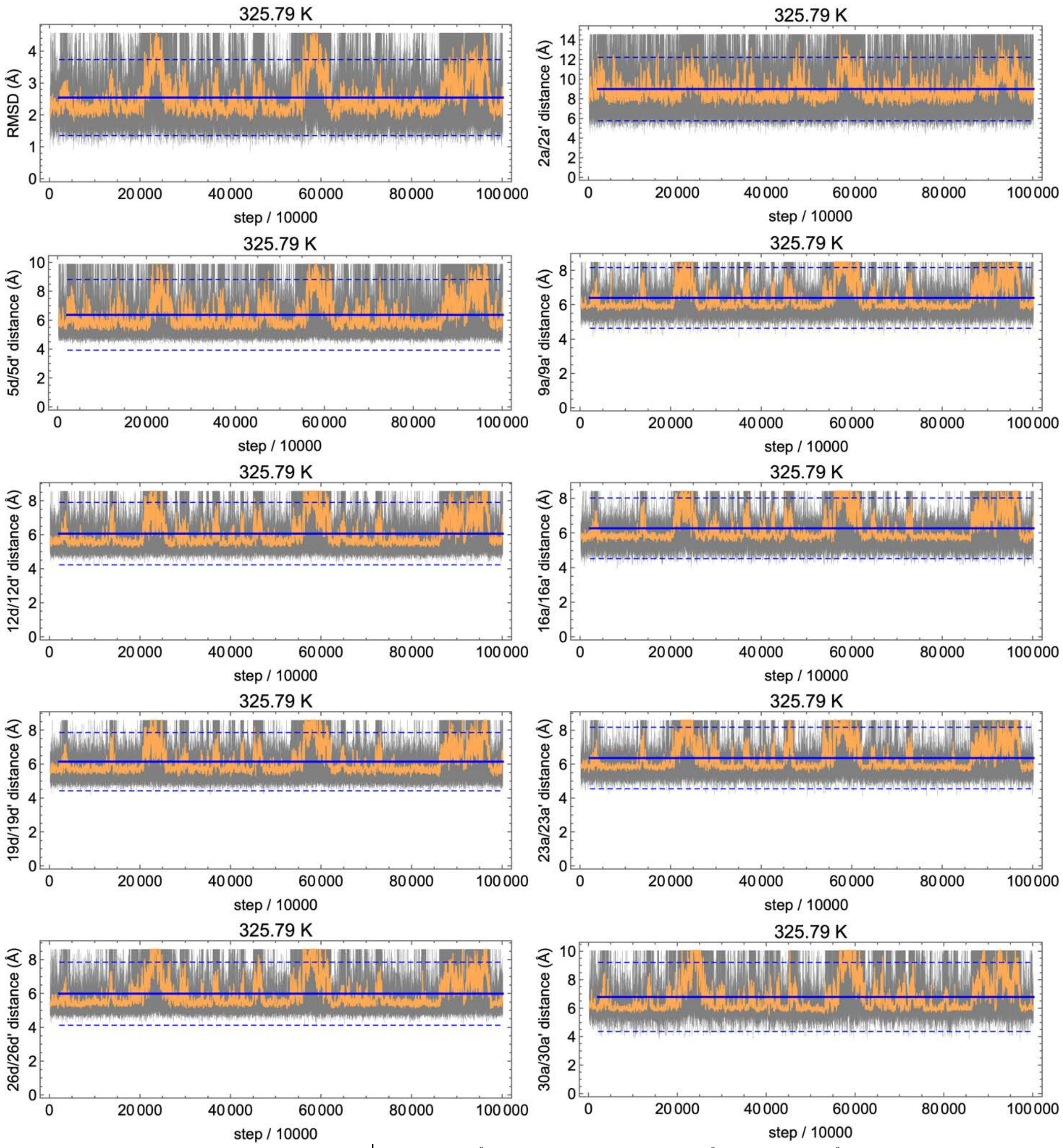

Measure at 325.79 K	Average (Å)	Standard Deviation (Å)	Slope (Å / μs)
RMSD	2.56531	1.19335	0.139313
5d/5d' distance	6.41773	2.44464	0.328499
12d/12d' distance	6.10306	1.83591	0.297327
19d/19d' distance	6.17993	1.71698	0.259241
26d/26d' distance	6.02773	1.86415	0.196727
2a/2a' distance	9.05539	3.23455	0.245012
9a/9a' distance	6.4351	1.76753	0.283012
16a/16a' distance	6.31154	1.75218	0.280605
23a/23a' distance	6.39893	1.81625	0.203395
30a/30a' distance	6.83347	2.42727	0.285363
Core Distance	6.64032	2.32428	

Figure S388. Plots of RMSD and residue packing distances for T-REMD simulations of variant **21f/24b'-xx** at 325.79 K.

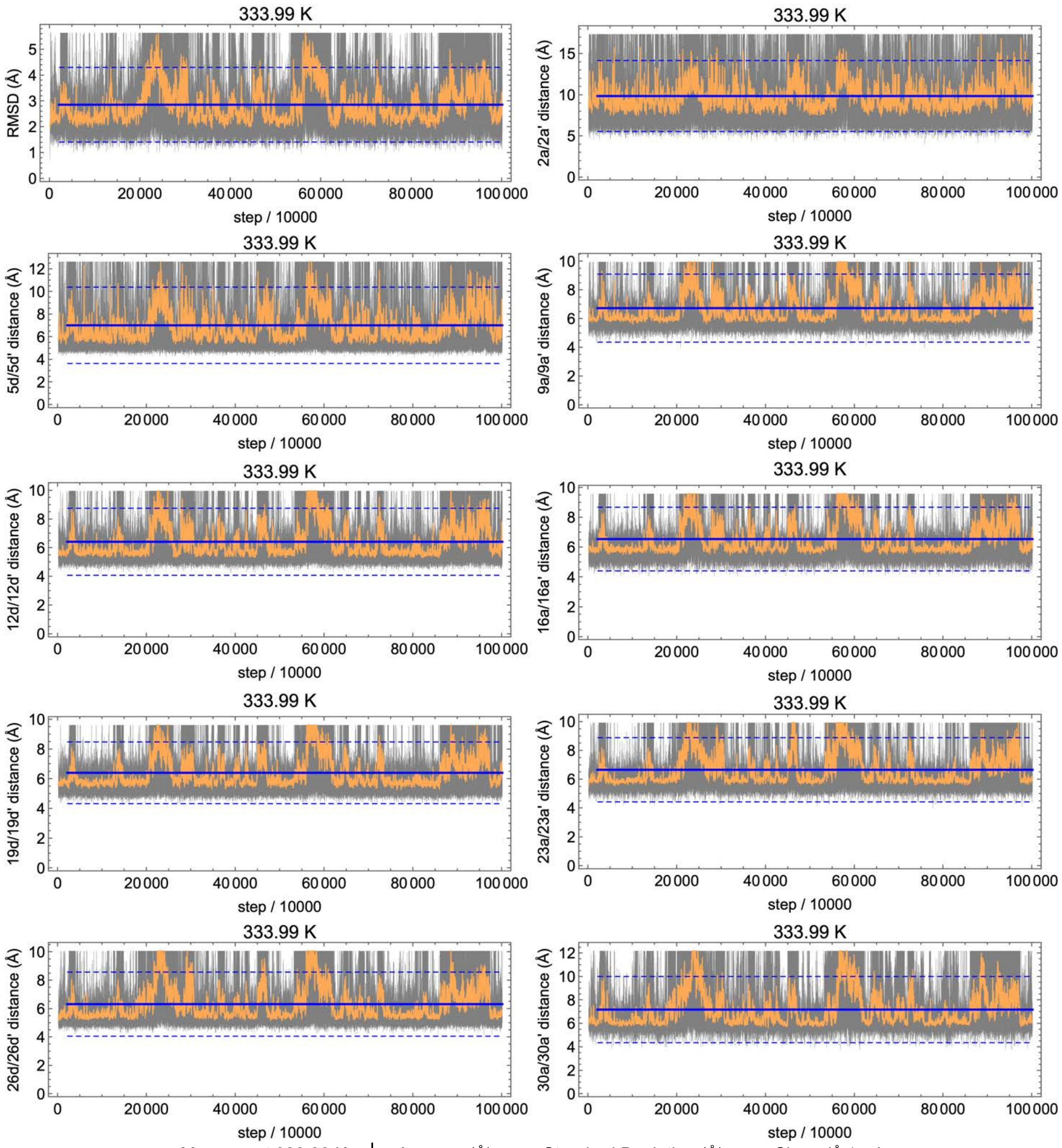

Measure at 333.99 K	Average (Å)	Standard Deviation (Å)	Slope (Å / μs)
RMSD	2.87784	1.44529	0.092238
5d/5d' distance	7.06495	3.37676	0.297874
12d/12d' distance	6.45493	2.33505	0.2483
19d/19d' distance	6.44081	2.07696	0.213684
26d/26d' distance	6.3577	2.2571	0.109615
2a/2a' distance	9.91623	4.3089	0.223333
9a/9a' distance	6.77331	2.36842	0.232007
16a/16a' distance	6.5824	2.13321	0.225054
23a/23a' distance	6.69571	2.2285	0.129649
30a/30a' distance	7.22879	2.82294	0.177772
Core Distance	7.0572	2.94008	

Figure S389. Plots of RMSD and residue packing distances for T-REMD simulations of variant **21f/24b'-xx** at 333.99 K.

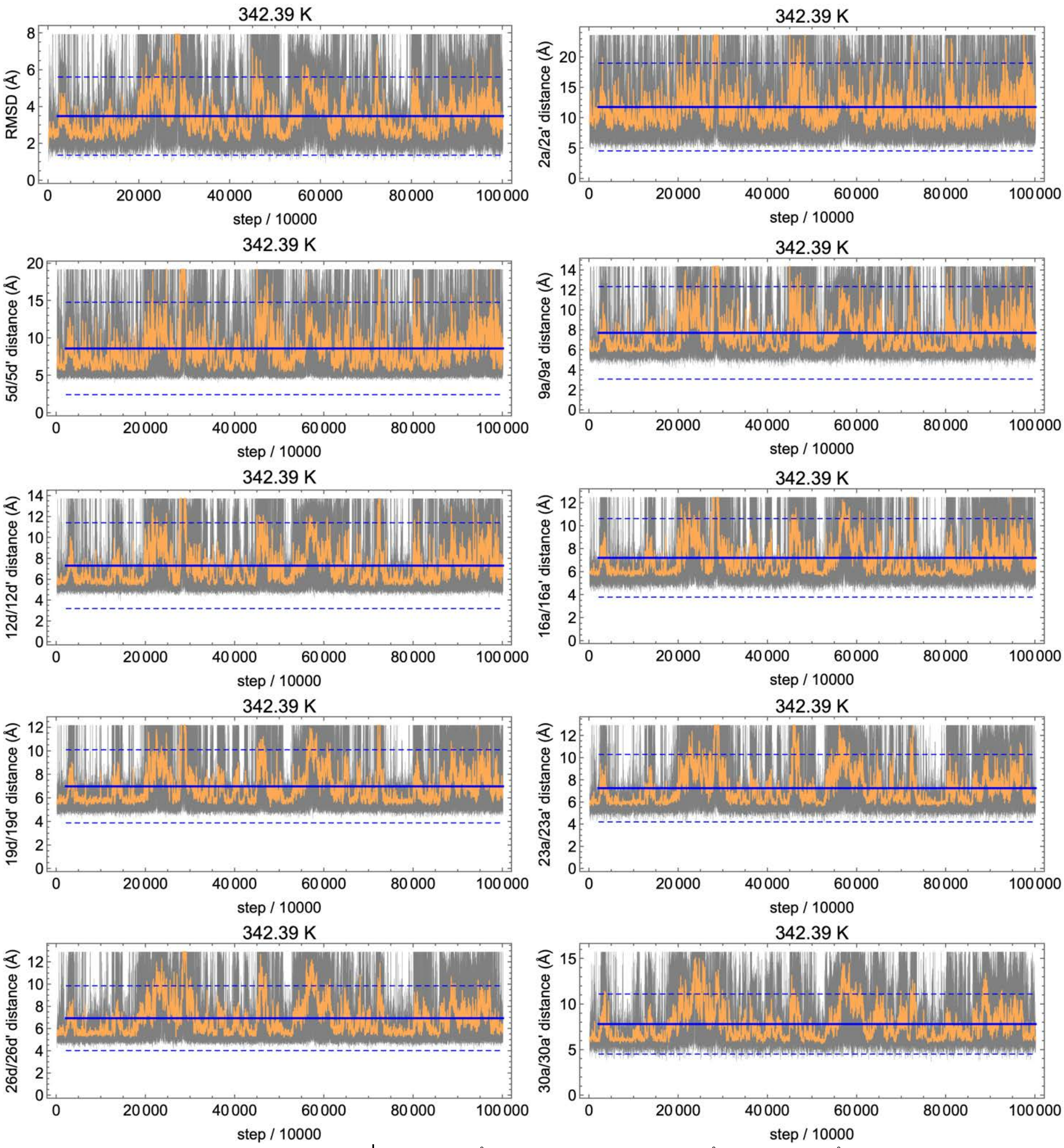

Measure at 342.39 K	Average (Å)	Standard Deviation (Å)	Slope (Å / μs)
RMSD	3.51703	2.12353	0.0699262
5d/5d' distance	8.68014	6.18048	0.330838
12d/12d' distance	7.35949	4.10873	0.22622
19d/19d' distance	7.03704	3.10729	0.179493
26d/26d' distance	6.98795	2.91732	0.0439705
2a/2a' distance	11.8687	7.22333	0.326212
9a/9a' distance	7.76789	4.62826	0.216753
16a/16a' distance	7.25646	3.41784	0.16369
23a/23a' distance	7.3062	3.04318	0.0695162
30a/30a' distance	7.87917	3.2958	0.0860507
Core Distance	8.0159	4.68494	

Figure S390. Plots of RMSD and residue packing distances for T-REMD simulations of variant **21f/24b'-xx** at 342.39 K.

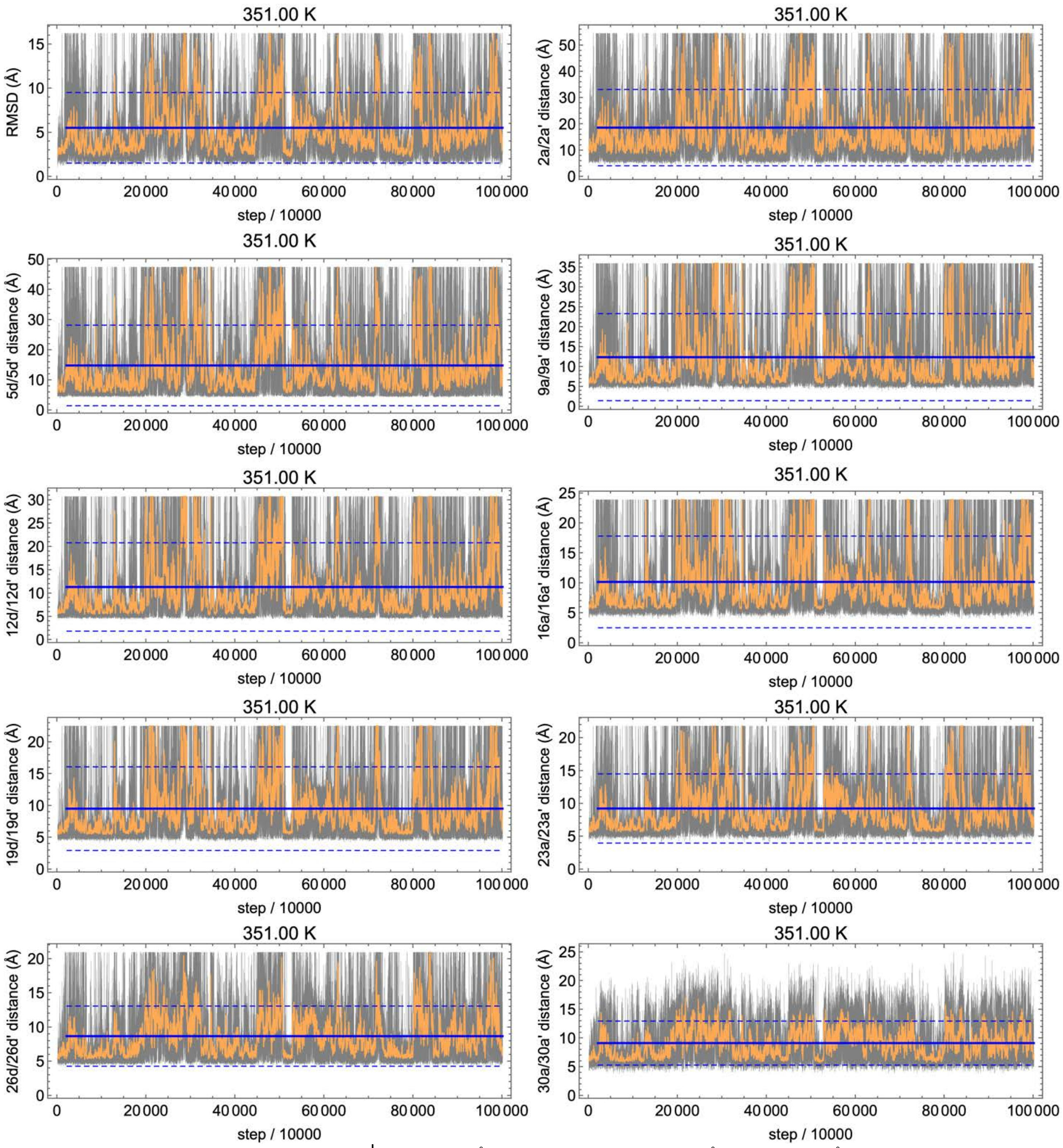

Measure at 351.00 K	Average (Å)	Standard Deviation (Å)	Slope (Å / μs)
RMSD	5.57384	3.99812	0.643028
5d/5d' distance	14.9912	13.3546	1.897
12d/12d' distance	11.4262	9.48689	1.41903
19d/19d' distance	9.59596	6.57869	1.06453
26d/26d' distance	8.75472	4.40086	0.637599
2a/2a' distance	18.7929	14.5322	1.83108
9a/9a' distance	12.5078	10.9244	1.58691
16a/16a' distance	10.2521	7.65325	1.11235
23a/23a' distance	9.31753	5.26984	0.809943
30a/30a' distance	9.21739	3.83525	0.466502
Core Distance	11.6506	9.72654	

Figure S391. Plots of RMSD and residue packing distances for T-REMD simulations of variant **21f/24b'-xx** at 351.00 K.

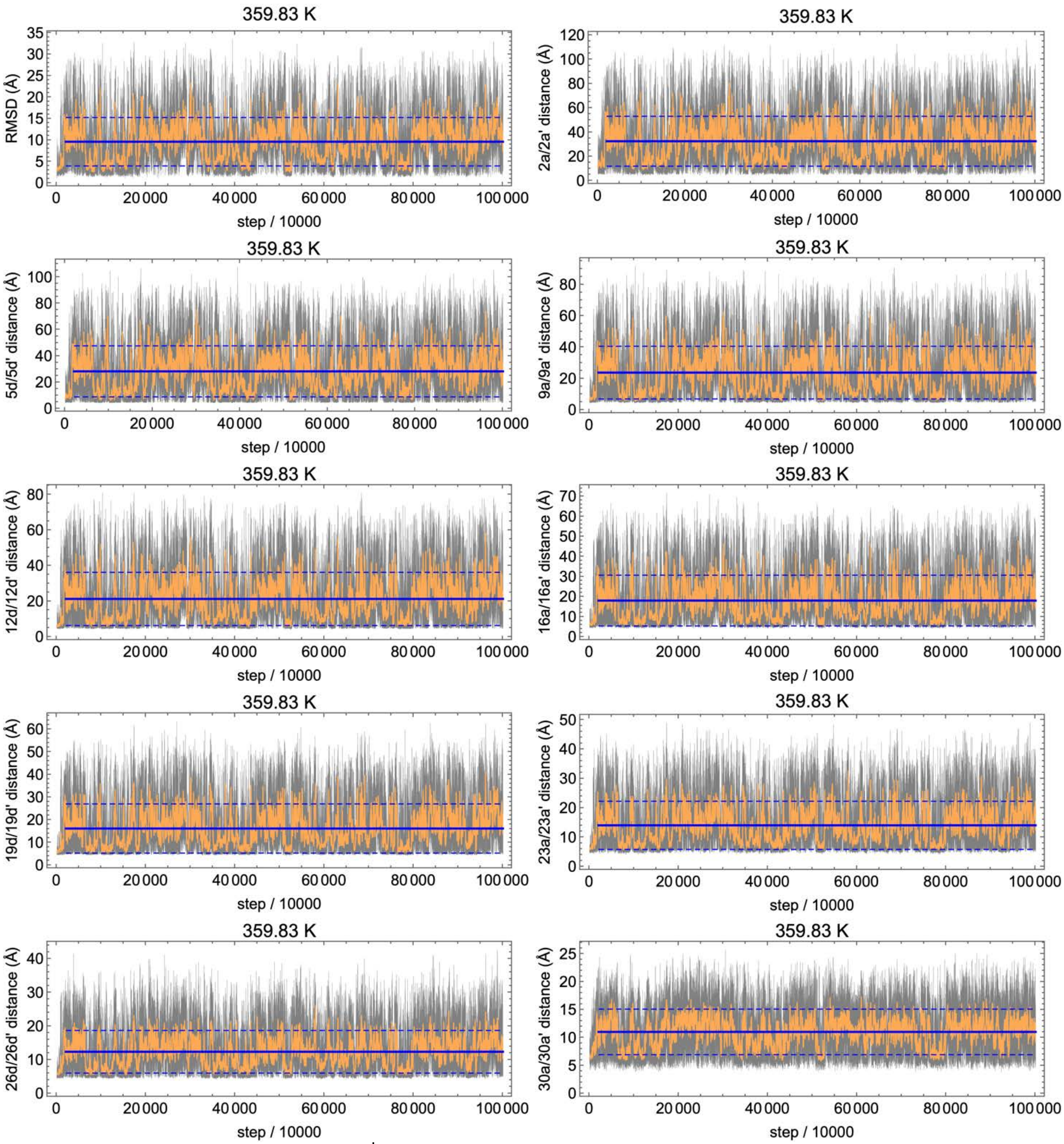

Measure at 359.83 K	Average (Å)	Standard Deviation (Å)	Slope (Å / μs)
RMSD	9.70028	5.64462	0.949598
5d/5d' distance	28.5146	19.4107	2.76045
12d/12d' distance	21.4785	14.9613	2.37015
19d/19d' distance	16.3139	10.8559	1.72732
26d/26d' distance	12.4882	6.33303	0.868967
2a/2a' distance	32.8461	20.5522	2.64525
9a/9a' distance	24.0134	16.8128	2.55163
16a/16a' distance	18.2162	12.6452	1.95305
23a/23a' distance	14.1748	8.20088	1.26503
30a/30a' distance	11.086	4.08031	0.453713
Core Distance	19.9035	15.4551	

Figure S392. Plots of RMSD and residue packing distances for T-REMD simulations of variant **21f/24b'-xx** at 359.83 K.

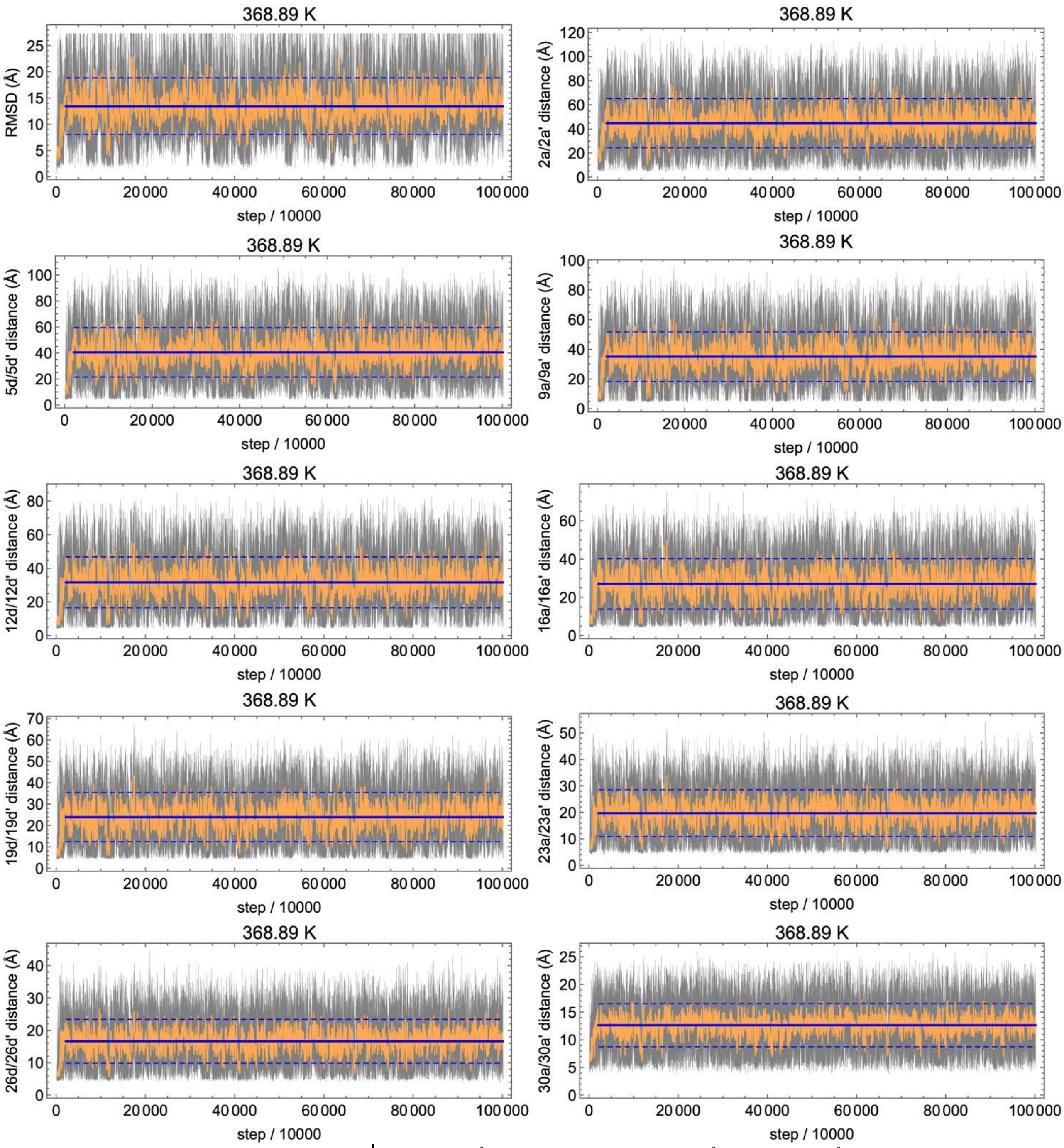

Measure at 368.89 K	Average (Å)	Standard Deviation (Å)	Slope (Å / μs)
RMSD	13.578	5.40031	0.39329
5d/5d' distance	40.9963	19.0224	1.19467
12d/12d' distance	32.0111	15.134	1.0945
19d/19d' distance	24.2354	11.4782	0.880868
26d/26d' distance	16.8269	6.76422	0.447598
2a/2a' distance	45.3338	20.3958	1.10551
9a/9a' distance	35.4874	16.6884	1.13447
16a/16a' distance	27.3445	13.2048	0.923981
23a/23a' distance	19.9477	8.83193	0.616223
30a/30a' distance	12.7693	3.89155	0.22494
Core Distance	28.328	17.3435	

Figure S393. Plots of RMSD and residue packing distances for T-REMD simulations of variant **21f/24b'-xx** at 368.89 K.

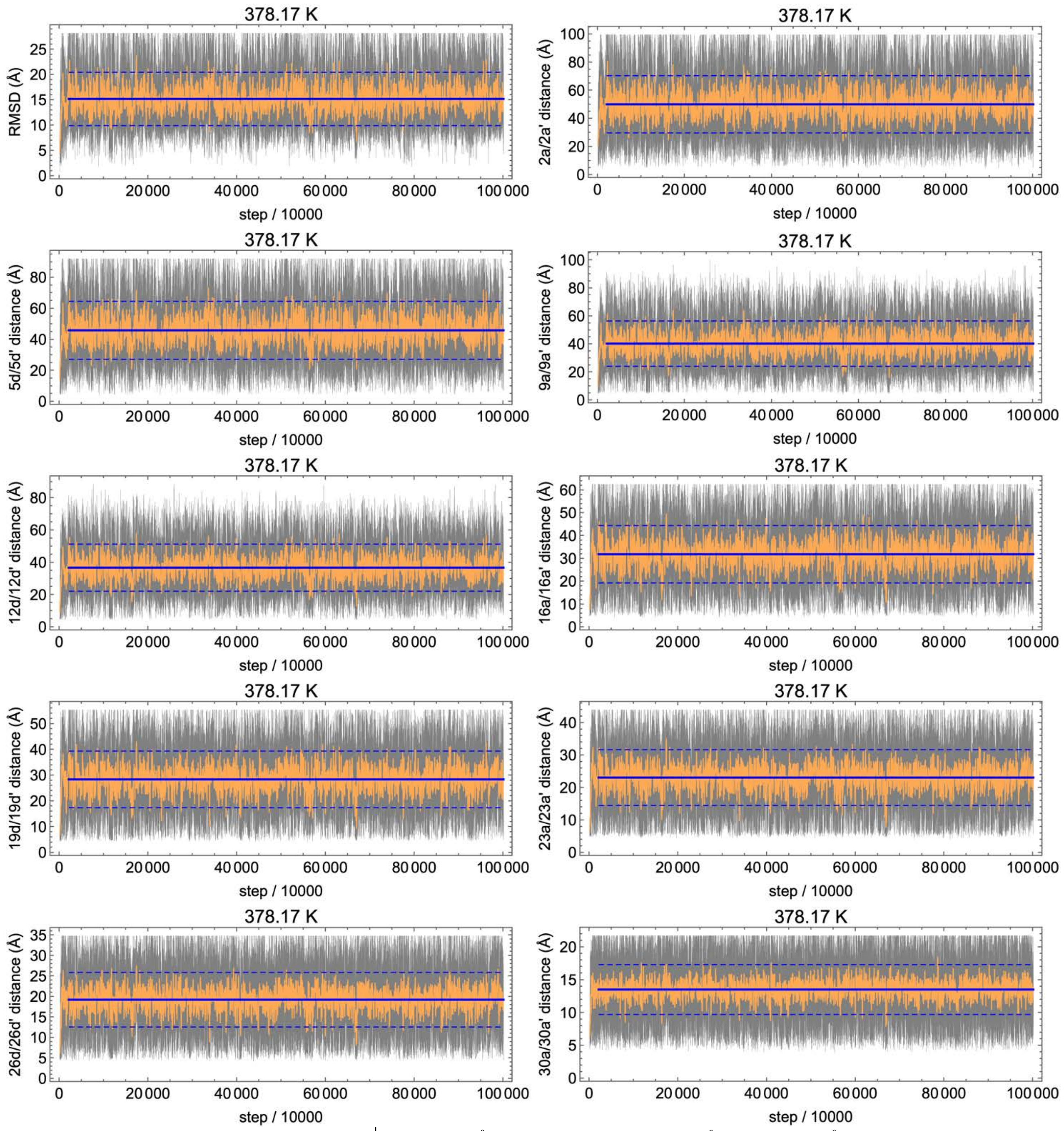

Measure at 378.17 K	Average (Å)	Standard Deviation (Å)	Slope (Å / μs)
RMSD	15.2967	5.26872	0.137376
5d/5d' distance	46.1972	18.7182	0.373358
12d/12d' distance	37.0473	14.5878	0.270088
19d/19d' distance	28.5718	10.9875	0.195813
26d/26d' distance	19.3526	6.65468	0.122251
2a/2a' distance	50.42	20.3547	0.341363
9a/9a' distance	40.6353	16.1702	0.339922
16a/16a' distance	32.0834	12.5804	0.166341
23a/23a' distance	23.2432	8.59374	0.102323
30a/30a' distance	13.5922	3.79713	0.016913
Core Distance	32.3492	17.8809	

Figure S394. Plots of RMSD and residue packing distances for T-REMD simulations of variant **21f/24b'-xx** at 378.17 K.

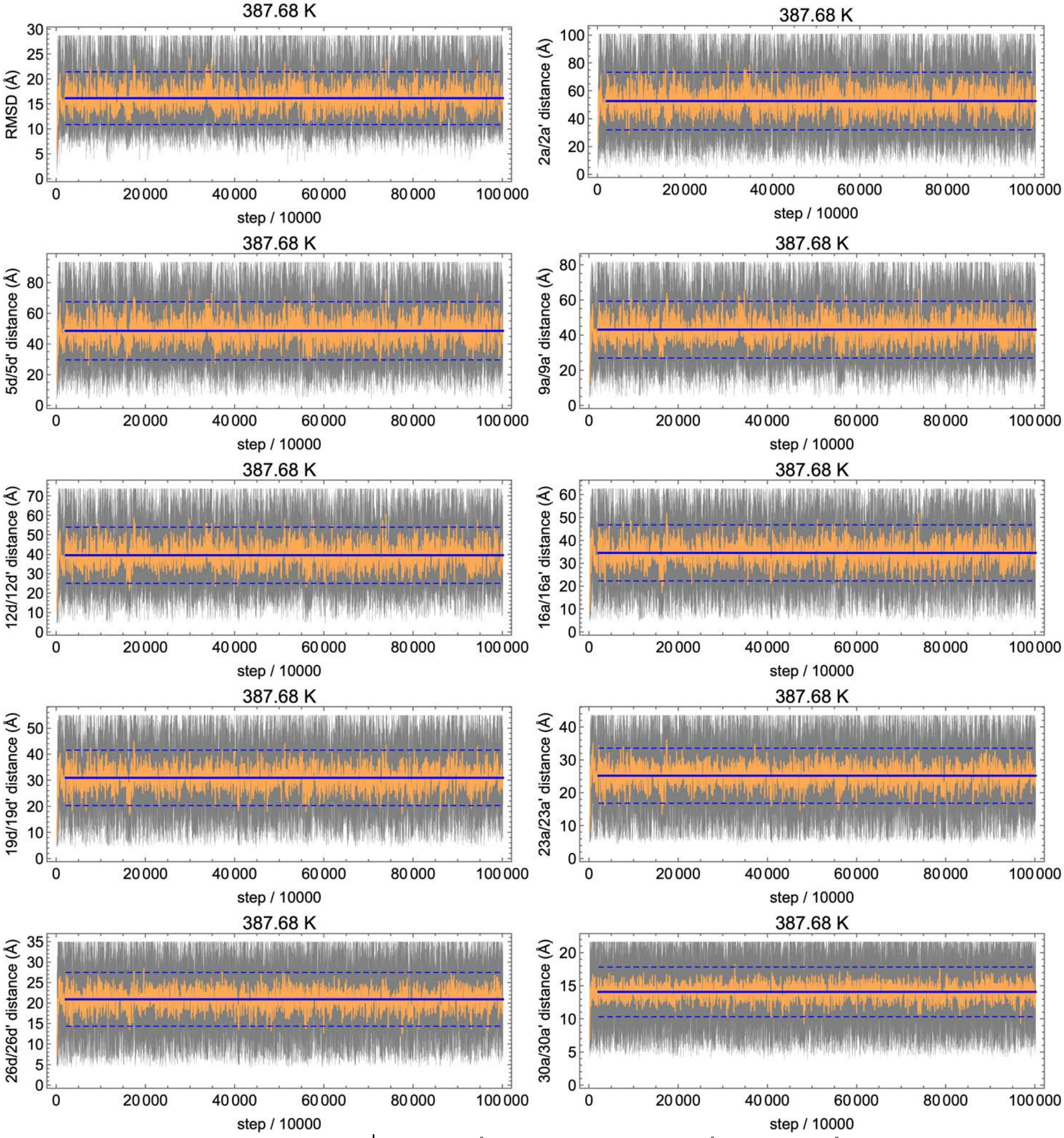

Measure at 387.68 K	Average (Å)	Standard Deviation (Å)	Slope (Å / μs)
RMSD	16.289	5.28962	0.0896851
5d/5d' distance	49.0078	18.9381	0.294986
12d/12d' distance	39.8297	14.4672	0.0903142
19d/19d' distance	31.186	10.622	0.0556952
26d/26d' distance	21.0809	6.55809	0.0422009
2a/2a' distance	53.1483	20.6883	0.275429
9a/9a' distance	43.4513	16.1886	0.140422
16a/16a' distance	34.8178	12.2372	0.0318042
23a/23a' distance	25.377	8.37201	−0.00326843
30a/30a' distance	14.1778	3.75348	0.0165476
Core Distance	34.6752	18.2624	

Figure S395. Plots of RMSD and residue packing distances for T-REMD simulations of variant **21f/24b'-xx** at 387.68 K.

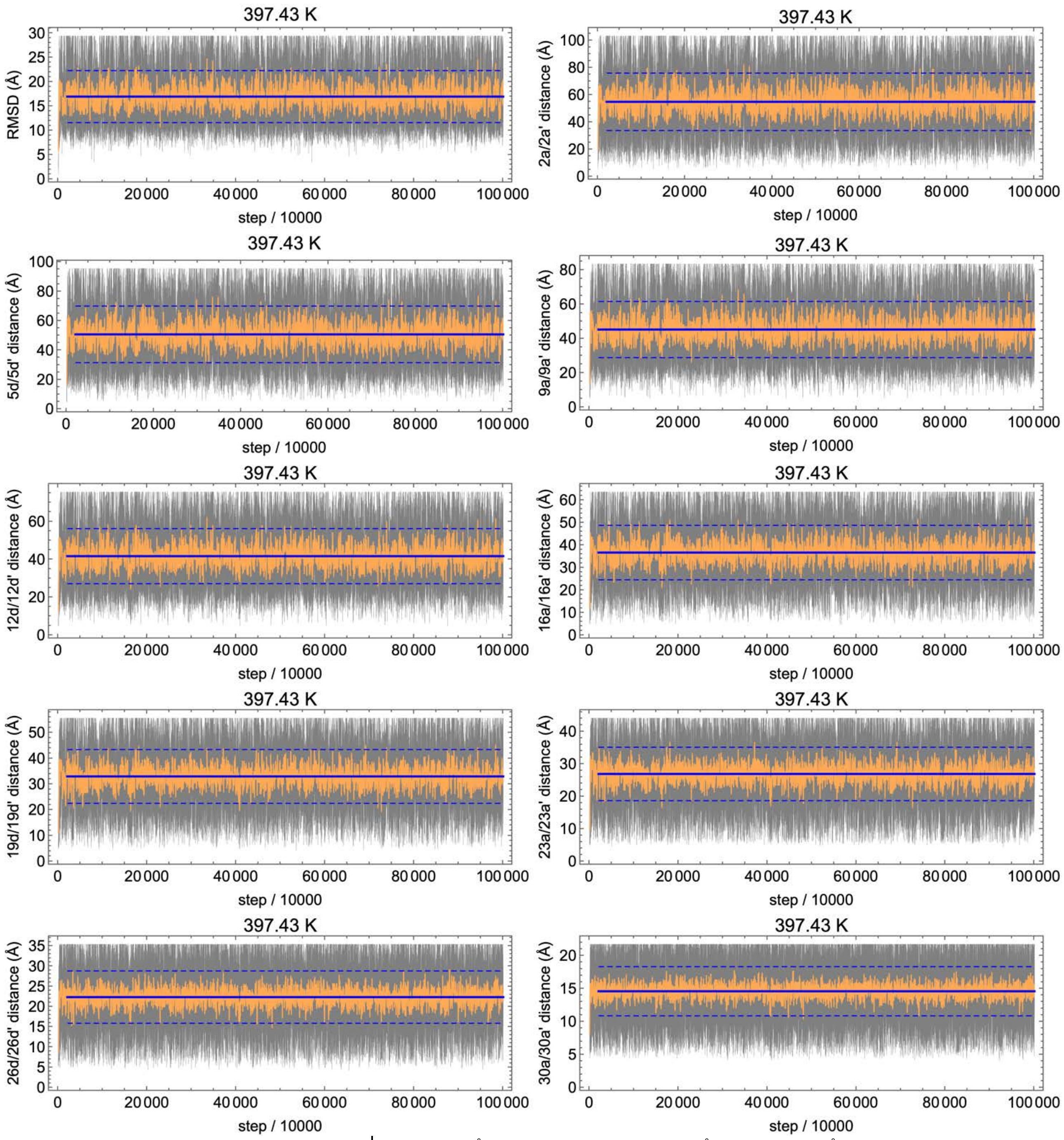

Measure at 397.43 K	Average (Å)	Standard Deviation (Å)	Slope (Å / μs)
RMSD	17.0294	5.34377	0.0578129
5d/5d' distance	50.9877	19.2463	0.231915
12d/12d' distance	41.8724	14.5258	−0.0109424
19d/19d' distance	33.1143	10.4471	−0.00355067
26d/26d' distance	22.4384	6.47109	0.0278857
2a/2a' distance	55.1154	21.1205	0.196313
9a/9a' distance	45.4386	16.3782	0.106801
16a/16a' distance	36.8247	12.0988	−0.0209901
23a/23a' distance	27.0283	8.23425	−0.0331572
30a/30a' distance	14.6533	3.72616	−0.000354399
Core Distance	36.3859	18.6075	

Figure S396. Plots of RMSD and residue packing distances for T-REMD simulations of variant **21f/24b'-xx** at 397.43 K.

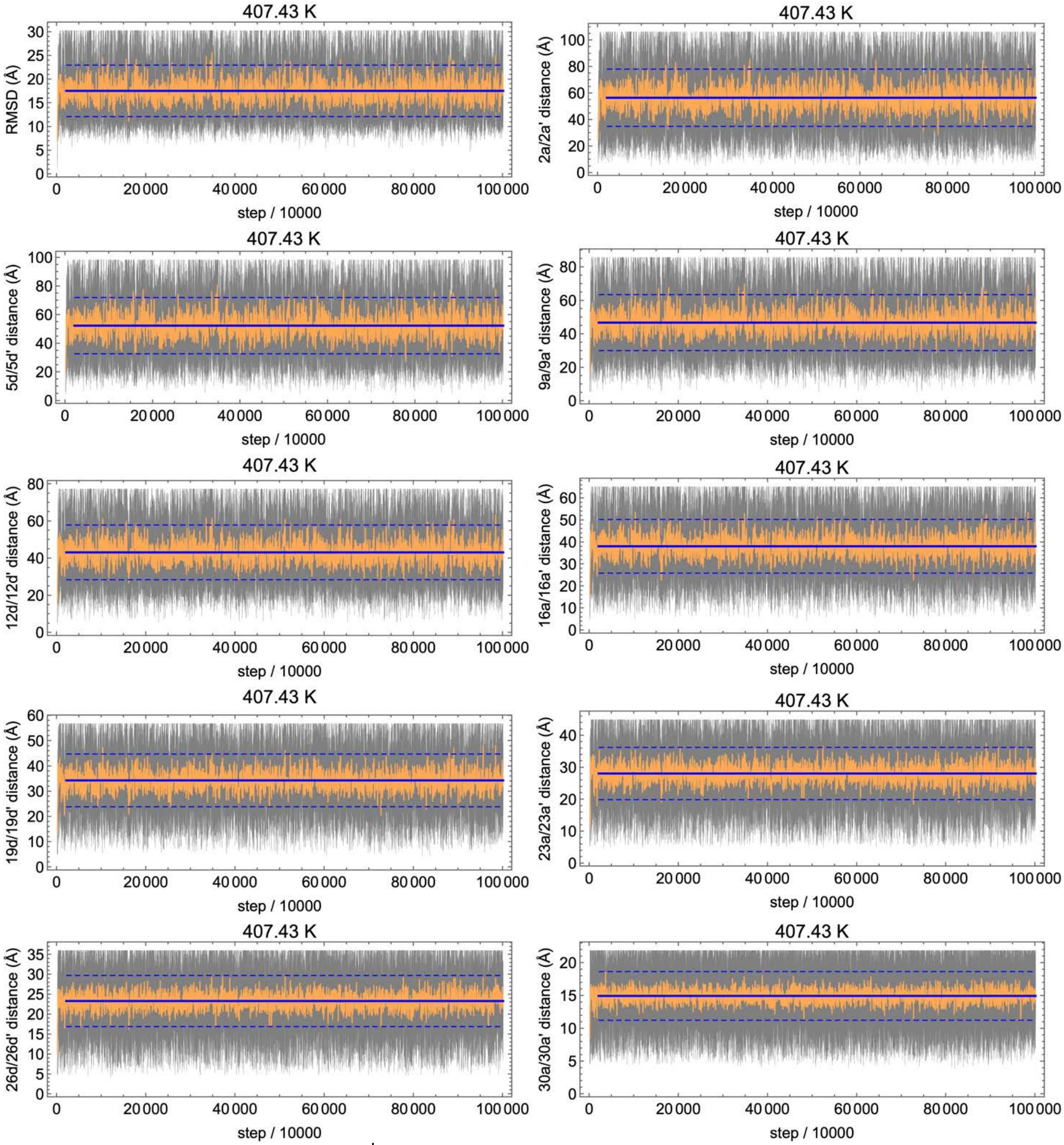

Measure at 407.43 K	Average (Å)	Standard Deviation (Å)	Slope (Å / μs)
RMSD	17.6574	5.43494	−0.0409056
5d/5d' distance	52.7064	19.6611	−0.185788
12d/12d' distance	43.4121	14.7351	−0.198572
19d/19d' distance	34.5205	10.4393	−0.106975
26d/26d' distance	23.4553	6.42018	0.00772004
2a/2a' distance	56.8811	21.6026	−0.211527
9a/9a' distance	47.0672	16.7029	−0.173657
16a/16a' distance	38.3038	12.1905	−0.188024
23a/23a' distance	28.2484	8.18157	−0.125747
30a/30a' distance	15.0364	3.71024	−0.0200334
Core Distance	37.7368	19.0282	

Figure S397. Plots of RMSD and residue packing distances for T-REMD simulations of variant **21f/24b'-xx** at 407.43 K.

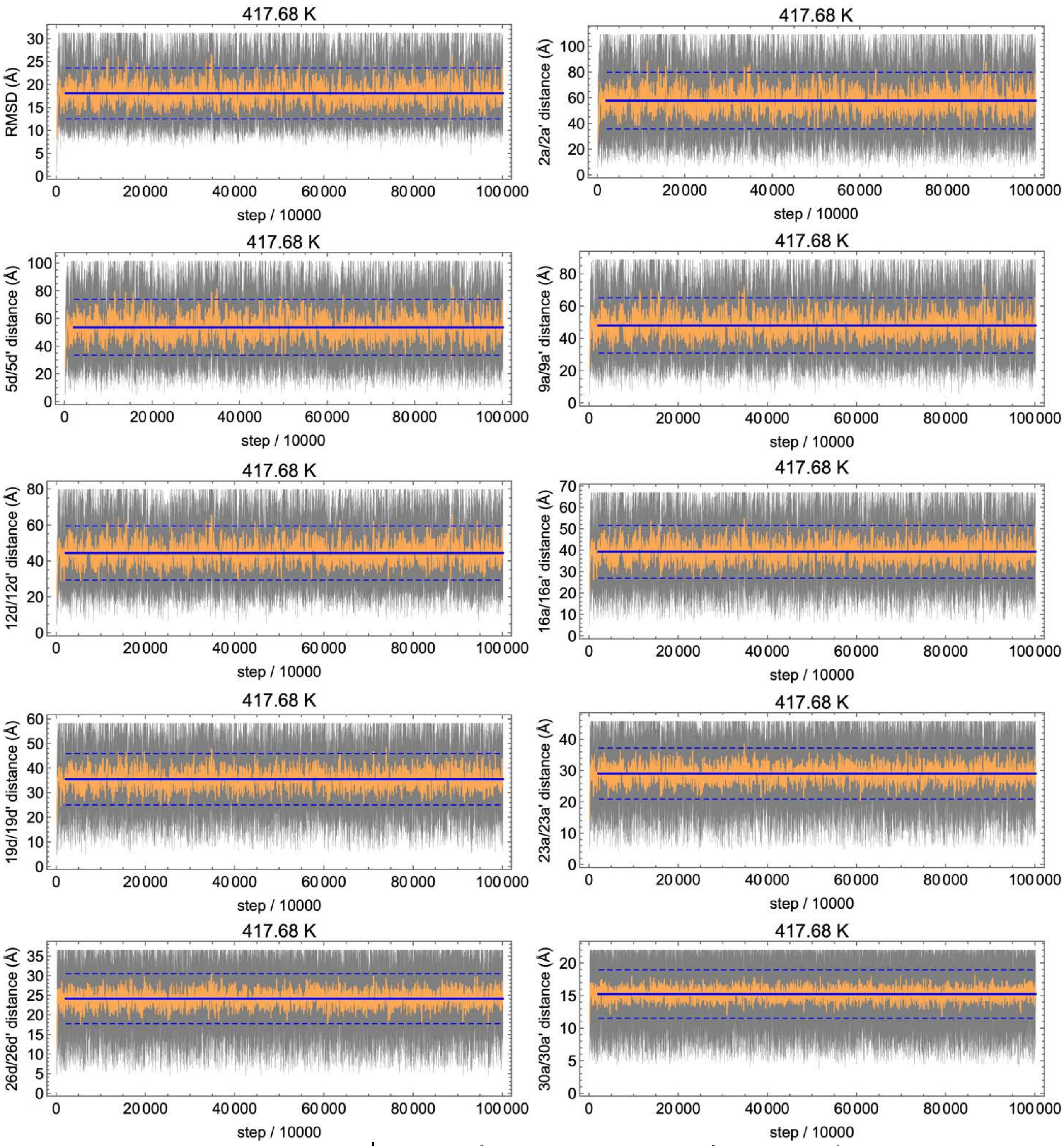

Measure at 417.68 K	Average (Å)	Standard Deviation (Å)	Slope (Å / μs)
RMSD	18.2018	5.54891	−0.0177028
5d/5d' distance	54.073	20.1122	−0.0522491
12d/12d' distance	44.7475	15.0541	−0.118645
19d/19d' distance	35.7104	10.4625	−0.0329528
26d/26d' distance	24.3132	6.36147	0.118515
2a/2a' distance	58.2729	22.1252	−0.145262
9a/9a' distance	48.4186	17.0996	−0.0945189
16a/16a' distance	39.5614	12.318	−0.0796883
23a/23a' distance	29.3048	8.1589	0.0255514
30a/30a' distance	15.3423	3.7003	0.0248604
Core Distance	38.8605	19.4242	

Figure S398. Plots of RMSD and residue packing distances for T-REMD simulations of variant **21f/24b'-xx** at 417.68 K.

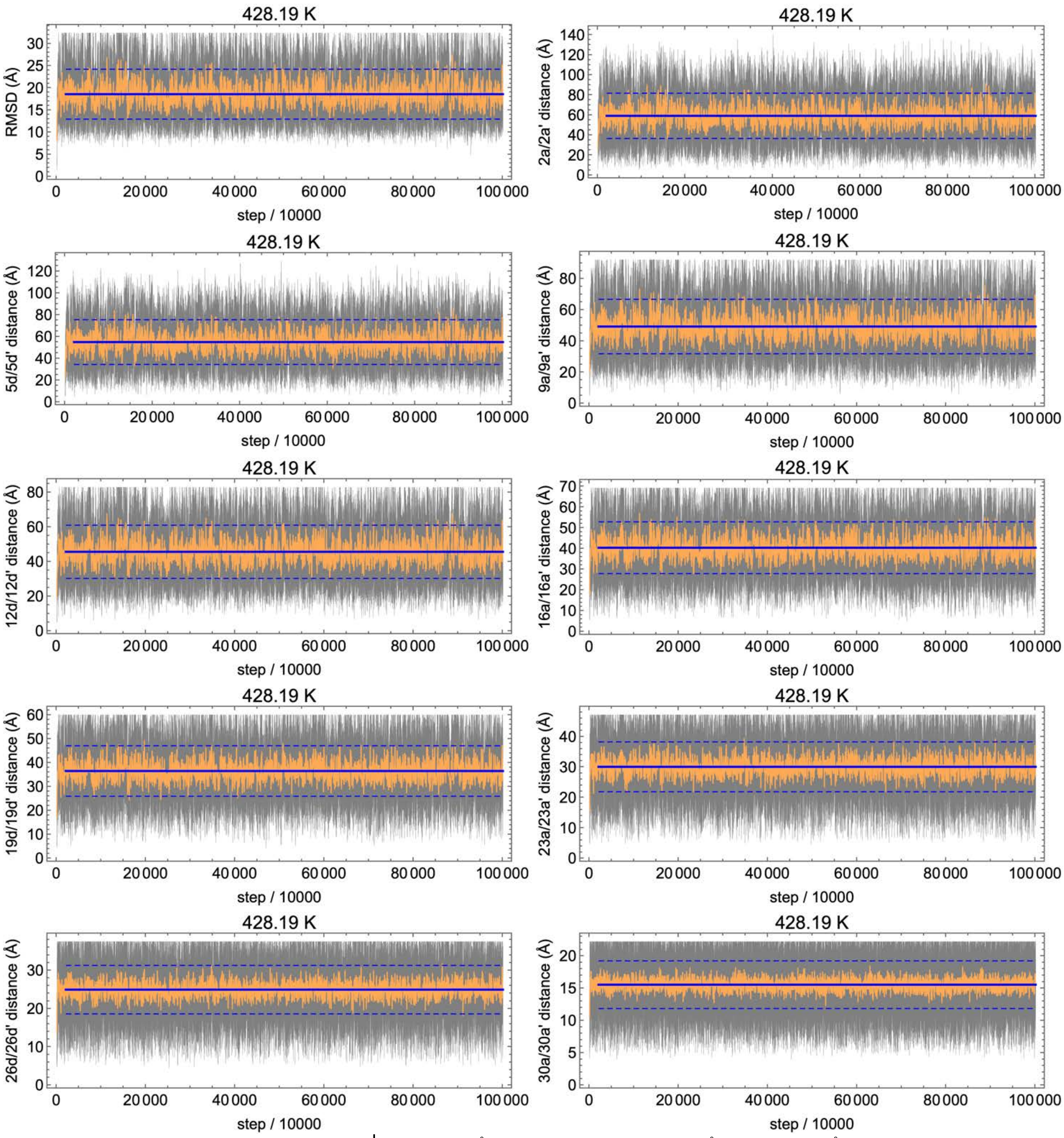

Measure at 428.19 K	Average (Å)	Standard Deviation (Å)	Slope (Å / μs)
RMSD	18.6767	5.62988	−0.0315204
5d/5d' distance	55.3192	20.5301	−0.152339
12d/12d' distance	45.8592	15.3579	−0.0943269
19d/19d' distance	36.7017	10.5654	−0.0275617
26d/26d' distance	25.0657	6.33848	0.0967606
2a/2a' distance	59.5763	22.5762	−0.206037
9a/9a' distance	49.5711	17.4787	−0.093413
16a/16a' distance	40.6044	12.4854	−0.0519472
23a/23a' distance	30.211	8.19302	−0.00469262
30a/30a' distance	15.6218	3.68792	−0.0444528
Core Distance	39.8367	19.8016	

Figure S399. Plots of RMSD and residue packing distances for T-REMD simulations of variant **21f/24b'-xx** at 428.19 K.

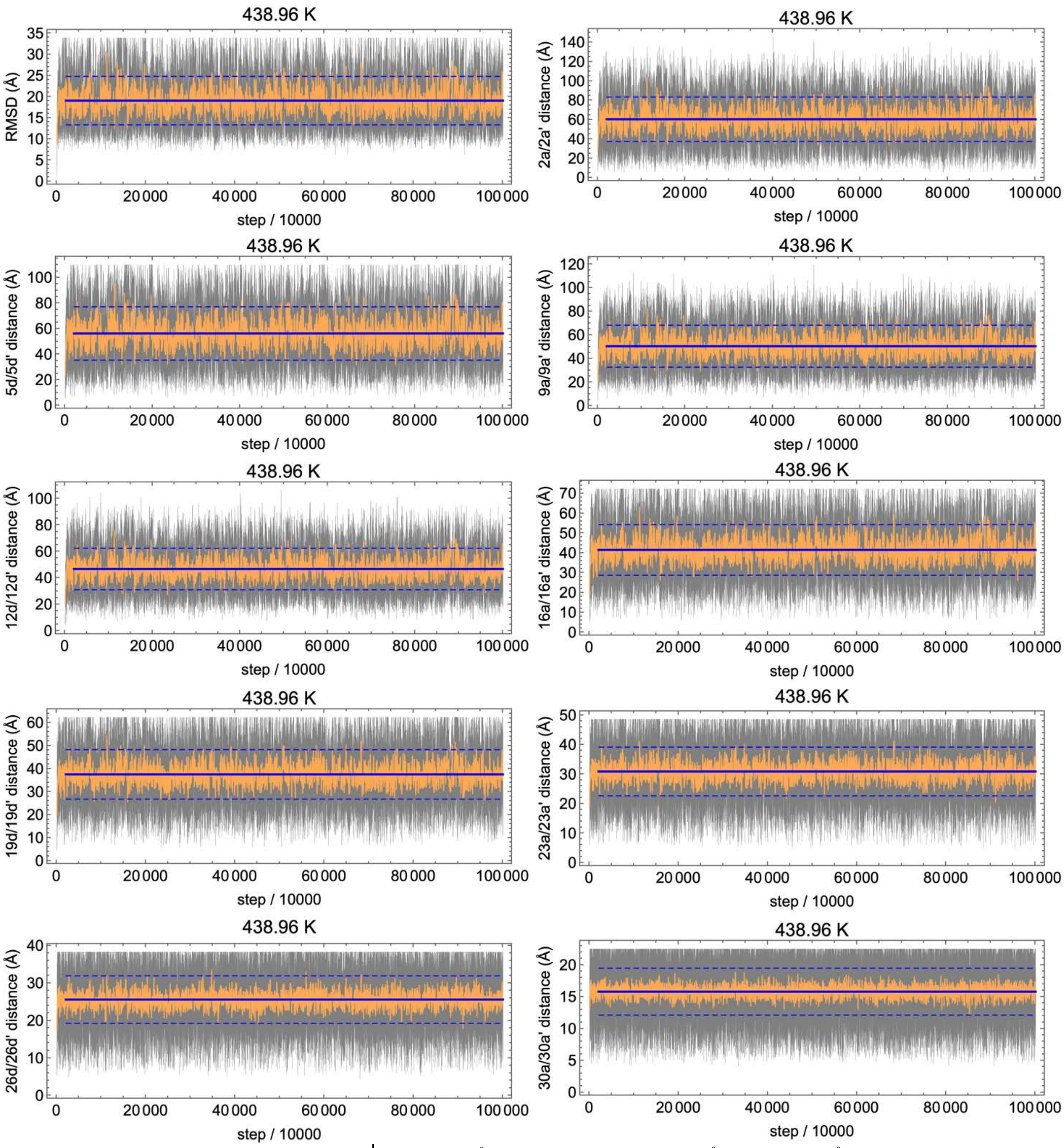

Measure at 438.96 K	Average (Å)	Standard Deviation (Å)	Slope (Å / μs)
RMSD	19.1553	5.71739	0.00693148
5d/5d' distance	56.5588	20.8637	−0.0138713
12d/12d' distance	47.0509	15.6967	0.052515
19d/19d' distance	37.6989	10.7417	0.0670913
26d/26d' distance	25.7031	6.3304	0.0686269
2a/2a' distance	60.8064	22.9664	−0.0136482
9a/9a' distance	50.8054	17.8301	0.00784693
16a/16a' distance	41.7085	12.7491	0.022669
23a/23a' distance	31.0378	8.25752	0.018601
30a/30a' distance	15.8668	3.67816	−0.0317809
Core Distance	40.804	20.1931	

Figure S400. Plots of RMSD and residue packing distances for T-REMD simulations of variant **21f/24b'-xx** at 438.96 K.

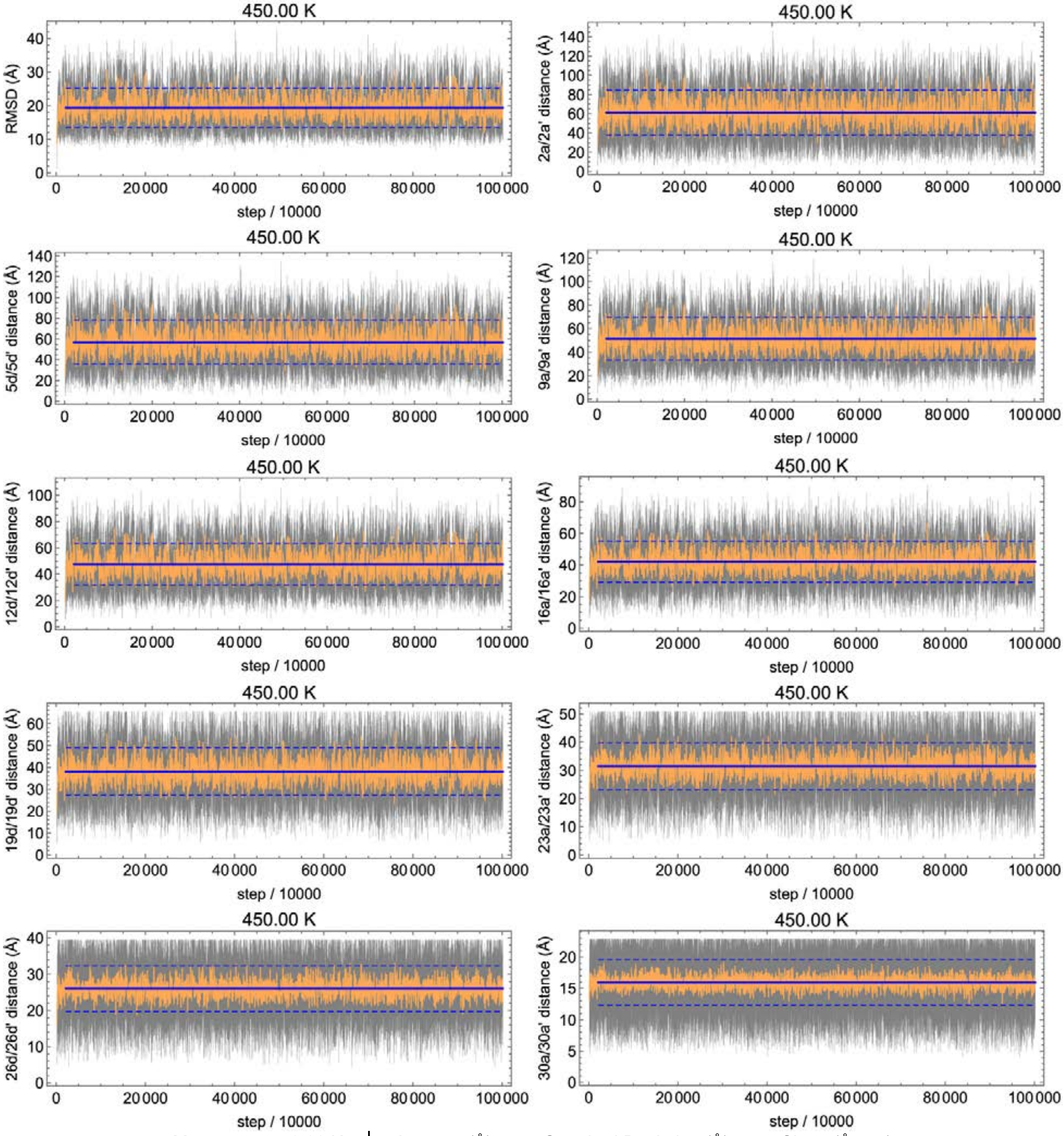

Measure at 450.00 K	Average (Å)	Standard Deviation (Å)	Slope (Å / μs)
RMSD	19.5566	5.79862	−0.0134755
5d/5d' distance	57.6429	21.2568	−0.0991599
12d/12d' distance	48.0212	15.967	0.0498687
19d/19d' distance	38.4613	10.8672	0.0792615
26d/26d' distance	26.2084	6.32887	−0.00709515
2a/2a' distance	61.9145	23.4314	−0.217762
9a/9a' distance	51.845	18.1462	−0.0152836
16a/16a' distance	42.5373	12.9835	0.0259386
23a/23a' distance	31.7178	8.31686	−0.0233411
30a/30a' distance	16.0568	3.66378	−0.0743007
Core Distance	41.6006	20.5707	

Figure S401. Plots of RMSD and residue packing distances for T-REMD simulations of variant **21f/24b'-xx** at 450.00 K.

(return to Table of Contents)

s21f/24b'-x4x

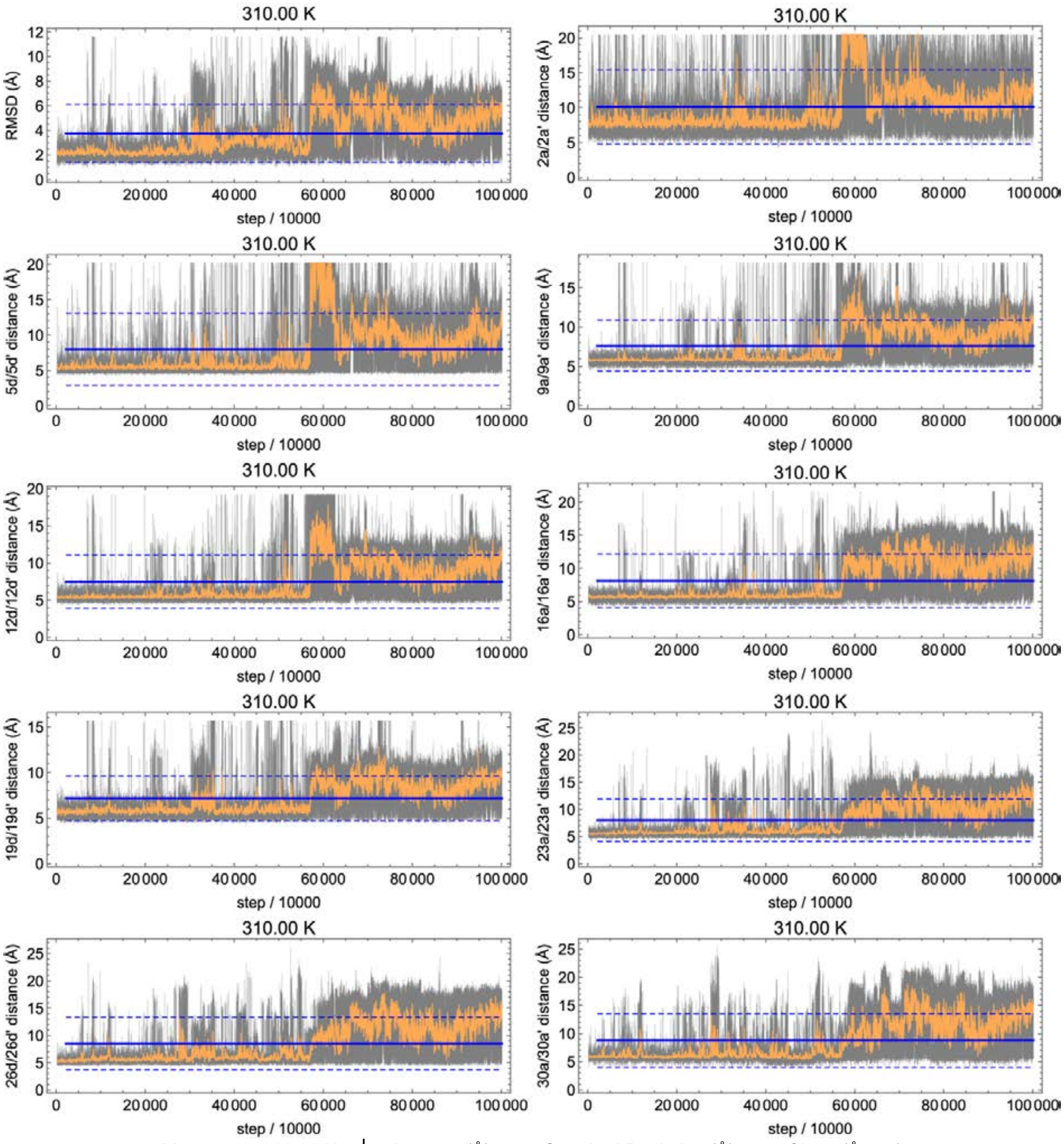

Measure at 310.00 K	Average (Å)	Standard Deviation (Å)	Slope (Å / μs)
RMSD	3.80996	2.3679	1.97019
5d/5d' distance	8.07558	5.12489	3.34588
12d/12d' distance	7.54856	3.5702	2.96875
19d/19d' distance	7.26947	2.45266	2.10503
26d/26d' distance	8.6475	4.81264	4.62292
2a/2a' distance	10.2086	5.33094	2.77343
9a/9a' distance	7.71864	3.21269	2.73068
16a/16a' distance	8.24313	4.02604	3.91101
23a/23a' distance	8.15197	3.90076	3.72711
30a/30a' distance	8.90445	4.78868	4.4875
Core Distance	8.30755	4.31344	

Figure S402. Plots of RMSD and residue packing distances for T-REMD simulations of variant **s21f/24b'-x4x** at 310.00 K.

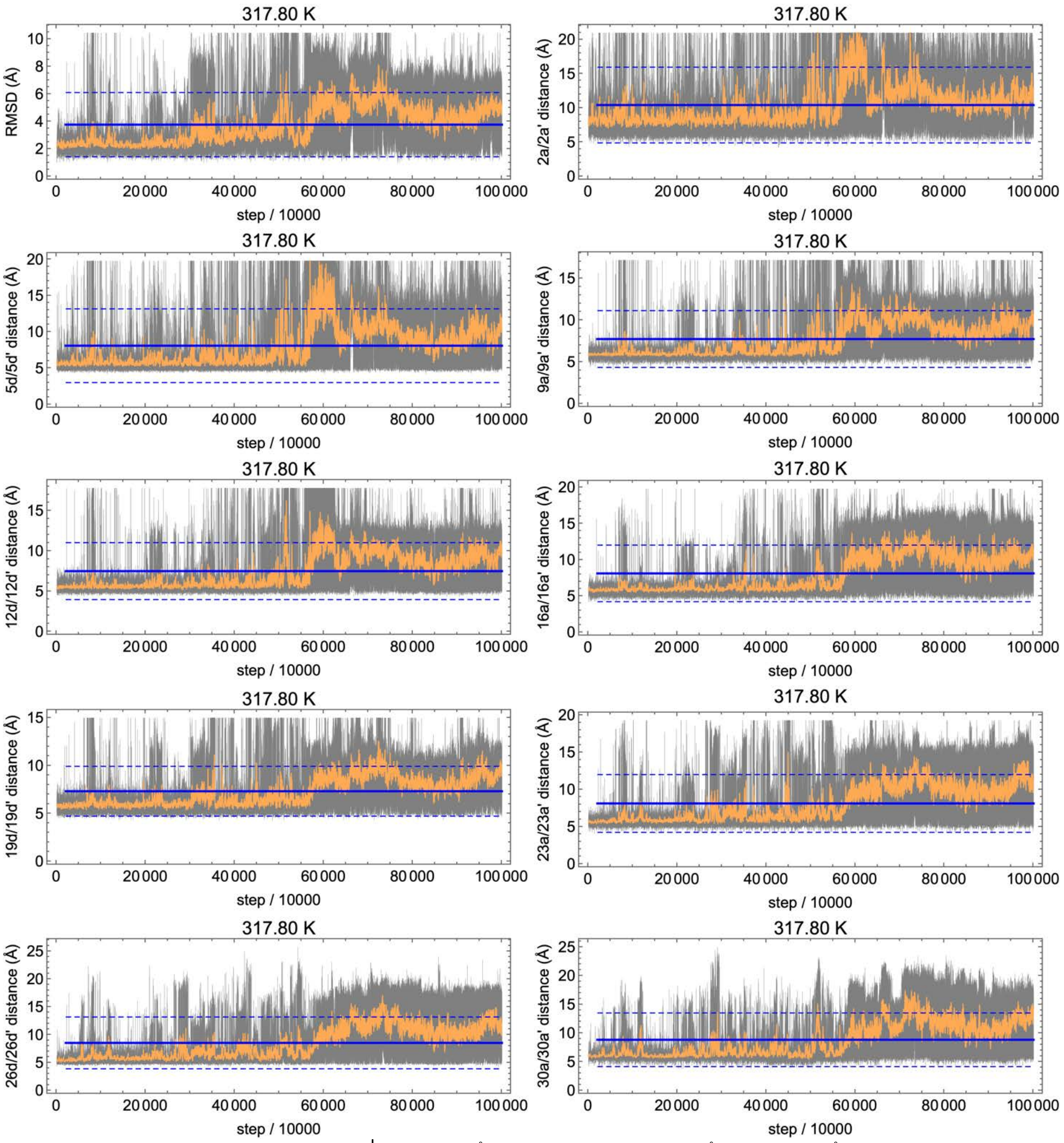

Measure at 317.80 K	Average (Å)	Standard Deviation (Å)	Slope (Å / μs)
RMSD	3.79033	2.33771	1.68121
5d/5d' distance	8.13527	5.08084	2.8736
12d/12d' distance	7.53895	3.5314	2.59006
19d/19d' distance	7.35739	2.60321	1.93069
26d/26d' distance	8.59868	4.65124	3.9155
2a/2a' distance	10.4698	5.53154	2.39041
9a/9a' distance	7.77313	3.39844	2.40894
16a/16a' distance	8.16508	3.90673	3.37699
23a/23a' distance	8.17568	3.88123	3.25516
30a/30a' distance	8.88989	4.67541	3.92323
Core Distance	8.34487	4.31999	

Figure S403. Plots of RMSD and residue packing distances for T-REMD simulations of variant **s21f/24b'-x4x** at 317.80 K.

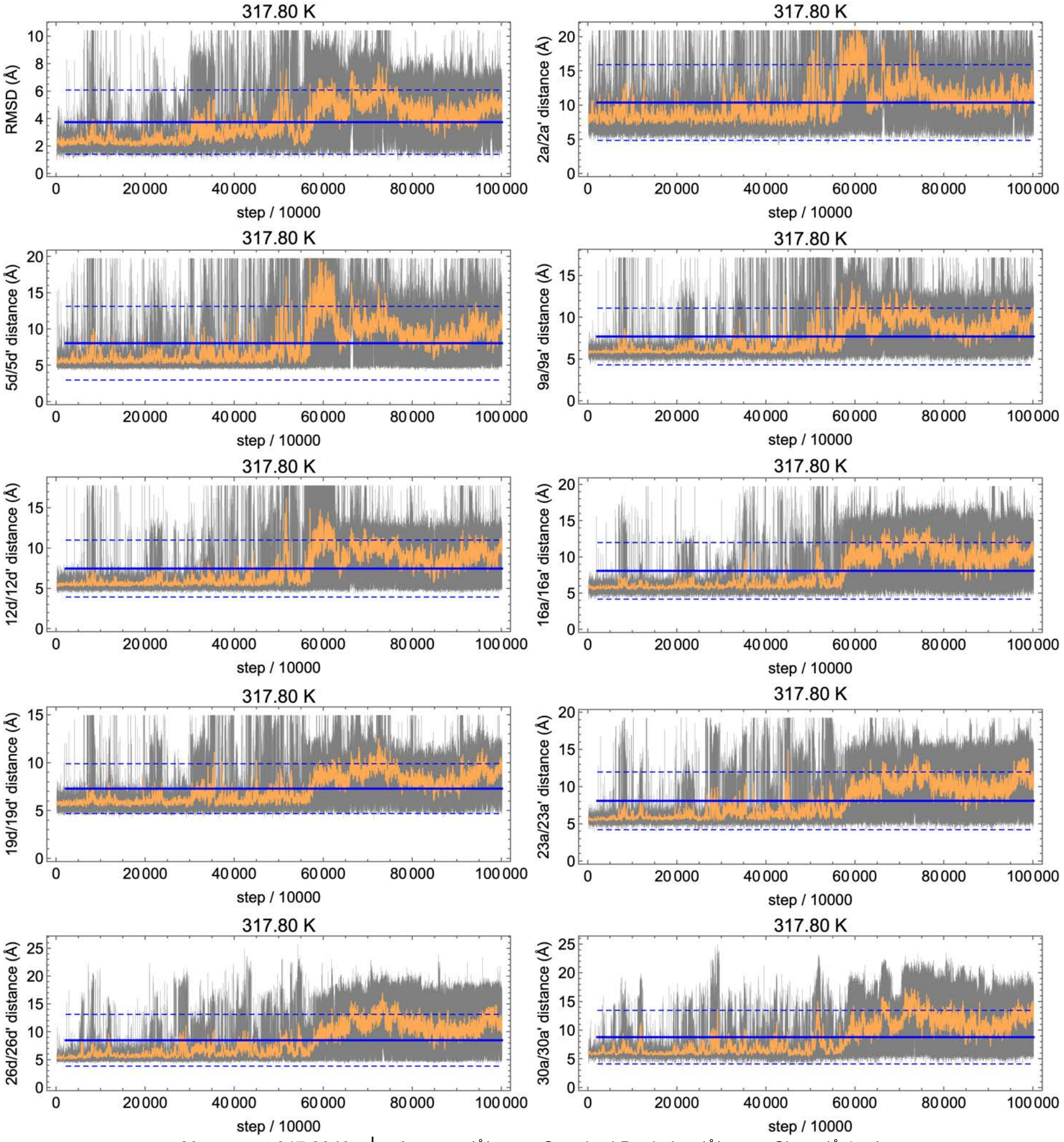

Measure at 317.80 K	Average (Å)	Standard Deviation (Å)	Slope (Å / μs)
RMSD	3.79033	2.33771	1.68121
5d/5d' distance	8.13527	5.08084	2.8736
12d/12d' distance	7.53895	3.5314	2.59006
19d/19d' distance	7.35739	2.60321	1.93069
26d/26d' distance	8.59868	4.65124	3.9155
2a/2a' distance	10.4698	5.53154	2.39041
9a/9a' distance	7.77313	3.39844	2.40894
16a/16a' distance	8.16508	3.90673	3.37699
23a/23a' distance	8.17568	3.88123	3.25516
30a/30a' distance	8.88989	4.67541	3.92323
Core Distance	8.34487	4.31999	

Figure S404. Plots of RMSD and residue packing distances for T-REMD simulations of variant **s21f/24b'-x4x** at 317.80 K.

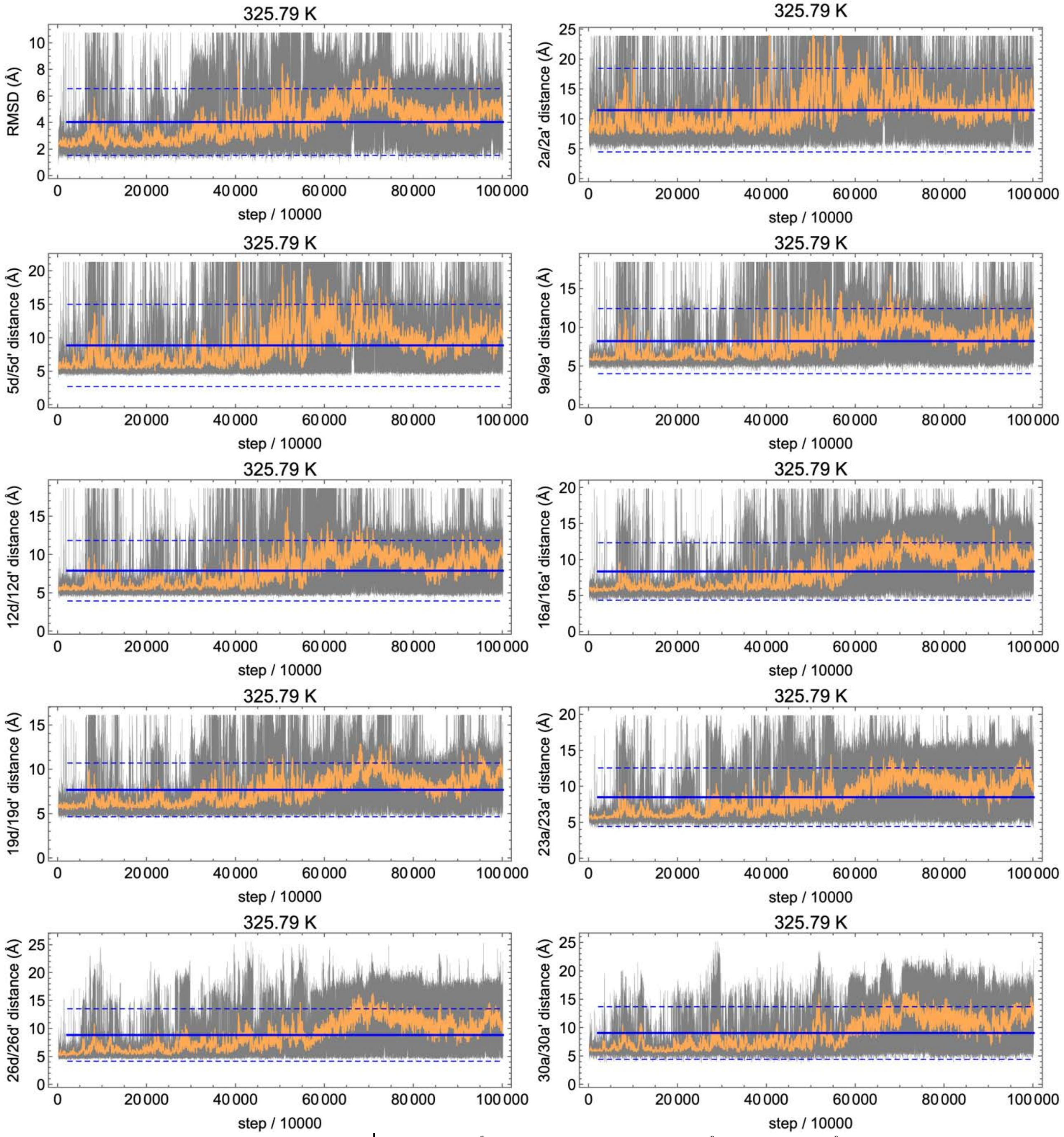

Measure at 325.79 K	Average (Å)	Standard Deviation (Å)	Slope (Å / μs)
RMSD	4.08317	2.51193	1.52876
5d/5d' distance	8.9637	6.1306	2.66537
12d/12d' distance	7.97063	3.9415	2.47586
19d/19d' distance	7.75068	3.02727	1.91823
26d/26d' distance	8.95312	4.68631	3.5019
2a/2a' distance	11.5722	6.98182	2.19898
9a/9a' distance	8.30215	4.20666	2.31689
16a/16a' distance	8.43204	3.99284	3.12323
23a/23a' distance	8.57163	4.06918	3.00701
30a/30a' distance	9.15775	4.63702	3.63186
Core Distance	8.85265	4.88479	

Figure S405. Plots of RMSD and residue packing distances for T-REMD simulations of variant **s21f/24b’-x4x** at 325.79 K.

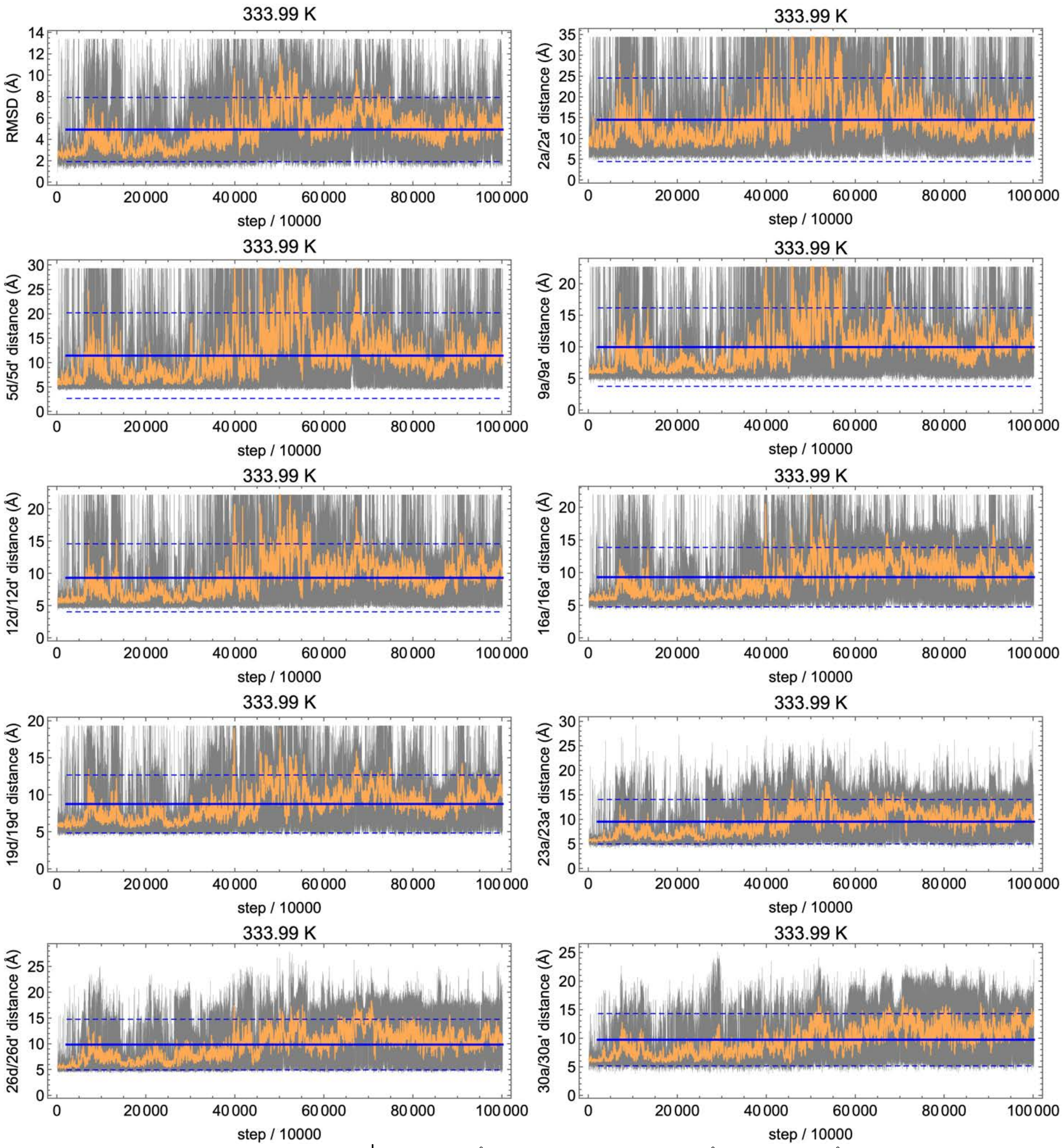

Measure at 333.99 K	Average (Å)	Standard Deviation (Å)	Slope (Å / μs)
RMSD	4.97354	2.99834	1.32694
5d/5d' distance	11.5686	8.76505	2.37836
12d/12d' distance	9.42289	5.27422	2.29791
19d/19d' distance	8.853	3.91569	1.85884
26d/26d' distance	9.9774	4.90743	2.99482
2a/2a' distance	14.65	10.0412	1.89253
9a/9a' distance	10.0566	6.20409	2.17183
16a/16a' distance	9.40615	4.55353	2.78554
23a/23a' distance	9.6551	4.53461	2.69457
30a/30a' distance	9.84211	4.57125	3.10933
Core Distance	10.3813	6.41602	

Figure S406. Plots of RMSD and residue packing distances for T-REMD simulations of variant **s21f/24b'-x4x** at 333.99 K.

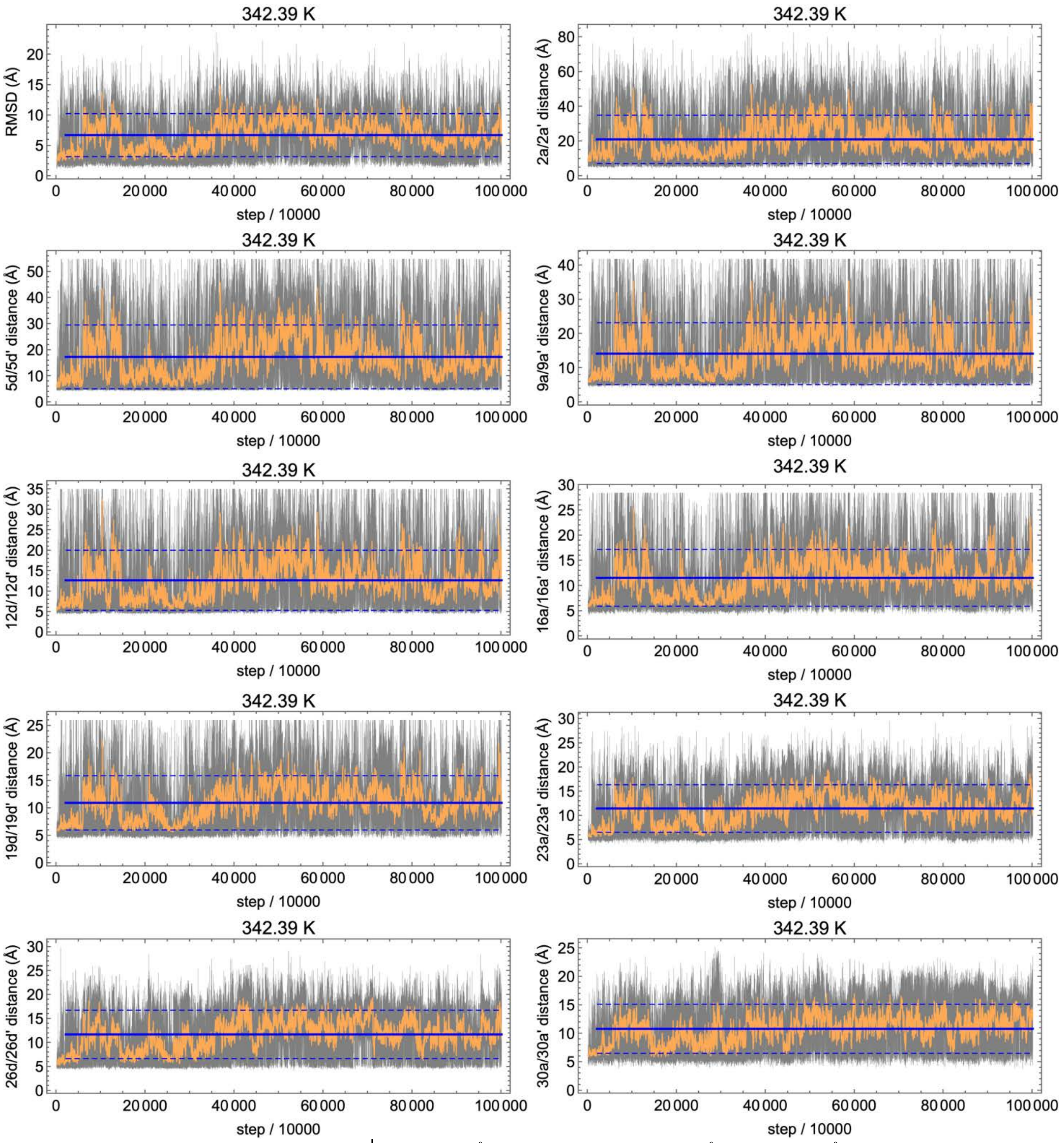

Measure at 342.39 K	Average (Å)	Standard Deviation (Å)	Slope (Å / μs)
RMSD	6.79054	3.53994	0.899403
5d/5d' distance	17.5049	12.2401	1.77379
12d/12d' distance	12.7921	7.34766	1.78445
19d/19d' distance	11.0363	4.93636	1.52619
26d/26d' distance	11.8111	5.03749	1.93808
2a/2a' distance	21.3312	13.898	1.35119
9a/9a' distance	14.2868	9.04068	1.7098
16a/16a' distance	11.6403	5.63553	2.08174
23a/23a' distance	11.5527	4.91159	1.90842
30a/30a' distance	10.9058	4.31123	1.87501
Core Distance	13.6512	8.84488	

Figure S407. Plots of RMSD and residue packing distances for T-REMD simulations of variant **s21f/24b'-x4x** at 342.39 K.

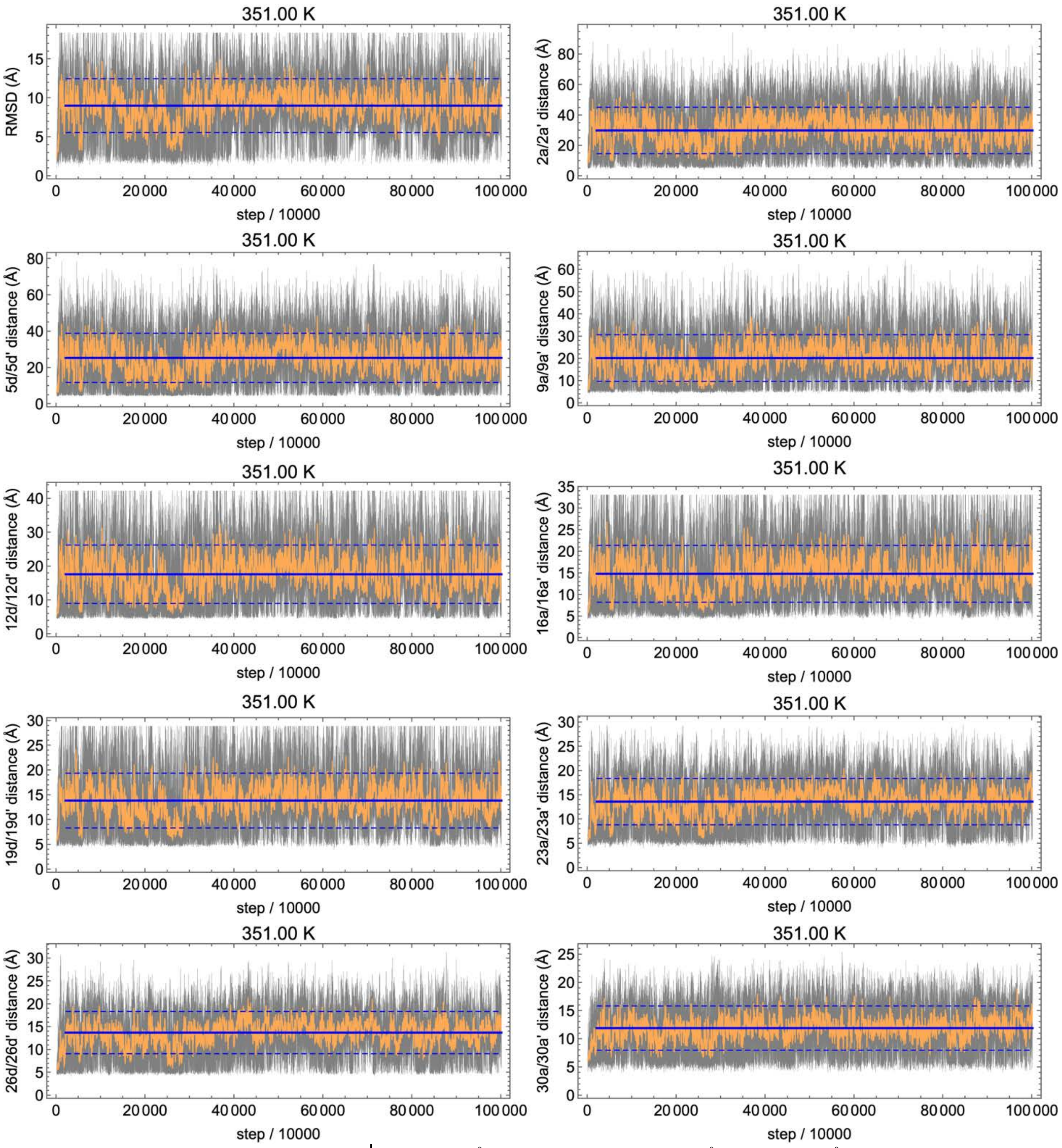

Measure at 351.00 K	Average (Å)	Standard Deviation (Å)	Slope (Å / μs)
RMSD	9.08541	3.45679	0.288515
5d/5d' distance	25.6128	13.5623	0.848276
12d/12d' distance	17.7729	8.6224	0.759717
19d/19d' distance	13.9628	5.53125	0.558413
26d/26d' distance	13.838	4.62296	0.638331
2a/2a' distance	30.2352	15.287	0.633612
9a/9a' distance	20.4279	10.4835	0.848046
16a/16a' distance	14.9395	6.57376	0.920186
23a/23a' distance	13.7133	4.78096	0.853105
30a/30a' distance	11.9977	3.92316	0.588284
Core Distance	18.0556	10.782	

Figure S408. Plots of RMSD and residue packing distances for T-REMD simulations of variant **s21f/24b’-x4x** at 351.00 K.

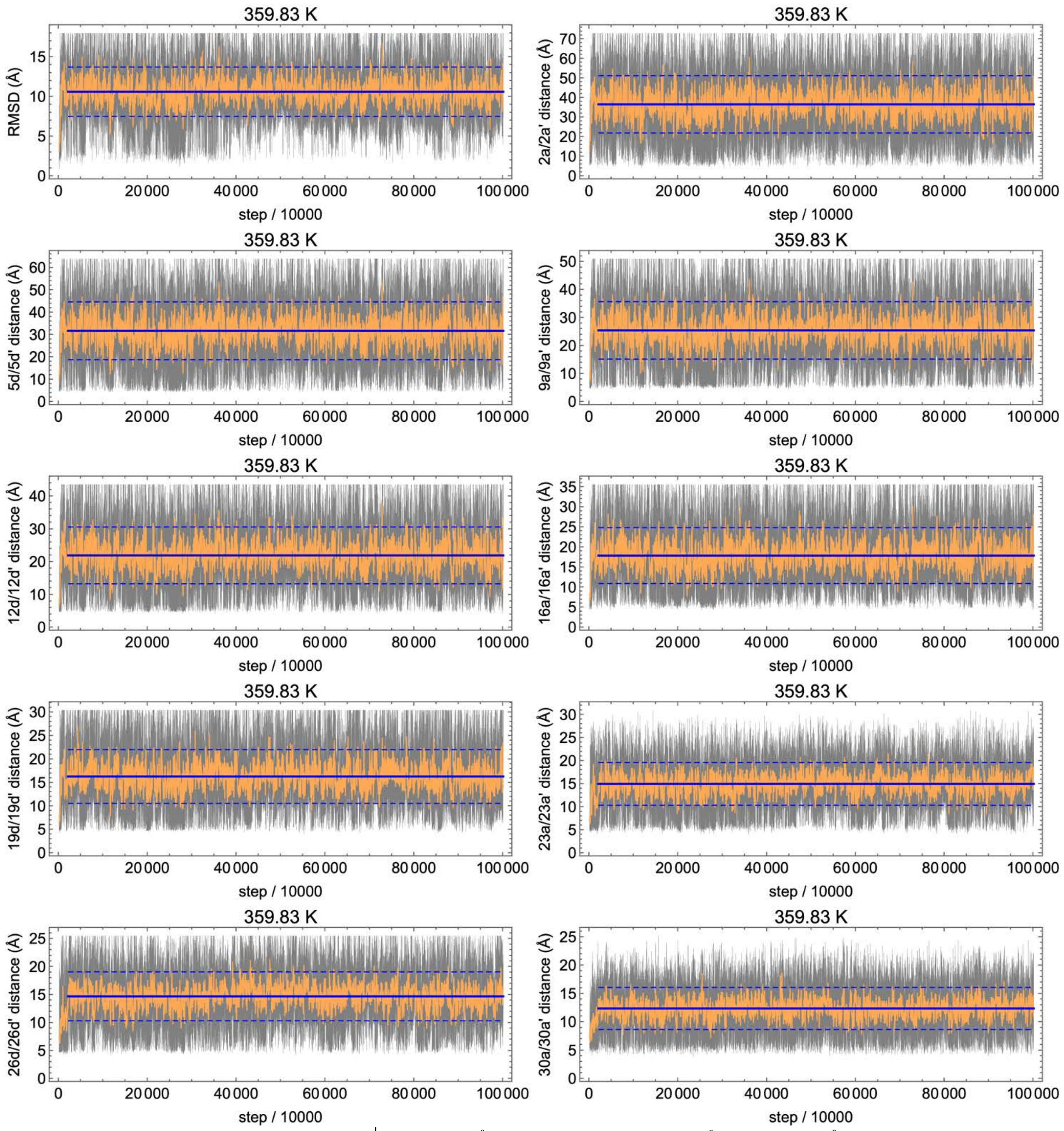

Measure at 359.83 K	Average (Å)	Standard Deviation (Å)	Slope (Å / μs)
RMSD	10.6686	3.10792	−0.0515474
5d/5d' distance	31.9139	12.9264	−0.0485461
12d/12d' distance	22.0849	8.66571	0.00467635
19d/19d' distance	16.3845	5.7182	−0.0851384
26d/26d' distance	14.7793	4.36268	0.0142891
2a/2a' distance	36.8583	14.6446	−0.0907934
9a/9a' distance	25.6041	10.2553	0.0446165
16a/16a' distance	17.9882	6.96394	0.0962839
23a/23a' distance	15.071	4.63008	0.157343
30a/30a' distance	12.4612	3.71155	0.0829039
Core Distance	21.4606	11.8577	

Figure S409. Plots of RMSD and residue packing distances for T-REMD simulations of variant **s21f/24b'-x4x** at 359.83 K.

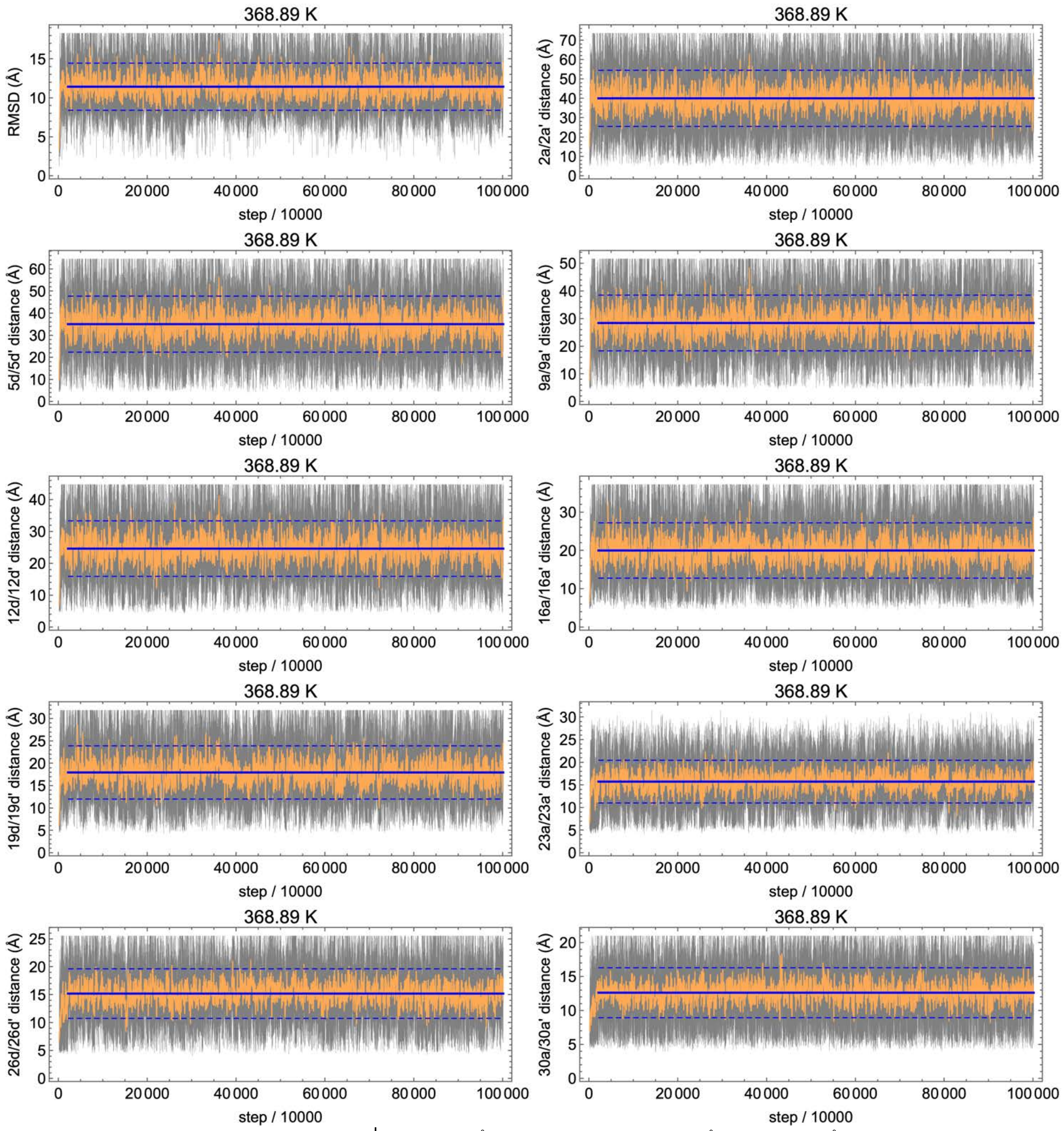

Measure at 368.89 K	Average (Å)	Standard Deviation (Å)	Slope (Å / μs)
RMSD	11.5126	3.02463	−0.0812242
5d/5d' distance	35.3217	12.6902	−0.118818
12d/12d' distance	24.8334	8.70956	−0.0662594
19d/19d' distance	18.0969	5.94427	−0.228189
26d/26d' distance	15.3059	4.43504	−0.0542865
2a/2a' distance	40.3183	14.4801	−0.182017
9a/9a' distance	28.6399	10.0855	−0.0345862
16a/16a' distance	20.1449	7.21118	−0.122653
23a/23a' distance	15.8674	4.71858	−0.0196531
30a/30a' distance	12.7038	3.67352	0.0194294
Core Distance	23.4702	12.5798	

Figure S410. Plots of RMSD and residue packing distances for T-REMD simulations of variant **s21f/24b’-x4x** at 368.89 K.

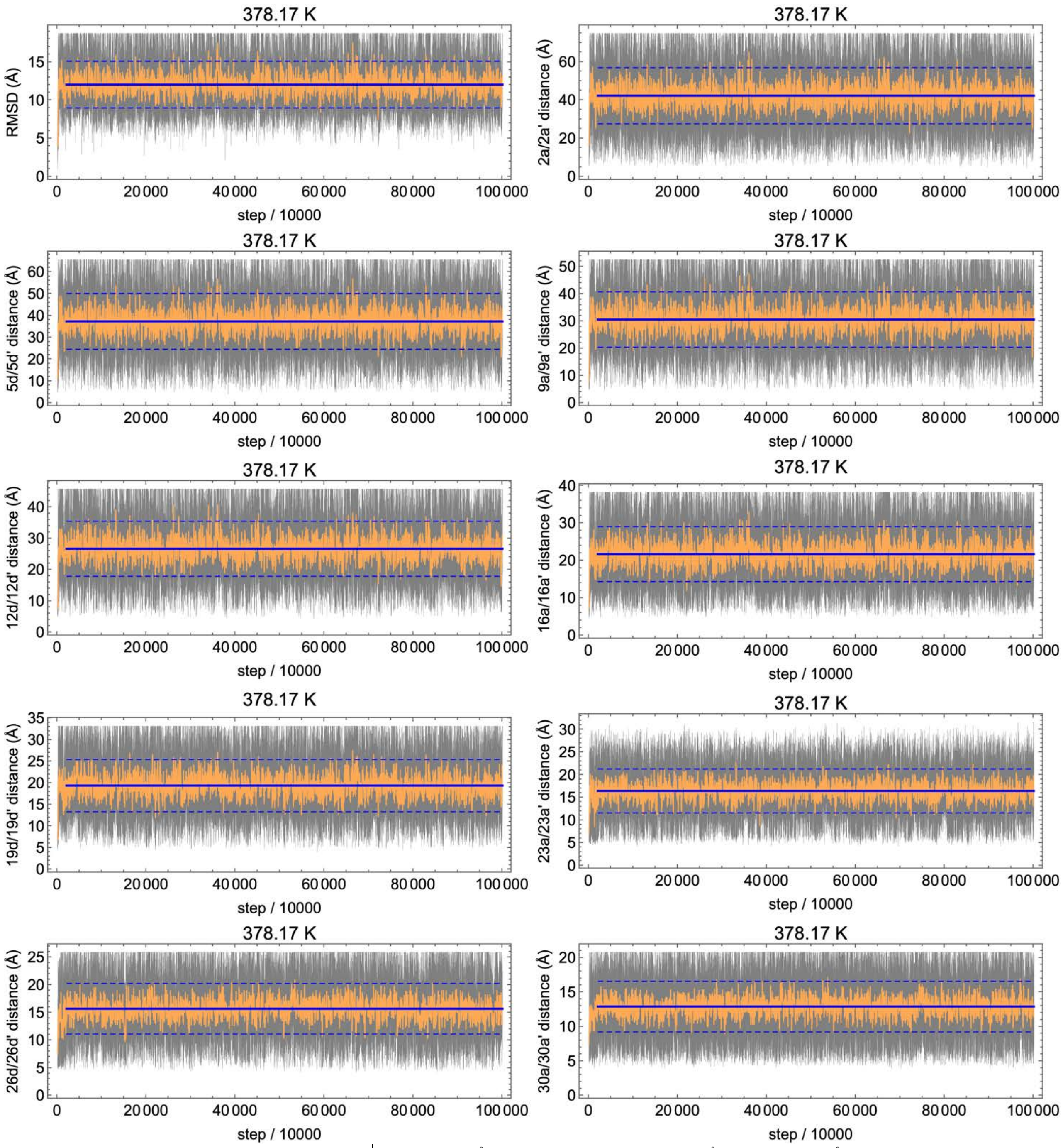

Measure at 378.17 K	Average (Å)	Standard Deviation (Å)	Slope (Å / μs)
RMSD	12.0935	3.05869	−0.054608
5d/5d' distance	37.4957	12.7626	−0.0323117
12d/12d' distance	26.8054	8.7902	−0.173476
19d/19d' distance	19.4748	6.05193	−0.235655
26d/26d' distance	15.7579	4.57486	−0.0750339
2a/2a' distance	42.4485	14.7036	0.0275333
9a/9a' distance	30.7375	10.1552	−0.108476
16a/16a' distance	21.8219	7.34678	−0.202726
23a/23a' distance	16.5232	4.84758	−0.0581595
30a/30a' distance	12.9559	3.67449	0.0398888
Core Distance	24.8912	13.1133	

Figure S411. Plots of RMSD and residue packing distances for T-REMD simulations of variant **s21f/24b'-x4x** at 378.17 K.

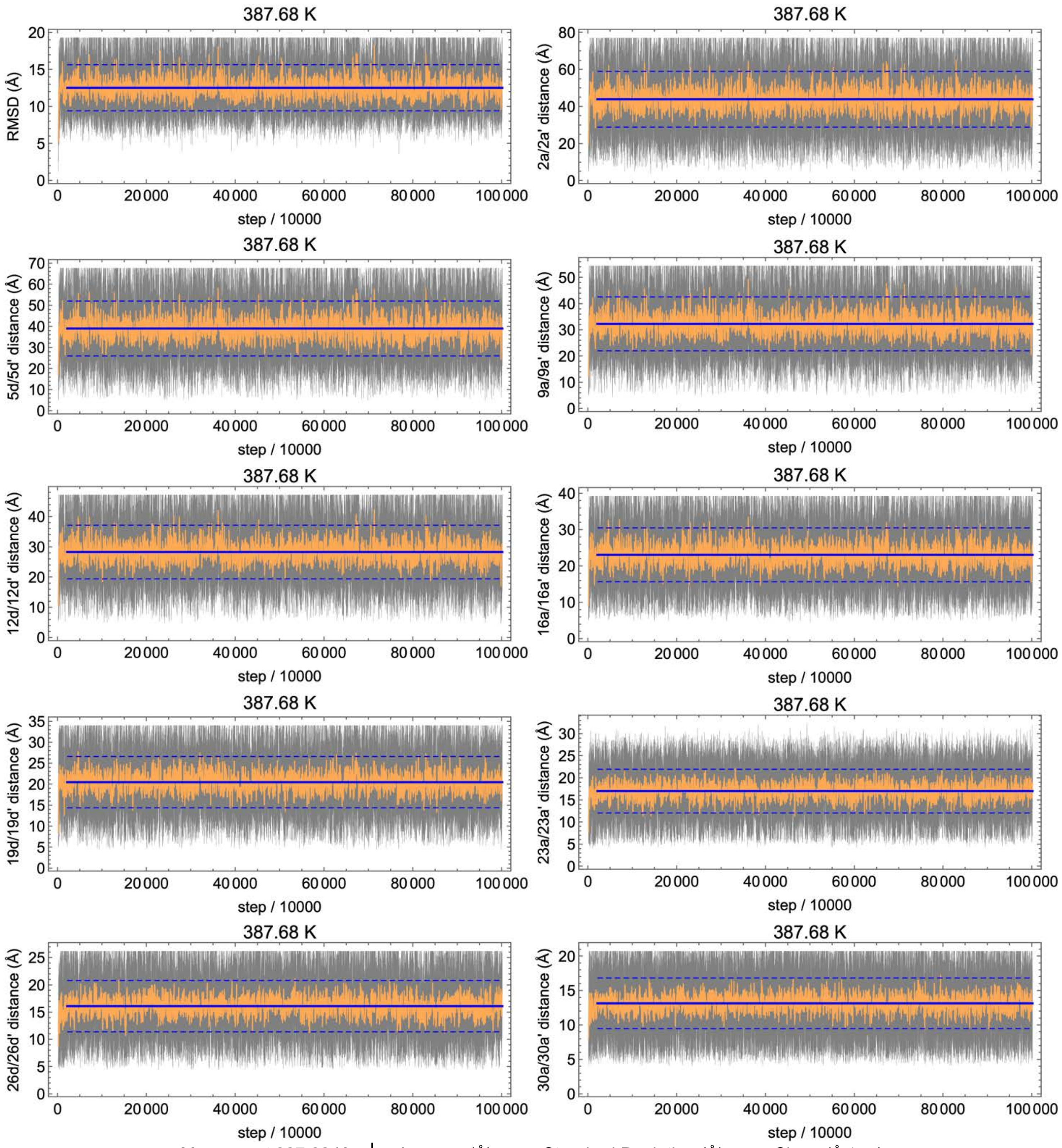

Measure at 387.68 K	Average (Å)	Standard Deviation (Å)	Slope (Å / μs)
RMSD	12.6151	3.12231	−0.052199
5d/5d' distance	39.3067	13.0155	−0.189962
12d/12d' distance	28.4971	8.87581	−0.185368
19d/19d' distance	20.6481	6.1191	−0.145592
26d/26d' distance	16.2073	4.71398	−0.0761566
2a/2a' distance	44.233	15.0523	−0.0950354
9a/9a' distance	32.5328	10.2925	−0.232026
16a/16a' distance	23.2602	7.41072	−0.191868
23a/23a' distance	17.1455	4.94884	0.0151241
30a/30a' distance	13.2317	3.68333	0.00138178
Core Distance	26.118	13.6019	

Figure S412. Plots of RMSD and residue packing distances for T-REMD simulations of variant **s21f/24b’-x4x** at 387.68 K.

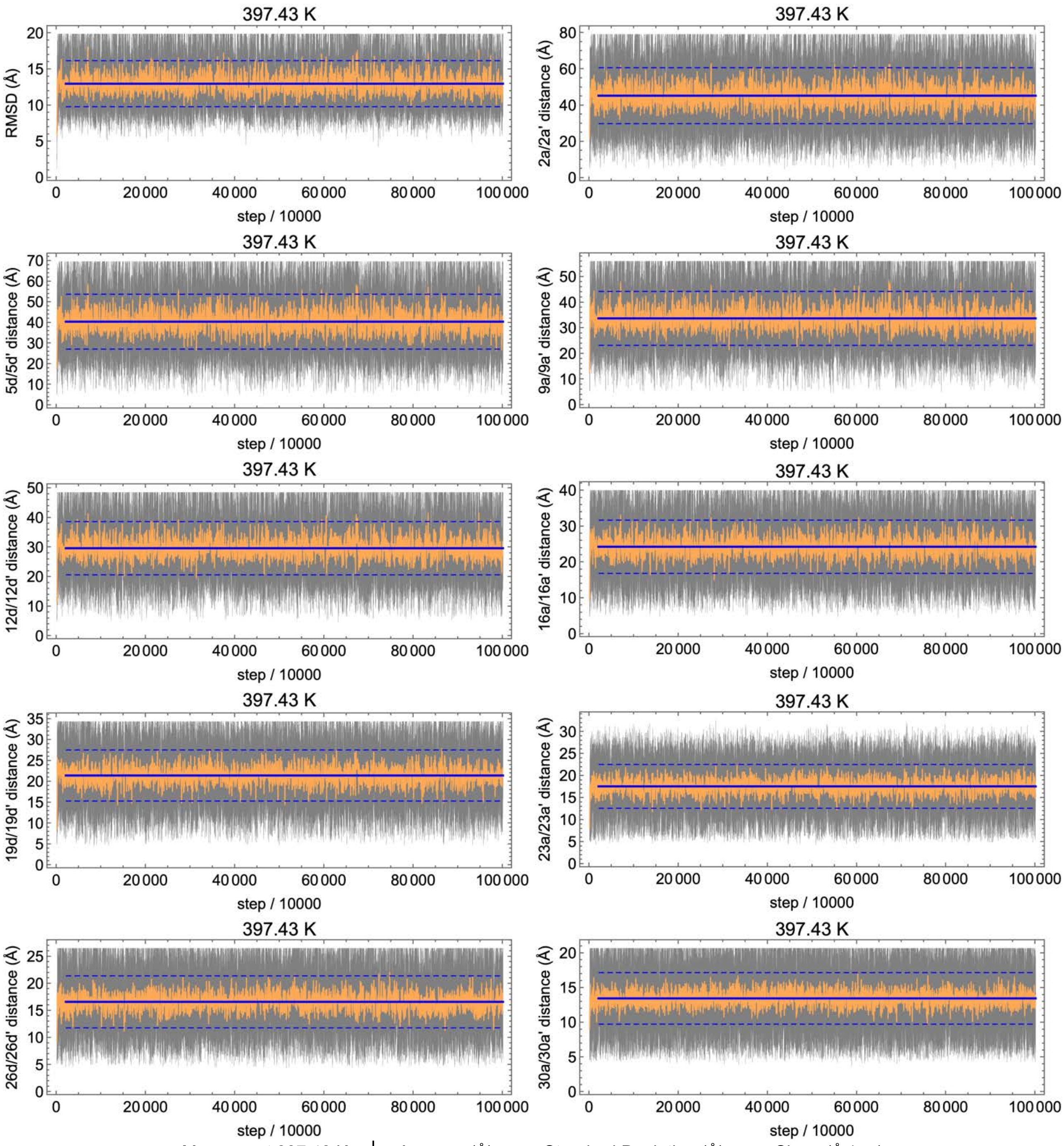

Measure at 397.43 K	Average (Å)	Standard Deviation (Å)	Slope (Å / μs)
RMSD	13.0412	3.18489	−0.0701663
5d/5d' distance	40.6708	13.3118	−0.244187
12d/12d' distance	29.7978	9.00258	−0.0809538
19d/19d' distance	21.5425	6.11891	−0.020044
26d/26d' distance	16.6873	4.79888	−0.0750609
2a/2a' distance	45.5185	15.4217	−0.243078
9a/9a' distance	33.9092	10.5219	−0.199596
16a/16a' distance	24.4107	7.42384	−0.0273349
23a/23a' distance	17.6796	4.967	0.0497975
30a/30a' distance	13.5327	3.71425	−0.0336429
Core Distance	27.0832	13.9731	

Figure S413. Plots of RMSD and residue packing distances for T-REMD simulations of variant **s21f/24b'-x4x** at 397.43 K.

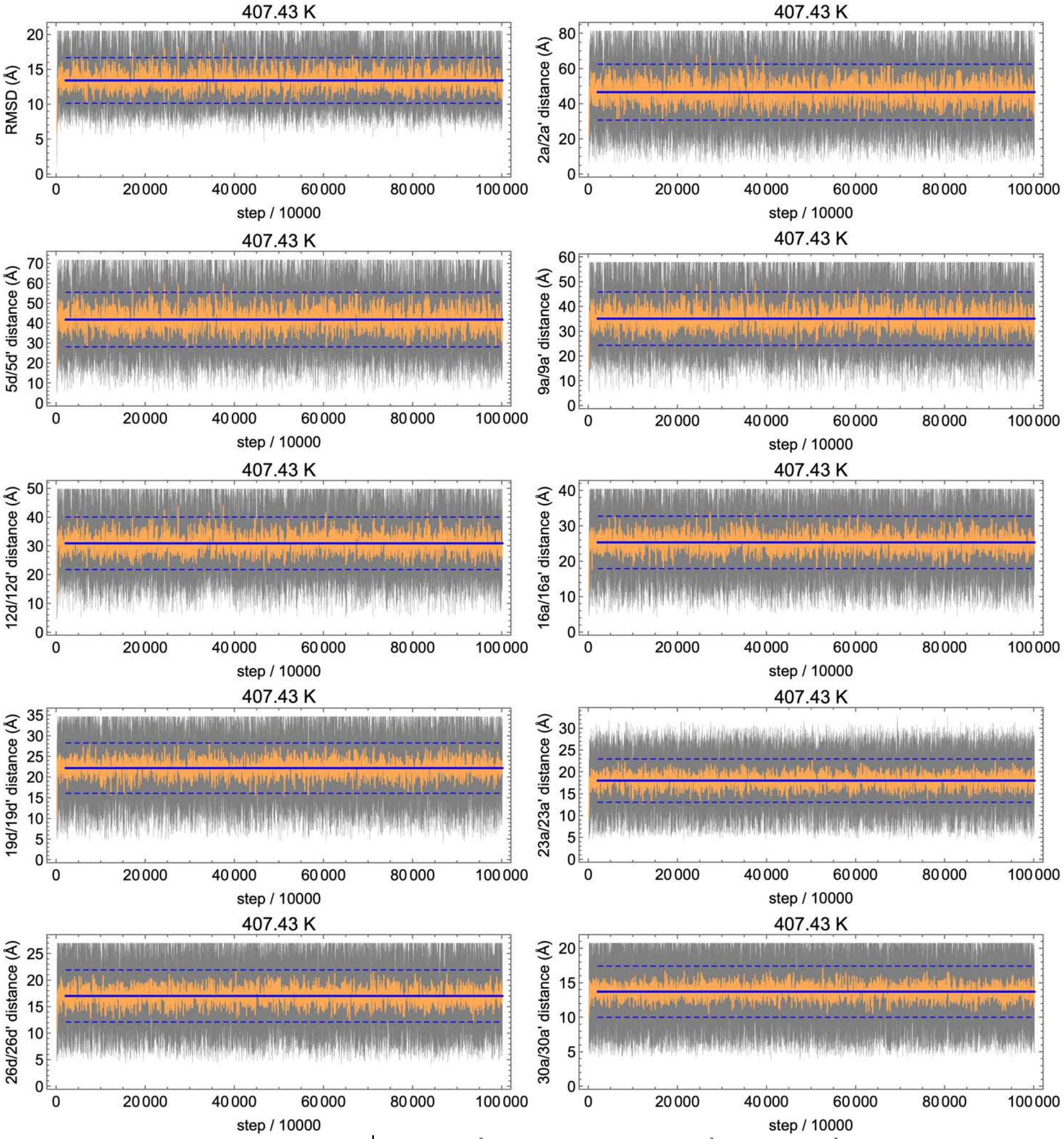

Measure at 407.43 K	Average (Å)	Standard Deviation (Å)	Slope (Å / μs)
RMSD	13.4971	3.26647	−0.0655346
5d/5d' distance	42.1165	13.6547	−0.287528
12d/12d' distance	31.1296	9.12927	−0.127997
19d/19d' distance	22.337	6.08895	−0.0812091
26d/26d' distance	17.1386	4.90525	−0.0909955
2a/2a' distance	46.9326	15.886	−0.388515
9a/9a' distance	35.3268	10.758	−0.228479
16a/16a' distance	25.5008	7.39856	−0.125663
23a/23a' distance	18.1536	4.95787	0.0148375
30a/30a' distance	13.8059	3.7087	−0.0660097
Core Distance	28.049	14.4067	

Figure S414. Plots of RMSD and residue packing distances for T-REMD simulations of variant **s21f/24b'-x4x** at 407.43 K.

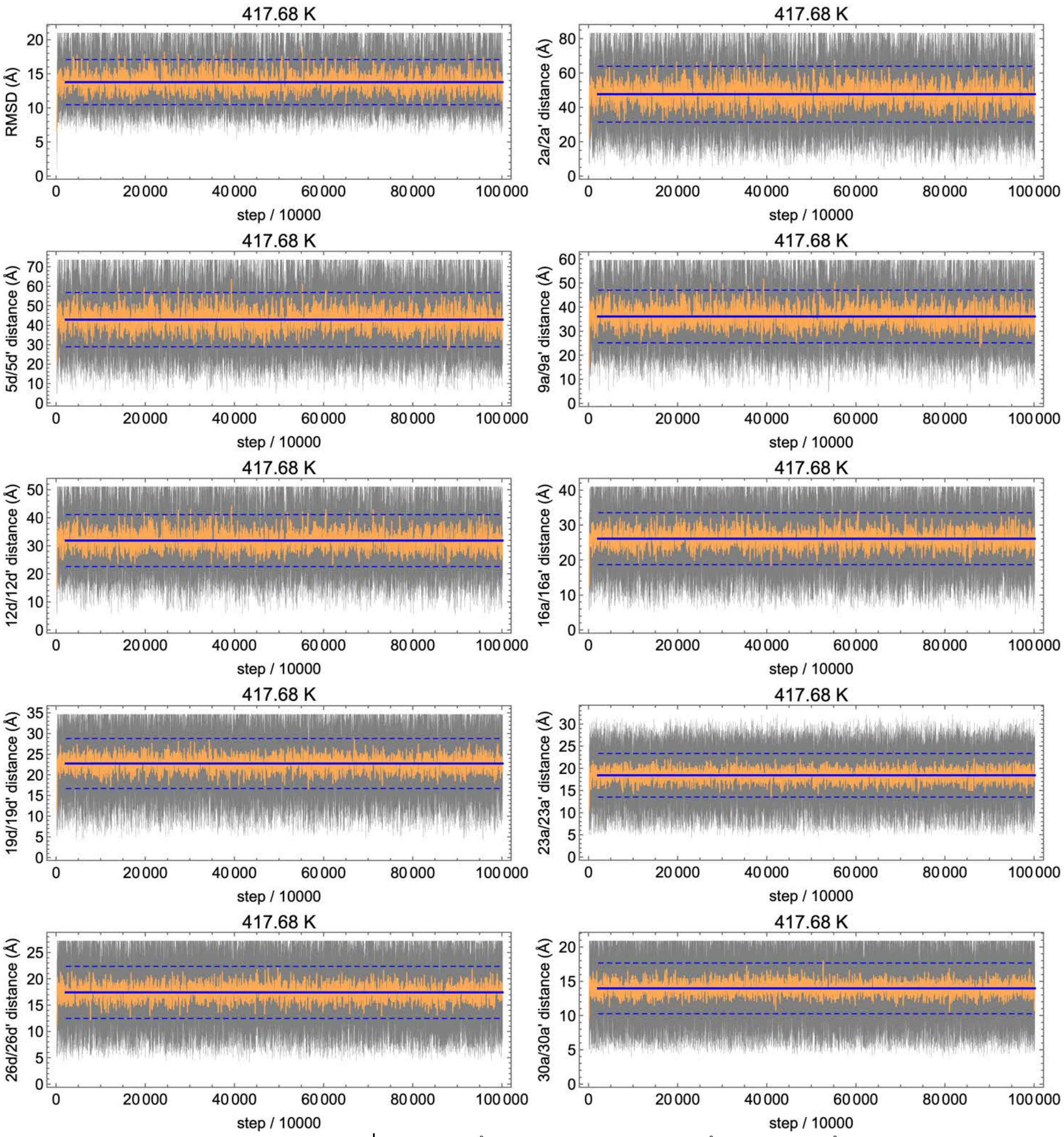

Measure at 417.68 K	Average (Å)	Standard Deviation (Å)	Slope (Å / μs)
RMSD	13.8761	3.31056	−0.110515
5d/5d' distance	43.1742	13.9427	−0.46595
12d/12d' distance	32.088	9.26472	−0.258767
19d/19d' distance	22.9041	6.04866	−0.0982314
26d/26d' distance	17.5533	4.94891	−0.0678931
2a/2a' distance	48.0635	16.2434	−0.542325
9a/9a' distance	36.3858	10.9447	−0.425284
16a/16a' distance	26.3049	7.43677	−0.136273
23a/23a' distance	18.5902	4.91622	−0.00563724
30a/30a' distance	14.0696	3.70339	−0.0504421
Core Distance	28.7926	14.7305	

Figure S415. Plots of RMSD and residue packing distances for T-REMD simulations of variant **s21f/24b'-x4x** at 417.68 K.

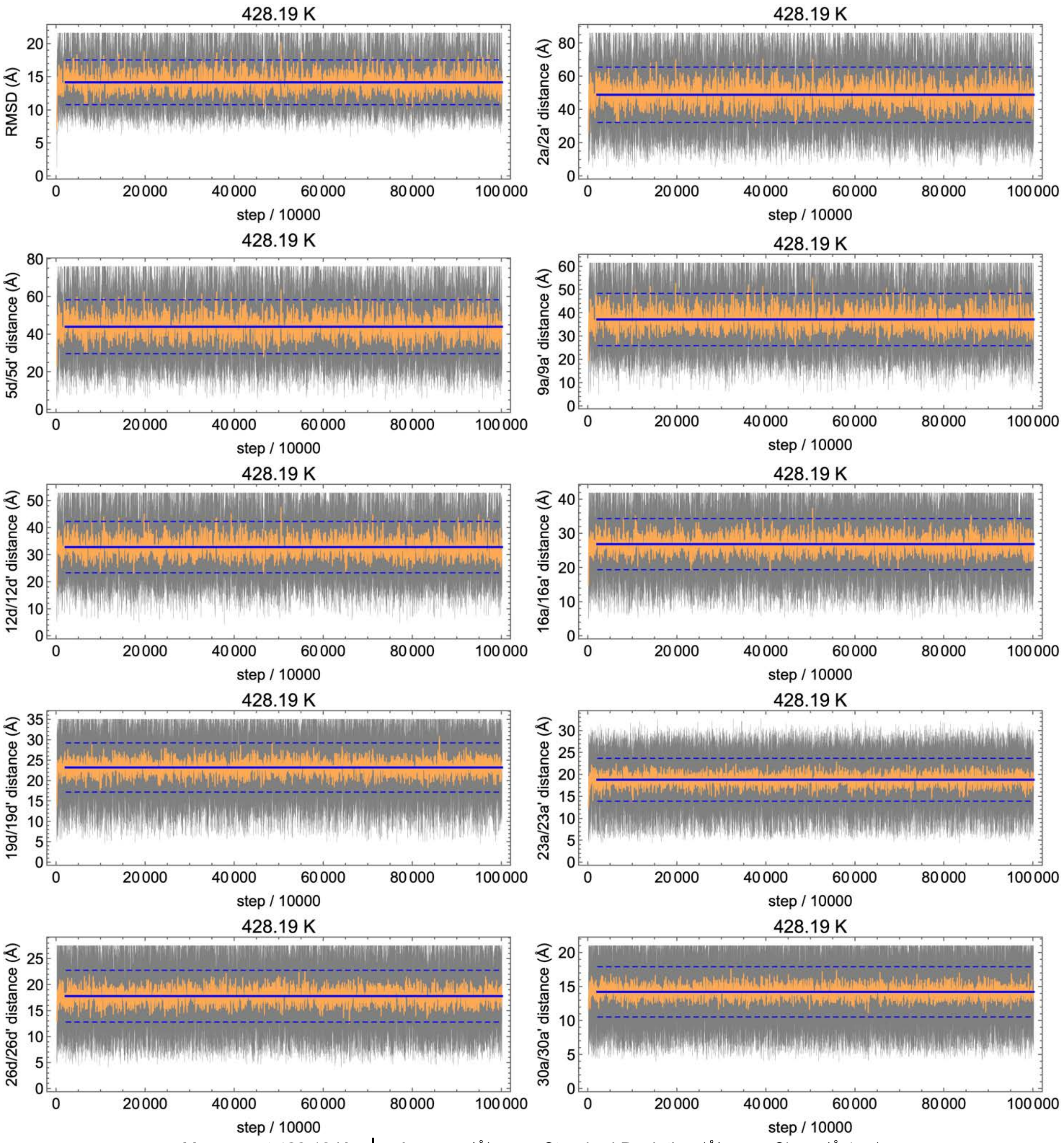

Measure at 428.19 K	Average (Å)	Standard Deviation (Å)	Slope (Å / μs)
RMSD	14.25	3.37913	−0.0920572
5d/5d' distance	44.2834	14.2985	−0.492941
12d/12d' distance	33.0278	9.49034	−0.160851
19d/19d' distance	23.3673	6.02149	0.0112293
26d/26d' distance	17.9145	4.97952	−0.00652953
2a/2a' distance	49.2159	16.6473	−0.531586
9a/9a' distance	37.4257	11.2409	−0.360946
16a/16a' distance	27.0312	7.48801	−0.0293686
23a/23a' distance	18.9418	4.89308	0.0793972
30a/30a' distance	14.312	3.68997	−0.0607393
Core Distance	29.5022	15.1118	

Figure S416. Plots of RMSD and residue packing distances for T-REMD simulations of variant **s21f/24b'-x4x** at 428.19 K.

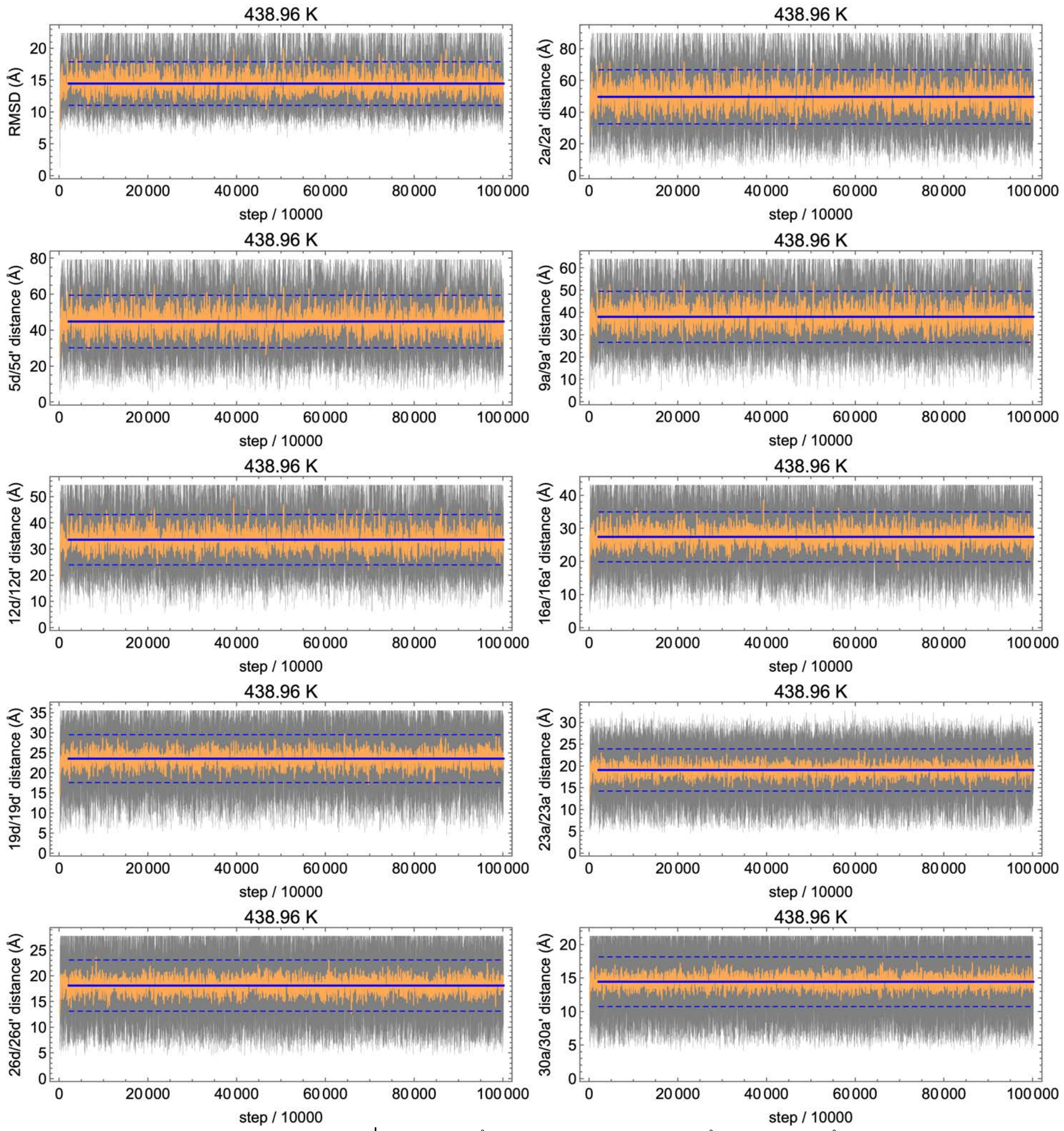

Measure at 438.96 K	Average (Å)	Standard Deviation (Å)	Slope (Å / μs)
RMSD	14.5654	3.4193	−0.112525
5d/5d' distance	45.1512	14.6297	−0.571639
12d/12d' distance	33.812	9.61444	−0.225499
19d/19d' distance	23.7252	5.97371	−0.0312899
26d/26d' distance	18.2349	4.98541	0.0434291
2a/2a' distance	50.1059	17.0573	−0.708907
9a/9a' distance	38.3403	11.4601	−0.404483
16a/16a' distance	27.6202	7.54467	−0.0606423
23a/23a' distance	19.2124	4.83073	0.0639743
30a/30a' distance	14.5366	3.70228	−0.0439986
Core Distance	30.0821	15.4202	

Figure S417. Plots of RMSD and residue packing distances for T-REMD simulations of variant **s21f/24b'-x4x** at 438.96 K.

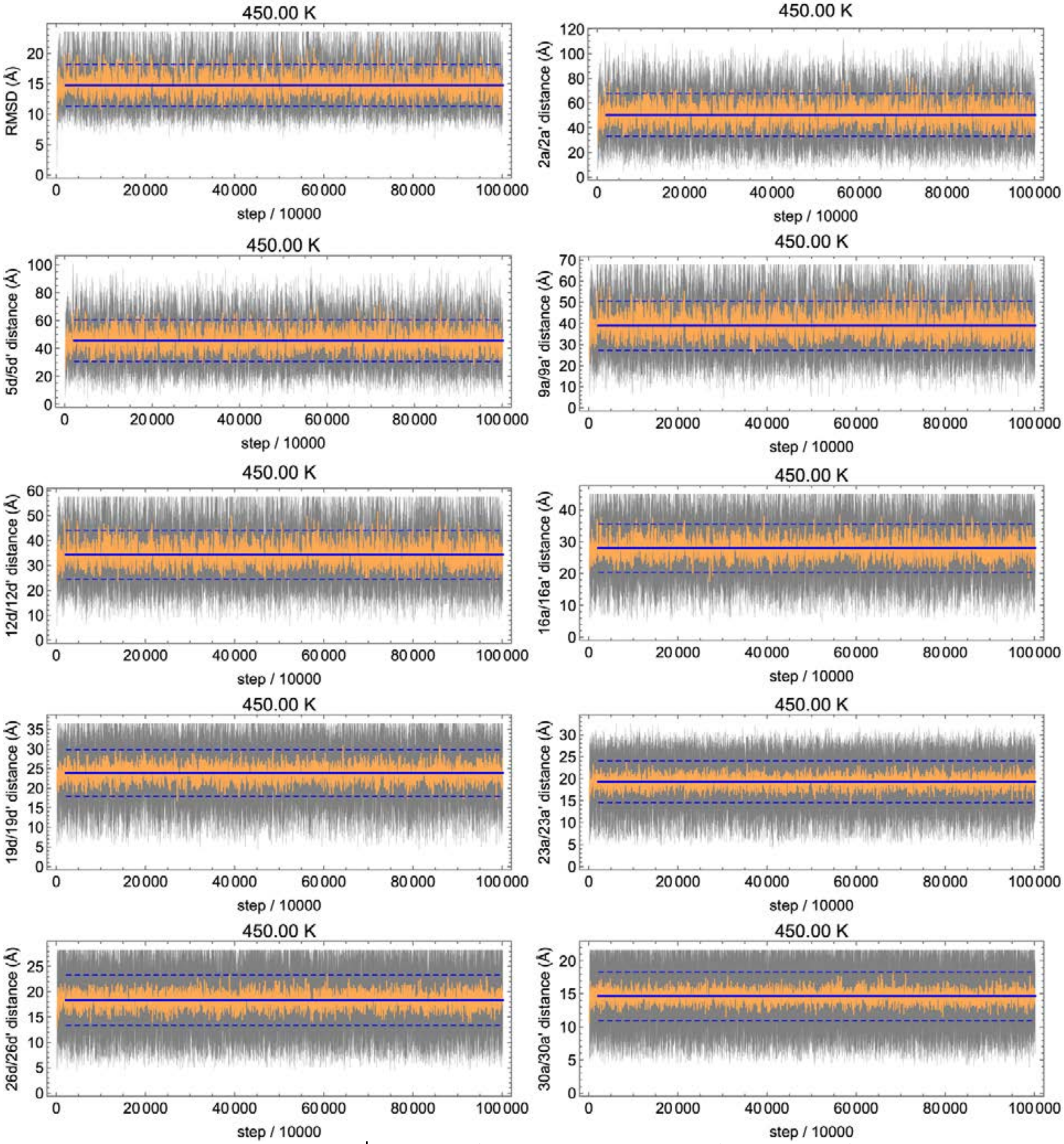

Measure at 450.00 K	Average (Å)	Standard Deviation (Å)	Slope (Å / μs)
RMSD	14.9064	3.46228	−0.0917727
5d/5d' distance	46.1263	14.9124	−0.458917
12d/12d' distance	34.5692	9.80085	−0.170787
19d/19d' distance	23.9908	5.97943	−0.0270841
26d/26d' distance	18.4983	4.96817	0.0445595
2a/2a' distance	51.1619	17.3766	−0.530325
9a/9a' distance	39.2034	11.7135	−0.380786
16a/16a' distance	28.1766	7.61448	−0.0292113
23a/23a' distance	19.4656	4.76251	0.0608534
30a/30a' distance	14.7358	3.68739	−0.0591655
Core Distance	30.6586	15.7675	

Figure S418. Plots of RMSD and residue packing distances for T-REMD simulations of variant **s21f/24b'-x4x** at 450.00 K.

(return to Table of Contents)

28f/31b'-xx

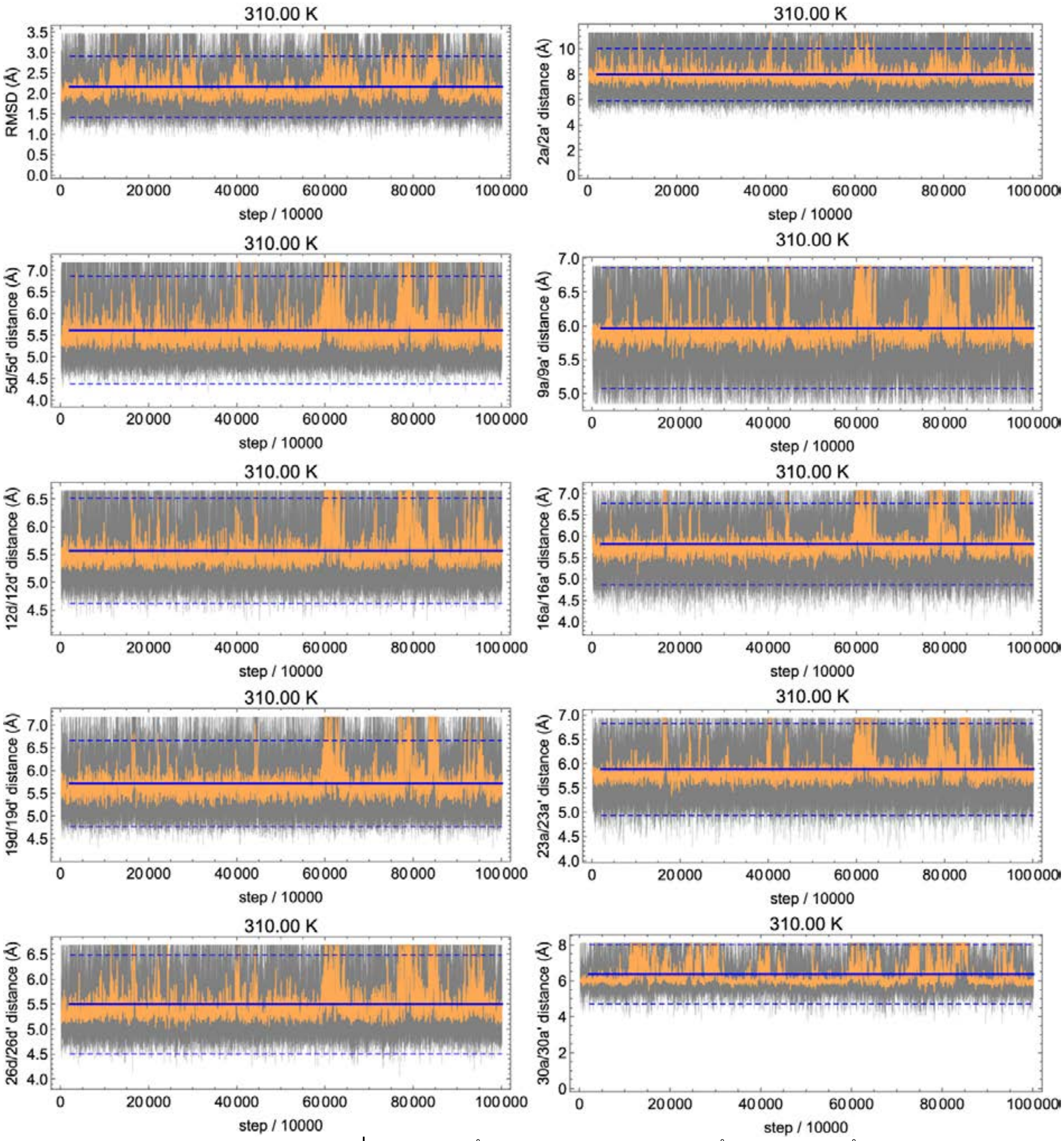

Measure at 310.00 K	Average (Å)	Standard Deviation (Å)	Slope (Å / μs)
RMSD	2.17941	0.753614	0.022357
5d/5d' distance	5.63353	1.24573	0.107897
12d/12d' distance	5.58092	0.94796	0.120127
19d/19d' distance	5.73021	0.949812	0.120823
26d/26d' distance	5.5115	0.991594	0.129323
2a/2a' distance	8.03611	2.07045	0.0506213
9a/9a' distance	5.97732	0.891422	0.089356
16a/16a' distance	5.83198	0.954803	0.10516
23a/23a' distance	5.89742	0.94454	0.114067
30a/30a' distance	6.39784	1.66081	0.0255511
Core Distance	6.06631	1.4493	

Figure S419. Plots of RMSD and residue packing distances for T-REMD simulations of variant **28f/31b'-xx** at 310.00 K.

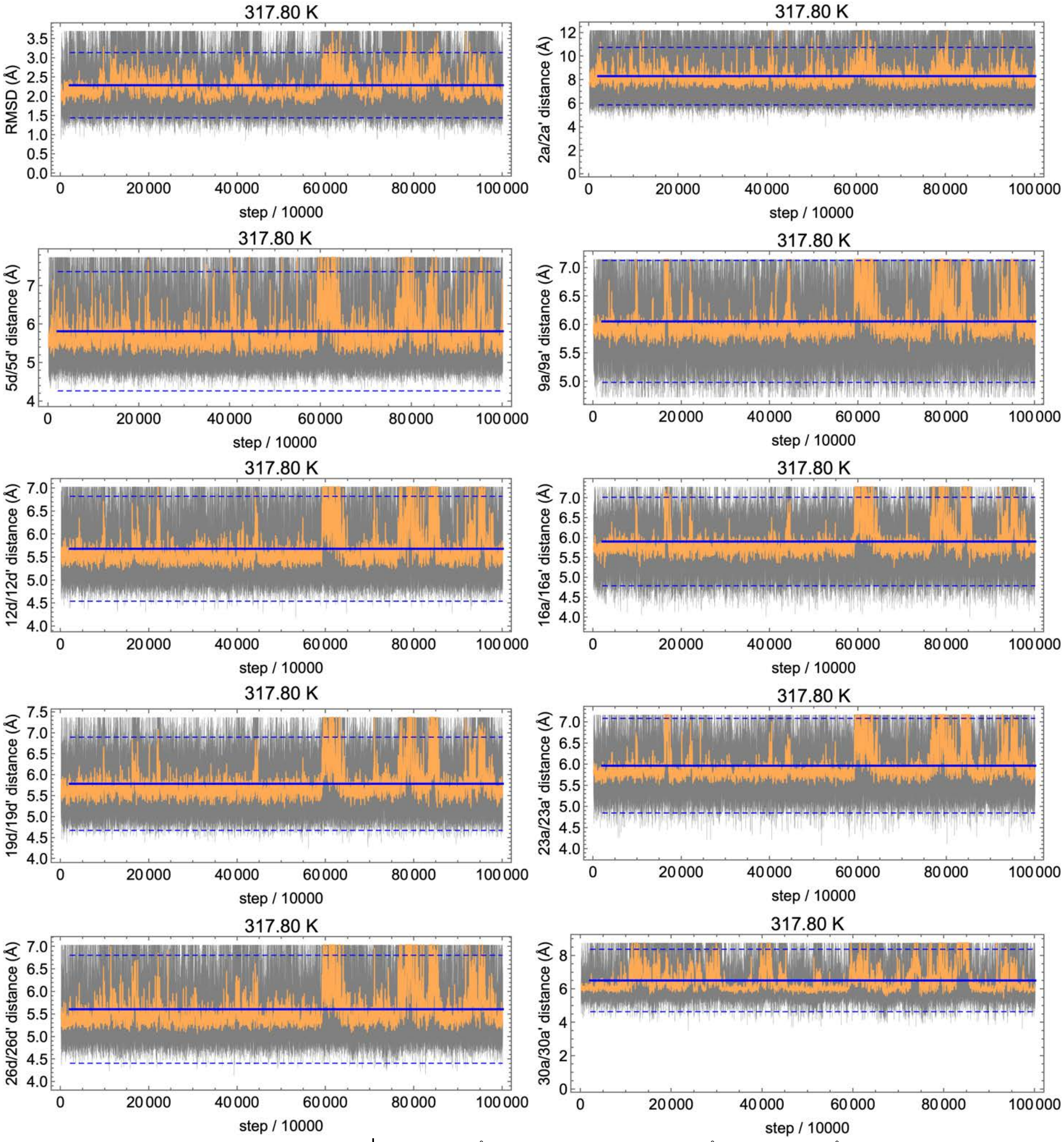

Measure at 317.80 K	Average (Å)	Standard Deviation (Å)	Slope (Å / μs)
RMSD	2.3039	0.849047	0.0675076
5d/5d' distance	5.82951	1.55034	0.189872
12d/12d' distance	5.69336	1.14001	0.189785
19d/19d' distance	5.79916	1.11252	0.18631
26d/26d' distance	5.61913	1.19838	0.198168
2a/2a' distance	8.34948	2.44332	0.104426
9a/9a' distance	6.06386	1.07196	0.15468
16a/16a' distance	5.91628	1.11347	0.166299
23a/23a' distance	5.97972	1.11987	0.178224
30a/30a' distance	6.54038	1.86806	0.104848
Core Distance	6.19899	1.67515	

Figure S420. Plots of RMSD and residue packing distances for T-REMD simulations of variant **28f/31b'-xx** at 317.80 K.

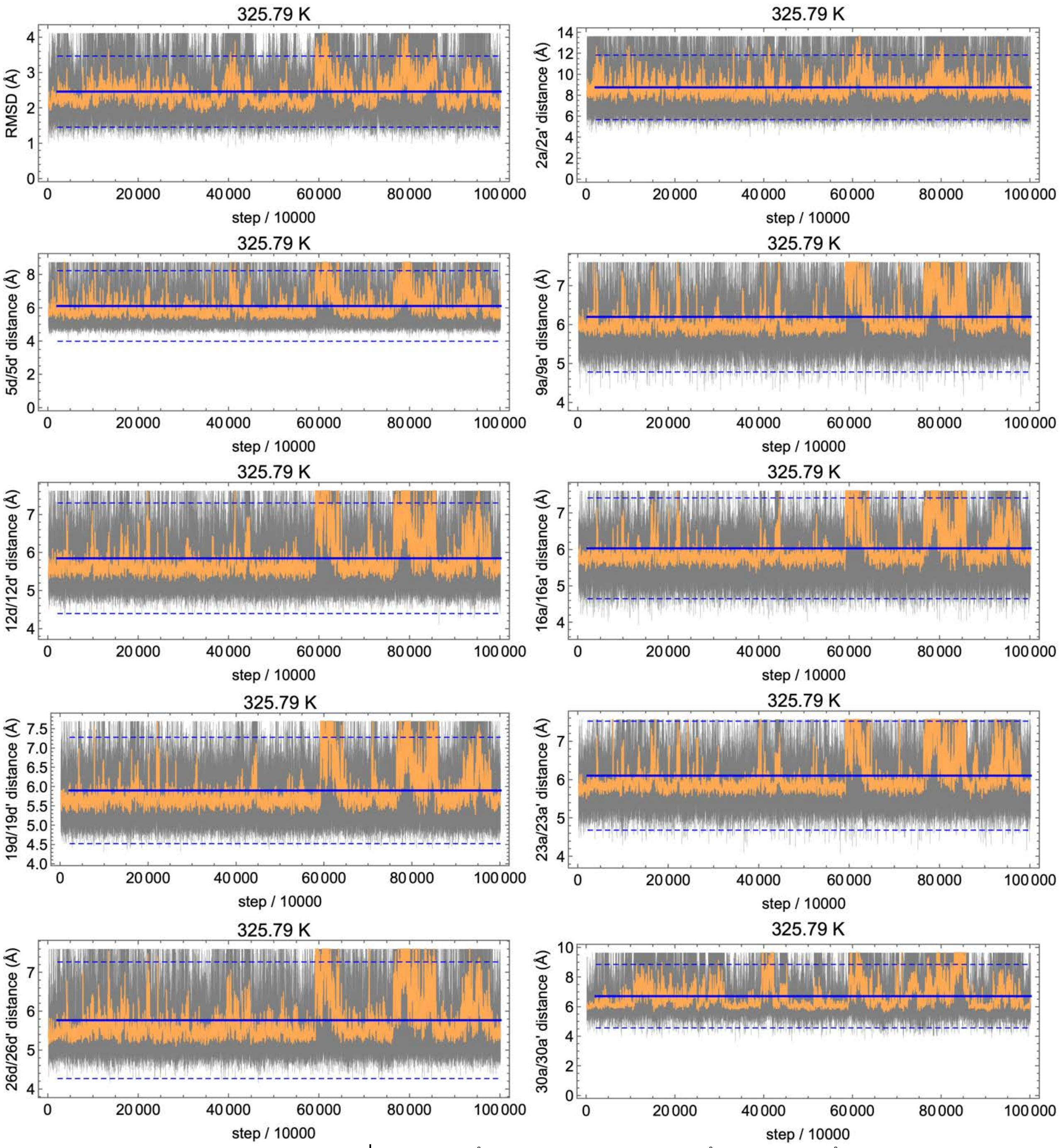

Measure at 325.79 K	Average (Å)	Standard Deviation (Å)	Slope (Å / μs)
RMSD	2.48045	1.00552	0.117997
5d/5d' distance	6.15051	2.12156	0.281026
12d/12d' distance	5.86548	1.45314	0.293373
19d/19d' distance	5.91757	1.37656	0.291116
26d/26d' distance	5.78565	1.5002	0.30558
2a/2a' distance	8.81555	3.07826	0.144954
9a/9a' distance	6.21766	1.4187	0.256004
16a/16a' distance	6.04993	1.38403	0.278599
23a/23a' distance	6.11952	1.41986	0.291575
30a/30a' distance	6.75466	2.14547	0.196927
Core Distance	6.4085	2.05301	

Figure S421. Plots of RMSD and residue packing distances for T-REMD simulations of variant **28f/31b'-xx** at 325.79 K.

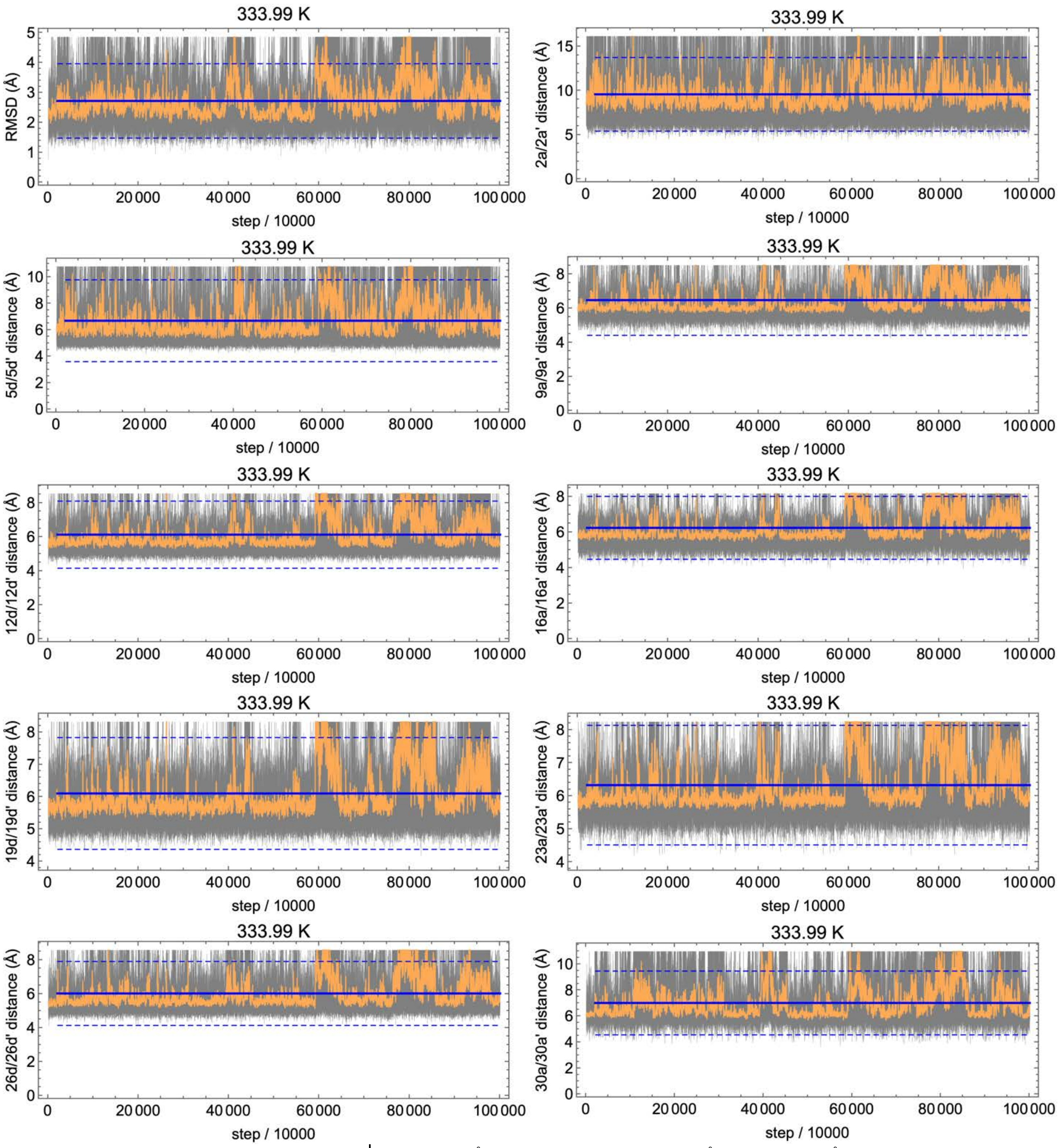

Measure at 333.99 K	Average (Å)	Standard Deviation (Å)	Slope (Å / μs)
RMSD	2.73204	1.24107	0.211782
5d/5d' distance	6.72081	3.09267	0.444248
12d/12d' distance	6.15272	1.97354	0.482145
19d/19d' distance	6.11479	1.73062	0.462732
26d/26d' distance	6.04389	1.88527	0.496054
2a/2a' distance	9.6161	4.16162	0.289976
9a/9a' distance	6.49801	2.06288	0.429392
16a/16a' distance	6.2652	1.7667	0.46284
23a/23a' distance	6.33696	1.80927	0.487073
30a/30a' distance	7.04136	2.45213	0.353509
Core Distance	6.75443	2.66705	

Figure S422. Plots of RMSD and residue packing distances for T-REMD simulations of variant **28f/31b'-xx** at 333.99 K.

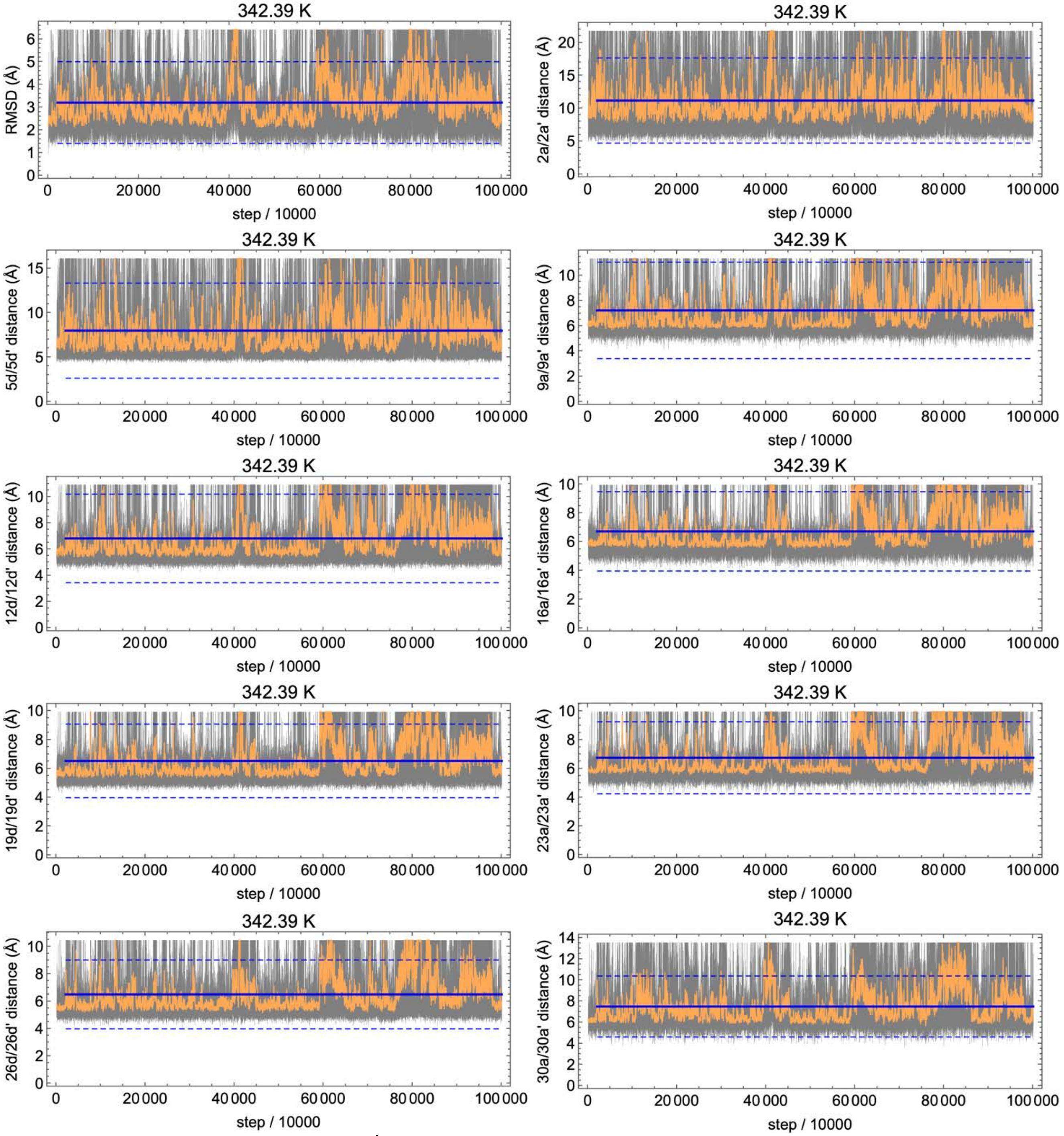

Measure at 342.39 K	Average (Å)	Standard Deviation (Å)	Slope (Å / μs)
RMSD	3.22465	1.79667	0.386442
5d/5d' distance	8.02991	5.3468	0.855674
12d/12d' distance	6.85005	3.37951	0.866579
19d/19d' distance	6.55525	2.55341	0.775772
26d/26d' distance	6.53084	2.51393	0.791016
2a/2a' distance	11.2431	6.46909	0.604
9a/9a' distance	7.25809	3.82601	0.811032
16a/16a' distance	6.7562	2.75692	0.793039
23a/23a' distance	6.77808	2.50386	0.801721
30a/30a' distance	7.53559	2.88413	0.586697
Core Distance	7.50412	4.07163	

Figure S423. Plots of RMSD and residue packing distances for T-REMD simulations of variant **28f/31b'-xx** at 342.39 K.

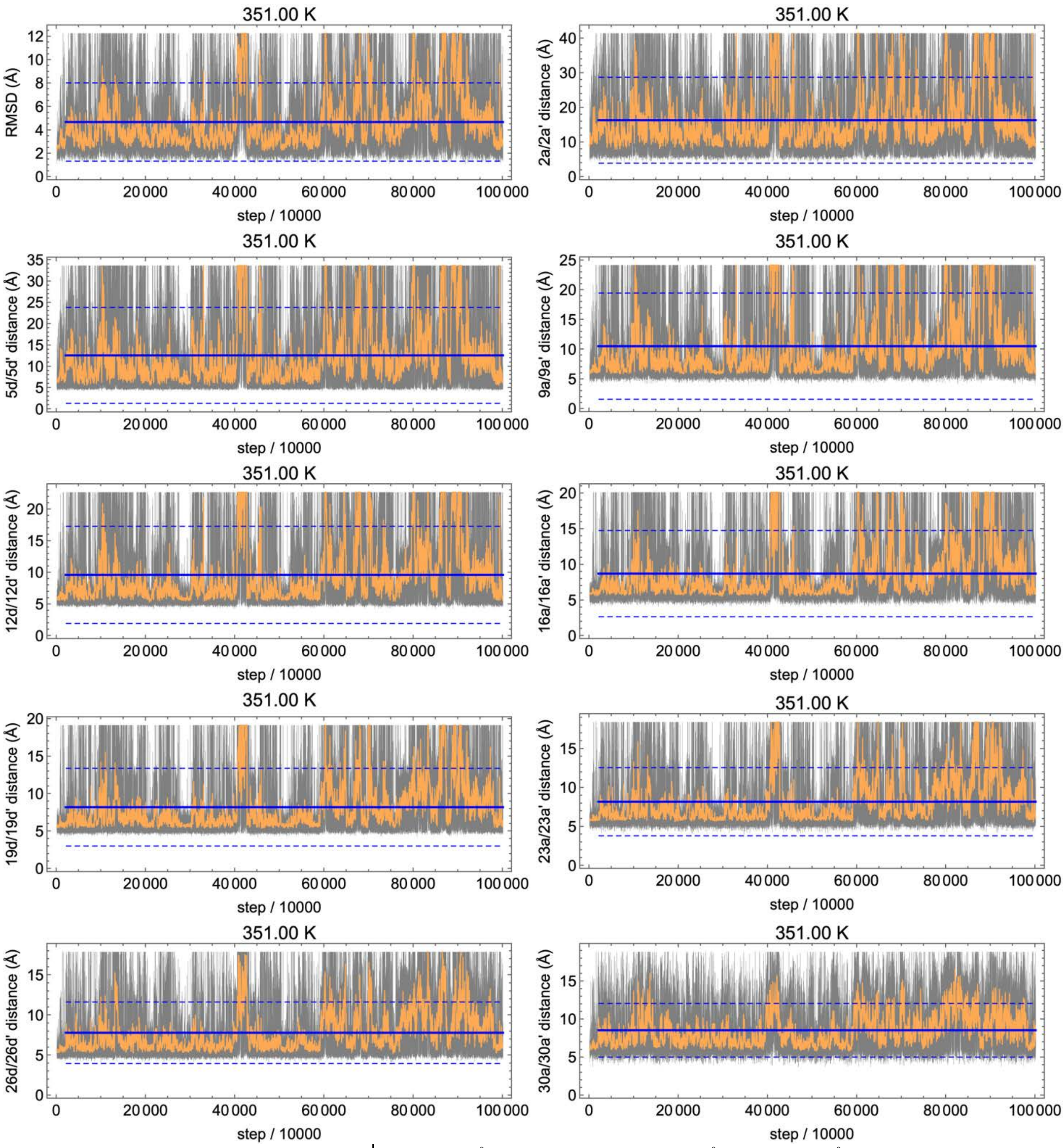

Measure at 351.00 K	Average (Å)	Standard Deviation (Å)	Slope (Å / μs)
RMSD	4.71932	3.34588	1.27543
5d/5d' distance	12.6997	11.2498	3.65025
12d/12d' distance	9.69547	7.67662	2.9403
19d/19d' distance	8.26668	5.18122	2.10183
26d/26d' distance	7.85783	3.83732	1.67708
2a/2a' distance	16.4663	12.4011	3.41109
9a/9a' distance	10.6016	8.94121	3.18303
16a/16a' distance	8.78636	6.04983	2.36749
23a/23a' distance	8.24757	4.37429	1.95131
30a/30a' distance	8.61084	3.52149	1.18054
Core Distance	10.1369	8.11293	

Figure S424. Plots of RMSD and residue packing distances for T-REMD simulations of variant **28f/31b'-xx** at 351.00 K.

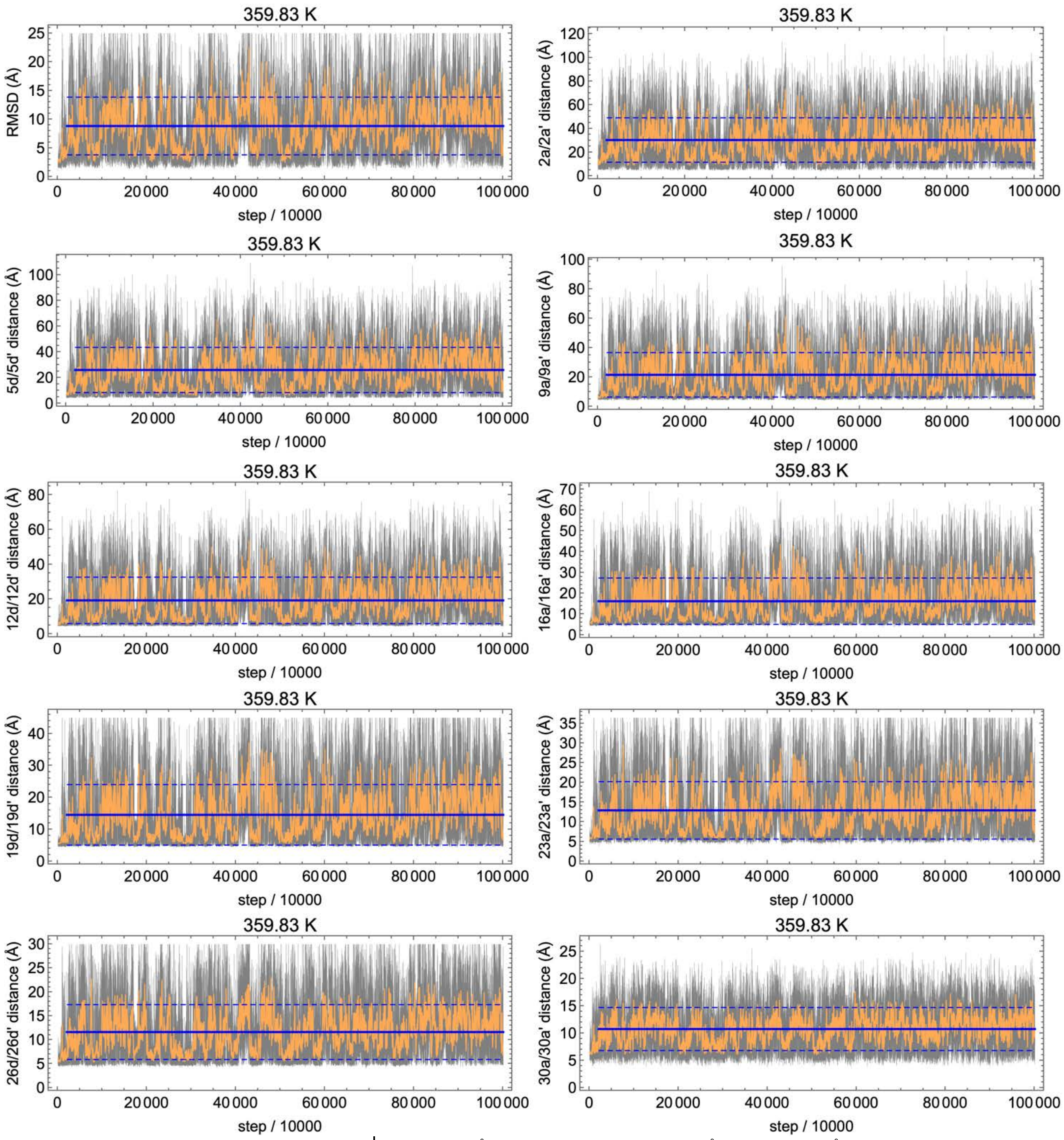

Measure at 359.83 K	Average (Å)	Standard Deviation (Å)	Slope (Å / μs)
RMSD	8.89527	5.03006	1.53724
5d/5d' distance	26.294	17.5996	4.92713
12d/12d' distance	19.4822	13.3703	4.00216
19d/19d' distance	14.6671	9.49412	2.90277
26d/26d' distance	11.6983	5.73279	1.68169
2a/2a' distance	30.6827	18.7529	4.81943
9a/9a' distance	21.8505	15.1233	4.41797
16a/16a' distance	16.3691	11.1593	3.34631
23a/23a' distance	13.0261	7.29892	2.21855
30a/30a' distance	10.8355	3.95439	0.934946
Core Distance	18.3228	13.9865	

Figure S425. Plots of RMSD and residue packing distances for T-REMD simulations of variant **28f/31b'-xx** at 359.83 K.

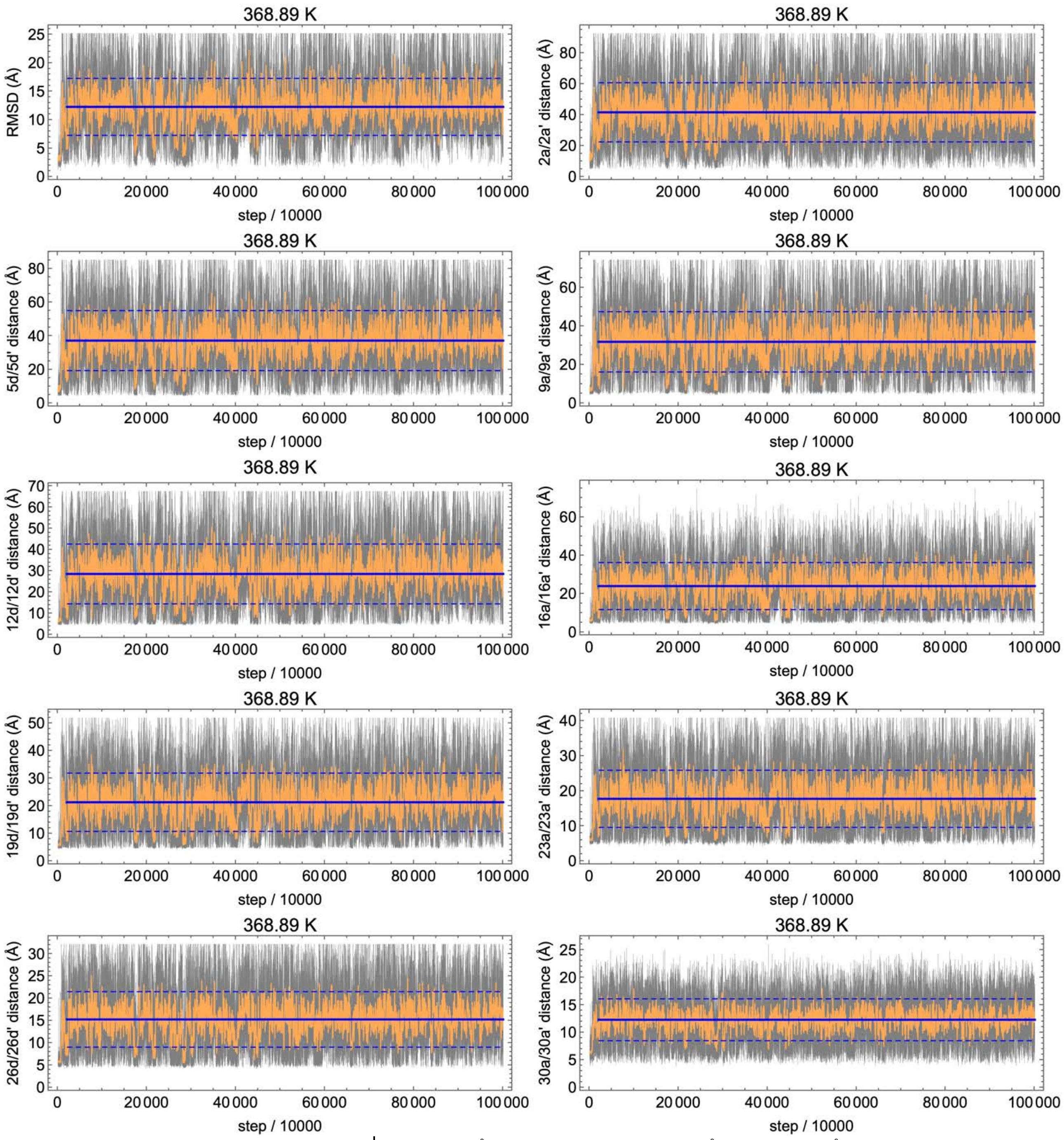

Measure at 368.89 K	Average (Å)	Standard Deviation (Å)	Slope (Å / μs)
RMSD	12.3429	5.00715	0.567714
5d/5d' distance	37.4132	17.8416	1.97489
12d/12d' distance	28.746	14.1022	1.77118
19d/19d' distance	21.4447	10.5693	1.35459
26d/26d' distance	15.365	6.22987	0.594475
2a/2a' distance	41.8715	19.1169	1.77446
9a/9a' distance	32.0338	15.647	1.99996
16a/16a' distance	24.1929	12.2637	1.52463
23a/23a' distance	17.86	8.15858	0.849742
30a/30a' distance	12.3789	3.80602	0.172181
Core Distance	25.7007	16.0681	

Figure S426. Plots of RMSD and residue packing distances for T-REMD simulations of variant **28f/31b'-xx** at 368.89 K.

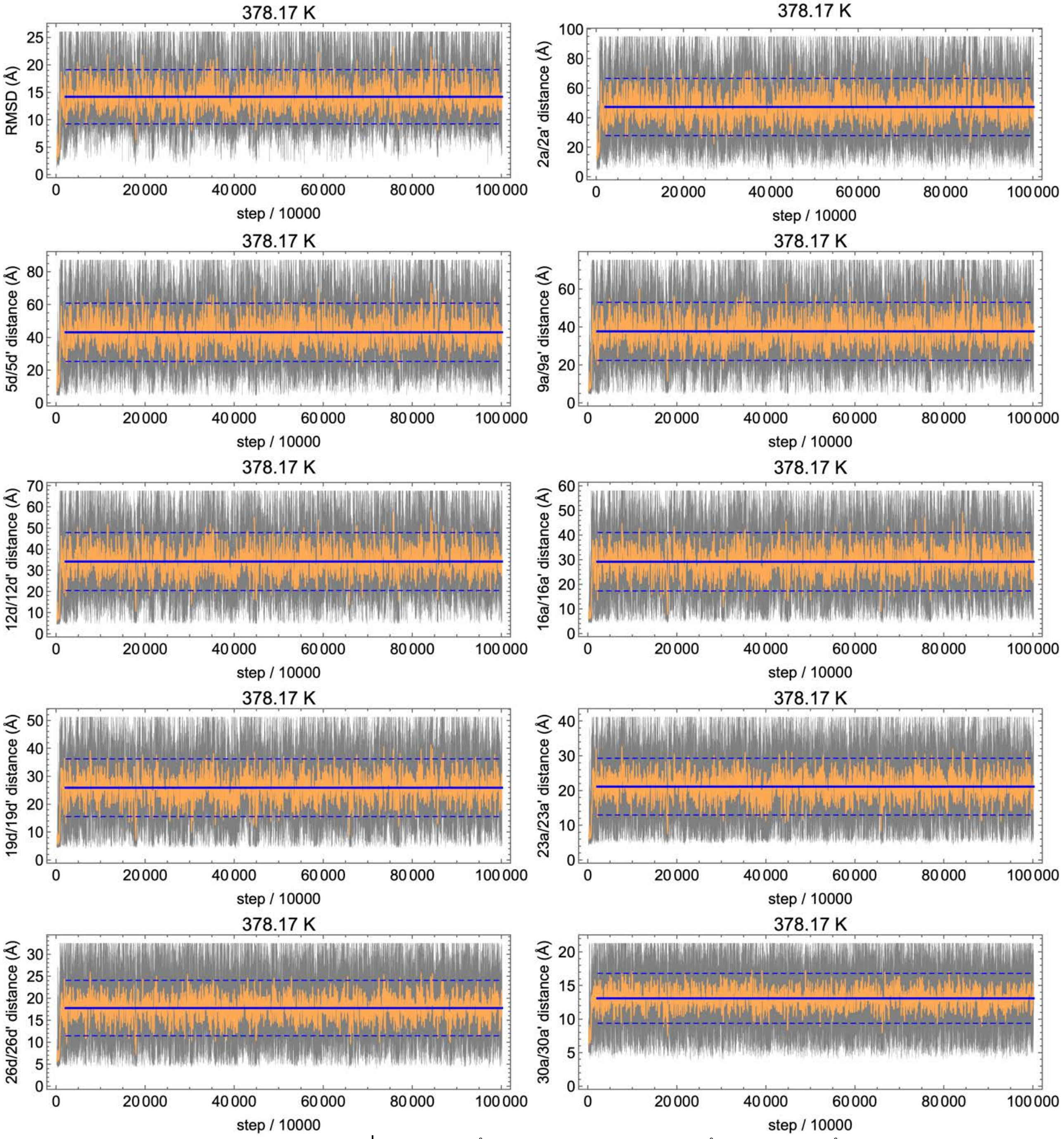

Measure at 378.17 K	Average (Å)	Standard Deviation (Å)	Slope (Å / μs)
RMSD	14.3022	4.93721	0.0733149
5d/5d' distance	43.4829	17.781	0.342605
12d/12d' distance	34.4558	13.6912	0.392652
19d/19d' distance	26.1205	10.332	0.371902
26d/26d' distance	17.9129	6.30458	0.0863956
2a/2a' distance	47.7185	19.3668	0.107264
9a/9a' distance	37.9995	15.2814	0.403121
16a/16a' distance	29.4498	11.8755	0.416546
23a/23a' distance	21.3144	8.17155	0.116781
30a/30a' distance	13.1886	3.71714	−0.0625322
Core Distance	30.1825	16.9283	

Figure S427. Plots of RMSD and residue packing distances for T-REMD simulations of variant **28f/31b'-xx** at 378.17 K.

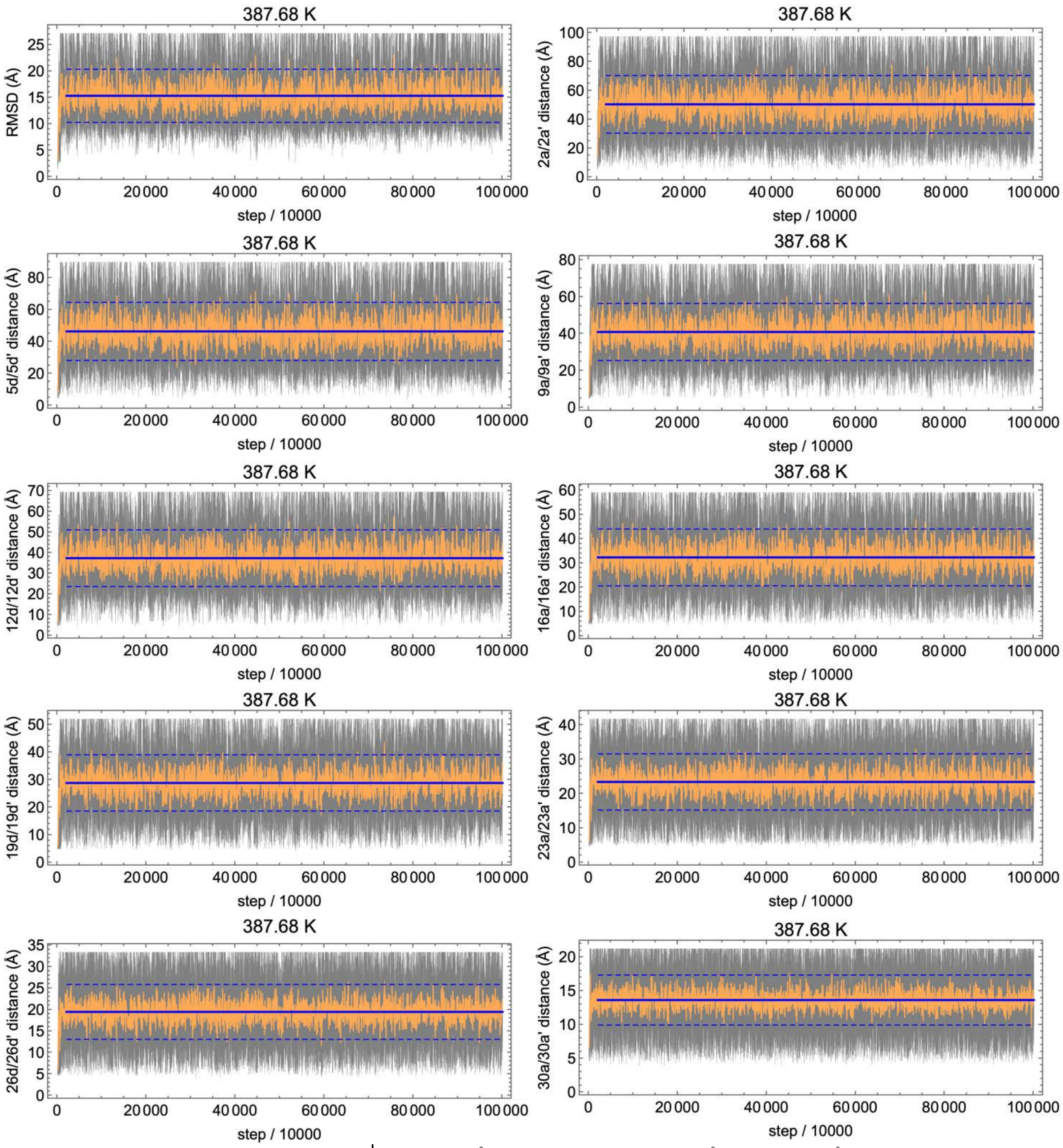

Measure at 387.68 K	Average (Å)	Standard Deviation (Å)	Slope (Å / μs)
RMSD	15.3934	5.03371	0.0121637
5d/5d' distance	46.5791	18.1885	0.0500092
12d/12d' distance	37.5723	13.7298	0.191572
19d/19d' distance	28.9554	10.2058	0.250054
26d/26d' distance	19.556	6.38271	0.0816406
2a/2a' distance	50.6865	19.9579	−0.155294
9a/9a' distance	41.131	15.4887	0.174771
16a/16a' distance	32.4943	11.7017	0.274663
23a/23a' distance	23.5122	8.18476	0.0945584
30a/30a' distance	13.6925	3.70178	−0.0893538
Core Distance	32.6866	17.5232	

Figure S428. Plots of RMSD and residue packing distances for T-REMD simulations of variant **28f/31b’-xx** at 387.68 K.

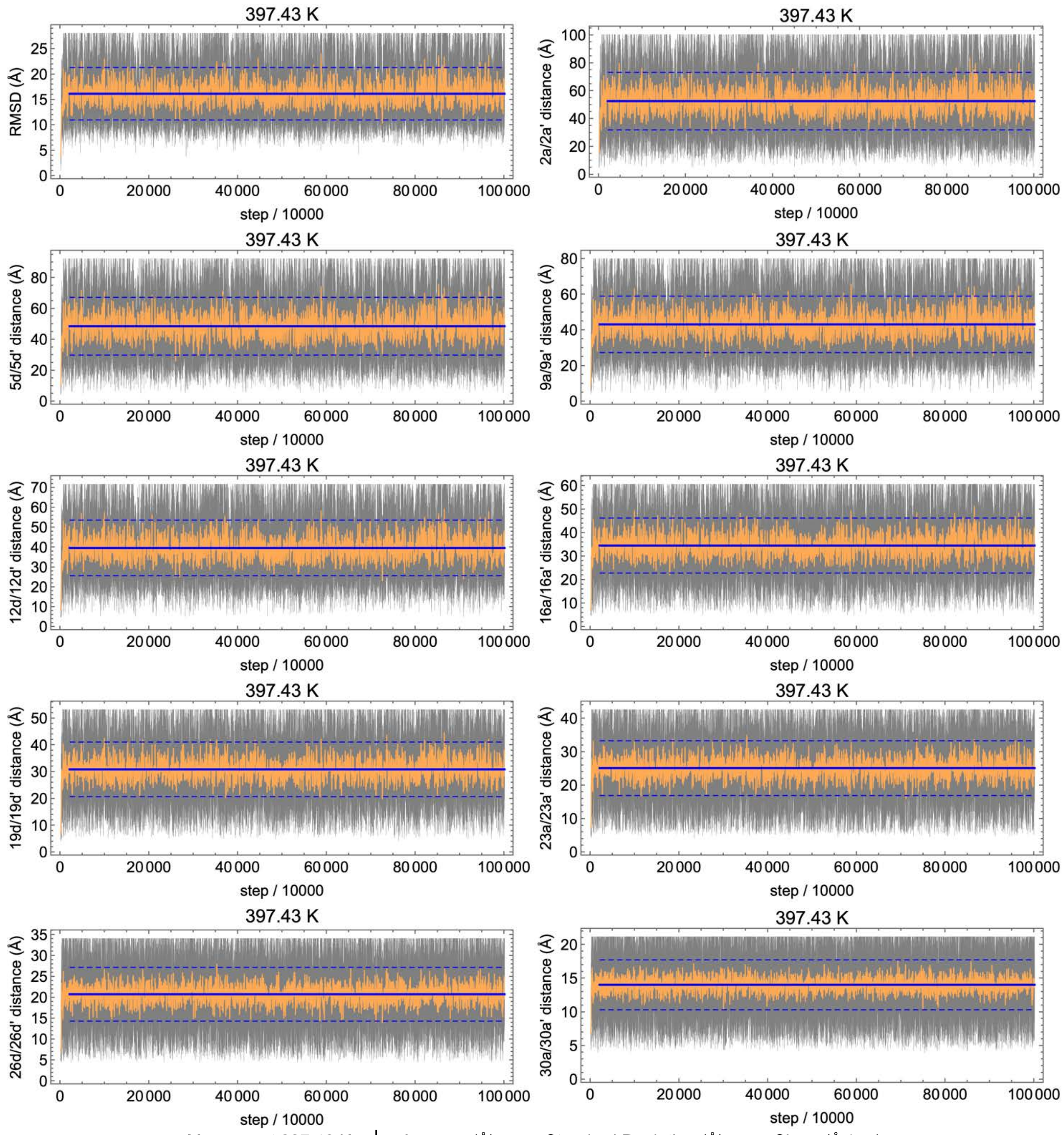

Measure at 397.43 K	Average (Å)	Standard Deviation (Å)	Slope (Å / μs)
RMSD	16.2385	5.15707	0.0399033
5d/5d' distance	48.8921	18.7021	0.121829
12d/12d' distance	39.8717	13.9911	0.189772
19d/19d' distance	31.0814	10.1854	0.217814
26d/26d' distance	20.8681	6.42218	0.063616
2a/2a' distance	52.9357	20.6131	0.0321818
9a/9a' distance	43.4411	15.8559	0.216445
16a/16a' distance	34.7377	11.7054	0.227631
23a/23a' distance	25.254	8.18362	0.0864292
30a/30a' distance	14.0983	3.70466	−0.0245729
Core Distance	34.5756	18.0726	

Figure S429. Plots of RMSD and residue packing distances for T-REMD simulations of variant **28f/31b'-xx** at 397.43 K.

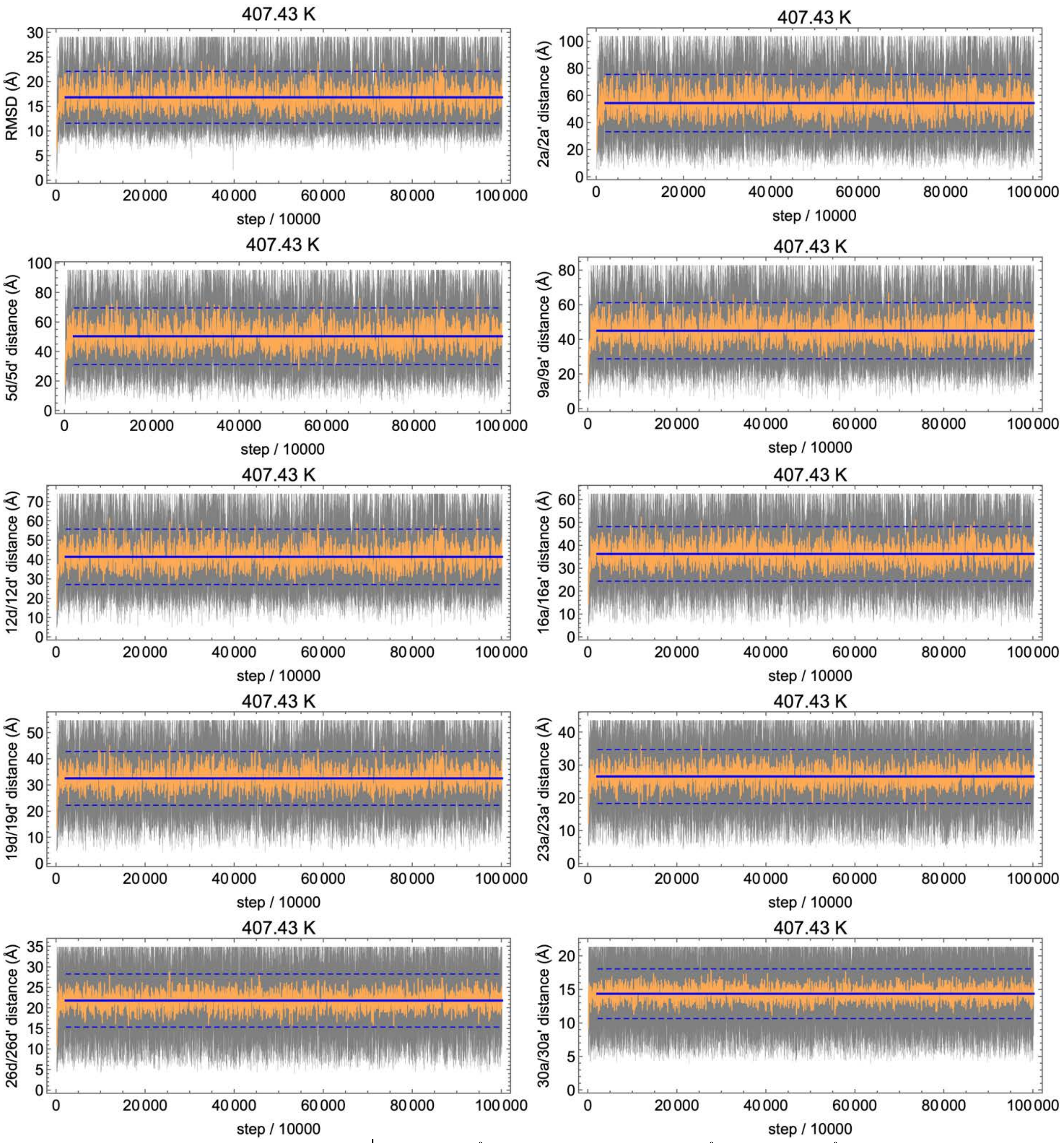

Measure at 407.43 K	Average (Å)	Standard Deviation (Å)	Slope (Å / μs)
RMSD	16.9633	5.27285	−0.0107307
5d/5d' distance	50.7976	19.1902	−0.0448702
12d/12d' distance	41.744	14.318	−0.00931965
19d/19d' distance	32.7953	10.2734	0.0887348
26d/26d' distance	21.9603	6.45393	0.0472332
2a/2a' distance	54.8169	21.1586	−0.0343745
9a/9a' distance	45.3276	16.222	0.00884953
16a/16a' distance	36.5333	11.9085	0.0344812
23a/23a' distance	26.7031	8.22618	0.0509068
30a/30a' distance	14.4601	3.70377	−0.0143889
Core Distance	36.1265	18.5755	

Figure S430. Plots of RMSD and residue packing distances for T-REMD simulations of variant **28f/31b'-xx** at 407.43 K.

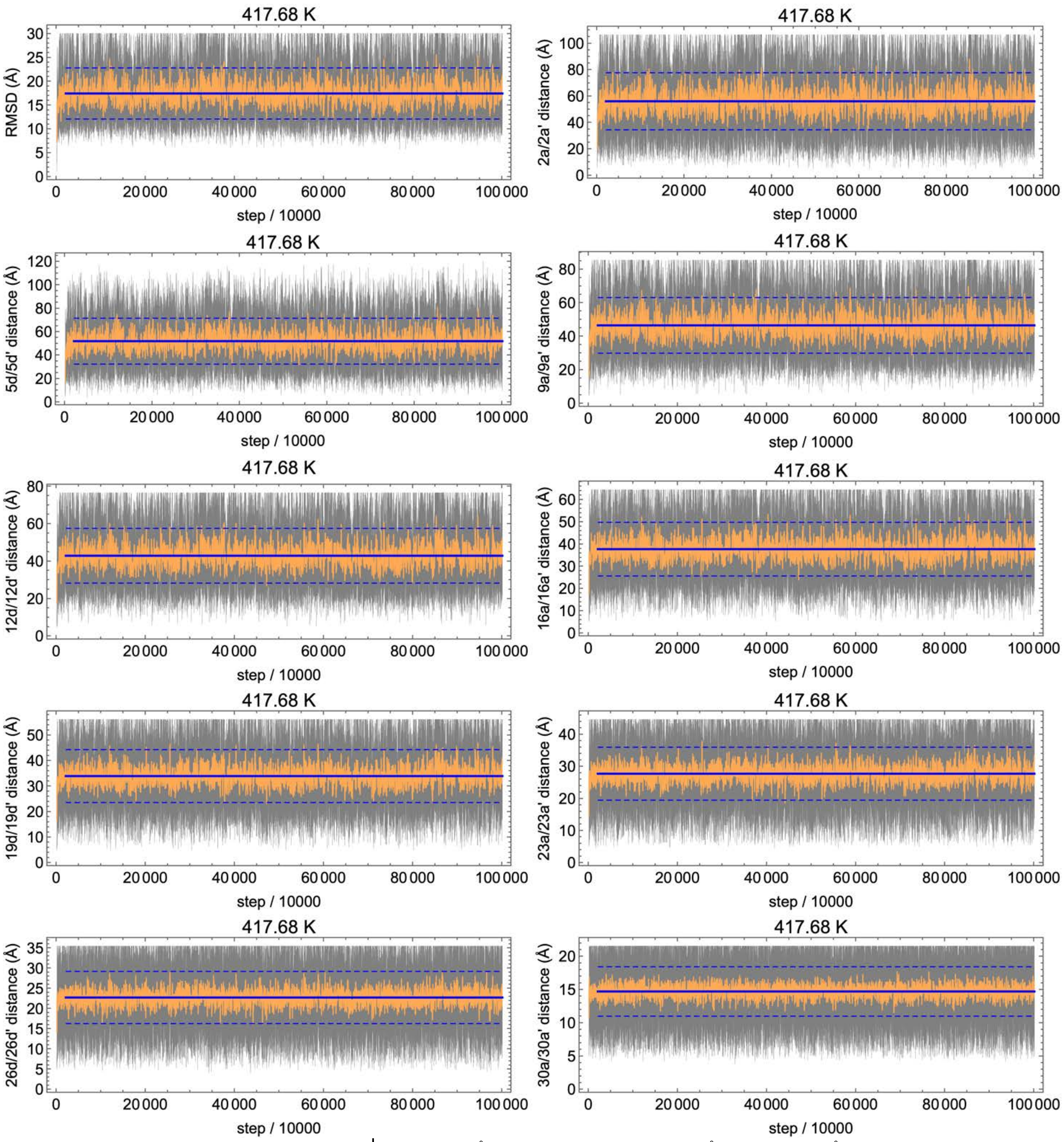

Measure at 417.68 K	Average (Å)	Standard Deviation (Å)	Slope (Å / μs)
RMSD	17.5612	5.35851	0.104835
5d/5d' distance	52.3772	19.6002	0.265501
12d/12d' distance	43.1885	14.62	0.189499
19d/19d' distance	34.1544	10.3682	0.265153
26d/26d' distance	22.8655	6.4576	0.134986
2a/2a' distance	56.4861	21.6187	0.340537
9a/9a' distance	46.8039	16.5964	0.250653
16a/16a' distance	37.974	12.0758	0.187204
23a/23a' distance	27.8935	8.24804	0.170169
30a/30a' distance	14.796	3.71484	−0.0129167
Core Distance	37.3932	19.0074	

Figure S431. Plots of RMSD and residue packing distances for T-REMD simulations of variant **28f/31b'-xx** at 417.68 K.

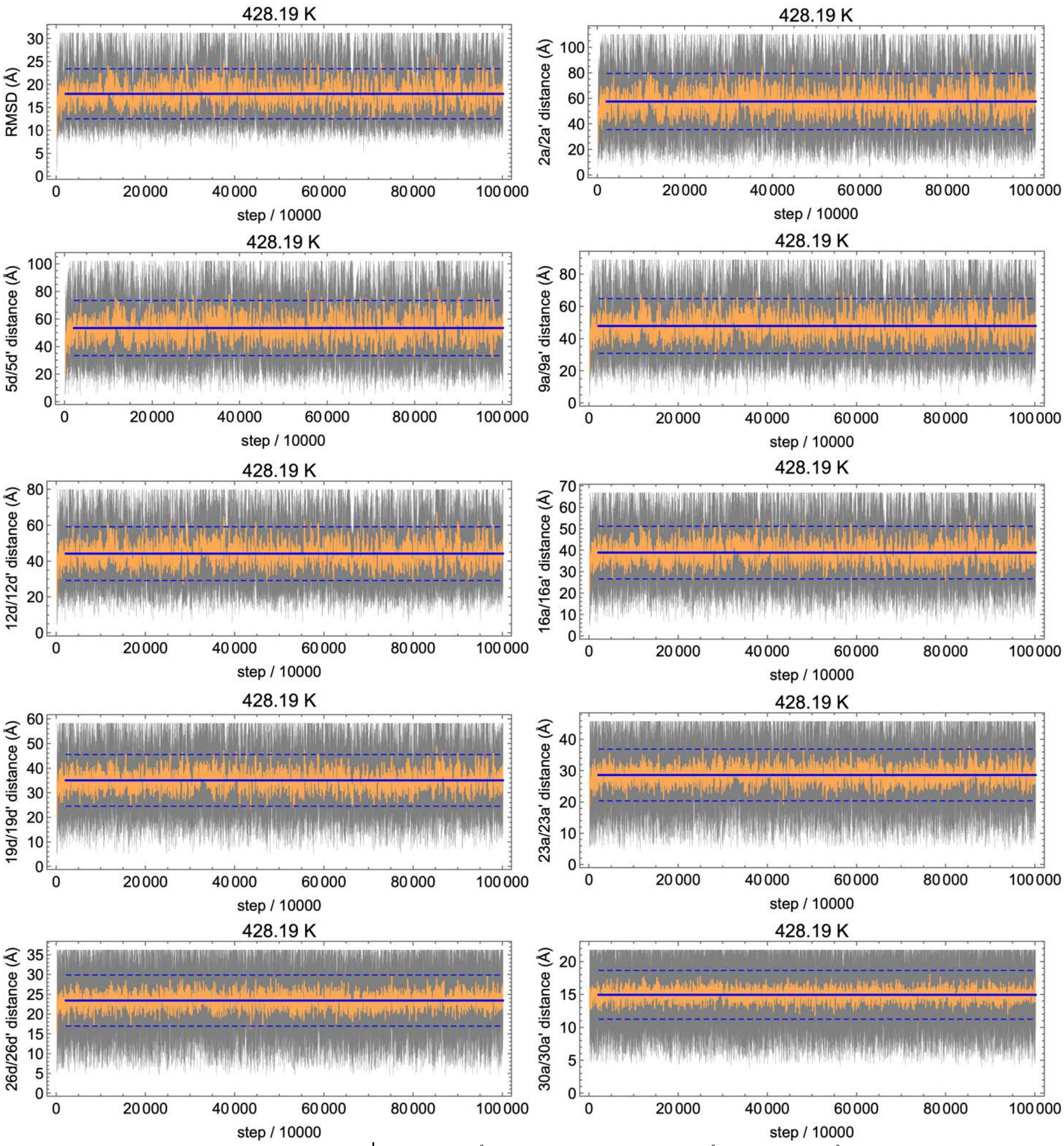

Measure at 428.19 K	Average (Å)	Standard Deviation (Å)	Slope (Å / μs)
RMSD	18.0993	5.4502	0.0657401
5d/5d' distance	53.8586	20.0057	0.202494
12d/12d' distance	44.5047	14.966	0.0518151
19d/19d' distance	35.2956	10.5008	0.138439
26d/26d' distance	23.5868	6.45627	0.156374
2a/2a' distance	57.998	22.0598	0.279341
9a/9a' distance	48.2194	16.98	0.133754
16a/16a' distance	39.2051	12.3035	0.0808253
23a/23a' distance	28.8379	8.27115	0.160678
30a/30a' distance	15.0478	3.70203	0.00919787
Core Distance	38.506	19.4585	

Figure S432. Plots of RMSD and residue packing distances for T-REMD simulations of variant **28f/31b'-xx** at 428.19 K.

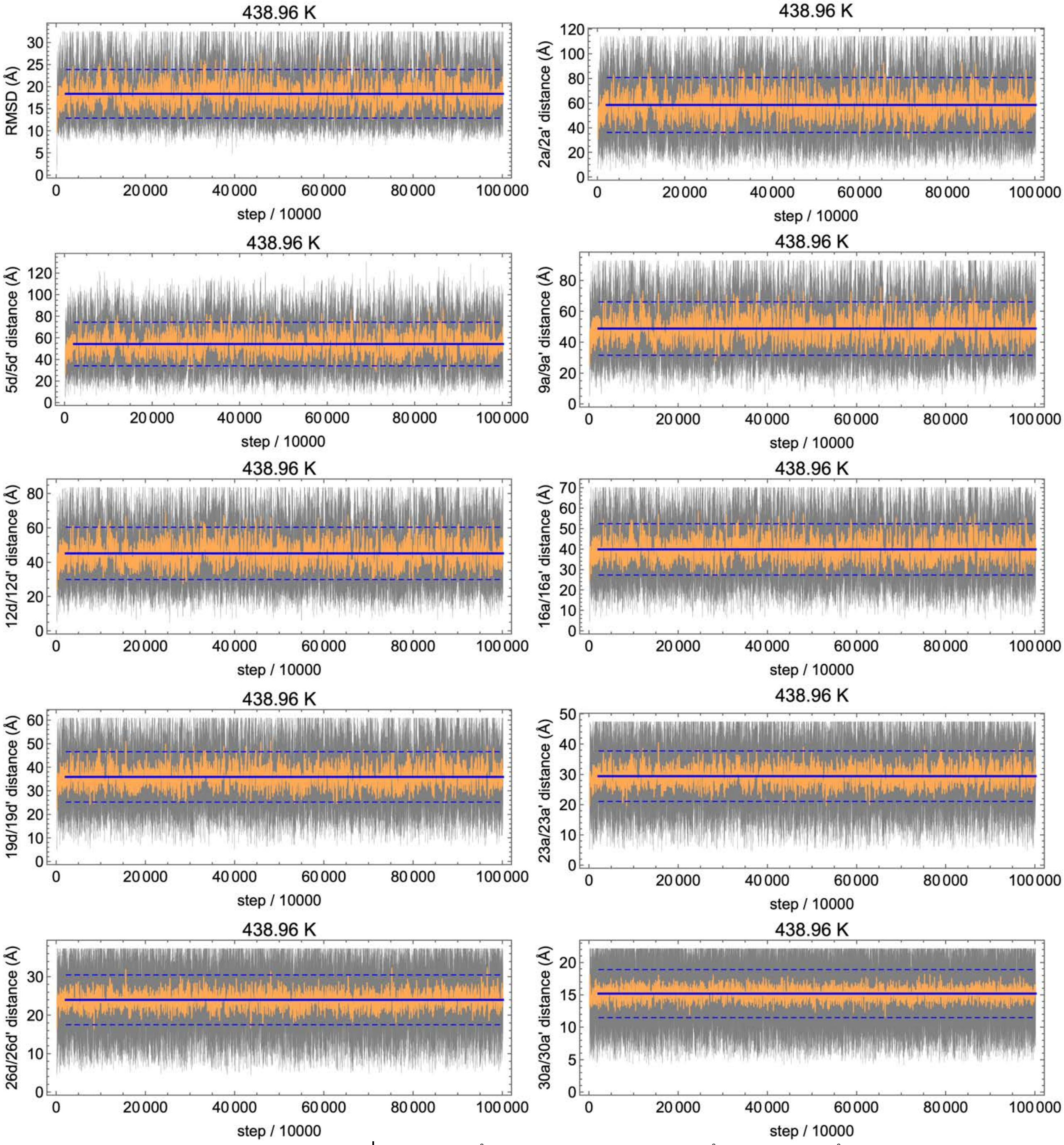

Measure at 438.96 K	Average (Å)	Standard Deviation (Å)	Slope (Å / μs)
RMSD	18.5385	5.49968	0.10003
5d/5d' distance	54.9145	20.2516	0.308978
12d/12d' distance	45.5239	15.239	0.16379
19d/19d' distance	36.2013	10.6903	0.138681
26d/26d' distance	24.1711	6.48282	0.153063
2a/2a' distance	59.0773	22.2786	0.327963
9a/9a' distance	49.2804	17.2788	0.24168
16a/16a' distance	40.1785	12.5404	0.159339
23a/23a' distance	29.6242	8.34461	0.119062
30a/30a' distance	15.2918	3.71046	−0.0395556
Core Distance	39.3626	19.7674	

Figure S433. Plots of RMSD and residue packing distances for T-REMD simulations of variant **28f/31b'-xx** at 438.96 K.

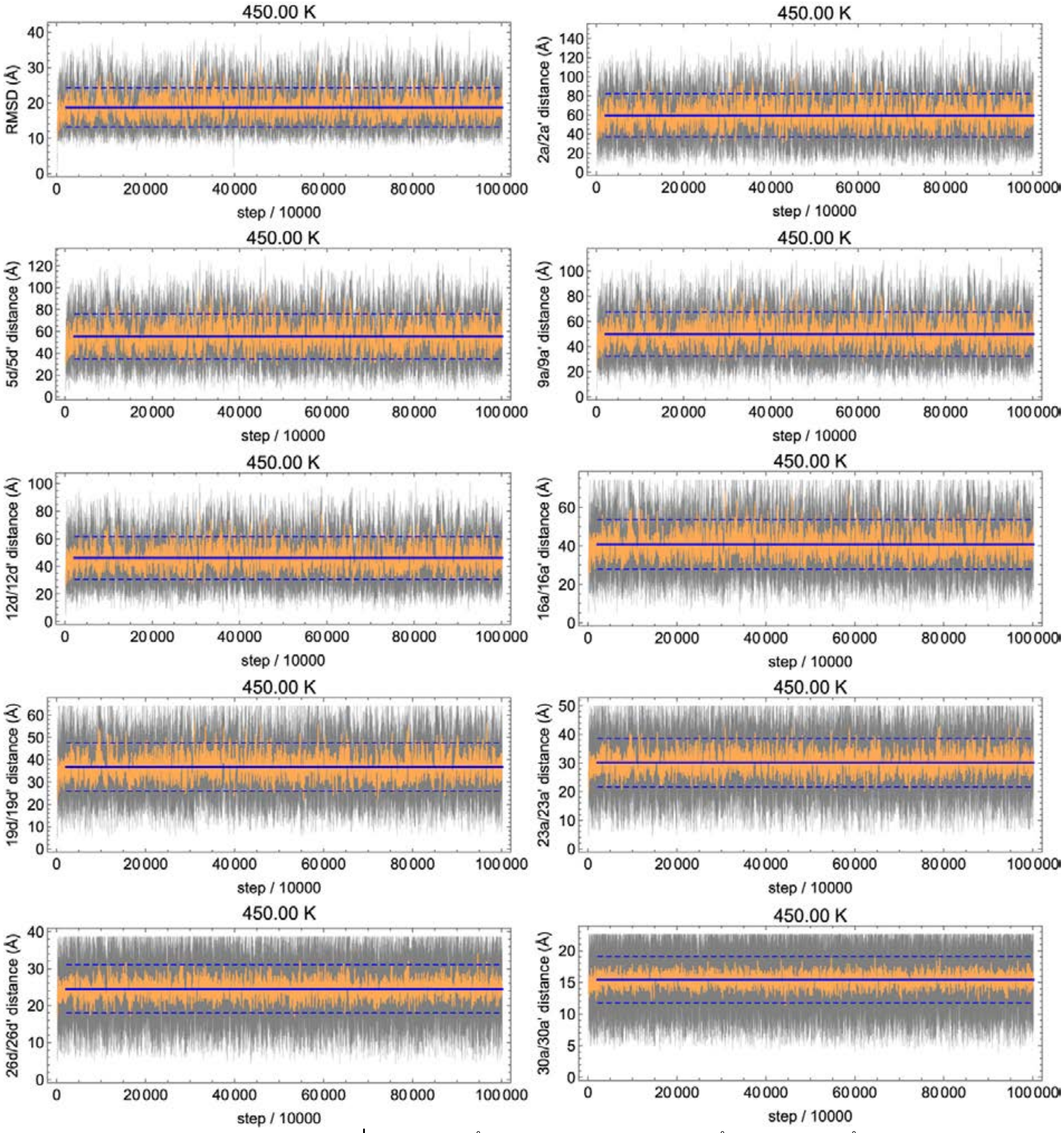

Measure at 450.00 K	Average (Å)	Standard Deviation (Å)	Slope (Å / μs)
RMSD	18.9609	5.55747	0.0598826
5d/5d' distance	56.0057	20.5312	0.19479
12d/12d' distance	46.5607	15.4931	0.0118977
19d/19d' distance	37.0534	10.8736	0.143287
26d/26d' distance	24.7166	6.52626	0.187286
2a/2a' distance	60.2678	22.5412	0.243035
9a/9a' distance	50.365	17.5265	0.10634
16a/16a' distance	41.1251	12.7832	0.107087
23a/23a' distance	30.3817	8.48419	0.154386
30a/30a' distance	15.5271	3.69944	0.0348893
Core Distance	40.2226	20.1072	

Figure S434. Plots of RMSD and residue packing distances for T-REMD simulations of variant **28f/31b'-xx** at 450.00 K.

(return to Table of Contents)

s28f/31b'-x4x

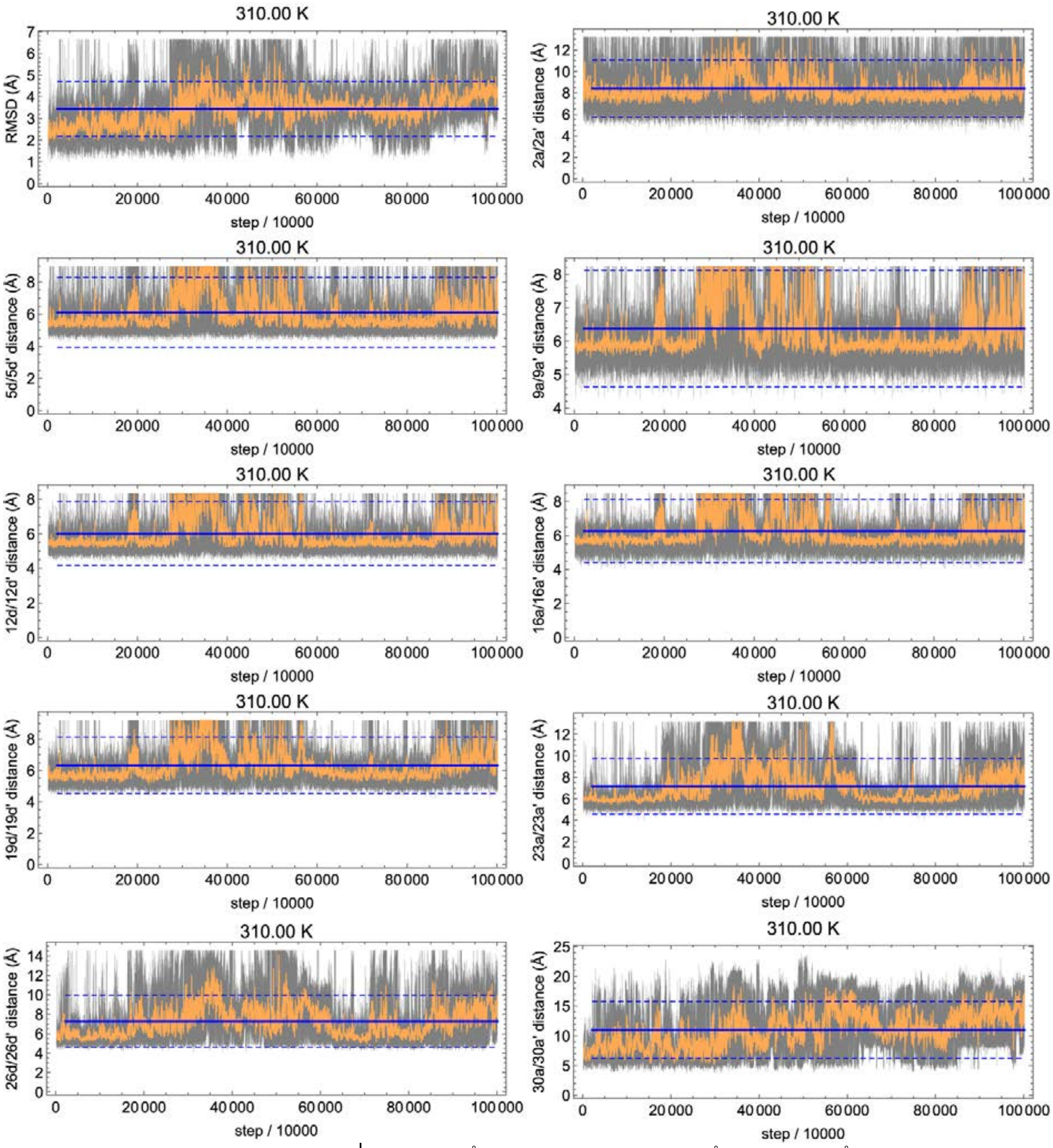

Measure at 310.00 K	Average (Å)	Standard Deviation (Å)	Slope (Å / μs)
RMSD	3.47576	1.25222	0.616604
5d/5d' distance	6.14784	2.17976	−0.0709452
12d/12d' distance	6.05431	1.85537	−0.0503374
19d/19d' distance	6.37902	1.79836	0.0737286
26d/26d' distance	7.3493	2.6574	0.417322
2a/2a' distance	8.47023	2.6623	−0.0877013
9a/9a' distance	6.39423	1.74155	−0.0720884
16a/16a' distance	6.30071	1.8588	−0.0777209
23a/23a' distance	7.24269	2.58441	0.129266
30a/30a' distance	11.1168	4.80336	3.04631
Core Distance	7.27279	3.04229	

Figure S435. Plots of RMSD and residue packing distances for T-REMD simulations of variant **s28f/31b'-x4x** at 310.00 K.

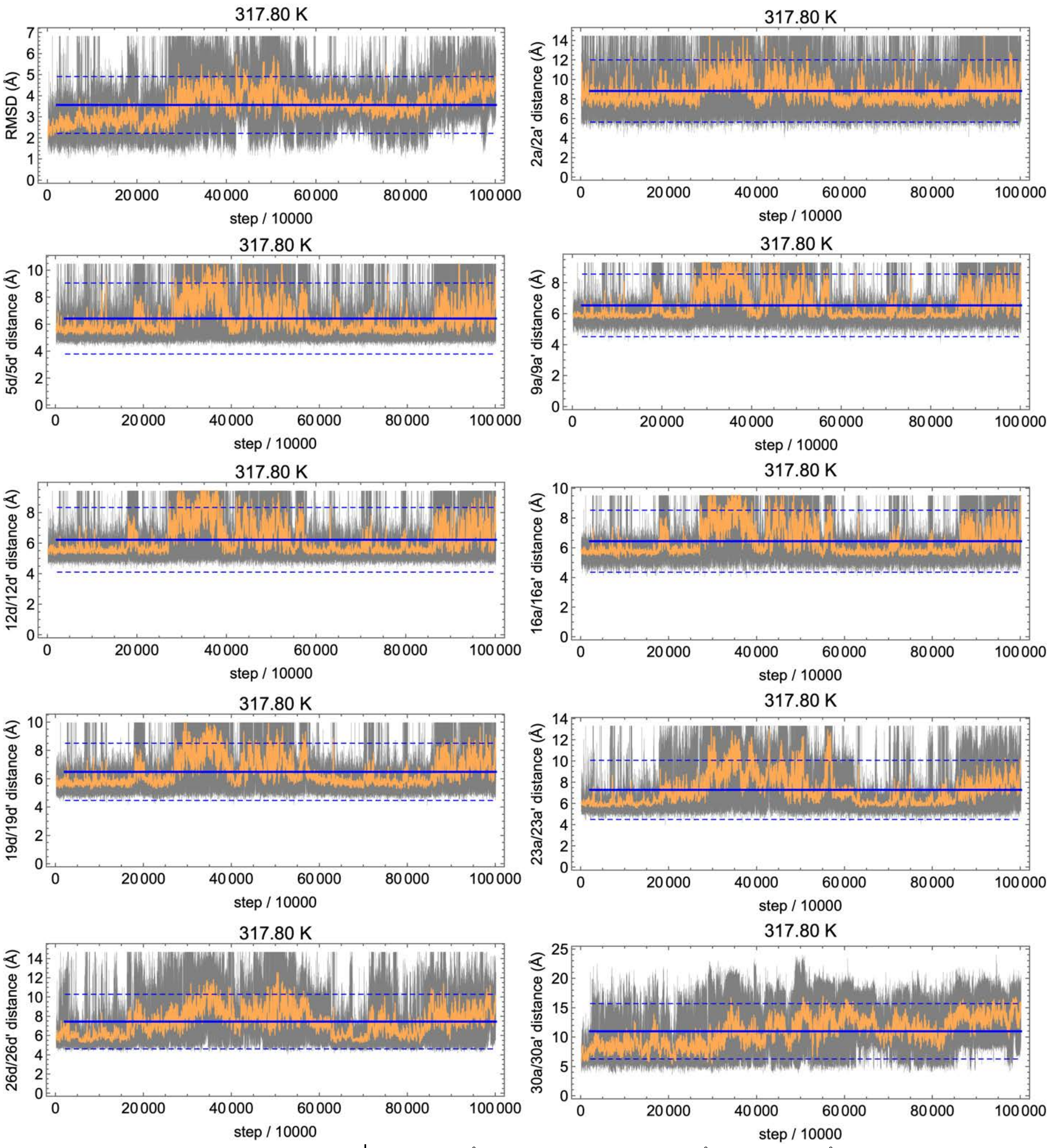

Measure at 317.80 K	Average (Å)	Standard Deviation (Å)	Slope (Å / μs)
RMSD	3.59501	1.34654	0.594247
5d/5d' distance	6.46465	2.63083	0.0465782
12d/12d' distance	6.26107	2.1145	0.0527937
19d/19d' distance	6.53277	2.01945	0.117866
26d/26d' distance	7.51666	2.84004	0.387715
2a/2a' distance	8.89022	3.18537	−0.0021409
9a/9a' distance	6.57689	2.02303	0.0249923
16a/16a' distance	6.48451	2.08812	0.021105
23a/23a' distance	7.34618	2.78857	0.0690809
30a/30a' distance	11.0962	4.71615	2.88007
Core Distance	7.46324	3.20514	

Figure S436. Plots of RMSD and residue packing distances for T-REMD simulations of variant **s28f/31b'-x4x** at 317.80 K.

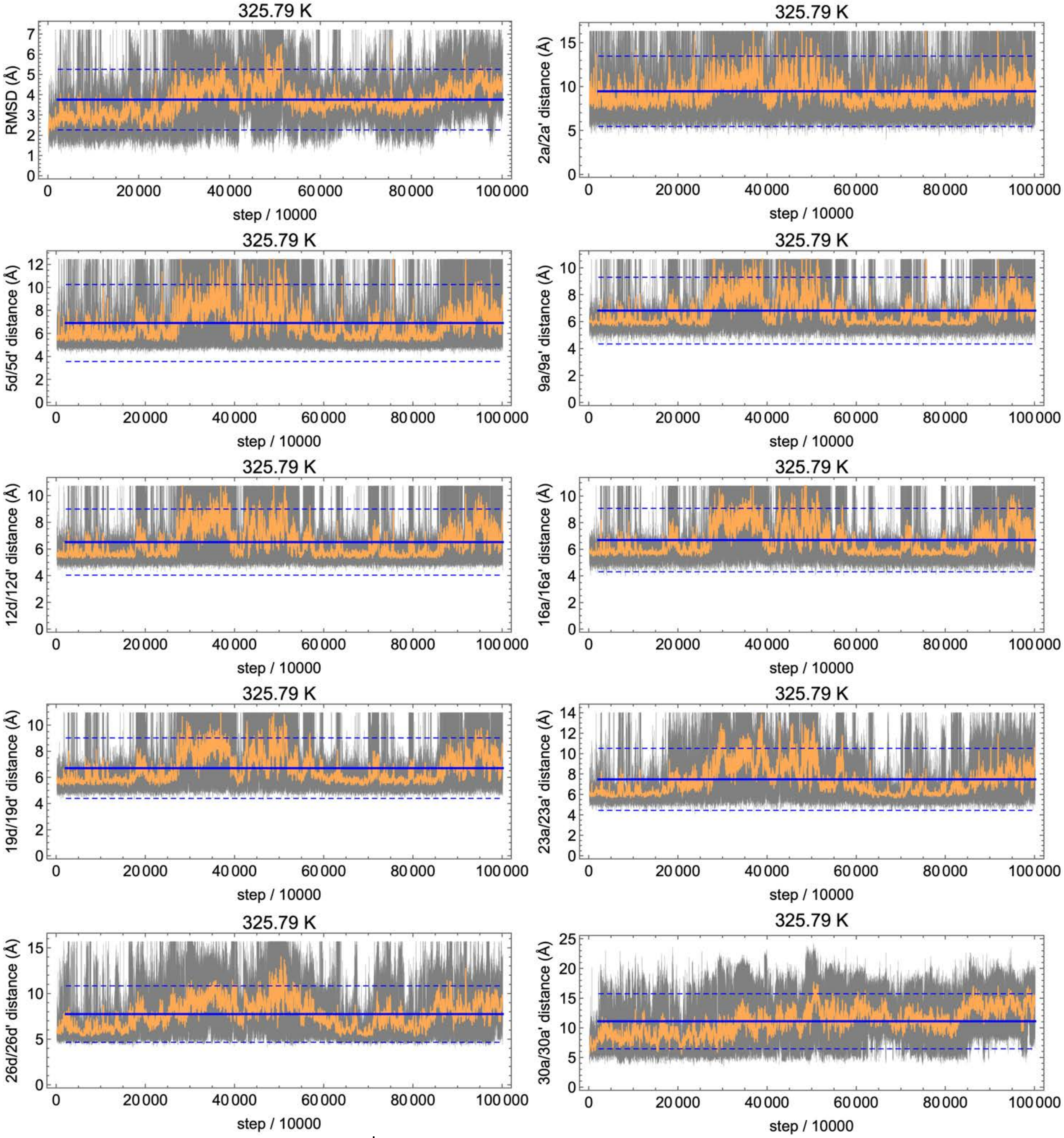

Measure at 325.79 K	Average (Å)	Standard Deviation (Å)	Slope (Å / μs)
RMSD	3.79153	1.49716	0.533927
5d/5d' distance	6.9632	3.34371	0.108275
12d/12d' distance	6.56666	2.47483	0.120224
19d/19d' distance	6.75848	2.31537	0.15213
26d/26d' distance	7.81854	3.08875	0.393442
2a/2a' distance	9.53712	4.01018	0.0224616
9a/9a' distance	6.8691	2.47579	0.0912268
16a/16a' distance	6.7435	2.38476	0.0985954
23a/23a' distance	7.5455	3.03423	0.0183061
30a/30a' distance	11.2125	4.64361	2.67975
Core Distance	7.77941	3.51009	

Figure S437. Plots of RMSD and residue packing distances for T-REMD simulations of variant **s28f/31b'-x4x** at 325.79 K.

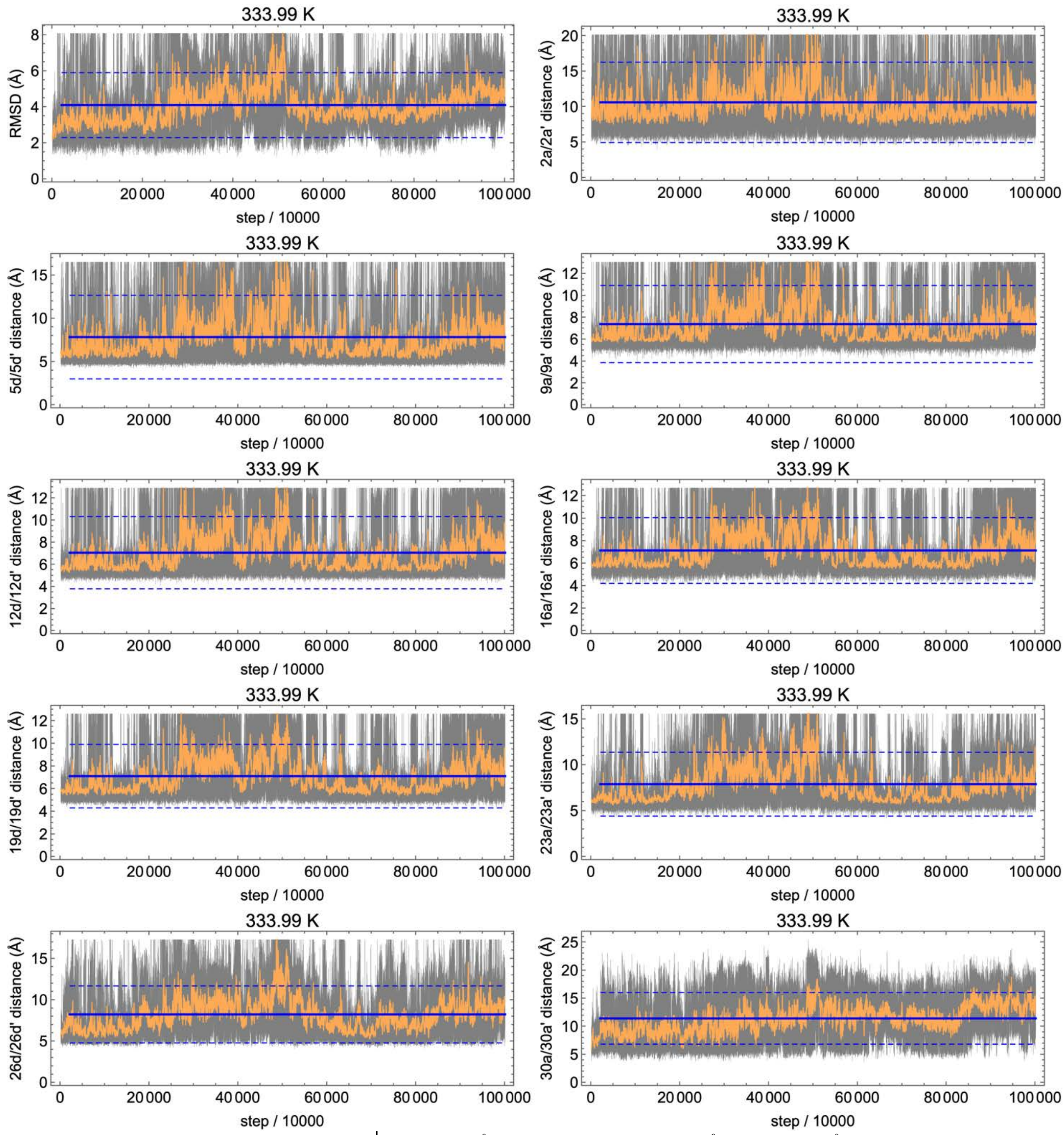

Measure at 333.99 K	Average (Å)	Standard Deviation (Å)	Slope (Å / μs)
RMSD	4.12834	1.8028	0.449512
5d/5d' distance	7.8959	4.83306	0.0675234
12d/12d' distance	7.10409	3.26379	0.116172
19d/19d' distance	7.15153	2.80491	0.127728
26d/26d' distance	8.29865	3.44672	0.392329
2a/2a' distance	10.6811	5.6674	−0.0422663
9a/9a' distance	7.43884	3.52812	0.0436751
16a/16a' distance	7.17679	2.91902	0.0990317
23a/23a' distance	7.94948	3.48036	−0.0387662
30a/30a' distance	11.5161	4.59634	2.44471
Core Distance	8.35695	4.23093	

Figure S438. Plots of RMSD and residue packing distances for T-REMD simulations of variant **s28f/31b'-x4x** at 333.99 K.

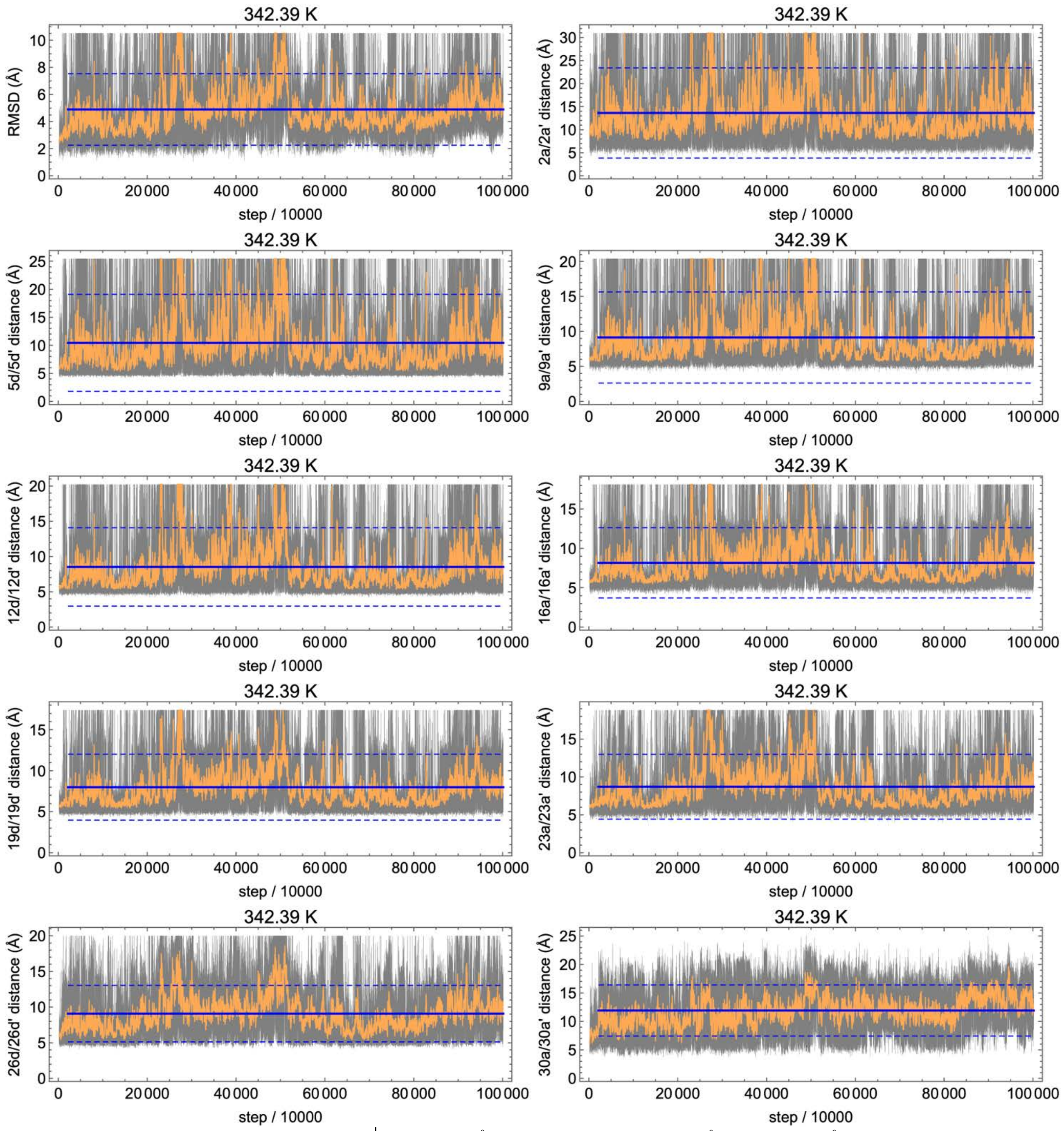

Measure at 342.39 K	Average (Å)	Standard Deviation (Å)	Slope (Å / μs)
RMSD	4.94616	2.63807	0.132321
5d/5d' distance	10.5633	8.65325	−0.604131
12d/12d' distance	8.62218	5.5544	−0.23822
19d/19d' distance	8.08197	4.02378	−0.109228
26d/26d' distance	9.17958	3.9658	0.199458
2a/2a' distance	13.7804	9.78423	−0.847917
9a/9a' distance	9.23592	6.51508	−0.44584
16a/16a' distance	8.25191	4.46573	−0.153865
23a/23a' distance	8.79212	4.2755	−0.279975
30a/30a' distance	12.0378	4.48602	1.95711
Core Distance	9.83836	6.35999	

Figure S439. Plots of RMSD and residue packing distances for T-REMD simulations of variant **s28f/31b’-x4x** at 342.39 K.

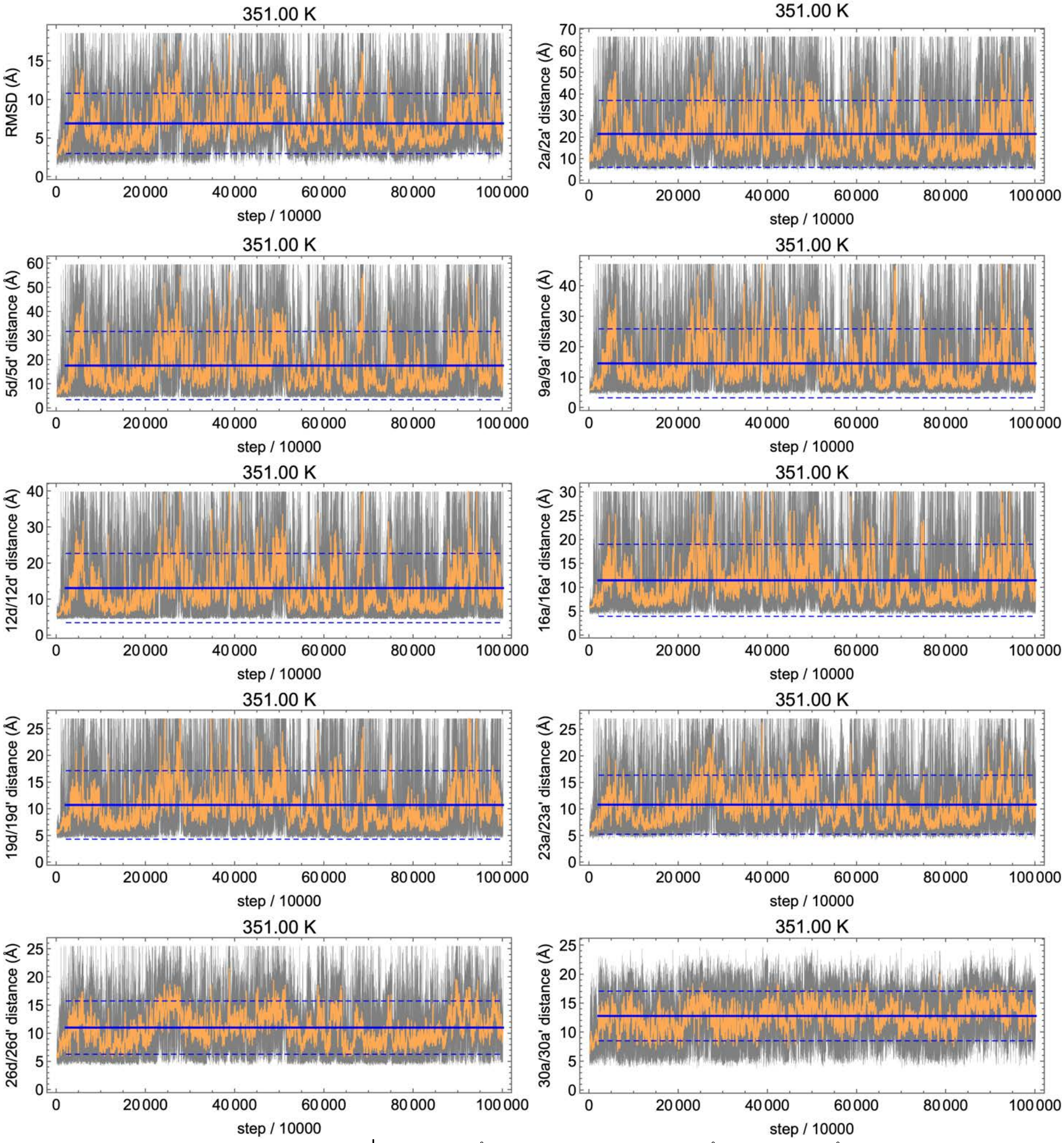

Measure at 351.00 K	Average (Å)	Standard Deviation (Å)	Slope (Å / μs)
RMSD	6.98681	3.90818	−0.0825039
5d/5d' distance	17.8336	14.1542	−1.0016
12d/12d' distance	13.2416	9.61338	−0.455053
19d/19d' distance	10.8326	6.4268	−0.207765
26d/26d' distance	11.1203	4.73564	−0.00386274
2a/2a' distance	21.7411	15.526	−1.27411
9a/9a' distance	14.7263	11.3311	−0.753019
16a/16a' distance	11.5976	7.55009	−0.308936
23a/23a' distance	10.9308	5.55897	−0.332749
30a/30a' distance	12.8941	4.28801	0.998177
Core Distance	13.8798	10.2337	

Figure S440. Plots of RMSD and residue packing distances for T-REMD simulations of variant **s28f/31b'-x4x** at 351.00 K.

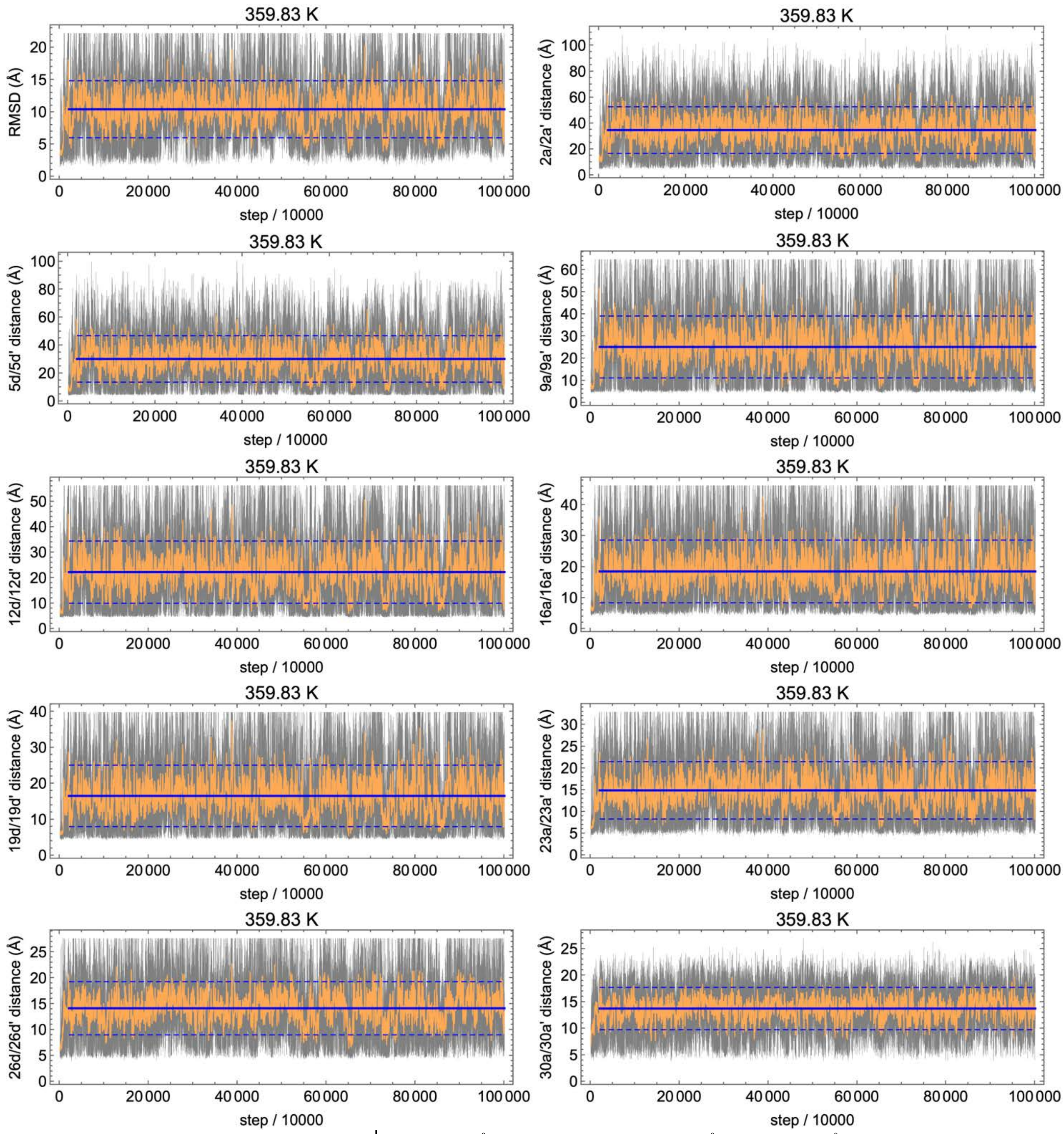

Measure at 359.83 K	Average (Å)	Standard Deviation (Å)	Slope (Å / μs)
RMSD	10.4721	4.41431	−0.0458168
5d/5d' distance	30.5111	16.579	−0.498653
12d/12d' distance	22.3812	12.2146	−0.116388
19d/19d' distance	16.6569	8.54414	0.0973686
26d/26d' distance	14.2242	5.13623	0.0797565
2a/2a' distance	34.992	17.9761	−0.752174
9a/9a' distance	25.317	13.9935	−0.305926
16a/16a' distance	18.6443	10.1312	−0.045694
23a/23a' distance	14.9888	6.59674	0.0111221
30a/30a' distance	13.8225	3.98063	0.213275
Core Distance	21.282	13.6155	

Figure S441. Plots of RMSD and residue packing distances for T-REMD simulations of variant **s28f/31b'-x4x** at 359.83 K.

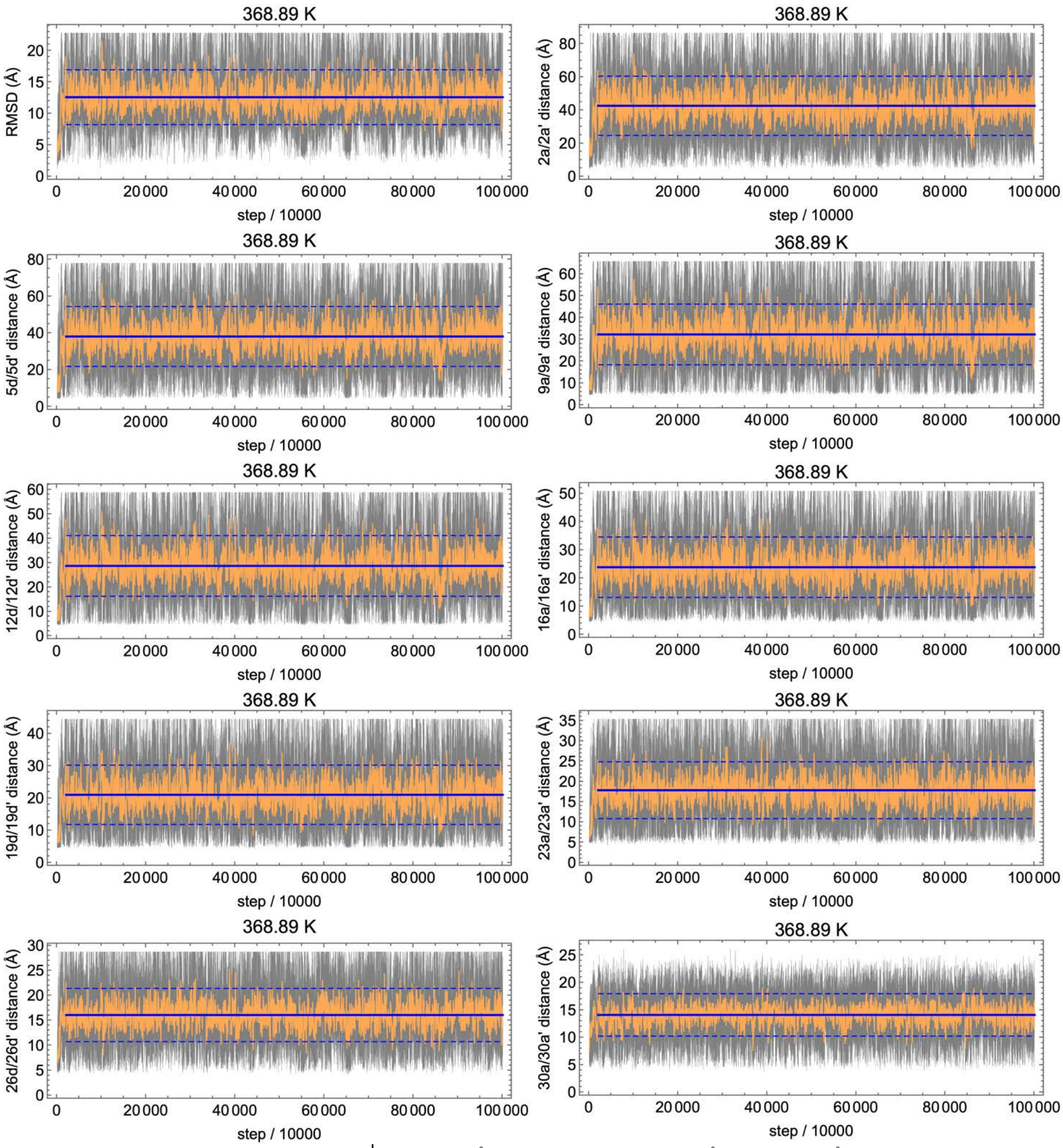

Measure at 368.89 K	Average (Å)	Standard Deviation (Å)	Slope (Å / μs)
RMSD	12.66	4.35783	0.0274612
5d/5d' distance	38.3182	16.2814	−0.0952754
12d/12d' distance	28.8595	12.4395	0.113085
19d/19d' distance	21.1288	9.20313	0.205349
26d/26d' distance	16.159	5.33356	0.139601
2a/2a' distance	42.8534	17.8562	−0.256178
9a/9a' distance	32.4582	13.9162	−0.0622992
16a/16a' distance	24.0156	10.7028	0.102338
23a/23a' distance	17.9485	7.01473	0.126321
30a/30a' distance	14.1726	3.86794	0.10613
Core Distance	26.2126	15.0505	

Figure S442. Plots of RMSD and residue packing distances for T-REMD simulations of variant **s28f/31b’-x4x** at 368.89 K.

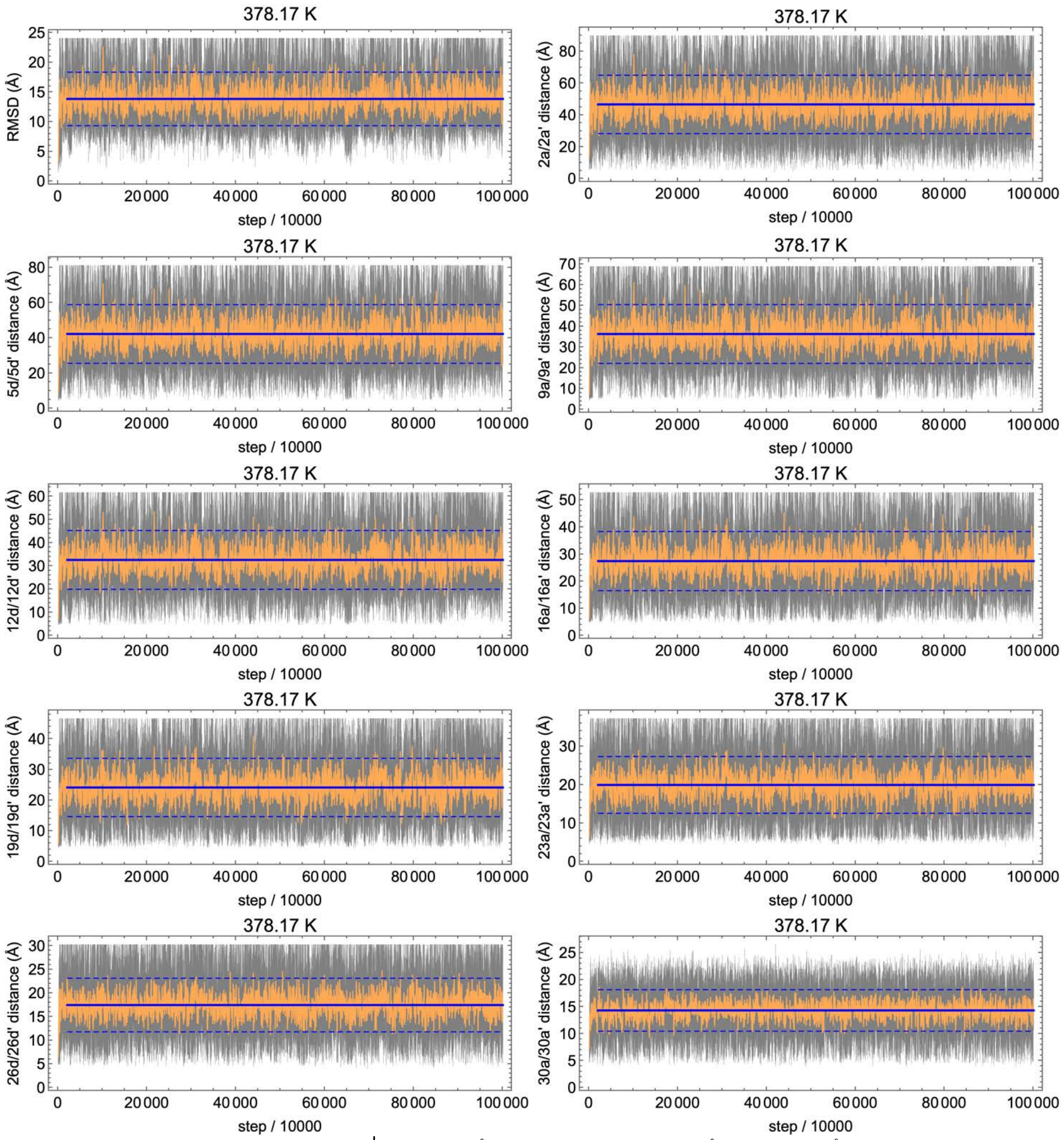

Measure at 378.17 K	Average (Å)	Standard Deviation (Å)	Slope (Å / μs)
RMSD	13.9156	4.50052	0.0628834
5d/5d' distance	42.4617	16.6302	0.0460574
12d/12d' distance	32.7511	12.665	0.216875
19d/19d' distance	24.2814	9.50132	0.204117
26d/26d' distance	17.5515	5.66884	0.123258
2a/2a' distance	46.8986	18.3432	−0.0579495
9a/9a' distance	36.5039	14.1485	0.136933
16a/16a' distance	27.5924	10.9029	0.174692
23a/23a' distance	20.0561	7.38702	0.182029
30a/30a' distance	14.3922	3.84831	0.003244
Core Distance	29.1654	16.0024	

Figure S443. Plots of RMSD and residue packing distances for T-REMD simulations of variant **s28f/31b'-x4x** at 378.17 K.

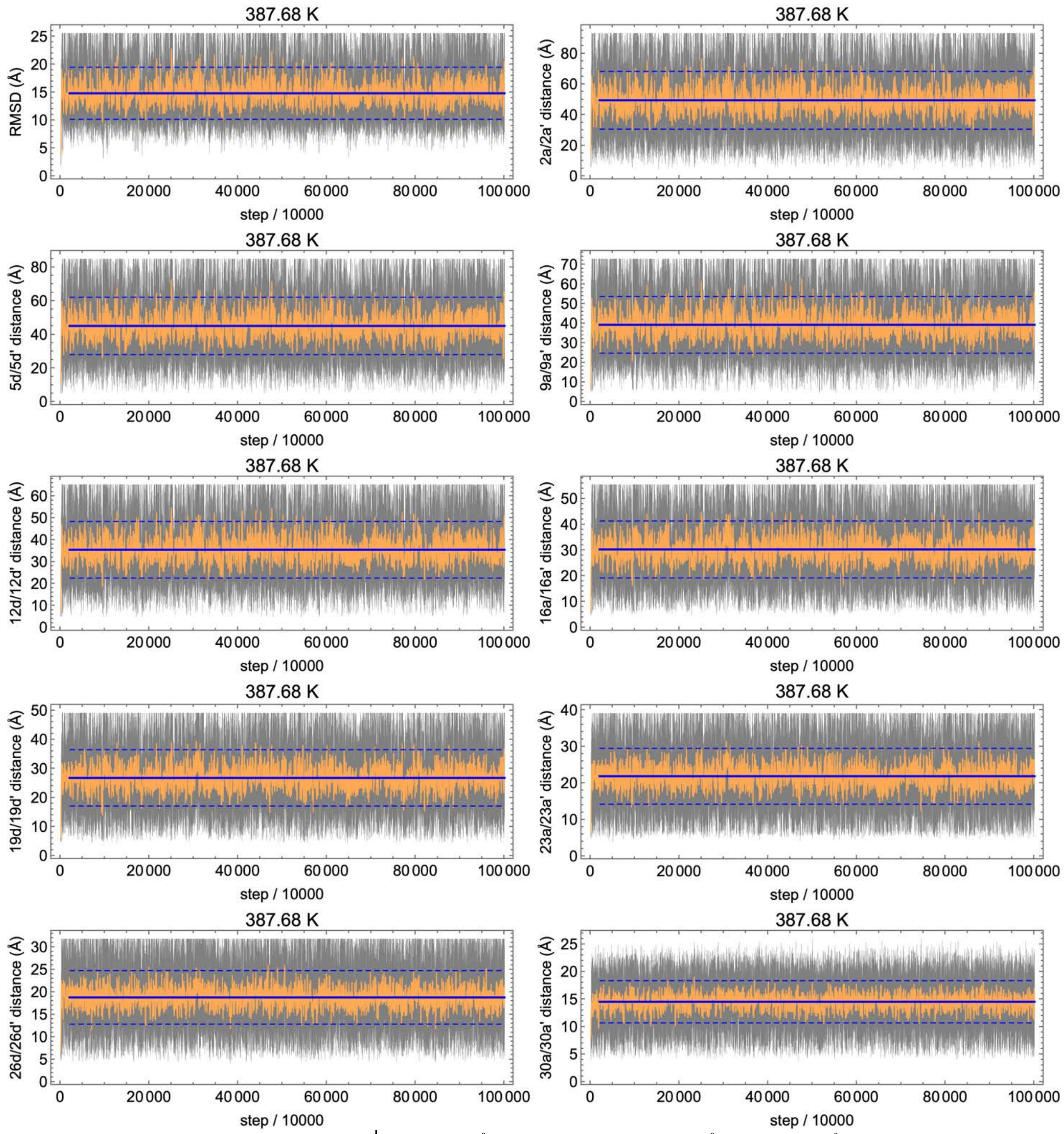

Measure at 387.68 K	Average (Å)	Standard Deviation (Å)	Slope (Å / μs)
RMSD	14.8965	4.65692	−0.0115817
5d/5d' distance	45.3421	17.0578	−0.151298
12d/12d' distance	35.6673	12.9468	0.0442896
19d/19d' distance	26.9219	9.68751	0.0291195
26d/26d' distance	18.889	5.9537	−0.0747442
2a/2a' distance	49.7253	18.851	−0.239326
9a/9a' distance	39.4339	14.4816	−0.0414046
16a/16a' distance	30.4255	11.0604	0.0595674
23a/23a' distance	21.9792	7.64501	−0.0685312
30a/30a' distance	14.6011	3.83721	−0.0658145
Core Distance	31.4428	16.6995	

Figure S444. Plots of RMSD and residue packing distances for T-REMD simulations of variant **s28f/31b'-x4x** at 387.68 K.

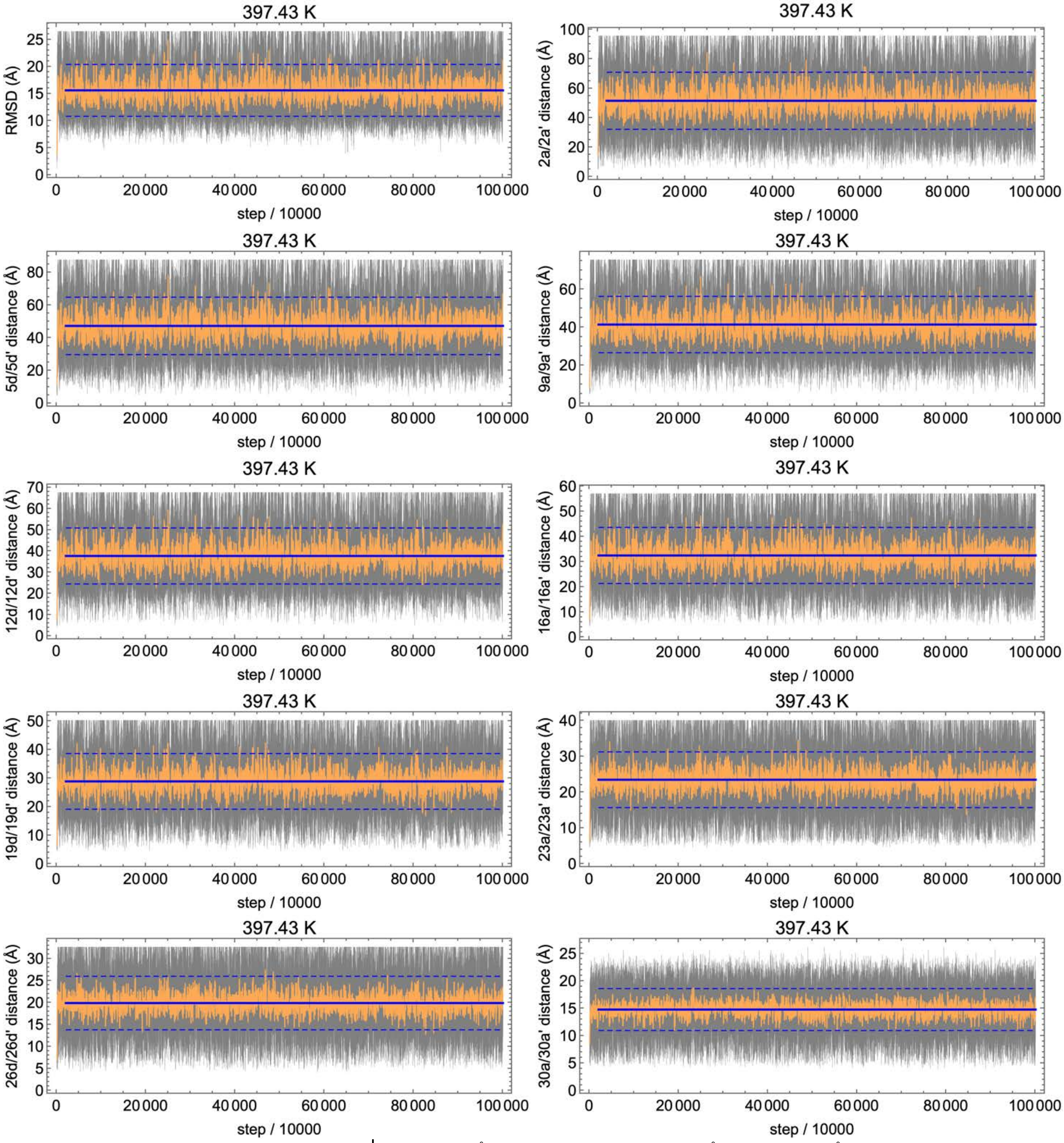

Measure at 397.43 K	Average (Å)	Standard Deviation (Å)	Slope (Å / μs)
RMSD	15.6767	4.78391	−0.0594134
5d/5d' distance	47.486	17.5421	−0.229696
12d/12d' distance	37.8759	13.188	−0.0832437
19d/19d' distance	29.0006	9.75123	−0.010948
26d/26d' distance	19.9829	6.08878	−0.117756
2a/2a' distance	51.8056	19.3968	−0.29598
9a/9a' distance	41.6238	14.8275	−0.13057
16a/16a' distance	32.6216	11.1464	0.0104828
23a/23a' distance	23.5727	7.78124	−0.116009
30a/30a' distance	14.8428	3.82854	−0.0881582
Core Distance	33.2013	17.2393	

Figure S445. Plots of RMSD and residue packing distances for T-REMD simulations of variant **s28f/31b’-x4x** at 397.43 K.

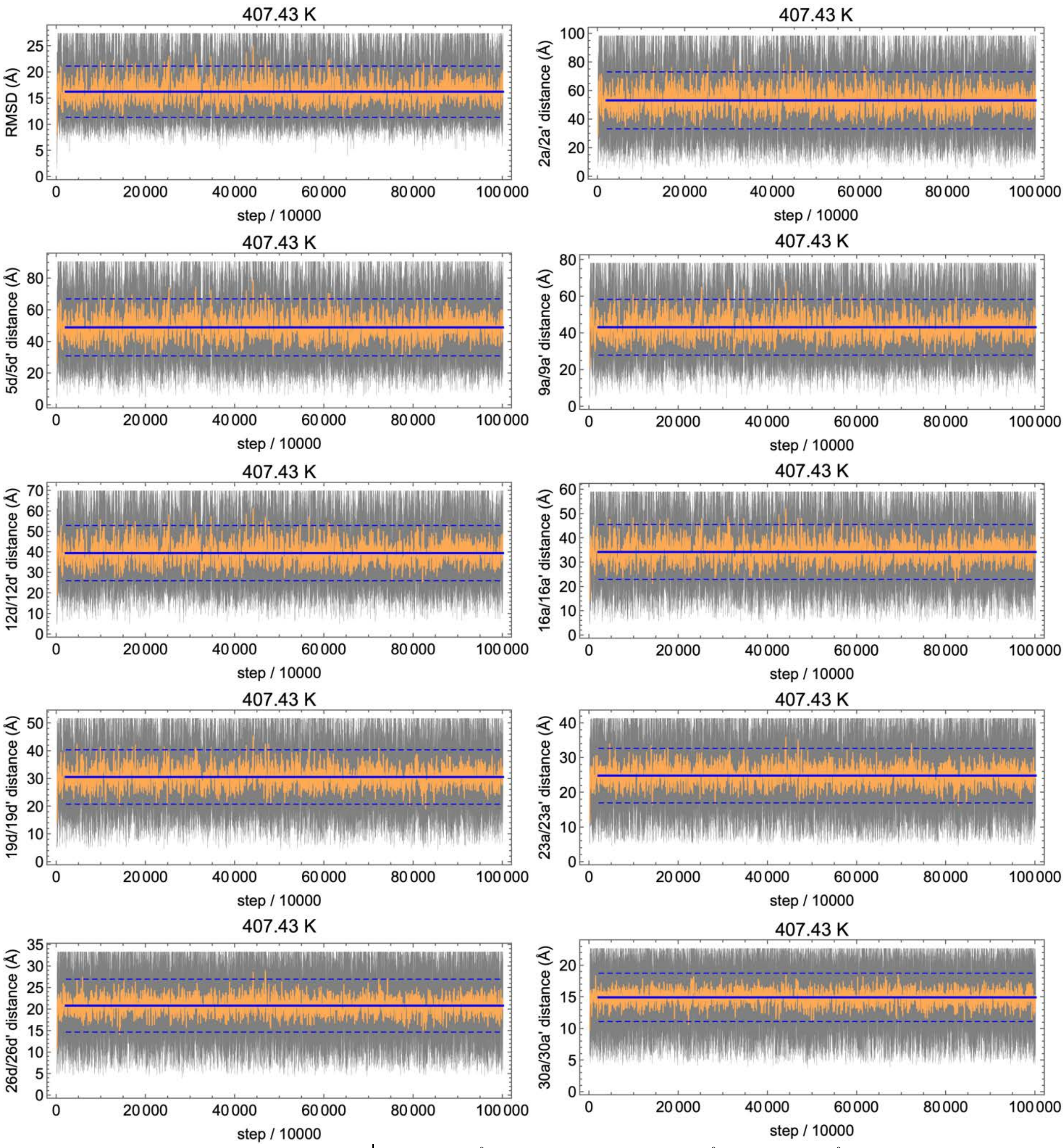

Measure at 407.43 K	Average (Å)	Standard Deviation (Å)	Slope (Å / μs)
RMSD	16.3507	4.8932	−0.0933946
5d/5d' distance	49.2909	18.0023	−0.298042
12d/12d' distance	39.738	13.4705	−0.206701
19d/19d' distance	30.7568	9.8031	−0.168291
26d/26d' distance	20.9501	6.15664	−0.154611
2a/2a' distance	53.5551	19.9635	−0.356249
9a/9a' distance	43.4733	15.1961	−0.207069
16a/16a' distance	34.467	11.291	−0.168655
23a/23a' distance	25.0006	7.86728	−0.210692
30a/30a' distance	15.0248	3.82677	−0.0686629
Core Distance	34.6952	17.7314	

Figure S446. Plots of RMSD and residue packing distances for T-REMD simulations of variant **s28f/31b'-x4x** at 407.43 K.

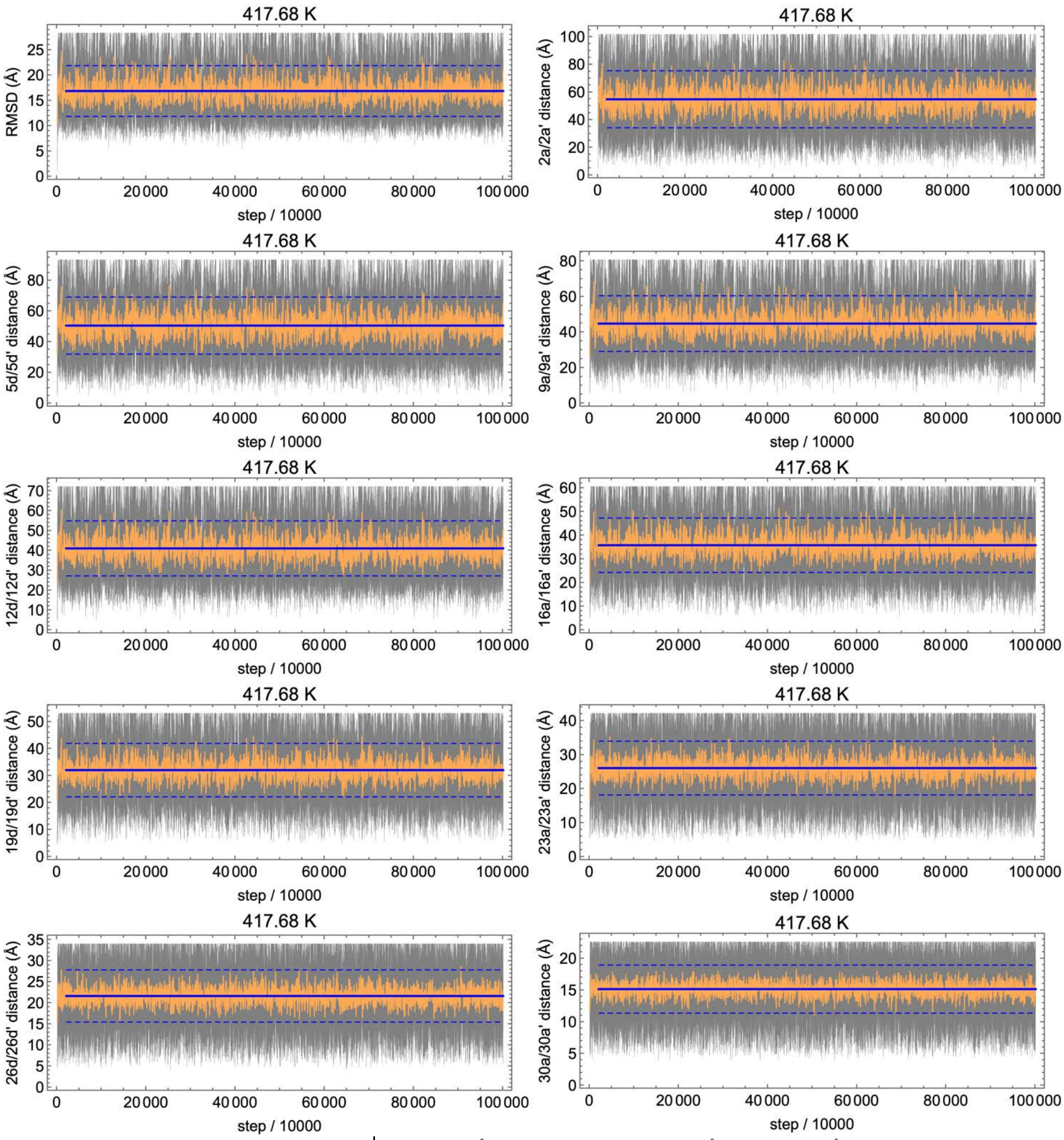

Measure at 417.68 K	Average (Å)	Standard Deviation (Å)	Slope (Å / μs)
RMSD	16.9604	5.01503	−0.0522514
5d/5d' distance	50.8446	18.5835	−0.00812114
12d/12d' distance	41.2835	13.8467	−0.157596
19d/19d' distance	32.2199	9.89925	−0.125212
26d/26d' distance	21.7485	6.18706	−0.0208712
2a/2a' distance	55.1068	20.6228	0.038441
9a/9a' distance	45.0531	15.6656	−0.102027
16a/16a' distance	35.9907	11.4829	−0.150666
23a/23a' distance	26.2042	7.91077	−0.0570088
30a/30a' distance	15.2315	3.78447	−0.0270211
Core Distance	35.9647	18.2355	

Figure S447. Plots of RMSD and residue packing distances for T-REMD simulations of variant **s28f/31b'-x4x** at 417.68 K.

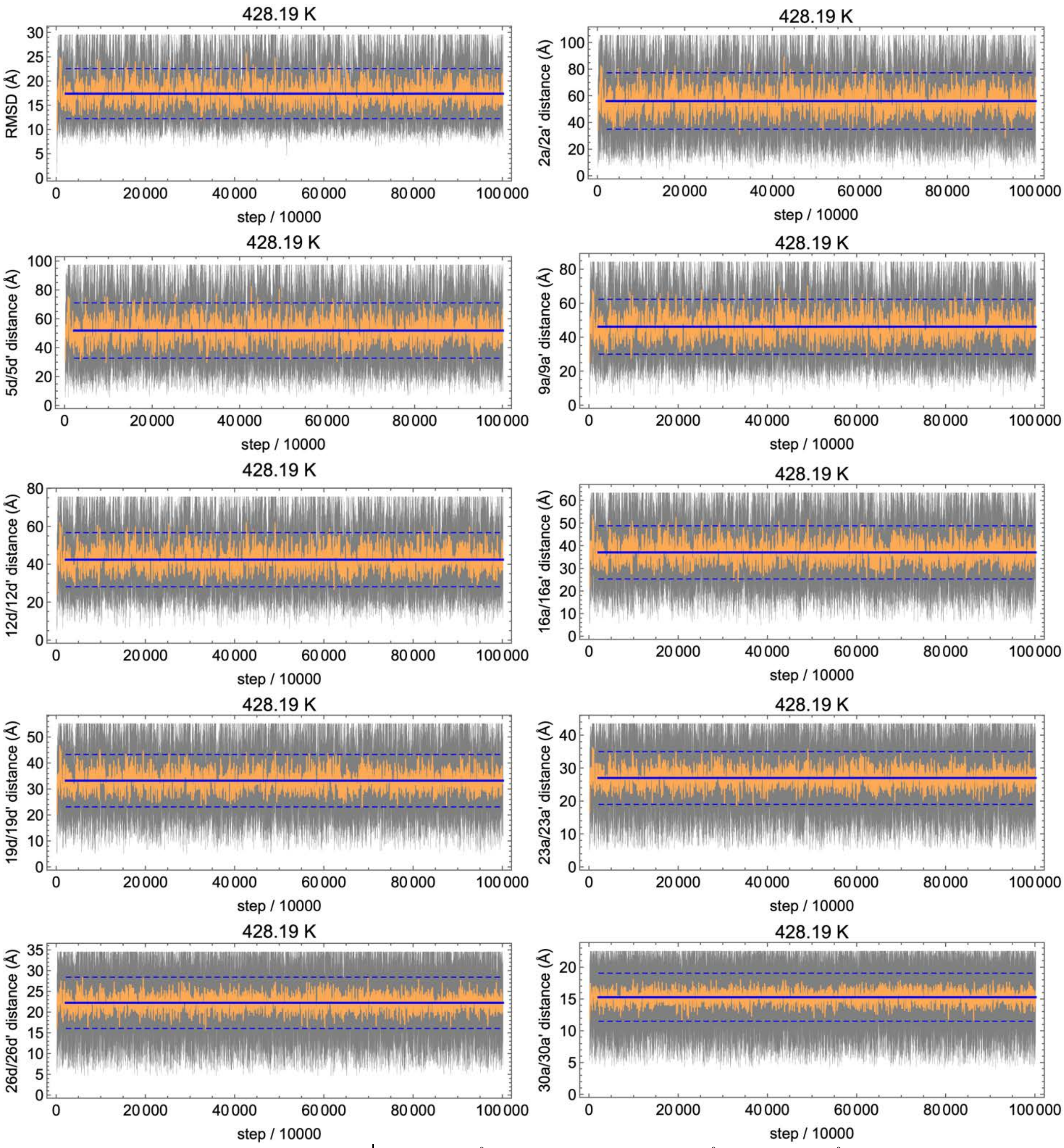

Measure at 428.19 K	Average (Å)	Standard Deviation (Å)	Slope (Å / μs)
RMSD	17.5281	5.16022	−0.0827367
5d/5d' distance	52.3456	19.127	−0.133005
12d/12d' distance	42.752	14.2672	−0.221206
19d/19d' distance	33.4493	10.0953	−0.18284
26d/26d' distance	22.4093	6.17911	−0.0131815
2a/2a' distance	56.6005	21.1619	−0.14675
9a/9a' distance	46.577	16.1817	−0.192256
16a/16a' distance	37.3509	11.7626	−0.264292
23a/23a' distance	27.1966	8.01001	−0.0909128
30a/30a' distance	15.3749	3.7772	0.0269961
Core Distance	37.1174	18.7666	

Figure S448. Plots of RMSD and residue packing distances for T-REMD simulations of variant **s28f/31b'-x4x** at 428.19 K.

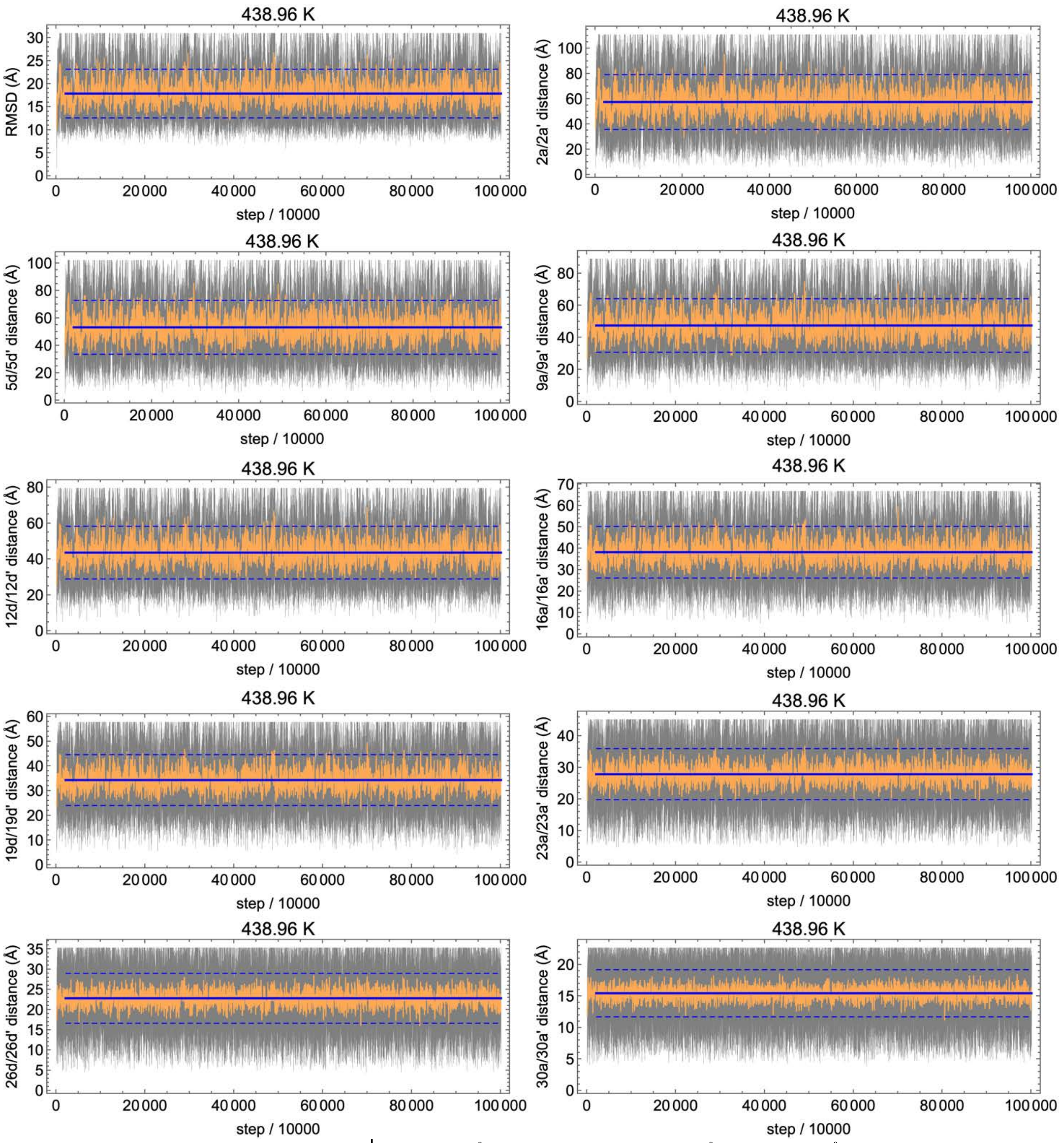

Measure at 438.96 K	Average (Å)	Standard Deviation (Å)	Slope (Å / μs)
RMSD	17.9866	5.27917	−0.0872501
5d/5d' distance	53.5411	19.6513	−0.176635
12d/12d' distance	43.8805	14.6787	−0.260037
19d/19d' distance	34.5082	10.2875	−0.131276
26d/26d' distance	22.9399	6.17775	0.0257047
2a/2a' distance	57.8288	21.7281	−0.132036
9a/9a' distance	47.7314	16.6673	−0.22453
16a/16a' distance	38.4497	12.0571	−0.254529
23a/23a' distance	28.0459	8.08395	−0.00545179
30a/30a' distance	15.5258	3.74162	0.0413485
Core Distance	38.0501	19.2284	

Figure S449. Plots of RMSD and residue packing distances for T-REMD simulations of variant **s28f/31b'-x4x** at 438.96 K.

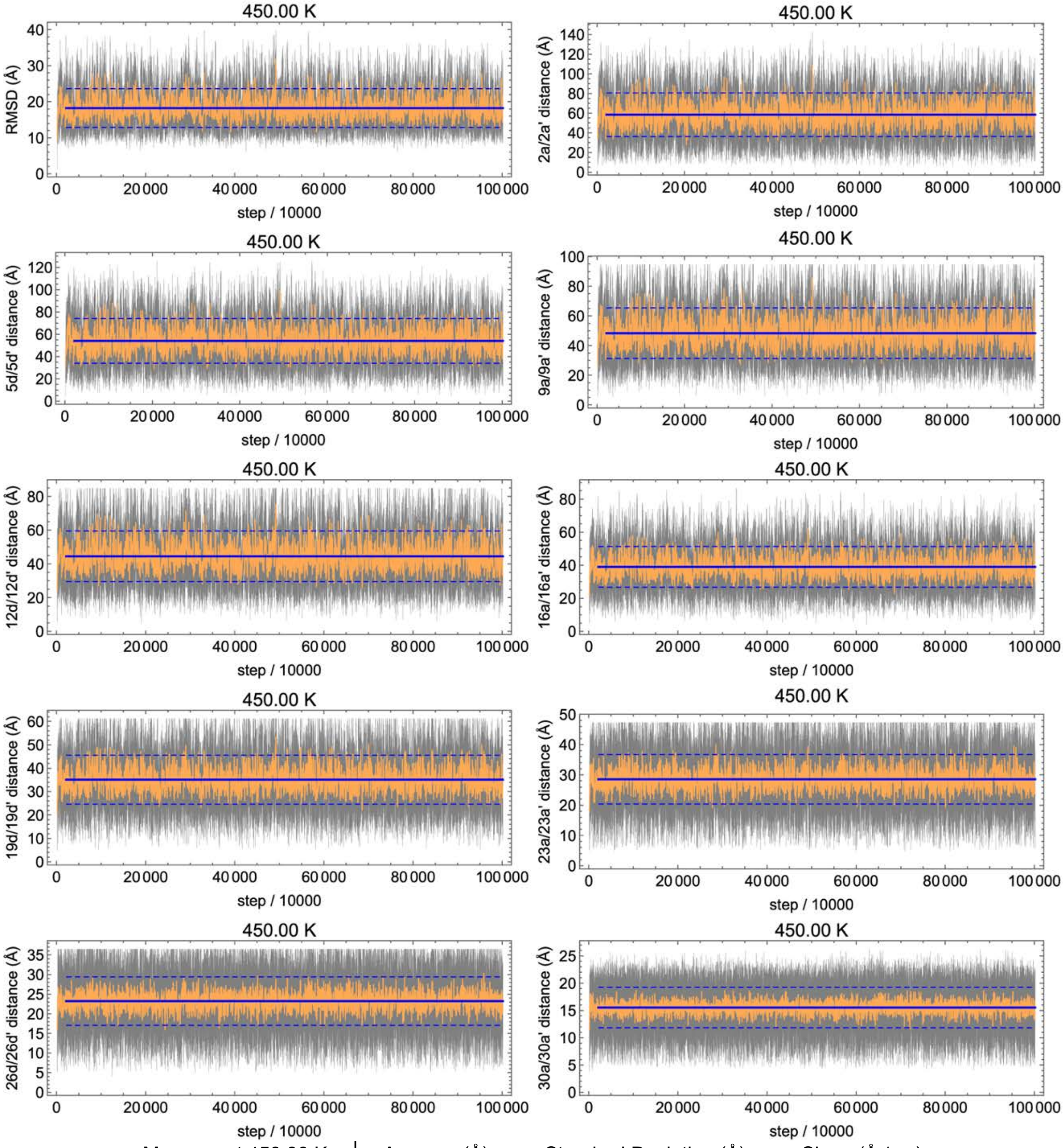

Measure at 450.00 K	Average (Å)	Standard Deviation (Å)	Slope (Å / μs)
RMSD	18.4224	5.37753	−0.102975
5d/5d' distance	54.6881	20.0503	−0.11724
12d/12d' distance	44.9049	15.0134	−0.348953
19d/19d' distance	35.3927	10.469	−0.151049
26d/26d' distance	23.4095	6.1663	0.0243762
2a/2a' distance	59.0922	22.1225	−0.0204447
9a/9a' distance	48.8224	17.0773	−0.279215
16a/16a' distance	39.4227	12.3389	−0.281229
23a/23a' distance	28.7688	8.16623	−0.0608097
30a/30a' distance	15.6474	3.71382	0.0772714
Core Distance	38.9054	19.653	

Figure S450. Plots of RMSD and residue packing distances for T-REMD simulations of variant **s28f/31b'-x4x** at 450.00 K.

(return to Table of Contents)

12. Measuring Cβ-Cβ distances within stapled and non-stapled coiled-coils at sixteen temperatures

We used *cpptraj* to measure the average distance between the β carbons (Cβ-Cβ distance) of the staple site residues during each of the temperature-centric T-REMD trajectories described above for each variant and its non-stapled counterpart. Plots of Cβ-Cβ distance vs. simulation step number for each variant appear below in Figures S451–S470 (20 variants, 16 temperatures per variant). In each plot, gray lines represent Cβ-Cβ distance as a function of simulation step number; orange lines represent the average Cβ-Cβ distance within a moving window of the 50 points that surround each corresponding point in the gray line; the solid blue line shows the trajectory average Cβ-Cβ distance from t = 0 – 2 μs. Dotted blue lines show this trajectory average Cβ-Cβ distance ± one standard deviation. We used linear regression to calculate the slope of Cβ-Cβ distance (the gray lines in the plots) vs. time from t = 0 – 2 μs; values of these slopes (in units of Å / μs) appear in tables below each plot, along with the trajectory average Cβ-Cβ distances and standard deviations. In each case, the slope is substantially smaller than the corresponding standard deviation, suggesting that the Cβ-Cβ distance measurement is not changing systematically over time outside of what would be expected given the observed standard deviation.

Standard deviation gives a sense of the variance of Cβ-Cβ distance within each temperature centric T-REMD trajectory. However, standard deviation alone is insufficient for comparing whether two trajectory average Cβ-Cβ distance values are significantly different. Getting a sense for the true uncertainty of the mean typically involves calculating standard error (*se*), which is usually the standard deviation (σ) divided by the square root of the number of observations (*n*; in each of the plots below, *n* = 100000; as explained above, we wrote trajectory details to file every 1000 steps, resulting in 1 million data points; to facilitate speedy data analysis and reasonable file sizes, we only used one out of every ten data points). However, using $se = \sigma / n^{1/2}$ may not be appropriate for properties derived from molecular dynamics trajectories, where each point may be correlated with (i.e., not independent of) the points that immediately surround it. If this is true, then $se = \sigma / n^{1/2}$ will underestimate the actual uncertainty of the mean.

To get a better sense of the uncertainty of each trajectory average Cβ-Cβ distance, we used block averaging, where we divided the *n* = 100000 Cβ-Cβ distance points for a given trajectory into *M* blocks of size *N* points each, such that $n = M * N$. We then calculated the average Cβ-Cβ distance for each block; the mean of these *M* block averages (which coincided with the average trajectory Cβ-Cβ distance in each case); the standard deviation of the *M* block averages (σ_{block}); and the standard error of the *M* block averages ($se_{block} = \sigma_{block} / M^{1/2}$). We tried several block sizes (*N* = 25 to 5000 and *M* = 4000 to 20) and found that se_{block} generally reaches a plateau for *N* = 2000, 2500, 3215, 4000, 5000. We averaged the resulting se_{block} values to estimate the actual uncertainty of the average Cβ-Cβ distances for a given trajectory. These average se_{block} values are presented with each Cβ-Cβ distance for each temperature-centric T-REMD trajectory in the summary tables below the

plots in Figures S451–S470 under the heading "Block standard error (Å)"; they are also the source of the uncertainties shown for the trajectory average Cβ-Cβ distances in the main text.

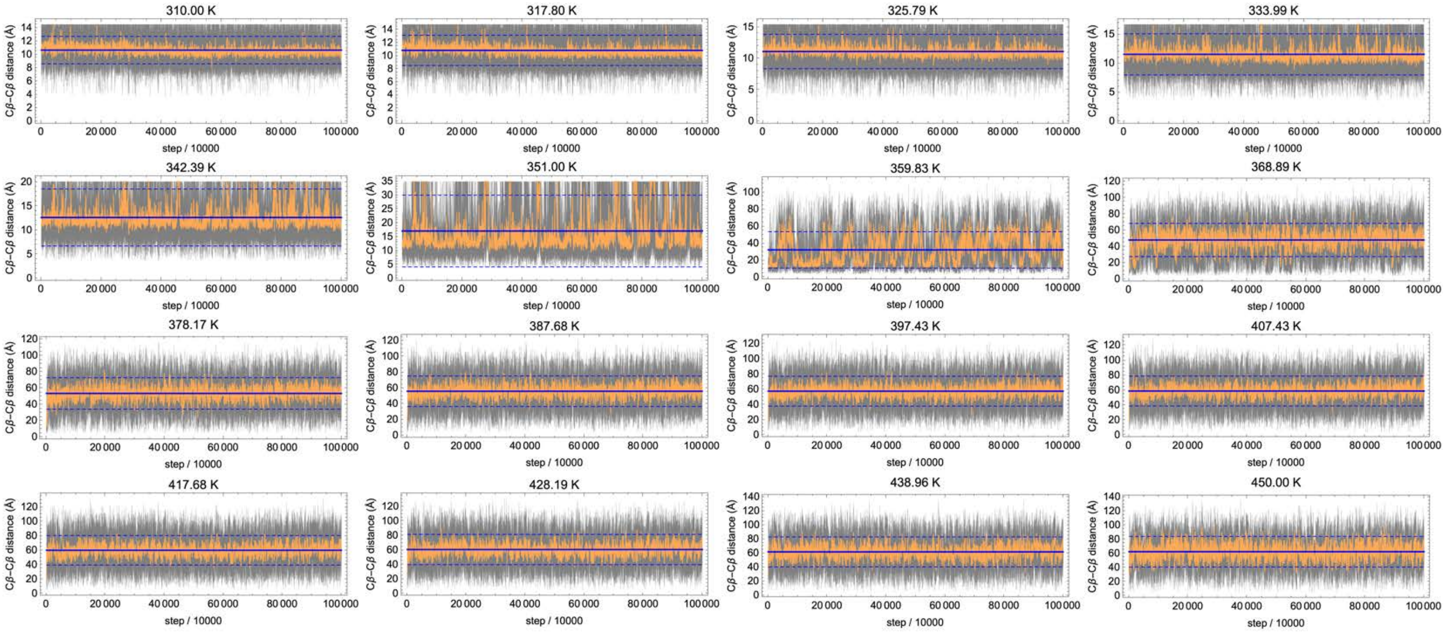

Temp (K)	Average Cβ–Cβ distance (Å)	Block Standard Error (Å)	Standard Deviation (Å)	Slope (Å / μs)
310.00	10.6604	0.0508649	2.05489	−0.402012
317.80	10.844	0.0537362	2.31417	−0.418624
325.79	11.111	0.0474964	2.73305	−0.339487
333.99	11.5463	0.0563245	3.54548	−0.234052
342.39	12.6562	0.136529	5.91866	0.223435
351.00	17.0925	0.7824	12.9469	3.16621
359.83	32.2661	1.5677	21.4511	7.58721
368.89	48.0633	0.684652	20.3734	2.8368
378.17	53.5033	0.33864	19.3297	1.21345
387.68	55.9685	0.272553	19.326	1.03037
397.43	57.6607	0.272342	19.5806	1.00032
407.43	58.9256	0.293544	19.988	0.858017
417.68	60.1075	0.331901	20.4838	0.76549
428.19	60.7799	0.345192	20.8124	0.559153
438.96	61.7822	0.381712	21.3279	0.403288
450.00	62.5236	0.419528	21.6328	0.409689

Figure S451. Plots of Cβ-Cβ distances for T-REMD simulations of variant **6e/1g'-xx** at 310.00, 317.80, 325.79, 333.99, 342.39, 351.00, 359.83, 368.89, 378.17, 387.68, 397.43, 407.43, 417.68, 428.19, 438.96, and 450.00 K.

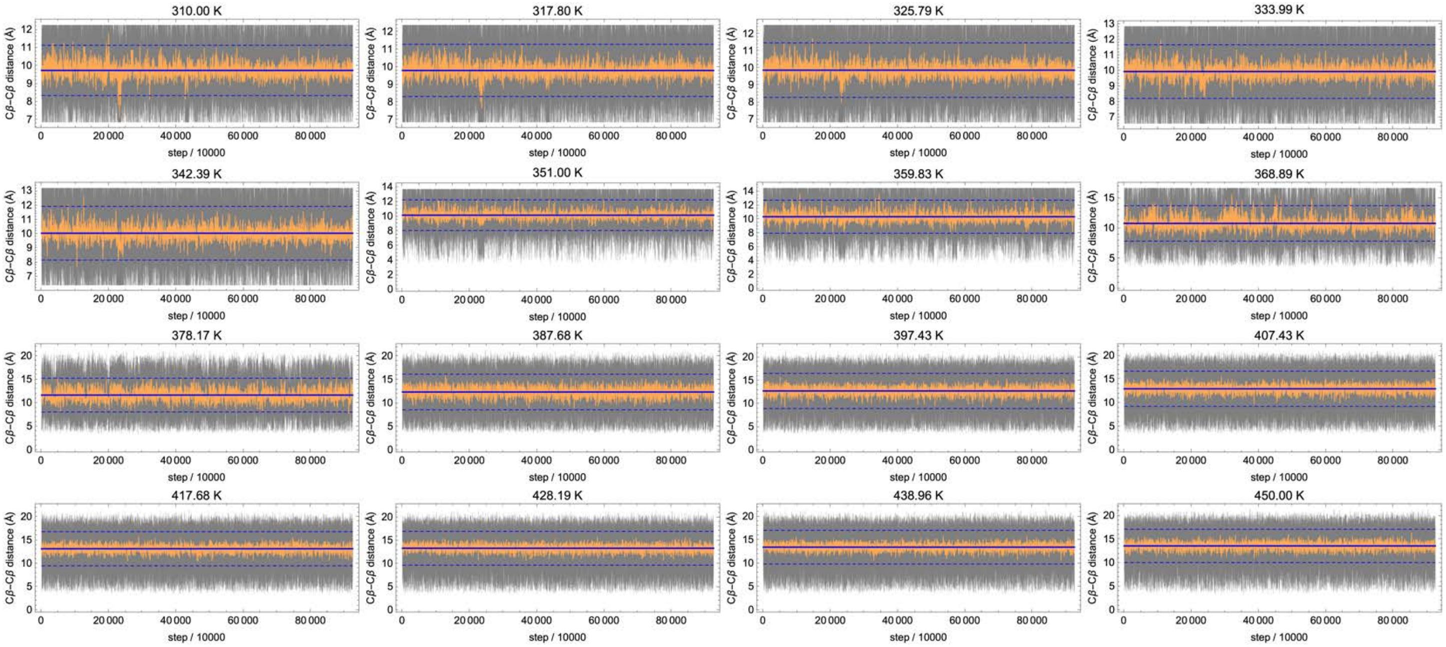

Temp (K)	Average Cβ–Cβ distance (Å)	Block Standard Error (Å)	Standard Deviation (Å)	Slope (Å / μs)
310.00	9.741	0.0166785	1.39219	−0.0690409
317.80	9.80452	0.0151121	1.48161	−0.045673
325.79	9.88003	0.0153044	1.59428	−0.0618231
333.99	9.94351	0.0148335	1.72231	−0.0501139
342.39	10.0484	0.0179843	1.89202	−0.0719967
351.00	10.1623	0.0173472	2.09088	−0.0690764
359.83	10.3641	0.0194207	2.38494	−0.0601184
368.89	10.8053	0.0524761	2.94086	−0.105142
378.17	11.6851	0.0501824	3.58375	−0.0641219
387.68	12.3635	0.0281551	3.80175	−0.0550952
397.43	12.7564	0.0205797	3.78518	−0.0432153
407.43	12.9781	0.01929	3.74541	−0.0340249
417.68	13.168	0.0174505	3.66891	−0.0549094
428.19	13.3303	0.0184992	3.64471	−0.0976978
438.96	13.4711	0.0164057	3.59269	−0.0216542
450.00	13.605	0.0159921	3.53458	−0.0355227

Figure S452. Plots of Cβ-Cβ distances for T-REMD simulations of variant **s6e/1g'-x4x** at 310.00, 317.80, 325.79, 333.99, 342.39, 351.00, 359.83, 368.89, 378.17, 387.68, 397.43, 407.43, 417.68, 428.19, 438.96, and 450.00 K.

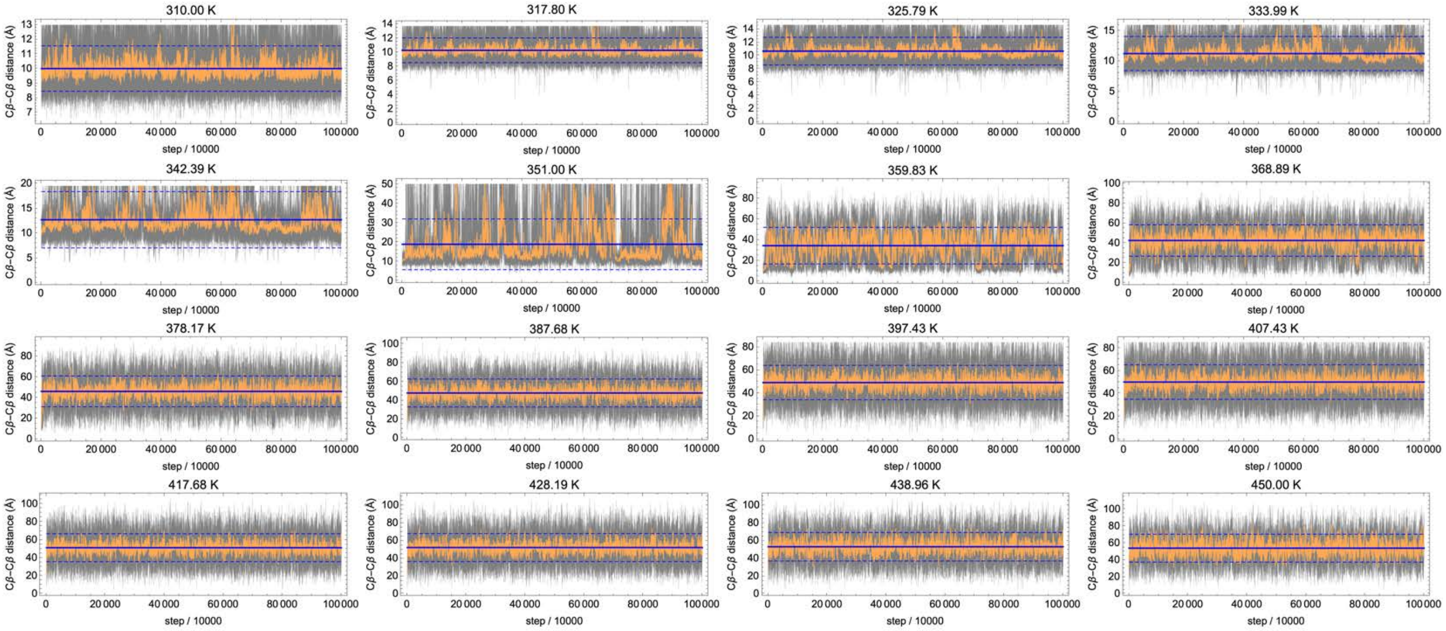

Temp (K)	Average $C\beta$–$C\beta$ distance (Å)	Block Standard Error (Å)	Standard Deviation (Å)	Slope (Å / μs)
310.00	10.0058	0.0353608	1.55368	−0.0570607
317.80	10.2864	0.0424228	1.74859	−0.0673553
325.79	10.667	0.060156	2.08727	−0.0994078
333.99	11.2352	0.0933322	2.81497	−0.133866
342.39	12.6982	0.273046	5.66347	−0.118756
351.00	18.944	0.939701	13.1322	1.84031
359.83	34.329	0.867307	17.6702	1.31372
368.89	42.5737	0.41152	15.8077	0.582732
378.17	46.0745	0.214009	14.8828	0.276814
387.68	47.8925	0.183655	14.8322	0.0397096
397.43	49.0887	0.1741	14.9449	0.00270978
407.43	50.17	0.185432	15.1752	−0.000335334
417.68	51.2572	0.186371	15.5255	0.0460899
428.19	52.224	0.216056	15.8061	0.0736648
438.96	53.1038	0.247773	16.1019	0.0663233
450.00	53.9398	0.273105	16.4656	0.285693

Figure S453. Plots of Cβ-Cβ distances for T-REMD simulations of variant **13e/8g'-xx** at 310.00, 317.80, 325.79, 333.99, 342.39, 351.00, 359.83, 368.89, 378.17, 387.68, 397.43, 407.43, 417.68, 428.19, 438.96, and 450.00 K.

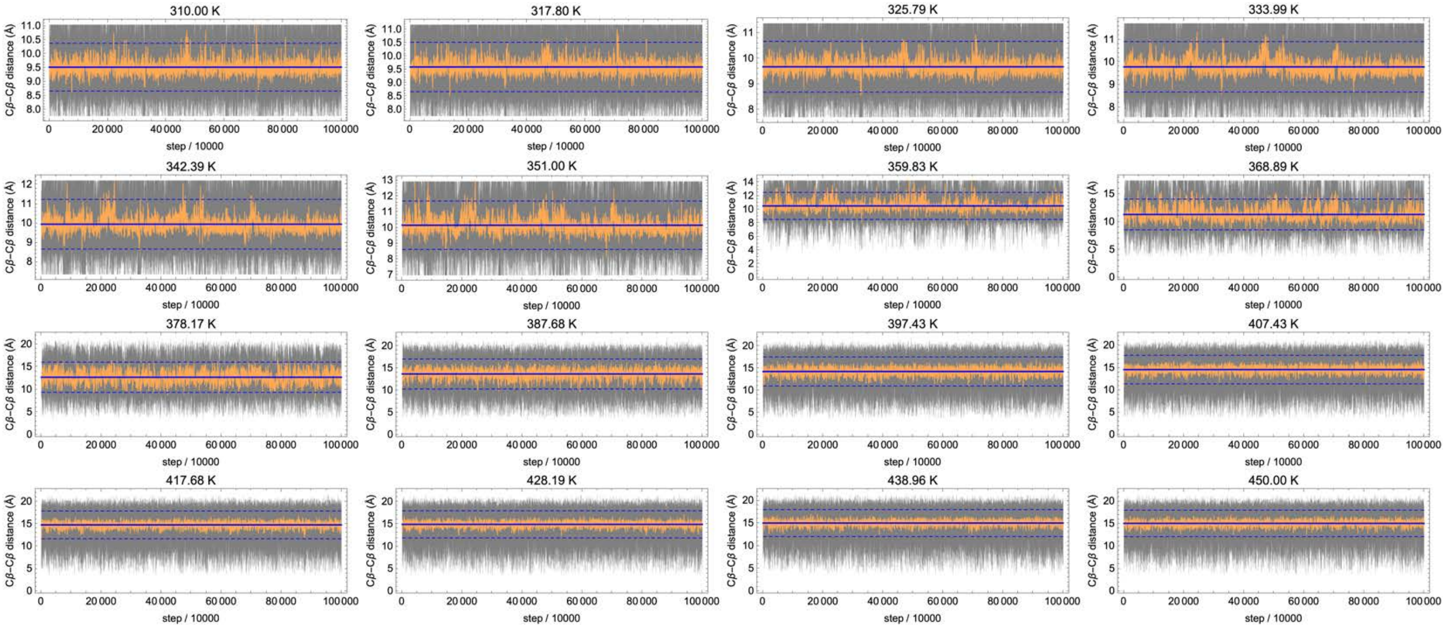

Temp (K)	Average Cβ−Cβ distance (Å)	Block Standard Error (Å)	Standard Deviation (Å)	Slope (Å / μs)
310.00	9.52445	0.00915654	0.858007	−0.00706672
317.80	9.59812	0.00985309	0.927141	−0.0190267
325.79	9.6769	0.0125613	0.99523	−0.0299656
333.99	9.79288	0.0212218	1.11787	−0.0595924
342.39	9.94928	0.02636	1.28428	−0.0994877
351.00	10.1608	0.0314111	1.5343	−0.116873
359.83	10.5346	0.0427988	1.98905	−0.124961
368.89	11.3271	0.0701327	2.75637	−0.152997
378.17	12.7107	0.0612193	3.35187	−0.0334753
387.68	13.7032	0.0288248	3.393	−0.0388128
397.43	14.2702	0.0199449	3.29246	0.0443973
407.43	14.5967	0.0137307	3.19486	0.0284637
417.68	14.8004	0.0151341	3.10724	0.0124994
428.19	14.9404	0.0151262	3.04746	−0.00988689
438.96	15.0382	0.0139428	2.9849	0.0374014
450.00	15.0818	0.0153601	2.94111	0.0741454

Figure S454. Plots of Cβ-Cβ distances for T-REMD simulations of variant **s13e/8g'-x4x** at 310.00, 317.80, 325.79, 333.99, 342.39, 351.00, 359.83, 368.89, 378.17, 387.68, 397.43, 407.43, 417.68, 428.19, 438.96, and 450.00 K.

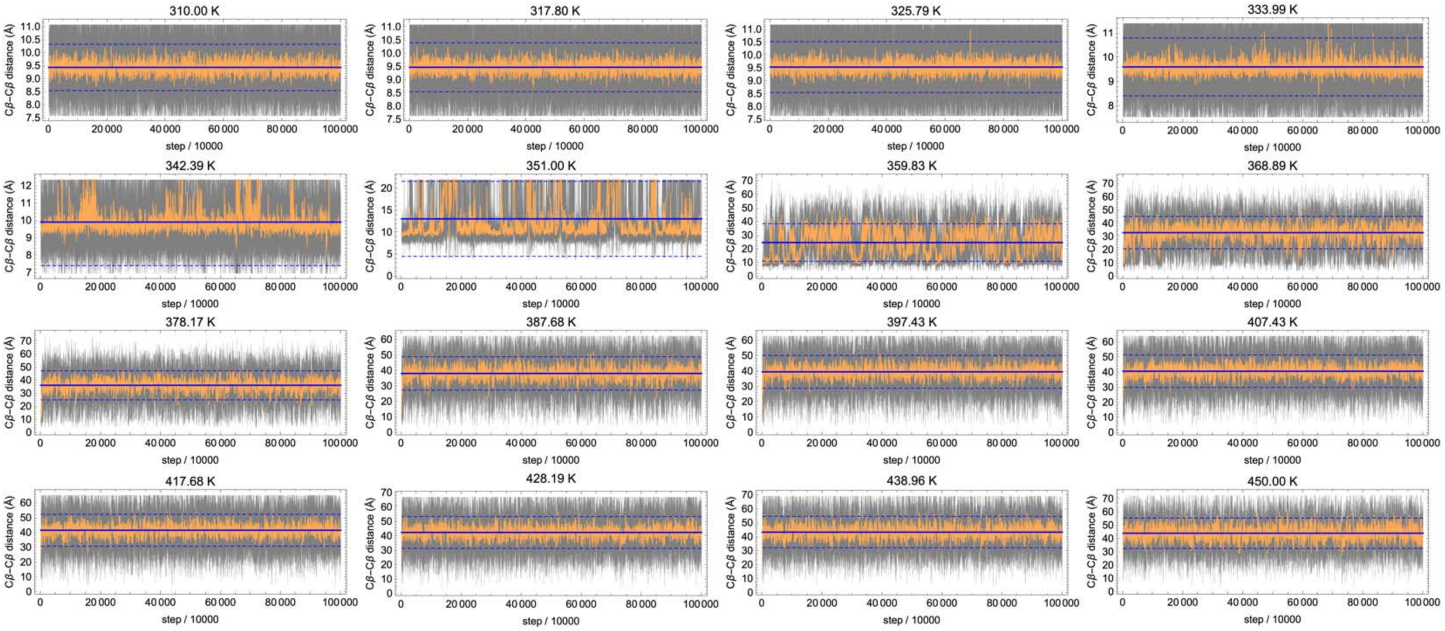

Temp (K)	Average Cβ–Cβ distance (Å)	Block Standard Error (Å)	Standard Deviation (Å)	Slope (Å / μs)
310.00	9.44884	0.00591939	0.888218	−0.0105269
317.80	9.48662	0.00523837	0.92734	−0.0126063
325.79	9.54534	0.00574995	0.990407	−0.00443114
333.99	9.61907	0.0084747	1.192	−0.00367161
342.39	9.92318	0.0482898	2.50389	−0.0121992
351.00	13.1177	0.644152	8.52027	−0.537047
359.83	24.9375	0.859786	13.7584	1.89449
368.89	33.1842	0.330465	12.22	0.295415
378.17	36.3813	0.194406	11.1272	0.113902
387.68	38.3289	0.14361	10.752	0.0888957
397.43	39.7108	0.128114	10.6535	0.258521
407.43	40.8414	0.130933	10.7228	0.262513
417.68	41.7619	0.118739	10.763	0.232361
428.19	42.657	0.125465	11.0266	0.0674109
438.96	43.5563	0.133387	11.2356	0.259334
450.00	44.2848	0.166563	11.4306	0.186973

Figure S455. Plots of Cβ-Cβ distances for T-REMD simulations of variant **20e/15g'-xx** at 310.00, 317.80, 325.79, 333.99, 342.39, 351.00, 359.83, 368.89, 378.17, 387.68, 397.43, 407.43, 417.68, 428.19, 438.96, and 450.00 K.

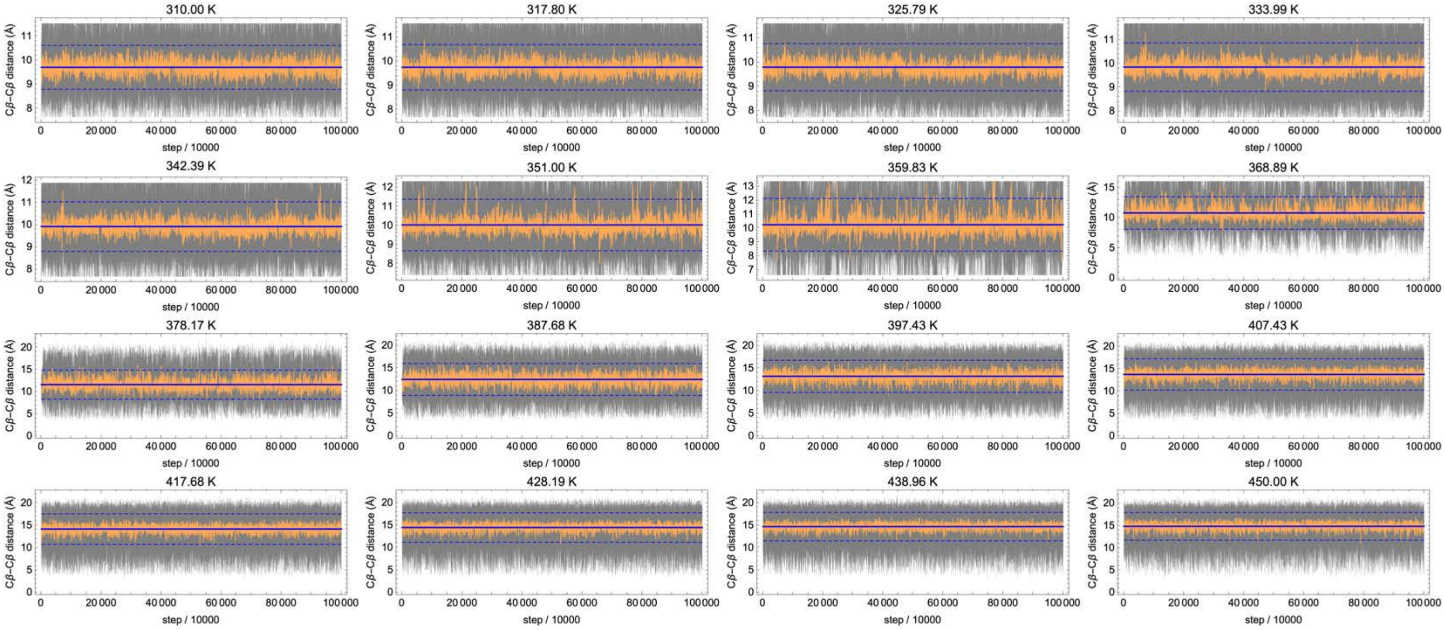

Temp (K)	Average Cβ–Cβ distance (Å)	Block Standard Error (Å)	Standard Deviation (Å)	Slope (Å / μs)
310.00	9.71086	0.0118383	0.910724	−0.0295793
317.80	9.75684	0.0138407	0.939456	−0.0265024
325.79	9.79799	0.0140721	0.976038	−0.0264245
333.99	9.8583	0.0141841	1.01883	−0.0274
342.39	9.92124	0.0131498	1.11915	−0.0081753
351.00	10.0341	0.0165507	1.34636	−0.00344945
359.83	10.2509	0.0278304	1.89247	0.0189704
368.89	10.8076	0.0393743	2.68936	−0.0135868
378.17	11.6395	0.0468859	3.27724	−0.0975302
387.68	12.5015	0.036996	3.51737	−0.0749156
397.43	13.2365	0.0299773	3.55654	−0.0637472
407.43	13.7889	0.0250589	3.50492	0.0167856
417.68	14.194	0.0206088	3.39641	0.0138984
428.19	14.4816	0.0202246	3.28352	0.015933
438.96	14.7072	0.0174001	3.18211	0.0379706
450.00	14.8472	0.019494	3.11323	0.0174693

Figure S456. Plots of Cβ-Cβ distances for T-REMD simulations of variant **s20e/15g’-x4x** at 310.00, 317.80, 325.79, 333.99, 342.39, 351.00, 359.83, 368.89, 378.17, 387.68, 397.43, 407.43, 417.68, 428.19, 438.96, and 450.00 K.

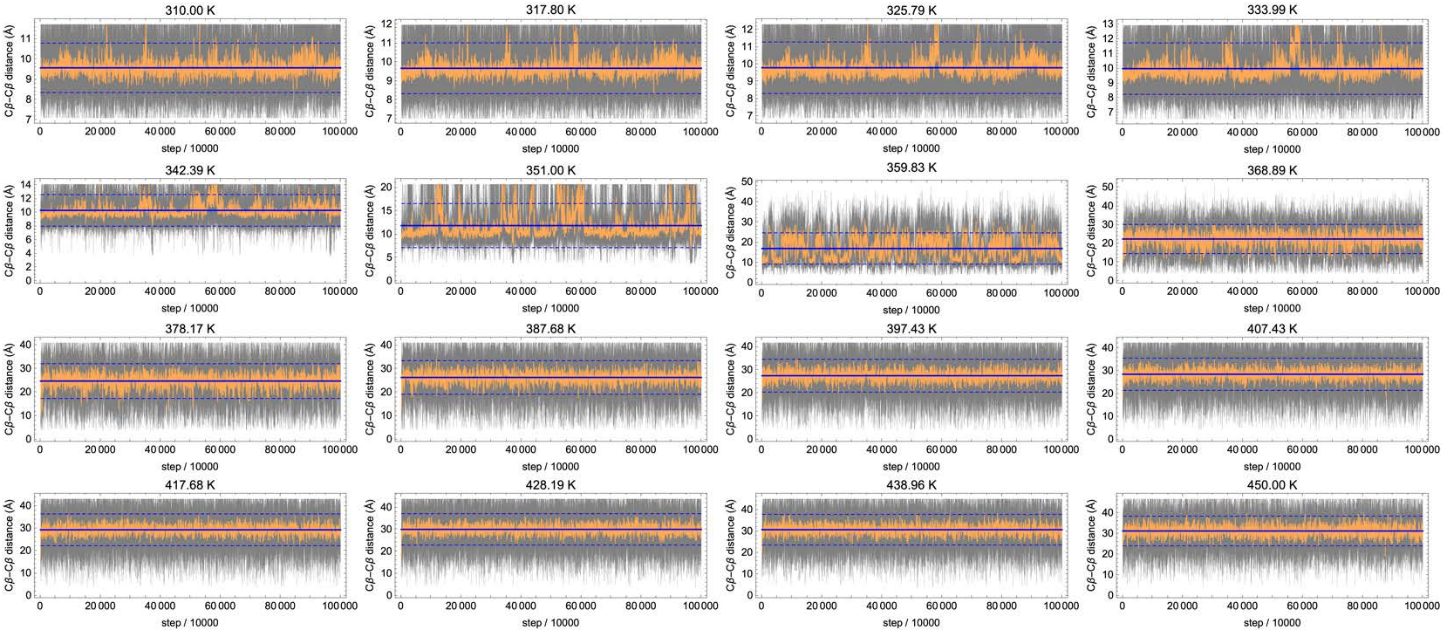

Temp (K)	Average Cβ–Cβ distance (Å)	Block Standard Error (Å)	Standard Deviation (Å)	Slope (Å / μs)
310.00	9.57843	0.0216386	1.22326	0.0282071
317.80	9.67521	0.0277312	1.34613	0.0815047
325.79	9.80064	0.0412265	1.49034	0.125552
333.99	9.98961	0.0651971	1.74937	0.190877
342.39	10.3077	0.0939565	2.30577	0.284715
351.00	11.8618	0.279357	4.71865	0.425243
359.83	17.0064	0.407233	7.75575	0.449505
368.89	22.4436	0.148367	7.77632	−0.078755
378.17	24.7638	0.106308	7.36792	−0.0618604
387.68	26.363	0.0732395	7.15256	−0.0116534
397.43	27.5181	0.0662488	7.06692	−0.101607
407.43	28.5808	0.0669276	7.07274	−0.117139
417.68	29.3631	0.0639226	7.06558	−0.103006
428.19	30.0622	0.0637119	7.10837	−0.115888
438.96	30.7223	0.0691365	7.13208	−0.152268
450.00	31.2722	0.0710002	7.20287	−0.0842301

Figure S457. Plots of Cβ-Cβ distances for T-REMD simulations of variant **27e/22g'-xx** at 310.00, 317.80, 325.79, 333.99, 342.39, 351.00, 359.83, 368.89, 378.17, 387.68, 397.43, 407.43, 417.68, 428.19, 438.96, and 450.00 K.

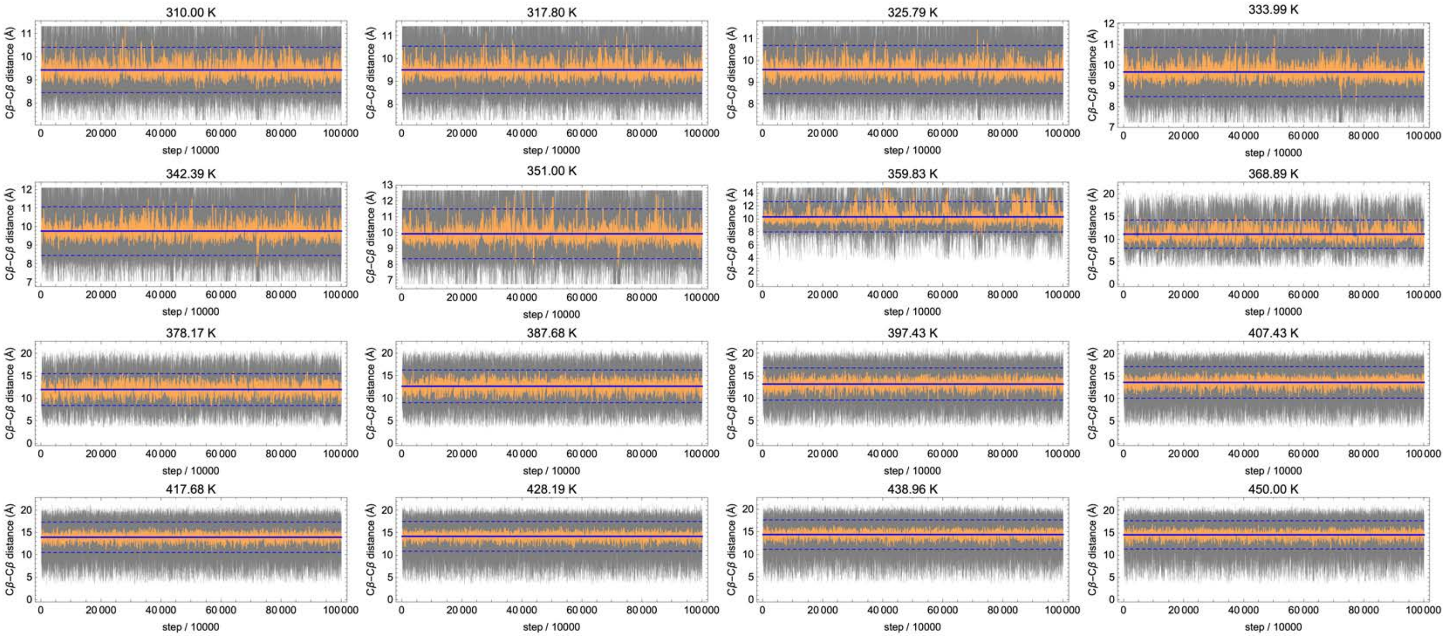

Temp (K)	Average Cβ–Cβ distance (Å)	Block Standard Error (Å)	Standard Deviation (Å)	Slope (Å / μs)
310.00	9.44711	0.0115628	0.977727	−0.00386515
317.80	9.52177	0.0122542	1.03995	−0.0131822
325.79	9.59561	0.0129168	1.10423	−0.0042825
333.99	9.68571	0.0145453	1.18554	−0.0159569
342.39	9.7865	0.0162865	1.31129	−0.0124433
351.00	9.94275	0.0238183	1.56844	−0.0124017
359.83	10.3883	0.0643078	2.31667	0.172032
368.89	11.1633	0.0614075	3.12307	0.124212
378.17	12.0472	0.0406371	3.54083	0.100822
387.68	12.7334	0.0313731	3.6205	0.0573067
397.43	13.2584	0.0265316	3.57318	0.0851957
407.43	13.6867	0.0246316	3.50962	0.0788344
417.68	14.0147	0.0187872	3.40572	0.00711773
428.19	14.2559	0.0125046	3.33352	−0.0260599
438.96	14.4545	0.0193805	3.23998	0.0188906
450.00	14.6117	0.0142628	3.16943	0.0080708

Figure S458. Plots of Cβ-Cβ distances for T-REMD simulations of variant **s27e/22g'-x4x** at 310.00, 317.80, 325.79, 333.99, 342.39, 351.00, 359.83, 368.89, 378.17, 387.68, 397.43, 407.43, 417.68, 428.19, 438.96, and 450.00 K.

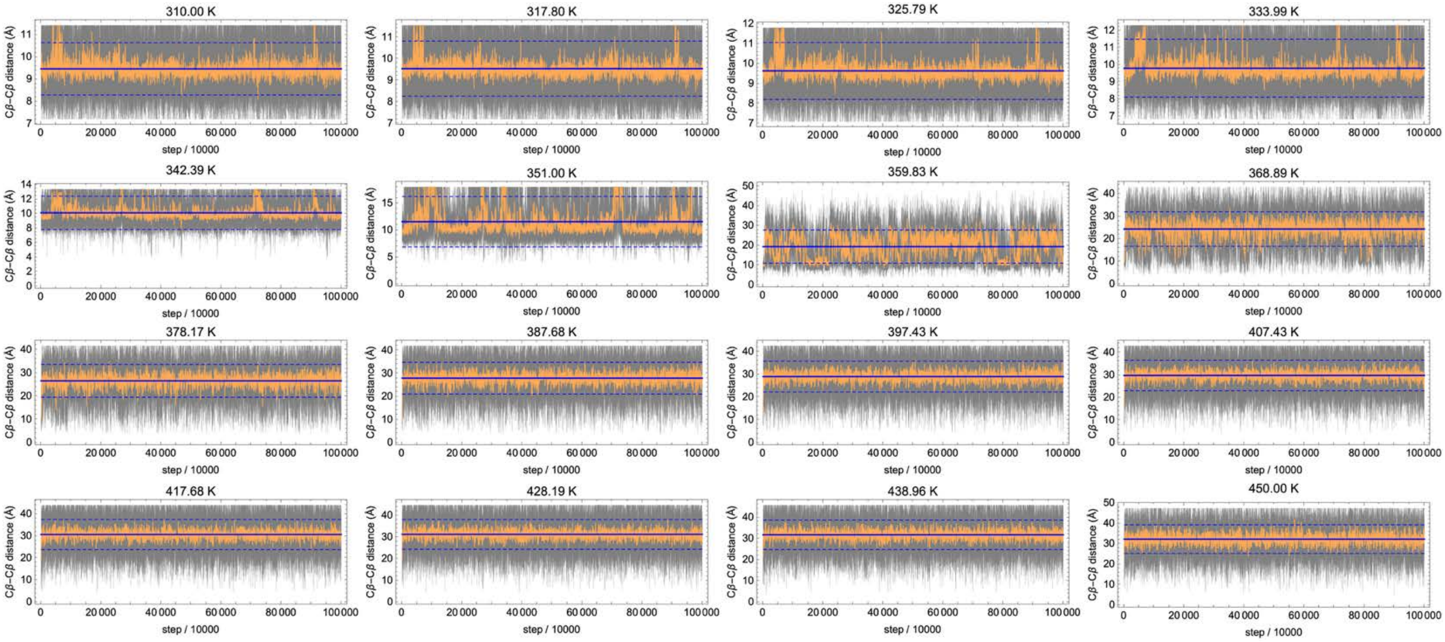

Temp (K)	Average Cβ–Cβ distance (Å)	Block Standard Error (Å)	Standard Deviation (Å)	Slope (Å / μs)
310.00	9.46382	0.0201375	1.17303	−0.0788369
317.80	9.54339	0.0252851	1.28027	−0.0988392
325.79	9.63763	0.0332142	1.42796	−0.102986
333.99	9.80037	0.0465934	1.68652	−0.117696
342.39	10.1301	0.0821757	2.29584	−0.172015
351.00	11.5722	0.297681	4.69268	−0.454959
359.83	19.3979	0.51797	8.42803	0.820862
368.89	24.376	0.178995	7.65407	0.502738
378.17	26.62	0.0860155	7.06106	0.242887
387.68	27.9568	0.0705949	6.87581	0.174447
397.43	29.0351	0.0584076	6.77426	0.0726371
407.43	29.8728	0.052068	6.78472	0.0346165
417.68	30.6692	0.0515002	6.82958	−0.0746393
428.19	31.2704	0.0525077	6.87103	−0.23893
438.96	31.7882	0.0547136	6.94342	−0.107822
450.00	32.2599	0.0667936	7.01388	0.0165427

Figure S459. Plots of Cβ-Cβ distances for T-REMD simulations of variant **27e/22g'-z4x** at 310.00, 317.80, 325.79, 333.99, 342.39, 351.00, 359.83, 368.89, 378.17, 387.68, 397.43, 407.43, 417.68, 428.19, 438.96, and 450.00 K.

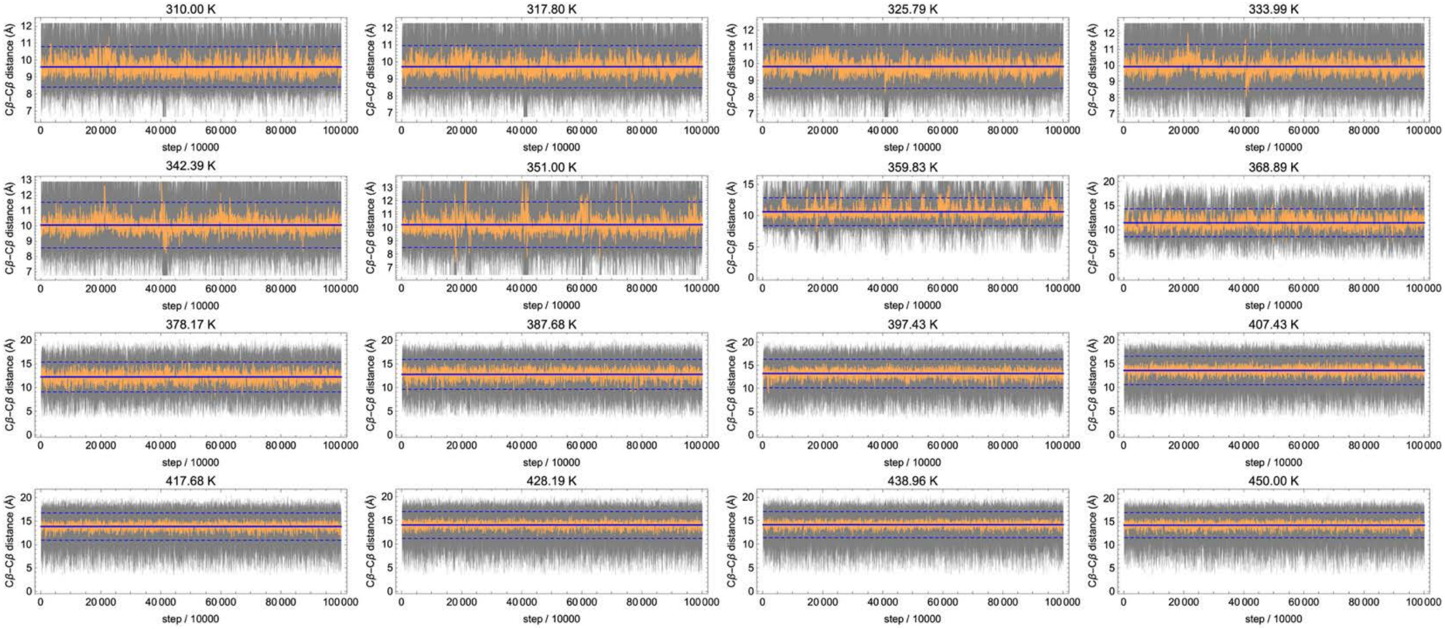

Temp (K)	Average Cβ–Cβ distance (Å)	Block Standard Error (Å)	Standard Deviation (Å)	Slope (Å / μs)
310.00	9.6162	0.0208992	1.18773	−0.0272621
317.80	9.74024	0.0213113	1.2487	−0.0283312
325.79	9.84562	0.0224886	1.30438	−0.04112
333.99	9.95112	0.024687	1.3721	−0.0425581
342.39	10.0766	0.0262465	1.48974	−0.0409878
351.00	10.2428	0.0337564	1.71332	−0.0214124
359.83	10.6735	0.0572436	2.24161	0.138786
368.89	11.5206	0.0553086	2.90429	0.157318
378.17	12.2988	0.0346667	3.14639	0.0625202
387.68	12.8761	0.0244994	3.15326	0.0128845
397.43	13.308	0.0214788	3.08705	−0.0137514
407.43	13.6607	0.0170303	3.00474	−0.00836671
417.68	13.9109	0.0173718	2.91618	−0.0143467
428.19	14.1073	0.0151677	2.84048	−0.0469705
438.96	14.2323	0.011794	2.77335	0.000469972
450.00	14.3275	0.0148025	2.72408	−0.00438478

Figure S460. Plots of Cβ-Cβ distances for T-REMD simulations of variant **s27e/22g'-z4x** at 310.00, 317.80, 325.79, 333.99, 342.39, 351.00, 359.83, 368.89, 378.17, 387.68, 397.43, 407.43, 417.68, 428.19, 438.96, and 450.00 K.

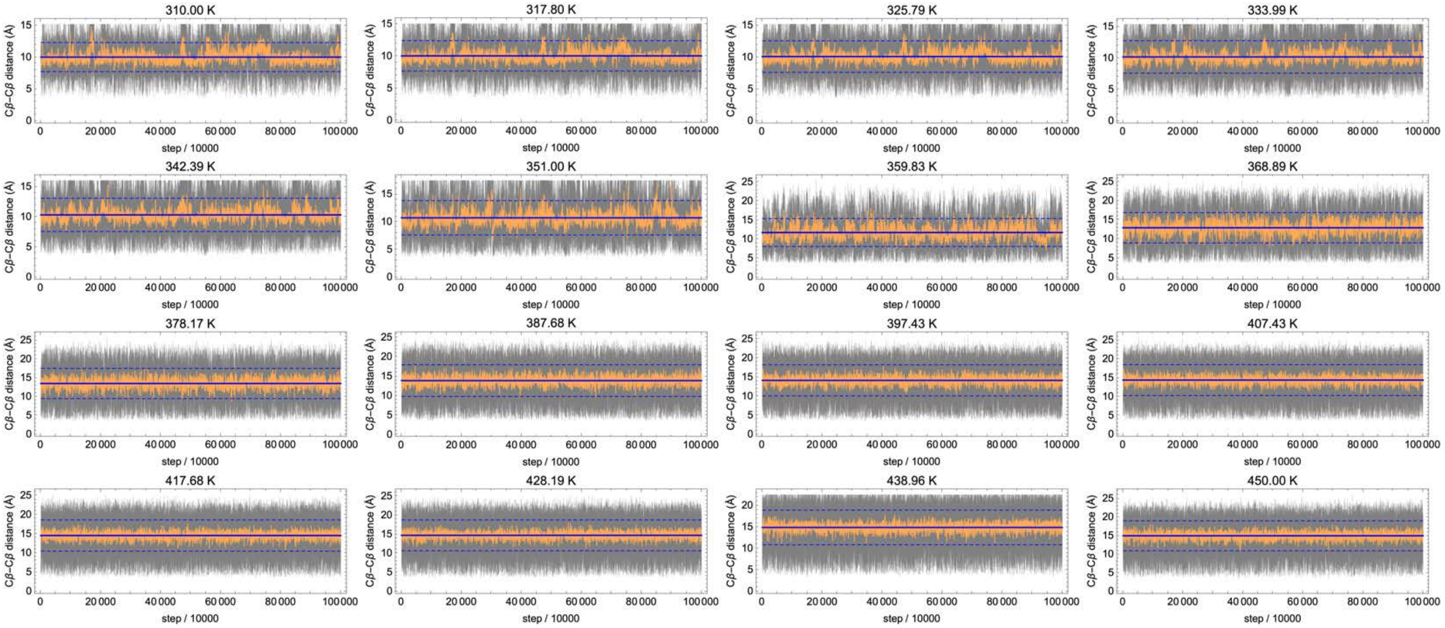

Temp (K)	Average Cβ–Cβ distance (Å)	Block Standard Error (Å)	Standard Deviation (Å)	Slope (Å / μs)
310.00	10.0221	0.0847244	2.28232	0.0751647
317.80	10.0543	0.0758271	2.35999	0.0893397
325.79	10.1011	0.0728944	2.4691	0.117204
333.99	10.1943	0.0679375	2.58422	0.118012
342.39	10.3365	0.0715435	2.75768	0.158965
351.00	10.7306	0.0865711	3.10018	0.227804
359.83	11.7462	0.113957	3.66468	0.0780811
368.89	12.9494	0.0735786	3.96268	0.0340545
378.17	13.5216	0.0404517	4.01998	0.0812509
387.68	13.8962	0.0311547	4.06494	0.0621822
397.43	14.1596	0.0328125	4.07544	0.0222294
407.43	14.3971	0.0265244	4.09772	−0.0124704
417.68	14.573	0.0193304	4.07899	−0.0080719
428.19	14.7437	0.0207716	4.05505	0.0261164
438.96	14.9032	0.0230074	4.04714	0.0372312
450.00	14.9779	0.022472	4.03842	0.0292716

Figure S461. Plots of Cβ-Cβ distances for T-REMD simulations of variant **34e/29g'-xx** at 310.00, 317.80, 325.79, 333.99, 342.39, 351.00, 359.83, 368.89, 378.17, 387.68, 397.43, 407.43, 417.68, 428.19, 438.96, and 450.00 K.

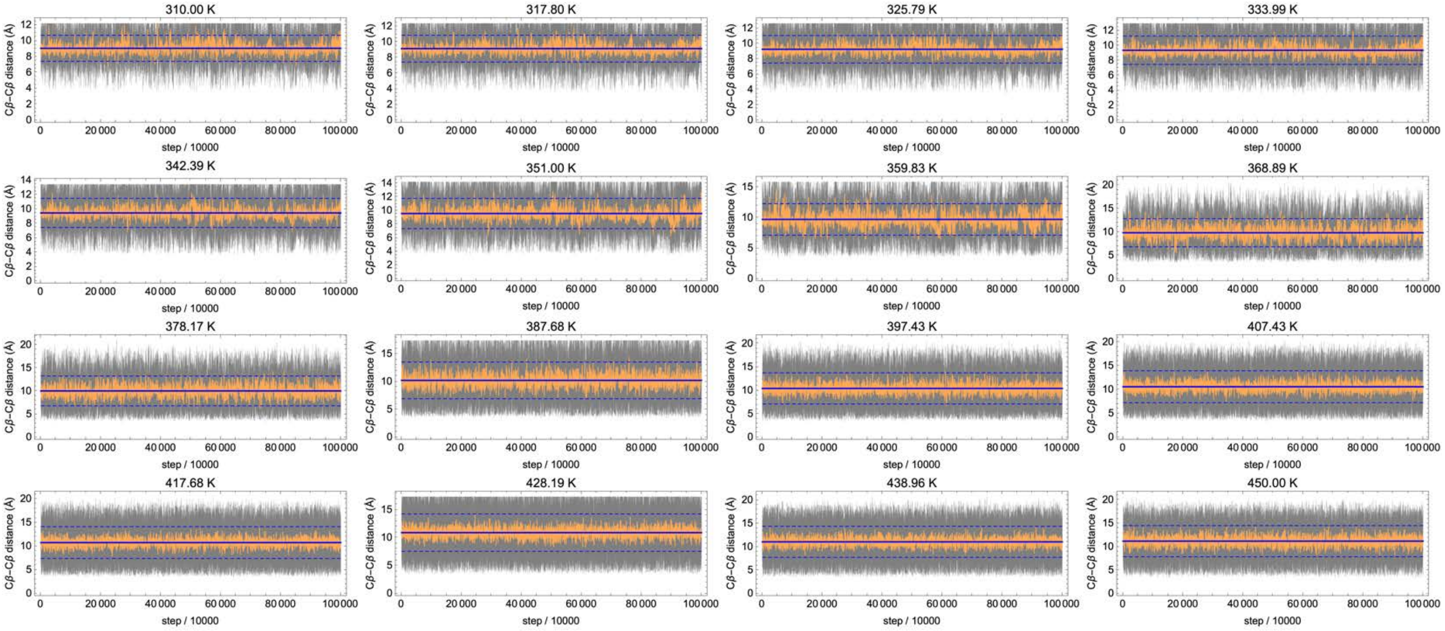

Temp (K)	Average Cβ–Cβ distance (Å)	Block Standard Error (Å)	Standard Deviation (Å)	Slope (Å / μs)
310.00	9.05627	0.0342339	1.64208	0.00278967
317.80	9.15475	0.0345543	1.71411	−0.0048656
325.79	9.25834	0.0370118	1.79731	0.0311464
333.99	9.37307	0.0366755	1.89957	−0.00964815
342.39	9.47651	0.0369502	2.02316	−0.0332567
351.00	9.57051	0.044248	2.21319	−0.0945138
359.83	9.70068	0.0542103	2.57514	−0.0296226
368.89	9.8425	0.0465191	3.01252	−0.0125904
378.17	10.0414	0.032806	3.2258	0.0539504
387.68	10.2246	0.026201	3.29658	0.0525183
397.43	10.4368	0.0238072	3.32836	0.0313127
407.43	10.6124	0.0247037	3.34882	−0.0150092
417.68	10.7694	0.0208611	3.35308	0.0206343
428.19	10.901	0.0220995	3.345	−0.0231436
438.96	11.0745	0.0206825	3.32974	−0.0128594
450.00	11.1967	0.020266	3.32535	−0.0336835

Figure S462. Plots of Cβ-Cβ distances for T-REMD simulations of variant **s34e/29g’-x4x** at 310.00, 317.80, 325.79, 333.99, 342.39, 351.00, 359.83, 368.89, 378.17, 387.68, 397.43, 407.43, 417.68, 428.19, 438.96, and 450.00 K.

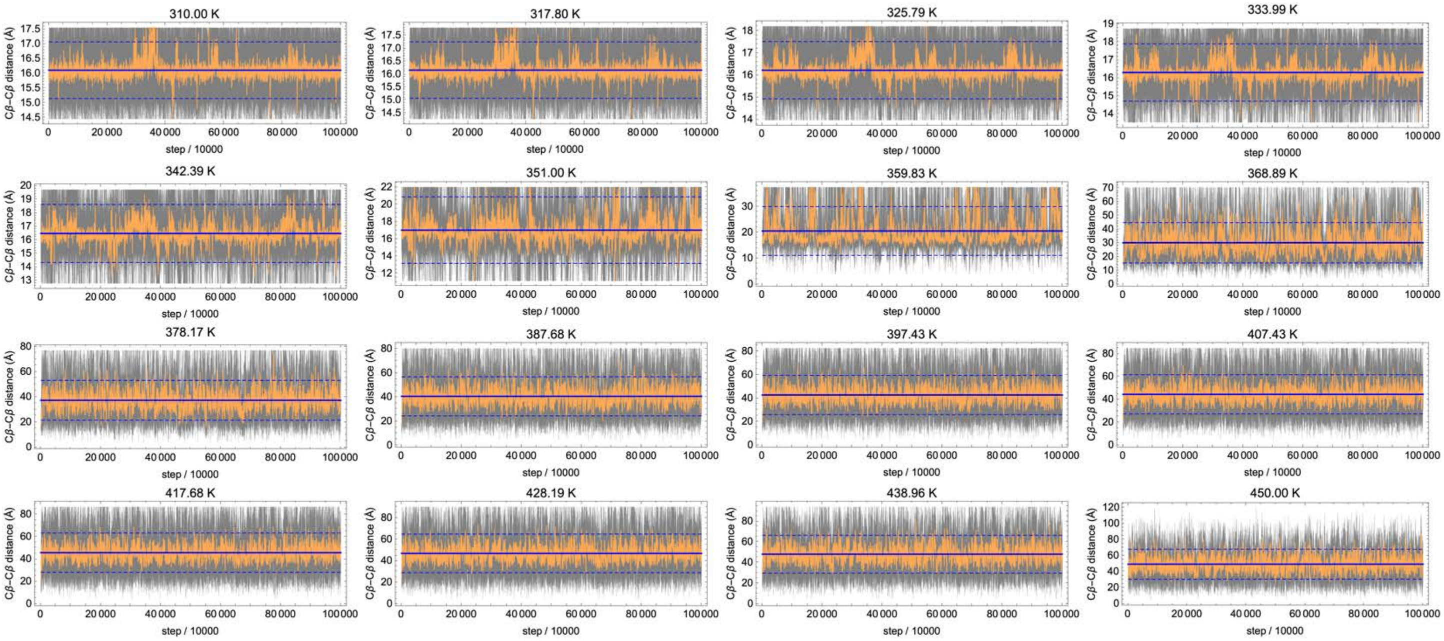

Temp (K)	Average Cβ–Cβ distance (Å)	Block Standard Error (Å)	Standard Deviation (Å)	Slope (Å / μs)
310.00	16.1086	0.0226003	0.960281	−0.0231745
317.80	16.1587	0.028131	1.09322	−0.0270226
325.79	16.2252	0.0382505	1.29611	−0.041092
333.99	16.3084	0.0471432	1.59297	−0.027143
342.39	16.4713	0.0590489	2.14182	0.0040012
351.00	17.0297	0.0956344	3.84631	0.202211
359.83	20.5664	0.460401	9.4268	0.6705
368.89	30.2211	0.451028	14.6707	0.683114
378.17	37.2961	0.343417	15.8288	0.667601
387.68	40.6318	0.262468	16.1948	0.608423
397.43	42.7665	0.232833	16.738	0.43184
407.43	44.4436	0.241461	17.1547	0.46542
417.68	45.7348	0.250679	17.5606	0.535133
428.19	46.9013	0.286084	17.9859	0.760294
438.96	48.0793	0.306144	18.323	0.679455
450.00	49.2309	0.325733	18.7056	0.695093

Figure S463. Plots of Cβ-Cβ distances for T-REMD simulations of variant **7f/10b'-xx** at 310.00, 317.80, 325.79, 333.99, 342.39, 351.00, 359.83, 368.89, 378.17, 387.68, 397.43, 407.43, 417.68, 428.19, 438.96, and 450.00 K.

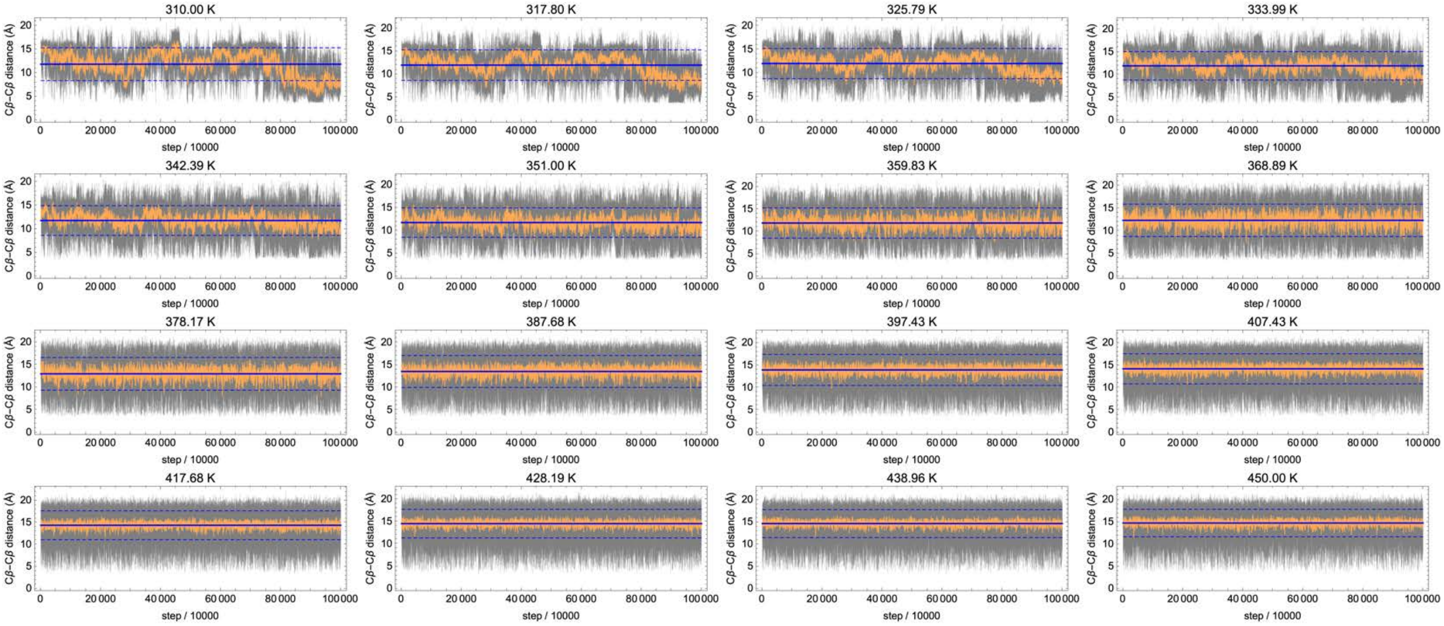

Temp (K)	Average Cβ–Cβ distance (Å)	Block Standard Error (Å)	Standard Deviation (Å)	Slope (Å / μs)
310.00	11.8349	0.392612	3.47339	−2.06379
317.80	11.9692	0.313004	3.31871	−1.75959
325.79	11.9967	0.241257	3.19379	−1.401
333.99	11.9326	0.181617	3.125	−1.07911
342.39	11.8024	0.139695	3.138	−0.822491
351.00	11.7345	0.103813	3.21227	−0.575518
359.83	11.8791	0.0696219	3.37738	−0.30896
368.89	12.3291	0.0468106	3.5664	−0.0966428
378.17	12.9783	0.0365002	3.61875	0.0142051
387.68	13.5542	0.0298267	3.54579	0.0125058
397.43	13.9394	0.0250941	3.45114	−0.0144298
407.43	14.1907	0.0199526	3.35664	−0.00487221
417.68	14.3725	0.0183427	3.26872	−0.0279014
428.19	14.5251	0.0145375	3.19775	−0.048106
438.96	14.6448	0.0137893	3.14188	−0.0251106
450.00	14.7309	0.0163837	3.08776	−0.0406689

Figure S464. Plots of Cβ-Cβ distances for T-REMD simulations of variant **s7f/10b’-x4x** at 310.00, 317.80, 325.79, 333.99, 342.39, 351.00, 359.83, 368.89, 378.17, 387.68, 397.43, 407.43, 417.68, 428.19, 438.96, and 450.00 K.

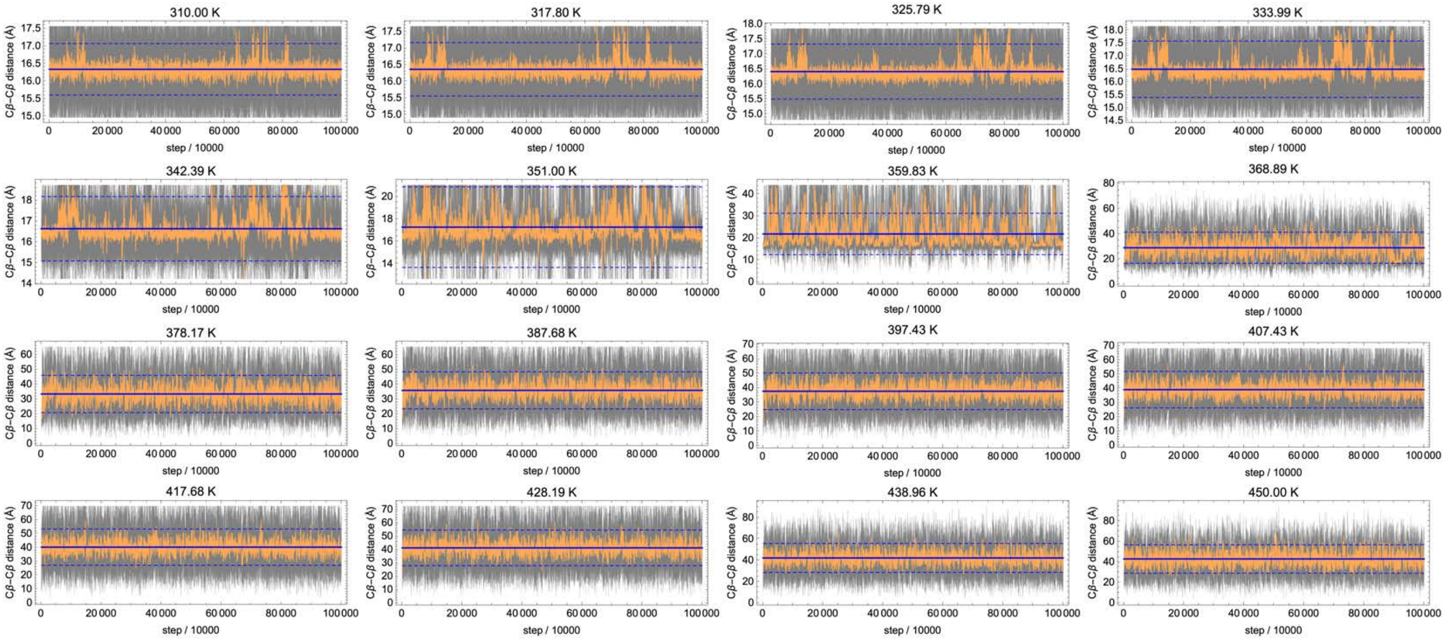

Temp (K)	Average Cβ–Cβ distance (Å)	Block Standard Error (Å)	Standard Deviation (Å)	Slope (Å / μs)
310.00	16.3398	0.0103641	0.731415	0.00600676
317.80	16.3665	0.0160755	0.806254	0.0103363
325.79	16.4166	0.0254581	0.915843	0.0393022
333.99	16.4929	0.0371746	1.09044	0.0448477
342.39	16.6448	0.055553	1.54448	0.0528573
351.00	17.2784	0.109589	3.59654	−0.155803
359.83	21.8815	0.369775	9.44094	−1.34548
368.89	29.0884	0.354314	12.2889	−0.770926
378.17	33.4718	0.199866	12.5497	−0.18828
387.68	36.0064	0.173592	12.5444	−0.214487
397.43	37.6688	0.165425	12.6259	−0.217193
407.43	39.1819	0.170411	12.8212	−0.205688
417.68	40.3144	0.18137	13.0801	−0.283536
428.19	41.3189	0.173518	13.3285	−0.152009
438.96	42.2323	0.191804	13.5621	−0.0835226
450.00	43.04	0.204072	13.8586	−0.0965857

Figure S465. Plots of Cβ-Cβ distances for T-REMD simulations of variant **14f/17b’-xx** at 310.00, 317.80, 325.79, 333.99, 342.39, 351.00, 359.83, 368.89, 378.17, 387.68, 397.43, 407.43, 417.68, 428.19, 438.96, and 450.00 K.

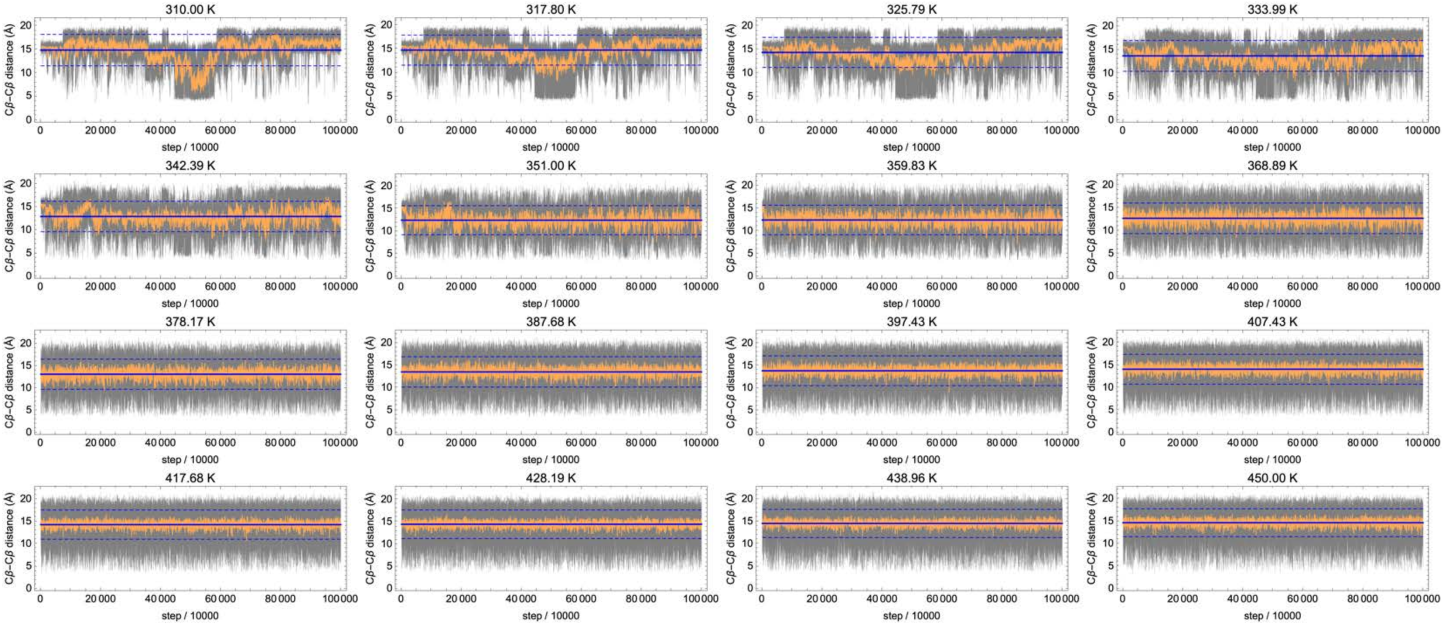

Temp (K)	Average Cβ–Cβ distance (Å)	Block Standard Error (Å)	Standard Deviation (Å)	Slope (Å / μs)
310.00	14.8463	0.36089	3.33114	0.531218
317.80	14.7308	0.266171	3.16919	0.578605
325.79	14.3384	0.230292	3.18461	0.598182
333.99	13.686	0.206529	3.28812	0.563835
342.39	12.9129	0.129471	3.28135	0.073427
351.00	12.4479	0.08432	3.22752	−0.196755
359.83	12.3991	0.0503446	3.25234	−0.171256
368.89	12.692	0.0330532	3.34324	−0.0284662
378.17	13.0884	0.0306006	3.38878	−0.00341783
387.68	13.5046	0.0270179	3.39057	−0.0156631
397.43	13.8301	0.0223336	3.34196	−0.0133258
407.43	14.0586	0.0203527	3.31364	−0.0515425
417.68	14.2711	0.0182128	3.25664	−0.0328148
428.19	14.4398	0.0155841	3.20361	−0.0317687
438.96	14.5588	0.0205161	3.14832	−0.0397085
450.00	14.6476	0.0170219	3.10056	−0.0490337

Figure S466. Plots of Cβ-Cβ distances for T-REMD simulations of variant **s14f/17b’-x4x** at 310.00, 317.80, 325.79, 333.99, 342.39, 351.00, 359.83, 368.89, 378.17, 387.68, 397.43, 407.43, 417.68, 428.19, 438.96, and 450.00 K.

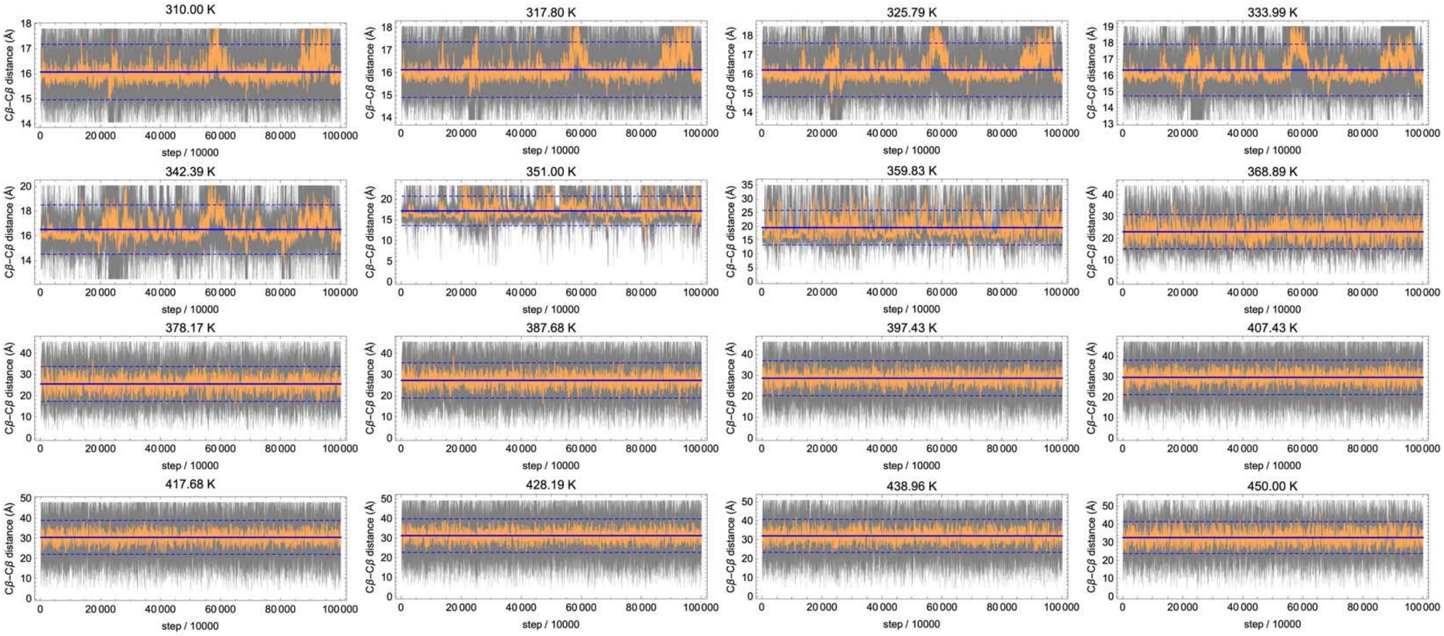

Temp (K)	Average Cβ–Cβ distance (Å)	Block Standard Error (Å)	Standard Deviation (Å)	Slope (Å / μs)
310.00	16.0832	0.0438723	1.10503	0.178286
317.80	16.1486	0.0574035	1.23006	0.221301
325.79	16.2361	0.0719939	1.39796	0.25381
333.99	16.3582	0.0856421	1.59421	0.231814
342.39	16.5374	0.0985416	1.99788	0.191092
351.00	17.2377	0.128841	3.56277	0.360116
359.83	19.6918	0.206528	6.28067	0.707849
368.89	23.172	0.150704	7.89048	0.472925
378.17	25.7006	0.106683	8.21727	0.0504601
387.68	27.4488	0.0908975	8.31082	−0.0313998
397.43	28.7948	0.0773168	8.3339	−0.00798063
407.43	29.8369	0.0621274	8.39258	−0.128847
417.68	30.6931	0.0777195	8.45699	0.0506053
428.19	31.4704	0.0765677	8.55288	−0.0163705
438.96	32.2016	0.0919801	8.69414	0.0782102
450.00	32.7846	0.0961445	8.79753	0.05806

Figure S467. Plots of Cβ-Cβ distances for T-REMD simulations of variant **21f/24b'-xx** at 310.00, 317.80, 325.79, 333.99, 342.39, 351.00, 359.83, 368.89, 378.17, 387.68, 397.43, 407.43, 417.68, 428.19, 438.96, and 450.00 K.

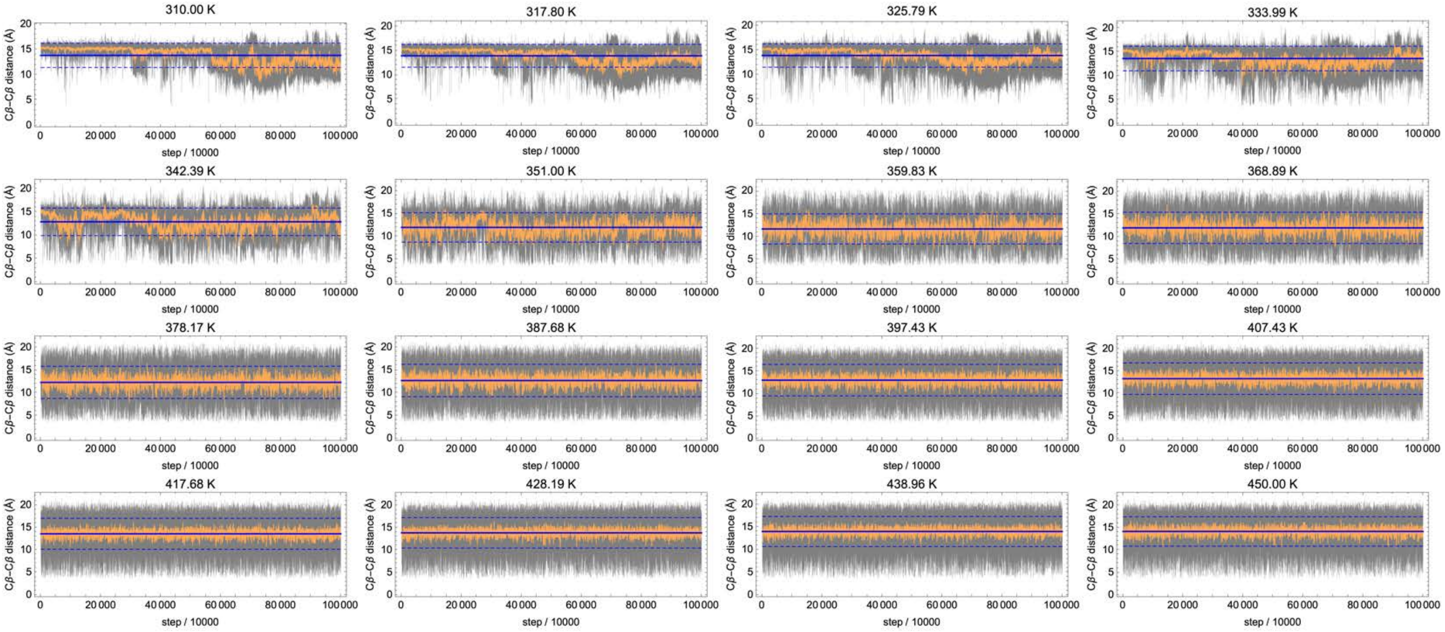

Temp (K)	Average Cβ–Cβ distance (Å)	Block Standard Error (Å)	Standard Deviation (Å)	Slope (Å / μs)
310.00	13.8152	0.237973	2.39543	−1.92298
317.80	13.9379	0.198062	2.34357	−1.50093
325.79	13.8979	0.172825	2.38337	−1.19345
333.99	13.6022	0.165517	2.58239	−0.846593
342.39	12.8915	0.18168	2.95259	−0.447691
351.00	11.972	0.124798	3.21707	−0.1319
359.83	11.7022	0.0694768	3.33777	−0.106178
368.89	11.9446	0.0548677	3.45345	−0.141084
378.17	12.2953	0.0426766	3.53633	−0.105686
387.68	12.6855	0.0312338	3.55385	−0.0333934
397.43	13.0228	0.0232758	3.52421	−0.00493232
407.43	13.3111	0.0231433	3.51644	−0.0413705
417.68	13.5766	0.0159444	3.44768	−0.00868001
428.19	13.8236	0.0174678	3.39872	−0.00188848
438.96	13.9903	0.0162797	3.33136	−0.0204675
450.00	14.1377	0.0189837	3.28461	−0.00919685

Figure S468. Plots of Cβ-Cβ distances for T-REMD simulations of variant **s21f/24b'-x4x** at 310.00, 317.80, 325.79, 333.99, 342.39, 351.00, 359.83, 368.89, 378.17, 387.68, 397.43, 407.43, 417.68, 428.19, 438.96, and 450.00 K.

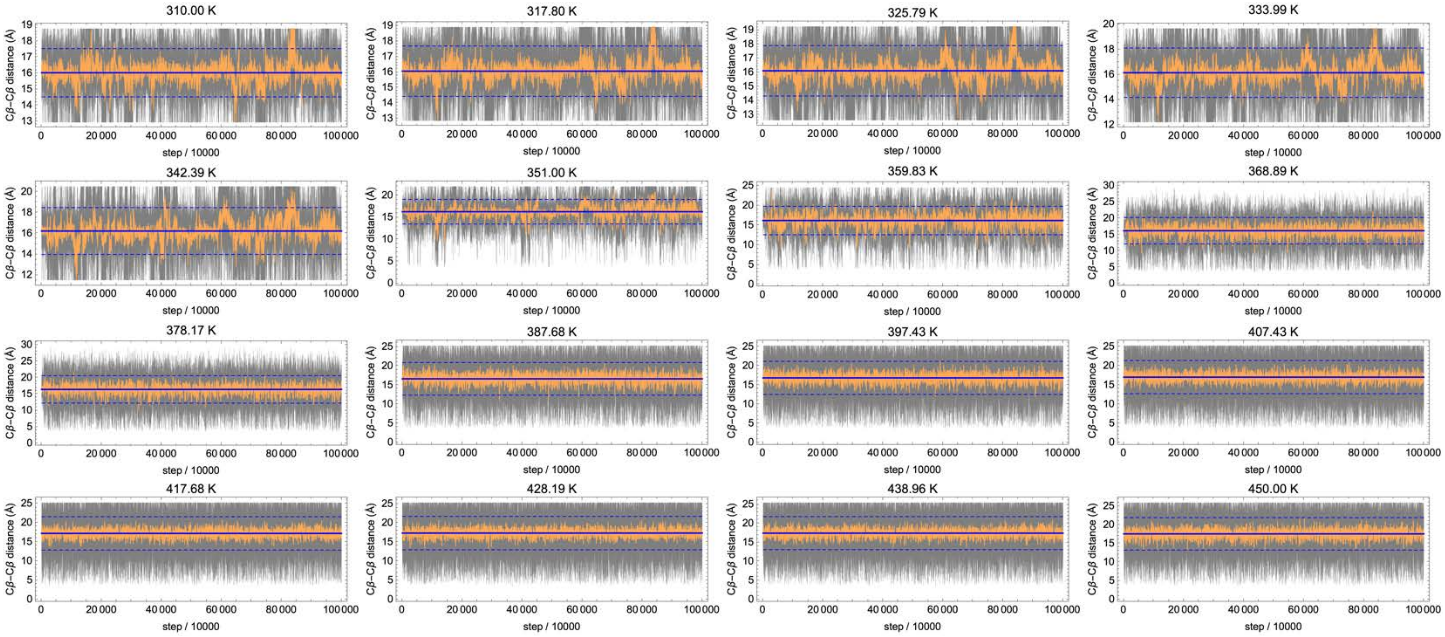

Temp (K)	Average Cβ–Cβ distance (Å)	Block Standard Error (Å)	Standard Deviation (Å)	Slope (Å / μs)
310.00	16.0255	0.049365	1.50417	0.0189155
317.80	16.059	0.0561115	1.63056	0.086987
325.79	16.1074	0.0620811	1.78734	0.131002
333.99	16.1447	0.0734647	1.9642	0.228158
342.39	16.2312	0.0883966	2.25015	0.317074
351.00	16.2767	0.100242	2.79057	0.386014
359.83	16.1711	0.084169	3.59807	0.0231765
368.89	16.2245	0.0528482	4.04675	−0.0112391
378.17	16.4268	0.0386174	4.18313	−0.107148
387.68	16.6337	0.0379535	4.26055	−0.128887
397.43	16.8482	0.0355011	4.30715	−0.0723717
407.43	17.0322	0.024938	4.33261	−0.0459051
417.68	17.2183	0.0255795	4.33769	0.0382159
428.19	17.3394	0.0245953	4.35728	0.0497292
438.96	17.4399	0.0227016	4.34961	−0.00644968
450.00	17.5677	0.024886	4.34615	0.0395567

Figure S469. Plots of Cβ-Cβ distances for T-REMD simulations of variant **28f/31b'-xx** at 310.00, 317.80, 325.79, 333.99, 342.39, 351.00, 359.83, 368.89, 378.17, 387.68, 397.43, 407.43, 417.68, 428.19, 438.96, and 450.00 K.

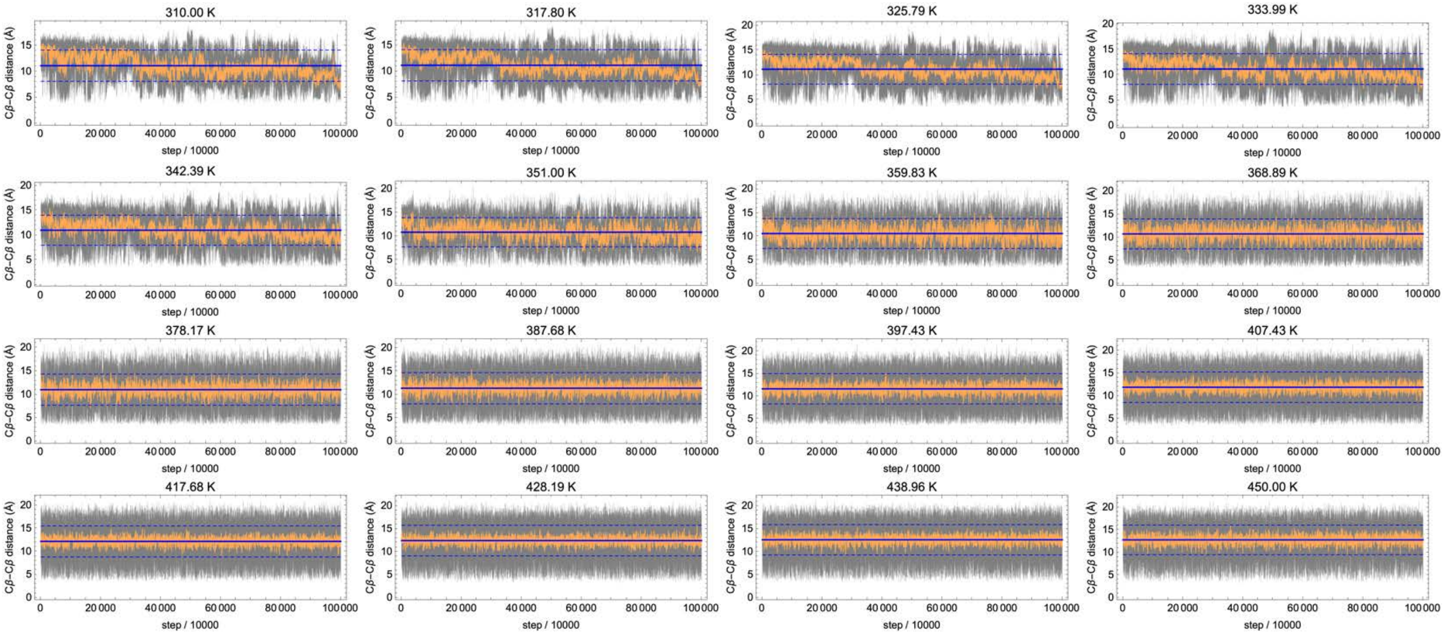

Temp (K)	Average Cβ–Cβ distance (Å)	Block Standard Error (Å)	Standard Deviation (Å)	Slope (Å / μs)
310.00	11.114	0.249205	3.01976	−2.02366
317.80	11.1547	0.23431	3.01047	−1.95812
325.79	11.1536	0.215124	3.00543	−1.80917
333.99	11.1338	0.200157	3.02552	−1.64158
342.39	11.0156	0.168398	3.03076	−1.32283
351.00	10.8229	0.132819	3.07054	−0.881987
359.83	10.6196	0.0858197	3.18358	−0.307702
368.89	10.7384	0.0438642	3.25673	−0.0964541
378.17	11.0127	0.0321494	3.3244	−0.0720079
387.68	11.3725	0.0236903	3.34433	−0.055526
397.43	11.646	0.0210849	3.3685	−0.0442958
407.43	11.9122	0.0168929	3.3531	−0.0725954
417.68	12.1904	0.01962	3.35178	−0.076559
428.19	12.4258	0.0175431	3.33186	−0.0252424
438.96	12.6171	0.0159961	3.30314	0.0352903
450.00	12.7859	0.0218932	3.27566	0.051225

Figure S470. Plots of Cβ-Cβ distances for T-REMD simulations of variant **s28f/31b'-x4x** at 310.00, 317.80, 325.79, 333.99, 342.39, 351.00, 359.83, 368.89, 378.17, 387.68, 397.43, 407.43, 417.68, 428.19, 438.96, and 450.00 K.

(return to Table of Contents)

13. Fitting the RMSD and residue packing distances to obtain T_m and ΔG for each variant

We plotted these average trajectory RMSD values and residue packing distances 2a/2a', 5d/5d', 9a/9a', 12d/12d', 16a/16a', 19d/19d', 23a/23a', 26d/26d', and 30a/30a' as a function of temperature for each variant. We fit these data globally to a model for a two-state monomer folding/unfolding equilibrium using the program Mathematica.[10] This model is described in equations S1–S10:

$$Average\ Trajectory\ RMSD = \frac{[K\cdot(rmsfi+rmsfs\cdot T)+(rmsui+rmsus\cdot T)]}{1+K}, \quad (S1)$$

$$Average\ 2a/2a'\ Packing\ Distance = \frac{[K\cdot(aa1fi+aa1fs\cdot T)+(aa1ui+aa1us\cdot T)]}{1+K}, \quad (S2)$$

$$Average\ 5d/5d'\ Packing\ Distance = \frac{[K\cdot(dd1fi+dd1fs\cdot T)+(dd1ui+dd1us\cdot T)]}{1+K}, \quad (S3)$$

$$Average\ 9a/9a'\ Packing\ Distance = \frac{[K\cdot(aa2fi+aa2fs\cdot T)+(aa2ui+aa2us\cdot T)]}{1+K}, \quad (S4)$$

$$Average\ 12d/12d'\ Packing\ Distance = \frac{[K\cdot(dd2fi+dd2fs\cdot T)+(dd2ui+dd2us\cdot T)]}{1+K}, \quad (S5)$$

$$Average\ 16a/16a'\ Packing\ Distance = \frac{[K\cdot(aa3fi+aa3fs\cdot T)+(aa3ui+aa3us\cdot T)]}{1+K}, \quad (S6)$$

$$Average\ 19d/19d'\ Packing\ Distance = \frac{[K\cdot(dd3fi+dd3fs\cdot T)+(dd3ui+dd3us\cdot T)]}{1+K}, \quad (S7)$$

$$Average\ 23a/23a'\ Packing\ Distance = \frac{[K\cdot(aa4fi+aa4fs\cdot T)+(aa4ui+aa4us\cdot T)]}{1+K}, \quad (S8)$$

$$Average\ 26d/26d'\ Packing\ Distance = \frac{[K\cdot(dd4fi+dd4fs\cdot T)+(dd4ui+dd4us\cdot T)]}{1+K}, \quad (S9)$$

$$Average\ 30a/30a'\ Packing\ Distance = \frac{[K\cdot(aa5fi+aa5fs\cdot T)+(aa5ui+aa5us\cdot T)]}{1+K}, \quad (S10)$$

where T is temperature in Kelvin; rmsfi and rmsfs are the *y*-intercept and slope of the folded pre-transition baseline and rmsui and rmsus are the *y*-intercept and slope of the unfolded post-transition baseline for the average trajectory RMSD data; aa1fi and aa1fs are the *y*-intercept and slope of the folded pre-transition baseline and aa1ui and aa1us are the *y*-intercept and slope of the unfolded post-transition baseline for the average trajectory 2a/2a' packing distance; dd1fi and dd1fs are the *y*-intercept and slope of the folded pre-transition baseline and dd1ui and dd1us are the *y*-intercept and slope of the unfolded post-transition baseline for the average trajectory 5d/5d' packing distance; aa2fi and aa2fs are the *y*-intercept and slope of the folded pre-transition baseline and aa2ui and aa2us are the *y*-intercept and slope of the unfolded post-transition baseline for the average trajectory 9a/9a' packing distance; dd2fi and dd2fs are the *y*-intercept and slope of the folded pre-transition baseline and dd2ui and dd2us are the *y*-intercept and slope of the unfolded post-transition baseline for the average trajectory 12d/12d' packing distance; aa3fi and aa3fs are the *y*-intercept and slope of the folded pre-transition baseline and aa3ui and aa3us are the *y*-intercept and slope of the unfolded post-transition baseline for the average trajectory 16a/16a' packing distance; dd3fi and dd3fs are the *y*-intercept and slope of the folded pre-transition baseline and dd3ui and dd3us are the *y*-intercept and slope of the unfolded post-transition baseline for the average trajectory 19d/19d'

packing distance; aa4fi and aa4fs are the *y*-intercept and slope of the folded pre-transition baseline and aa4ui and aa4us are the *y*-intercept and slope of the unfolded post-transition baseline for the average trajectory 23a/23a' packing distance; dd4fi and dd4fs are the *y*-intercept and slope of the folded pre-transition baseline and dd4ui and dd4us are the *y*-intercept and slope of the unfolded post-transition baseline for the average trajectory 26d/26d' packing distance; aa5fi and aa5fs are the *y*-intercept and slope of the folded pre-transition baseline and aa5ui and aa5us are the *y*-intercept and slope of the unfolded post-transition baseline for the average trajectory 30a/30a' packing distance; and K is the temperature-dependent folding equilibrium constant. K is related to the temperature-dependent free energy of folding ΔG according to equation S11:

$$K = \exp\left[-\frac{\Delta \mathrm{G}}{\mathrm{RT}}\right], \tag{S11}$$

where R is the universal gas constant (0.0019872 kcal/mol/K), and ΔG is also a function of temperature, as shown in equation S12:

$$\Delta \mathrm{G} = \frac{\Delta \mathrm{H}_0 \cdot (\mathrm{T_m} - \mathrm{T})}{\mathrm{T_m}} + \Delta \mathrm{C_p} \cdot \left(\mathrm{T} - \mathrm{T_m} - \mathrm{T} \cdot \ln\left[\frac{\mathrm{T}}{\mathrm{T_m}}\right]\right), \tag{S12}$$

where T_m is the midpoint of the unfolding transition and the temperature at which $\Delta G_f = 0$; ΔH_0 is the change in enthalpy upon folding at $T = T_m$; and ΔC_p is the change in heat capacity upon folding. We used the fit parameters for each variant to calculate the ΔG values given in the main text; we calculated the uncertainty for each ΔG value by propagation of error using the standard errors of the fit parameters. Plots of average trajectory RMSD and average trajectory core packing distances as a function of temperature for each variant are shown in Figures S433–S450, along with the fit parameters ± standard error and statistics.

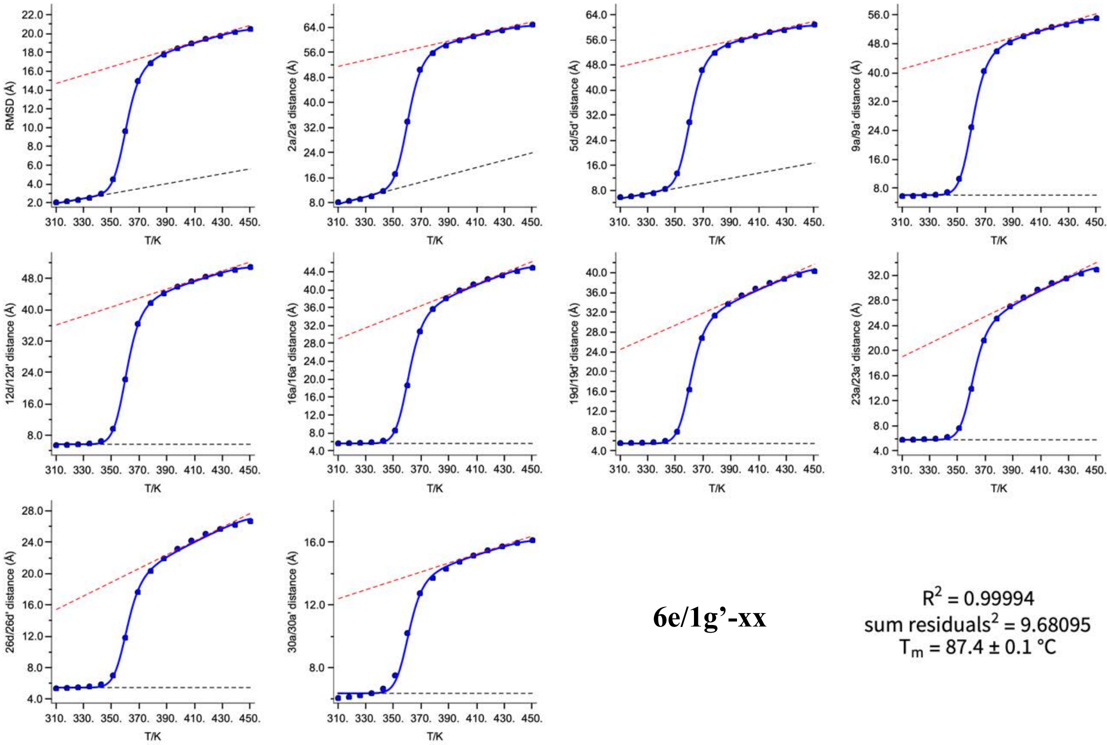

Parameters	Values	Units	P–Values
ΔH_0	–51.71 ± 0.70	kcal/mol	<0.001
T_m	87.4 ± 0.1	°C	---
ΔC_p	0.952 ± 0.023	kcal/mol/K	<0.001
rmsfi	3.32 ± 0.27	deg cm^{-2} dmol^{-1} K^{-1} x 10^{-3}	<0.001
rmsfs	0.026 ± 0.008	deg cm^{-2} dmol^{-1} x 10^{-3}	0.001
rmsui	16.98 ± 0.22	deg cm^{-2} dmol^{-1} x 10^{-3}	<0.001
rmsus	0.044 ± 0.004	deg cm^{-2} dmol^{-1} x 10^{-3}	<0.001
aa1fi	13.68 ± 0.29	deg cm^{-2} dmol^{-1} K^{-1} x 10^{-3}	<0.001
aa1fs	0.117 ± 0.008	deg cm^{-2} dmol^{-1} x 10^{-3}	<0.001
aa1ui	56.81 ± 0.26	deg cm^{-2} dmol^{-1} x 10^{-3}	<0.001
aa1us	0.102 ± 0.005	deg cm^{-2} dmol^{-1} x 10^{-3}	<0.001
dd1fi	9.63 ± 0.29	deg cm^{-2} dmol^{-1} K^{-1} x 10^{-3}	<0.001
dd1fs	0.081 ± 0.008	deg cm^{-2} dmol^{-1} x 10^{-3}	<0.001
dd1ui	52.81 ± 0.27	deg cm^{-2} dmol^{-1} x 10^{-3}	<0.001
dd1us	0.104 ± 0.005	deg cm^{-2} dmol^{-1} x 10^{-3}	<0.001
aa2fi	6.26 ± 0.12	deg cm^{-2} dmol^{-1} K^{-1} x 10^{-3}	<0.001
aa2fs	---	---	---
aa2ui	46.65 ± 0.26	deg cm^{-2} dmol^{-1} x 10^{-3}	<0.001
aa2us	0.109 ± 0.005	deg cm^{-2} dmol^{-1} x 10^{-3}	<0.001
dd2fi	5.81 ± 0.12	deg cm^{-2} dmol^{-1} K^{-1} x 10^{-3}	<0.001
dd2fs	---	---	---
dd2ui	42.07 ± 0.25	deg cm^{-2} dmol^{-1} x 10^{-3}	<0.001
dd2us	0.115 ± 0.005	deg cm^{-2} dmol^{-1} x 10^{-3}	<0.001
aa3fi	5.77 ± 0.12	deg cm^{-2} dmol^{-1} K^{-1} x 10^{-3}	<0.001
aa3fs	---	---	---
aa3ui	35.37 ± 0.24	deg cm^{-2} dmol^{-1} x 10^{-3}	<0.001
aa3us	0.123 ± 0.004	deg cm^{-2} dmol^{-1} x 10^{-3}	<0.001
dd3fi	5.59 ± 0.12	deg cm^{-2} dmol^{-1} K^{-1} x 10^{-3}	<0.001
dd3fs	---	---	---
dd3ui	30.83 ± 0.23	deg cm^{-2} dmol^{-1} x 10^{-3}	<0.001
dd3us	0.123 ± 0.004	deg cm^{-2} dmol^{-1} x 10^{-3}	<0.001
aa4fi	5.85 ± 0.12	deg cm^{-2} dmol^{-1} K^{-1} x 10^{-3}	<0.001
aa4fs	---	---	---
aa4ui	24.53 ± 0.22	deg cm^{-2} dmol^{-1} x 10^{-3}	<0.001
aa4us	0.107 ± 0.004	deg cm^{-2} dmol^{-1} x 10^{-3}	<0.001
dd4fi	5.51 ± 0.11	deg cm^{-2} dmol^{-1} K^{-1} x 10^{-3}	<0.001
dd4fs	---	---	---
dd4ui	19.90 ± 0.21	deg cm^{-2} dmol^{-1} x 10^{-3}	<0.001
dd4us	0.088 ± 0.004	deg cm^{-2} dmol^{-1} x 10^{-3}	<0.001
aa4fi	6.39 ± 0.11	deg cm^{-2} dmol^{-1} K^{-1} x 10^{-3}	<0.001
aa5fs	---	---	---
aa5ui	13.87 ± 0.20	deg cm^{-2} dmol^{-1} x 10^{-3}	<0.001
aa5us	0.029 ± 0.004	deg cm^{-2} dmol^{-1} x 10^{-3}	<0.001

Figure S471. Plots of average trajectory RMSD and average trajectory core distances as a function of temperature for variant **6e/1g'-xx**, along with the fit parameters ± standard error and statistics.

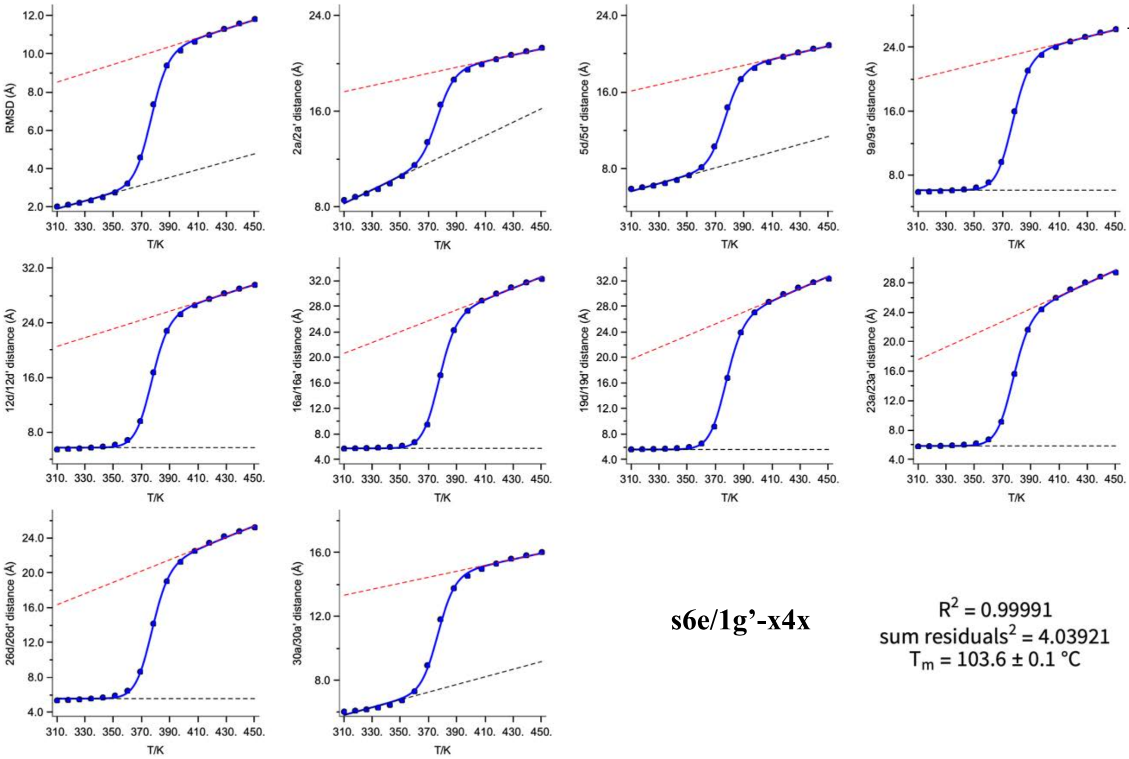

Parameters	Values	Units	P–Values
ΔH_0	–47.32 ± 0.67	kcal/mol	<0.001
T_m	103.6 ± 0.1	°C	<0.001
ΔC_p	---	---	---
rmsfi	3.30 ± 0.16	deg cm^{-2} dmol^{-1} K^{-1} x 10^{-3}	<0.001
rmsfs	0.020 ± 0.004	deg cm^{-2} dmol^{-1} x 10^{-3}	<0.001
rmsui	10.09 ± 0.16	deg cm^{-2} dmol^{-1} x 10^{-3}	<0.001
rmsus	0.023 ± 0.003	deg cm^{-2} dmol^{-1} x 10^{-3}	<0.001
aa1fi	12.12 ± 0.16	deg cm^{-2} dmol^{-1} K^{-1} x 10^{-3}	<0.001
aa1fs	0.057 ± 0.004	deg cm^{-2} dmol^{-1} x 10^{-3}	<0.001
aa1ui	19.38 ± 0.16	deg cm^{-2} dmol^{-1} x 10^{-3}	<0.001
aa1us	0.026 ± 0.003	deg cm^{-2} dmol^{-1} x 10^{-3}	<0.001
dd1fi	8.43 ± 0.16	deg cm^{-2} dmol^{-1} K^{-1} x 10^{-3}	<0.001
dd1fs	0.041 ± 0.004	deg cm^{-2} dmol^{-1} x 10^{-3}	<0.001
dd1ui	18.40 ± 0.16	deg cm^{-2} dmol^{-1} x 10^{-3}	<0.001
dd1us	0.034 ± 0.003	deg cm^{-2} dmol^{-1} x 10^{-3}	<0.001
aa2fi	6.16 ± 0.07	deg cm^{-2} dmol^{-1} K^{-1} x 10^{-3}	<0.001
aa2fs	---	---	---
aa2ui	23.07 ± 0.17	deg cm^{-2} dmol^{-1} x 10^{-3}	<0.001
aa2us	0.044 ± 0.003	deg cm^{-2} dmol^{-1} x 10^{-3}	<0.001
dd2fi	5.80 ± 0.07	deg cm^{-2} dmol^{-1} K^{-1} x 10^{-3}	<0.001
dd2fs	---	---	---
dd2ui	24.93 ± 0.17	deg cm^{-2} dmol^{-1} x 10^{-3}	<0.001
dd2us	0.065 ± 0.003	deg cm^{-2} dmol^{-1} x 10^{-3}	<0.001
aa3fi	5.82 ± 0.07	deg cm^{-2} dmol^{-1} K^{-1} x 10^{-3}	<0.001
aa3fs	---	---	---
aa3ui	26.42 ± 0.18	deg cm^{-2} dmol^{-1} x 10^{-3}	<0.001
aa3us	0.086 ± 0.004	deg cm^{-2} dmol^{-1} x 10^{-3}	<0.001
dd3fi	5.65 ± 0.07	deg cm^{-2} dmol^{-1} K^{-1} x 10^{-3}	<0.001
dd3fs	---	---	---
dd3ui	25.98 ± 0.18	deg cm^{-2} dmol^{-1} x 10^{-3}	<0.001
dd3us	0.093 ± 0.004	deg cm^{-2} dmol^{-1} x 10^{-3}	<0.001
aa4fi	5.91 ± 0.07	deg cm^{-2} dmol^{-1} K^{-1} x 10^{-3}	<0.001
aa4fs	---	---	---
aa4ui	23.41 ± 0.17	deg cm^{-2} dmol^{-1} x 10^{-3}	<0.001
aa4us	0.087 ± 0.003	deg cm^{-2} dmol^{-1} x 10^{-3}	<0.001
dd4fi	5.61 ± 0.07	deg cm^{-2} dmol^{-1} K^{-1} x 10^{-3}	<0.001
dd4fs	---	---	---
dd4ui	20.72 ± 0.16	deg cm^{-2} dmol^{-1} x 10^{-3}	<0.001
dd4us	0.065 ± 0.003	deg cm^{-2} dmol^{-1} x 10^{-3}	<0.001
aa4fi	7.45 ± 0.16	deg cm^{-2} dmol^{-1} K^{-1} x 10^{-3}	<0.001
aa5fs	0.024 ± 0.004	deg cm^{-2} dmol^{-1} x 10^{-3}	<0.001
aa5ui	14.61 ± 0.16	deg cm^{-2} dmol^{-1} x 10^{-3}	<0.001
aa5us	0.019 ± 0.003	deg cm^{-2} dmol^{-1} x 10^{-3}	<0.001

Figure S472. Plots of average trajectory RMSD and average trajectory core distances as a function of temperature for variant **s6e/1g'-x4x**, along with the fit parameters ± standard error and statistics.

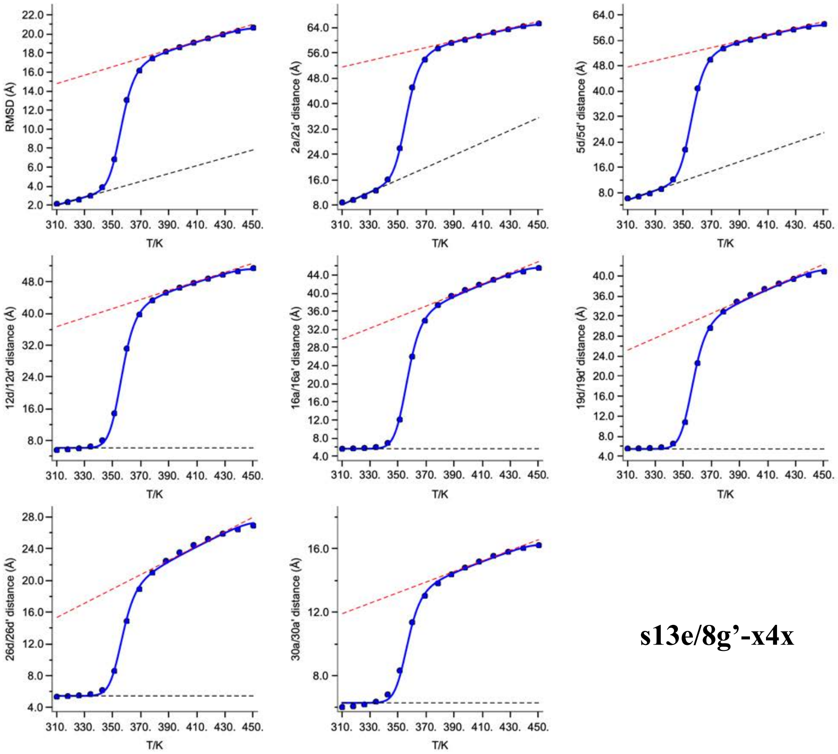

s13e/8g'-x4x

R^2 = 0.99993
sum residuals^2 = 11.31004
T_m = 82.9 ± 0.1 °C

Parameters	Values	Units	P–Values
ΔH_0	−53.02 ± 0.74	kcal/mol	<0.001
T_m	82.9 ± 0.1	°C	<0.001
ΔC_p	0.963 ± 0.023	kcal/mol/K	<0.001
rmsfi	3.99 ± 0.30	deg cm^{-2} dmol^{-1} K^{-1} x 10^{-3}	<0.001
rmsfs	0.041 ± 0.010	deg cm^{-2} dmol^{-1} x 10^{-3}	<0.001
rmsui	16.89 ± 0.22	deg cm^{-2} dmol^{-1} x 10^{-3}	<0.001
rmsus	0.045 ± 0.004	deg cm^{-2} dmol^{-1} x 10^{-3}	<0.001
aa1fi	17.39 ± 0.33	deg cm^{-2} dmol^{-1} K^{-1} x 10^{-3}	<0.001
aa1fs	0.196 ± 0.010	deg cm^{-2} dmol^{-1} x 10^{-3}	<0.001
aa1ui	56.45 ± 0.26	deg cm^{-2} dmol^{-1} x 10^{-3}	<0.001
aa1us	0.103 ± 0.004	deg cm^{-2} dmol^{-1} x 10^{-3}	<0.001
dd1fi	12.80 ± 0.33	deg cm^{-2} dmol^{-1} K^{-1} x 10^{-3}	<0.001
dd1fs	0.152 ± 0.010	deg cm^{-2} dmol^{-1} x 10^{-3}	<0.001
dd1ui	52.45 ± 0.26	deg cm^{-2} dmol^{-1} x 10^{-3}	<0.001
dd1us	0.102 ± 0.004	deg cm^{-2} dmol^{-1} x 10^{-3}	<0.001
aa2fi	8.00 ± 0.32	deg cm^{-2} dmol^{-1} K^{-1} x 10^{-3}	<0.001
aa2fs	0.048 ± 0.010	deg cm^{-2} dmol^{-1} x 10^{-3}	<0.001
aa2ui	46.03 ± 0.26	deg cm^{-2} dmol^{-1} x 10^{-3}	<0.001
aa2us	0.113 ± 0.005	deg cm^{-2} dmol^{-1} x 10^{-3}	<0.001
dd2fi	6.24 ± 0.13	deg cm^{-2} dmol^{-1} K^{-1} x 10^{-3}	<0.001
dd2fs	---	---	---
dd2ui	42.10 ± 0.25	deg cm^{-2} dmol^{-1} x 10^{-3}	<0.001
dd2us	0.114 ± 0.005	deg cm^{-2} dmol^{-1} x 10^{-3}	<0.001
aa3fi	5.78 ± 0.13	deg cm^{-2} dmol^{-1} K^{-1} x 10^{-3}	<0.001
aa3fs	---	---	---
aa3ui	35.59 ± 0.24	deg cm^{-2} dmol^{-1} x 10^{-3}	<0.001
aa3us	0.122 ± 0.004	deg cm^{-2} dmol^{-1} x 10^{-3}	<0.001
dd3fi	5.57 ± 0.13	deg cm^{-2} dmol^{-1} K^{-1} x 10^{-3}	<0.001
dd3fs	---	---	---
dd3ui	30.96 ± 0.24	deg cm^{-2} dmol^{-1} x 10^{-3}	<0.001
dd3us	0.123 ± 0.004	deg cm^{-2} dmol^{-1} x 10^{-3}	<0.001
aa4fi	5.84 ± 0.13	deg cm^{-2} dmol^{-1} K^{-1} x 10^{-3}	<0.001
aa4fs	---	---	---
aa4ui	24.41 ± 0.23	deg cm^{-2} dmol^{-1} x 10^{-3}	<0.001
aa4us	0.108 ± 0.004	deg cm^{-2} dmol^{-1} x 10^{-3}	<0.001
dd4fi	5.51 ± 0.13	deg cm^{-2} dmol^{-1} K^{-1} x 10^{-3}	<0.001
dd4fs	---	---	---
dd4ui	19.57 ± 0.22	deg cm^{-2} dmol^{-1} x 10^{-3}	<0.001
dd4us	0.090 ± 0.004	deg cm^{-2} dmol^{-1} x 10^{-3}	<0.001
aa4fi	6.31 ± 0.13	deg cm^{-2} dmol^{-1} K^{-1} x 10^{-3}	<0.001
aa5fs	---	---	---
aa5ui	13.48 ± 0.21	deg cm^{-2} dmol^{-1} x 10^{-3}	<0.001
aa5us	0.033 ± 0.004	deg cm^{-2} dmol^{-1} x 10^{-3}	<0.001

Figure S473. Plots of average trajectory RMSD and average trajectory core distances as a function of temperature for variant **13e/8g'-xx**, along with the fit parameters ± standard error and statistics.

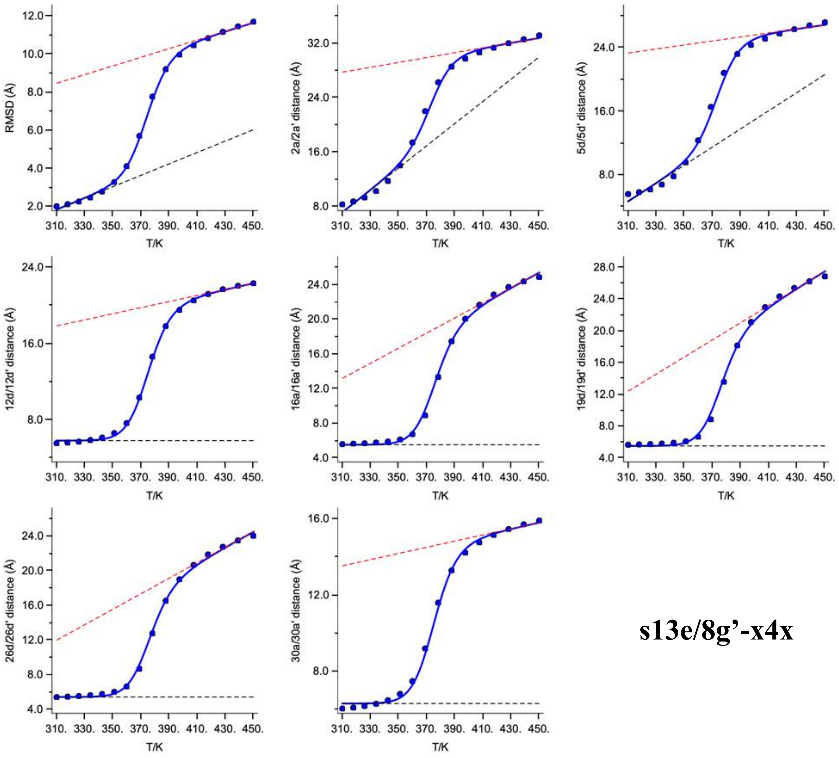

s13e/8g'-x4x

R^2 = 0.99954
sum residuals^2 = 18.90995
T_m = 101.7 ± 0.4 °C

Parameters	Values	Units	P–Values
ΔH_0	−35.32 ± 1.32	kcal/mol	<0.001
T_m	101.7 ± 0.4	°C	<0.001
ΔC_p	---	---	---
rmsfi	3.79 ± 0.38	deg cm^{-2} dmol^{-1} K^{-1} x 10^{-3}	<0.001
rmsfs	0.030 ± 0.009	deg cm^{-2} dmol^{-1} x 10^{-3}	<0.001
rmsui	9.95 ± 0.38	deg cm^{-2} dmol^{-1} x 10^{-3}	<0.001
rmsus	0.023 ± 0.008	deg cm^{-2} dmol^{-1} x 10^{-3}	0.003
aa1fi	17.76 ± 0.42	deg cm^{-2} dmol^{-1} K^{-1} x 10^{-3}	<0.001
aa1fs	0.163 ± 0.009	deg cm^{-2} dmol^{-1} x 10^{-3}	<0.001
aa1ui	30.09 ± 0.41	deg cm^{-2} dmol^{-1} x 10^{-3}	<0.001
aa1us	0.036 ± 0.008	deg cm^{-2} dmol^{-1} x 10^{-3}	<0.001
dd1fi	12.08 ± 0.41	deg cm^{-2} dmol^{-1} K^{-1} x 10^{-3}	<0.001
dd1fs	0.114 ± 0.009	deg cm^{-2} dmol^{-1} x 10^{-3}	<0.001
dd1ui	24.99 ± 0.42	deg cm^{-2} dmol^{-1} x 10^{-3}	<0.001
dd1us	0.025 ± 0.008	deg cm^{-2} dmol^{-1} x 10^{-3}	0.002
aa2fi	6.10 ± 0.15	deg cm^{-2} dmol^{-1} K^{-1} x 10^{-3}	<0.001
aa2fs	---	---	---
aa2ui	20.27 ± 0.40	deg cm^{-2} dmol^{-1} x 10^{-3}	<0.001
aa2us	0.021 ± 0.008	deg cm^{-2} dmol^{-1} x 10^{-3}	0.006
dd2fi	5.84 ± 0.15	deg cm^{-2} dmol^{-1} K^{-1} x 10^{-3}	<0.001
dd2fs	---	---	---
dd2ui	19.95 ± 0.40	deg cm^{-2} dmol^{-1} x 10^{-3}	<0.001
dd2us	0.032 ± 0.008	deg cm^{-2} dmol^{-1} x 10^{-3}	<0.001
aa3fi	5.55 ± 0.15	deg cm^{-2} dmol^{-1} K^{-1} x 10^{-3}	<0.001
aa3fs	---	---	---
aa3ui	18.86 ± 0.41	deg cm^{-2} dmol^{-1} x 10^{-3}	<0.001
aa3us	0.087 ± 0.008	deg cm^{-2} dmol^{-1} x 10^{-3}	<0.001
dd3fi	5.54 ± 0.15	deg cm^{-2} dmol^{-1} K^{-1} x 10^{-3}	<0.001
dd3fs	---	---	---
dd3ui	19.42 ± 0.43	deg cm^{-2} dmol^{-1} x 10^{-3}	<0.001
dd3us	0.108 ± 0.008	deg cm^{-2} dmol^{-1} x 10^{-3}	<0.001
aa4fi	5.74 ± 0.15	deg cm^{-2} dmol^{-1} K^{-1} x 10^{-3}	<0.001
aa4fs	---	---	---
aa4ui	18.80 ± 0.42	deg cm^{-2} dmol^{-1} x 10^{-3}	<0.001
aa4us	0.113 ± 0.008	deg cm^{-2} dmol^{-1} x 10^{-3}	<0.001
dd4fi	5.45 ± 0.15	deg cm^{-2} dmol^{-1} K^{-1} x 10^{-3}	<0.001
dd4fs	---	---	---
dd4ui	17.80 ± 0.41	deg cm^{-2} dmol^{-1} x 10^{-3}	<0.001
dd4us	0.089 ± 0.008	deg cm^{-2} dmol^{-1} x 10^{-3}	<0.001
aa4fi	6.32 ± 0.15	deg cm^{-2} dmol^{-1} K^{-1} x 10^{-3}	<0.001
aa5fs	---	---	---
aa5ui	14.59 ± 0.35	deg cm^{-2} dmol^{-1} x 10^{-3}	<0.001
aa5us	0.016 ± 0.007	deg cm^{-2} dmol^{-1} x 10^{-3}	0.023

Figure S474. Plots of average trajectory RMSD and average trajectory core distances as a function of temperature for variant **s13e/8g'-x4x**, along with the fit parameters ± standard error and statistics.

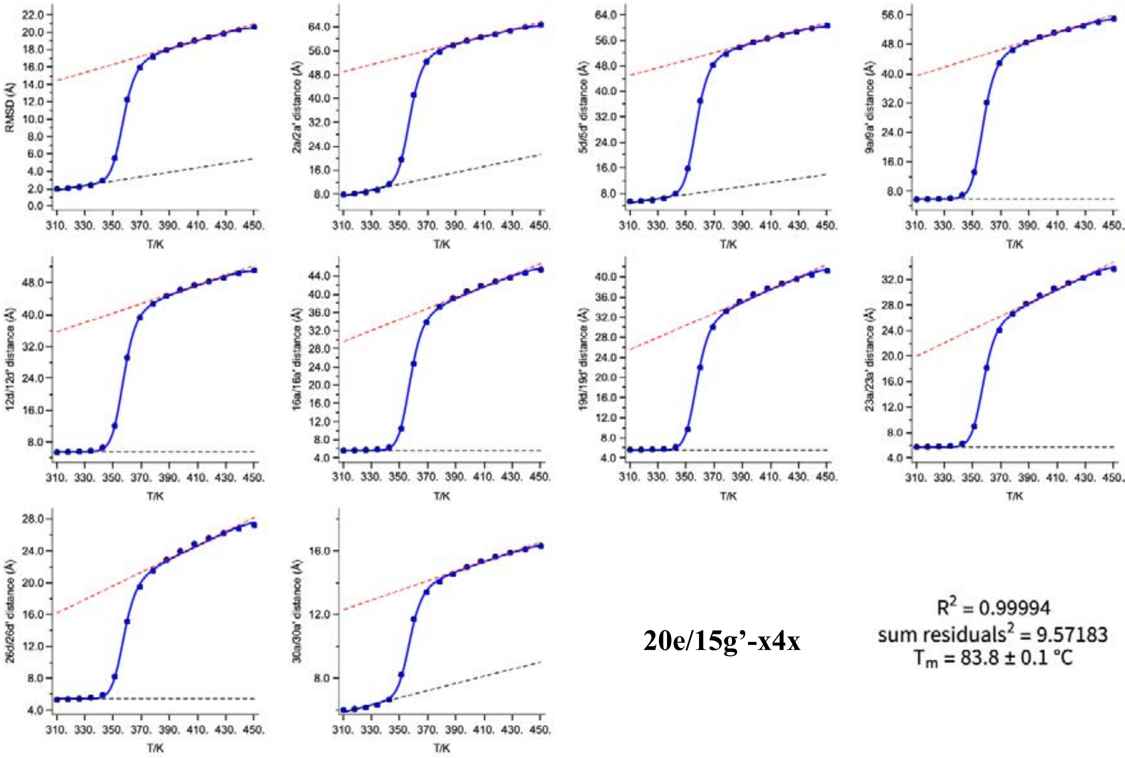

Parameters	Values	Units	P–Values
ΔH_0	–57.85 ± 0.70	kcal/mol	<0.001
T_m	83.8 ± 0.1	°C	---
ΔC_p	1.067 ± 0.022	kcal/mol/K	<0.001
rmsfi	3.10 ± 0.27	deg cm^{-2} dmol^{-1} K^{-1} x 10^{-3}	<0.001
rmsfs	0.026 ± 0.009	deg cm^{-2} dmol^{-1} x 10^{-3}	0.004
rmsui	16.69 ± 0.20	deg cm^{-2} dmol^{-1} x 10^{-3}	<0.001
rmsus	0.047 ± 0.004	deg cm^{-2} dmol^{-1} x 10^{-3}	<0.001
aa1fi	12.14 ± 0.29	deg cm^{-2} dmol^{-1} K^{-1} x 10^{-3}	<0.001
aa1fs	0.099 ± 0.009	deg cm^{-2} dmol^{-1} x 10^{-3}	<0.001
aa1ui	54.73 ± 0.23	deg cm^{-2} dmol^{-1} x 10^{-3}	<0.001
aa1us	0.119 ± 0.004	deg cm^{-2} dmol^{-1} x 10^{-3}	<0.001
dd1fi	8.19 ± 0.29	deg cm^{-2} dmol^{-1} K^{-1} x 10^{-3}	<0.001
dd1fs	0.063 ± 0.009	deg cm^{-2} dmol^{-1} x 10^{-3}	<0.001
dd1ui	50.74 ± 0.24	deg cm^{-2} dmol^{-1} x 10^{-3}	<0.001
dd1us	0.119 ± 0.004	deg cm^{-2} dmol^{-1} x 10^{-3}	<0.001
aa2fi	6.11 ± 0.12	deg cm^{-2} dmol^{-1} K^{-1} x 10^{-3}	<0.001
aa2fs	---	---	---
aa2ui	45.15 ± 0.23	deg cm^{-2} dmol^{-1} x 10^{-3}	<0.001
aa2us	0.119 ± 0.004	deg cm^{-2} dmol^{-1} x 10^{-3}	<0.001
dd2fi	5.72 ± 0.12	deg cm^{-2} dmol^{-1} K^{-1} x 10^{-3}	<0.001
dd2fs	---	---	---
dd2ui	41.38 ± 0.22	deg cm^{-2} dmol^{-1} x 10^{-3}	<0.001
dd2us	0.119 ± 0.004	deg cm^{-2} dmol^{-1} x 10^{-3}	<0.001
aa3fi	5.68 ± 0.12	deg cm^{-2} dmol^{-1} K^{-1} x 10^{-3}	<0.001
aa3fs	---	---	---
aa3ui	35.42 ± 0.22	deg cm^{-2} dmol^{-1} x 10^{-3}	<0.001
aa3us	0.122 ± 0.004	deg cm^{-2} dmol^{-1} x 10^{-3}	<0.001
dd3fi	5.61 ± 0.12	deg cm^{-2} dmol^{-1} K^{-1} x 10^{-3}	<0.001
dd3fs	---	---	---
dd3ui	31.34 ± 0.21	deg cm^{-2} dmol^{-1} x 10^{-3}	<0.001
dd3us	0.121 ± 0.004	deg cm^{-2} dmol^{-1} x 10^{-3}	<0.001
aa4fi	5.78 ± 0.12	deg cm^{-2} dmol^{-1} K^{-1} x 10^{-3}	<0.001
aa4fs	---	---	---
aa4ui	25.01 ± 0.20	deg cm^{-2} dmol^{-1} x 10^{-3}	<0.001
aa4us	0.105 ± 0.004	deg cm^{-2} dmol^{-1} x 10^{-3}	<0.001
dd4fi	5.49 ± 0.12	deg cm^{-2} dmol^{-1} K^{-1} x 10^{-3}	<0.001
dd4fs	---	---	---
dd4ui	20.28 ± 0.20	deg cm^{-2} dmol^{-1} x 10^{-3}	<0.001
dd4us	0.086 ± 0.004	deg cm^{-2} dmol^{-1} x 10^{-3}	<0.001
aa4fi	6.97 ± 0.27	deg cm^{-2} dmol^{-1} K^{-1} x 10^{-3}	<0.001
aa5fs	0.022 ± 0.009	deg cm^{-2} dmol^{-1} x 10^{-3}	0.010
aa5ui	13.76 ± 0.20	deg cm^{-2} dmol^{-1} x 10^{-3}	<0.001
aa5us	0.030 ± 0.003	deg cm^{-2} dmol^{-1} x 10^{-3}	<0.001

Figure S475. Plots of average trajectory RMSD and average trajectory core distances as a function of temperature for variant **20e/15g'-xx**, along with the fit parameters ± standard error and statistics.

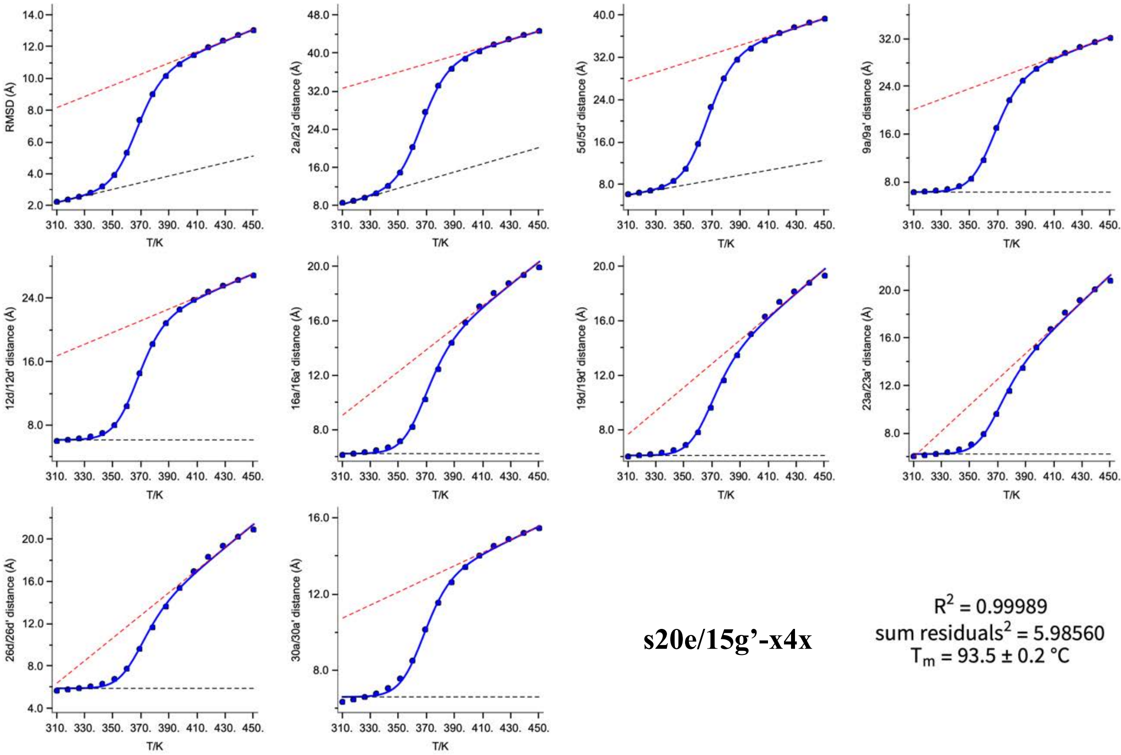

Parameters	Values	Units	P–Values
ΔH_0	–29.94 ± 0.60	kcal/mol	<0.001
T_m	93.5 ± 0.2	°C	<0.001
ΔC_p	---	---	---
rmsfi	3.41 ± 0.25	deg cm^{-2} dmol^{-1} K^{-1} x 10^{-3}	<0.001
rmsfs	0.021 ± 0.006	deg cm^{-2} dmol^{-1} x 10^{-3}	<0.001
rmsui	10.19 ± 0.22	deg cm^{-2} dmol^{-1} x 10^{-3}	<0.001
rmsus	0.035 ± 0.004	deg cm^{-2} dmol^{-1} x 10^{-3}	<0.001
aa1fi	13.17 ± 0.32	deg cm^{-2} dmol^{-1} K^{-1} x 10^{-3}	<0.001
aa1fs	0.085 ± 0.007	deg cm^{-2} dmol^{-1} x 10^{-3}	<0.001
aa1ui	37.57 ± 0.30	deg cm^{-2} dmol^{-1} x 10^{-3}	<0.001
aa1us	0.086 ± 0.005	deg cm^{-2} dmol^{-1} x 10^{-3}	<0.001
dd1fi	8.73 ± 0.31	deg cm^{-2} dmol^{-1} K^{-1} x 10^{-3}	<0.001
dd1fs	0.047 ± 0.007	deg cm^{-2} dmol^{-1} x 10^{-3}	<0.001
dd1ui	32.39 ± 0.30	deg cm^{-2} dmol^{-1} x 10^{-3}	<0.001
dd1us	0.085 ± 0.005	deg cm^{-2} dmol^{-1} x 10^{-3}	<0.001
aa2fi	6.33 ± 0.10	deg cm^{-2} dmol^{-1} K^{-1} x 10^{-3}	<0.001
aa2fs	---	---	---
aa2ui	25.19 ± 0.25	deg cm^{-2} dmol^{-1} x 10^{-3}	<0.001
aa2us	0.087 ± 0.004	deg cm^{-2} dmol^{-1} x 10^{-3}	<0.001
dd2fi	6.20 ± 0.10	deg cm^{-2} dmol^{-1} K^{-1} x 10^{-3}	<0.001
dd2fs	---	---	---
dd2ui	20.98 ± 0.22	deg cm^{-2} dmol^{-1} x 10^{-3}	<0.001
dd2us	0.074 ± 0.004	deg cm^{-2} dmol^{-1} x 10^{-3}	<0.001
aa3fi	6.28 ± 0.09	deg cm^{-2} dmol^{-1} K^{-1} x 10^{-3}	<0.001
aa3fs	---	---	---
aa3ui	13.67 ± 0.19	deg cm^{-2} dmol^{-1} x 10^{-3}	<0.001
aa3us	0.081 ± 0.003	deg cm^{-2} dmol^{-1} x 10^{-3}	<0.001
dd3fi	6.14 ± 0.09	deg cm^{-2} dmol^{-1} K^{-1} x 10^{-3}	<0.001
dd3fs	---	---	---
dd3ui	12.62 ± 0.19	deg cm^{-2} dmol^{-1} x 10^{-3}	<0.001
dd3us	0.087 ± 0.003	deg cm^{-2} dmol^{-1} x 10^{-3}	<0.001
aa4fi	6.29 ± 0.09	deg cm^{-2} dmol^{-1} K^{-1} x 10^{-3}	<0.001
aa4fs	---	---	---
aa4ui	12.23 ± 0.19	deg cm^{-2} dmol^{-1} x 10^{-3}	<0.001
aa4us	0.109 ± 0.003	deg cm^{-2} dmol^{-1} x 10^{-3}	<0.001
dd4fi	5.93 ± 0.09	deg cm^{-2} dmol^{-1} K^{-1} x 10^{-3}	<0.001
dd4fs	---	---	---
dd4ui	12.50 ± 0.19	deg cm^{-2} dmol^{-1} x 10^{-3}	<0.001
dd4us	0.107 ± 0.003	deg cm^{-2} dmol^{-1} x 10^{-3}	<0.001
aa4fi	6.62 ± 0.09	deg cm^{-2} dmol^{-1} K^{-1} x 10^{-3}	<0.001
aa5fs	---	---	---
aa5ui	12.71 ± 0.18	deg cm^{-2} dmol^{-1} x 10^{-3}	<0.001
aa5us	0.034 ± 0.003	deg cm^{-2} dmol^{-1} x 10^{-3}	<0.001

Figure S476. Plots of average trajectory RMSD and average trajectory core distances as a function of temperature for variant **s20e/15g'-x4x**, along with the fit parameters ± standard error and statistics.

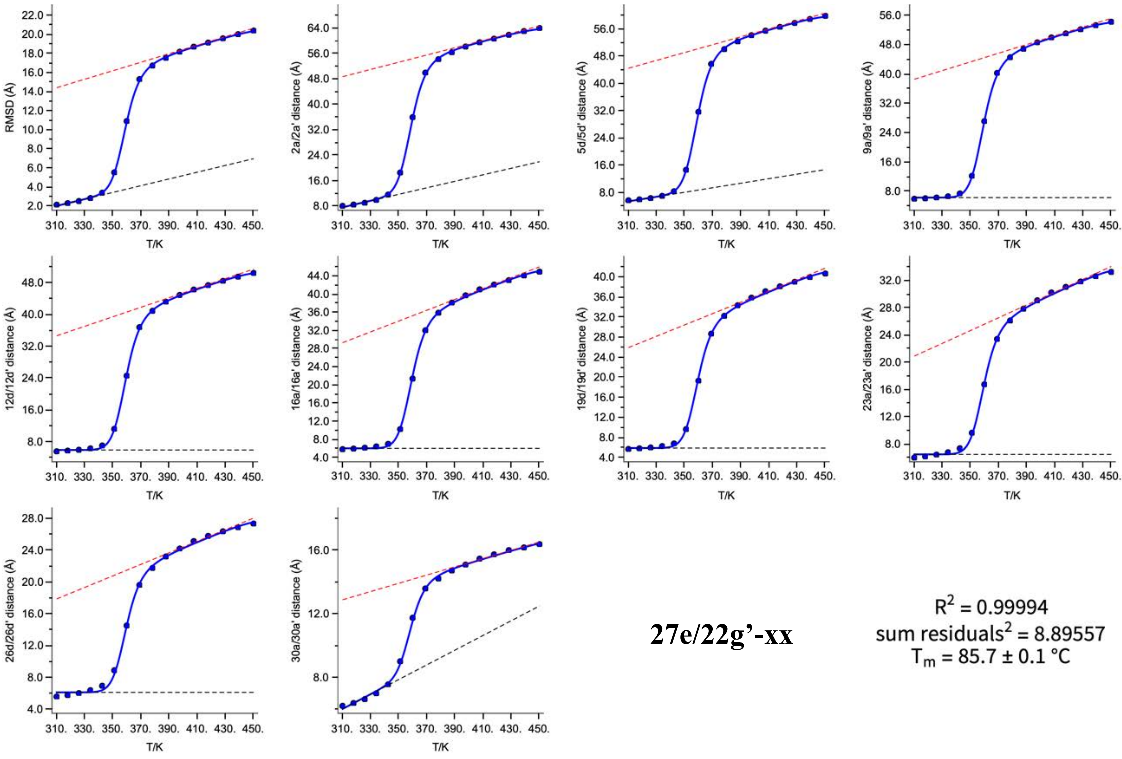

Parameters	Values	Units	P–Values
ΔH_0	–50.28 ± 0.64	kcal/mol	<0.001
T_m	85.7 ± 0.1	°C	<0.001
ΔC_p	0.881 ± 0.025	kcal/mol/K	<0.001
rmsfi	3.78 ± 0.26	deg cm^{-2} dmol^{-1} K^{-1} x 10^{-3}	<0.001
rmsfs	0.035 ± 0.008	deg cm^{-2} dmol^{-1} x 10^{-3}	<0.001
rmsui	16.62 ± 0.21	deg cm^{-2} dmol^{-1} x 10^{-3}	<0.001
rmsus	0.045 ± 0.004	deg cm^{-2} dmol^{-1} x 10^{-3}	<0.001
aa1fi	12.71 ± 0.29	deg cm^{-2} dmol^{-1} K^{-1} x 10^{-3}	<0.001
aa1fs	0.102 ± 0.009	deg cm^{-2} dmol^{-1} x 10^{-3}	<0.001
aa1ui	54.36 ± 0.25	deg cm^{-2} dmol^{-1} x 10^{-3}	<0.001
aa1us	0.114 ± 0.004	deg cm^{-2} dmol^{-1} x 10^{-3}	<0.001
dd1fi	8.73 ± 0.29	deg cm^{-2} dmol^{-1} K^{-1} x 10^{-3}	<0.001
dd1fs	0.067 ± 0.009	deg cm^{-2} dmol^{-1} x 10^{-3}	<0.001
dd1ui	50.19 ± 0.25	deg cm^{-2} dmol^{-1} x 10^{-3}	<0.001
dd1us	0.116 ± 0.005	deg cm^{-2} dmol^{-1} x 10^{-3}	<0.001
aa2fi	6.39 ± 0.12	deg cm^{-2} dmol^{-1} K^{-1} x 10^{-3}	<0.001
aa2fs	---	---	---
aa2ui	44.44 ± 0.24	deg cm^{-2} dmol^{-1} x 10^{-3}	<0.001
aa2us	0.119 ± 0.004	deg cm^{-2} dmol^{-1} x 10^{-3}	<0.001
dd2fi	6.00 ± 0.12	deg cm^{-2} dmol^{-1} K^{-1} x 10^{-3}	<0.001
dd2fs	---	---	---
dd2ui	40.59 ± 0.23	deg cm^{-2} dmol^{-1} x 10^{-3}	<0.001
dd2us	0.120 ± 0.004	deg cm^{-2} dmol^{-1} x 10^{-3}	<0.001
aa3fi	6.12 ± 0.11	deg cm^{-2} dmol^{-1} K^{-1} x 10^{-3}	<0.001
aa3fs	---	---	---
aa3ui	35.20 ± 0.22	deg cm^{-2} dmol^{-1} x 10^{-3}	<0.001
aa3us	0.119 ± 0.004	deg cm^{-2} dmol^{-1} x 10^{-3}	<0.001
dd3fi	5.97 ± 0.11	deg cm^{-2} dmol^{-1} K^{-1} x 10^{-3}	<0.001
dd3fs	---	---	---
dd3ui	31.51 ± 0.22	deg cm^{-2} dmol^{-1} x 10^{-3}	<0.001
dd3us	0.113 ± 0.004	deg cm^{-2} dmol^{-1} x 10^{-3}	<0.001
aa4fi	6.52 ± 0.11	deg cm^{-2} dmol^{-1} K^{-1} x 10^{-3}	<0.001
aa4fs	---	---	---
aa4ui	25.54 ± 0.21	deg cm^{-2} dmol^{-1} x 10^{-3}	<0.001
aa4us	0.094 ± 0.004	deg cm^{-2} dmol^{-1} x 10^{-3}	<0.001
dd4fi	6.16 ± 0.11	deg cm^{-2} dmol^{-1} K^{-1} x 10^{-3}	<0.001
dd4fs	---	---	---
dd4ui	21.46 ± 0.20	deg cm^{-2} dmol^{-1} x 10^{-3}	<0.001
dd4us	0.073 ± 0.004	deg cm^{-2} dmol^{-1} x 10^{-3}	<0.001
aa4fi	8.30 ± 0.26	deg cm^{-2} dmol^{-1} K^{-1} x 10^{-3}	<0.001
aa5fs	0.046 ± 0.008	deg cm^{-2} dmol^{-1} x 10^{-3}	<0.001
aa5ui	14.17 ± 0.20	deg cm^{-2} dmol^{-1} x 10^{-3}	<0.001
aa5us	0.026 ± 0.004	deg cm^{-2} dmol^{-1} x 10^{-3}	<0.001

Figure S477. Plots of average trajectory RMSD and average trajectory core distances as a function of temperature for variant **27e/22g'-xx**, along with the fit parameters ± standard error and statistics.

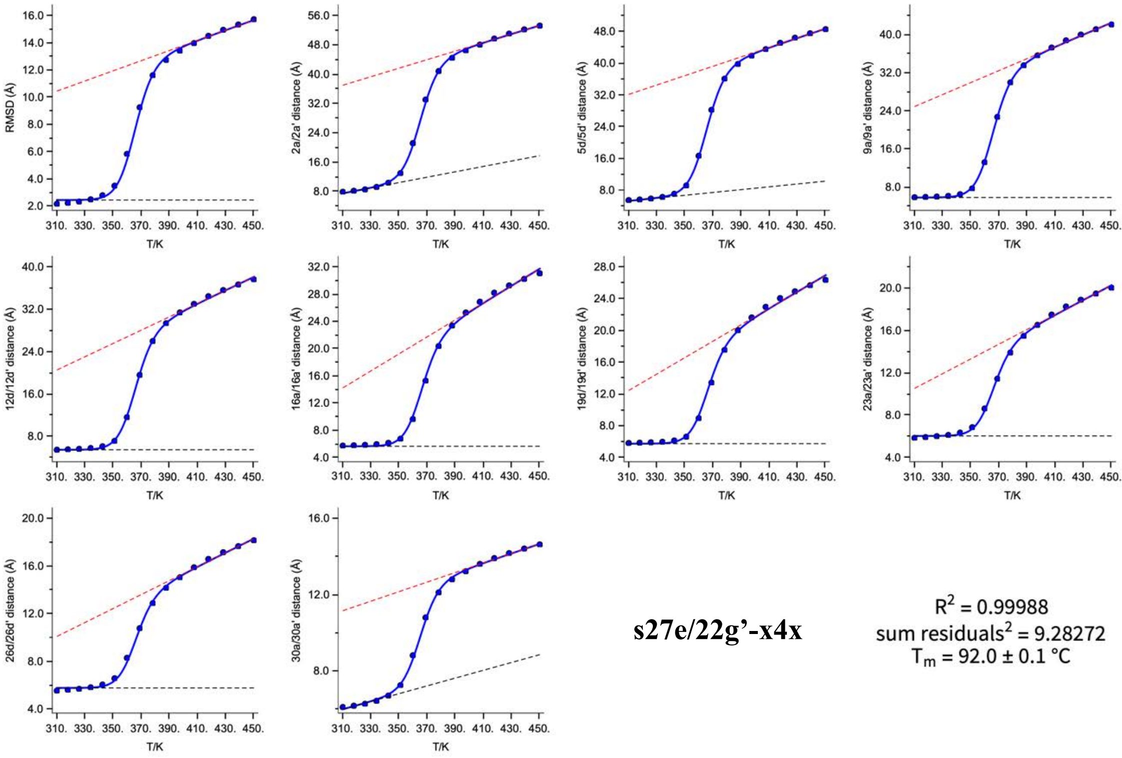

Parameters	Values	Units	P–Values
ΔH_0	–41.11 ± 0.69	kcal/mol	<0.001
T_m	92.0 ± 0.1	°C	<0.001
ΔC_p	---	---	---
rmsfi	2.47 ± 0.11	deg cm^{-2} dmol^{-1} K^{-1} x 10^{-3}	<0.001
rmsfs	---	---	---
rmsui	12.53 ± 0.21	deg cm^{-2} dmol^{-1} x 10^{-3}	<0.001
rmsus	0.037 ± 0.004	deg cm^{-2} dmol^{-1} x 10^{-3}	<0.001
aa1fi	11.64 ± 0.32	deg cm^{-2} dmol^{-1} K^{-1} x 10^{-3}	<0.001
aa1fs	0.073 ± 0.008	deg cm^{-2} dmol^{-1} x 10^{-3}	<0.001
aa1ui	43.50 ± 0.27	deg cm^{-2} dmol^{-1} x 10^{-3}	<0.001
aa1us	0.116 ± 0.005	deg cm^{-2} dmol^{-1} x 10^{-3}	<0.001
dd1fi	7.37 ± 0.31	deg cm^{-2} dmol^{-1} K^{-1} x 10^{-3}	<0.001
dd1fs	0.036 ± 0.008	deg cm^{-2} dmol^{-1} x 10^{-3}	<0.001
dd1ui	38.75 ± 0.27	deg cm^{-2} dmol^{-1} x 10^{-3}	<0.001
dd1us	0.118 ± 0.005	deg cm^{-2} dmol^{-1} x 10^{-3}	<0.001
aa2fi	5.88 ± 0.11	deg cm^{-2} dmol^{-1} K^{-1} x 10^{-3}	<0.001
aa2fs	---	---	---
aa2ui	31.91 ± 0.25	deg cm^{-2} dmol^{-1} x 10^{-3}	<0.001
aa2us	0.126 ± 0.004	deg cm^{-2} dmol^{-1} x 10^{-3}	<0.001
dd2fi	5.54 ± 0.11	deg cm^{-2} dmol^{-1} K^{-1} x 10^{-3}	<0.001
dd2fs	---	---	---
dd2ui	27.53 ± 0.24	deg cm^{-2} dmol^{-1} x 10^{-3}	<0.001
dd2us	0.126 ± 0.004	deg cm^{-2} dmol^{-1} x 10^{-3}	<0.001
aa3fi	5.71 ± 0.11	deg cm^{-2} dmol^{-1} K^{-1} x 10^{-3}	<0.001
aa3fs	---	---	---
aa3ui	21.19 ± 0.22	deg cm^{-2} dmol^{-1} x 10^{-3}	<0.001
aa3us	0.125 ± 0.004	deg cm^{-2} dmol^{-1} x 10^{-3}	<0.001
dd3fi	5.79 ± 0.11	deg cm^{-2} dmol^{-1} K^{-1} x 10^{-3}	<0.001
dd3fs	---	---	---
dd3ui	18.21 ± 0.22	deg cm^{-2} dmol^{-1} x 10^{-3}	<0.001
dd3us	0.104 ± 0.004	deg cm^{-2} dmol^{-1} x 10^{-3}	<0.001
aa4fi	6.07 ± 0.11	deg cm^{-2} dmol^{-1} K^{-1} x 10^{-3}	<0.001
aa4fs	---	---	---
aa4ui	14.41 ± 0.21	deg cm^{-2} dmol^{-1} x 10^{-3}	<0.001
aa4us	0.070 ± 0.004	deg cm^{-2} dmol^{-1} x 10^{-3}	<0.001
dd4fi	5.79 ± 0.11	deg cm^{-2} dmol^{-1} K^{-1} x 10^{-3}	<0.001
dd4fs	---	---	---
dd4ui	13.35 ± 0.21	deg cm^{-2} dmol^{-1} x 10^{-3}	<0.001
dd4us	0.059 ± 0.004	deg cm^{-2} dmol^{-1} x 10^{-3}	<0.001
aa4fi	7.13 ± 0.27	deg cm^{-2} dmol^{-1} K^{-1} x 10^{-3}	<0.001
aa5fs	0.020 ± 0.007	deg cm^{-2} dmol^{-1} x 10^{-3}	0.005
aa5ui	12.56 ± 0.22	deg cm^{-2} dmol^{-1} x 10^{-3}	<0.001
aa5us	0.025 ± 0.004	deg cm^{-2} dmol^{-1} x 10^{-3}	<0.001

Figure S478. Plots of average trajectory RMSD and average trajectory core distances as a function of temperature for variant **s27e/22g'-x4x**, along with the fit parameters ± standard error and statistics.

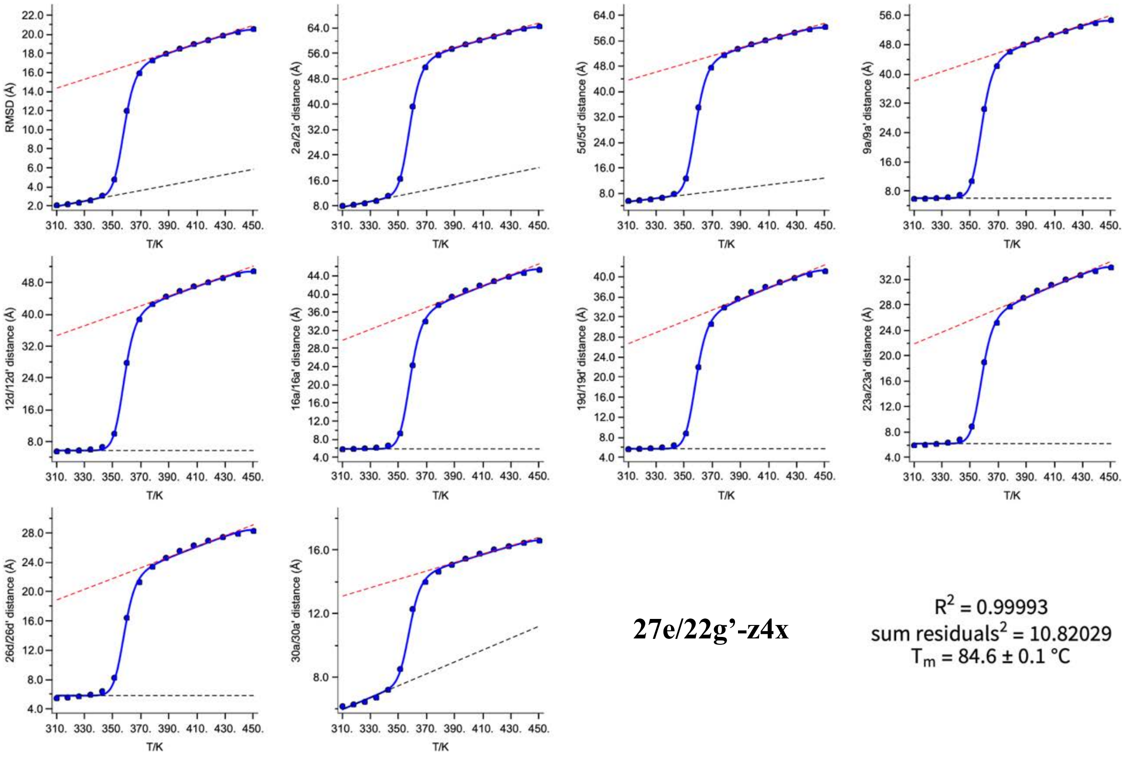

Parameters	Values	Units	P–Values
ΔH_0	–64.81 ± 0.88	kcal/mol	<0.001
T_m	84.6 ± 0.1	°C	–––
ΔC_p	1.243 ± 0.025	kcal/mol/K	<0.001
rmsfi	3.34 ± 0.28	deg cm^{-2} dmol^{-1} K^{-1} x 10^{-3}	<0.001
rmsfs	0.028 ± 0.009	deg cm^{-2} dmol^{-1} x 10^{-3}	0.002
rmsui	16.66 ± 0.21	deg cm^{-2} dmol^{-1} x 10^{-3}	<0.001
rmsus	0.047 ± 0.004	deg cm^{-2} dmol^{-1} x 10^{-3}	<0.001
aa1fi	12.05 ± 0.30	deg cm^{-2} dmol^{-1} K^{-1} x 10^{-3}	<0.001
aa1fs	0.089 ± 0.009	deg cm^{-2} dmol^{-1} x 10^{-3}	<0.001
aa1ui	53.96 ± 0.24	deg cm^{-2} dmol^{-1} x 10^{-3}	<0.001
aa1us	0.128 ± 0.004	deg cm^{-2} dmol^{-1} x 10^{-3}	<0.001
dd1fi	8.03 ± 0.30	deg cm^{-2} dmol^{-1} K^{-1} x 10^{-3}	<0.001
dd1fs	0.053 ± 0.009	deg cm^{-2} dmol^{-1} x 10^{-3}	<0.001
dd1ui	49.88 ± 0.24	deg cm^{-2} dmol^{-1} x 10^{-3}	<0.001
dd1us	0.128 ± 0.004	deg cm^{-2} dmol^{-1} x 10^{-3}	<0.001
aa2fi	6.25 ± 0.13	deg cm^{-2} dmol^{-1} K^{-1} x 10^{-3}	<0.001
aa2fs	–––	–––	–––
aa2ui	44.34 ± 0.23	deg cm^{-2} dmol^{-1} x 10^{-3}	<0.001
aa2us	0.128 ± 0.004	deg cm^{-2} dmol^{-1} x 10^{-3}	<0.001
dd2fi	5.86 ± 0.13	deg cm^{-2} dmol^{-1} K^{-1} x 10^{-3}	<0.001
dd2fs	–––	–––	–––
dd2ui	40.78 ± 0.23	deg cm^{-2} dmol^{-1} x 10^{-3}	<0.001
dd2us	0.125 ± 0.004	deg cm^{-2} dmol^{-1} x 10^{-3}	<0.001
aa3fi	5.99 ± 0.12	deg cm^{-2} dmol^{-1} K^{-1} x 10^{-3}	<0.001
aa3fs	–––	–––	–––
aa3ui	35.68 ± 0.22	deg cm^{-2} dmol^{-1} x 10^{-3}	<0.001
aa3us	0.120 ± 0.004	deg cm^{-2} dmol^{-1} x 10^{-3}	<0.001
dd3fi	5.83 ± 0.12	deg cm^{-2} dmol^{-1} K^{-1} x 10^{-3}	<0.001
dd3fs	–––	–––	–––
dd3ui	32.14 ± 0.22	deg cm^{-2} dmol^{-1} x 10^{-3}	<0.001
dd3us	0.112 ± 0.004	deg cm^{-2} dmol^{-1} x 10^{-3}	<0.001
aa4fi	6.25 ± 0.12	deg cm^{-2} dmol^{-1} K^{-1} x 10^{-3}	<0.001
aa4fs	–––	–––	–––
aa4ui	26.38 ± 0.21	deg cm^{-2} dmol^{-1} x 10^{-3}	<0.001
aa4us	0.093 ± 0.004	deg cm^{-2} dmol^{-1} x 10^{-3}	<0.001
dd4fi	5.88 ± 0.12	deg cm^{-2} dmol^{-1} K^{-1} x 10^{-3}	<0.001
dd4fs	–––	–––	–––
dd4ui	22.43 ± 0.21	deg cm^{-2} dmol^{-1} x 10^{-3}	<0.001
dd4us	0.073 ± 0.004	deg cm^{-2} dmol^{-1} x 10^{-3}	<0.001
aa4fi	7.80 ± 0.28	deg cm^{-2} dmol^{-1} K^{-1} x 10^{-3}	<0.001
aa5fs	0.037 ± 0.009	deg cm^{-2} dmol^{-1} x 10^{-3}	<0.001
aa5ui	14.40 ± 0.21	deg cm^{-2} dmol^{-1} x 10^{-3}	<0.001
aa5us	0.026 ± 0.004	deg cm^{-2} dmol^{-1} x 10^{-3}	<0.001

Figure S479. Plots of average trajectory RMSD and average trajectory core distances as a function of temperature for variant **27e/22g'-z4x**, along with the fit parameters ± standard error and statistics.

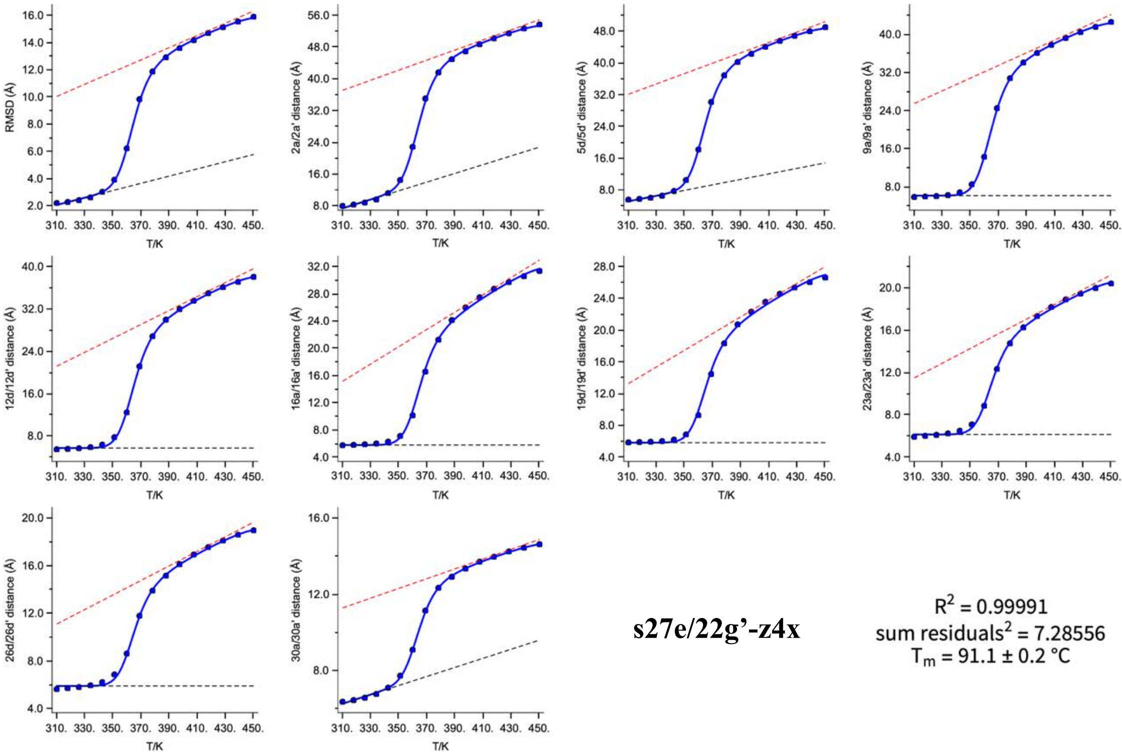

Parameters	Values	Units	P–Values
ΔH_0	–41.85 ± 0.79	kcal/mol	<0.001
T_m	91.1 ± 0.2	°C	<0.001
ΔC_p	0.765 ± 0.027	kcal/mol/K	<0.001
rmsfi	3.54 ± 0.24	deg cm^{-2} dmol^{-1} K^{-1} x 10^{-3}	<0.001
rmsfs	0.026 ± 0.006	deg cm^{-2} dmol^{-1} x 10^{-3}	<0.001
rmsui	12.51 ± 0.21	deg cm^{-2} dmol^{-1} x 10^{-3}	<0.001
rmsus	0.045 ± 0.004	deg cm^{-2} dmol^{-1} x 10^{-3}	<0.001
aa1fi	13.47 ± 0.27	deg cm^{-2} dmol^{-1} K^{-1} x 10^{-3}	<0.001
aa1fs	0.110 ± 0.007	deg cm^{-2} dmol^{-1} x 10^{-3}	<0.001
aa1ui	44.11 ± 0.31	deg cm^{-2} dmol^{-1} x 10^{-3}	<0.001
aa1us	0.127 ± 0.005	deg cm^{-2} dmol^{-1} x 10^{-3}	<0.001
dd1fi	9.01 ± 0.27	deg cm^{-2} dmol^{-1} K^{-1} x 10^{-3}	<0.001
dd1fs	0.069 ± 0.007	deg cm^{-2} dmol^{-1} x 10^{-3}	<0.001
dd1ui	39.31 ± 0.31	deg cm^{-2} dmol^{-1} x 10^{-3}	<0.001
dd1us	0.131 ± 0.006	deg cm^{-2} dmol^{-1} x 10^{-3}	<0.001
aa2fi	6.23 ± 0.10	deg cm^{-2} dmol^{-1} K^{-1} x 10^{-3}	<0.001
aa2fs	–––	–––	–––
aa2ui	32.82 ± 0.29	deg cm^{-2} dmol^{-1} x 10^{-3}	<0.001
aa2us	0.133 ± 0.005	deg cm^{-2} dmol^{-1} x 10^{-3}	<0.001
dd2fi	5.81 ± 0.10	deg cm^{-2} dmol^{-1} K^{-1} x 10^{-3}	<0.001
dd2fs	–––	–––	–––
dd2ui	28.43 ± 0.27	deg cm^{-2} dmol^{-1} x 10^{-3}	<0.001
dd2us	0.132 ± 0.005	deg cm^{-2} dmol^{-1} x 10^{-3}	<0.001
aa3fi	5.85 ± 0.10	deg cm^{-2} dmol^{-1} K^{-1} x 10^{-3}	<0.001
aa3fs	–––	–––	–––
aa3ui	22.12 ± 0.24	deg cm^{-2} dmol^{-1} x 10^{-3}	<0.001
aa3us	0.127 ± 0.004	deg cm^{-2} dmol^{-1} x 10^{-3}	<0.001
dd3fi	5.89 ± 0.10	deg cm^{-2} dmol^{-1} K^{-1} x 10^{-3}	<0.001
dd3fs	–––	–––	–––
dd3ui	19.04 ± 0.22	deg cm^{-2} dmol^{-1} x 10^{-3}	<0.001
dd3us	0.105 ± 0.004	deg cm^{-2} dmol^{-1} x 10^{-3}	<0.001
aa4fi	6.19 ± 0.10	deg cm^{-2} dmol^{-1} K^{-1} x 10^{-3}	<0.001
aa4fs	–––	–––	–––
aa4ui	15.31 ± 0.20	deg cm^{-2} dmol^{-1} x 10^{-3}	<0.001
aa4us	0.069 ± 0.004	deg cm^{-2} dmol^{-1} x 10^{-3}	<0.001
dd4fi	5.93 ± 0.10	deg cm^{-2} dmol^{-1} K^{-1} x 10^{-3}	<0.001
dd4fs	–––	–––	–––
dd4ui	14.45 ± 0.20	deg cm^{-2} dmol^{-1} x 10^{-3}	<0.001
dd4us	0.061 ± 0.004	deg cm^{-2} dmol^{-1} x 10^{-3}	<0.001
aa4fi	7.57 ± 0.23	deg cm^{-2} dmol^{-1} K^{-1} x 10^{-3}	<0.001
aa5fs	0.024 ± 0.006	deg cm^{-2} dmol^{-1} x 10^{-3}	<0.001
aa5ui	12.70 ± 0.20	deg cm^{-2} dmol^{-1} x 10^{-3}	<0.001
aa5us	0.026 ± 0.004	deg cm^{-2} dmol^{-1} x 10^{-3}	<0.001

Figure S480. Plots of average trajectory RMSD and average trajectory core distances as a function of temperature for variant **s27e/22g'-z4x**, along with the fit parameters ± standard error and statistics.

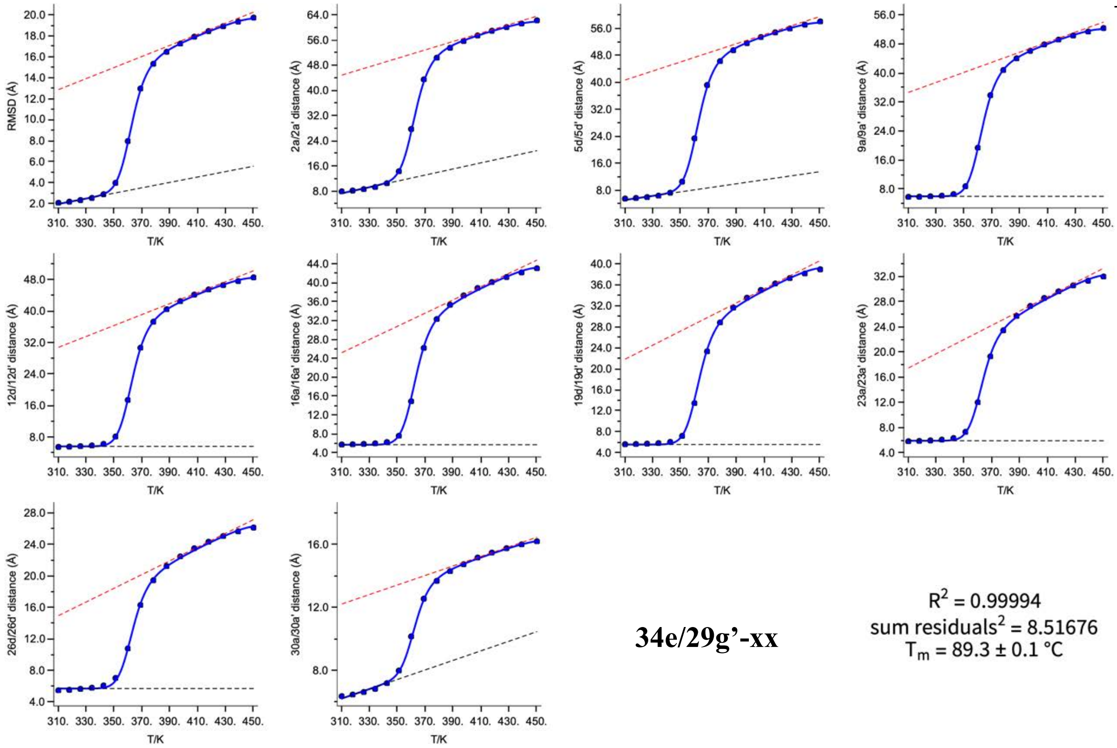

Parameters	Values	Units	P–Values
ΔH_0	−48.56 ± 0.68	kcal/mol	<0.001
T_m	89.3 ± 0.1	°C	<0.001
ΔC_p	0.909 ± 0.022	kcal/mol/K	<0.001
rmsfi	3.36 ± 0.25	deg cm^{-2} dmol^{-1} K^{-1} x 10^{-3}	<0.001
rmsfs	0.026 ± 0.007	deg cm^{-2} dmol^{-1} x 10^{-3}	<0.001
rmsui	15.68 ± 0.21	deg cm^{-2} dmol^{-1} x 10^{-3}	<0.001
rmsus	0.053 ± 0.004	deg cm^{-2} dmol^{-1} x 10^{-3}	<0.001
aa1fi	12.52 ± 0.27	deg cm^{-2} dmol^{-1} K^{-1} x 10^{-3}	<0.001
aa1fs	0.097 ± 0.008	deg cm^{-2} dmol^{-1} x 10^{-3}	<0.001
aa1ui	52.01 ± 0.28	deg cm^{-2} dmol^{-1} x 10^{-3}	<0.001
aa1us	0.134 ± 0.005	deg cm^{-2} dmol^{-1} x 10^{-3}	<0.001
dd1fi	8.39 ± 0.27	deg cm^{-2} dmol^{-1} K^{-1} x 10^{-3}	<0.001
dd1fs	0.060 ± 0.008	deg cm^{-2} dmol^{-1} x 10^{-3}	<0.001
dd1ui	47.87 ± 0.28	deg cm^{-2} dmol^{-1} x 10^{-3}	<0.001
dd1us	0.135 ± 0.005	deg cm^{-2} dmol^{-1} x 10^{-3}	<0.001
aa2fi	6.14 ± 0.11	deg cm^{-2} dmol^{-1} K^{-1} x 10^{-3}	<0.001
aa2fs	---	---	---
aa2ui	42.03 ± 0.26	deg cm^{-2} dmol^{-1} x 10^{-3}	<0.001
aa2us	0.138 ± 0.005	deg cm^{-2} dmol^{-1} x 10^{-3}	<0.001
dd2fi	5.73 ± 0.11	deg cm^{-2} dmol^{-1} K^{-1} x 10^{-3}	<0.001
dd2fs	---	---	---
dd2ui	38.21 ± 0.25	deg cm^{-2} dmol^{-1} x 10^{-3}	<0.001
dd2us	0.139 ± 0.005	deg cm^{-2} dmol^{-1} x 10^{-3}	<0.001
aa3fi	5.79 ± 0.11	deg cm^{-2} dmol^{-1} K^{-1} x 10^{-3}	<0.001
aa3fs	---	---	---
aa3ui	32.64 ± 0.24	deg cm^{-2} dmol^{-1} x 10^{-3}	<0.001
aa3us	0.140 ± 0.004	deg cm^{-2} dmol^{-1} x 10^{-3}	<0.001
dd3fi	5.65 ± 0.11	deg cm^{-2} dmol^{-1} K^{-1} x 10^{-3}	<0.001
dd3fs	---	---	---
dd3ui	28.97 ± 0.23	deg cm^{-2} dmol^{-1} x 10^{-3}	<0.001
dd3us	0.134 ± 0.004	deg cm^{-2} dmol^{-1} x 10^{-3}	<0.001
aa4fi	5.97 ± 0.11	deg cm^{-2} dmol^{-1} K^{-1} x 10^{-3}	<0.001
aa4fs	---	---	---
aa4ui	23.48 ± 0.22	deg cm^{-2} dmol^{-1} x 10^{-3}	<0.001
aa4us	0.113 ± 0.004	deg cm^{-2} dmol^{-1} x 10^{-3}	<0.001
dd4fi	5.74 ± 0.11	deg cm^{-2} dmol^{-1} K^{-1} x 10^{-3}	<0.001
dd4fs	---	---	---
dd4ui	19.59 ± 0.21	deg cm^{-2} dmol^{-1} x 10^{-3}	<0.001
dd4us	0.087 ± 0.004	deg cm^{-2} dmol^{-1} x 10^{-3}	<0.001
aa4fi	7.83 ± 0.25	deg cm^{-2} dmol^{-1} K^{-1} x 10^{-3}	<0.001
aa5fs	0.030 ± 0.007	deg cm^{-2} dmol^{-1} x 10^{-3}	<0.001
aa5ui	13.83 ± 0.21	deg cm^{-2} dmol^{-1} x 10^{-3}	<0.001
aa5us	0.030 ± 0.004	deg cm^{-2} dmol^{-1} x 10^{-3}	<0.001

Figure S481. Plots of average trajectory RMSD and average trajectory core distances as a function of temperature for variant **34e/29g'-xx**, along with the fit parameters ± standard error and statistics.

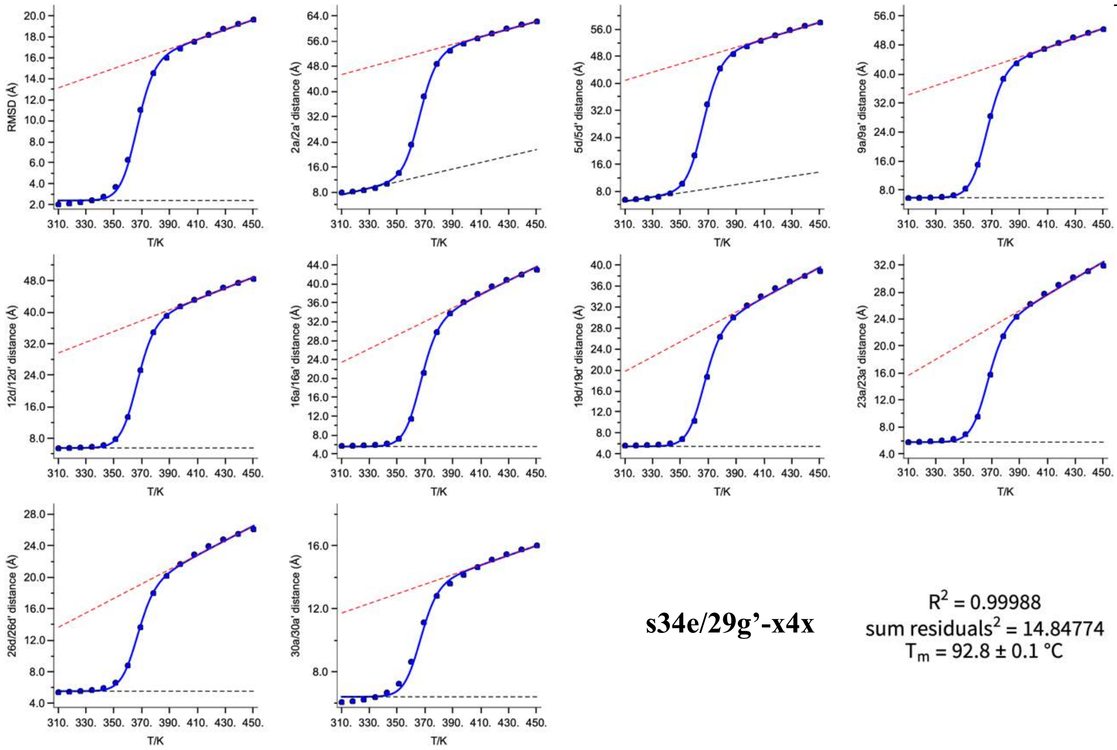

Parameters	Values	Units	P–Values
ΔH_0	−43.84 ± 0.67	kcal/mol	<0.001
T_m	92.8 ± 0.1	°C	<0.001
ΔC_p	---	---	---
rmsfi	2.45 ± 0.14	deg cm^{-2} dmol^{-1} K^{-1} x 10^{-3}	<0.001
rmsfs	---	---	---
rmsui	15.78 ± 0.26	deg cm^{-2} dmol^{-1} x 10^{-3}	<0.001
rmsus	0.046 ± 0.005	deg cm^{-2} dmol^{-1} x 10^{-3}	<0.001
aa1fi	13.09 ± 0.37	deg cm^{-2} dmol^{-1} K^{-1} x 10^{-3}	<0.001
aa1fs	0.102 ± 0.009	deg cm^{-2} dmol^{-1} x 10^{-3}	<0.001
aa1ui	52.25 ± 0.32	deg cm^{-2} dmol^{-1} x 10^{-3}	<0.001
aa1us	0.121 ± 0.006	deg cm^{-2} dmol^{-1} x 10^{-3}	<0.001
dd1fi	8.65 ± 0.37	deg cm^{-2} dmol^{-1} K^{-1} x 10^{-3}	<0.001
dd1fs	0.062 ± 0.009	deg cm^{-2} dmol^{-1} x 10^{-3}	<0.001
dd1ui	47.90 ± 0.32	deg cm^{-2} dmol^{-1} x 10^{-3}	<0.001
dd1us	0.123 ± 0.006	deg cm^{-2} dmol^{-1} x 10^{-3}	<0.001
aa2fi	6.07 ± 0.14	deg cm^{-2} dmol^{-1} K^{-1} x 10^{-3}	<0.001
aa2fs	---	---	---
aa2ui	41.70 ± 0.30	deg cm^{-2} dmol^{-1} x 10^{-3}	<0.001
aa2us	0.131 ± 0.005	deg cm^{-2} dmol^{-1} x 10^{-3}	<0.001
dd2fi	5.65 ± 0.14	deg cm^{-2} dmol^{-1} K^{-1} x 10^{-3}	<0.001
dd2fs	---	---	---
dd2ui	37.49 ± 0.29	deg cm^{-2} dmol^{-1} x 10^{-3}	<0.001
dd2us	0.137 ± 0.005	deg cm^{-2} dmol^{-1} x 10^{-3}	<0.001
aa3fi	5.68 ± 0.14	deg cm^{-2} dmol^{-1} K^{-1} x 10^{-3}	<0.001
aa3fs	---	---	---
aa3ui	31.62 ± 0.28	deg cm^{-2} dmol^{-1} x 10^{-3}	<0.001
aa3us	0.145 ± 0.005	deg cm^{-2} dmol^{-1} x 10^{-3}	<0.001
dd3fi	5.54 ± 0.14	deg cm^{-2} dmol^{-1} K^{-1} x 10^{-3}	<0.001
dd3fs	---	---	---
dd3ui	27.78 ± 0.28	deg cm^{-2} dmol^{-1} x 10^{-3}	<0.001
dd3us	0.142 ± 0.005	deg cm^{-2} dmol^{-1} x 10^{-3}	<0.001
aa4fi	5.83 ± 0.14	deg cm^{-2} dmol^{-1} K^{-1} x 10^{-3}	<0.001
aa4fs	---	---	---
aa4ui	22.46 ± 0.27	deg cm^{-2} dmol^{-1} x 10^{-3}	<0.001
aa4us	0.120 ± 0.005	deg cm^{-2} dmol^{-1} x 10^{-3}	<0.001
dd4fi	5.59 ± 0.14	deg cm^{-2} dmol^{-1} K^{-1} x 10^{-3}	<0.001
dd4fs	---	---	---
dd4ui	18.87 ± 0.27	deg cm^{-2} dmol^{-1} x 10^{-3}	<0.001
dd4us	0.092 ± 0.005	deg cm^{-2} dmol^{-1} x 10^{-3}	<0.001
aa4fi	6.44 ± 0.14	deg cm^{-2} dmol^{-1} K^{-1} x 10^{-3}	<0.001
aa5fs	---	---	---
aa5ui	13.47 ± 0.26	deg cm^{-2} dmol^{-1} x 10^{-3}	<0.001
aa5us	0.031 ± 0.005	deg cm^{-2} dmol^{-1} x 10^{-3}	<0.001

Figure S482. Plots of average trajectory RMSD and average trajectory core distances as a function of temperature for variant **s34e/29g'-x4x**, along with the fit parameters ± standard error and statistics.

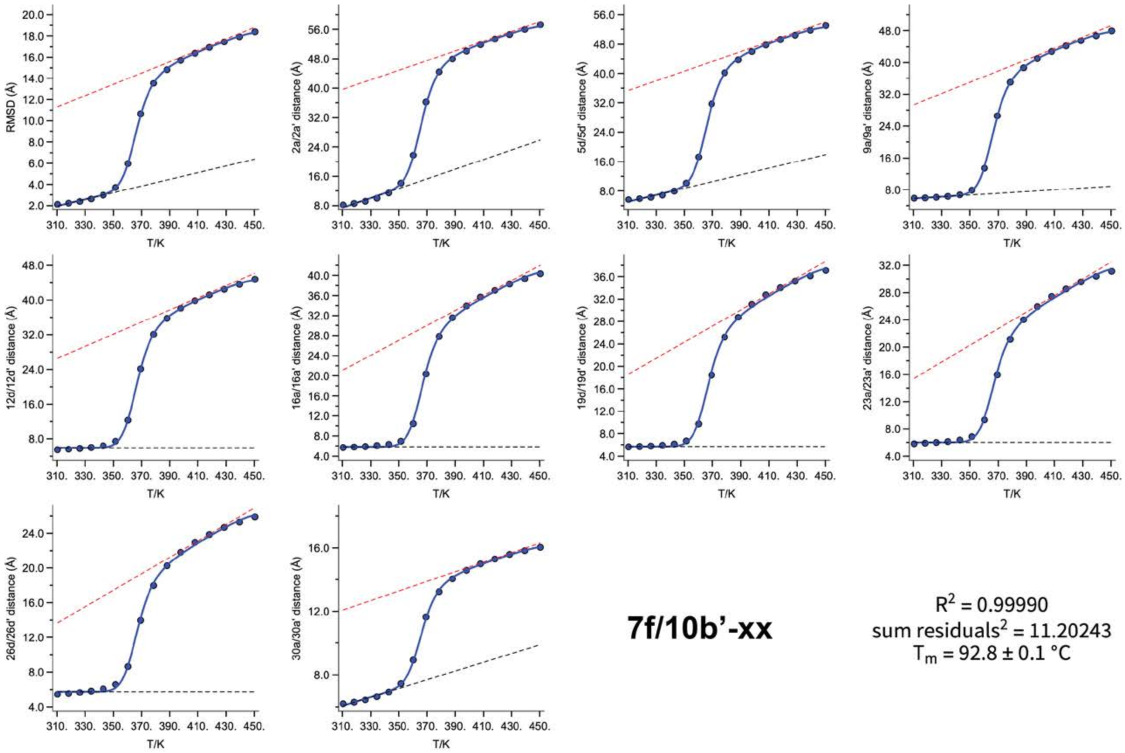

Parameters	Values	Units	P- Values
ΔH_0	- 48.23 ± 0.91	kcal/mol	<0.001
T_m	92.8 ± 0.1	°C	<0.001
ΔC_p	0.916 ± 0.031	kcal/mol/K	<0.001
rmsfi	3.79 ± 0.28	deg cm^{-2} dmol^{-1} K^{-1} x 10^{-3}	<0.001
rmsfs	0.032 ± 0.008	deg cm^{-2} dmol^{-1} x 10^{-3}	<0.001
rmsui	14.37 ± 0.25	deg cm^{-2} dmol^{-1} x 10^{-3}	<0.001
rmsus	0.054 ± 0.005	deg cm^{-2} dmol^{-1} x 10^{-3}	<0.001
aa1fi	14.99 ± 0.31	deg cm^{-2} dmol^{-1} K^{-1} x 10^{-3}	<0.001
aa1fs	0.132 ± 0.008	deg cm^{-2} dmol^{-1} x 10^{-3}	<0.001
aa1ui	47.23 ± 0.32	deg cm^{-2} dmol^{-1} x 10^{-3}	<0.001
aa1us	0.132 ± 0.006	deg cm^{-2} dmol^{-1} x 10^{-3}	<0.001
dd1fi	10.39 ± 0.31	deg cm^{-2} dmol^{-1} K^{-1} x 10^{-3}	<0.001
dd1fs	0.092 ± 0.008	deg cm^{-2} dmol^{-1} x 10^{-3}	<0.001
dd1ui	43.00 ± 0.33	deg cm^{-2} dmol^{-1} x 10^{-3}	<0.001
dd1us	0.134 ± 0.006	deg cm^{-2} dmol^{-1} x 10^{-3}	<0.001
aa2fi	7.17 ± 0.30	deg cm^{-2} dmol^{-1} K^{-1} x 10^{-3}	<0.001
aa2fs	0.022 ± 0.008	deg cm^{-2} dmol^{-1} x 10^{-3}	0.006
aa2ui	37.49 ± 0.33	deg cm^{-2} dmol^{-1} x 10^{-3}	<0.001
aa2us	0.142 ± 0.006	deg cm^{-2} dmol^{-1} x 10^{-3}	<0.001
dd2fi	6.04 ± 0.12	deg cm^{-2} dmol^{-1} K^{-1} x 10^{-3}	<0.001
dd2fs	---	---	---
dd2ui	34.55 ± 0.31	deg cm^{-2} dmol^{-1} x 10^{-3}	<0.001
dd2us	0.140 ± 0.006	deg cm^{-2} dmol^{-1} x 10^{-3}	<0.001
aa3fi	5.97 ± 0.12	deg cm^{-2} dmol^{-1} K^{-1} x 10^{-3}	<0.001
aa3fs	---	---	---
aa3ui	29.61 ± 0.29	deg cm^{-2} dmol^{-1} x 10^{-3}	<0.001
aa3us	0.149 ± 0.006	deg cm^{-2} dmol^{-1} x 10^{-3}	<0.001
dd3fi	5.81 ± 0.12	deg cm^{-2} dmol^{-1} K^{-1} x 10^{-3}	<0.001
dd3fs	---	---	---
dd3ui	26.77 ± 0.28	deg cm^{-2} dmol^{-1} x 10^{-3}	<0.001
dd3us	0.144 ± 0.005	deg cm^{-2} dmol^{-1} x 10^{-3}	<0.001
aa4fi	6.09 ± 0.12	deg cm^{-2} dmol^{-1} K^{-1} x 10^{-3}	<0.001
aa4fs	---	---	---
aa4ui	22.35 ± 0.26	deg cm^{-2} dmol^{-1} x 10^{-3}	<0.001
aa4us	0.122 ± 0.005	deg cm^{-2} dmol^{-1} x 10^{-3}	<0.001
dd4fi	5.80 ± 0.12	deg cm^{-2} dmol^{-1} K^{-1} x 10^{-3}	<0.001
dd4fs	---	---	---
dd4ui	19.05 ± 0.25	deg cm^{-2} dmol^{-1} x 10^{-3}	<0.001
dd4us	0.095 ± 0.005	deg cm^{-2} dmol^{-1} x 10^{-3}	<0.001
aa4fi	7.64 ± 0.28	deg cm^{-2} dmol^{-1} K^{-1} x 10^{-3}	<0.001
aa5fs	0.027 ± 0.007	deg cm^{-2} dmol^{-1} x 10^{-3}	<0.001
aa5ui	13.81 ± 0.25	deg cm^{-2} dmol^{-1} x 10^{-3}	<0.001
aa5us	0.030 ± 0.005	deg cm^{-2} dmol^{-1} x 10^{-3}	<0.001

Figure S483. Plots of average trajectory RMSD and average trajectory core distances as a function of temperature for variant **7f/10b'-xx**, along with the fit parameters ± standard error and statistics.

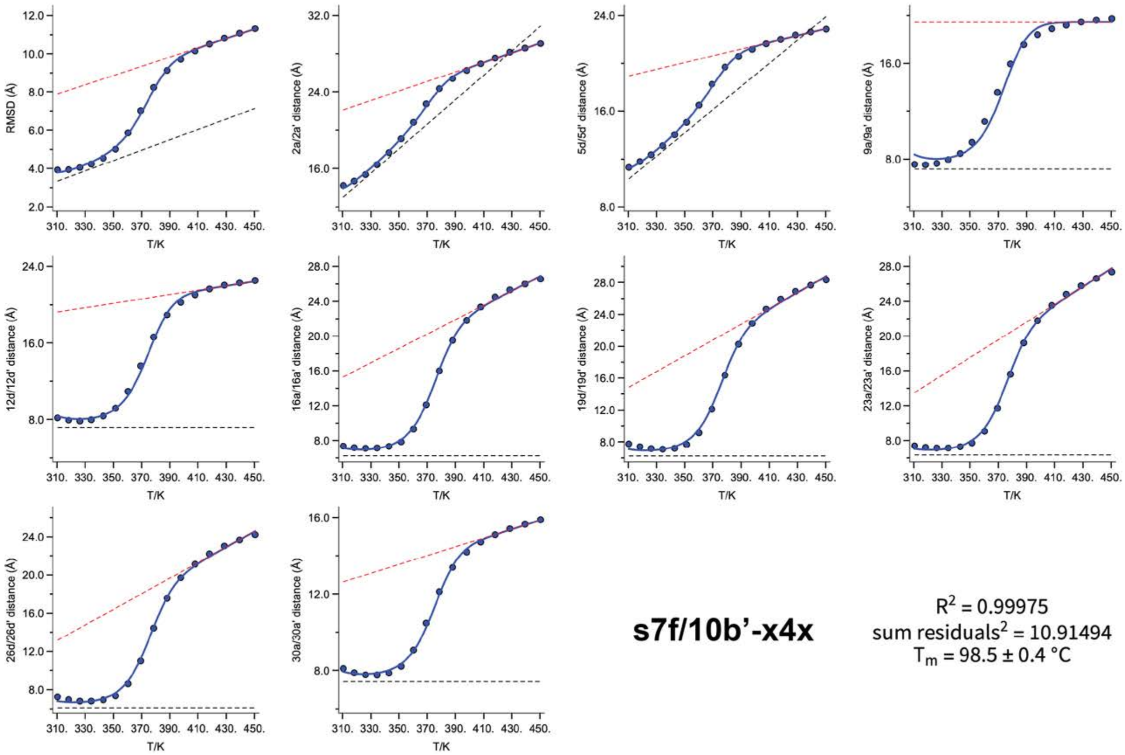

Parameters	Values	Units	P- Values
ΔH_0	- 28.17 ± 1.16	kcal/mol	<0.001
T_m	98.5 ± 0.4	°C	<0.001
ΔC_p	- 0.611 ± 0.027	kcal/mol/K	<0.001
rmsfi	5.05 ± 0.34	deg cm^{-2} dmol^{-1} K^{-1} x 10^{-3}	<0.001
rmsfs	0.027 ± 0.007	deg cm^{-2} dmol^{-1} x 10^{-3}	<0.001
rmsui	9.42 ± 0.29	deg cm^{-2} dmol^{-1} x 10^{-3}	<0.001
rmsus	0.024 ± 0.006	deg cm^{-2} dmol^{-1} x 10^{-3}	<0.001
aa1fi	20.95 ± 0.35	deg cm^{-2} dmol^{-1} K^{-1} x 10^{-3}	<0.001
aa1fs	0.128 ± 0.008	deg cm^{-2} dmol^{-1} x 10^{-3}	<0.001
aa1ui	25.26 ± 0.29	deg cm^{-2} dmol^{-1} x 10^{-3}	<0.001
aa1us	0.050 ± 0.005	deg cm^{-2} dmol^{-1} x 10^{-3}	<0.001
dd1fi	16.37 ± 0.34	deg cm^{-2} dmol^{-1} K^{-1} x 10^{-3}	<0.001
dd1fs	0.097 ± 0.008	deg cm^{-2} dmol^{-1} x 10^{-3}	<0.001
dd1ui	20.74 ± 0.29	deg cm^{-2} dmol^{-1} x 10^{-3}	<0.001
dd1us	0.028 ± 0.006	deg cm^{-2} dmol^{-1} x 10^{-3}	<0.001
aa2fi	7.20 ± 0.23	deg cm^{-2} dmol^{-1} K^{-1} x 10^{-3}	<0.001
aa2fs	---	---	---
aa2ui	19.51 ± 0.12	deg cm^{-2} dmol^{-1} x 10^{-3}	<0.001
aa2us	---	---	---
dd2fi	7.24 ± 0.25	deg cm^{-2} dmol^{-1} K^{-1} x 10^{-3}	<0.001
dd2fs	---	---	---
dd2ui	20.73 ± 0.30	deg cm^{-2} dmol^{-1} x 10^{-3}	<0.001
dd2us	0.023 ± 0.006	deg cm^{-2} dmol^{-1} x 10^{-3}	<0.001
aa3fi	6.29 ± 0.23	deg cm^{-2} dmol^{-1} K^{-1} x 10^{-3}	<0.001
aa3fs	---	---	---
aa3ui	20.48 ± 0.32	deg cm^{-2} dmol^{-1} x 10^{-3}	<0.001
aa3us	0.083 ± 0.006	deg cm^{-2} dmol^{-1} x 10^{-3}	<0.001
dd3fi	6.30 ± 0.23	deg cm^{-2} dmol^{-1} K^{-1} x 10^{-3}	<0.001
dd3fs	---	---	---
dd3ui	21.06 ± 0.33	deg cm^{-2} dmol^{-1} x 10^{-3}	<0.001
dd3us	0.100 ± 0.006	deg cm^{-2} dmol^{-1} x 10^{-3}	<0.001
aa4fi	6.38 ± 0.21	deg cm^{-2} dmol^{-1} K^{-1} x 10^{-3}	<0.001
aa4fs	---	---	---
aa4ui	19.86 ± 0.32	deg cm^{-2} dmol^{-1} x 10^{-3}	<0.001
aa4us	0.102 ± 0.006	deg cm^{-2} dmol^{-1} x 10^{-3}	<0.001
dd4fi	6.17 ± 0.20	deg cm^{-2} dmol^{-1} K^{-1} x 10^{-3}	<0.001
dd4fs	---	---	---
dd4ui	18.25 ± 0.30	deg cm^{-2} dmol^{-1} x 10^{-3}	<0.001
dd4us	0.081 ± 0.006	deg cm^{-2} dmol^{-1} x 10^{-3}	<0.001
aa4fi	7.45 ± 0.17	deg cm^{-2} dmol^{-1} K^{-1} x 10^{-3}	<0.001
aa5fs	---	---	---
aa5ui	14.09 ± 0.27	deg cm^{-2} dmol^{-1} x 10^{-3}	<0.001
aa5us	0.023 ± 0.005	deg cm^{-2} dmol^{-1} x 10^{-3}	<0.001

Figure S484. Plots of average trajectory RMSD and average trajectory core distances as a function of temperature for variant **s7f/10b'-x4x**, along with the fit parameters ± standard error and statistics.

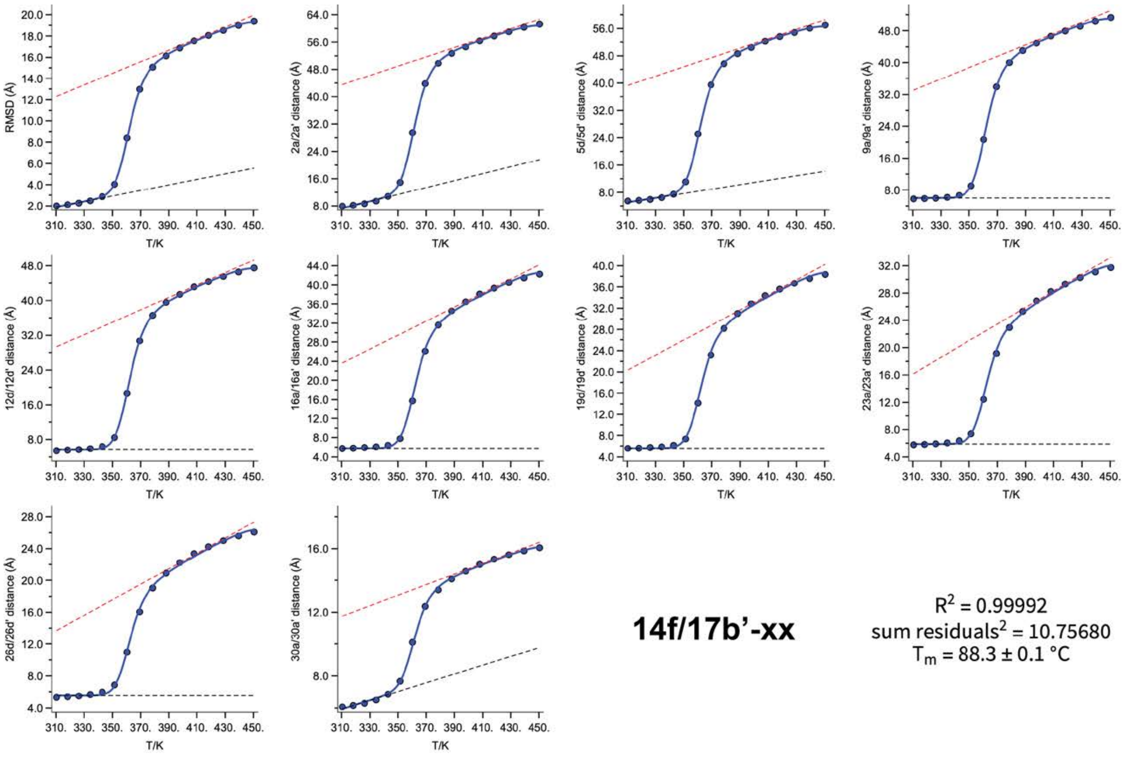

Parameters	Values	Units	P- Values
ΔH_0	- 49.00 ± 0.81	kcal/mol	<0.001
T_m	88.3 ± 0.1	°C	<0.001
ΔC_p	0.921 ± 0.024	kcal/mol/K	<0.001
rmsfi	3.30 ± 0.28	deg cm^{-2} dmol^{-1} K^{-1} x 10^{-3}	<0.001
rmsfs	0.026 ± 0.008	deg cm^{-2} dmol^{-1} x 10^{-3}	0.002
rmsui	15.19 ± 0.24	deg cm^{-2} dmol^{-1} x 10^{-3}	<0.001
rmsus	0.055 ± 0.004	deg cm^{-2} dmol^{-1} x 10^{-3}	<0.001
aa1fi	12.73 ± 0.31	deg cm^{-2} dmol^{-1} K^{-1} x 10^{-3}	<0.001
aa1fs	0.102 ± 0.009	deg cm^{-2} dmol^{-1} x 10^{-3}	<0.001
aa1ui	50.76 ± 0.30	deg cm^{-2} dmol^{-1} x 10^{-3}	<0.001
aa1us	0.137 ± 0.005	deg cm^{-2} dmol^{-1} x 10^{-3}	<0.001
dd1fi	8.58 ± 0.31	deg cm^{-2} dmol^{-1} K^{-1} x 10^{-3}	<0.001
dd1fs	0.065 ± 0.009	deg cm^{-2} dmol^{-1} x 10^{-3}	<0.001
dd1ui	46.55 ± 0.31	deg cm^{-2} dmol^{-1} x 10^{-3}	<0.001
dd1us	0.138 ± 0.005	deg cm^{-2} dmol^{-1} x 10^{-3}	<0.001
aa2fi	6.13 ± 0.12	deg cm^{-2} dmol^{-1} K^{-1} x 10^{-3}	<0.001
aa2fs	---	---	---
aa2ui	40.57 ± 0.29	deg cm^{-2} dmol^{-1} x 10^{-3}	<0.001
aa2us	0.143 ± 0.005	deg cm^{-2} dmol^{-1} x 10^{-3}	<0.001
dd2fi	5.79 ± 0.12	deg cm^{-2} dmol^{-1} K^{-1} x 10^{-3}	<0.001
dd2fs	---	---	---
dd2ui	36.82 ± 0.28	deg cm^{-2} dmol^{-1} x 10^{-3}	<0.001
dd2us	0.143 ± 0.005	deg cm^{-2} dmol^{-1} x 10^{-3}	<0.001
aa3fi	5.89 ± 0.12	deg cm^{-2} dmol^{-1} K^{-1} x 10^{-3}	<0.001
aa3fs	---	---	---
aa3ui	31.33 ± 0.26	deg cm^{-2} dmol^{-1} x 10^{-3}	<0.001
aa3us	0.147 ± 0.005	deg cm^{-2} dmol^{-1} x 10^{-3}	<0.001
dd3fi	5.70 ± 0.12	deg cm^{-2} dmol^{-1} K^{-1} x 10^{-3}	<0.001
dd3fs	---	---	---
dd3ui	27.79 ± 0.25	deg cm^{-2} dmol^{-1} x 10^{-3}	<0.001
dd3us	0.142 ± 0.005	deg cm^{-2} dmol^{-1} x 10^{-3}	<0.001
aa4fi	5.95 ± 0.12	deg cm^{-2} dmol^{-1} K^{-1} x 10^{-3}	<0.001
aa4fs	---	---	---
aa4ui	22.55 ± 0.24	deg cm^{-2} dmol^{-1} x 10^{-3}	<0.001
aa4us	0.122 ± 0.004	deg cm^{-2} dmol^{-1} x 10^{-3}	<0.001
dd4fi	5.61 ± 0.12	deg cm^{-2} dmol^{-1} K^{-1} x 10^{-3}	<0.001
dd4fs	---	---	---
dd4ui	18.79 ± 0.23	deg cm^{-2} dmol^{-1} x 10^{-3}	<0.001
dd4us	0.097 ± 0.004	deg cm^{-2} dmol^{-1} x 10^{-3}	<0.001
aa4fi	7.38 ± 0.28	deg cm^{-2} dmol^{-1} K^{-1} x 10^{-3}	<0.001
aa5fs	0.027 ± 0.008	deg cm^{-2} dmol^{-1} x 10^{-3}	<0.001
aa5ui	13.50 ± 0.23	deg cm^{-2} dmol^{-1} x 10^{-3}	<0.001
aa5us	0.033 ± 0.004	deg cm^{-2} dmol^{-1} x 10^{-3}	<0.001

Figure S485. Plots of average trajectory RMSD and average trajectory core distances as a function of temperature for variant **14f/17b'-xx**, along with the fit parameters ± standard error and statistics.

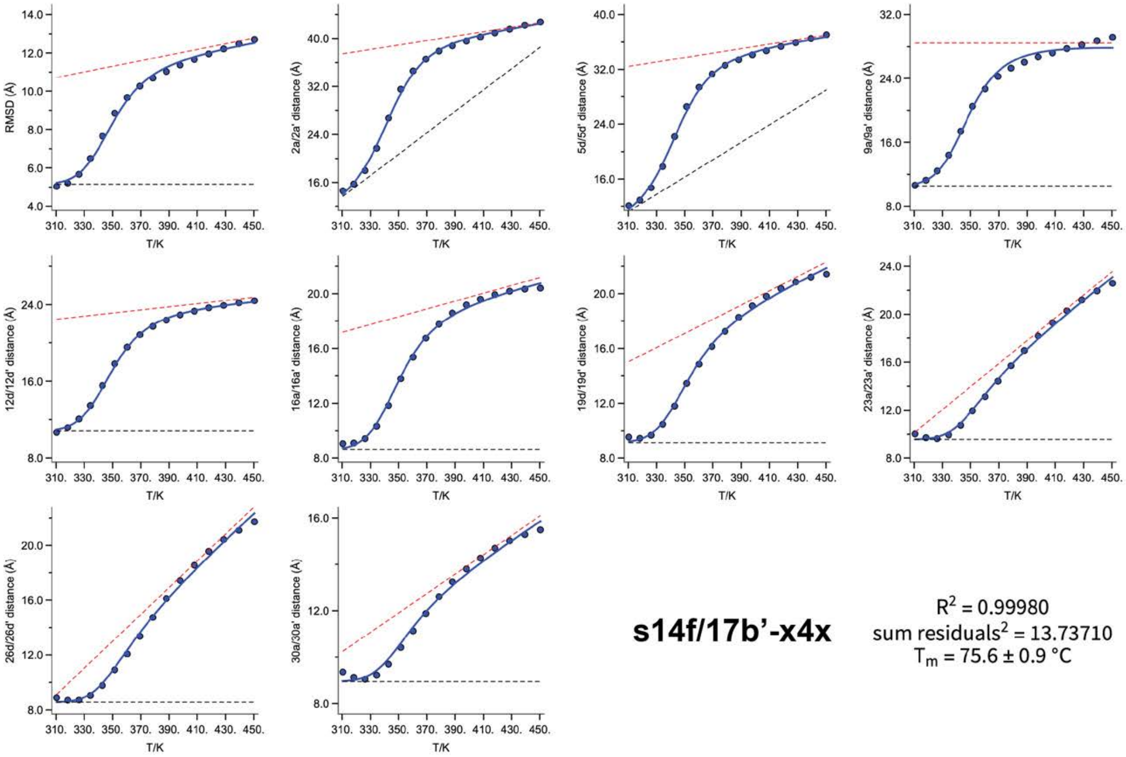

Parameters	Values	Units	P- Values
ΔH_0	- 19.50 ± 1.08	kcal/mol	<0.001
T_m	75.6 ± 0.9	°C	<0.001
ΔC_p	0.198 ± 0.062	kcal/mol/K	0.002
rmsfi	5.18 ± 0.19	deg cm^{-2} dmol^{-1} K^{-1} x 10^{-3}	<0.001
rmsfs	---	---	---
rmsui	11.32 ± 0.33	deg cm^{-2} dmol^{-1} x 10^{-3}	<0.001
rmsus	0.015 ± 0.004	deg cm^{-2} dmol^{-1} x 10^{-3}	<0.001
aa1fi	20.65 ± 0.83	deg cm^{-2} dmol^{-1} K^{-1} x 10^{-3}	<0.001
aa1fs	0.178 ± 0.022	deg cm^{-2} dmol^{-1} x 10^{-3}	<0.001
aa1ui	39.00 ± 0.75	deg cm^{-2} dmol^{-1} x 10^{-3}	<0.001
aa1us	0.037 ± 0.007	deg cm^{-2} dmol^{-1} x 10^{-3}	<0.001
dd1fi	16.25 ± 0.79	deg cm^{-2} dmol^{-1} K^{-1} x 10^{-3}	<0.001
dd1fs	0.127 ± 0.022	deg cm^{-2} dmol^{-1} x 10^{-3}	<0.001
dd1ui	33.80 ± 0.71	deg cm^{-2} dmol^{-1} x 10^{-3}	<0.001
dd1us	0.033 ± 0.006	deg cm^{-2} dmol^{-1} x 10^{-3}	<0.001
aa2fi	10.63 ± 0.23	deg cm^{-2} dmol^{-1} K^{-1} x 10^{-3}	<0.001
aa2fs	---	---	---
aa2ui	28.52 ± 0.58	deg cm^{-2} dmol^{-1} x 10^{-3}	<0.001
aa2us	---	---	---
dd2fi	10.90 ± 0.22	deg cm^{-2} dmol^{-1} K^{-1} x 10^{-3}	<0.001
dd2fs	---	---	---
dd2ui	23.18 ± 0.46	deg cm^{-2} dmol^{-1} x 10^{-3}	<0.001
dd2us	0.016 ± 0.005	deg cm^{-2} dmol^{-1} x 10^{-3}	<0.001
aa3fi	8.65 ± 0.20	deg cm^{-2} dmol^{-1} K^{-1} x 10^{-3}	<0.001
aa3fs	---	---	---
aa3ui	18.34 ± 0.41	deg cm^{-2} dmol^{-1} x 10^{-3}	<0.001
aa3us	0.029 ± 0.005	deg cm^{-2} dmol^{-1} x 10^{-3}	<0.001
dd3fi	9.17 ± 0.20	deg cm^{-2} dmol^{-1} K^{-1} x 10^{-3}	<0.001
dd3fs	---	---	---
dd3ui	17.10 ± 0.38	deg cm^{-2} dmol^{-1} x 10^{-3}	<0.001
dd3us	0.052 ± 0.005	deg cm^{-2} dmol^{-1} x 10^{-3}	<0.001
aa4fi	9.61 ± 0.19	deg cm^{-2} dmol^{-1} K^{-1} x 10^{-3}	<0.001
aa4fs	---	---	---
aa4ui	13.93 ± 0.33	deg cm^{-2} dmol^{-1} x 10^{-3}	<0.001
aa4us	0.096 ± 0.006	deg cm^{-2} dmol^{-1} x 10^{-3}	<0.001
dd4fi	8.60 ± 0.19	deg cm^{-2} dmol^{-1} K^{-1} x 10^{-3}	<0.001
dd4fs	---	---	---
dd4ui	12.95 ± 0.33	deg cm^{-2} dmol^{-1} x 10^{-3}	<0.001
dd4us	0.098 ± 0.006	deg cm^{-2} dmol^{-1} x 10^{-3}	<0.001
aa4fi	8.97 ± 0.18	deg cm^{-2} dmol^{-1} K^{-1} x 10^{-3}	<0.001
aa5fs	---	---	---
aa5ui	11.89 ± 0.29	deg cm^{-2} dmol^{-1} x 10^{-3}	<0.001
aa5us	0.042 ± 0.004	deg cm^{-2} dmol^{-1} x 10^{-3}	<0.001

Figure S486. Plots of average trajectory RMSD and average trajectory core distances as a function of temperature for variant **s14f/17b'-x4x**, along with the fit parameters ± standard error and statistics.

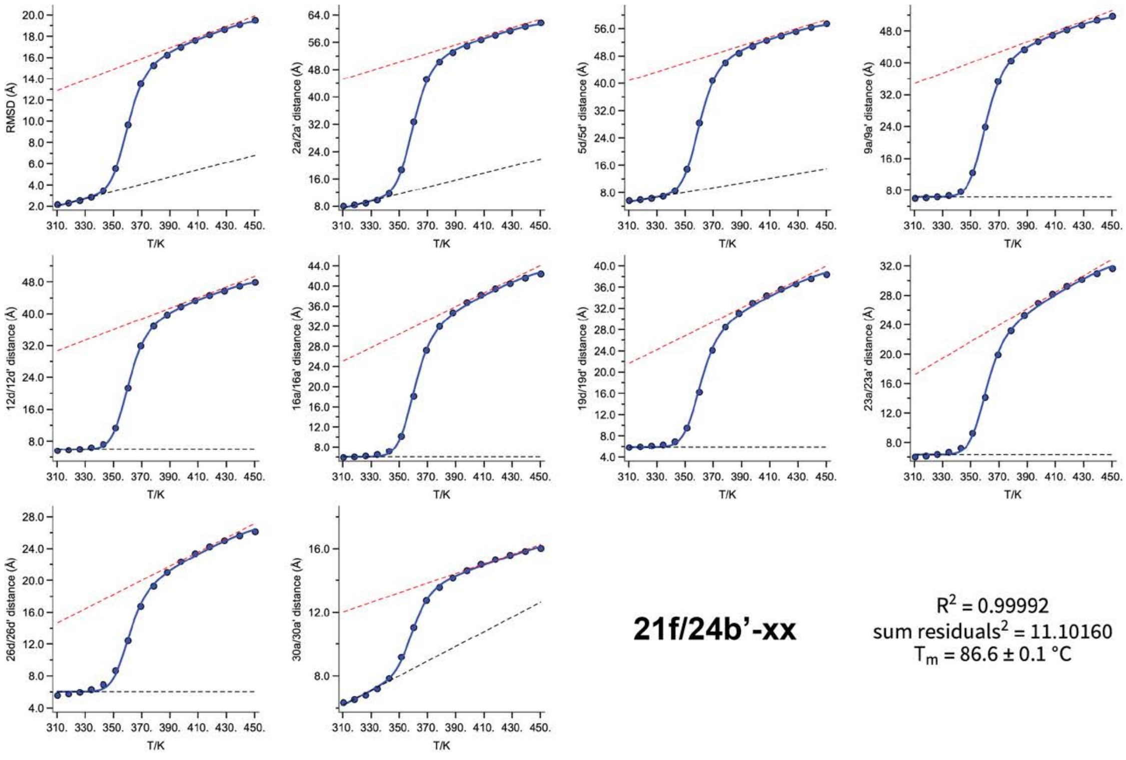

Parameters	Values	Units	P- Values
ΔH_0	- 41.30 ± 0.68	kcal/mol	<0.001
T_m	86.6 ± 0.1	°C	<0.001
ΔC_p	0.712 ± 0.024	kcal/mol/K	<0.001
rmsfi	3.79 ± 0.30	deg cm^{-2} dmol^{-1} K^{-1} x 10^{-3}	<0.001
rmsfs	0.034 ± 0.009	deg cm^{-2} dmol^{-1} x 10^{-3}	<0.001
rmsui	15.46 ± 0.25	deg cm^{-2} dmol^{-1} x 10^{-3}	<0.001
rmsus	0.050 ± 0.004	deg cm^{-2} dmol^{-1} x 10^{-3}	<0.001
aa1fi	12.77 ± 0.34	deg cm^{-2} dmol^{-1} K^{-1} x 10^{-3}	<0.001
aa1fs	0.102 ± 0.010	deg cm^{-2} dmol^{-1} x 10^{-3}	<0.001
aa1ui	51.68 ± 0.34	deg cm^{-2} dmol^{-1} x 10^{-3}	<0.001
aa1us	0.126 ± 0.006	deg cm^{-2} dmol^{-1} x 10^{-3}	<0.001
dd1fi	8.89 ± 0.34	deg cm^{-2} dmol^{-1} K^{-1} x 10^{-3}	<0.001
dd1fs	0.069 ± 0.010	deg cm^{-2} dmol^{-1} x 10^{-3}	<0.001
dd1ui	47.39 ± 0.34	deg cm^{-2} dmol^{-1} x 10^{-3}	<0.001
dd1us	0.128 ± 0.006	deg cm^{-2} dmol^{-1} x 10^{-3}	<0.001
aa2fi	6.44 ± 0.13	deg cm^{-2} dmol^{-1} K^{-1} x 10^{-3}	<0.001
aa2fs	---	---	---
aa2ui	41.52 ± 0.32	deg cm^{-2} dmol^{-1} x 10^{-3}	<0.001
aa2us	0.131 ± 0.006	deg cm^{-2} dmol^{-1} x 10^{-3}	<0.001
dd2fi	6.06 ± 0.13	deg cm^{-2} dmol^{-1} K^{-1} x 10^{-3}	<0.001
dd2fs	---	---	---
dd2ui	37.53 ± 0.30	deg cm^{-2} dmol^{-1} x 10^{-3}	<0.001
dd2us	0.134 ± 0.006	deg cm^{-2} dmol^{-1} x 10^{-3}	<0.001
aa3fi	6.15 ± 0.13	deg cm^{-2} dmol^{-1} K^{-1} x 10^{-3}	<0.001
aa3fs	---	---	---
aa3ui	31.97 ± 0.28	deg cm^{-2} dmol^{-1} x 10^{-3}	<0.001
aa3us	0.136 ± 0.005	deg cm^{-2} dmol^{-1} x 10^{-3}	<0.001
dd3fi	6.04 ± 0.13	deg cm^{-2} dmol^{-1} K^{-1} x 10^{-3}	<0.001
dd3fs	---	---	---
dd3ui	28.29 ± 0.27	deg cm^{-2} dmol^{-1} x 10^{-3}	<0.001
dd3us	0.131 ± 0.005	deg cm^{-2} dmol^{-1} x 10^{-3}	<0.001
aa4fi	6.39 ± 0.13	deg cm^{-2} dmol^{-1} K^{-1} x 10^{-3}	<0.001
aa4fs	---	---	---
aa4ui	22.91 ± 0.25	deg cm^{-2} dmol^{-1} x 10^{-3}	<0.001
aa4us	0.112 ± 0.005	deg cm^{-2} dmol^{-1} x 10^{-3}	<0.001
dd4fi	6.12 ± 0.13	deg cm^{-2} dmol^{-1} K^{-1} x 10^{-3}	<0.001
dd4fs	---	---	---
dd4ui	19.20 ± 0.24	deg cm^{-2} dmol^{-1} x 10^{-3}	<0.001
dd4us	0.089 ± 0.004	deg cm^{-2} dmol^{-1} x 10^{-3}	<0.001
aa4fi	8.52 ± 0.30	deg cm^{-2} dmol^{-1} K^{-1} x 10^{-3}	<0.001
aa5fs	0.046 ± 0.009	deg cm^{-2} dmol^{-1} x 10^{-3}	<0.001
aa5ui	13.57 ± 0.24	deg cm^{-2} dmol^{-1} x 10^{-3}	<0.001
aa5us	0.030 ± 0.004	deg cm^{-2} dmol^{-1} x 10^{-3}	<0.001

Figure S487. Plots of average trajectory RMSD and average trajectory core distances as a function of temperature for variant **21f/24b'-xx**, along with the fit parameters ± standard error and statistics.

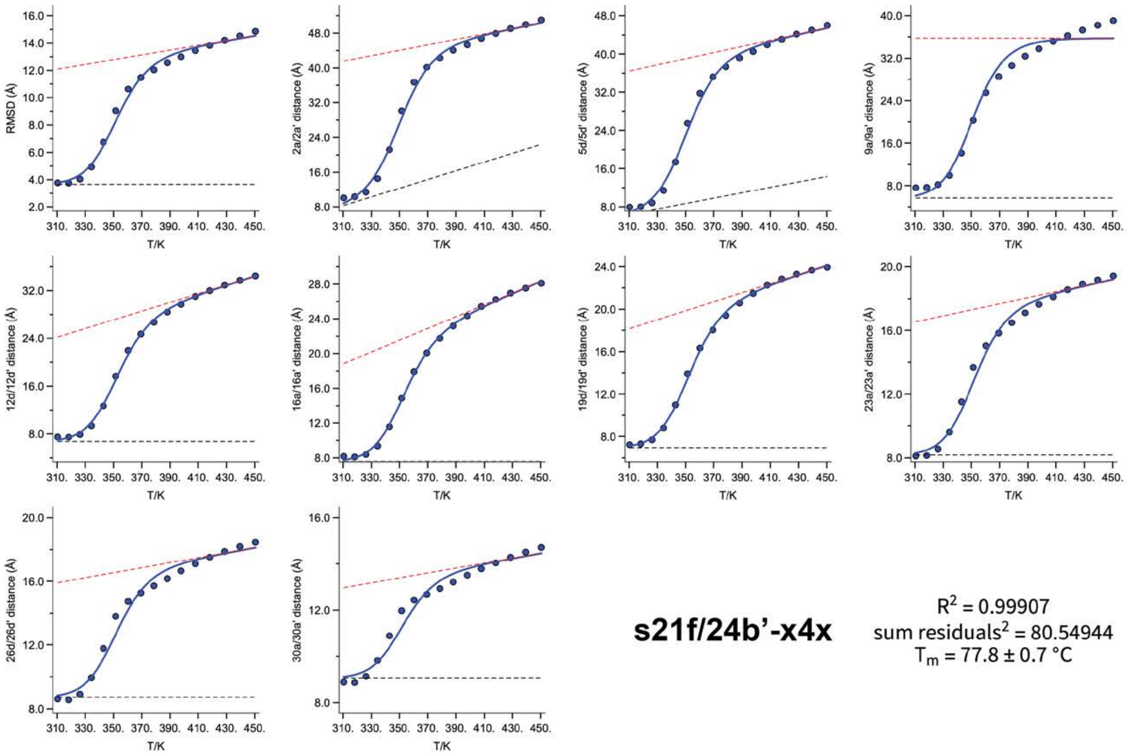

Parameters	Values	Units	P- Values
ΔH_0	- 22.03 ± 1.09	kcal/mol	<0.001
T_m	77.8 ± 0.7	°C	<0.001
ΔC_p	---	---	---
rmsfi	3.70 ± 0.43	deg cm^{-2} dmol^{-1} K^{-1} x 10^{-3}	<0.001
rmsfs	---	---	---
rmsui	12.86 ± 0.63	deg cm^{-2} dmol^{-1} x 10^{-3}	<0.001
rmsus	0.017 ± 0.010	deg cm^{-2} dmol^{-1} x 10^{-3}	0.077
aa1fi	12.57 ± 1.63	deg cm^{-2} dmol^{-1} K^{-1} x 10^{-3}	<0.001
aa1fs	0.101 ± 0.045	deg cm^{-2} dmol^{-1} x 10^{-3}	0.028
aa1ui	44.29 ± 1.02	deg cm^{-2} dmol^{-1} x 10^{-3}	<0.001
aa1us	0.063 ± 0.014	deg cm^{-2} dmol^{-1} x 10^{-3}	<0.001
dd1fi	8.88 ± 1.58	deg cm^{-2} dmol^{-1} K^{-1} x 10^{-3}	<0.001
dd1fs	0.057 ± 0.045	deg cm^{-2} dmol^{-1} x 10^{-3}	0.207
dd1ui	39.24 ± 1.02	deg cm^{-2} dmol^{-1} x 10^{-3}	<0.001
dd1us	0.064 ± 0.014	deg cm^{-2} dmol^{-1} x 10^{-3}	<0.001
aa2fi	5.77 ± 0.53	deg cm^{-2} dmol^{-1} K^{-1} x 10^{-3}	<0.001
aa2fs	---	---	---
aa2ui	35.89 ± 0.31	deg cm^{-2} dmol^{-1} x 10^{-3}	<0.001
aa2us	---	---	---
dd2fi	6.82 ± 0.48	deg cm^{-2} dmol^{-1} K^{-1} x 10^{-3}	<0.001
dd2fs	---	---	---
dd2ui	27.30 ± 0.73	deg cm^{-2} dmol^{-1} x 10^{-3}	<0.001
dd2us	0.073 ± 0.011	deg cm^{-2} dmol^{-1} x 10^{-3}	<0.001
aa3fi	7.65 ± 0.45	deg cm^{-2} dmol^{-1} K^{-1} x 10^{-3}	<0.001
aa3fs	---	---	---
aa3ui	21.69 ± 0.67	deg cm^{-2} dmol^{-1} x 10^{-3}	<0.001
aa3us	0.068 ± 0.010	deg cm^{-2} dmol^{-1} x 10^{-3}	<0.001
dd3fi	6.99 ± 0.45	deg cm^{-2} dmol^{-1} K^{-1} x 10^{-3}	<0.001
dd3fs	---	---	---
dd3ui	19.97 ± 0.66	deg cm^{-2} dmol^{-1} x 10^{-3}	<0.001
dd3us	0.043 ± 0.010	deg cm^{-2} dmol^{-1} x 10^{-3}	<0.001
aa4fi	8.21 ± 0.43	deg cm^{-2} dmol^{-1} K^{-1} x 10^{-3}	<0.001
aa4fs	---	---	---
aa4ui	17.36 ± 0.63	deg cm^{-2} dmol^{-1} x 10^{-3}	<0.001
aa4us	0.019 ± 0.010	deg cm^{-2} dmol^{-1} x 10^{-3}	0.053
dd4fi	8.77 ± 0.43	deg cm^{-2} dmol^{-1} K^{-1} x 10^{-3}	<0.001
dd4fs	---	---	---
dd4ui	16.59 ± 0.62	deg cm^{-2} dmol^{-1} x 10^{-3}	<0.001
dd4us	0.016 ± 0.010	deg cm^{-2} dmol^{-1} x 10^{-3}	0.106
aa4fi	9.08 ± 0.42	deg cm^{-2} dmol^{-1} K^{-1} x 10^{-3}	<0.001
aa5fs	---	---	---
aa5ui	13.43 ± 0.61	deg cm^{-2} dmol^{-1} x 10^{-3}	<0.001
aa5us	0.011 ± 0.010	deg cm^{-2} dmol^{-1} x 10^{-3}	0.268

Figure S488. Plots of average trajectory RMSD and average trajectory core distances as a function of temperature for variant **s21f/24b'-x4x**, along with the fit parameters ± standard error and statistics.

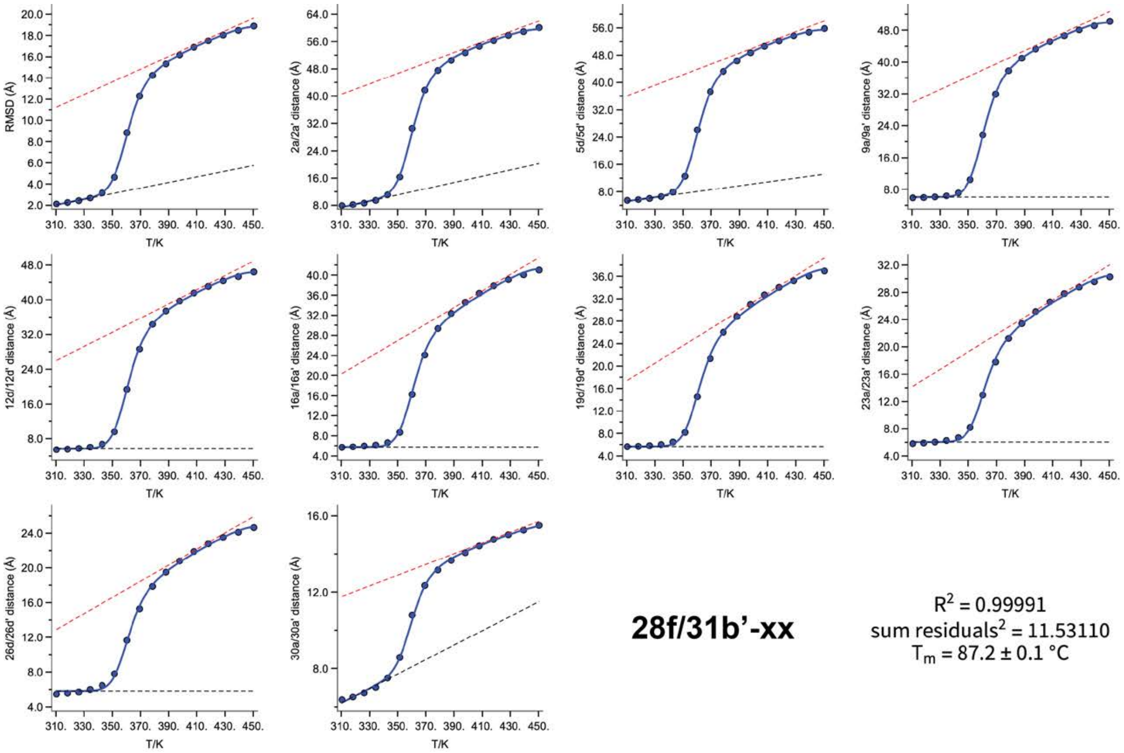

Parameters	Values	Units	P- Values
ΔH_0	- 41.89 ± 0.78	kcal/mol	<0.001
T_m	87.2 ± 0.1	°C	<0.001
ΔC_p	0.771 ± 0.022	kcal/mol/K	<0.001
rmsfi	3.43 ± 0.30	deg cm^{-2} dmol^{-1} K^{-1} x 10^{-3}	<0.001
rmsfs	0.026 ± 0.009	deg cm^{-2} dmol^{-1} x 10^{-3}	0.004
rmsui	14.33 ± 0.26	deg cm^{-2} dmol^{-1} x 10^{-3}	<0.001
rmsus	0.060 ± 0.004	deg cm^{-2} dmol^{-1} x 10^{-3}	<0.001
aa1fi	12.19 ± 0.34	deg cm^{-2} dmol^{-1} K^{-1} x 10^{-3}	<0.001
aa1fs	0.091 ± 0.010	deg cm^{-2} dmol^{-1} x 10^{-3}	<0.001
aa1ui	48.47 ± 0.36	deg cm^{-2} dmol^{-1} x 10^{-3}	<0.001
aa1us	0.154 ± 0.006	deg cm^{-2} dmol^{-1} x 10^{-3}	<0.001
dd1fi	8.23 ± 0.34	deg cm^{-2} dmol^{-1} K^{-1} x 10^{-3}	<0.001
dd1fs	0.057 ± 0.010	deg cm^{-2} dmol^{-1} x 10^{-3}	<0.001
dd1ui	44.16 ± 0.36	deg cm^{-2} dmol^{-1} x 10^{-3}	<0.001
dd1us	0.157 ± 0.006	deg cm^{-2} dmol^{-1} x 10^{-3}	<0.001
aa2fi	6.19 ± 0.13	deg cm^{-2} dmol^{-1} K^{-1} x 10^{-3}	<0.001
aa2fs	---	---	---
aa2ui	38.24 ± 0.33	deg cm^{-2} dmol^{-1} x 10^{-3}	<0.001
aa2us	0.163 ± 0.006	deg cm^{-2} dmol^{-1} x 10^{-3}	<0.001
dd2fi	5.80 ± 0.13	deg cm^{-2} dmol^{-1} K^{-1} x 10^{-3}	<0.001
dd2fs	---	---	---
dd2ui	34.39 ± 0.32	deg cm^{-2} dmol^{-1} x 10^{-3}	<0.001
dd2us	0.164 ± 0.006	deg cm^{-2} dmol^{-1} x 10^{-3}	<0.001
aa3fi	5.89 ± 0.13	deg cm^{-2} dmol^{-1} K^{-1} x 10^{-3}	<0.001
aa3fs	---	---	---
aa3ui	28.83 ± 0.30	deg cm^{-2} dmol^{-1} x 10^{-3}	<0.001
aa3us	0.165 ± 0.005	deg cm^{-2} dmol^{-1} x 10^{-3}	<0.001
dd3fi	5.78 ± 0.13	deg cm^{-2} dmol^{-1} K^{-1} x 10^{-3}	<0.001
dd3fs	---	---	---
dd3ui	25.41 ± 0.28	deg cm^{-2} dmol^{-1} x 10^{-3}	<0.001
dd3us	0.156 ± 0.005	deg cm^{-2} dmol^{-1} x 10^{-3}	<0.001
aa4fi	6.12 ± 0.13	deg cm^{-2} dmol^{-1} K^{-1} x 10^{-3}	<0.001
aa4fs	---	---	---
aa4ui	20.71 ± 0.26	deg cm^{-2} dmol^{-1} x 10^{-3}	<0.001
aa4us	0.127 ± 0.005	deg cm^{-2} dmol^{-1} x 10^{-3}	<0.001
dd4fi	5.88 ± 0.13	deg cm^{-2} dmol^{-1} K^{-1} x 10^{-3}	<0.001
dd4fs	---	---	---
dd4ui	17.66 ± 0.25	deg cm^{-2} dmol^{-1} x 10^{-3}	<0.001
dd4us	0.093 ± 0.004	deg cm^{-2} dmol^{-1} x 10^{-3}	<0.001
aa4fi	8.15 ± 0.30	deg cm^{-2} dmol^{-1} K^{-1} x 10^{-3}	<0.001
aa5fs	0.038 ± 0.009	deg cm^{-2} dmol^{-1} x 10^{-3}	<0.001
aa5ui	13.22 ± 0.25	deg cm^{-2} dmol^{-1} x 10^{-3}	<0.001
aa5us	0.028 ± 0.004	deg cm^{-2} dmol^{-1} x 10^{-3}	<0.001

Figure S489. Plots of average trajectory RMSD and average trajectory core distances as a function of temperature for variant **28f/31b'-xx**, along with the fit parameters ± standard error and statistics.

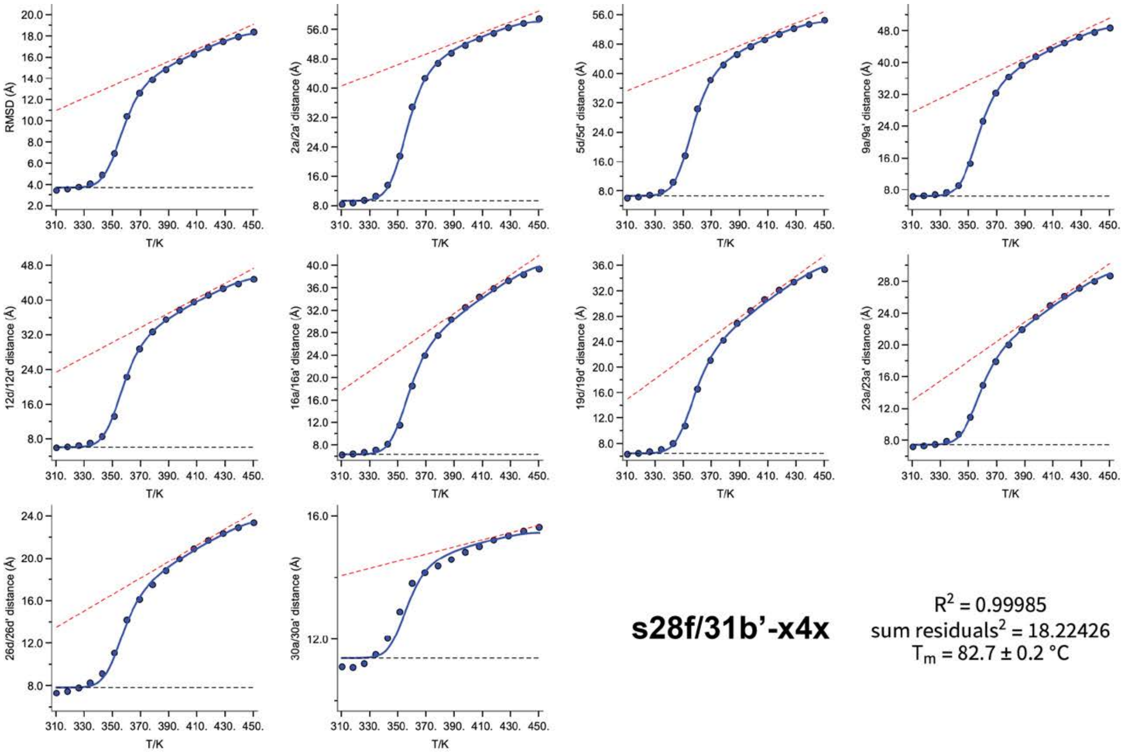

Parameters	Values	Units	P- Values
ΔH_0	- 35.34 ± 0.85	kcal/mol	<0.001
T_m	82.7 ± 0.2	°C	<0.001
ΔC_p	0.595 ± 0.024	kcal/mol/K	<0.001
rmsfi	3.78 ± 0.17	deg cm^{-2} dmol^{-1} K^{-1} x 10^{-3}	<0.001
rmsfs	---	---	---
rmsui	13.70 ± 0.30	deg cm^{-2} dmol^{-1} x 10^{-3}	<0.001
rmsus	0.058 ± 0.005	deg cm^{-2} dmol^{-1} x 10^{-3}	<0.001
aa1fi	9.46 ± 0.18	deg cm^{-2} dmol^{-1} K^{-1} x 10^{-3}	<0.001
aa1fs	---	---	---
aa1ui	47.46 ± 0.50	deg cm^{-2} dmol^{-1} x 10^{-3}	<0.001
aa1us	0.146 ± 0.008	deg cm^{-2} dmol^{-1} x 10^{-3}	<0.001
dd1fi	6.77 ± 0.18	deg cm^{-2} dmol^{-1} K^{-1} x 10^{-3}	<0.001
dd1fs	---	---	---
dd1ui	42.51 ± 0.49	deg cm^{-2} dmol^{-1} x 10^{-3}	<0.001
dd1us	0.154 ± 0.008	deg cm^{-2} dmol^{-1} x 10^{-3}	<0.001
aa2fi	6.51 ± 0.17	deg cm^{-2} dmol^{-1} K^{-1} x 10^{-3}	<0.001
aa2fs	---	---	---
aa2ui	35.49 ± 0.44	deg cm^{-2} dmol^{-1} x 10^{-3}	<0.001
aa2us	0.169 ± 0.007	deg cm^{-2} dmol^{-1} x 10^{-3}	<0.001
dd2fi	6.21 ± 0.17	deg cm^{-2} dmol^{-1} K^{-1} x 10^{-3}	<0.001
dd2fs	---	---	---
dd2ui	31.40 ± 0.42	deg cm^{-2} dmol^{-1} x 10^{-3}	<0.001
dd2us	0.171 ± 0.007	deg cm^{-2} dmol^{-1} x 10^{-3}	<0.001
aa3fi	6.41 ± 0.17	deg cm^{-2} dmol^{-1} K^{-1} x 10^{-3}	<0.001
aa3fs	---	---	---
aa3ui	25.76 ± 0.38	deg cm^{-2} dmol^{-1} x 10^{-3}	<0.001
aa3us	0.172 ± 0.006	deg cm^{-2} dmol^{-1} x 10^{-3}	<0.001
dd3fi	6.51 ± 0.17	deg cm^{-2} dmol^{-1} K^{-1} x 10^{-3}	<0.001
dd3fs	---	---	---
dd3ui	22.47 ± 0.35	deg cm^{-2} dmol^{-1} x 10^{-3}	<0.001
dd3us	0.161 ± 0.006	deg cm^{-2} dmol^{-1} x 10^{-3}	<0.001
aa4fi	7.51 ± 0.17	deg cm^{-2} dmol^{-1} K^{-1} x 10^{-3}	<0.001
aa4fs	---	---	---
aa4ui	18.78 ± 0.32	deg cm^{-2} dmol^{-1} x 10^{-3}	<0.001
aa4us	0.123 ± 0.005	deg cm^{-2} dmol^{-1} x 10^{-3}	<0.001
dd4fi	7.88 ± 0.17	deg cm^{-2} dmol^{-1} K^{-1} x 10^{-3}	<0.001
dd4fs	---	---	---
dd4ui	17.12 ± 0.30	deg cm^{-2} dmol^{-1} x 10^{-3}	<0.001
dd4us	0.077 ± 0.005	deg cm^{-2} dmol^{-1} x 10^{-3}	<0.001
aa4fi	11.40 ± 0.17	deg cm^{-2} dmol^{-1} K^{-1} x 10^{-3}	<0.001
aa5fs	---	---	---
aa5ui	14.62 ± 0.28	deg cm^{-2} dmol^{-1} x 10^{-3}	<0.001
aa5us	0.012 ± 0.005	deg cm^{-2} dmol^{-1} x 10^{-3}	0.017

Figure S490. Plots of average trajectory RMSD and average trajectory core distances as a function of temperature for variant **s28f/31b'-x4x**, along with the fit parameters ± standard error and statistics.

(return to Table of Contents)

14. Calculating the difference in RMSF for each atom in the 310-K trajectories of each stapled variant relative to its non-stapled counterpart

We used *cpptraj* to align the backbone atoms from each frame in the 310-K T-REMD trajectory of each coiled-coil variant to corresponding atoms within a reference structure derived from the crystal structure of the parent coiled coil **A/B** (PDB 1kd9). We used *cpptraj* to calculate the root-mean-square fluctuation (RMSF) of each atom in each coiled-coil variant. We then used subtraction to compare the RMSF values of each atom in a stapled variant with the RMSF values for corresponding atoms in its non-stapled counterpart ($\Delta RMSF = RMSF_{stapled} - RMSF_{non\text{-}stapled}$). We then used Pymol to generate the main-text images of each stapled variant based on the median-energy frame of the 310-K T-REMD trajectory of that variant, with atoms colored according to their ΔRMSF values.

15. Correlation between simulated and observed values of melting temperatures and stabilization of stapled variants compared to their non-stapled counterparts

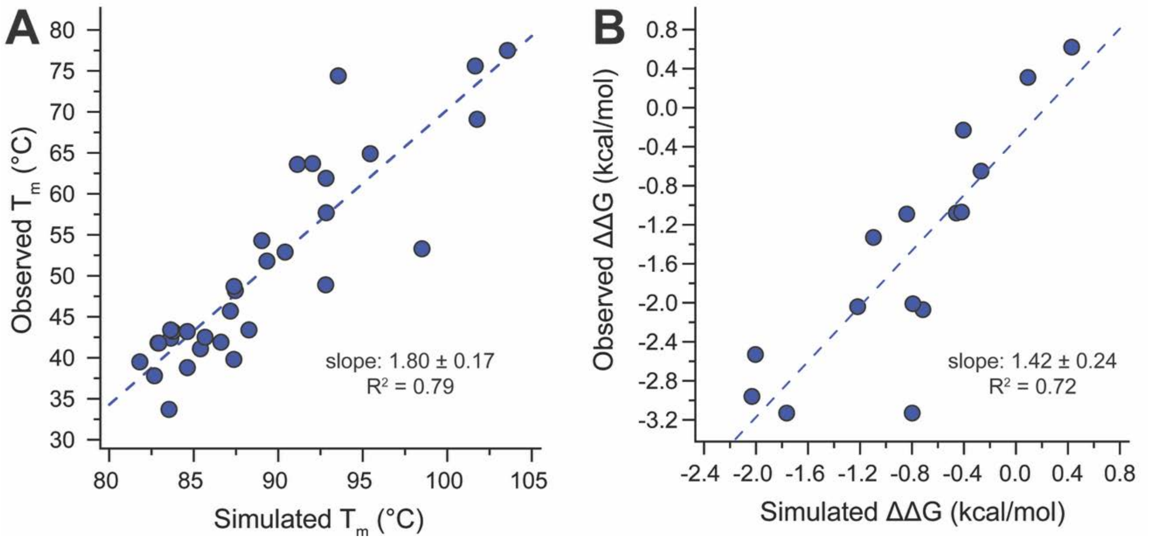

Figure S491. Correlations between simulated vs. observed values of (A) the melting temperatures T_m of stapled variants stapled variants **s6e/8g'-z4x**, **s13e/15g'-z4x**, **s20e/22g'-z4x**, **s27e/29g'-z4x**, and **s27e/29g'-x4x** (nsb e/g' sites); **s6e/1g'-x4x**, **s13e/8g'-x4x**, **s20e/15g'-x4x**, **s27e/22g'-z4x**, **s27e/22g'-x4x**, and **s34e/29g'-x4x** (sb e/g' sites); **s7f/10b'-x4x**, **s14f/17b'-x4x**, **s21f/24b'-x4x**, and **s28f/31b'-x4x** (f/b' sites); and their non-stapled counterparts; and **(B)** the stabilization ΔΔG of each stapled variant relative to its non-stapled counterpart. Dotted blue lines represent fits of these data to linear equations, with the R^2 values indicated. Observed T_m and ΔΔG values for stapled variants **s6e/8g'-z4x**, **s13e/15g'-z4x**, **s20e/22g'-z4x, s27e/29g'-z4x**, **s27e/29g'-x4x** and their non-stapled counterparts are from reference 53, whereas simulated T_m and ΔΔG values for stapled variants **s6e/8g'-z4x**, **s13e/15g'-z4x**, **s20e/22g'-z4x, s27e/29g'-x4x** and their non-stapled counterparts are from reference 55. Observed and simulated T_m and ΔΔG values for all other variants are newly reported here.

As shown in Figure S491A, the simulated T_m values are higher in magnitude than the corresponding observed T_m values. This difference likely reflects the difference between our experiments, which were conducted in 20 mM phosphate buffer + 4 M guanidinium chloride denaturant (as explained in Section _ above), and our simulations, which were done without denaturant in implicit solvent. We included denaturant in our experiments

because many of these variants do not completely unfold in the absence of denaturant, even at 95 °C, the highest experimentally accessible temperature. For simplicity, and because our implicit solvent T-REMD simulations are not limited to temperatures below 100 °C, we did not include denaturants in our simulations. We did this knowing that the observed T_m values would necessarily be lower than they would have been without denaturant, and therefore likely lower than the simulated T_m values. However, we reasoned that comparison of T_m values among variants would still be valid, and in any case, we were less interested in the absolute stability of each variant than in the stability of each stapled variant relative to its non-stapled counterpart. Despite the difference in absolute values described above, the correlation between simulated and observed T_m values is strong (R^2 = 0.79), suggesting that the implicit-solvent T-REMD simulations recapitulate relationships between stability of each variant relative to that of all the other variants explored here, and not just the difference between a stapled-variant and its non-stapled counterpart.

Figure S491B shows the strong correlation (R^2 = 0.72) between simulated and observed ΔΔG values for each stapled variant relative to its non-stapled counterpart. The simulated ΔΔG values are generally smaller than their observed counterparts, for reasons that are not clear. However, the simulations correctly identify three of the four most stabilizing stapling sites from our observations and the three most destabilizing stapling sites, suggesting the potential for similar simulations to predicting which among many prospective stapling sites in a protein is likely to be most stabilizing.

(return to Table of Contents)

ESI References

1. Q. Xiao, D. S. Ashton, Z. B. Jones, K. P. Thompson and J. L. Price, *RSC Chem. Biol.*, 2020, **1**, 273–280.
2. P. B. Lawrence, Y. Gavrilov, S. S. Matthews, M. I. Langlois, D. Shental-Bechor, H. M. Greenblatt, B. K. Pandey, M. S. Smith, R. Paxman, C. D. Torgerson, J. P. Merrell, C. C. Ritz, M. B. Prigozhin, Y. Levy and J. L. Price, *J Am Chem Soc*, 2014, **136**, 17547-17560.
3. Dennington, R.; Keith, T. A.; Millam, J. M. GaussView 6; Semichem Inc.: Shawnee Mission, KS, 2016.
4. J. L. Price, 2025, **129**, 866–875.
5. Tian, C.; Kasavajhala, K.; Belfon, K. A. A.; Raguette, L.; Huang, H.; Migues, A. N.; et al. ff19SB: AminoAcid-Specific Protein Backbone Parameters Trained against Quantum Mechanics Energy Surfaces in Solution. J. Chem. Theory. Comput. 2020, 16 (1), 528–552. DOI: 10.1021/acs.jctc.9b00591.
6. D.A. Case, H. M. A., K. Belfon, I.Y. Ben-Shalom, J.T. Berryman, S.R. Brozell, D.S. Cerutti, T.E. Cheatham, III, G.A. Cisneros, V.W.D. Cruzeiro, T.A. Darden, R.E. Duke, G. Giambasu, M.K. Gilson, H. Gohlke, A.W. Goetz, R. Harris, S. Izadi, S.A. Izmailov, K. Kasavajhala, M.C. Kaymak, E. King, A. Kovalenko, T. Kurtzman, T.S. Lee, S. LeGrand, P. Li, C. Lin, J. Liu, T. Luchko, R. Luo, M. Machado, V. Man, M. Manathunga, K.M. Merz, Y. Miao, O. Mikhailovskii, G. Monard, H. Nguyen, K.A. O'Hearn, A. Onufriev, F. Pan, S. Pantano, R. Qi, A. Rahnamoun, D.R. Roe, A. Roitberg, C. Sagui, S. Schott-Verdugo, A. Shajan, J. Shen, C.L. Simmerling, N.R. Skrynnikov, J. Smith, J. Swails, R.C. Walker, J. Wang, J. Wang, H. Wei, R.M. Wolf, X. Wu, Y. Xiong, Y. Xue, D.M. York, S. Zhao, and P.A. Kollman Amber 2020 2020.
7. He, X.; Man, V. H.; Yang, W.; Lee, T.-S.; Wang, J. A fast and high-quality charge model for the next generation general AMBER force field. J. Chem. Phys. 2020, 153 (11). DOI: 10.1063/5.0019056.

8. Onufriev, A.; Bashford, D.; Case, D. A. Exploring protein native states and large-scale conformational changes with a modified generalized born model. Proteins-Structure Function and Bioinformatics 2004, 55 (2), 383–394. DOI: 10.1002/prot.20033.
9. Ryckaert, J. P.; Ciccotti, G.; Berendsen, H. J. C. Numerical-Integration of Cartesian Equations of Motion of a System with Constraints - Molecular-Dynamics of N-Alkanes. J. Comput. Phys. 1977, 23 (3), 327–341. DOI: 10.1016/0021-9991(77)90098-5.
10. Mathematica Version 13.3; Wolfram Research, Inc.: Champaign, Illinois, 2023.
